# Combinatorial discovery of small-molecule 1,2,3-triazolium ionic liquids exhibiting lower critical solution temperature phase transition

**DOI:** 10.1038/s41598-020-75392-z

**Published:** 2020-10-26

**Authors:** Yen-Ho Chu, Mou-Fu Cheng, Yung-Hsin Chiang

**Affiliations:** Department of Chemistry and Biochemistry, National Chung Cheng University, Chiayi, 62102 Taiwan, ROC

**Keywords:** Biotechnology, Chemistry, Materials science

## Abstract

Both lower and upper critical solution temperature (LCST and UCST) systems are two typical phase behaviors of thermoresponsive materials with solvents, in which LCST is far less common than UCST. Recent studies on ionic liquids carrying LCST phase transitions have predominantly focused on quaternary ammonium- and phosphonium-based ionic salts. Based on the 1,2,3-triazole core structure assemblable by azide-alkyne cycloaddition click reaction, this work reports the combinatorial synthesis of 1,3,4-trialkylated 1,2,3-triazolium ionic liquids in three libraries with a total of 160 ionic liquids and demonstrates, for the first time, their values in temperature-switchable phase transition with water. In this work, the successful discovery of a new thermoresponsive ionic liquid **b26**, based on the structure-and-phase separation study of **b8** and **b9**, perfectly exemplified the true value of the tunability of ionic liquid fine structures. For all 160 ionic liquids synthesized, 155 are liquid at room temperature and 22 room-temperature ionic liquids were found to exhibit thermoresponsive phase transitions having low *T*_*c*_ values in water. To the best of our knowledge, this comprehensive study is the first report of small-molecule 1,2,3-triazolium ionic liquids that exhibit LCST property in water.

## Introduction

Based on click chemistry^1^, this work reports structurally tunable ionic liquids tailored specifically for concise design and combinatorial synthesis of thermoresponsive ionic liquids (TILs)^2–4^ carrying lower critical solution temperature (LCST)-type phase transitions. Ionic liquids are entirely made up of ions^2^ and carry negligible vapor pressure, and, not surprisingly, many of them are miscible in water. As ionic liquids contribute insignificant pollution to air, some of them exhibit unique properties with water such as temperature-driven phase transition (e.g., LCST). This phase behavior of ionic liquids in water is highly dependent on the component ions and imperative in various aspects of reaction catalysis, extraction and separation^2–4^. Moreover, their structures can be synthetically altered, tuned, and controlled by the engineering of cations or anions. In fact, significant progress has been made in exploring functionalized ionic liquids through the incorporation of appropriate groups to equip novel ionic liquids with tailored properties for targeted applications^2–4^.

**Figure 1:**
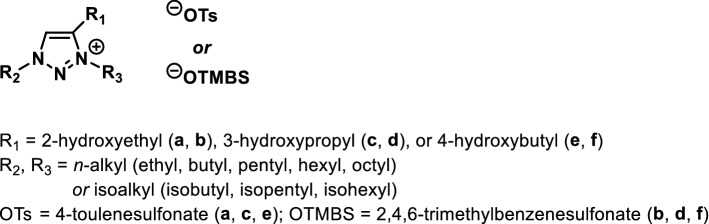
Structures of 1,2,3-triazolium ionic liquids (**a**–**f**).

To date, a myriad of researches and studies have focused on imidazolium-based ionic liquids. Based specifically on the 1,2,3-triazole core structure assemblable by click reaction^5,6^, this work develops combinatorial synthesis of 1,3,4-trialkylated 1,2,3-triazolium ionic liquids **a–f** (three sets of libraries with 160 ionic liquids in total, Fig. 1) and demonstrates, for the first time, their values in temperature-switchable phase transition with water.

LCST and upper critical solution temperature (UCST) systems are two typical phase behaviors of TILs, in which LCST is far less common than UCST. Ohno and coworkers have been pioneers on the studies of quaternary ammonium- and phosphonium-based TILs with LCST phase transitions^3,4,7^. Albeit certain ionic salts and neutral polymers were found LCST transition with water^4^, no small-molecule triazolium TILs have been reported in literature and there was also no comprehensive and systematic study on structural design of ionic liquids to demonstrate temperature responsiveness. We are interested in developing new ionic liquids^8–10^, were intrigued by the recent advances of TILs^2–4,7,11–13^, and envisaged that ionic liquid libraries (**a**–**f**) embedded with the core structure of 1,2,3-triazole (Fig. 1) are non-volatile, structurally tunable salts and should be of new candidate TILs (other than known ammonium- and phosphonium-based TILs) for the structure-and-phase separation relationship (SPS) study. For exactly this reason, we set out to develop a generalized library synthesis for 1,3,4-trialkylated 1,2,3-triazolium ionic liquids **a**–**f** and aimed at the combinatorial discovery of new TILs with temperature-switchable phase separation.

## Results and discussion

### First library of 57 room-temperature ionic liquids (a1–a28 and b1–b29)

**Figure 2:**
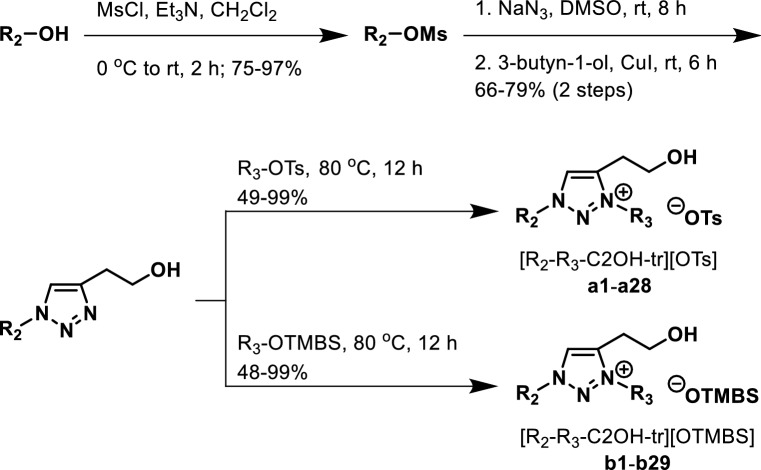
Synthesis of thermoresponsive ionic liquids, **a1**–**a28** and **b1**–**b29**.

Using 4-toulenesulfonate (OTs) and 2,4,6-trimethylbenzenesulfonate (OTMBS) as anions, we aimed at providing their potential to participate in π-π interactions with triazolium cations and, accordingly, synthesized our ionic liquid library with the hope to facilitate the discovery of TILs. Figure 2 illustrates the synthesis of our first library of 57 room-temperature ionic liquids, [R_2_-R_3_-C2OH-tr][OTs] (**a1**–**a28**) and [R_2_-R_3_-C2OH-tr][OTMBS] (**b1**–**b29**), of which the core 1,2,3-triazole could be readily assembled from a copper(I)-catalyzed azide-alkyne cycloaddition (CuAAC) reaction^5,6^ at room temperature. We commenced our synthesis from commercial alcohols, which underwent a sequence of reactions (i.e., alcohol mesylation, nucleophilic substitution with sodium azide, the key CuAAC reaction, and, finally, ionic liquid forming reaction with corresponding arylsulfonate) to ultimately afford the desired ionic liquids. The R_1_, R_2_ and R_3_ components in product structures (Fig. 1) are all from commercial aliphatic alcohols. The ionic liquid syntheses were chemically straightforward and the overall isolated yields, in our hands, for these 4-step syntheses of **a1**–**a28** and **b1**–**b29** were acceptable: 31–73% and 28–65%, respectively (Fig. 2). All **a1**–**a28** and **b1**–**b29** ionic liquids obtained are liquidous at room temperature. Detailed experimental procedures, ^1^H and ^13^C NMR, and high-resolution mass spectrometry (HRMS) spectra and data of all 57 room-temperature ionic liquids are summarized in the Supporting Information (ESI-1).

Ionic liquid in water can display phase transition and its phase behavior is governed by the total hydrophobicity/hydrophilicity as well as its aggregation state of the constituent cation and anion of the ionic liquid^4,7,11^. Typically, for an aqueous ionic liquid mixture having LCST phase transition, a single phase appears at low temperatures, and, upon heating above a critical temperature *T*_*c*_, the solution separates into two immiscible phases. This is most likely due to strong intermolecular interactions (e.g., hydrogen bonding) between TIL and water leading to aggregates formation at lower temperatures, but those intermolecular interactions are ruptured upon heating above *T*_*c*_.

**Figure 3:**
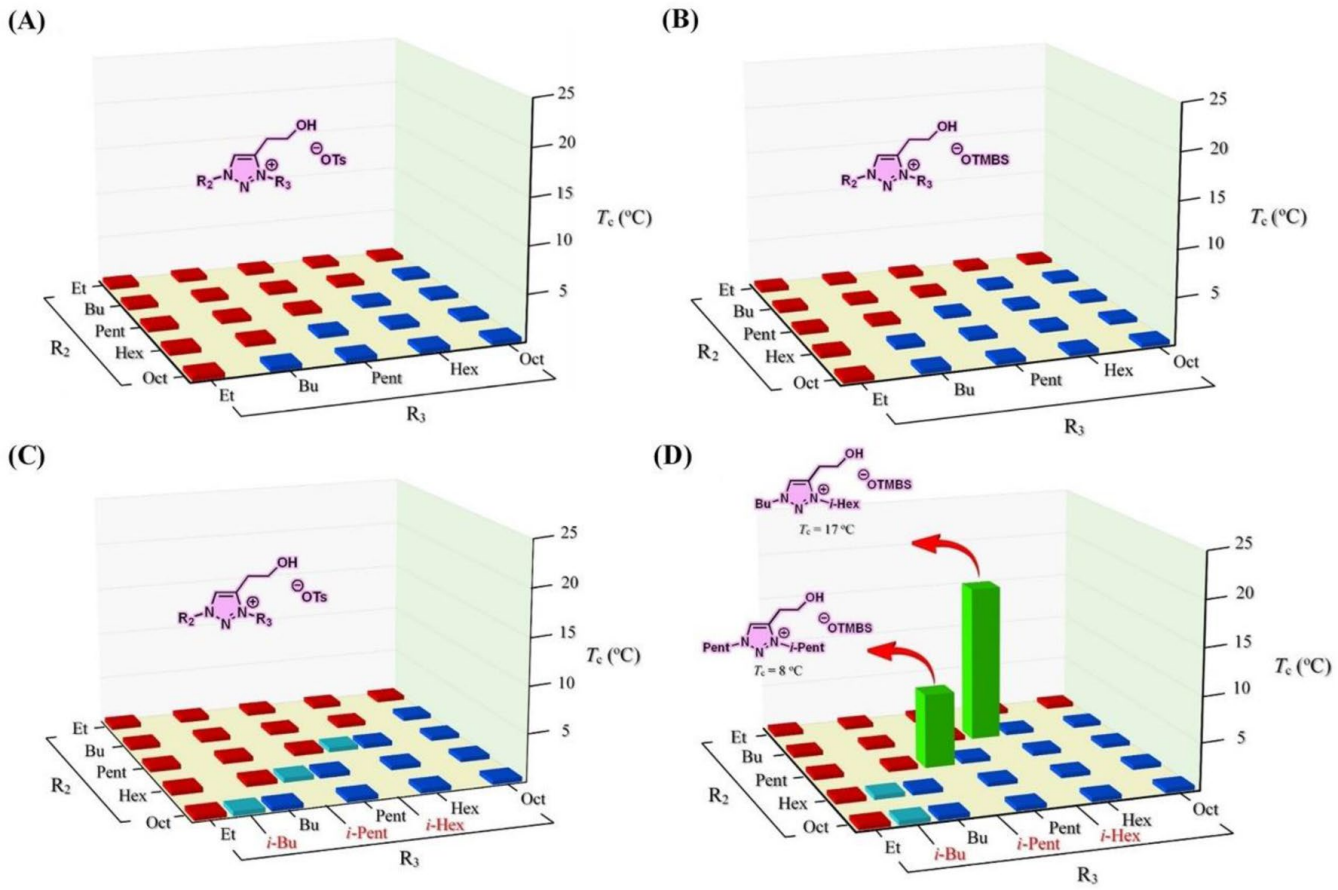
Phase transitions of a library of 57 room-temperature ionic liquids upon mixing with water (1:2, w/w) at temperatures between 2 °C and 90 °C: (**A**) ionic liquids **a1**–**a25**, (**B**) ionic liquids **b1–b25**, (**C**) ionic liquids **a1**–**a25** along with 3 additional ionic liquids having branched sidechains **a26**–**a28**, and (**D**) ionic liquids **b1**–**b25** along with 4 additional ionic liquids with branched sidechains **b26**–**b29**. Phase transition results shown in red and blue indicate entirely homogeneous (one-phase) and heterogeneous (two-phase) solutions, respectively, between 2 °C and 90 °C. In this library, two ionic liquids show phase transitions: **b26** ([Bu-*i*-Hex-C2OH-tr][OTMBS]; *T*_c_ = 17 °C), and **b27** ([Pent-*i*-Pent-C2OH-tr][OTMBS]; *T*_c_ = 8 °C).

Figure 3 shows a library of 57 room-temperature ionic liquids, [R_2_-R_3_-C2OH-tr][OTs] (**a1**–**a28**) and [R_2_-R_3_-C2OH-tr][OTMBS] (**b1**–**b29**), and their phase transitions toward temperature changes in water. In this work, to discover ionic liquids with thermoresponsiveness, each ionic salt was mixed with water in a mass ratio of 1:2 (w/w) and the mixture was then placed in a 2 °C ice bath followed by gradual heating until it reached 90 °C. Phase transition temperature (*T*_c_) for LCST was determined at the temperature point when the aqueous solution turned cloudy during heating by naked eyes^3,7^. At first, our results from screening of the library of 50 ionic liquids, **a1**–**a25** and **b1**–**b25**, were all disappointed (Fig. 3A,B) and no single ionic liquid showed any promising LCST phase transition. Among them, only single-phase, hydrophilic (labeled in red) and two-phase, hydrophobic (labeled in blue) ionic liquids were observed. Not surprisingly, more hydrophobic ionic liquids were experimentally observed in [OTMBS]-based (thirteen blues in Fig. 3B) than those of [OTs]-based (ten blues in Fig. 3A) ionic liquids.

Since it was reported^4,7^ that phase behavior of a TIL is a fine balance between hydrophobicity and hydrophilicity of the ionic liquid investigated, we therefore reasoned that, if R_3_ group could be tuned further and altered slightly in its structure (i.e., *n*-alkyl amended to *iso*-alkyl) for ionic liquids that are on the rim between being totally hydrophilic and being totally hydrophobic, the phase transition might be experimentally obtained. Here, R_3_ group, rather than R_2_ group, was targeted for the reason of its ease as the final step in the synthesis of ionic liquids (Fig. 2). Accordingly, seven ionic liquids **a26**–**a28** and **b26**–**b29**, labeled in green and light blue (Fig. 3C,D), all appeared on the edge between hydrophilic red and hydrophobic blue were identified and then synthesized. Gratifyingly, among these seven ionic liquids tested, two were discovered having LCST phase transition: [Bu-*i*-Hex-C2OH-tr][OTMBS] (**b26**) and [Pent-*i*-Pent-C2OH-tr][OTMBS] (**b27**) (Fig. 3D). It is worth highlighting that both **b26** and **b27** ionic liquids labeled in green carry *T*_*c*_ values that are below room temperature: *T*_c_ = 17 °C and 8 °C, respectively.

**Figure 4:**
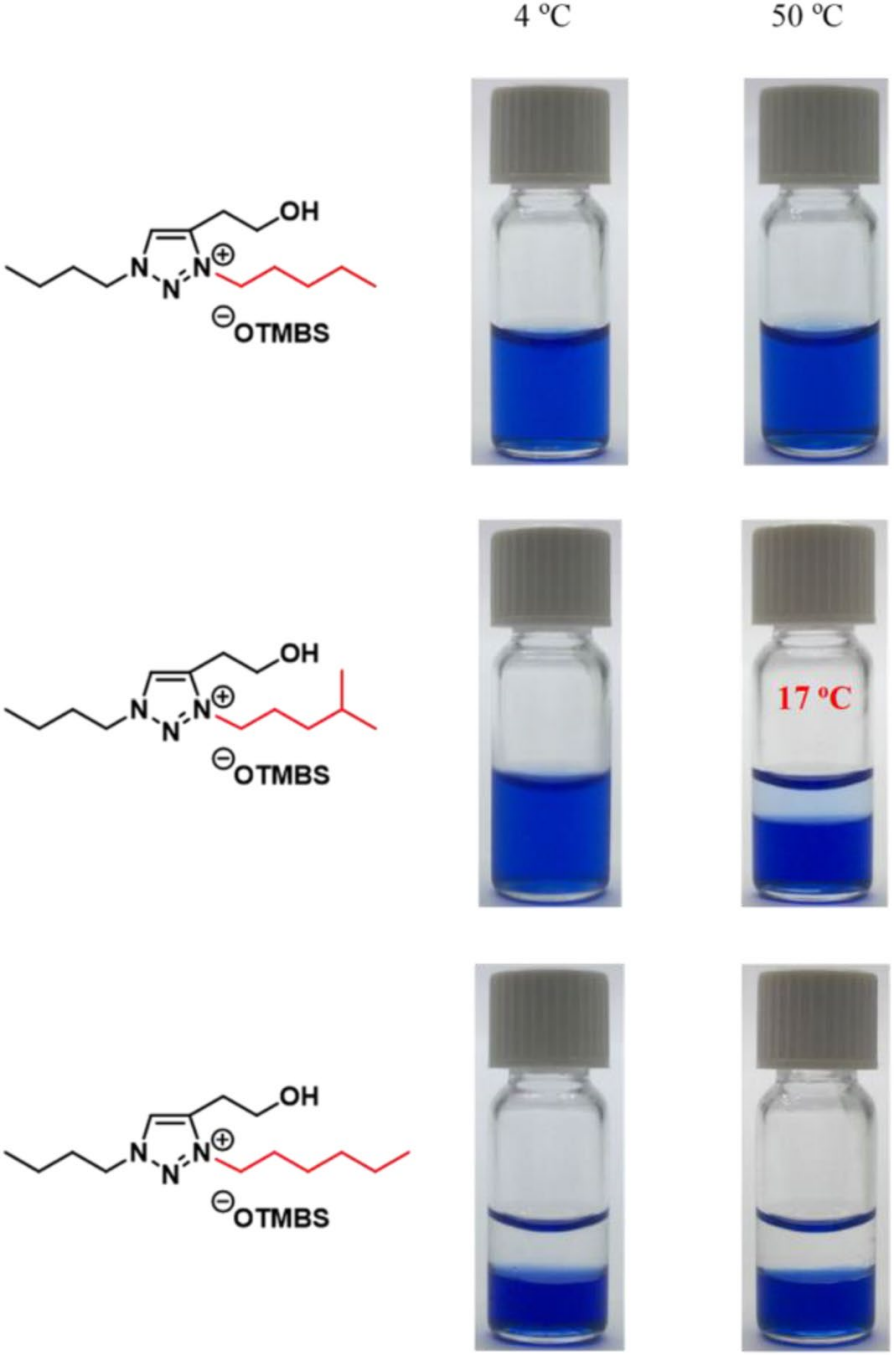
Temperature-dependent phase transitions of binary mixtures (2:1, w/w) of water with [Bu-Pent-C2OH-tr][OTMBS] (**b8**), [Bu-*i*-Hex-C2OH-tr][OTMBS] (**b26**), and [Bu-Hex-C2OH-tr][OTMBS] (**b9**), respectively. The coomassie brilliant blue R-250 (0.006 wt% in water) was added to accentuate the phase separation. Only [Bu-*i*-Hex-C2OH-tr][OTMBS] (**b26**) ionic liquid shows phase transition (*T*_c_ = 17 °C), whereas [Bu-Pent-C2OH-tr][OTMBS] (**b8**) and [Bu-Hex-C2OH-tr][OTMBS] (**b9**) are totally miscible and immiscible, respectively, with water at temperatures between 2 °C and 90 °C.

Figure 4 shows photos of phase behavior for a set of three representative ionic liquids, [Bu-Pent-C2OH-tr][OTMBS] (**b8**), [Bu-*i*-Hex-C2OH-tr][OTMBS] (**b26**), and [Bu-Hex-C2OH-tr][OTMBS] (**b9**). The result unambiguously demonstrated that the engineering of R_3_ sidechain from *n*-pentyl (Pent) group in **b8** to a ‘one-carbon-longer’ *n*-hexyl (Hex) group in **b9** completely changed their phase behavior from being totally hydrophilic to completely hydrophobic. Most significantly, the incorporation of an isohexyl (*i*-Hex; 4-methyl-1-pentyl) group to replace the Pent or Hex group stunningly changed phase behavior and evidently made **b26** a new small-molecule TIL with LCST phase transition in water (Fig. 4). This successful development of **b26** as TIL clearly highlighted the real value of fine-tunability of ionic liquid structures, and both **b26** and **b27** were two fruitful TIL examples using the SPS-based discovery platform developed in this work.

**Figure 5:**
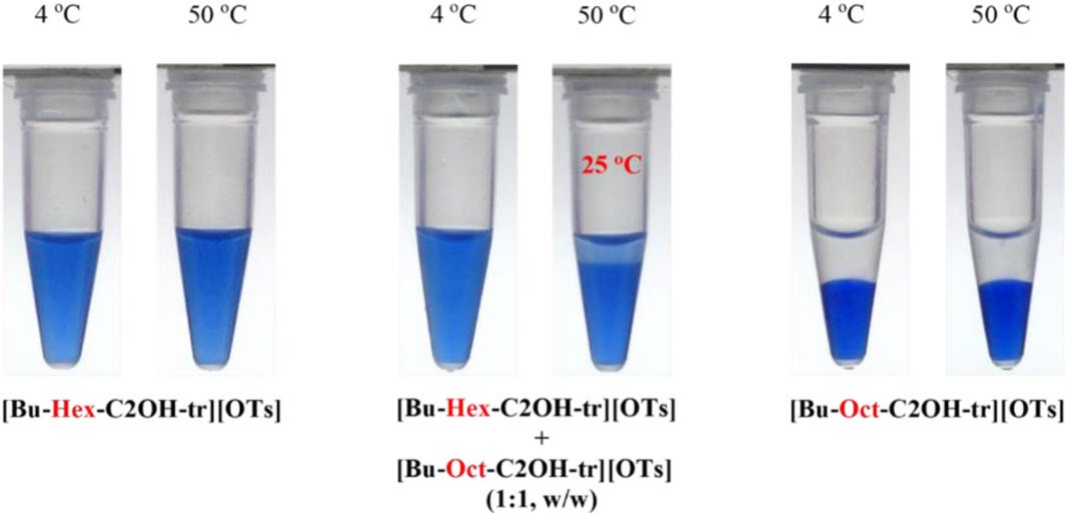
Temperature dependence (4 °C and 50 °C) of phase behavior of mixtures (2:1, w/w) of water with [Bu-Hex-C2OH-tr][OTs] (**a9**) alone, an equal mass mixture of [Bu-Hex-C2OH-tr][OTs] (**a9**) and [Bu-Oct-C2OH-tr][OTs] (**a10**) (1:1, w/w; *T*_*c*_ = 25 °C), and [Bu-Oct-C2OH-tr][OTs] (**a10**) alone, respectively.

We investigated further to test whether mixtures of a hydrophilic ionic liquid and a neighboring hydrophobic ionic liquid shown in Fig. 3 might possibly change their overall phase behavior in water^7^. As illustrated in Fig. 3A, the [Bu-Hex-C2OH-tr][OTs] (**a9**) is hydrophilic and homogeneous with water, but the [Bu-Oct-C2OH-tr][OTs] (**a10**) is hydrophobic and water immiscible, at temperatures between 2 °C and 90 °C; that is, both are not thermoresponsive toward temperature changes. Here, we demonstrated that a simple mixing of **a9** and **a10** with water is a convenient method to control total hydrophobicity toward phase transition and, for this example, an equal mass mixture of **a9** and **a10** in water readily formed a phase separation with a *T*_*c*_ at 25 °C (Fig. 5). In addition, this aqueous mixture returned to a homogeneous solution when cooled to 4 °C, confirming the successful development of a three-component LCST system. This SPS platform likely opens the possibility to a wide range of ionic salts for combinatorial discovery of new TIL pairs.

**Figure 6:**
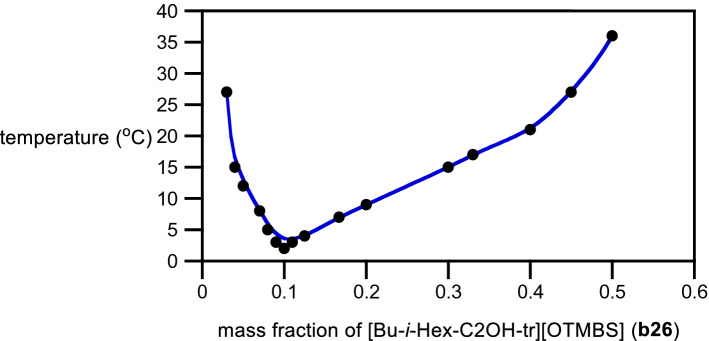
Phase diagram of a mixture of [Bu-*i*-Hex-C2OH-tr][OTMBS] (**b26**) and water. Solid line is a guide for the eye.

In a LCST system, the solubility of an ionic liquid in water decreases upon heating and its *T*_*c*_ depends on the mass fraction of ionic liquid in water. Figure 6 shows a thermally induced demixing of [Bu-*i*-Hex-C2OH-tr][OTMBS] (**b26**) with water. The phase separation temperature varies with TIL weight fraction in water and can be tuned between 90 °C and 2 °C. As expected for a LCST-phase behavior, its phase diagram is of a concave curve with the lowest *T*_*c*_ near its mass ratio of 1:9 (w/w) with water, confirming that **b26** is indeed a room-temperature TIL with the LCST phase behavior.

### Second library of 52 ionic liquids (c1–c26 and d1–d26)

Encouraged by the combinatorial discovery of new TILs from our first library of 57 ionic liquids (**a1**–**a28** and **b1**–**b29**), we decided to extend further our investigation to a second library: [R_2_-R_3_-C3OH-tr][OTs] (**c1**–**c26**) and [R_2_-R_3_-C3OH-tr][OTMBS] (**d1**–**d26**) (Fig. 1). The preparation of this library followed the similar synthetic route as depicted in Fig. 2. Figure S1 (ESI) summarized the synthesis of this library of 52 ionic liquids. In short, the overall isolated yields for these 4-step syntheses of **c1**–**c26** and **d1**–**d26** were moderate: 47–75% and 32–74%, respectively (Figure S1). For this library, four ionic liquids were found solid at room temperature (**d2**–**d5**, m.p. 65–71 °C) and the rest of 48 are all room-temperature ionic liquids. Detailed experimental procedures, NMR and HRMS spectra and data of 52 ionic liquids are provided in the Supporting Information (ESI-2).

**Figure 7:**
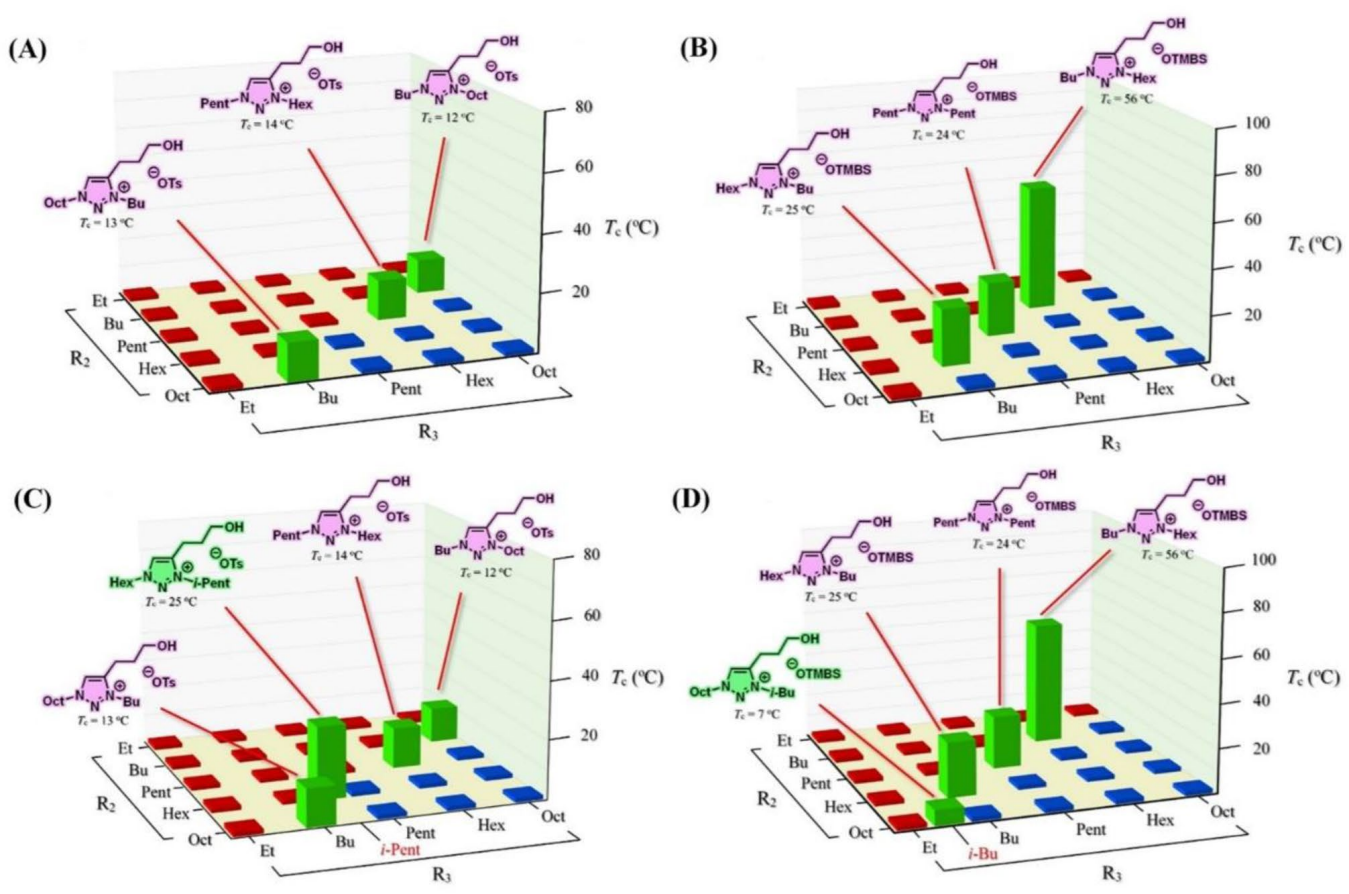
LCST Phase transitions of a library of 52 ionic liquids upon mixing with water (1:2, w/w) at temperatures between 2 °C and 90 °C: (**A**) ionic liquids **c1**–**c25**, (**B**) ionic liquids **d1**–**d25**, (**C**) ionic liquids **c1–c25** along with one additional ionic liquid **c26** having an isopentyl sidechain, and (**D**) ionic liquids **d1**–**d25** along with one additional ionic liquid **d26** with an isobutyl sidechain. In this library, 8 ionic liquids in total show phase transitions: **c10** ([Bu-Oct-C3OH-tr][OTs]; *T*_c_ = 12 °C), **c14** ([Pent-Hex-C3OH-tr][OTs]; *T*_c_ = 14 °C), **c22** ([Oct-Bu-C3OH-tr][OTs]; *T*_c_ = 13 °C), **c26** ([Hex-*i*-Pent-C3OH-tr][OTs]; *T*_c_ = 25 °C), **d9** ([Bu-Hex-C3OH-tr][OTMBS]; *T*_c_ = 56 °C), **d13** ([Pent-Pent-C3OH-tr][OTMBS]; *T*_c_ = 24 °C), **d17** ([Hex-Bu-C3OH-tr][OTMBS]; *T*_c_ = 25 °C), and **d26** ([Oct-*i*-Bu-C3OH-tr][OTMBS]; *T*_c_ = 7 °C).

Figure 7 shows screening results of 52 ionic liquids and their phase transitions toward temperature changes in water. As expected, the total number of hydrophobic ionic liquids found in [OTMBS]-based exceeded that of [OTs]-based ionic liquids (Fig. 7B vs. A). To our delight, eight room-temperature ionic liquids (**c10**, **c14**, **c22**, **c26**, **d9**, **d13**, **d17**, and **d26**), labeled in green, were found transporting LCST phase transitions (Fig. 7). Among these, two TILs carry branched sidechains at R_3_: [Hex-*i*-Pent-C3OH-tr][OTs] (**c26**, Fig. 7C) and [Oct-*i*-Bu-C3OH-tr][OTMBS] (**d26**, Fig. 7D). Furthermore, these eight TILs obtained were all identified to reside on the rim between being totally hydrophilic (red) and totally hydrophobic (blue) in Fig. 7. It is also of interest to note that, in this library, the ‘1,5-isomer’ of [Bu-Oct-C3OH-tr][OTs] (**c10**)—[Oct-Bu-C3OH-tr][OTs] (**c22**)—was found thermoresponsive toward temperature change in water (Fig. 7A). Remarkably, both **c10** and **c22** carry essentially identical *T*_*c*_ value (12 °C and 13 °C, respectively) with water. One additional ‘1,4- and 1,5-isomer’ pair of TILs—[Bu-Hex-C3OH-tr][OTMBS] (**d9**) and [Hex-Bu-C3OH-tr][OTMBS] (**d17**)—were identified in [OTMBS]-based library (Fig. 7B). All eight room-temperature TILs identified in our second library carry attractive low *T*_*c*_ phase transitions (7–56 °C) with water. Figure S2 shows a concave phase diagram of the representative [Bu-Hex-C3OH-tr][OTMBS] (**d9**) in water (ESI).

### Third library of 51 ionic liquids (e1–e25 and f1–f26)

To further prove the value and usefulness of the combinatorial platform developed in this work, we offered to expand our study to a third library of 51 ionic liquids: [R_2_-R_3_-C4OH-tr][OTs] (**e1**–**e25**) and [R_2_-R_3_-C4OH-tr][OTMBS] (**f1**–**f26**) (Fig. 1). The preparation of this library followed the similar synthetic approach highlighted in Fig. 2. The library synthesis is summarized in Figure S3 (ESI). The overall isolated yields for syntheses of **e1**–**e25** and **f1**–**f26** were good: 44–71% and 42–69%, respectively (ESI). For this library, only one ionic liquid was found solid at room temperature (**f2**, m.p. 66 °C) and the rest of 50 are room-temperature ionic liquids. Detailed experimental procedures, NMR and HRMS spectra and data of 51 ionic liquids are provided in the Supporting Information (ESI-3).

**Figure 8:**
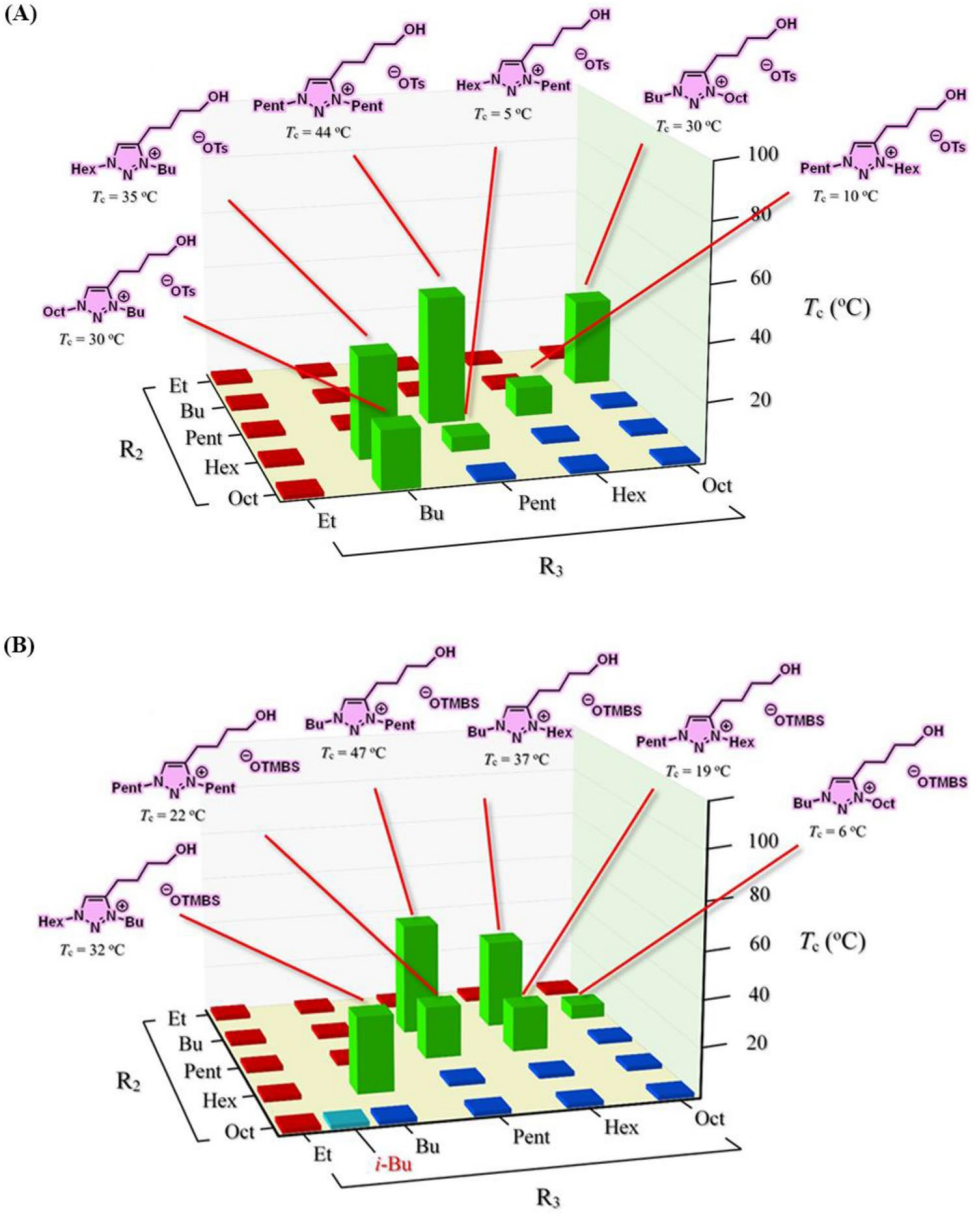
LCST Phase transitions of a library of 51 ionic liquids upon mixing with water (1:2, w/w) at temperatures between 2 °C and 90 °C: (**A**) ionic liquids **e1**–**e25** and (**B**) ionic liquids **f1**–**f26**. In this library, 12 ionic liquids in total show phase transitions: **e10** ([Bu-Oct-C4OH-tr][OTs]; *T*_c_ = 30 °C), **e13** ([Pent-Pent-C4OH-tr][OTs]; *T*_c_ = 44 °C), **e14** ([Pent-Hex-C4OH-tr][OTs]; *T*_c_ = 10 °C), **e17** ([Hex-Bu-C4OH-tr][OTs]; *T*_c_ = 35 °C), **e18** ([Hex-Pent-C4OH-tr][OTs]; *T*_c_ = 5 °C), **e22** ([Oct-Bu-C4OH-tr][OTs]; *T*_c_ = 30 °C), **f8** ([Bu-Pent-C4OH-tr][OTMBS]; *T*_c_ = 47 °C), **f9** ([Bu-Hex-C4OH-tr][OTMBS]; *T*_c_ = 37 °C), **f10** ([Bu-Oct-C4OH-tr][OTMBS]; *T*_c_ = 6 °C), **f13** ([Pent-Pent-C4OH-tr][OTMBS]; *T*_c_ = 22 °C), **f14** ([Pent-Hex-C4OH-tr][OTMBS]; *T*_c_ = 19 °C), and **f17** ([Hex-Bu-C4OH-tr][OTMBS]; *T*_c_ = 32 °C).

Figure 8 presents the screening results from a library of 51 ionic liquids and their phase transitions with water on temperature changes. We were pleased that, in this library, 12 room-temperature ionic liquids (**e10**, **e13**, **e14**, **e17**, **e18**, **e22**, **f8**, **f9**, **f10**, **f13**, **f14**, and **f17**), labeled in green, were discovered exhibiting LCST phase transitions with six each from [OTs]- and [OTMBS]-based ionic liquids (Fig. 8A,B, respectively). Again, these 12 TILs identified were all situated on the rim between being totally hydrophilic (red) and totally hydrophobic (blue) in Fig. 8. It is also noted that three ‘1,4- and 1,5-isomer’ pairs of ionic liquids were found thermoresponsive toward temperature change in water: **e10** and **e22**; **e14** and **e18**; **f9** and **f17**. All 12 TILs obtained in third library carry attractive low *T*_*c*_ phase transitions (5–47 °C) with water. The phase diagram of a representative [Hex-Bu-C4OH-tr][OTMBS] (**e17**) is shown in Figure S4 (ESI), in which the reversible LCST is only observed at mass fraction between 10 and 40% in water.

## Conclusion

In summary, we reported in this work the development of a SPS platform for the successful discovery of new small-molecule 1,2,3-triazolium TILs, which unambiguously illustrated and highlighted the value and structure tunability of ionic liquids (e.g., discovery of **b26** from **b8** and **b9**, Fig. 4). This work also demonstrated that, among three libraries of 160 ionic liquids prepared, 22 room-temperature TILs obtained are eminently capable of performing LCST phase transitions with low *T*_*c*_ values in water; that is, a 14% successful rate in library screening. Albeit totally preliminary, it is worth noting that most, if not all, compounds with LCST property identified from three ionic liquid libraries had a common combined carbon count of 11 ± 1 at alkyl (R_2_ and R_3_) appendage: 10 (Fig. 3), 10–12 (Fig. 7), and 10–12 (with an exception of 9 for the ionic liquid **f8**, Fig. 8). Reagents used in chemical synthesis of ionic liquid libraries were all from inexpensive aliphatic alcohols (i.e., R_1_OH, R_2_OH, and R_3_OH, Fig. 1) and the synthesis of 1,2,3-triazolium ionic liquids involved a key CuAAC click reaction with good overall isolated yields. Click chemistry-based combinatorial approaches permit highly effective synthesis of a myriad of 1,2,3-triazole compounds simultaneously and have been important tools in many areas of research, including discovery of small-molecule enzyme inhibitors and synthesis of ionic liquids^14–20^. To our knowledge, this is the first comprehensive and systematic study of small-molecule triazolium ionic liquids that exhibit thermoresponsive LCST property in water. The results presented in this work hold compelling possibilities of the use of TILs as functional materials for advancing affinity extractions, biomolecule separations and bio-catalysis aiming at target analytes for a range of new applications. The study of biomolecules with TILs in aqueous solution is being actively pursued in this laboratory, and the result will be reported in due course.

## Methods

### Combinatorial synthesis of three libraries of 160 ionic liquids: [R_2_-R_3_-C2OH-tr][OTs] (a1–a28), [R_2_-R_3_-C2OH-tr][OTMBS] (b1–b29), [R_2_-R_3_-C3OH-tr][OTs] (c1–c26), [R_2_-R_3_-C3OH-tr][OTMBS] (d1–d26), [R_2_-R_3_-C4OH-tr][OTs] (e1–e25) and [R_2_-R_3_-C4OH-tr][OTMBS] (f1–f26)

Detailed synthetic procedures and spectral characterization (^1^H- and ^13^C-NMR, and HRMS) of all 160 ionic liquids were given in the Supporting Information (ESI-1 for **a1–a28** and **b1–b29**, ESI-2 for **c1–c26** and **d1–d26**, and ESI-3 for **e1–e25** and **f1–f26**). For all 160 ionic liquids synthesized, 155 are liquid at room temperature.

For spectral characterization of ionic liquids, [Bu-*i*-Hex-C2OH-tr][OTMBS] (**b26**) was used as a representative room-temperature ionic liquid: 93% yield, light yellow liquid; ^1^H NMR (400 MHz, CDCl_3_) δ 0.89 (d, *J* = 6.0 Hz, 2 × CHC*H*_*3*_, 6H), 0.95 (t, *J* = 8.0 Hz, CH_3_, 3H), 1.18–1.25 (m, CH_2_, 2H), 1.31**–**1.40 (m, CH_2_, 2H), 1.53**–**1.63 (m, C*H*CH_3_, 1H), 1.89**–**2.01 (m, 2 × NCH_2_C*H*_*2*_ , 4H), 2.22 (s, *p*-CH_3_, 3H), 2.63 (s, 2 × *o*-CH_3_, 6H), 3.07 (t, *J* = 6.0 Hz, CH=CC*H*_2_, 2H), 4.05 (t, *J* = 6.0 Hz, CH_2_C*H*_2_OH, 2H), 4.44 (t, *J* = 8.0 Hz, CNC*H*_2,_ 2H), 4.60 (t, *J* = 6.0 Hz, CHNC*H*_2,_ 2H), 6.81 (s, aryl H, 2H), 9.19 (s, C*H*=CCH_2_, 1H); ^13^C NMR (100 MHz, CDCl_3_) δ 13.47, 19.63, 20.93, 22.47, 23.15, 27.03, 27.11, 27.68, 31.24, 35.37, 51.53, 53.86, 58.70, 130.38, 130.79, 137.14, 138.44, 140.25, 142.67; EI-HRMS *m/z* [M^+^] calcd for C_14_H_28_N_3_O 254.2232, found 254.2226.

## Supplementary information

Supplementary Information 1.

Supplementary Information 2.

Supplementary Information 3.

Supplementary Information 4.
