# Supplementary material for: Combinatorial discovery of small-molecule 1,2,3-triazolium ionic liquids exhibiting lower critical solution temperature phase transition
Source: Sci Rep. 2020 Oct 26;10:18247. doi: 10.1038/s41598-020-75392-z (PMC7589527; doi:10.1038/s41598-020-75392-z)
Supplement: Supplementary file 1 — Supplementary Information 1. [file 41598_2020_75392_MOESM1_ESM.pdf]

Supporting Information (ESI): Figure S1, Figure S2, Figure S3, Figure S4.

Combinatorial Discovery of Small-Molecule 1,2,3-Triazolium Ionic Liquids Exhibiting Lower Critical Solution Temperature Phase Transition

Yen-Ho Chu,* Mou-Fu Cheng and Yung-Hsin Chiang

Department of Chemistry and Biochemistry, National Chung Cheng University, Chiayi 62102, Taiwan, Republic of China

* Corresponding author. Tel: 886 52729139; fax: 886 52721040; e-mail: cheyhc@ccu.edu.tw

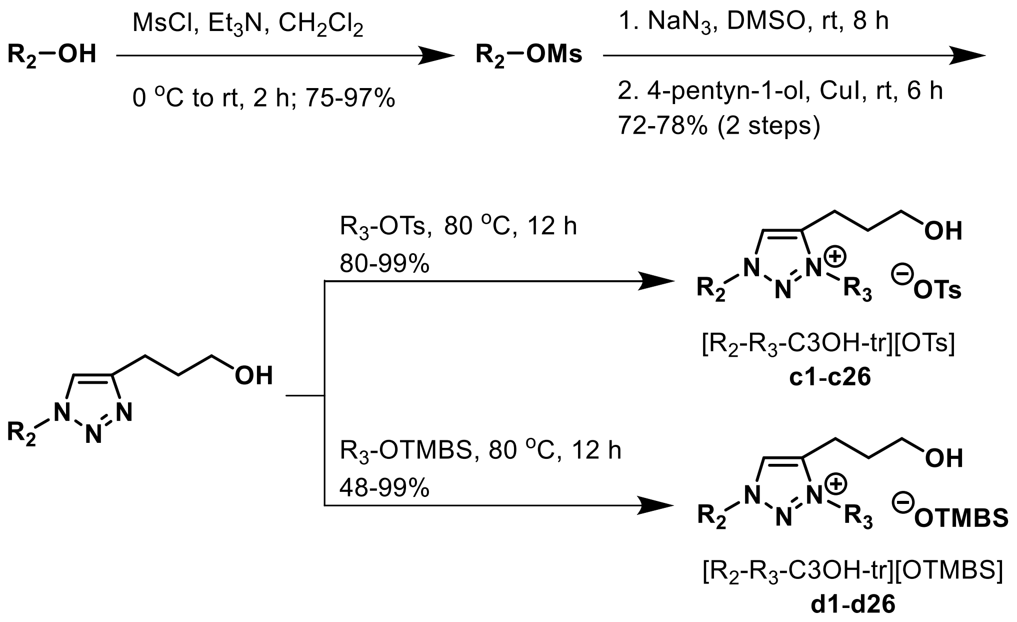

Figure S1. Synthesis of thermoresponsive ionic liquids, **c1-c26** and **d1-d26**.

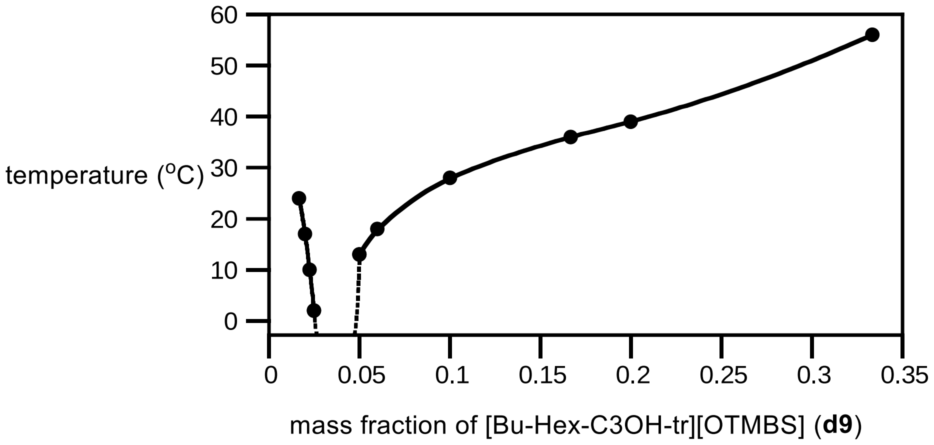

Figure S2. Phase diagram of a mixture of [Bu-Hex-C3OH-tr][OTMBS] (**d9**) and water. Solid line is a guide for the eye. Dashed line indicates limit of experimental measurements due to the freezing temperature of water.

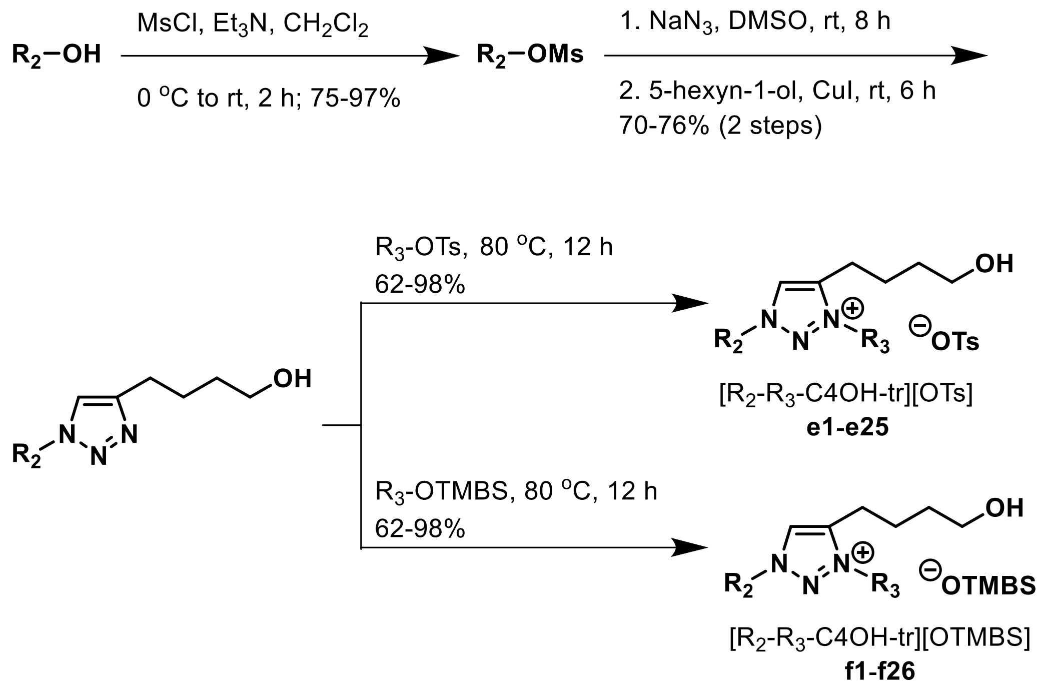

Figure S3. Synthesis of thermoresponsive ionic liquids, **e1-e25** and **f1-f26**.

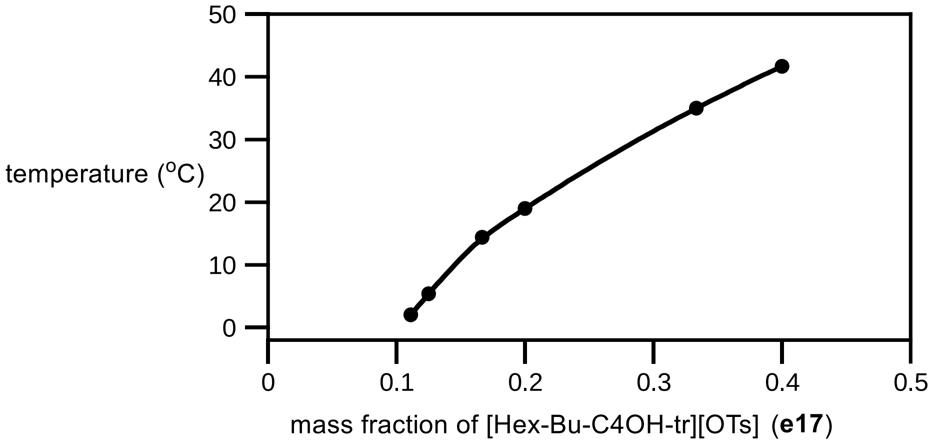

Figure S4. Phase diagram of a mixture of [Hex-Bu-C4OH-tr][OTMBS] (**e17**) and water. The reversible LCST is only observed at mass fraction between 10% and 40% in water. Solid line is a guide for the eye. In this system of **e17**, the experimental observation of a complete bowl-shaped LCST curve was limited by the freezing temperature of water.
