# Supplementary material for: Combinatorial discovery of small-molecule 1,2,3-triazolium ionic liquids exhibiting lower critical solution temperature phase transition
Source: Sci Rep. 2020 Oct 26;10:18247. doi: 10.1038/s41598-020-75392-z (PMC7589527; doi:10.1038/s41598-020-75392-z)
Supplement: Supplementary file 2 — Supplementary Information 2. [file 41598_2020_75392_MOESM2_ESM.pdf]

Supporting Information (ESI-1) for [R_2-R_3-C2OH-tr][OTs] (**a1**-**a28**) and [R_2-R_3-C2OH-tr][OTMBS] (**b1**-**b29**) Room-Temperature Ionic Liquids:

Combinatorial Discovery of Small-Molecule 1,2,3-Triazolium Ionic Liquids Exhibiting Lower Critical Solution Temperature Phase Transition

Yen-Ho Chu*, Mou-Fu Cheng and Yung-Hsin Chiang

* Corresponding author. E-mail: cheyhc@ccu.edu.tw

Department of Chemistry and Biochemistry, National Chung Cheng University, Chiayi 62102, Taiwan, Republic of China

Combinatorial Synthesis of a Library of 28 [R_2-R_3-C2OH-tr][OTs] Room-Temperature Ionic Liquids

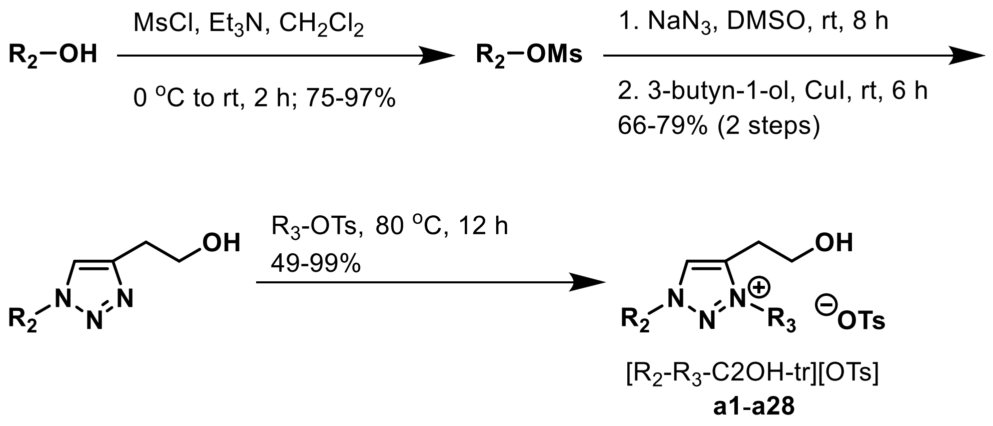

Generalized Procedure for the Synthesis of Alkyl Methanesulfonates

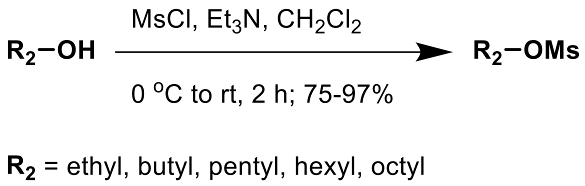

To a round-bottomed flask containing an alcohol (R_2OH, 54 mmol) in dichloromethane (0.35 M) was added triethylamine (108 mmol, 2 equiv) and homogeneously stirred in ice bath. The mixture was added MsCl (64 mmol, 1.2 equiv) slowly in ice bath for 30 min, then the reaction was proceeded at room temperature for another 1.5 h. The progress of reaction could be readily monitored by TLC. After reaction completed, the resulting solution remained colorless. It was then extracted with 10 wt% citric acid (three times) and 10 wt% sodium bicarbonate (three times). The organic layer was collected, dried with anhydrous sodium sulfate, filtered, and concentrated to afford alkyl methanesulfonate (R_2OMs).

Ethyl methanesulfonate (Et-OMs): 75% yield, colorless liquid; ^{1}H NMR (400 MHz, $CDCl_3$) δ 1.42 (t, *J* = 8.0 Hz, CH_2CH_3, 3H), 3.01 (s, CH_3, 3H), 4.31 (q, *J* = 8.0 Hz, OCH_2, 2H).

Butyl methanesulfonate (Bu-OMs): 97% yield, colorless liquid; ^{1}H NMR (400 MHz, $CDCl_3$) δ 0.96 (t, *J* = 8.0 Hz, CH_2CH_3, 3H), 1.42-1.49 (m, CH_2CH_3, 2H), 1.70-1.77

(m, CH_2CH$_2$CH$_3$, 2H), 3.01 (s, CH$_3$, 3H), 4.24 (t, J = 6.0 Hz, OCH_2, 2H).

Pentyl methanesulfonate (Pent-OMs): 92% yield, colorless liquid; ^{1}H NMR (400 MHz, CDCl$_3$) δ 0.92 (t, J = 8.0 Hz, CH$_2$CH_3, 3H), 1.34-1.40 (m, 2 x CH$_2$, 4H), 1.72-1.79 (m, OCH$_2$CH_2, 2H), 3.00 (s, CH$_3$, 3H), 4.23 (t, J = 6.0 Hz, OCH_2, 2H).

Hexyl methanesulfonate (Hex-OMs): 93% yield, colorless liquid; ^{1}H NMR (400 MHz, CDCl$_3$) δ 0.90 (t, J = 8.0 Hz, CH$_2$CH_3, 3H), 1.29-1.44 (m, 3 x CH$_2$, 6H), 1.71-1.78 (m, OCH$_2$CH_2, 2H), 3.01 (s, CH$_3$, 3H), 4.23 (t, J = 6.0 Hz, OCH_2, 2H).

Octyl methanesulfonate (Oct-OMs): 96% yield, colorless liquid; ^{1}H NMR (400 MHz, CDCl$_3$) δ 0.88 (t, J = 6.0 Hz, CH$_2$CH_3, 3H), 1.28-1.41 (m, 5 x CH$_2$, 10H), 1.71-1.78 (m, OCH$_2$CH_2, 2H), 3.00 (s, CH$_3$, 3H), 4.22 (t, J = 6.0 Hz, OCH_2, 2H).

Generalized Procedure for the Synthesis of 1,4-Disubstituted 1,2,3-Triazoles (R_2-C2OH-tr)

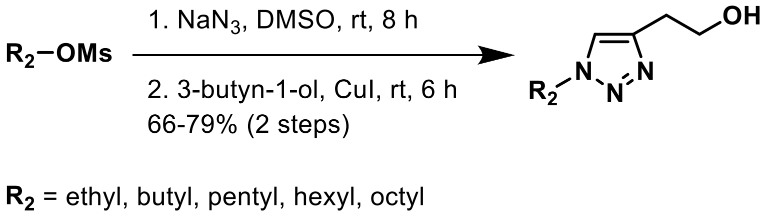

To a round-bottomed flask containing alkyl methanesulfonate (R_2-OMs, 4.6 mmol) in dimethyl sulfoxide (1.0 M) was added sodium azide (9.3 mmol, 2 equiv) and the

reaction was allowed to proceed at room temperature for 8 h. The progress of reaction could be monitored by TLC. The resulting reaction mixture was then added copper(I) iodide (0.5 mmol, 0.1 equiv) and 3-butyn-1-ol (5.5 mmol 1.2 equiv). The subsequent [3+2] cycloaddition reaction was proceeded for 6 h at room temperature. The progress of reaction again was readily monitored by TLC. After reaction completed, crude product was filtered and concentrated under reduced pressure. The residue was purified by column chromatography (ethyl acetate/hexane = 1:5 to 1:0) to finally afford the desired R_2-C2OH-tr.

Et-C2OH-tr: 71% yield, light yellow liquid; ^{1}H NMR (400 MHz, $CDCl_3$) δ 1.55 (t, J = 6.0 Hz, NCH_2CH_3, 3H), 2.76 (s, OH, 1H), 2.95 (t, J = 6.0 Hz, CH=CCH_2, 2H), 3.95 (q, J = 6.0 Hz, CH_2CH_2OH, 2H), 4.39 (q, J = 6.0 Hz, NCH_2, 2H), 7.40 (s, CH=CCH_2, 1H).

Bu-C2OH-tr: 66% yield, light yellow liquid; ^{1}H NMR (400 MHz, $CDCl_3$) δ 0.96 (t, J = 8.0 Hz, CH_2CH_2CH_3, 3H), 1.32-1.41 (m, CH_2C$H_2$$CH_3$, 2H), 1.85-1.91 (m, C$H_2$$CH_2CH_3$, 2H), 2.63 (t, J = 6.0 Hz, OH, 1H), 2.95 (t, J = 6.0 Hz, CH=CCH_2, 2H), 3.95 (t, J = 6.0 Hz, CH_2CH_2OH, 2H), 4.34 (t, J = 6.0 Hz, NCH_2, 2H), 7.37 (s, CH=CCH_2, 1H).

Pent-C2OH-tr: 79% yield, light yellow liquid; ^{1}H NMR (400 MHz, $CDCl_3$) δ 0.90 (t, J = 6.0 Hz, CH_2CH_2CH_3, 3H), 1.31-1.39 (m, 2 x CH_2, 4H), 1.86-1.94 (m, NCH_2CH_2,

2H), 2.76-2.77 (m, O*H*, 1H), 2.95 (t, *J* = 6.0 Hz, CH=CC*H*$_2$, 2H), 3.95 (t, *J* = 6.0 Hz, CH_2C*H*$_2$OH, 2H), 4.32 (t, *J* = 8.0 Hz, N*CH*$_2$, 2H), 7.38 (s, C*H*=CCH_2, 1H).

Hex-C2OH-tr: 67% yield, light yellow liquid; ^{1}H NMR (400 MHz, $CDCl_3$) δ 0.88 (t, *J* = 6.0 Hz, CH_2CH_2C*H*$_3$, 3H), 1.32 (m, 3 x CH_2, 6H), 1.88-1.91 (m, NCH_2C*H*$_2$, 2H), 2.60-2.63 (t, *J* = 6.0 Hz, O*H*, 1H), 2.95 (t, *J* = 6.0 Hz, CH=CC*H*$_2$, 2H), 3.96 (t, *J* = 6.0 Hz, CH_2C*H*$_2$OH, 2H), 4.32 (t, *J* = 8.0 Hz, N*CH*$_2$, 2H), 7.37 (s, C*H*=CCH_2, 1H).

Oct-C2OH-tr: 79% yield, light yellow solid; ^{1}H NMR (400 MHz, $CDCl_3$) δ 0.88 (t, *J* = 6.0 Hz, CH_2CH_2C*H*$_3$, 3H), 1.26-1.32 (m, 5 x CH_2 10H), 1.86-1.93 (m, NCH_2C*H*$_2$, 2H), 2.61 (s, O*H*, 1H), 2.95 (t, *J* = 4.0 Hz, CH=CC*H*$_2$, 2H), 3.96 (m, CH_2C*H*$_2$OH, 2H), 4.32 (t, *J* = 8.0 Hz, N*CH*$_2$, 2H), 7.36 (s, C*H*=CCH_2, 1H).

Generalized Procedure for the Synthesis of [R$_2$-R$_3$-C2OH-tr][OTs] Ionic Liquids

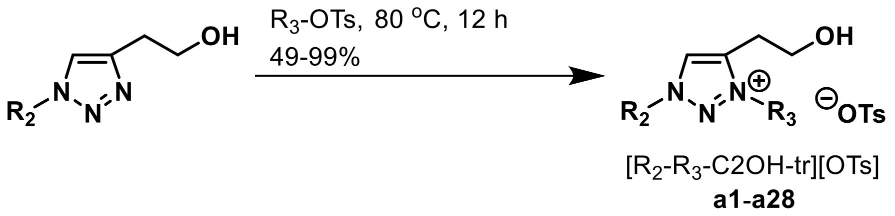

R_3 = ethyl, butyl, isobutyl, pentyl, isopentyl, hexyl, isohexyl, octyl

To a round-bottomed flask containing R$_2$-C2OH-tr (0.5 mmol) was added alkyl 4-methylbenzenesulfonate (R$_3$-OTs, 0.6 mmol, 1.3 equiv). The mixture was heated at

80 ^{o}C for 12 h. The progress of the ionic liquid forming reaction could be readily monitored by TLC. After reaction was completed, the reaction mixture was washed with ether (3 mL, 3 times) and then with *n*-hexane (3 mL, 3 times) to finally afford the desired [R_2-R_3-C2OH-tr][OTs] as room-temperature ionic liquids (**a1-a28**).

[Et-Et-C2OH-tr][OTs] (a1): 99% yield, light yellow liquid; ^{1}H NMR (400 MHz, $CDCl_3$) δ 1.57-1.65 (m, 2 x CH_3, 6H), 2.34 (s, *p*-CH_3, 3H), 3.10 (t, *J* = 6.0 Hz, CH=CCH_2, 2H), 4.03 (t, *J* = 6.0 Hz, CH_2CH_2OH, 2H), 4.54 (q, *J* = 8.0 Hz, CNC$H_2$$CH_3$, 2H), 4.65 (q, *J* = 8.0 Hz, CHNC$H_2$$CH_3$, 2H), 7.14 (d, *J* = 8.0 Hz, aryl H, 2H), 7.72 (d, *J* = 8.0 Hz, aryl H, 2H), 9.01 (s, C*H*=CCH_2, 1H); ^{13}C NMR (100 MHz, $CDCl_3$) δ 14.14, 14.31, 21.44, 26.94, 46.77, 49.37, 59.10, 126.01, 128.89, 129.62, 140.00, 142.51, 142.71; EI-HRMS *m/z* [M^+] calcd for $C_8H_{16}N_3O$ 170.1293, found 170.1284.

[Et-Bu-C2OH-tr][OTs] (a2): 98% yield, light yellow liquid; ^{1}H NMR (400 MHz, $CDCl_3$) δ 0.96 (t, *J* = 8.0 Hz, CH_2CH_2CH_3, 3H), 1.33-1.43 (m, CH_2C$H_2$$CH_3$, 2H), 1.63 (t, *J* = 8.0 Hz, NCH_2CH_3, 3H), 1.87-1.95 (m, C$H_2$$CH_2CH_3$, 2H), 2.34 (s, *p*-$CH_3$, 3H), 3.08 (t, *J* = 6.0 Hz, CH=CCH_2, 2H), 4.04 (t, *J* = 6.0 Hz, CH_2CH_2OH, 2H), 4.46 (t, *J* = 8.0 Hz, NC$H_2$$CH_2$, 2H), 4.64 (q, *J* = 8.0 Hz, NC$H_2$$CH_3$, 2H), 7.14 (d, *J* = 8.0 Hz, aryl H, 2H), 7.71 (d, *J* = 8.0 Hz, aryl H, 2H), 9.02 (s, C*H*=CCH_2, 1H); ^{13}C NMR (100 MHz, $CDCl_3$) δ 13.51, 14.30, 19.67, 21.45, 27.00, 30.91, 49.39, 51.08, 59.08, 126.08,

128.92, 129.65, 140.25, 142.13, 142.59; EI-HRMS *m/z* [M^+] calcd for $C_{10}H_{20}N_3O$ 198.1606, found 198.1595.

[Et-Pent-C2OH-tr][OTs] (a3): 92% yield, light yellow liquid; ^1H NMR (400 MHz, $CDCl_3$) δ 0.91 (t, *J* = 8.0 Hz, $CH_2CH_2CH_3$, 3H), 1.31-1.37 (m, 2 x CH_2, 4H), 1.65 (t, *J* = 8.0 Hz, NCH_2CH_3, 3H), 1.90-1.98 (m, NCH_2CH_2, 2H), 2.34 (s, *p*-CH_3, 3H), 3.07 (t, *J* = 6.0 Hz, CH=CCH_2, 2H), 4.05 (t, *J* = 6.0 Hz, CH_2CH_2OH, 2H), 4.46 (t, *J* = 8.0 Hz, NCH_2CH_2, 2H), 4.67 (q, *J* = 8.0 Hz, NCH_2CH_3, 2H), 7.14 (d, *J* = 8.0 Hz, aryl H, 2H), 7.73 (d, *J* = 8.0 Hz, aryl H, 2H), 9.10 (s, C*H*=CCH_2, 1H); ^{13}C NMR (100 MHz, $CDCl_3$) δ 13.91, 14.38, 21.46, 22.15, 27.11, 28.48, 28.76, 49.46, 51.33, 58.99, 126.06, 128.86, 129.89, 139.88, 142.62, 142.91; EI-HRMS *m/z* [M^+] calcd for $C_{11}H_{22}N_3O$ 212.1763, found 212.1751.

[Et-Hex-C2OH-tr][OTs] (a4): 92% yield, light yellow liquid; ^1H NMR (400 MHz, $CDCl_3$) δ 0.89 (t, *J* = 6.0 Hz, $CH_2CH_2CH_3$, 3H), 1.28-1.36 (m, 3 x CH_2, 6H), 1.65 (t, *J* = 8.0 Hz, NCH_2CH_3, 3H), 1.89-1.96 (m, NCH_2CH_2, 2H), 2.34 (s, *p*-CH_3, 3H), 3.07 (t, *J* = 6.0 Hz, CH=CCH_2, 2H), 4.05 (t, *J* = 6.0 Hz, CH_2CH_2OH, 2H), 4.45 (t, *J* = 6.0 Hz, NCH_2CH_2, 2H), 4.66 (q, *J* = 8.0 Hz, NCH_2CH_3, 2H), 7.14 (d, *J* = 8.0 Hz, aryl H, 2H), 7.73 (d, *J* = 8.0 Hz, aryl H, 2H), 9.09 (s, C*H*=CCH_2, 1H); ^{13}C NMR (100 MHz, $CDCl_3$) δ 14.05, 14.37, 21.46, 22.49, 26.09, 27.11, 29.02, 31.15, 49.44, 51.34, 58.99,

126.05, 128.85, 129.89, 139.85, 142.62, 142.93; EI-HRMS *m/z* [M^+] calcd for $C_{12}H_{24}N_3O$ 226.1919, found 226.1909.

[Et-Oct-C2OH-tr][OTs] (a5): 82% yield, light yellow liquid; ^{1}H NMR (400 MHz, $CDCl_3$) δ 0.88 (t, *J* = 6.0 Hz, $CH_2CH_2CH_3$, 3H), 1.26-1.34 (m, 5 x CH_2, 10H), 1.66 (t, *J* = 8.0 Hz, NCH_2CH_3, 3H), 1.91-1.98 (m, NCH_2CH_2, 2H), 2.34 (s, *p*-CH_3, 3H), 3.07 (t, *J* = 6.0 Hz, CH=CCH_2, 2H), 4.07 (t, *J* = 6.0 Hz, CH_2CH_2OH, 2H), 4.46 (t, *J* = 8.0 Hz, NCH_2CH_2, 2H), 4.68 (q, *J* = 8.0 Hz, NCH_2CH_3, 2H), 7.14 (d, *J* = 8.0 Hz, aryl H, 2H), 7.73 (d, *J* = 8.0 Hz, aryl H, 2H), 9.09 (s, C*H*=CCH_2, 1H); ^{13}C NMR (100 MHz, $CDCl_3$) δ 14.21, 14.35, 21.46, 22.73, 26.43, 27.07, 29.00, 29.05, 29.12, 31.82, 49.44, 51.33, 59.00, 126.07, 128.88, 129.84, 139.98, 142.58, 142.66; EI-HRMS *m/z* [M^+] calcd for $C_{14}H_{28}N_3O$ 254.2232, found 254.2221.

[Bu-Et-C2OH-tr][OTs] (a6): 98% yield, light yellow liquid; ^{1}H NMR (400 MHz, $CDCl_3$) δ 0.93 (t, *J* = 6.0 Hz, $CH_2CH_2CH_3$, 3H), 1.30-1.40 (m, $CH_2CH_2CH_3$, 2H), 1.59 (t, *J* = 8.0 Hz, NCH_2CH_3, 3H), 1.91-1.98 (m, $CH_2CH_2CH_3$, 2H), 2.34 (s, *p*-CH_3, 3H), 3.11 (t, *J* = 6.0 Hz, CH=CCH_2, 2H), 4.03 (t, *J* = 6.0 Hz, CH_2CH_2OH, 2H), 4.52-4.57 (m, NCH_2CH_2 + NCH_2CH_3, 4H), 7.14 (d, *J* = 8.0 Hz, aryl H, 2H), 7.71 (d, *J* = 8.0 Hz, aryl H, 2H), 8.95 (s, C*H*=CCH_2, 1H); ^{13}C NMR (100 MHz, $CDCl_3$) δ 13.47, 14.18, 19.62, 21.48, 26.93, 31.16, 46.80, 53.80, 59.06, 126.09, 128.96, 129.95, 140.21, 142.33, 142.50; EI-HRMS *m/z* [M^+] calcd for $C_{10}H_{20}N_3O$ 198.1606, found 198.1595.

[Bu-Bu-C2OH-tr][OTs] (a7): 90% yield, light yellow liquid; ^{1}H NMR (400 MHz, $CDCl_3$) δ 0.93-0.99 (m, 2 x CH_3, 6H), 1.33-1.41 (m, 2 x C*H*$_2$$CH_3$, 4H), 1.88-2.00 (m, 2 x NCH_2C*H*$_2$, 4H), 2.34 (s, *p*-CH_3, 3H), 3.09 (t, *J* = 6.0 Hz, CH=CC*H*$_2$, 2H), 4.06 (t, *J* = 6.0 Hz, CH_2C*H*$_2$OH, 2H), 4.48 (t, *J* = 8.0 Hz, CNC*H*$_2$, 2H), 4.59 (t, *J* = 8.0 Hz, CHNC*H*$_2$, 2H), 7.14 (d, *J* = 8.0 Hz, aryl H, 2H), 7.72 (d, *J* = 8.0 Hz, aryl H, 2H), 9.04 (s, C*H*=CCH_2, 1H); ^{13}C NMR (100 MHz, $CDCl_3$) δ 13.46, 13.54, 19.60, 19.67, 21.47, 27.05, 30.97, 31.18, 51.09, 53.81, 58.97, 126.10, 128.89, 130.13, 139.98, 142.62, 142.69; EI-HRMS *m/z* [M^+] calcd for $C_{12}H_{24}N_3O$ 226.1919, found 226.1913.

[Bu-Pent-C2OH-tr][OTs] (a8): 88% yield, light yellow liquid; ^{1}H NMR (400 MHz, $CDCl_3$) δ 0.91 (t, *J* = 6.0 Hz, CH_3, 3H), 0.95 (t, *J* = 8.0 Hz, CH_3, 3H), 1.31-1.40 (m, 3 x CH_2, 6H), 1.92-2.02 (m, 2 x NCH_2C*H*$_2$, 4H), 2.34 (s, *p*-CH_3, 3H), 3.07 (t, *J* = 6.0 Hz, CH=CC*H*$_2$, 2H), 4.07 (t, *J* = 6.0 Hz, CH_2C*H*$_2$OH, 2H), 4.47 (t, *J* = 8.0 Hz, CNC*H*$_2$, 2H), 4.61 (t, *J* = 8.0 Hz, CHNC*H*$_2$, 2H), 7.14 (d, *J* = 8.0 Hz, aryl H, 2H), 7.74 (d, *J* = 8.0 Hz, aryl H, 2H), 9.07 (s, C*H*=CCH_2, 1H); ^{13}C NMR (100 MHz, $CDCl_3$) δ 13.42, 13.87, 19.55, 21.42, 22.10, 27.04, 28.40, 28.69, 31.16, 51.30, 53.71, 59.04, 126.03, 128.81, 130.05, 139.82, 142.69, 142.93; EI-HRMS *m/z* [M^+] calcd for $C_{13}H_{26}N_3O$ 240.2076, found 240.2067.

[Bu-Hex-C2OH-tr][OTs] (a9): 98% yield, light yellow liquid; ^{1}H NMR (400 MHz, $CDCl_3$) δ 0.88 (t, *J* = 6.0 Hz, CH_3, 3H), 0.93 (t, *J* = 8.0 Hz, CH_3, 3H), 1.31-1.37 (m, 4

x CH_2, 8H), 1.89-1.99 (m, 2 x NCH_2CH_2, 4H), 2.34 (s, *p*-CH_3, 3H), 3.08 (t, *J* = 6.0 Hz, CH=CCH_2, 2H), 4.05 (t, *J* = 6.0 Hz, CH_2CH_2OH, 2H), 4.46 (t, *J* = 6.0 Hz, $CNCH_2$, 2H), 4.58 (t, *J* = 8.0 Hz, $CHNCH_2$, 2H), 7.14 (d, *J* = 8.0 Hz, aryl H, 2H), 7.72 (d, *J* = 8.0 Hz, aryl H, 2H), 9.01 (s, CH=CCH_2, 1H); ^{13}C NMR (100 MHz, $CDCl_3$) δ 13.45, 14.05, 19.59, 21.48, 22.50, 26.07, 27.04, 28.99, 31.15, 31.17, 51.34, 53.80, 59.00, 126.14, 128.92, 130.11, 140.17, 142.30, 142.60; EI-HRMS *m/z* [M^+] calcd for $C_{14}H_{28}N_3O$ 254.2232, found 254.2222.

[Bu-Oct-C2OH-tr][OTs] (a10): 49% yield, light yellow liquid; ^1H NMR (400 MHz, $CDCl_3$) δ 0.88 (t, *J* = 8.0 Hz, CH_3, 3H), 0.95 (t, *J* = 8.0 Hz, CH_3, 3H), 1.31-1.37 (m, 6 x CH_2, 12H), 1.92-2.02 (m, 2 x NCH_2CH_2, 4H), 2.34 (s, *p*-CH_3, 3H), 3.08 (t, *J* = 6.0 Hz, CH=CCH_2, 2H), 4.06 (t, *J* = 6.0 Hz, CH_2CH_2OH, 2H), 4.46 (t, *J* = 6.0 Hz, $CNCH_2$, 2H), 4.58 (t, *J* = 8.0 Hz, $CHNCH_2$, 2H), 7.14 (d, *J* = 8.0 Hz, aryl H, 2H), 7.73 (d, *J* = 8.0 Hz, aryl H, 2H), 9.07 (s, CH=CCH_2, 1H); ^{13}C NMR (100 MHz, $CDCl_3$) δ 13.46, 14.22, 19.60, 21.47, 22.74, 26.41, 27.05, 29.00, 29.04, 29.14, 31.19, 31.81, 51.33, 53.80, 58.95, 126.08, 128.89, 130.15, 139.96, 142.58, 142.71; EI-HRMS *m/z* [M^+] calcd for $C_{16}H_{32}N_3O$ 282.2545, found 282.2541.

[Pent-Et-C2OH-tr][OTs] (a11): 97% yield, light yellow liquid; ^1H NMR (400 MHz, $CDCl_3$) δ 0.88 (t, *J* = 6.0 Hz, CH_3, 3H), 1.27-1.36 (m, 2 x CH_2, 4H), 1.60 (t, *J* = 8.0 Hz, CH_3, 3H), 1.94-2.01 (m, 2 x NCH_2CH_2, 4H), 2.33 (s, *p*-CH_3, 3H), 3.11 (t, *J* = 6.0

Hz, CH=CCH_2, 2H), 4.05 (t, J = 6.0 Hz, CH_2CH_2OH, 2H), 4.54-4.57 (m, 2 x NCH_2, 4H), 7.14 (d, J = 8.0 Hz, aryl H, 2H), 7.71 (d, J = 8.0 Hz, aryl H, 2H), 8.96 (s, CH=CCH_2, 1H); ^{13}C NMR (100 MHz, $CDCl_3$) δ 13.89, 14.17, 21.45, 22.06, 26.91, 28.36, 28.94, 46.78, 54.00, 59.05, 126.08, 128.92, 129.91, 140.25, 142.13, 142.47; EI-HRMS *m/z* [M$^+$] calcd for $C_{11}H_{22}N_3O$ 212.1763, found 212.1751.

[Pent-Bu-C2OH-tr][OTs] (a12): 85% yield, light yellow liquid; ^{1}H NMR (400 MHz, $CDCl_3$) δ 0.89 (t, J = 8.0 Hz, CH_3, 3H), 0.97 (t, J = 8.0 Hz, CH_3, 3H), 1.29-1.43 (m, 3 x CH_2, 6H), 1.89-2.02 (m, 2 x NCH$_2$CH_2, 4H), 2.34 (s, *p*-CH_3, 3H), 3.08 (t, J = 6.0 Hz, CH=CCH_2, 2H), 4.06 (t, J = 6.0 Hz, CH$_2$CH_2OH, 2H), 4.48 (t, J = 8.0 Hz, CNCH_2, 2H), 4.58 (t, J = 6.0 Hz, CHNCH_2, 2H), 7.14 (d, J = 8.0 Hz, aryl H, 2H), 7.73 (d, J = 8.0 Hz, aryl H, 2H), 9.05 (s, CH=CCH$_2$, 1H); ^{13}C NMR (100 MHz, $CDCl_3$) δ 13.53, 13.93, 19.69, 21.47, 22.09, 27.09, 28.39, 29.00, 30.97, 51.10, 54.03, 58.98, 126.09, 128.86, 130.15, 139.92, 142.66, 142.79; EI-HRMS *m/z* [M$^+$] calcd for $C_{13}H_{26}N_3O$ 240.2076, found 240.2065.

[Pent-Pent-C2OH-tr][OTs] (a13): 83% yield, light yellow liquid; ^{1}H NMR (400 MHz, $CDCl_3$) δ 0.87-0.92 (m, 2 x CH_3, 6H), 1.25-1.39 (m, 4 x CH_2, 8H), 1.90-2.02 (m, 2 x NCH$_2$CH_2, 4H), 2.33 (s, *p*-CH_3, 3H), 3.08 (t, J = 6.0 Hz, CH=CCH_2, 2H), 4.06 (t, J = 6.0 Hz, CH$_2$CH_2OH, 2H), 4.47 (t, J = 8.0 Hz, CNCH_2, 2H), 4.59 (t, J = 6.0 Hz, CHNCH_2, 2H), 7.14 (d, J = 8.0 Hz, aryl H, 2H), 7.73 (d, J = 8.0 Hz, aryl H, 2H),

9.06 (s, C*H*=CCH_2, 1H); ^{13}C NMR (100 MHz, $CDCl_3$) δ 13.93, 13.93, 21.47, 22.10, 22.15, 27.11, 28.40, 28.47, 28.75, 29.01, 51.33, 54.06, 58.96, 126.08, 128.85, 130.19, 139.83, 142.64, 142.98; EI-HRMS *m/z* [M^+] calcd for $C_{14}H_{28}N_3O$ 254.2232, found 254.2229.

[Pent-Hex-C2OH-tr][OTs] (a14): 67% yield, light yellow liquid; ^{1}H NMR (400 MHz, $CDCl_3$) δ 0.87-0.91 (m, 2 x CH_3, 6H), 1.30-1.32 (m, 5 x CH_2, 10H), 1.92-2.01 (m, 2 x NCH_2CH_2, 4H), 2.34 (s, *p*-CH_3, 3H), 3.08 (t, J = 6.0 Hz, CH=CCH_2, 2H), 4.06 (t, J = 6.0 Hz, CH_2CH_2OH, 2H), 4.47 (t, J = 8.0 Hz, $CNCH_2$, 2H), 4.60 (t, J = 6.0 Hz, $CHNCH_2$, 2H), 7.14 (d, J = 8.0 Hz, aryl H, 2H), 7.73 (d, J = 8.0 Hz, aryl H, 2H), 9.06 (s, C*H*=CCH_2, 1H); ^{13}C NMR (100 MHz, $CDCl_3$) δ 13.93, 14.05, 21.47, 22.10, 22.51, 26.08, 27.11, 28.40, 29.01, 29.02, 31.16, 51.35, 54.07, 58.94, 126.08, 128.85, 130.21, 139.83, 142.62, 142.97; EI-HRMS *m/z* [M^+] calcd for $C_{15}H_{30}N_3O$ 268.2389, found 268.2378.

[Pent-Oct-C2OH-tr][OTs] (a15): 84% yield, light yellow liquid; ^{1}H NMR (400 MHz, $CDCl_3$) δ 0.87-0.91 (m, 2 x CH_3, 6H), 1.26-1.34 (m, 7 x CH_2, 10H), 1.92-2.03 (m, 2 x NCH_2CH_2, 4H), 2.34 (s, *p*-CH_3, 3H), 3.08 (t, J = 6.0 Hz, CH=CCH_2, 2H), 4.06 (t, J = 6.0 Hz, CH_2CH_2OH, 2H), 4.47 (t, J = 8.0 Hz, $CNCH_2$, 2H), 4.59 (t, J = 8.0 Hz, $CHNCH_2$, 2H), 7.14 (d, J = 8.0 Hz, aryl H, 2H), 7.73 (d, J = 8.0 Hz, aryl H, 2H), 9.07 (s, C*H*=CCH_2, 1H); ^{13}C NMR (100 MHz, $CDCl_3$) δ 13.93, 14.22, 21.47, 22.10, 22.74,

26.43, 27.08, 28.39, 29.01, 29.01, 29.06, 29.15, 31.82, 51.33, 54.06, 58.92, 126.06, 128.86, 130.18, 139.83, 142.58, 142.95; EI-HRMS *m/z* [M^+] calcd for $C_{17}H_{34}N_3O$ 296.2702, found 296.2698.

[Hex-Et-C2OH-tr][OTs] (a16): 92% yield, light yellow liquid; ^{1}H NMR (400 MHz, $CDCl_3$) δ 0.87 (t, *J* = 6.0 Hz, CH_3, 3H), 1.25-1.31 (m, 3 x CH_2, 6H), 1.59 (t, *J* = 8.0 Hz, CH_3, 3H), 1.92-1.99 (m, NCH_2CH_2, 2H), 2.34 (s, *p*-CH_3, 3H), 3.11 (t, *J* = 6.0 Hz, CH=CCH_2, 2H), 4.04 (t, *J* = 6.0 Hz, CH_2CH_2OH, 2H), 4.52-4.57 (m, CNCH_2 + CHNCH_2, 4H), 7.11 (d, *J* = 8.0 Hz, aryl H, 2H), 7.73 (d, *J* = 8.0 Hz, aryl H, 2H), 8.94 (s, C*H*=CCH_2, 1H); ^{13}C NMR (100 MHz, $CDCl_3$) δ 14.08, 14.14, 21.46, 22.51, 26.00, 26.83, 29.21, 31.12, 46.77, 54.01, 59.04, 126.04, 128.98, 129.80, 140.15, 142.43; EI-HRMS *m/z* [M^+] calcd for $C_{12}H_{24}N_3O$ 226.1919, found 226.1919.

[Hex-Bu-C2OH-tr][OTs] (a17): 66% yield, light yellow liquid; ^{1}H NMR (400 MHz, $CDCl_3$) δ 0.87 (t, *J* = 6.0 Hz, CH_3, 3H), 0.98 (t, *J* = 6.0 Hz, CH_3, 3H), 1.30-1.42 (m, 4 x CH_2, 8H), 1.89-2.00 (m, 2 x NCH_2CH_2, 4H), 2.34 (s, *p*-CH_3, 3H), 3.08 (t, *J* = 6.0 Hz, CH=CCH_2, 2H), 4.07 (t, *J* = 6.0 Hz, CH_2CH_2OH, 2H), 4.48 (t, *J* = 8.0 Hz, CNCH_2, 2H), 4.59 (t, *J* = 8.0 Hz, CHNCH_2, 2H), 7.14 (d, *J* = 8.0 Hz, aryl H, 2H), 7.73 (d, *J* = 8.0 Hz, aryl H, 2H), 9.05 (s, C*H*=CCH_2, 1H); ^{13}C NMR (100 MHz, $CDCl_3$) δ 13.52, 14.06, 19.69, 21.46, 22.51, 26.00, 27.07, 29.26, 30.96, 31.11, 51.08, 54.05,

58.97, 126.06, 128.85, 130.12, 139.83, 142.62, 142.98; EI-HRMS *m/z* [M^+] calcd for $C_{14}H_{28}N_3O$ 254.2232, found 254.2221.

[Hex-Pent-C2OH-tr][OTs] (a18): 85% yield, light yellow liquid; ^{1}H NMR (400 MHz, $CDCl_3$) δ 0.87-0.93 (m, 2 x CH_3, 6H), 1.30-1.34 (m, 5 x CH_2, 10H), 1.93-1.98 (m, 2 x NCH_2CH_2, 4H), 2.33 (s, *p*-CH_3, 3H), 3.08 (t, *J* = 6.0 Hz, CH=CCH_2, 2H), 4.06 (t, *J* = 6.0 Hz, CH_2CH_2OH, 2H), 4.47 (t, *J* = 8.0 Hz, CNCH_2, 2H), 4.60 (t, *J* = 6.0 Hz, CHNCH_2, 2H), 7.14 (d, *J* = 8.0 Hz, aryl H, 2H), 7.74 (d, *J* = 8.0 Hz, aryl H, 2H), 9.09 (s, C*H*=CCH_2, 1H); ^{13}C NMR (100 MHz, $CDCl_3$) δ 13.90, 14.06, 21.15, 22.14, 22.52, 26.01, 27.12, 28.46, 28.76, 29.28, 31.12, 51.31, 54.08, 58.93, 126.05, 128.79, 130.22, 139.60, 142.63, 143.34; EI-HRMS *m/z* [M^+] calcd for $C_{15}H_{30}N_3O$ 268.2389, found 268.2382.

[Hex-Hex-C2OH-tr][OTs] (a19): 79% yield, light yellow liquid; ^{1}H NMR (400 MHz, $CDCl_3$) δ 0.85-0.91 (m, 2 x CH_3, 6H), 1.30-1.32 (m, 6 x CH_2, 12H), 1.91-2.01 (m, 2 x NCH_2CH_2, 4H), 2.34 (s, *p*-CH_3, 3H), 3.08 (t, *J* = 6.0 Hz, CH=CCH_2, 2H), 4.08 (t, *J* = 6.0 Hz, CH_2CH_2OH, 2H), 4.47 (t, *J* = 8.0 Hz, CNCH_2, 2H), 4.60 (t, *J* = 8.0 Hz, CHNCH_2, 2H), 7.14 (d, *J* = 8.0 Hz, aryl H, 2H), 7.73 (d, *J* = 8.0 Hz, aryl H, 2H), 9.05 (s, C*H*=CCH_2, 1H); ^{13}C NMR (100 MHz, $CDCl_3$) δ 14.07, 14.07, 19.69, 21.47, 22.52, 26.01, 26.08, 27.06, 29.00, 29.26, 31.12, 31.16, 51.34, 54.09, 58.95, 126.09, 128.90,

130.14, 139.98, 142.56, 142.68; EI-HRMS *m/z* [M^+] calcd for $C_{16}H_{32}N_3O$ 282.2545, found 282.2532.

[Hex-Oct-C2OH-tr][OTs] (a20): 69% yield, light yellow liquid; ^{1}H NMR (400 MHz, $CDCl_3$) δ 0.87-0.91 (m, 2 x CH_3 , 6H), 1.26-1.33 (m, 8 x CH_2, 16H), 1.92-2.00 (m, 2 x NCH_2CH_2 , 4H), 2.34 (s, *p*-CH_3, 3H), 3.08 (t, *J* = 6.0 Hz, CH=CCH_2, 2H), 4.06 (t, *J* = 6.0 Hz, CH_2CH_2OH, 2H), 4.47 (t, *J* = 8.0 Hz, CNCH_2, 2H), 4.59 (t, *J* = 8.0 Hz, CHNCH_2, 2H), 7.14 (d, *J* = 8.0 Hz, aryl H, 2H), 7.73 (d, *J* = 8.0 Hz, aryl H, 2H), 9.06 (s, C*H*=CCH_2, 1H); ^{13}C NMR (100 MHz, $CDCl_3$) δ 14.08, 14.22, 21.47, 22.53, 22.75, 26.02, 26.44, 27.10, 29.02, 29.07, 29.15, 29.28, 31.13, 31.83, 51.34, 54.09, 58.93, 126.08, 128.86, 130.20, 139.84, 142.60, 142.94; EI-HRMS *m/z* [M^+] calcd for $C_{18}H_{36}N_3O$ 310.2858, found 310.2847.

[Oct-Et-C2OH-tr][OTs] (a21): 96% yield, light yellow liquid; ^{1}H NMR (400 MHz, $CDCl_3$) δ 0.88 (t, *J* = 6.0 Hz, CH_3, 3H), 1.25-1.30 (m, 5 x CH_2, 10H), 1.62 (t, *J* = 8.0 Hz, CH_3, 3H), 1.95-2.02 (m, NCH_2CH_2 , 2H), 2.34 (s, *p*-CH_3, 3H), 3.10 (t, *J* = 6.0 Hz, CH=CCH_2, 2H), 4.07 (t, *J* = 6.0 Hz, CH_2CH_2OH, 2H), 4.53-4.60 (m, CNCH_2 + CHNCH_2, 4H), 7.14 (d, *J* = 8.0 Hz, aryl H, 2H), 7.72 (d, *J* = 8.0 Hz, aryl H, 2H), 8.98 (s, C*H*=CCH_2, 1H); ^{13}C NMR (100 MHz, $CDCl_3$) δ 14.19, 14.21, 21.44, 22.73, 26.33, 26.95, 28.95, 29.13, 29.28, 31.83, 46.78, 53.99, 59.08, 126.03, 128.87, 129.92, 139.92, 142.52, 142.78; EI-HRMS *m/z* [M^+] calcd for $C_{14}H_{28}N_3O$ 254.2232, found 254.2226.

[Oct-Bu-C2OH-tr][OTs] (a22): 79% yield, light yellow liquid; ^{1}H NMR (400 MHz, $CDCl_3$) δ 0.87 (t, *J* = 6.0 Hz, CH_3, 3H), 0.97 (t, *J* = 6.0 Hz, CH_3, 3H), 1.25-1.43 (m, 6 x CH_2, 12H), 1.89-2.00 (m, 2 x NCH_2CH_2, 4H), 2.33 (s, *p*-CH_3, 3H), 3.08 (t, *J* = 6.0 Hz, $CH{=}CCH_2$, 2H), 4.06 (t, *J* = 6.0 Hz, CH_2CH_2OH, 2H), 4.48 (t, *J* = 8.0 Hz, $CNCH_2$, 2H), 4.59 (t, *J* = 8.0 Hz, $CHNCH_2$, 2H), 7.14 (d, *J* = 8.0 Hz, aryl H, 2H), 7.73 (d, *J* = 8.0 Hz, aryl H, 2H), 9.06 (s, $CH{=}CCH_2$, 1H); ^{13}C NMR (100 MHz, $CDCl_3$) δ 13.54, 14.24, 19.71, 21.47, 22.76, 26.36, 27.12, 28.97, 29.18, 29.33, 31.00, 31.85, 51.10, 54.09, 58.95, 126.09, 128.85, 130.20, 139.79, 142.64, 143.02; EI-HRMS *m/z* [M^+] calcd for $C_{16}H_{32}N_3O$ 282.2545, found 282.2539.

[Oct-Pent-C2OH-tr][OTs] (a23): 82% yield, light yellow liquid; ^{1}H NMR (400 MHz, $CDCl_3$) δ 0.86-0.93 (m, 2 x CH_3, 6H), 1.25-1.38 (m, 7 x CH_2, 16H), 1.91-2.00 (m, 2 x NCH_2CH_2, 4H), 2.33 (s, *p*-CH_3, 3H), 3.08 (t, *J* = 4.0 Hz, $CH{=}CCH_2$, 2H), 4.06 (t, *J* = 4.0 Hz, CH_2CH_2OH, 2H), 4.47 (t, *J* = 8.0 Hz, $CNCH_2$, 2H), 4.59 (t, *J* = 8.0 Hz, $CHNCH_2$, 2H), 7.14 (d, *J* = 8.0 Hz, aryl H, 2H), 7.74 (d, *J* = 8.0 Hz, aryl H, 2H), 9.07 (s, $CH{=}CCH_2$, 1H); ^{13}C NMR (100 MHz, $CDCl_3$) δ 13.91, 14.23, 21.46, 22.15, 22.75, 26.37, 27.14, 28.46, 28.76, 28.97, 29.17, 29.34, 31.85, 51.33, 54.07, 58.97, 126.07, 128.81, 130.21, 139.67, 142.67, 143.25; EI-HRMS *m/z* [M^+] calcd for $C_{17}H_{34}N_3O$ 296.2702, found 296.2689.

[Oct-Hex-C2OH-tr][OTs] (a24): 95% yield, light yellow liquid; ^{1}H NMR (400 MHz, $CDCl_3$) δ 0.86-0.91 (m, 2 x CH_3, 6H), 1.25-1.32 (m, 8 x CH_2, 16H), 1.93-2.01 (m, 2 x $NCH_2C\mathit{H_2}$, 4H), 2.34 (s, *p*-CH_3, 3H), 3.08 (t, *J* = 6.0 Hz, CH=CC$\mathit{H_2}$, 2H), 4.08 (t, *J* = 6.0 Hz, $CH_2C\mathit{H_2}OH$, 2H), 4.47 (t, *J* = 8.0 Hz, CNC$\mathit{H_2}$, 2H), 4.60 (t, *J* = 8.0 Hz, CHNC$\mathit{H_2}$, 2H), 7.14 (d, *J* = 8.0 Hz, aryl H, 2H), 7.73 (d, *J* = 8.0 Hz, aryl H, 2H), 9.05 (s, C*H*=CCH_2, 1H); ^{13}C NMR (100 MHz, $CDCl_3$) δ 14.06, 14.23, 21.47, 22.52, 22.77, 26.08, 26.35, 27.00, 28.98, 28.99, 29.18, 29.31, 31.16, 31.86, 51.31, 54.07, 58.95, 126.07, 128.90, 130.07, 139.94, 142.53; EI-HRMS *m/z* [M^+] calcd for $C_{18}H_{36}N_3O$ 310.2858, found 310.2846.

[Oct-Oct-C2OH-tr][OTs] (a25): 61% yield, light yellow liquid; ^{1}H NMR (400 MHz, $CDCl_3$) δ 0.86-0.89 (m, 2 x CH_3, 6H), 1.26-1.32 (m, 10 x CH_2, 20H), 1.95-2.01 (m, 2 x $NCH_2C\mathit{H_2}$, 4H), 2.34 (s, *p*-CH_3, 3H), 3.08 (t, *J* = 6.0 Hz, CH=CC$\mathit{H_2}$, 2H), 4.09 (t, *J* = 6.0 Hz, $CH_2C\mathit{H_2}OH$, 2H), 4.47 (t, *J* = 6.0 Hz, CNC$\mathit{H_2}$, 2H), 4.61 (t, *J* = 6.0 Hz, CHNC$\mathit{H_2}$, 2H), 7.14 (d, *J* = 8.0 Hz, aryl H, 2H), 7.73 (d, *J* = 8.0 Hz, aryl H, 2H), 9.05 (s, C*H*=CCH_2, 1H); ^{13}C NMR (100 MHz, $CDCl_3$) δ 14.24, 14.24, 21.48, 22.77, 22.77, 26.36, 26.44, 27.07, 28.99, 29.03, 29.03, 29.17, 29.19, 29.32, 31.85, 31.87, 51.33, 54.09, 59.02, 126.09, 128.92, 130.11, 139.98, 142.53, 142.72; EI-HRMS *m/z* [M^+] calcd for $C_{20}H_{40}N_3O$ 338.3171, found 338.3157.

[Pent-*i*-Hex-C2OH-tr][OTs] (a26): 67% yield, light yellow liquid; ^{1}H NMR (400 MHz, $CDCl_3$) δ 0.87-0.90 (m, 3 x CH_3, 9H), 1.18-1.37 (m, 3 x CH_2, 6H), 1.53-1.63 (m, C*H*CH_3, 1H), 1.90-2.02 (m, 2 x $NCH_2C\mathit{H}_2$, 4H), 2.34 (s, *p*-CH_3, 3H), 3.08 (t, *J* = 6.0 Hz, CH=CC*H*$_2$, 2H), 4.06 (m, $CH_2C\mathit{H}_2OH$, 2H), 4.46 (t, *J* = 8.0 Hz, CNC*H*$_2$, 2H), 4.59 (t, *J* = 8.0 Hz, CHNC*H*$_2$, 2H), 7.14 (d, *J* = 8.0 Hz, aryl H, 2H), 7.73 (d, *J* = 8.0 Hz, aryl H, 2H), 9.07 (s, C*H*=CCH_2, 1H); ^{13}C NMR (100 MHz, $CDCl_3$) δ 13.92, 21.45, 22.08, 22.44, 26.96, 27.06, 27.64, 28.36, 28.98, 35.31, 51.53, 54.00, 59.00, 126.05, 128.85, 130.10, 139.86, 142.65, 142.88; EI-HRMS *m/z* [M^+] calcd for $C_{15}H_{30}N_3O$ 268.2389, found 268.2379.

[Hex-*i*-Pent-C2OH-tr][OTs] (a27): 85% yield, light yellow liquid; ^{1}H NMR (400 MHz, $CDCl_3$) δ 0.87 (t, *J* = 8.0 Hz, CH_3, 3H), 0.98 (d, *J* = 6.0 Hz, 2 x CHC*H*$_3$, 6H), 1.29 (m, 3 x CH_2, 6H), 1.57-1.68 (m, C*H*CH_3, 1H), 1.80-1.85 (m, $NCH_2C\mathit{H}_2$, 2H), 1.94-2.02 (m, $NCH_2C\mathit{H}_2$, 2H), 2.33 (s, *p*-CH_3, 3H), 3.08 (t, *J* = 6.0 Hz, CH=CC*H*$_2$, 2H), 4.07 (t, *J* = 6.0 Hz, $CH_2C\mathit{H}_2OH$, 2H), 4.49 (t, *J* = 6.0 Hz, CNC*H*$_2$, 2H), 4.59 (t, *J* = 6.0 Hz, CHNC*H*$_2$, 2H), 7.14 (d, *J* = 8.0 Hz, aryl H, 2H), 7.72 (d, *J* = 8.0 Hz, aryl H, 2H), 9.05 (s, C*H*=CCH_2, 1H); ^{13}C NMR (100 MHz, $CDCl_3$) δ 14.08, 21.49, 22.27, 22.54, 25.77, 26.04, 27.13, 29.28, 31.13, 37.71, 49.84, 54.13, 58.88, 126.12, 128.89, 130.27, 139.97, 142.53, 142.67; EI-HRMS *m/z* [M^+] calcd for $C_{15}H_{30}N_3O$ 268.2389, found 268.2381.

[Oct-*i*-Bu-C2OH-tr][OTs] (a28): 81% yield, light yellow liquid; ^{1}H NMR (400 MHz, $CDCl_3$) δ 0.87 (t, *J* = 8.0 Hz, CH_3, 3H), 0.98 (d, *J* = 6.0 Hz, 2 x $CHCH_3$, 6H), 1.24-1.30 (m, 5 x CH_2, 12H), 1.95-2.01 (m, NCH_2CH_2, 2H), 2.23-2.32 (m, $CHCH_3$, 1H), 2.34 (s, *p*-CH_3, 3H), 3.07 (t, *J* = 6.0 Hz, $CH{=}CCH_2$, 2H), 4.04 (t, *J* = 6.0 Hz, CH_2CH_2OH, 2H), 4.31 (d, *J* = 4.0 Hz, $CNCH_2$, 2H), 4.60 (t, *J* = 8.0 Hz, $CHNCH_2$, 2H), 7.15 (d, *J* = 8.0 Hz, aryl H, 2H), 7.74 (d, *J* = 8.0 Hz, aryl H, 2H), 9.08 (s, $CH{=}CCH_2$, 1H); ^{13}C NMR (100 MHz, $CDCl_3$) δ 13.54, 14.24, 19.71, 21.47, 22.76, 26.36, 27.12, 28.97, 29.33, 31.00, 31.85, 51.10, 54.09, 58.95, 126.09, 128.85, 130.20, 139.79, 142.64, 143.02; EI-HRMS *m/z* [M^+] calcd for $C_{16}H_{32}N_3O$ 282.2545, found 282.2533.

Combinatorial Synthesis of a Library of 29 [R2-R3-C2OH-tr][OTMBS] Room-Temperature Ionic Liquids

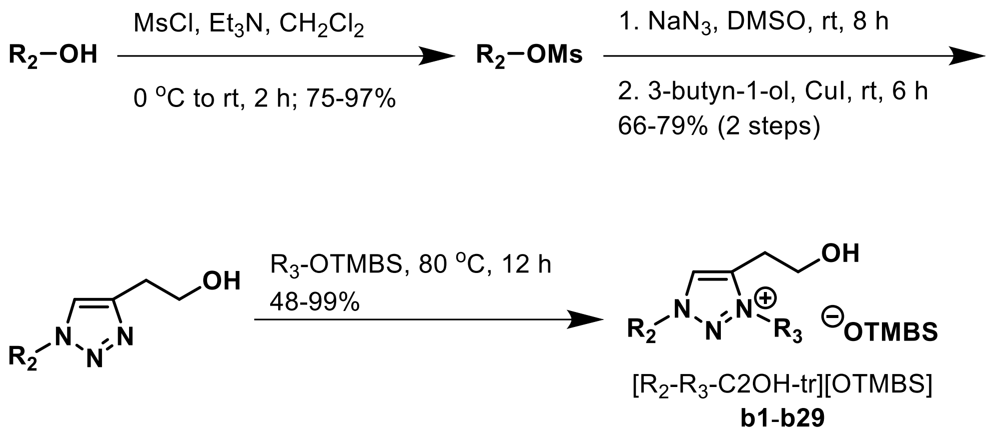

Generalized Procedure for the Synthesis of [R_2-R_3-C2OH-tr][OTMBS] Ionic Liquids

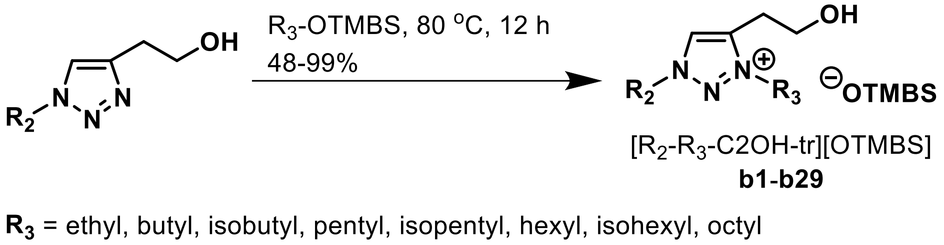

To a round-bottomed flask containing R_2-C2OH-tr (0.6 mmol) was added alkyl 2,4,6-trimethylbenzenesulfonate (R_3-OTMBS, 0.8 mmol, 1.3 equiv). The mixture was heated at 80 °C for 12 h. The progress of the ionic liquid forming reaction could be readily monitored by TLC. After reaction was completed, the reaction mixture was washed with ether (3 mL, 3 times) and then with *n*-hexane (3 mL, 3 times) to finally afford the desired [R_2-R_3-C2OH-tr][OTMBS] as room-temperature ionic liquids (**a1-a29**).

[Et-Et-C2OH-tr][OTMBS] (b1): 99% yield, light yellow liquid; ^{1}H NMR (400 MHz, $CDCl_3$) δ 1.60-1.69 (m, 2 x CH_3, 6H), 2.22 (s, *p*-CH_3, 3H), 2.63 (s, 2 x *o*-CH_3, 6H), 3.08 (t, J = 6.0 Hz, CH=CCH_2, 2H), 4.07 (t, J = 6.0 Hz, CH_2CH_2OH, 2H), 4.53 (q, J = 8.0 Hz, CNC$H_2$$CH_3$, 2H), 4.68 (q, J = 8.0 Hz, CHNC$H_2$$CH_3$, 2H), 6.81 (s, aryl H, 2H), 9.16 (s, C*H*=CCH_2, 1H); ^{13}C NMR (100 MHz, $CDCl_3$) δ 14.13, 14.32, 20.91, 23.08,

26.90, 46.72, 49.43, 58.89, 129.77, 130.81, 137.09, 138.57, 140.13, 142.49; EI-HRMS *m/z* [M^+] calcd for $C_8H_{16}N_3O$ 170.1293, found 170.1284.

[Et-Bu-C2OH-tr][OTMBS] (b2): 93% yield, light yellow liquid; ^{1}H NMR (400 MHz, $CDCl_3$) δ 0.98 (t, *J* = 8.0 Hz, $CH_2CH_2CH_3$, 3H), 1.35-1.45 (m, $CH_2CH_2CH_3$, 2H), 1.67 (t, *J* = 8.0 Hz, NCH_2CH_3, 3H), 1.90-1.97 (m, $CH_2CH_2CH_3$, 2H), 2.22 (s, *p*-CH_3, 3H), 2.65 (s, 2 x *o*-CH_3, 6H), 3.06 (t, *J* = 6.0 Hz, $CH{=}CCH_2$, 2H), 4.08 (t, *J* = 6.0 Hz, CH_2CH_2OH, 2H), 4.46 (t, *J* = 8.0 Hz, NCH_2CH_2, 2H), 4.69 (q, *J* = 8.0 Hz, NCH_2CH_3, 2H), 6.81 (s, aryl H, 2H), 9.24 (s, $CH{=}CCH_2$, 1H); ^{13}C NMR (100 MHz, $CDCl_3$) δ 13.54, 14.39, 19.76, 20.92, 23.13, 27.08, 31.01, 49.54, 51.06, 58.72, 130.09, 130.80, 137.11, 138.48, 140.25, 142.60; EI-HRMS *m/z* [M^+] calcd for $C_{10}H_{20}N_3O$ 198.1606, found 198.1595.

[Et-Pent-C2OH-tr][OTMBS] (b3): 82% yield, light yellow liquid; ^{1}H NMR (400 MHz, $CDCl_3$) δ 0.90 (t, *J* = 6.0 Hz, $CH_2CH_2CH_3$, 3H), 1.32-1.39 (m, 2 x CH_2, 4H), 1.66 (t, *J* = 8.0 Hz, NCH_2CH_3, 3H), 1.91-1.98 (m, NCH_2CH_2, 2H), 2.22 (s, *p*-CH_3, 3H), 2.64 (s, 2 x *o*-CH_3, 6H), 3.07 (t, *J* = 6.0 Hz, $CH{=}CCH_2$, 2H), 4.07 (t, *J* = 6.0 Hz, CH_2CH_2OH, 2H), 4.45 (t, *J* = 8.0 Hz, NCH_2CH_2, 2H), 4.68 (q, *J* = 8.0 Hz, NCH_2CH_3, 2H), 6.81 (s, aryl H, 2H), 9.25 (s, $CH{=}CCH_2$, 1H); ^{13}C NMR (100 MHz, $CDCl_3$) δ 13.91, 14.41, 20.91, 22.15, 23.14, 27.12, 28.48, 28.77, 49.44, 51.29, 58.78, 130.06,

130.75, 137.06, 138.35, 140.44, 142.70; EI-HRMS *m/z* [M^+] calcd for $C_{11}H_{22}N_3O$ 212.1763, found 212.1752.

[Et-Hex-C2OH-tr][OTMBS] (b4): 69% yield, light yellow liquid; ^1H NMR (400 MHz, $CDCl_3$) δ 0.89 (t, *J* = 6.0 Hz, $CH_2CH_2CH_3$, 3H), 1.33-1.35 (m, 3 x CH_2, 6H), 1.67 (t, *J* = 8.0 Hz, NCH_2CH_3, 3H), 1.90-1.98 (m, NCH_2CH_2, 2H), 2.22 (s, *p*-CH_3, 3H), 2.64 (s, 2 x *o*-CH_3, 6H), 3.06 (t, *J* = 4.0 Hz, $CH{=}CCH_2$, 2H), 4.07 (t, *J* = 4.0 Hz, CH_2CH_2OH, 2H), 4.45 (t, *J* = 6.0 Hz, NCH_2CH_2, 2H), 4.68 (q, *J* = 8.0 Hz, NCH_2CH_3, 2H), 6.81 (s, aryl H, 2H), 9.24 (s, $CH{=}CCH_2$, 1H); ^{13}C NMR (100 MHz, $CDCl_3$) δ 14.06, 14.35, 20.91, 22.50, 23.09, 26.08, 26.95, 28.97, 31.15, 49.43, 51.26, 58.82, 129.84, 130.83, 137.09, 138.62, 140.01, 142.55; EI-HRMS *m/z* [M^+] calcd for $C_{12}H_{24}N_3O$ 226.1919, found 226.1912.

[Et-Oct-C2OH-tr][OTMBS] (b5): 53% yield, light yellow liquid; ^1H NMR (400 MHz, $CDCl_3$) δ 0.88 (t, *J* = 6.0 Hz, $CH_2CH_2CH_3$, 3H), 1.26-1.34 (m, 5 x CH_2, 10H), 1.67 (t, *J* = 6.0 Hz, NCH_2CH_3, 3H), 1.92-1.96 (m, NCH_2CH_2, 2H), 2.22 (s, *p*-CH_3, 3H), 2.65 (s, 2 x *o*-CH_3, 6H), 3.07 (t, *J* = 6.0 Hz, $CH{=}CCH_2$, 2H), 4.07 (t, *J* = 6.0 Hz, CH_2CH_2OH, 2H), 4.44 (t, *J* = 8.0 Hz, NCH_2CH_2, 2H), 4.69 (q, *J* = 6.0 Hz, NCH_2CH_3, 2H), 6.81 (s, aryl H, 2H), 9.27 (s, $CH{=}CCH_2$, 1H); ^{13}C NMR (100 MHz, $CDCl_3$) δ 14.22, 14.41, 20.91, 22.74, 23.15, 26.44, 27.13, 29.00, 29.08, 29.12, 31.82, 49.44,

51.31, 58.76, 130.09, 130.75, 137.05, 138.31, 140.49, 142.69; EI-HRMS *m/z* [M^+] calcd for $C_{14}H_{28}N_3O$ 254.2232, found 254.2221.

[Bu-Et-C2OH-tr][OTMBS] (b6): 70% yield, light yellow liquid; ^{1}H NMR (400 MHz, $CDCl_3$) δ 0.96 (t, *J* = 6.0 Hz, $CH_2CH_2CH_3$, 3H), 1.33-1.43 (m, $CH_2CH_2CH_3$, 2H), 1.62 (t, *J* = 8.0 Hz, NCH_2CH_3 , 3H), 1.95-2.02 (m, NCH_2CH_2, 2H), 2.22 (s, *p*-CH_3, 3H), 2.62 (s, 2 x *o*-CH_3, 6H), 3.09 (t, *J* = 6.0 Hz, CH=CCH_2, 2H), 4.07 (t, *J* = 6.0 Hz, CH_2CH_2OH, 2H), 4.52-4.62 (m, NCH_2CH_2 + NCH_2CH_3, 4H), 6.81 (s, aryl H, 2H), 9.12 (s, C*H*=CCH_2, 1H); ^{13}C NMR (100 MHz, $CDCl_3$) δ 13.47, 14.21, 19.63, 20.92, 23.10, 26.98, 31.21, 46.77, 53.80, 58.88, 130.15, 130.79, 137.13, 138.55, 140.05, 142.59; EI-HRMS *m/z* [M^+] calcd for $C_{10}H_{20}N_3O$ 198.1606, found 198.1596.

[Bu-Bu-C2OH-tr][OTMBS] (b7): 48% yield, light yellow liquid; ^{1}H NMR (400 MHz, $CDCl_3$) δ 0.93-1.00 (m, 2 x CH_3 , 6H), 1.32-1.44 (m, 2 x $CH_2CH_2CH_3$, 4H), 1.89-2.02 (m, 2 x NCH_2CH_2 , 4H), 2.22 (s, *p*-CH_3, 3H), 2.63 (s, 2 x *o*-CH_3, 6H), 3.07 (t, *J* = 6.0 Hz, CH=CCH_2, 2H), 4.07 (t, *J* = 6.0 Hz, CH_2CH_2OH, 2H), 4.46 (t, *J* = 8.0 Hz, CNCH_2, 2H), 4.60 (t, *J* = 8.0 Hz, CHNCH_2, 2H), 6.81 (s, aryl H, 2H), 9.19 (s, C*H*=CCH_2, 1H); ^{13}C NMR (100 MHz, $CDCl_3$) δ 13.46, 13.52, 19.61, 19.69, 20.91, 23.12, 27.10, 30.98, 31.22, 51.07, 53.78, 58.78, 130.32, 130.74, 137.11, 138.37, 140.31, 142.75; EI-HRMS *m/z* [M^+] calcd for $C_{12}H_{24}N_3O$ 226.1919, found 226.1908.

[Bu-Pent-C2OH-tr][OTMBS] (b8): 55% yield, light yellow liquid; ^{1}H NMR (400 MHz, $CDCl_3$) δ 0.90-0.98 (m, 2 x CH_3, 6H), 1.33-1.40 (m, 3 x CH_2, 6H), 1.94-2.03 (m, 2 x $NCH_2C\mathit{H_2}$, 4H), 2.22 (s, *p*-CH_3, 3H), 2.65 (s, 2 x *o*-CH_3, 6H), 3.07 (t, *J* = 4.0 Hz, $CH{=}CC\mathit{H_2}$, 2H), 4.09 (t, *J* = 4.0 Hz, $CH_2C\mathit{H_2}OH$, 2H), 4.44 (t, *J* = 6.0 Hz, $CNC\mathit{H_2}$, 2H), 4.62 (t, *J* = 8.0 Hz, $CHNC\mathit{H_2}$, 2H), 6.81 (s, aryl H, 2H), 9.24 (s, $C\mathit{H}{=}CCH_2$, 1H); ^{13}C NMR (100 MHz, $CDCl_3$) δ 13.45, 13.91, 19.61, 20.91, 22.14, 23.14, 27.08, 28.46, 28.75, 31.22, 51.28, 53.82, 58.73, 130.36, 130.77, 137.08, 138.36, 140.40, 142.67; EI-HRMS *m/z* [M^+] calcd for $C_{13}H_{26}N_3O$ 240.2076, found 240.2064.

[Bu-Hex-C2OH-tr][OTMBS] (b9): 73% yield, light yellow liquid; ^{1}H NMR (400 MHz, $CDCl_3$) δ 0.89 (t, *J* = 6.0 Hz, CH_3, 3H), 0.96 (t, *J* = 8.0 Hz, $C\mathit{H_3}$, 3H), 1.31-1.41 (m, 4 x CH_2, 8H), 1.94-2.04 (m, 2 x $NCH_2C\mathit{H_2}$, 4H), 2.22 (s, *p*-CH_3, 3H), 2.66 (s, 2 x *o*-CH_3, 6H), 3.06 (t, *J* = 6.0 Hz, $CH{=}CC\mathit{H_2}$, 2H), 4.10 (t, *J* = 6.0 Hz, $CH_2C\mathit{H_2}OH$, 2H), 4.45 (t, *J* = 6.0 Hz, $CNC\mathit{H_2}$, 2H), 4.62 (t, *J* = 6.0 Hz, $CHNC\mathit{H_2}$, 2H), 6.81 (s, aryl H, 2H), 9.26 (s, $C\mathit{H}{=}CCH_2$, 1H); ^{13}C NMR (100 MHz, $CDCl_3$) δ 13.48, 14.06, 19.67, 20.94, 22.53, 23.19, 26.12, 27.22, 29.08, 31.16, 31.26, 51.33, 53.91, 58.55, 130.60, 130.74, 137.10, 138.20, 140.67, 142.71; EI-HRMS *m/z* [M^+] calcd for $C_{14}H_{28}N_3O$ 254.2232, found 254.2223.

[Bu-Oct-C2OH-tr][OTMBS] (b10): 75% yield, light yellow liquid; ^{1}H NMR (400 MHz, $CDCl_3$) δ 0.88 (t, *J* = 8.0 Hz, CH_3, 3H), 0.95 (t, *J* = 8.0 Hz, CH_3, 3H), 1.26-1.41

(m, 6 x CH_2, 12H), 1.85-2.03 (m, 2 x NCH_2CH_2, 4H), 2.22 (s, *p*-CH_3, 3H), 2.65 (s, 2 x *o*-CH_3, 6H), 3.07 (t, J = 6.0 Hz, CH=CCH_2, 2H), 4.07 (t, J = 6.0 Hz, CH_2CH_2OH, 2H), 4.45 (t, J = 8.0 Hz, CNCH_2, 2H), 4.60 (t, J = 8.0 Hz, CHNCH_2, 2H), 6.81 (s, aryl H, 2H), 9.24 (s, C*H*=CCH_2, 1H); ^{13}C NMR (100 MHz, $CDCl_3$) δ 13.47, 14.23, 19.65, 20.93, 22.75, 23.18, 26.43, 27.17, 29.00, 29.09, 29.14, 31.25, 31.82, 51.32, 53.83, 58.67, 130.47, 130.75, 137.09, 138.26, 140.55, 142.74; EI-HRMS *m/z* [M^+] calcd for $C_{16}H_{32}N_3O$ 282.2545, found 282.2540.

[Pent-Et-C2OH-tr][OTMBS] (b11): 85% yield, light yellow liquid; ^{1}H NMR (400 MHz, $CDCl_3$) δ 0.90 (t, J = 6.0 Hz, CH_3, 3H), 1.33-1.36 (m, 2 x CH_2, 4H), 1.62 (t, J = 8.0 Hz, CH_3, 3H), 1.97-2.04 (m, NCH_2CH_2, 4H), 2.22 (s, *p*-CH_3, 3H), 2.62 (s, 2 x *o*-CH_3, 6H), 3.10 (t, J = 4.0 Hz, CH=CCH_2, 2H), 4.08 (t, J = 4.0 Hz, CH_2CH_2OH, 2H), 4.52-4.61 (m, 2 x NCH_2, 4H), 6.80 (s, aryl H, 2H), 9.15 (s, C*H*=CCH_2, 1H); ^{13}C NMR (100 MHz, $CDCl_3$) δ 13.92, 14.23, 20.92, 22.10, 23.09, 27.02, 28.40, 29.03, 46.78, 54.02, 58.90, 130.17, 130.79, 137.14, 138.55, 140.04, 142.65; EI-HRMS *m/z* [M^+] calcd for $C_{11}H_{22}N_3O$ 212.1763, found 212.1755.

[Pent-Bu-C2OH-tr][OTMBS] (b12): 64% yield, light yellow liquid; ^{1}H NMR (400 MHz, $CDCl_3$) δ 0.90 (t, J = 6.0 Hz, CH_3, 3H), 0.99 (t, J = 6.0 Hz, CH_3, 3H), 1.33-1.43 (m, 3 x CH_2, 6H), 1.91-2.05 (m, 2 x NCH_2CH_2, 4H), 2.22 (s, *p*-CH_3, 3H), 2.64 (s, 2 x *o*-CH_3, 6H), 3.07 (t, J = 6.0 Hz, CH=CCH_2, 2H), 4.09 (t, J = 6.0 Hz, CH_2CH_2OH, 2H),

4.47 (t, J = 8.0 Hz, CNCH_2, 2H), 4.61 (t, J = 6.0 Hz, CHNCH_2, 2H), 6.81 (s, aryl H, 2H), 9.21 (s, CH=CCH_2, 1H); ^{13}C NMR (100 MHz, $CDCl_3$) δ 13.53, 13.94, 19.73, 20.94, 22.11, 23.13, 27.13, 28.43, 29.05, 31.01, 51.08, 54.10, 58.70, 130.38, 130.82, 137.17, 138.58, 140.05, 142.70; EI-HRMS m/z [M^+] calcd for $C_{13}H_{26}N_3O$ 240.2076, found 240.2064.

[Pent-Pent-C2OH-tr][OTMBS] (b13): 60% yield, light yellow liquid; ^{1}H NMR (400 MHz, $CDCl_3$) δ 0.88-0.93 (m, 2 x CH_3, 6H), 1.31-1.40 (m, 4 x CH_2, 8H), 1.92-2.05 (m, 2 x NCH_2CH_2, 4H), 2.22 (s, p-CH_3, 3H), 2.65 (s, 2 x o-CH_3, 6H), 3.07 (t, J = 6.0 Hz, CH=CCH_2, 2H), 4.09 (t, J = 6.0 Hz, CH_2CH_2OH, 2H), 4.46 (t, J = 6.0 Hz, CNCH_2, 2H), 4.60 (t, J = 6.0 Hz, CHNCH_2, 2H), 6.81 (s, aryl H, 2H), 9.24 (s, CH=CCH_2, 1H); ^{13}C NMR (100 MHz, $CDCl_3$) δ 13.93, 13.95, 20.94, 22.13, 22.17, 23.18, 27.19, 28.45, 28.50, 28.80, 29.07, 51.31, 54.13, 58.63, 130.52, 130.77, 137.12, 138.33, 140.45, 142.71; EI-HRMS m/z [M^+] calcd for $C_{14}H_{28}N_3O$ 254.2232, found 254.2226.

[Pent-Hex-C2OH-tr][OTMBS] (b14): 90% yield, light yellow liquid; ^{1}H NMR (400 MHz, $CDCl_3$) δ 0.88-0.91 (m, 2 x CH_3, 6H), 1.31-1.36 (m, 5 x CH_2, 8H), 1.91-2.05 (m, 2 x NCH_2CH_2, 4H), 2.22 (s, p-CH_3, 3H), 2.65 (s, 2 x o-CH_3, 6H), 3.07 (t, J = 6.0 Hz, CH=CCH_2, 2H), 4.09 (t, J = 6.0 Hz, CH_2CH_2OH, 2H), 4.46 (t, J = 6.0 Hz, CNCH_2, 2H), 4.60 (t, J = 6.0 Hz, CHNCH_2, 2H), 6.81 (s, aryl H, 2H), 9.24 (s,

C*H*=CCH$_2$, 1H); ^{13}C NMR (100 MHz, CDCl$_3$) δ 13.92, 14.05, 20.90, 22.09, 22.50, 23.16, 26.06, 27.13, 28.40, 29.01, 29.04, 31.15, 51.31, 54.02, 58.76, 130.35, 130.73, 137.05, 138.33, 140.56, 142.74; EI-HRMS *m/z* [M$^+$] calcd for $C_{15}H_{30}N_3O$ 268.2389, found 268.2383.

[Pent-Oct-C2OH-tr][OTMBS] (b15): 74% yield, light yellow liquid; ^{1}H NMR (400 MHz, CDCl$_3$) δ 0.87-0.91 (m, 2 x CH$_3$, 6H), 1.26-1.36 (m, 7 x CH$_2$, 8H), 1.91-2.05 (m, 2 x NCH$_2$C*H$_2$*, 4H), 2.22 (s, *p*-CH$_3$, 3H), 2.65 (s, 2 x *o*-CH$_3$, 6H), 3.07 (t, *J* = 6.0 Hz, CH=CC*H*$_2$, 2H), 4.09 (t, *J* = 6.0 Hz, CH$_2$C*H*$_2$OH, 2H), 4.45 (t, *J* = 8.0 Hz, CNC*H*$_2$, 2H), 4.60 (t, *J* = 6.0 Hz, CHNC*H*$_2$, 2H), 6.81 (s, aryl H, 2H), 9.24 (s, C*H*=CCH$_2$, 1H); ^{13}C NMR (100 MHz, CDCl$_3$) δ 13.93, 14.21, 20.91, 22.10, 22.74, 23.17, 26.42, 27.15, 28.41, 29.00, 29.06, 29.07, 29.13, 31.81, 51.32, 54.03, 58.73, 130.40, 130.74, 137.06, 138.24, 140.58, 142.74; EI-HRMS *m/z* [M$^+$] calcd for $C_{17}H_{34}N_3O$ 296.2702, found 296.2690.

[Hex-Et-C2OH-tr][OTMBS] (b16): 79% yield, light yellow liquid; ^{1}H NMR (400 MHz, CDCl$_3$) δ 0.88 (t, *J* = 6.0 Hz, CH$_3$, 3H), 1.29-1.35 (m, 3 x CH$_2$, 6H), 1.61 (t, *J* = 8.0 Hz, CH$_3$, 3H), 1.95-2.03 (m, NCH$_2$C*H$_2$*, 2H), 2.22 (s, *p*-CH$_3$, 3H), 2.63 (s, 2 x *o*-CH$_3$, 6H), 3.10 (t, *J* = 6.0 Hz, CH=CC*H*$_2$, 2H), 4.07 (t, *J* = 6.0 Hz, CH$_2$C*H*$_2$OH, 2H), 4.52-4.60 (m, 2 x NC*H*$_2$, 4H), 6.80 (s, aryl H, 2H), 9.14 (s, C*H*=CCH$_2$, 1H); ^{13}C NMR (100 MHz, CDCl$_3$) δ 14.07, 14.25, 20.92, 22.52, 23.12, 26.04, 27.05, 29.30, 31.13,

46.78, 54.08, 58.86, 130.24, 130.79, 137.12, 138.47, 140.21, 142.65; EI-HRMS *m/z* [M^+] calcd for $C_{12}H_{24}N_3O$ 226.1919, found 226.1908.

[Hex-Bu-C2OH-tr][OTMBS] (b17): 87% yield, light yellow liquid; ^{1}H NMR (400 MHz, $CDCl_3$) δ 0.88 (t, *J* = 6.0 Hz, CH_3, 3H), 0.98 (t, *J* = 6.0 Hz, CH_3, 3H), 1.31-1.48 (m, 4 x CH_2, 8H), 1.90-2.02 (m, 2 x $NCH_2C\mathit{H_2}$, 4H), 2.22 (s, *p*-CH_3, 3H), 2.64 (s, 2 x *o*-CH_3, 6H), 3.07 (t, *J* = 4.0 Hz, $CH{=}CC\mathit{H}_2$, 2H), 4.09 (t, *J* = 4.0 Hz, $CH_2C\mathit{H}_2OH$, 2H), 4.47 (t, *J* = 8.0 Hz, $CNC\mathit{H}_2$, 2H), 4.60 (t, *J* = 6.0 Hz, $CHNC\mathit{H}_2$, 2H), 6.81 (s, aryl H, 2H), 9.20 (s, $C\mathit{H}{=}CCH_2$, 1H); ^{13}C NMR (100 MHz, $CDCl_3$) δ 13.53, 14.07, 19.73, 20.94, 22.54, 23.13, 26.04, 27.13, 29.31, 31.01, 31.12, 51.07, 54.13, 58.69, 130.40, 130.79, 137.17, 138.48, 140.17, 142.67; EI-HRMS *m/z* [M^+] calcd for $C_{14}H_{28}N_3O$ 254.2232, found 254.2224.

[Hex-Pent-C2OH-tr][OTMBS] (b18): 78% yield, light yellow liquid; ^{1}H NMR (400 MHz, $CDCl_3$) δ 0.86-0.93 (m, 2 x CH_3, 6H), 1.28-1.40 (m, 5 x CH_2, 10H), 1.90-2.04 (m, 2 x $NCH_2C\mathit{H_2}$, 4H), 2.22 (s, *p*-CH_3, 3H), 2.65 (s, 2 x *o*-CH_3, 6H), 3.07 (t, *J* = 6.0 Hz, $CH{=}CC\mathit{H}_2$, 2H), 4.08 (t, *J* = 6.0 Hz, $CH_2C\mathit{H}_2OH$, 2H), 4.46 (t, *J* = 6.0 Hz, $CNC\mathit{H}_2$, 2H), 4.60 (t, *J* = 8.0 Hz, $CHNC\mathit{H}_2$, 2H), 6.81 (s, aryl H, 2H), 9.23 (s, $C\mathit{H}{=}CCH_2$, 1H); ^{13}C NMR (100 MHz, $CDCl_3$) δ 13.89, 14.06, 20.90, 22.14, 22.52, 23.16, 26.01, 27.12, 28.45, 28.75, 29.30, 31.11, 51.29, 54.05, 58.75, 130.36, 130.73,

137.05, 138.25, 140.54, 142.72; EI-HRMS *m/z* [M^+] calcd for $C_{15}H_{30}N_3O$ 268.2389, found 268.2379.

[Hex-Hex-C2OH-tr][OTMBS] (b19): 82% yield, light yellow liquid; ^1H NMR (400 MHz, $CDCl_3$) δ 0.86-0.91 (m, 2 x CH_3, 6H), 1.30-1.32 (m, 6 x CH_2, 12H), 1.93-2.02 (m, 2 x NCH_2CH_2, 4H), 2.22 (s, *p*-CH_3, 3H), 2.62 (s, 2 x *o*-CH_3, 6H), 3.08 (t, *J* = 6.0 Hz, CH=CCH_2, 2H), 4.09 (t, *J* = 6.0 Hz, CH_2CH_2OH, 2H), 4.46 (t, *J* = 8.0 Hz, CNCH_2, 2H), 4.60 (t, *J* = 8.0 Hz, CHNCH_2, 2H), 6.80 (s, aryl H, 2H), 9.19 (s, C*H*=CCH_2, 1H); ^{13}C NMR (100 MHz, $CDCl_3$) δ 14.07, 14.07, 20.94, 22.57, 22.57, 23.10, 26.04, 26.11, 27.10, 29.03, 29.30, 31.13, 31.16, 51.32, 54.13, 58.68, 130.35, 130.85, 137.22, 138.70, 139.79, 142.61; EI-HRMS *m/z* [M^+] calcd for $C_{16}H_{32}N_3O$ 282.2545, found 282.2540.

[Hex-Oct-C2OH-tr][OTMBS] (b20): 80% yield, light yellow liquid; ^1H NMR (400 MHz, $CDCl_3$) δ 0.87-0.90 (m, 2 x CH_3 , 6H), 1.26-1.34 (m, 8 x CH, 16H), 1.93-2.02 (m, 2 x NCH_2CH_2, 4H), 2.22 (s, *p*-CH_3, 3H), 2.63 (s, 2 x *o*-CH_3, 6H), 3.07 (t, *J* = 6.0 Hz, CH=CCH_2, 2H), 4.10 (t, *J* = 6.0 Hz, CH_2CH_2OH, 2H), 4.46 (t, *J* = 6.0 Hz, CNCH_2, 2H), 4.61 (t, *J* = 8.0 Hz, CHNCH_2, 2H), 6.81 (s, aryl H, 2H), 9.21 (s, C*H*=CCH_2, 1H); ^{13}C NMR (100 MHz, $CDCl_3$) δ 14.08, 14.23, 20.94, 22.54, 22.76, 23.13, 26.04, 26.45, 27.10, 29.02, 29.08, 29.15, 29.30, 31.14, 31.84, 51.31, 54.11,

58.73, 130.36, 130.82, 137.17, 138.55, 140.04, 142.62; EI-HRMS *m/z* [M^+] calcd for $C_{18}H_{36}N_3O$ 310.2858, found 310.2846.

[Oct-Et-C2OH-tr][OTMBS] (b21): 61% yield, light yellow liquid; ^{1}H NMR (400 MHz, $CDCl_3$) δ 0.88 (t, *J* = 6.0 Hz, CH_3, 3H), 1.25-1.32 (m, 5 x CH_2, 10H), 1.64 (t, *J* = 6.0 Hz, CH_3, 3H), 1.97-2.03 (m, NCH_2CH_2, 2H), 2.22 (s, *p*-CH_3, 3H), 2.64 (s, 2 x *o*-CH_3, 6H), 3.09 (t, *J* = 6.0 Hz, $CH{=}CCH_2$, 2H), 4.10 (t, *J* = 6.0 Hz, CH_2CH_2OH, 2H), 4.52-4.62 (m, 2 x NCH_2, 4H), 6.81 (s, aryl H, 2H), 9.16 (s, $CH{=}CCH_2$, 1H); ^{13}C NMR (100 MHz, $CDCl_3$) δ 14.22, 14.23, 20.90, 22.74, 23.13, 26.36, 27.02, 28.95, 29.14, 29.34, 31.83, 46.77, 54.02, 58.91, 130.16, 130.75, 137.06, 138.36, 140.41, 142.65; EI-HRMS *m/z* [M^+] calcd for $C_{14}H_{28}N_3O$ 254.2232, found 254.2229.

[Oct-Bu-C2OH-tr][OTMBS] (b22): 77% yield, light yellow liquid; ^{1}H NMR (400 MHz, $CDCl_3$) δ 0.88 (t, *J* = 6.0 Hz, CH_3, 3H), 0.98 (t, *J* = 8.0 Hz, CH_3, 3H), 1.25-1.31 (m, 6 x CH_2, 12H), 1.90-2.01 (m, 2 x NCH_2CH_2, 4H), 2.22 (s, *p*-CH_3, 3H), 2.65 (s, 2 x *o*-CH_3, 6H), 3.07 (t, *J* = 6.0 Hz, $CH{=}CCH_2$, 2H), 4.08 (t, *J* = 6.0 Hz, CH_2CH_2OH, 2H), 4.47 (t, *J* = 8.0 Hz, $CNCH_2$, 2H), 4.60 (t, *J* = 8.0 Hz, $CHNCH_2$, 2H), 6.81 (s, aryl H, 2H), 9.21 (s, $CH{=}CCH_2$, 1H); ^{13}C NMR (100 MHz, $CDCl_3$) δ 13.52, 14.23, 19.70, 20.91, 22.75, 23.16, 26.36, 27.13, 28.95, 29.17, 29.35, 30.99, 31.83, 51.07, 54.06, 58.76, 130.34, 130.77, 137.08, 138.35, 140.45, 142.76; EI-HRMS *m/z* [M^+] calcd for $C_{16}H_{32}N_3O$ 282.2545, found 282.2533.

[Oct-Pent-C2OH-tr][OTMBS] (b23): 58% yield, light yellow liquid; ^{1}H NMR (400 MHz, $CDCl_3$) δ 0.86-0.93 (m, 2 x CH_3 , 6H), 1.25-1.37 (m, 7 x CH_2, 14H), 1.92-2.02 (m, 2 x NCH_2CH_2 , 4H), 2.22 (s, *p*-CH_3, 3H), 2.64 (s, 2 x *o*-CH_3, 6H), 3.07 (t, J = 4.0 Hz, $CH{=}CCH_2$, 2H), 4.09 (t, J = 4.0 Hz, CH_2CH_2OH, 2H), 4.46 (t, J = 6.0 Hz, $CNCH_2$, 2H), 4.60 (t, J = 8.0 Hz, $CHNCH_2$, 2H), 6.81 (s, aryl H, 2H), 9.23 (s, $CH{=}CCH_2$, 1H); ^{13}C NMR (100 MHz, $CDCl_3$) δ 13.91, 14.23, 20.92, 22.16, 22.76, 23.18, 26.38, 27.16, 28.48, 28.78, 28.97, 29.08, 29.37, 31.85, 51.30, 54.09, 58.72, 130.43, 130.75, 137.08, 138.25, 140.59, 142.73; EI-HRMS *m/z* [M^+] calcd for $C_{17}H_{34}N_3O$ 296.2702, found 296.2689.

[Oct-Hex-C2OH-tr][OTMBS] (b24): 59% yield, light yellow liquid; ^{1}H NMR (400 MHz, $CDCl_3$) δ 0.88-0.91 (m, 2 x CH_3 , 6H), 1.25-1.33 (m, 8 x CH_2, 16H), 1.93-2.02 (m, 2 x NCH_2CH_2 , 4H), 2.22 (s, *p*-CH_3, 3H), 2.64 (s, 2 x *o*-CH_3, 6H), 3.07 (t, J = 6.0 Hz, $CH{=}CCH_2$, 2H), 4.10 (t, J = 6.0 Hz, CH_2CH_2OH, 2H), 4.46 (t, J = 8.0 Hz, $CNCH_2$, 2H), 4.61 (t, J = 8.0 Hz, $CHNCH_2$, 2H), 6.81 (s, aryl H, 2H), 9.22 (s, $CH{=}CCH_2$, 1H); ^{13}C NMR (100 MHz, $CDCl_3$) δ 14.07, 14.25, 20.95, 22.53, 22.78, 23.15, 26.12, 26.40, 27.16, 28.99, 29.06, 29.19, 29.37, 31.17, 31.87, 51.33, 54.16, 58.66, 130.45, 130.82, 137.18, 138.52, 140.13, 142.66; EI-HRMS *m/z* [M^+] calcd for $C_{18}H_{36}N_3O$ 310.2858, found 310.2560.

[Oct-Oct-C2OH-tr][OTMBS] (b25): 68% yield, light yellow liquid; ^{1}H NMR (400 MHz, $CDCl_3$) δ 0.88-0.90 (m, 2 x CH_3 , 6H), 1.26-1.34 (m, 10 x CH_2, 20H), 1.93-2.02 (m, 2 x NCH_2CH_2, 4H), 2.22 (s, *p*-CH_3, 3H), 2.65 (s, 2 x *o*-CH_3, 6H), 3.07 (t, J = 6.0 Hz, CH=CCH_2, 2H), 4.09 (t, J = 6.0 Hz, CH_2CH_2OH, 2H), 4.46 (t, J = 6.0 Hz, CNCH_2, 2H), 4.60 (t, J = 8.0 Hz, CHNCH_2, 2H), 6.81 (s, aryl H, 2H), 9.23 (s, C*H*=CCH_2, 1H); ^{13}C NMR (100 MHz, $CDCl_3$) δ 14.23, 14.23, 20.93, 22.76, 22.76, 23.18, 26.39, 26.45, 27.12, 28.99, 29.01, 29.09, 29.15, 29.19, 29.37, 31.83, 31.86, 51.31, 54.12, 58.70, 130.41, 130.77, 137.08, 138.30, 140.47, 142.62; EI-HRMS *m/z* [M^+] calcd for $C_{20}H_{40}N_3O$ 338.3171, found 338.3160.

[Bu-*i*-Hex-C2OH-tr][OTMBS] (b26): 93% yield, light yellow liquid; ^{1}H NMR (400 MHz, $CDCl_3$) δ 0.89 (d, J = 6.0 Hz, 2 x CHCH_3, 6H), 0.95 (t, J = 8.0 Hz, CH_3, 3H), 1.18-1.25 (m, CH_2, 2H), 1.31-1.40 (m, CH_2, 2H), 1.53-1.63 (m, C*H*CH_3, 1H), 1.89-2.01 (m, 2 x NCH_2CH_2, 4H), 2.22 (s, *p*-CH_3, 3H), 2.63 (s, 2 x *o*-CH_3, 6H), 3.07 (t, J = 6.0 Hz, CH=CCH_2, 2H), 4.05 (t, J = 6.0 Hz, CH_2CH_2OH, 2H), 4.44 (t, J = 8.0 Hz, CNCH_2, 2H), 4.60 (t, J = 6.0 Hz, CHNCH_2, 2H), 6.81 (s, aryl H, 2H), 9.19 (s, C*H*=CCH_2, 1H); ^{13}C NMR (100 MHz, $CDCl_3$) δ 13.47, 19.63, 20.93, 22.47, 23.15, 27.03, 27.11, 27.68, 31.24, 35.37, 51.53, 53.86, 58.70, 130.38, 130.79, 137.14, 138.44, 140.25, 142.67; EI-HRMS *m/z* [M^+] calcd for $C_{14}H_{28}N_3O$ 254.2232, found 254.2226.

[Pent-*i*-Pent-C2OH-tr][OTMBS] (b27): 80% yield, light yellow liquid; ^{1}H NMR (400 MHz, $CDCl_3$) δ 0.89 (t, *J* = 8.0 Hz, CH_3, 3H), 0.98 (d, *J* = 6.0 Hz, 2 x $CHCH_3$, 6H), 1.27-1.38 (m, 2 x CH_2, 4H), 1.57-1.67 (m, $CHCH_3$, 1H), 1.79-1.85 (m, NCH_2CH_2 , 2H), 1.95-2.03 (m, NCH_2CH_2 , 2H), 2.22 (s, *p*-CH_3, 3H), 2.64 (s, 2 x *o*-CH_3, 6H), 3.07 (t, *J* = 6.0 Hz, $CH{=}CCH_2$, 2H), 4.06 (t, *J* = 6.0 Hz, CH_2CH_2OH, 2H), 4.48 (t, *J* = 6.0 Hz, $CNCH_2$, 2H), 4.58 (t, *J* = 6.0 Hz, $CHNCH_2$, 2H), 6.81 (s, aryl H, 2H), 9.19 (s, $CH{=}CCH_2$, 1H); ^{13}C NMR (100 MHz, $CDCl_3$) δ 13.94, 20.91, 22.10, 22.25, 23.15, 25.74, 27.12, 28.41, 29.04, 37.69, 49.79, 54.05, 58.70, 130.39, 130.75, 137.08, 138.31, 140.45, 142.62; EI-HRMS *m/z* [M^+] calcd for $C_{14}H_{28}N_3O$ 254.2232, found 254.2222.

[Hex-*i*-Bu-C2OH-tr][OTMBS] (b28): 88% yield, light yellow liquid; ^{1}H NMR (400 MHz, $CDCl_3$) δ 0.87 (t, *J* = 8.0 Hz, CH_3, 3H), 0.99 (d, *J* = 6.0 Hz, 2 x $CHCH_3$, 6H), 1.28-1.34 (m, 3 x CH_2, 6H), 1.97-2.04 (m, NCH_2CH_2 , 2H), 2.22 (s, *p*-CH_3, 3H), 2.27-2.33 (m, $CHCH_3$, 1H), 2.59 (s, 2 x *o*-CH_3, 6H), 3.08 (t, *J* = 6.0 Hz, $CH{=}CCH_2$, 2H), 4.09 (t, *J* = 4.0 Hz, CH_2CH_2OH, 2H), 4.30 (d, *J* = 8.0 Hz, $CNCH_2$, 2H), 4.62 (t, *J* = 8.0 Hz, $CHNCH_2$, 2H), 6.80 (s, aryl H, 2H), 9.17 (s, $CH{=}CCH_2$, 1H); ^{13}C NMR (100 MHz, $CDCl_3$) δ 14.06, 19.81, 20.96, 22.52, 23.03, 25.99, 27.17, 29.03, 29.28, 31.09, 54.10, 57.92, 58.84, 130.25, 130.88, 137.37, 139.04, 142.93; EI-HRMS *m/z* [M^+] calcd for $C_{14}H_{28}N_3O$ 254.2232, found 254.2229.

[Oct-*i*-Bu-C2OH-tr][OTMBS] (b29): 78% yield, light yellow liquid; ^{1}H NMR (400 MHz, $CDCl_3$) δ 0.87 (t, *J* = 6.0 Hz, CH_3, 3H), 1.00 (d, *J* = 6.0 Hz, 2 x $CHCH_3$, 6H), 1.2541.31 (m, 5 x CH_2, 10H), 1.98-2.04 (m, NCH_2CH_2 , 2H), 2.22 (s, *p*-CH_3, 3H), 2.27-2.34 (m, $CHCH_3$, 1H), 2.60 (s, 2 x *o*-CH_3, 6H), 3.07 (t, *J* = 6.0 Hz, $CH{=}CCH_2$, 2H), 4.10 (t, *J* = 6.0 Hz, CH_2CH_2OH, 2H), 4.30 (d, *J* = 8.0 Hz, $CNCH_2$, 2H), 4.63 (t, *J* = 6.0 Hz, $CHNCH_2$, 2H), 6.80 (s, aryl H, 2H), 9.18 (s, $CH{=}CCH_2$, 1H); ^{13}C NMR (100 MHz, $CDCl_3$) δ 14.24, 19.82, 20.95, 22.75, 23.04, 26.33, 27.18, 28.94, 29.04, 29.16, 29.33, 31.83, 54.11, 57.92, 58.82, 130.28, 130.87, 137.37, 139.01, 139.09, 142.93; EI-HRMS *m/z* [M^+] calcd for $C_{16}H_{32}N_3O$ 282.2545, found 282.2539.

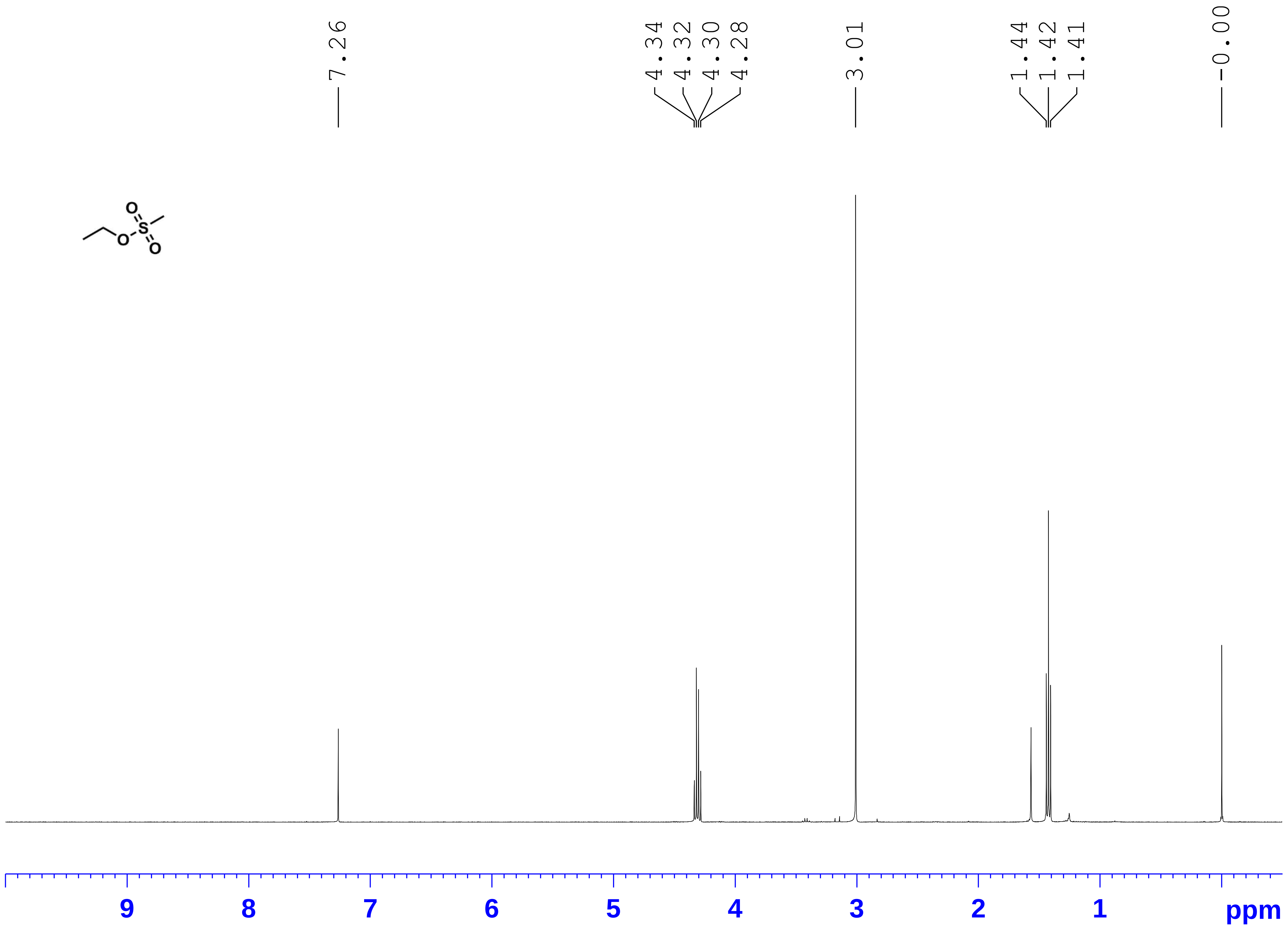

7.26
4.34
4.32
4.30
4.28
3.01
1.44
1.42
1.41
0.00
O
S
O
O
9
8
7
6
5
4
3
2
1
ppm

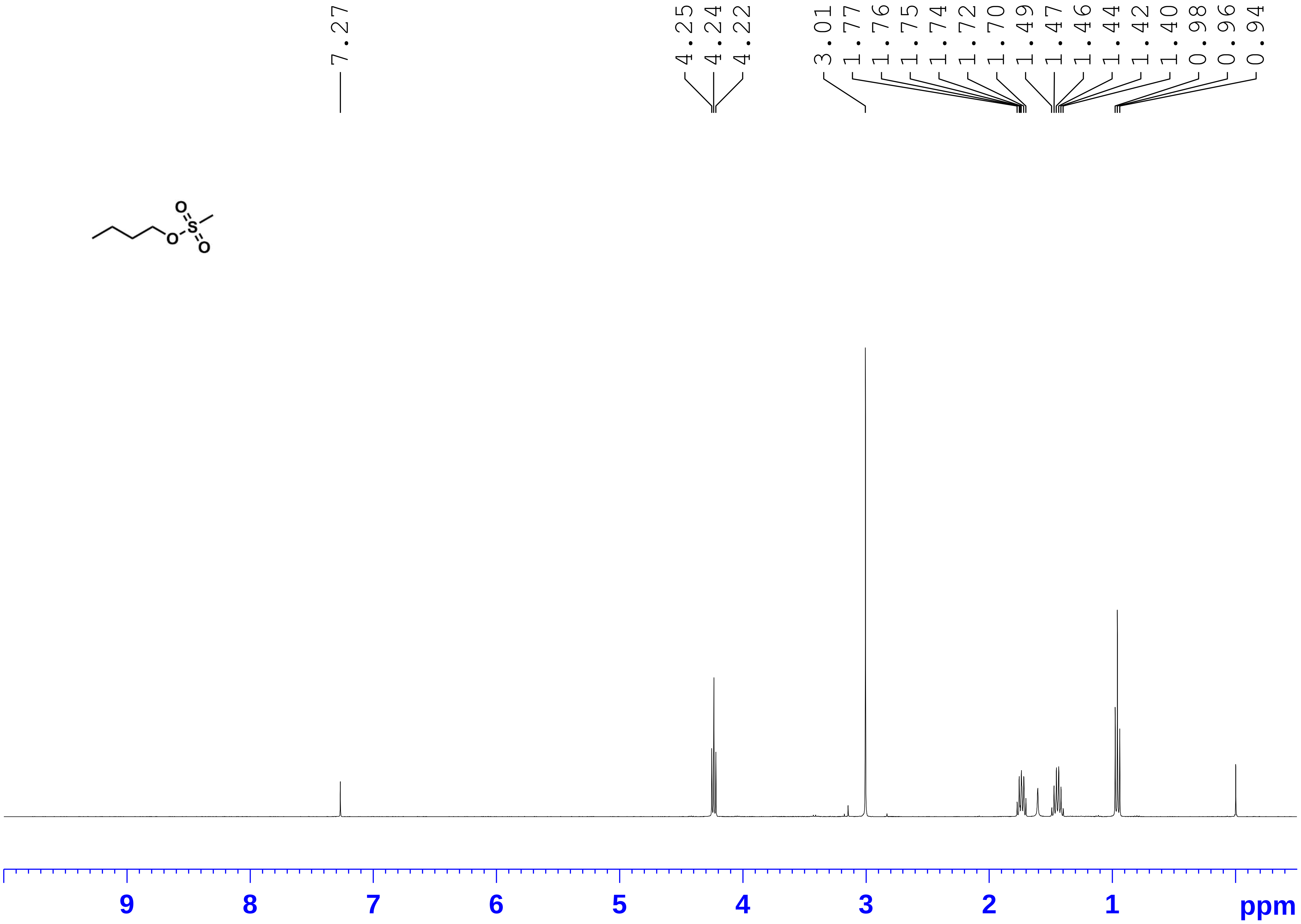
7.27
4.25
4.24
4.22
3.01
1.77
1.76
1.75
1.74
1.72
1.70
1.49
1.47
1.46
1.44
1.42
1.40
0.98
0.96
0.94
O
S
O
O
9
8
7
6
5
4
3
2
1
ppm

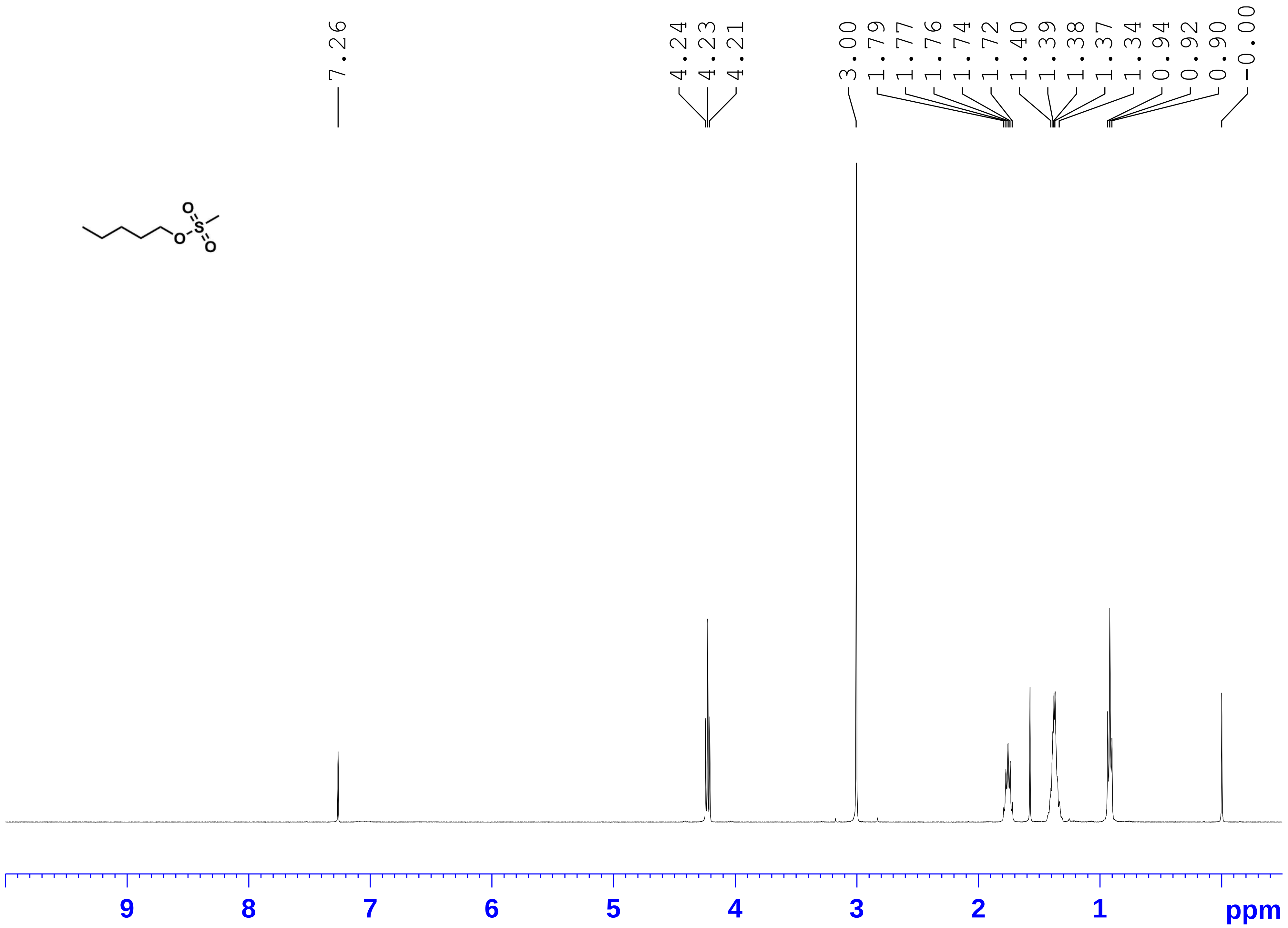

7.26
4.24
4.23
4.21
3.00
1.79
1.77
1.76
1.74
1.72
1.40
1.39
1.38
1.37
1.34
0.94
0.92
0.90
0.00
9
8
7
6
5
4
3
2
1
ppm

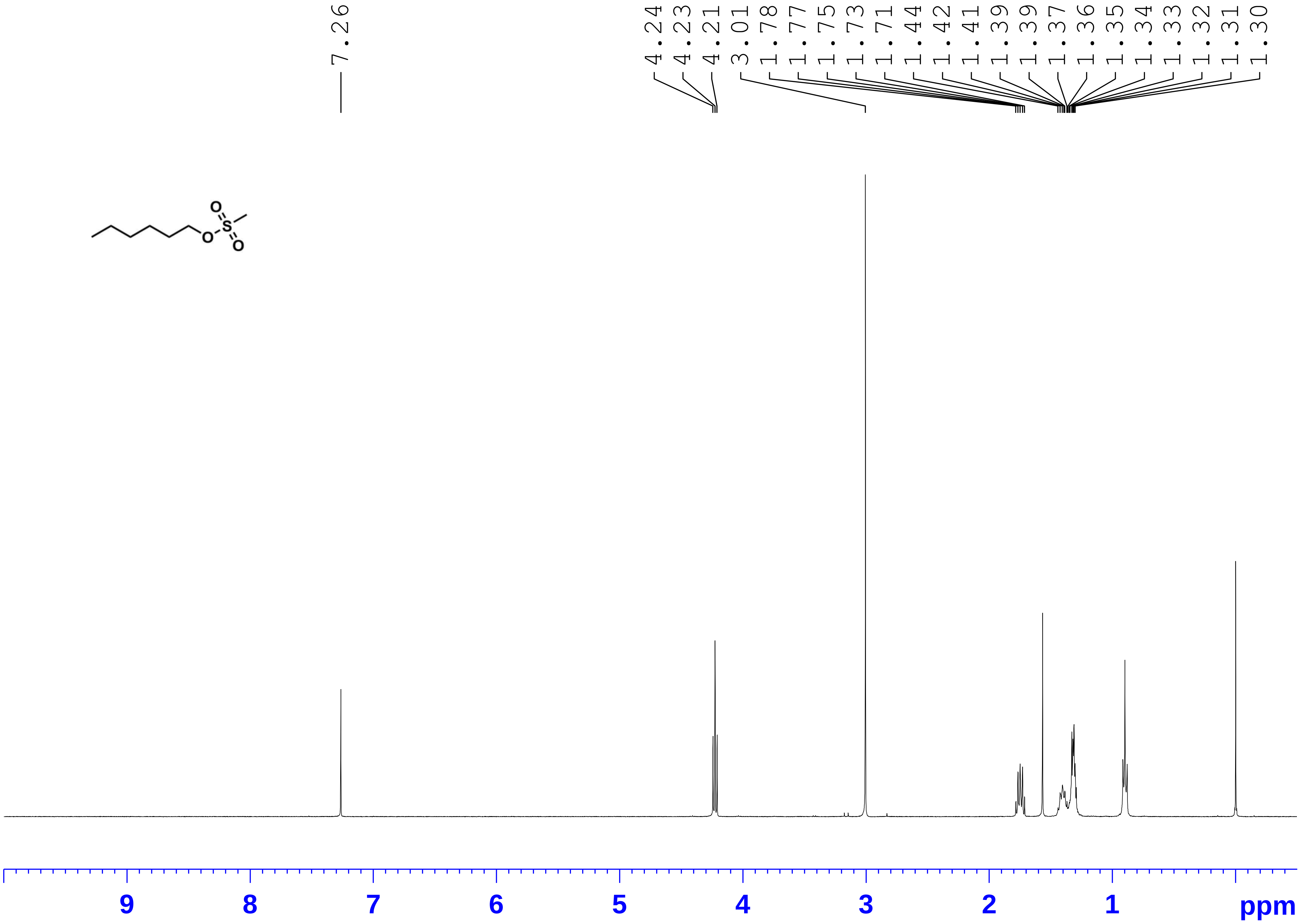
7.26
4.24
4.23
4.21
3.01
1.78
1.77
1.75
1.73
1.71
1.44
1.42
1.41
1.39
1.39
1.37
1.36
1.35
1.34
1.33
1.32
1.31
1.30
O
S
O
O
9
8
7
6
5
4
3
2
1
ppm

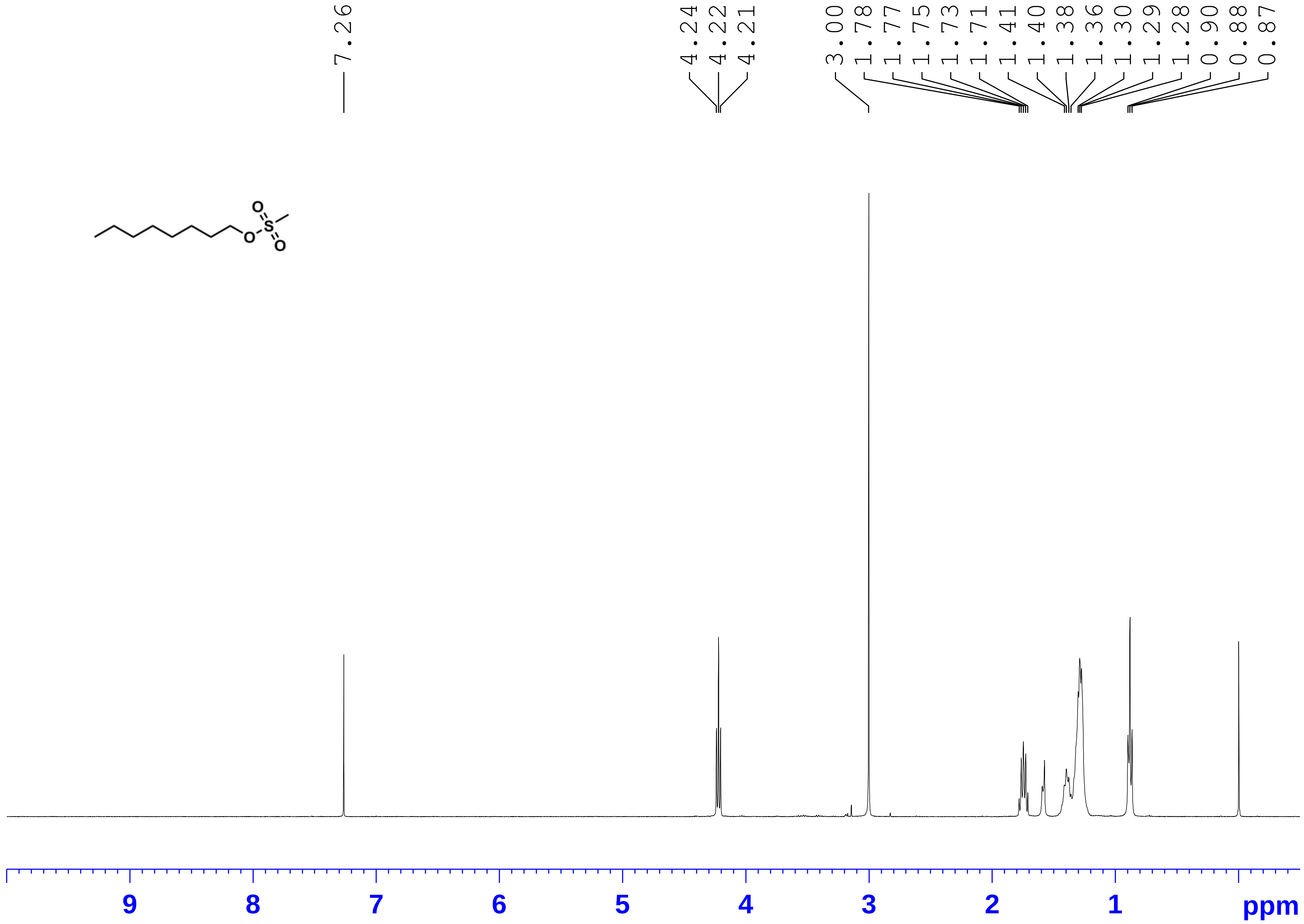
7.26
4.24
4.22
4.21
3.00
1.78
1.77
1.75
1.73
1.71
1.41
1.40
1.38
1.36
1.30
1.29
1.28
0.90
0.88
0.87
O
S
O
O
9
8
7
6
5
4
3
2
1
ppm

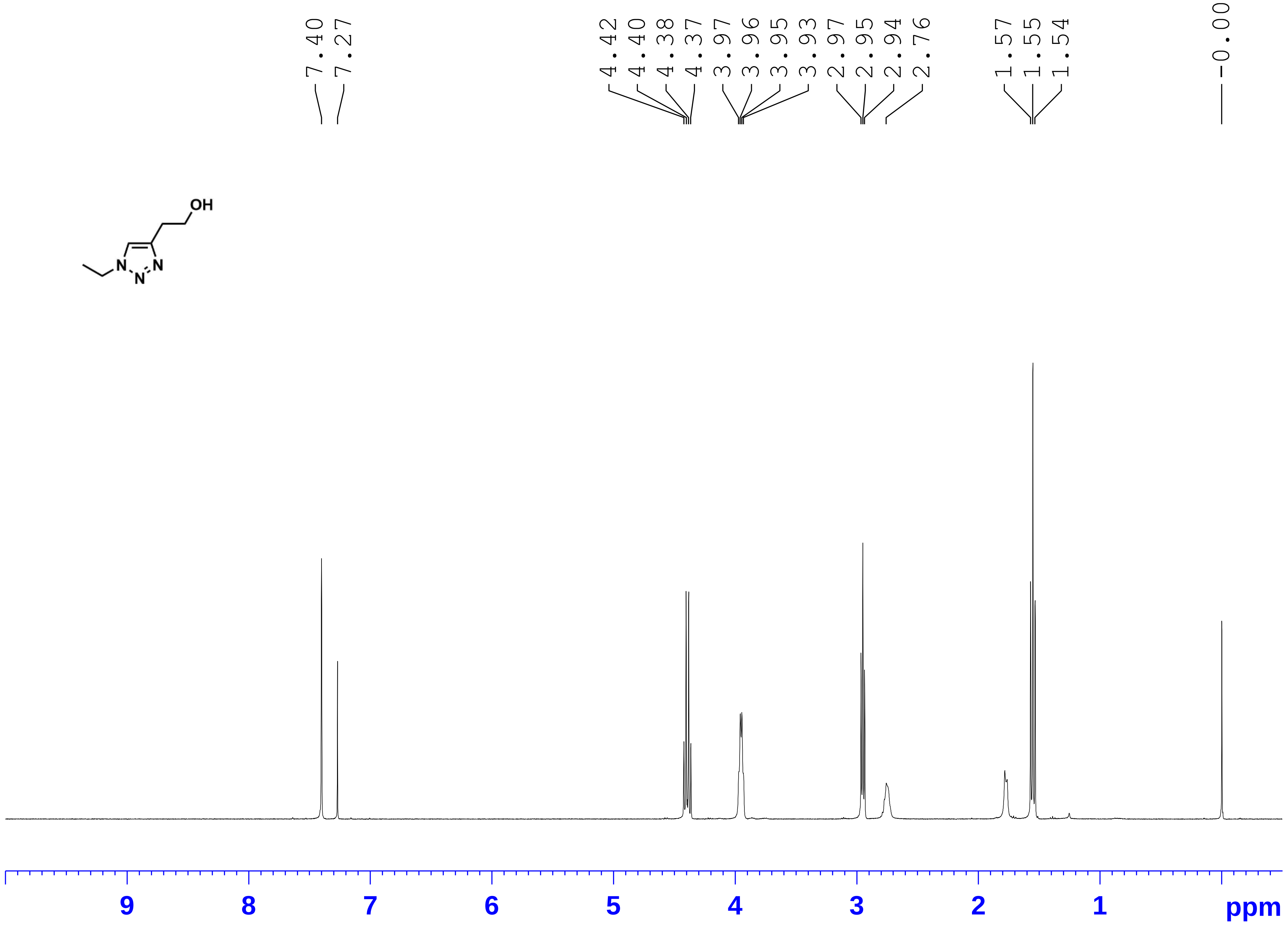

7.40
7.27
4.42
4.40
4.38
4.37
3.97
3.96
3.95
3.93
2.97
2.95
2.94
2.76
1.57
1.55
1.54
0.00
OH
N
N
N
9
8
7
6
5
4
3
2
1
ppm

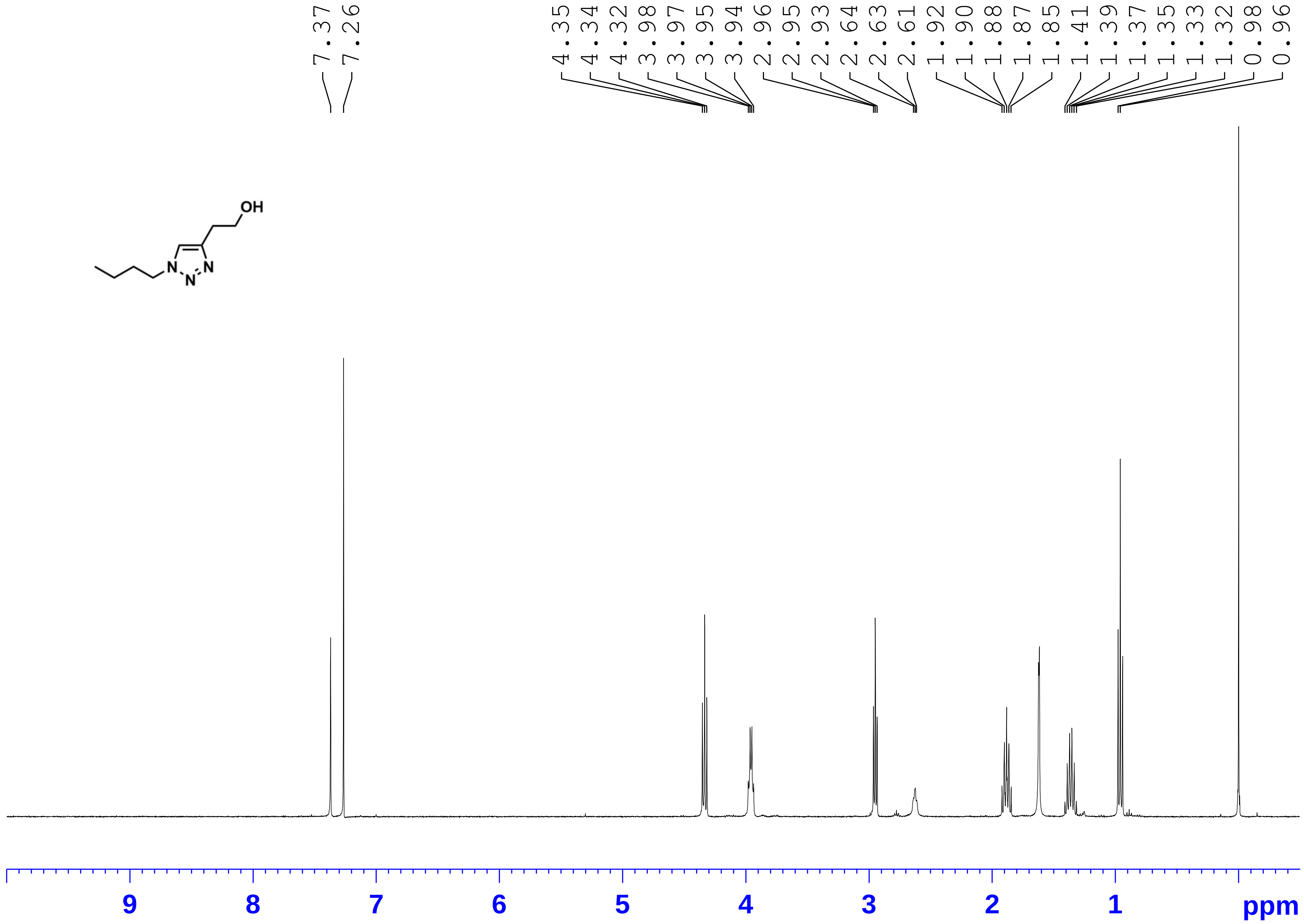
7.37
7.26
4.35
4.34
4.32
3.98
3.97
3.95
3.94
2.96
2.95
2.93
2.64
2.63
2.61
1.92
1.90
1.88
1.87
1.85
1.41
1.39
1.37
1.35
1.33
1.32
0.98
0.96
OH
N
N
N
9
8
7
6
5
4
3
2
1
ppm

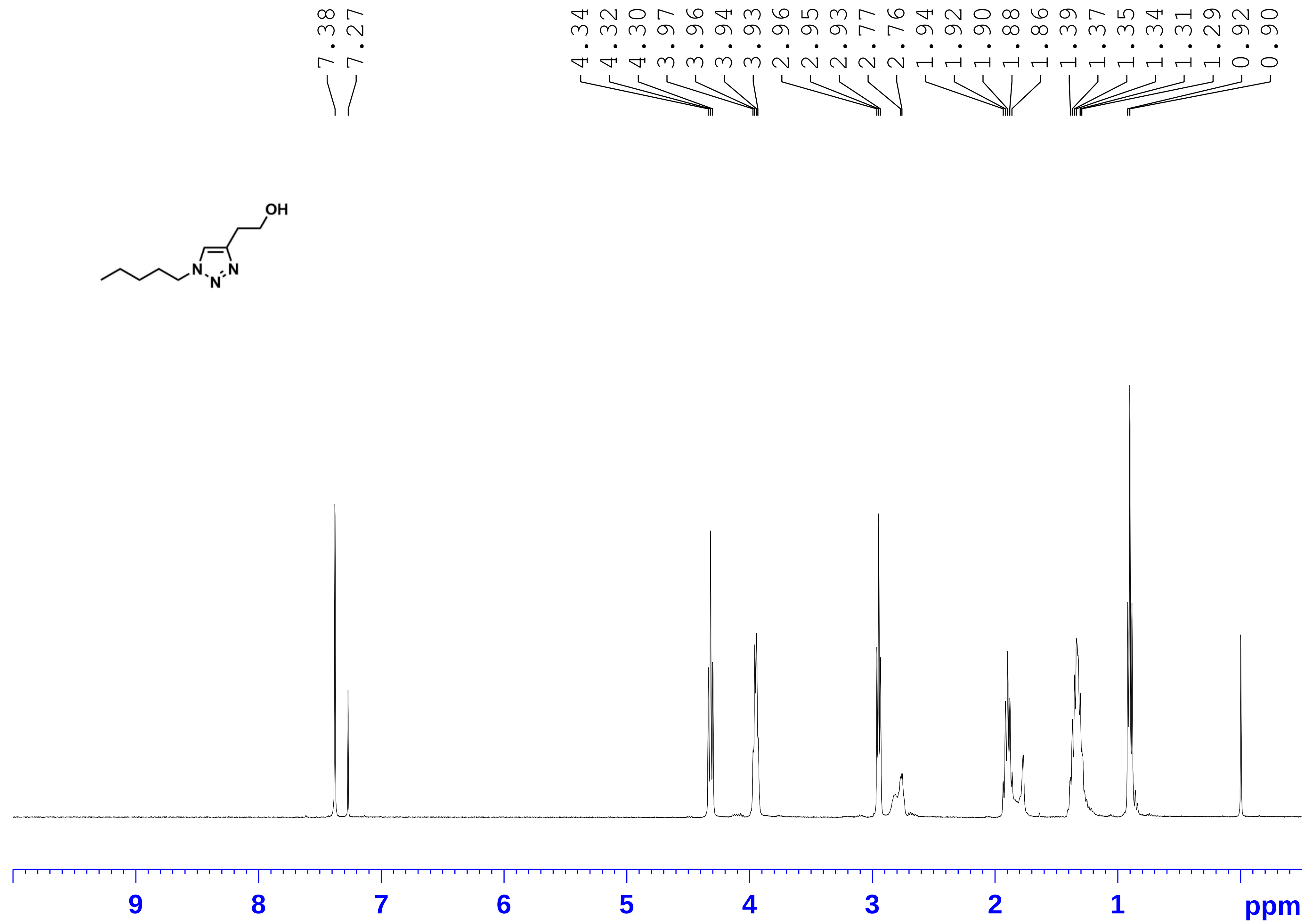
7.38
7.27
4.34
4.32
4.30
3.97
3.96
3.94
3.93
2.96
2.95
2.93
2.77
2.76
1.94
1.92
1.90
1.88
1.86
1.39
1.37
1.35
1.34
1.31
1.29
0.92
0.90
OH
N
N
N
9
8
7
6
5
4
3
2
1
ppm

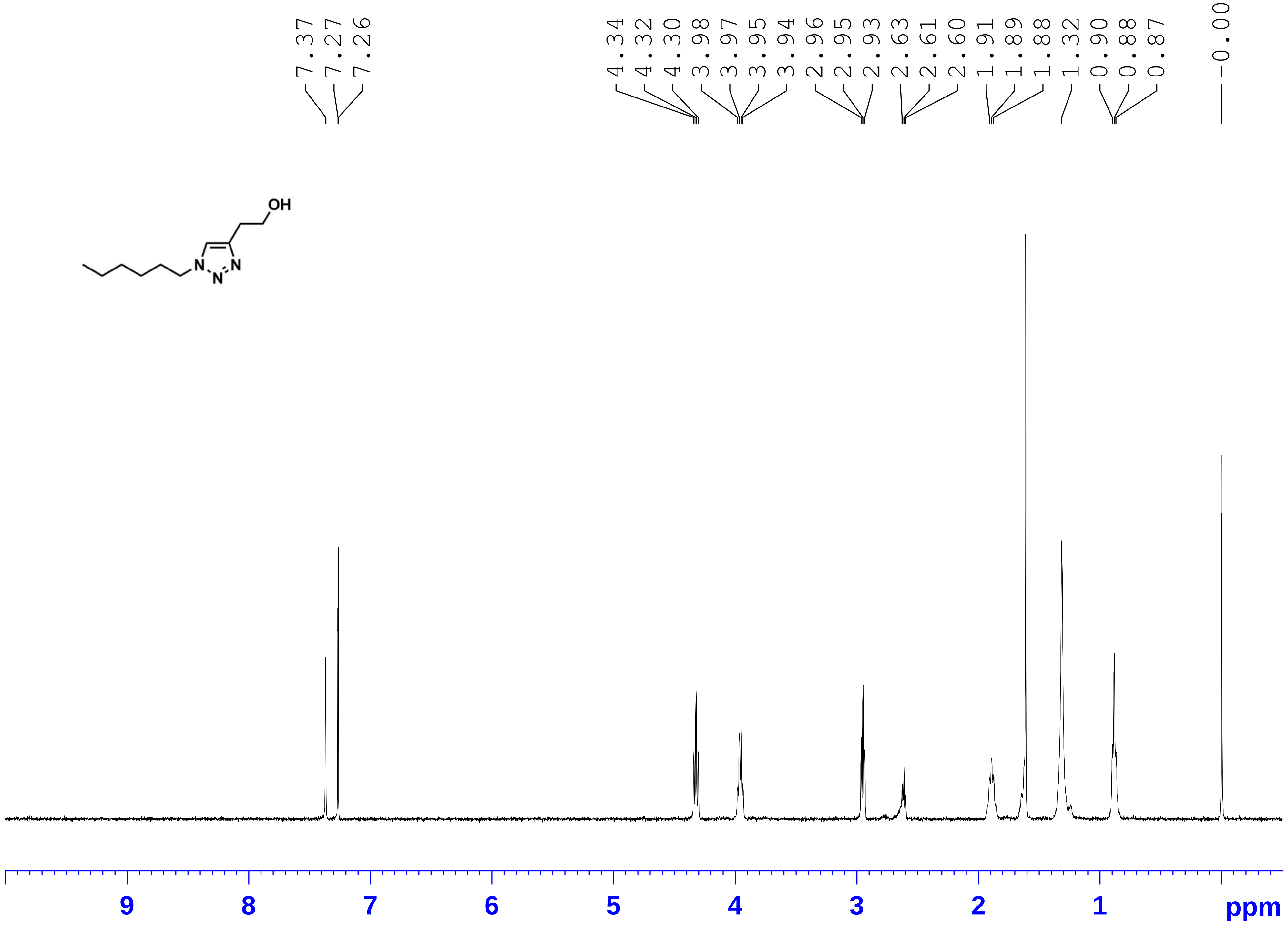
7.37
7.27
7.26
4.34
4.32
4.30
3.98
3.97
3.95
3.94
2.96
2.95
2.93
2.63
2.61
2.60
1.91
1.89
1.88
1.32
0.90
0.88
0.87
0.00
OH
N
N
N
9
8
7
6
5
4
3
2
1
ppm

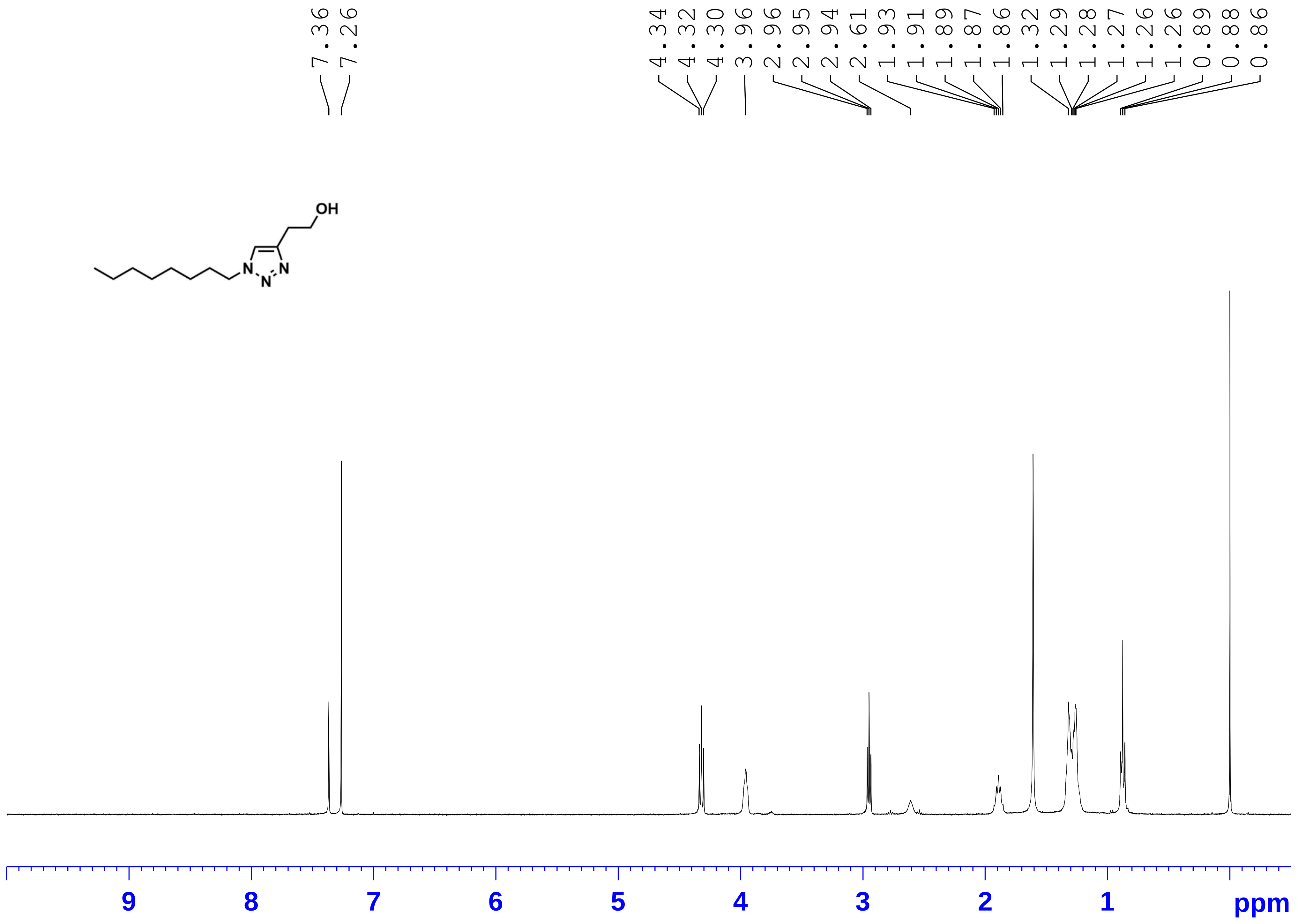

7.36
7.26
4.34
4.32
4.30
3.96
2.96
2.95
2.94
2.61
1.93
1.91
1.89
1.87
1.86
1.32
1.29
1.28
1.27
1.26
1.26
0.89
0.88
0.86
OH
N
N
N
9
8
7
6
5
4
3
2
1
ppm

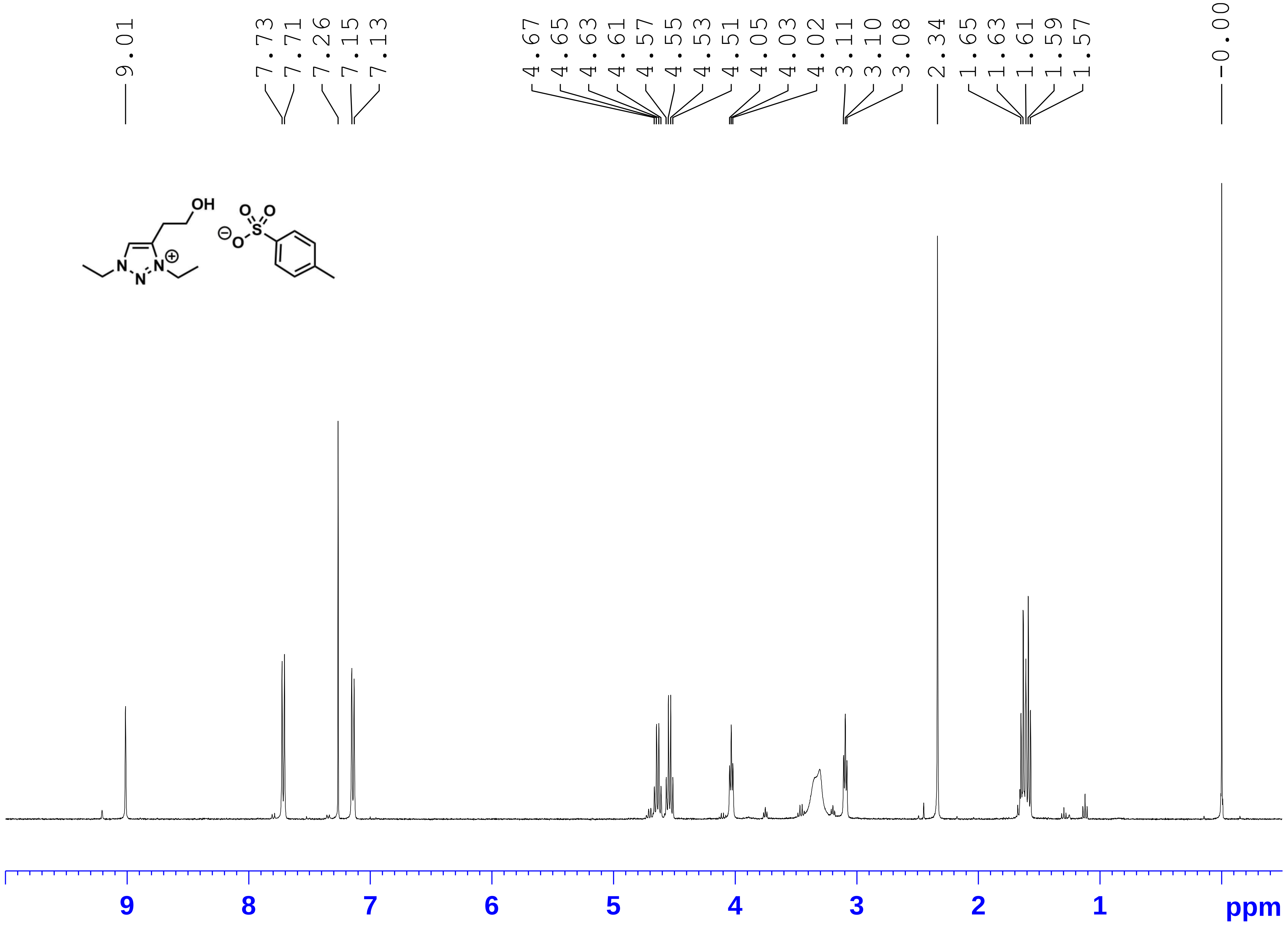
9.01
7.73
7.71
7.26
7.15
7.13
4.67
4.65
4.63
4.61
4.57
4.55
4.53
4.51
4.05
4.03
4.02
3.11
3.10
3.08
2.34
1.65
1.63
1.61
1.59
1.57
0.00
OH
O
S
N
N
N
9
8
7
6
5
4
3
2
1
ppm

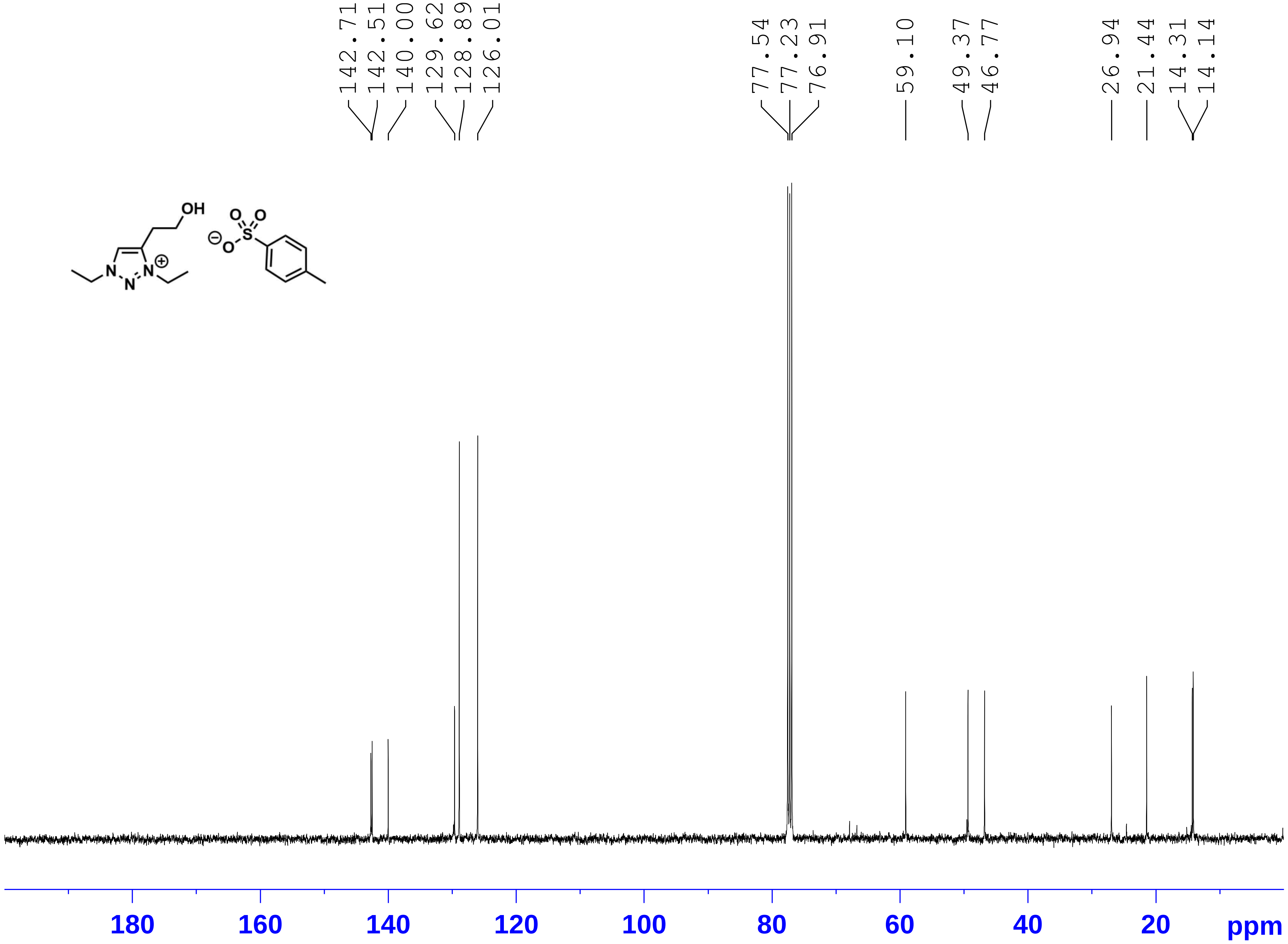
142.71
142.51
140.00
129.62
128.89
126.01
77.54
77.23
76.91
59.10
49.37
46.77
26.94
21.44
14.31
14.14
OH
O
O
S
O
N
N
N
180
160
140
120
100
80
60
40
20
ppm

Spectrum

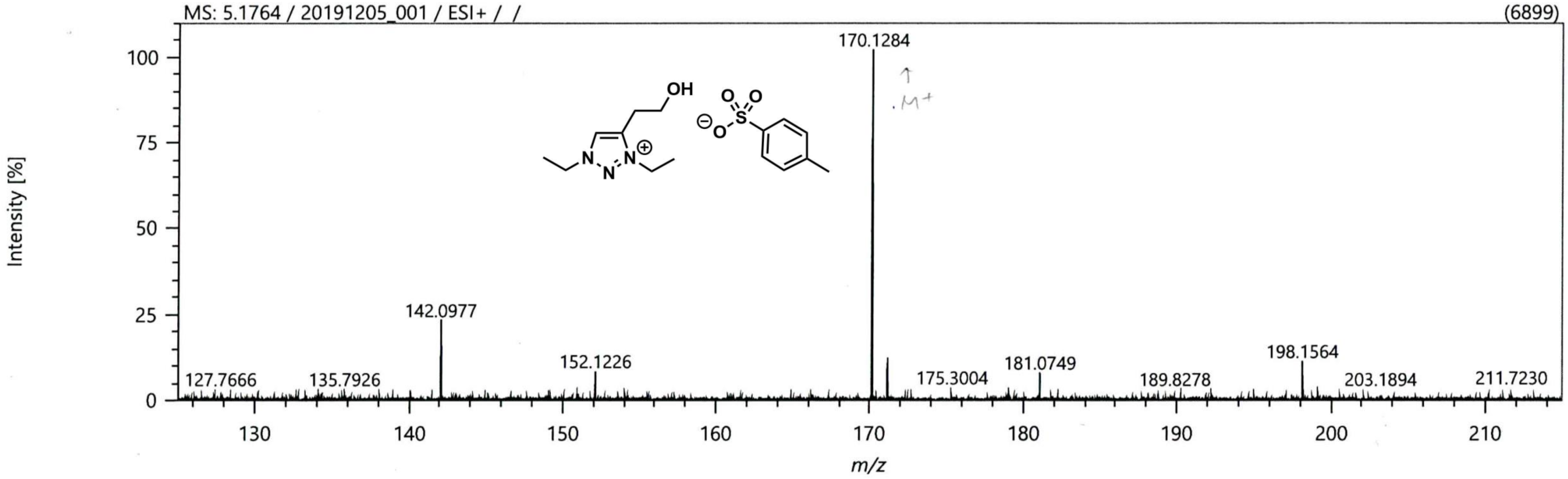

Elemental Composition

Parameters		Elements Set 1:										
Tolerance:	±10.00 ppm	Symbol	C	H	O	Na	N	S	P	Si	F	Cl
Electron:	Odd/Even	Min	0	0	1	0	3	0	0	0	0	0
Charge:	+1	Max	400	1000	1	0	3	0	0	0	0	0
DBE:	-1.5 - 999.0											

Results

Mass	Formula	Calculated Mass	Mass Difference [mDa]	Mass Difference [ppm]	DBE
170.12837	C8 H16 N3 O	170.12879	-0.42	-2.49	2.5

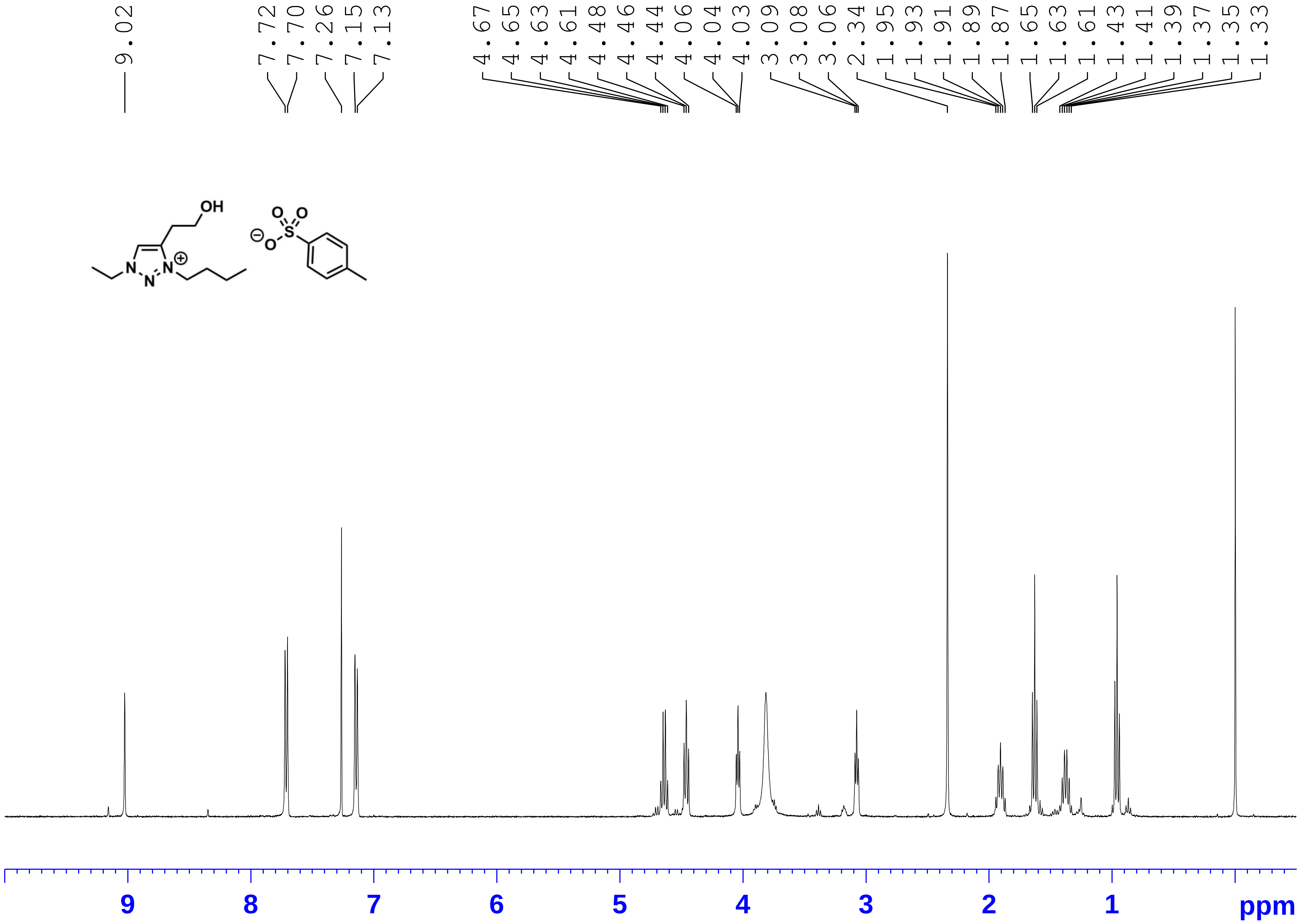
9.02
7.72
7.70
7.26
7.15
7.13
4.67
4.65
4.63
4.61
4.48
4.46
4.44
4.06
4.04
4.03
3.09
3.08
3.06
2.34
1.95
1.93
1.91
1.89
1.87
1.65
1.63
1.61
1.43
1.41
1.39
1.37
1.35
1.33
9
8
7
6
5
4
3
2
1
ppm

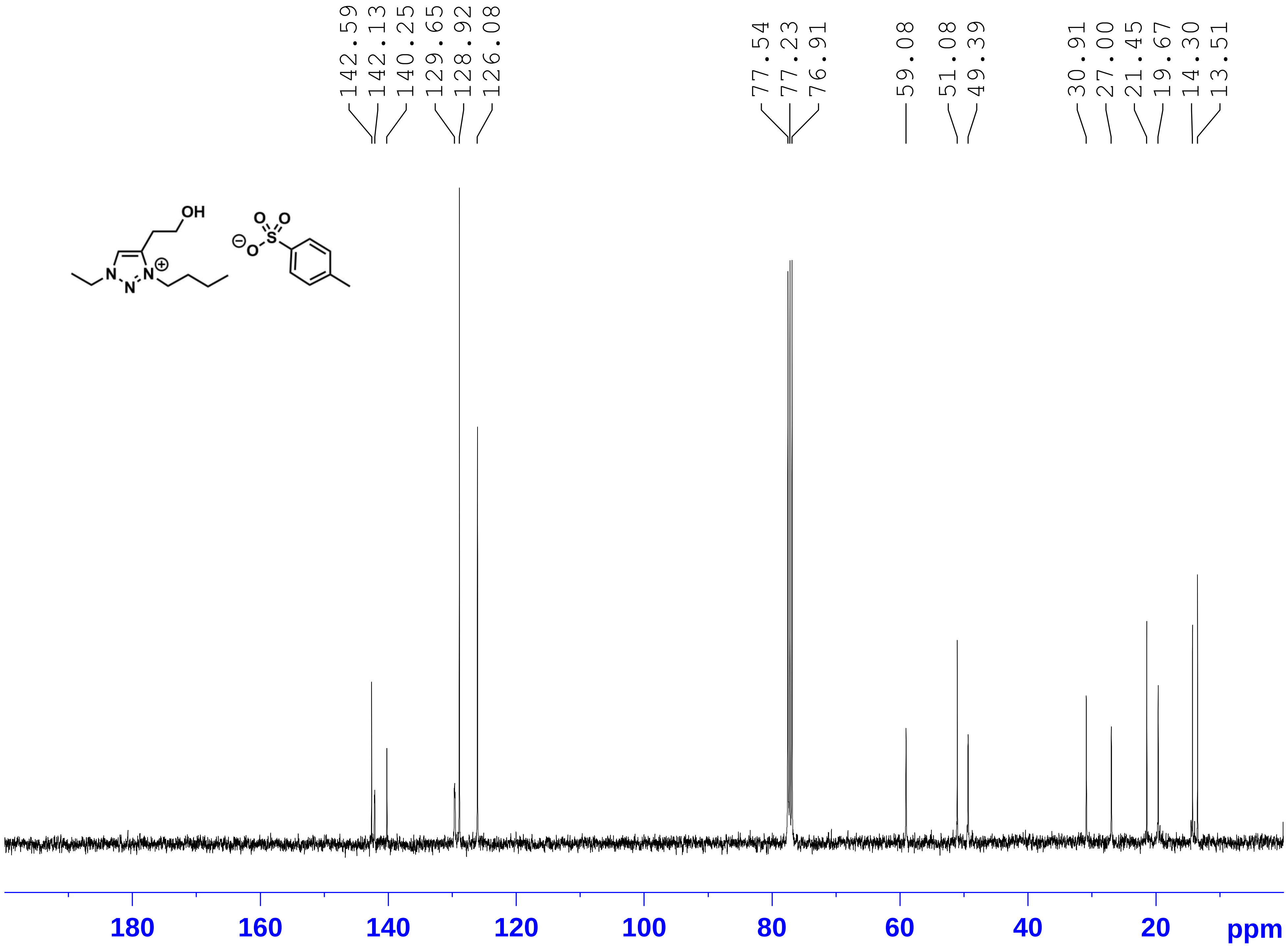

142.59
142.13
140.25
129.65
128.92
126.08
77.54
77.23
76.91
59.08
51.08
49.39
30.91
27.00
21.45
19.67
14.30
13.51
OH
O
O
S
O
N
N
N
180
160
140
120
100
80
60
40
20
ppm

Spectrum

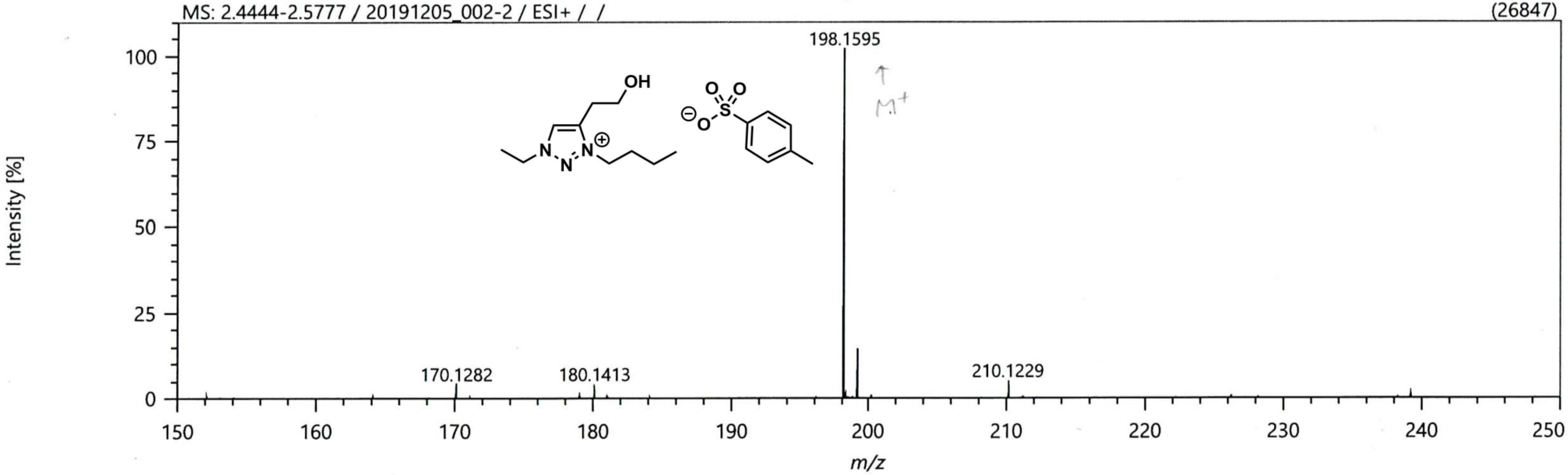

Elemental Composition

Parameters

Tolerance: ±10.00 ppm
Electron: Odd/Even
Charge: +1
DBE: -1.5 - 999.0

Elements Set 1:

Symbol	C	H	O	Na	N	S	P	Si	F	Cl
Min	0	0	1	0	3	0	0	0	0	0
Max	400	1000	1	0	3	0	0	0	0	0

Results

Mass	Formula	Calculated Mass	Mass Difference [mDa]	Mass Difference [ppm]	DBE
198.15954	C10 H20 N3 O	198.16009	-0.55	-2.79	2.5

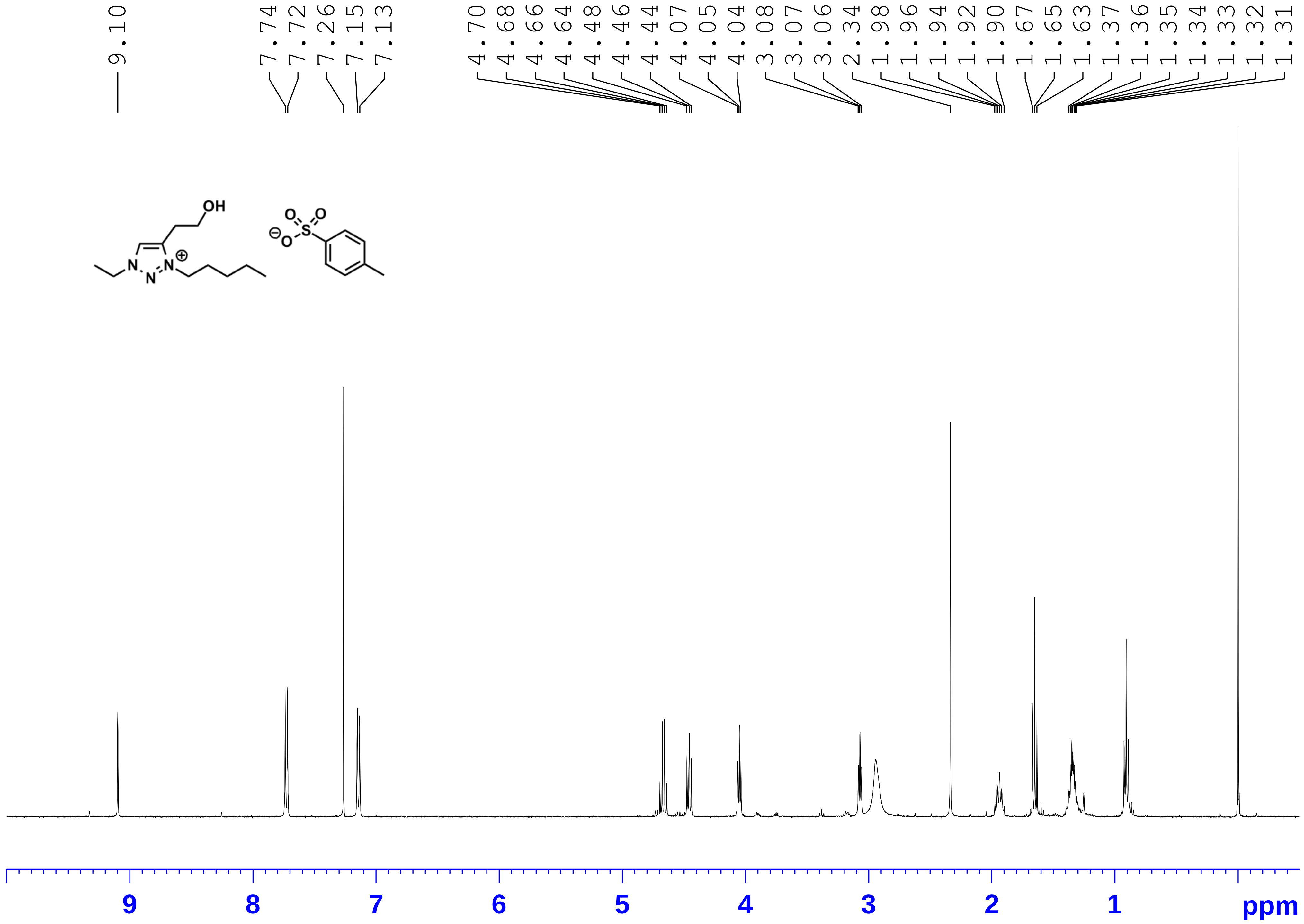

9.10
7.74
7.72
7.26
7.15
7.13
4.70
4.68
4.66
4.64
4.48
4.46
4.44
4.07
4.05
4.04
3.08
3.07
3.06
2.34
1.98
1.96
1.94
1.92
1.90
1.67
1.65
1.63
1.37
1.36
1.35
1.34
1.33
1.32
1.31
OH
N
N
N
O
O
S
O
9
8
7
6
5
4
3
2
1
ppm

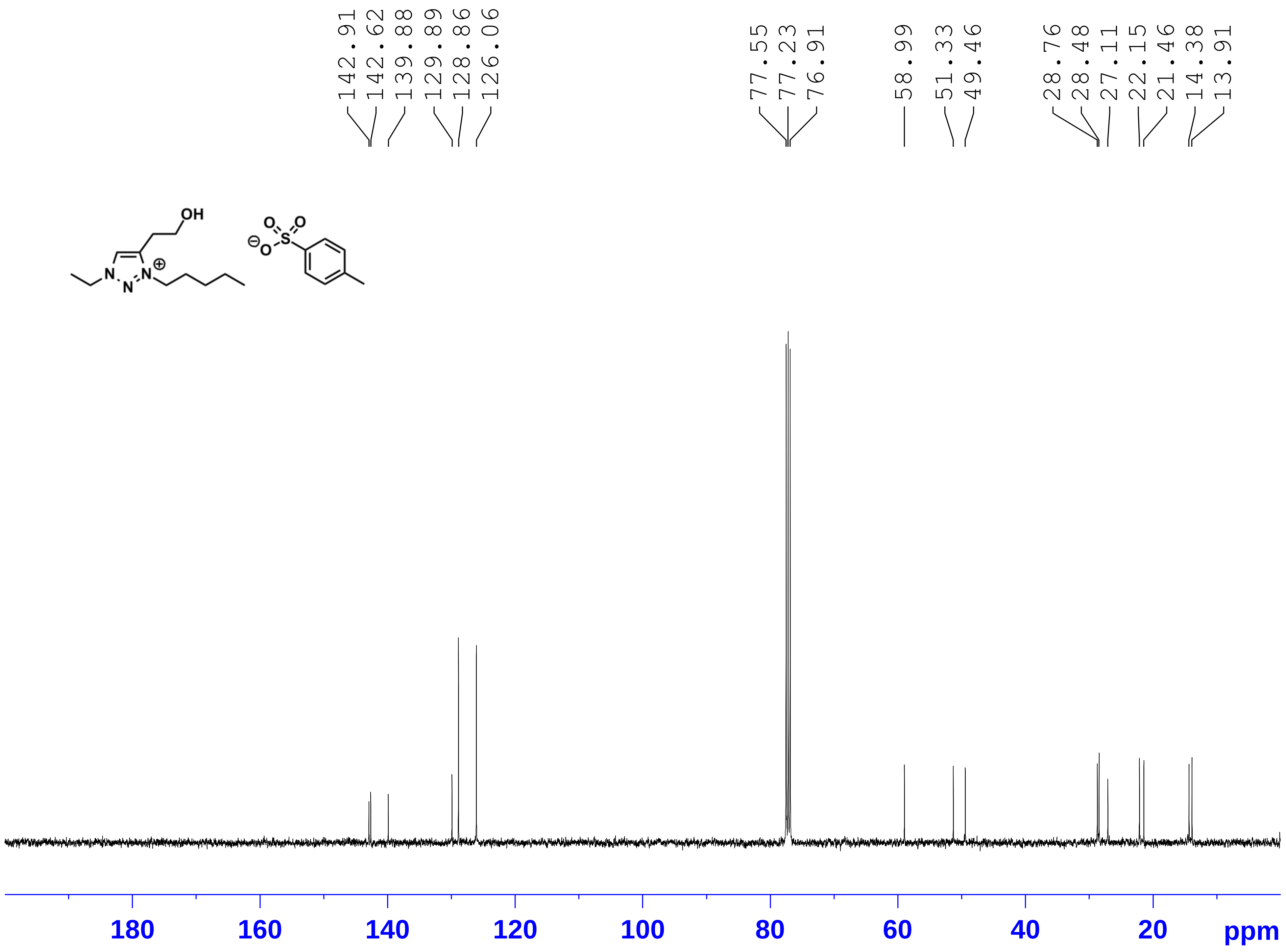

142.91
142.62
139.88
129.89
128.86
126.06
77.55
77.23
76.91
58.99
51.33
49.46
28.76
28.48
27.11
22.15
21.46
14.38
13.91
OH
O
S
N
180
160
140
120
100
80
60
40
20
ppm

Spectrum

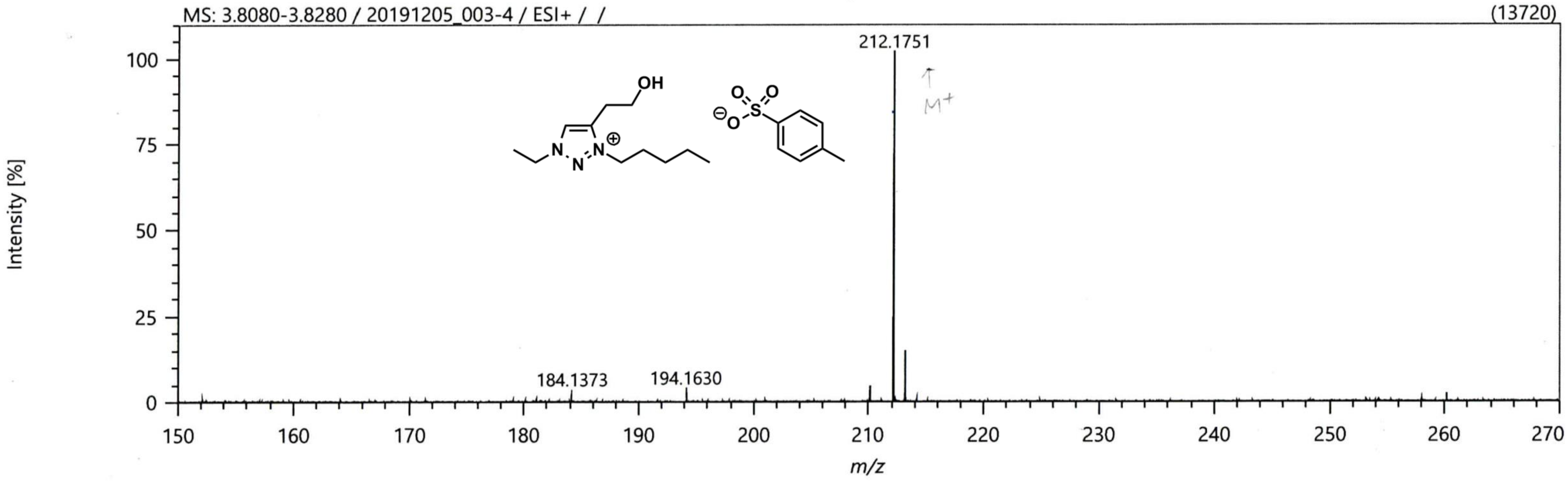

Elemental Composition

Parameters

Tolerance: ±50.00 ppm

Electron: Odd/Even

Charge: +1

DBE: -1.5 - 999.0

Elements Set 1:

Symbol	C	H	O	Na	N	S	P	Si	F	Cl
Min	0	0	1	0	3	0	0	0	0	0
Max	400	1000	1	0	3	0	0	0	0	0

Results

Mass	Formula	Calculated Mass	Mass Difference [mDa]	Mass Difference [ppm]	DBE
212.17511	C11 H22 N3 O	212.17574	-0.62	-2.94	2.5

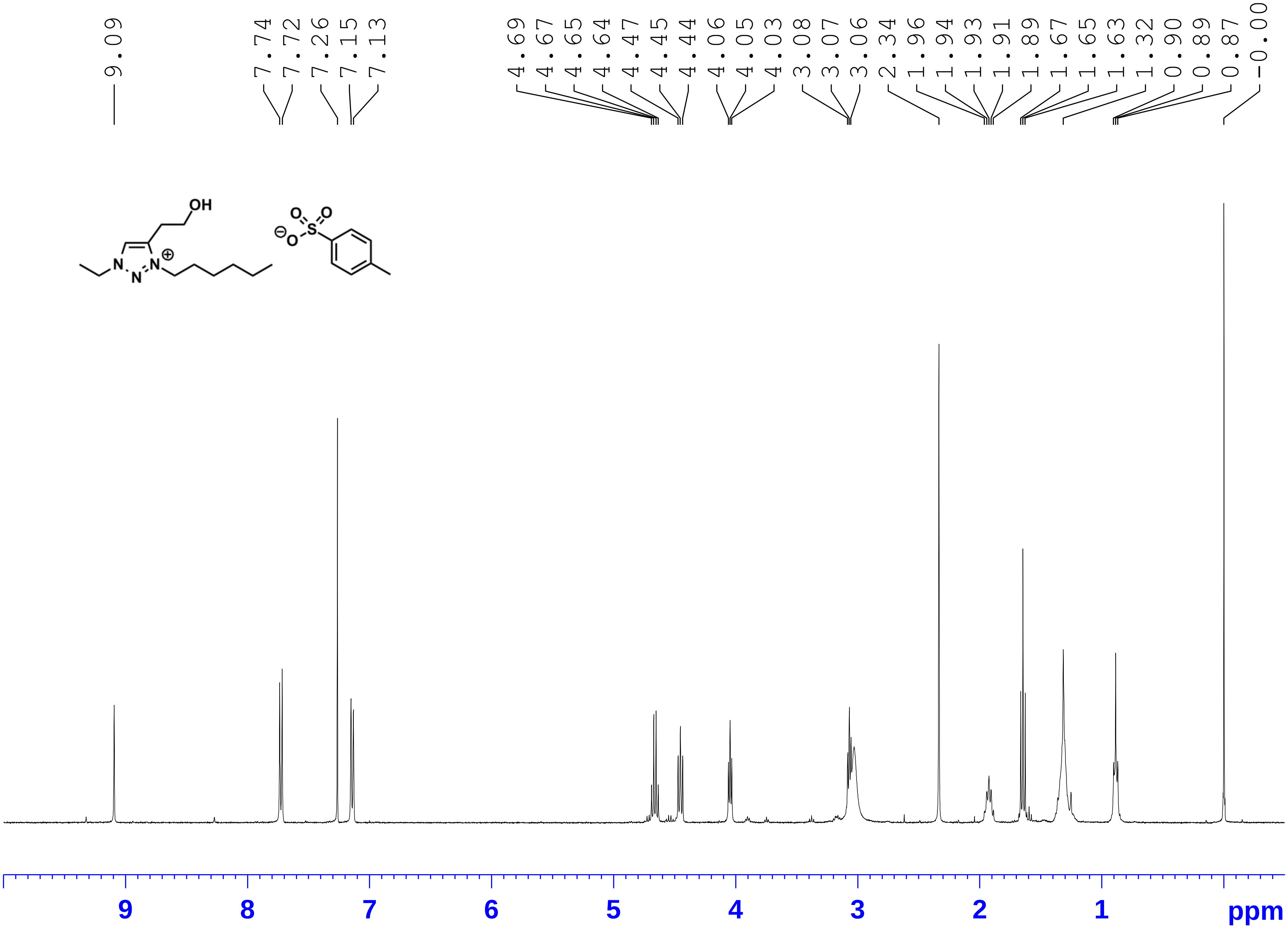
9.09
7.74
7.72
7.26
7.15
7.13
4.69
4.67
4.65
4.64
4.47
4.45
4.44
4.06
4.05
4.03
3.08
3.07
3.06
2.34
1.96
1.94
1.93
1.91
1.89
1.67
1.65
1.63
1.32
0.90
0.89
0.87
-0.00
OH
N
N
N
O
O
S
O
9
8
7
6
5
4
3
2
1
ppm

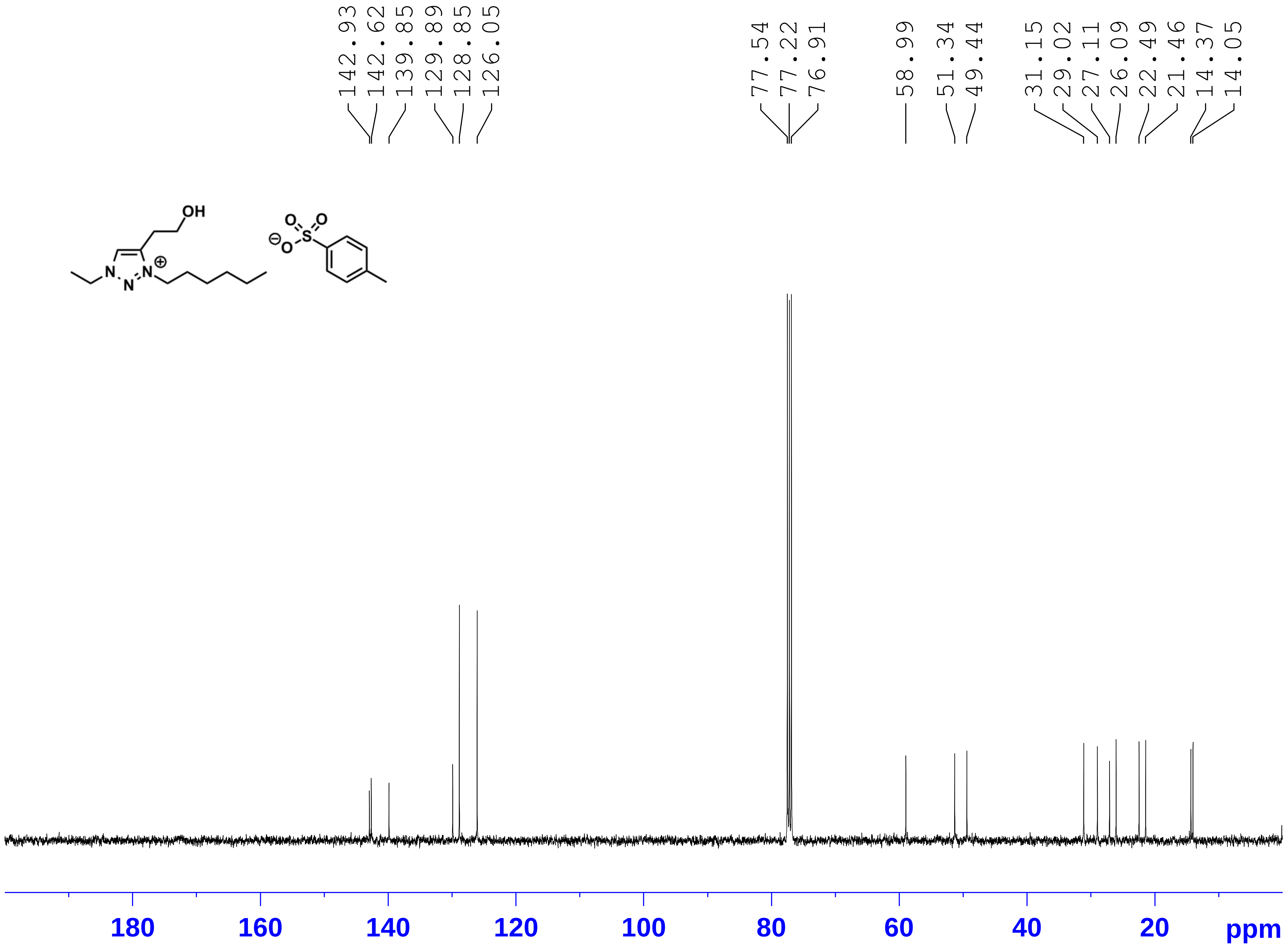
142.93
142.62
139.85
129.89
128.85
126.05
77.54
77.22
76.91
58.99
51.34
49.44
31.15
29.02
27.11
26.09
22.49
21.46
14.37
14.05
OH
N
N
N
S
O
O
O
180
160
140
120
100
80
60
40
20
ppm

Spectrum

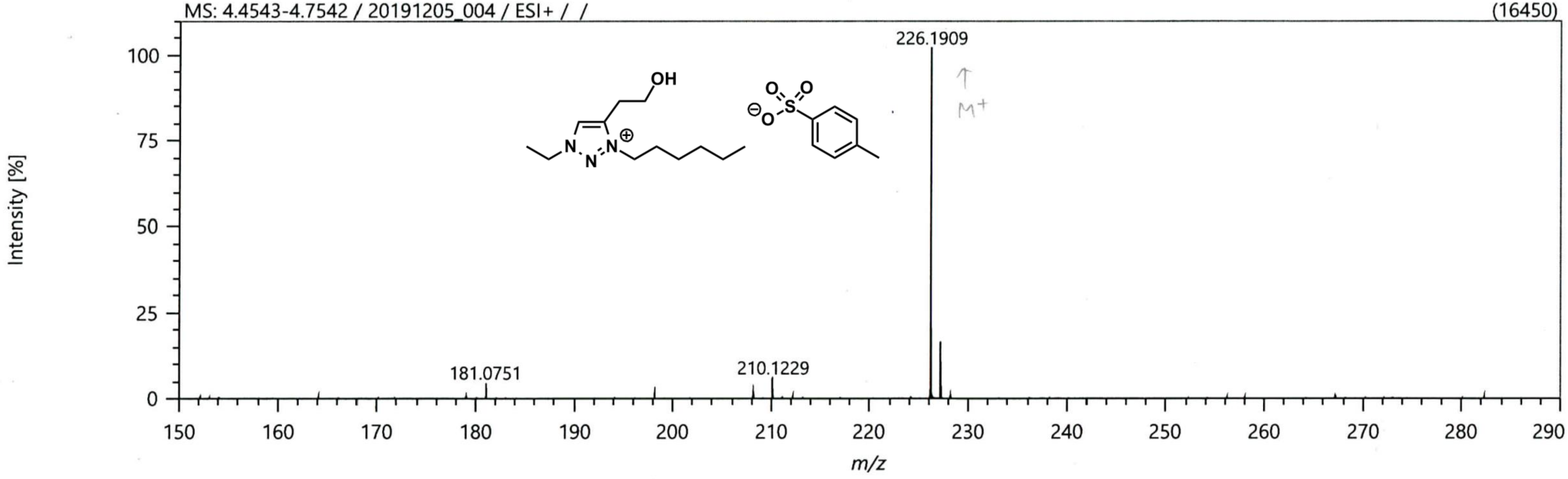

Elemental Composition

Parameters

Tolerance:	±10.00 ppm
Electron:	Odd/Even
Charge:	+1
DBE:	-1.5 - 999.0

Elements Set 1:

Symbol	C	H	O	Na	N	S	P	Si	F	Cl
Min	0	0	1	0	3	0	0	0	0	0
Max	400	1000	1	0	3	0	0	0	0	0

Results

Mass	Formula	Calculated Mass	Mass Difference [mDa]	Mass Difference [ppm]	DBE
226.19087	C12 H24 N3 O	226.19139	-0.52	-2.29	2.5

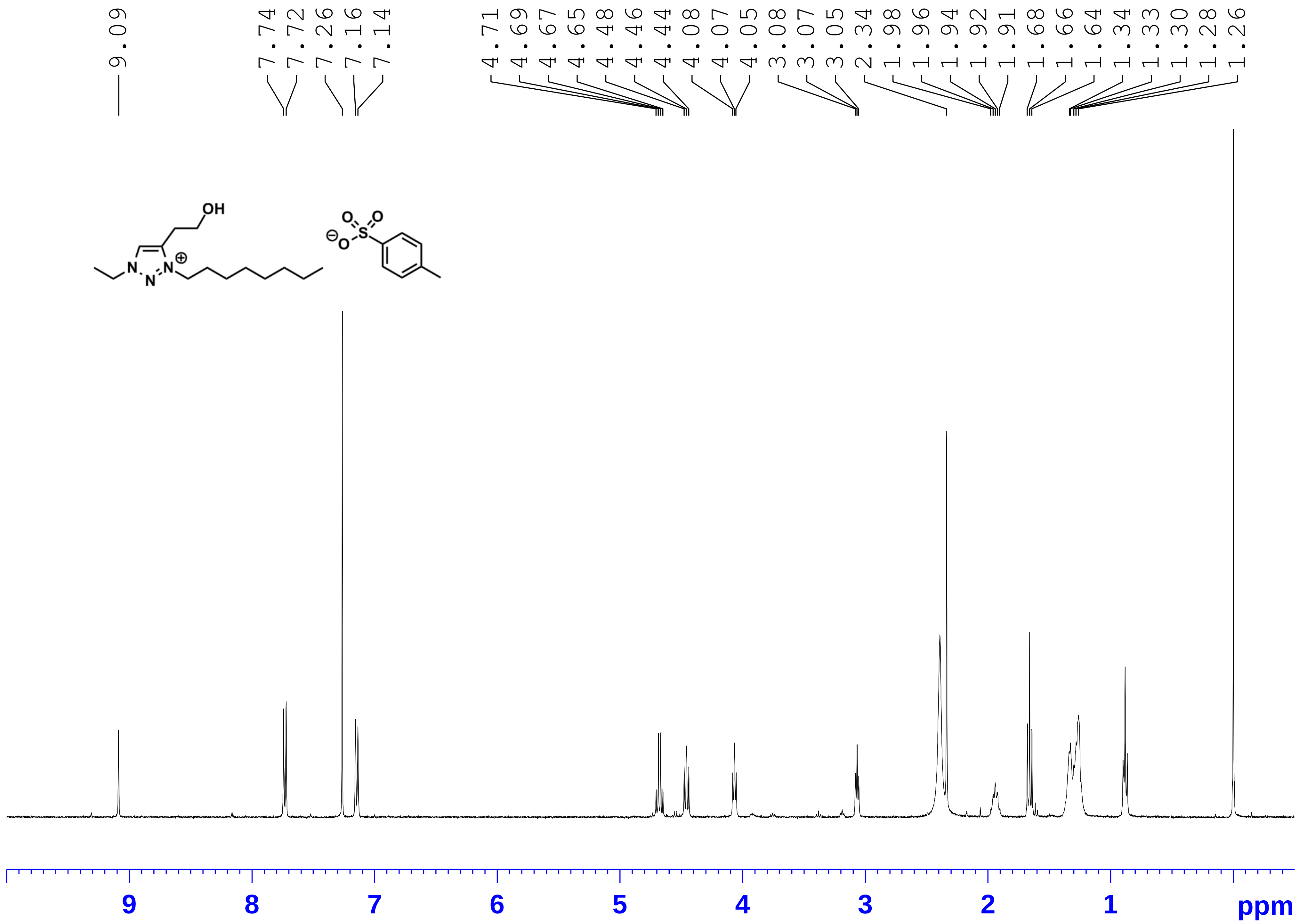

9.09
7.74
7.72
7.26
7.16
7.14
4.71
4.69
4.67
4.65
4.48
4.46
4.44
4.08
4.07
4.05
3.08
3.07
3.05
2.34
1.98
1.96
1.94
1.92
1.91
1.68
1.66
1.64
1.34
1.33
1.30
1.28
1.26
OH
N
N
N
O
O
S
O
9
8
7
6
5
4
3
2
1
ppm

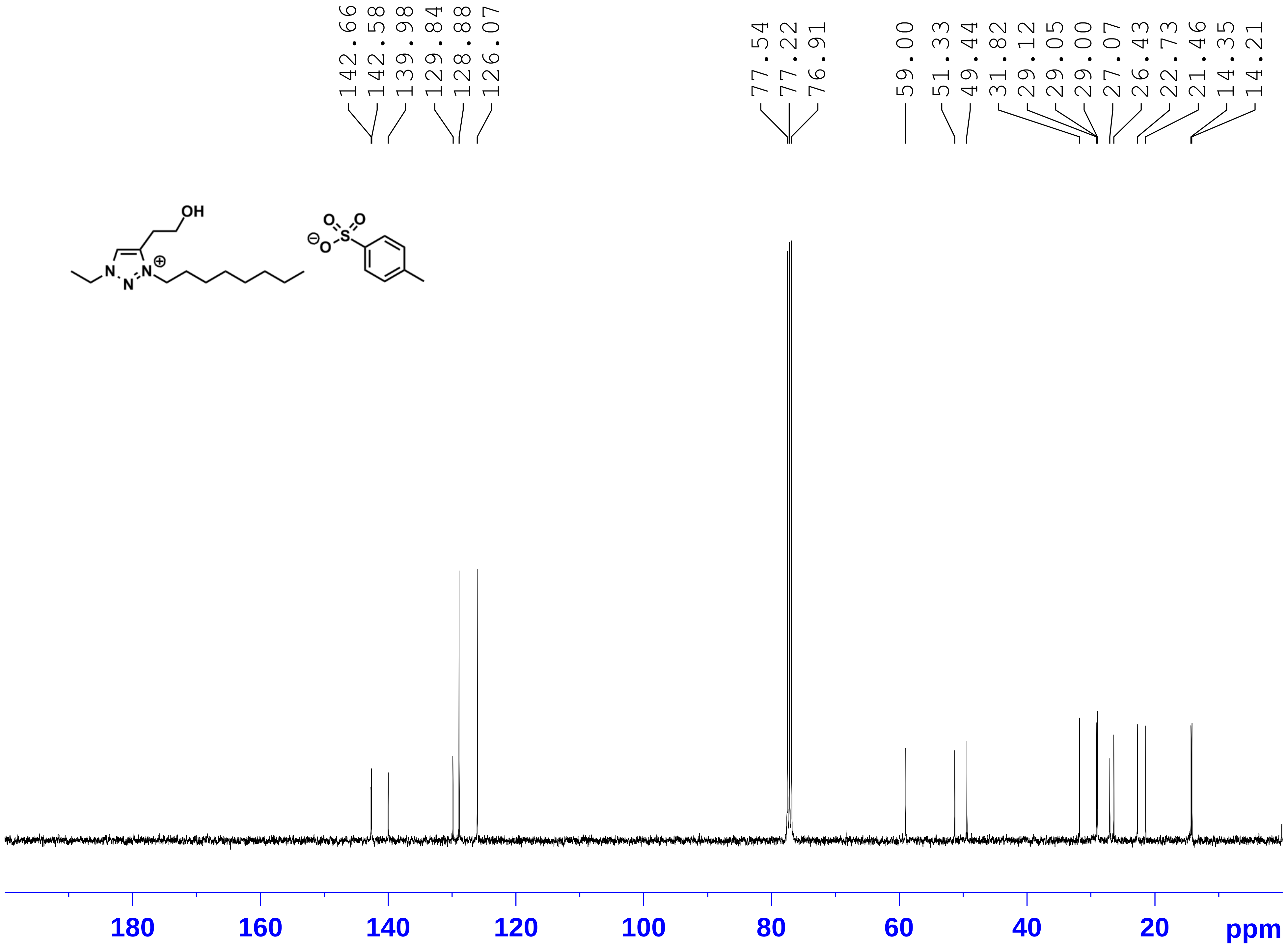

142.66
142.58
139.98
129.84
128.88
126.07
77.54
77.22
76.91
59.00
51.33
49.44
31.82
29.12
29.05
29.00
27.07
26.43
22.73
21.46
14.35
14.21
OH
N
N
N
O
S
O
O
180
160
140
120
100
80
60
40
20
ppm

Spectrum

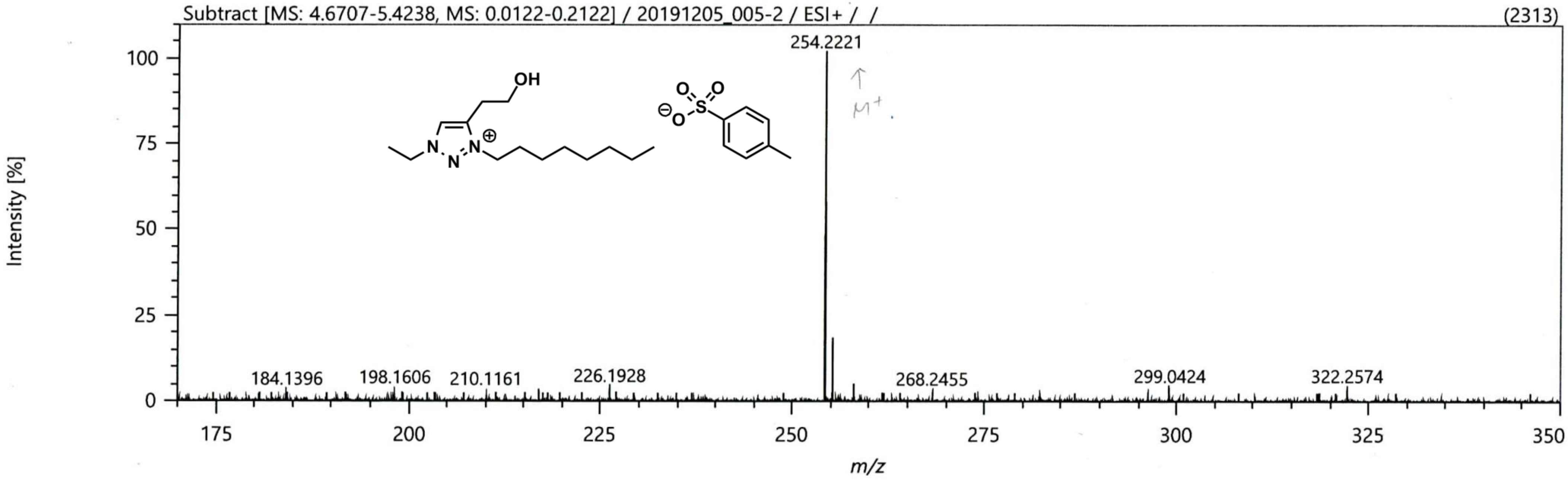

Elemental Composition

Parameters		Elements Set 1:										
Tolerance:	±50.00 ppm	Symbol	C	H	O	Na	N	S	P	Si	F	Cl
Electron:	Odd/Even	Min	0	0	1	0	3	0	0	0	0	0
Charge:	+1	Max	400	1000	1	0	3	0	0	0	0	0
DBE:	-1.5 - 999.0											

Results

Mass	Formula	Calculated Mass	Mass Difference [mDa]	Mass Difference [ppm]	DBE
254.22213	C14 H28 N3 O	254.22269	-0.56	-2.19	2.5

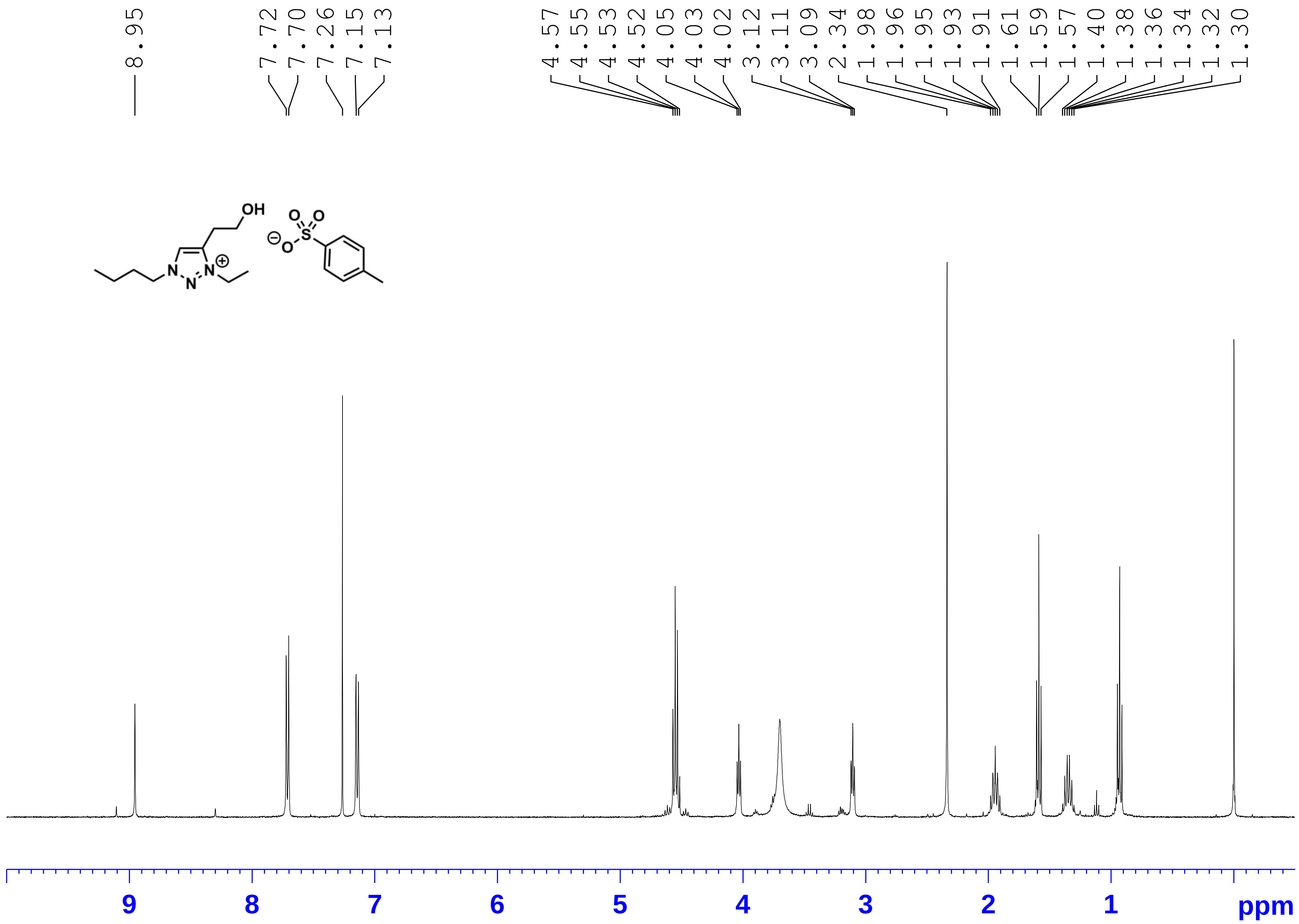
8.95
7.72
7.70
7.26
7.15
7.13
4.57
4.55
4.53
4.52
4.05
4.03
4.02
3.12
3.11
3.09
2.34
1.98
1.96
1.95
1.93
1.91
1.61
1.59
1.57
1.40
1.38
1.36
1.34
1.32
1.30
OH
O
S
O
O
N
N
N
9
8
7
6
5
4
3
2
1
ppm

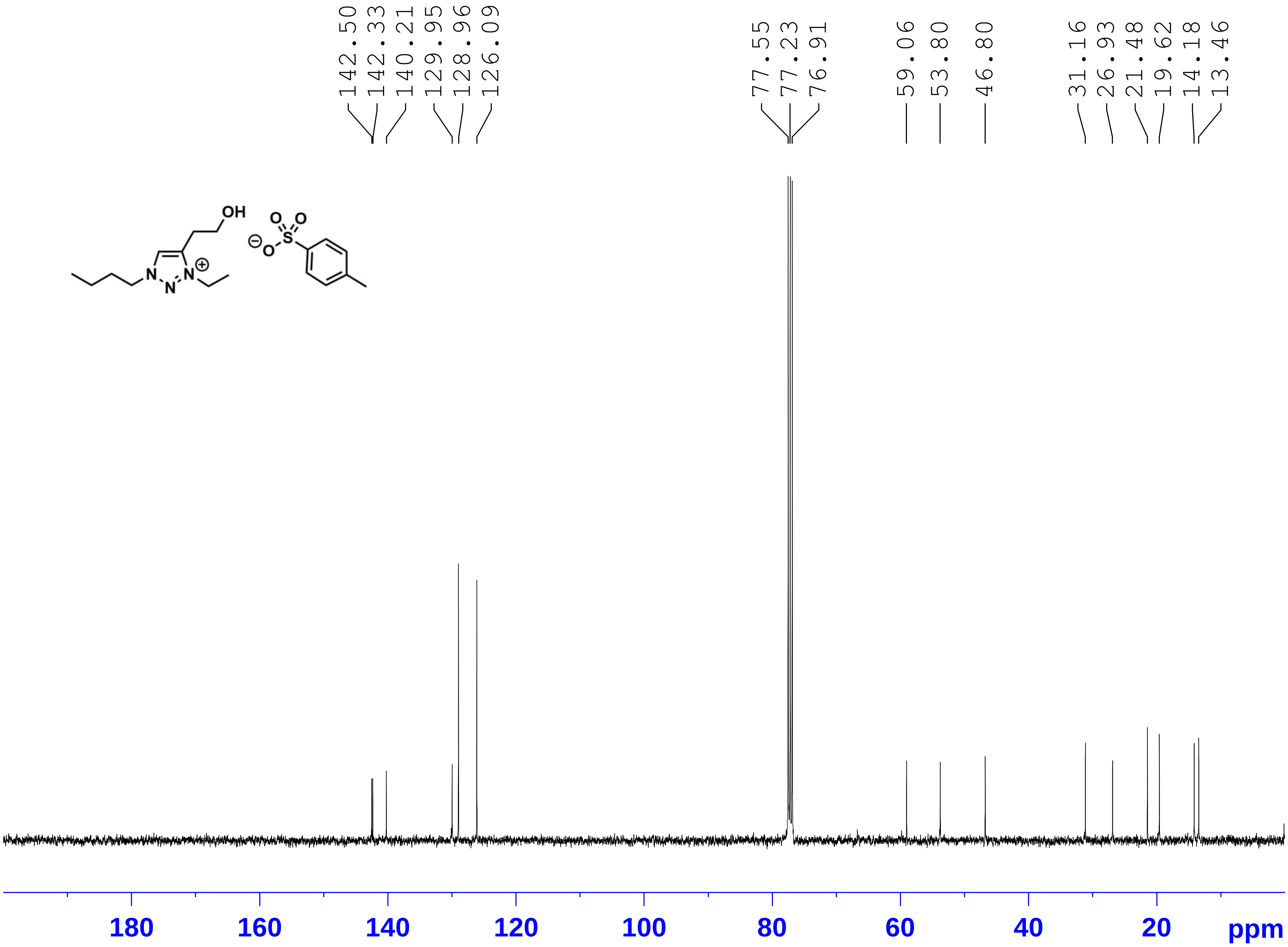

142.50
142.33
140.21
129.95
128.96
126.09
77.55
77.23
76.91
59.06
53.80
46.80
31.16
26.93
21.48
19.62
14.18
13.46
OH
O
O
S
O
N
N
N
180
160
140
120
100
80
60
40
20
ppm

Spectrum

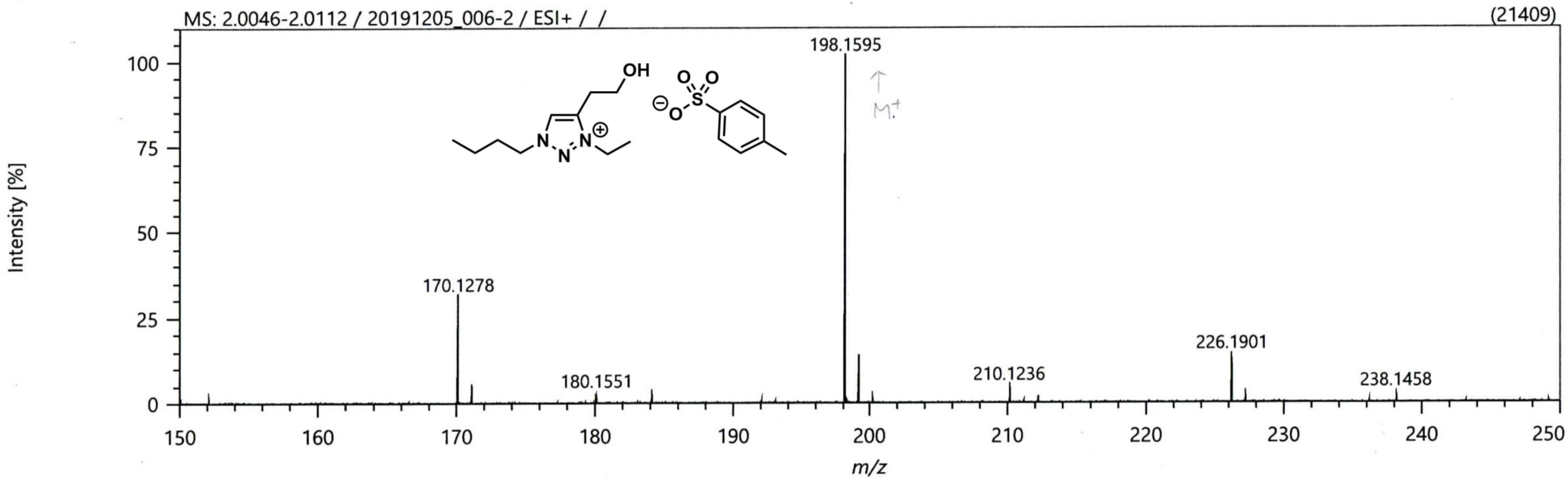

Elemental Composition

Parameters	
Tolerance:	±50.00 ppm
Electron:	Odd/Even
Charge:	+1
DBE:	-1.5 - 999.0

Elements Set 1:

Symbol	C	H	O	Na	N	S	P	Si	F	Cl
Min	0	0	1	0	3	0	0	0	0	0
Max	400	1000	1	0	3	0	0	0	0	0

Results

Mass	Formula	Calculated Mass	Mass Difference [mDa]	Mass Difference [ppm]	DBE
198.15951	C10 H20 N3 O	198.16009	-0.58	-2.92	2.5

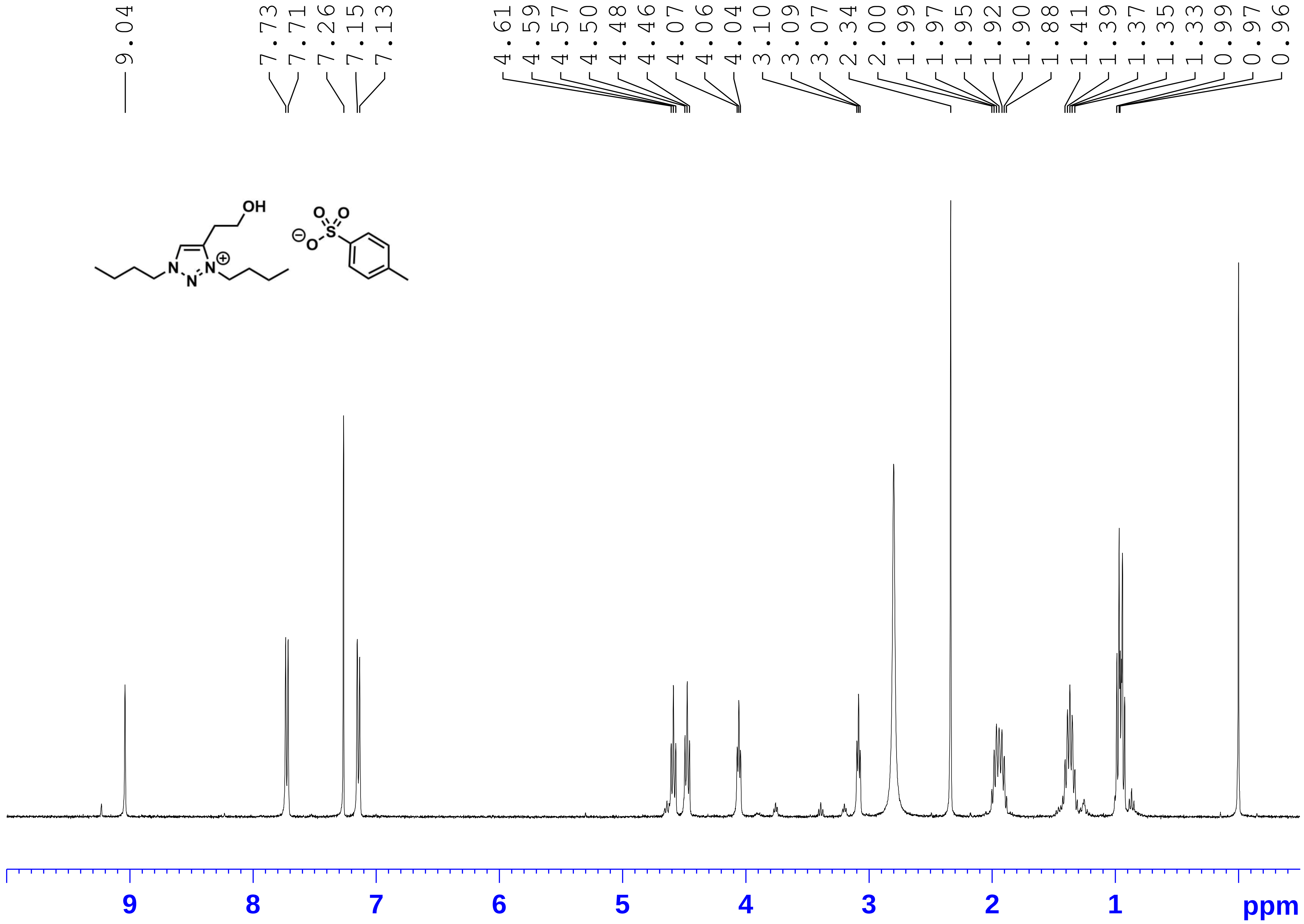
9.04
7.73
7.71
7.26
7.15
7.13
4.61
4.59
4.57
4.50
4.48
4.46
4.07
4.06
4.04
3.10
3.09
3.07
2.34
2.00
1.99
1.97
1.95
1.92
1.90
1.88
1.41
1.39
1.37
1.35
1.33
0.99
0.97
0.96
OH
O
O
S
O
N
N
N
9
8
7
6
5
4
3
2
1
ppm

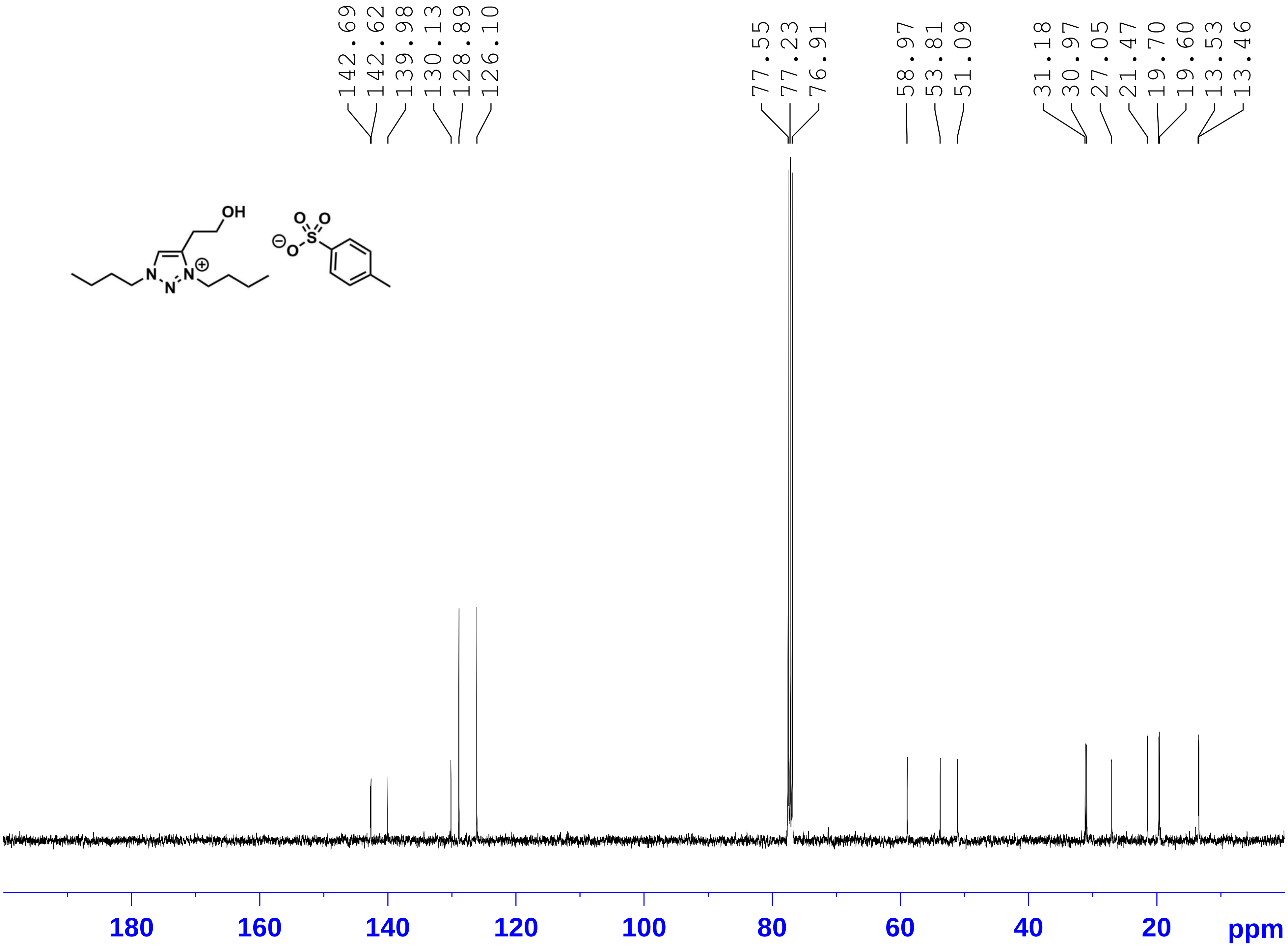

142.69
142.62
139.98
130.13
128.89
126.10
77.55
77.23
76.91
58.97
53.81
51.09
31.18
30.97
27.05
21.47
19.70
19.60
13.53
13.46
OH
O
S
N
180
160
140
120
100
80
60
40
20
ppm

Spectrum

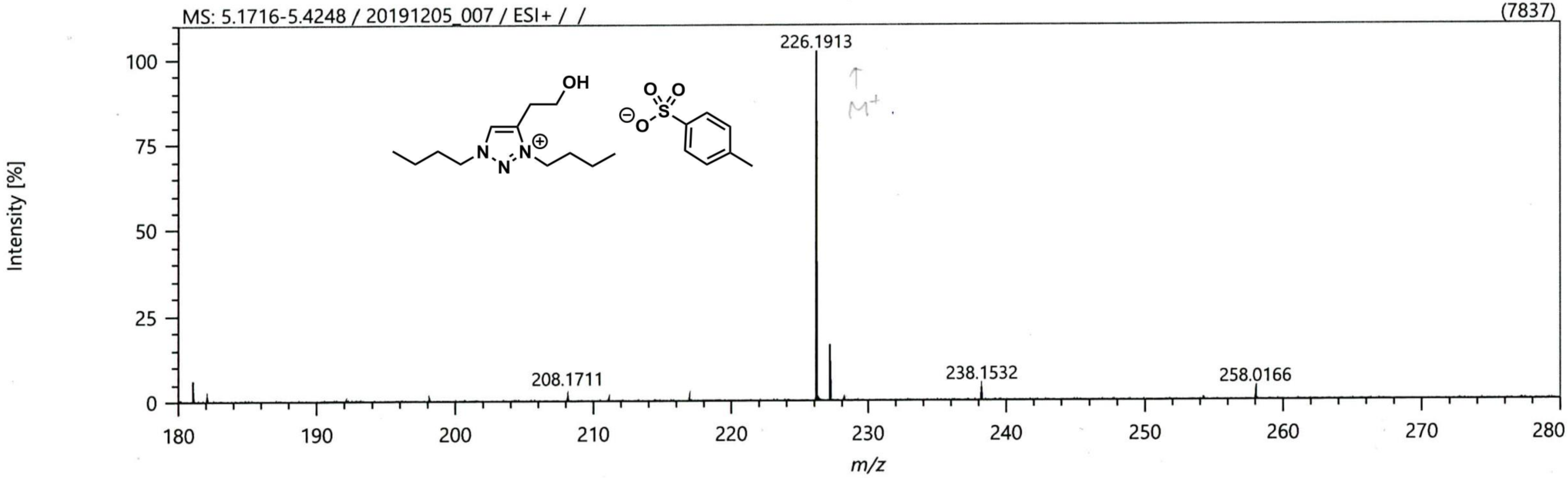

Elemental Composition

Parameters		Elements Set 1:										
Tolerance:	±50.00 ppm	Symbol	C	H	O	Na	N	S	P	Si	F	Cl
Electron:	Odd/Even	Min	0	0	1	0	3	0	0	0	0	0
Charge:	+1	Max	400	1000	1	0	3	0	0	0	0	0
DBE:	-1.5 - 999.0											

Results

Mass	Formula	Calculated Mass	Mass Difference [mDa]	Mass Difference [ppm]	DBE
226.19131	C12 H24 N3 O	226.19139	-0.07	-0.33	2.5

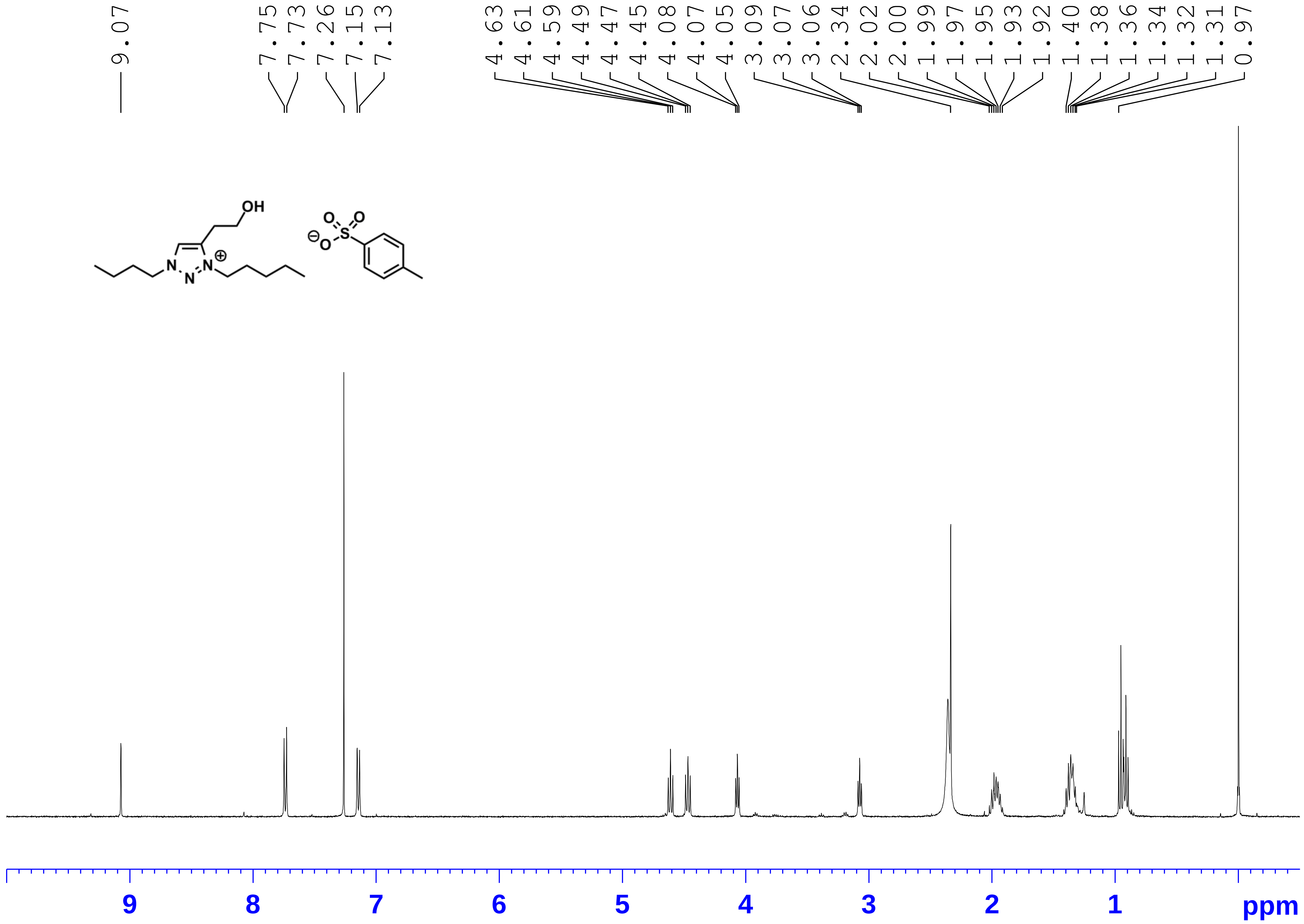

9.07
7.75
7.73
7.26
7.15
7.13
4.63
4.61
4.59
4.49
4.47
4.45
4.08
4.07
4.05
3.09
3.07
3.06
2.34
2.02
2.00
1.99
1.97
1.95
1.93
1.92
1.40
1.38
1.36
1.34
1.32
1.31
0.97
OH
O
S
O
O
N
N
N
9
8
7
6
5
4
3
2
1
ppm

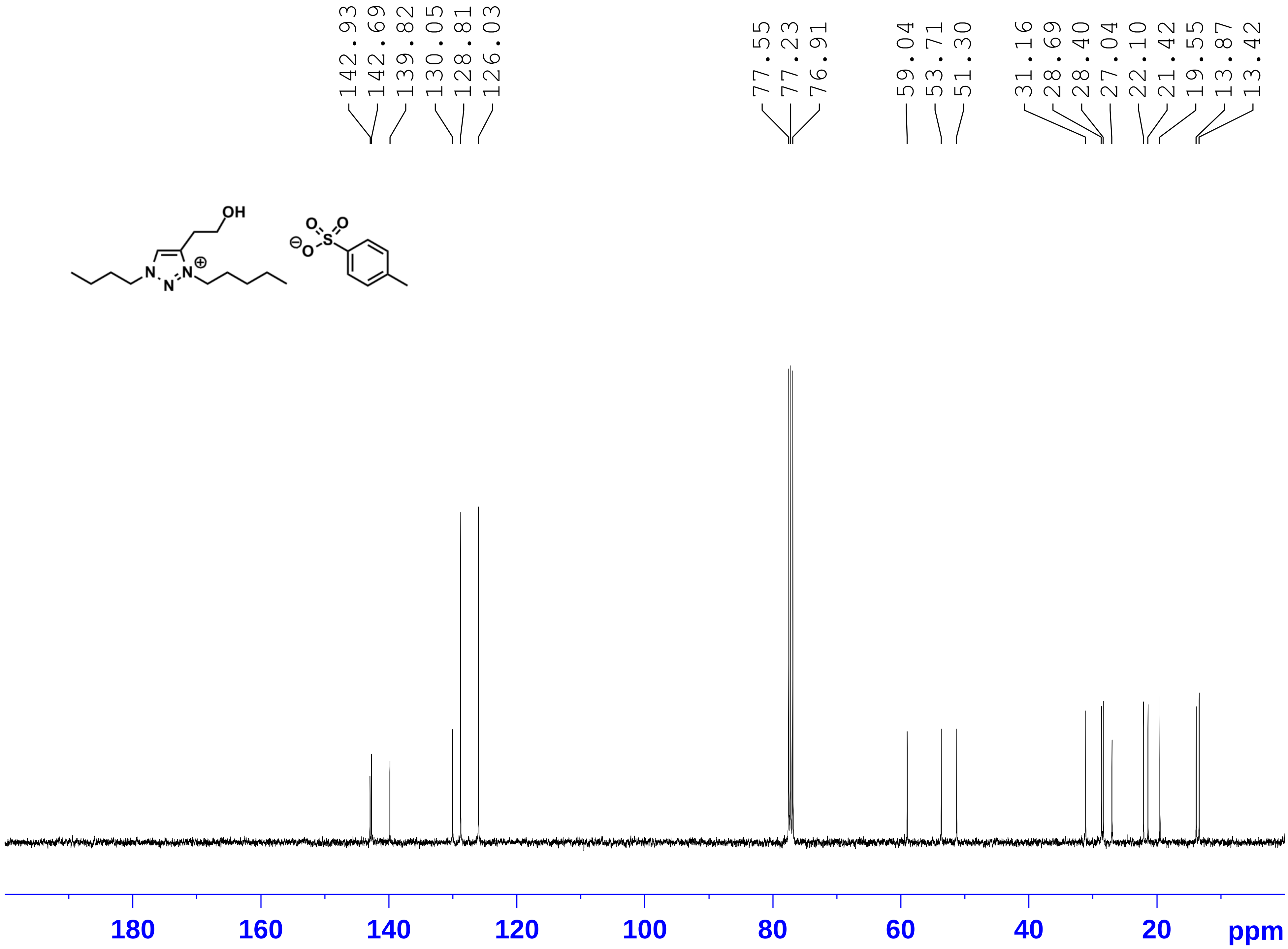
142.93
142.69
139.82
130.05
128.81
126.03
77.55
77.23
76.91
59.04
53.71
51.30
31.16
28.69
28.40
27.04
22.10
21.42
19.55
13.87
13.42
OH
N
N
N
O
S
O
O
180
160
140
120
100
80
60
40
20
ppm

Spectrum

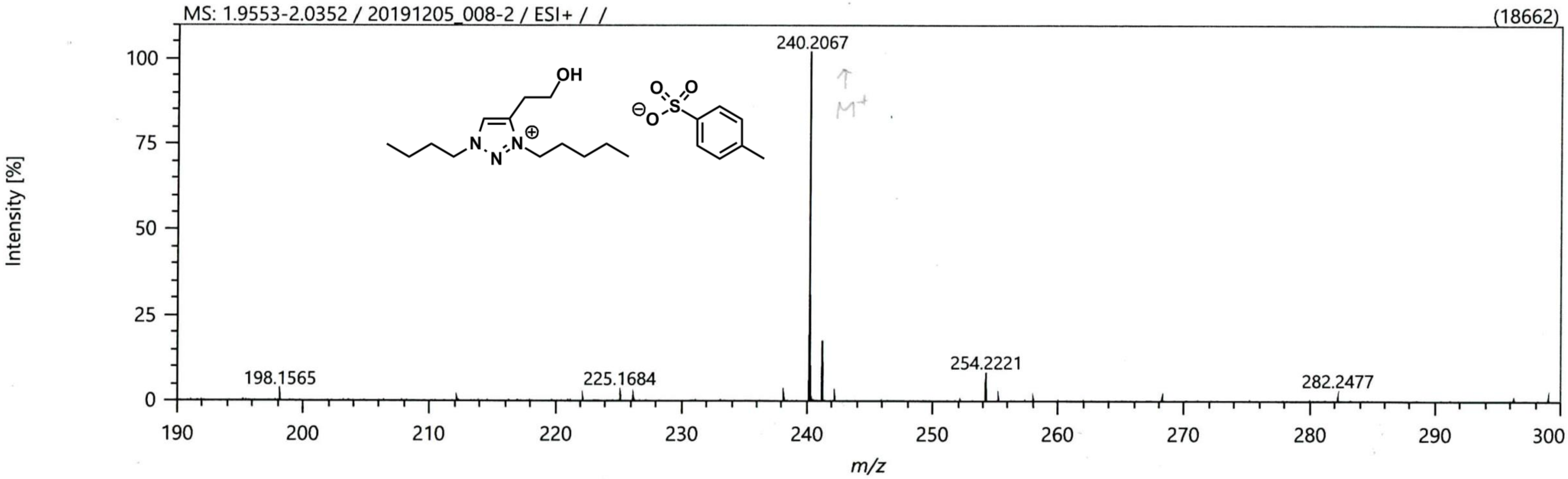

Elemental Composition

Parameters

Tolerance:	±50.00 ppm
Electron:	Odd/Even
Charge:	+1
DBE:	-1.5 - 999.0

Elements Set 1:

Symbol	C	H	O	Na	N	S	P	Si	F	Cl
Min	0	0	1	0	3	0	0	0	0	0
Max	400	1000	1	0	3	0	0	0	0	0

Results

Mass	Formula	Calculated Mass	Mass Difference [mDa]	Mass Difference [ppm]	DBE
240.20667	C13 H26 N3 O	240.20704	-0.36	-1.52	2.5

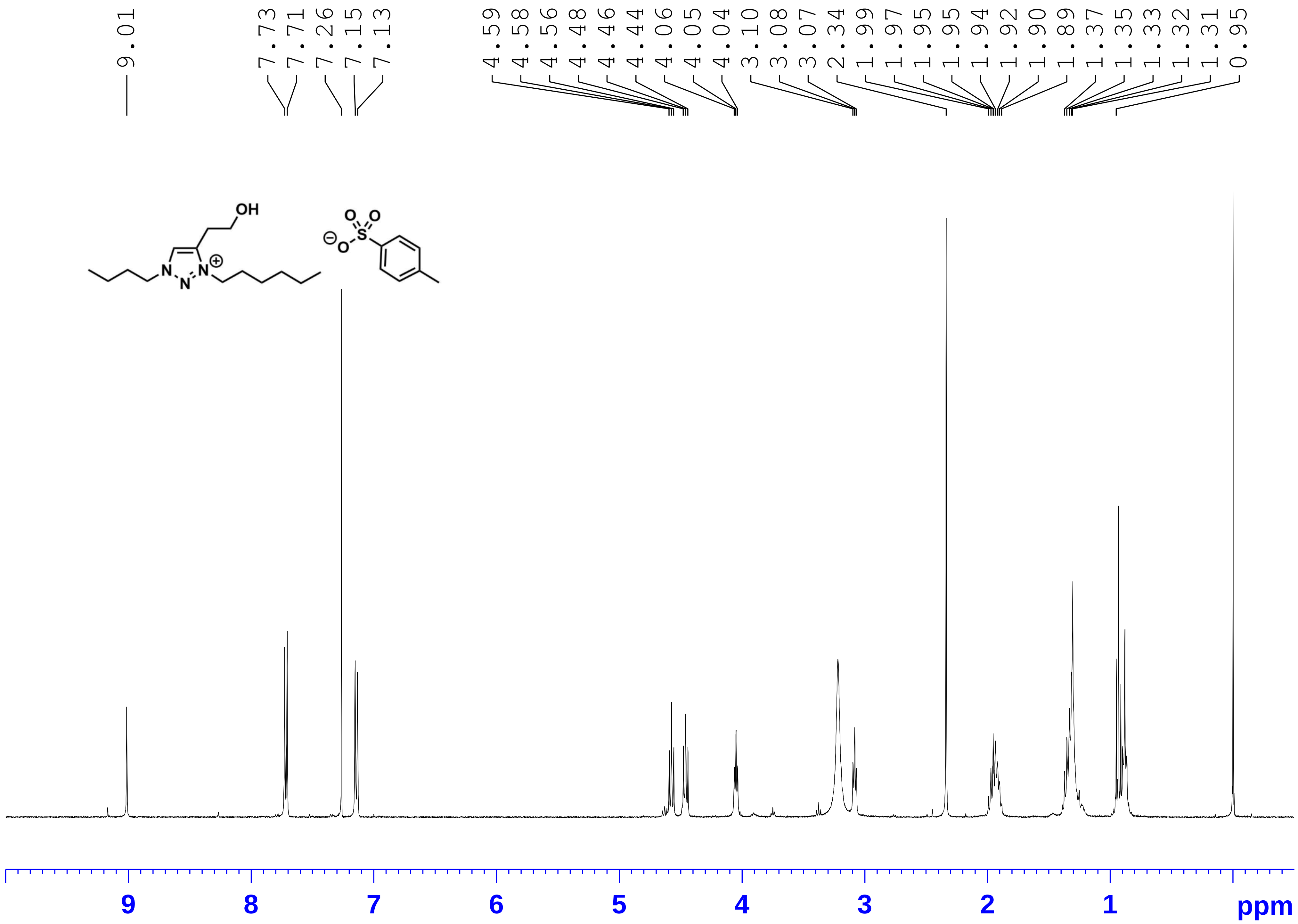

9.01
7.73
7.71
7.26
7.15
7.13
4.59
4.58
4.56
4.48
4.46
4.44
4.06
4.05
4.04
3.10
3.08
3.07
2.34
1.99
1.97
1.95
1.95
1.94
1.92
1.90
1.89
1.37
1.35
1.33
1.32
1.31
0.95
OH
O
O
S
O
N
N
N
9
8
7
6
5
4
3
2
1
ppm

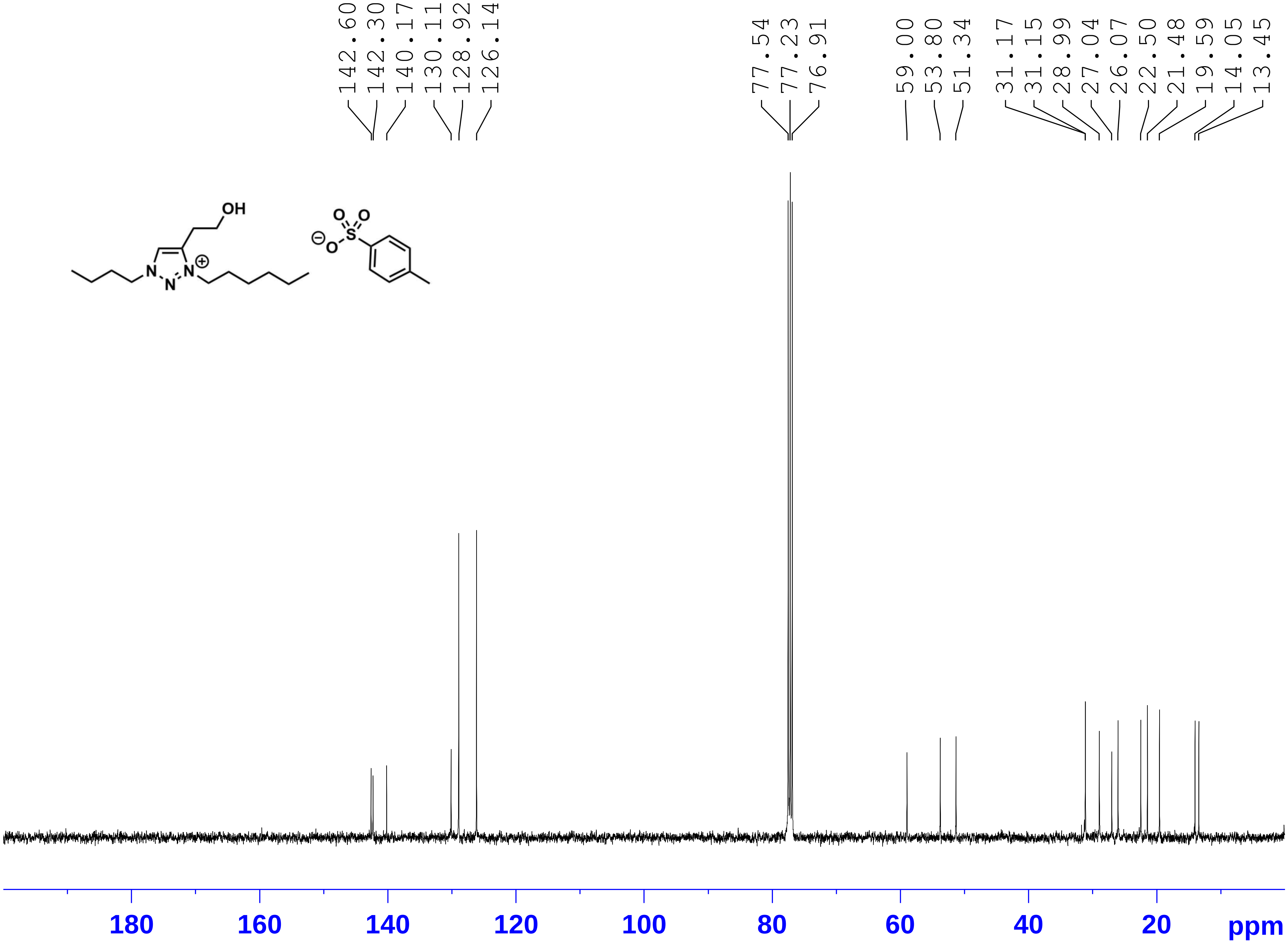

142.60
142.30
140.17
130.11
128.92
126.14
77.54
77.23
76.91
59.00
53.80
51.34
31.17
31.15
28.99
27.04
26.07
22.50
21.48
19.59
14.05
13.45
OH
O
O
S
O
N
N
N
180
160
140
120
100
80
60
40
20
ppm

Spectrum

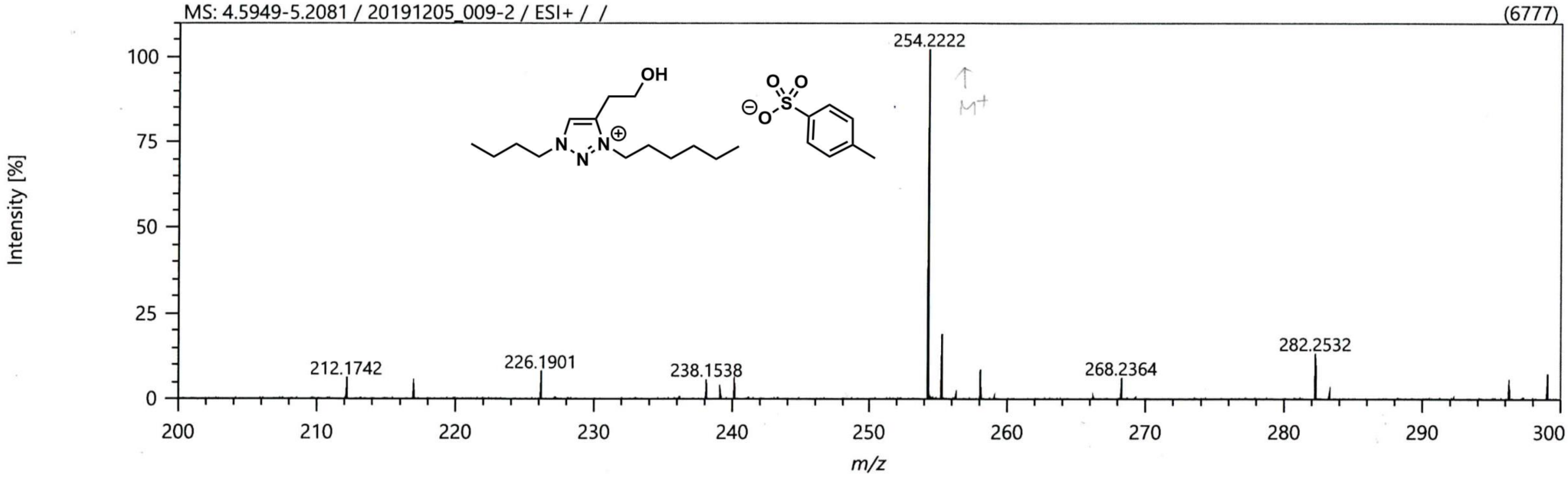

Elemental Composition

Parameters		Elements Set 1:									
Tolerance:	±50.00 ppm	Symbol	C	H	O	Na	N	S	P	Si	F	Cl
Electron:	Odd/Even	Min	0	0	1	0	3	0	0	0	0	0
Charge:	+1	Max	400	1000	1	0	3	0	0	0	0	0
DBE:	-1.5 - 999.0											

Results

Mass	Formula	Calculated Mass	Mass Difference [mDa]	Mass Difference [ppm]	DBE
254.22220	C14 H28 N3 O	254.22269	-0.49	-1.93	2.5

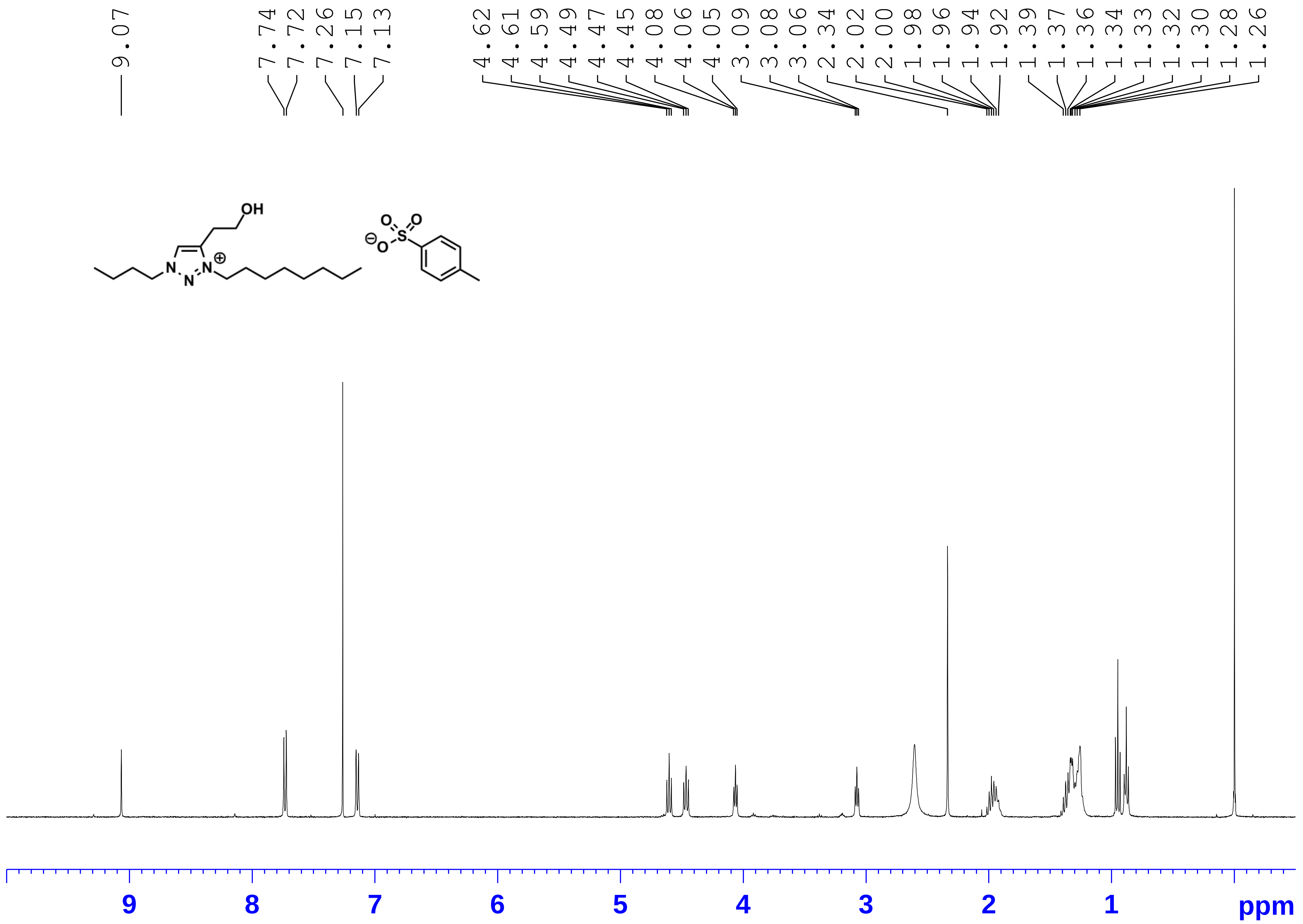
9.07
7.74
7.72
7.26
7.15
7.13
4.62
4.61
4.59
4.49
4.47
4.45
4.08
4.06
4.05
3.09
3.08
3.06
2.34
2.02
2.00
1.98
1.96
1.94
1.92
1.39
1.37
1.36
1.34
1.33
1.32
1.30
1.28
1.26
OH
N
N
N
O
O
S
O
9
8
7
6
5
4
3
2
1
ppm

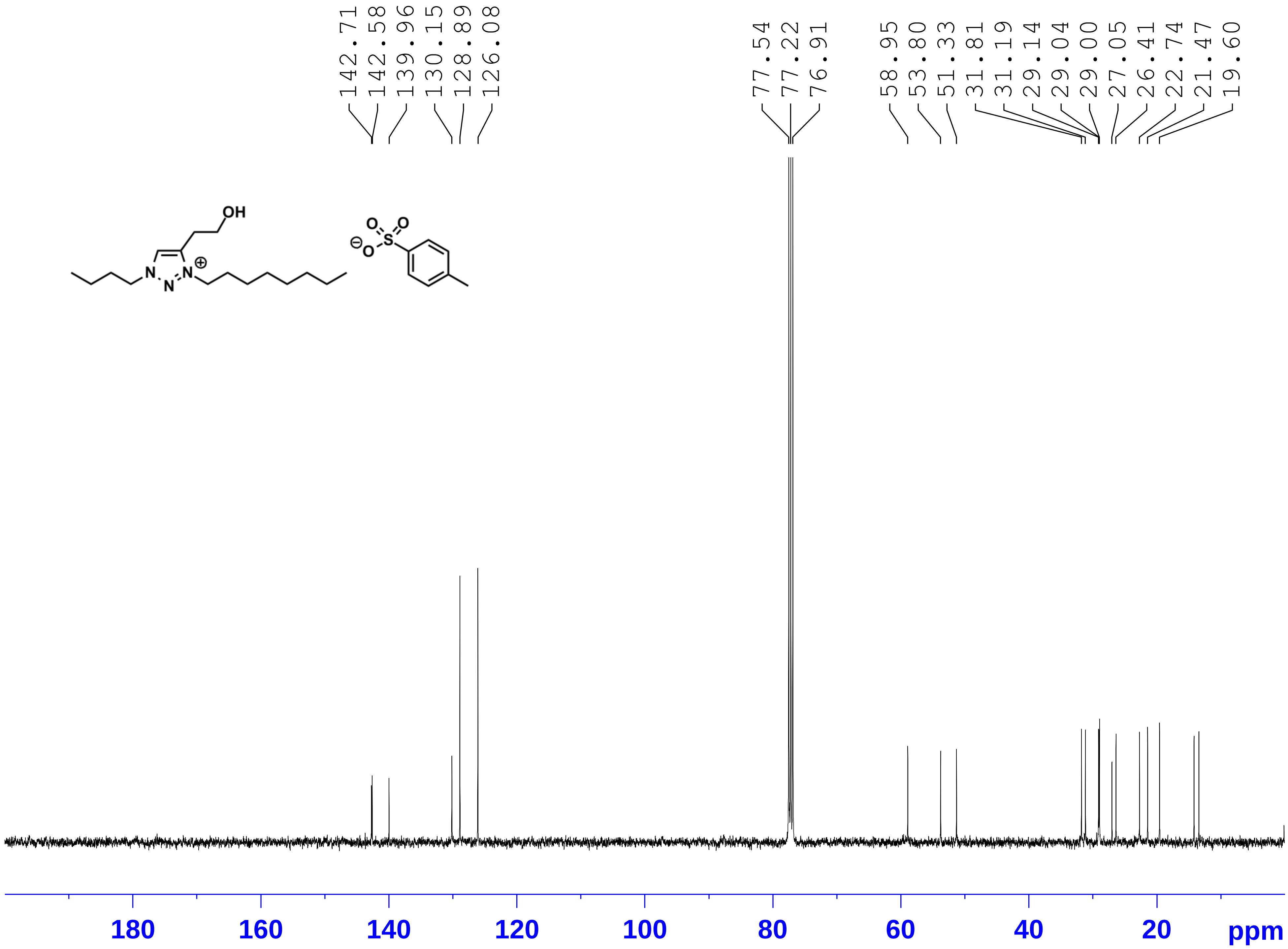
142.71
142.58
139.96
130.15
128.89
126.08
77.54
77.22
76.91
58.95
53.80
51.33
31.81
31.19
29.14
29.04
29.00
27.05
26.41
22.74
21.47
19.60
OH
O
S
O
O
N
N
N
180
160
140
120
100
80
60
40
20
ppm

Spectrum

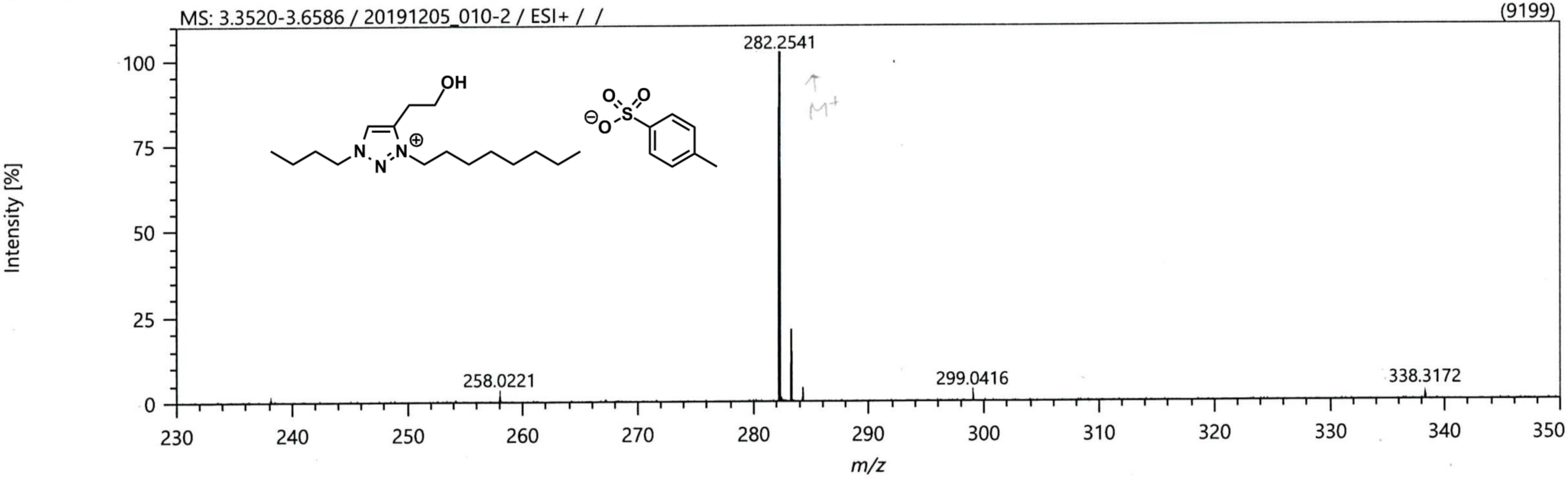

Elemental Composition

Parameters		Elements Set 1:										
Tolerance:	±50.00 ppm	Symbol	C	H	O	Na	N	S	P	Si	F	Cl
Electron:	Odd/Even	Min	0	0	1	0	3	0	0	0	0	0
Charge:	+1	Max	400	1000	1	0	3	0	0	0	0	0
DBE:	-1.5 - 999.0											

Results

Mass	Formula	Calculated Mass	Mass Difference [mDa]	Mass Difference [ppm]	DBE
282.25415	C16 H32 N3 O	282.25399	0.16	0.57	2.5

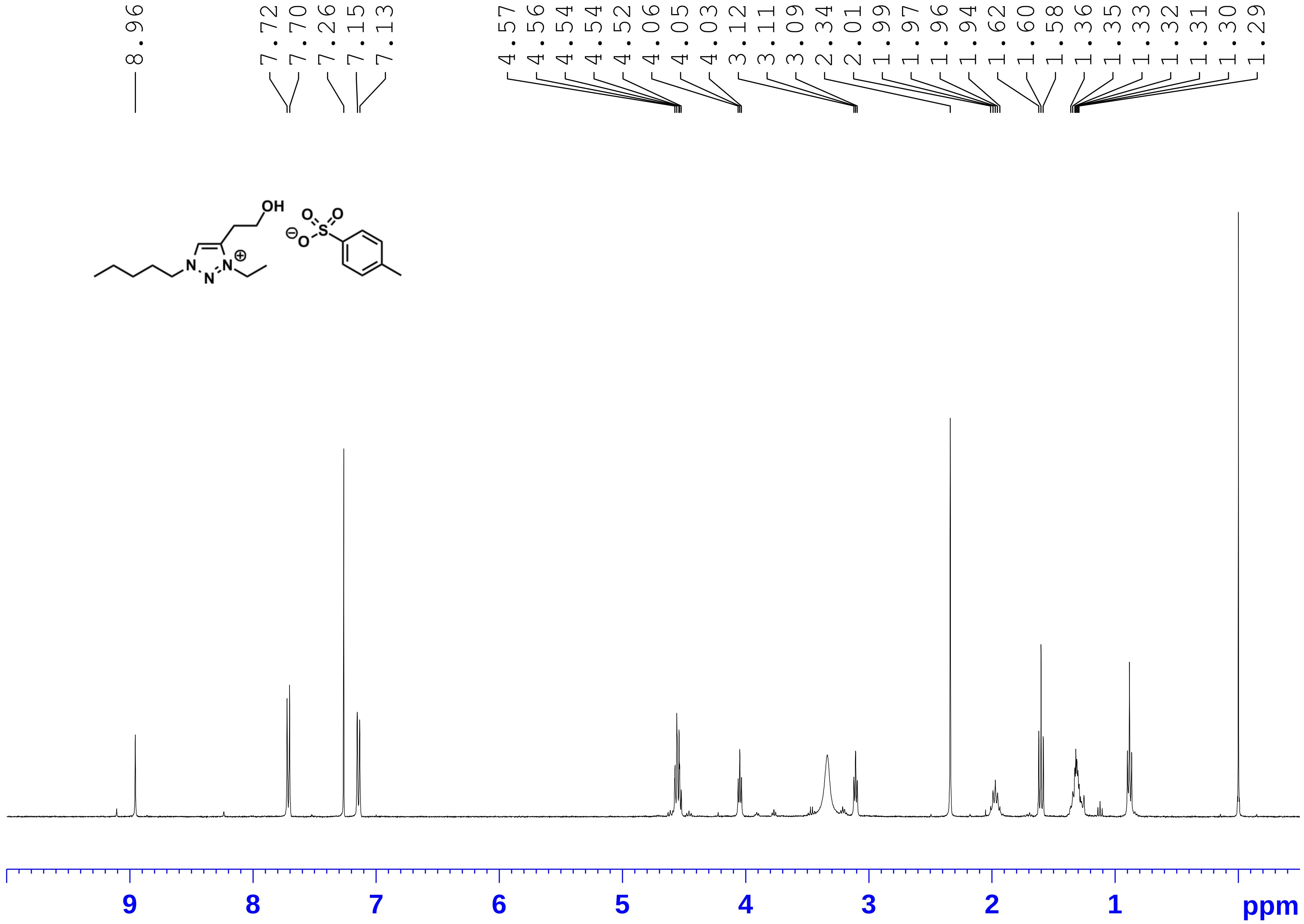
8.96
7.72
7.70
7.26
7.15
7.13
4.57
4.56
4.54
4.54
4.52
4.06
4.05
4.03
3.12
3.11
3.09
2.34
2.01
1.99
1.97
1.96
1.94
1.62
1.60
1.58
1.36
1.35
1.33
1.32
1.31
1.30
1.29
OH
O
S
O
O
N
N
N
9
8
7
6
5
4
3
2
1
ppm

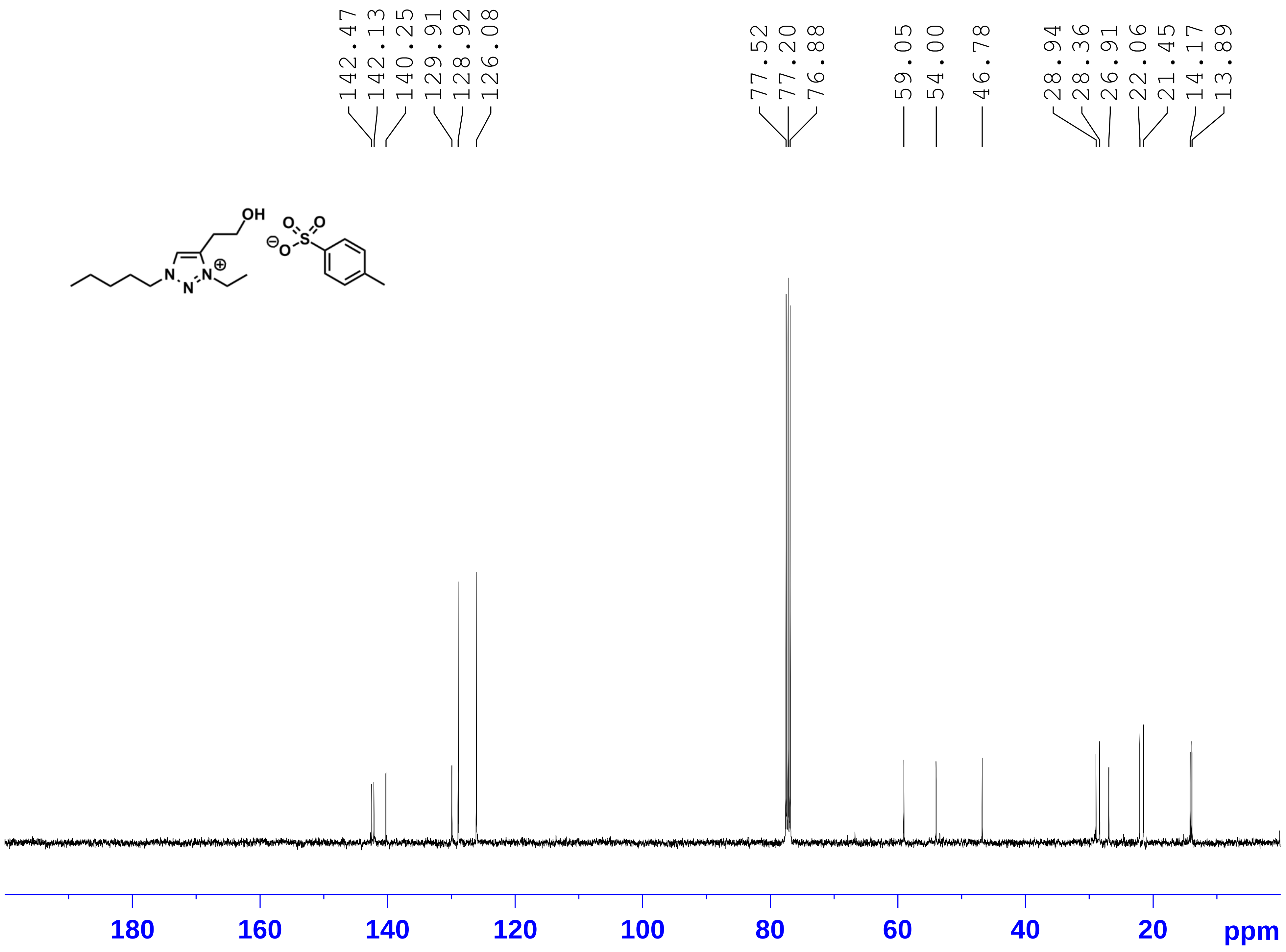

142.47
142.13
140.25
129.91
128.92
126.08
77.52
77.20
76.88
59.05
54.00
46.78
28.94
28.36
26.91
22.06
21.45
14.17
13.89
OH
O
O
S
O
N
N
N
180
160
140
120
100
80
60
40
20
ppm

Spectrum

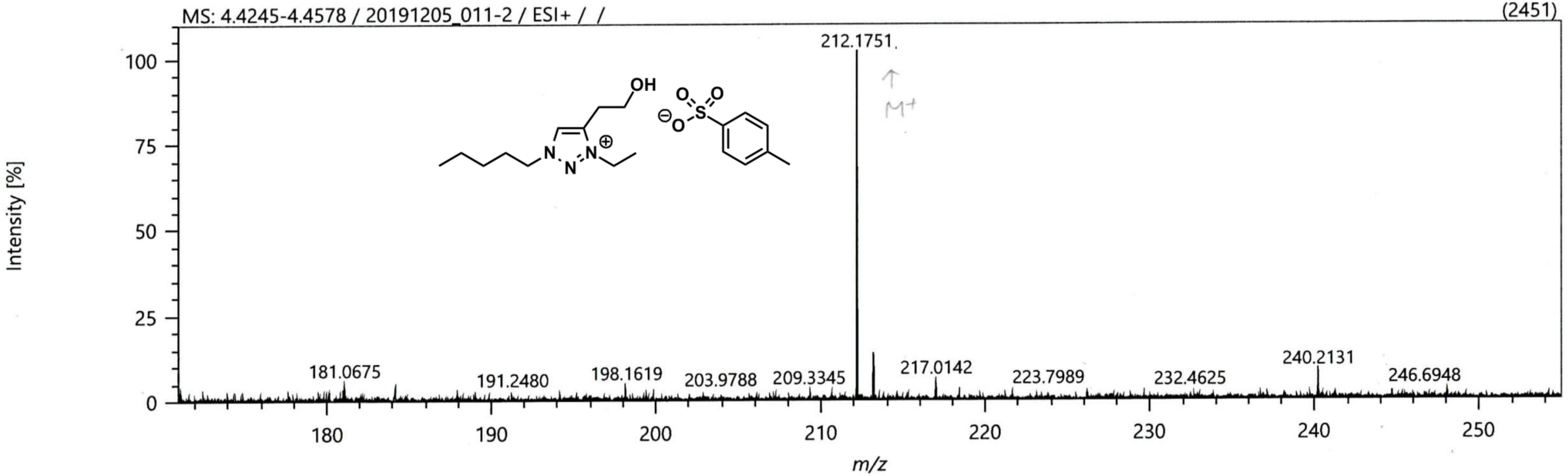

Elemental Composition

Parameters		Elements Set 1:										
Tolerance:	±50.00 ppm	Symbol	C	H	O	Na	N	S	P	Si	F	Cl
Electron:	Odd/Even	Min	0	0	1	0	3	0	0	0	0	0
Charge:	+1	Max	400	1000	1	0	3	0	0	0	0	0
DBE:	-1.5 - 999.0											

Results

Mass	Formula	Calculated Mass	Mass Difference [mDa]	Mass Difference [ppm]	DBE
212.17513	C11 H22 N3 O	212.17574	-0.61	-2.88	2.5

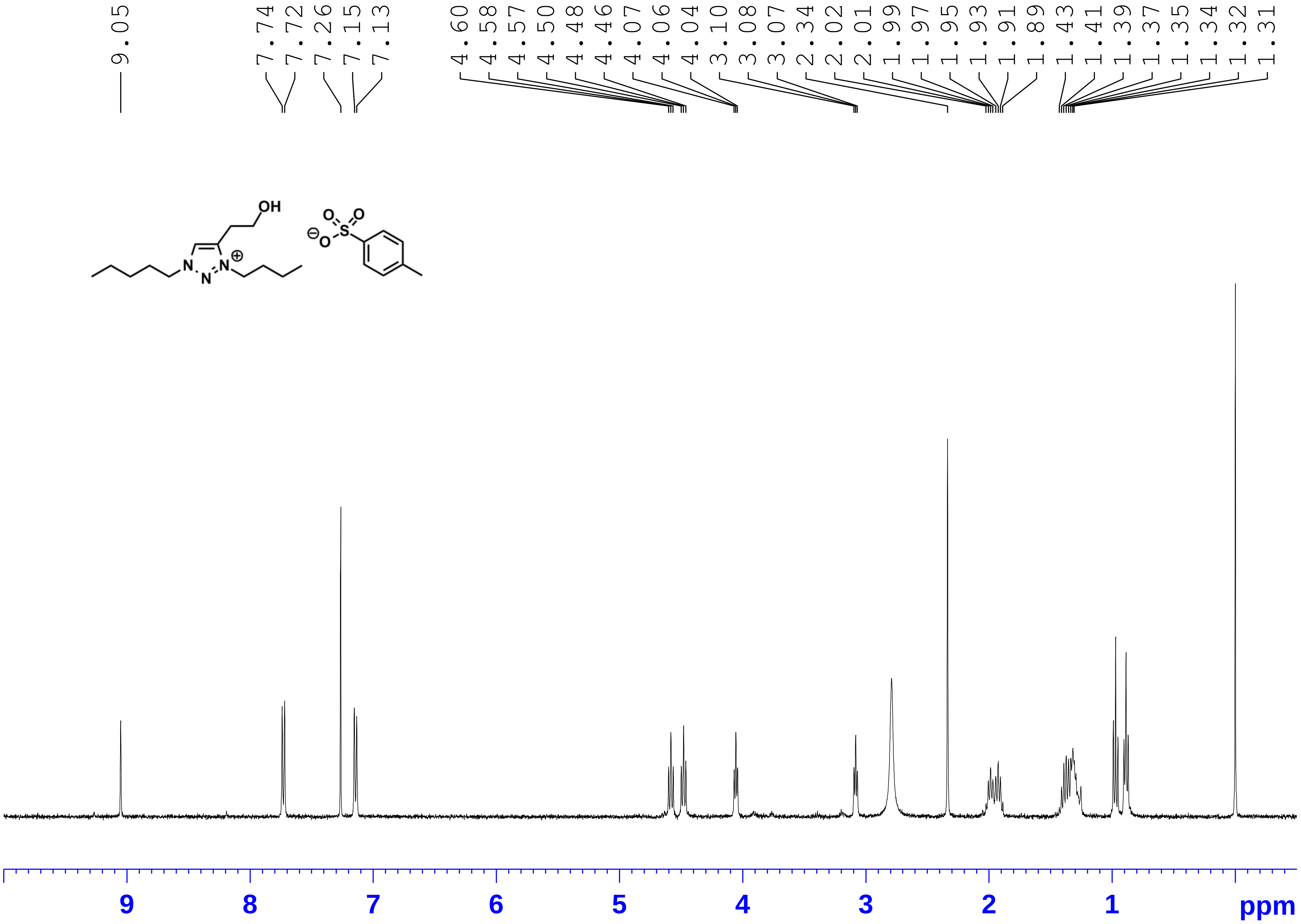
9.05
7.74
7.72
7.26
7.15
7.13
4.60
4.58
4.57
4.50
4.48
4.46
4.07
4.06
4.04
3.10
3.08
3.07
2.34
2.02
2.01
1.99
1.97
1.95
1.93
1.91
1.89
1.43
1.41
1.39
1.37
1.35
1.34
1.32
1.31
OH
O
O
S
O
N
N
N
9
8
7
6
5
4
3
2
1
ppm

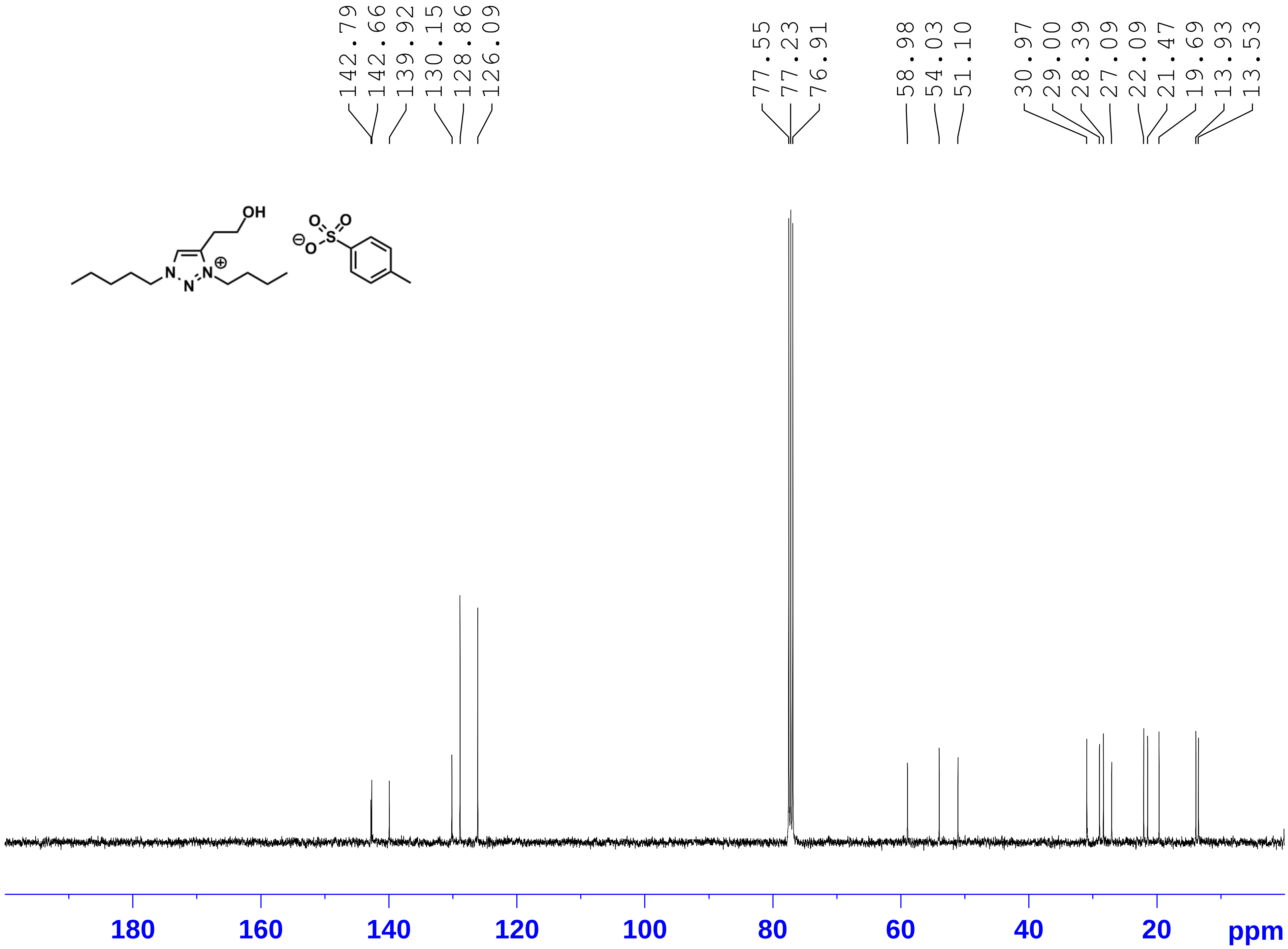
142.79
142.66
139.92
130.15
128.86
126.09
77.55
77.23
76.91
58.98
54.03
51.10
30.97
29.00
28.39
27.09
22.09
21.47
19.69
13.93
13.53
OH
N
N
N
O
S
O
O
180
160
140
120
100
80
60
40
20
ppm

Spectrum

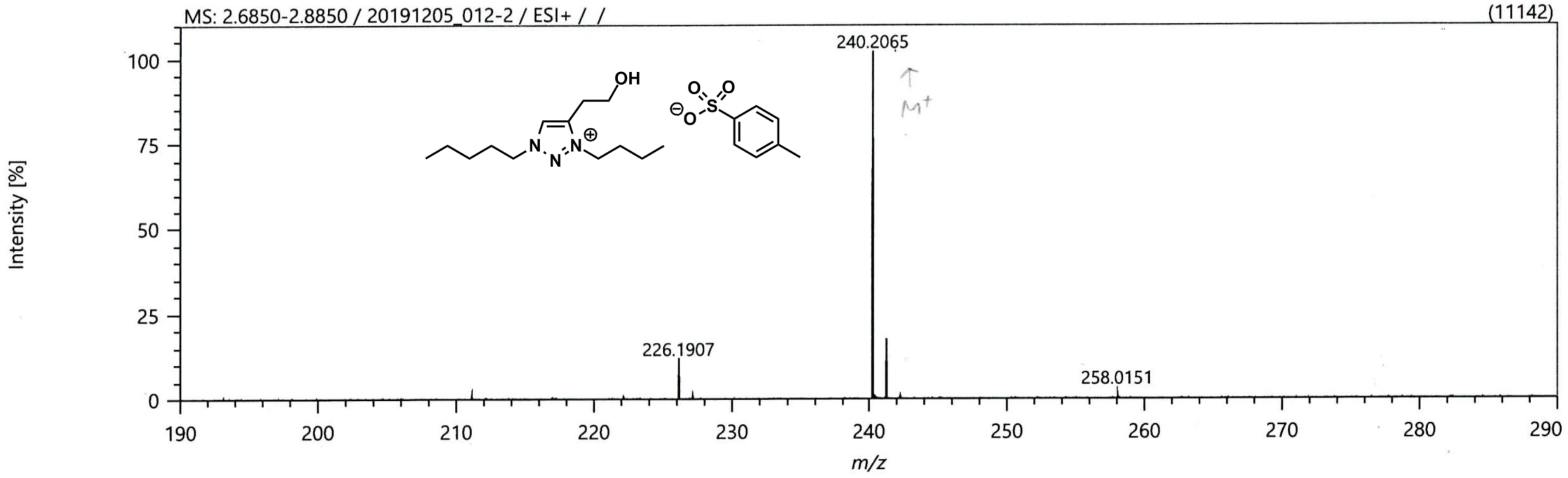

Elemental Composition

Parameters	
Tolerance:	±50.00 ppm
Electron:	Odd/Even
Charge:	+1
DBE:	-1.5 - 999.0

Elements Set 1:

Symbol	C	H	O	Na	N	S	P	Si	F	Cl
Min	0	0	1	0	3	0	0	0	0	0
Max	400	1000	1	0	3	0	0	0	0	0

Results

Mass	Formula	Calculated Mass	Mass Difference [mDa]	Mass Difference [ppm]	DBE
240.20652	C13 H26 N3 O	240.20704	-0.52	-2.17	2.5

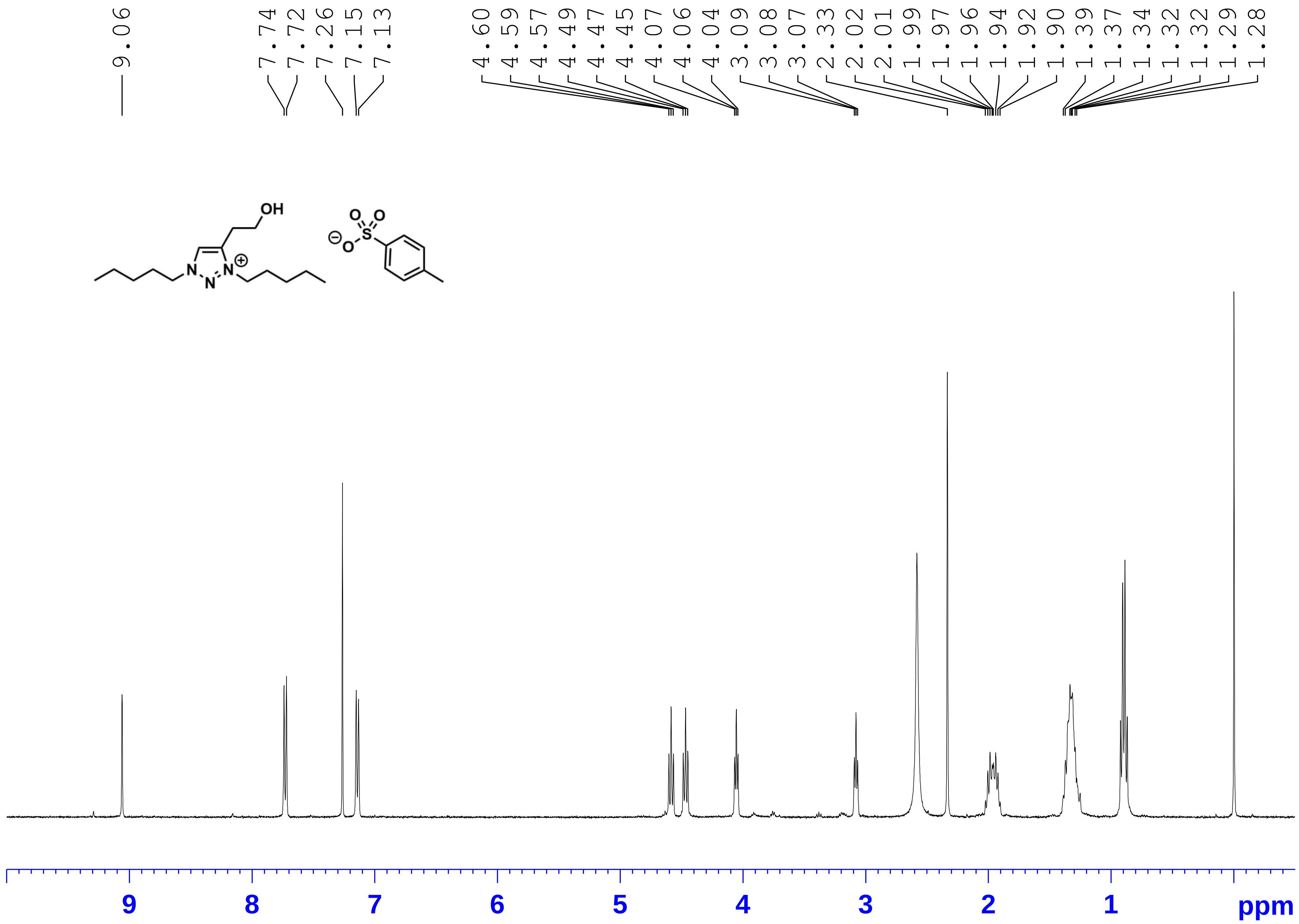
9.06
7.74
7.72
7.26
7.15
7.13
4.60
4.59
4.57
4.49
4.47
4.45
4.07
4.06
4.04
3.09
3.08
3.07
2.33
2.02
2.01
1.99
1.97
1.96
1.94
1.92
1.90
1.39
1.37
1.34
1.32
1.32
1.29
1.28
OH
N
N
N
O
O
S
O
9
8
7
6
5
4
3
2
1
ppm

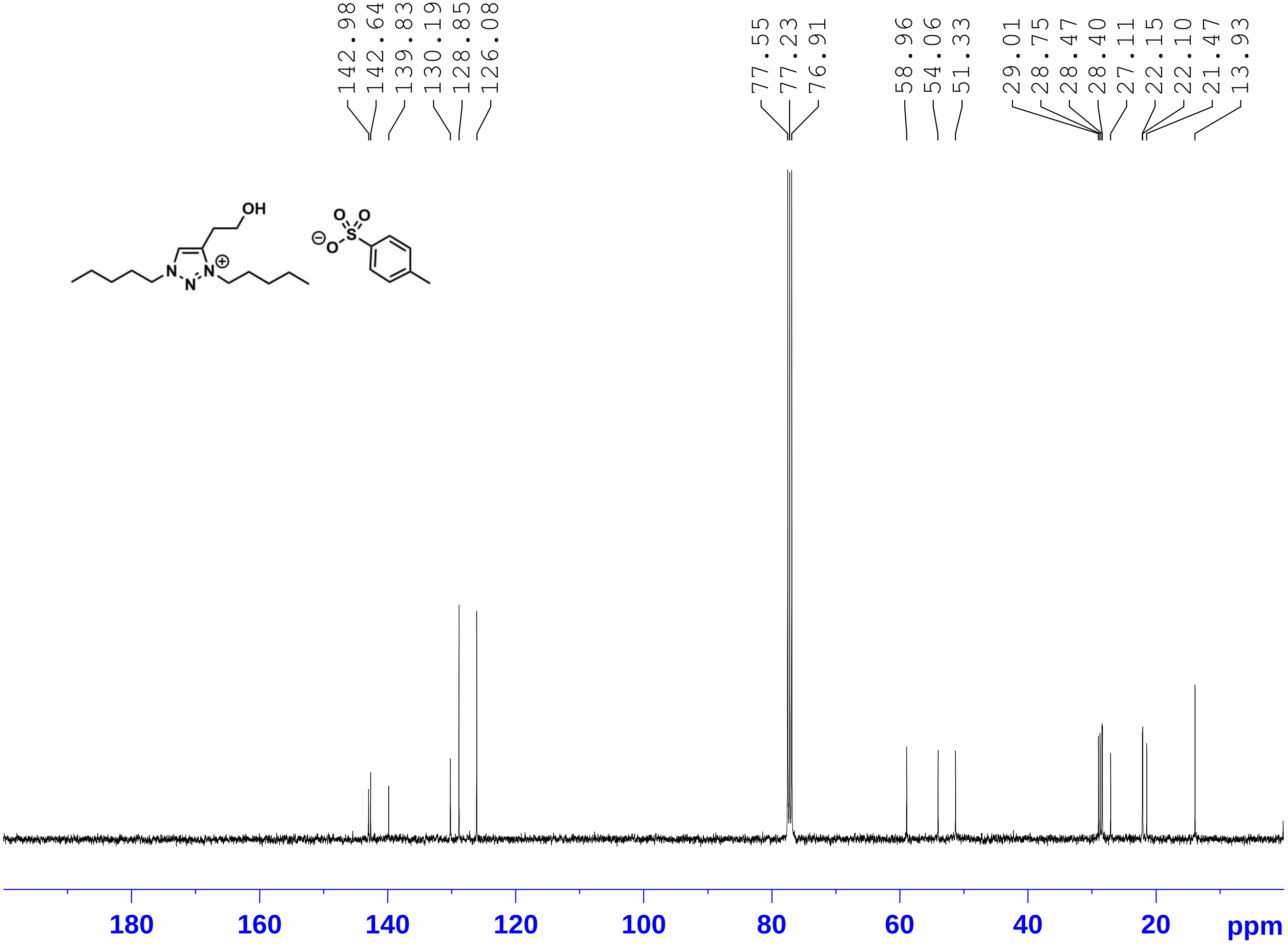

142.98
142.64
139.83
130.19
128.85
126.08
77.55
77.23
76.91
58.96
54.06
51.33
29.01
28.75
28.47
28.40
27.11
22.15
22.10
21.47
13.93
OH
N
N
N
S
O
O
O
180
160
140
120
100
80
60
40
20
ppm

Spectrum

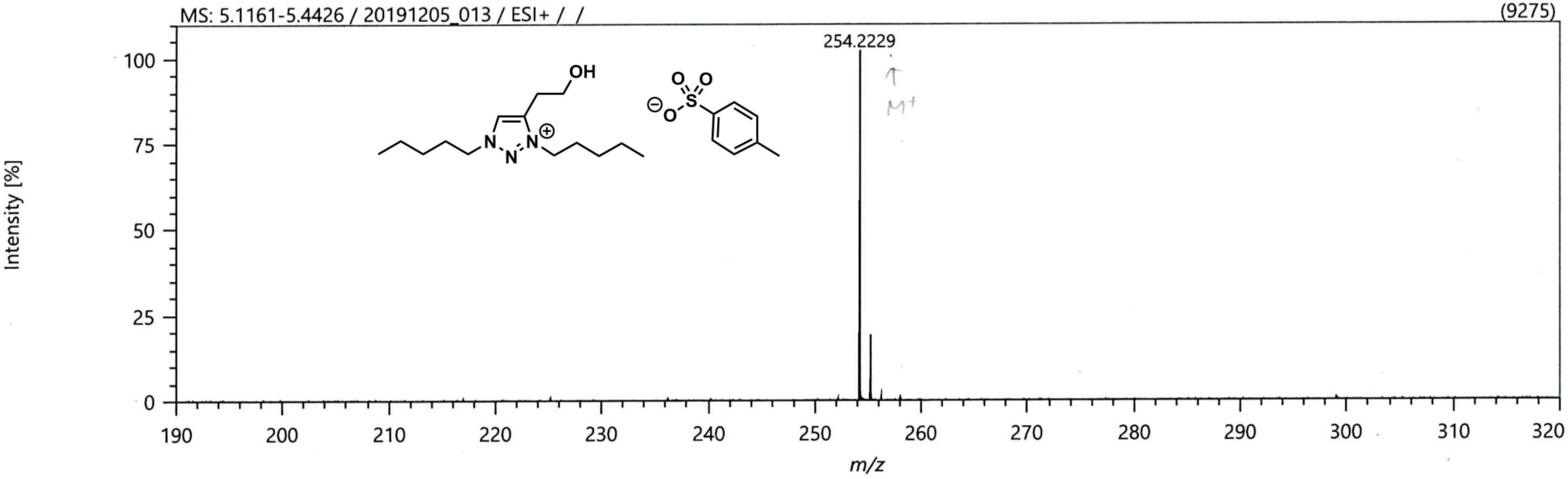

Elemental Composition

Parameters

Tolerance:	±50.00 ppm
Electron:	Odd/Even
Charge:	+1
DBE:	-1.5 - 999.0

Elements Set 1:

Symbol	C	H	O	Na	N	S	P	Si	F	Cl
Min	0	0	1	0	3	0	0	0	0	0
Max	400	1000	1	0	3	0	0	0	0	0

Results

Mass	Formula	Calculated Mass	Mass Difference [mDa]	Mass Difference [ppm]	DBE
254.22286	C14 H28 N3 O	254.22269	0.17	0.68	2.5

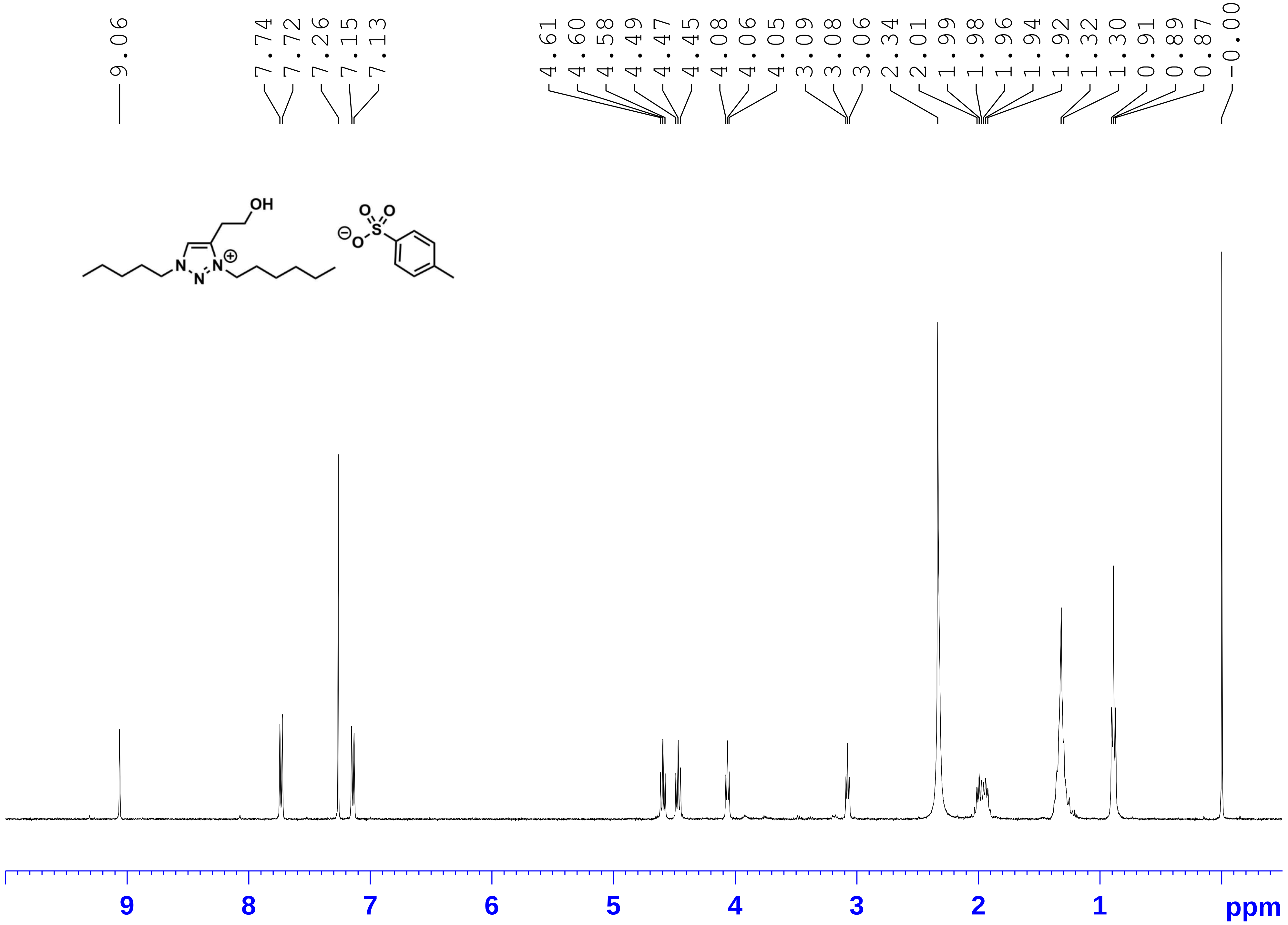
9.06
7.74
7.72
7.26
7.15
7.13
4.61
4.60
4.58
4.49
4.47
4.45
4.08
4.06
4.05
3.09
3.08
3.06
2.34
2.01
1.99
1.98
1.96
1.94
1.92
1.32
1.30
0.91
0.89
0.87
-0.00
OH
N
N
N
O
O
S
O
9
8
7
6
5
4
3
2
1
ppm

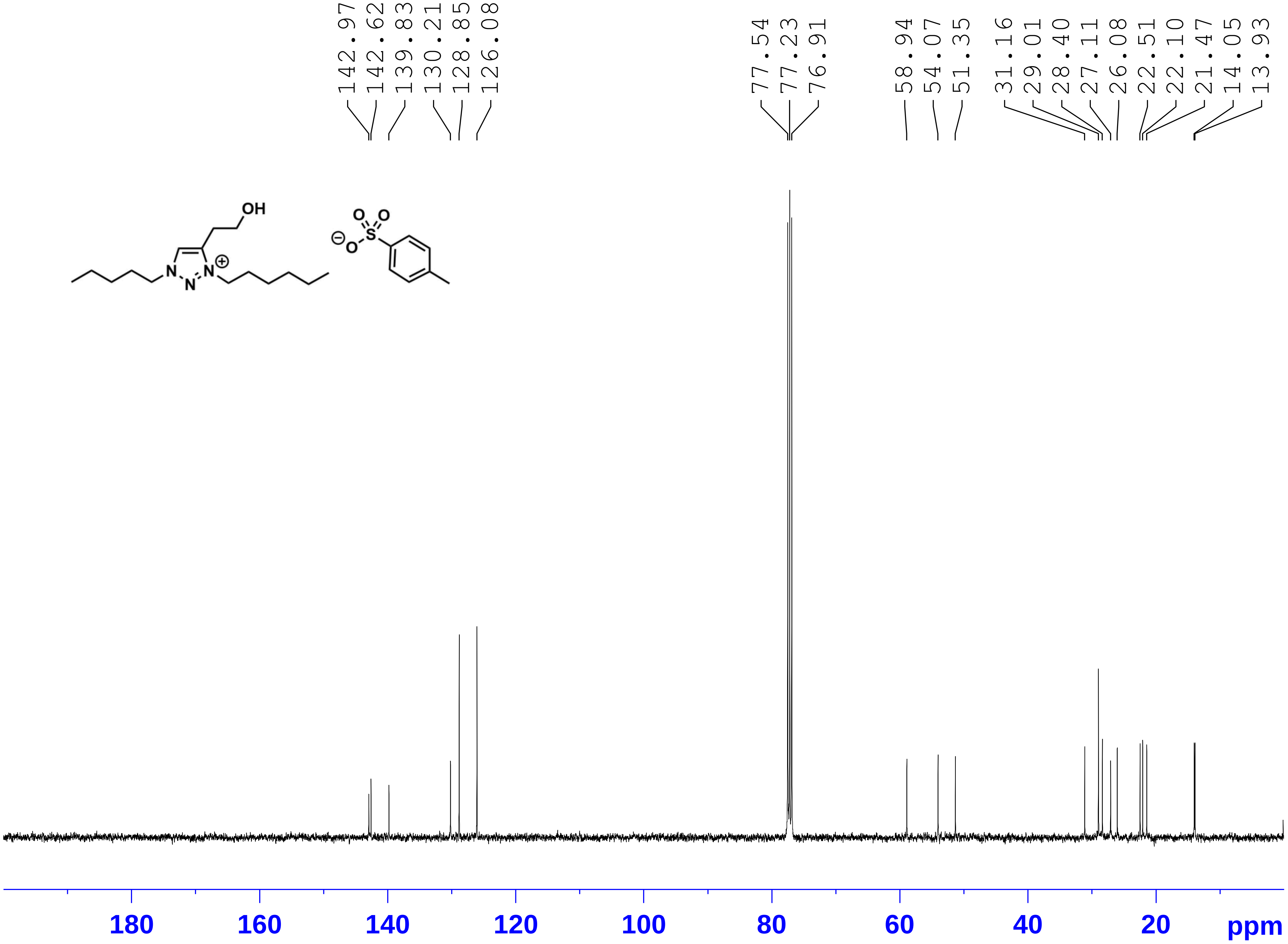
142.97
142.62
139.83
130.21
128.85
126.08
77.54
77.23
76.91
58.94
54.07
51.35
31.16
29.01
28.40
27.11
26.08
22.51
22.10
21.47
14.05
13.93
OH
N
N
N
O
O
S
O
180
160
140
120
100
80
60
40
20
ppm

Spectrum

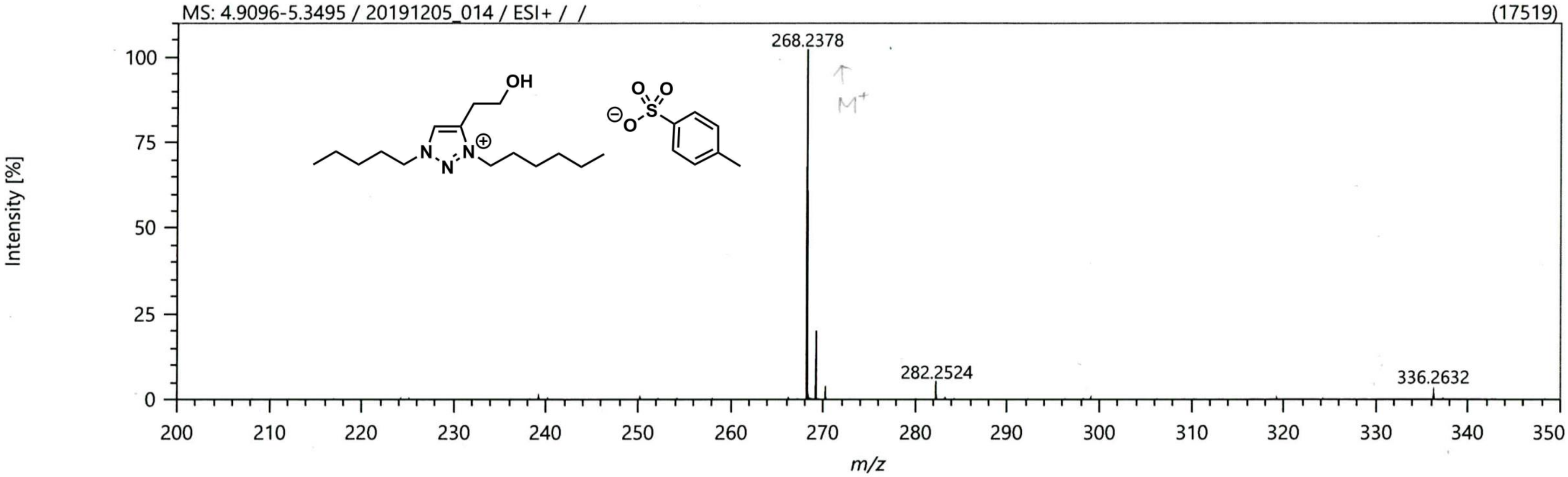

Elemental Composition

Parameters		Elements Set 1:										
Tolerance:	±50.00 ppm	Symbol	C	H	O	Na	N	S	P	Si	F	Cl
Electron:	Odd/Even	Min	0	0	1	0	3	0	0	0	0	0
Charge:	+1	Max	400	1000	1	0	3	0	0	0	0	0
DBE:	-1.5 - 999.0											

Results

Mass	Formula	Calculated Mass	Mass Difference [mDa]	Mass Difference [ppm]	DBE
268.23778	C15 H30 N3 O	268.23834	-0.56	-2.10	2.5

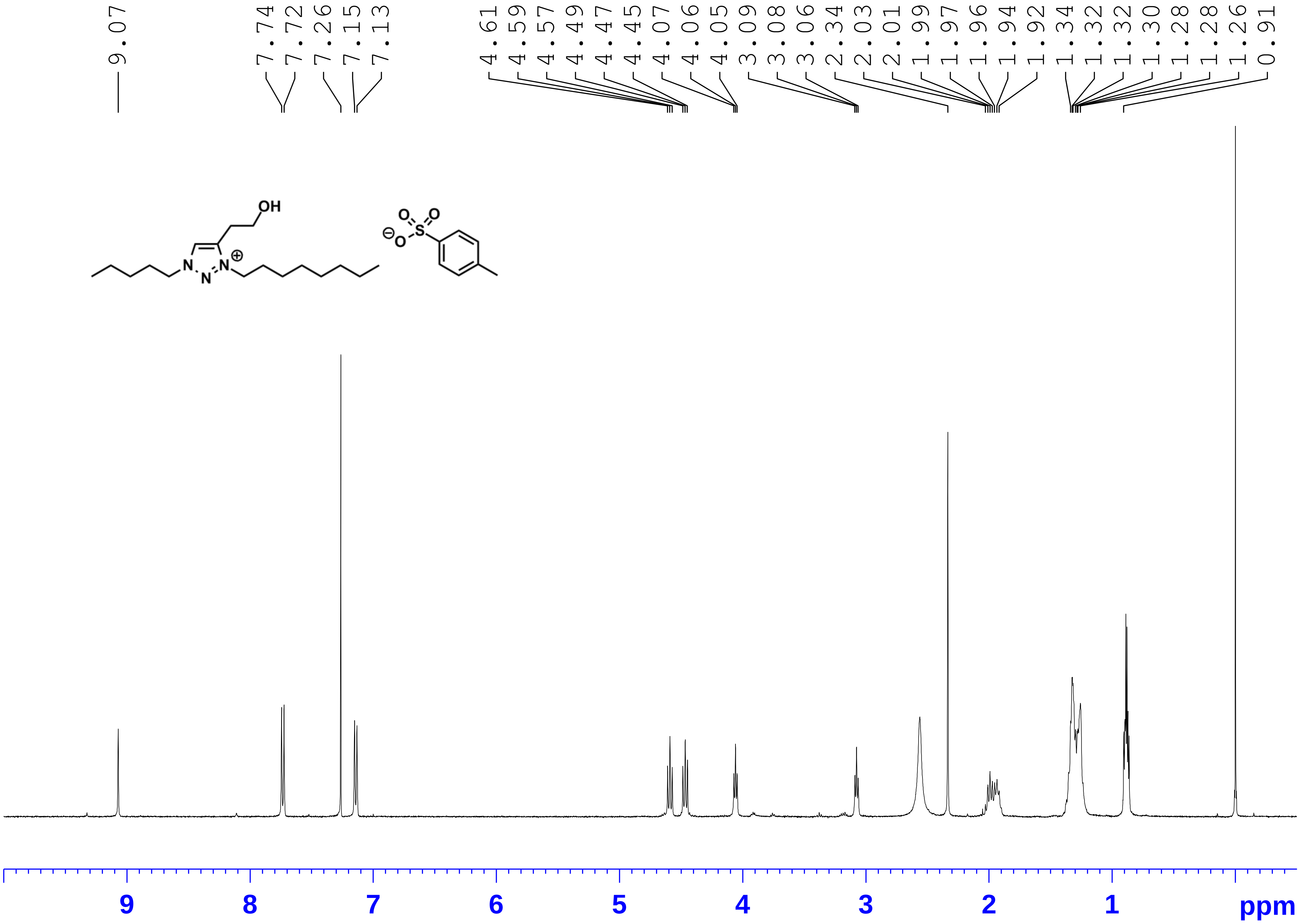

9.07
7.74
7.72
7.26
7.15
7.13
4.61
4.59
4.57
4.49
4.47
4.45
4.07
4.06
4.05
3.09
3.08
3.06
2.34
2.03
2.01
1.99
1.97
1.96
1.94
1.92
1.34
1.32
1.32
1.30
1.28
1.28
1.26
0.91
OH
N
N
N
O
O
S
O
9
8
7
6
5
4
3
2
1
ppm

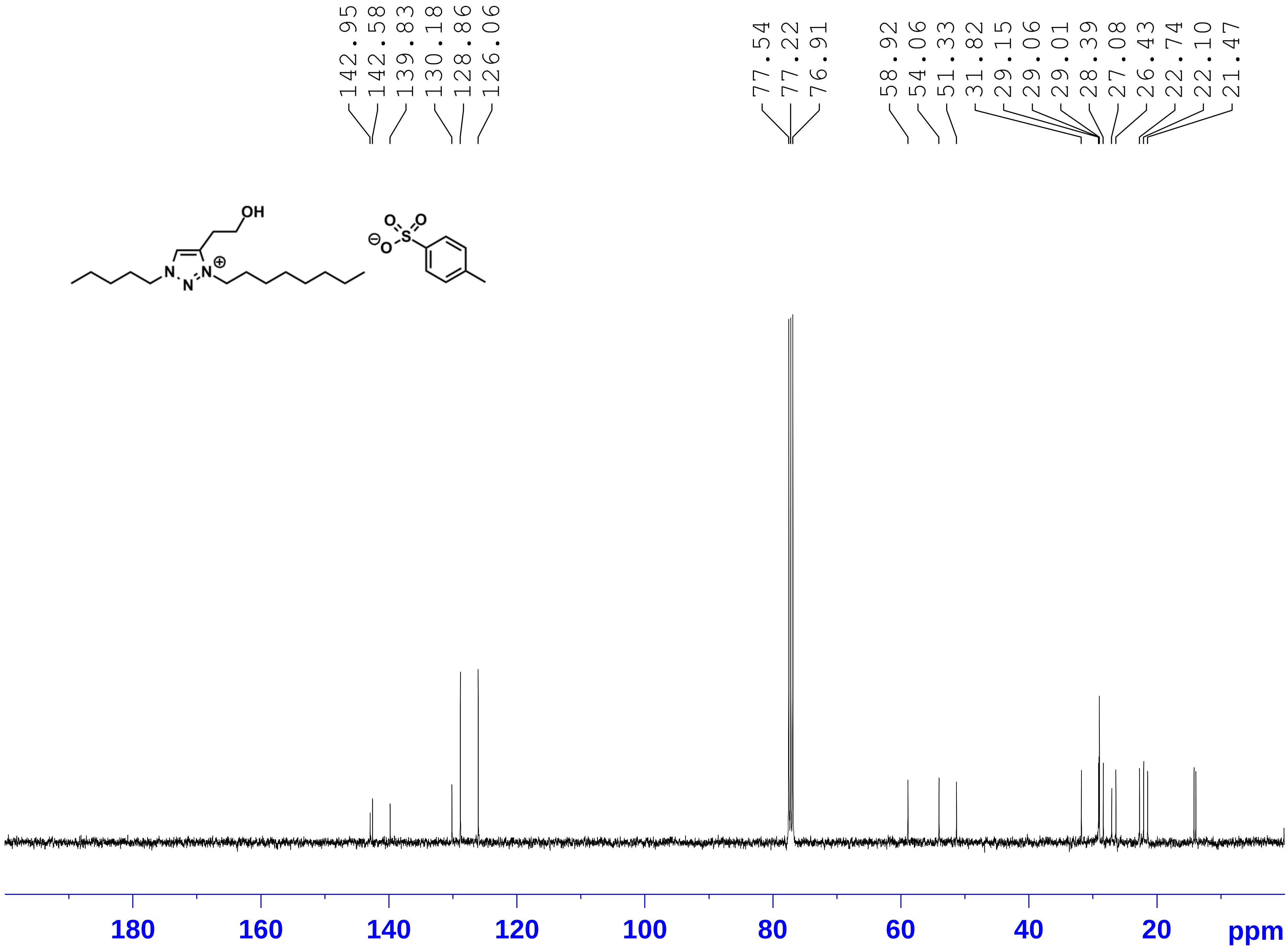

142.95
142.58
139.83
130.18
128.86
126.06
77.54
77.22
76.91
58.92
54.06
51.33
31.82
29.15
29.06
29.01
28.39
27.08
26.43
22.74
22.10
21.47
OH
N
N
N
O
S
O
O
180
160
140
120
100
80
60
40
20
ppm

Spectrum

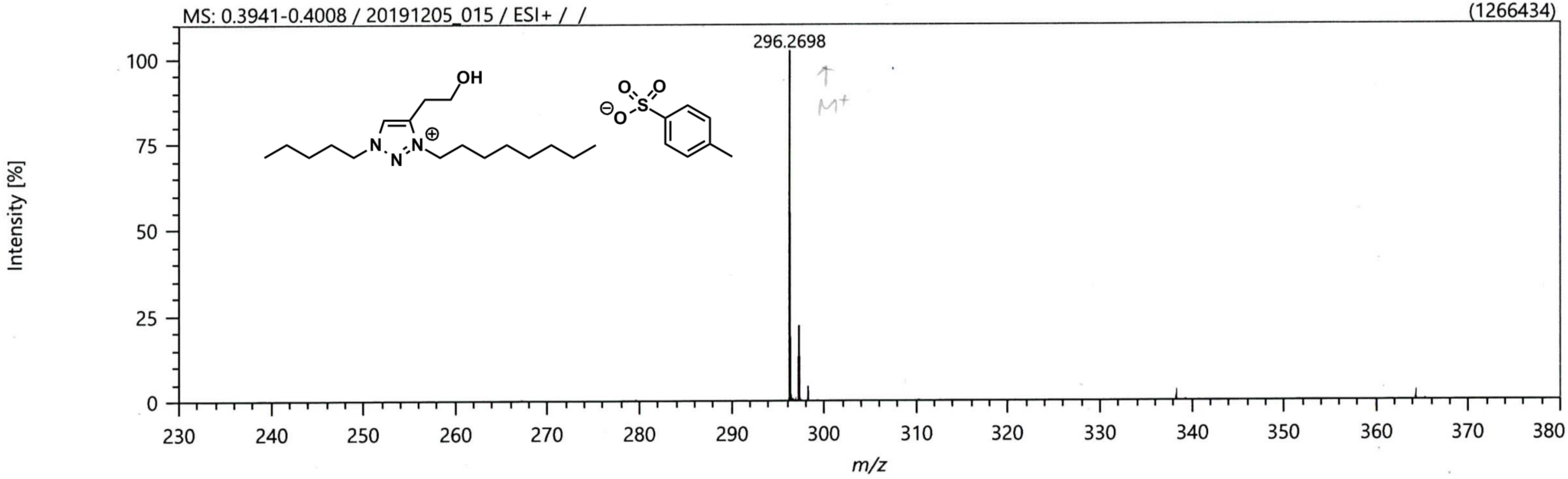

Elemental Composition

Parameters	
Tolerance:	±50.00 ppm
Electron:	Odd/Even
Charge:	+1
DBE:	-1.5 - 999.0

Elements Set 1:

Symbol	C	H	O	Na	N	S	P	Si	F	Cl
Min	0	0	1	0	3	0	0	0	0	0
Max	400	1000	1	0	3	0	0	0	0	0

Results

Mass	Formula	Calculated Mass	Mass Difference [mDa]	Mass Difference [ppm]	DBE
296.26983	C17 H34 N3 O	296.26964	0.19	0.63	2.5

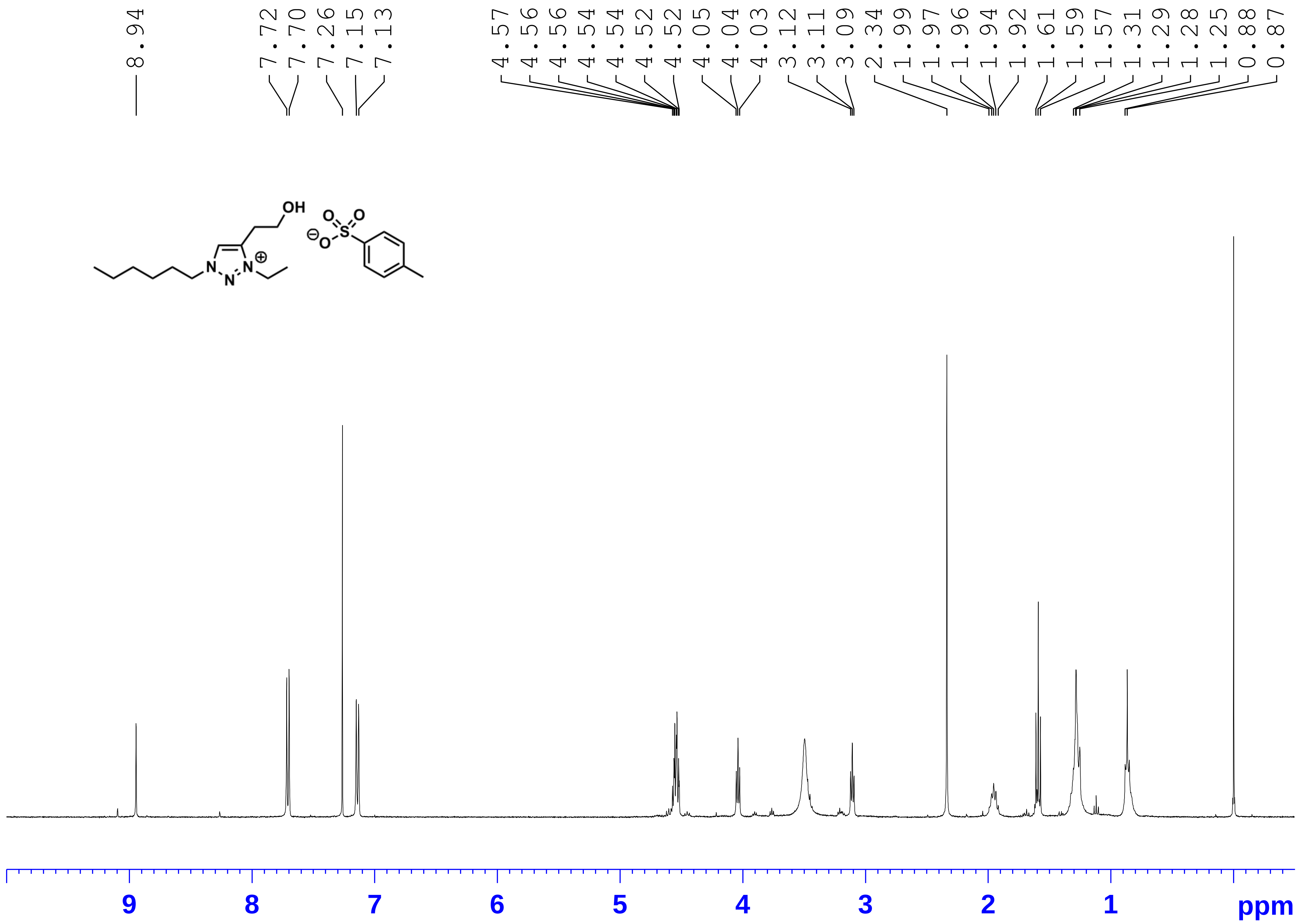
8.94
7.72
7.70
7.26
7.15
7.13
4.57
4.56
4.56
4.54
4.54
4.52
4.52
4.05
4.04
4.03
3.12
3.11
3.09
2.34
1.99
1.97
1.96
1.94
1.92
1.61
1.59
1.57
1.31
1.29
1.28
1.25
0.88
0.87
OH
O
O
S
O
N
N
N
9
8
7
6
5
4
3
2
1
ppm

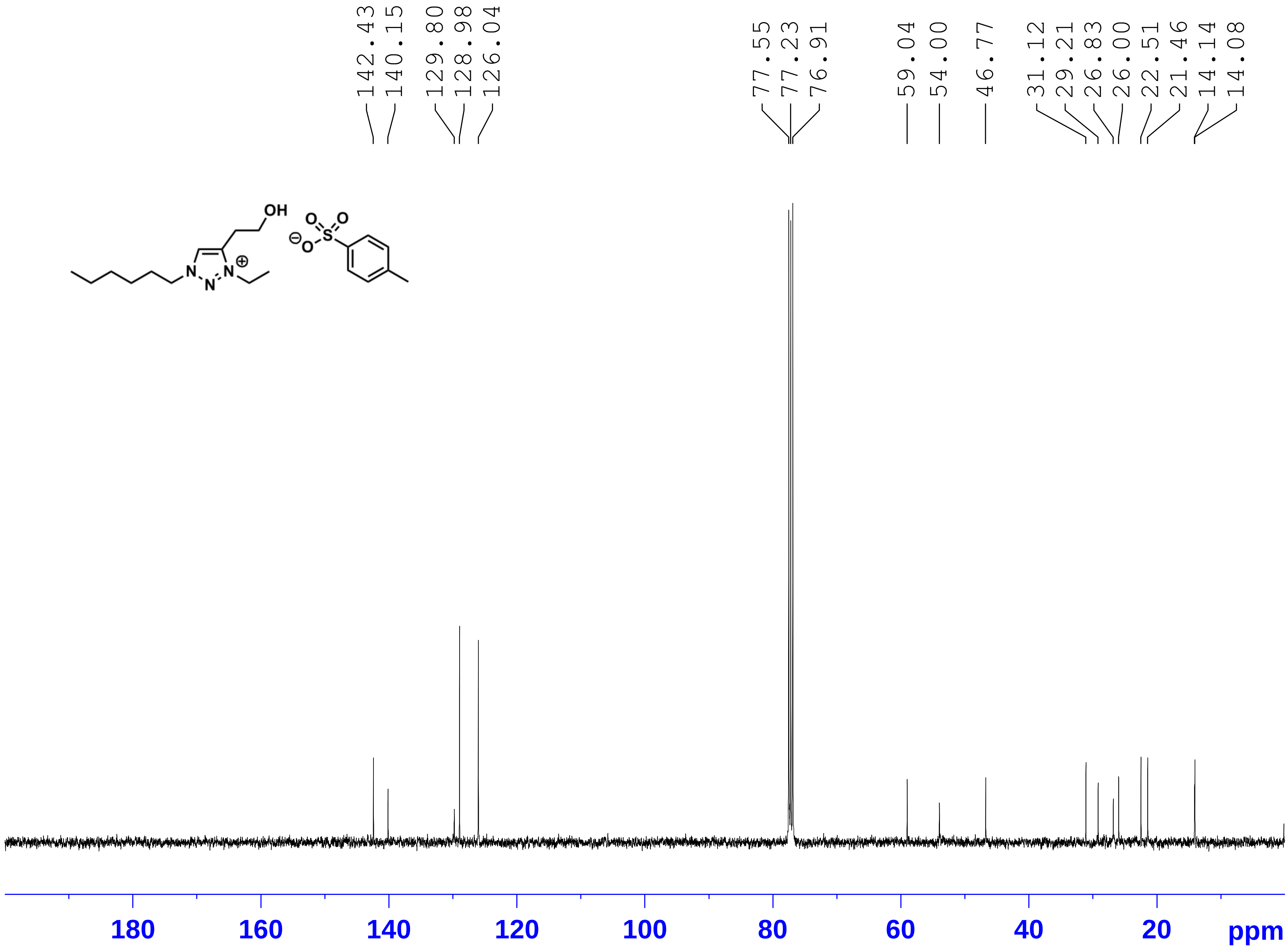

142.43
140.15
129.80
128.98
126.04
77.55
77.23
76.91
59.04
54.00
46.77
31.12
29.21
26.83
26.00
22.51
21.46
14.14
14.08
OH
O
S
N
180
160
140
120
100
80
60
40
20
ppm

Spectrum

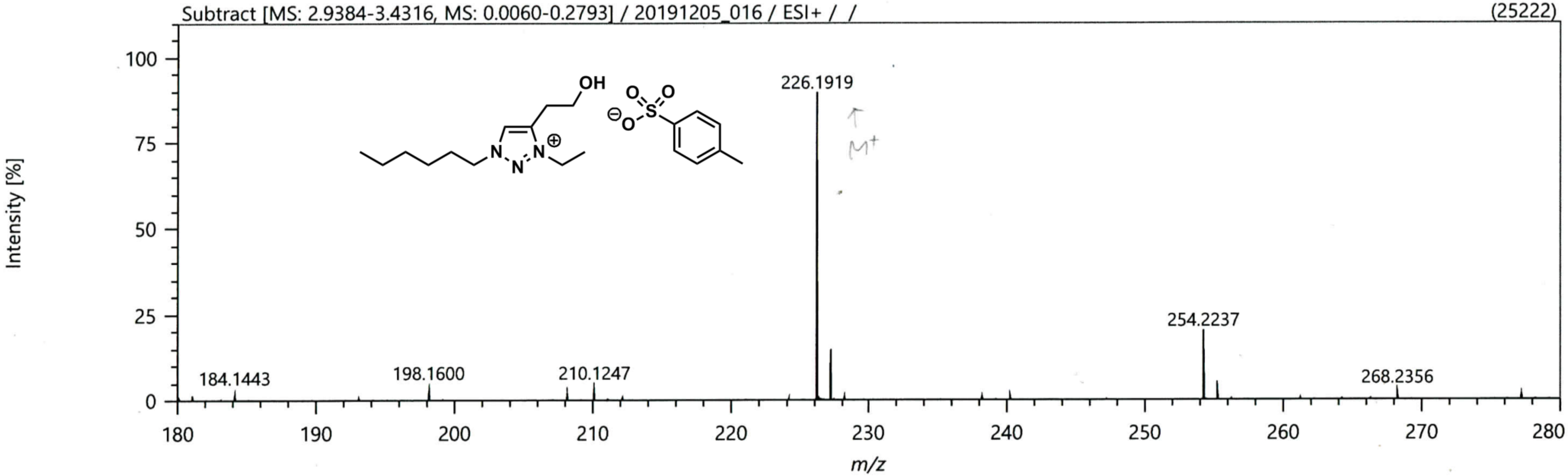

Elemental Composition

Parameters	
Tolerance:	±50.00 ppm
Electron:	Odd/Even
Charge:	+1
DBE:	-1.5 - 999.0

Elements Set 1:

Symbol	C	H	O	Na	N	S	P	Si	F	Cl
Min	0	0	1	0	3	0	0	0	0	0
Max	400	1000	1	0	3	0	0	0	0	0

Results

Mass	Formula	Calculated Mass	Mass Difference [mDa]	Mass Difference [ppm]	DBE
226.19192	C12 H24 N3 O	226.19139	0.53	2.35	2.5

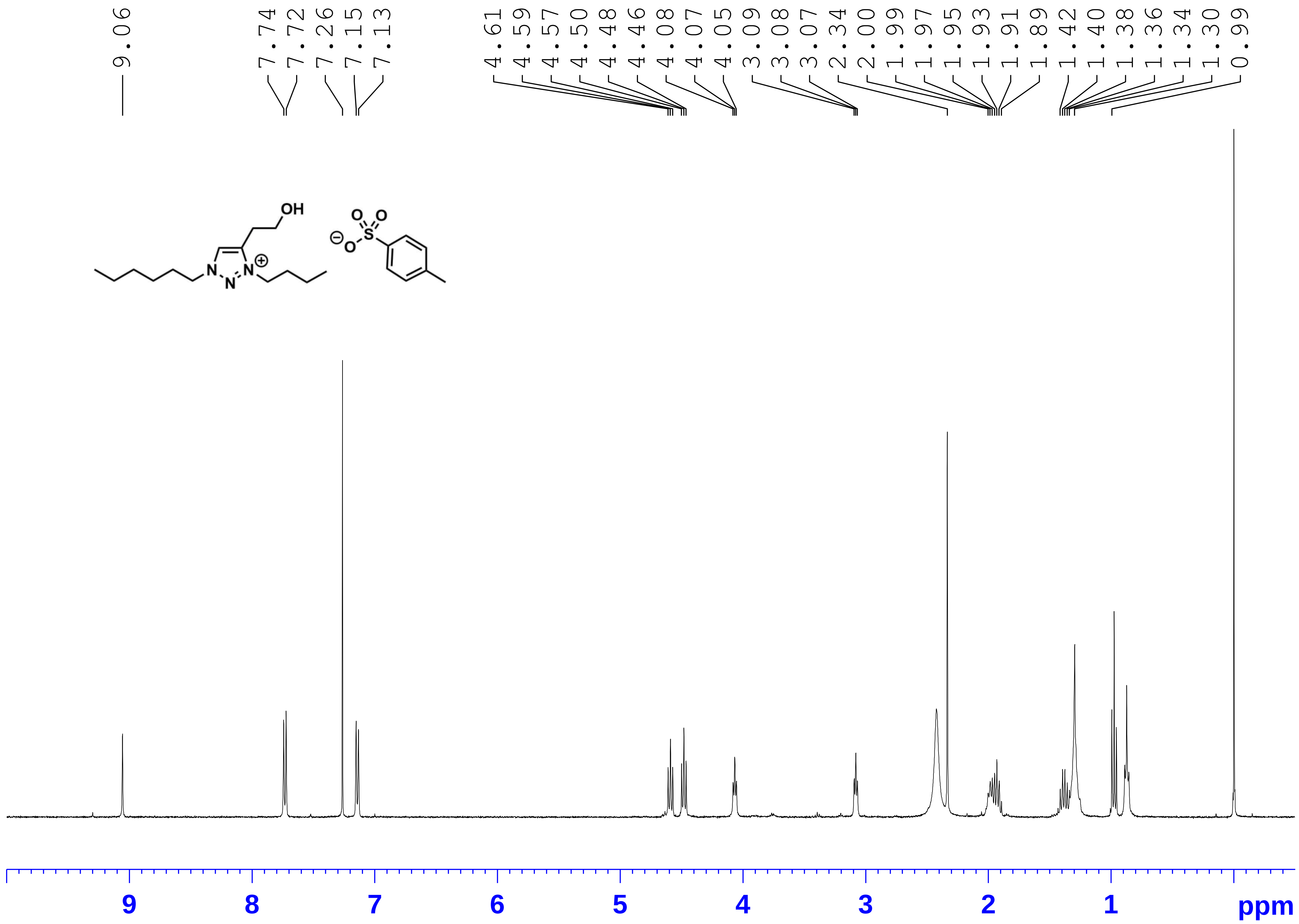

9.06
7.74
7.72
7.26
7.15
7.13
4.61
4.59
4.57
4.50
4.48
4.46
4.08
4.07
4.05
3.09
3.08
3.07
2.34
2.00
1.99
1.97
1.95
1.93
1.91
1.89
1.42
1.40
1.38
1.36
1.34
1.30
0.99
OH
O
O
S
O
N
N
N
9
8
7
6
5
4
3
2
1
ppm

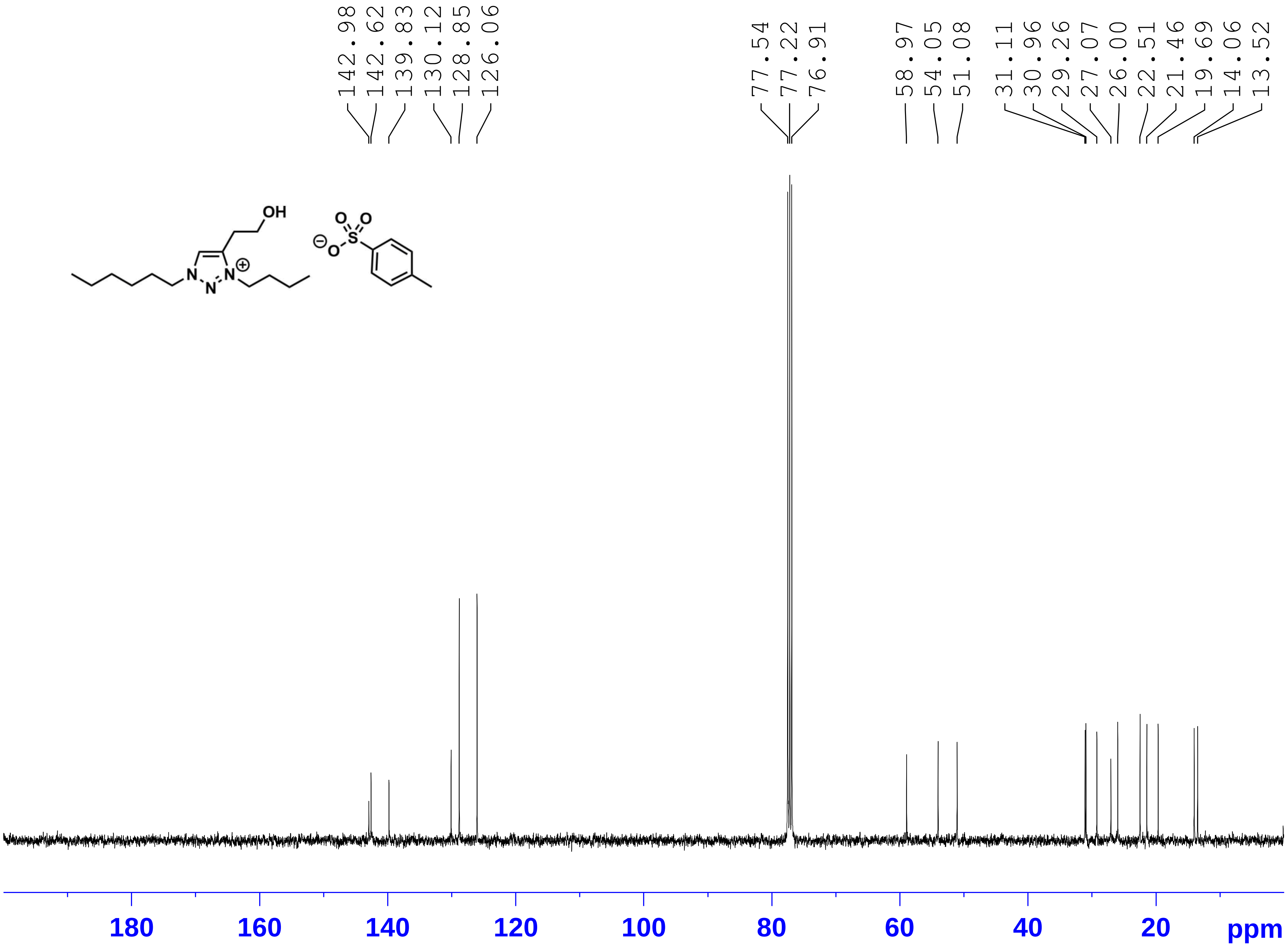

142.98
142.62
139.83
130.12
128.85
126.06
77.54
77.22
76.91
58.97
54.05
51.08
31.11
30.96
29.26
27.07
26.00
22.51
21.46
19.69
14.06
13.52
OH
O
O
S
O
N
N
N
180
160
140
120
100
80
60
40
20
ppm

Spectrum

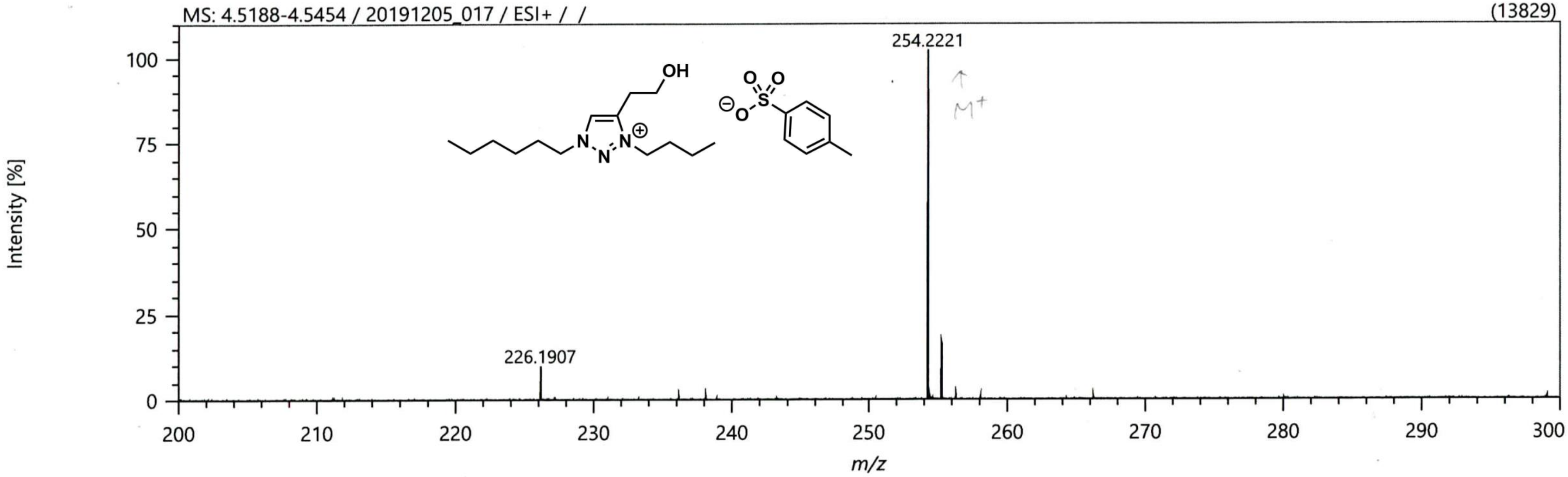

Elemental Composition

Parameters	
Tolerance:	±50.00 ppm
Electron:	Odd/Even
Charge:	+1
DBE:	-1.5 - 999.0

Elements Set 1:

Symbol	C	H	O	Na	N	S	P	Si	F	Cl
Min	0	0	1	0	3	0	0	0	0	0
Max	400	1000	1	0	3	0	0	0	0	0

Results

Mass	Formula	Calculated Mass	Mass Difference [mDa]	Mass Difference [ppm]	DBE
254.22205	C14 H28 N3 O	254.22269	-0.64	-2.51	2.5

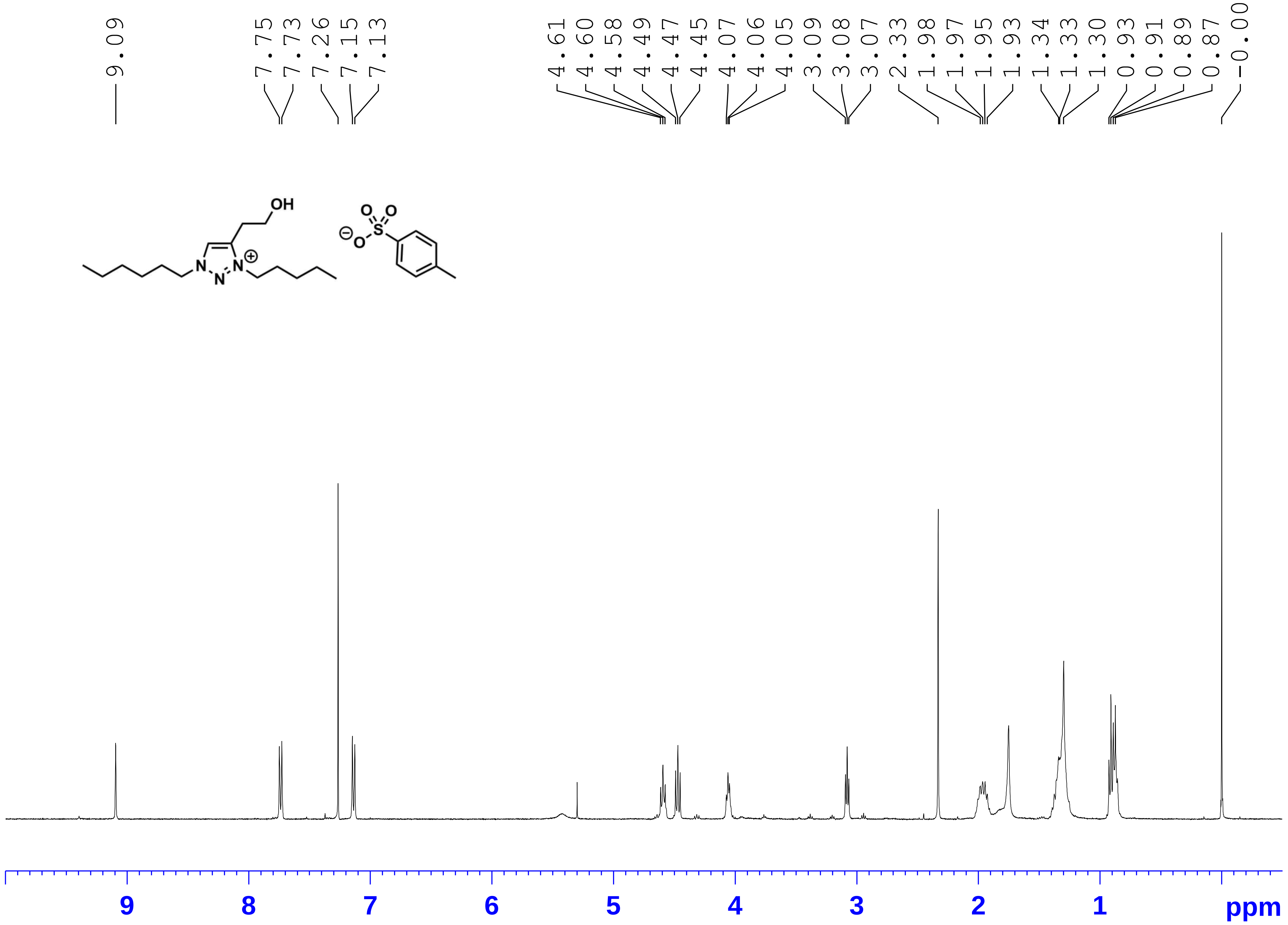
9.09
7.75
7.73
7.26
7.15
7.13
4.61
4.60
4.58
4.49
4.47
4.45
4.07
4.06
4.05
3.09
3.08
3.07
2.33
1.98
1.97
1.95
1.93
1.34
1.33
1.30
0.93
0.91
0.89
0.87
-0.00
OH
N
N
N
O
O
S
O
9
8
7
6
5
4
3
2
1
ppm

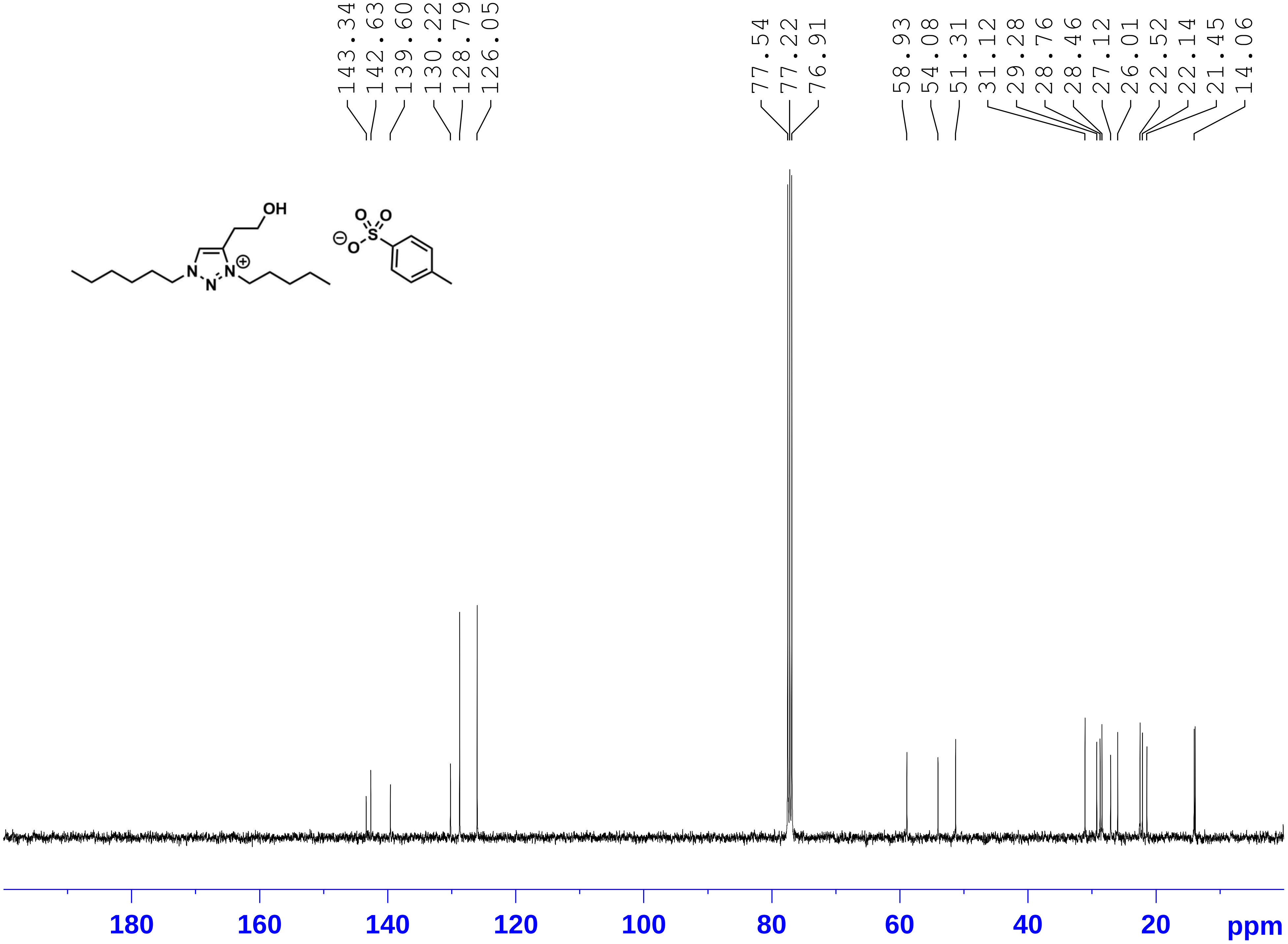

143.34
142.63
139.60
130.22
128.79
126.05
77.54
77.22
76.91
58.93
54.08
51.31
31.12
29.28
28.76
28.46
27.12
26.01
22.52
22.14
21.45
14.06
OH
N
N
N
O
O
S
O
180
160
140
120
100
80
60
40
20
ppm

Spectrum

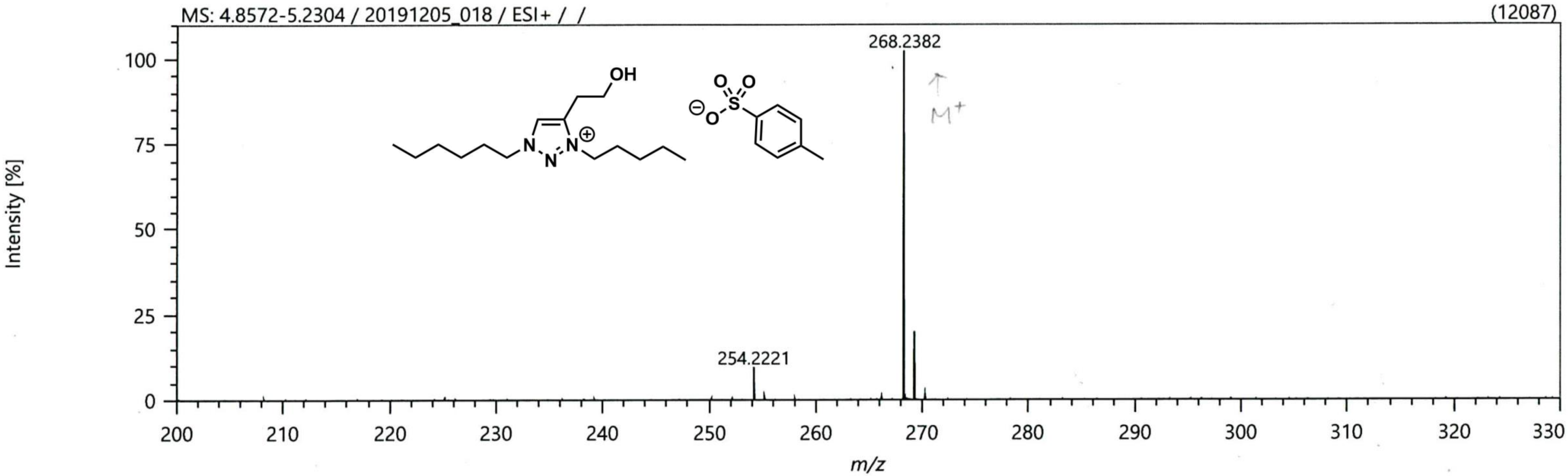

Elemental Composition

Parameters		Elements Set 1:										
Tolerance:	±50.00 ppm	Symbol	C	H	O	Na	N	S	P	Si	F	Cl
Electron:	Odd/Even	Min	0	0	1	0	3	0	0	0	0	0
Charge:	+1	Max	400	1000	1	0	3	0	0	0	0	0
DBE:	-1.5 - 999.0											

Results

Mass	Formula	Calculated Mass	Mass Difference [mDa]	Mass Difference [ppm]	DBE
268.23824	C15 H30 N3 O	268.23834	-0.10	-0.38	2.5

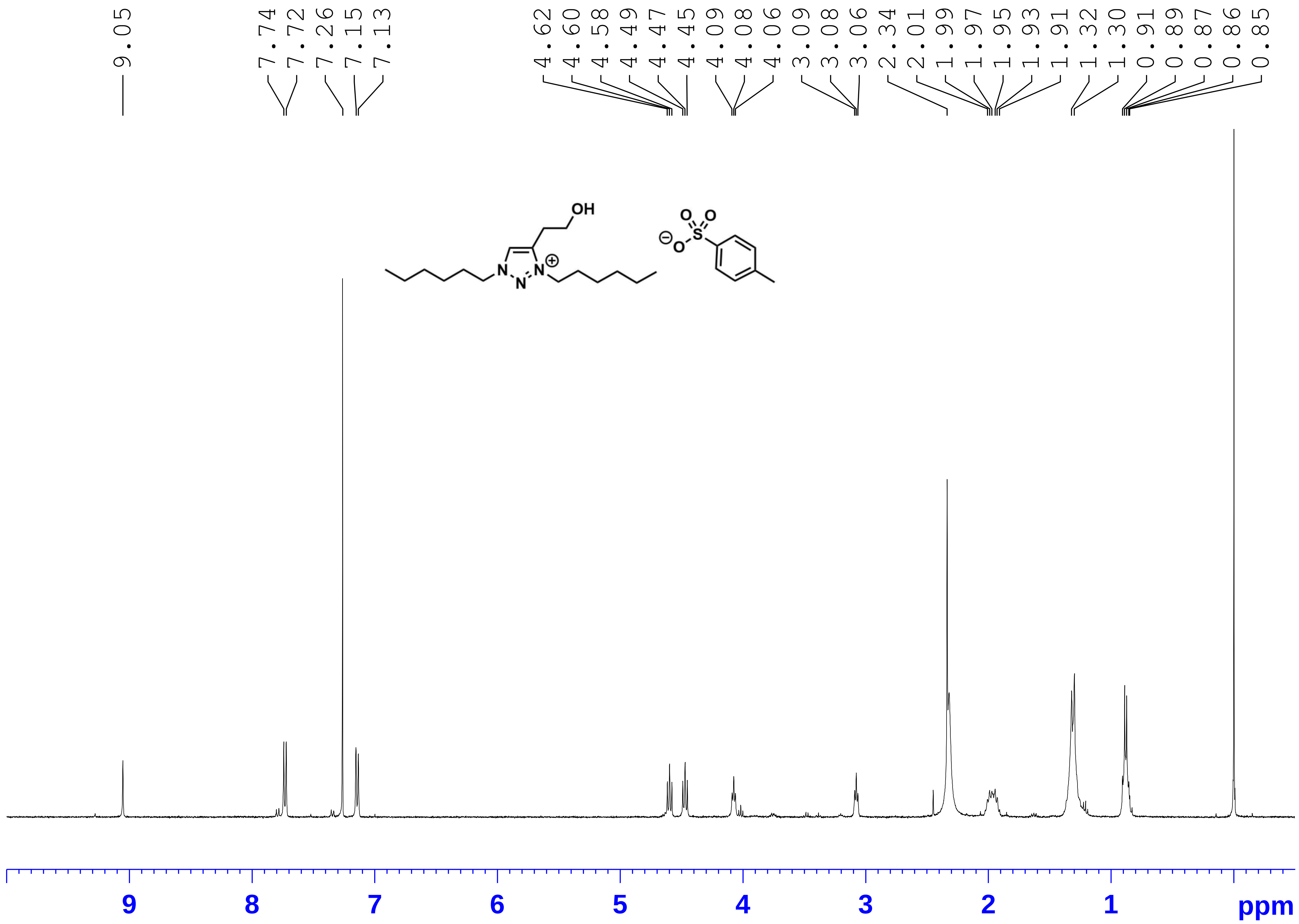

9.05
7.74
7.72
7.26
7.15
7.13
4.62
4.60
4.58
4.49
4.47
4.45
4.09
4.08
4.06
3.09
3.08
3.06
2.34
2.01
1.99
1.97
1.95
1.93
1.91
1.32
1.30
0.91
0.89
0.87
0.86
0.85
OH
N
N
N
O
O
S
O
9
8
7
6
5
4
3
2
1
ppm

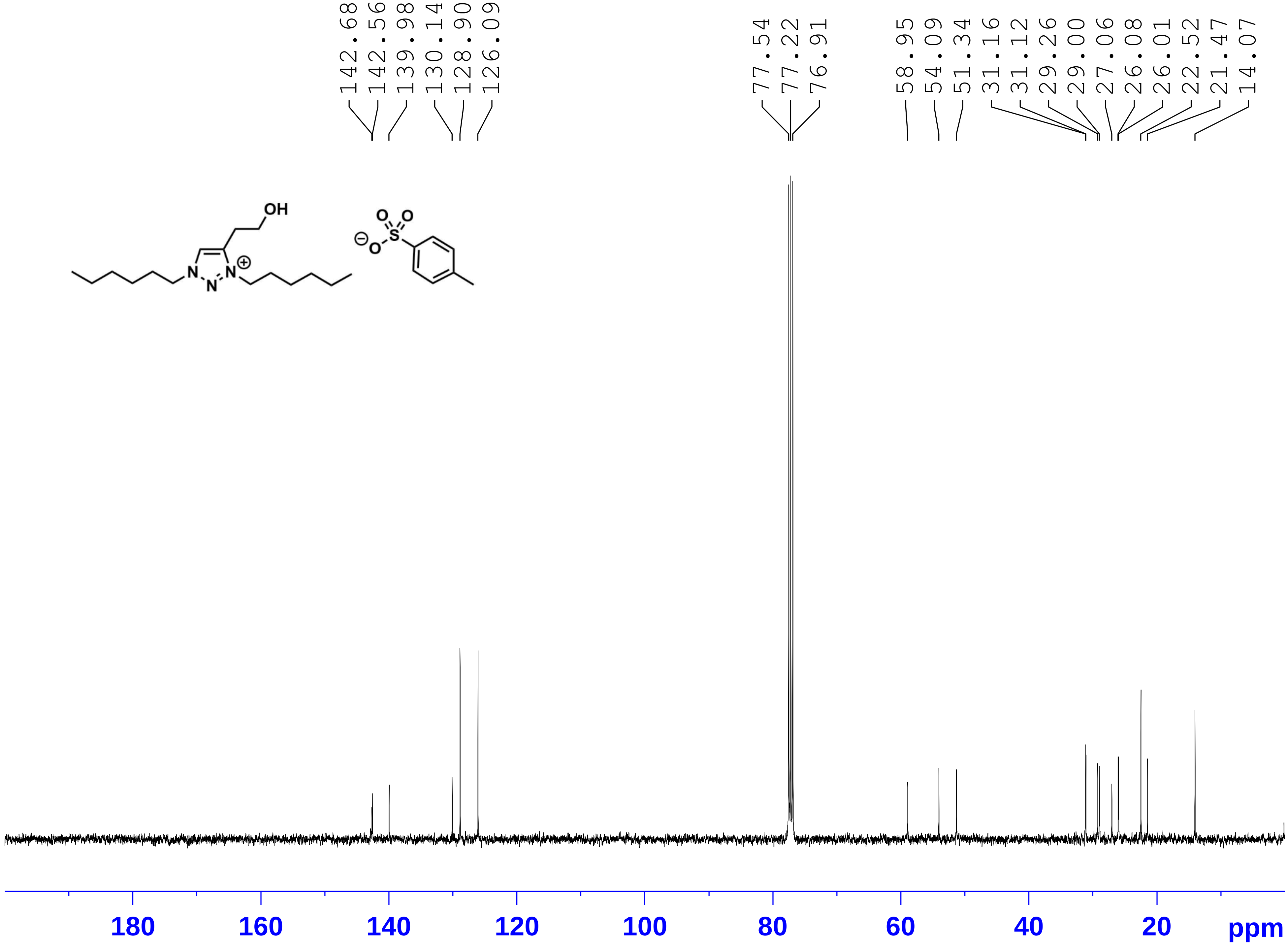

142.68
142.56
139.98
130.14
128.90
126.09
77.54
77.22
76.91
58.95
54.09
51.34
31.16
31.12
29.26
29.00
27.06
26.08
26.01
22.52
21.47
14.07
OH
N
N
N
S
O
O
O
180
160
140
120
100
80
60
40
20
ppm

Spectrum

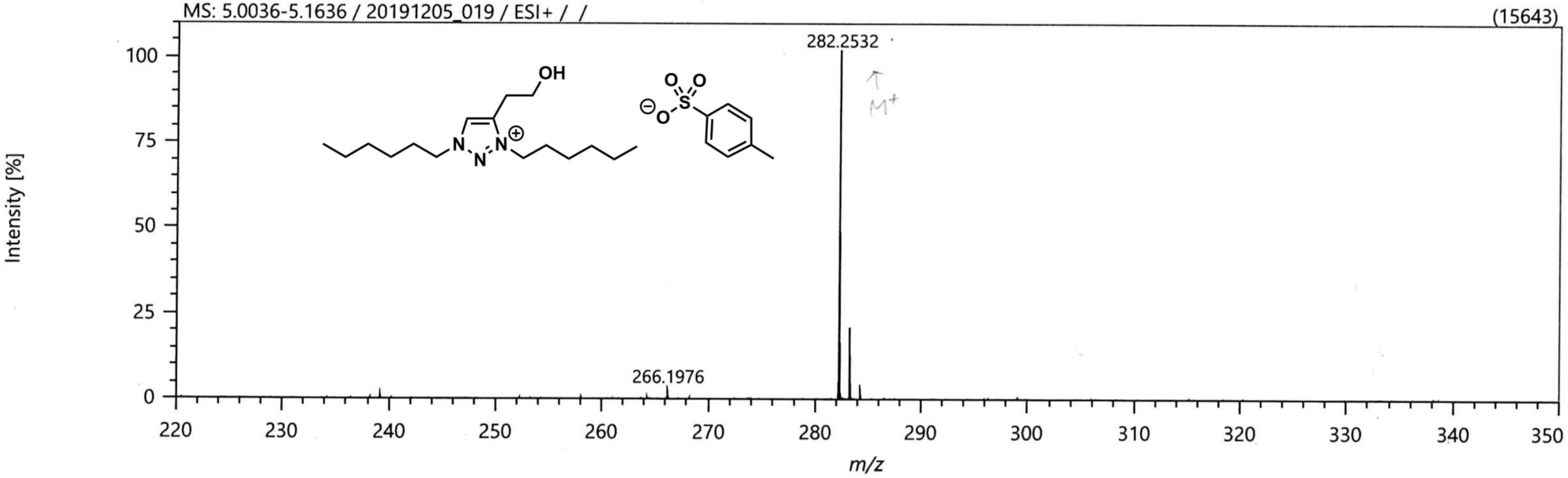

Elemental Composition

Parameters

Tolerance:	±50.00 ppm
Electron:	Odd/Even
Charge:	+1
DBE:	-1.5 - 999.0

Elements Set 1:

Symbol	C	H	O	Na	N	S	P	Si	F	Cl
Min	0	0	1	0	3	0	0	0	0	0
Max	400	1000	1	0	3	0	0	0	0	0

Results

Mass	Formula	Calculated Mass	Mass Difference [mDa]	Mass Difference [ppm]	DBE
282.25320	C16 H32 N3 O	282.25399	-0.79	-2.79	2.5

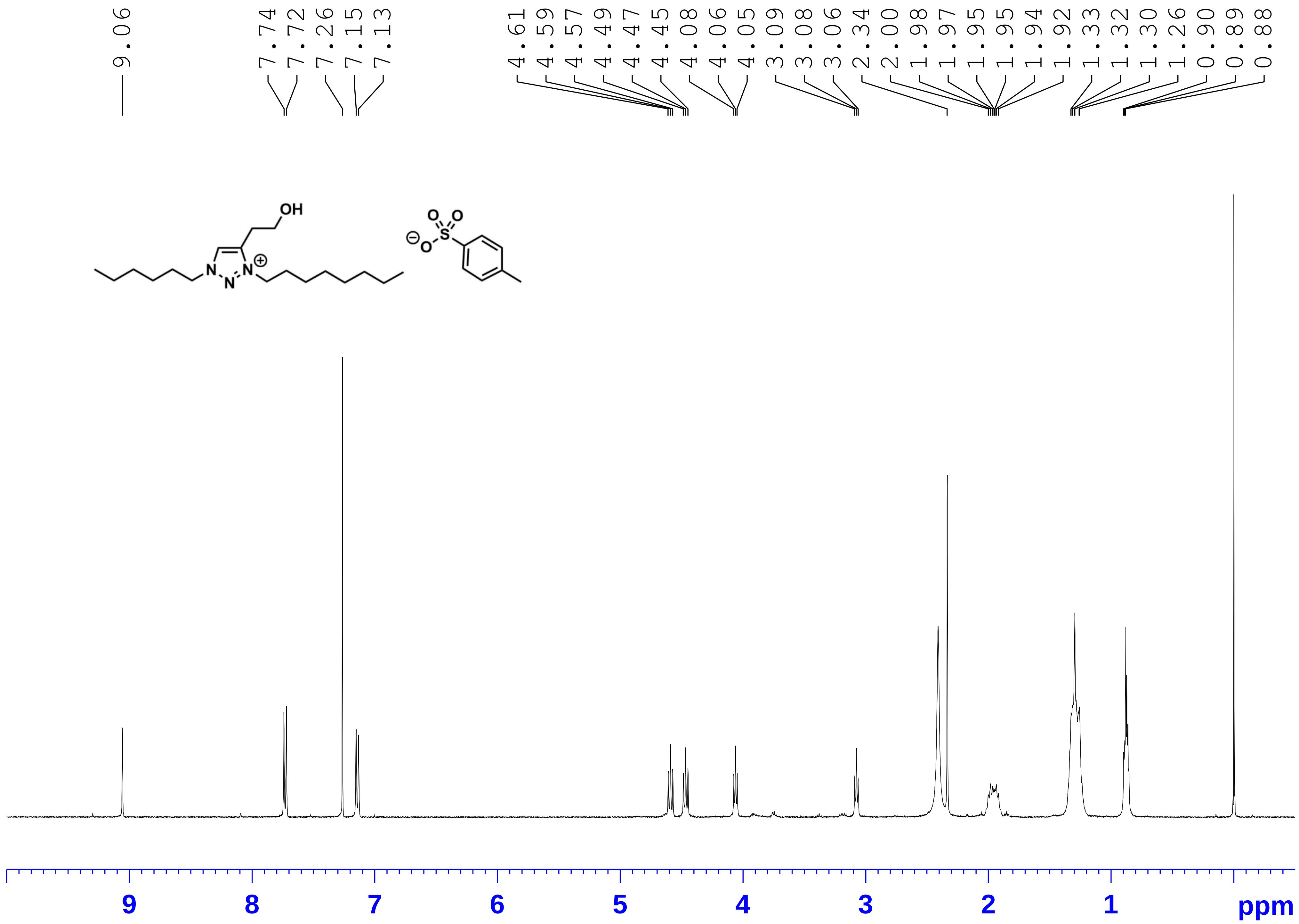
9.06
7.74
7.72
7.26
7.15
7.13
4.61
4.59
4.57
4.49
4.47
4.45
4.08
4.06
4.05
3.09
3.08
3.06
2.34
2.00
1.98
1.97
1.95
1.95
1.94
1.92
1.33
1.32
1.30
1.26
0.90
0.89
0.88
OH
N
N
N
O
O
S
O
9
8
7
6
5
4
3
2
1
ppm

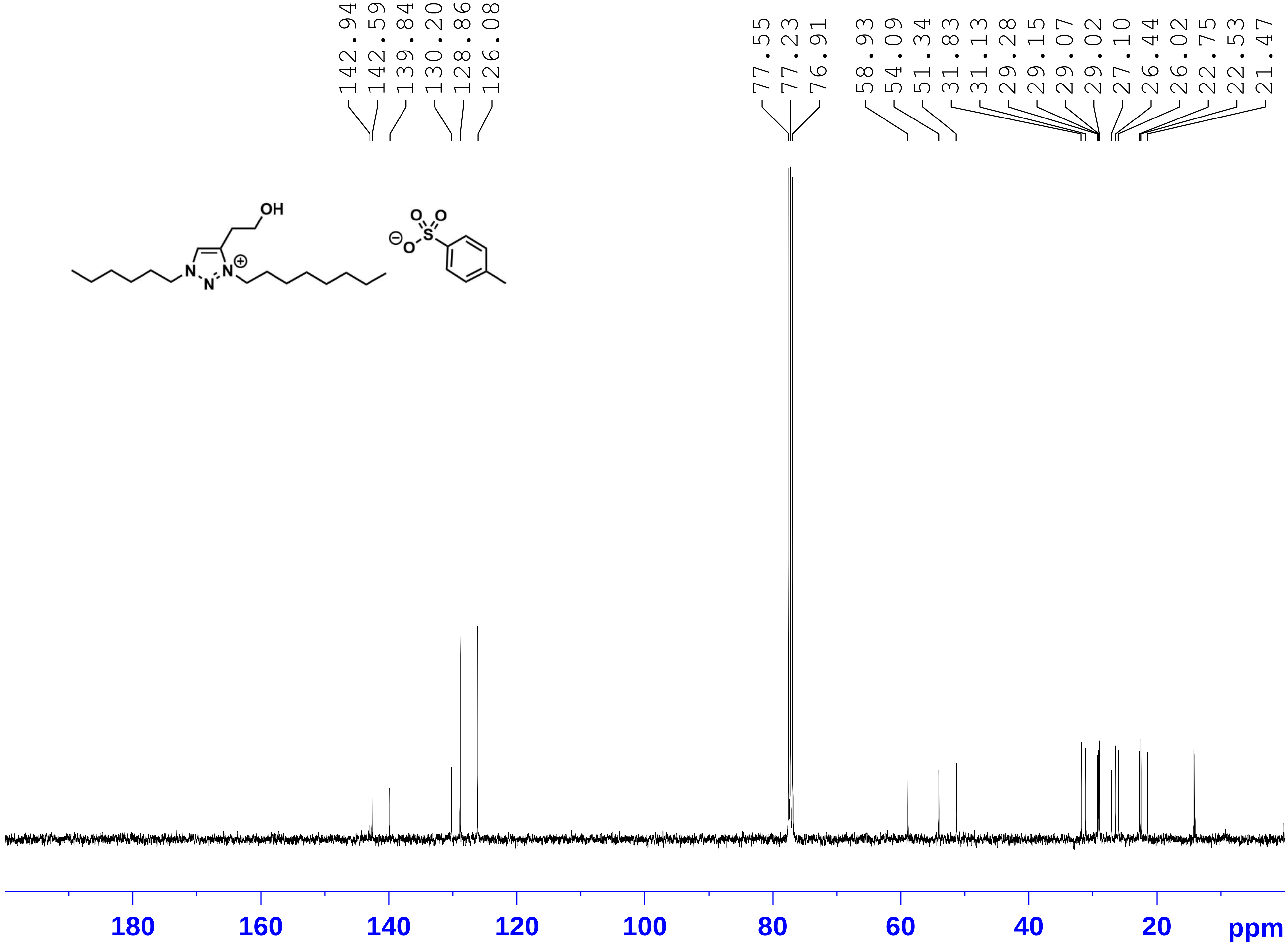

142.94
142.59
139.84
130.20
128.86
126.08
77.55
77.23
76.91
58.93
54.09
51.34
31.83
31.13
29.28
29.15
29.07
29.02
27.10
26.44
26.02
22.75
22.53
21.47
OH
N
N
N
O
O
S
O
180
160
140
120
100
80
60
40
20
ppm

Spectrum

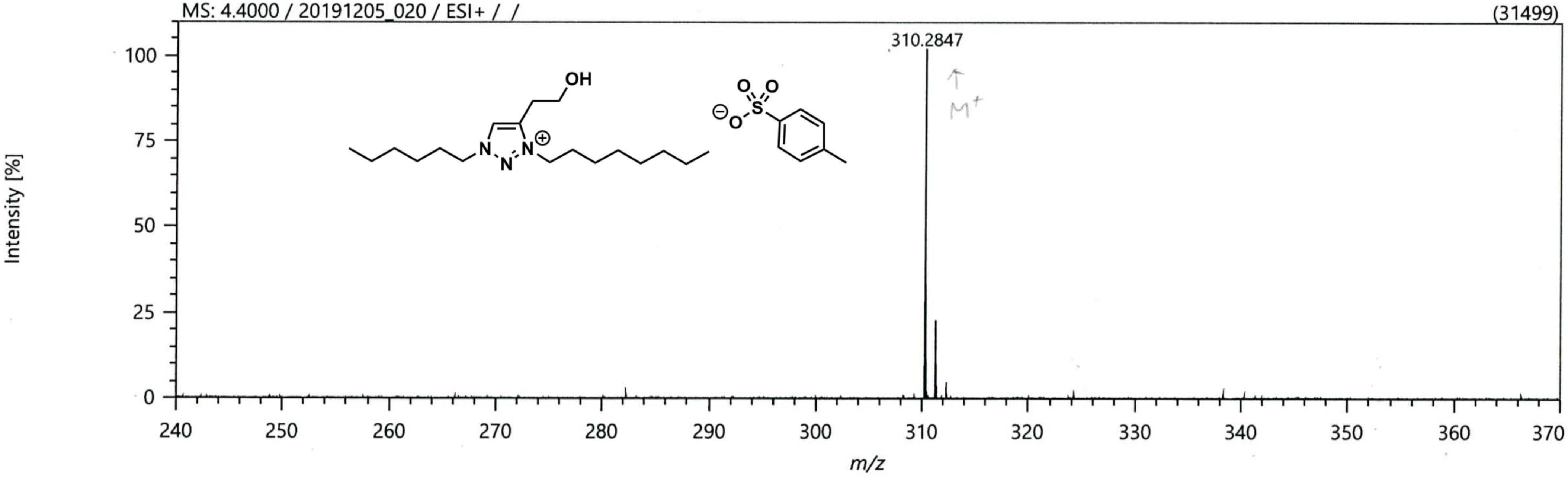

Elemental Composition

Parameters	
Tolerance:	±50.00 ppm
Electron:	Odd/Even
Charge:	+1
DBE:	-1.5 - 999.0

Elements Set 1:

Symbol	C	H	O	Na	N	S	P	Si	F	Cl
Min	0	0	1	0	3	0	0	0	0	0
Max	400	1000	1	0	3	0	0	0	0	0

Results

Mass	Formula	Calculated Mass	Mass Difference [mDa]	Mass Difference [ppm]	DBE
310.28465	C18 H36 N3 O	310.28529	-0.64	-2.06	2.5

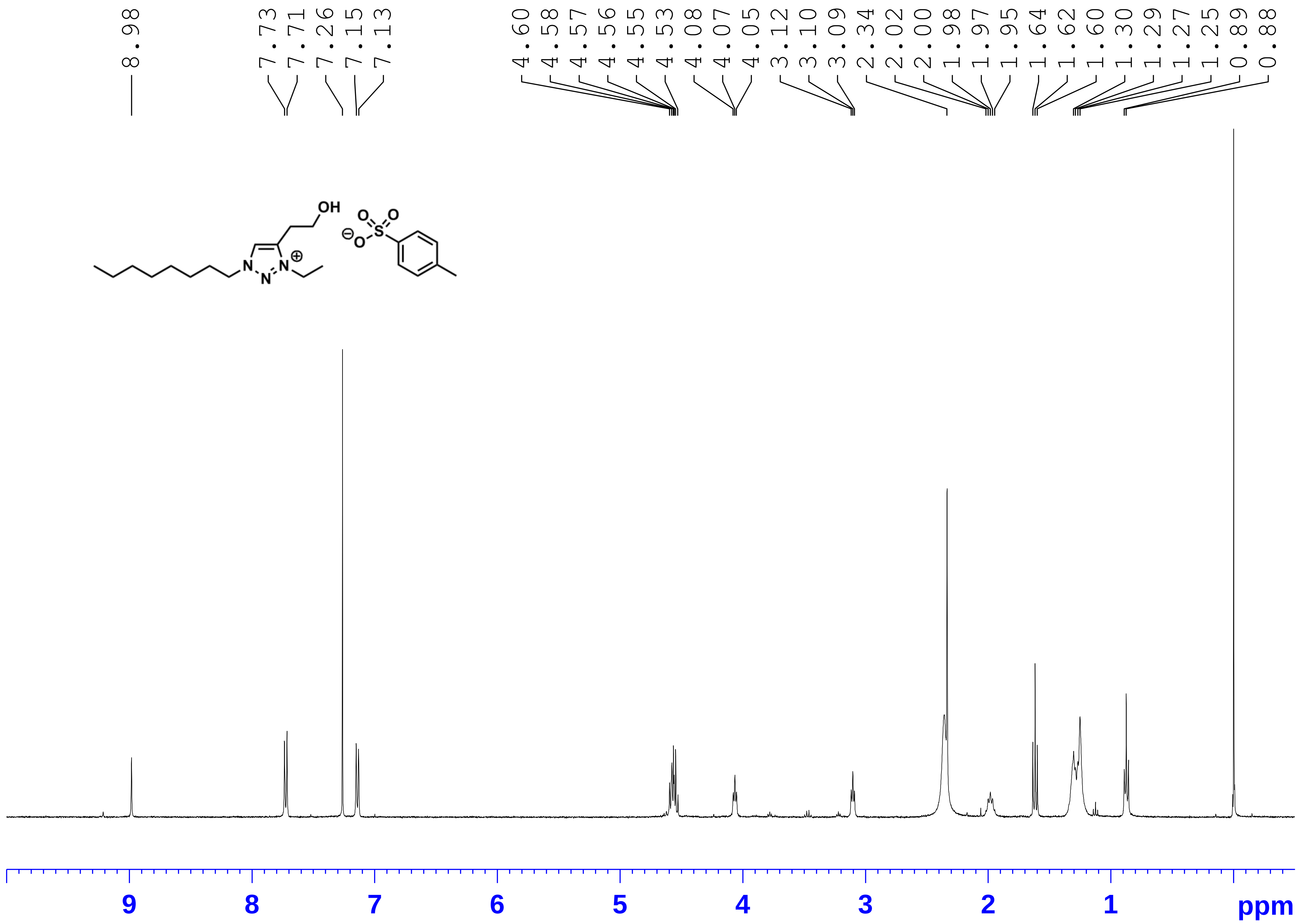

8.98
7.73
7.71
7.26
7.15
7.13
4.60
4.58
4.57
4.56
4.55
4.53
4.08
4.07
4.05
3.12
3.10
3.09
2.34
2.02
2.00
1.98
1.97
1.95
1.64
1.62
1.60
1.30
1.29
1.27
1.25
0.89
0.88
OH
O
O
S
O
N
N
N
9
8
7
6
5
4
3
2
1
ppm

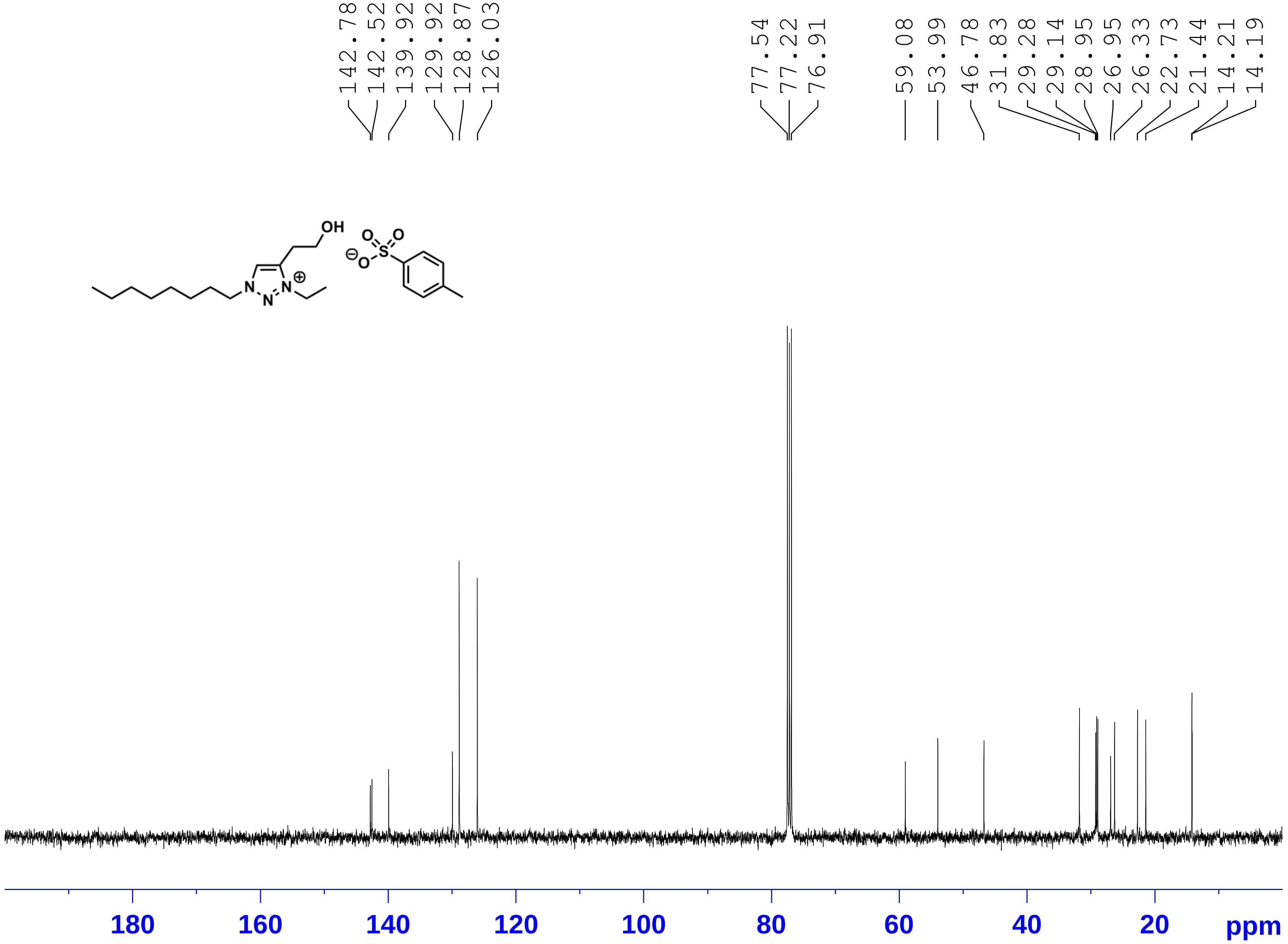
142.78
142.52
139.92
129.92
128.87
126.03
77.54
77.22
76.91
59.08
53.99
46.78
31.83
29.28
29.14
28.95
26.95
26.33
22.73
21.44
14.21
14.19
OH
O
O
S
O
N
N
N
180
160
140
120
100
80
60
40
20
ppm

Spectrum

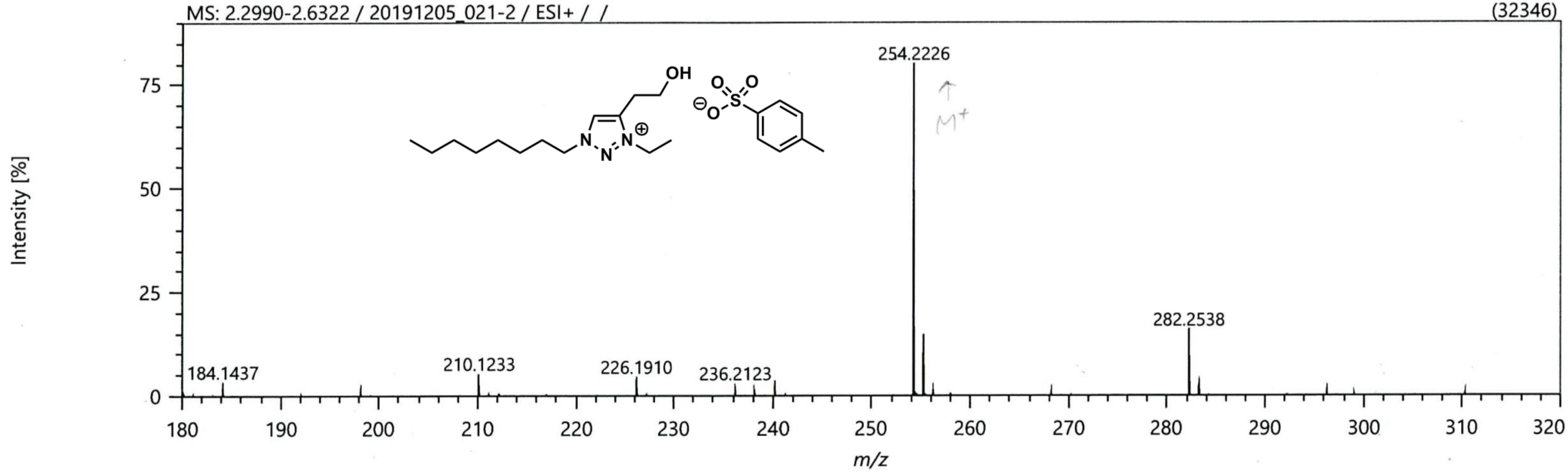

Elemental Composition

Parameters		Elements Set 1:										
Tolerance:	±50.00 ppm	Symbol	C	H	O	Na	N	S	P	Si	F	Cl
Electron:	Odd/Even	Min	0	0	1	0	3	0	0	0	0	0
Charge:	+1	Max	400	1000	1	0	3	0	0	0	0	0
DBE:	-1.5 - 999.0											

Results

Mass	Formula	Calculated Mass	Mass Difference [mDa]	Mass Difference [ppm]	DBE
254.22257	C14 H28 N3 O	254.22269	-0.12	-0.48	2.5

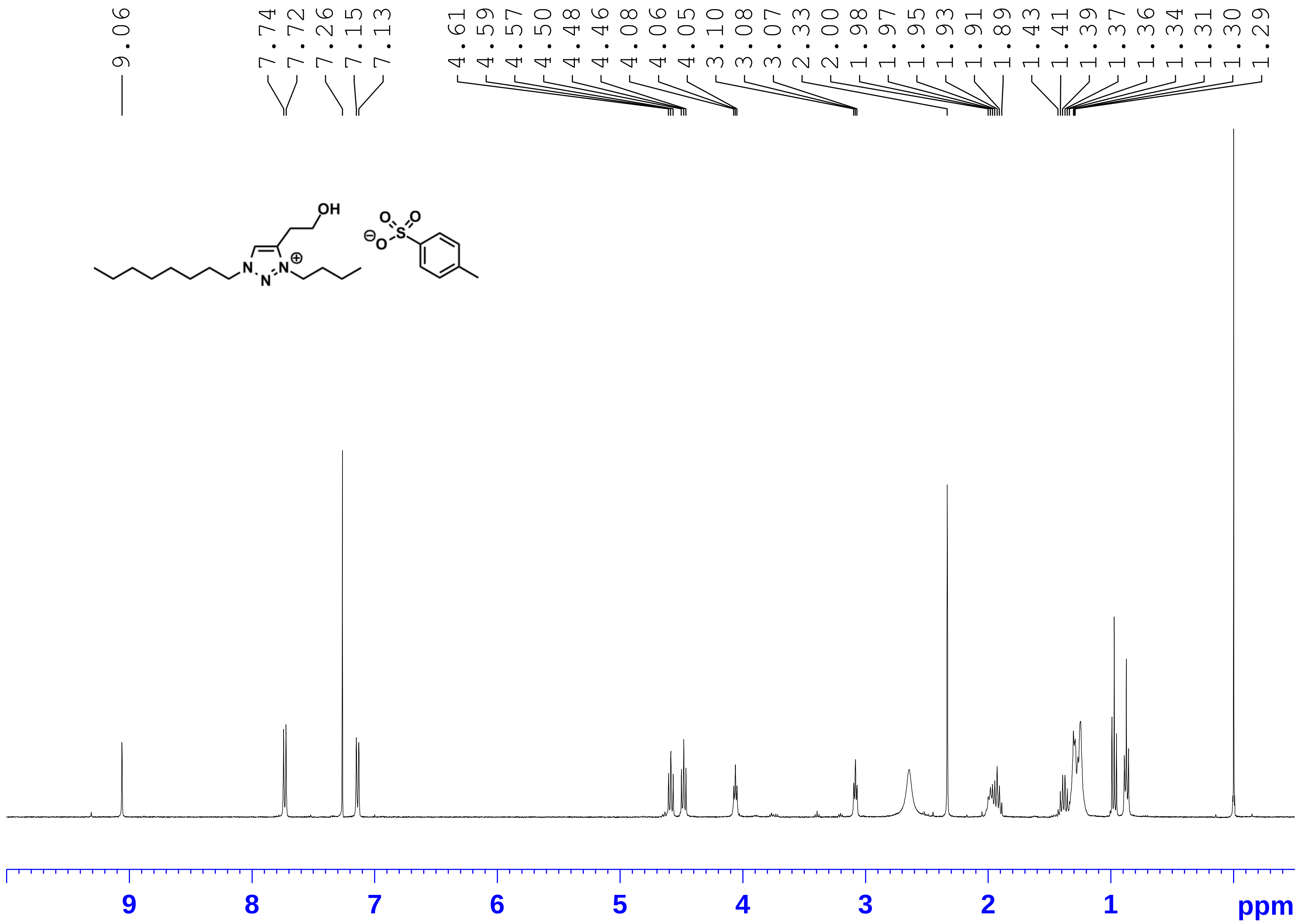
9.06
7.74
7.72
7.26
7.15
7.13
4.61
4.59
4.57
4.50
4.48
4.46
4.08
4.06
4.05
3.10
3.08
3.07
2.33
2.00
1.98
1.97
1.95
1.93
1.91
1.89
1.43
1.41
1.39
1.37
1.36
1.34
1.31
1.30
1.29
OH
N
N
N
O
O
S
O
9
8
7
6
5
4
3
2
1
ppm

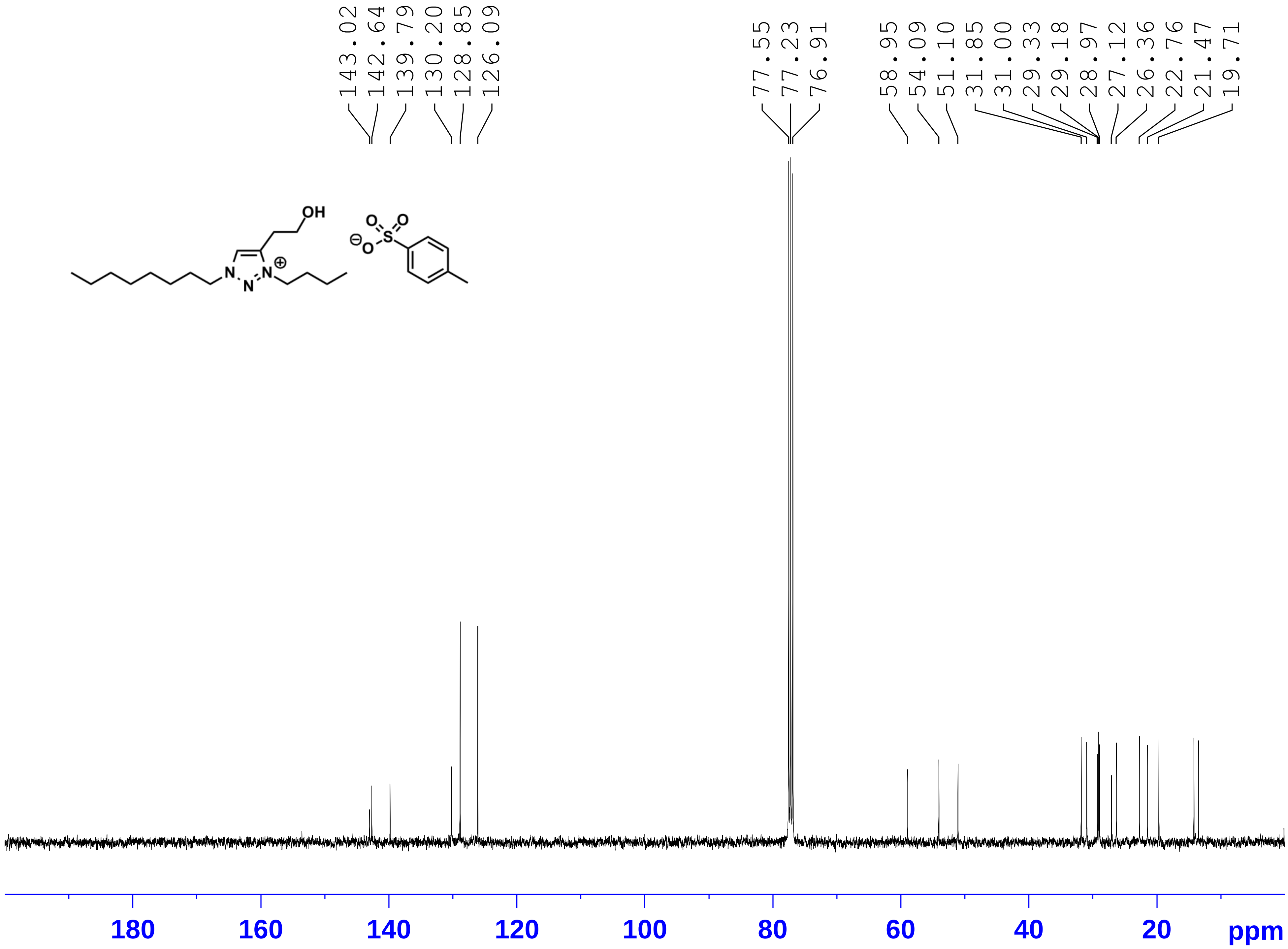

143.02
142.64
139.79
130.20
128.85
126.09
77.55
77.23
76.91
58.95
54.09
51.10
31.85
31.00
29.33
29.18
28.97
27.12
26.36
22.76
21.47
19.71
OH
O
S
O
O
N
N
N
180
160
140
120
100
80
60
40
20
ppm

Spectrum

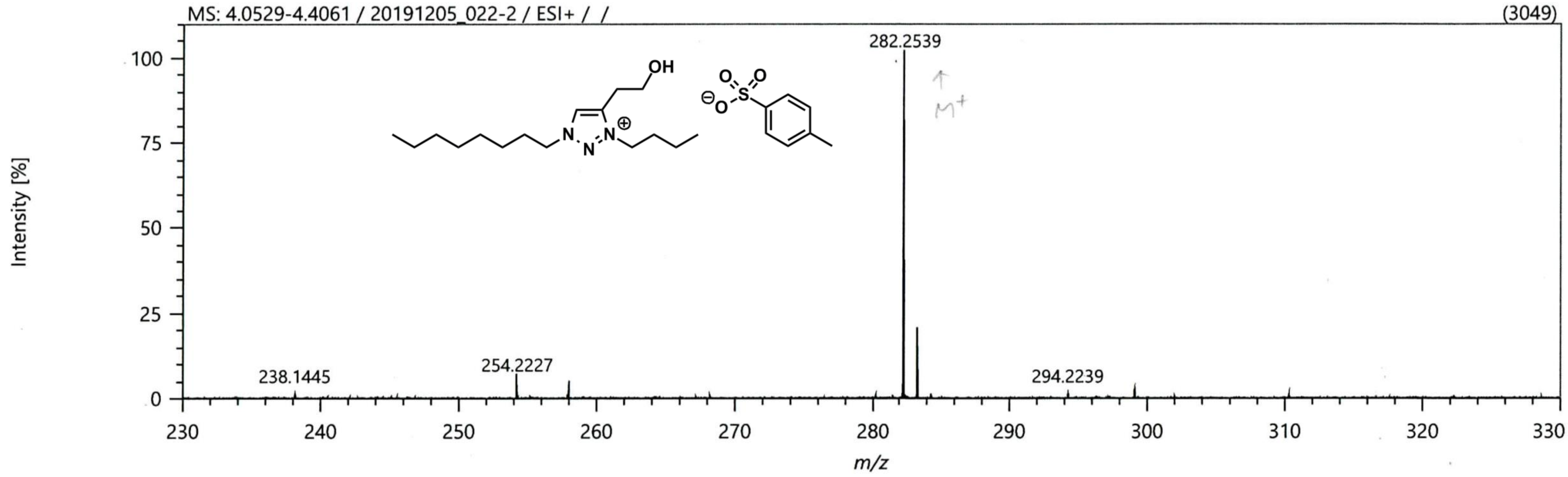

Elemental Composition

Parameters	
Tolerance:	±50.00 ppm
Electron:	Odd/Even
Charge:	+1
DBE:	-1.5 - 999.0

Elements Set 1:

Symbol	C	H	O	Na	N	S	P	Si	F	Cl
Min	0	0	1	0	3	0	0	0	0	0
Max	400	1000	1	0	3	0	0	0	0	0

Results

Mass	Formula	Calculated Mass	Mass Difference [mDa]	Mass Difference [ppm]	DBE
282.25391	C16 H32 N3 O	282.25399	-0.07	-0.26	2.5

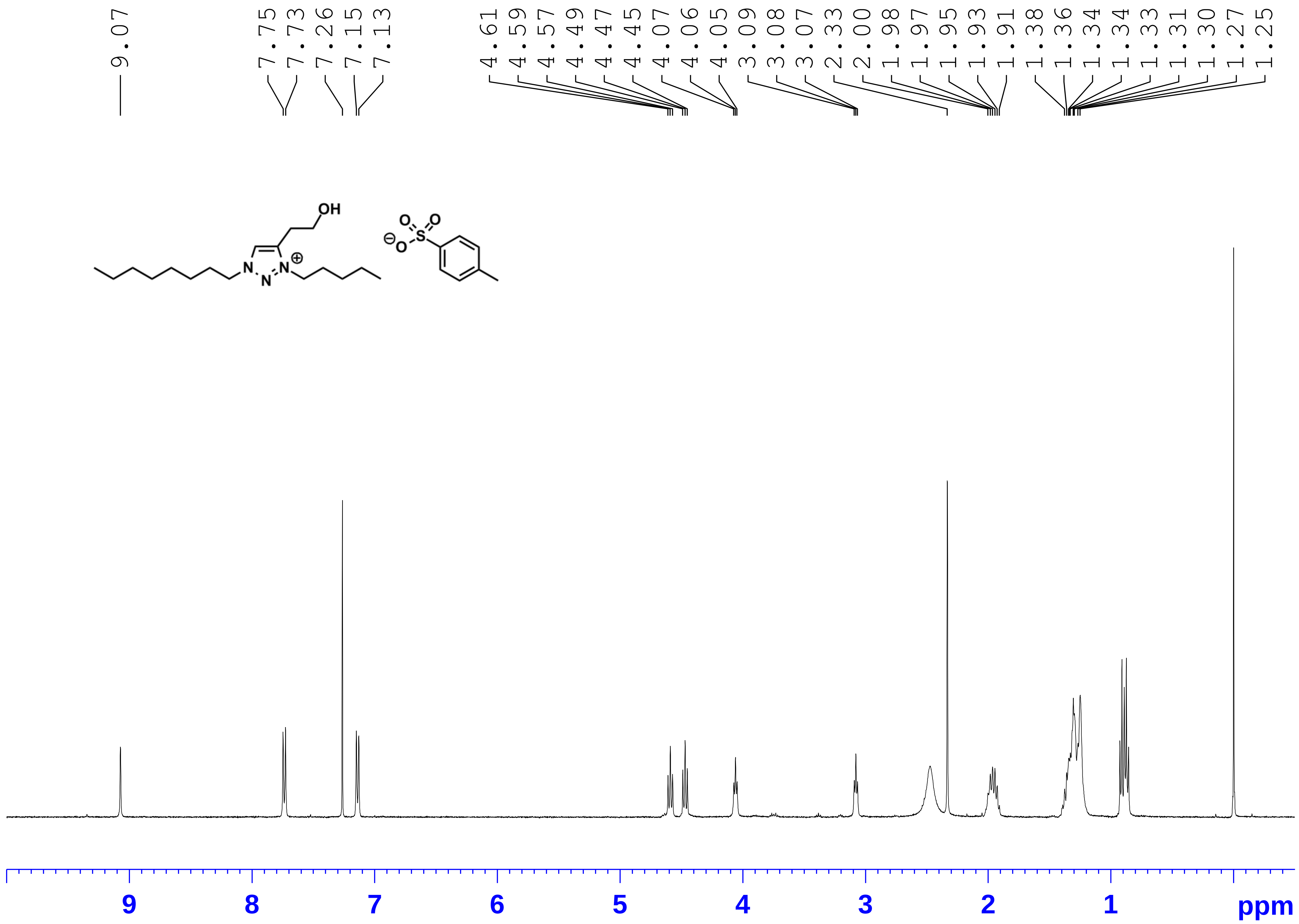
9.07
7.75
7.73
7.26
7.15
7.13
4.61
4.59
4.57
4.49
4.47
4.45
4.07
4.06
4.05
3.09
3.08
3.07
2.33
2.00
1.98
1.97
1.95
1.93
1.91
1.38
1.36
1.34
1.34
1.33
1.31
1.30
1.27
1.25
OH
O
O
S
O
N
N
N
9
8
7
6
5
4
3
2
1
ppm

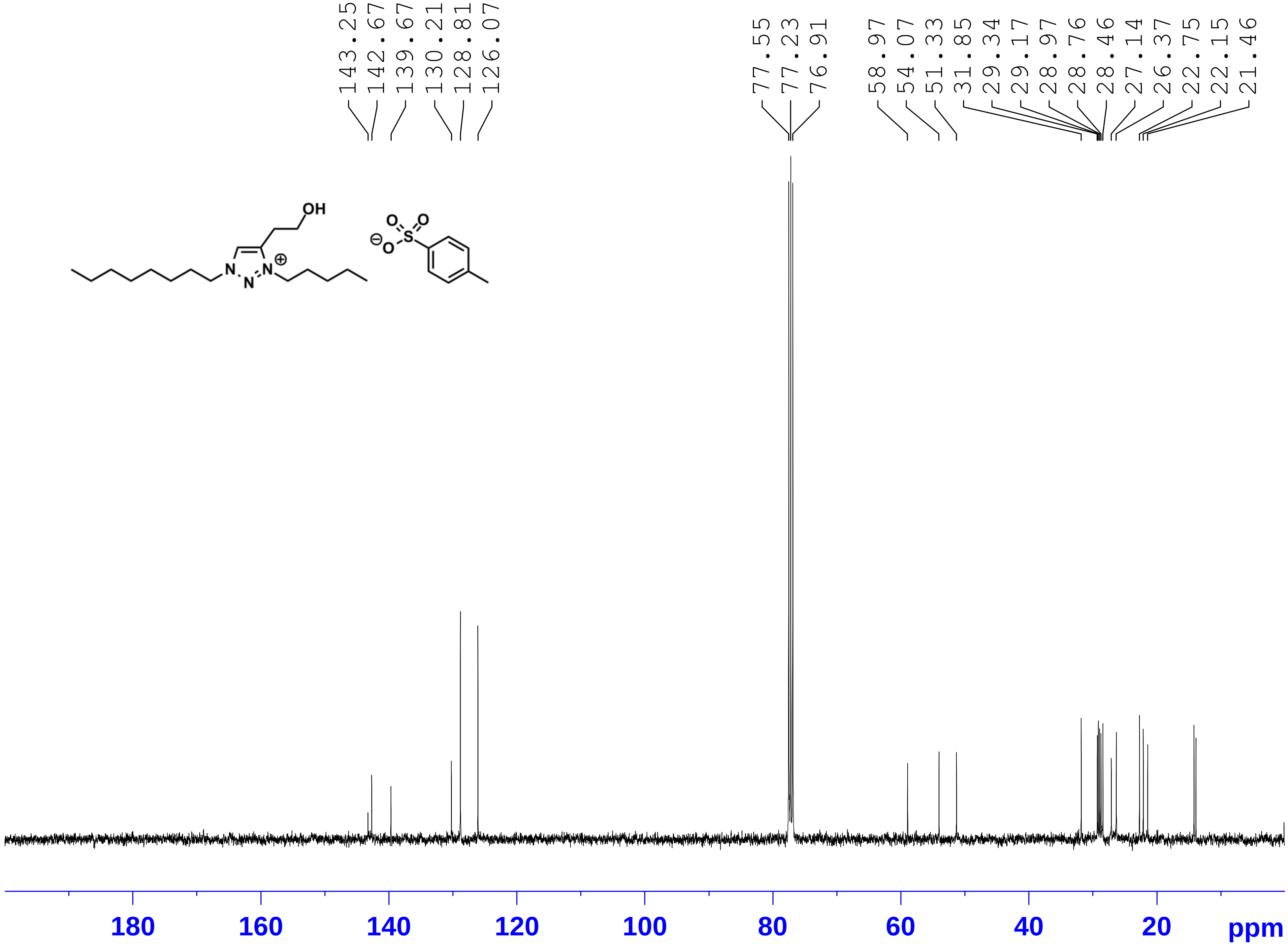

143.25
142.67
139.67
130.21
128.81
126.07
77.55
77.23
76.91
58.97
54.07
51.33
31.85
29.34
29.17
28.97
28.76
28.46
27.14
26.37
22.75
22.15
21.46
OH
N
N
N
O
S
O
O
180
160
140
120
100
80
60
40
20
ppm

Spectrum

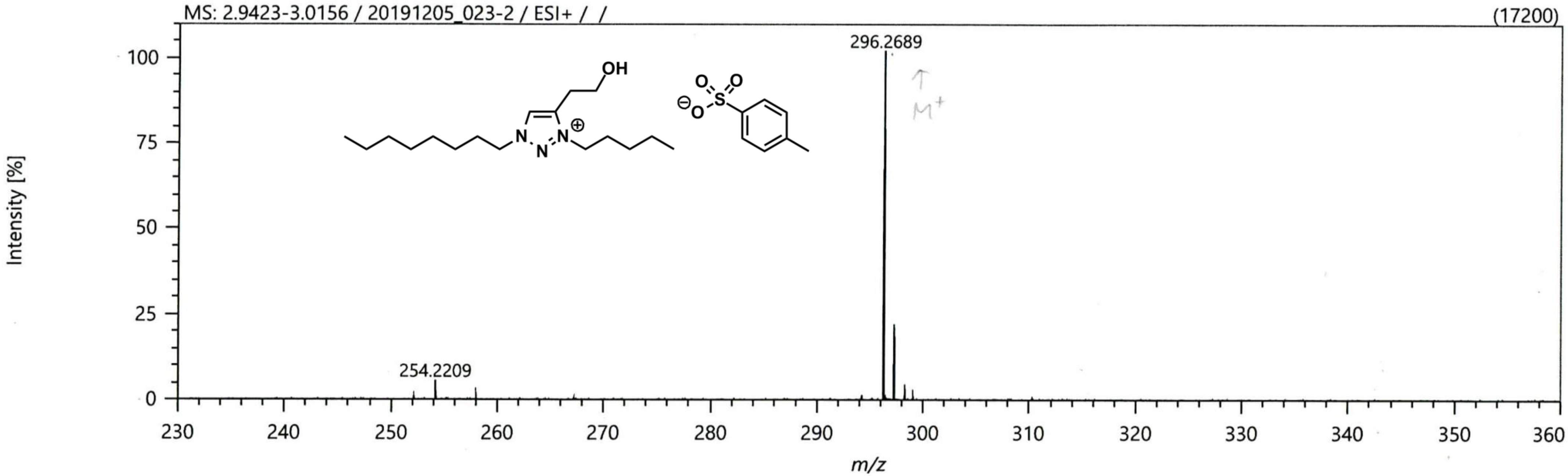

Elemental Composition

Parameters

Tolerance:	±50.00 ppm
Electron:	Odd/Even
Charge:	+1
DBE:	-1.5 - 999.0

Elements Set 1:

Symbol	C	H	O	Na	N	S	P	Si	F	Cl
Min	0	0	1	0	3	0	0	0	0	0
Max	400	1000	1	0	3	0	0	0	0	0

Results

Mass	Formula	Calculated Mass	Mass Difference [mDa]	Mass Difference [ppm]	DBE
296.26889	C17 H34 N3 O	296.26964	-0.75	-2.54	2.5

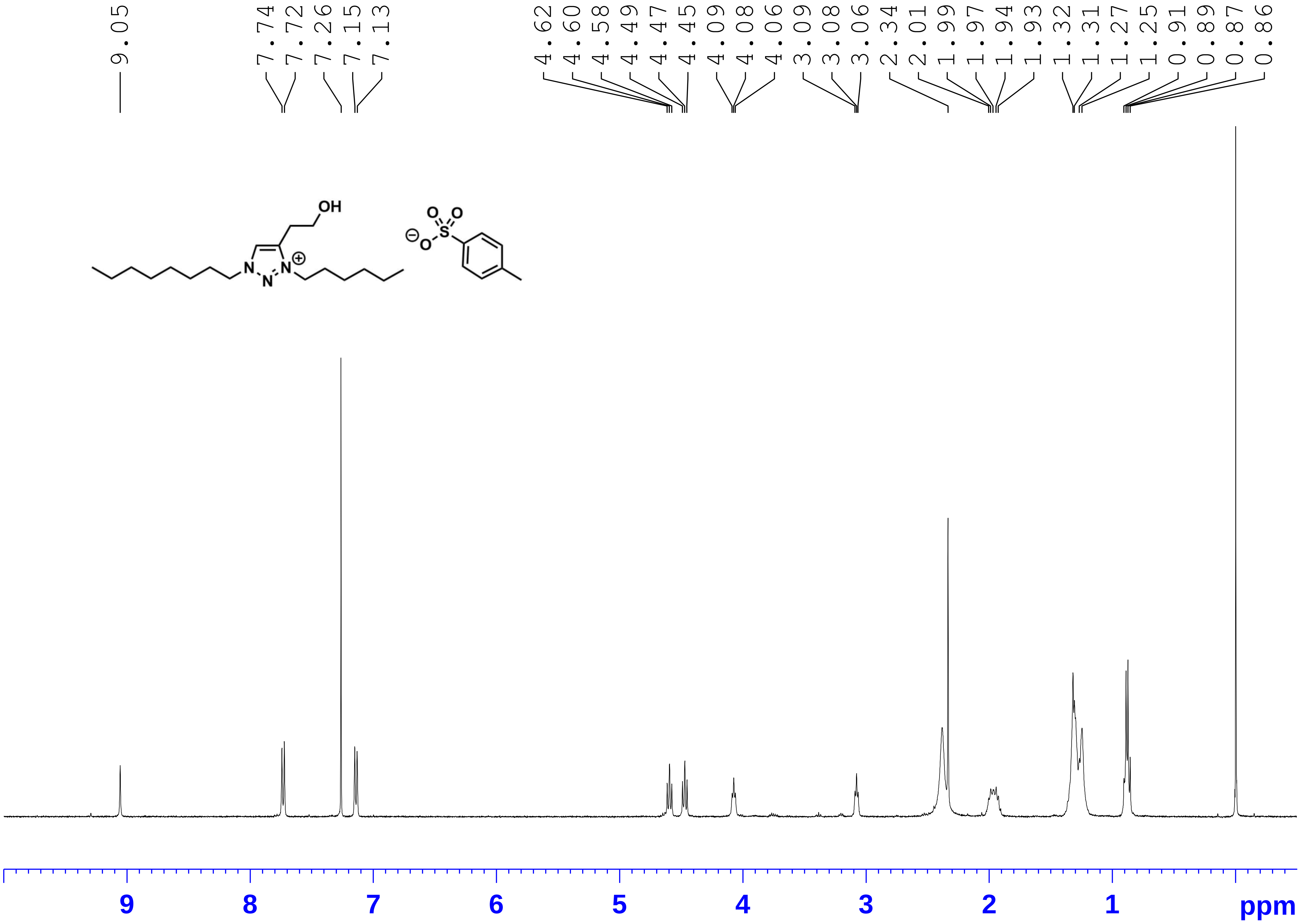
9.05
7.74
7.72
7.26
7.15
7.13
4.62
4.60
4.58
4.49
4.47
4.45
4.09
4.08
4.06
3.09
3.08
3.06
2.34
2.01
1.99
1.97
1.94
1.93
1.32
1.31
1.27
1.25
0.91
0.89
0.87
0.86
OH
N
N
N
O
S
O
O
9
8
7
6
5
4
3
2
1
ppm

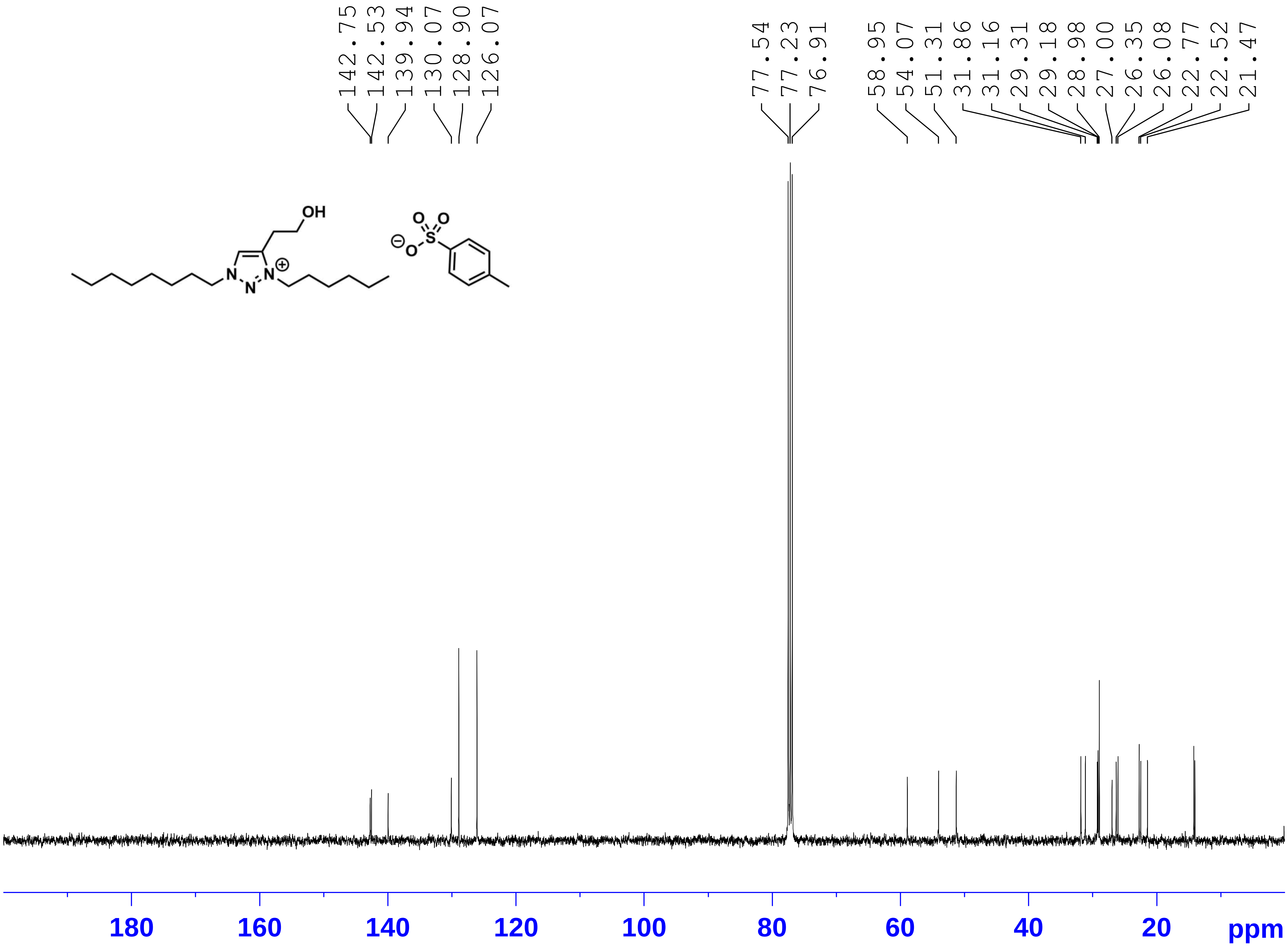

142.75
142.53
139.94
130.07
128.90
126.07
77.54
77.23
76.91
58.95
54.07
51.31
31.86
31.16
29.31
29.18
28.98
27.00
26.35
26.08
22.77
22.52
21.47
OH
O
O
S
O
N
N
N
180
160
140
120
100
80
60
40
20
ppm

Spectrum

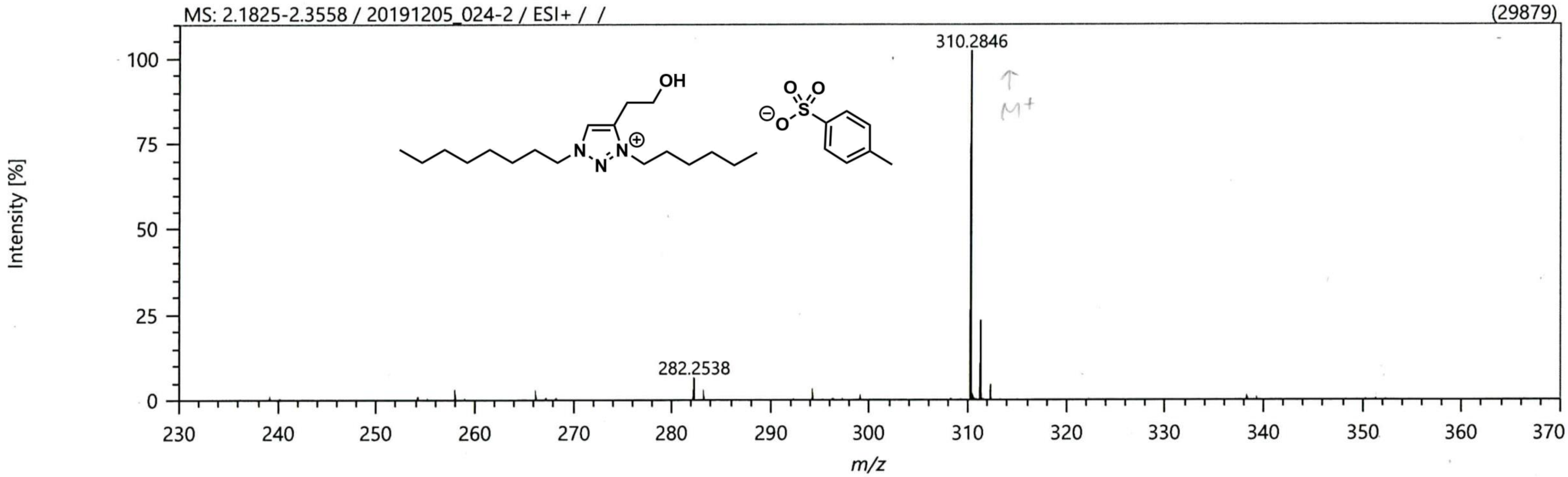

Elemental Composition

Parameters	
Tolerance:	±50.00 ppm
Electron:	Odd/Even
Charge:	+1
DBE:	-1.5 - 999.0

Elements Set 1:

Symbol	C	H	O	Na	N	S	P	Si	F	Cl
Min	0	0	1	0	3	0	0	0	0	0
Max	400	1000	1	0	3	0	0	0	0	0

Results

Mass	Formula	Calculated Mass	Mass Difference [mDa]	Mass Difference [ppm]	DBE
310.28461	C18 H36 N3 O	310.28529	-0.68	-2.18	2.5

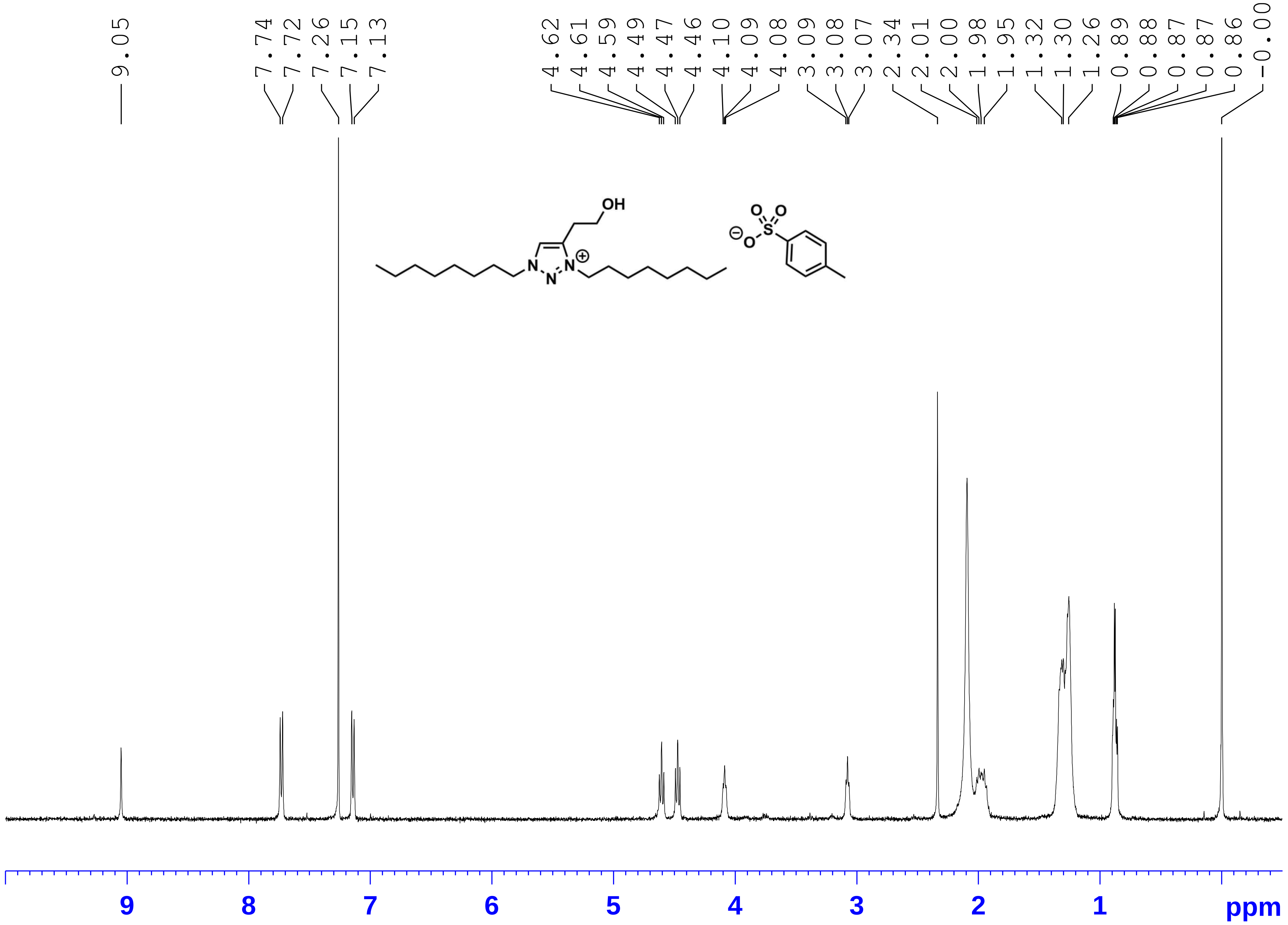

9.05
7.74
7.72
7.26
7.15
7.13
4.62
4.61
4.59
4.49
4.47
4.46
4.10
4.09
4.08
3.09
3.08
3.07
2.34
2.01
2.00
1.98
1.95
1.32
1.30
1.26
0.89
0.88
0.87
0.87
0.86
-0.00
OH
N
N
N
O
O
S
O
9
8
7
6
5
4
3
2
1
ppm

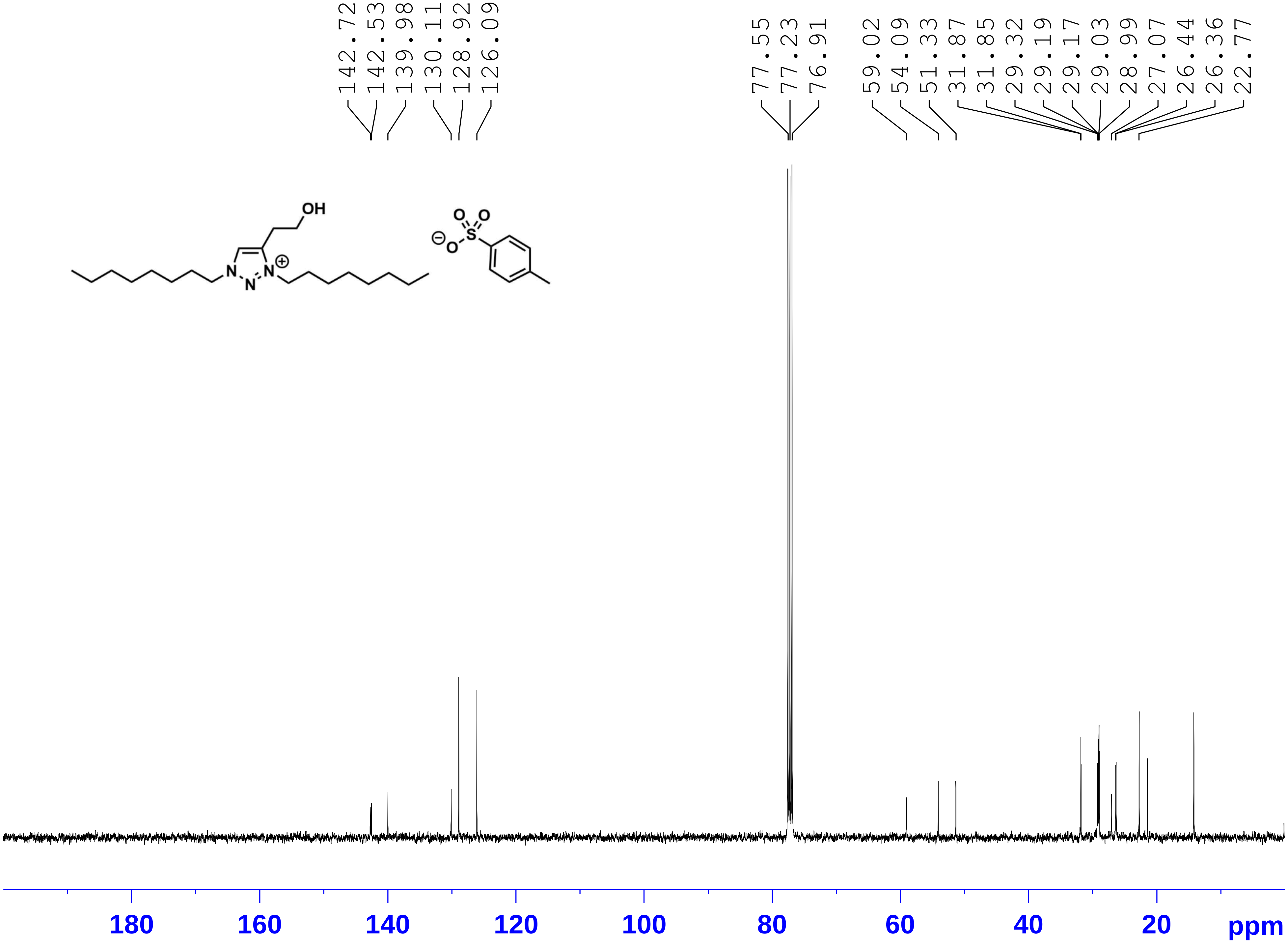

142.72
142.53
139.98
130.11
128.92
126.09
77.55
77.23
76.91
59.02
54.09
51.33
31.87
31.85
29.32
29.19
29.17
29.03
28.99
27.07
26.44
26.36
22.77
OH
N
N
N
O
O
S
O
180
160
140
120
100
80
60
40
20
ppm

Spectrum

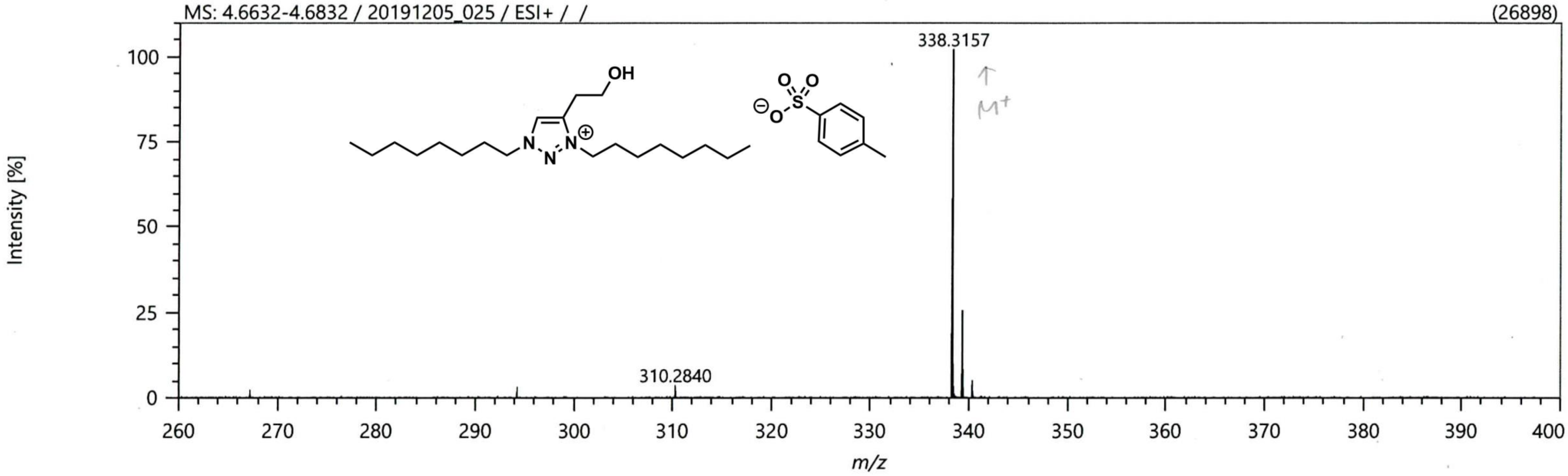

Elemental Composition

Parameters		Elements Set 1:										
Tolerance:	±50.00 ppm	Symbol	C	H	O	Na	N	S	P	Si	F	Cl
Electron:	Odd/Even	Min	0	0	1	0	3	0	0	0	0	0
Charge:	+1	Max	400	1000	1	0	3	0	0	0	0	0
DBE:	-1.5 - 999.0											

Results

Mass	Formula	Calculated Mass	Mass Difference [mDa]	Mass Difference [ppm]	DBE
338.31569	C20 H40 N3 O	338.31659	-0.90	-2.66	2.5

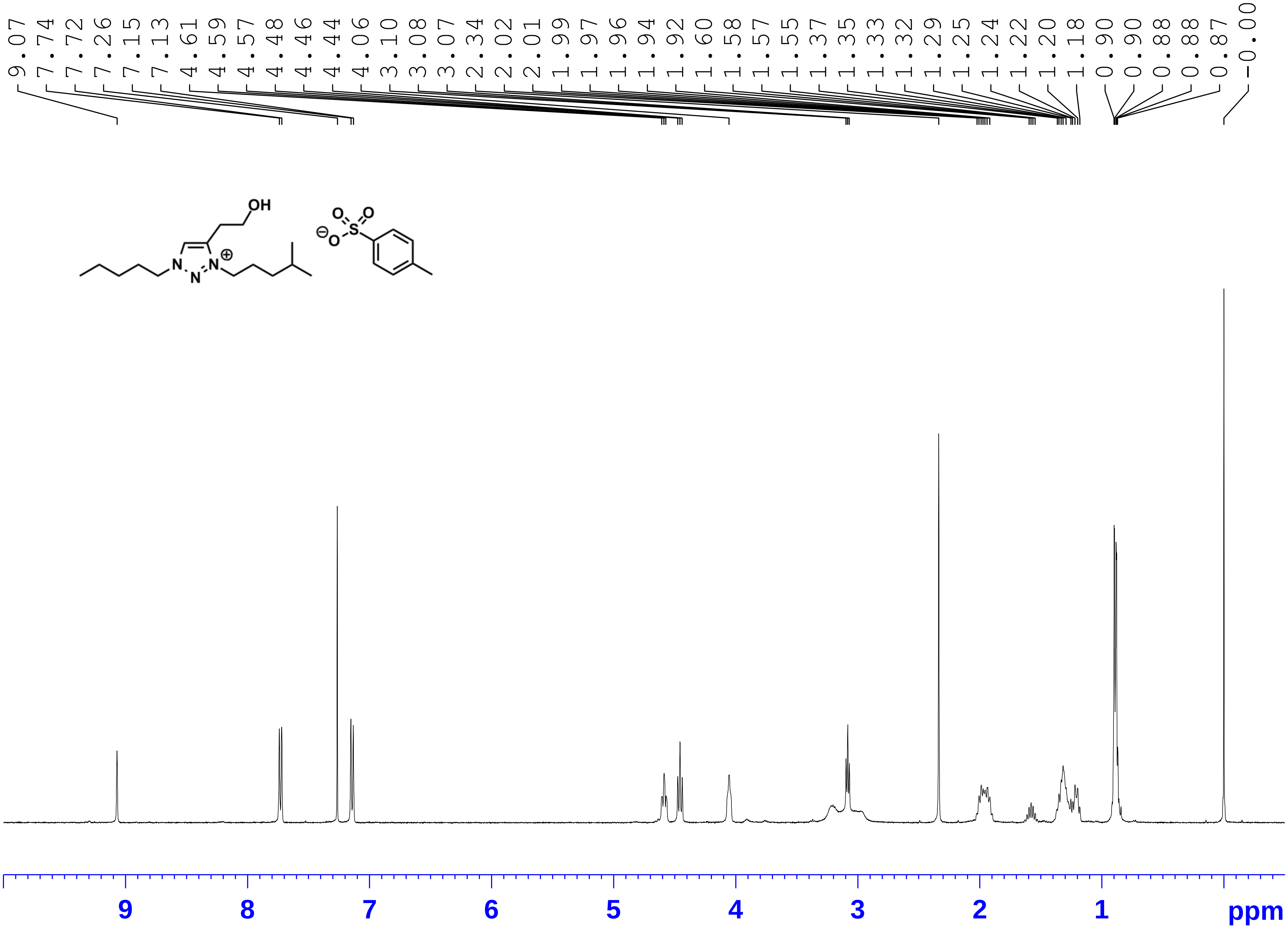
9.07
7.74
7.72
7.26
7.15
7.13
4.61
4.59
4.57
4.48
4.46
4.44
4.06
3.10
3.08
3.07
2.34
2.02
2.01
1.99
1.97
1.96
1.94
1.92
1.60
1.58
1.57
1.55
1.37
1.35
1.33
1.32
1.29
1.25
1.24
1.22
1.20
1.18
0.90
0.90
0.88
0.88
0.87
-0.00
OH
O
O
S
O
N
N
N
9
8
7
6
5
4
3
2
1
ppm

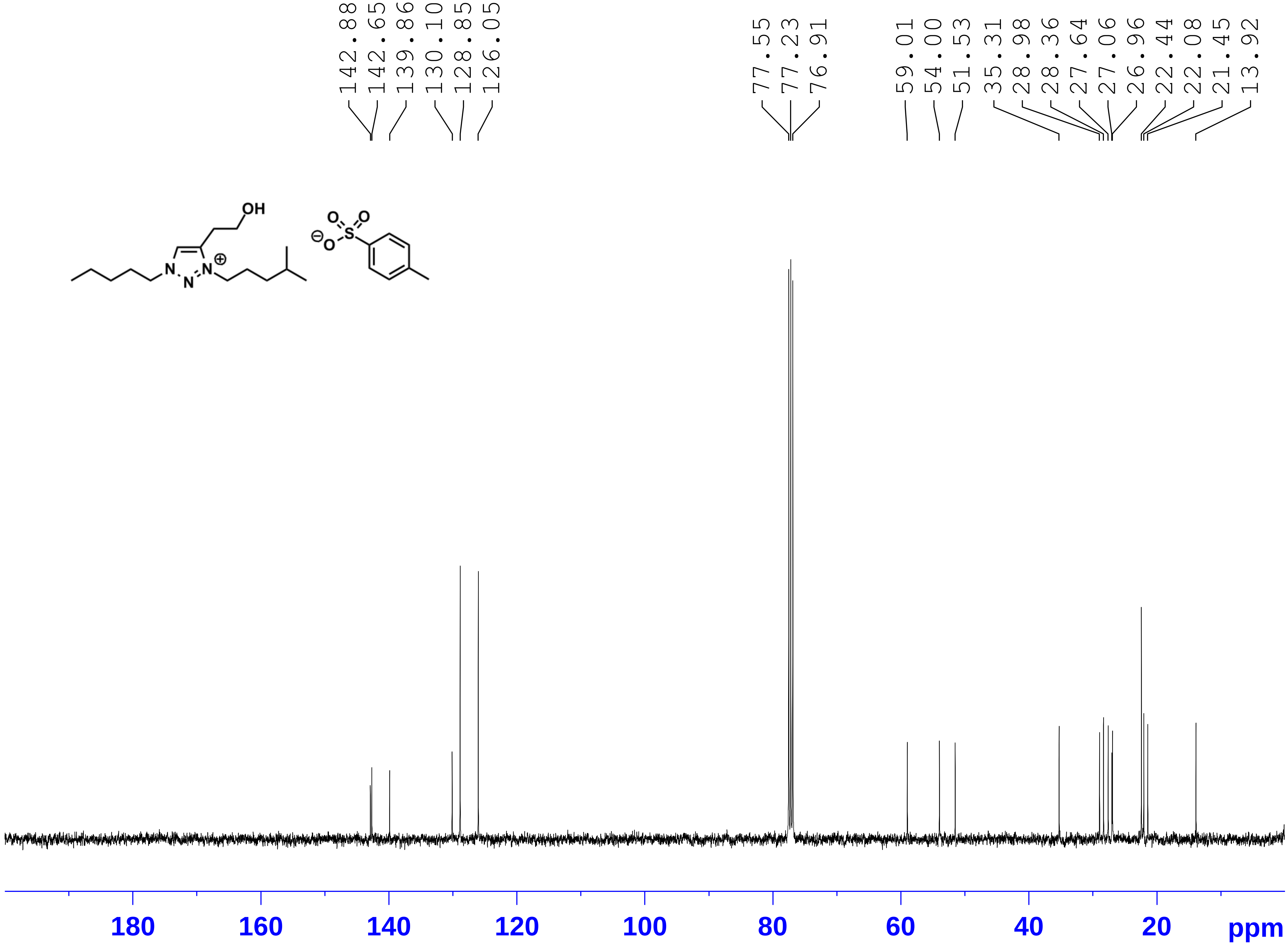
142.88
142.65
139.86
130.10
128.85
126.05
77.55
77.23
76.91
59.01
54.00
51.53
35.31
28.98
28.36
27.64
27.06
26.96
22.44
22.08
21.45
13.92
OH
O
O
S
O
N
N
N
180
160
140
120
100
80
60
40
20
ppm

Spectrum

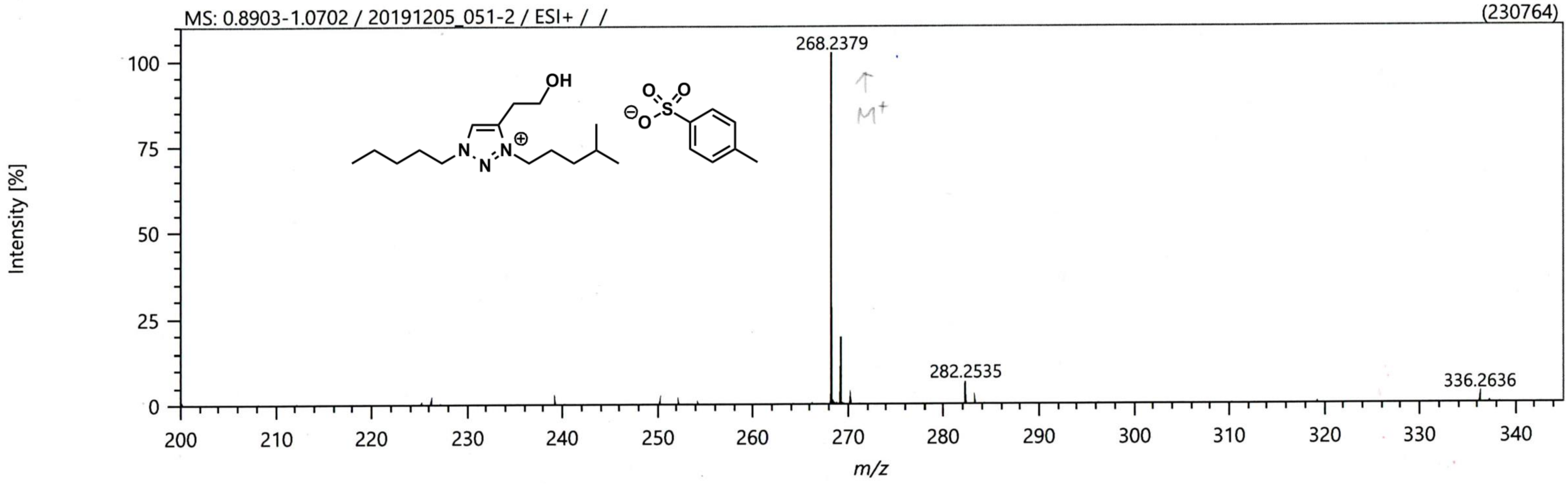

Elemental Composition

Parameters		Elements Set 1:										
Tolerance:	±50.00 ppm	Symbol	C	H	O	Na	N	S	P	Si	F	Cl
Electron:	Odd/Even	Min	0	0	1	0	3	0	0	0	0	0
Charge:	+1	Max	400	1000	1	0	3	0	0	0	0	0
DBE:	-1.5 - 999.0											

Results

Mass	Formula	Calculated Mass	Mass Difference [mDa]	Mass Difference [ppm]	DBE
268.23793	C15 H30 N3 O	268.23834	-0.41	-1.52	2.5

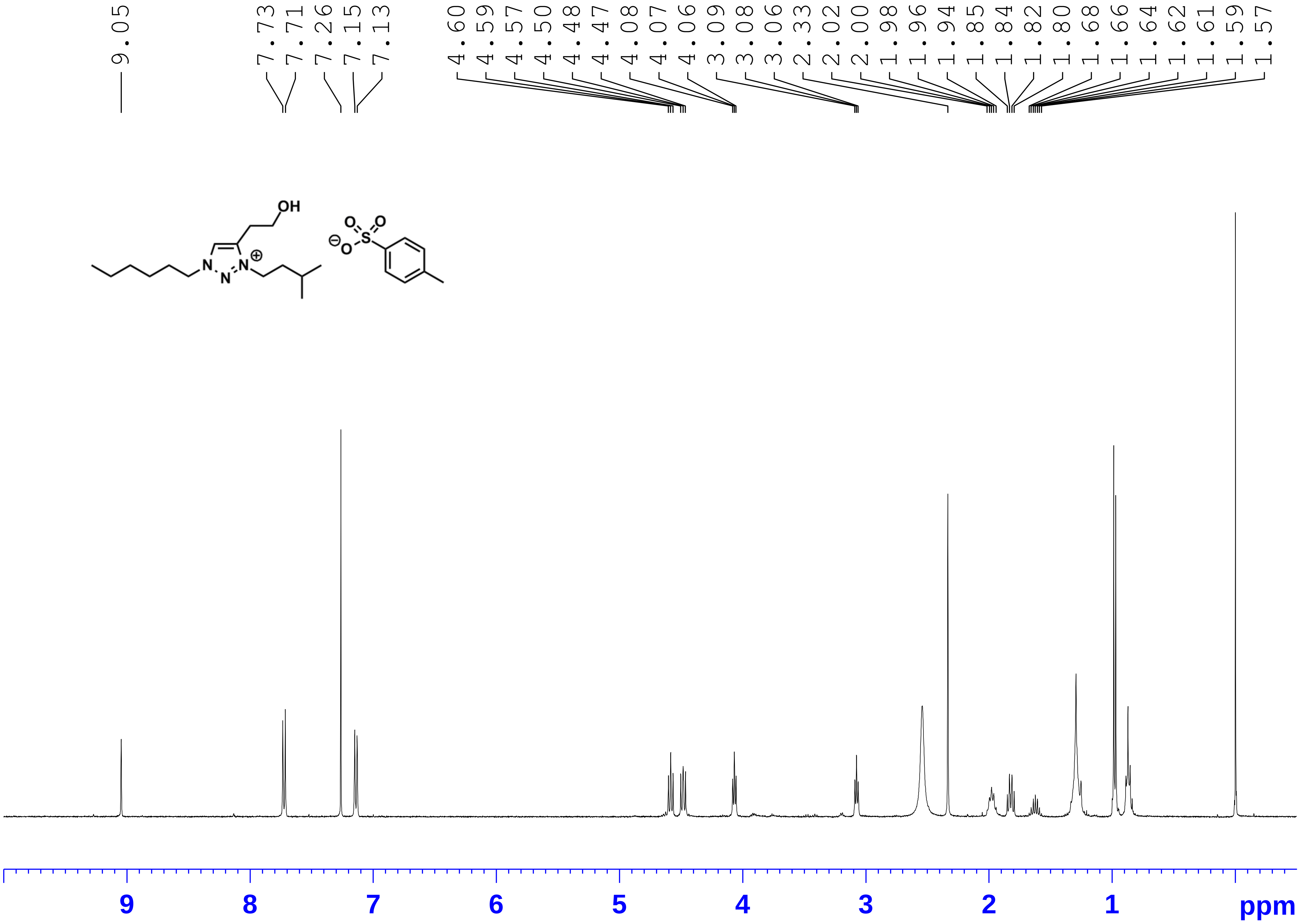
9.05
7.73
7.71
7.26
7.15
7.13
4.60
4.59
4.57
4.50
4.48
4.47
4.08
4.07
4.06
3.09
3.08
3.06
2.33
2.02
2.00
1.98
1.96
1.94
1.85
1.84
1.82
1.80
1.68
1.66
1.64
1.62
1.61
1.59
1.57
OH
N
N
N
O
O
S
O
9
8
7
6
5
4
3
2
1
ppm

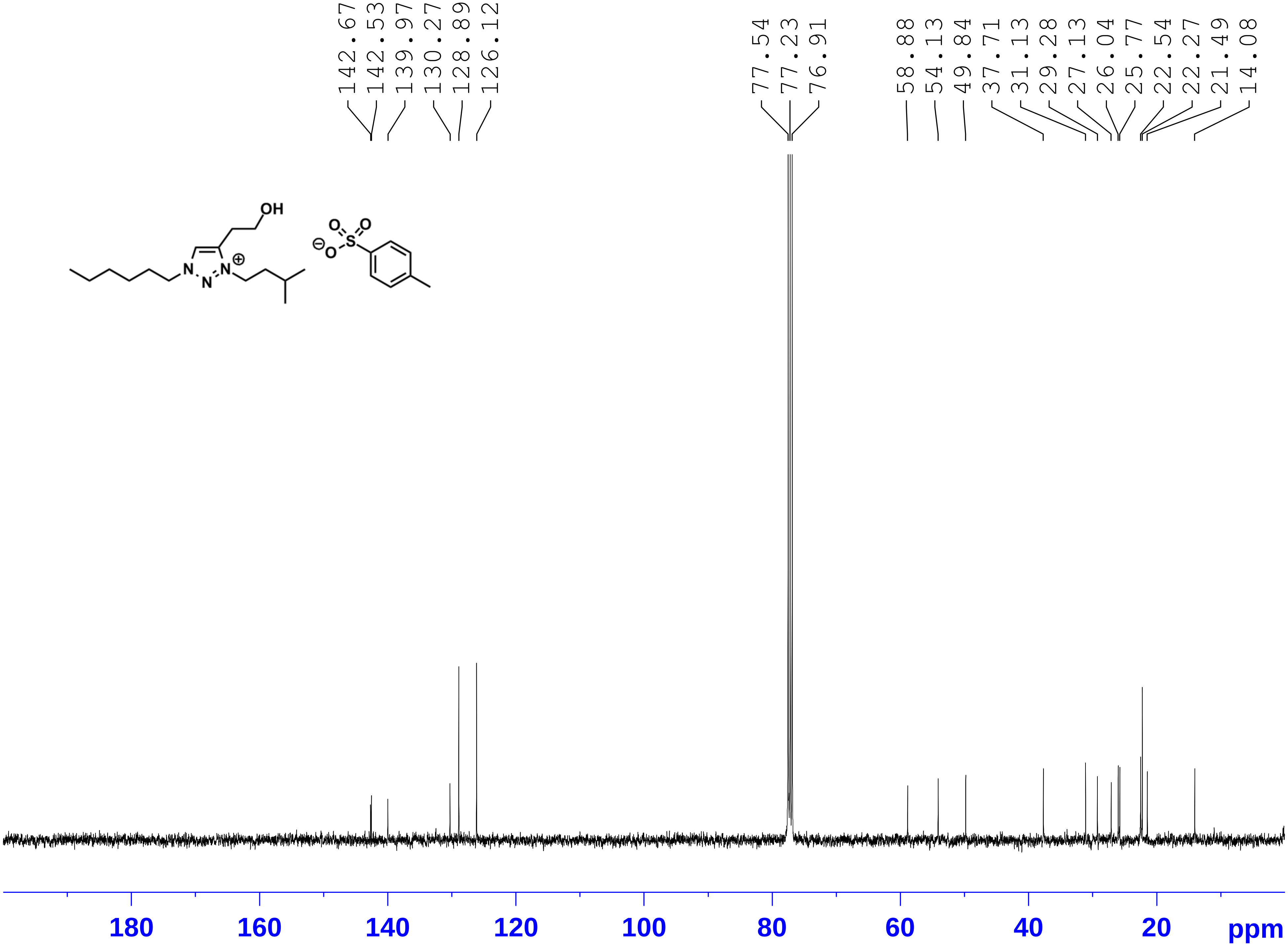
142.67
142.53
139.97
130.27
128.89
126.12
77.54
77.23
76.91
58.88
54.13
49.84
37.71
31.13
29.28
27.13
26.04
25.77
22.54
22.27
21.49
14.08
OH
N
N
N
O
S
O
O
180
160
140
120
100
80
60
40
20
ppm

Spectrum

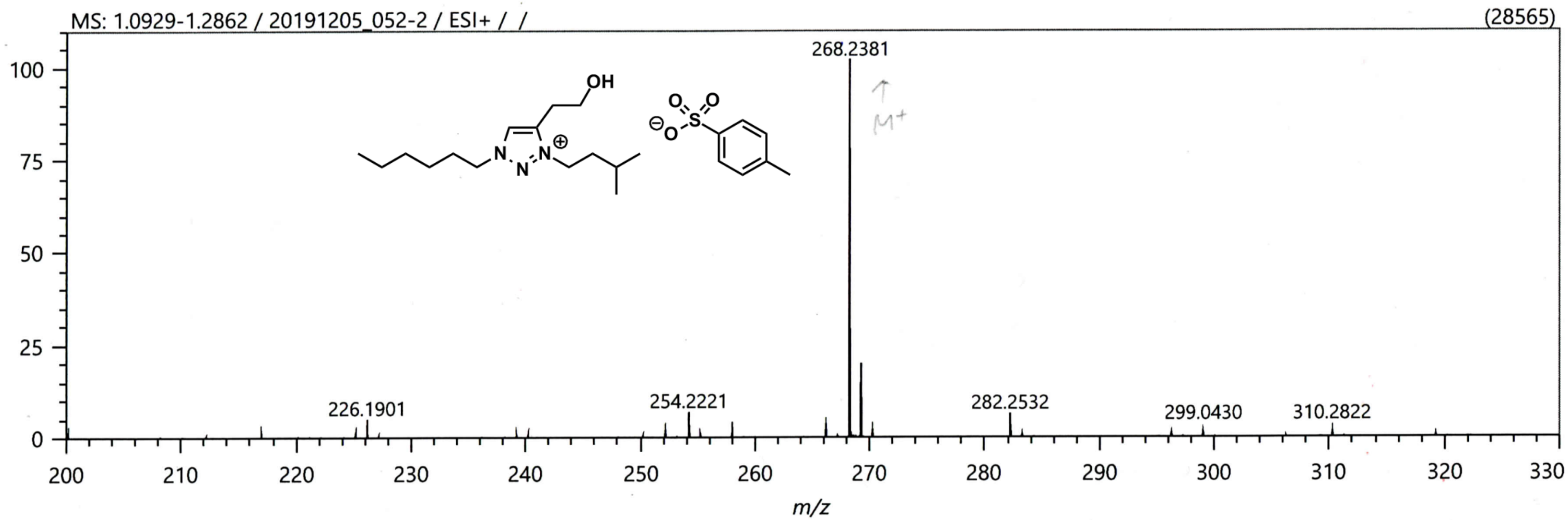

Elemental Composition

Parameters

Tolerance:	±50.00 ppm
Electron:	Odd/Even
Charge:	+1
DBE:	-1.5 - 999.0

Elements Set 1:

Symbol	C	H	O	Na	N	S	P	Si	F	Cl
Min	0	0	1	0	3	0	0	0	0	0
Max	400	1000	1	0	3	0	0	0	0	0

Results

Mass	Formula	Calculated Mass	Mass Difference [mDa]	Mass Difference [ppm]	DBE
268.23806	C15 H30 N3 O	268.23834	-0.28	-1.04	2.5

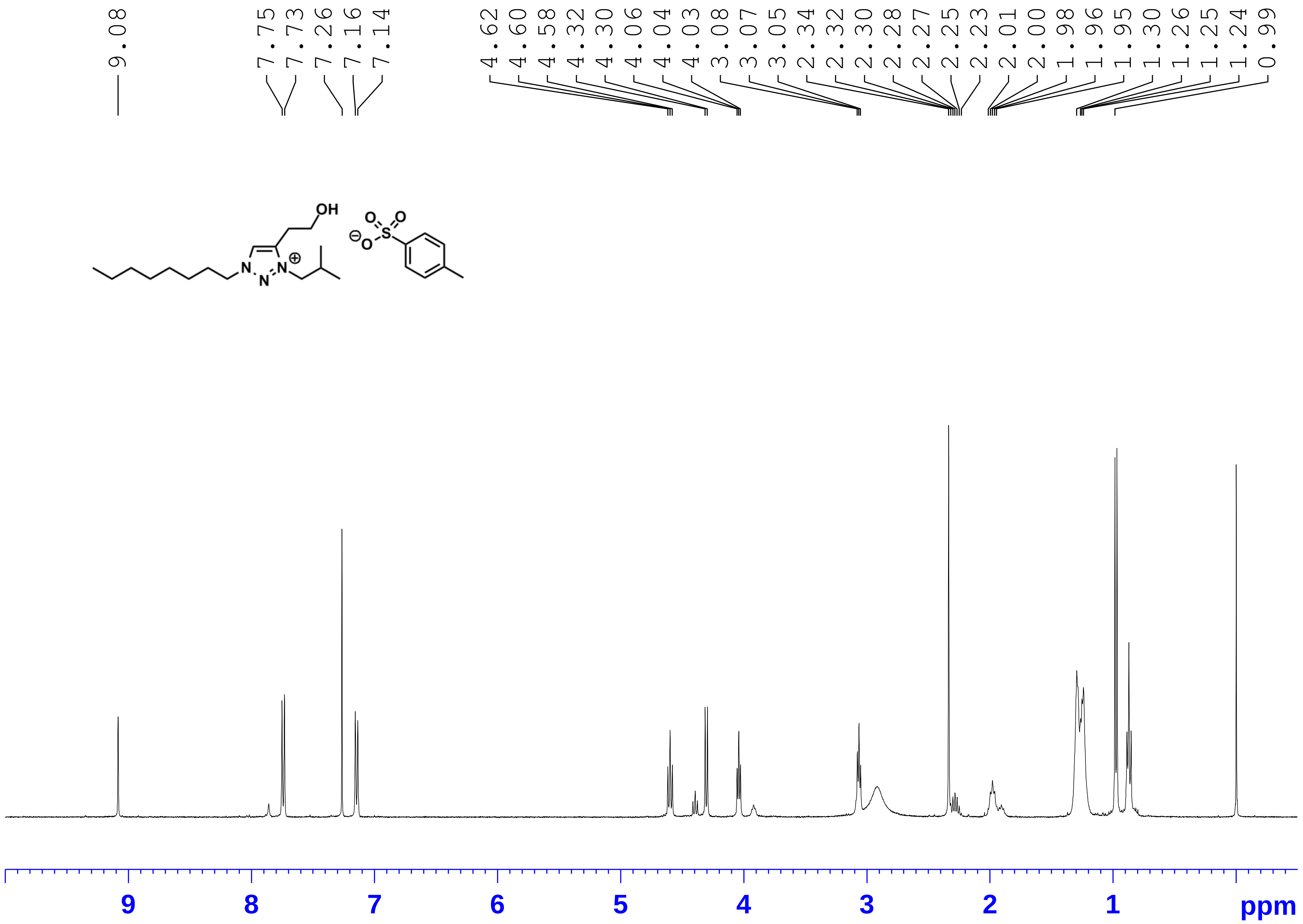

9.08
7.75
7.73
7.26
7.16
7.14
4.62
4.60
4.58
4.32
4.30
4.06
4.04
4.03
3.08
3.07
3.05
2.34
2.32
2.30
2.28
2.27
2.25
2.23
2.01
2.00
1.98
1.96
1.95
1.30
1.26
1.25
1.24
0.99
OH
N
N
N
O
O
S
O
9
8
7
6
5
4
3
2
1
ppm

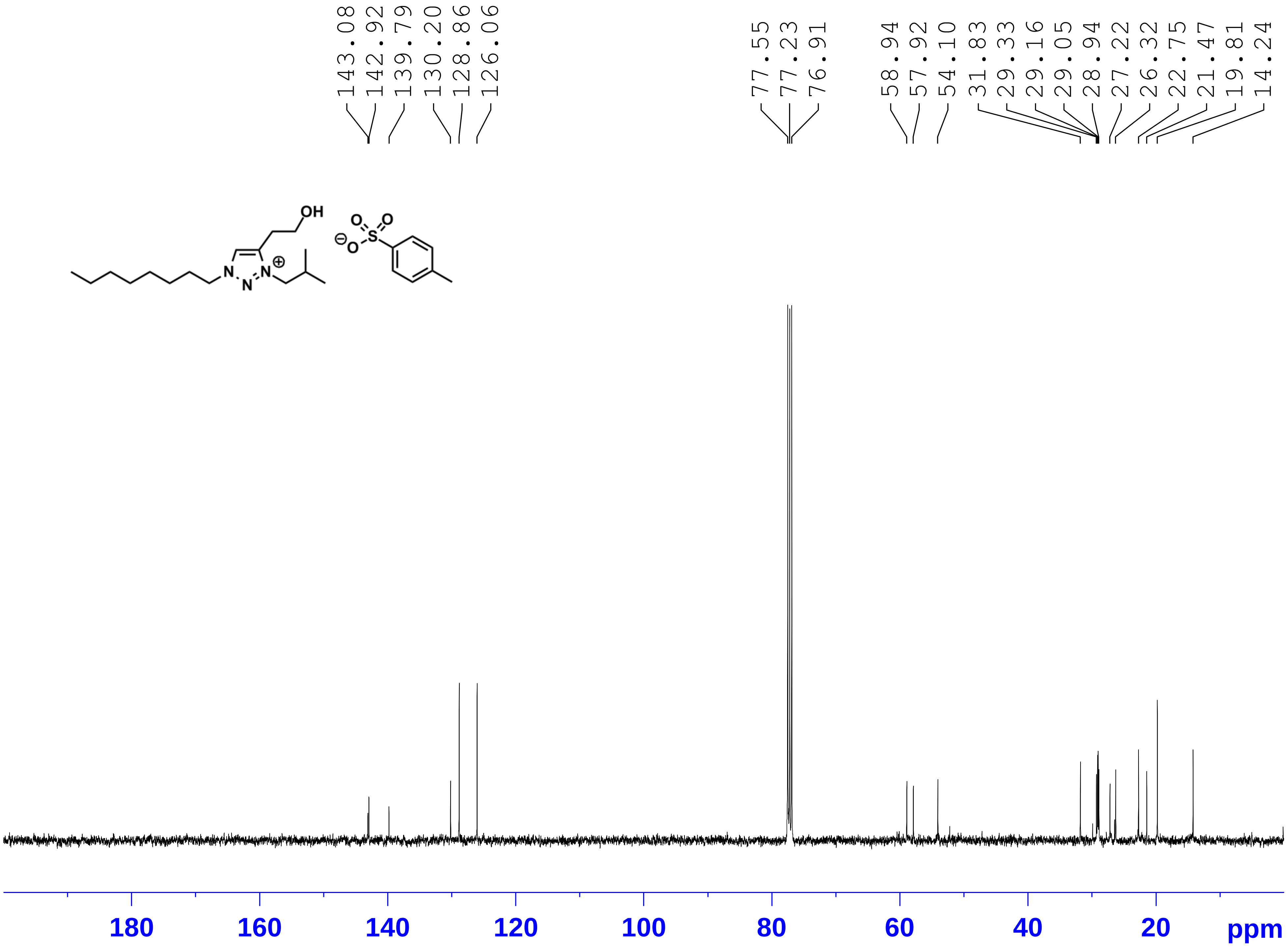

143.08
142.92
139.79
130.20
128.86
126.06
77.55
77.23
76.91
58.94
57.92
54.10
31.83
29.33
29.16
29.05
28.94
27.22
26.32
22.75
21.47
19.81
14.24
OH
O
S
O
O
N
N
N
180
160
140
120
100
80
60
40
20
ppm

Spectrum

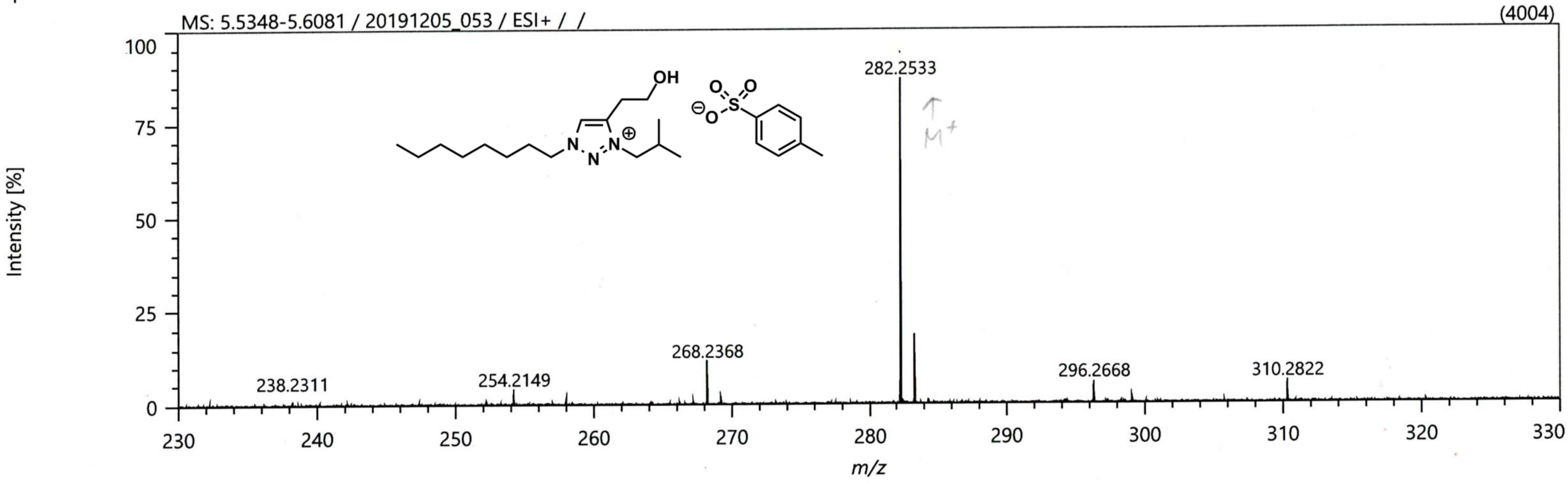

Elemental Composition

Parameters	
Tolerance:	±50.00 ppm
Electron:	Odd/Even
Charge:	+1
DBE:	-1.5 - 999.0

Elements Set 1:

Symbol	C	H	O	Na	N	S	P	Si	F	Cl
Min	0	0	1	0	3	0	0	0	0	0
Max	400	1000	1	0	3	0	0	0	0	0

Results

Mass	Formula	Calculated Mass	Mass Difference [mDa]	Mass Difference [ppm]	DBE
282.25325	C16 H32 N3 O	282.25399	-0.74	-2.61	2.5

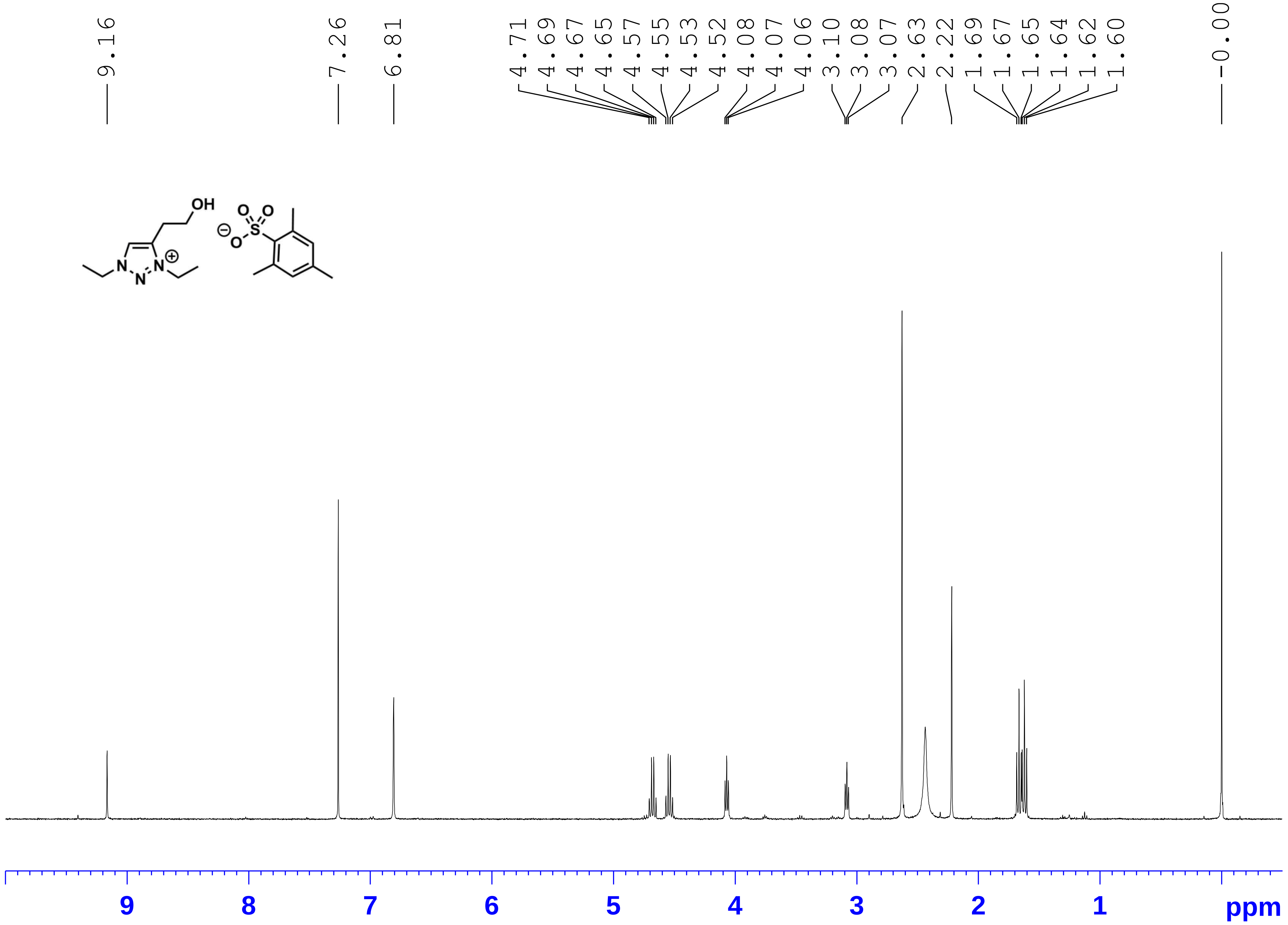
9.16
7.26
6.81
4.71
4.69
4.67
4.65
4.57
4.55
4.53
4.52
4.08
4.07
4.06
3.10
3.08
3.07
2.63
2.22
1.69
1.67
1.65
1.64
1.62
1.60
0.00
OH
N
N
N
O
O
S
O
9
8
7
6
5
4
3
2
1
ppm

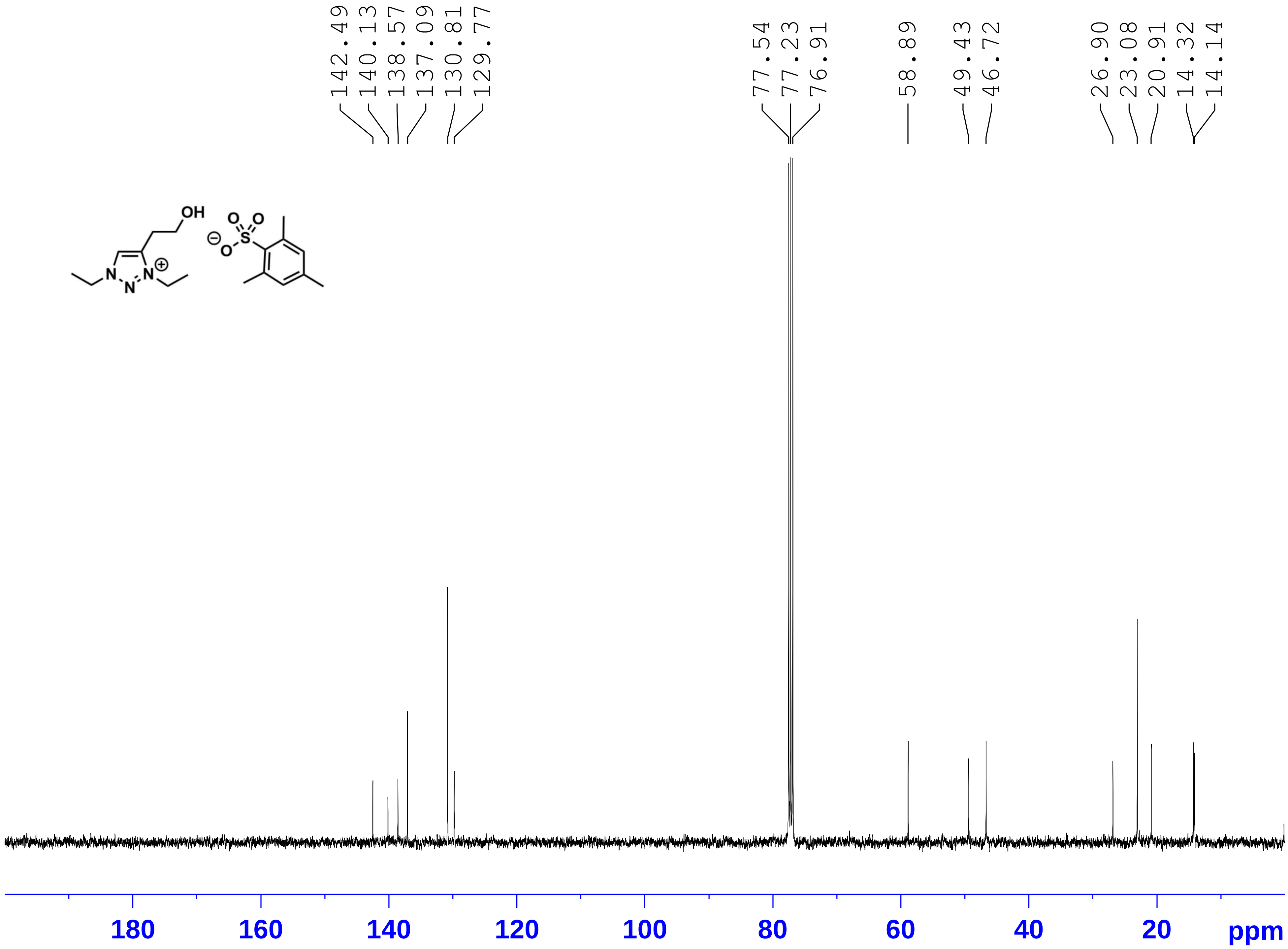

142.49
140.13
138.57
137.09
130.81
129.77
77.54
77.23
76.91
58.89
49.43
46.72
26.90
23.08
20.91
14.32
14.14
OH
O
O
S
O
N
N
N
180
160
140
120
100
80
60
40
20
ppm

Spectrum

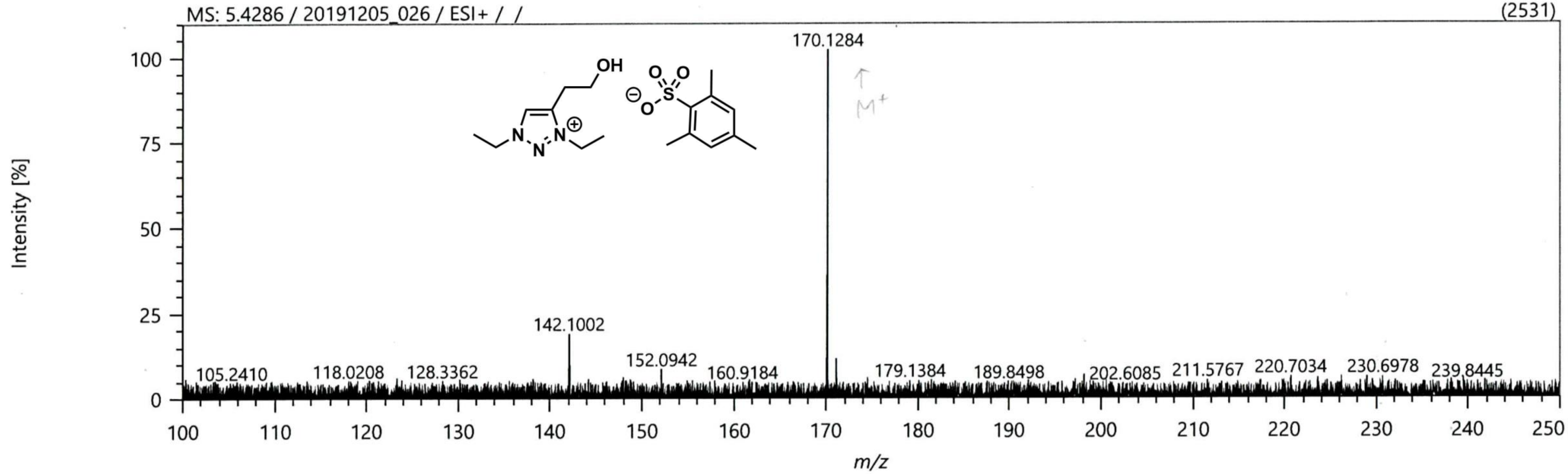

Elemental Composition

Parameters	
Tolerance:	±50.00 ppm
Electron:	Odd/Even
Charge:	+1
DBE:	-1.5 - 999.0

Elements Set 1:

Symbol	C	H	O	Na	N	S	P	Si	F	Cl
Min	0	0	1	0	3	0	0	0	0	0
Max	400	1000	1	0	3	0	0	0	0	0

Results

Mass	Formula	Calculated Mass	Mass Difference [mDa]	Mass Difference [ppm]	DBE
170.12837	C8 H16 N3 O	170.12879	-0.42	-2.49	2.5

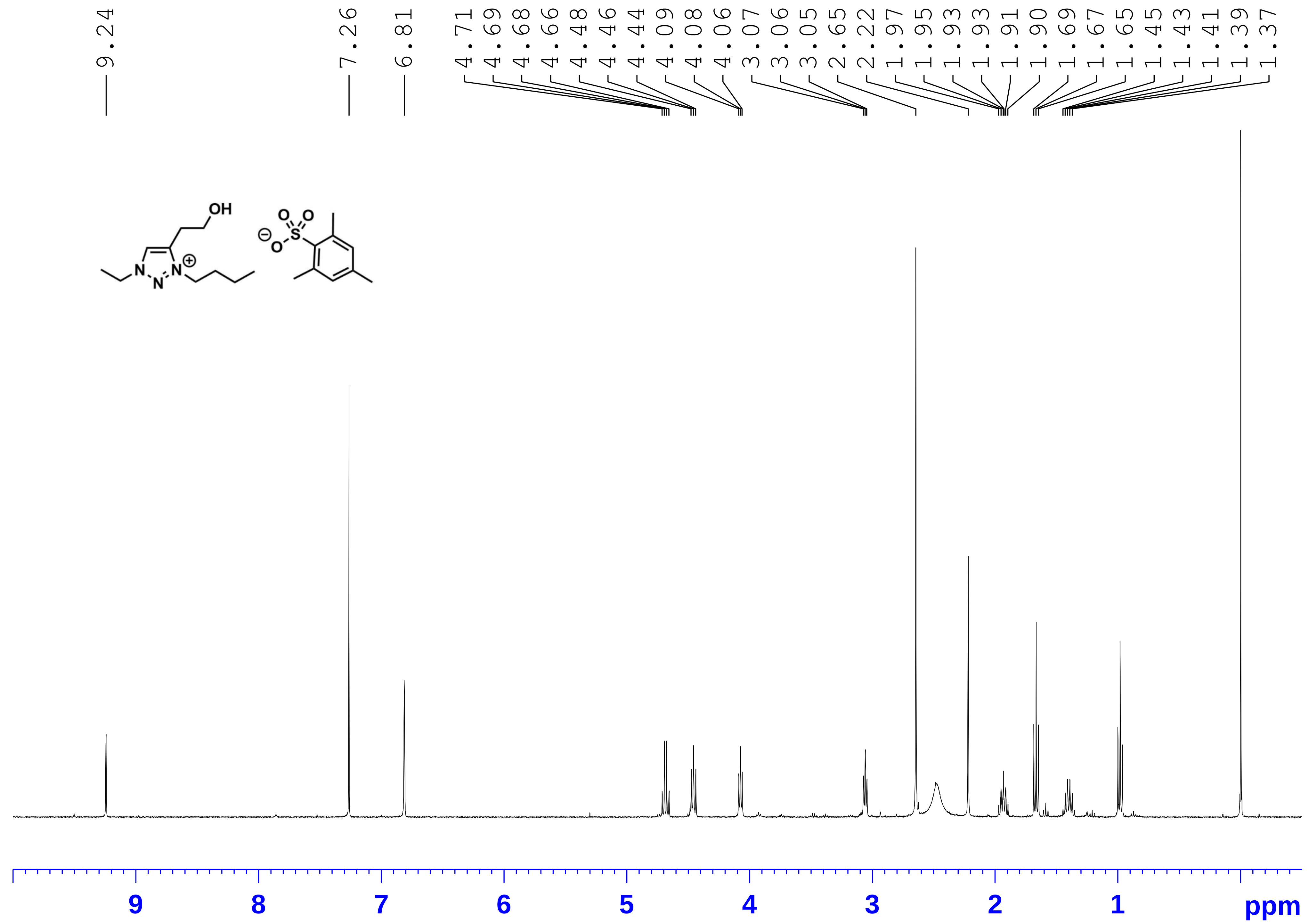
9.24
7.26
6.81
4.71
4.69
4.68
4.66
4.48
4.46
4.44
4.09
4.08
4.06
3.07
3.06
3.05
2.65
2.22
1.97
1.95
1.93
1.93
1.91
1.90
1.69
1.67
1.65
1.45
1.43
1.41
1.39
1.37
OH
O
S
O
N
N
N
9
8
7
6
5
4
3
2
1
ppm

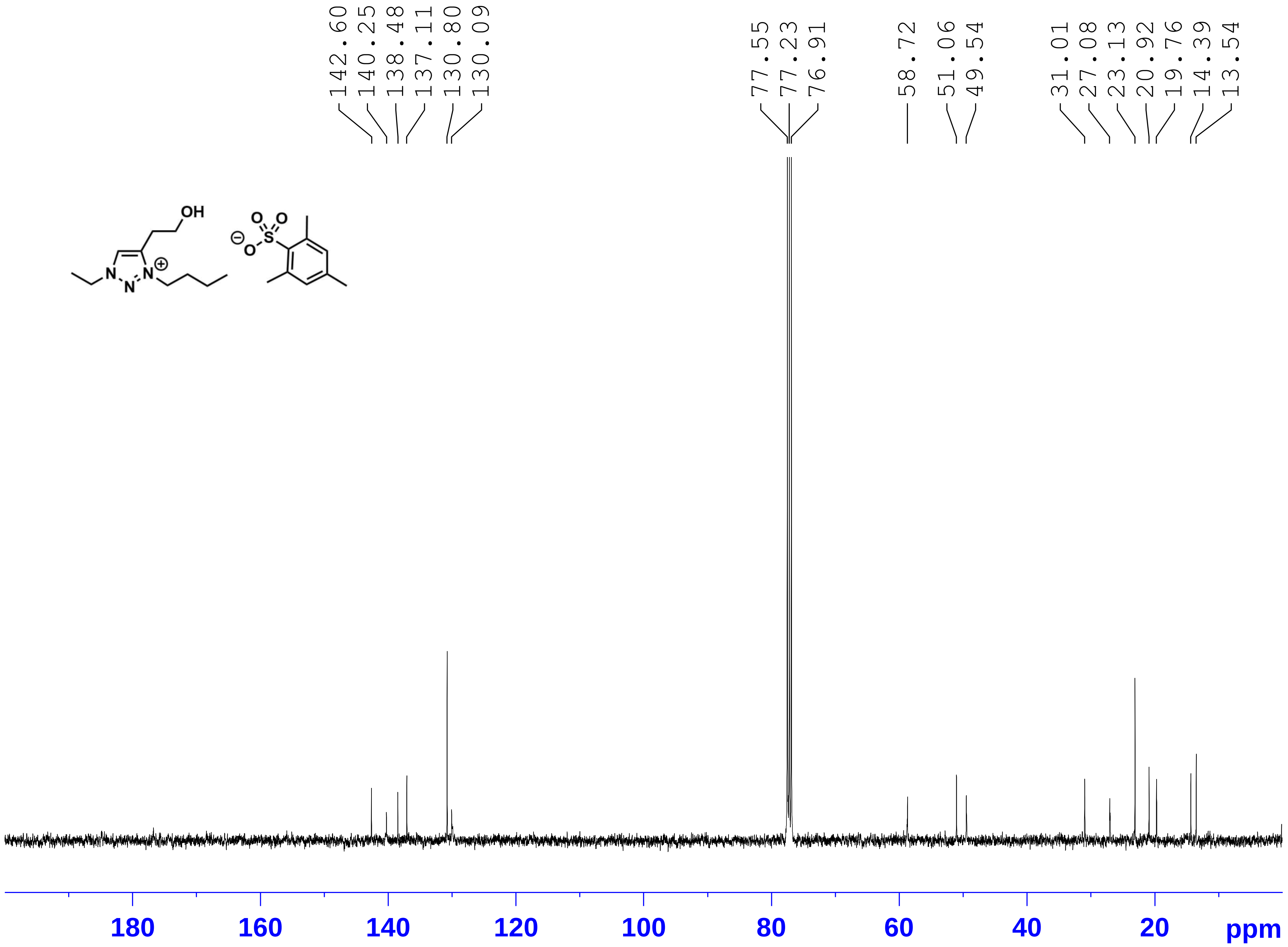

142.60
140.25
138.48
137.11
130.80
130.09
77.55
77.23
76.91
58.72
51.06
49.54
31.01
27.08
23.13
20.92
19.76
14.39
13.54
OH
O
S
N
180
160
140
120
100
80
60
40
20
ppm

Spectrum

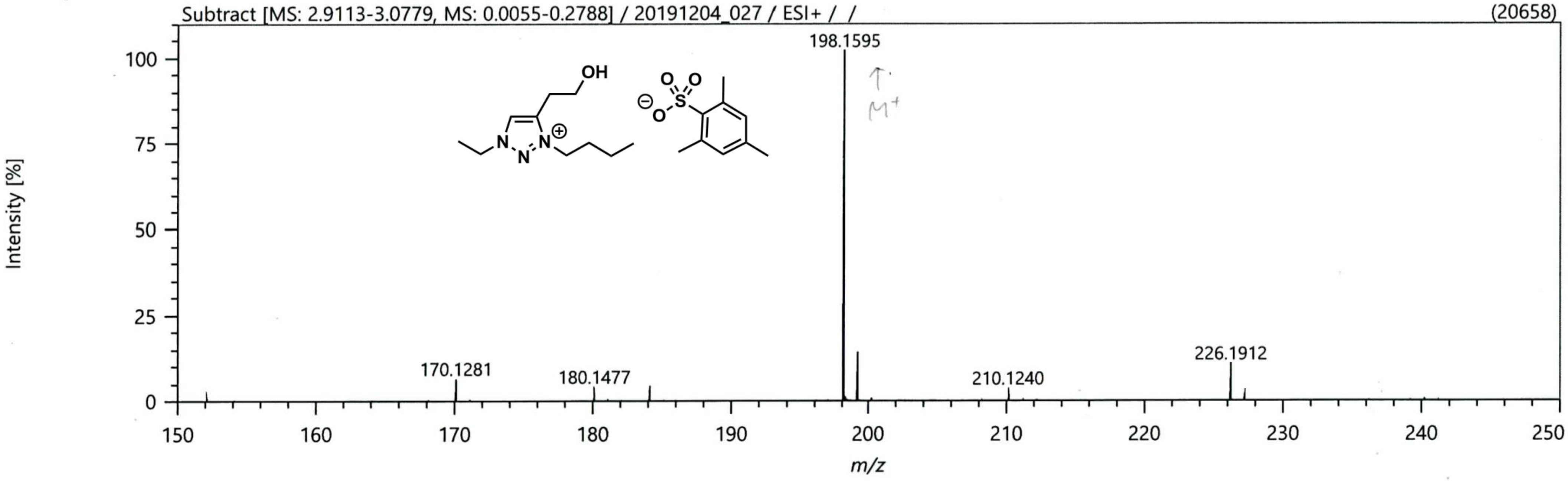

Elemental Composition

Parameters

Tolerance:	±10.00 ppm
Electron:	Odd/Even
Charge:	+1
DBE:	-1.5 - 999.0

Elements Set 1:

Symbol	C	H	O	Na	N	S	P	Si	F	Cl
Min	0	0	1	0	3	0	0	0	0	0
Max	400	1000	1	0	3	0	0	0	0	0

Results

Mass	Formula	Calculated Mass	Mass Difference [mDa]	Mass Difference [ppm]	DBE
198.15954	C10 H20 N3 O	198.16009	-0.55	-2.78	2.5

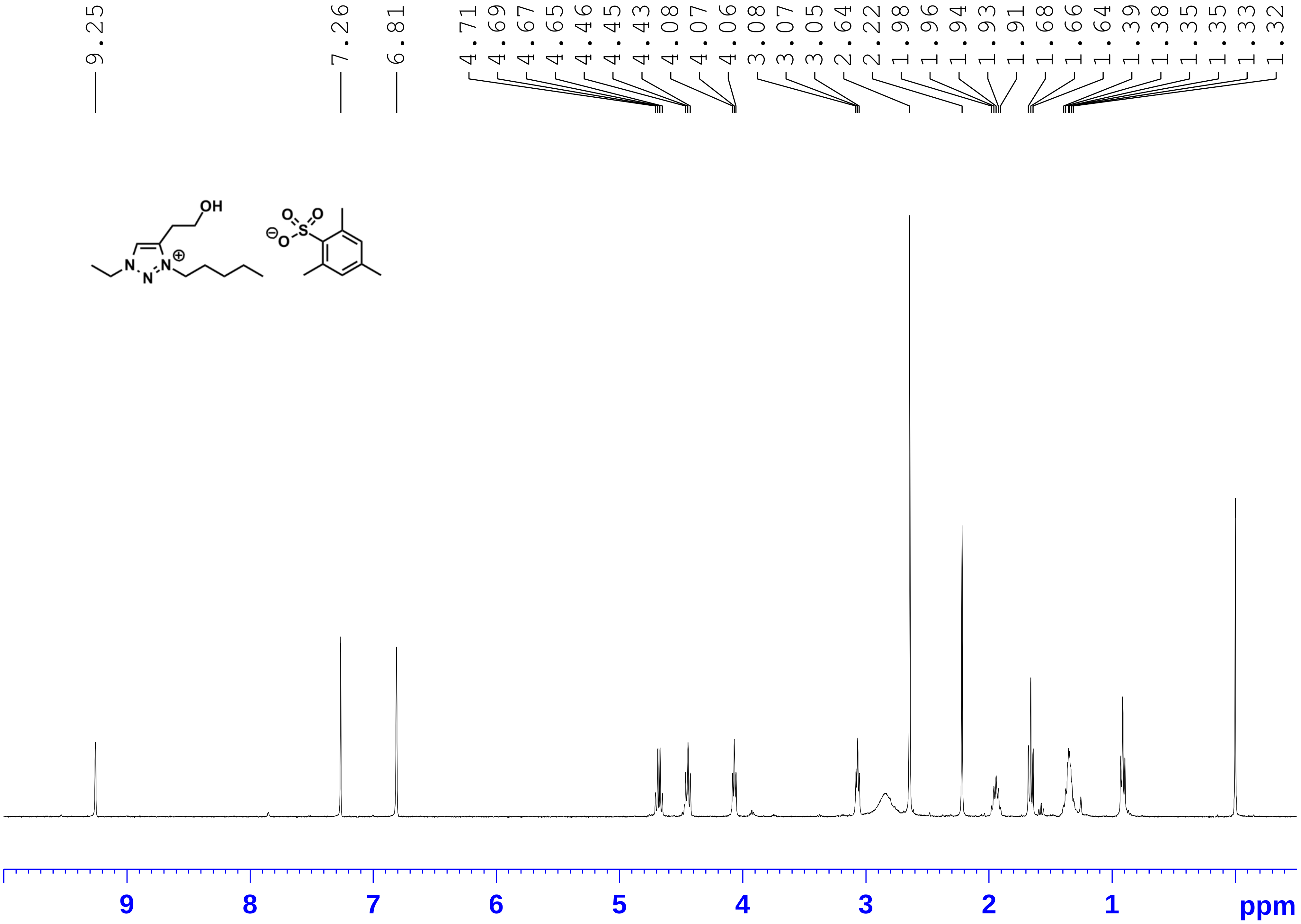
9.25
7.26
6.81
4.71 4.69 4.67 4.65 4.46 4.45 4.43 4.08 4.07 4.06 3.08 3.07 3.05 2.64 2.22 1.98 1.96 1.94 1.93 1.91 1.68 1.66 1.64 1.39 1.38 1.35 1.35 1.33 1.32
OH
N
N
N
O
O
S
O
9 8 7 6 5 4 3 2 1 ppm

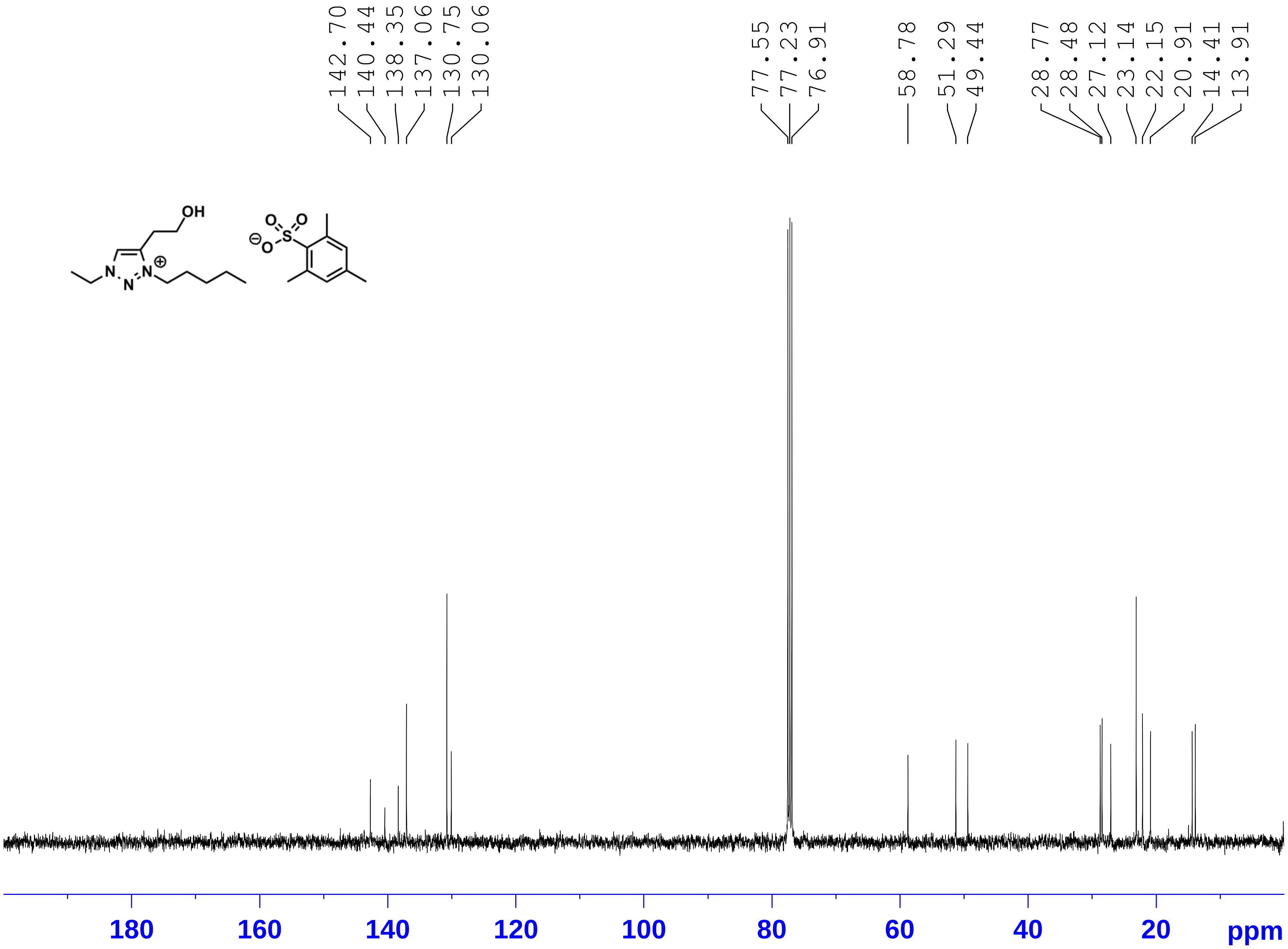
142.70
140.44
138.35
137.06
130.75
130.06
77.55
77.23
76.91
58.78
51.29
49.44
28.77
28.48
27.12
23.14
22.15
20.91
14.41
13.91
OH
180
160
140
120
100
80
60
40
20
ppm

Spectrum

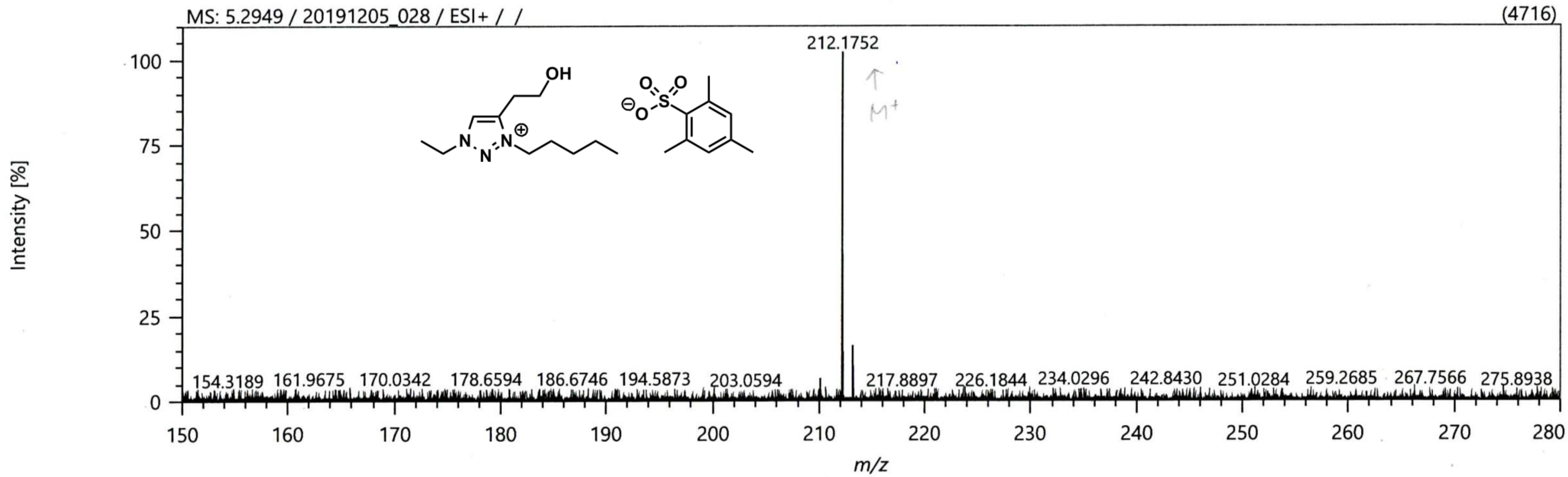

Elemental Composition

Parameters

Tolerance:	±50.00 ppm
Electron:	Odd/Even
Charge:	+1
DBE:	-1.5 - 999.0

Elements Set 1:

Symbol	C	H	O	Na	N	S	P	Si	F	Cl
Min	0	0	1	0	3	0	0	0	0	0
Max	400	1000	1	0	3	0	0	0	0	0

Results

Mass	Formula	Calculated Mass	Mass Difference [mDa]	Mass Difference [ppm]	DBE
212.17521	C11 H22 N3 O	212.17574	-0.53	-2.49	2.5

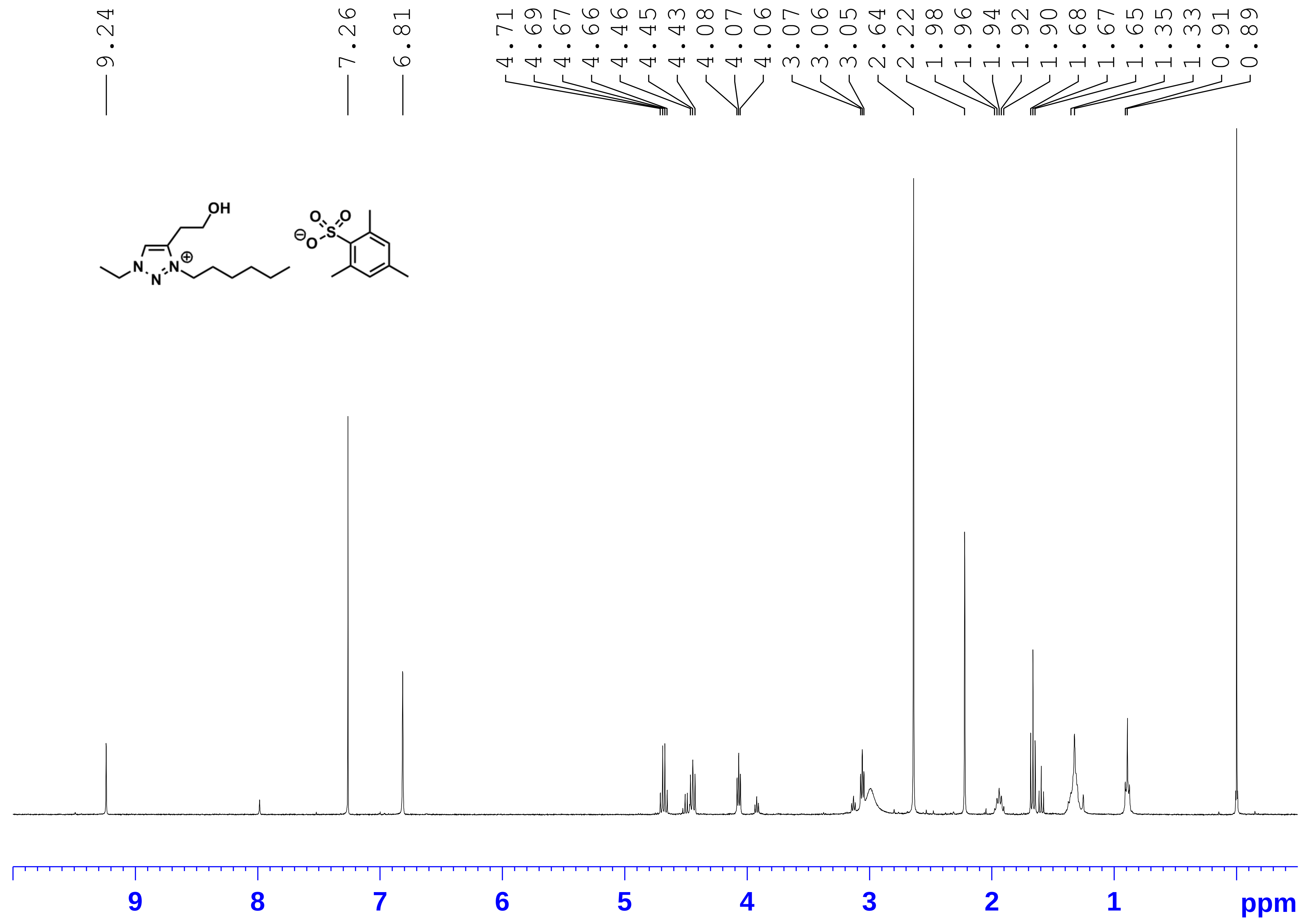

9.24
7.26
6.81
4.71
4.69
4.67
4.66
4.46
4.45
4.43
4.08
4.07
4.06
3.07
3.06
3.05
2.64
2.22
1.98
1.96
1.94
1.92
1.90
1.68
1.67
1.65
1.35
1.33
0.91
0.89
OH
N
N
N
O
S
O
O
9
8
7
6
5
4
3
2
1
ppm

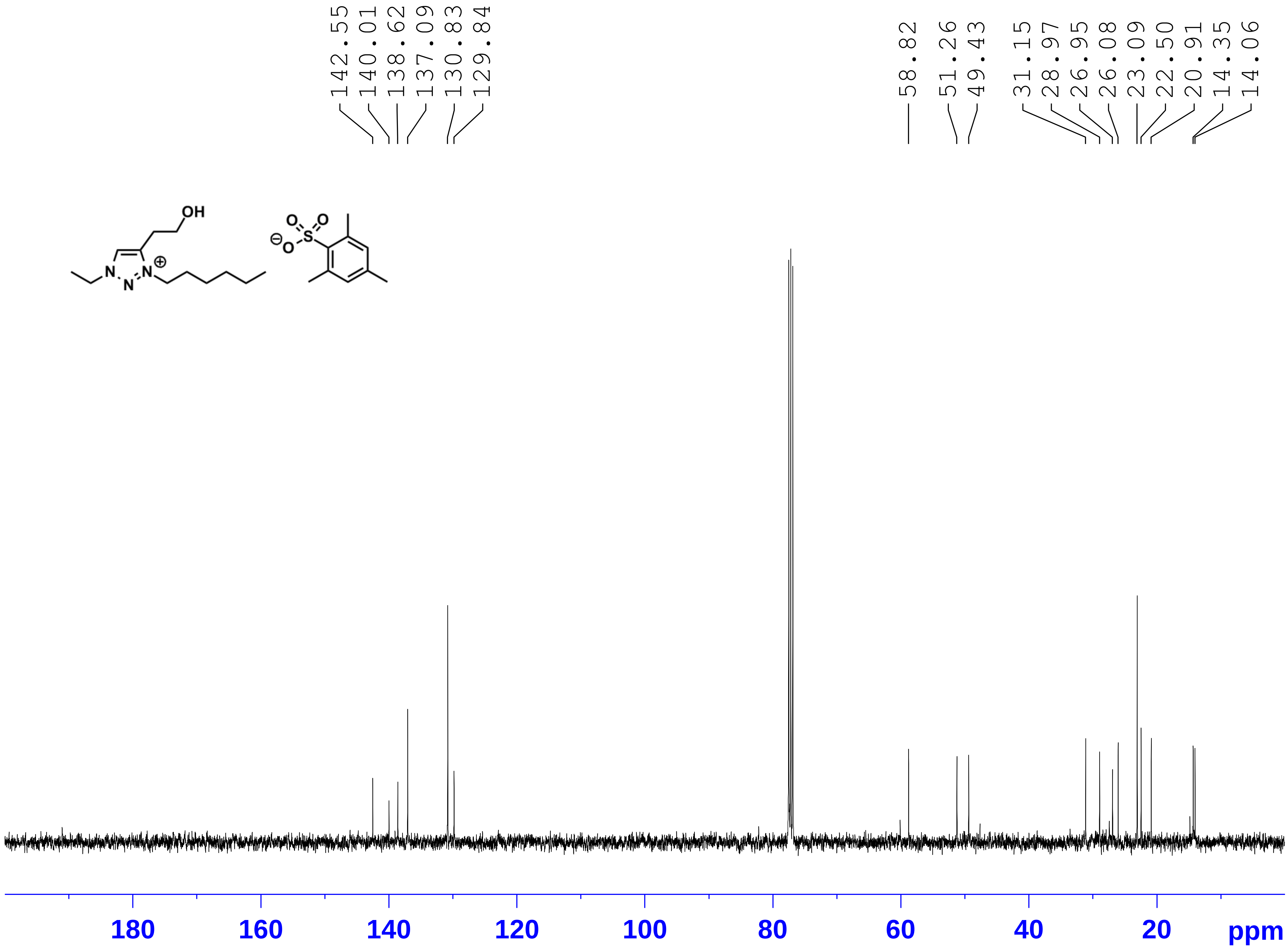

142.55
140.01
138.62
137.09
130.83
129.84
58.82
51.26
49.43
31.15
28.97
26.95
26.08
23.09
22.50
20.91
14.35
14.06
OH
O
S
O
N
N
N
180
160
140
120
100
80
60
40
20
ppm

Spectrum

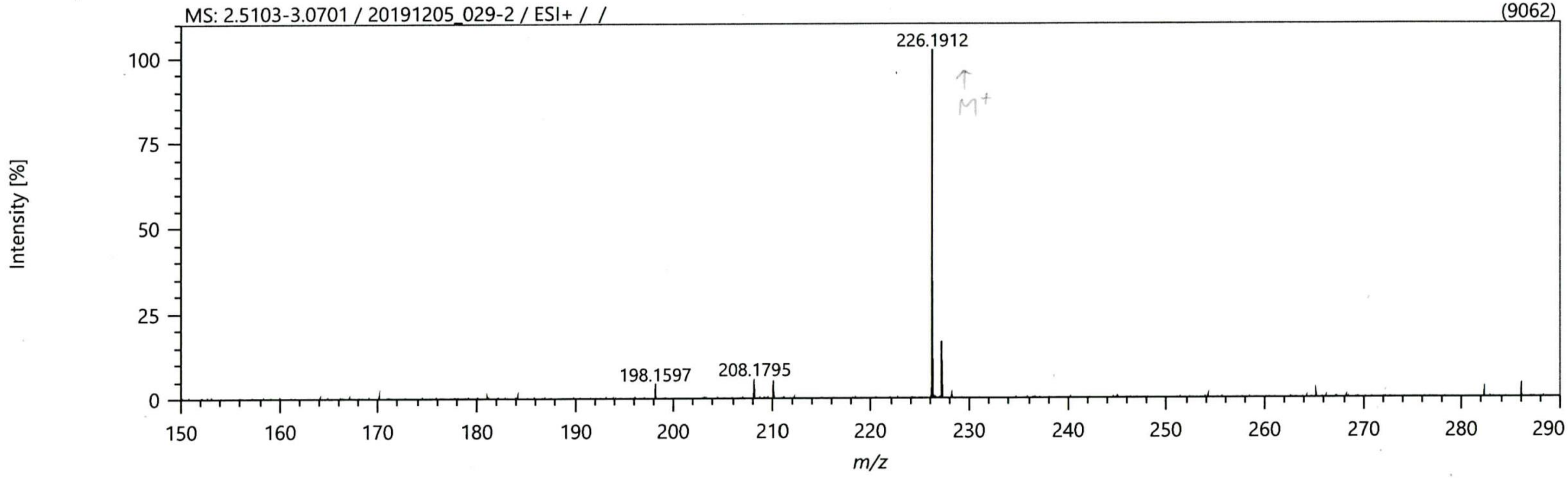

Elemental Composition

Parameters	
Tolerance:	±50.00 ppm
Electron:	Odd/Even
Charge:	+1
DBE:	-1.5 - 999.0

Elements Set 1:

Symbol	C	H	O	Na	N	S	P	Si	F	Cl
Min	0	0	1	0	3	0	0	0	0	0
Max	400	1000	1	0	3	0	0	0	0	0

Results

Mass	Formula	Calculated Mass	Mass Difference [mDa]	Mass Difference [ppm]	DBE
226.19118	C12 H24 N3 O	226.19139	-0.21	-0.93	2.5

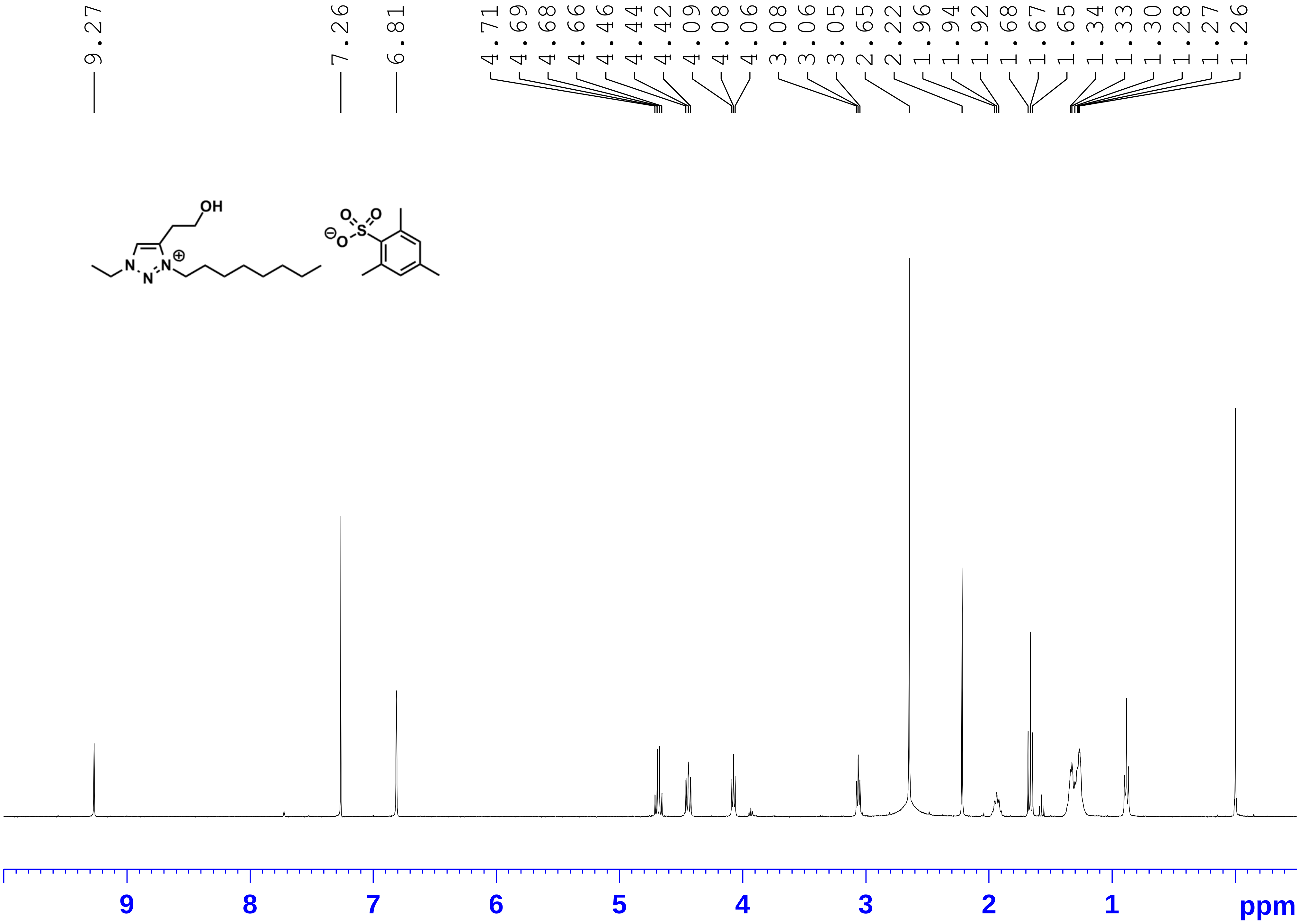

9.27
7.26
6.81
4.71
4.69
4.68
4.66
4.46
4.44
4.42
4.09
4.08
4.06
3.08
3.06
3.05
2.65
2.22
1.96
1.94
1.92
1.68
1.67
1.65
1.34
1.33
1.30
1.28
1.27
1.26
OH
N
N
N
O
O
S
O
9
8
7
6
5
4
3
2
1
ppm

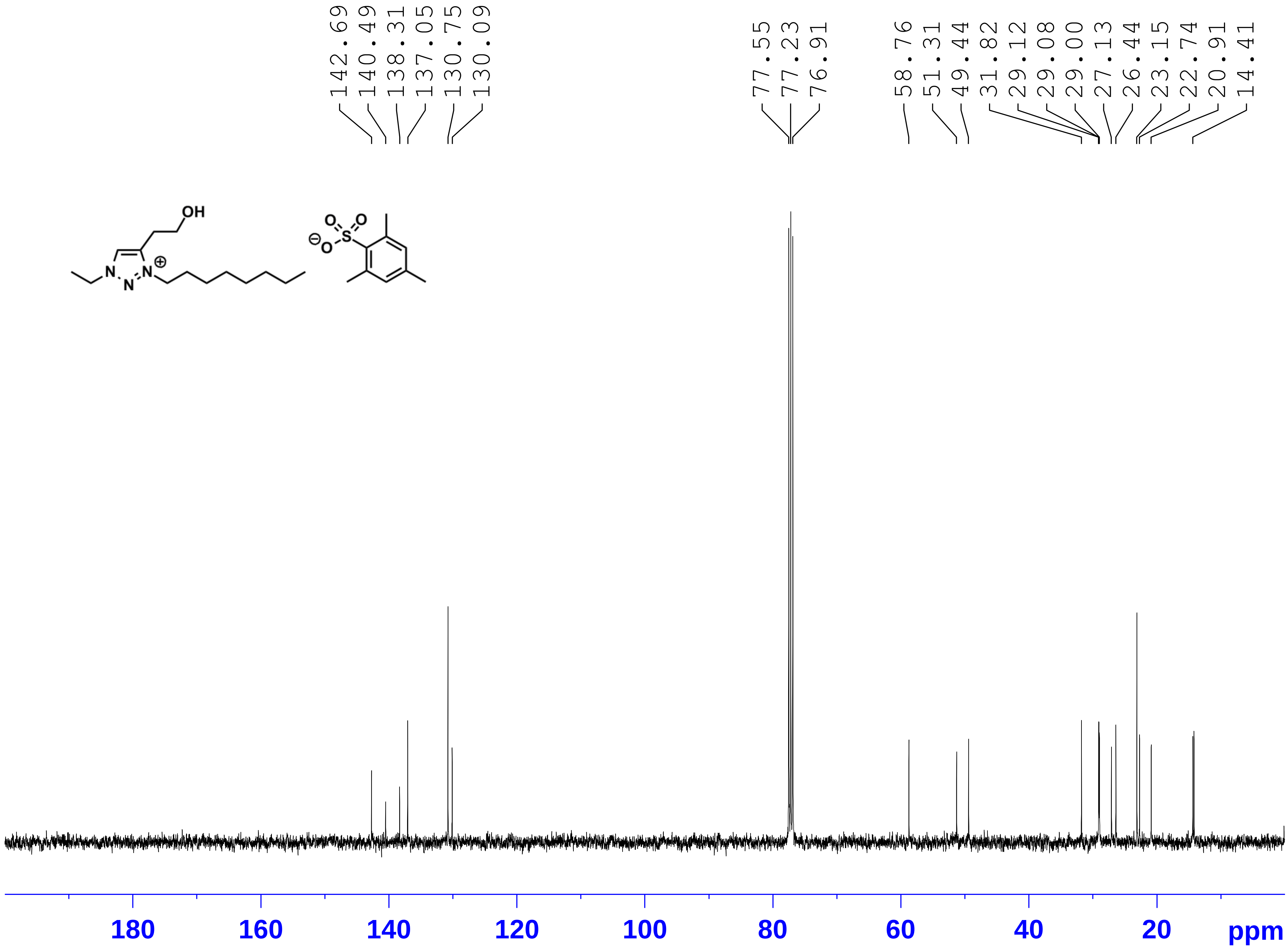

142.69
140.49
138.31
137.05
130.75
130.09
77.55
77.23
76.91
58.76
51.31
49.44
31.82
29.12
29.08
29.00
27.13
26.44
23.15
22.74
20.91
14.41
OH
N
N
N
O
O
S
O
180
160
140
120
100
80
60
40
20
ppm

Spectrum

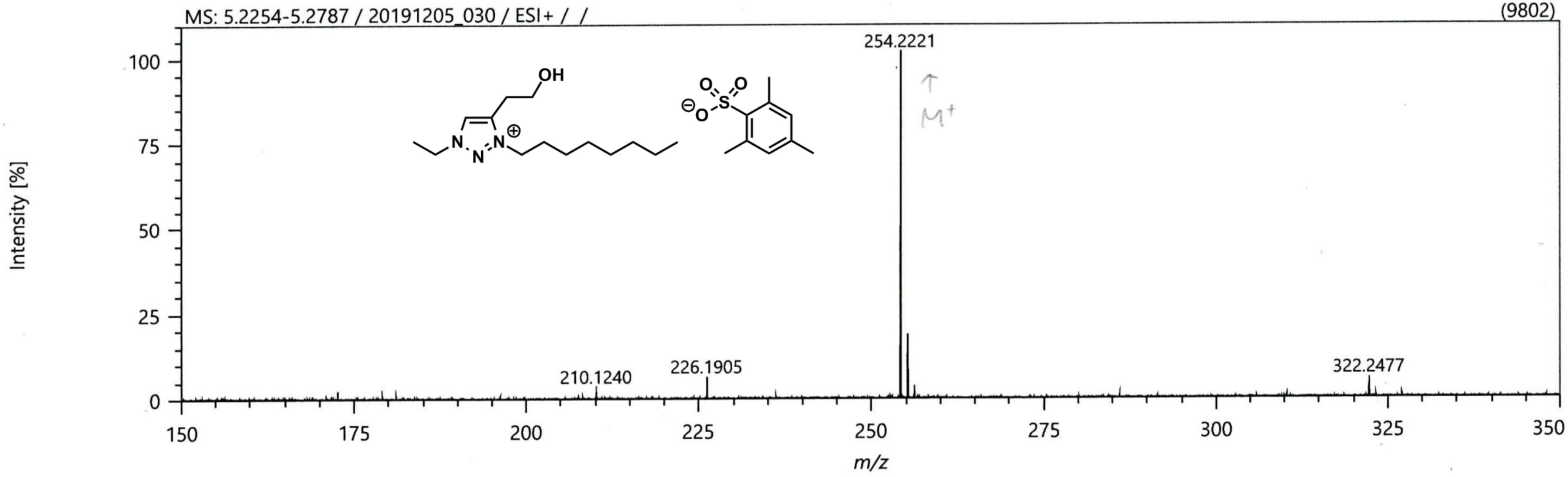

Elemental Composition

Parameters		Elements Set 1:										
Tolerance:	±50.00 ppm	Symbol	C	H	O	Na	N	S	P	Si	F	Cl
Electron:	Odd/Even	Min	0	0	1	0	3	0	0	0	0	0
Charge:	+1	Max	400	1000	1	0	3	0	0	0	0	0
DBE:	-1.5 - 999.0											

Results

Mass	Formula	Calculated Mass	Mass Difference [mDa]	Mass Difference [ppm]	DBE
254.22211	C14 H28 N3 O	254.22269	-0.58	-2.28	2.5

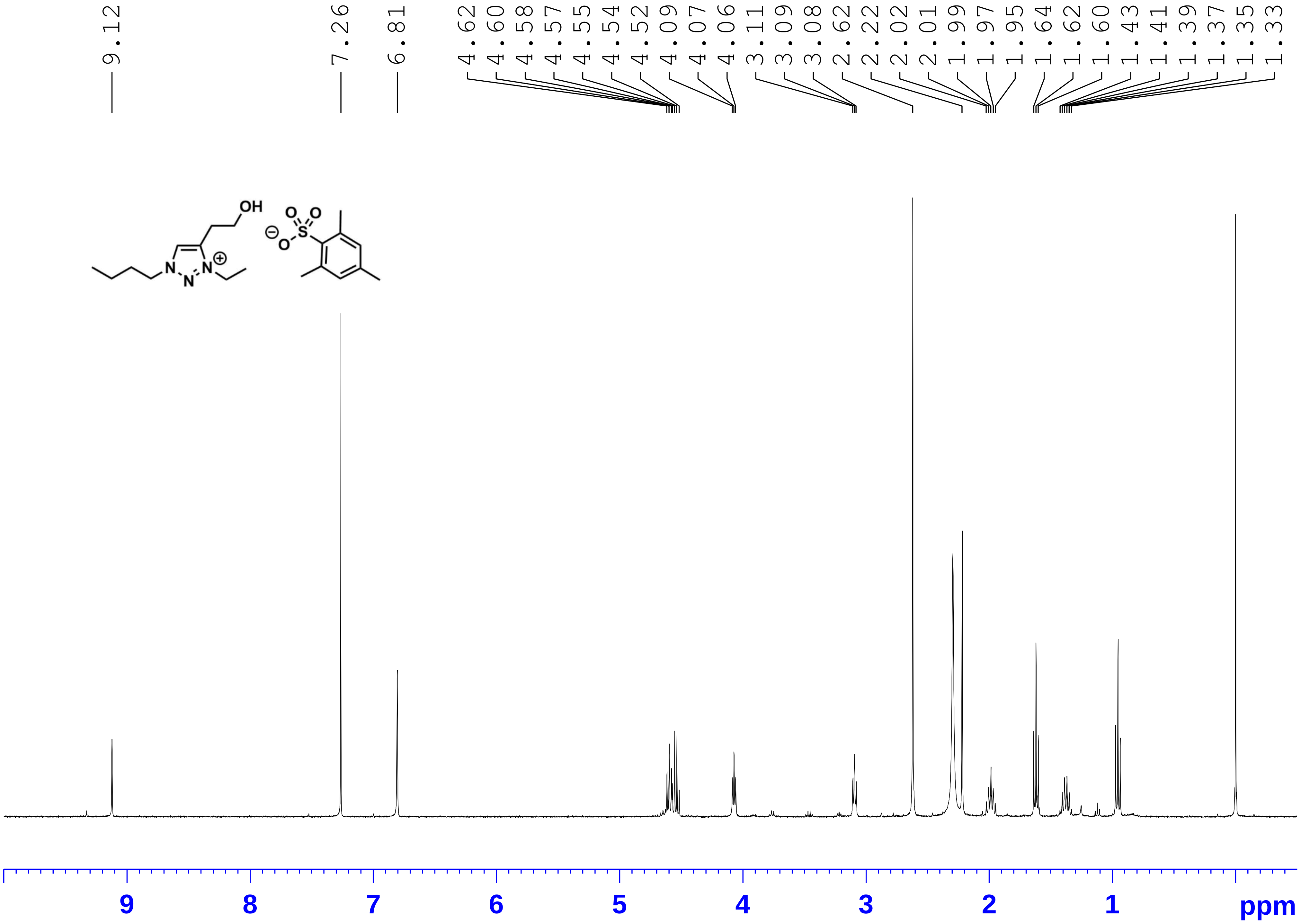

9.12
7.26
6.81
4.62
4.60
4.58
4.57
4.55
4.54
4.52
4.09
4.07
4.06
3.11
3.09
3.08
2.62
2.22
2.02
2.01
1.99
1.97
1.95
1.64
1.62
1.60
1.43
1.41
1.39
1.37
1.35
1.33
OH
N
N
N
O
O
S
O
9
8
7
6
5
4
3
2
1
ppm

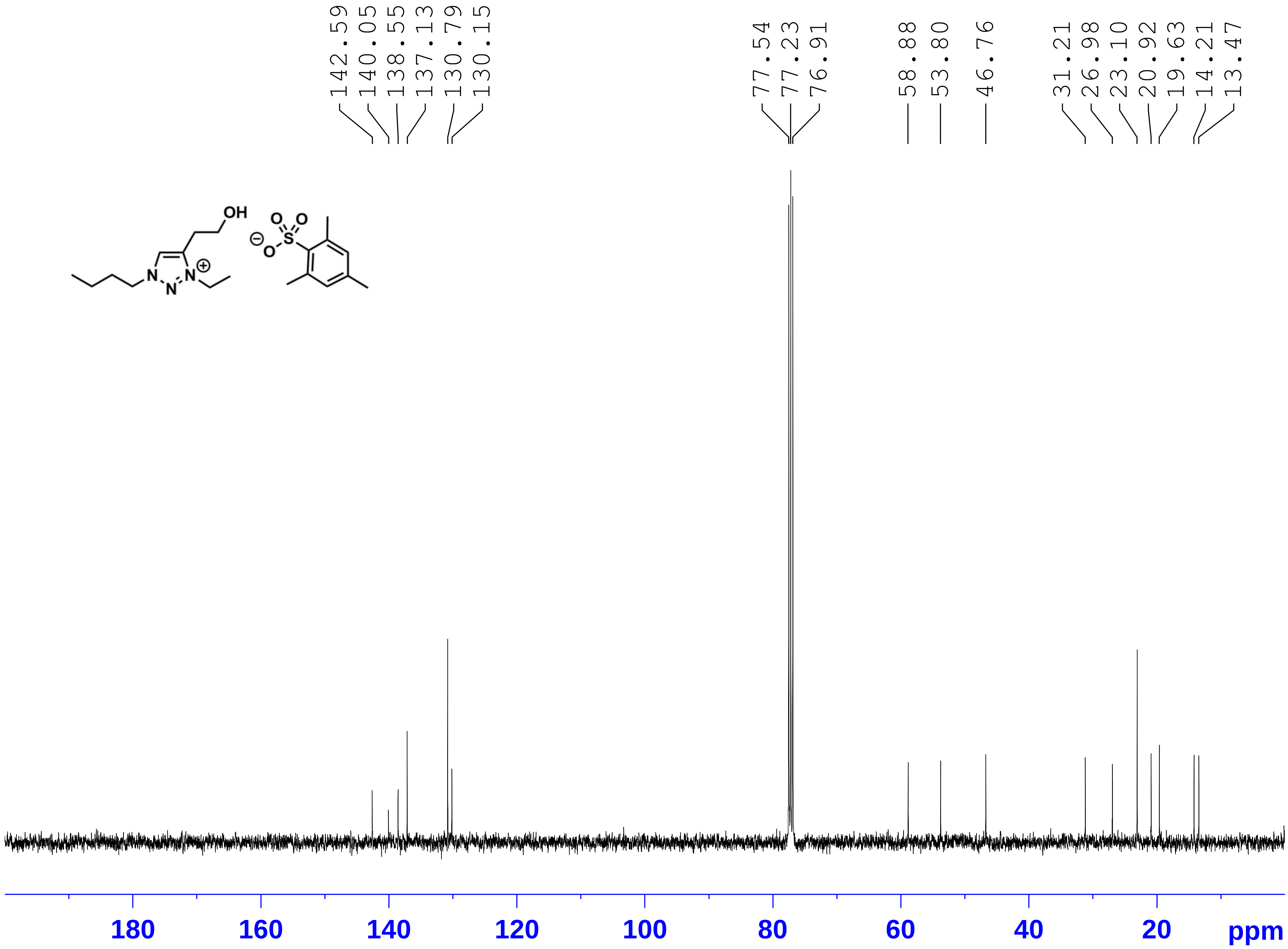

142.59
140.05
138.55
137.13
130.79
130.15
77.54
77.23
76.91
58.88
53.80
46.76
31.21
26.98
23.10
20.92
19.63
14.21
13.47
OH
O
S
N
N
N
180
160
140
120
100
80
60
40
20
ppm

Spectrum

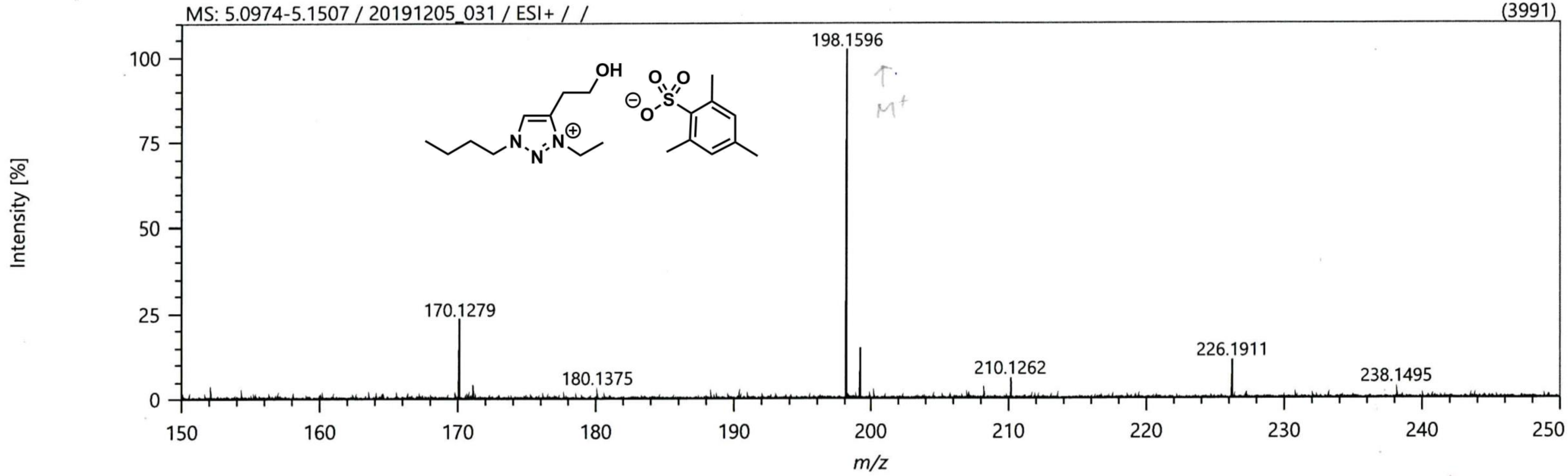

Elemental Composition

Parameters		Elements Set 1:										
Tolerance:	±50.00 ppm	Symbol	C	H	O	Na	N	S	P	Si	F	Cl
Electron:	Odd/Even	Min	0	0	1	0	3	0	0	0	0	0
Charge:	+1	Max	400	1000	1	0	3	0	0	0	0	0
DBE:	-1.5 - 999.0											

Results

Mass	Formula	Calculated Mass	Mass Difference [mDa]	Mass Difference [ppm]	DBE
198.15958	C10 H20 N3 O	198.16009	-0.51	-2.57	2.5

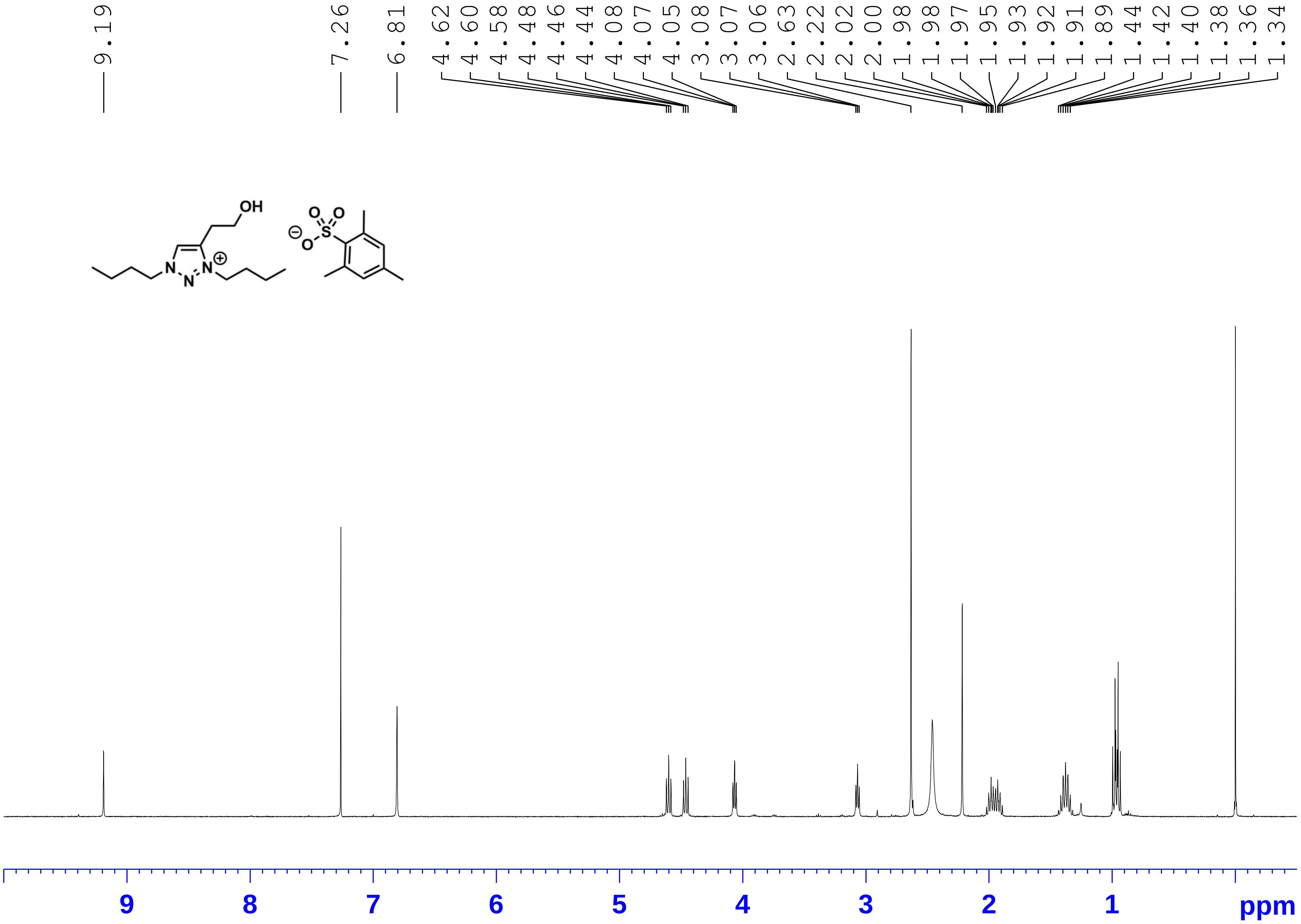
9.19
7.26
6.81
4.62
4.60
4.58
4.48
4.46
4.44
4.08
4.07
4.05
3.08
3.07
3.06
2.63
2.22
2.02
2.00
1.98
1.98
1.97
1.95
1.93
1.92
1.91
1.89
1.44
1.42
1.40
1.38
1.36
1.34
OH
N
N
N
O
O
S
O
9
8
7
6
5
4
3
2
1
ppm

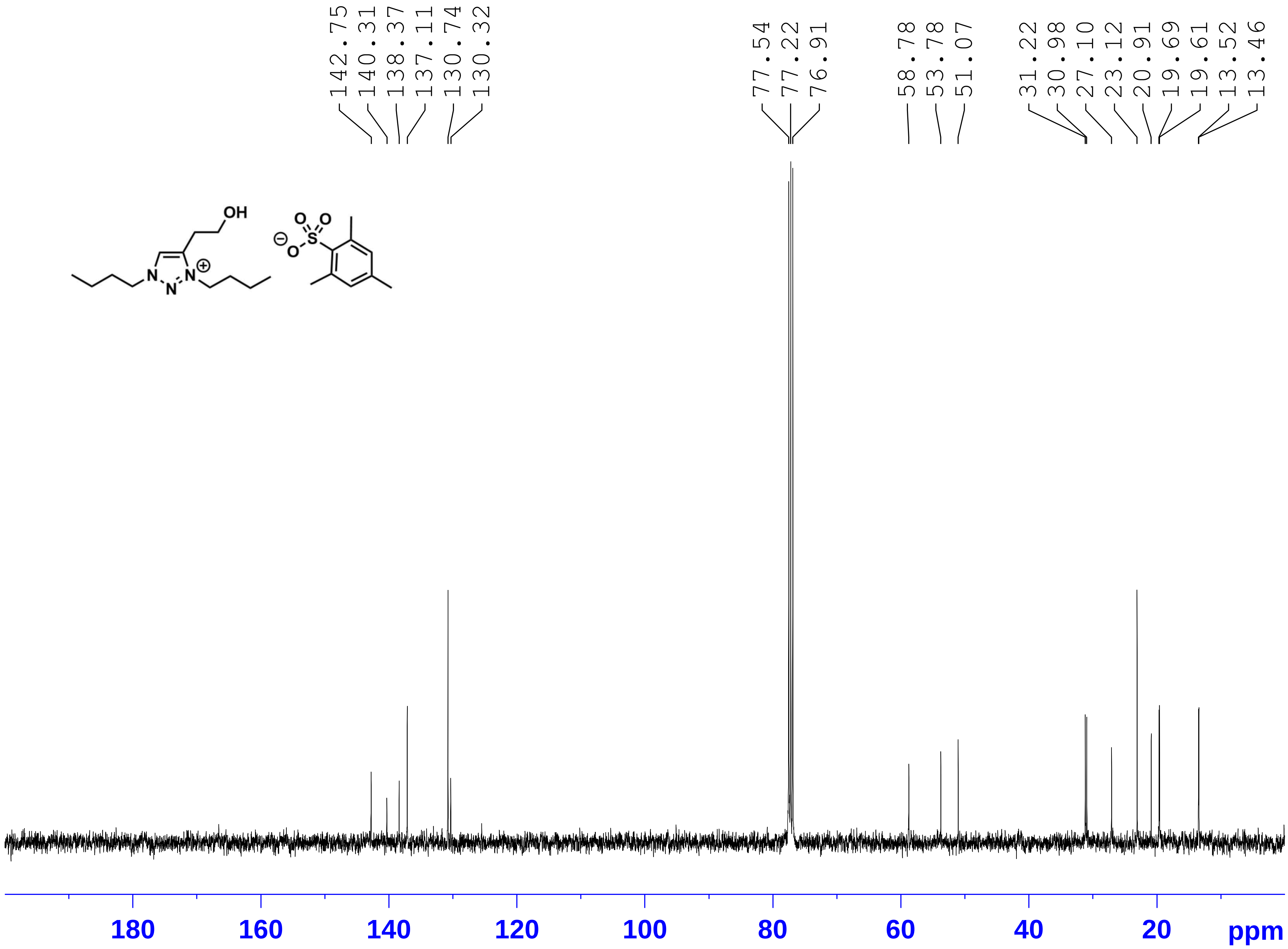

142.75
140.31
138.37
137.11
130.74
130.32
77.54
77.22
76.91
58.78
53.78
51.07
31.22
30.98
27.10
23.12
20.91
19.69
19.61
13.52
13.46
OH
O
O
S
O
N
N
N
180
160
140
120
100
80
60
40
20
ppm

Spectrum

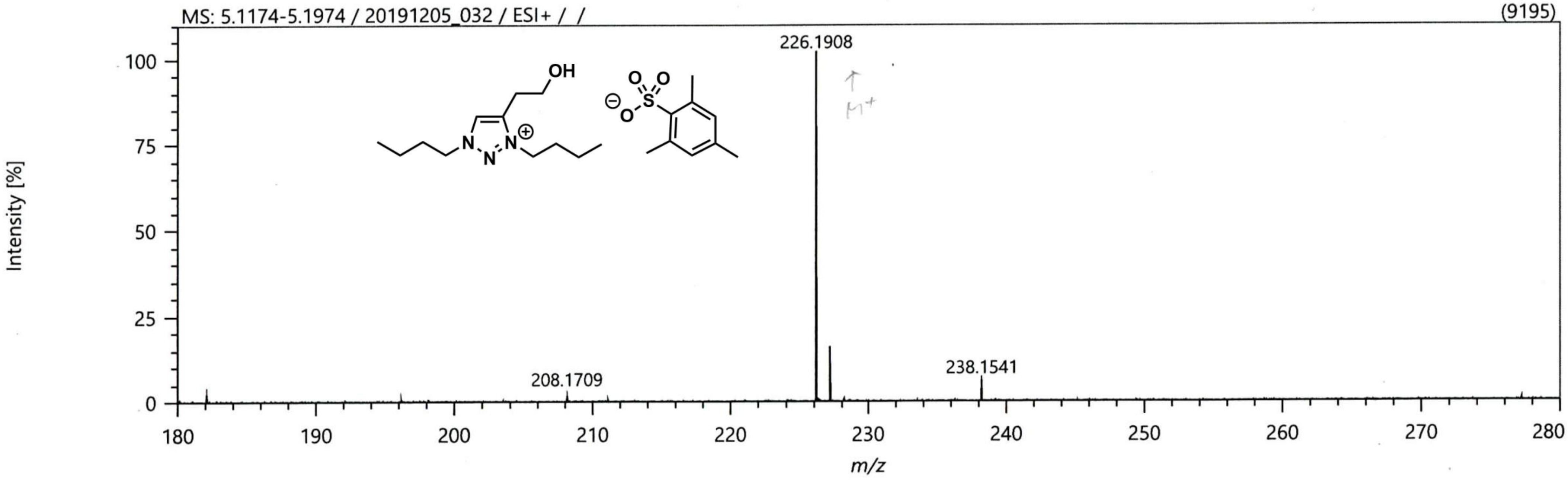

Elemental Composition

Parameters

Tolerance:	±50.00 ppm
Electron:	Odd/Even
Charge:	+1
DBE:	-1.5 - 999.0

Elements Set 1:

Symbol	C	H	O	Na	N	S	P	Si	F	Cl
Min	0	0	1	0	3	0	0	0	0	0
Max	400	1000	1	0	3	0	0	0	0	0

Results

Mass	Formula	Calculated Mass	Mass Difference [mDa]	Mass Difference [ppm]	DBE
226.19082	C12 H24 N3 O	226.19139	-0.57	-2.52	2.5

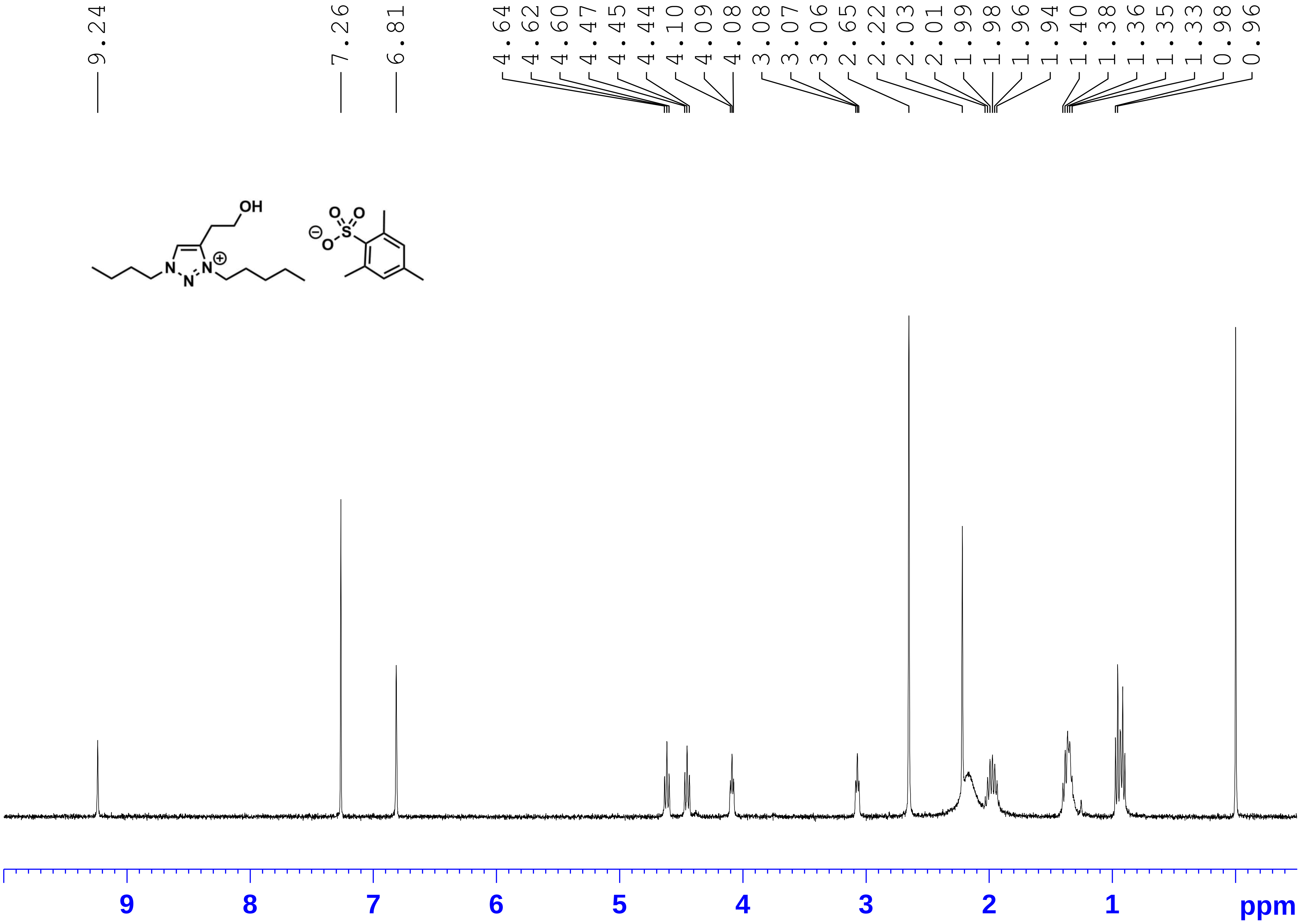
9.24
7.26
6.81
4.64
4.62
4.60
4.47
4.45
4.44
4.10
4.09
4.08
3.08
3.07
3.06
2.65
2.22
2.03
2.01
1.99
1.98
1.96
1.94
1.40
1.38
1.36
1.35
1.33
0.98
0.96
OH
N
N
N
O
S
O
O
9
8
7
6
5
4
3
2
1
ppm

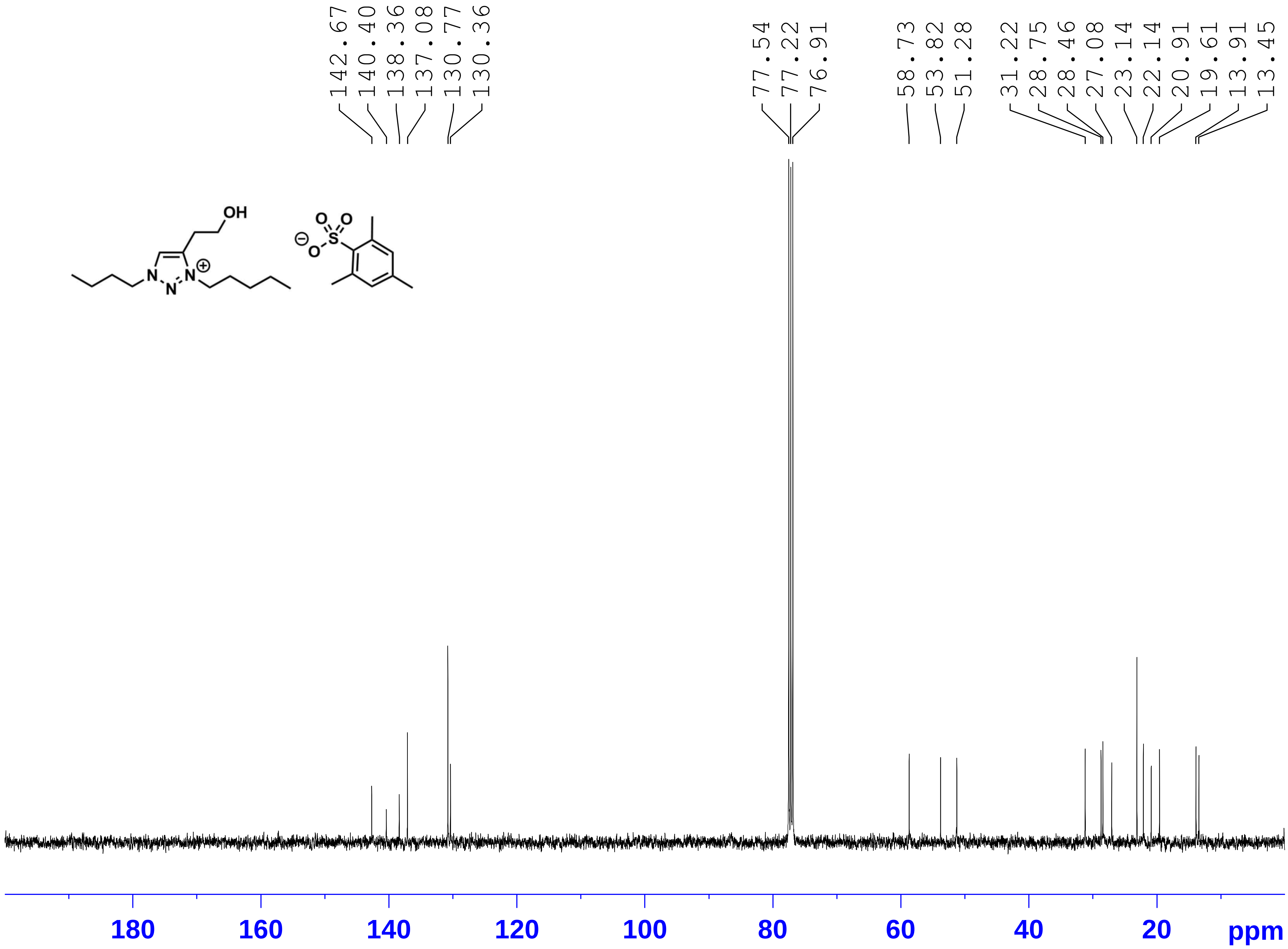
142.67
140.40
138.36
137.08
130.77
130.36
77.54
77.22
76.91
58.73
53.82
51.28
31.22
28.75
28.46
27.08
23.14
22.14
20.91
19.61
13.91
13.45
OH
O
O
S
O
N
N
N
180
160
140
120
100
80
60
40
20
ppm

Spectrum

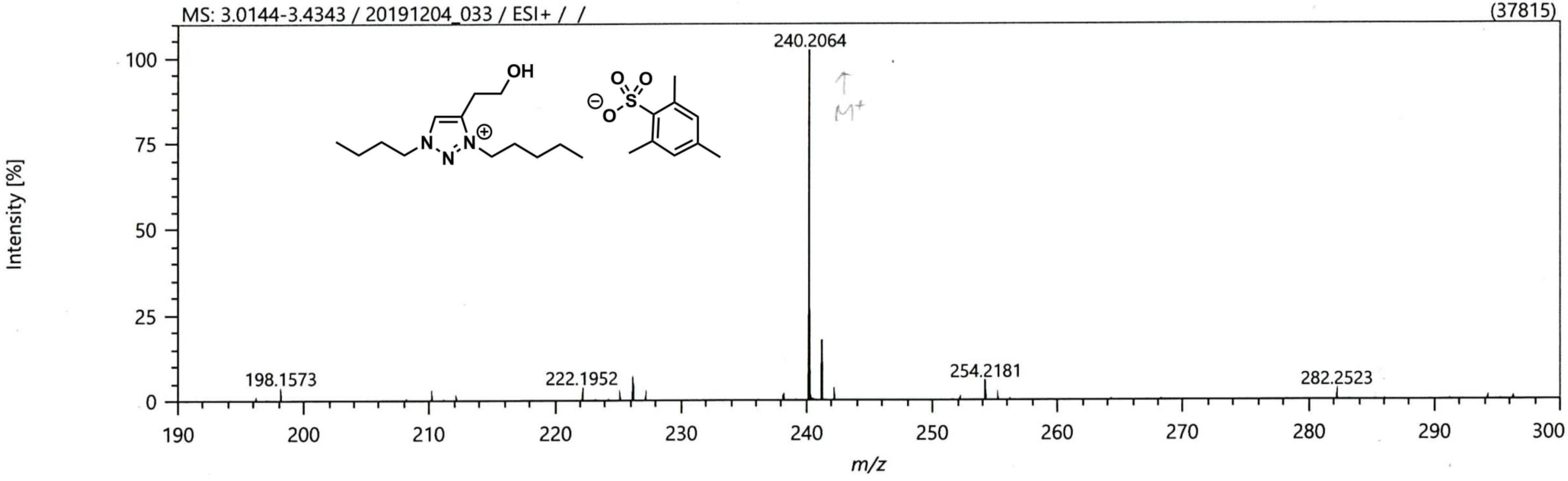

Elemental Composition

Parameters

Tolerance:	±10.00 ppm
Electron:	Odd/Even
Charge:	+1
DBE:	-1.5 - 999.0

Elements Set 1:

Symbol	C	H	O	Na	N	S	P	Si	F	Cl
Min	0	0	1	0	3	0	0	0	0	0
Max	400	1000	1	0	3	0	0	0	0	0

Results

Mass	Formula	Calculated Mass	Mass Difference [mDa]	Mass Difference [ppm]	DBE
240.20638	C13 H26 N3 O	240.20704	-0.66	-2.75	2.5

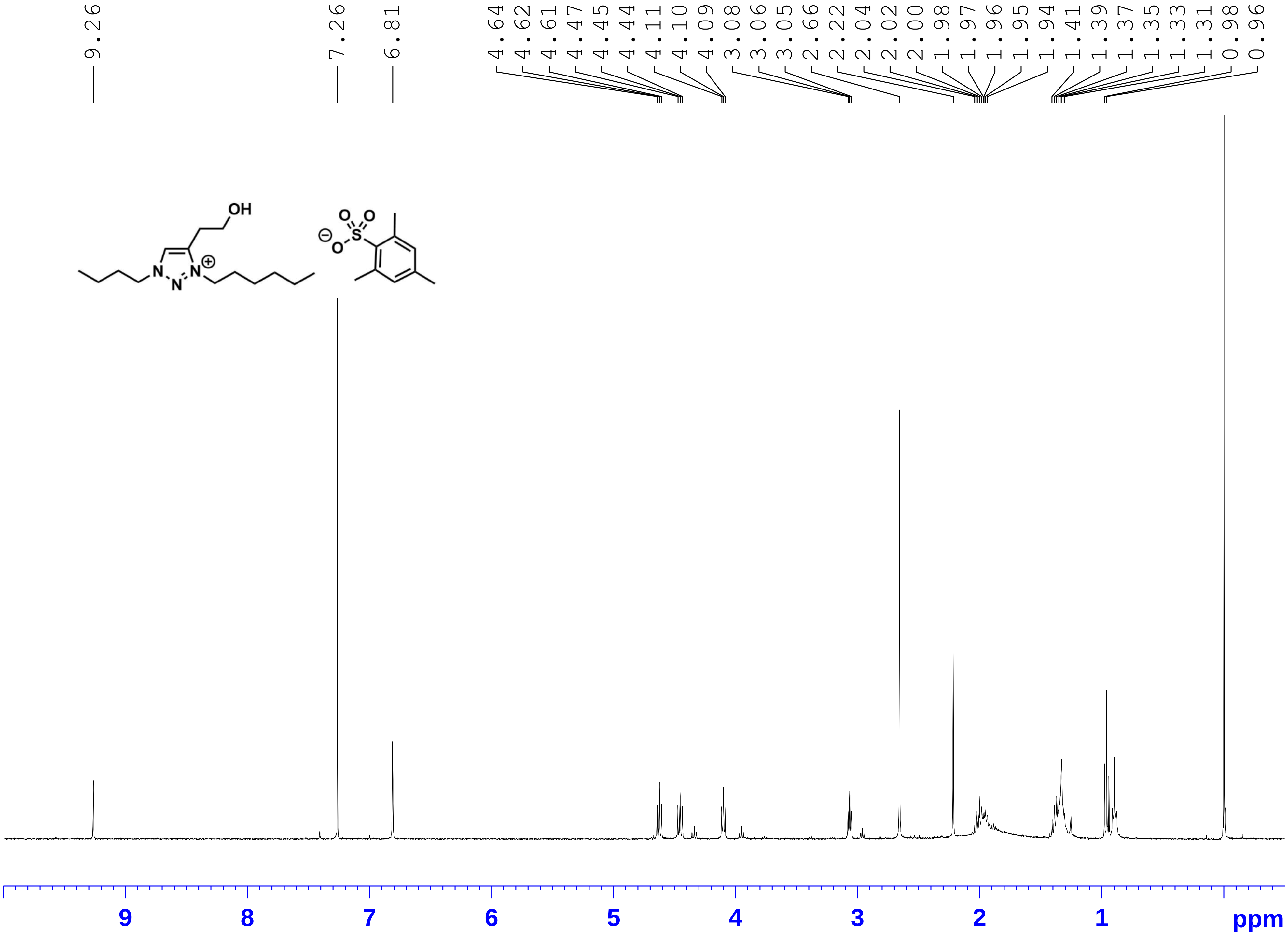

9.26
7.26
6.81
4.64
4.62
4.61
4.47
4.45
4.44
4.11
4.10
4.09
3.08
3.06
3.05
2.66
2.22
2.04
2.02
2.00
1.98
1.97
1.96
1.95
1.94
1.41
1.39
1.37
1.35
1.33
1.31
0.98
0.96
OH
N
N
N
O
O
O
S
9
8
7
6
5
4
3
2
1
ppm

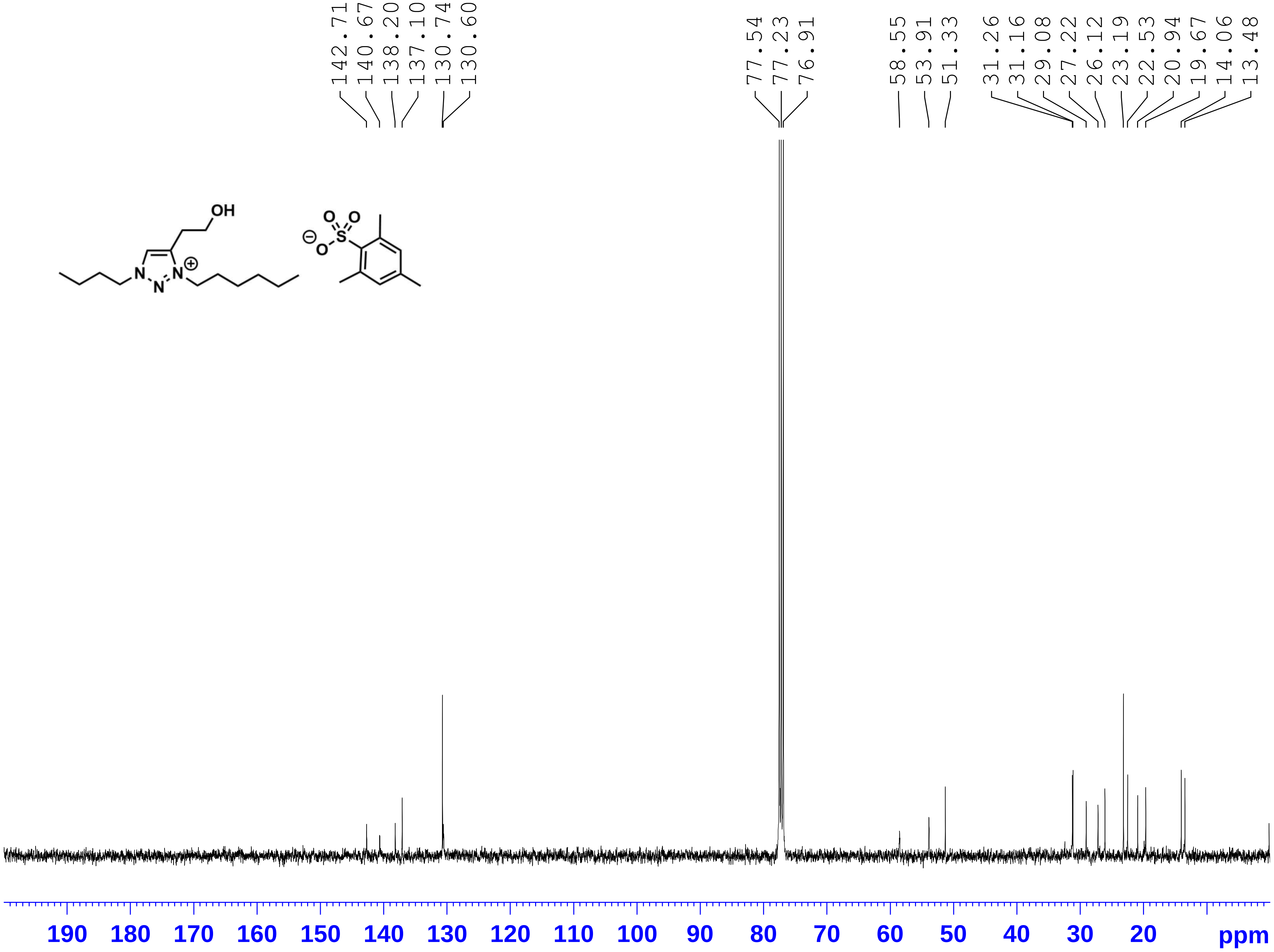
142.71
140.67
138.20
137.10
130.74
130.60
77.54
77.23
76.91
58.55
53.91
51.33
31.26
31.16
29.08
27.22
26.12
23.19
22.53
20.94
19.67
14.06
13.48
OH
N
N
N
O
O
S
O
190
180
170
160
150
140
130
120
110
100
90
80
70
60
50
40
30
20
ppm

Spectrum

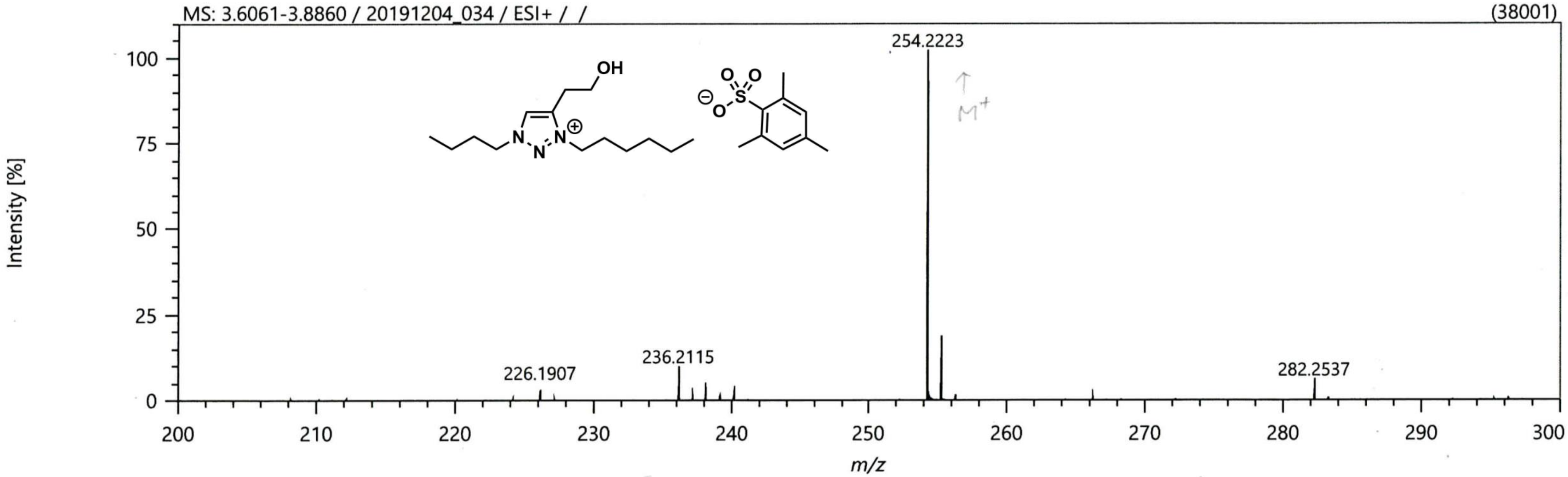

Elemental Composition

Parameters

Tolerance:	±10.00 ppm
Electron:	Odd/Even
Charge:	+1
DBE:	-1.5 - 999.0

Elements Set 1:

Symbol	C	H	O	Na	N	S	P	Si	F	Cl
Min	0	0	1	0	3	0	0	0	0	0
Max	400	1000	1	0	3	0	0	0	0	0

Results

Mass	Formula	Calculated Mass	Mass Difference [mDa]	Mass Difference [ppm]	DBE
254.22234	C14 H28 N3 O	254.22269	-0.35	-1.39	2.5

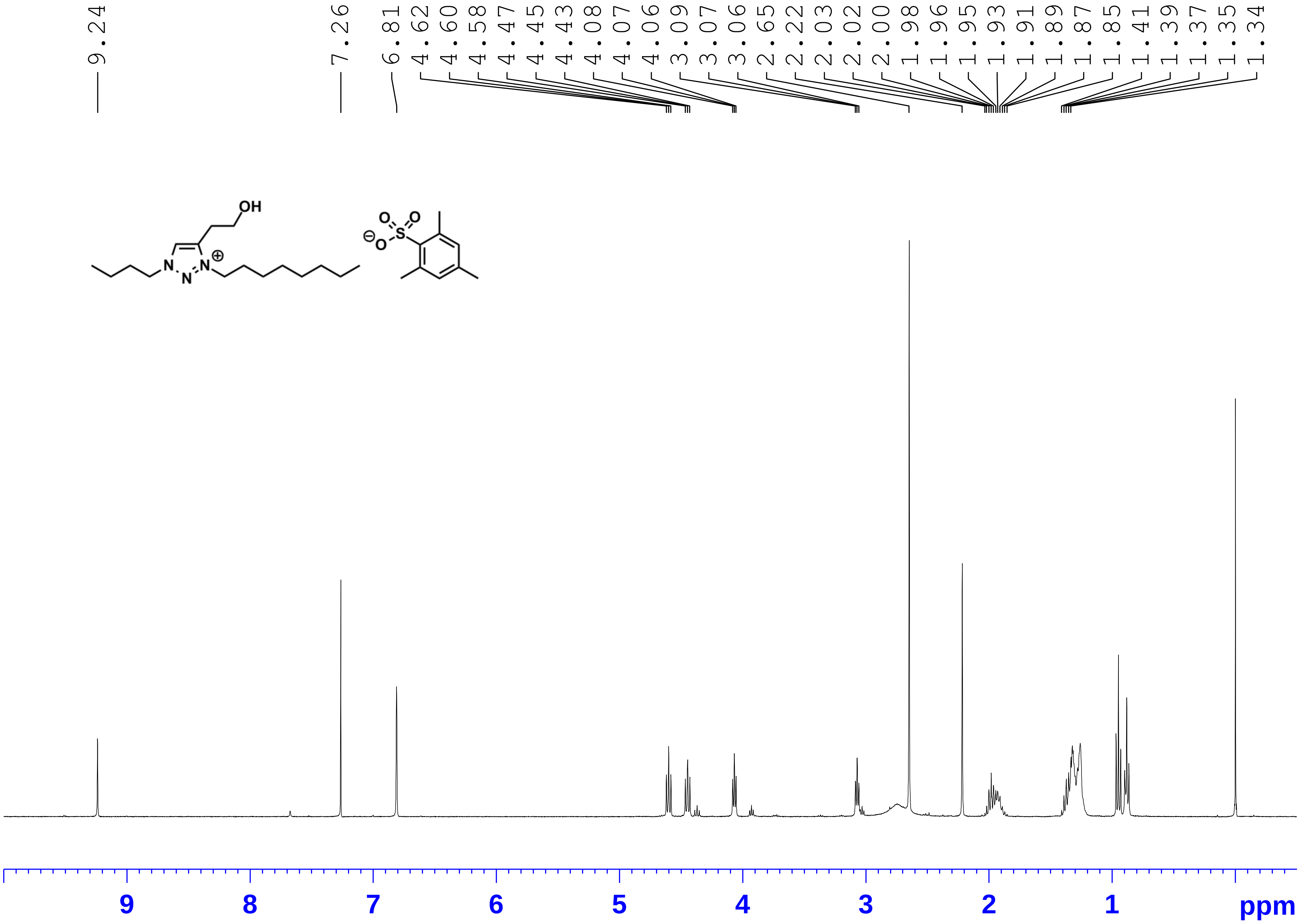

9.24
7.26
6.81
4.62
4.60
4.58
4.47
4.45
4.43
4.08
4.07
4.06
3.09
3.07
3.06
2.65
2.22
2.03
2.02
2.00
1.98
1.96
1.95
1.93
1.91
1.89
1.87
1.85
1.41
1.39
1.37
1.35
1.34
OH
N
N
N
O
O
O
S
9
8
7
6
5
4
3
2
1
ppm

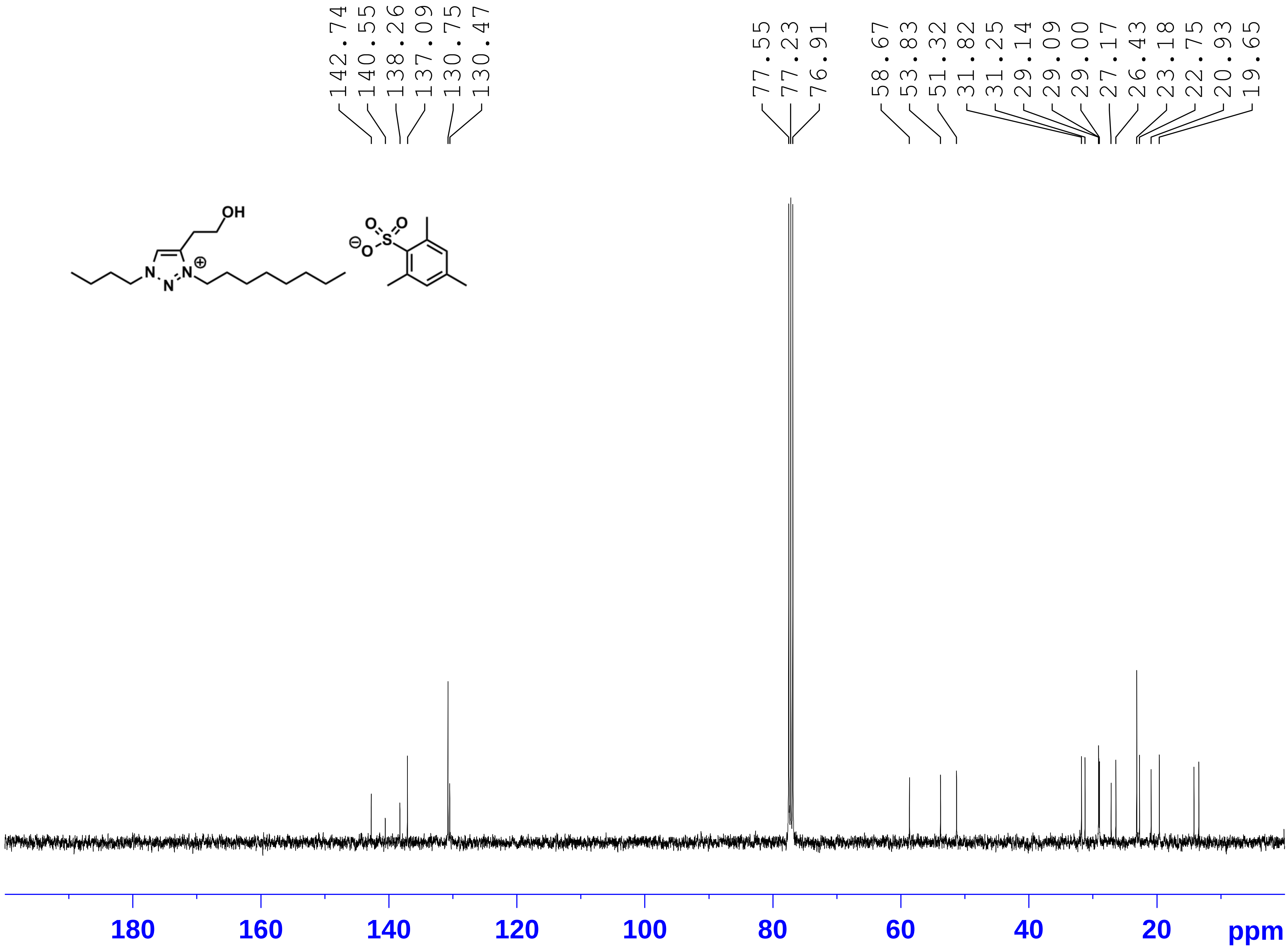

142.74
140.55
138.26
137.09
130.75
130.47
77.55
77.23
76.91
58.67
53.83
51.32
31.82
31.25
29.14
29.09
29.00
27.17
26.43
23.18
22.75
20.93
19.65
OH
N
N
N
O
O
S
O
180
160
140
120
100
80
60
40
20
ppm

Spectrum

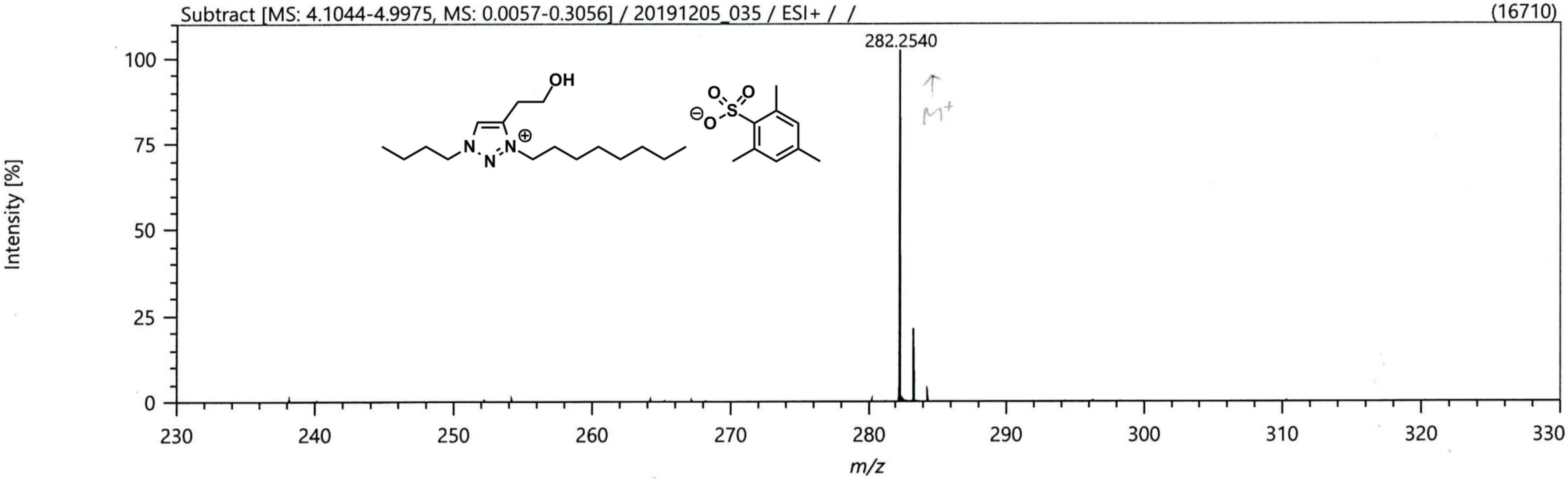

Elemental Composition

Parameters

Tolerance:	±50.00 ppm
Electron:	Odd/Even
Charge:	+1
DBE:	-1.5 - 999.0

Elements Set 1:

Symbol	C	H	O	Na	N	S	P	Si	F	Cl
Min	0	0	1	0	3	0	0	0	0	0
Max	400	1000	1	0	3	0	0	0	0	0

Results

Mass	Formula	Calculated Mass	Mass Difference [mDa]	Mass Difference [ppm]	DBE
282.25397	C16 H32 N3 O	282.25399	-0.02	-0.08	2.5

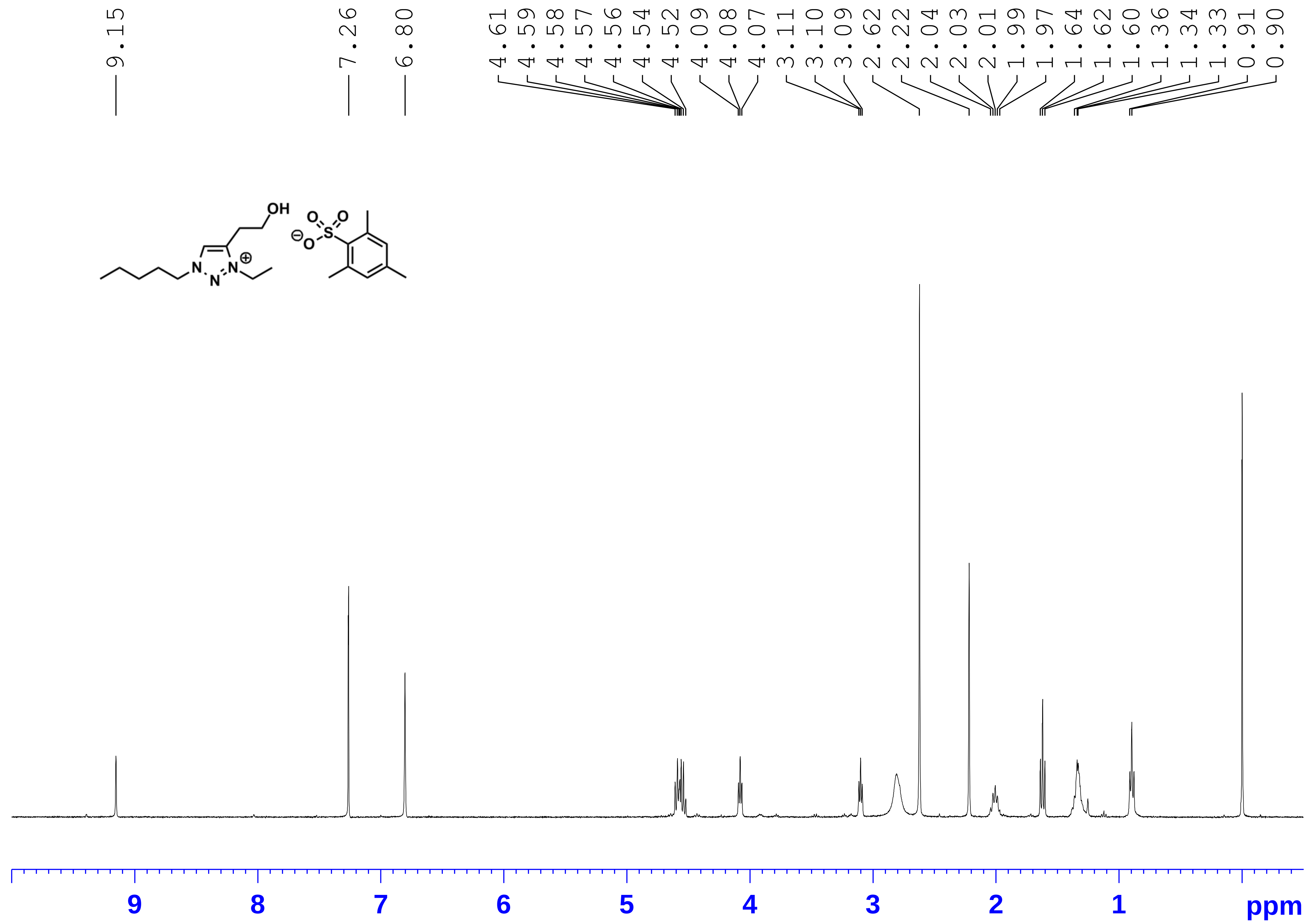
9.15
7.26
6.80
4.61
4.59
4.58
4.57
4.56
4.54
4.52
4.09
4.08
4.07
3.11
3.10
3.09
2.62
2.22
2.04
2.03
2.01
1.99
1.97
1.64
1.62
1.60
1.36
1.34
1.33
0.91
0.90
OH
O
O
S
O
N
N
N
9
8
7
6
5
4
3
2
1
ppm

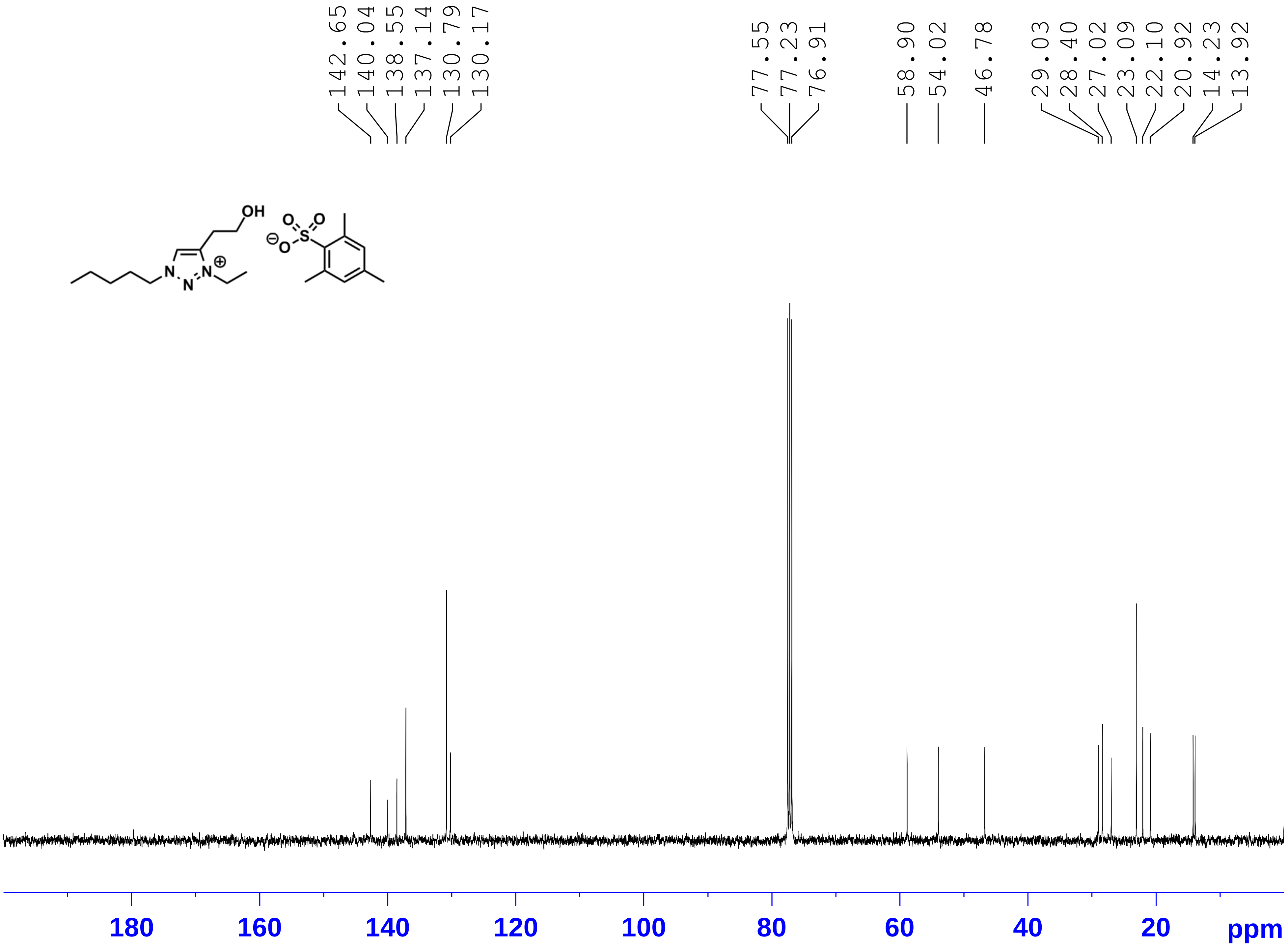
142.65
140.04
138.55
137.14
130.79
130.17
77.55
77.23
76.91
58.90
54.02
46.78
29.03
28.40
27.02
23.09
22.10
20.92
14.23
13.92
OH
O
O
S
O
O
N
N
N
180
160
140
120
100
80
60
40
20
ppm

Spectrum

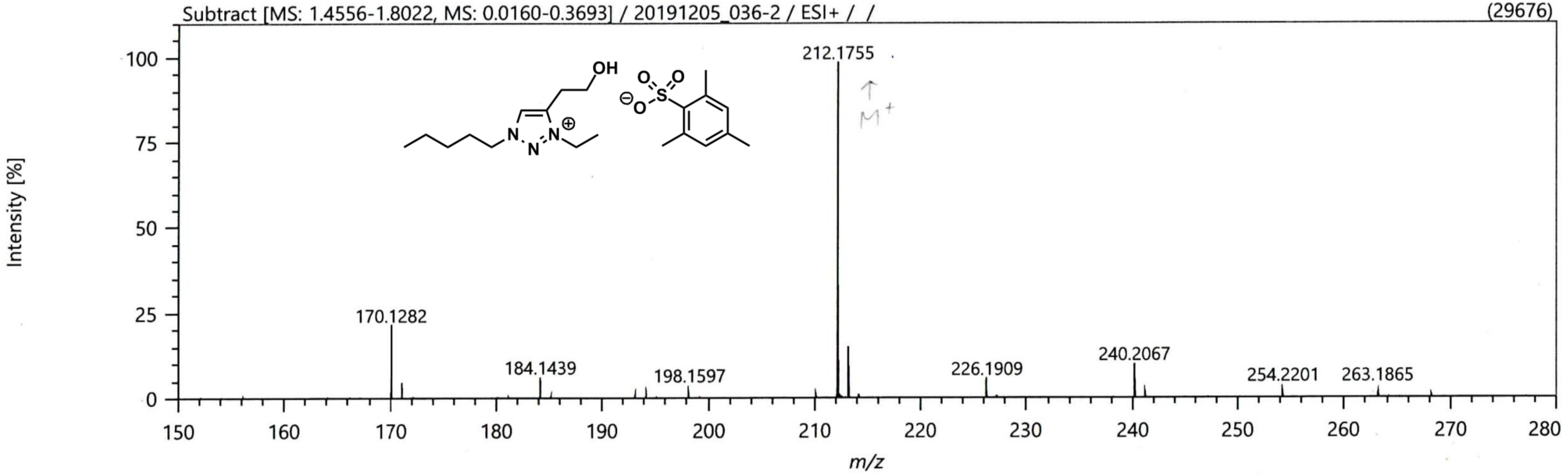

Elemental Composition

Parameters		Elements Set 1:										
Tolerance:	±50.00 ppm	Symbol	C	H	O	Na	N	S	P	Si	F	Cl
Electron:	Odd/Even	Min	0	0	1	0	3	0	0	0	0	0
Charge:	+1	Max	400	1000	1	0	3	0	0	0	0	0
DBE:	-1.5 - 999.0											

Results

Mass	Formula	Calculated Mass	Mass Difference [mDa]	Mass Difference [ppm]	DBE
212.17550	C11 H22 N3 O	212.17574	-0.24	-1.13	2.5

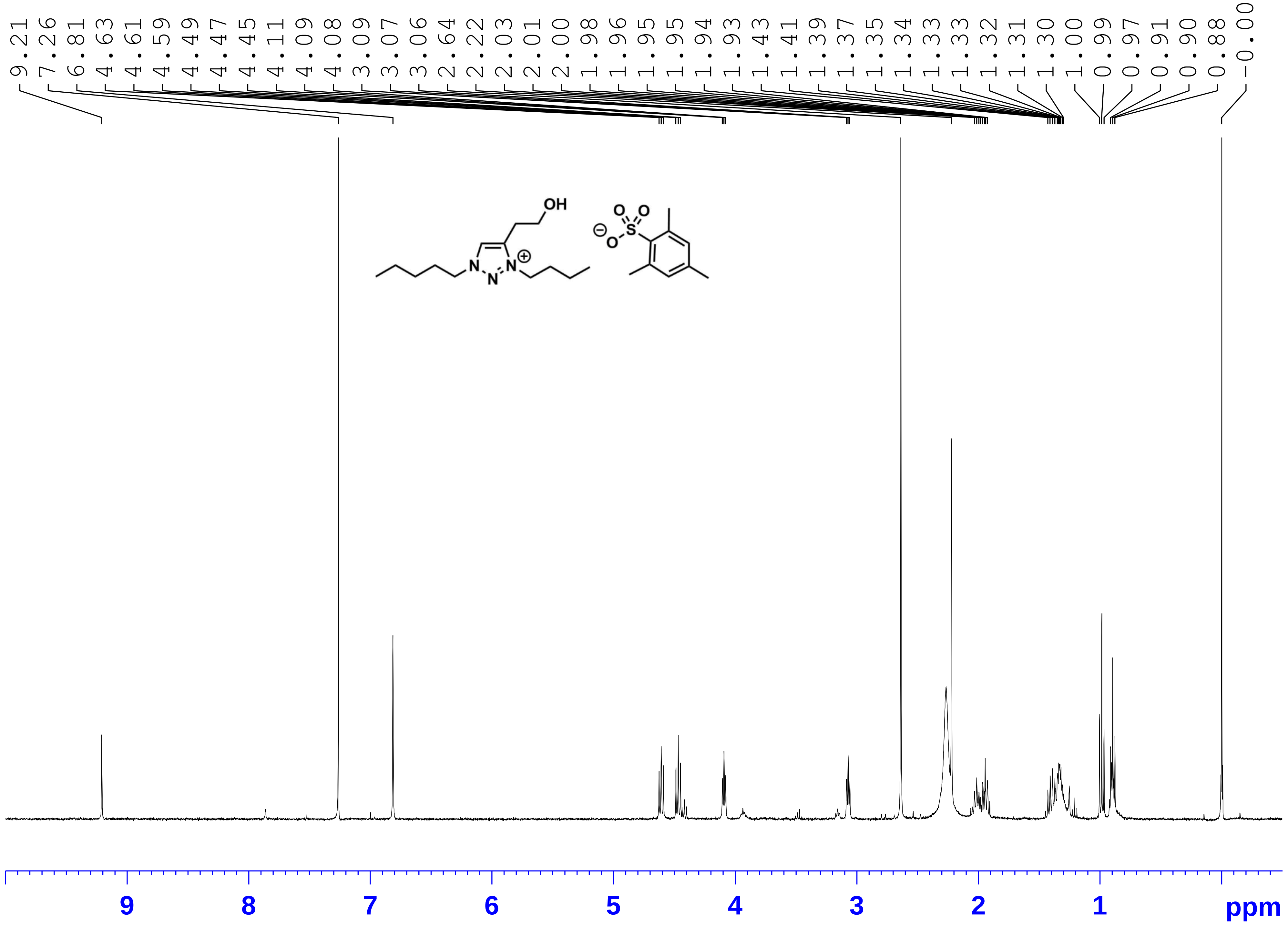
9.21
7.26
6.81
4.63
4.61
4.59
4.49
4.47
4.45
4.11
4.09
4.08
3.09
3.07
3.06
2.64
2.22
2.03
2.01
2.00
1.98
1.96
1.95
1.95
1.94
1.93
1.43
1.41
1.39
1.37
1.35
1.34
1.33
1.33
1.32
1.31
1.30
1.00
0.99
0.97
0.91
0.90
0.88
-0.00
OH
N
N
N
O
O
S
O
9
8
7
6
5
4
3
2
1
ppm

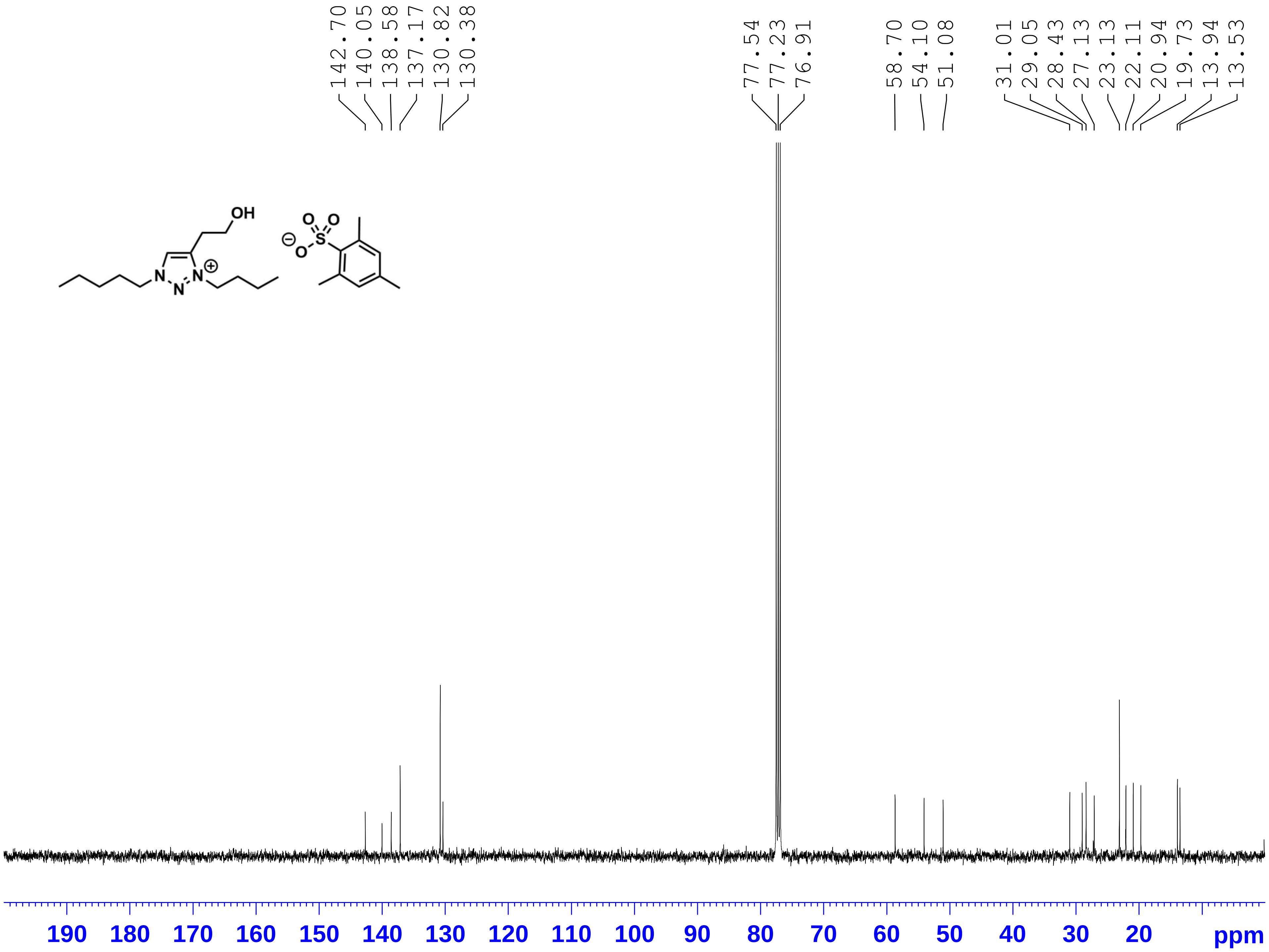

142.70
140.05
138.58
137.17
130.82
130.38
77.54
77.23
76.91
58.70
54.10
51.08
31.01
29.05
28.43
27.13
23.13
22.11
20.94
19.73
13.94
13.53
OH
O
O
S
O
N
N
N
190
180
170
160
150
140
130
120
110
100
90
80
70
60
50
40
30
20
ppm

Spectrum

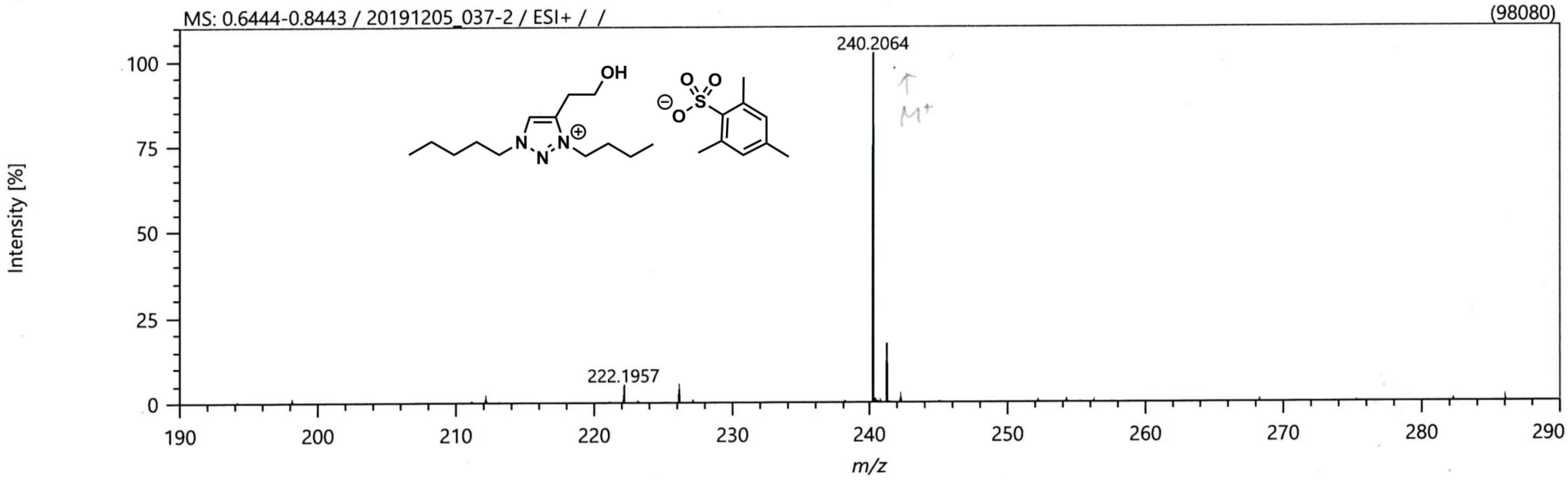

Elemental Composition

Parameters		Elements Set 1:										
Tolerance:	±50.00 ppm	Symbol	C	H	O	Na	N	S	P	Si	F	Cl
Electron:	Odd/Even	Min	0	0	1	0	3	0	0	0	0	0
Charge:	+1	Max	400	1000	1	0	3	0	0	0	0	0
DBE:	-1.5 - 999.0											

Results

Mass	Formula	Calculated Mass	Mass Difference [mDa]	Mass Difference [ppm]	DBE
240.20635	C13 H26 N3 O	240.20704	-0.69	-2.86	2.5

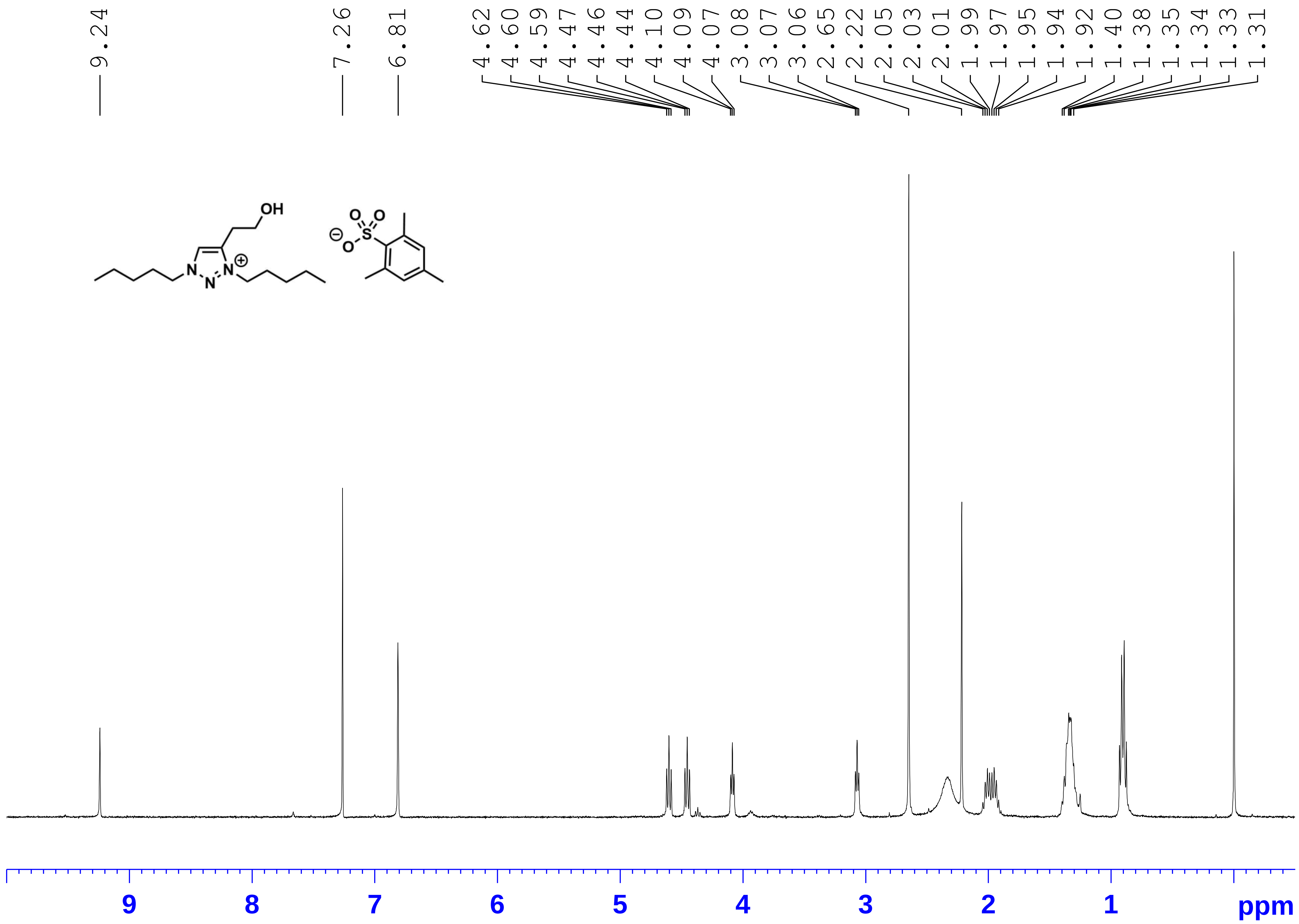
9.24
7.26
6.81
4.62
4.60
4.59
4.47
4.46
4.44
4.10
4.09
4.07
3.08
3.07
3.06
2.65
2.22
2.05
2.03
2.01
1.99
1.97
1.95
1.94
1.92
1.40
1.38
1.35
1.34
1.33
1.31
OH
N
N
N
O
O
S
O
9
8
7
6
5
4
3
2
1
ppm

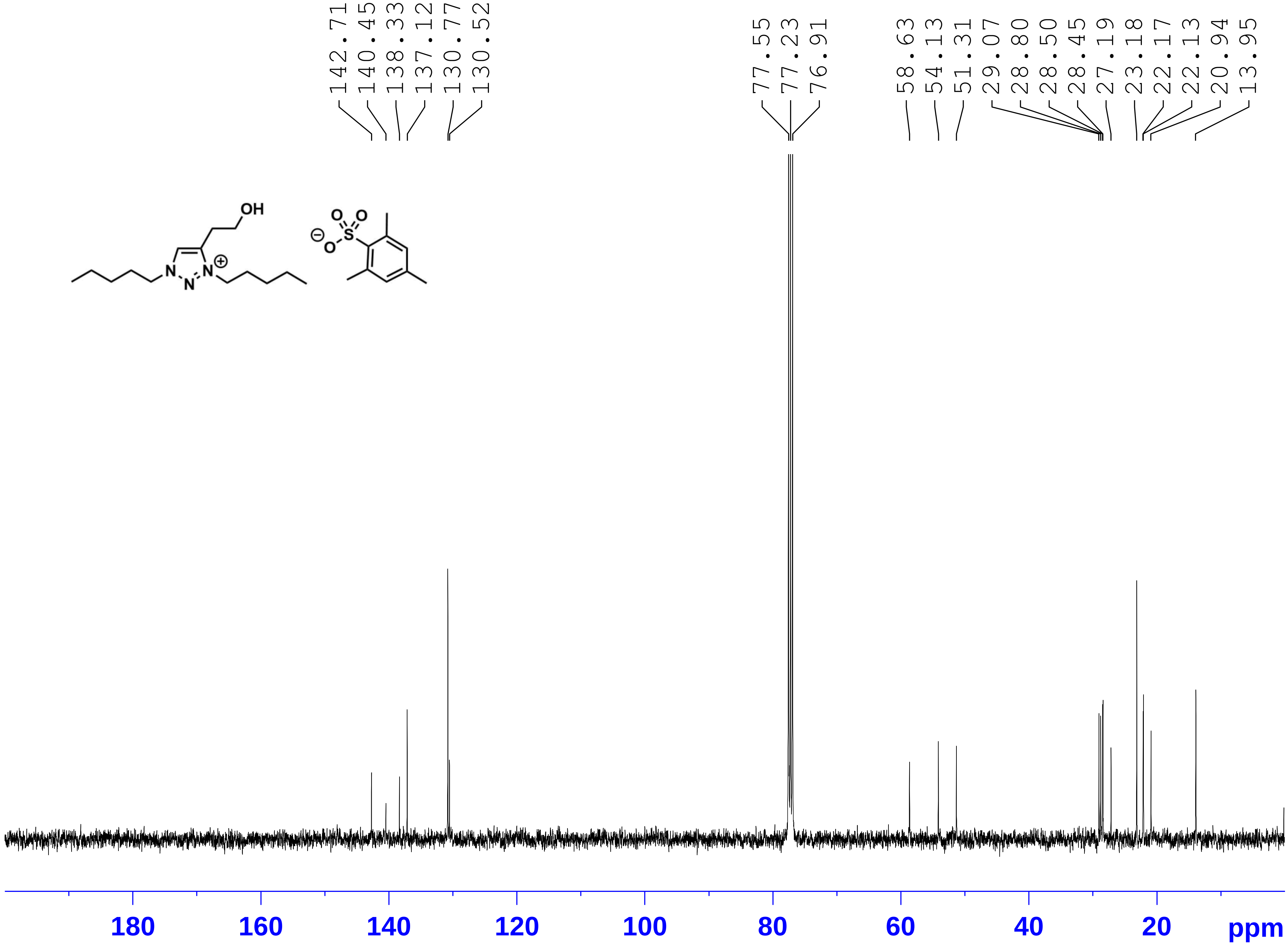
142.71
140.45
138.33
137.12
130.77
130.52
77.55
77.23
76.91
58.63
54.13
51.31
29.07
28.80
28.50
28.45
27.19
23.18
22.17
22.13
20.94
13.95
OH
N
N
N
O
O
S
O
180
160
140
120
100
80
60
40
20
ppm

Spectrum

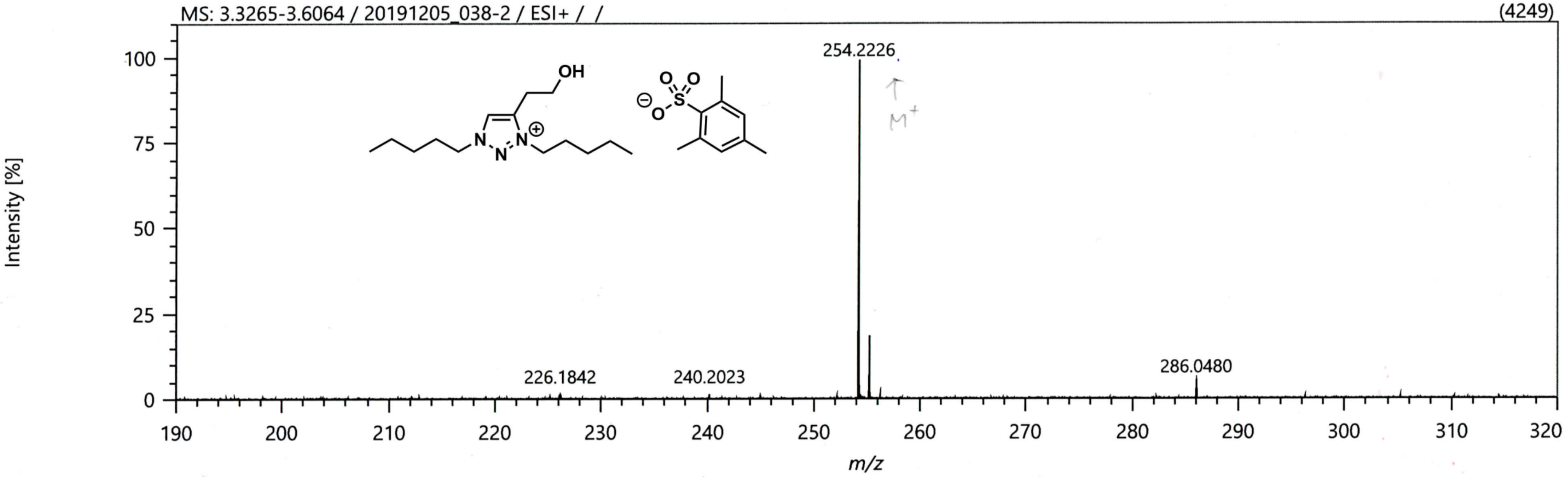

Elemental Composition

Parameters

Tolerance:	±50.00 ppm
Electron:	Odd/Even
Charge:	+1
DBE:	-1.5 - 999.0

Elements Set 1:

Symbol	C	H	O	Na	N	S	P	Si	F	Cl
Min	0	0	1	0	3	0	0	0	0	0
Max	400	1000	1	0	3	0	0	0	0	0

Results

Mass	Formula	Calculated Mass	Mass Difference [mDa]	Mass Difference [ppm]	DBE
254.22265	C14 H28 N3 O	254.22269	-0.04	-0.16	2.5

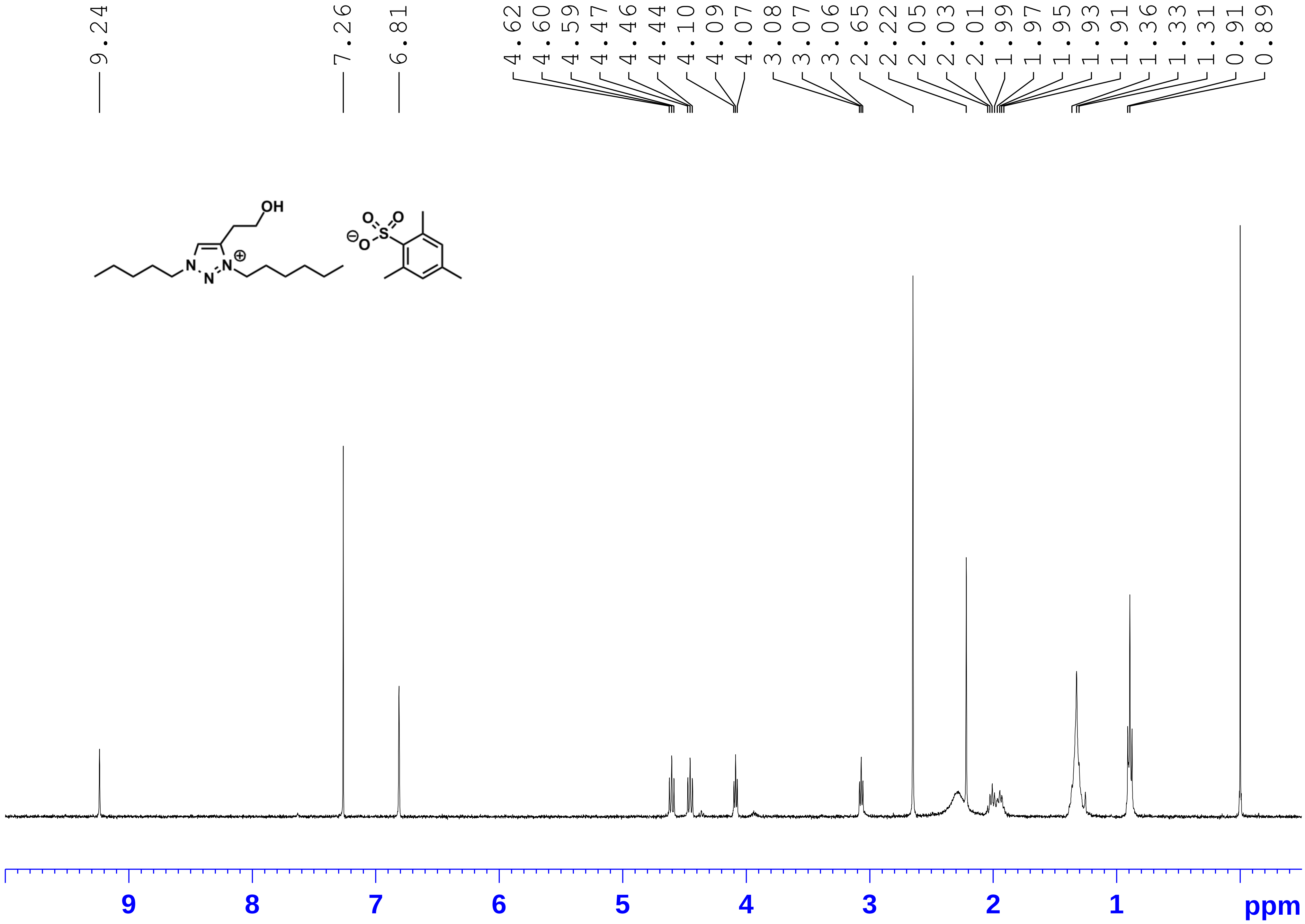
9.24
7.26
6.81
4.62
4.60
4.59
4.47
4.46
4.44
4.10
4.09
4.07
3.08
3.07
3.06
2.65
2.22
2.05
2.03
2.01
1.99
1.97
1.95
1.93
1.91
1.36
1.33
1.31
0.91
0.89
OH
N
N
N
⊕
⊖
O
S
O
O
9
8
7
6
5
4
3
2
1
ppm

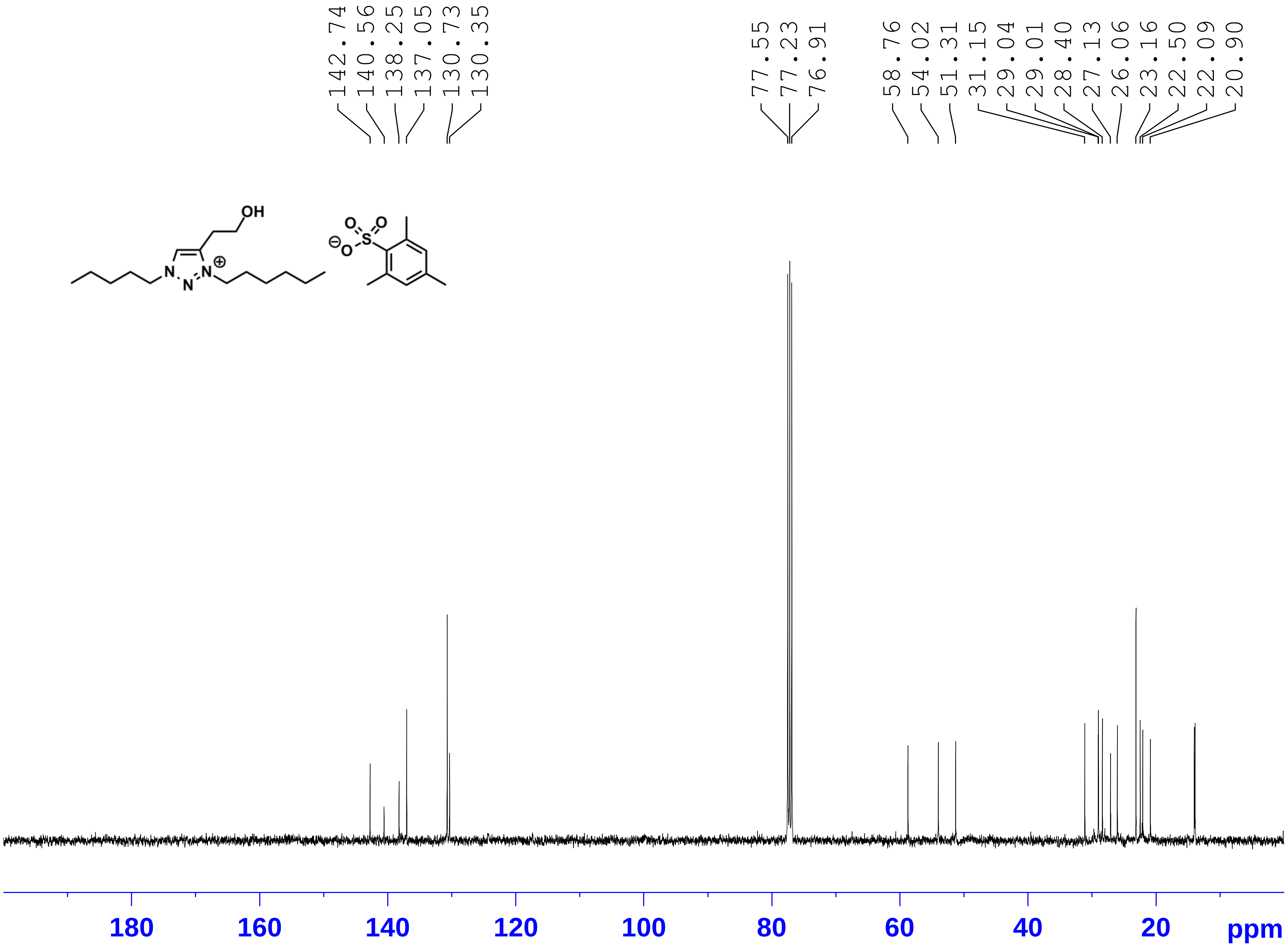

142.74
140.56
138.25
137.05
130.73
130.35
77.55
77.23
76.91
58.76
54.02
51.31
31.15
29.04
29.01
28.40
27.13
26.06
23.16
22.50
22.09
20.90
OH
N
N
N
O
S
O
O
180
160
140
120
100
80
60
40
20
ppm

Spectrum

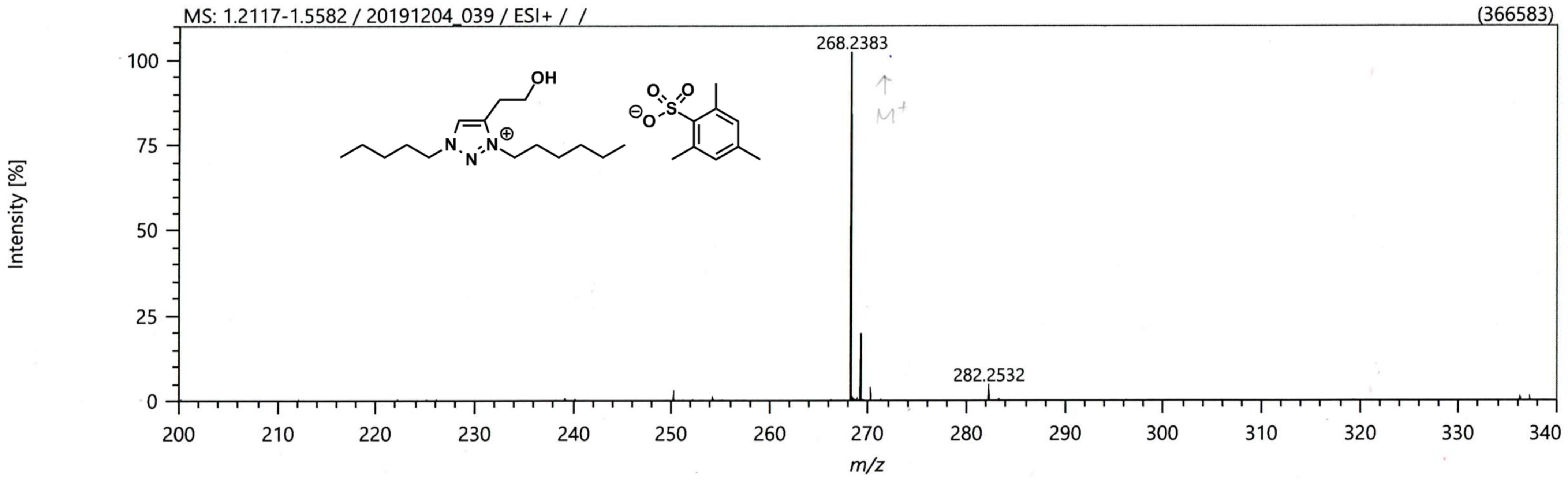

Elemental Composition

Parameters	
Tolerance:	±10.00 ppm
Electron:	Odd/Even
Charge:	+1
DBE:	-1.5 - 999.0

Elements Set 1:

Symbol	C	H	O	Na	N	S	P	Si	F	Cl
Min	0	0	1	0	3	0	0	0	0	0
Max	400	1000	1	0	3	0	0	0	0	0

Results

Mass	Formula	Calculated Mass	Mass Difference [mDa]	Mass Difference [ppm]	DBE
268.23831	C15 H30 N3 O	268.23834	-0.03	-0.13	2.5

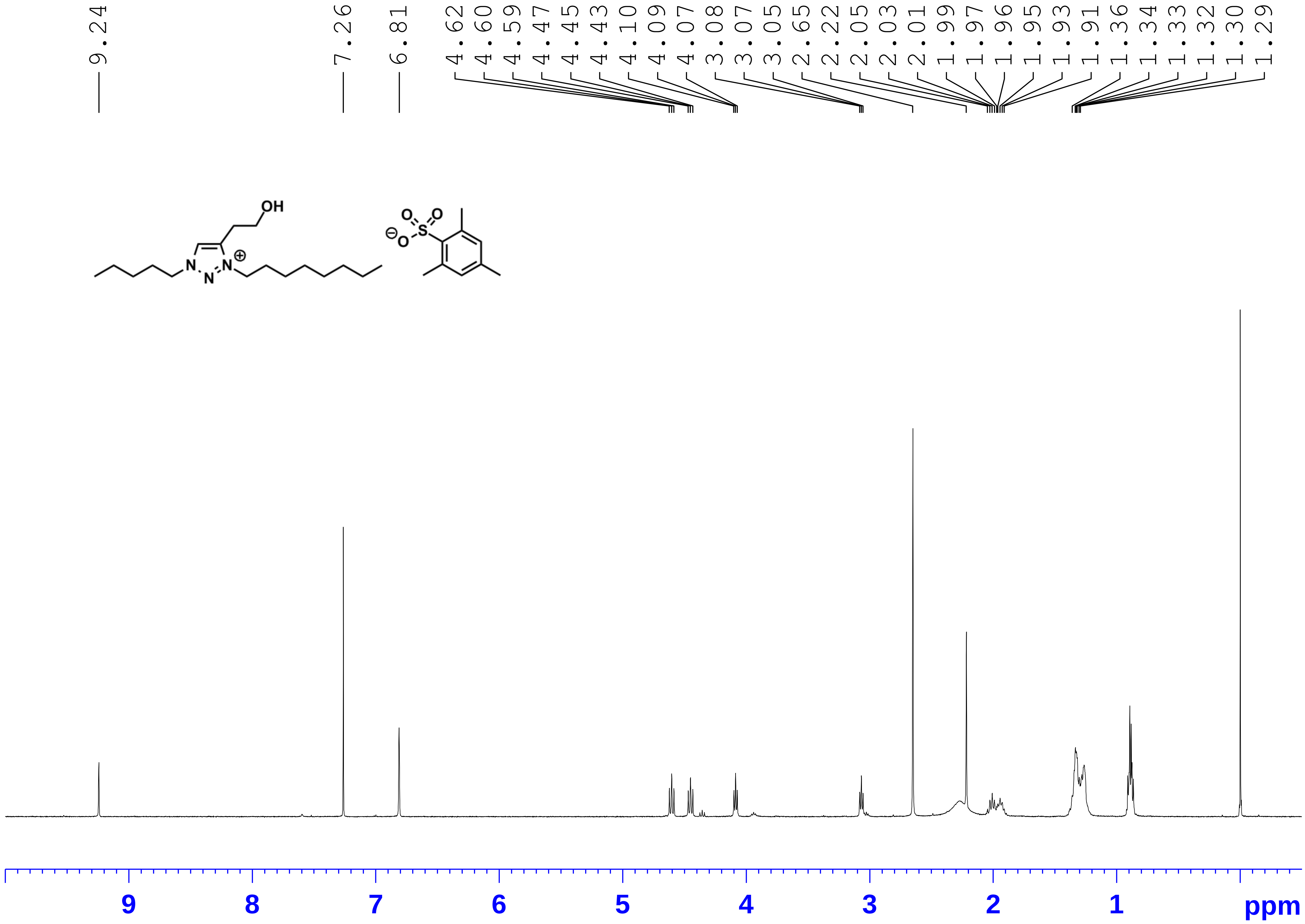
9.24
7.26
6.81
4.62
4.60
4.59
4.47
4.45
4.43
4.10
4.09
4.07
3.08
3.07
3.05
2.65
2.22
2.05
2.03
2.01
1.99
1.97
1.96
1.95
1.93
1.91
1.36
1.34
1.33
1.32
1.30
1.29
OH
N
N
N
O
O
S
O
9
8
7
6
5
4
3
2
1
ppm

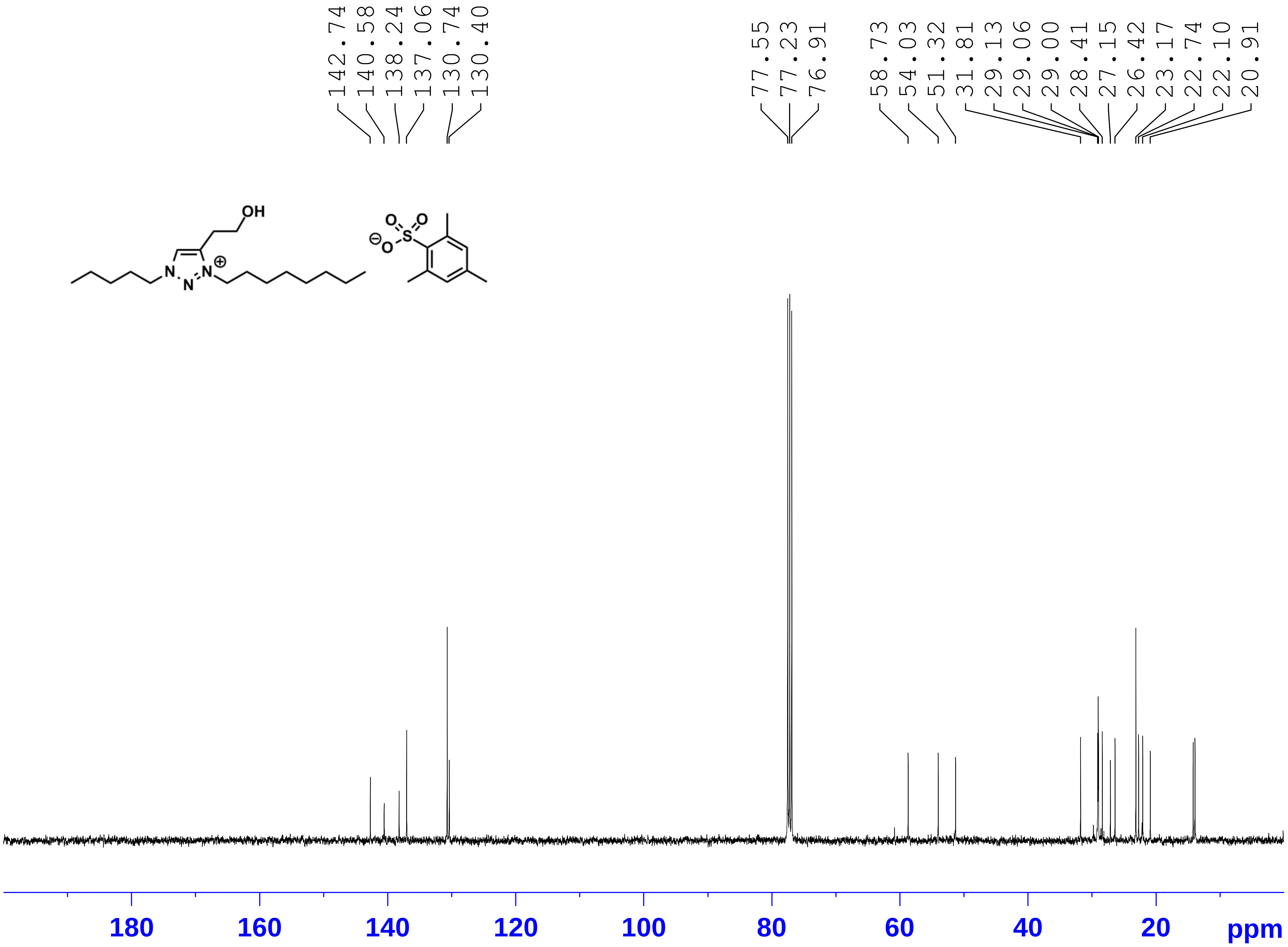
142.74
140.58
138.24
137.06
130.74
130.40
77.55
77.23
76.91
58.73
54.03
51.32
31.81
29.13
29.06
29.00
28.41
27.15
26.42
23.17
22.74
22.10
20.91
OH
N
N
N
S
O
O
O
180
160
140
120
100
80
60
40
20
ppm

Spectrum

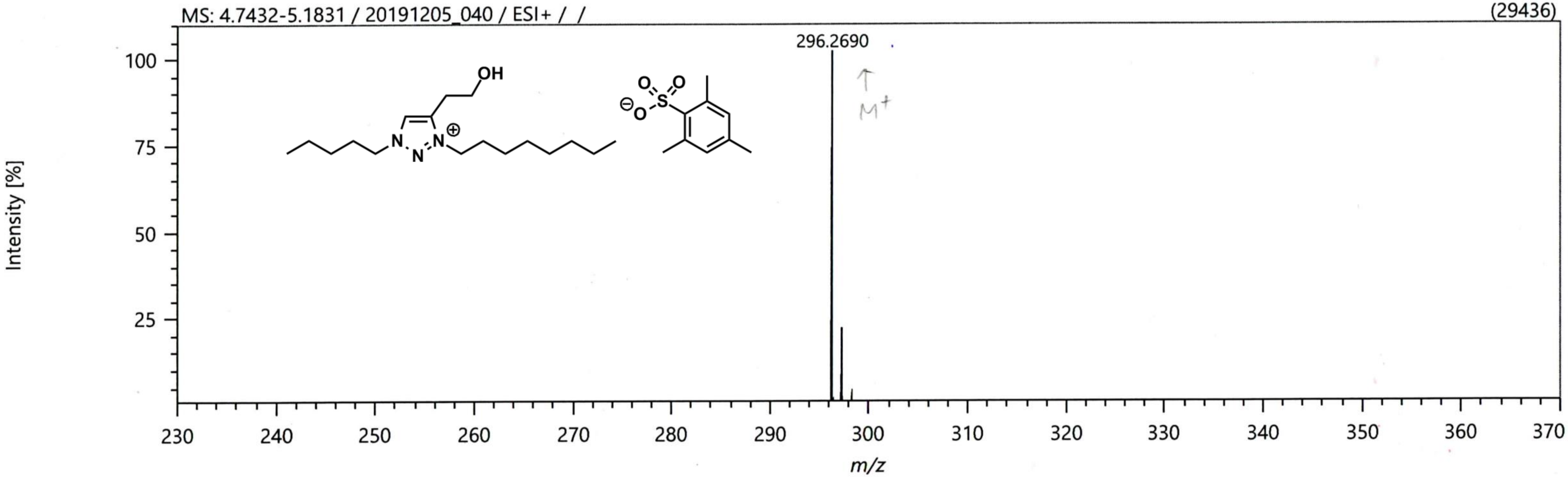

Elemental Composition

Parameters

Tolerance:	±50.00 ppm
Electron:	Odd/Even
Charge:	+1
DBE:	-1.5 - 999.0

Elements Set 1:

Symbol	C	H	O	Na	N	S	P	Si	F	Cl
Min	0	0	1	0	3	0	0	0	0	0
Max	400	1000	1	0	3	0	0	0	0	0

Results

Mass	Formula	Calculated Mass	Mass Difference [mDa]	Mass Difference [ppm]	DBE
296.26897	C17 H34 N3 O	296.26964	-0.67	-2.25	2.5

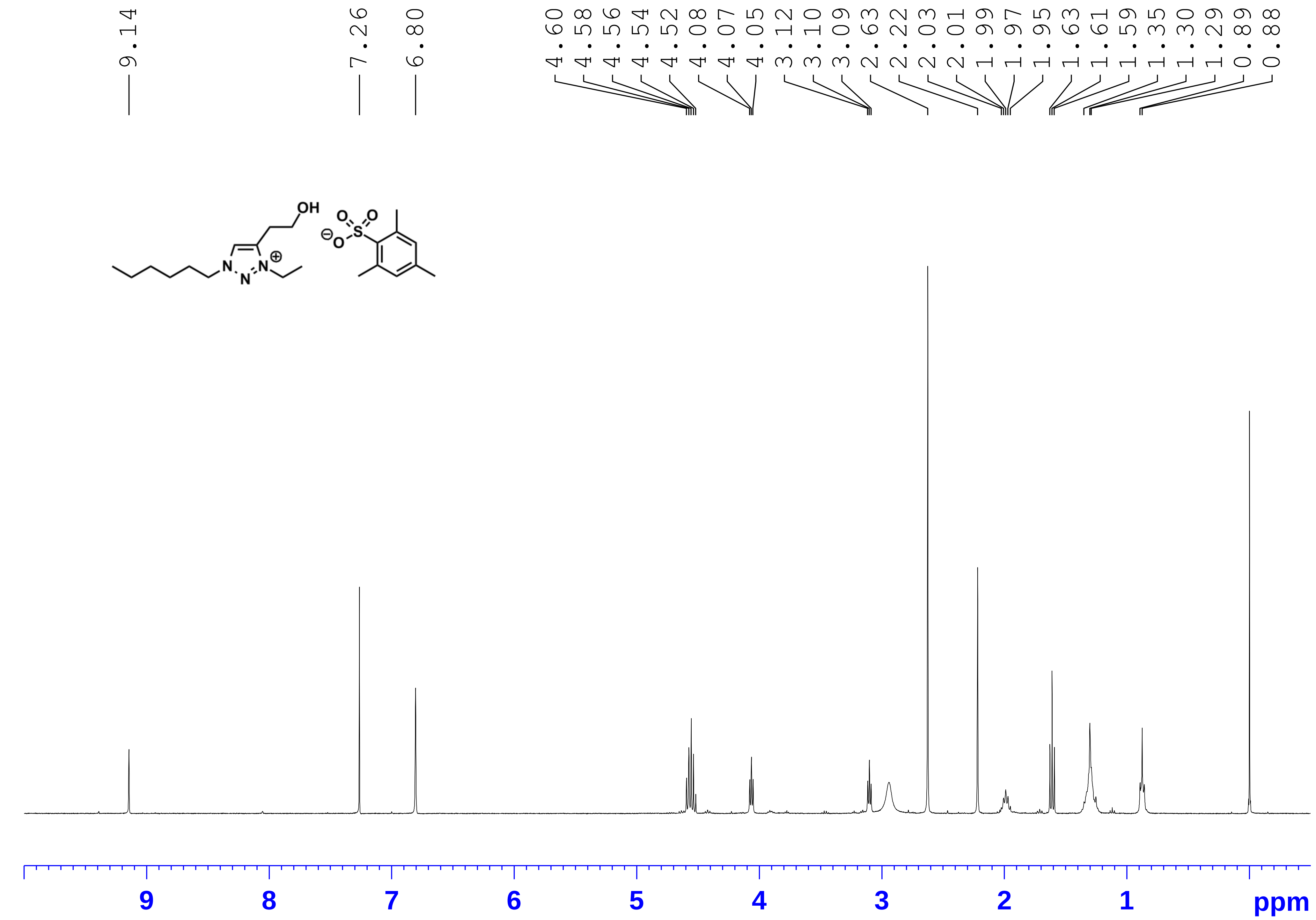
9.14
7.26
6.80
4.60
4.58
4.56
4.54
4.52
4.08
4.07
4.05
3.12
3.10
3.09
2.63
2.22
2.03
2.01
1.99
1.97
1.95
1.63
1.61
1.59
1.35
1.30
1.29
0.89
0.88
OH
N
N
N
S
O
O
O
9
8
7
6
5
4
3
2
1
ppm

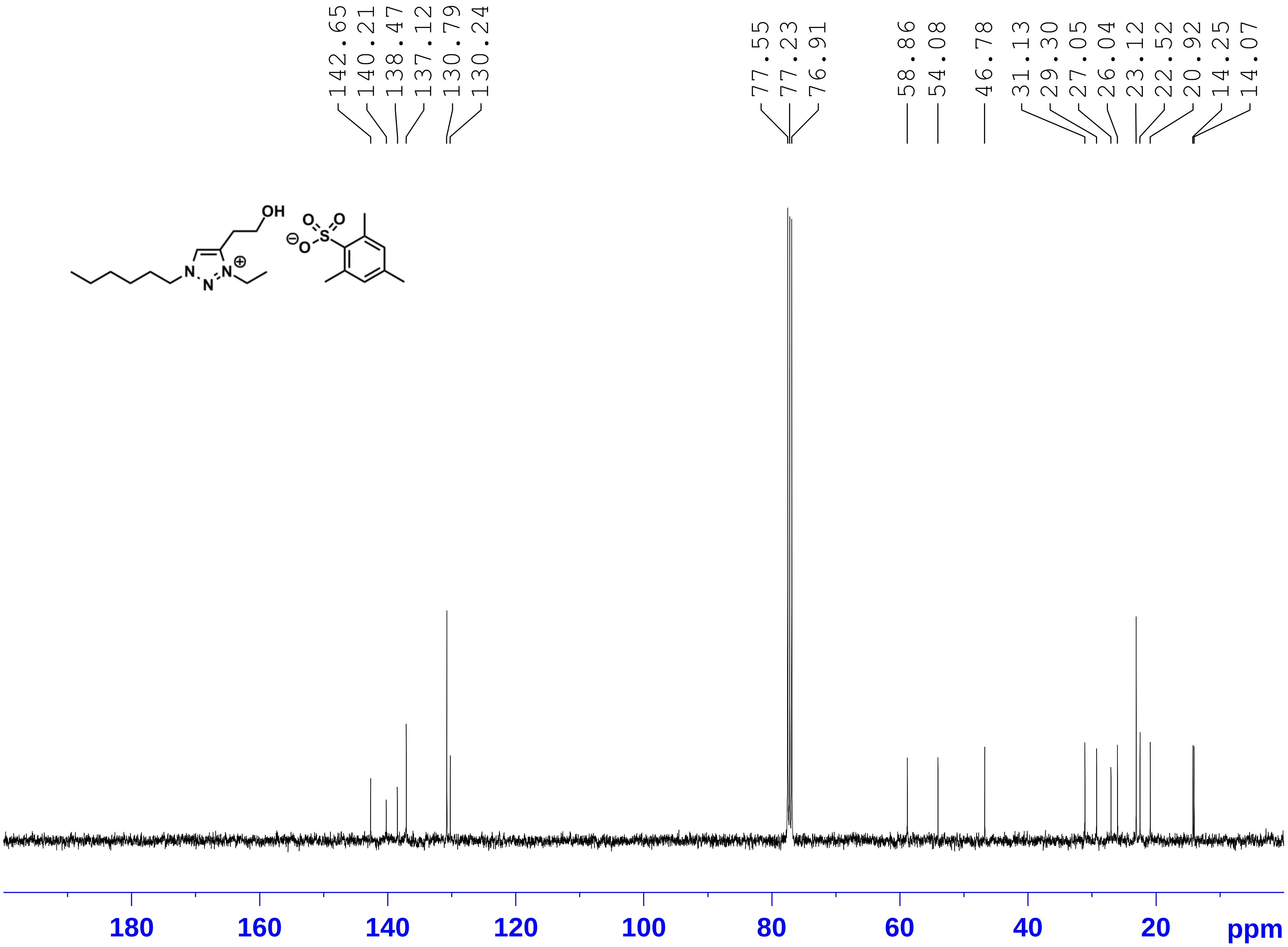
142.65
140.21
138.47
137.12
130.79
130.24
77.55
77.23
76.91
58.86
54.08
46.78
31.13
29.30
27.05
26.04
23.12
22.52
20.92
14.25
14.07
OH
O
S
O
O
N
N
N
180
160
140
120
100
80
60
40
20
ppm

Spectrum

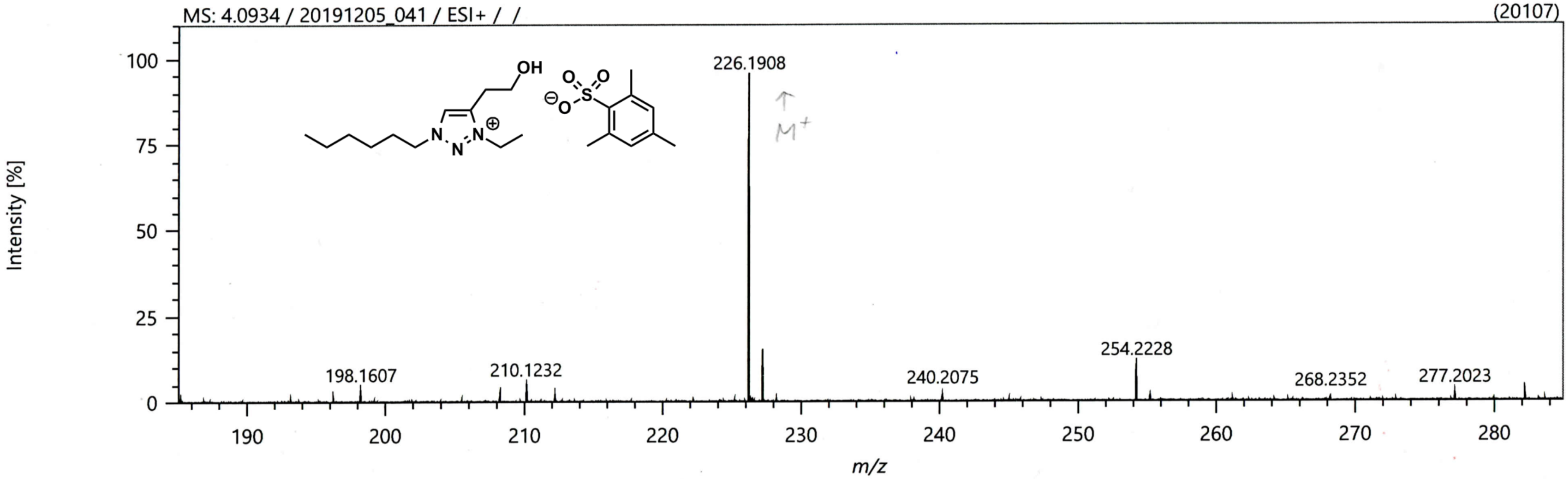

Elemental Composition

Parameters

Tolerance:	±50.00 ppm
Electron:	Odd/Even
Charge:	+1
DBE:	-1.5 - 999.0

Elements Set 1:

Symbol	C	H	O	Na	N	S	P	Si	F	Cl
Min	0	0	1	0	3	0	0	0	0	0
Max	400	1000	1	0	3	0	0	0	0	0

Results

Mass	Formula	Calculated Mass	Mass Difference [mDa]	Mass Difference [ppm]	DBE
226.19076	C12 H24 N3 O	226.19139	-0.63	-2.79	2.5

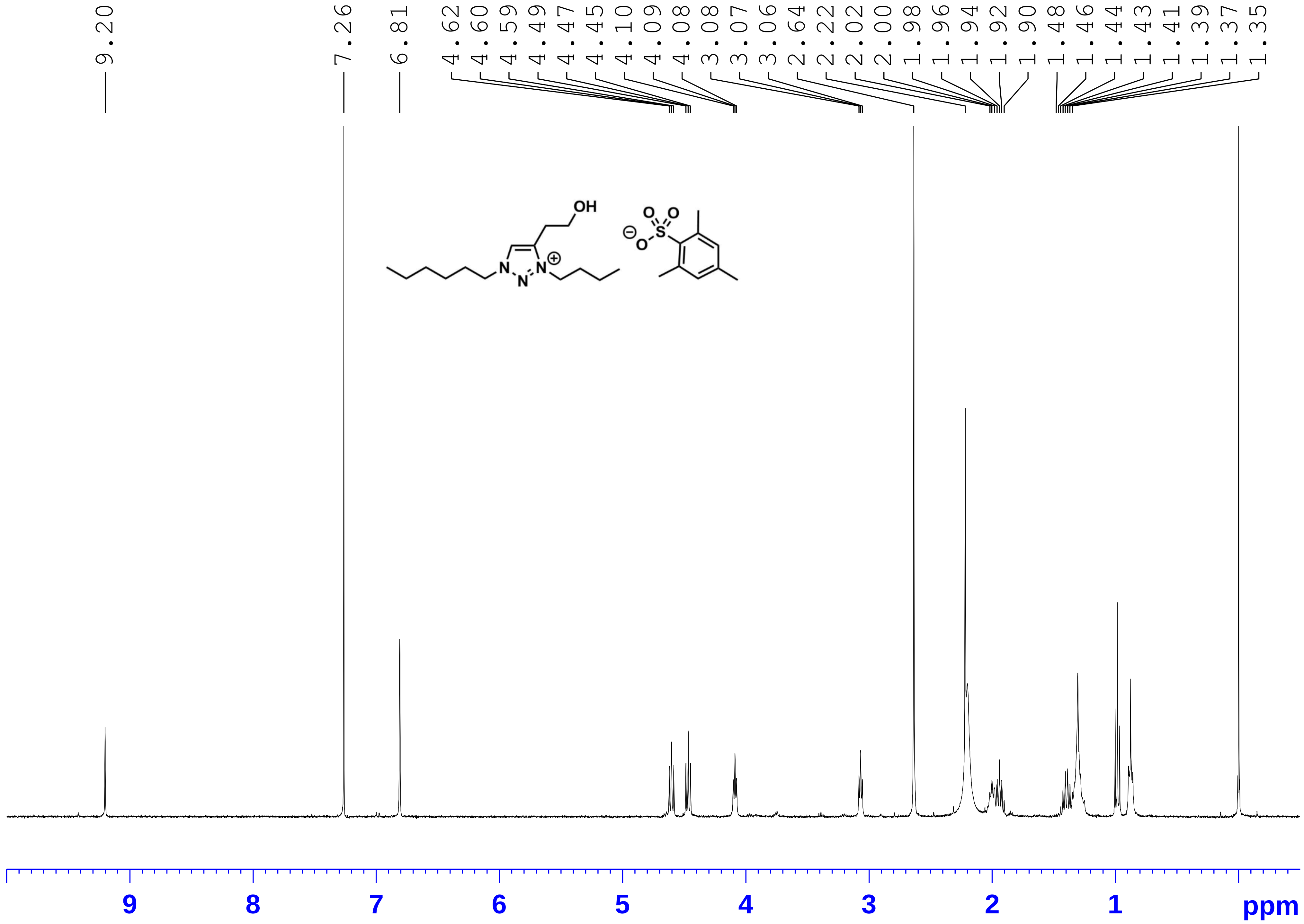
9.20
7.26
6.81
4.62
4.60
4.59
4.49
4.47
4.45
4.10
4.09
4.08
3.08
3.07
3.06
2.64
2.22
2.02
2.00
1.98
1.96
1.94
1.92
1.90
1.48
1.46
1.44
1.43
1.41
1.39
1.37
1.35
OH
N
N
N
O
O
S
O
9
8
7
6
5
4
3
2
1
ppm

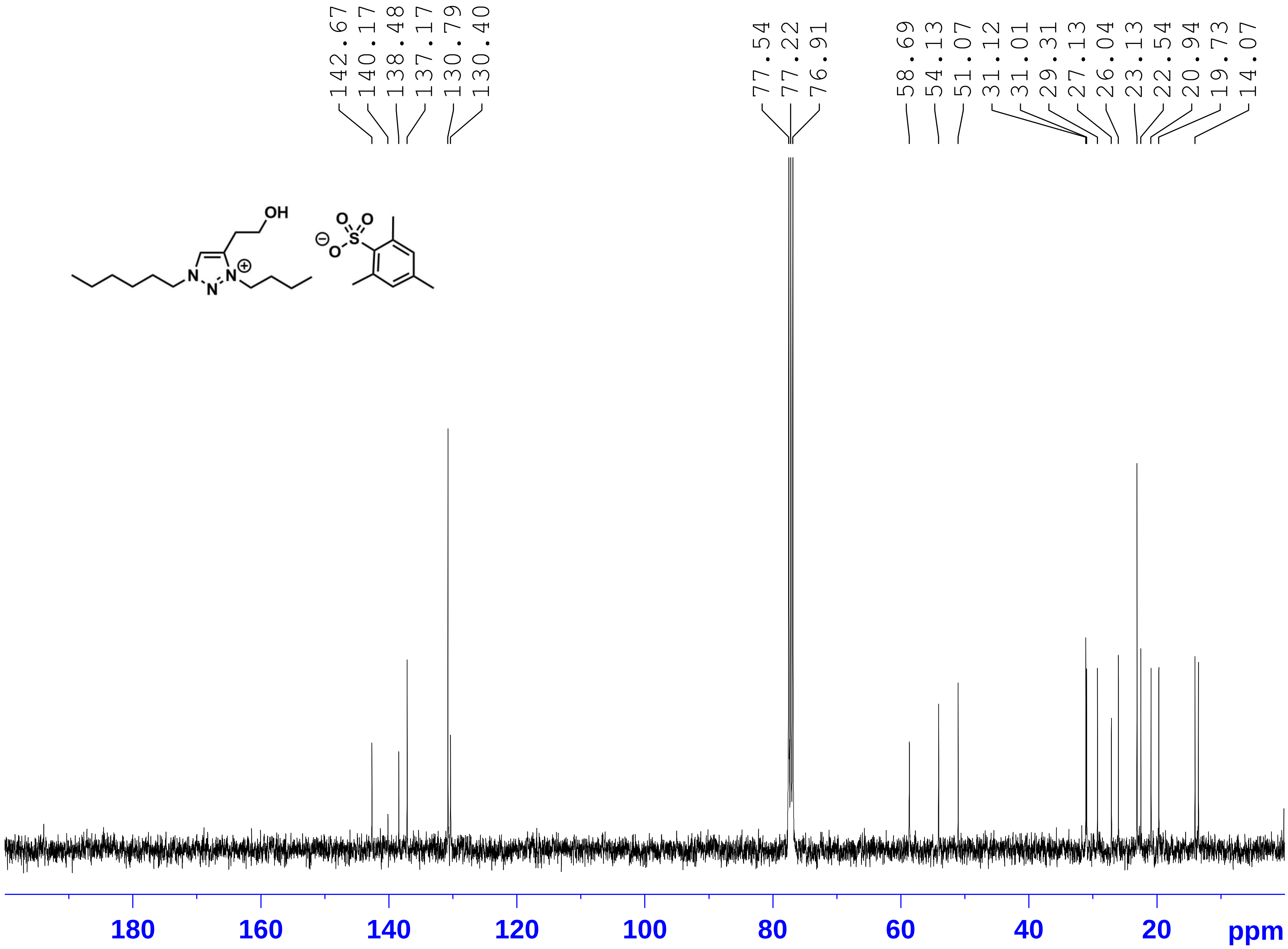

142.67
140.17
138.48
137.17
130.79
130.40
77.54
77.22
76.91
58.69
54.13
51.07
31.12
31.01
29.31
27.13
26.04
23.13
22.54
20.94
19.73
14.07
OH
O
O
S
O
N
N
N
180
160
140
120
100
80
60
40
20
ppm

Spectrum

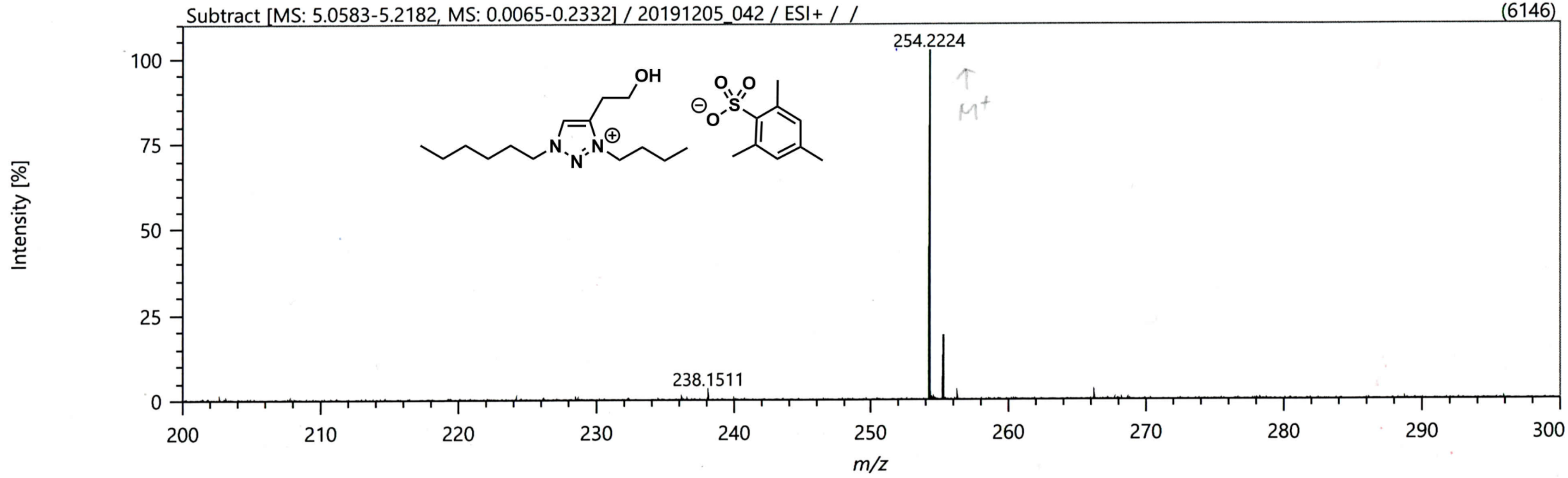

Elemental Composition

Parameters

Tolerance:	±50.00 ppm
Electron:	Odd/Even
Charge:	+1
DBE:	-1.5 - 999.0

Elements Set 1:

Symbol	C	H	O	Na	N	S	P	Si	F	Cl
Min	0	0	1	0	3	0	0	0	0	0
Max	400	1000	1	0	3	0	0	0	0	0

Results

Mass	Formula	Calculated Mass	Mass Difference [mDa]	Mass Difference [ppm]	DBE
254.22237	C14 H28 N3 O	254.22269	-0.32	-1.27	2.5

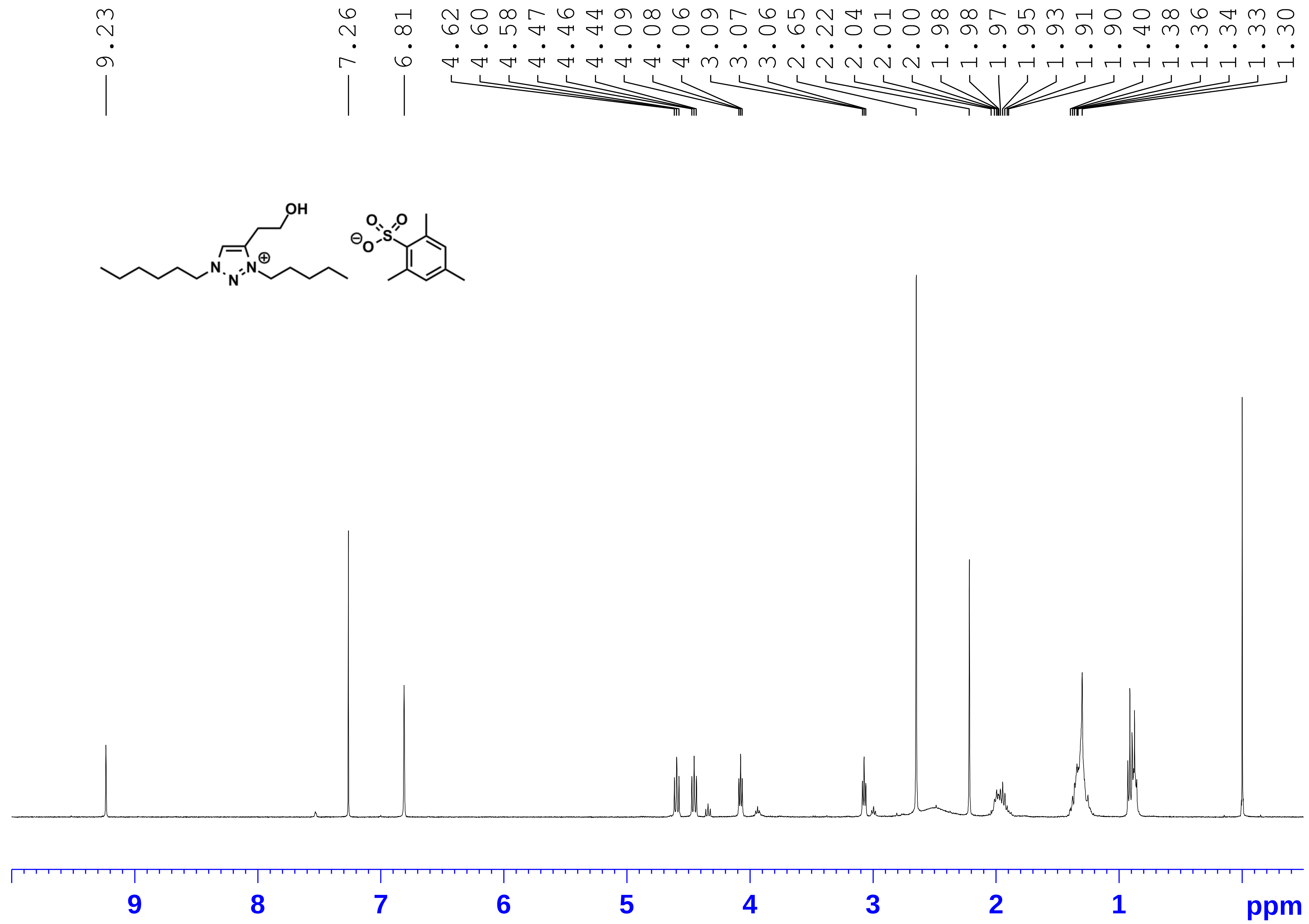
9.23
7.26
6.81
4.62
4.60
4.58
4.47
4.46
4.44
4.09
4.08
4.06
3.09
3.07
3.06
2.65
2.22
2.04
2.01
2.00
1.98
1.98
1.97
1.95
1.93
1.91
1.90
1.40
1.38
1.36
1.34
1.33
1.30
OH
O
S
O
O
N
N
N
9
8
7
6
5
4
3
2
1
ppm

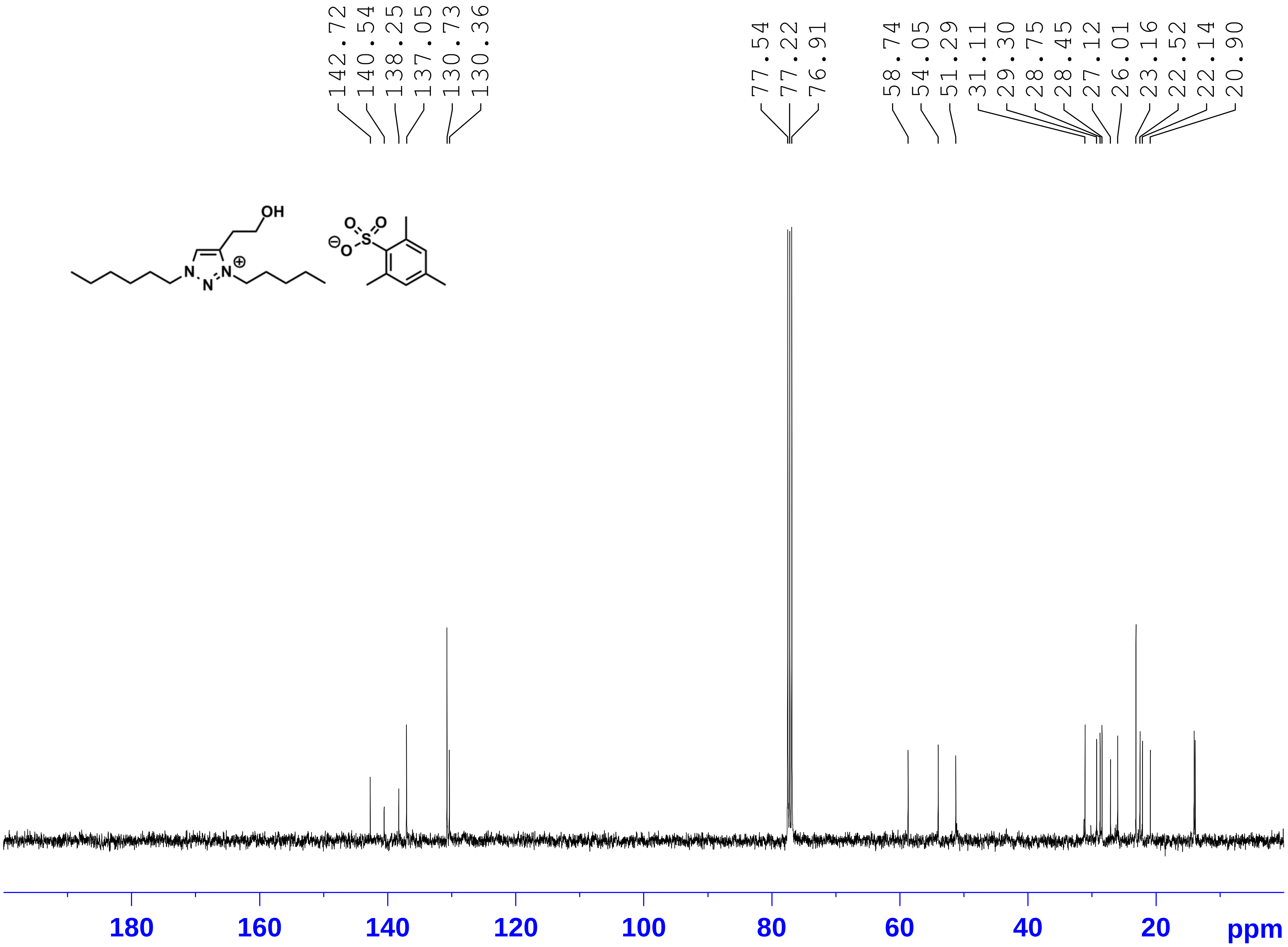
142.72
140.54
138.25
137.05
130.73
130.36
77.54
77.22
76.91
58.74
54.05
51.29
31.11
29.30
28.75
28.45
27.12
26.01
23.16
22.52
22.14
20.90
OH
O
O
S
O
N
N
N
180
160
140
120
100
80
60
40
20
ppm

Spectrum

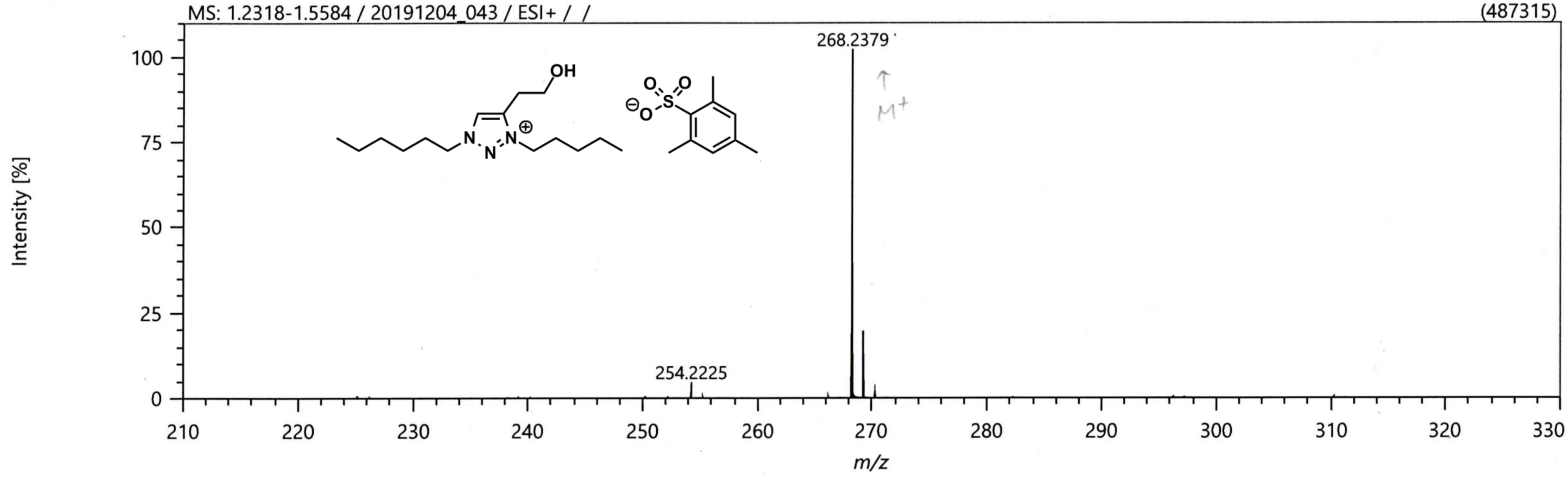

Elemental Composition

Parameters

Tolerance:	±10.00 ppm
Electron:	Odd/Even
Charge:	+1
DBE:	-1.5 - 999.0

Elements Set 1:

Symbol	C	H	O	Na	N	S	P	Si	F	Cl
Min	0	0	1	0	3	0	0	0	0	0
Max	400	1000	1	0	3	0	0	0	0	0

Results

Mass	Formula	Calculated Mass	Mass Difference [mDa]	Mass Difference [ppm]	DBE
268.23793	C15 H30 N3 O	268.23834	-0.41	-1.53	2.5

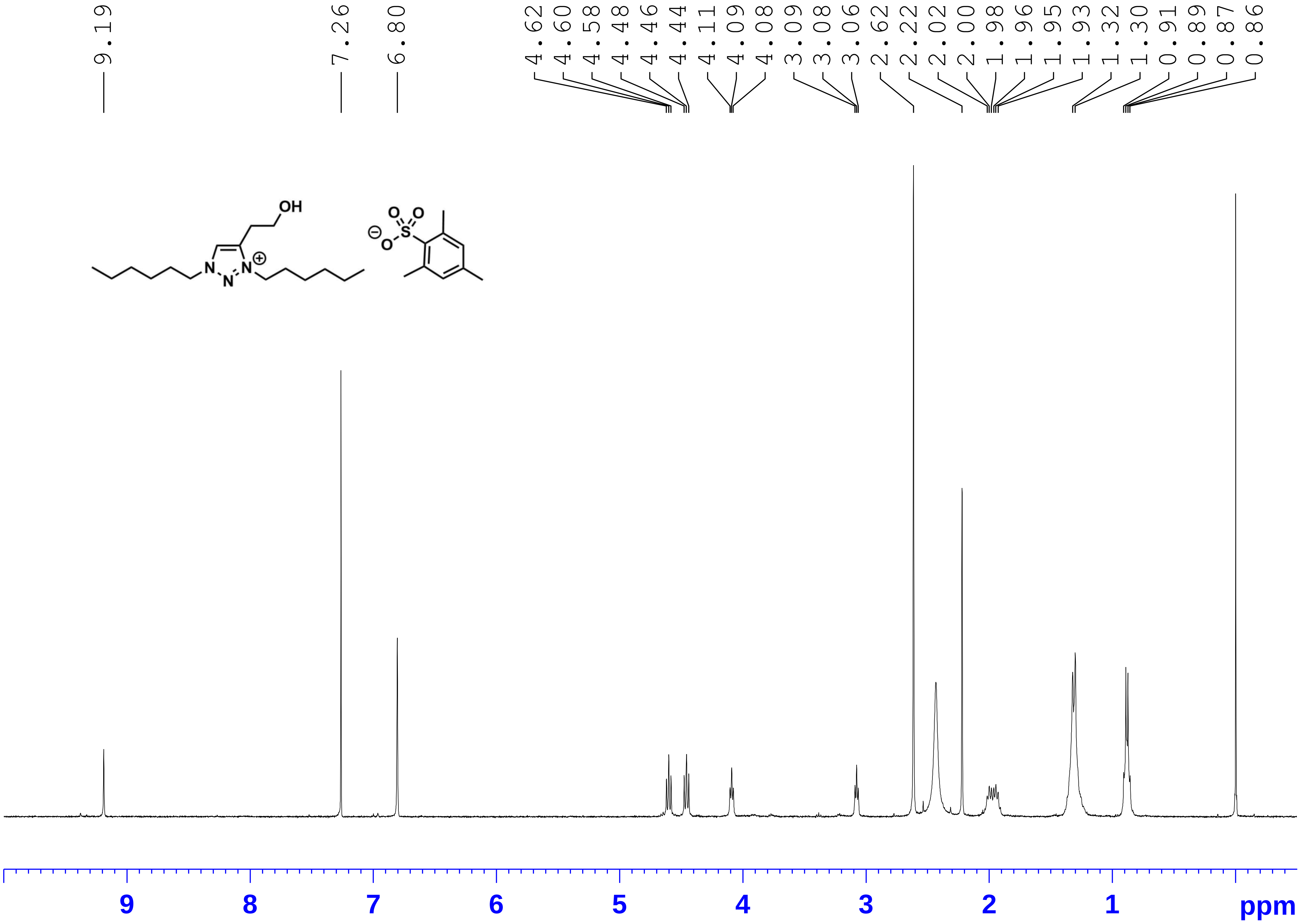

9.19
7.26
6.80
4.62
4.60
4.58
4.48
4.46
4.44
4.11
4.09
4.08
3.09
3.08
3.06
2.62
2.22
2.02
2.00
1.98
1.96
1.95
1.93
1.32
1.30
0.91
0.89
0.87
0.86
OH
N
N
N
O
O
S
O
9
8
7
6
5
4
3
2
1
ppm

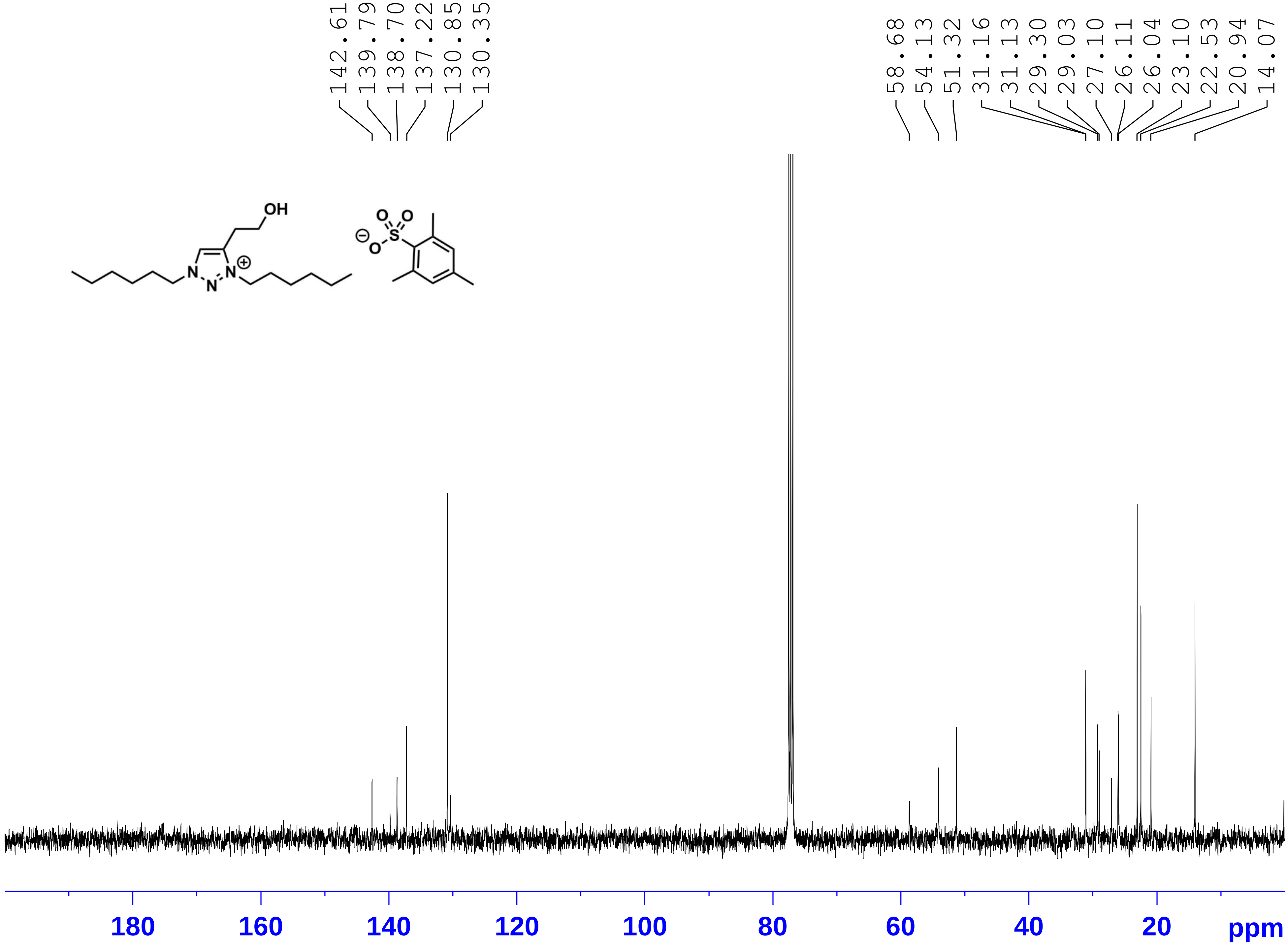

142.61
139.79
138.70
137.22
130.85
130.35
58.68
54.13
51.32
31.16
31.13
29.30
29.03
27.10
26.11
26.04
23.10
22.53
20.94
14.07
OH
N
N
N
S
O
O
O
180
160
140
120
100
80
60
40
20
ppm

Spectrum

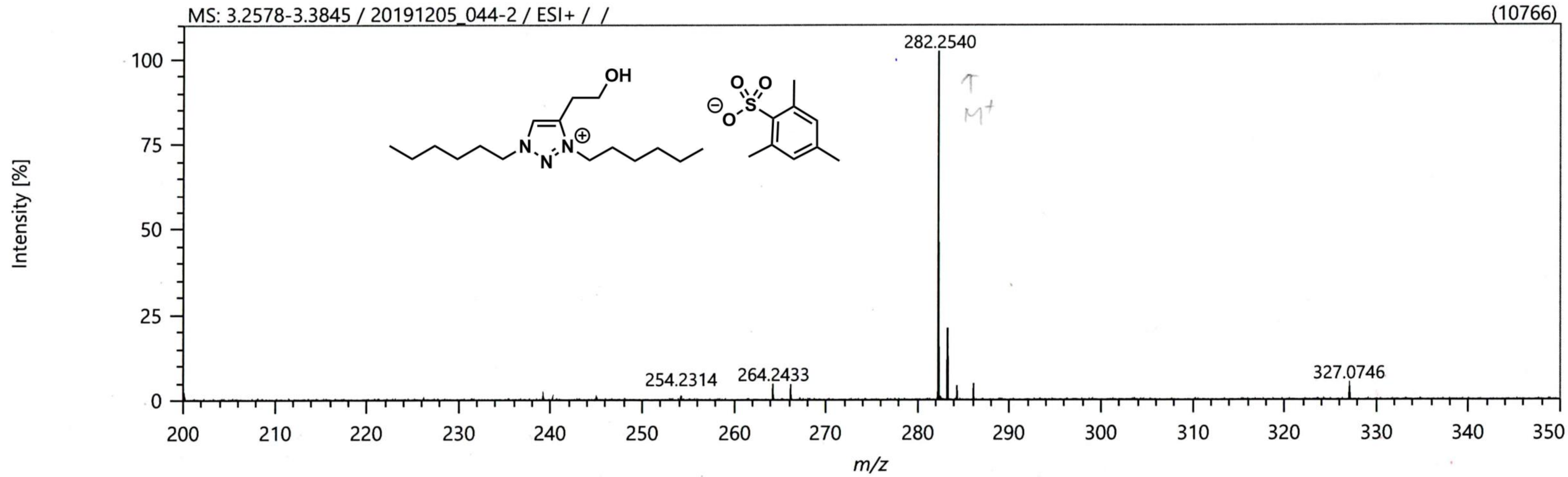

Elemental Composition

Parameters

Tolerance:	±50.00 ppm
Electron:	Odd/Even
Charge:	+1
DBE:	-1.5 - 999.0

Elements Set 1:

Symbol	C	H	O	Na	N	S	P	Si	F	Cl
Min	0	0	1	0	3	0	0	0	0	0
Max	400	1000	1	0	3	0	0	0	0	0

Results

Mass	Formula	Calculated Mass	Mass Difference [mDa]	Mass Difference [ppm]	DBE
282.25402	C16 H32 N3 O	282.25399	0.03	0.12	2.5

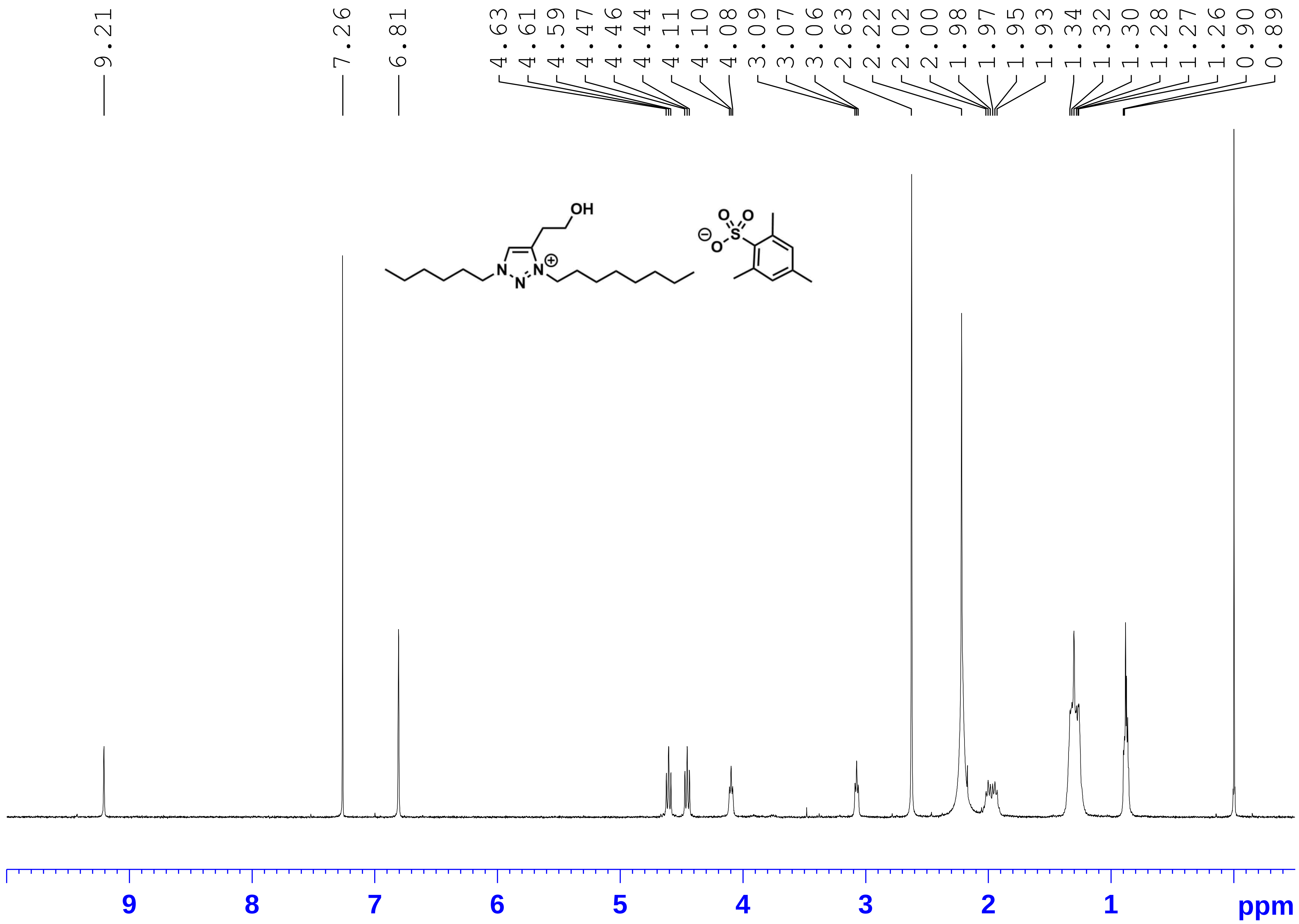
9.21
7.26
6.81
4.63
4.61
4.59
4.47
4.46
4.44
4.11
4.10
4.08
3.09
3.07
3.06
2.63
2.22
2.02
2.00
1.98
1.97
1.95
1.93
1.34
1.32
1.30
1.28
1.27
1.26
0.90
0.89
OH
N
N
N
O
O
S
O
O
9
8
7
6
5
4
3
2
1
ppm

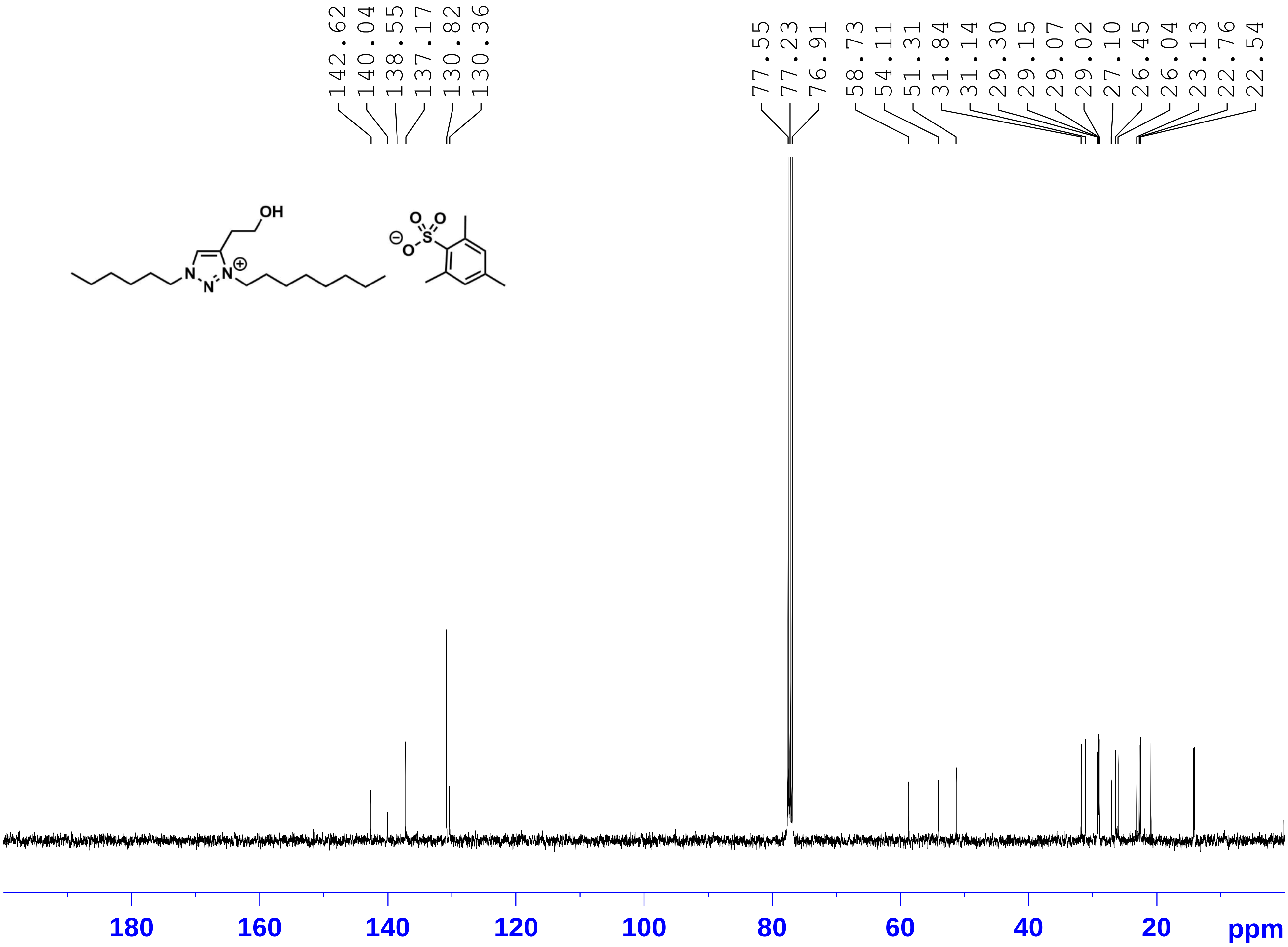
142.62
140.04
138.55
137.17
130.82
130.36
77.55
77.23
76.91
58.73
54.11
51.31
31.84
31.14
29.30
29.15
29.07
29.02
27.10
26.45
26.04
23.13
22.76
22.54
OH
180
160
140
120
100
80
60
40
20
ppm

Spectrum

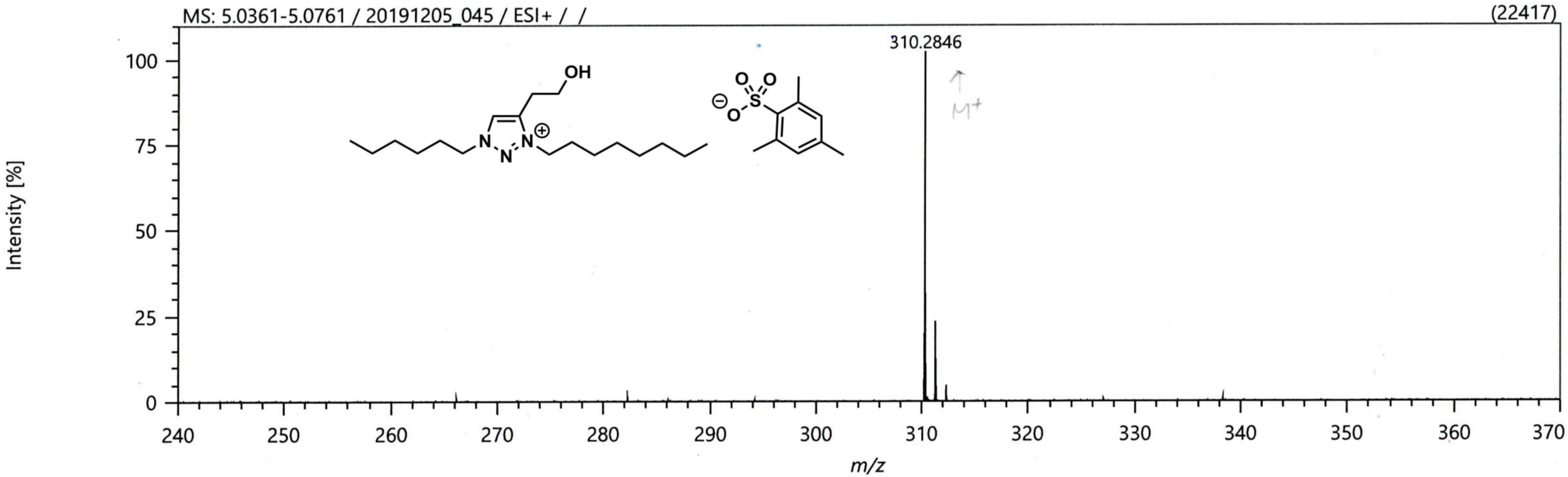

Elemental Composition

Parameters

Tolerance:	±50.00 ppm
Electron:	Odd/Even
Charge:	+1
DBE:	-1.5 - 999.0

Elements Set 1:

Symbol	C	H	O	Na	N	S	P	Si	F	Cl
Min	0	0	1	0	3	0	0	0	0	0
Max	400	1000	1	0	3	0	0	0	0	0

Results

Mass	Formula	Calculated Mass	Mass Difference [mDa]	Mass Difference [ppm]	DBE
310.28455	C18 H36 N3 O	310.28529	-0.74	-2.38	2.5

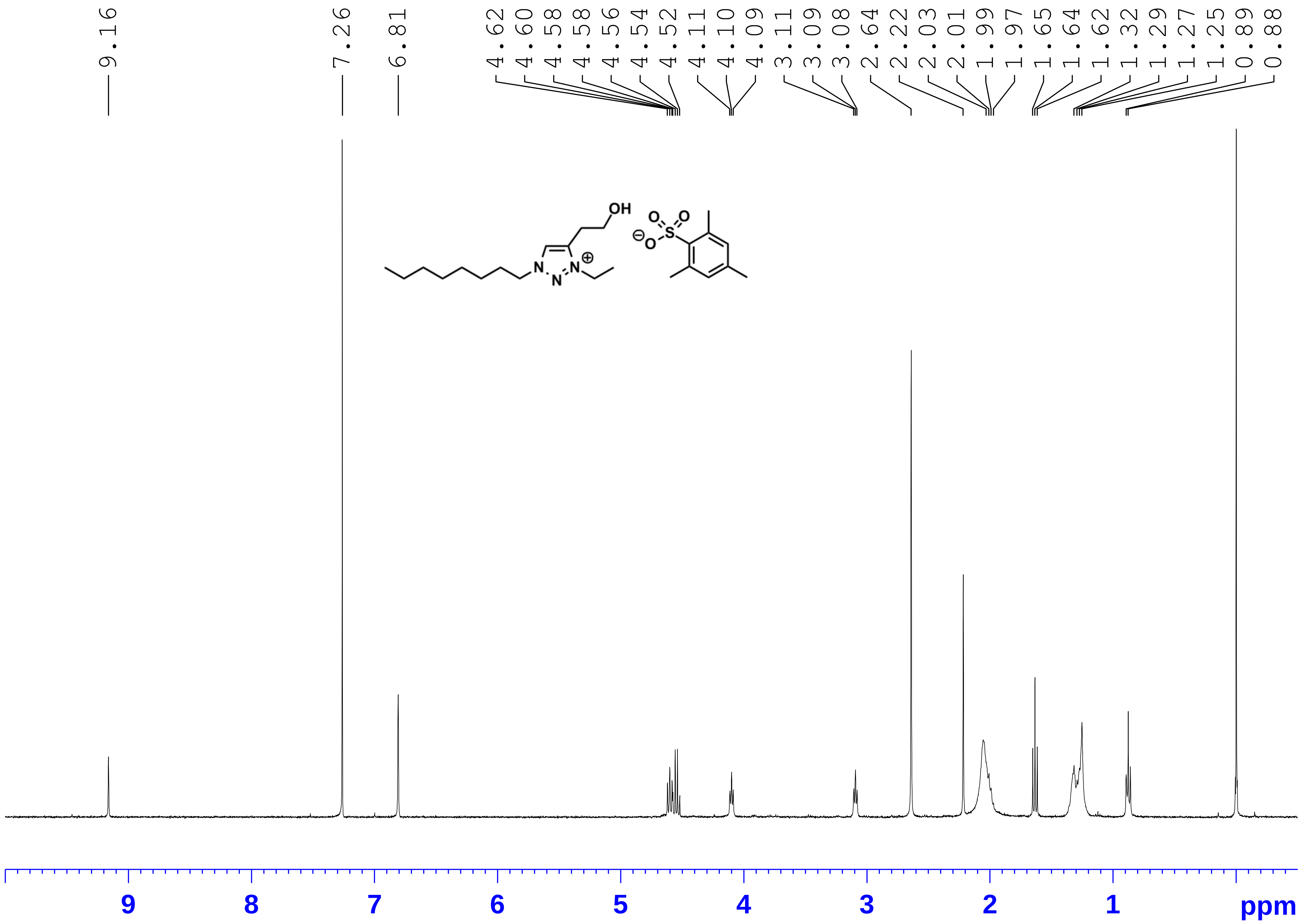

9.16
7.26
6.81
4.62
4.60
4.58
4.58
4.56
4.54
4.52
4.11
4.10
4.09
3.11
3.09
3.08
2.64
2.22
2.03
2.01
1.99
1.97
1.65
1.64
1.62
1.32
1.29
1.27
1.25
0.89
0.88
OH
N
N
N
O
S
O
O
9
8
7
6
5
4
3
2
1
ppm

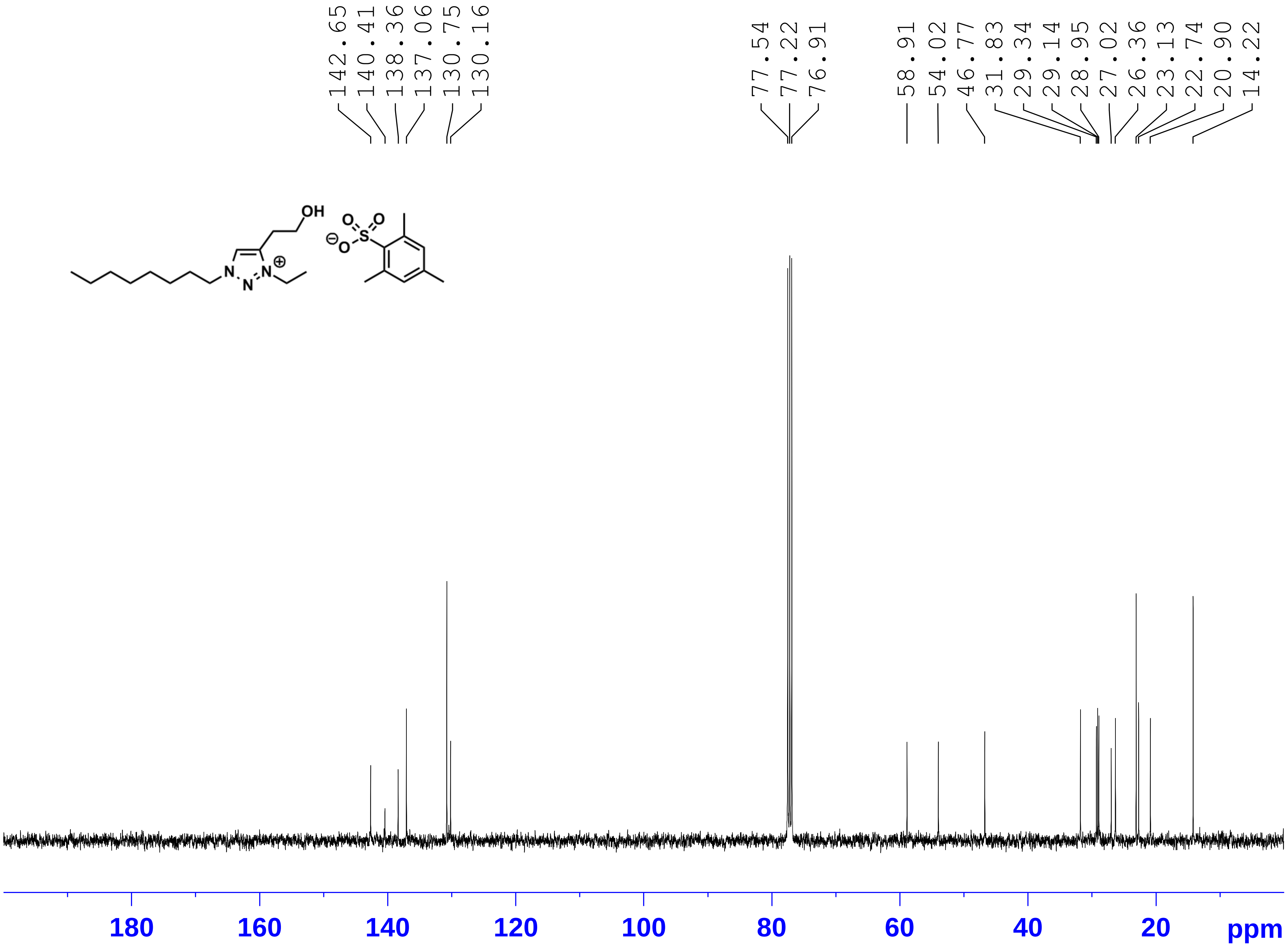

142.65
140.41
138.36
137.06
130.75
130.16
77.54
77.22
76.91
58.91
54.02
46.77
31.83
29.34
29.14
28.95
27.02
26.36
23.13
22.74
20.90
14.22
180
160
140
120
100
80
60
40
20
ppm

Spectrum

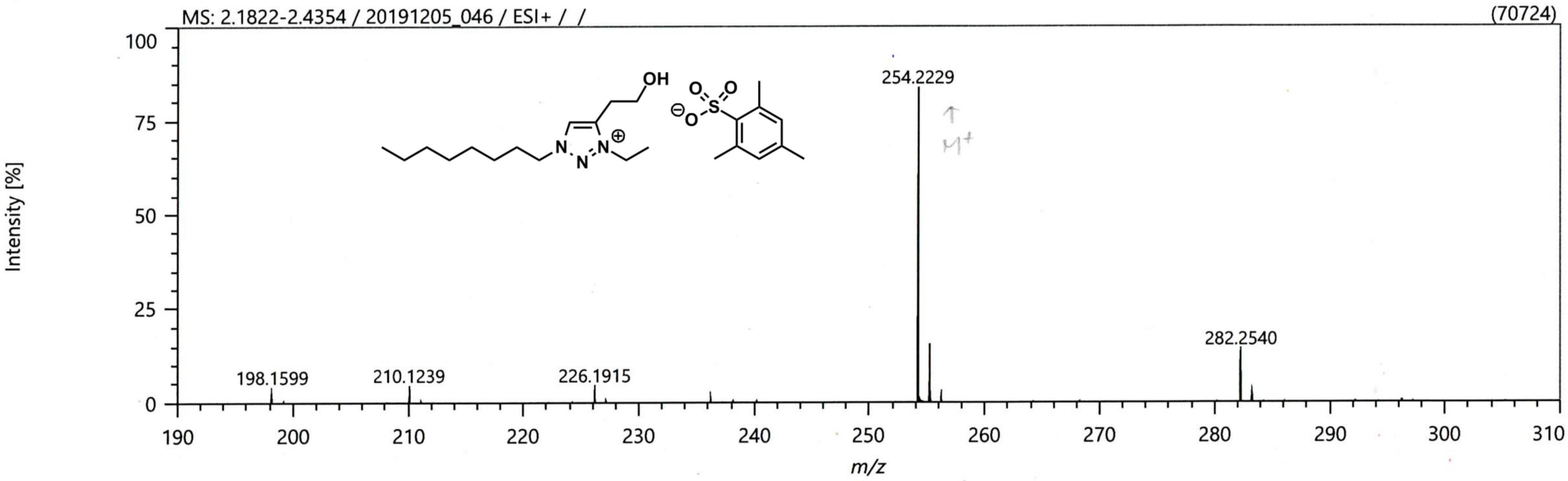

Elemental Composition

Parameters	
Tolerance:	±50.00 ppm
Electron:	Odd/Even
Charge:	+1
DBE:	-1.5 - 999.0

Elements Set 1:

Symbol	C	H	O	Na	N	S	P	Si	F	Cl
Min	0	0	1	0	3	0	0	0	0	0
Max	400	1000	1	0	3	0	0	0	0	0

Results

Mass	Formula	Calculated Mass	Mass Difference [mDa]	Mass Difference [ppm]	DBE
254.22288	C14 H28 N3 O	254.22269	0.19	0.76	2.5

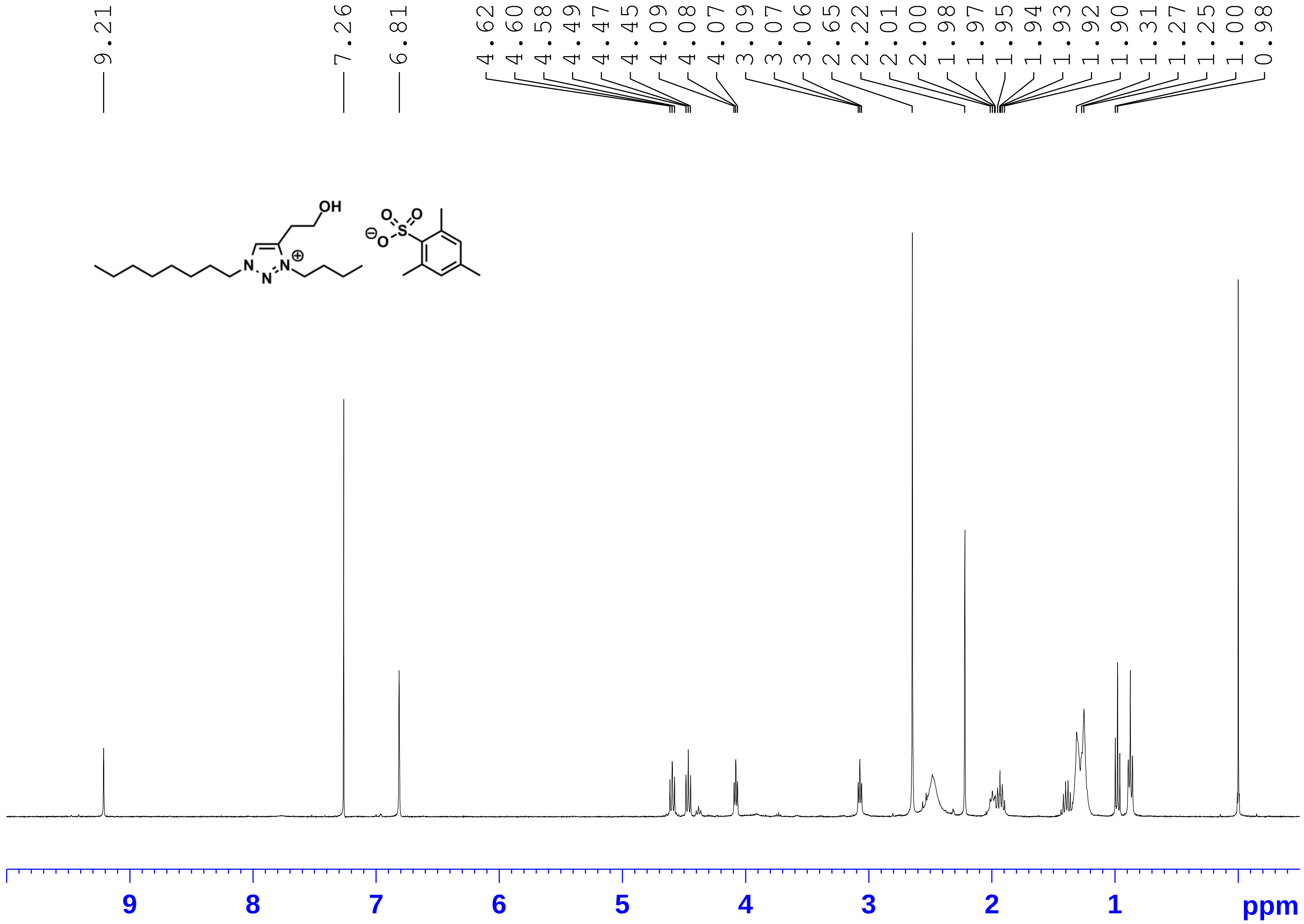
9.21
7.26
6.81
4.62
4.60
4.58
4.49
4.47
4.45
4.09
4.08
4.07
3.09
3.07
3.06
2.65
2.22
2.01
2.00
1.98
1.97
1.95
1.94
1.93
1.92
1.90
1.31
1.27
1.25
1.00
0.98
OH
O
S
O
O
N
N
N
9
8
7
6
5
4
3
2
1
ppm

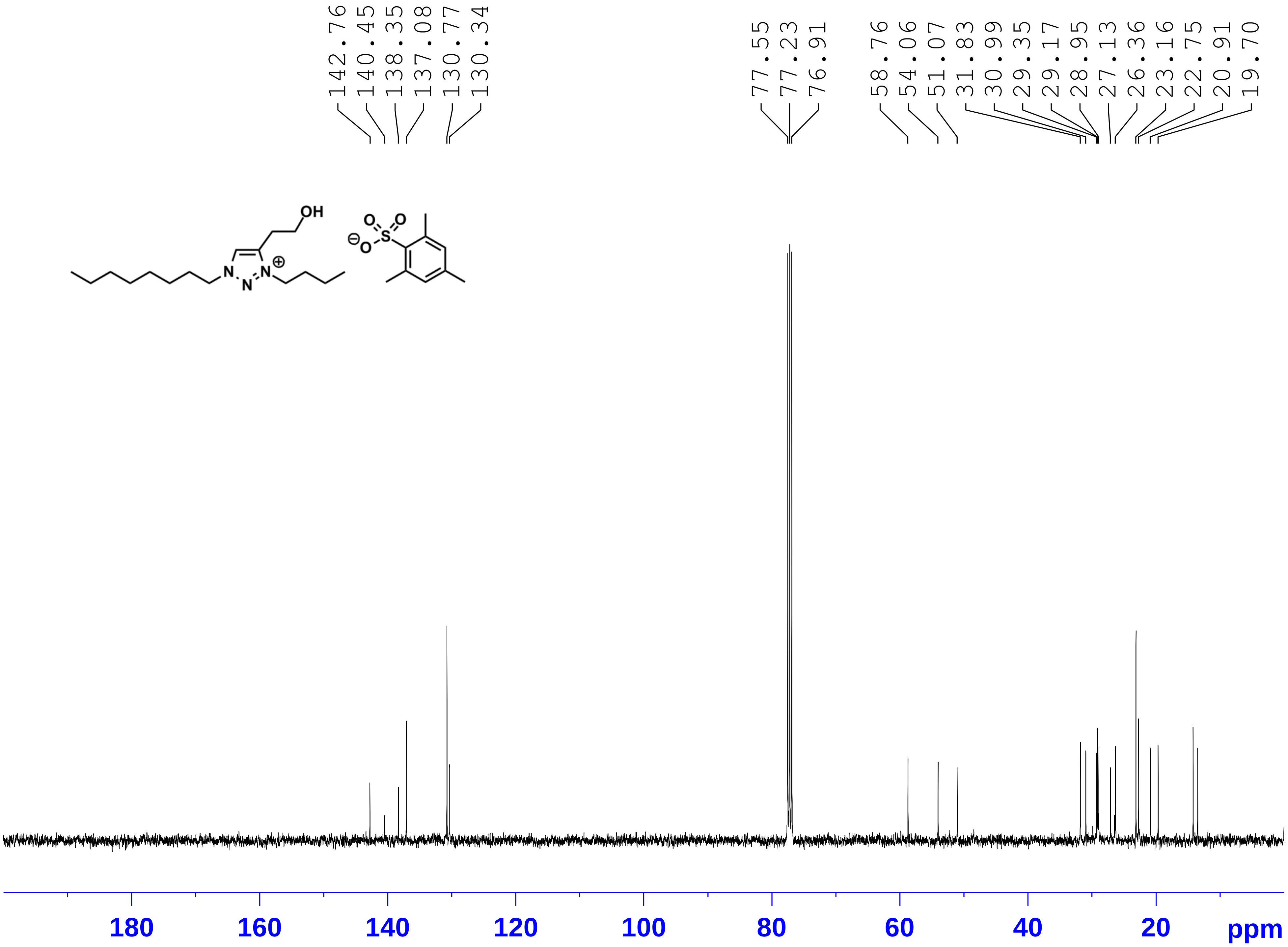
142.76
140.45
138.35
137.08
130.77
130.34
77.55
77.23
76.91
58.76
54.06
51.07
31.83
30.99
29.35
29.17
28.95
27.13
26.36
23.16
22.75
20.91
19.70
OH
O
O
S
O
N
N
N
180
160
140
120
100
80
60
40
20
ppm

Spectrum

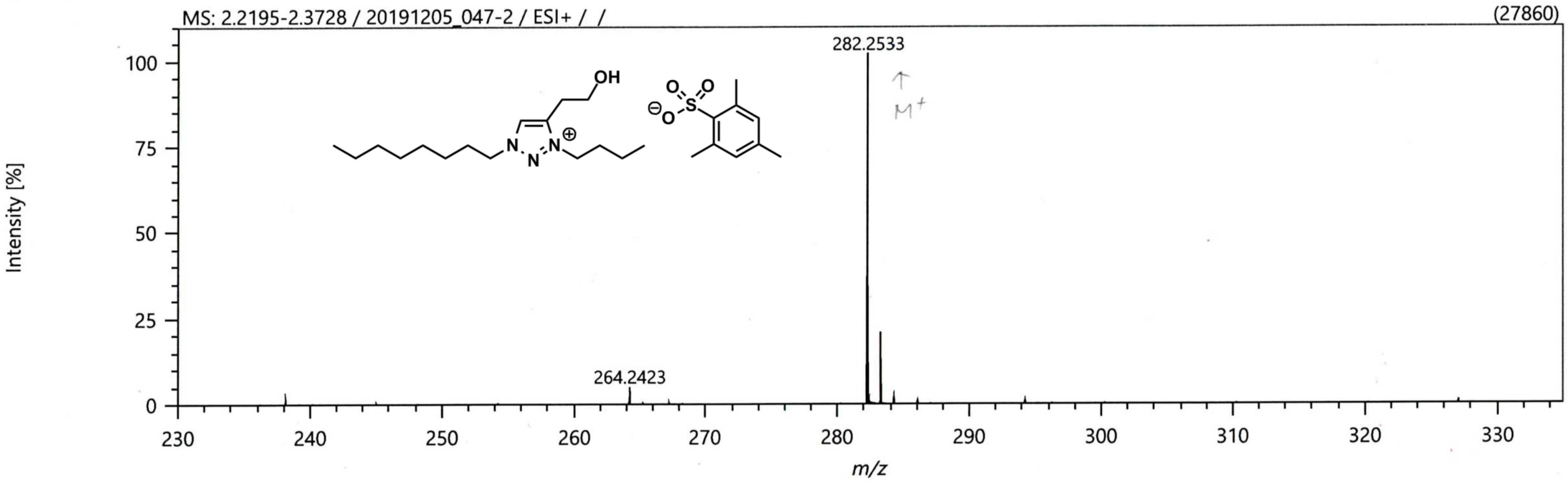

Elemental Composition

Parameters

Tolerance:	±50.00 ppm
Electron:	Odd/Even
Charge:	+1
DBE:	-1.5 - 999.0

Elements Set 1:

Symbol	C	H	O	Na	N	S	P	Si	F	Cl
Min	0	0	1	0	3	0	0	0	0	0
Max	400	1000	1	0	3	0	0	0	0	0

Results

Mass	Formula	Calculated Mass	Mass Difference [mDa]	Mass Difference [ppm]	DBE
282.25325	C16 H32 N3 O	282.25399	-0.74	-2.60	2.5

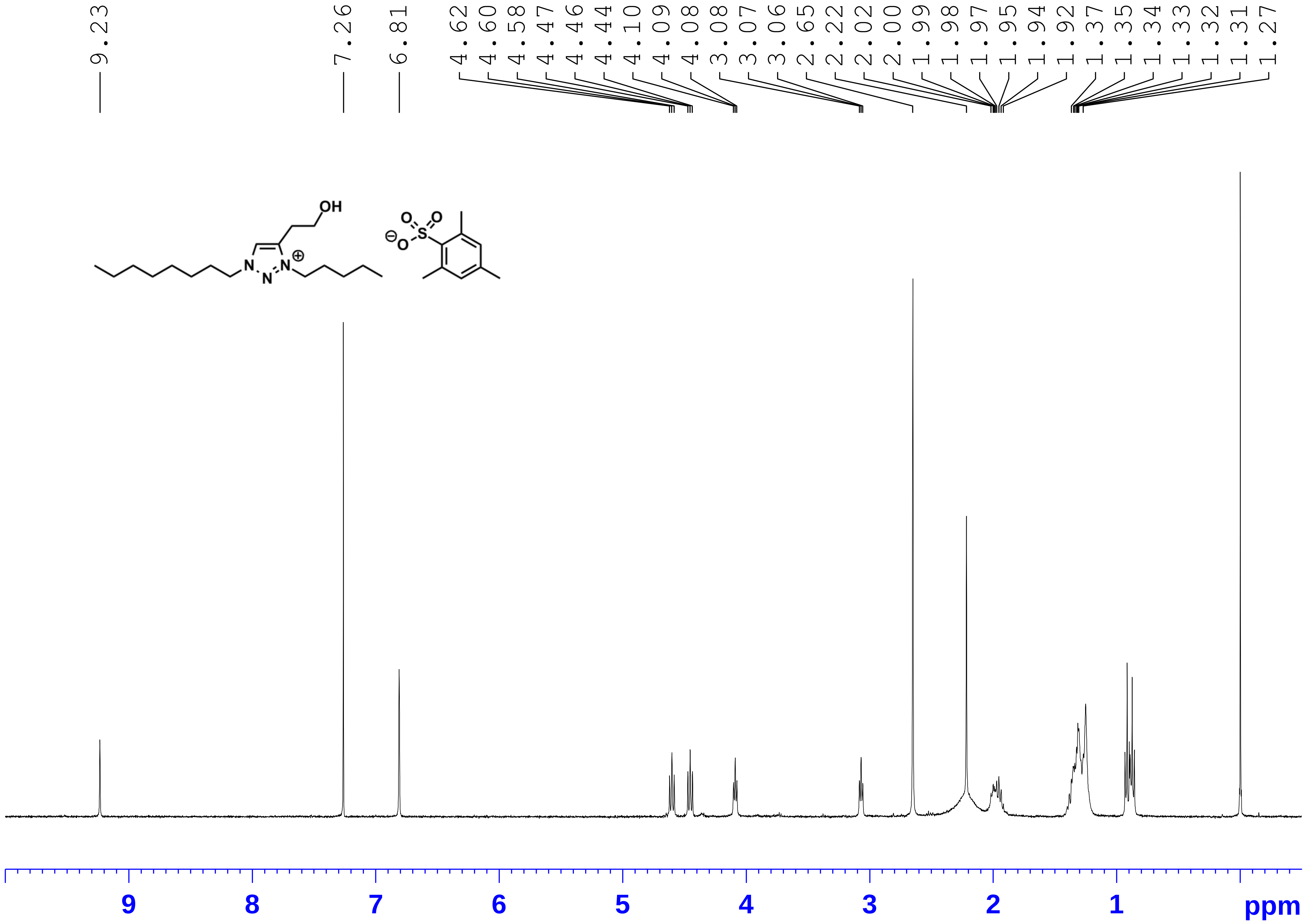

9.23
7.26
6.81
4.62
4.60
4.58
4.47
4.46
4.44
4.10
4.09
4.08
3.08
3.07
3.06
2.65
2.22
2.02
2.00
1.99
1.98
1.97
1.95
1.94
1.92
1.37
1.35
1.34
1.33
1.32
1.31
1.27
OH
N
N
N
O
O
S
O
9
8
7
6
5
4
3
2
1
ppm

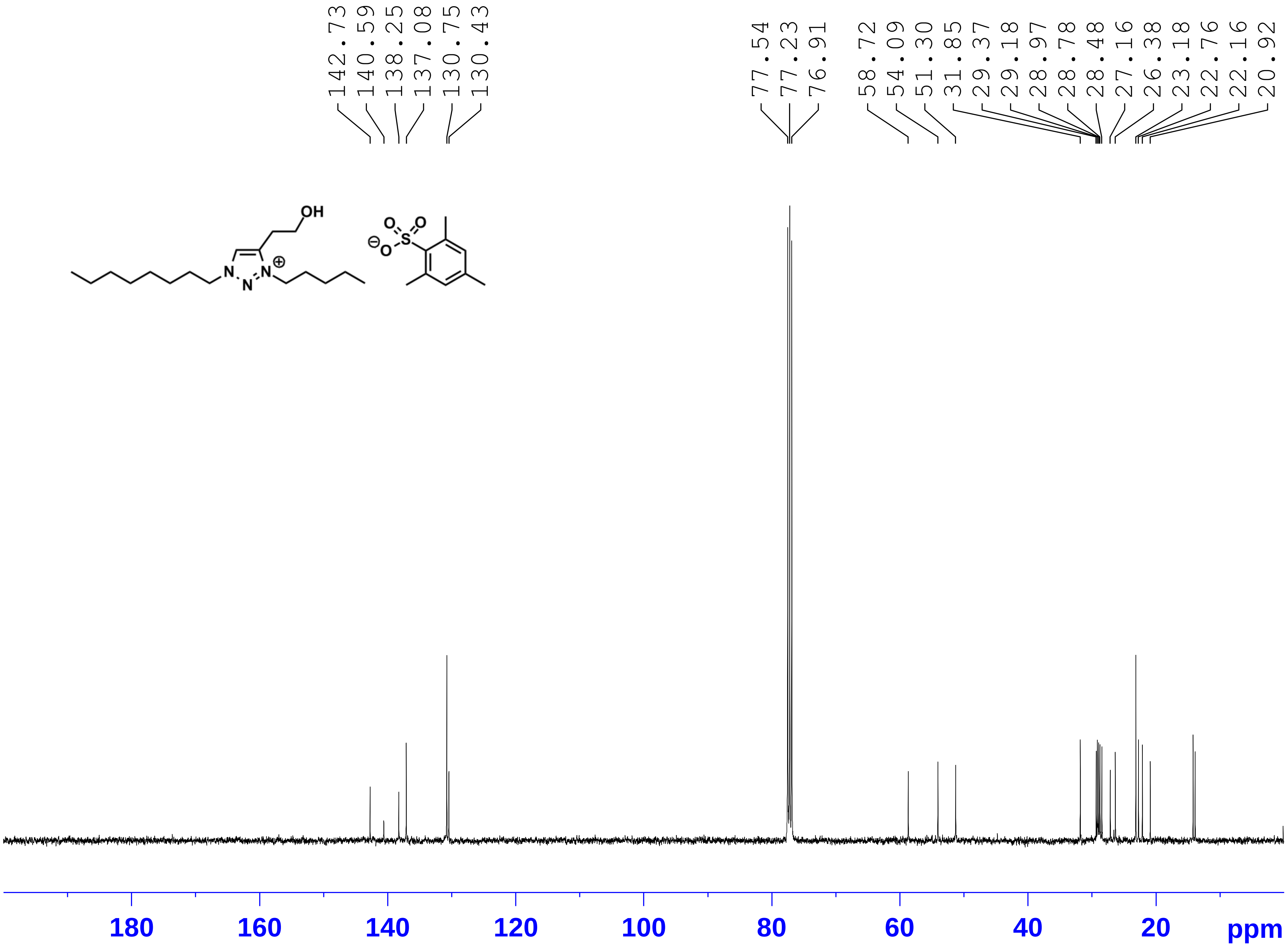
142.73
140.59
138.25
137.08
130.75
130.43
77.54
77.23
76.91
58.72
54.09
51.30
31.85
29.37
29.18
28.97
28.78
28.48
27.16
26.38
23.18
22.76
22.16
20.92
OH
O
S
O
O
N
N
N
180
160
140
120
100
80
60
40
20
ppm

Spectrum

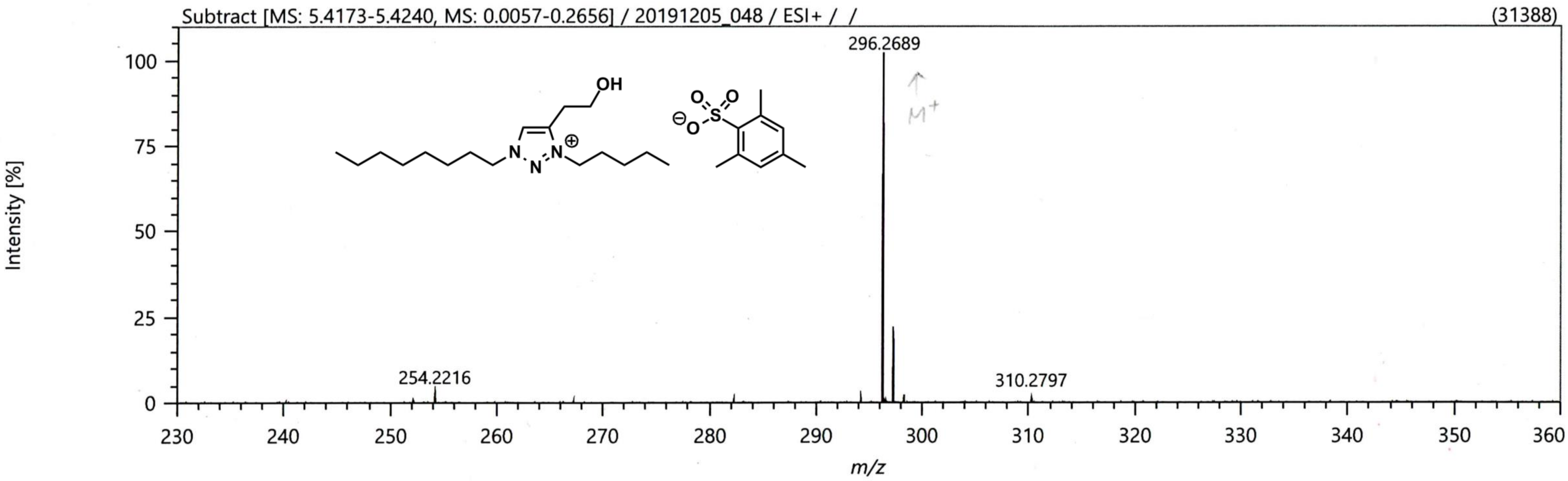

Elemental Composition

Parameters

Tolerance:	±50.00 ppm
Electron:	Odd/Even
Charge:	+1
DBE:	-1.5 - 999.0

Elements Set 1:

Symbol	C	H	O	Na	N	S	P	Si	F	Cl
Min	0	0	1	0	3	0	0	0	0	0
Max	400	1000	1	0	3	0	0	0	0	0

Results

Mass	Formula	Calculated Mass	Mass Difference [mDa]	Mass Difference [ppm]	DBE
296.26888	C17 H34 N3 O	296.26964	-0.76	-2.58	2.5

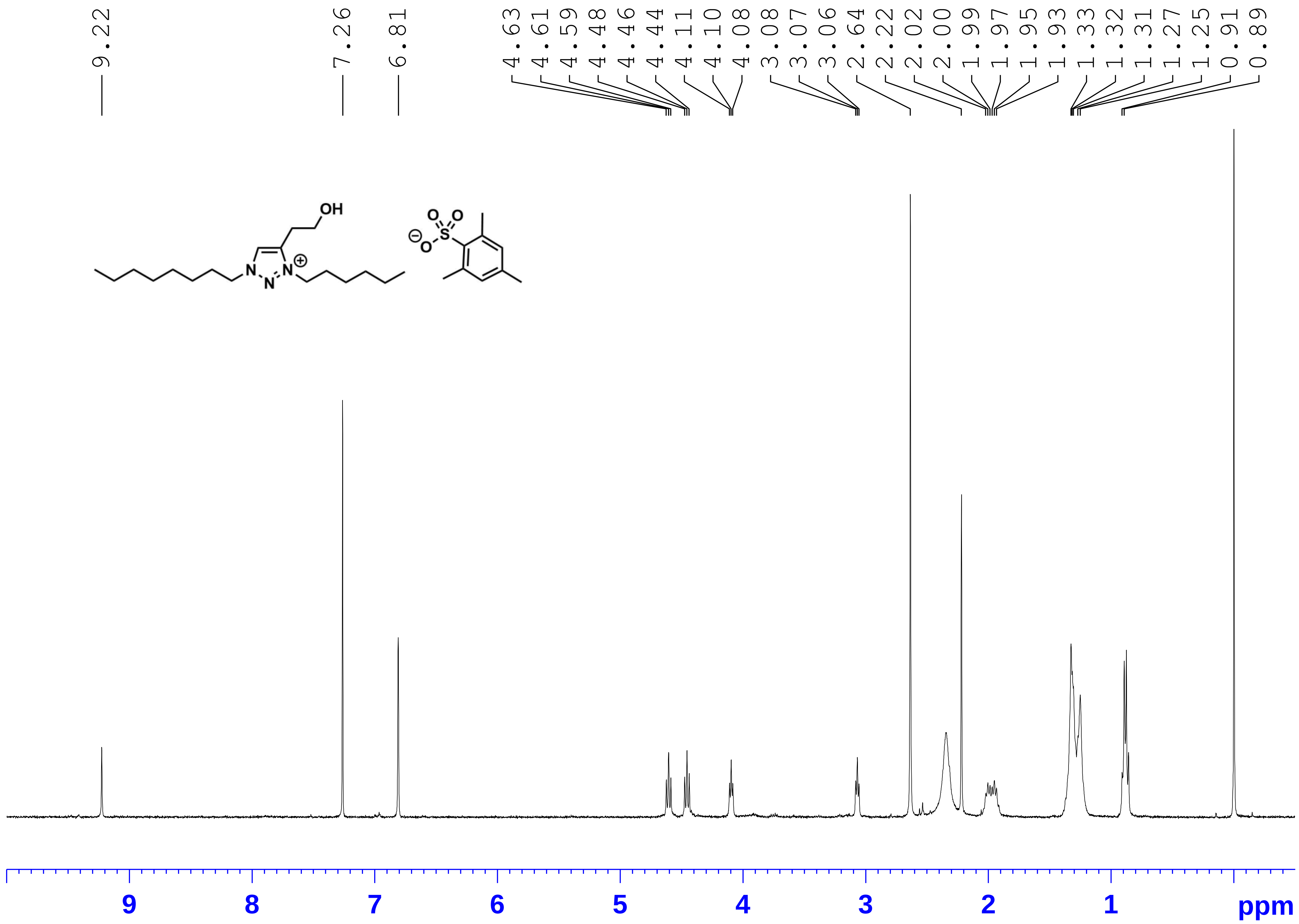
OH
N
N
N
O
O
S
O
9.22
7.26
6.81
4.63
4.61
4.59
4.48
4.46
4.44
4.11
4.10
4.08
3.08
3.07
3.06
2.64
2.22
2.02
2.00
1.99
1.97
1.95
1.93
1.33
1.32
1.31
1.27
1.25
0.91
0.89
9
8
7
6
5
4
3
2
1
ppm

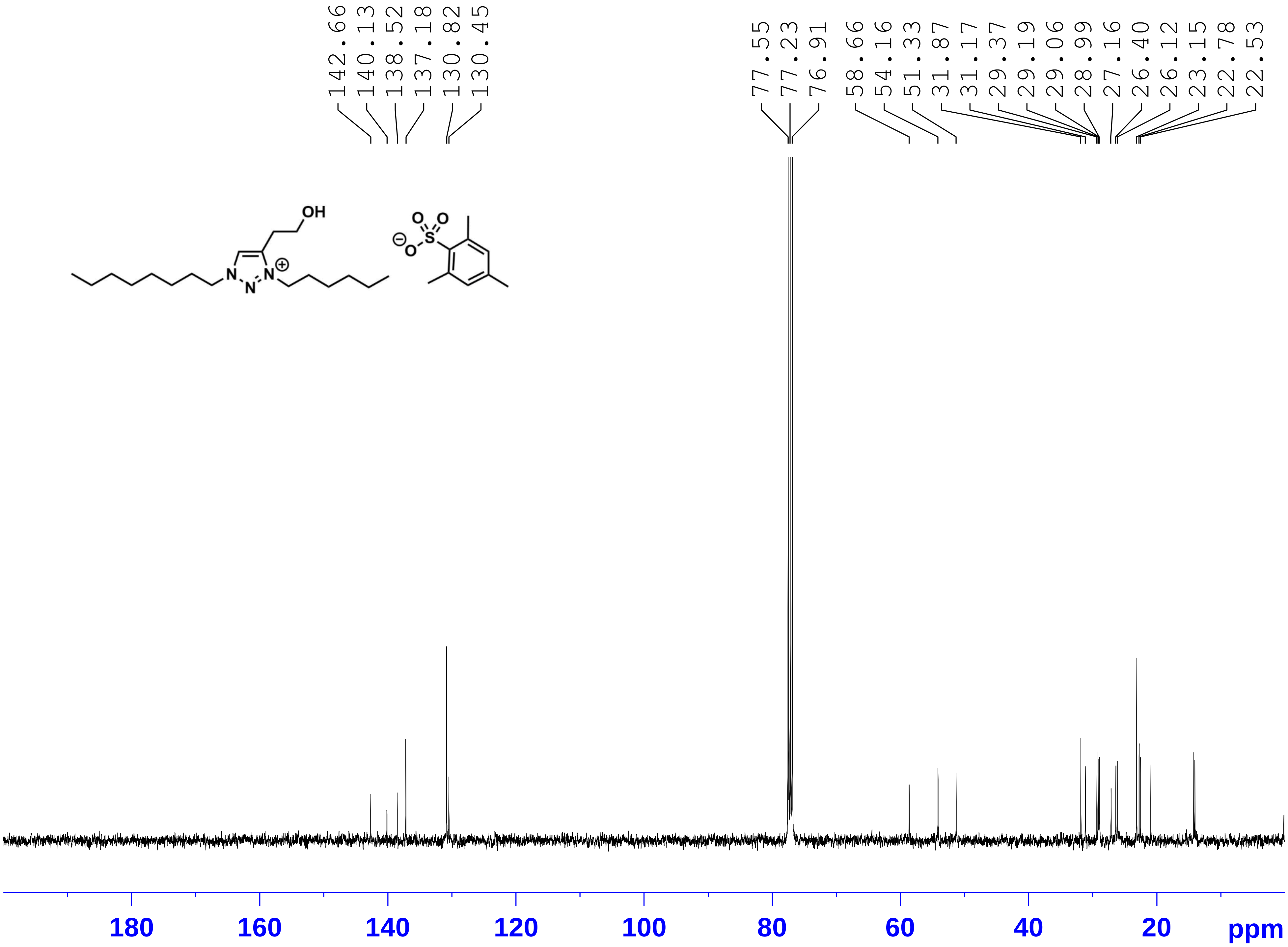

142.66
140.13
138.52
137.18
130.82
130.45
77.55
77.23
76.91
58.66
54.16
51.33
31.87
31.17
29.37
29.19
29.06
28.99
27.16
26.40
26.12
23.15
22.78
22.53
180
160
140
120
100
80
60
40
20
ppm

Spectrum

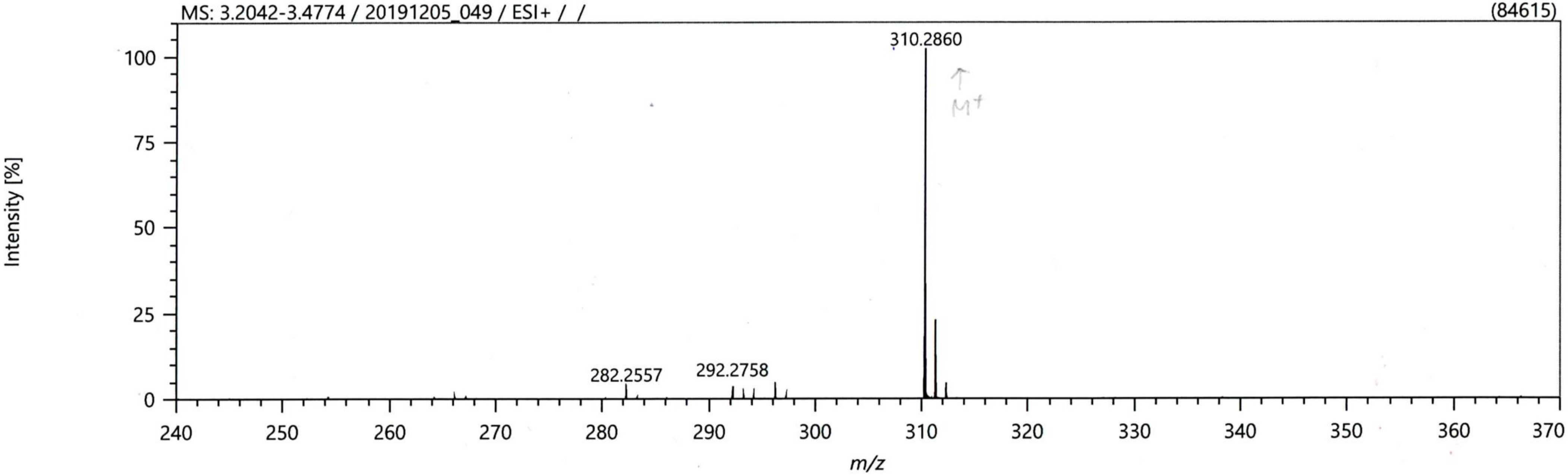

Elemental Composition

Parameters	
Tolerance:	±50.00 ppm
Electron:	Odd/Even
Charge:	+1
DBE:	-1.5 - 999.0

Elements Set 1:

Symbol	C	H	O	Na	N	S	P	Si	F	Cl
Min	0	0	1	0	3	0	0	0	0	0
Max	400	1000	1	0	3	0	0	0	0	0

Results

Mass	Formula	Calculated Mass	Mass Difference [mDa]	Mass Difference [ppm]	DBE
310.28602	C18 H36 N3 O	310.28529	0.73	2.34	2.5

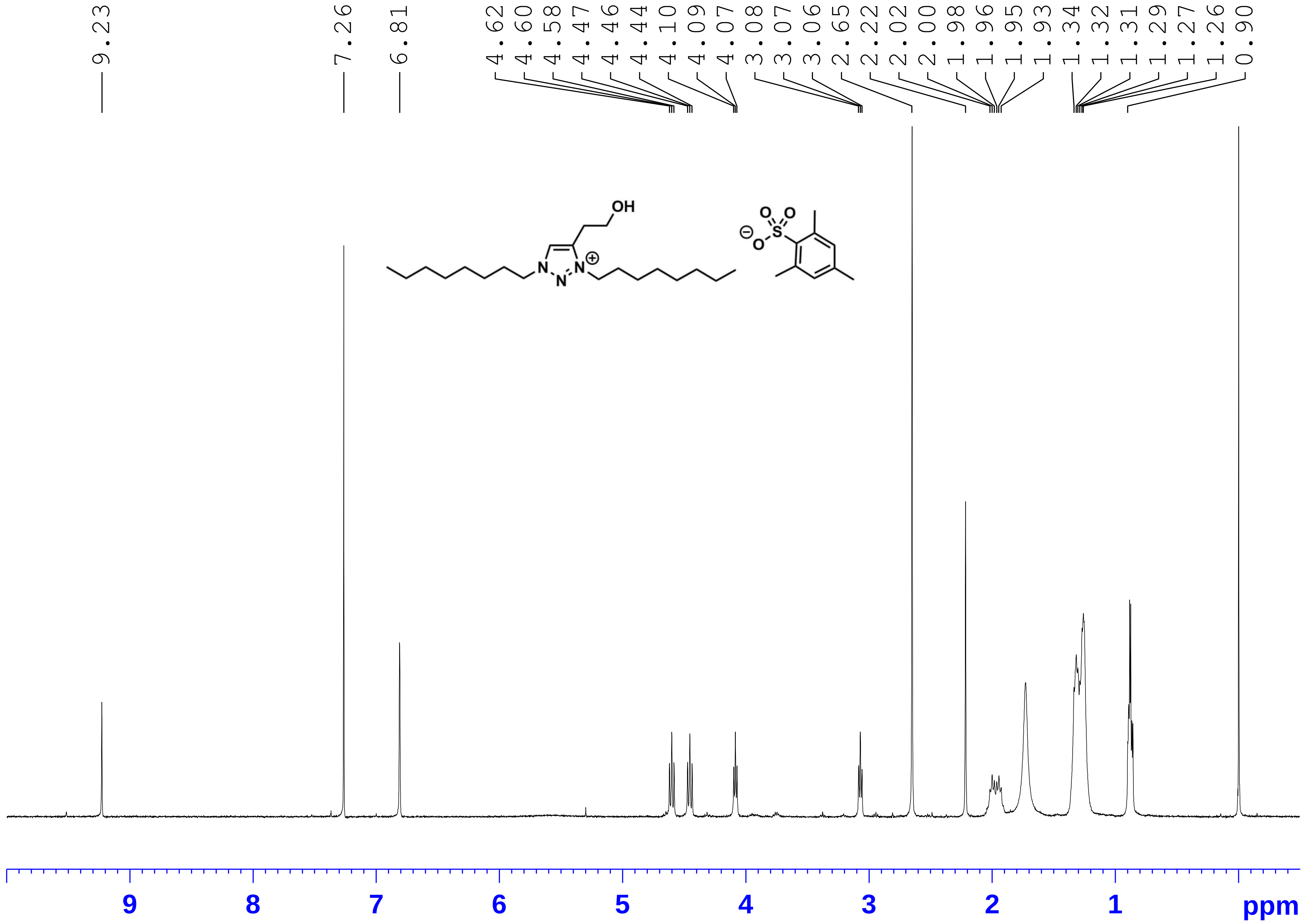
9.23
7.26
6.81
4.62
4.60
4.58
4.47
4.46
4.44
4.10
4.09
4.07
3.08
3.07
3.06
2.65
2.22
2.02
2.00
1.98
1.96
1.95
1.93
1.34
1.32
1.31
1.29
1.27
1.26
0.90
OH
N
N
N
O
O
S
O
9
8
7
6
5
4
3
2
1
ppm

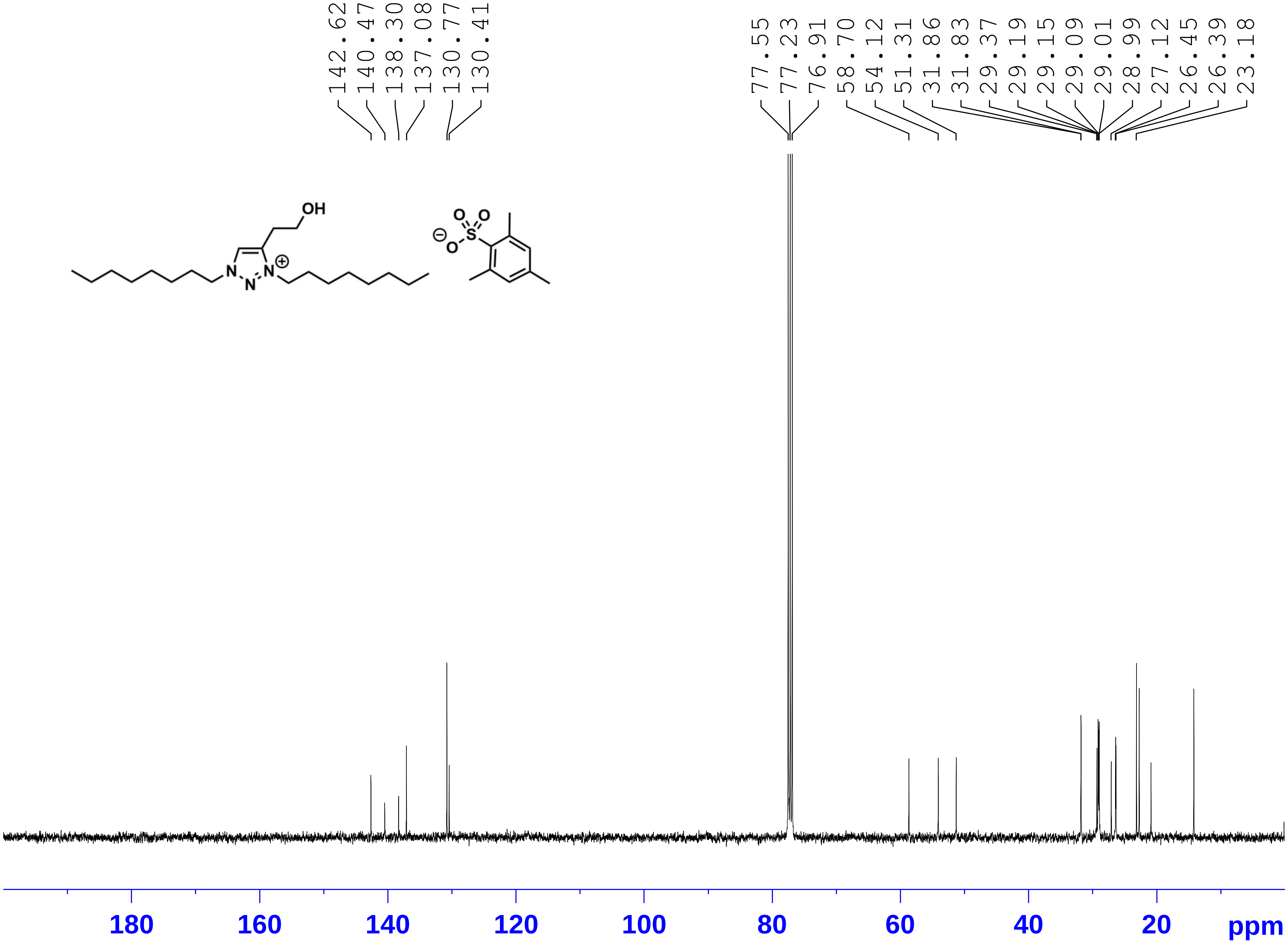

142.62
140.47
138.30
137.08
130.77
130.41
77.55
77.23
76.91
58.70
54.12
51.31
31.86
31.83
29.37
29.19
29.15
29.09
29.01
28.99
27.12
26.45
26.39
23.18
OH
N
N
N
O
O
S
O
180
160
140
120
100
80
60
40
20
ppm

Spectrum

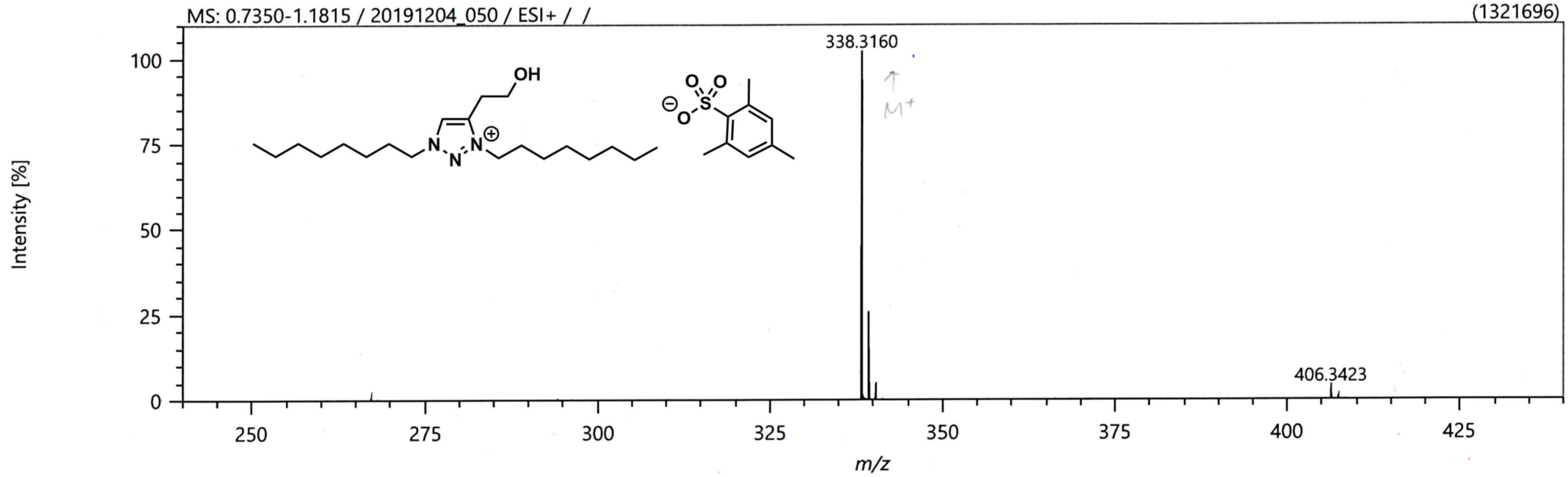

Elemental Composition

Parameters	
Tolerance:	±10.00 ppm
Electron:	Odd/Even
Charge:	+1
DBE:	-1.5 - 999.0

Elements Set 1:

Symbol	C	H	O	Na	N	S	P	Si	F	Cl
Min	0	0	1	0	3	0	0	0	0	0
Max	400	1000	1	0	3	0	0	0	0	0

Results

Mass	Formula	Calculated Mass	Mass Difference [mDa]	Mass Difference [ppm]	DBE
338.31603	C20 H40 N3 O	338.31659	-0.56	-1.66	2.5

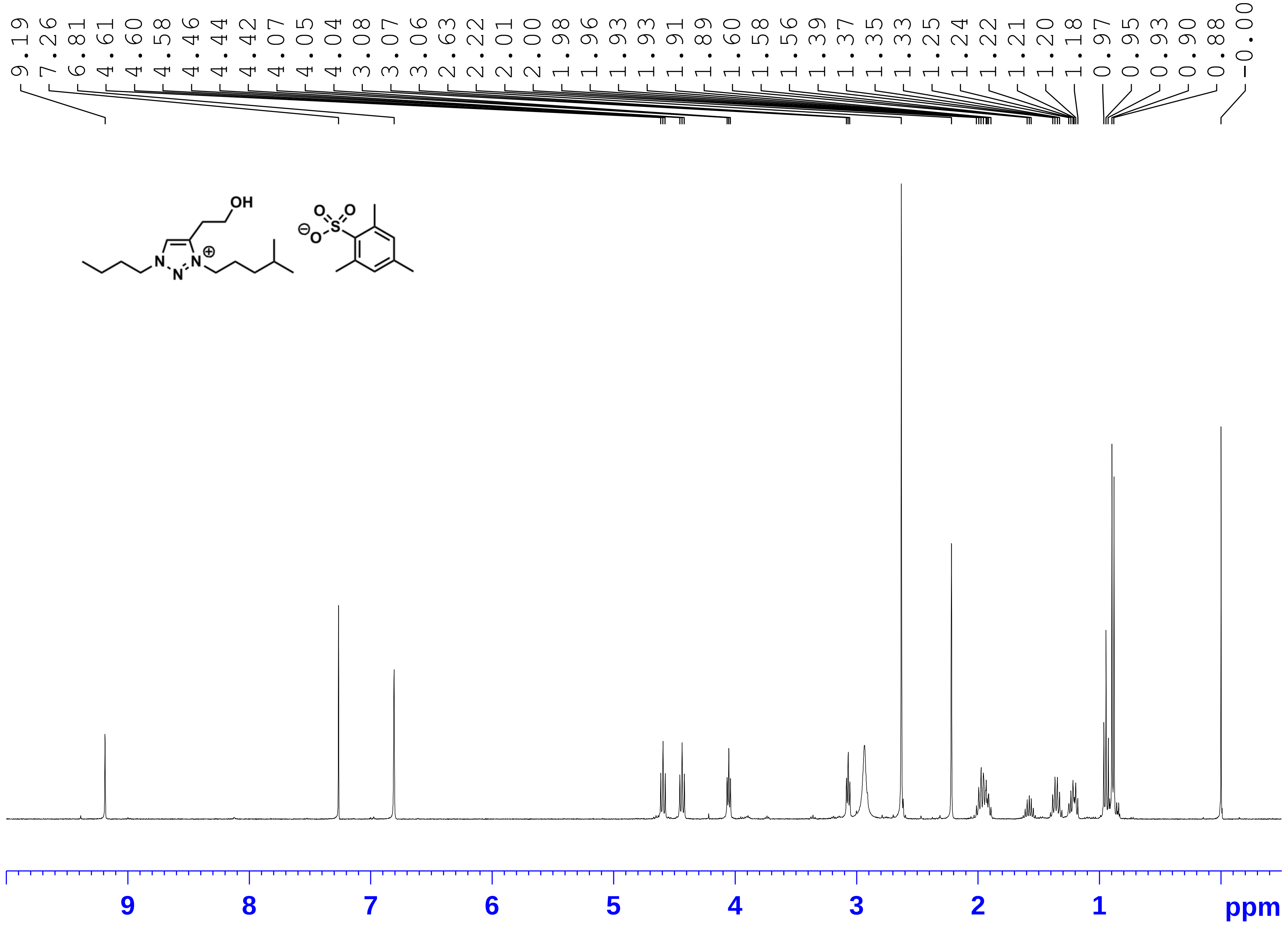
9.19
7.26
6.81
4.61
4.60
4.58
4.46
4.44
4.42
4.07
4.05
4.04
3.08
3.07
3.06
2.63
2.22
2.01
2.00
1.98
1.96
1.93
1.93
1.91
1.89
1.60
1.58
1.56
1.39
1.37
1.35
1.33
1.25
1.24
1.22
1.21
1.20
1.18
0.97
0.95
0.93
0.90
0.88
-0.00
OH
N
N
N
O
O
S
O
9
8
7
6
5
4
3
2
1
ppm

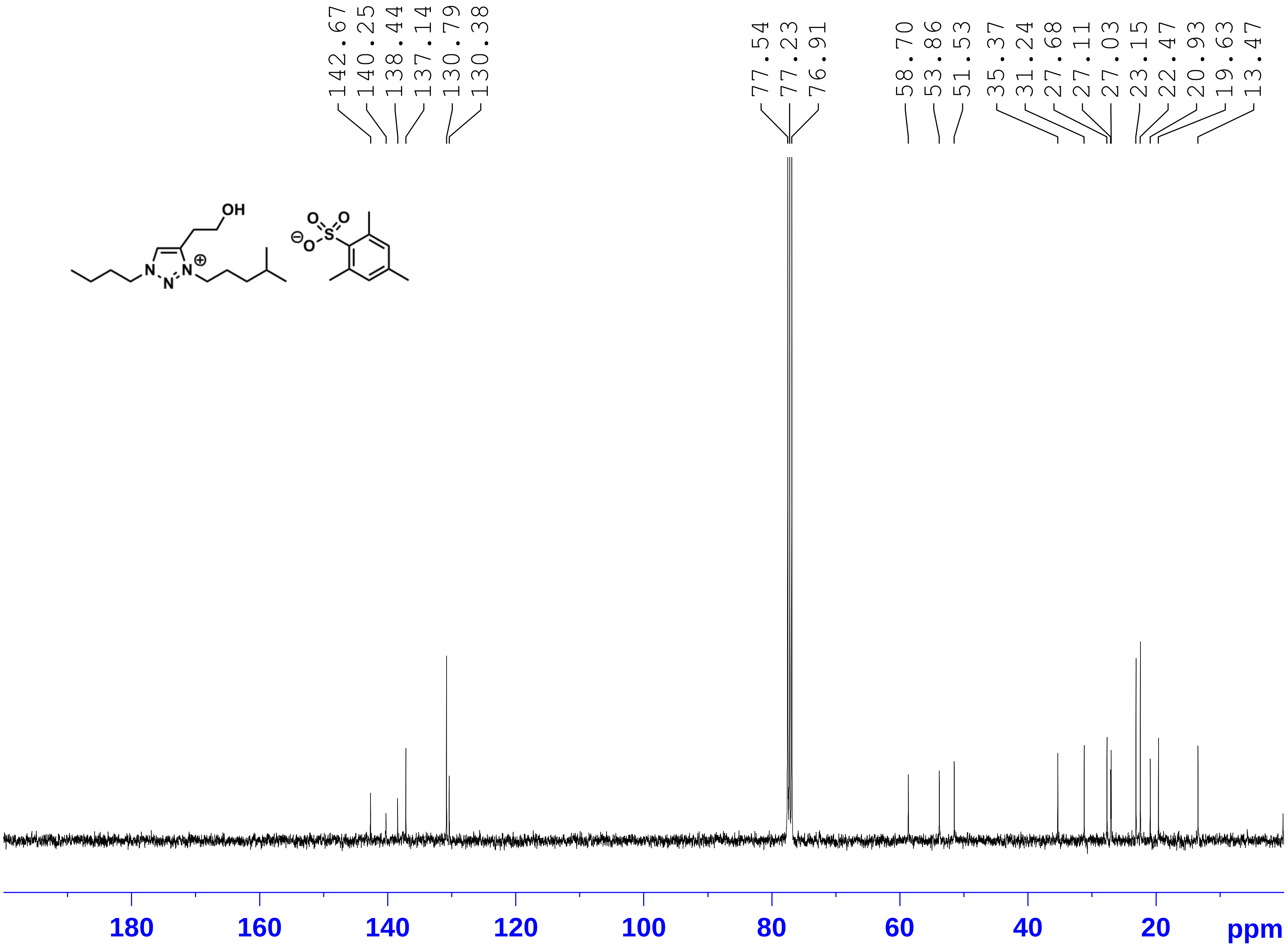

142.67
140.25
138.44
137.14
130.79
130.38
77.54
77.23
76.91
58.70
53.86
51.53
35.37
31.24
27.68
27.11
27.03
23.15
22.47
20.93
19.63
13.47
OH
O
O
S
O
N
N
N
180
160
140
120
100
80
60
40
20
ppm

Spectrum

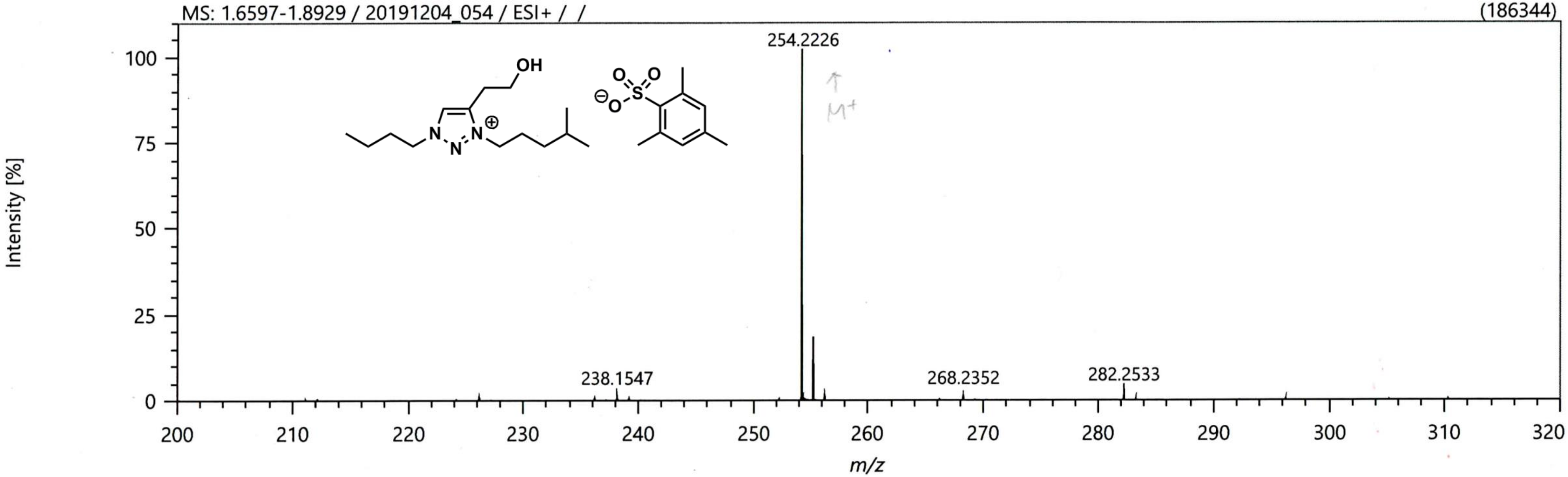

Elemental Composition

Parameters		Elements Set 1:										
Tolerance:	±10.00 ppm	Symbol	C	H	O	Na	N	S	P	Si	F	Cl
Electron:	Odd/Even	Min	0	0	1	0	3	0	0	0	0	0
Charge:	+1	Max	400	1000	1	0	3	0	0	0	0	0
DBE:	-1.5 - 999.0											

Results

Mass	Formula	Calculated Mass	Mass Difference [mDa]	Mass Difference [ppm]	DBE
254.22256	C14 H28 N3 O	254.22269	-0.13	-0.52	2.5

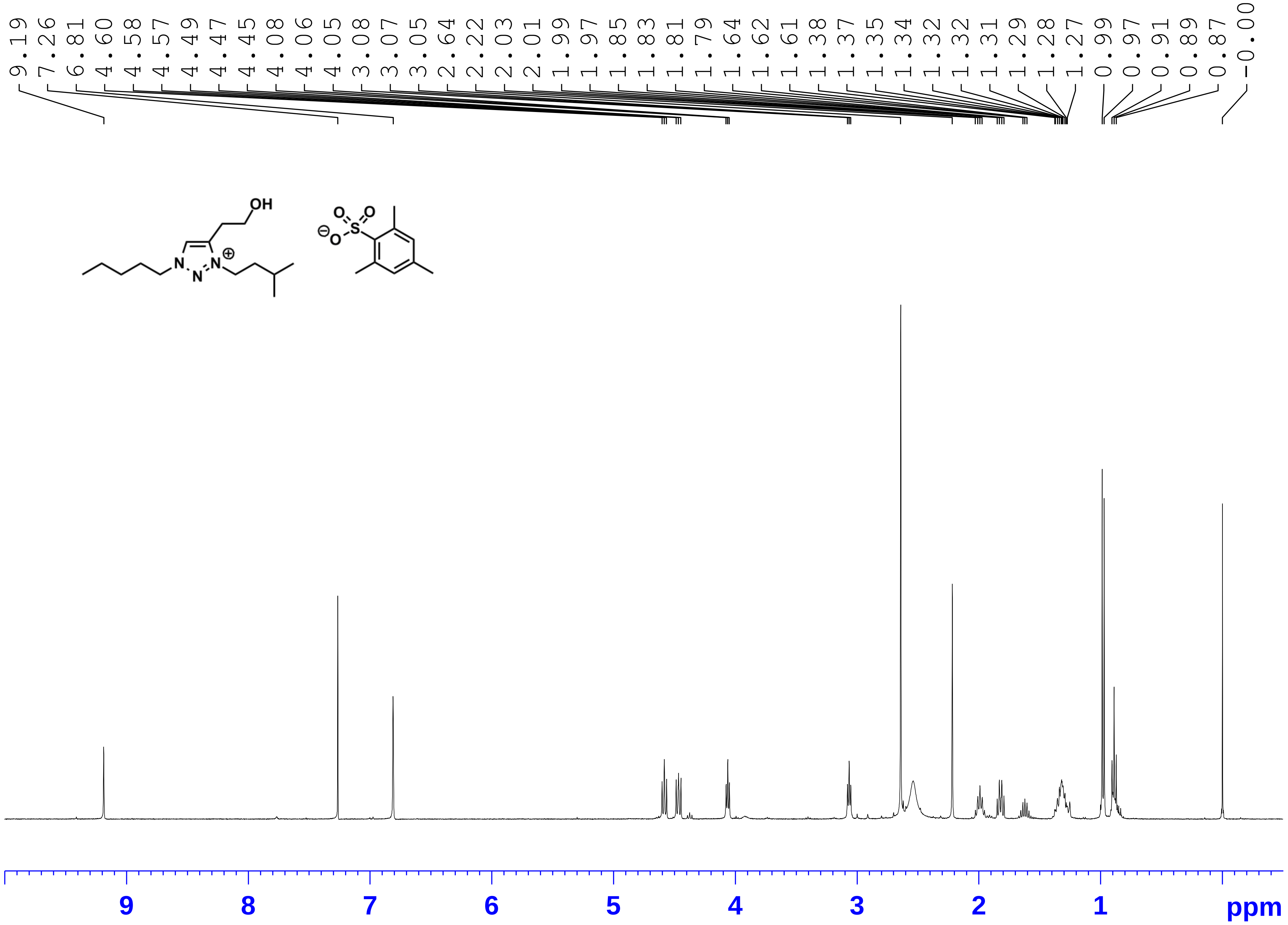
9.19
7.26
6.81
4.60
4.58
4.57
4.49
4.47
4.45
4.08
4.06
4.05
3.08
3.07
3.05
2.64
2.22
2.03
2.01
1.99
1.97
1.85
1.83
1.81
1.79
1.64
1.62
1.61
1.38
1.37
1.35
1.34
1.32
1.32
1.31
1.29
1.28
1.27
0.99
0.97
0.91
0.89
0.87
-0.00
OH
N
N
N
O
O
S
O
9
8
7
6
5
4
3
2
1
ppm

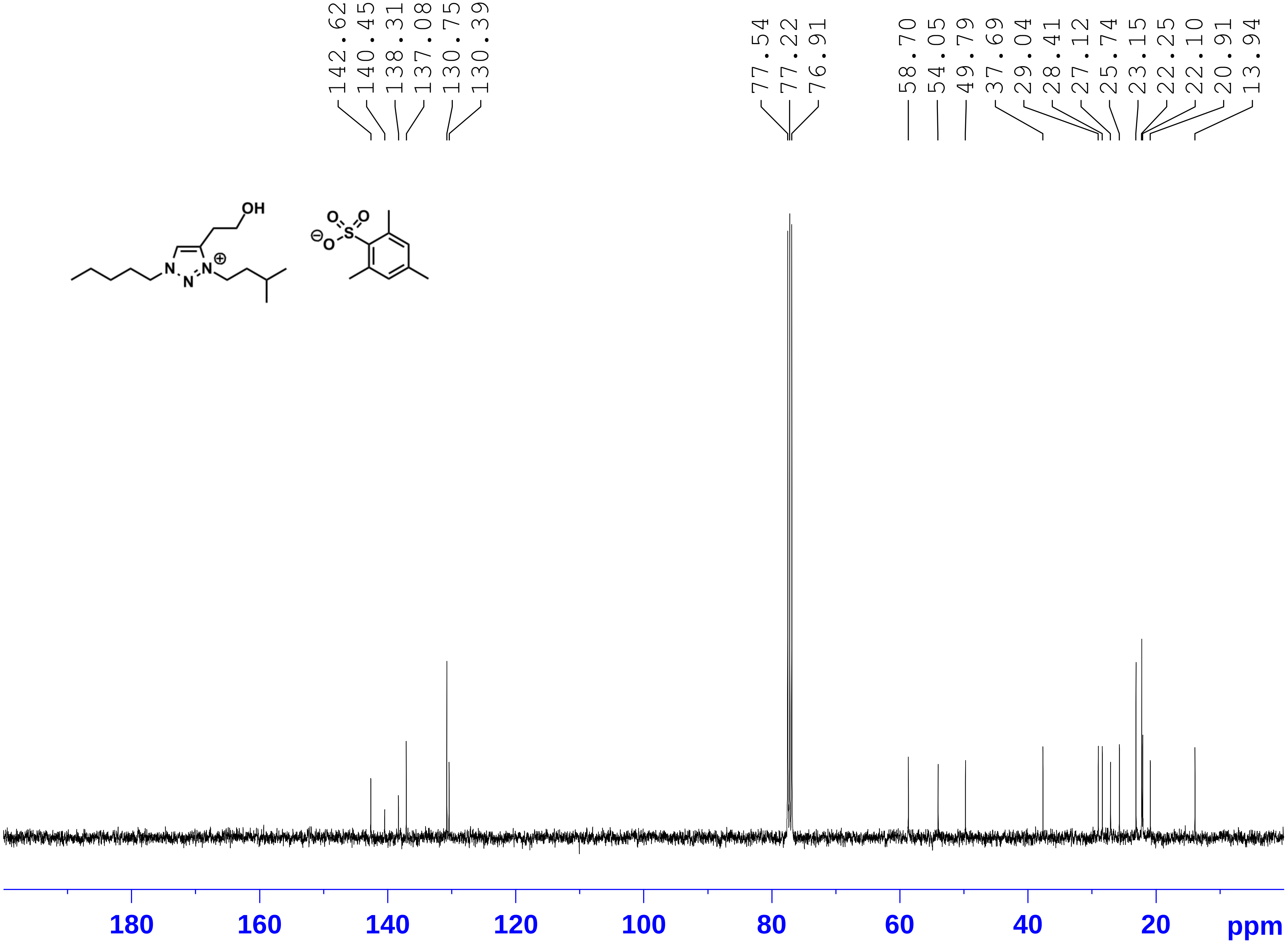
142.62
140.45
138.31
137.08
130.75
130.39
77.54
77.22
76.91
58.70
54.05
49.79
37.69
29.04
28.41
27.12
25.74
23.15
22.25
22.10
20.91
13.94
OH
N
N
N
S
O
O
O
180
160
140
120
100
80
60
40
20
ppm

Spectrum

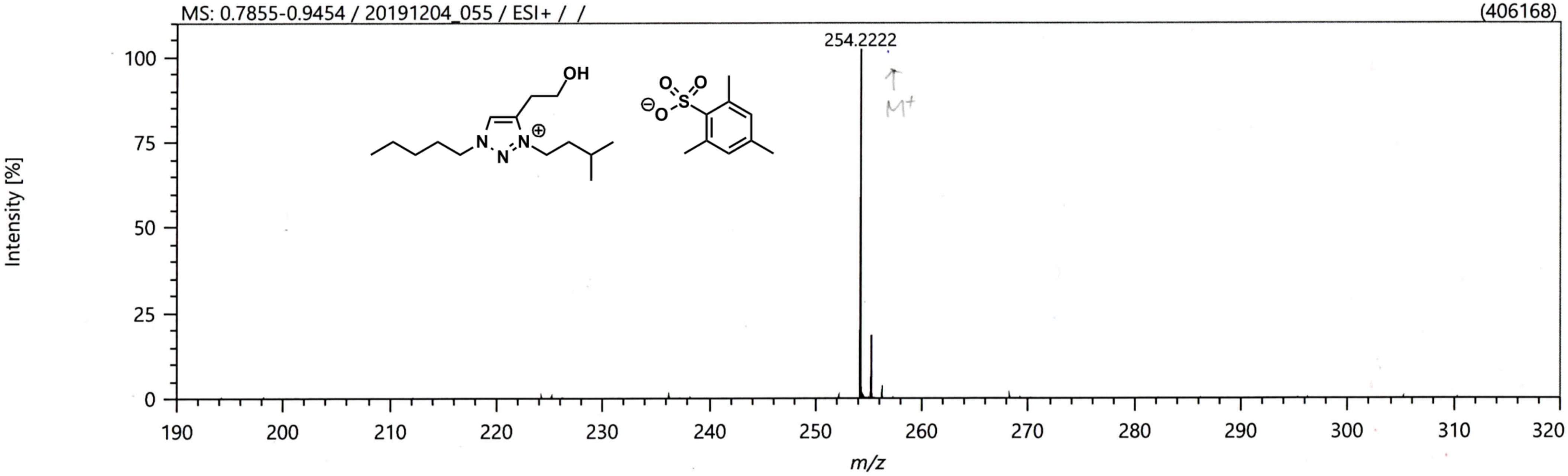

Elemental Composition

Parameters	
Tolerance:	±10.00 ppm
Electron:	Odd/Even
Charge:	+1
DBE:	-1.5 - 999.0

Elements Set 1:

Symbol	C	H	O	Na	N	S	P	Si	F	Cl
Min	0	0	1	0	3	0	0	0	0	0
Max	400	1000	1	0	3	0	0	0	0	0

Results

Mass	Formula	Calculated Mass	Mass Difference [mDa]	Mass Difference [ppm]	DBE
254.22220	C14 H28 N3 O	254.22269	-0.49	-1.92	2.5

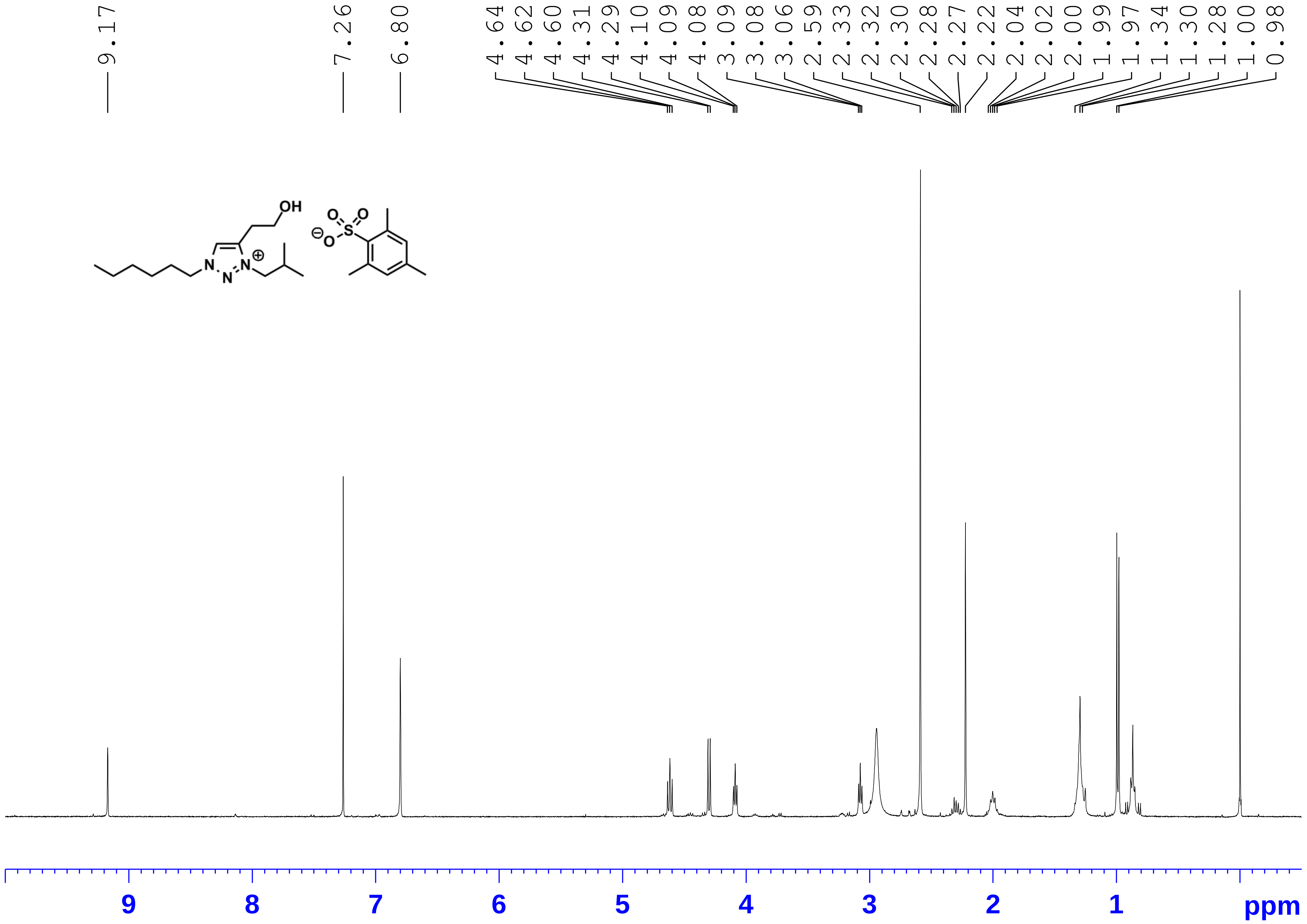

9.17
7.26
6.80
4.64
4.62
4.60
4.31
4.29
4.10
4.09
4.08
3.09
3.08
3.06
2.59
2.33
2.32
2.30
2.28
2.27
2.22
2.04
2.02
2.00
1.99
1.97
1.34
1.30
1.28
1.00
0.98
OH
N
N
N
O
S
O
O
9
8
7
6
5
4
3
2
1
ppm

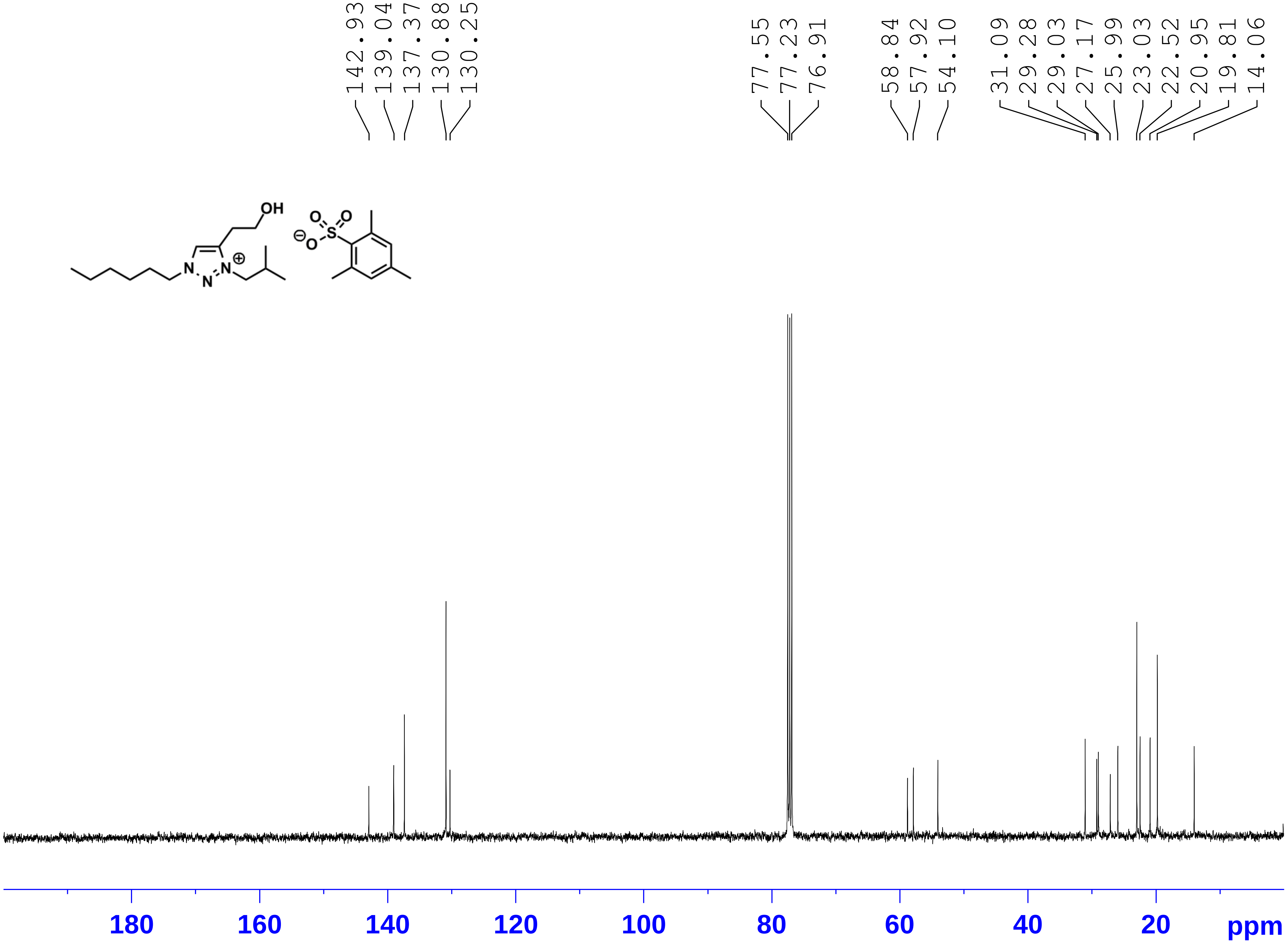
142.93
139.04
137.37
130.88
130.25
77.55
77.23
76.91
58.84
57.92
54.10
31.09
29.28
29.03
27.17
25.99
23.03
22.52
20.95
19.81
14.06
180
160
140
120
100
80
60
40
20
ppm

Spectrum

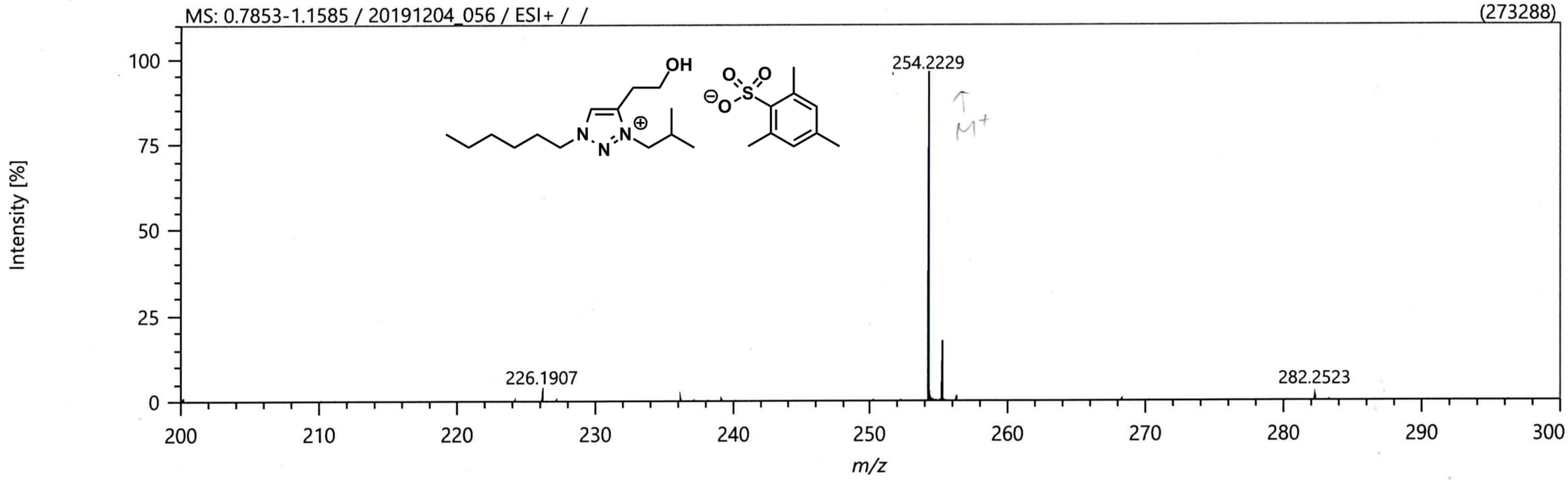

Elemental Composition

Parameters		Elements Set 1:										
Tolerance:	±10.00 ppm	Symbol	C	H	O	Na	N	S	P	Si	F	Cl
Electron:	Odd/Even	Min	0	0	1	0	3	0	0	0	0	0
Charge:	+1	Max	400	1000	1	0	3	0	0	0	0	0
DBE:	-1.5 - 999.0											

Results

Mass	Formula	Calculated Mass	Mass Difference [mDa]	Mass Difference [ppm]	DBE
254.22291	C14 H28 N3 O	254.22269	0.22	0.88	2.5

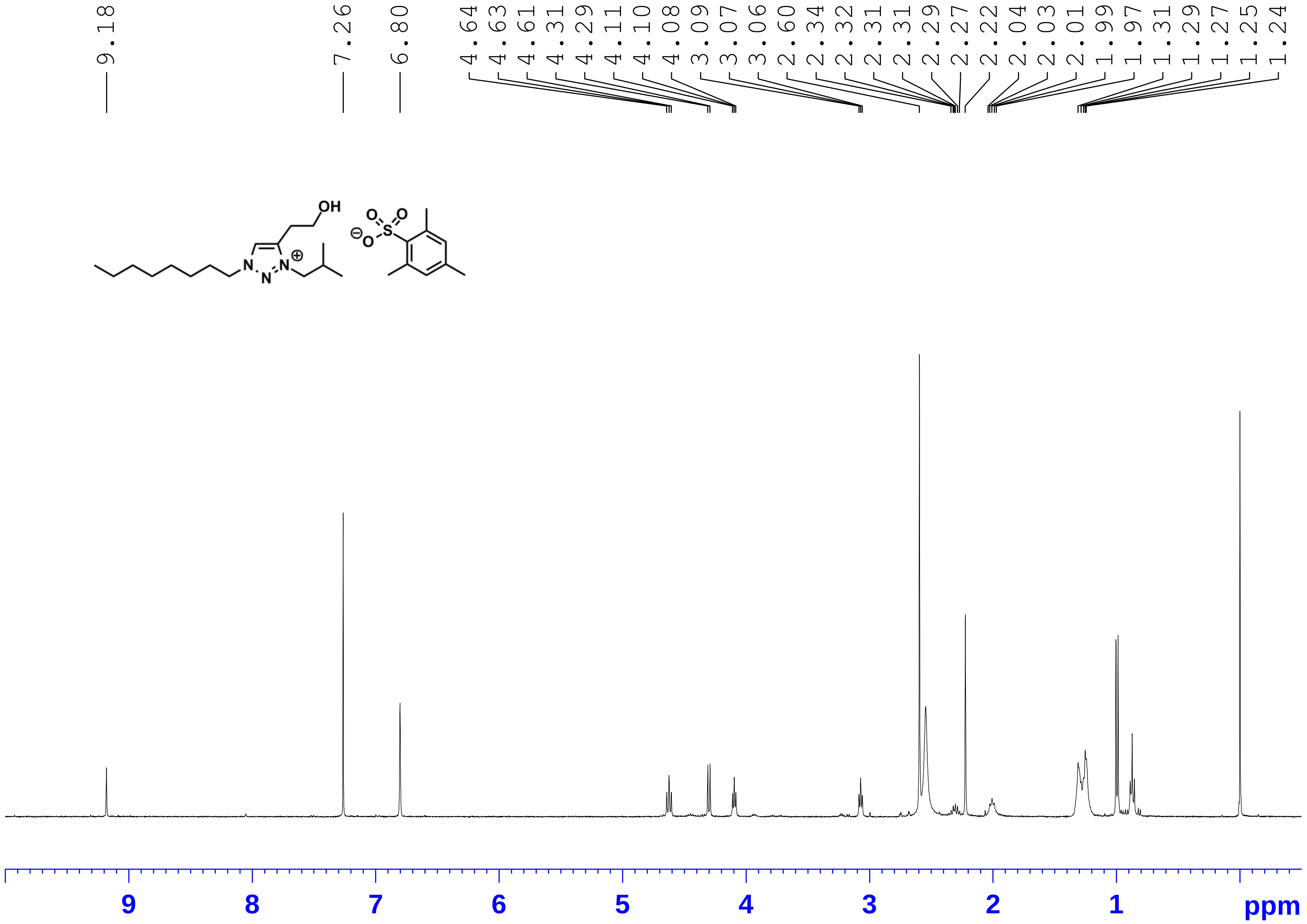
9.18
7.26
6.80
4.64
4.63
4.61
4.31
4.29
4.11
4.10
4.08
3.09
3.07
3.06
2.60
2.34
2.32
2.31
2.31
2.29
2.27
2.22
2.04
2.03
2.01
1.99
1.97
1.31
1.29
1.27
1.25
1.24
OH
O
S
O
O
N
N
N
9
8
7
6
5
4
3
2
1
ppm

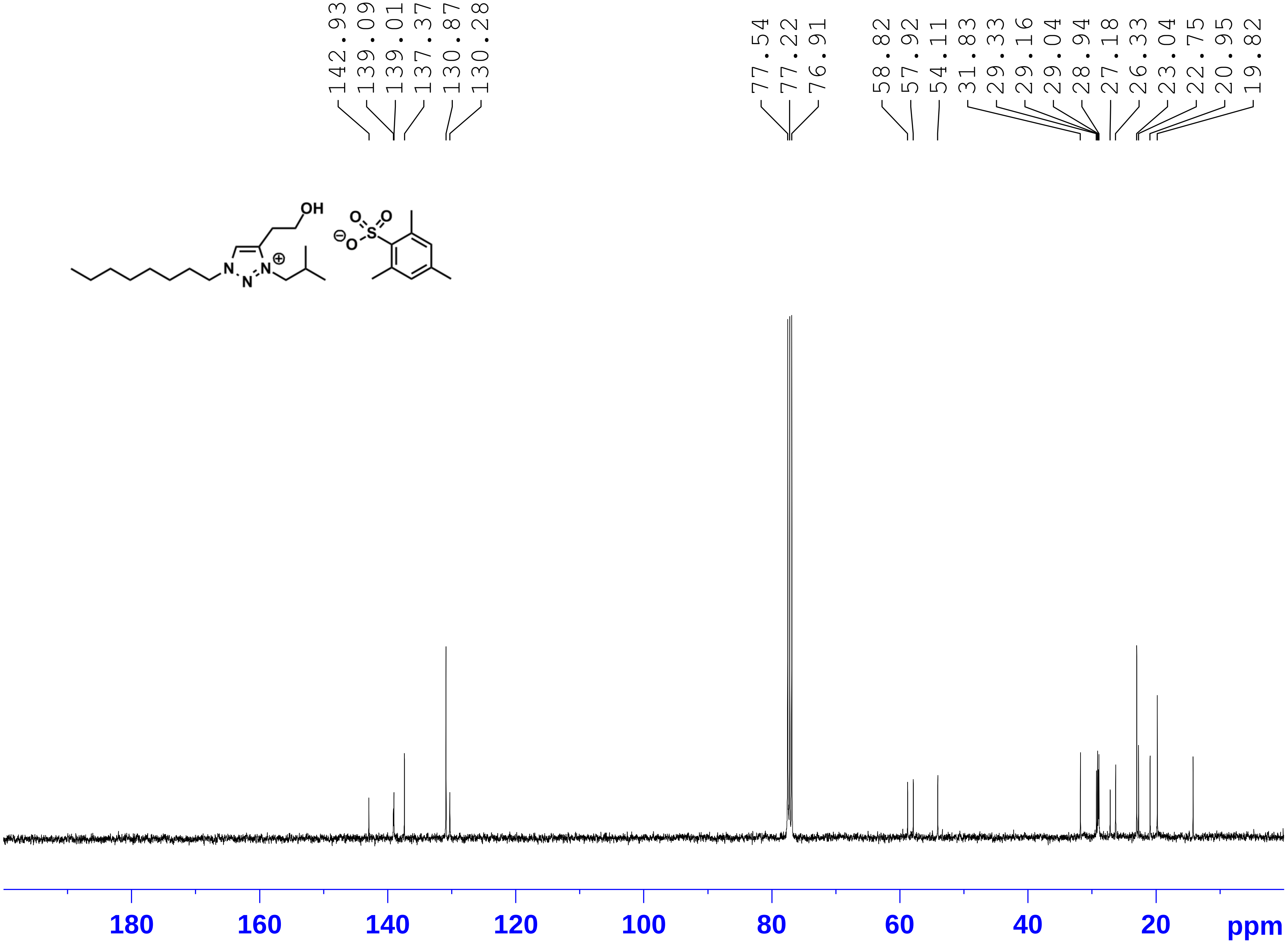
142.93
139.09
139.01
137.37
130.87
130.28
77.54
77.22
76.91
58.82
57.92
54.11
31.83
29.33
29.16
29.04
28.94
27.18
26.33
23.04
22.75
20.95
19.82
OH
O
S
O
O
N
N
N
180
160
140
120
100
80
60
40
20
ppm

Spectrum

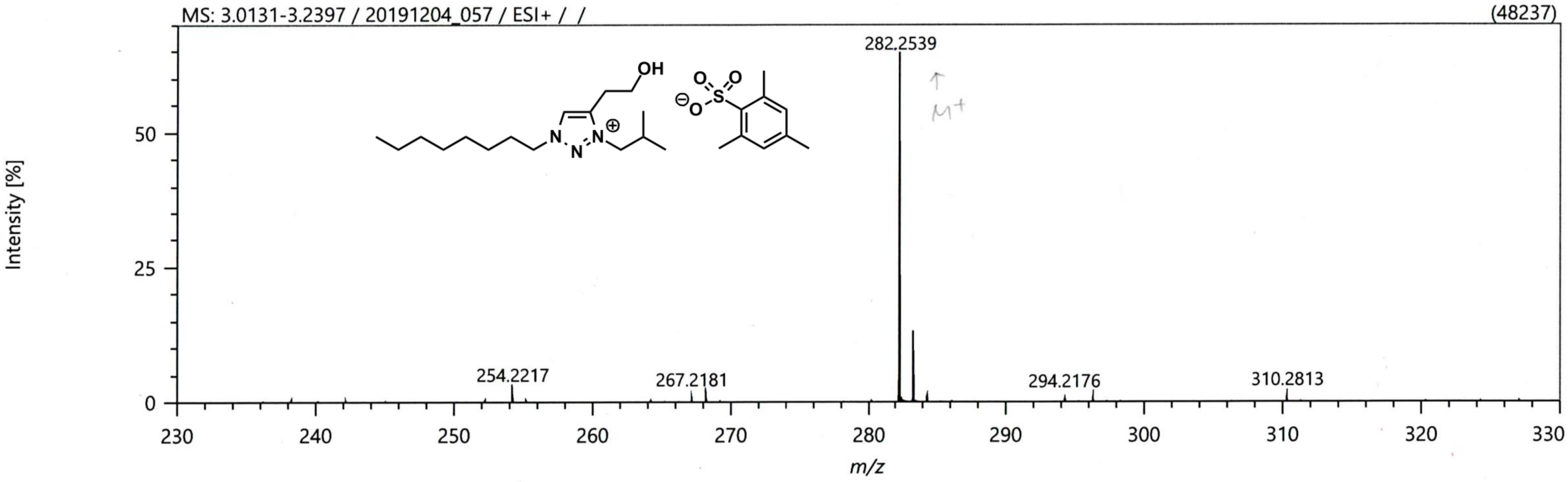

Elemental Composition

Parameters		Elements Set 1:									
Tolerance:	±10.00 ppm	Symbol	C	H	O	Na	N	S	P	Si	F	Cl
Electron:	Odd/Even	Min	0	0	1	0	3	0	0	0	0	0
Charge:	+1	Max	400	1000	1	0	3	0	0	0	0	0
DBE:	-1.5 - 999.0											

Results

Mass	Formula	Calculated Mass	Mass Difference [mDa]	Mass Difference [ppm]	DBE
282.25393	C16 H32 N3 O	282.25399	-0.06	-0.20	2.5
