# Supplementary material for: Combinatorial discovery of small-molecule 1,2,3-triazolium ionic liquids exhibiting lower critical solution temperature phase transition
Source: Sci Rep. 2020 Oct 26;10:18247. doi: 10.1038/s41598-020-75392-z (PMC7589527; doi:10.1038/s41598-020-75392-z)
Supplement: Supplementary file 3 — Supplementary Information 3. [file 41598_2020_75392_MOESM3_ESM.pdf]

Supporting Information (ESI-2) for [R_2-R_3-C3OH-tr][OTs] (**c1**-**c26**) and [R_2-R_3-C3OH-tr][OTMBS] (**d1**-**d26**) Ionic Liquids:

Combinatorial Discovery of Small-Molecule 1,2,3-Triazolium Ionic Liquids Exhibiting Lower Critical Solution Temperature Phase Transition

Yen-Ho Chu*, Mou-Fu Cheng and Yung-Hsin Chiang

* Corresponding author. E-mail: cheyhc@ccu.edu.tw

Department of Chemistry and Biochemistry, National Chung Cheng University, Chiayi 62102, Taiwan, Republic of China

Combinatorial Synthesis of a Library of 26 [R_2-R_3-C3OH-tr][OTs]

Room-Temperature Ionic Liquids

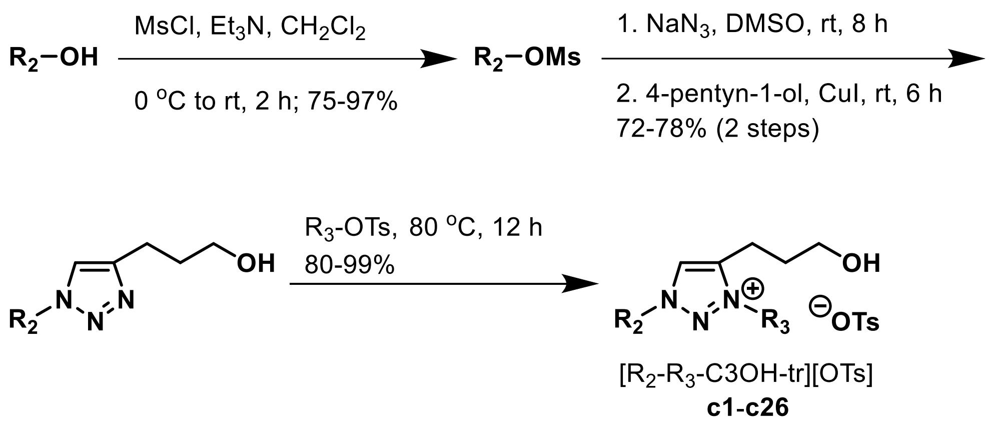

Generalized Procedure for the Synthesis of 1,4-Disubstituted 1,2,3-Triazoles (R_2-C3OH-tr)

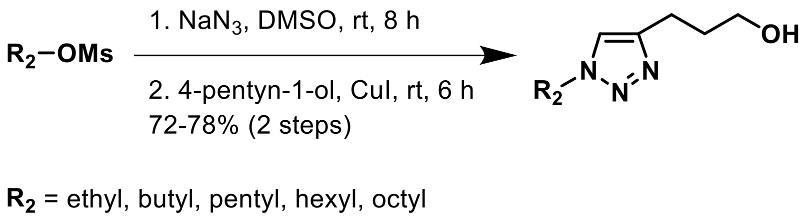

To a round-bottomed flask containing alkyl methanesulfonate (R_2-OMs, 7.9 mmol) in dimethyl sulfoxide (1.0 M) was added sodium azide (15.8 mmol, 2 equiv) and the reaction was allowed to proceed at room temperature for 8 h. The progress of reaction could be monitored by TLC. The resulting reaction mixture was then added copper(I) iodide (0.8 mmol, 0.1 equiv) and 4-pentyn-1-ol (9.5 mmol 1.2 equiv). The subsequent [3+2] cycloaddition reaction was proceeded for 6 h at room temperature. The progress of reaction again was readily monitored by TLC. After reaction completed, crude product was filtered and concentrated under reduced pressure. The residue was purified by column chromatography (ethyl acetate/hexane = 1:5 to 1:0) to finally afford the desired R_2-C3OH-tr.

Et-C3OH-tr: 75% yield, light yellow liquid; ^{1}H NMR (400 MHz, $CDCl_3$) δ 1.55 (t, *J* = 6.0 Hz, NCH_2CH_3, 3H), 1.92-1.98 (m, $CH_2CH_2CH_2OH$, 2H), 2.42 (s, O*H*, 1H), 2.84

(t, J = 8.0 Hz, CH=CCH_2, 2H), 3.72 (t, J = 6.0 Hz, CH_2CH_2OH, 2H), 4.38 (q, J = 6.0 Hz, NCH_2CH_3, 2H), 7.32 (s, C*H*=CCH_2, 1H).

Bu-C3OH-tr: 78% yield, light yellow liquid; ^{1}H NMR (400 MHz, $CDCl_3$) δ 0.96 (t, J = 6.0 Hz, $CH_2CH_2CH_3$, 3H), 1.31-1.40 (m, $CH_2CH_2CH_3$, 2H), 1.84-1.97 (m, 2 x CH_2, 4H), 2.42 (s, O*H*, 1H), 2.84 (t, J = 6.0 Hz, CH=CCH_2, 2H), 3.71 (t, J = 4.0 Hz, CH_2CH_2OH, 2H), 4.32 (t, J = 8.0 Hz, NCH_2CH_2, 2H), 7.29 (s, C*H*=CCH_2, 1H).

Pent-C3OH-tr: 72% yield, light yellow liquid; ^{1}H NMR (400 MHz, $CDCl_3$) δ 0.90 (t, J = 8.0 Hz, $CH_2CH_2CH_3$, 3H), 1.28-1.39 (m, 2 x CH_2, 4H), 1.85-1.98 (m, 2 x CH_2, 4H), 2.32 (s, O*H*, 1H), 2.84 (t, J = 8.0 Hz, CH=CCH_2, 2H), 3.72 (t, J = 6.0 Hz, CH_2CH_2OH, 2H), 4.31 (t, J = 8.0 Hz, NCH_2CH_2, 2H), 7.29 (s, C*H*=CCH_2, 1H).

Hex-C3OH-tr: 73% yield, light yellow liquid; ^{1}H NMR (400 MHz, $CDCl_3$) δ 0.88 (t, J = 8.0 Hz, $CH_2CH_2CH_3$, 3H), 1.30-1.31 (m, 3 x CH_2, 6H), 1.86-1.97 (m, 2 x CH_2, 4H), 2.46 (s, O*H*, 1H), 2.84 (t, J = 8.0 Hz, CH=CCH_2, 2H), 3.72 (t, J = 6.0 Hz, CH_2CH_2OH, 2H), 4.31 (t, J = 8.0 Hz, NCH_2CH_2, 2H), 7.29 (s, C*H*=CCH_2, 1H).

Oct-C3OH-tr: 75% yield, light yellow liquid; ^{1}H NMR (400 MHz, $CDCl_3$) δ 0.88 (t, J = 6.0 Hz, $CH_2CH_2CH_3$, 3H), 1.25-1.31 (m, 5 x CH_2, 10H), 1.85-1.91 (m, NCH_2CH_2, 2H), 1.93-1.98 (m, $CH_2CH_2CH_2$OH, 2H), 2.18 (t, J = 6.0 Hz, O*H*, 1H), 2.84 (t, J = 8.0 Hz, CH=CCH_2, 2H), 3.72 (q, J = 6.0 Hz, CH_2CH_2OH, 2H), 4.31 (t, J = 6.0 Hz, NCH_2, 2H), 7.28 (s, C*H*=CCH_2, 1H).

Generalized Procedure for the Synthesis of [R_2-R_3-C3OH-tr][OTs] Ionic Liquids

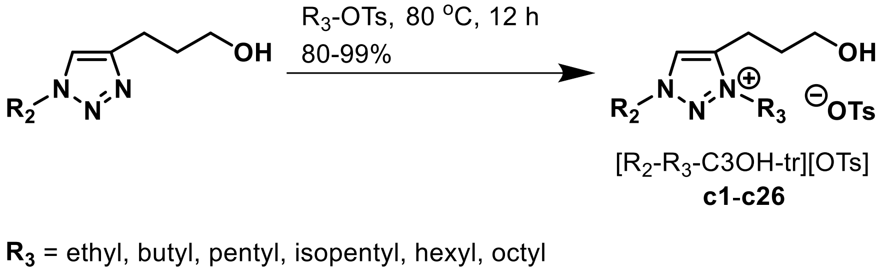

To a round-bottomed flask containing R_2-C3OH-tr (0.6 mmol) was added alkyl 4-methylbenzenesulfonate (R_3-OTs, 0.7 mmol, 1.3 equiv). The mixture was heated at 80 °C for 12 h. The progress of the ionic liquid forming reaction could be readily monitored by TLC. After reaction was completed, the reaction mixture was washed with ether (3 mL, 3 times) and then with *n*-hexane (3 mL, 3 times) to finally afford the desired [R_2-R_3-C3OH-tr][OTs] as room-temperature ionic liquids (**c1**-**c26**).

[Et-Et-C3OH-tr][OTs] (c1): 99% yield, light yellow liquid; ^{1}H NMR (400 MHz, $CDCl_3$) δ 1.57-1.64 (m, 2 x CH_3, 6H), 2.02-2.18 (m, $CH_2CH_2CH_2OH$, 2H), 2.34 (s, *p*-CH_3, 3H), 3.02 (t, *J* = 6.0 Hz, CH=CCH_2, 2H), 3.72 (t, *J* = 6.0 Hz, CH_2CH_2OH, 2H), 4.49 (q, *J* = 8.0 Hz, CNCH_2CH_3, 2H), 4.64 (q, *J* = 8.0 Hz, CHNCH_2CH_3, 2H), 7.15 (d, *J* = 8.0 Hz, aryl H, 2H), 7.73 (d, *J* = 8.0 Hz, aryl H, 2H), 9.09 (s, C*H*=CCH_2, 1H); ^{13}C NMR (100 MHz, $CDCl_3$) δ 14.07, 14.47, 20.22, 21.46, 29.67, 46.54, 49.42, 60.44,

126.04, 128.91, 129.41, 140.05, 142.65, 144.25; EI-HRMS *m/z* [M^+] calcd for $C_9H_{18}N_3O$ 184.1450, found 184.1445.

[Et-Bu-C3OH-tr][OTs] (c2): 97% yield, light yellow liquid; ^{1}H NMR (400 MHz, $CDCl_3$) δ 0.98 (t, *J* = 8.0 Hz, $CH_2CH_2CH_3$, 3H), 1.41 (q, *J* = 8.0 Hz, $CH_2CH_2CH_3$, 2H), 1.63 (t, *J* = 8.0 Hz, NCH_2CH_3, 3H), 1.88-1.96 (m, $CH_2CH_2CH_3$, 2H), 2.04-2.10 (m, $CH_2CH_2CH_2OH$, 2H), 2.34 (s, *p*-CH_3, 3H), 3.01 (t, *J* = 6.0 Hz, CH=CCH_2, 2H), 3.72 (t, *J* = 6.0 Hz, CH_2CH_2OH, 2H), 4.42 (t, *J* = 8.0 Hz, NCH_2CH_2, 2H), 4.69 (q, *J* = 8.0 Hz, NCH_2CH_3, 2H), 7.15 (d, *J* = 8.0 Hz, aryl *H*, 2H), 7.75 (d, *J* = 8.0 Hz, aryl *H*, 2H), 9.20 (s, C*H*=CCH_2, 1H); ^{13}C NMR (100 MHz, $CDCl_3$) δ 13.54, 14.52, 19.72, 20.37, 21.47, 29.72, 30.88, 49.48, 50.87, 60.43, 126.08, 128.89, 129.64, 139.94, 142.85, 144.41; EI-HRMS *m/z* [M^+] calcd for $C_{11}H_{22}N_3O$ 212.1763, found 212.1754.

[Et-Pent-C3OH-tr][OTs] (c3): 87% yield, light yellow liquid; ^{1}H NMR (400 MHz, $CDCl_3$) δ 0.91 (t, *J* = 8.0 Hz, $CH_2CH_2CH_3$, 3H), 1.30-1.39 (m, 2 x CH_2, 4H), 1.62 (t, *J* = 8.0 Hz, NCH_2CH_3 , 3H), 1.89-1.96 (m, NCH_2CH_2 , 2H), 2.03-2.19 (m, $CH_2CH_2CH_2OH$, 2H), 2.34 (s, *p*-CH_3, 3H), 3.00 (t, *J* = 8.0 Hz, CH=CCH_2, 2H), 3.72 (t, *J* = 6.0 Hz, CH_2CH_2OH, 2H), 4.40 (t, *J* = 8.0 Hz, NCH_2CH_2, 2H), 4.67 (q, *J* = 8.0 Hz, NCH_2CH_3, 2H), 7.15 (d, *J* = 8.0 Hz, aryl H, 2H), 7.74 (d, *J* = 8.0 Hz, aryl H, 2H), 9.17 (s, C*H*=CCH_2, 1H); ^{13}C NMR (100 MHz, $CDCl_3$) δ 13.91, 14.53, 20.34, 21.47,

22.14, 28.46, 28.64, 29.71, 49.46, 51.10, 60.41, 126.08, 128.89, 129.60, 139.99, 142.74, 144.38; EI-HRMS *m/z* [M^+] calcd for $C_{12}H_{24}N_3O$ 226.1919, found 226.1913.

[Et-Hex-C3OH-tr][OTs] (c4): 84% yield, light yellow liquid; ^{1}H NMR (400 MHz, $CDCl_3$) δ 0.89 (t, *J* = 8.0 Hz, $CH_2CH_2CH_3$, 3H), 1.28-1.36 (m, 3 x CH_2, 6H), 1.63 (t, *J* = 6.0 Hz, NCH_2CH_3 , 3H), 1.89-1.96 (m, NCH_2CH_2 , 2H), 2.03-2.09 (m, $CH_2CH_2CH_2OH$, 2H), 2.34 (s, *p*-CH_3, 3H), 3.00 (t, *J* = 8.0 Hz, $CH{=}CCH_2$, 2H), 3.72 (t, *J* = 4.0 Hz, CH_2CH_2OH, 2H), 4.40 (t, *J* = 8.0 Hz, NCH_2CH_2, 2H), 4.67 (q, *J* = 8.0 Hz, NCH_2CH_3, 2H), 7.15 (d, *J* = 8.0 Hz, aryl H, 2H), 7.74 (d, *J* = 8.0 Hz, aryl H, 2H), 9.18 (s, $CH{=}CCH_2$, 1H); ^{13}C NMR (100 MHz, $CDCl_3$) δ 14.05, 14.48, 20.28, 21.45, 22.48, 26.06, 28.86, 29.71, 31.14, 49.43, 51.07, 60.41, 126.02, 128.88, 129.46, 139.91, 142.87, 144.35; EI-HRMS *m/z* [M^+] calcd for $C_{13}H_{26}N_3O$ 240.2076, found 240.2066.

[Et-Oct-C3OH-tr][OTs] (c5): 87% yield, light yellow liquid; ^{1}H NMR (400 MHz, $CDCl_3$) δ 0.88 (t, *J* = 8.0 Hz, $CH_2CH_2CH_3$, 3H), 1.27-1.33 (m, 5 x CH_2, 10H), 1.63 (t, *J* = 8.0 Hz, NCH_2CH_3 , 3H), 1.89-1.97 (m, NCH_2CH_2 , 2H), 2.04-2.11 (m, $CH_2CH_2CH_2OH$, 2H), 2.34 (s, *p*-CH_3, 3H), 3.01 (t, *J* = 6.0 Hz, $CH{=}CCH_2$, 2H), 3.73 (t, *J* = 6.0 Hz, CH_2CH_2OH, 2H), 4.40 (t, *J* = 8.0 Hz, NCH_2CH_2, 2H), 4.68 (q, *J* = 8.0 Hz, NCH_2CH_3, 2H), 7.15 (d, *J* = 8.0 Hz, aryl H, 2H), 7.74 (d, *J* = 8.0 Hz, aryl H, 2H), 9.19 (s, $CH{=}CCH_2$, 1H); ^{13}C NMR (100 MHz, $CDCl_3$) δ 14.23, 14.54, 20.37, 21.49, 22.74, 26.44, 28.97, 29.00, 29.13, 29.65, 31.82, 49.48, 51.12, 60.43, 126.12, 128.91,

129.68, 140.10, 142.46, 144.35; EI-HRMS *m/z* [M^+] calcd for $C_{15}H_{30}N_3O$ 268.2389, found 268.2378.

[Bu-Et-C3OH-tr][OTs] (c6): 95% yield, light yellow liquid; ^1H NMR (400 MHz, $CDCl_3$) δ 0.92 (t, *J* = 8.0 Hz, $CH_2CH_2CH_3$, 3H), 1.29-1.38 (m, $CH_2CH_2CH_3$, 2H), 1.59 (t, *J* = 8.0 Hz, NCH_2CH_3, 3H), 1.90-1.97 (m, $CH_2CH_2CH_3$, 2H), 2.03-2.09 (m, $CH_2CH_2CH_2OH$, 2H), 2.34 (s, *p*-CH_3, 3H), 3.03 (t, *J* = 8.0 Hz, CH=CCH_2, 2H), 3.73 (t, *J* = 6.0 Hz, CH_2CH_2OH, 2H), 4.50 (q, *J* = 8.0 Hz, NCH_2CH_3, 2H), 4.57 (q, *J* = 8.0 Hz, NCH_2CH_2, 2H), 7.14 (d, *J* = 8.0 Hz, aryl H, 2H), 7.73 (d, *J* = 8.0 Hz, aryl H, 2H), 9.07 (s, CH=CCH_2, 1H); ^{13}C NMR (100 MHz, $CDCl_3$) δ 13.45, 14.11, 19.56, 20.25, 21.47, 29.68, 31.27, 46.56, 53.75, 60.45, 126.11, 128.89, 129.73, 140.16, 142.37, 144.28; EI-HRMS *m/z* [M^+] calcd for $C_{11}H_{22}N_3O$ 212.1763, found 212.1754.

[Bu-Bu-C3OH-tr][OTs] (c7): 81% yield, light yellow liquid; ^1H NMR (400 MHz, $CDCl_3$) δ 0.91-0.99 (m, 2 x CH_3, 6H), 1.31-1.41 (m, 2 x $CH_2CH_2CH_3$, 4H), 1.88-1.99 (m, 2 x NCH_2CH_2, 4H), 2.04-2.10 (m, $CH_2CH_2CH_2OH$, 2H), 2.34 (s, *p*-CH_3, 3H), 3.02 (t, *J* = 6.0 Hz, CH=CCH_2, 2H), 3.73 (t, *J* = 6.0 Hz, CH_2CH_2OH, 2H), 4.43 (t, *J* = 6.0 Hz, $CNCH_2$, 2H), 4.60 (t, *J* = 8.0 Hz, $CHNCH_2$, 2H), 7.15 (d, *J* = 8.0 Hz, aryl *H*, 2H), 7.74 (d, *J* = 8.0 Hz, aryl *H*, 2H), 9.16 (s, CH=CCH_2, 1H); ^{13}C NMR (100 MHz, $CDCl_3$) δ 13.46, 13.53, 19.57, 19.70, 20.41, 21.47, 29.74, 30.87, 31.30, 50.86, 53.80,

60.49, 126.10, 128.89, 129.93, 139.97, 142.79, 144.45; EI-HRMS *m/z* [M^+] calcd for $C_{13}H_{26}N_3O$ 240.2076, found 240.2068.

[Bu-Pent-C3OH-tr][OTs] (c8): 94% yield, light yellow liquid; ^{1}H NMR (400 MHz, $CDCl_3$) δ 0.89-0.94 (m, 2 x CH_3, 6H), 1.28-1.37 (m, 3 x CH_2, 6H), 1.89-1.98 (m, 2 x NCH_2CH_2, 4H), 2.03-2.09 (m, $CH_2CH_2CH_2OH$, 2H), 2.34 (s, *p*-CH_3, 3H), 3.01 (t, *J* = 8.0 Hz, $CH{=}CCH_2$, 2H), 3.71 (t, *J* = 6.0 Hz, CH_2CH_2OH, 2H), 4.42 (t, *J* = 6.0 Hz, $CNCH_2$, 2H), 4.60 (t, *J* = 8.0 Hz, $CHNCH_2$, 2H), 7.14 (d, *J* = 8.0 Hz, aryl H, 2H), 7.75 (d, *J* = 8.0 Hz, aryl H, 2H), 9.19 (s, $CH{=}CCH_2$, 1H); ^{13}C NMR (100 MHz, $CDCl_3$) δ 13.43, 13.89, 19.52, 20.33, 21.44, 22.11, 28.40, 28.63, 29.78, 31.31, 51.07, 53.73, 60.34, 126.06, 128.81, 129.93, 139.78, 143.04, 144.41; EI-HRMS *m/z* [M^+] calcd for $C_{14}H_{28}N_3O$ 254.2232, found 254.2230.

[Bu-Hex-C3OH-tr][OTs] (c9): 94% yield, light yellow liquid; ^{1}H NMR (400 MHz, $CDCl_3$) δ 0.87-0.95 (m, 2 x CH_3, 6H), 1.28-1.37 (m, 4 x CH_2, 8H), 1.93-2.00 (m, 2 x NCH_2CH_2, 4H), 2.05-2.11 (m, $CH_2CH_2CH_2OH$, 2H), 2.34 (s, *p*-CH_3, 3H), 3.02 (t, *J* = 6.0 Hz, $CH{=}CCH_2$, 2H), 3.75 (t, *J* = 6.0 Hz, CH_2CH_2OH, 2H), 4.42 (t, *J* = 8.0 Hz, $CNCH_2$, 2H), 4.62 (t, *J* = 8.0 Hz, $CHNCH_2$, 2H), 7.15 (d, *J* = 8.0 Hz, aryl *H*, 2H), 7.75 (d, *J* = 8.0 Hz, aryl *H*, 2H), 9.18 (s, $CH{=}CCH_2$, 1H); ^{13}C NMR (100 MHz, $CDCl_3$) δ 13.44, 14.05, 19.53, 20.33, 21.45, 22.51, 26.05, 28.84, 29.76, 31.15, 31.24, 51.05,

53.76, 60.54, 126.06, 128.91, 129.69, 139.96, 142.78, 144.42; EI-HRMS *m/z* [M^+] calcd for $C_{15}H_{28}N_3O$ 268.2383, found 268.2376.

[Bu-Oct-C3OH-tr][OTs] (c10): 99% yield, light yellow liquid; ^{1}H NMR (400 MHz, $CDCl_3$) δ 0.88 (t, *J* = 6.0 Hz, CH_3, 3H), 0.93 (t, *J* = 6.0 Hz, CH_3, 3H), 1.27-1.37 (m, 6 x CH_2, 12H), 1.91-2.00 (m, 2 x NCH_2CH_2, 4H), 2.04-2.10 (m, $CH_2CH_2CH_2OH$, 2H), 2.34 (s, *p*-CH_3, 3H), 3.01 (t, *J* = 6.0 Hz, $CH{=}CCH_2$, 2H), 3.73 (t, *J* = 6.0 Hz, CH_2CH_2OH, 2H), 4.42 (t, *J* = 6.0 Hz, $CNCH_2$, 2H), 4.62 (t, *J* = 8.0 Hz, $CHNCH_2$, 2H), 7.15 (d, *J* = 8.0 Hz, aryl H, 2H), 7.75 (d, *J* = 8.0 Hz, aryl H, 2H), 9.22 (s, $CH{=}CCH_2$, 1H); ^{13}C NMR (100 MHz, $CDCl_3$) δ 13.44, 14.20, 19.53, 20.34, 21.44, 22.71, 26.36, 28.94, 28.96, 29.11, 29.77, 31.31, 31.78, 51.09, 53.73, 60.34, 126.07, 128.80, 129.97, 139.76, 143.06, 144.40; EI-HRMS *m/z* [M^+] calcd for $C_{17}H_{34}N_3O$ 296.2702, found 296.2689.

[Pent-Et-C3OH-tr][OTs] (c11): 96% yield, light yellow liquid; ^{1}H NMR (400 MHz, $CDCl_3$) δ 0.87 (t, *J* = 6.0 Hz, CH_3, 3H), 1.24-1.34 (m, 2 x CH_2, 4H), 1.58 (t, *J* = 8.0 Hz, CH_3, 3H), 1.90-2.00 (m, 2 x NCH_2CH_2, 4H), 2.03-2.09 (m, $CH_2CH_2CH_2OH$, 2H), 2.34 (s, *p*-CH_3, 3H), 3.03 (t, *J* = 8.0 Hz, $CH{=}CCH_2$, 2H), 3.73 (t, *J* = 4.0 Hz, CH_2CH_2OH, 2H), 4.47-4.55 (m, 2 x NCH_2, 4H), 7.14 (d, *J* = 8.0 Hz, aryl H, 2H), 7.72 (d, *J* = 8.0 Hz, aryl H, 2H), 9.01 (s, $CH{=}CCH_2$, 1H); ^{13}C NMR (100 MHz, $CDCl_3$) δ 13.92, 14.08, 20.25, 21.48, 22.07, 28.34, 29.05, 29.61, 46.56, 53.98, 60.54, 126.13,

128.95, 129.62, 140.32, 142.08, 144.26; EI-HRMS *m/z* [M^+] calcd for $C_{12}H_{24}N_3O$ 226.1919, found 226.1918.

[Pent-Bu-C3OH-tr][OTs] (c12): 92% yield, light yellow liquid; ^{1}H NMR (400 MHz, $CDCl_3$) δ 0.87 (t, *J* = 6.0 Hz, CH_3, 3H), 0.96 (t, *J* = 8.0 Hz, CH_3, 3H), 1.23-1.39 (m, 3 x CH_2, 6H), 1.86-1.99 (m, 2 x NCH_2CH_2, 4H), 2.03-2.09 (m, $CH_2CH_2CH_2OH$, 2H), 2.34 (s, *p*-CH_3, 3H), 3.01 (t, *J* = 8.0 Hz, $CH{=}CCH_2$, 2H), 3.72 (t, *J* = 6.0 Hz, CH_2CH_2OH, 2H), 4.43 (t, *J* = 6.0 Hz, $CNCH_2$, 2H), 4.57 (t, *J* = 8.0 Hz, $CHNCH_2$, 2H), 7.14 (d, *J* = 8.0 Hz, aryl H, 2H), 7.74 (d, *J* = 8.0 Hz, aryl H, 2H), 9.13 (s, $CH{=}CCH_2$, 1H); ^{13}C NMR (100 MHz, $CDCl_3$) δ 13.51, 13.92, 19.65, 20.35, 21.45, 22.06, 28.32, 29.09, 29.73, 30.84, 50.85, 53.97, 60.42, 126.09, 128.86, 129.84, 139.95, 142.71, 144.41; EI-HRMS *m/z* [M^+] calcd for $C_{14}H_{28}N_3O$ 254.2232, found 254.2221.

[Pent-Pent-C3OH-tr][OTs] (c13): 89% yield, light yellow liquid; ^{1}H NMR (400 MHz, $CDCl_3$) δ 0.86-0.93 (m, 2 x CH_3, 6H), 1.28-1.38 (m, 4 x CH_2, 8H), 1.91-2.02 (m, 2 x NCH_2CH_2, 4H), 2.06-2.10 (m, $CH_2CH_2CH_2OH$, 2H), 2.34 (s, *p*-CH_3, 3H), 3.02 (t, *J* = 6.0 Hz, $CH{=}CCH_2$, 2H), 3.73 (t, *J* = 4.0 Hz, CH_2CH_2OH, 2H), 4.42 (t, *J* = 8.0 Hz, $CNCH_2$, 2H), 4.61 (t, *J* = 6.0 Hz, $CHNCH_2$, 2H), 7.15 (d, *J* = 8.0 Hz, aryl *H*, 2H), 7.76 (d, *J* = 8.0 Hz, aryl *H*, 2H), 9.20 (s, $CH{=}CCH_2$, 1H); ^{13}C NMR (100 MHz, $CDCl_3$) δ 13.93, 13.94, 20.45, 21.47, 22.09, 22.15, 28.37, 28.48, 28.65, 29.10, 29.80,

51.07, 54.07, 60.53, 126.08, 128.89, 129.94, 139.87, 142.99, 144.44; EI-HRMS *m/z* [M^+] calcd for $C_{15}H_{30}N_3O$ 268.2389, found 268.2387.

[Pent-Hex-C3OH-tr][OTs] (c14): 80% yield, light yellow liquid; ^1H NMR (400 MHz, $CDCl_3$) δ 0.86-0.91 (m, 2 x CH_3, 6H), 1.28-1.32 (m, 5 x CH_2, 10H), 1.93-2.02 (m, 2 x NCH_2CH_2, 4H), 2.05-2.11 (m, $CH_2CH_2CH_2OH$, 2H), 2.34 (s, *p*-CH_3, 3H), 3.02 (t, *J* = 6.0 Hz, CH=CCH_2, 2H), 3.74 (t, *J* = 6.0 Hz, CH_2CH_2OH, 2H), 4.42 (t, *J* = 8.0 Hz, $CNCH_2$, 2H), 4.61 (t, *J* = 8.0 Hz, $CHNCH_2$, 2H), 7.15 (d, *J* = 8.0 Hz, aryl *H*, 2H), 7.76 (d, *J* = 8.0 Hz, aryl *H*, 2H), 9.20 (s, C*H*=CCH_2, 1H); ^{13}C NMR (100 MHz, $CDCl_3$) δ 13.94, 14.06, 20.49, 21.48, 22.11, 22.52, 26.10, 28.38, 28.95, 29.15, 29.78, 31.16, 51.12, 54.08, 60.48, 126.11, 128.87, 130.10, 139.82, 143.06, 144.43; EI-HRMS *m/z* [M^+] calcd for $C_{16}H_{32}N_3O$ 282.2545, found 282.2536.

[Pent-Oct-C3OH-tr][OTs] (c15): 88% yield, light yellow liquid; ^1H NMR (400 MHz, $CDCl_3$) δ 0.86-0.90 (m, 2 x CH_3, 6H), 1.27-1.32 (m, 7 x CH_2, 10H), 1.91-2.01 (m, 2 x NCH_2CH_2, 4H), 2.04-2.10 (m, $CH_2CH_2CH_2OH$, 2H), 2.34 (s, *p*-CH_3, 3H), 3.01 (t, *J* = 6.0 Hz, CH=CCH_2, 2H), 3.73(t, *J* = 4.0 Hz, CH_2CH_2OH, 2H), 4.42 (t, *J* = 8.0 Hz, $CNCH_2$, 2H), 4.60 (t, *J* = 6.0 Hz, $CHNCH_2$, 2H), 7.15 (d, *J* = 8.0 Hz, aryl H, 2H), 7.75 (d, *J* = 8.0 Hz, aryl H, 2H), 9.19 (s, C*H*=CCH_2, 1H); ^{13}C NMR (100 MHz, $CDCl_3$) δ 13.93, 14.21, 20.40, 21.47, 22.08, 22.73, 26.40, 28.35, 28.95, 29.00, 29.13, 29.14,

29.74, 31.81, 51.11, 54.01, 60.43, 126.11, 128.86, 129.97, 139.89, 142.83, 144.40; EI-HRMS *m/z* [M^+] calcd for $C_{18}H_{36}N_3O$ 310.2858, found 310.2852.

[Hex-Et-C3OH-tr][OTs] (c16): 99% yield, light yellow liquid; ^{1}H NMR (400 MHz, $CDCl_3$) δ 0.84-0.87 (m, CH_3, 3H), 1.26-1.28 (m, 3 x CH_2, 6H), 1.58 (t, *J* = 8.0 Hz, CH_3, 3H), 1.90-1.97 (m, $NCH_2C\mathit{H}_2$, 2H), 2.04-2.10 (m, $CH_2C\mathit{H}_2CH_2OH$, 2H), 2.34 (s, *p*-CH_3, 3H), 3.02 (t, *J* = 6.0 Hz, $CH{=}CC\mathit{H}_2$, 2H), 3.75 (m, $CH_2C\mathit{H}_2OH$, 2H), 4.47-4.55 (m, $CNC\mathit{H}_2$ + $CHNC\mathit{H}_2$, 4H), 7.14 (d, *J* = 8.0 Hz, aryl H, 2H), 7.72 (d, *J* = 8.0 Hz, aryl H, 2H), 9.00 (s, $C\mathit{H}{=}CCH_2$, 1H); ^{13}C NMR (100 MHz, $CDCl_3$) δ 14.09, 14.09, 20.26, 21.50, 22.52, 25.99, 29.33, 29.65, 31.12, 46.58, 54.02, 60.58, 126.17, 128.98, 129.60, 140.25, 142.08, 144.28; EI-HRMS *m/z* [M^+] calcd for $C_{13}H_{26}N_3O$ 240.2076, found 240.2065.

[Hex-Bu-C3OH-tr][OTs] (c17): 93% yield, light yellow liquid; ^{1}H NMR (400 MHz, $CDCl_3$) δ 0.86 (t, *J* = 6.0 Hz, CH_3, 3H), 0.97 (t, *J* = 6.0 Hz, CH_3, 3H), 1.28-1.41 (m, 4 x CH_2, 8H), 1.88-1.98 (m, 2 x $NCH_2C\mathit{H}_2$, 4H), 2.08 (m, $CH_2C\mathit{H}_2CH_2OH$, 2H), 2.34 (s, *p*-$C\mathit{H}_3$, 3H), 3.02 (t, *J* = 8.0 Hz, $CH{=}CC\mathit{H}_2$, 2H), 3.75 (m, $CH_2C\mathit{H}_2OH$, 2H), 4.43 (t, *J* = 8.0 Hz, $CNC\mathit{H}_2$, 2H), 4.59 (t, *J* = 8.0 Hz, $CHNC\mathit{H}_2$, 2H), 7.15 (d, *J* = 8.0 Hz, aryl *H*, 2H), 7.75 (d, *J* = 8.0 Hz, aryl *H*, 2H), 9.16 (s, $C\mathit{H}{=}CCH_2$, 1H); ^{13}C NMR (100 MHz, $CDCl_3$) δ 13.54, 14.08, 19.74, 20.54, 21.50, 22.54, 26.01, 29.40, 29.75, 30.93,

31.13, 50.87, 54.14, 60.54, 126.14, 128.90, 130.12, 139.93, 142.85, 144.45; EI-HRMS *m/z* [M^+] calcd for $C_{15}H_{30}N_3O$ 268.2389, found 268.2384.

[Hex-Pent-C3OH-tr][OTs] (c18): 89% yield, light yellow liquid; ^{1}H NMR (400 MHz, $CDCl_3$) δ 0.87-0.93 (m, 2 x CH_3, 6H), 1.29-1.38 (m, 5 x CH_2, 10H), 1.93-1.97 (m, 2 x $NCH_2C\mathit{H_2}$, 4H), 2.08 (m, $CH_2C\mathit{H_2}CH_2OH$, 2H), 2.34 (s, *p*-$C\mathit{H_3}$, 3H), 3.02 (t, *J* = 6.0 Hz, CH=$CC\mathit{H_2}$, 2H), 3.74 (m, $CH_2C\mathit{H_2}OH$, 2H), 4.42 (t, *J* = 6.0 Hz, $CNC\mathit{H_2}$, 2H), 4.61 (t, *J* = 8.0 Hz, $CHNC\mathit{H_2}$, 2H), 7.15 (d, *J* = 8.0 Hz, aryl *H*, 2H), 7.76 (d, *J* = 8.0 Hz, aryl *H*, 2H), 9.23 (s, C*H*=CCH_2, 1H); ^{13}C NMR (100 MHz, $CDCl_3$) δ 13.92, 14.08, 20.55, 21.49, 22.16, 22.54, 26.01, 28.50, 28.71, 29.41, 29.77, 31.13, 51.10, 54.15, 60.52, 126.14, 128.88, 130.18, 139.86, 143.01, 144.44; EI-HRMS *m/z* [M^+] calcd for $C_{16}H_{32}N_3O$ 282.2545, found 282.2548.

[Hex-Hex-C3OH-tr][OTs] (c19): 92% yield, light yellow liquid; ^{1}H NMR (400 MHz, $CDCl_3$) δ 0.85-0.90 (m, 2 x CH_3, 6H), 1.28-1.31 (m, 6 x CH_2, 12H), 1.91-1.98 (m, 2 x $NCH_2C\mathit{H_2}$, 4H), 2.08 (m, $CH_2C\mathit{H_2}CH_2OH$, 2H), 2.34 (s, *p*-$C\mathit{H_3}$, 3H), 3.02 (t, *J* = 6.0 Hz, CH=$CC\mathit{H_2}$, 2H), 3.74 (m, $CH_2C\mathit{H_2}OH$, 2H), 4.42 (t, *J* = 8.0 Hz, $CNC\mathit{H_2}$, 2H), 4.59 (t, *J* = 8.0 Hz, $CHNC\mathit{H_2}$, 2H), 7.15 (d, *J* = 8.0 Hz, aryl *H*, 2H), 7.75 (d, *J* = 8.0 Hz, aryl *H*, 2H), 9.18 (s, C*H*=CCH_2, 1H)); ^{13}C NMR (100 MHz, $CDCl_3$) δ 14.07, 14.08, 20.43, 21.47, 22.52, 22.53, 25.98, 26.08, 28.90, 29.35, 29.78, 31.12, 31.16,

51.08, 54.08, 60.56, 126.09, 128.91, 129.85, 139.93, 142.84, 144.42; EI-HRMS *m/z* [M^+] calcd for $C_{17}H_{34}N_3O$ 296.2702, found 296.2693.

[Hex-Oct-C3OH-tr][OTs] (c20): 83% yield, light yellow liquid; ^1H NMR (400 MHz, $CDCl_3$) δ 0.85-0.90 (m, 2 x CH_3 , 6H), 1.27-1.34 (m, 8 x CH_2, 16H), 1.93-2.00 (m, 2 x NCH_2CH_2 , 4H), 2.08 (m, $CH_2CH_2CH_2OH$, 2H), 2.34 (s, *p*-CH_3, 3H), 3.02 (t, *J* = 6.0 Hz, $CH=CCH_2$, 2H), 3.75 (m, CH_2CH_2OH, 2H), 4.42 (t, *J* = 8.0 Hz, $CNCH_2$, 2H), 4.62 (t, *J* = 6.0 Hz, $CHNCH_2$, 2H), 7.15 (d, *J* = 8.0 Hz, aryl H, 2H), 7.76 (d, *J* = 8.0 Hz, aryl H, 2H), 9.23 (s, $CH=CCH_2$, 1H); ^{13}C NMR (100 MHz, $CDCl_3$) δ 14.08, 14.22, 20.39, 21.47, 22.52, 22.75, 25.97, 26.41, 28.94, 29.01, 29.15, 29.36, 29.78, 31.11, 31.83, 51.07, 54.03, 60.48, 126.06, 128.87, 129.85, 139.84, 142.96, 144.39; EI-HRMS *m/z* [M^+] calcd for $C_{19}H_{38}N_3O$ 324.3015, found 324.3005.

[Oct-Et-C3OH-tr][OTs] (c21): 99% yield, light yellow liquid; ^1H NMR (400 MHz, $CDCl_3$) δ 0.87 (t, *J* = 8.0 Hz, CH_3, 3H), 1.24-1.28 (m, 5 x CH_2, 10H), 1.59 (t, *J* = 6.0 Hz, CH_3, 3H), 1.95 (m, NCH_2CH_2 , 2H), 2.08 (m, $CH_2CH_2CH_2OH$, 2H), 2.34 (s, *p*-CH_3, 3H), 3.05 (m, $CH=CCH_2$, 2H), 3.80 (m, CH_2CH_2OH, 2H), 4.50 (t, *J* = 6.0 Hz, $CNCH_2$, 2H), 4.56 (t, *J* = 8.0 Hz, $CHNCH_2$, 2H), 7.15 (d, *J* = 8.0 Hz, aryl H, 2H), 7.73 (d, *J* = 8.0 Hz, aryl H, 2H), 9.09 (s, $CH=CCH_2$, 1H); ^{13}C NMR (100 MHz, $CDCl_3$) δ 14.10, 14.21, 20.26, 21.44, 22.73, 26.30, 26.31, 28.94, 29.13, 29.39, 31.83, 46.55,

53.98, 60.46, 126.11, 128.87, 129.70, 139.99, 142.86, 144.29; EI-HRMS *m/z* [M^+] calcd for $C_{15}H_{30}N_3O$ 268.2389, found 268.2384.

[Oct-Bu-C3OH-tr][OTs] (c22): 86% yield, light yellow liquid; ^{1}H NMR (400 MHz, $CDCl_3$) δ 0.87 (t, *J* = 8.0 Hz, CH_3, 3H), 0.98 (t, *J* = 8.0 Hz, CH_3, 3H), 1.24-1.28 (m, 5 x CH_2, 10H), 1.34-1.44 (m, CH_2, 2H), 1.89-1.97 (m, 2 x NCH_2CH_2, 4H), 2.10 (m, $CH_2CH_2CH_2OH$, 2H), 2.34 (s, *p*-CH_3, 3H), 3.03 (m, $CH{=}CCH_2$, 2H), 3.08 (m, CH_2CH_2OH, 2H), 4.43 (t, *J* = 6.0 Hz, $CNCH_2$, 2H), 4.60 (t, *J* = 8.0 Hz, $CHNCH_2$, 2H), 7.15 (d, *J* = 8.0 Hz, aryl H, 2H), 7.75 (d, *J* = 8.0 Hz, aryl H, 2H), 9.21 (s, $CH{=}CCH_2$, 1H); ^{13}C NMR (100 MHz, $CDCl_3$) δ 13.52, 14.22, 19.67, 20.41, 21.45, 22.75, 26.30, 26.30, 28.95, 29.16, 29.44, 30.88, 31.83, 50.86, 54.02, 60.42, 126.12, 128.83, 129.96, 139.77, 143.31, 144.46; EI-HRMS *m/z* [M^+] calcd for $C_{17}H_{34}N_3O$ 296.2702, found 296.2700.

[Oct-Pent-C3OH-tr][OTs] (c23): 98% yield, light yellow liquid; ^{1}H NMR (400 MHz, $CDCl_3$) δ 0.87 (t, *J* = 8.0 Hz, CH_3, 3H), 0.92 (t, *J* = 6.0 Hz, CH_3, 3H), 1.24-1.37 (m, 7 x CH_2, 16H), 1.93-1.99 (m, 2 x NCH_2CH_2, 4H), 2.10 (m, $CH_2CH_2CH_2OH$, 2H), 2.34 (s, *p*-CH_3, 3H), 3.03 (m, $CH{=}CCH_2$, 2H), 3.80 (m, CH_2CH_2OH, 2H), 4.42 (t, *J* = 6.0 Hz, $CNCH_2$, 2H), 4.61 (t, *J* = 8.0 Hz, $CHNCH_2$, 2H), 7.15 (d, *J* = 8.0 Hz, aryl H, 2H), 7.76 (d, *J* = 8.0 Hz, aryl H, 2H), 9.23 (s, $CH{=}CCH_2$, 1H); ^{13}C NMR (100 MHz, $CDCl_3$) δ 13.90, 14.21, 20.39, 21.44, 22.13, 22.74, 26.30, 26.31, 28.43, 28.64, 28.95,

29.16, 29.43, 31.83, 51.08, 54.01, 60.39, 126.11, 128.82, 129.95, 139.73, 143.32, 144.44; EI-HRMS *m/z* [M^+] calcd for $C_{18}H_{36}N_3O$ 310.2858, found 310.2846.

[Oct-Hex-C3OH-tr][OTs] (c24): 90% yield, light yellow liquid; ^{1}H NMR (400 MHz, $CDCl_3$) δ 0.85-0.89 (m, 2 x CH_3 , 6H), 1.24-1.32 (m, 8 x CH_2, 16H), 1.92-1.99 (m, 2 x $NCH_2C\mathit{H_2}$, 4H), 2.10 (m, $CH_2C\mathit{H_2}CH_2OH$, 2H), 2.34 (s, *p*-CH_3, 3H), 3.03 (m, $CH{=}CC\mathit{H_2}$, 2H), 3.80 (m, $CH_2C\mathit{H_2}OH$, 2H), 4.42 (t, *J* = 8.0 Hz, $CNC\mathit{H_2}$, 2H), 4.60 (t, *J* = 6.0 Hz, $CHNC\mathit{H_2}$, 2H), 7.15 (d, *J* = 8.0 Hz, aryl H, 2H), 7.75 (d, *J* = 8.0 Hz, aryl H, 2H), 9.22 (s, $C\mathit{H}{=}CCH_2$, 1H); ^{13}C NMR (100 MHz, $CDCl_3$) δ 14.05, 14.22, 20.43, 21.46, 22.50, 22.75, 26.06, 26.32, 26.32, 28.92, 28.97, 29.17, 29.45, 31.15, 31.85, 51.11, 54.04, 60.42, 126.14, 128.84, 130.02, 139.80, 143.28, 144.42; EI-HRMS *m/z* [M^+] calcd for $C_{19}H_{38}N_3O$ 324.3015, found 324.3010.

[Oct-Oct-C3OH-tr][OTs] (c25): 87% yield, light yellow liquid; ^{1}H NMR (400 MHz, $CDCl_3$) δ 0.86-0.89 (m, 2 x CH_3 , 6H), 1.26-1.32 (m, 10 x CH_2, 20H), 1.95-2.01 (m, 2 x $NCH_2C\mathit{H_2}$, 4H), 2.34 (s, *p*-CH_3, 3H), 3.08 (t, *J* = 6.0 Hz, $CH{=}CC\mathit{H_2}$, 2H), 4.09 (t, *J* = 6.0 Hz, $CH_2C\mathit{H_2}OH$, 2H), 4.47 (t, *J* = 6.0 Hz, $CNC\mathit{H_2}$, 2H), 4.61 (t, *J* = 6.0 Hz, $CHNC\mathit{H_2}$, 2H), 7.14 (d, *J* = 8.0 Hz, aryl H, 2H), 7.73 (d, *J* = 8.0 Hz, aryl H, 2H), 9.05 (s, $C\mathit{H}{=}CCH_2$, 1H); ^{13}C NMR (100 MHz, $CDCl_3$) δ 14.23,14.24, 21.48, 22.76, 22.77, 26.36, 26.43, 26.44, 27.07, 28.99, 29.03, 29.04, 29.17, 29.19, 29.32, 31.85, 31.87,

51.33, 54.09, 59.02, 126.09, 128.92, 130.11, 139.98, 142.53, 142.72; EI-HRMS *m/z* [M^+] calcd for $C_{21}H_{42}N_3O$ 352.3328, found 352.3320.

[Hex-*i*-Pent-C3OH-tr][OTs] (c26): 87% yield, light yellow liquid; ^{1}H NMR (400 MHz, $CDCl_3$) δ 0.85-0.89 (m, CH_3 , 3H), 0.99 (d, *J* = 4.0 Hz, 2 x CHC*H*$_3$, 6H), 1.28-1.29 (m, 3 x CH_2, 6H), 1.58-1.68 (m, C*H*CH_3, 1H), 1.80-1.86 (m, NCH_2C*H*$_2$CH, 2H), 1.92-1.99 (m, NCH_2C*H*$_2$$CH_2$, 2H), 2.07-2.10 (m, CH_2C*H*$_2$$CH_2$OH, 2H), 2.34 (s, *p*-C*H*$_3$, 3H), 3.02 (t, *J* = 6.0 Hz, CH=CC*H*$_2$, 2H), 3.75 (m, CH_2C*H*$_2$OH, 2H), 4.44 (t, *J* = 8.0 Hz, CNC*H*$_2$, 2H), 4.61 (t, *J* = 8.0 Hz, CHNC*H*$_2$, 2H), 7.15 (d, *J* = 8.0 Hz, aryl *H*, 2H), 7.76 (d, *J* = 8.0 Hz, aryl *H*, 2H), 9.22 (s, C*H*=CCH_2, 1H); ^{13}C NMR (100 MHz, $CDCl_3$) δ 14.07, 20.41, 21.46, 22.25, 22.53, 25.73, 25.97, 29.34, 29.78, 31.11, 37.57, 49.56, 54.06, 60.53, 126.07, 128.89, 129.88, 139.87, 142.96, 144.37; EI-HRMS *m/z* [M^+] calcd for $C_{16}H_{32}N_3O$ 282.2545, found 282.2545.

Combinatorial Synthesis of a Library of 26 [R_2-R_3-C3OH-tr][OTMBS] Ionic Liquids

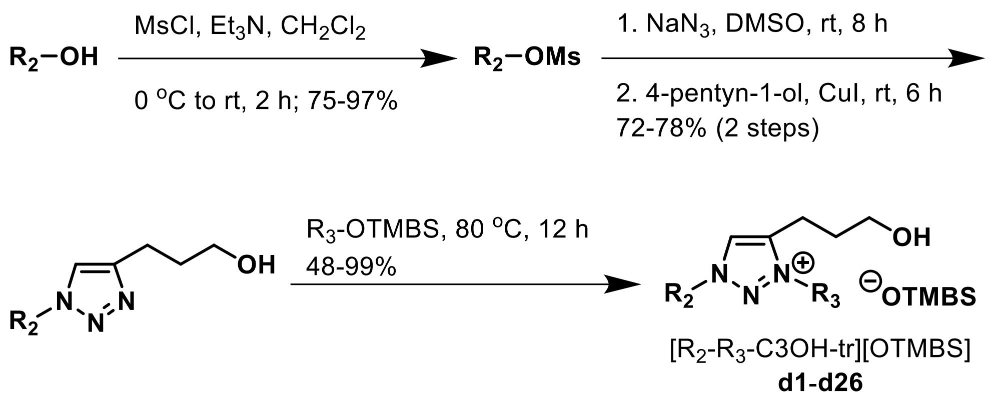

Generalized Procedure for the Synthesis of [R_2-R_3-C3OH-tr][OTMBS] Ionic Liquids

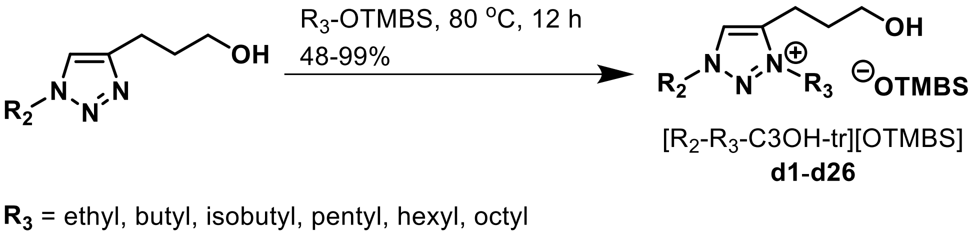

To a round-bottomed flask containing R_2-C3OH-tr (0.6 mmol) was added alkyl 2,4,6-trimethylbenzenesulfonate (R_3-OTMBS, 0.7 mmol, 1.3 equiv). The mixture was heated at 80 °C for 12 h. The progress of the ionic liquid forming reaction could be readily monitored by TLC. After reaction was completed, the reaction mixture was washed with ether (3 mL, 3 times) and then with *n*-hexane (3 mL, 3 times) to finally afford the desired [R_2-R_3-C3OH-tr][OTMBS] as ionic liquids (**d1-d26**).

[Et-Et-C3OH-tr][OTMBS] (d1): 99% yield, light yellow liquid; ^{1}H NMR (400 MHz, $CDCl_3$) δ 1.59-1.66 (m, 2 x CH_3, 6H), 2.03-2.09 (m, $CH_2CH_2CH_2OH$, 2H), 2.22 (s, *p*-CH_3, 3H), 2.64 (s, 2 x *o*-CH_3, 6H), 3.02 (t, *J* = 6.0 Hz, CH=CCH_2, 2H), 3.72 (t, *J* = 6.0 Hz, CH_2CH_2OH, 2H), 4.49 (q, *J* = 8.0 Hz, CNCH_2CH_3, 2H), 4.69 (q, *J* = 8.0 Hz, CHNCH_2CH_3, 2H), 6.81 (s, aryl H, 2H), 9.27 (s, C*H*=CCH_2, 1H); ^{13}C NMR (100 MHz, $CDCl_3$) δ 14.13, 14.55, 20.31, 20.93, 23.11, 29.73, 46.51, 49.45, 60.32, 129.79,

130.78, 137.13, 138.53, 140.16, 144.30; EI-HRMS *m/z* [M^+] calcd for $C_9H_{18}N_3O$ 184.1419, found 184.1448.

[Et-Bu-C3OH-tr][OTMBS] (d2): 56% yield, light yellow solid; mp 65-66 ^oC; ^1H NMR (400 MHz, $CDCl_3$) δ 0.99 (t, *J* = 6.0 Hz, $CH_2CH_2CH_3$, 3H), 1.41 (q, *J* = 8.0 Hz, $CH_2CH_2CH_3$, 2H), 1.66 (t, *J* = 8.0 Hz, NCH_2CH_3 , 3H), 1.90-1.98 (m, $CH_2CH_2CH_3$, 2H), 2.05-2.11 (m, $CH_2CH_2CH_2OH$, 2H), 2.22 (s, *p*-CH_3, 3H), 2.68 (s, 2 x *o*-CH_3, 6H), 3.02 (t, *J* = 6.0 Hz, CH=CCH_2, 2H), 3.72 (t, *J* = 6.0 Hz, CH_2CH_2OH, 2H), 4.41 (t, *J* = 6.0 Hz, NCH_2CH_2, 2H), 4.73 (q, *J* = 8.0 Hz, NCH_2CH_3, 2H), 6.82 (s, aryl *H*, 2H), 9.44 (s, C*H*=CCH_2, 1H); ^{13}C NMR (100 MHz, $CDCl_3$) δ 13.53, 14.58, 19.74, 20.44, 20.91, 23.17, 29.79, 30.94, 49.49, 50.82, 60.31, 130.04, 130.72, 137.06, 138.19, 140.73, 144.42; EI-HRMS *m/z* [M^+] calcd for $C_{11}H_{22}N_3O$ 212.1763, found 212.1759.

[Et-Pent-C3OH-tr][OTMBS] (d3): 77% yield, light yellow solid; mp 67-68 ^oC; ^1H NMR (400 MHz, $CDCl_3$) δ 0.92 (t, *J* = 8.0 Hz, CH_3 , 3H), 1.32-1.39 (m, 2 x CH_2 , 4H), 1.65 (t, *J* = 8.0 Hz, NCH_2CH_3 , 3H), 1.91-1.99 (m, NCH_2CH_2 , 2H), 2.04-2.10 (m, $CH_2CH_2CH_2OH$, 2H), 2.22 (s, *p*-CH_3, 3H), 2.65 (s, 2 x *o*-CH_3, 6H), 3.01 (t, *J* = 6.0 Hz, CH=CCH_2, 2H), 3.72 (t, *J* = 6.0 Hz, CH_2CH_2OH, 2H), 4.40 (t, *J* = 8.0 Hz, NCH_2CH_2, 2H), 4.71 (q, *J* = 8.0 Hz, NCH_2CH_3, 2H), 6.82 (s, aryl H, 2H), 9.39 (s, C*H*=CCH_2, 1H); ^{13}C NMR (100 MHz, $CDCl_3$) δ 13.92, 14.62, 20.46, 20.94, 22.16,

23.15, 28.50, 28.74, 29.73, 49.51, 51.07, 60.28, 130.11, 130.78, 137.14, 138.43, 140.32, 144.39; EI-HRMS *m/z* [M^+] calcd for $C_{12}H_{24}N_3O$ 226.1919, found 226.1919.

[Et-Hex-C3OH-tr][OTMBS] (d4): 76% yield, light yellow solid; mp 68-69 °C; ^{1}H NMR (400 MHz, $CDCl_3$) δ 0.89 (t, *J* = 8.0 Hz, $CH_2CH_2CH_3$, 3H), 1.29-1.36 (m, 3 x CH_2, 6H), 1.63 (t, *J* = 6.0 Hz, NCH_2CH_3 , 3H), 1.88-1.96 (m, NCH_2CH_2, 2H), 2.02-2.09 (m, $CH_2CH_2CH_2OH$, 2H), 2.22 (s, *p*-CH_3, 3H), 2.65 (s, 2 x *o*-CH_3, 6H), 3.00 (t, *J* = 6.0 Hz, $CH{=}CCH_2$, 2H), 3.70 (t, *J* = 6.0 Hz, CH_2CH_2OH, 2H), 4.40 (t, *J* = 6.0 Hz, NCH_2CH_2, 2H), 4.70 (q, *J* = 8.0 Hz, NCH_2CH_3, 2H), 6.81 (s, aryl H, 2H), 9.36 (s, $CH{=}CCH_2$, 1H); ^{13}C NMR (100 MHz, $CDCl_3$) δ 14.03, 14.59, 20.30, 20.88, 22.46, 23.13, 26.04, 28.92, 29.77, 31.11, 49.39, 51.05, 60.14, 129.85, 130.70, 137.02, 138.25, 140.56, 144.36; EI-HRMS *m/z* [M^+] calcd for $C_{13}H_{26}N_3O$ 240.2076, found 240.2071.

[Et-Oct-C3OH-tr][OTMBS] (d5): 88% yield, light yellow solid; mp 70-71 °C; ^{1}H NMR (400 MHz, $CDCl_3$) δ 0.89 (t, *J* = 8.0 Hz, $CH_2CH_2CH_3$, 3H), 1.26-1.37 (m, 5 x CH_2, 10H), 1.67 (t, *J* = 6.0 Hz, NCH_2CH_3 , 3H), 1.94-2.01 (m, NCH_2CH_2, 2H), 2.07-2.13 (m, $CH_2CH_2CH_2OH$, 2H), 2.22 (s, *p*-CH_3, 3H), 2.65 (s, 2 x *o*-CH_3, 6H), 3.02 (t, *J* = 8.0 Hz, $CH{=}CCH_2$, 2H), 3.75 (t, *J* = 6.0 Hz, CH_2CH_2OH, 2H), 4.41 (t, *J* = 8.0 Hz, NCH_2CH_2, 2H), 4.74 (q, *J* = 8.0 Hz, NCH_2CH_3, 2H), 6.81 (s, aryl H, 2H), 9.45 (s, $CH{=}CCH_2$, 1H); ^{13}C NMR (100 MHz, $CDCl_3$) δ 14.23, 14.59, 20.43, 20.93,

22.74, 23.14, 26.44, 29.00, 29.01, 29.13, 29.72, 31.82, 49.48, 51.07, 60.30, 130.01, 130.77, 137.14, 138.45, 140.25, 144.37; EI-HRMS *m/z* [M^+] calcd for $C_{15}H_{30}N_3O$ 268.2389, found 268.2389.

[Bu-Et-C3OH-tr][OTMBS] (d6): 98% yield, light yellow liquid; ^{1}H NMR (400 MHz, $CDCl_3$) δ 0.92 (t, *J* = 8.0 Hz, $CH_2CH_2CH_3$, 3H), 1.28-1.38 (m, $CH_2CH_2CH_3$, 2H), 1.28 (t, *J* = 8.0 Hz, NCH_2CH_3, 3H), 1.90-1.98 (m, NCH_2CH_2, 2H), 2.00-2.07 (m, $CH_2CH_2CH_2OH$, 2H), 2.22 (s, *p*-CH_3, 3H), 2.63 (s, 2 x *o*-CH_3, 6H), 3.03 (t, *J* = 8.0 Hz, CH=CCH_2, 2H), 3.68 (t, *J* = 6.0 Hz, CH_2CH_2OH, 2H), 4.50 (q, *J* = 8.0 Hz, NCH_2CH_3, 2H), 4.58 (t, *J* = 8.0 Hz, NCH_2CH_2, 2H), 6.80 (s, aryl H, 2H), 9.24 (s, CH=CCH_2, 1H); ^{13}C NMR (100 MHz, $CDCl_3$) δ 13.42, 14.12, 19.54, 20.22, 20.88, 23.09, 29.78, 31.31, 46.51, 53.69, 60.24, 129.92, 130.71, 137.06, 138.39, 140.31, 144.29; EI-HRMS *m/z* [M^+] calcd for $C_{11}H_{22}N_3O$ 212.1763, found 212.1759.

[Bu-Bu-C3OH-tr][OTMBS] (d7): 94% yield, light yellow liquid; ^{1}H NMR (400 MHz, $CDCl_3$) δ 0.96-1.01 (m, 2 x CH_3, 6H), 1.33-1.43 (m, 2 x $CH_2CH_2CH_3$, 4H), 1.90-2.00 (m, 2 x NCH_2CH_2, 4H), 2.10 (m, $CH_2CH_2CH_2OH$, 2H), 2.23 (s, *p*-CH_3, 3H), 2.62 (s, 2 x *o*-CH_3, 6H), 3.04 (m, CH=CCH_2, 2H), 3.77 (m, CH_2CH_2OH, 2H), 4.43 (t, *J* = 8.0 Hz, $CNCH_2$, 2H), 4.64 (t, *J* = 8.0 Hz, $CHNCH_2$, 2H), 6.81 (s, aryl *H*, 2H), 9.31 (s, CH=CCH_2, 1H); ^{13}C NMR (100 MHz, $CDCl_3$) δ 13.46, 13.53, 19.59, 19.72, 20.51, 20.93, 23.14, 29.76, 30.94, 31.37, 50.83, 53.84, 60.37, 130.31, 130.80,

137.17, 138.53, 140.18, 144.46; EI-HRMS *m/z* [M^+] calcd for $C_{13}H_{26}N_3O$ 240.2076, found 240.2068.

[Bu-Pent-C3OH-tr][OTMBS] (d8): 85% yield, light yellow liquid; ^{1}H NMR (400 MHz, $CDCl_3$) δ 0.90-0.96 (m, 2 x CH_3, 6H), 1.33-1.40 (m, 3 x CH_2, 6H), 1.95-2.02 (m, 2 x $NCH_2C\textit{H}_2$, 4H), 2.05-2.11 (m, $CH_2C\textit{H}_2CH_2OH$, 2H), 2.22 (s, *p*-$C\textit{H}_3$, 3H), 2.65 (s, 2 x *o*-$C\textit{H}_3$, 6H), 3.02 (t, *J* = 6.0 Hz, $CH{=}CC\textit{H}_2$, 2H), 3.73 (t, *J* = 6.0 Hz, $CH_2C\textit{H}_2OH$, 2H), 4.41 (t, *J* = 8.0 Hz, $CNC\textit{H}_2$, 2H), 4.65 (t, *J* = 8.0 Hz, $CHNC\textit{H}_2$, 2H), 6.81 (s, aryl *H*, 2H), 9.39 (s, $C\textit{H}{=}CCH_2$, 1H); ^{13}C NMR (100 MHz, $CDCl_3$) δ 13.43, 13.89, 19.55, 20.40, 20.90, 22.12, 23.13, 28.43, 28.67, 29.80, 31.35, 51.04, 53.76, 60.28, 130.20, 130.74, 137.11, 138.37, 140.39, 144.44; EI-HRMS *m/z* [M^+] calcd for $C_{14}H_{28}N_3O$ 254.2232, found 254.2231.

[Bu-Hex-C3OH-tr][OTMBS] (d9): 80% yield, light yellow liquid; ^{1}H NMR (400 MHz, $CDCl_3$) δ 0.92-0.97 (m, 2 x CH_3, 6H), 1.31-1.39 (m, 4 x CH_2, 8H), 1.97-2.02 (m, 2 x $NCH_2C\textit{H}_2$, 4H), 2.07-2.10 (m, $CH_2C\textit{H}_2CH_2OH$, 2H), 2.22 (s, *p*-$C\textit{H}_3$, 3H), 2.66 (s, 2 x *o*-$C\textit{H}_3$, 6H), 3.02 (t, *J* = 6.0 Hz, $CH{=}CC\textit{H}_2$, 2H), 3.75 (t, *J* = 6.0 Hz, $CH_2C\textit{H}_2OH$, 2H), 4.41 (t, *J* = 8.0 Hz, $CNC\textit{H}_2$, 2H), 4.66 (t, *J* = 6.0 Hz, $CHNC\textit{H}_2$, 2H), 6.82 (s, aryl *H*, 2H), 9.39 (s, $C\textit{H}{=}CCH_2$, 1H); ^{13}C NMR (100 MHz, $CDCl_3$) δ 13.46, 14.05, 19.59, 20.48, 20.93, 22.52, 23.16, 26.09, 28.97, 29.79, 31.15, 31.37, 51.07,

53.83, 60.36, 130.28, 130.80, 137.13, 138.46, 140.32, 144.44; EI-HRMS *m/z* [M^+] calcd for $C_{15}H_{30}N_3O$ 268.2389, found 268.2382.

[Bu-Oct-C3OH-tr][OTMBS] (d10): 90% yield, light yellow liquid; ^1H NMR (400 MHz, $CDCl_3$) δ 0.88 (t, *J* = 6.0 Hz, CH_3, 3H), 0.94 (t, *J* = 6.0 Hz, CH_3, 3H), 1.26-1.37 (m, 6 x CH_2, 12H), 1.89-2.01 (m, 2 x NCH_2CH_2, 4H), 2.03-2.10 (m, $CH_2CH_2CH_2OH$, 2H), 2.22 (s, *p*-CH_3, 3H), 2.66 (s, 2 x *o*-CH_3, 6H), 3.01 (t, *J* = 6.0 Hz, $CH{=}CCH_2$, 2H), 3.72 (t, *J* = 6.0 Hz, CH_2CH_2OH, 2H), 4.41 (t, *J* = 8.0 Hz, $CNCH_2$, 2H), 4.63 (t, *J* = 8.0 Hz, $CHNCH_2$, 2H), 6.81 (s, aryl H, 2H), 9.36 (s, $CH{=}CCH_2$, 1H); ^{13}C NMR (100 MHz, $CDCl_3$) δ 13.44, 14.20, 19.55, 20.38, 20.90, 22.72, 23.16, 26.37, 28.97, 28.98, 29.11, 29.80, 31.37, 31.78, 51.05, 53.74, 60.21, 130.24, 130.71, 137.07, 138.25, 140.54, 144.40; EI-HRMS *m/z* [M^+] calcd for $C_{17}H_{34}N_3O$ 296.2702, found 296.2698.

[Pent-Et-C3OH-tr][OTMBS] (d11): 87% yield, light yellow liquid; ^1H NMR (400 MHz, $CDCl_3$) δ 0.88 (t, *J* = 6.0 Hz, CH_3, 3H), 1.25-1.33 (m, 2 x CH_2, 4H), 1.59 (t, *J* = 8.0 Hz, CH_3, 3H), 1.93-2.00 (m, NCH_2CH_2, 4H), 2.02-2.08 (m, $CH_2CH_2CH_2OH$, 2H), 2.22 (s, *p*-CH_3, 3H), 2.63 (s, 2 x *o*-CH_3, 6H), 3.04 (t, *J* = 8.0 Hz, $CH{=}CCH_2$, 2H), 3.70 (t, *J* = 6.0 Hz, CH_2CH_2OH, 2H), 4.50 (q, *J* = 8.0 Hz, NCH_2CH_3, 2H), 4.58 (t, *J* = 8.0 Hz, NCH_2CH_2, 2H), 6.80 (s, aryl H, 2H), 9.25 (s, $CH{=}CCH_2$, 1H); ^{13}C NMR (100 MHz, $CDCl_3$) δ 13.91, 14.14, 20.28, 20.90, 22.07, 23.10, 28.33, 29.14, 29.77, 46.51,

53.96, 60.30, 129.96, 130.75, 137.11, 138.50, 140.12, 144.30; EI-HRMS *m/z* [M^+] calcd for $C_{12}H_{24}N_3O$ 226.1919, found 226.1916.

[Pent-Bu-C3OH-tr][OTMBS] (d12): 65% yield, light yellow liquid; ^{1}H NMR (400 MHz, $CDCl_3$) δ 0.88 (t, *J* = 6.0 Hz, CH_3, 3H), 0.99 (t, *J* = 6.0 Hz, CH_3, 3H), 1.32-1.43 (m, 3 x CH_2, 6H), 1.91-2.04 (m, 2 x $NCH_2C\mathit{H}_2$, 4H), 2.07-2.10 (m, $CH_2C\mathit{H}_2CH_2OH$, 2H), 2.22 (s, *p*-C*H*$_3$, 3H), 2.65 (s, 2 x *o*-C*H*$_3$, 6H), 3.03 (t, *J* = 6.0 Hz, CH=CC*H*$_2$, 2H), 3.74 (t, *J* = 6.0 Hz, $CH_2C\mathit{H}_2OH$, 2H), 4.43 (t, *J* = 8.0 Hz, CNC*H*$_2$, 2H), 4.64 (t, *J* = 8.0 Hz, CHNC*H*$_2$, 2H), 6.81 (s, aryl *H*, 2H), 9.39 (s, C*H*=CCH_2, 1H); ^{13}C NMR (100 MHz, $CDCl_3$) δ 13.53, 13.94, 19.72, 20.50, 20.94, 22.10, 23.15, 28.38, 29.19, 29.78, 30.95, 50.84, 54.07, 60.35, 130.32, 130.80, 137.18, 138.50, 140.26, 144.47; EI-HRMS *m/z* [M^+] calcd for $C_{14}H_{28}N_3O$ 254.2232, found 254.2232.

[Pent-Pent-C3OH-tr][OTMBS] (d13): 64% yield, light yellow liquid; ^{1}H NMR (400 MHz, $CDCl_3$) δ 0.86-0.94 (m, 2 x CH_3, 6H), 1.29-1.39 (m, 4 x CH_2, 8H), 1.92-2.04 (m, 2 x $NCH_2C\mathit{H}_2$, 4H), 2.07-2.10 (m, $CH_2C\mathit{H}_2CH_2OH$, 2H), 2.22 (s, *p*-C*H*$_3$, 3H), 2.66 (s, 2 x *o*-C*H*$_3$, 6H), 3.02 (t, *J* = 6.0 Hz, CH=CC*H*$_2$, 2H), 3.74 (t, *J* = 4.0 Hz, $CH_2C\mathit{H}_2OH$, 2H), 4.42 (t, *J* = 6.0 Hz, CNC*H*$_2$, 2H), 4.64 (t, *J* = 8.0 Hz, CHNC*H*$_2$, 2H), 6.81 (s, aryl *H*, 2H), 9.40 (s, C*H*=CCH_2, 1H); ^{13}C NMR (100 MHz, $CDCl_3$) δ 13.92, 13.92, 20.46, 20.92, 22.10, 22.14, 23.16, 28.36, 28.46, 28.69, 29.18, 29.82, 51.05,

43.03, 60.32, 130.27, 130.76, 137.11, 138.34, 140.47, 144.45; EI-HRMS *m/z* [M^+] calcd for $C_{15}H_{30}N_3O$ 268.2389, found 268.2390.

[Pent-Hex-C3OH-tr][OTMBS] (d14): 55% yield, light yellow liquid; ^{1}H NMR (400 MHz, $CDCl_3$) δ 0.86-0.91 (m, 2 x CH_3, 6H), 1.26-1.36 (m, 5 x CH_2, 10H), 1.90-2.04 (m, 2 x NCH_2CH_2, 4H), 2.04-2.10 (m, $CH_2CH_2CH_2OH$, 2H), 2.22 (s, *p*-CH_3, 3H), 2.64 (s, 2 x *o*-CH_3, 6H), 3.02 (t, *J* = 8.0 Hz, CH=CCH_2, 2H), 3.73 (t, *J* = 6.0 Hz, CH_2CH_2OH, 2H), 4.41 (t, *J* = 8.0 Hz, $CNCH_2$, 2H), 4.62 (t, *J* = 8.0 Hz, $CHNCH_2$, 2H), 6.81 (s, aryl *H*, 2H), 9.34 (s, C*H*=CCH_2, 1H); ^{13}C NMR (100 MHz, $CDCl_3$) δ 13.53, 13.94, 19.72, 20.50, 20.94, 22.10, 23.15, 26.07, 28.36, 28.38, 29.19, 29.78, 30.95, 50.84, 54.07, 60.35, 130.32, 130.80, 137.18, 138.50, 140.26, 144.47; EI-HRMS *m/z* [M^+] calcd for $C_{16}H_{32}N_3O$ 282.2545, found 282.2542.

[Pent-Oct-C3OH-tr][OTMBS] (d15): 81% yield, light yellow liquid; ^{1}H NMR (400 MHz, $CDCl_3$) δ 0.86-0.90 (m, 2 x CH_3, 6H), 1.27-1.33 (m, 7 x CH_2, 8H), 1.92-2.01 (m, 2 x NCH_2CH_2, 4H), 2.03-2.10 (m, $CH_2CH_2CH_2OH$, 2H), 2.22 (s, *p*-CH_3, 3H), 2.66 (s, 2 x *o*-CH_3, 6H), 3.02 (t, *J* = 6.0 Hz, CH=CCH_2, 2H), 3.73 (t, *J* = 6.0 Hz, CH_2CH_2OH, 2H), 4.41 (t, *J* = 8.0 Hz, $CNCH_2$, 2H), 4.63 (t, *J* = 8.0 Hz, $CHNCH_2$, 2H), 6.82 (s, aryl H, 2H), 9.37 (s, C*H*=CCH_2, 1H); ^{13}C NMR (100 MHz, $CDCl_3$) δ 13.92, 14.21, 20.41, 20.91, 22.09, 22.73, 23.18, 26.39, 28.34, 28.99, 29.12, 29.19, 29.81,

31.80, 31.80, 51.06, 59.98, 60.22, 130.28, 130.72, 137.07, 138.24, 140.58, 144.40; EI-HRMS *m/z* [M^+] calcd for $C_{18}H_{36}N_3O$ 310.2858, found 310.2849.

[Hex-Et-C3OH-tr][OTMBS] (d16): 79% yield, light yellow liquid; ^{1}H NMR (400 MHz, $CDCl_3$) δ 0.87 (t, *J* = 6.0 Hz, CH_3, 3H), 1.25-1.33 (m, 3 x CH_2, 6H), 1.62 (t, *J* = 6.0 Hz, CH_3, 3H), 1.94-2.01 (m, $NCH_2C\mathit{H_2}$, 2H), 2.05-2.11 (m, $CH_2C\mathit{H_2}CH_2OH$, 2H), 2.22 (s, *p*-CH_3, 3H), 2.62 (s, 2 x *o*-CH_3, 6H), 3.05 (t, *J* = 8.0 Hz, $CH{=}CC\mathit{H_2}$, 2H), 3.74 (t, *J* = 6.0 Hz, $CH_2C\mathit{H_2}OH$, 2H), 4.51 (q, *J* = 8.0 Hz, $N\mathit{CH_2}CH_3$, 2H), 4.61 (t, *J* = 6.0 Hz, $N\mathit{CH_2}CH_2$, 2H), 6.80 (s, aryl H, 2H), 9.27 (s, $C\mathit{H}{=}CCH_2$, 1H); ^{13}C NMR (100 MHz, $CDCl_3$) δ 14.07, 14.18, 20.35, 20.93, 22.51, 23.10, 25.98, 29.43, 29.74, 31.11, 46.51, 54.02, 60.32, 130.09, 130.78, 137.18, 138.59, 139.92, 144.30; EI-HRMS *m/z* [M^+] calcd for $C_{13}H_{26}N_3O$ 240.2076, found 240.2068.

[Hex-Bu-C3OH-tr][OTMBS] (d17): 81% yield, light yellow liquid; ^{1}H NMR (400 MHz, $CDCl_3$) δ 0.87 (t, *J* = 6.0 Hz, CH_3, 3H), 0.99 (t, *J* = 8.0 Hz, CH_3, 3H), 1.29-1.45 (m, 4 x CH_2, 8H), 1.91-2.01 (m, 2 x $NCH_2C\mathit{H_2}$ + $CH_2C\mathit{H_2}CH_2OH$, 6H), 2.22 (s, *p*-$C\mathit{H_3}$, 3H), 2.65 (s, 2 x *o*-$C\mathit{H_3}$, 6H), 3.03 (t, *J* = 6.0 Hz, $CH{=}CC\mathit{H_2}$, 2H), 3.75 (t, *J* = 4.0 Hz, $CH_2C\mathit{H_2}OH$, 2H), 4.43 (t, *J* = 6.0 Hz, $CNC\mathit{H_2}$, 2H), 4.64 (t, *J* = 8.0 Hz, $CHNC\mathit{H_2}$, 2H), 6.81 (s, aryl *H*, 2H), 9.37 (s, $C\mathit{H}{=}CCH_2$, 1H); ^{13}C NMR (100 MHz, $CDCl_3$) δ 13.50, 14.05, 19.67, 20.39, 20.90, 22.51, 23.14, 25.95, 29.38, 29.83, 30.87,

31.09, 50.80, 54.01, 60.38, 130.00, 130.78, 137.09, 138.45, 140.30, 144.47; EI-HRMS *m/z* [M^+] calcd for $C_{15}H_{30}N_3O$ 268.2389, found 268.2382.

[Hex-Pent-C3OH-tr][OTMBS] (d18): 75% yield, light yellow liquid; ^{1}H NMR (400 MHz, $CDCl_3$) δ 0.87 (t, *J* = 6.0 Hz, CH_3, 3H), 0.92 (t, *J* = 8.0 Hz, CH_3, 3H), 1.25-1.39 (m, 5 x CH_2, 10H), 1.92-2.01 (m, 2 x NCH_2CH_2, 4H), 2.05-2.12 (m, $CH_2CH_2CH_2OH$, 2H), 2.22 (s, *p*-CH_3, 3H), 2.65 (s, 2 x *o*-CH_3, 6H), 3.03 (t, *J* = 6.0 Hz, $CH{=}CCH_2$, 2H), 3.74 (t, *J* = 6.0 Hz, CH_2CH_2OH, 2H), 4.42 (t, *J* = 6.0 Hz, $CNCH_2$, 2H), 4.64 (t, *J* = 6.0 Hz, $CHNCH_2$, 2H), 6.81 (s, aryl H, 2H), 9.38 (s, $CH{=}CCH_2$, 1H); ^{13}C NMR (100 MHz, $CDCl_3$) δ 13.91, 14.08, 20.46, 20.93, 22.15, 22.53, 23.16, 25.98, 28.46, 28.71, 29.46, 29.78, 31.12, 51.05, 54.05, 60.29, 130.32, 130.76, 137.15, 138.41, 140.26, 144.42; EI-HRMS *m/z* [M^+] calcd for $C_{16}H_{32}N_3O$ 282.2545, found 282.2542.

[Hex-Hex-C3OH-tr][OTMBS] (d19): 80% yield, light yellow liquid; ^{1}H NMR (400 MHz, $CDCl_3$) δ 0.88-0.91 (m, 2 x CH_3 , 6H), 1.29-1.33 (m, 6 x CH_2, 12H), 1.91-2.00 (m, 2 x NCH_2CH_2 , 4H), 2.05-2.09 (m, $CH_2CH_2CH_2OH$, 2H), 2.22 (s, *p*-CH_3, 3H), 2.65 (s, 2 x *o*-CH_3, 6H), 3.02 (t, *J* = 6.0 Hz, $CH{=}CCH_2$, 2H), 3.73 (t, *J* = 6.0 Hz, CH_2CH_2OH, 2H), 4.41 (t, *J* = 6.0 Hz, $CNCH_2$, 2H), 4.63 (t, *J* = 8.0 Hz, $CHNCH_2$, 2H), 7.15 (d, *J* = 8.0 Hz, aryl H, 2H), 7.75 (d, *J* = 8.0 Hz, aryl H, 2H), 9.38 (s, $CH{=}CCH_2$, 1H); ^{13}C NMR (100 MHz, $CDCl_3$) δ 14.04, 14.05, 20.43, 20.90, 22.50, 22.51, 23.15, 25.97, 26.06, 28.94, 29.42, 29.83, 31.11, 31.14, 51.07, 54.03, 60.30, 130.19, 130.77,

137.10, 138.38, 140.42, 144.44; EI-HRMS *m/z* [M^+] calcd for $C_{17}H_{34}N_3O$ 296.2702, found 296.2701.

[Hex-Oct-C3OH-tr][OTMBS] (d20): 48% yield, light yellow liquid; ^{1}H NMR (400 MHz, $CDCl_3$) δ 0.85-0.90 (m, 2 x CH_3, 6H), 1.27-1.34 (m, 8 x CH, 16H), 1.93-2.01 (m, 2 x NCH_2CH_2, 4H), 2.06-2.12 (m, $CH_2CH_2CH_2OH$, 2H), 2.22 (s, *p*-CH_3, 3H), 2.67 (s, 2 x *o*-CH_3, 6H), 3.02 (t, J = 8.0 Hz, CH=CCH_2, 2H), 3.74 (t, J = 4.0 Hz, CH_2CH_2OH, 2H), 4.41 (t, J = 8.0 Hz, CNCH_2, 2H), 4.64 (t, J = 8.0 Hz, CHNCH_2, 2H), 6.81 (s, aryl H, 2H), 9.43 (s, C*H*=CCH_2, 1H); ^{13}C NMR (100 MHz, $CDCl_3$) δ 14.06, 14.21, 20.46, 20.91, 22.53, 22.74, 23.19, 25.98, 26.42, 29.00, 29.01, 29.13, 29.43, 29.83, 31.12, 31.81, 51.05, 54.05, 60.32, 130.27, 130.75, 137.07, 138.26, 140.61, 144.41; EI-HRMS *m/z* [M^+] calcd for $C_{19}H_{38}N_3O$ 324.3015, found 324.3004.

[Oct-Et-C3OH-tr][OTMBS] (d21): 60% yield, light yellow liquid; ^{1}H NMR (400 MHz, $CDCl_3$) δ 0.87 (t, J = 8.0 Hz, CH_3, 3H), 1.24-1.28 (m, 5 x CH_2, 10H), 1.59 (t, J = 6.0 Hz, CH_3, 3H), 1.94-1.98 (m, NCH_2CH_2, 2H), 2.08 (m, $CH_2CH_2CH_2OH$, 2H), 2.23 (s, *p*-CH_3, 3H), 2.64 (s, 2 x *o*-CH_3, 6H), 3.05 (m, CH=CCH_2, 2H), 3.77 (m, J = 6.0 Hz, CH_2CH_2OH, 2H), 4.51 (q, J = 8.0 Hz, NCH_2CH_3, 2H), 4.58 (t, J = 8.0 Hz, NCH_2CH_2, 2H), 6.81 (s, aryl H, 2H), 9.28 (s, C*H*=CCH_2, 1H); ^{13}C NMR (100 MHz, $CDCl_3$) δ 14.17, 14.23, 20.34, 20.91, 22.74, 23.15, 26.32, 28.96, 29.15, 29.48, 29.90,

31.83, 46.51, 54.01, 60.30, 130.08, 130.75, 137.10, 138.36, 140.56, 144.33; EI-HRMS *m/z* [M^+] calcd for $C_{15}H_{30}N_3O$ 268.2389, found 268.2386.

[Oct-Bu-C3OH-tr][OTMBS] (d22): 85% yield, light yellow liquid; ^{1}H NMR (400 MHz, $CDCl_3$) δ 0.87 (t, *J* = 8.0 Hz, CH_3, 3H), 0.98 (t, *J* = 8.0 Hz, CH_3, 3H), 1.24-1.28 (m, 5 x CH_2, 10H), 1.34-1.43 (m, CH_2, 2H), 1.88-1.99 (m, 2 x $NCH_2C\mathit{H_2}$, 4H), 2.08 (m, $CH_2C\mathit{H}_2CH_2OH$, 2H), 2.23 (s, *p*-CH_3, 3H), 2.66 (s, 2 x *o*-CH_3, 6H), 3.03 (m, $CH{=}CC\mathit{H}_2$, 2H), 3.74 (m, $CH_2C\mathit{H}_2OH$, 2H), 4.42 (t, *J* = 6.0 Hz, $CNC\mathit{H}_2$, 2H), 4.61 (t, *J* = 8.0 Hz, $CHNC\mathit{H}_2$, 2H), 6.82 (s, aryl H, 2H), 9.36 (s, $C\mathit{H}{=}CCH_2$, 1H); ^{13}C NMR (100 MHz, $CDCl_3$) δ 13.52, 14.23, 19.69, 20.45, 20.91, 22.75, 23.17, 26.31, 28.95, 29.16, 29.50, 29.89, 30.93, 31.83, 50.83, 54.04, 60.27, 130.28, 130.76, 137.12, 138.31, 140.71, 144.46; EI-HRMS *m/z* [M^+] calcd for $C_{17}H_{34}N_3O$ 296.2702, found 296.2688.

[Oct-Pent-C3OH-tr][OTMBS] (d23): 77% yield, light yellow liquid; ^{1}H NMR (400 MHz, $CDCl_3$) δ 0.85-0.93 (m, 2 x CH_3, 6H), 1.24-1.38 (m, 7 x CH_2, 14H), 1.90-1.99 (m, 2 x $NCH_2C\mathit{H_2}$, 4H), 2.07 (m, $CH_2C\mathit{H}_2CH_2OH$, 2H), 2.22 (s, *p*-CH_3, 3H), 2.67 (s, 2 x *o*-CH_3, 6H), 3.02 (m, $CH{=}CC\mathit{H}_2$, 2H), 3.74 (m, $CH_2C\mathit{H}_2OH$, 2H), 4.41 (t, *J* = 6.0 Hz, $CNC\mathit{H}_2$, 2H), 4.61 (t, *J* = 8.0 Hz, $CHNC\mathit{H}_2$, 2H), 6.82 (s, aryl H, 2H), 9.38 (s, $C\mathit{H}{=}CCH_2$, 1H); ^{13}C NMR (100 MHz, $CDCl_3$) δ 13.90, 14.22, 20.42, 20.90, 22.14, 22.74, 23.19, 26.31, 28.44, 28.70, 28.96, 29.16, 29.50, 29.90, 31.83, 51.04, 54.02,

60.22, 130.29, 130.72, 137.07, 138.17, 144.44; EI-HRMS *m/z* [M^+] calcd for $C_{18}H_{36}N_3O$ 310.2858, found 310.2845.

[Oct-Hex-C3OH-tr][OTMBS] (d24): 80% yield, light yellow liquid; ^1H NMR (400 MHz, $CDCl_3$) δ 0.85-0.91 (m, 2 x CH_3 , 6H), 1.24-1.33 (m, 8 x CH_2, 16H), 1.91-1.99 (m, 2 x NCH_2CH_2 , 4H), 2.09 (m, $CH_2CH_2CH_2OH$, 2H), 2.22 (s, *p*-CH_3, 3H), 2.67 (s, 2 x *o*-CH_3, 6H), 3.02 (m, CH=CCH_2, 2H), 3.75 (m, CH_2CH_2OH, 2H), 4.41 (t, *J* = 8.0 Hz, CNCH_2, 2H), 4.63 (t, *J* = 6.0 Hz, CHNCH_2, 2H), 6.82 (s, aryl H, 2H), 9.42 (s, C*H*=CCH_2, 1H); ^{13}C NMR (100 MHz, $CDCl_3$) δ 14.04, 14.22, 20.43, 20.90, 22.50, 22.74, 23.20, 26.06, 26.31, 28.96, 28.96, 29.16, 29.50, 29.90, 31.14, 31.83, 51.06, 54.02, 60.24, 130.30, 130.72, 137.07, 138.17, 144.42; EI-HRMS *m/z* [M^+] calcd for $C_{19}H_{38}N_3O$ 324.3015, found 324.3005.

[Oct-Oct-C3OH-tr][OTMBS] (d25): 65% yield, light yellow liquid; ^1H NMR (400 MHz, $CDCl_3$) δ 0.85-0.90 (m, 2 x CH_3 , 6H), 1.24-1.34 (m, 10 x CH_2, 20H), 1.93-2.01 (m, 2 x NCH_2CH_2 , 4H), 2.09 (m, $CH_2CH_2CH_2OH$, 2H), 2.23 (s, *p*-CH_3, 3H), 2.67 (s, 2 x *o*-CH_3, 6H), 3.02 (m, CH=CCH_2, 2H), 3.77 (m, CH_2CH_2OH, 2H), 4.41 (t, *J* = 8.0 Hz, CNCH_2, 2H), 4.64 (t, *J* = 8.0 Hz, CHNCH_2, 2H), 6.82 (s, aryl H, 2H), 9.42 (s, C*H*=CCH_2, 1H); ^{13}C NMR (100 MHz, $CDCl_3$) δ 14.22, 14.23, 20.42, 20.90, 20.90, 22.74, 22.74, 23.20, 26.31, 26.39, 28.96, 28.99, 29.13, 29.16, 29.50,

29.90, 31.81, 31.84, 51.05, 54.06, 60.22, 130.27, 130.73, 137.08, 138.19, 144.41; EI-HRMS *m/z* [M^+] calcd for $C_{21}H_{42}N_3O$ 352.3328, found 352.3317.

[Oct-*i*-Bu-C3OH-tr][OTMBS] (d26): 82% yield, light yellow liquid; ^{1}H NMR (400 MHz, $CDCl_3$) δ 0.85-0.89 (m, CH_3, 3H), 0.99 (d, J = 4.0 Hz, 2 x $CHCH_3$, 6H), 1.23-1.28 (m, 5 x CH_2, 12H), 1.98 (m, NCH_2CH_2 , 2H), 2.23 (s, *p*-CH_3, 3H), 2.33 (m, C*H*$CH_{3,}$ 1H), 2.66 (s, 2 x *o*-CH_3, 6H), 3.02 (m, CH=CCH_2, 2H), 3.77 (m, CH_2CH_2OH, 2H), 4.24 (d, J = 4.0 Hz, CNC$H_{2,}$ 2H), 4.63 (t, J = 6.0 Hz, CHNC$H_{2,}$ 2H), 6.82 (s, aryl H, 2H), 9.39 (s, C*H*=CCH_2, 1H); ^{13}C NMR (100 MHz, $CDCl_3$) δ 14.22, 19.77, 20.53, 20.91, 22.74, 23.17, 26.26, 28.92, 27.94, 29.15, 29.47, 29.92, 31.81, 54.03, 57.65, 60.31, 130.14, 130.77, 137.12, 138.41, 140.44, 142.76; EI-HRMS *m/z* [M^+] calcd for $C_{17}H_{34}N_3O$ 296.2702, found 296.2697.

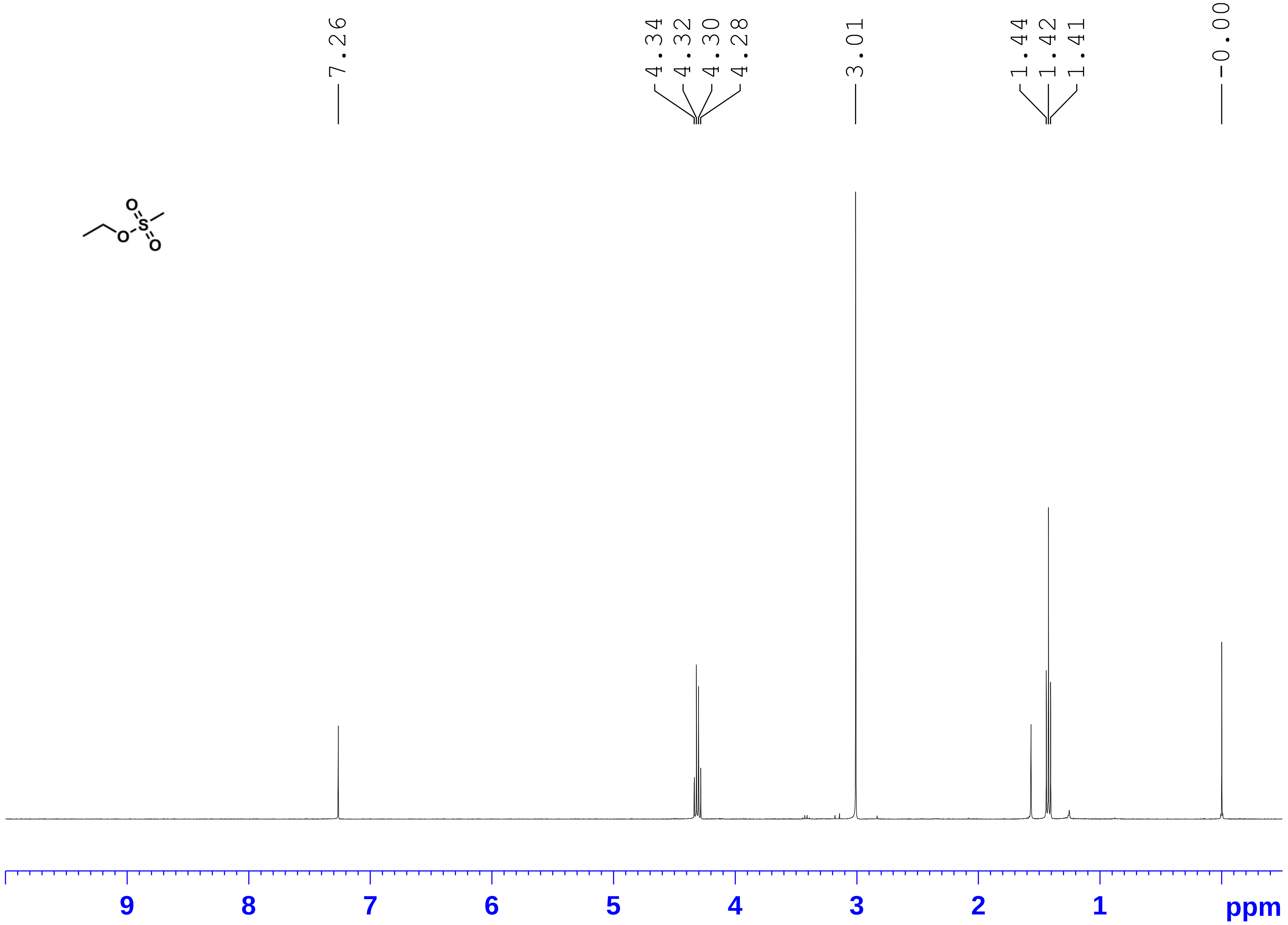
7.26
4.34
4.32
4.30
4.28
3.01
1.44
1.42
1.41
0.00
O
S
O
O
9
8
7
6
5
4
3
2
1
ppm

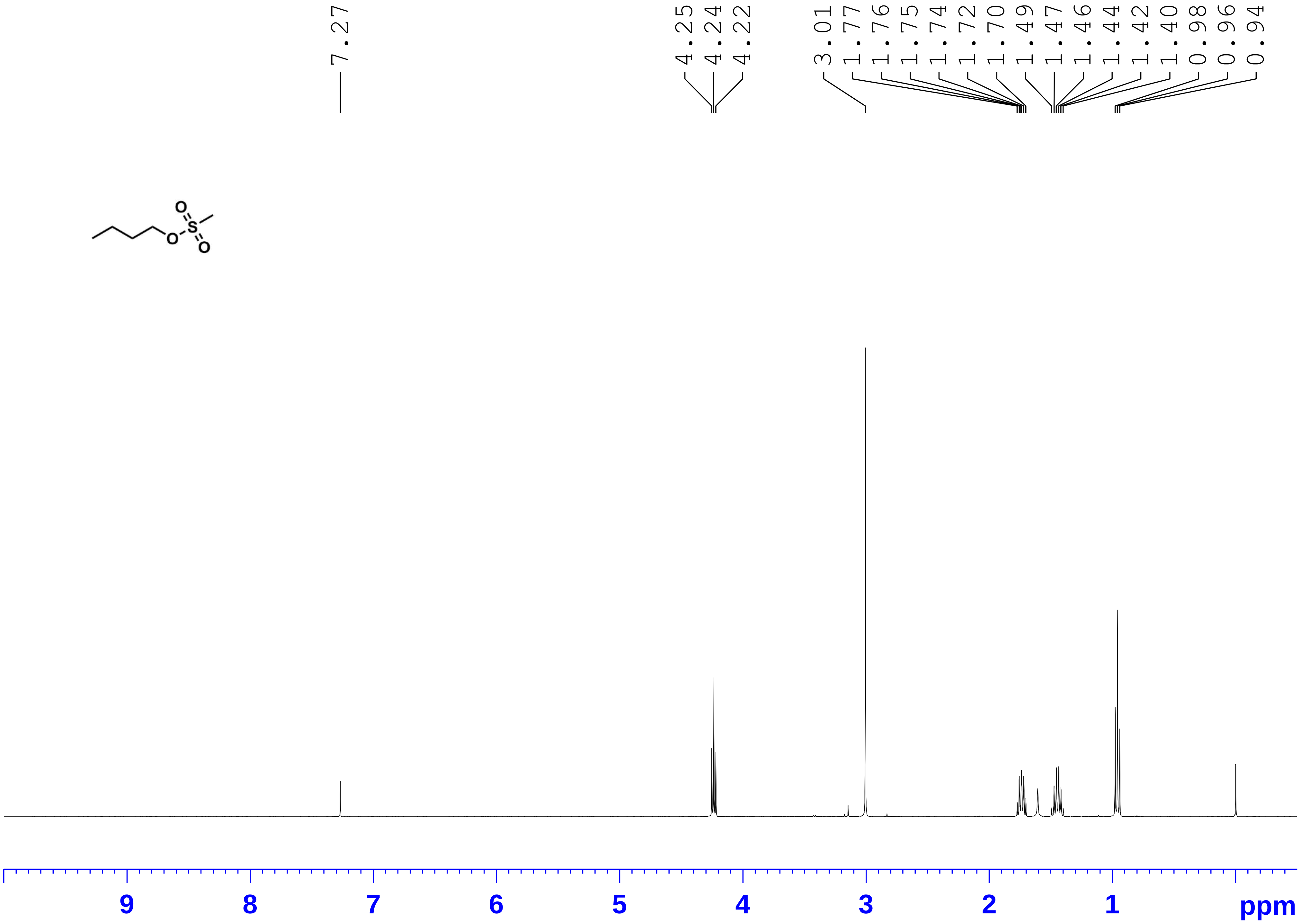

7.27
4.25
4.24
4.22
3.01
1.77
1.76
1.75
1.74
1.72
1.70
1.49
1.47
1.46
1.44
1.42
1.40
0.98
0.96
0.94
O
S
O
O
9
8
7
6
5
4
3
2
1
ppm

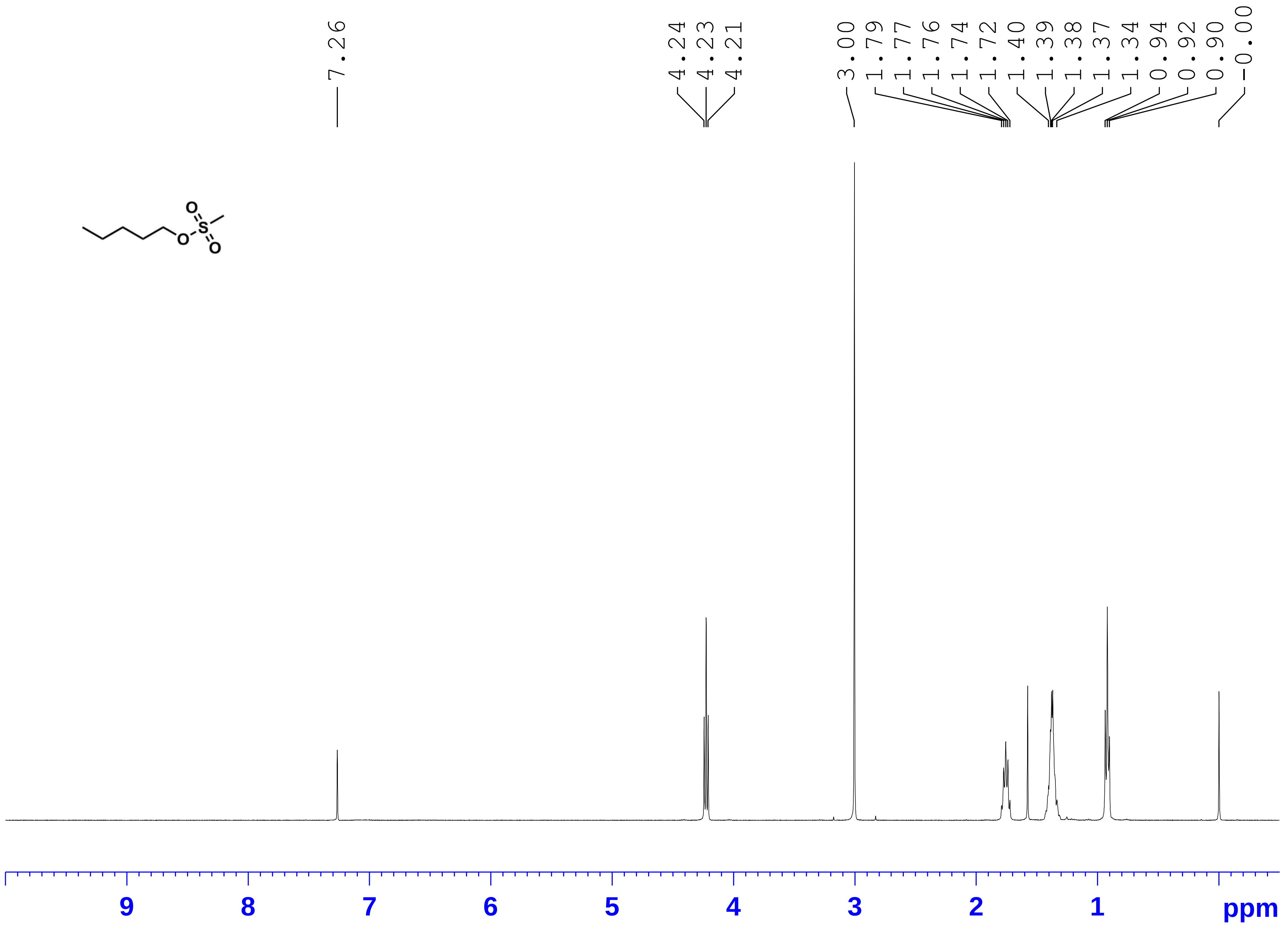

7.26
4.24
4.23
4.21
3.00
1.79
1.77
1.76
1.74
1.72
1.40
1.39
1.38
1.37
1.34
0.94
0.92
0.90
-0.00
O
S
O
O
9
8
7
6
5
4
3
2
1
ppm

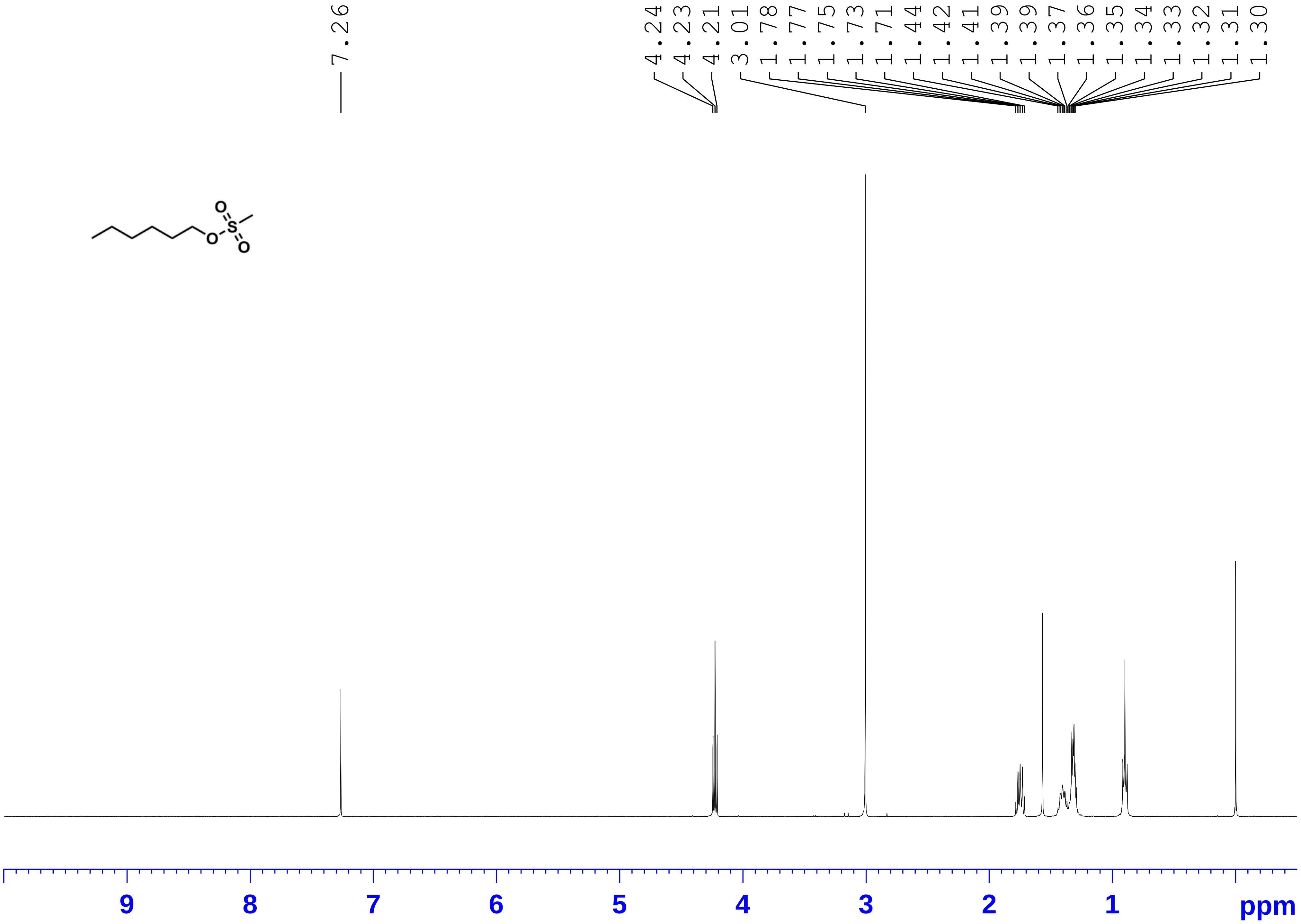
7.26
4.24
4.23
4.21
3.01
1.78
1.77
1.75
1.73
1.71
1.44
1.42
1.41
1.39
1.39
1.37
1.36
1.35
1.34
1.33
1.32
1.31
1.30
9
8
7
6
5
4
3
2
1
ppm

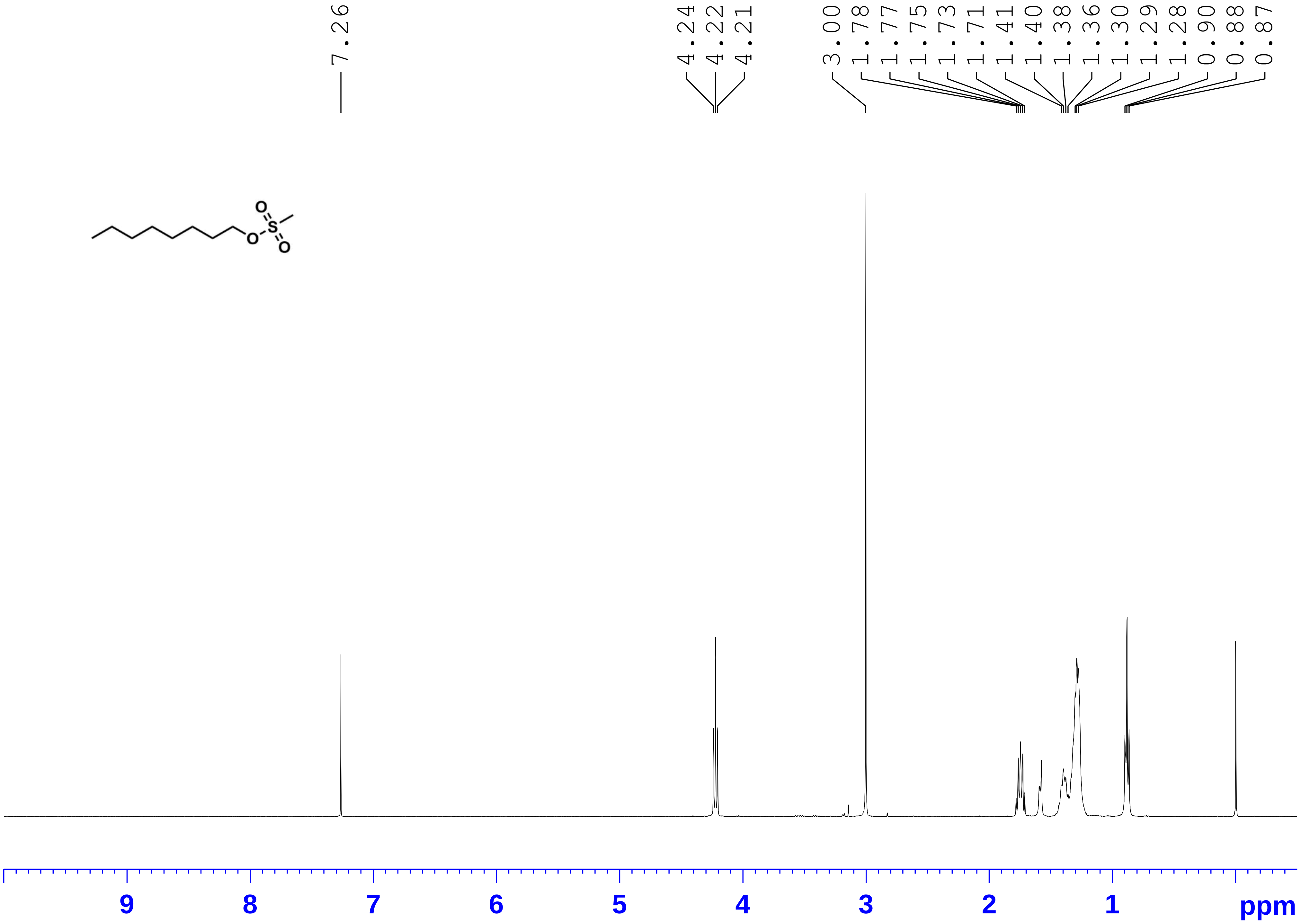
7.26
4.24
4.22
4.21
3.00
1.78
1.77
1.75
1.73
1.71
1.41
1.40
1.38
1.36
1.30
1.29
1.28
0.90
0.88
0.87
9
8
7
6
5
4
3
2
1
ppm

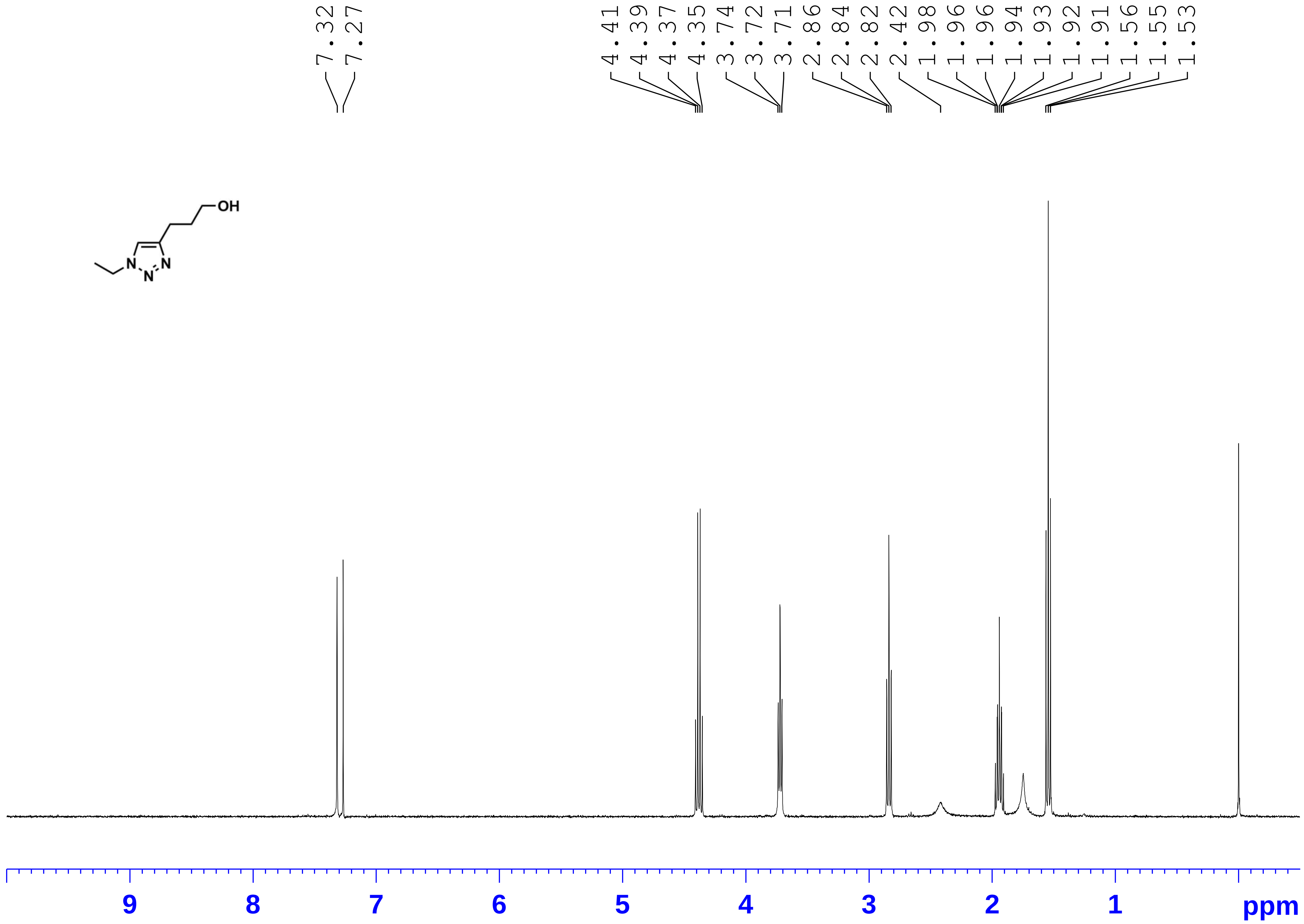
7.32
7.27
4.41
4.39
4.37
4.35
3.74
3.72
3.71
2.86
2.84
2.82
2.42
1.98
1.96
1.96
1.94
1.93
1.92
1.91
1.56
1.55
1.53
OH
N
N
N
9
8
7
6
5
4
3
2
1
ppm

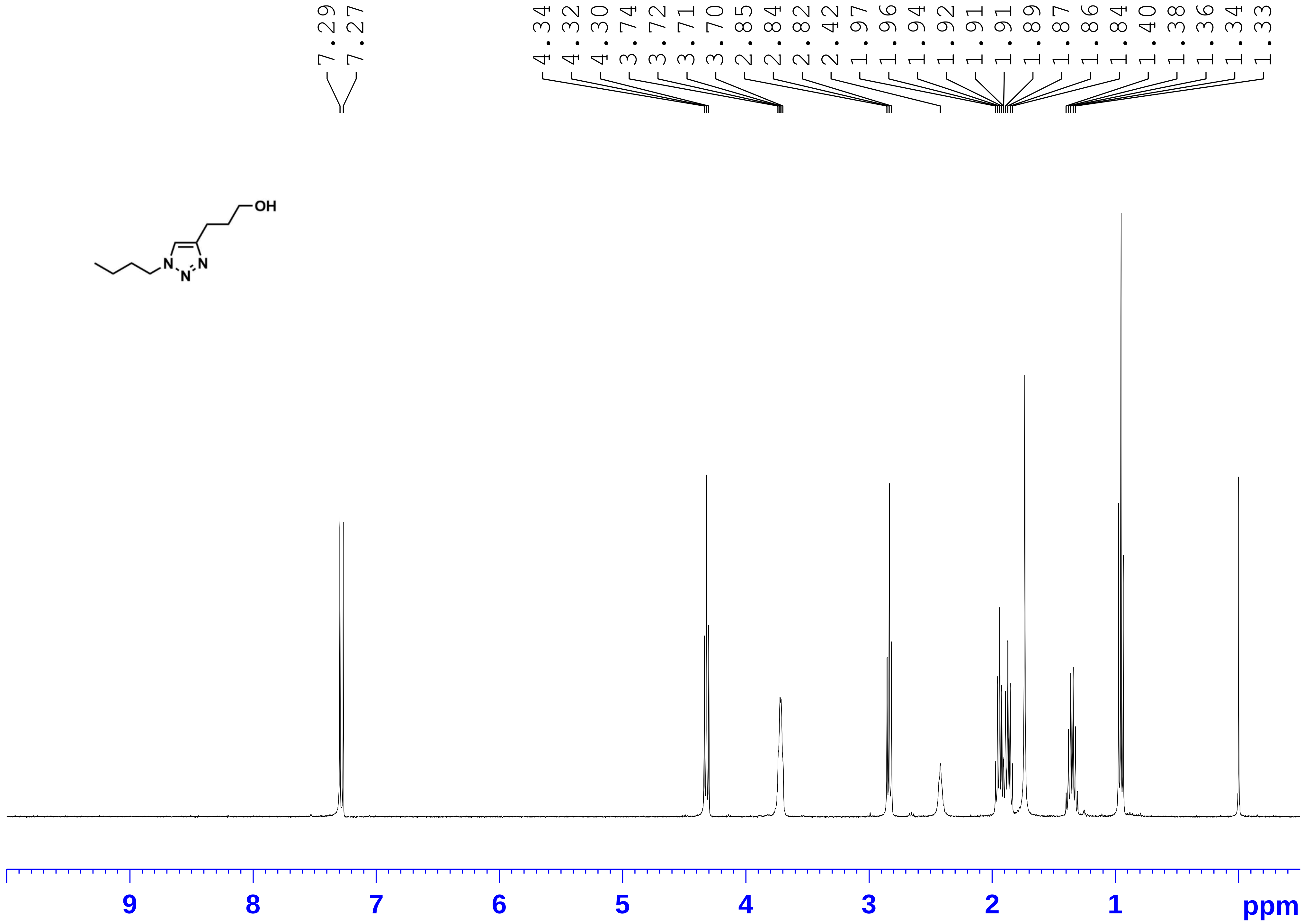
7.29
7.27
4.34
4.32
4.30
3.74
3.72
3.71
3.70
2.85
2.84
2.82
2.42
1.97
1.96
1.94
1.92
1.91
1.91
1.89
1.87
1.86
1.84
1.40
1.38
1.36
1.34
1.33
OH
N
N
N
9
8
7
6
5
4
3
2
1
ppm

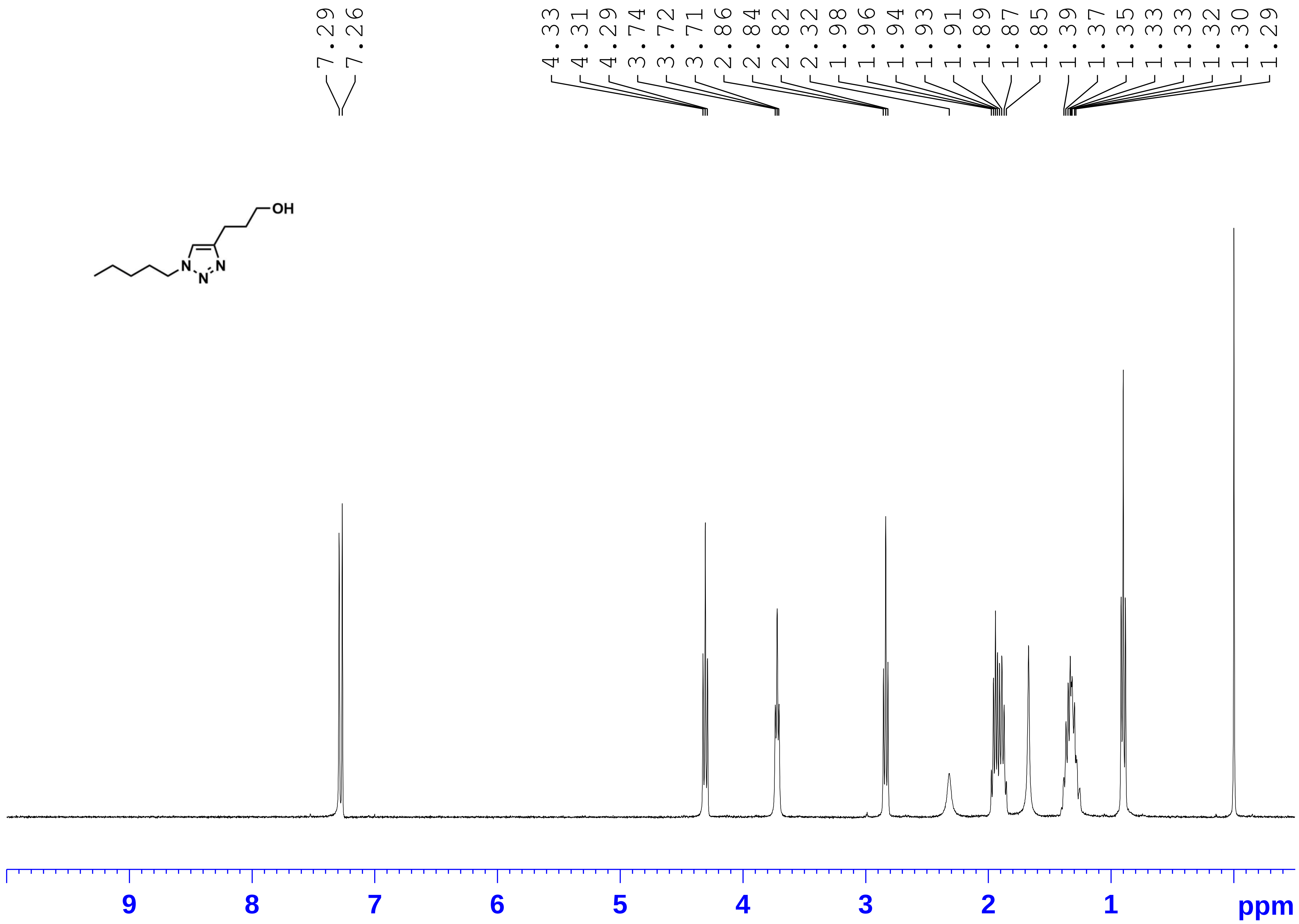
7.29
7.26
4.33
4.31
4.29
3.74
3.72
3.71
2.86
2.84
2.82
2.32
1.98
1.96
1.94
1.93
1.91
1.89
1.87
1.85
1.39
1.37
1.35
1.33
1.33
1.32
1.30
1.29
OH
N
N
N
9
8
7
6
5
4
3
2
1
ppm

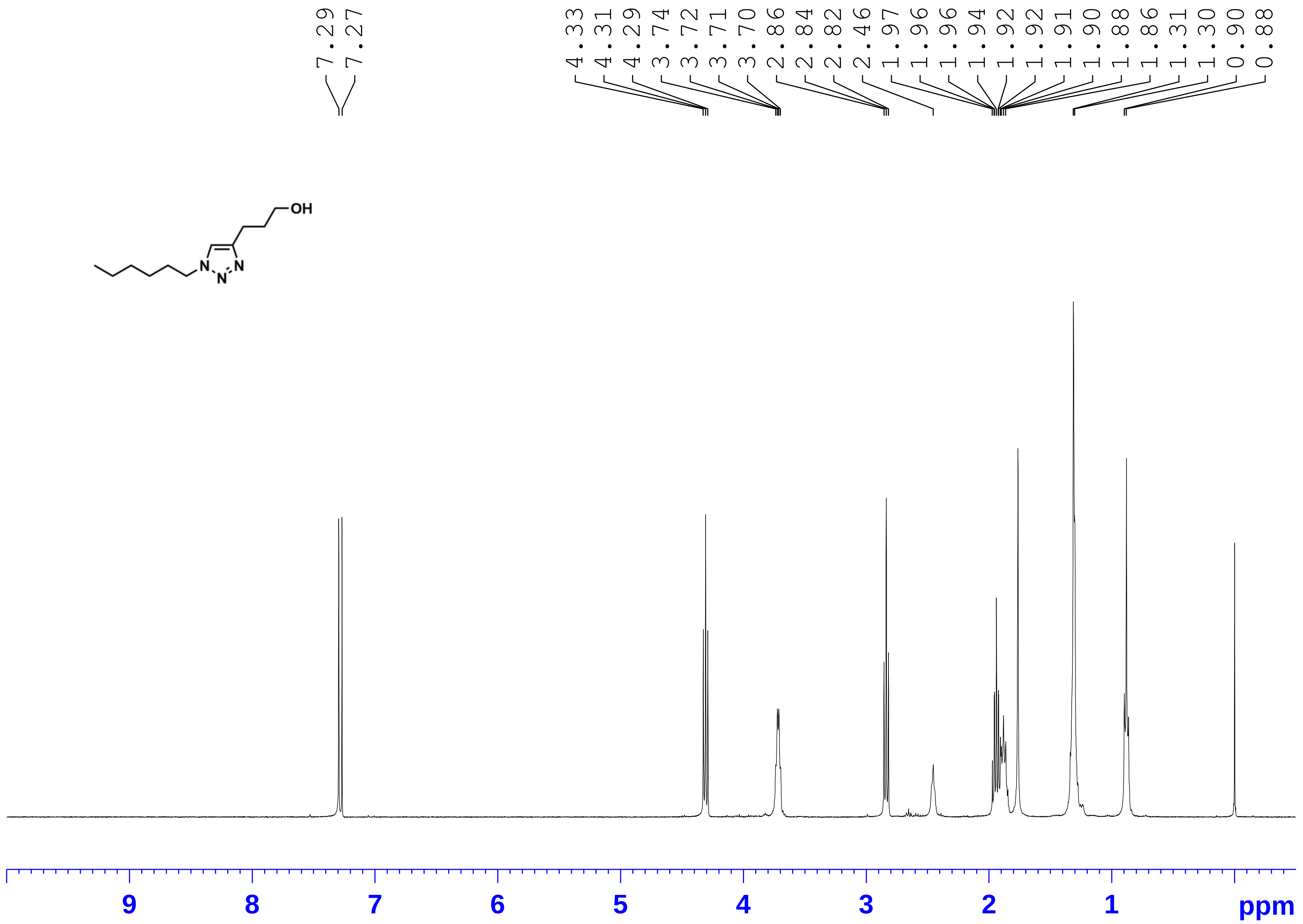

7.29
7.27
4.33
4.31
4.29
3.74
3.72
3.71
3.70
2.86
2.84
2.82
2.46
1.97
1.96
1.96
1.94
1.92
1.92
1.91
1.90
1.88
1.86
1.31
1.30
0.90
0.88
OH
N
N
N
9
8
7
6
5
4
3
2
1
ppm

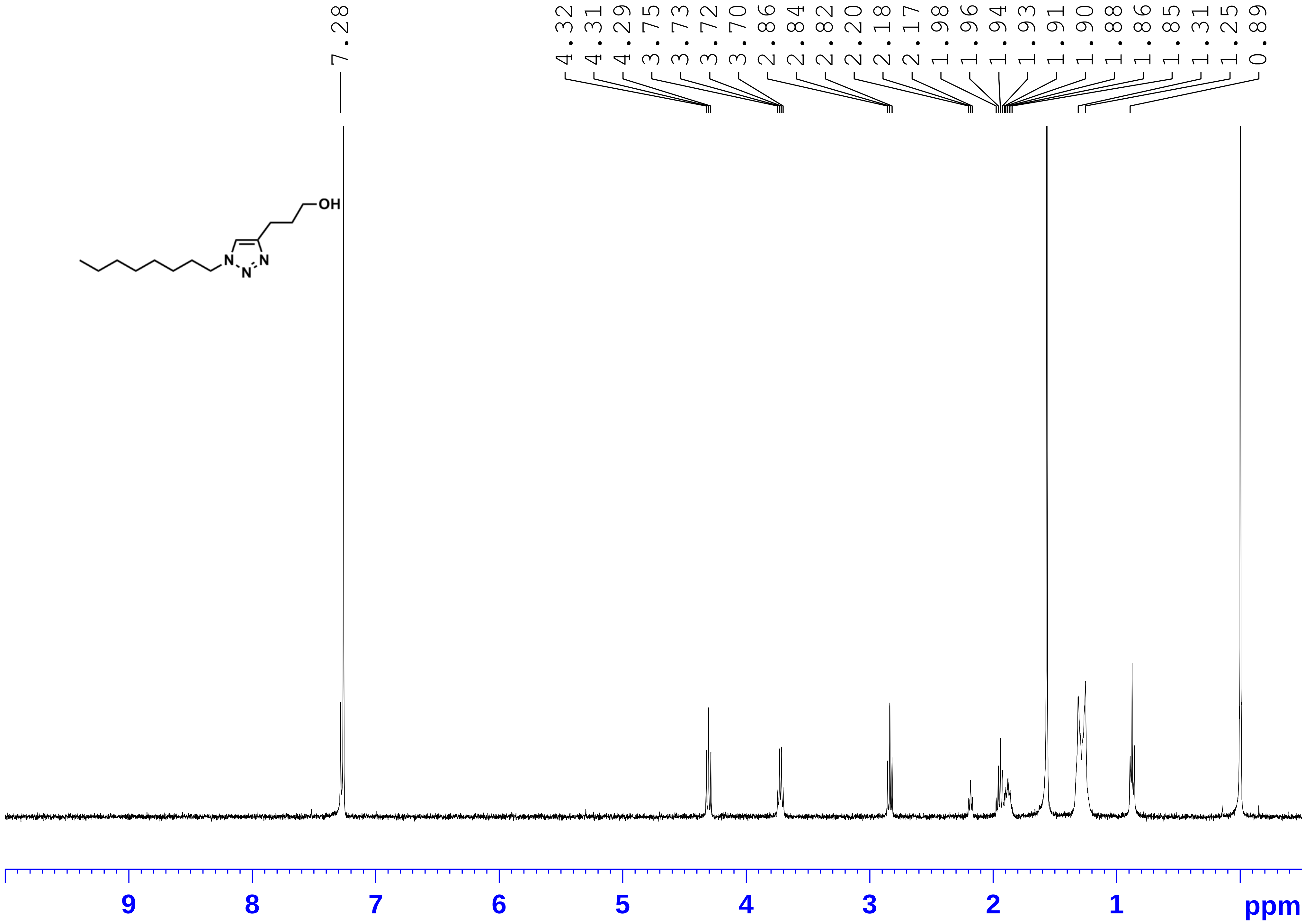
7.28
4.32
4.31
4.29
3.75
3.73
3.72
3.70
2.86
2.84
2.82
2.20
2.18
2.17
1.98
1.96
1.94
1.93
1.91
1.90
1.88
1.86
1.85
1.31
1.25
0.89
OH
N
N
N
9
8
7
6
5
4
3
2
1
ppm

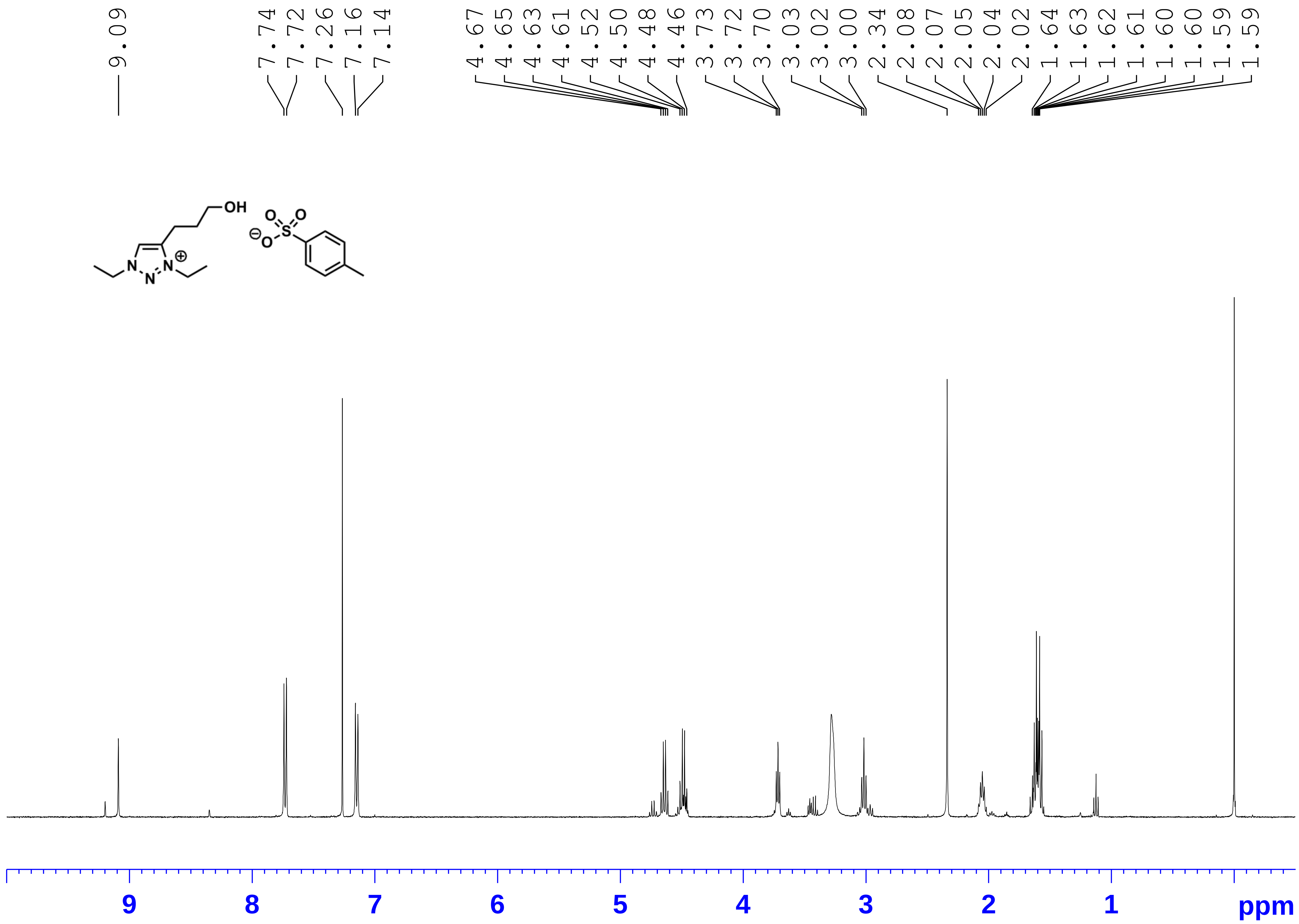
9.09
7.74
7.72
7.26
7.16
7.14
4.67
4.65
4.63
4.61
4.52
4.50
4.48
4.46
3.73
3.72
3.70
3.03
3.02
3.00
2.34
2.08
2.07
2.05
2.04
2.02
1.64
1.63
1.62
1.61
1.60
1.60
1.59
1.59
OH
O
O
S
O
N
N
N
9
8
7
6
5
4
3
2
1
ppm

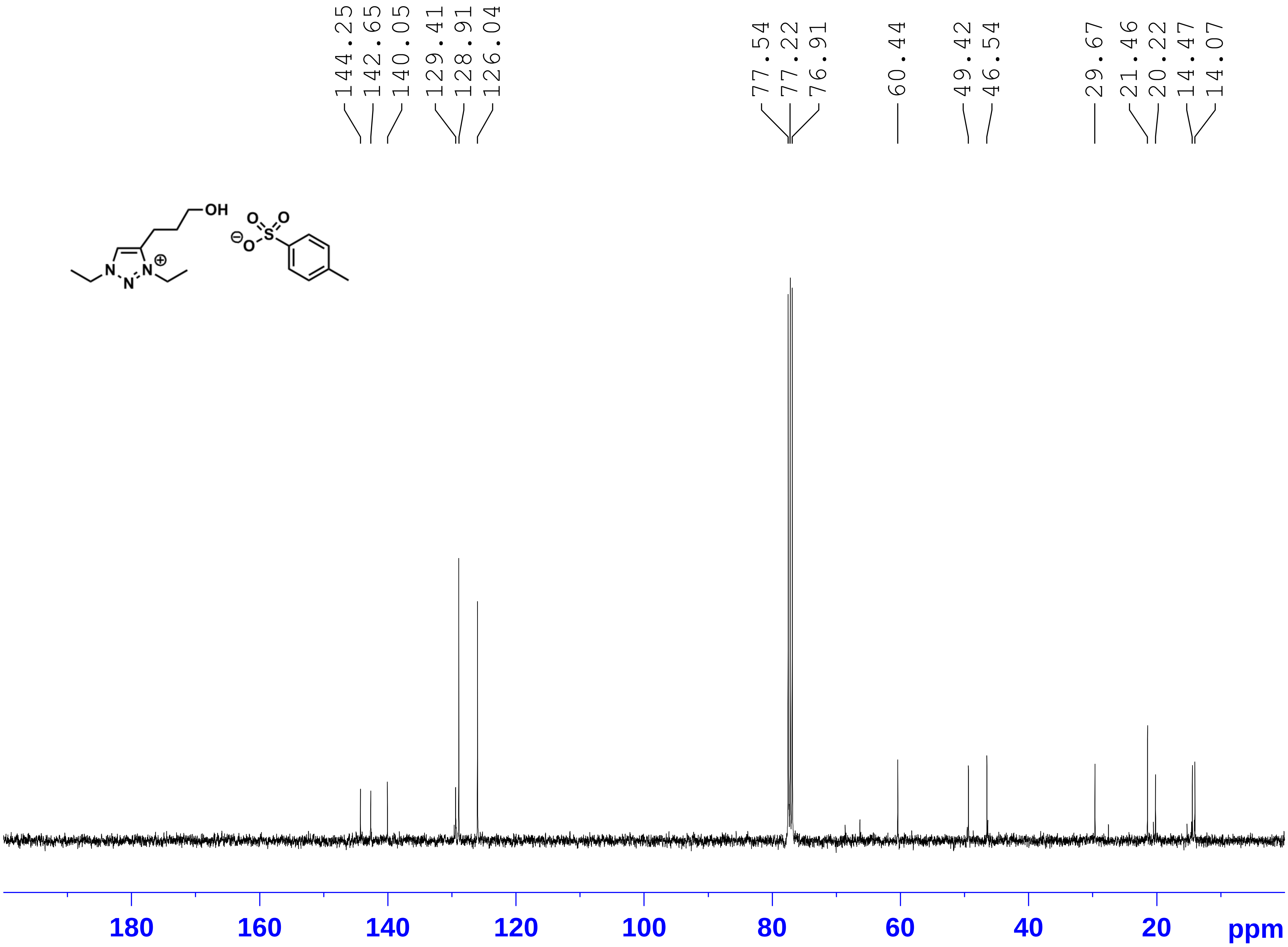
144.25
142.65
140.05
129.41
128.91
126.04
77.54
77.22
76.91
60.44
49.42
46.54
29.67
21.46
20.22
14.47
14.07
180
160
140
120
100
80
60
40
20
ppm

Spectrum

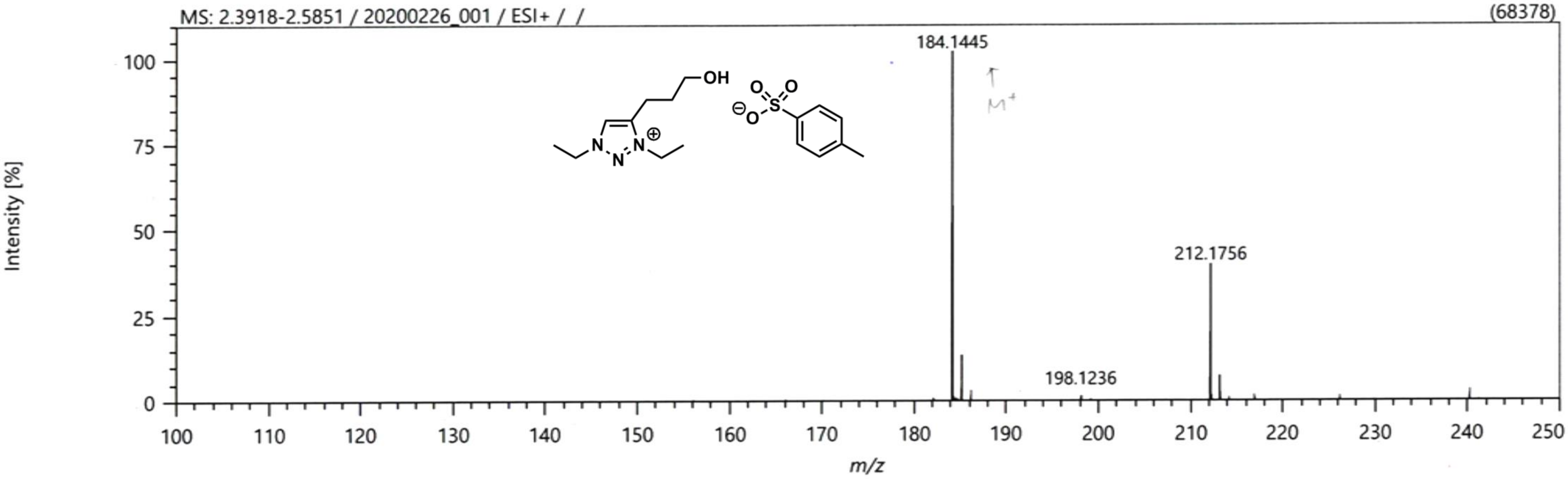

Elemental Composition

Parameters

		Elements Set 1:							
Tolerance:	±10.00 ppm	Symbol	C	H	O	Na	N	S	Ni	F
Electron:	Odd/Even	Min	0	0	1	0	3	0	0	0
Charge:	+1	Max	1000	4000	1	0	3	0	0	0
DBE:	-99.0 - 999.0									

Results

Mass	Formula	Calculated Mass	Mass Difference [mDa]	Mass Difference [ppm]	DBE
184.14447	C9 H18 N3 O	184.14444	0.03	0.16	2.5

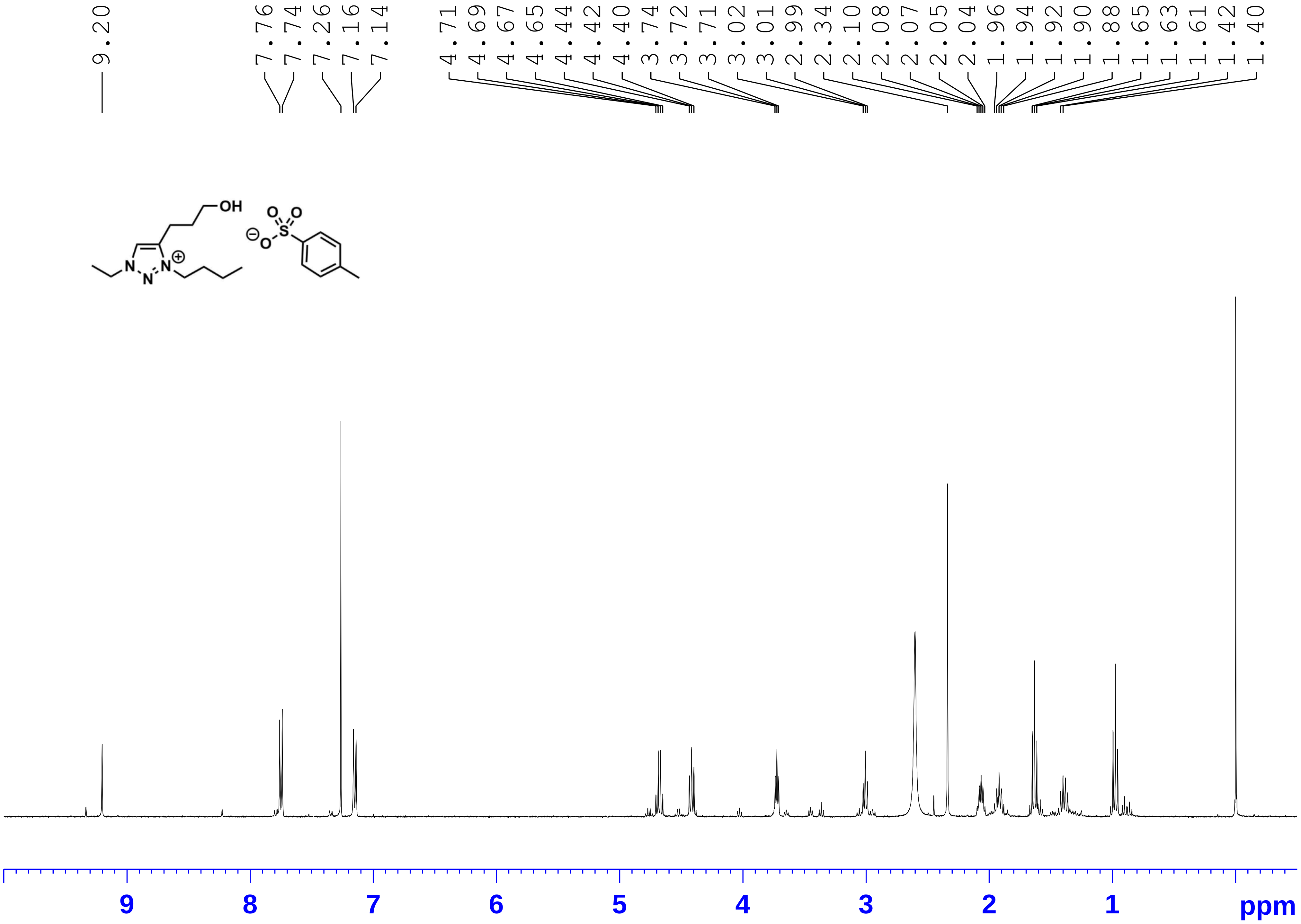
9.20
7.76
7.74
7.26
7.16
7.14
4.71
4.69
4.67
4.65
4.44
4.42
4.40
3.74
3.72
3.71
3.02
3.01
2.99
2.34
2.10
2.08
2.07
2.05
2.04
1.96
1.94
1.92
1.90
1.88
1.65
1.63
1.61
1.42
1.40
OH
O
S
O
O
N
N
N
9
8
7
6
5
4
3
2
1
ppm

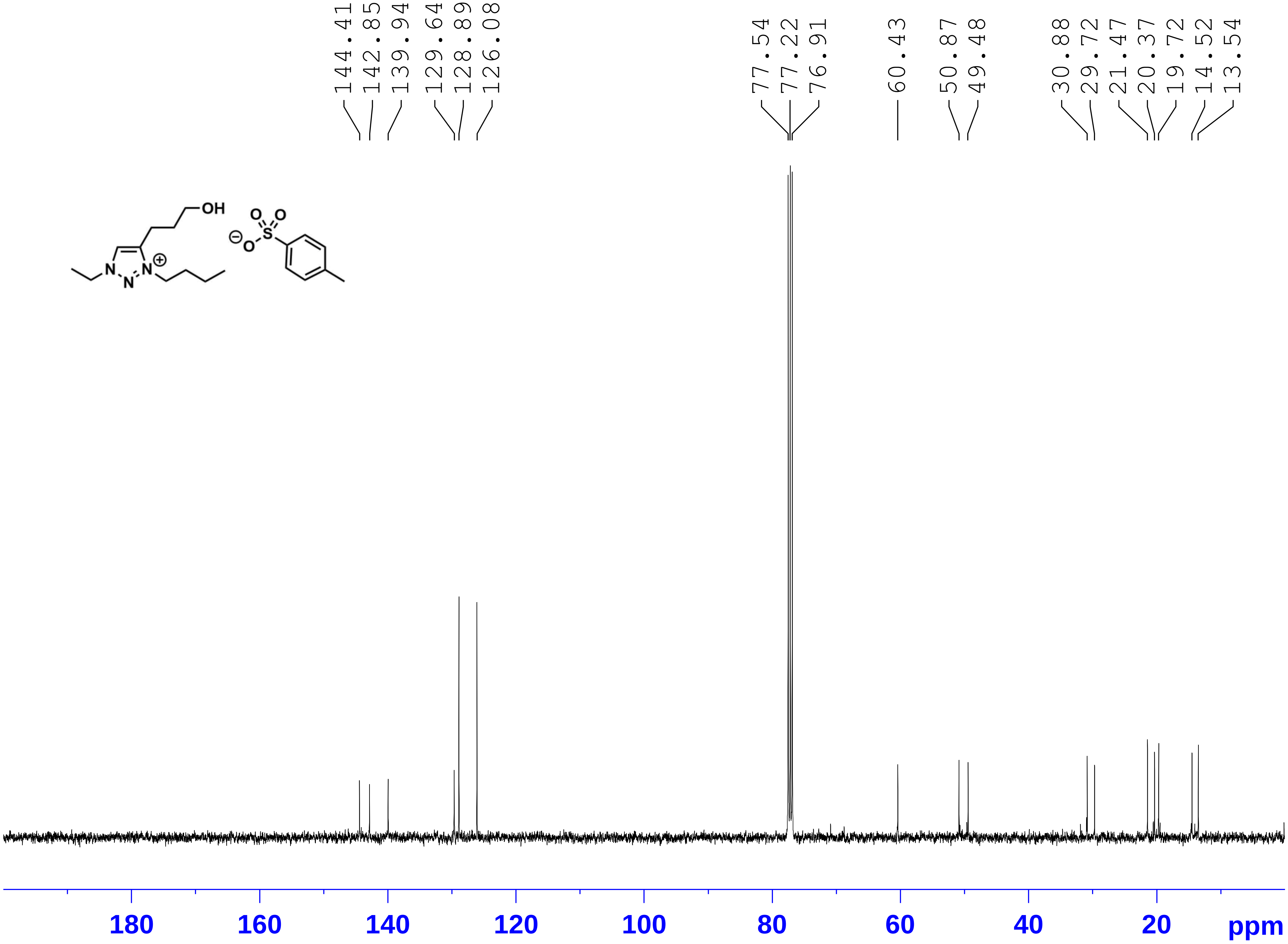
144.41
142.85
139.94
129.64
128.89
126.08
77.54
77.22
76.91
60.43
50.87
49.48
30.88
29.72
21.47
20.37
19.72
14.52
13.54
OH
180
160
140
120
100
80
60
40
20
ppm

Spectrum

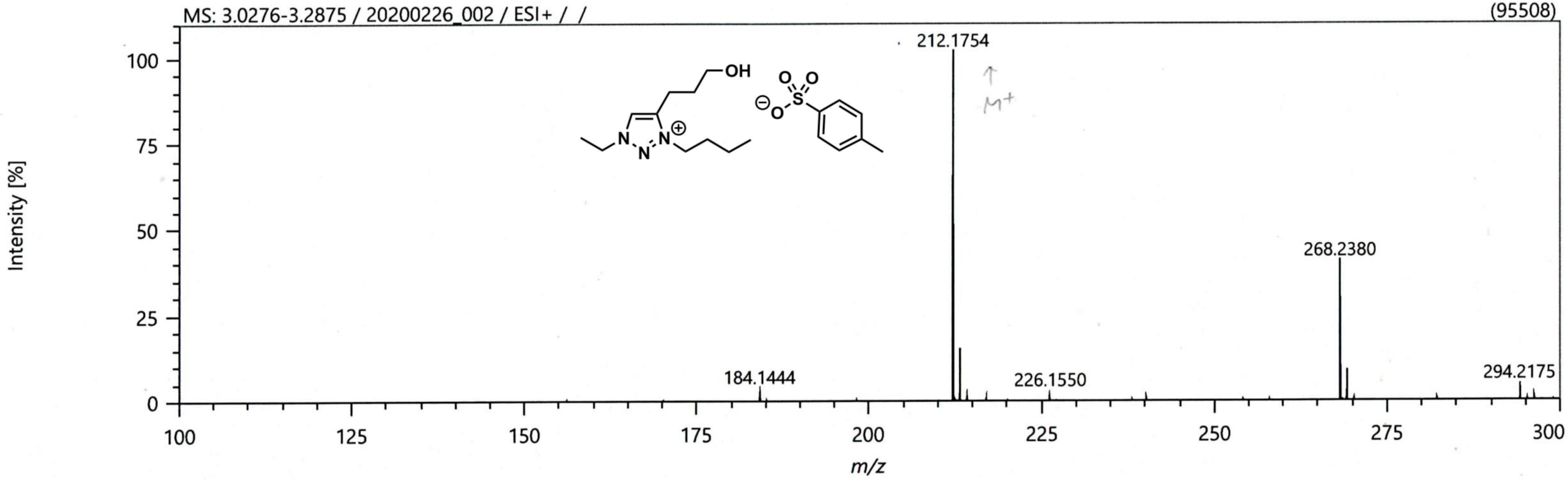

Elemental Composition

Parameters

			Elements Set 1:							
Tolerance:	±10.00 ppm	Symbol	C	H	O	Na	N	S	Ni	F
Electron:	Odd/Even	Min	0	0	1	0	3	0	0	0
Charge:	+1	Max	1000	4000	1	0	3	0	0	0
DBE:	-99.0 - 999.0									

Results

Mass	Formula	Calculated Mass	Mass Difference [mDa]	Mass Difference [ppm]	DBE
212.17535	C11 H22 N3 O	212.17574	-0.39	-1.82	2.5

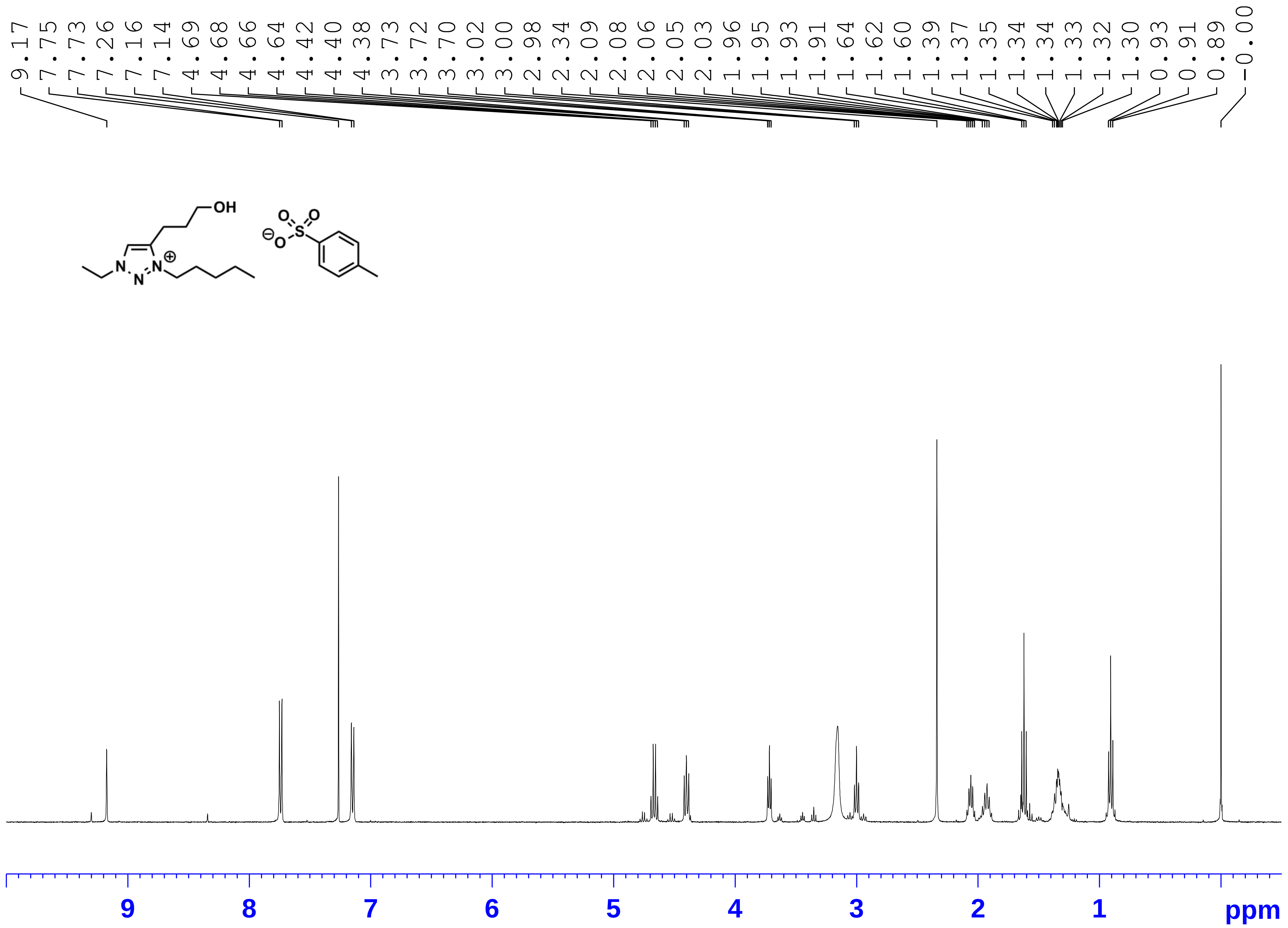

9.17
7.75
7.73
7.26
7.16
7.14
4.69
4.68
4.66
4.64
4.42
4.40
4.38
3.73
3.72
3.70
3.02
3.00
2.98
2.34
2.09
2.08
2.06
2.05
2.03
1.96
1.95
1.93
1.91
1.64
1.62
1.60
1.39
1.37
1.35
1.34
1.34
1.33
1.33
1.32
1.30
0.93
0.91
0.89
-0.00
OH
N
N
N
O
S
O
O
9
8
7
6
5
4
3
2
1
ppm

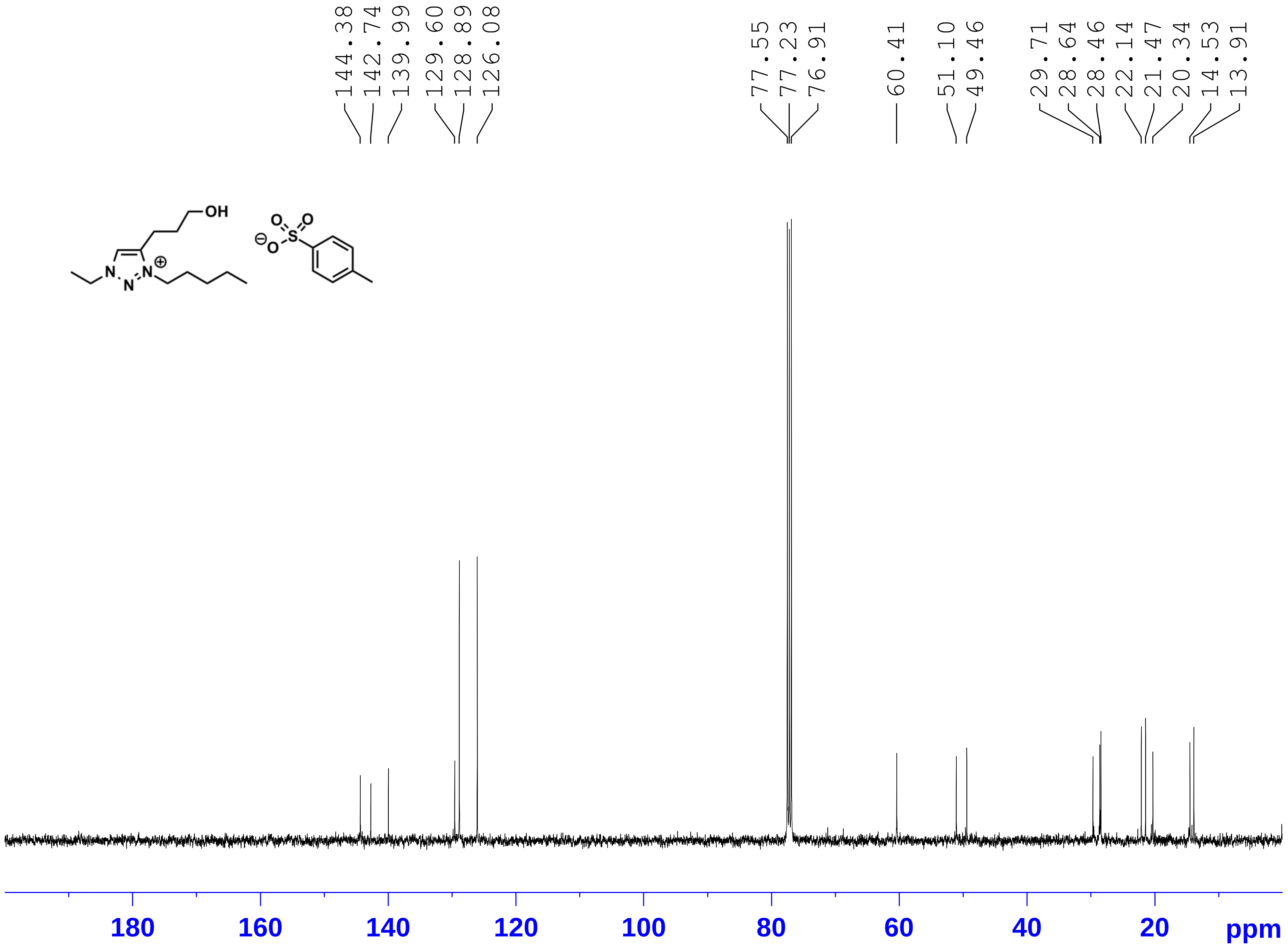
144.38
142.74
139.99
129.60
128.89
126.08
77.55
77.23
76.91
60.41
51.10
49.46
29.71
28.64
28.46
22.14
21.47
20.34
14.53
13.91
OH
N
N
N
O
O
S
O
180
160
140
120
100
80
60
40
20
ppm

Spectrum

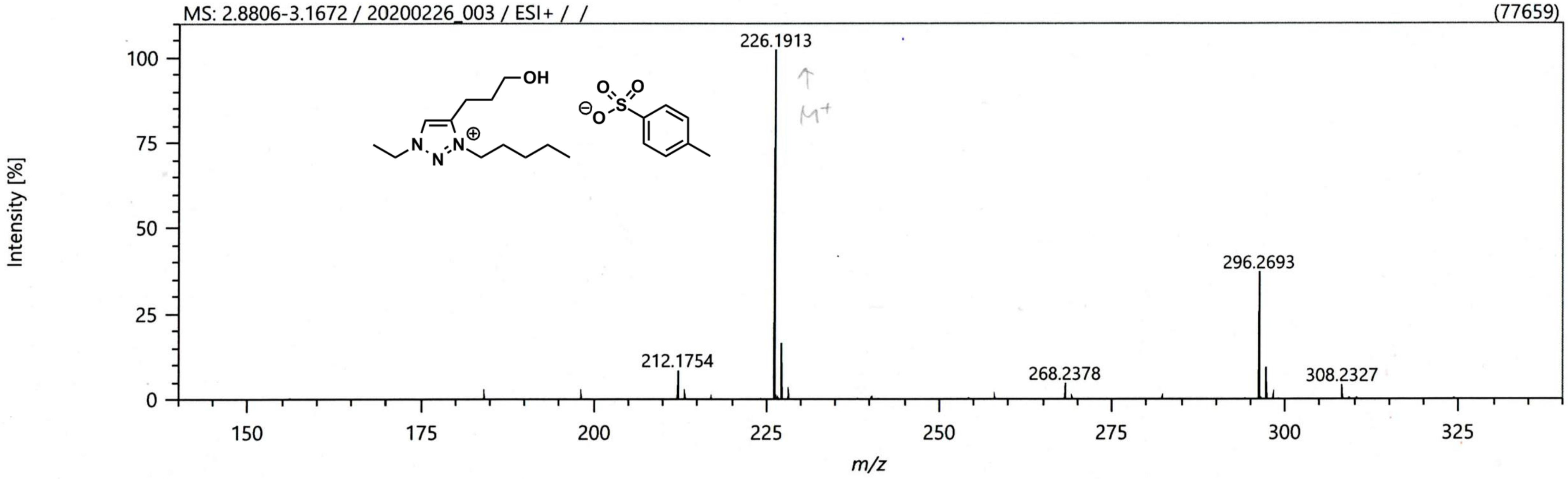

Elemental Composition

Parameters

Tolerance:	±10.00 ppm
Electron:	Odd/Even
Charge:	+1
DBE:	-99.0 - 999.0

Elements Set 1:

Symbol	C	H	O	Na	N	S	Ni	F
Min	0	0	1	0	3	0	0	0
Max	1000	4000	1	0	3	0	0	0

Results

Mass	Formula	Calculated Mass	Mass Difference [mDa]	Mass Difference [ppm]	DBE
226.19126	C12 H24 N3 O	226.19139	-0.13	-0.58	2.5

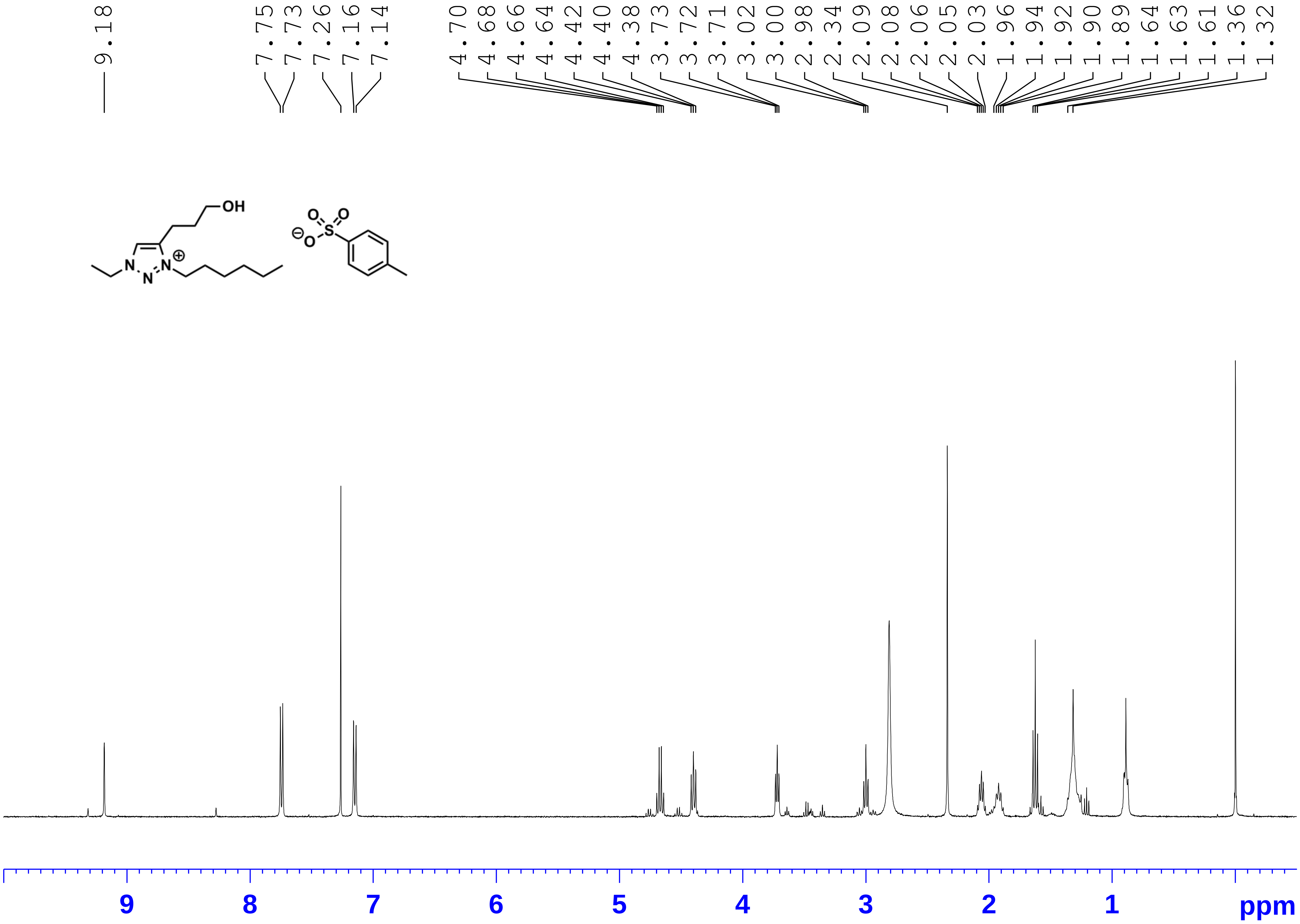

9.18
7.75
7.73
7.26
7.16
7.14
4.70
4.68
4.66
4.64
4.42
4.40
4.38
3.73
3.72
3.71
3.02
3.00
2.98
2.34
2.09
2.08
2.06
2.05
2.03
1.96
1.94
1.92
1.90
1.89
1.64
1.63
1.61
1.36
1.32
OH
O
O
S
O
N
N
N
9
8
7
6
5
4
3
2
1
ppm

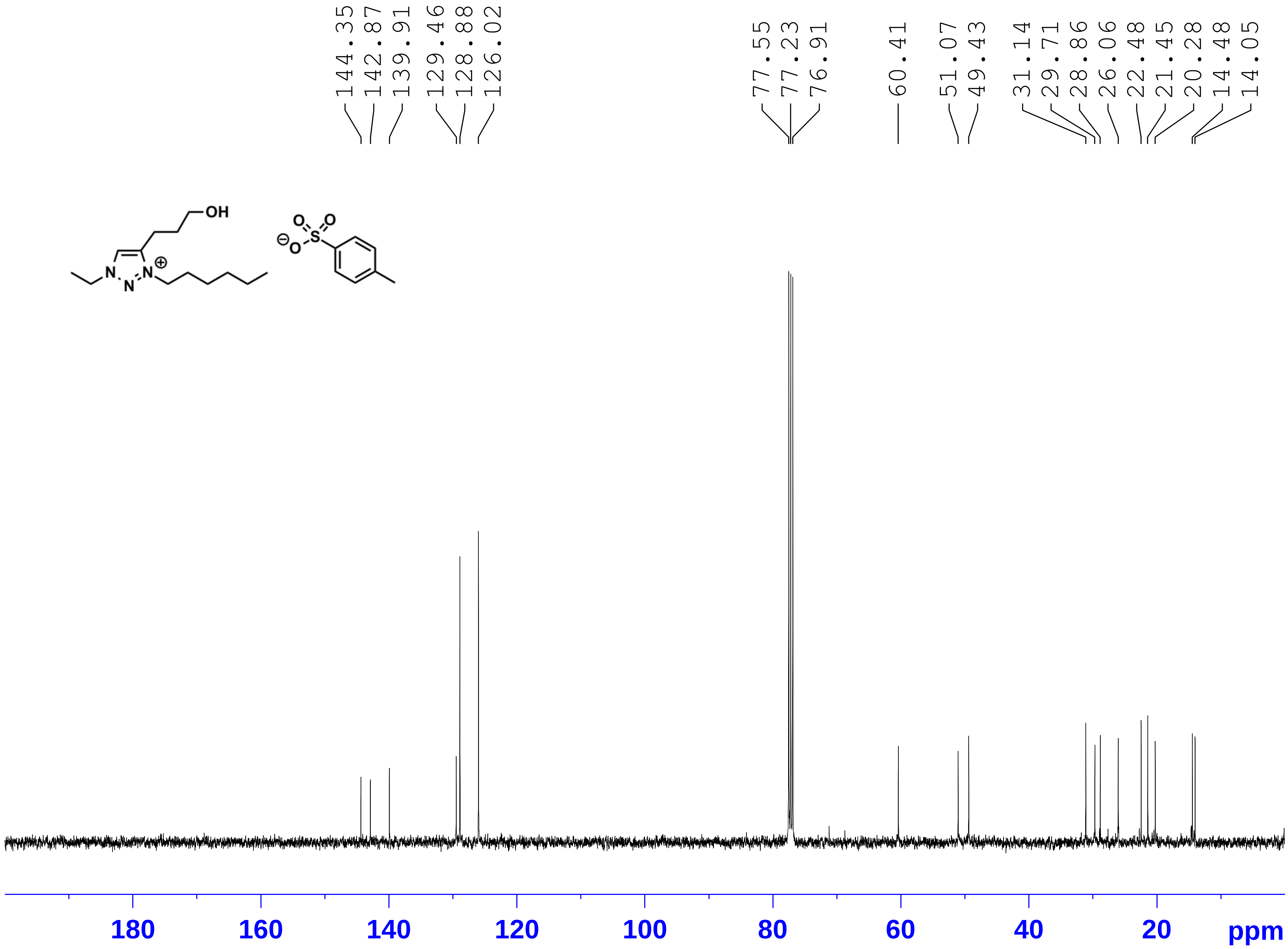
144.35
142.87
139.91
129.46
128.88
126.02
77.55
77.23
76.91
60.41
51.07
49.43
31.14
29.71
28.86
26.06
22.48
21.45
20.28
14.48
14.05
OH
N
N
N
O
S
O
O
180
160
140
120
100
80
60
40
20
ppm

Spectrum

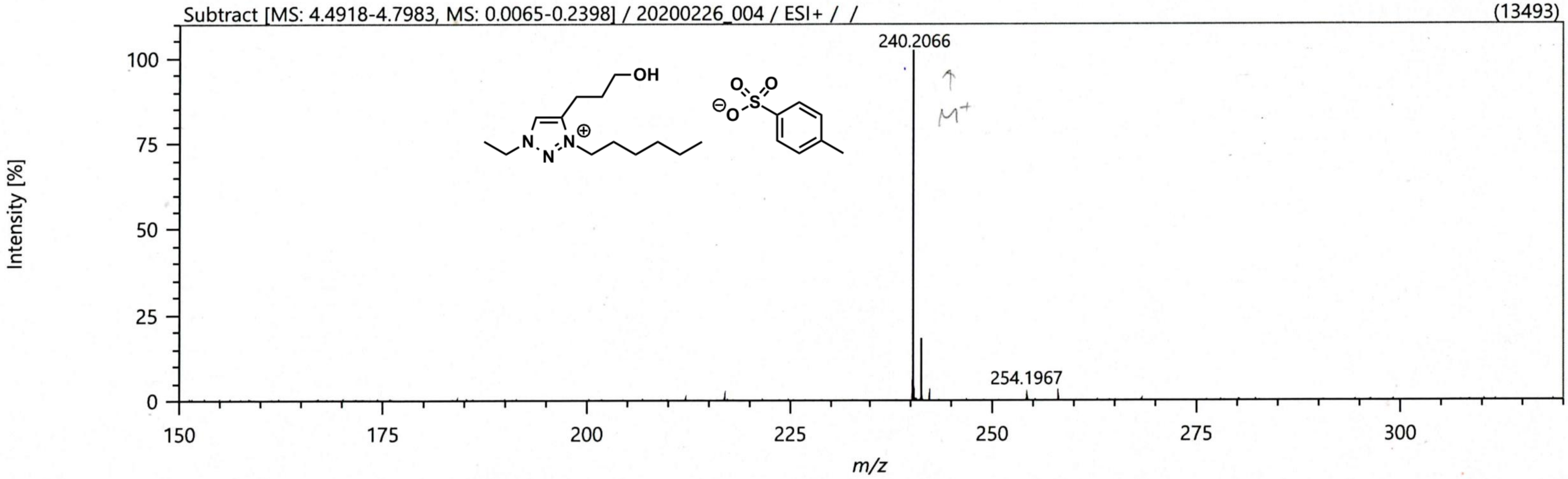

Elemental Composition

Parameters

Tolerance:	±10.00 ppm
Electron:	Odd/Even
Charge:	+1
DBE:	-99.0 - 999.0

Elements Set 1:

Symbol	C	H	O	Na	N	S	Ni	F
Min	0	0	1	0	3	0	0	0
Max	1000	4000	1	0	3	0	0	0

Results

Mass	Formula	Calculated Mass	Mass Difference [mDa]	Mass Difference [ppm]	DBE
240.20662	C13 H26 N3 O	240.20704	-0.42	-1.73	2.5

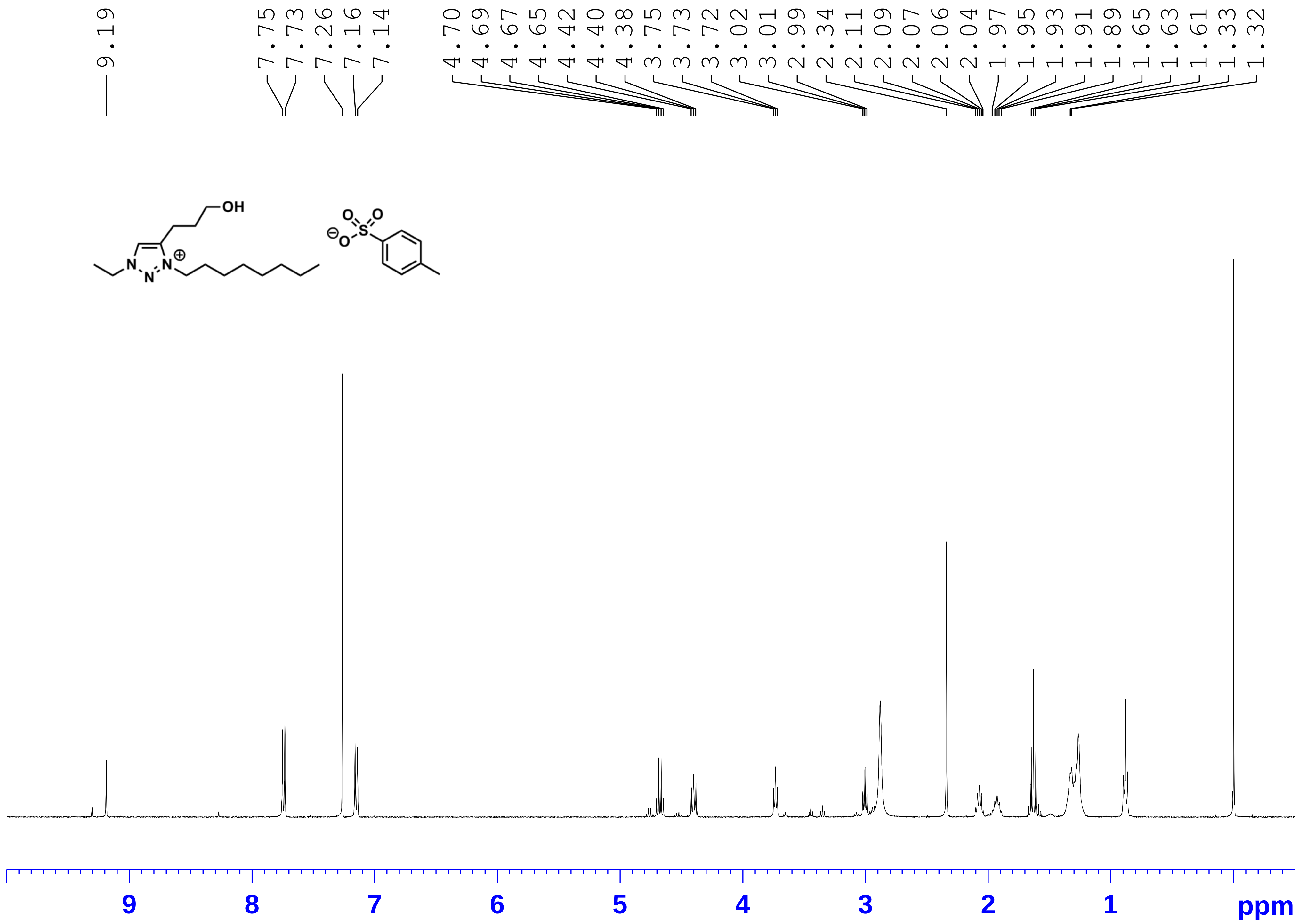

9.19
7.75
7.73
7.26
7.16
7.14
4.70
4.69
4.67
4.65
4.42
4.40
4.38
3.75
3.73
3.72
3.02
3.01
2.99
2.34
2.11
2.09
2.07
2.06
2.04
1.97
1.95
1.93
1.91
1.89
1.65
1.63
1.61
1.33
1.32
OH
N
N
N
O
O
S
O
9
8
7
6
5
4
3
2
1
ppm

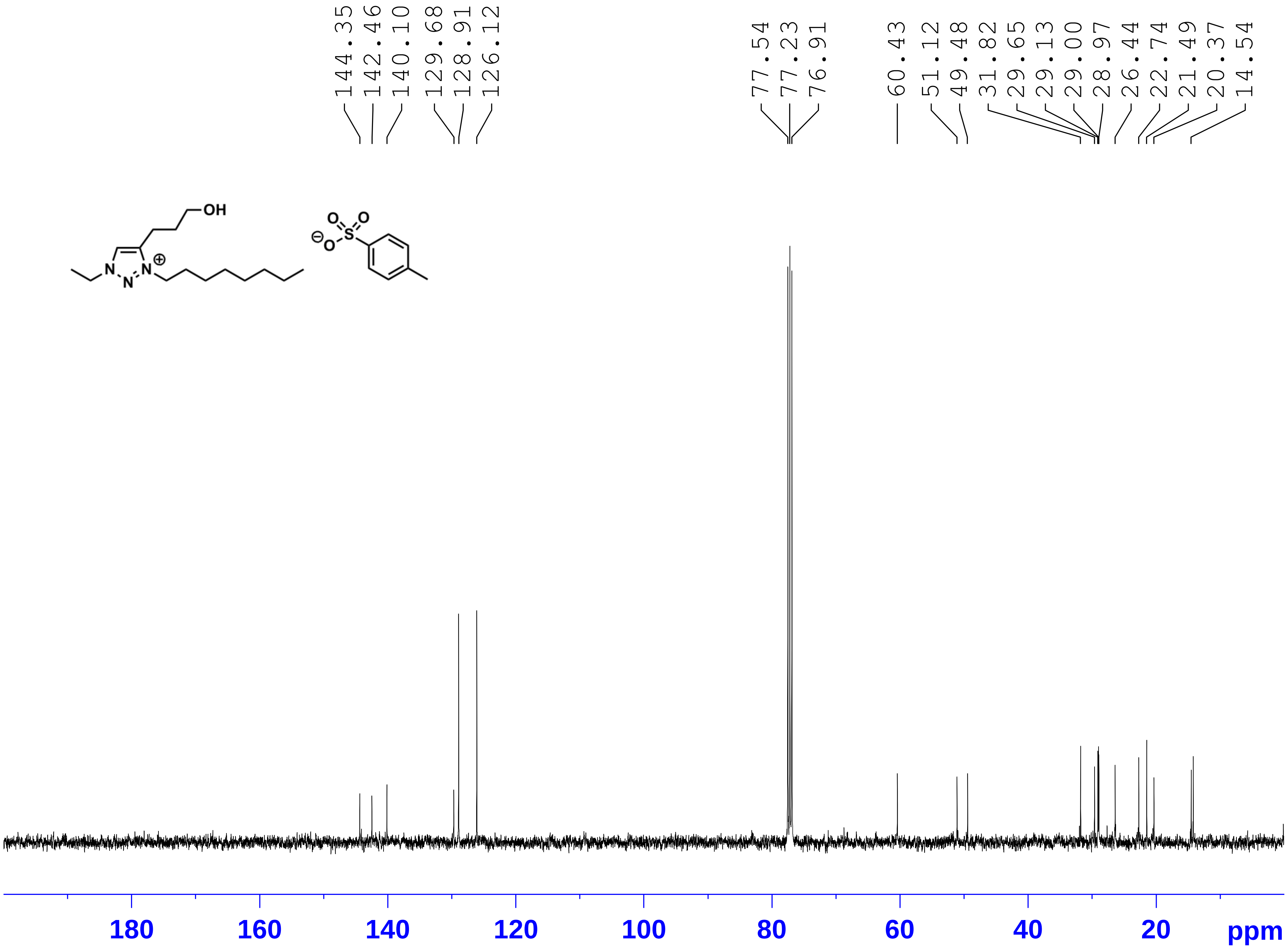
144.35
142.46
140.10
129.68
128.91
126.12
77.54
77.23
76.91
60.43
51.12
49.48
31.82
29.65
29.13
29.00
28.97
26.44
22.74
21.49
20.37
14.54
OH
N
N
N
O
O
S
O
180
160
140
120
100
80
60
40
20
ppm

Spectrum

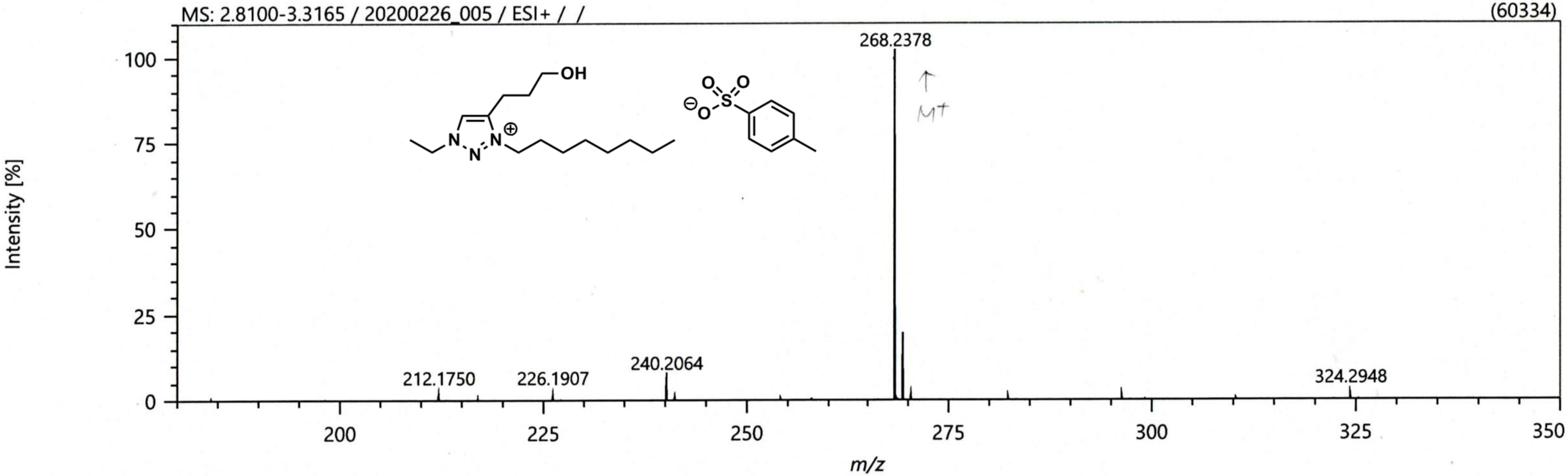

Elemental Composition

Parameters

Tolerance:	±10.00 ppm
Electron:	Odd/Even
Charge:	+1
DBE:	-99.0 - 999.0

Elements Set 1:

Symbol	C	H	O	Na	N	S	Ni	F
Min	0	0	1	0	3	0	0	0
Max	1000	4000	1	0	3	0	0	0

Results

Mass	Formula	Calculated Mass	Mass Difference [mDa]	Mass Difference [ppm]	DBE
268.23780	C15 H30 N3 O	268.23834	-0.53	-1.99	2.5

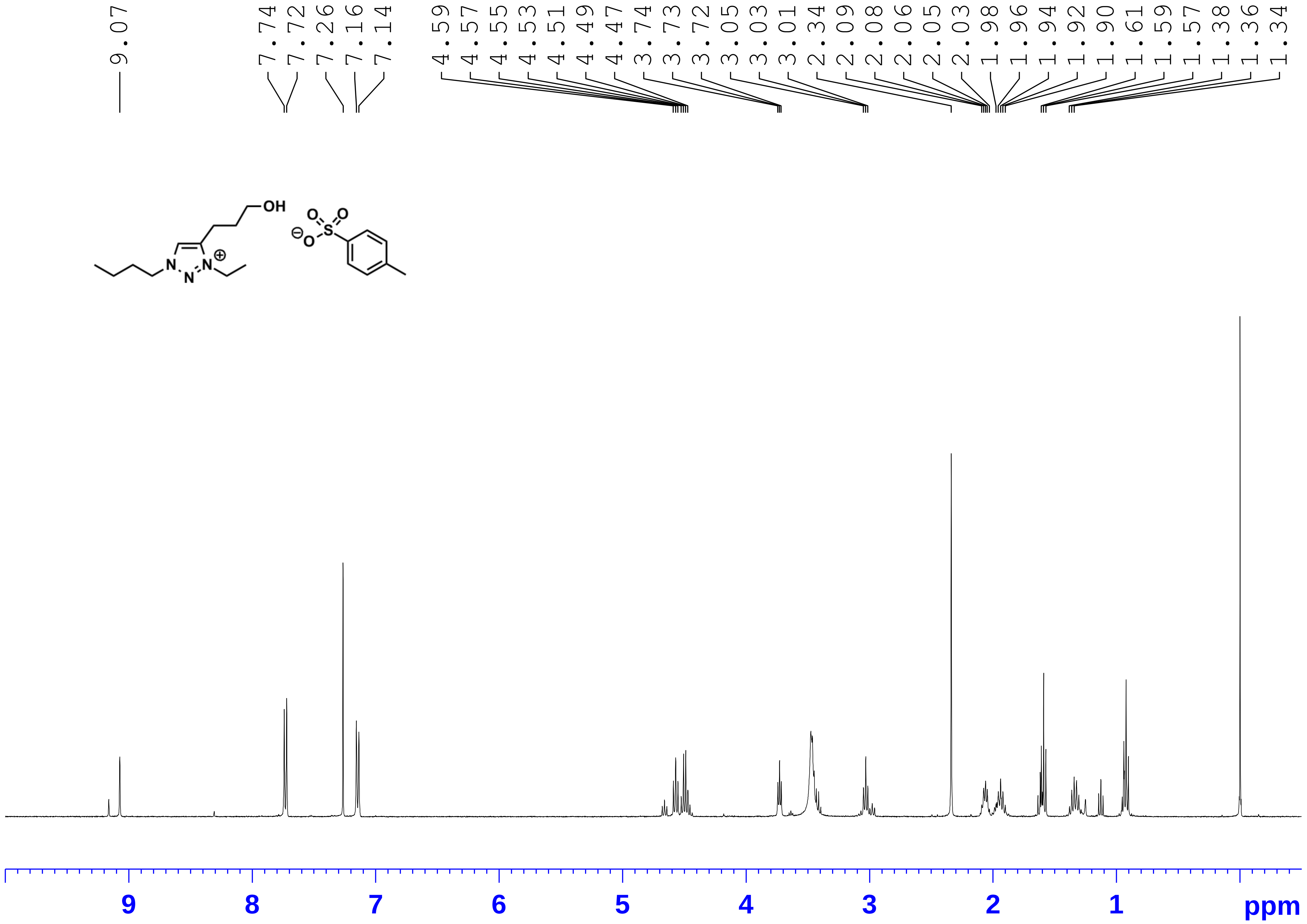
9.07
7.74
7.72
7.26
7.16
7.14
4.59
4.57
4.55
4.53
4.51
4.49
4.47
3.74
3.73
3.72
3.05
3.03
3.01
2.34
2.09
2.08
2.06
2.05
2.03
1.98
1.96
1.94
1.92
1.90
1.61
1.59
1.57
1.38
1.36
1.34
OH
O
O
S
O
N
N
N
9
8
7
6
5
4
3
2
1
ppm

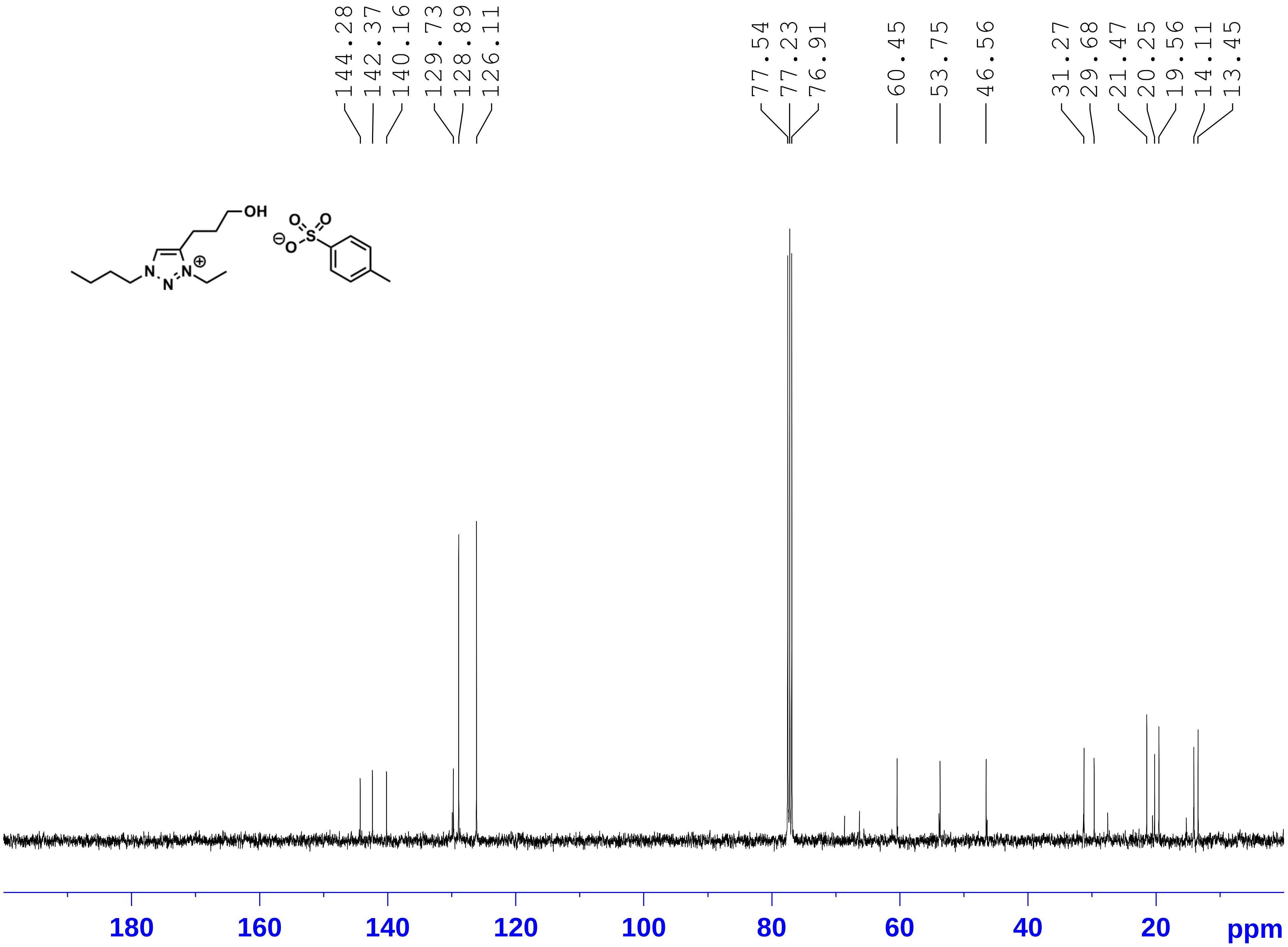

144.28
142.37
140.16
129.73
128.89
126.11
77.54
77.23
76.91
60.45
53.75
46.56
31.27
29.68
21.47
20.25
19.56
14.11
13.45
OH
O
S
O
N
N
N
180
160
140
120
100
80
60
40
20
ppm

Spectrum

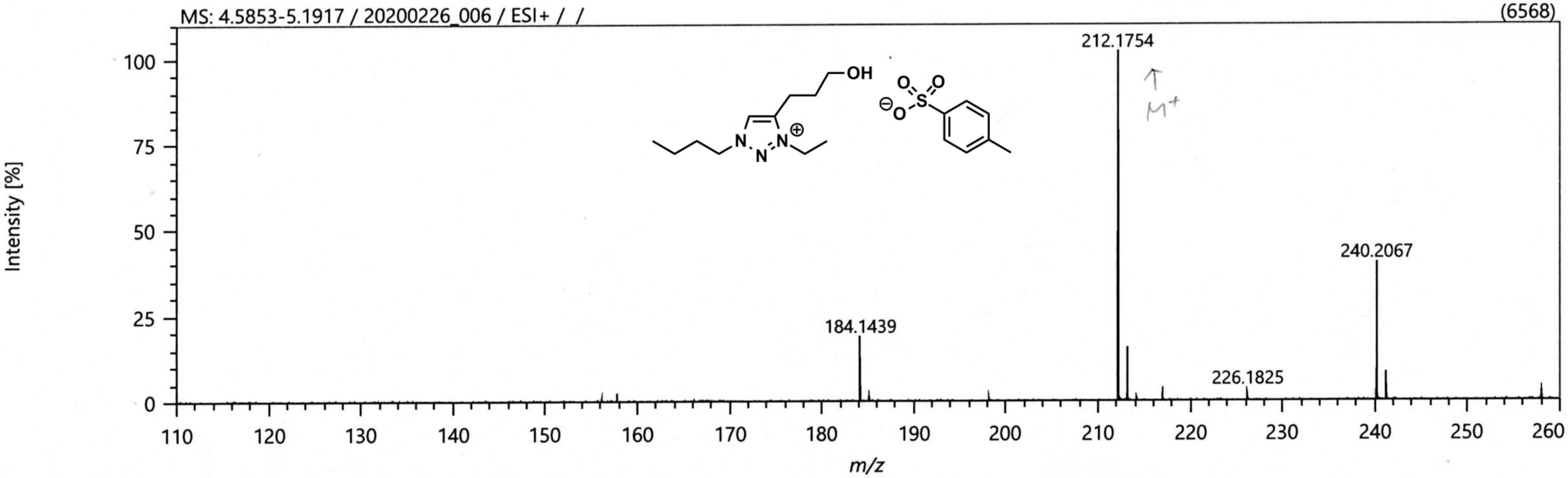

Elemental Composition

Parameters		Elements Set 1:								
Tolerance:	±10.00 ppm	Symbol	C	H	O	Na	N	S	Ni	F
Electron:	Odd/Even	Min	0	0	1	0	3	0	0	0
Charge:	+1	Max	1000	4000	1	0	3	0	0	0
DBE:	-99.0 - 999.0									

Results

Mass	Formula	Calculated Mass	Mass Difference [mDa]	Mass Difference [ppm]	DBE
212.17537	C11 H22 N3 O	212.17574	-0.36	-1.72	2.5

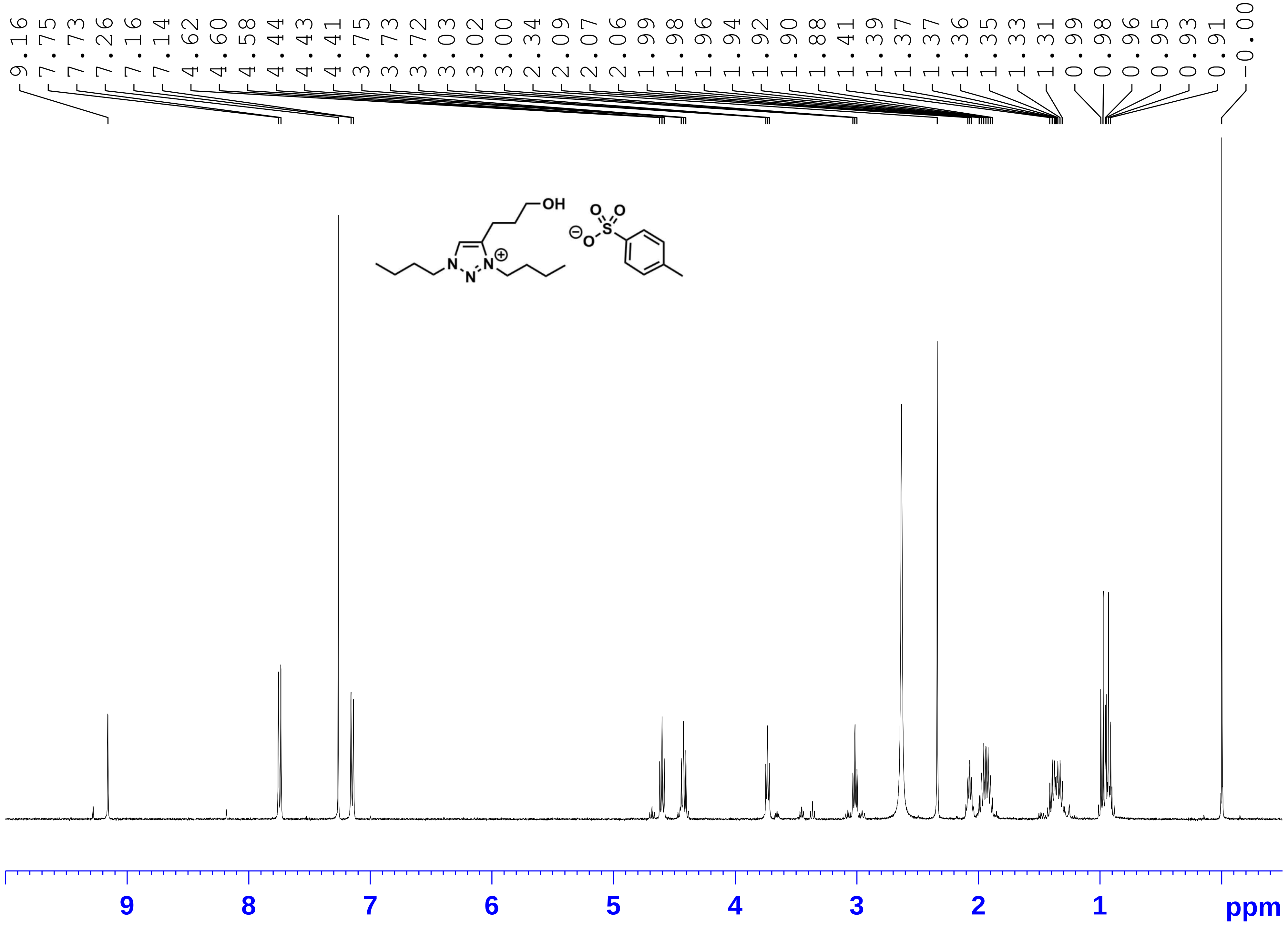
9.16
7.75
7.73
7.26
7.16
7.14
4.62
4.60
4.58
4.44
4.43
4.41
3.75
3.73
3.72
3.03
3.02
3.00
2.34
2.09
2.07
2.06
1.99
1.98
1.96
1.94
1.92
1.90
1.88
1.41
1.39
1.37
1.37
1.36
1.35
1.33
1.31
0.99
0.98
0.96
0.95
0.93
0.91
-0.00
OH
O
O
S
O
N
N
N
9
8
7
6
5
4
3
2
1
ppm

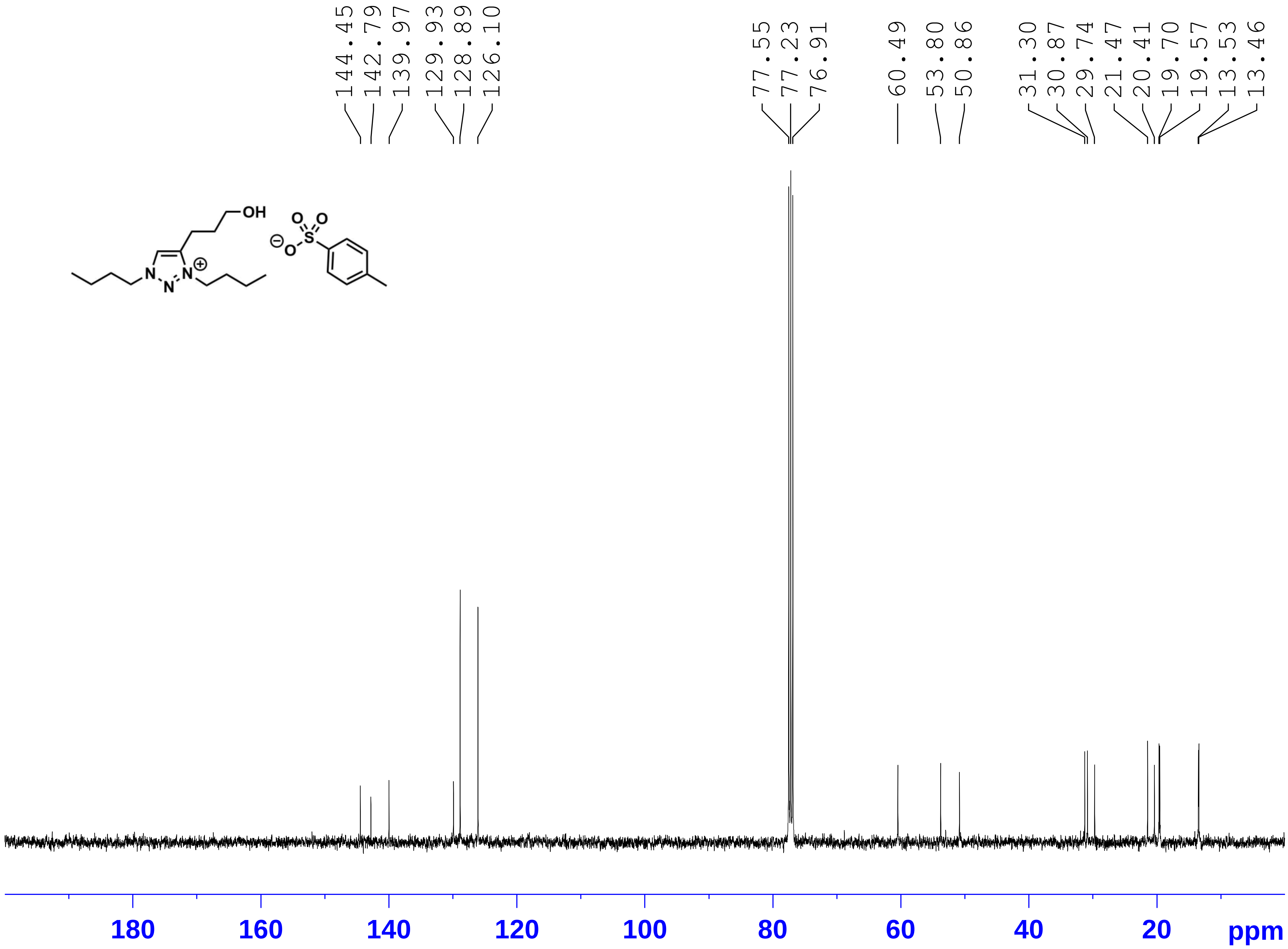
144.45
142.79
139.97
129.93
128.89
126.10
77.55
77.23
76.91
60.49
53.80
50.86
31.30
30.87
29.74
21.47
20.41
19.70
19.57
13.53
13.46
OH
O
O
S
O
N
N
N
180
160
140
120
100
80
60
40
20
ppm

Spectrum

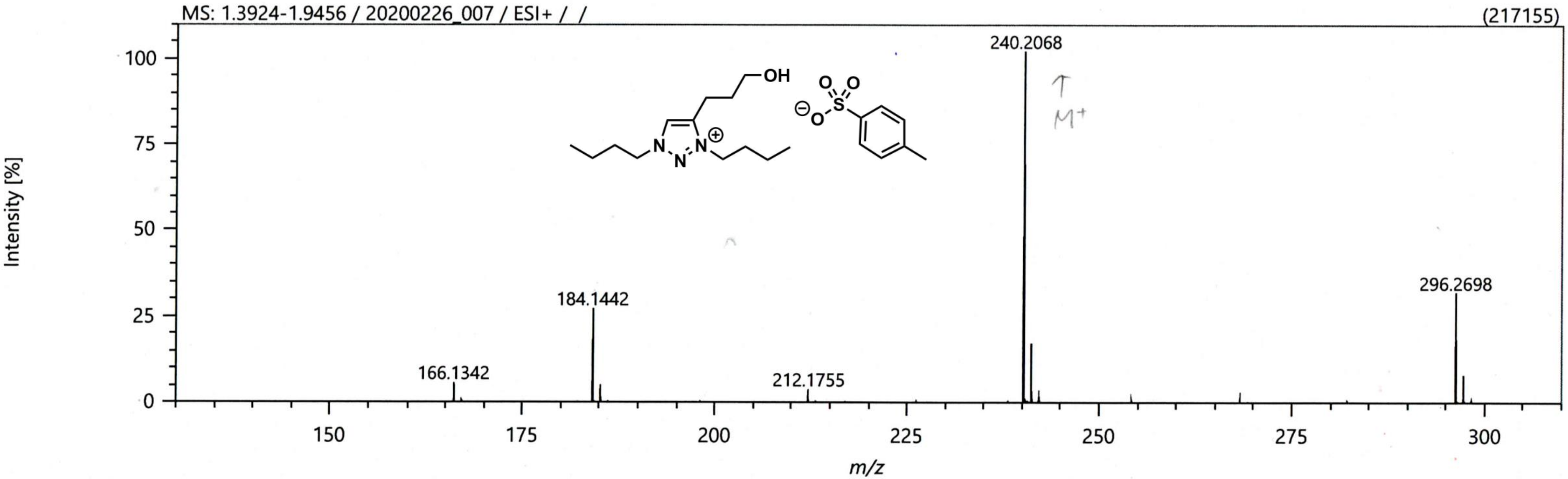

Elemental Composition

Parameters

		Elements Set 1:								
Tolerance:	±10.00 ppm	Symbol	C	H	O	Na	N	S	Ni	F
Electron:	Odd/Even	Min	0	0	1	0	3	0	0	0
Charge:	+1	Max	1000	4000	1	0	3	0	0	0
DBE:	-99.0 - 999.0									

Results

Mass	Formula	Calculated Mass	Mass Difference [mDa]	Mass Difference [ppm]	DBE
240.20684	C13 H26 N3 O	240.20704	-0.20	-0.82	2.5

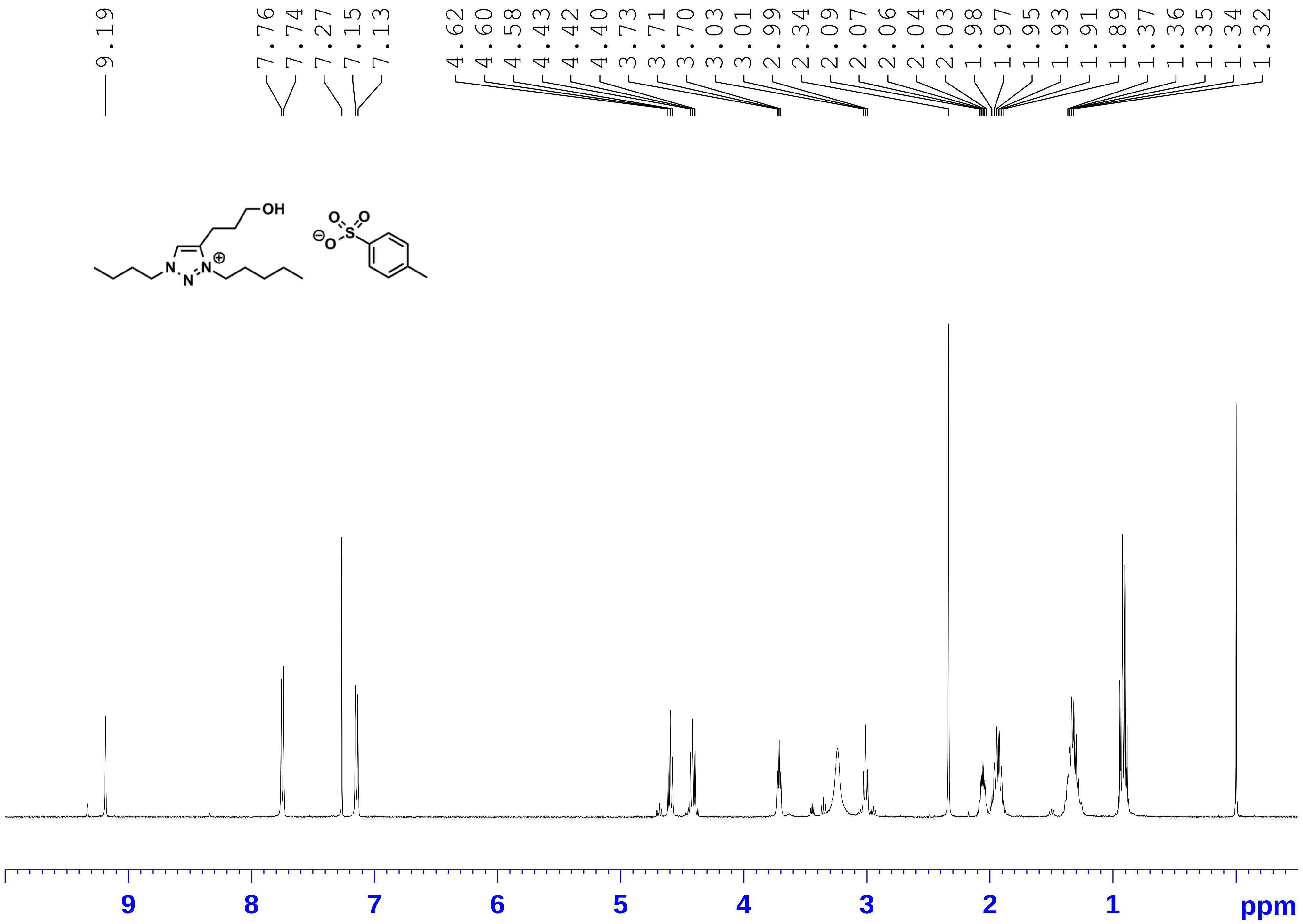
9.19
7.76
7.74
7.27
7.15
7.13
4.62
4.60
4.58
4.43
4.42
4.40
3.73
3.71
3.70
3.03
3.01
2.99
2.34
2.09
2.07
2.06
2.04
2.03
1.98
1.97
1.95
1.93
1.91
1.89
1.37
1.36
1.35
1.34
1.32
OH
N
N
N
O
O
O
S
9
8
7
6
5
4
3
2
1
ppm

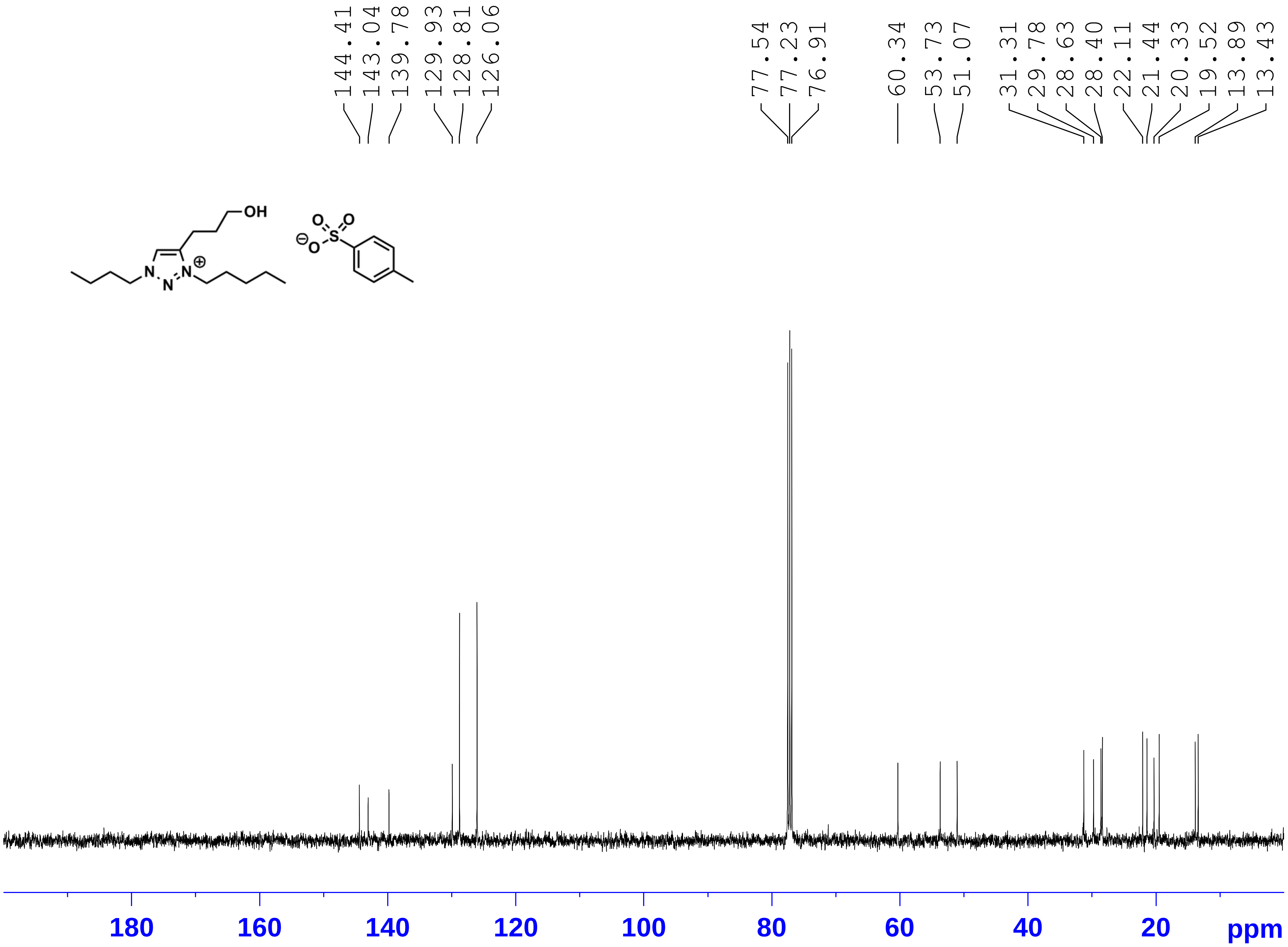
144.41
143.04
139.78
129.93
128.81
126.06
77.54
77.23
76.91
60.34
53.73
51.07
31.31
29.78
28.63
28.40
22.11
21.44
20.33
19.52
13.89
13.43
OH
O
S
O
⊖O
N
N
N⊕
180
160
140
120
100
80
60
40
20
ppm

Spectrum

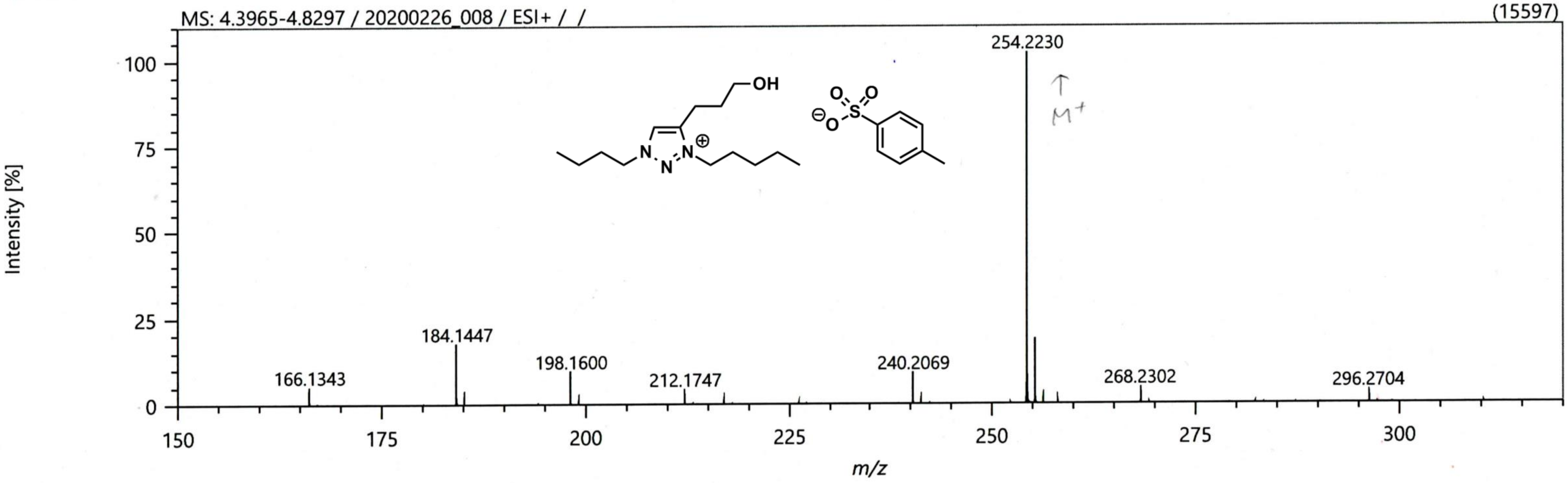

Elemental Composition

Parameters		Elements Set 1:								
Tolerance:	±10.00 ppm	Symbol	C	H	O	Na	N	S	Ni	F
Electron:	Odd/Even	Min	0	0	1	0	3	0	0	0
Charge:	+1	Max	1000	4000	1	0	3	0	0	0
DBE:	-99.0 - 999.0									

Results

Mass	Formula	Calculated Mass	Mass Difference [mDa]	Mass Difference [ppm]	DBE
254.22299	C14 H28 N3 O	254.22269	0.30	1.19	2.5

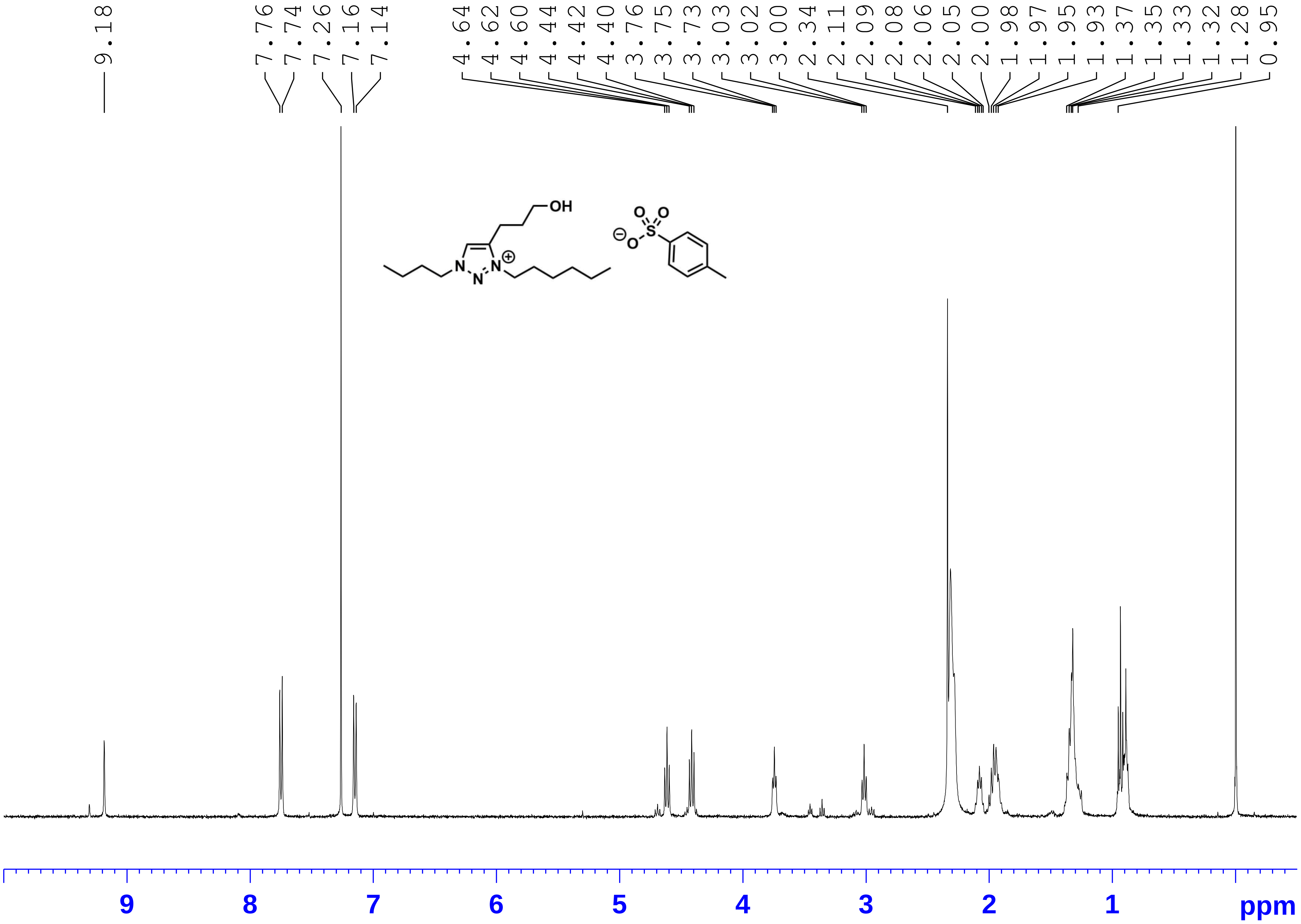
9.18
7.76
7.74
7.26
7.16
7.14
4.64
4.62
4.60
4.44
4.42
4.40
3.76
3.75
3.73
3.03
3.02
3.00
2.34
2.11
2.09
2.08
2.06
2.05
2.00
1.98
1.97
1.95
1.93
1.37
1.35
1.33
1.32
1.28
0.95
OH
O
O
S
O
N
N
N
9
8
7
6
5
4
3
2
1
ppm

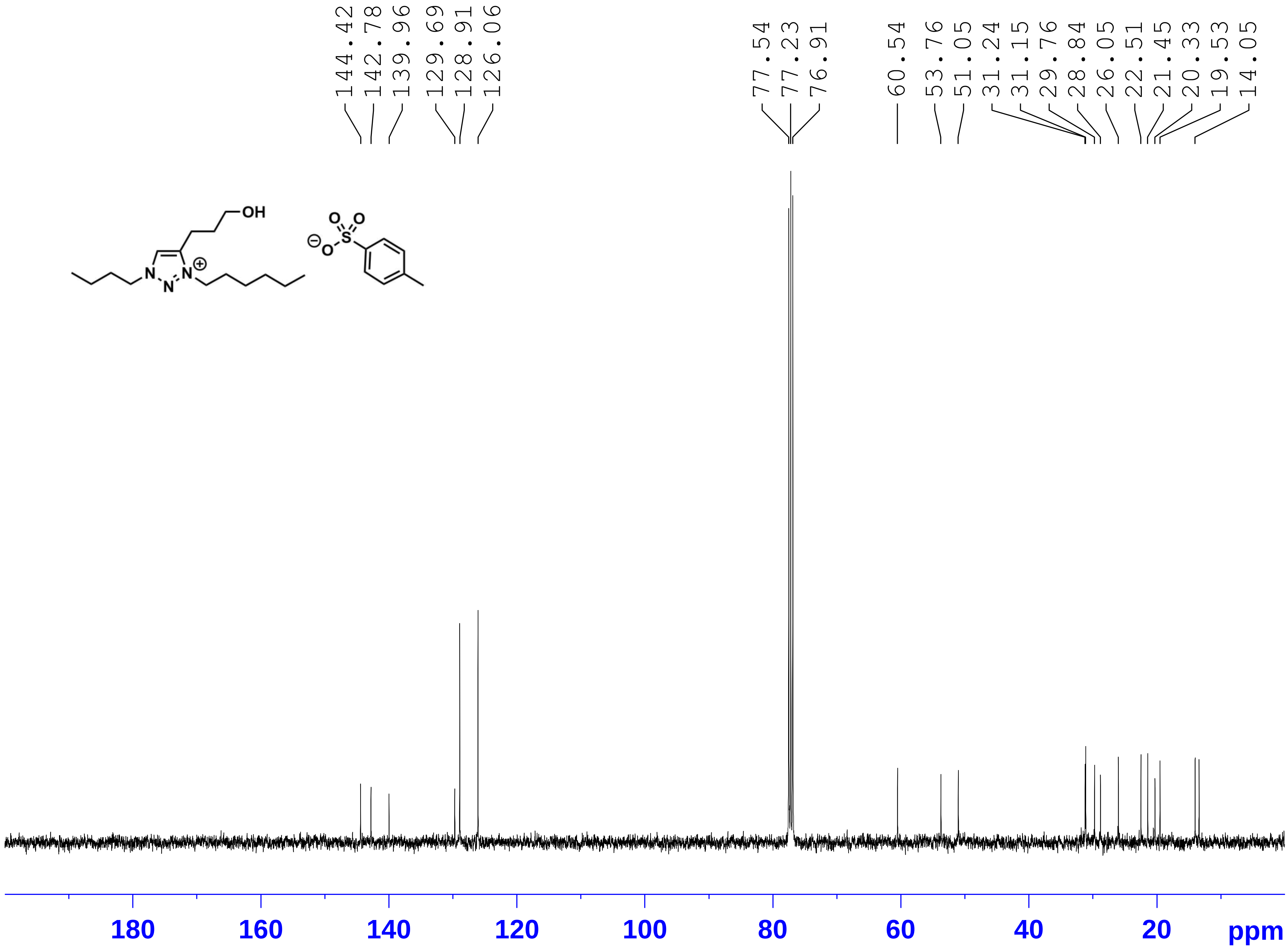
144.42
142.78
139.96
129.69
128.91
126.06
77.54
77.23
76.91
60.54
53.76
51.05
31.24
31.15
29.76
28.84
26.05
22.51
21.45
20.33
19.53
14.05
OH
N
N
N
O
O
S
O
180
160
140
120
100
80
60
40
20
ppm

Spectrum

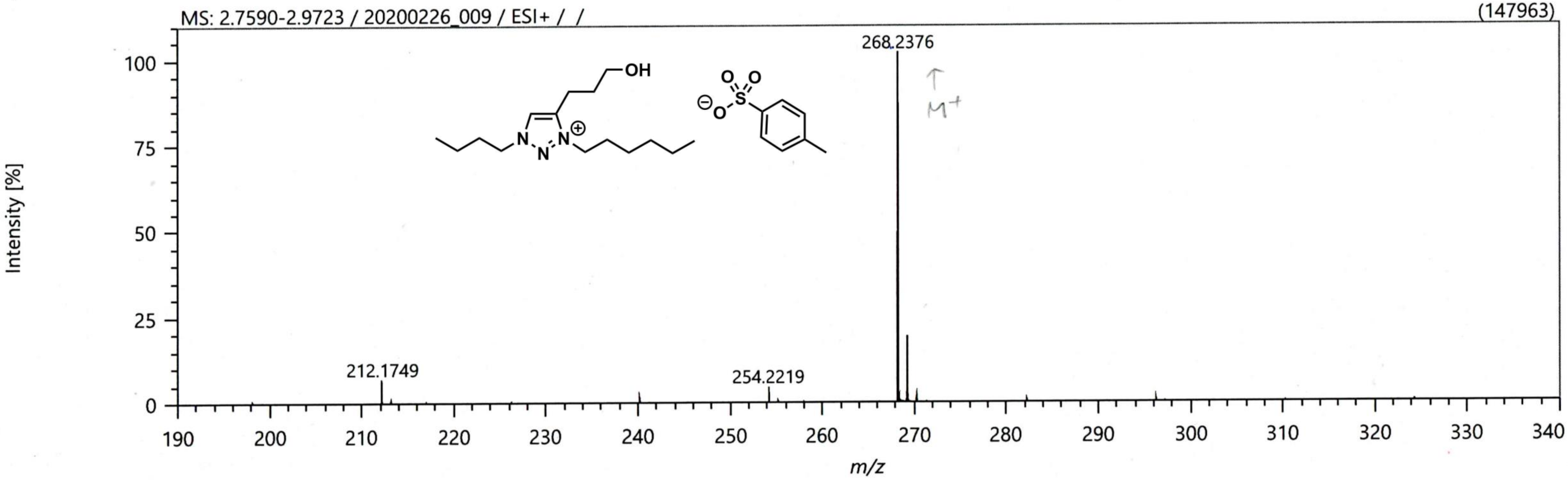

Elemental Composition

Parameters

Tolerance:	±10.00 ppm
Electron:	Odd/Even
Charge:	+1
DBE:	-99.0 - 999.0

Elements Set 1:

Symbol	C	H	O	Na	N	S	Ni	F
Min	0	0	1	0	3	0	0	0
Max	1000	4000	1	0	3	0	0	0

Results

Mass	Formula	Calculated Mass	Mass Difference [mDa]	Mass Difference [ppm]	DBE
268.23763	C15 H30 N3 O	268.23834	-0.71	-2.66	2.5

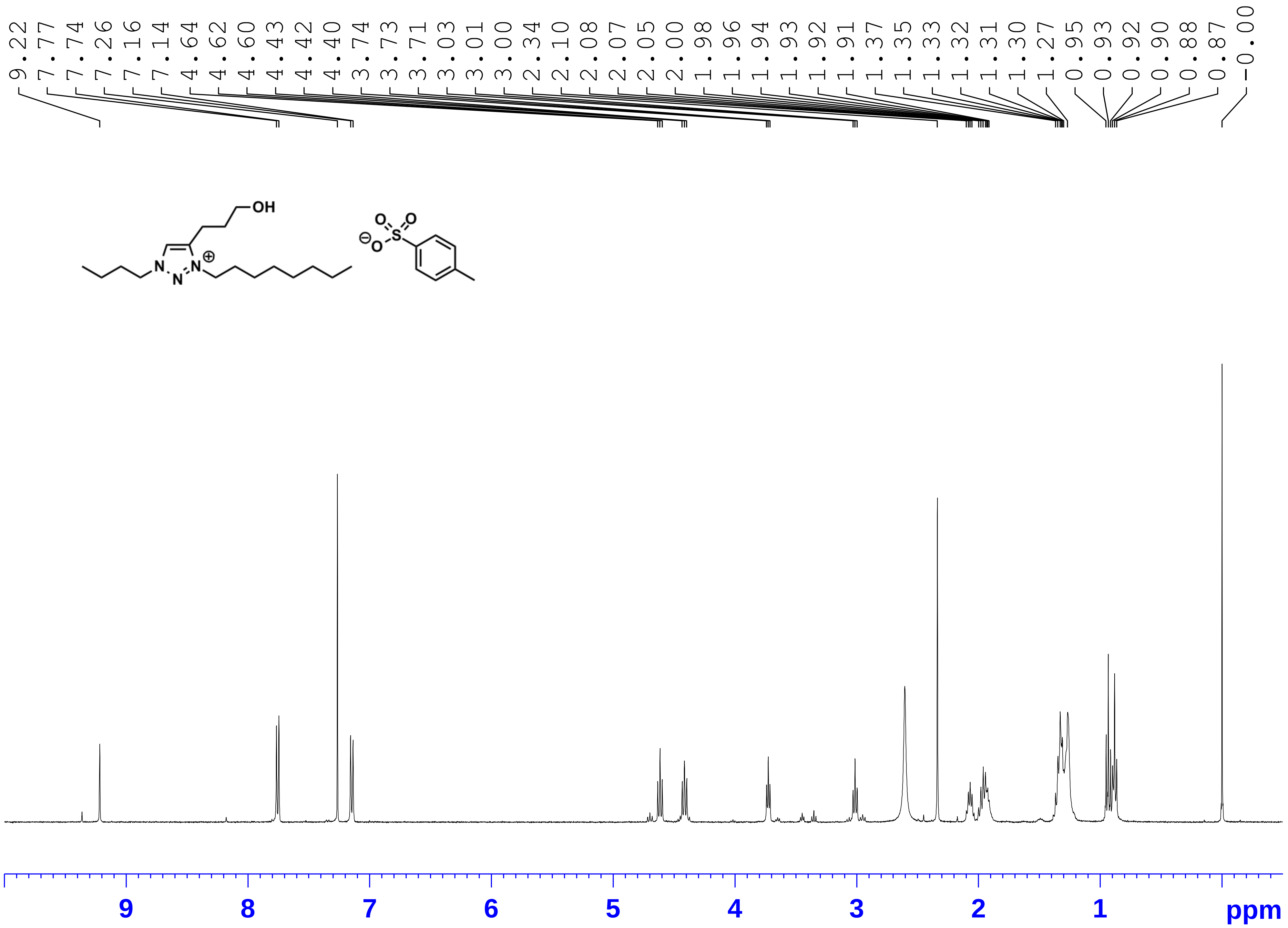
9.22
7.77
7.74
7.26
7.16
7.14
4.64
4.62
4.60
4.43
4.42
4.40
3.74
3.73
3.71
3.03
3.01
3.00
2.34
2.10
2.08
2.07
2.05
2.00
1.98
1.96
1.94
1.93
1.92
1.91
1.37
1.35
1.33
1.32
1.31
1.30
1.27
0.95
0.93
0.92
0.90
0.88
0.87
-0.00
OH
N
N
N
O
O
S
O
9
8
7
6
5
4
3
2
1
ppm

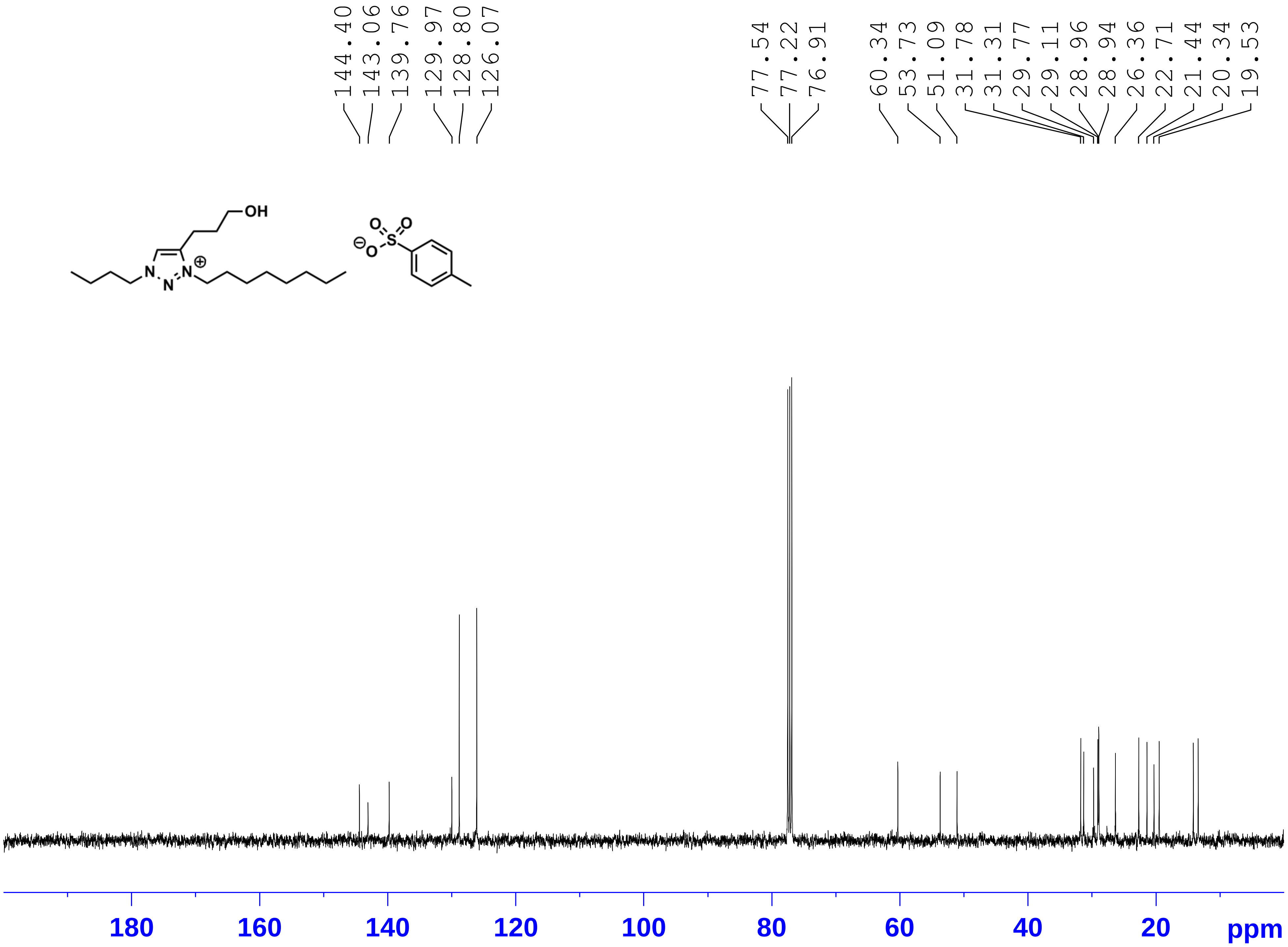

144.40
143.06
139.76
129.97
128.80
126.07
77.54
77.22
76.91
60.34
53.73
51.09
31.78
31.31
29.77
29.11
28.96
28.94
26.36
22.71
21.44
20.34
19.53
OH
O
S
O
N
N
N
180
160
140
120
100
80
60
40
20
ppm

Spectrum

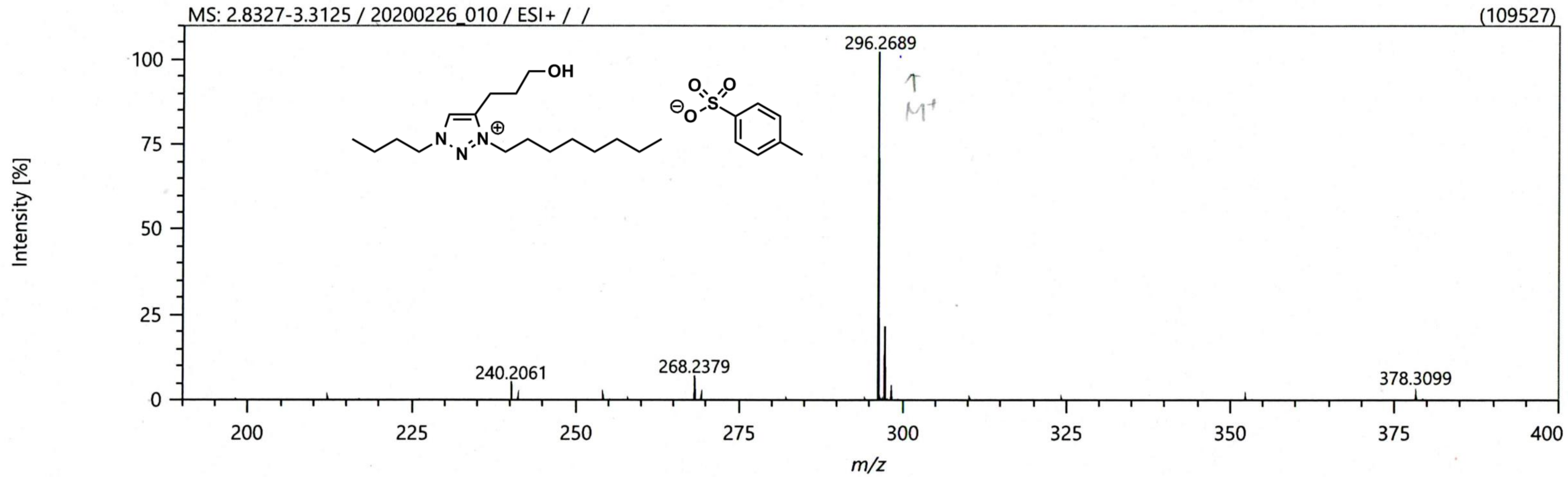

Elemental Composition

Parameters	
Tolerance:	±10.00 ppm
Electron:	Odd/Even
Charge:	+1
DBE:	-99.0 - 999.0

Elements Set 1:

Symbol	C	H	O	Na	N	S	Ni	F
Min	0	0	1	0	3	0	0	0
Max	1000	4000	1	0	3	0	0	0

Results

Mass	Formula	Calculated Mass	Mass Difference [mDa]	Mass Difference [ppm]	DBE
296.26890	C17 H34 N3 O	296.26964	-0.74	-2.50	2.5

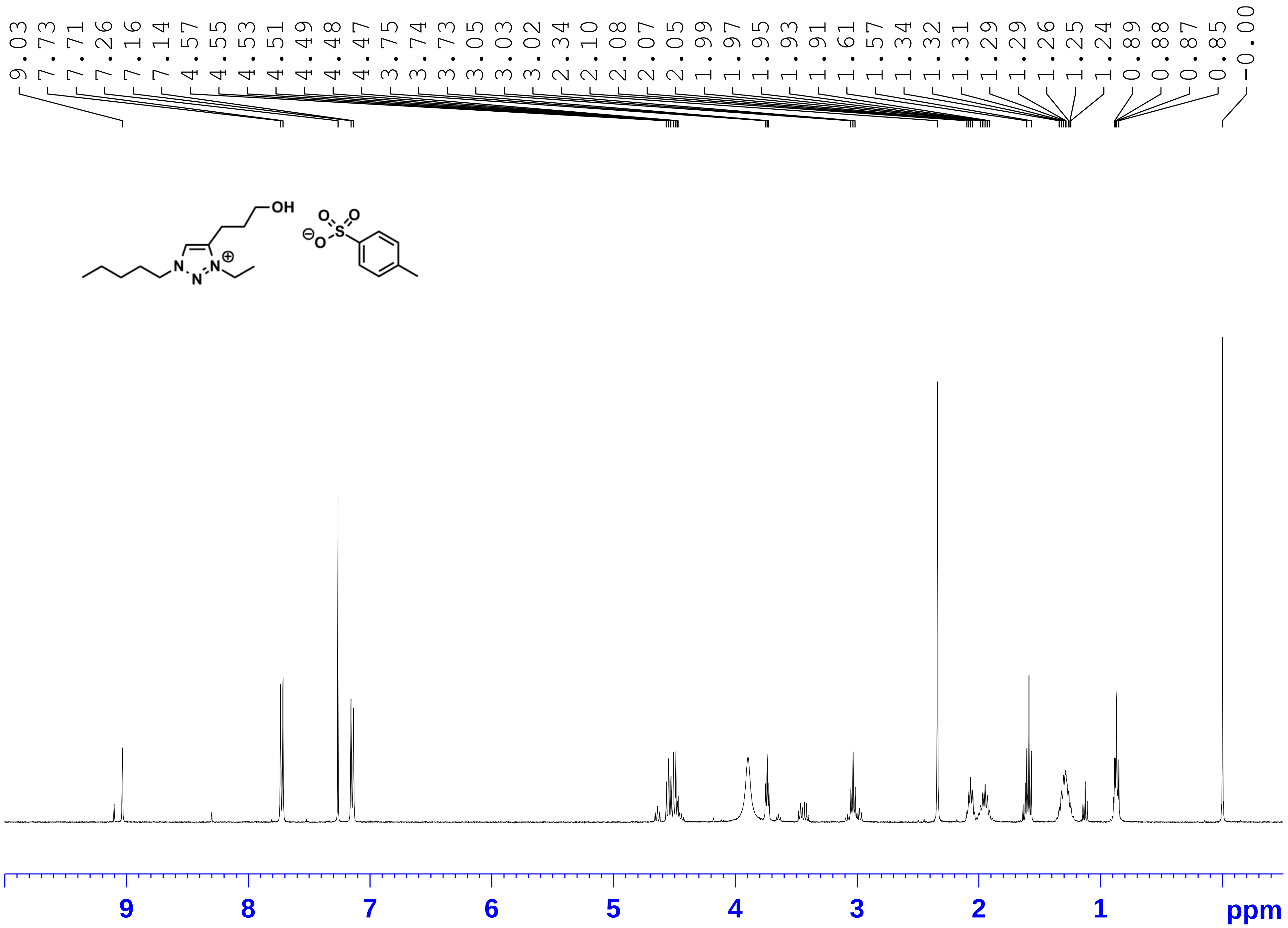
9.03
7.73
7.71
7.26
7.16
7.14
4.57
4.55
4.53
4.51
4.49
4.48
4.47
3.75
3.74
3.73
3.05
3.03
3.02
2.34
2.10
2.08
2.07
2.05
1.99
1.97
1.95
1.93
1.91
1.61
1.57
1.34
1.32
1.31
1.29
1.29
1.26
1.25
1.24
0.89
0.88
0.87
0.85
-0.00
OH
N
N
N
O
S
O
O
9
8
7
6
5
4
3
2
1
ppm

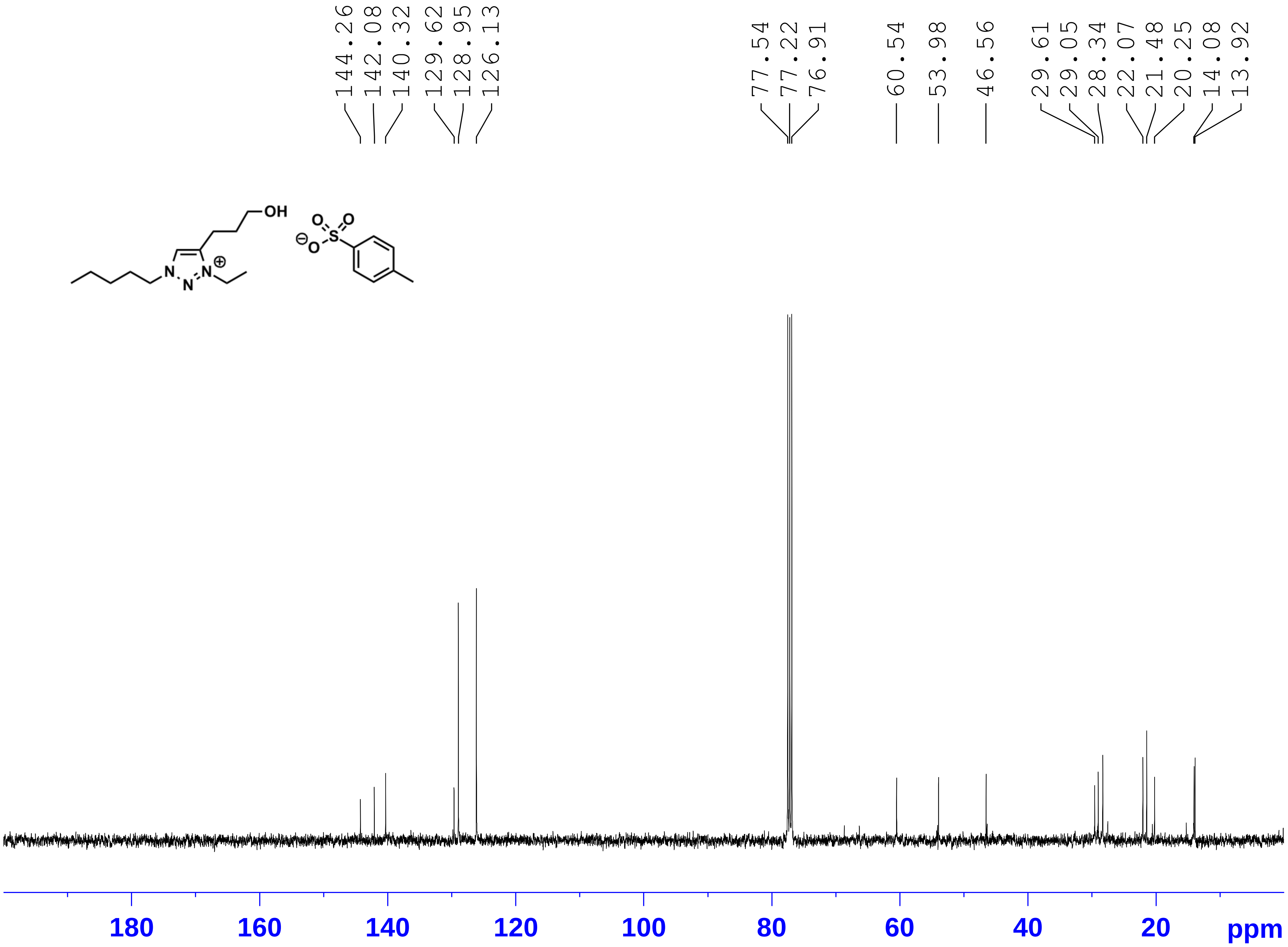

144.26
142.08
140.32
129.62
128.95
126.13
77.54
77.22
76.91
60.54
53.98
46.56
29.61
29.05
28.34
22.07
21.48
20.25
14.08
13.92
OH
180
160
140
120
100
80
60
40
20
ppm

Spectrum

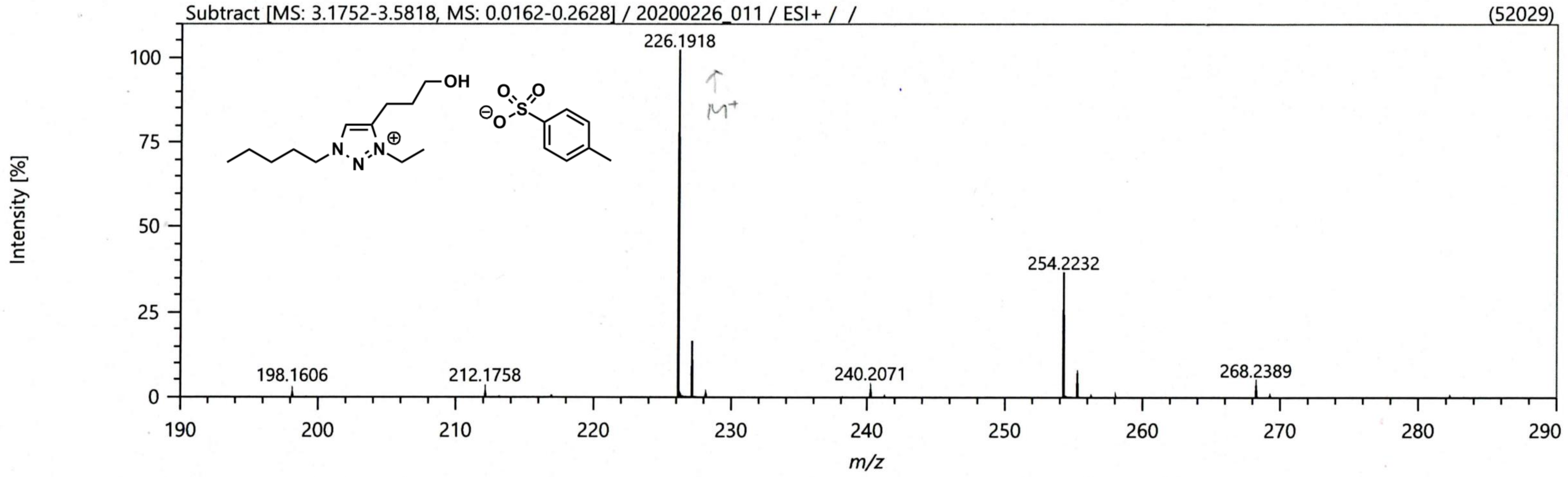

Elemental Composition

Parameters

Tolerance:	±10.00 ppm
Electron:	Odd/Even
Charge:	+1
DBE:	-99.0 - 999.0

Elements Set 1:

Symbol	C	H	O	Na	N	S	Ni	F
Min	0	0	1	0	3	0	0	0
Max	1000	4000	1	0	3	0	0	0

Results

Mass	Formula	Calculated Mass	Mass Difference [mDa]	Mass Difference [ppm]	DBE
226.19182	C12 H24 N3 O	226.19139	0.43	1.89	2.5

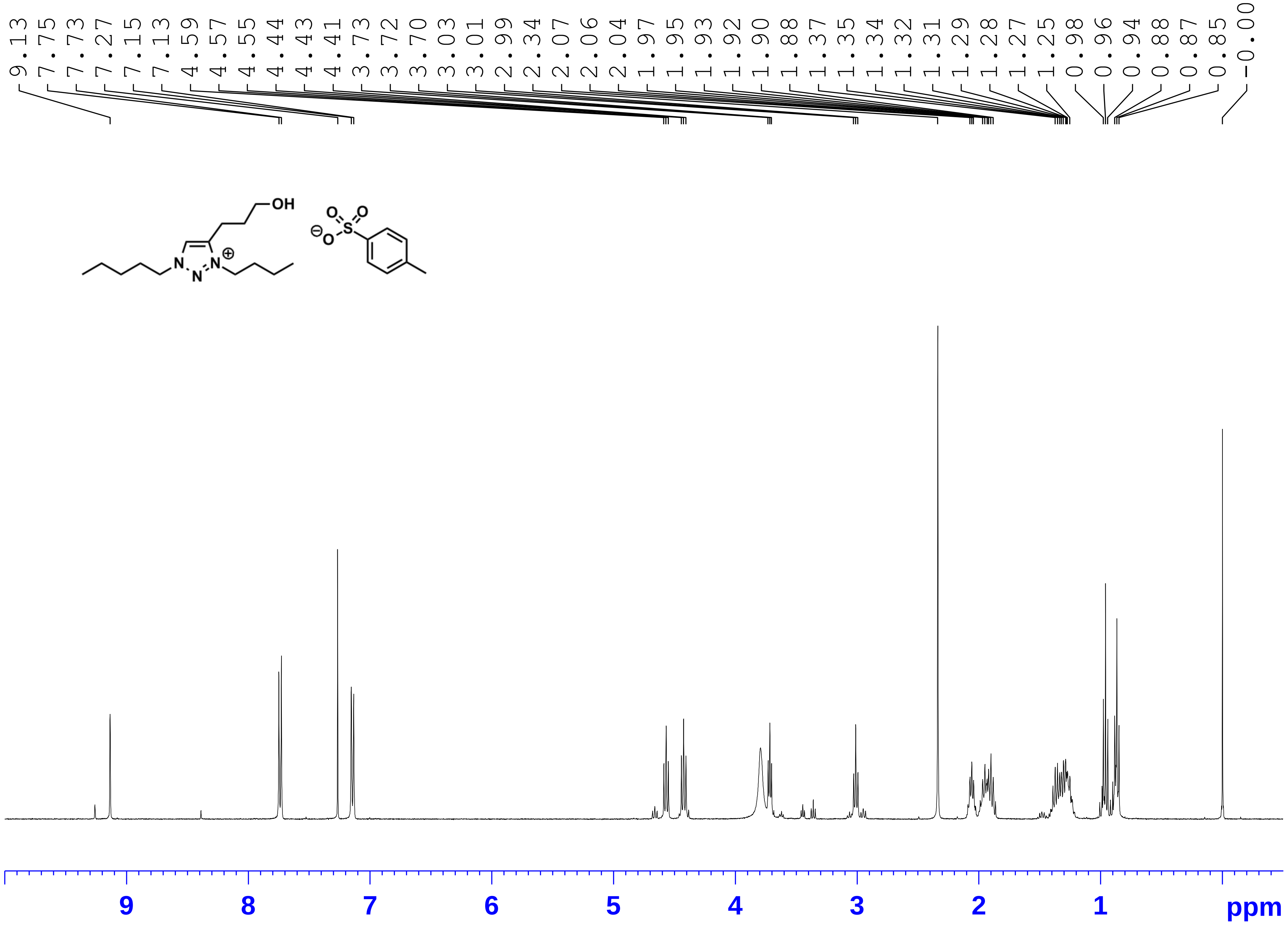
9.13
7.75
7.73
7.27
7.15
7.13
4.59
4.57
4.55
4.44
4.43
4.41
3.73
3.72
3.70
3.03
3.01
2.99
2.34
2.07
2.06
2.04
1.97
1.95
1.93
1.92
1.90
1.88
1.37
1.35
1.34
1.32
1.31
1.29
1.28
1.27
1.25
0.98
0.96
0.94
0.88
0.87
0.85
-0.00
OH
N
N
N
O
O
S
O
9
8
7
6
5
4
3
2
1
ppm

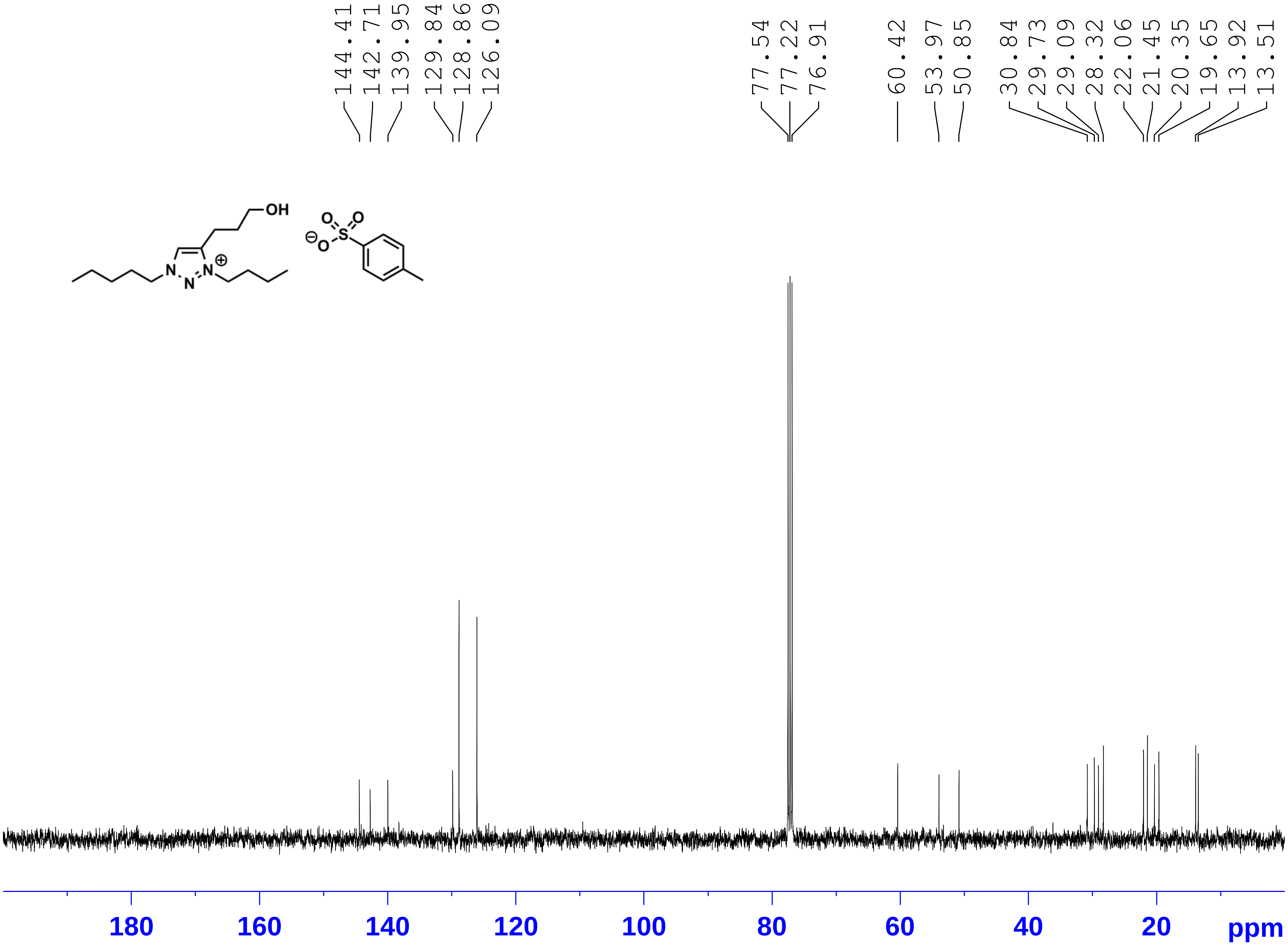
144.41
142.71
139.95
129.84
128.86
126.09
77.54
77.22
76.91
60.42
53.97
50.85
30.84
29.73
29.09
28.32
22.06
21.45
20.35
19.65
13.92
13.51
OH
O
S
O
O
N
N
N
180
160
140
120
100
80
60
40
20
ppm

Spectrum

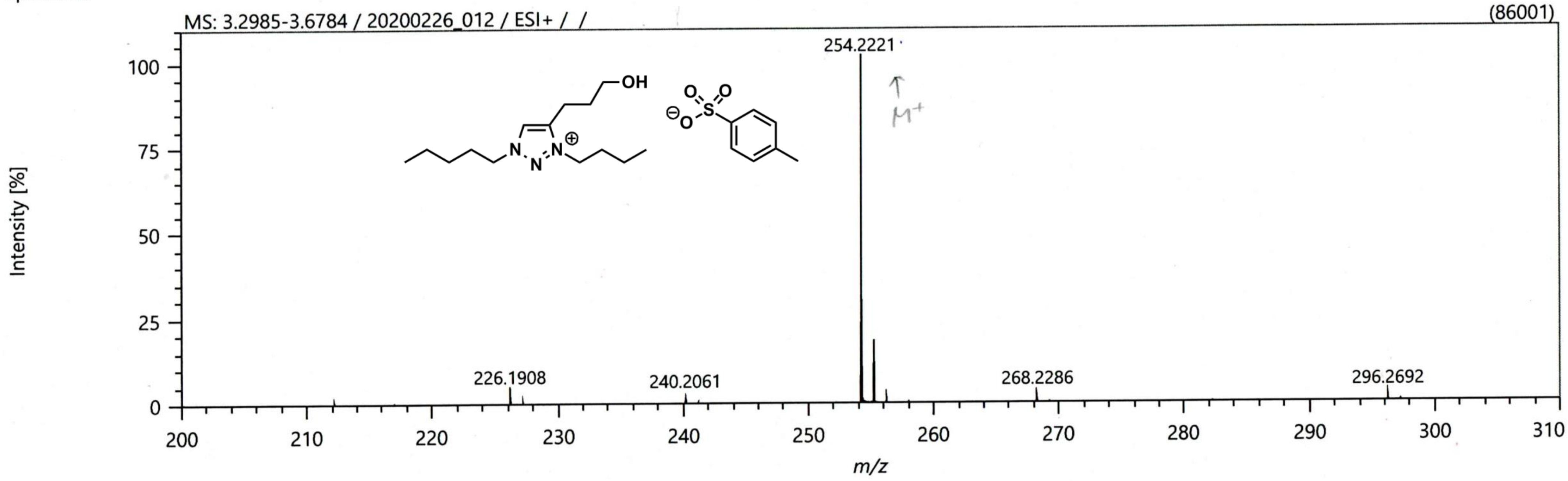

Elemental Composition

Parameters

Tolerance:	±10.00 ppm
Electron:	Odd/Even
Charge:	+1
DBE:	-99.0 - 999.0

Elements Set 1:

Symbol	C	H	O	Na	N	S	Ni	F
Min	0	0	1	0	3	0	0	0
Max	1000	4000	1	0	3	0	0	0

Results

Mass	Formula	Calculated Mass	Mass Difference [mDa]	Mass Difference [ppm]	DBE
254.22212	C14 H28 N3 O	254.22269	-0.57	-2.24	2.5

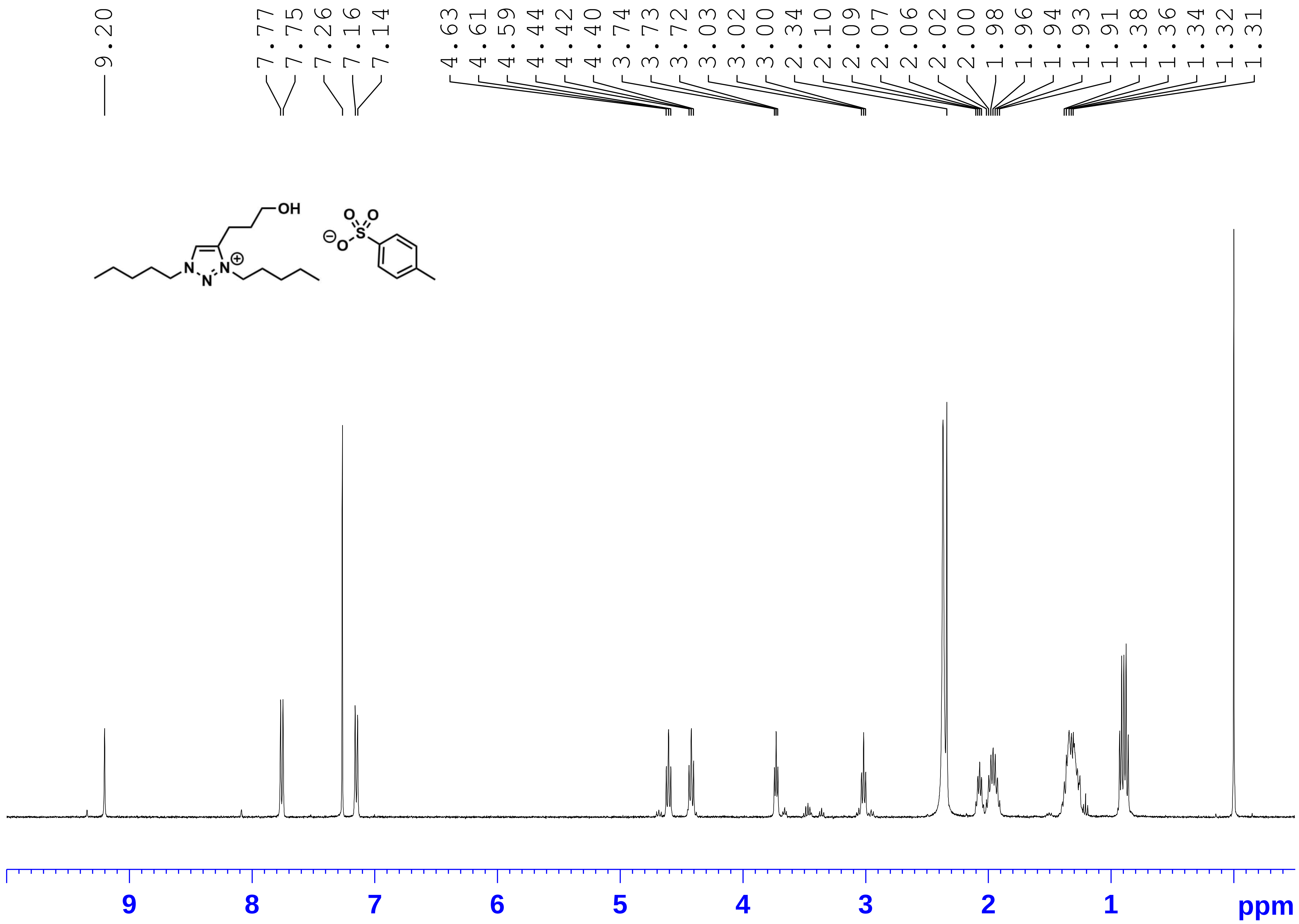

9.20
7.77
7.75
7.26
7.16
7.14
4.63
4.61
4.59
4.44
4.42
4.40
3.74
3.73
3.72
3.03
3.02
3.00
2.34
2.10
2.09
2.07
2.06
2.02
2.00
1.98
1.96
1.94
1.93
1.91
1.38
1.36
1.34
1.32
1.31
OH
N
N
N
O
O
S
O
9
8
7
6
5
4
3
2
1
ppm

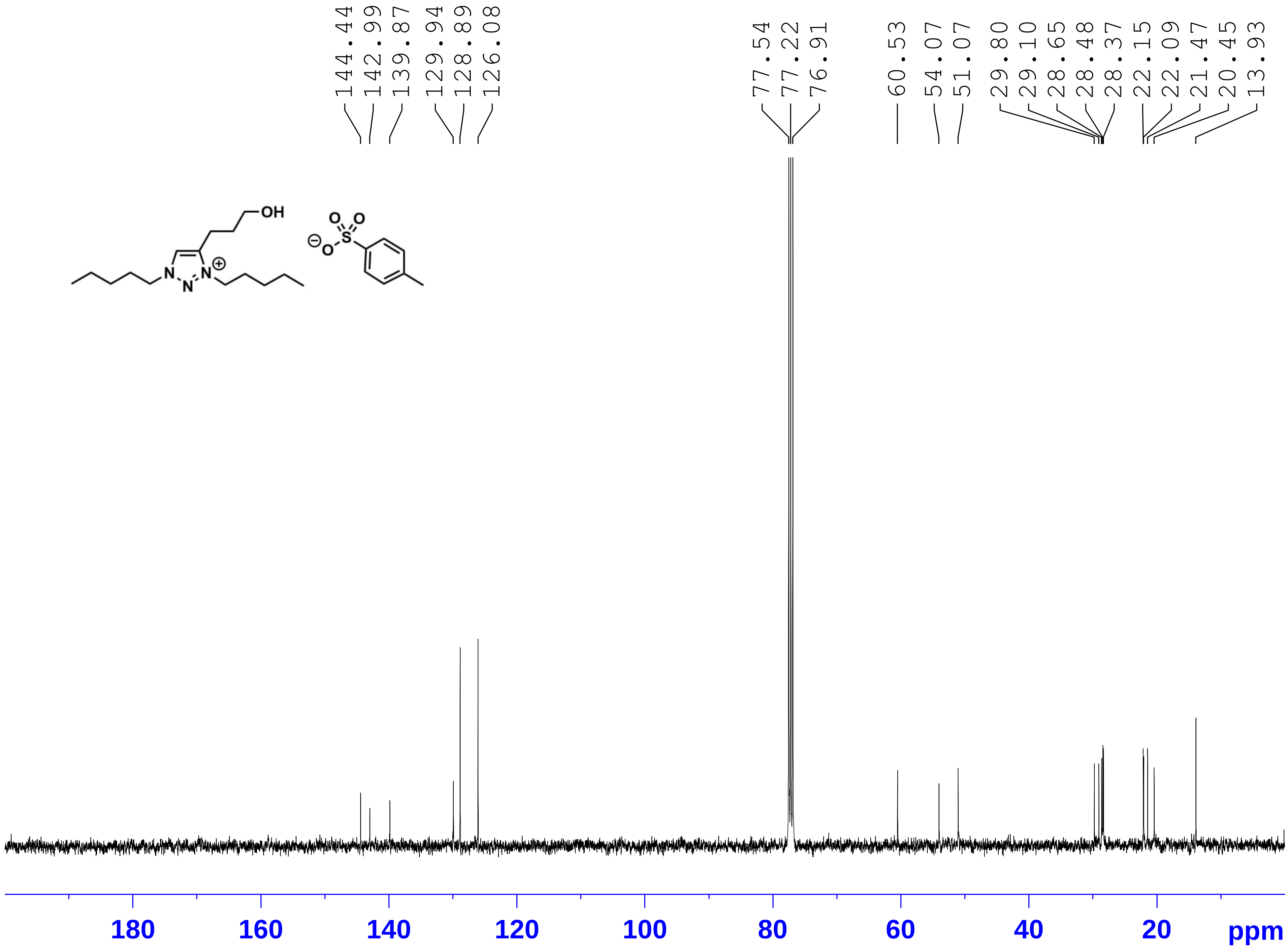
144.44
142.99
139.87
129.94
128.89
126.08
77.54
77.22
76.91
60.53
54.07
51.07
29.80
29.10
28.65
28.48
28.37
22.15
22.09
21.47
20.45
13.93
OH
O
O
S
O
N
N
N
180
160
140
120
100
80
60
40
20
ppm

Spectrum

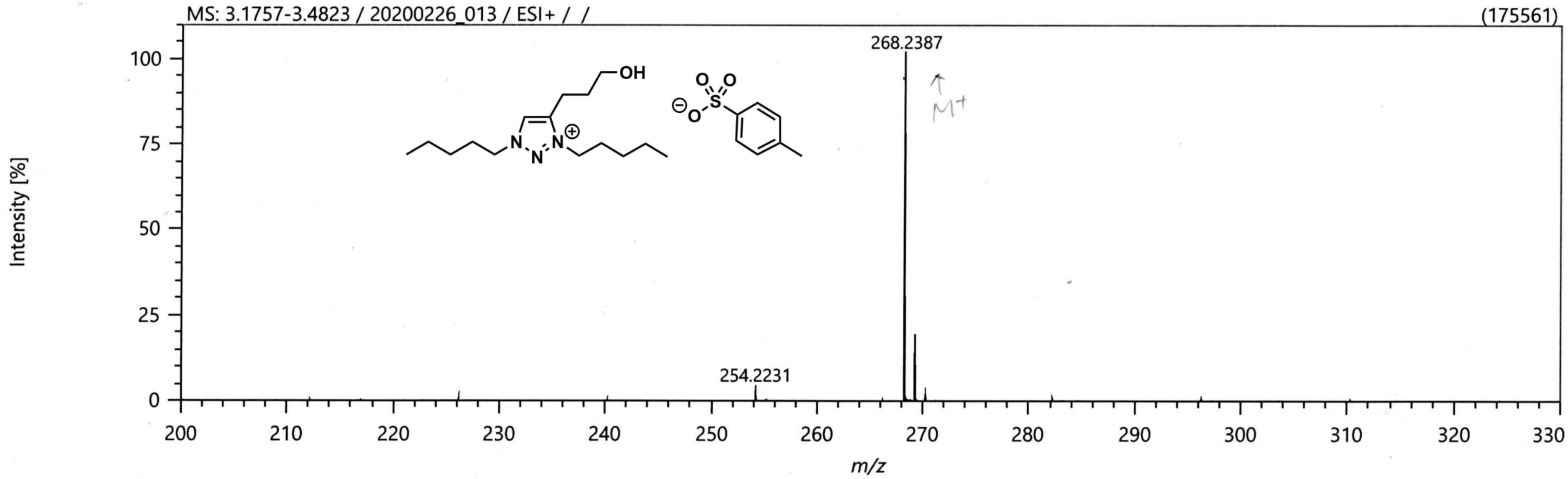

Elemental Composition

Parameters

Tolerance:	±10.00 ppm
Electron:	Odd/Even
Charge:	+1
DBE:	-99.0 - 999.0

Elements Set 1:

Symbol	C	H	O	Na	N	S	Ni	F
Min	0	0	1	0	3	0	0	0
Max	1000	4000	1	0	3	0	0	0

Results

Mass	Formula	Calculated Mass	Mass Difference [mDa]	Mass Difference [ppm]	DBE
268.23871	C15 H30 N3 O	268.23834	0.37	1.38	2.5

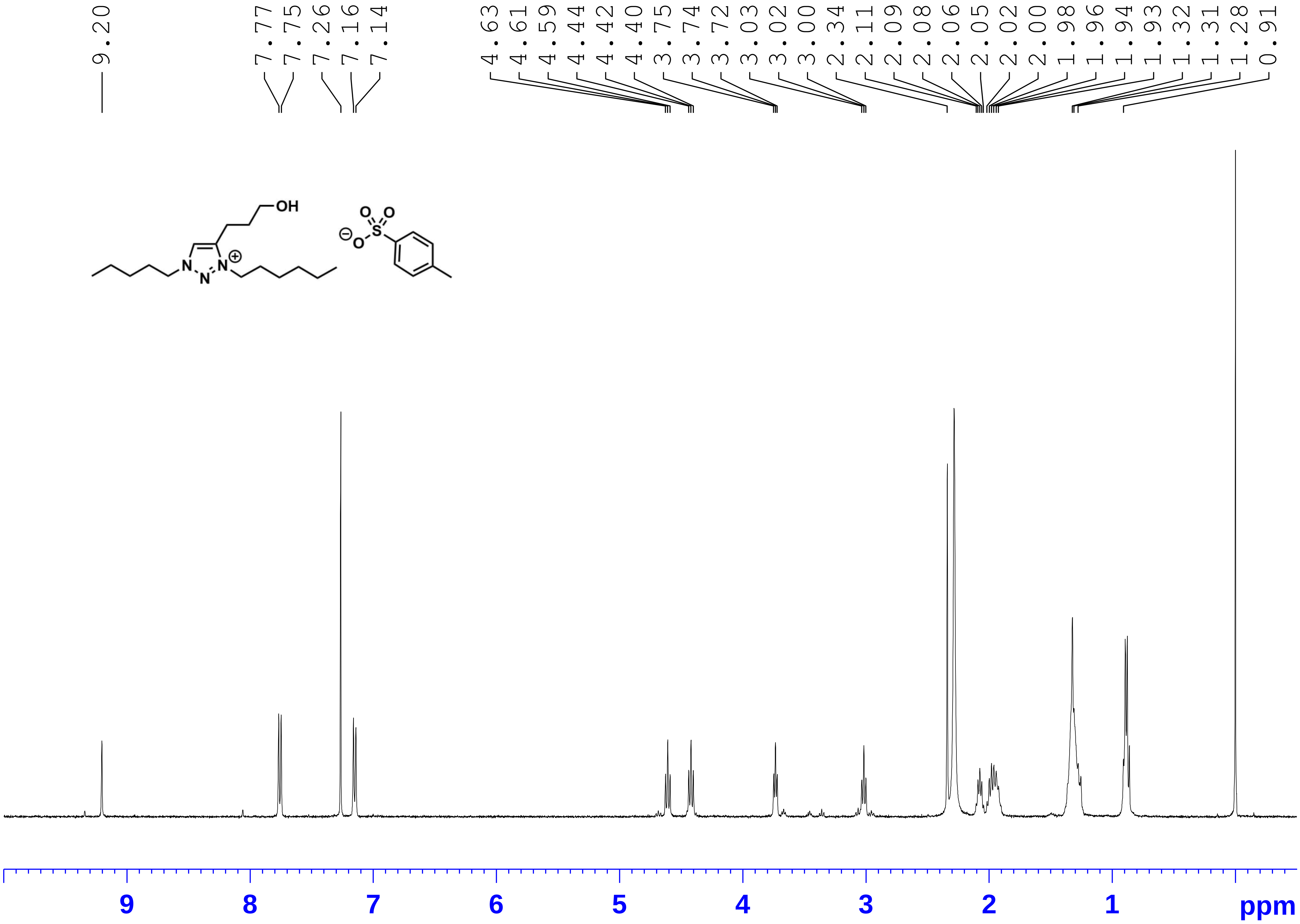
9.20
7.77
7.75
7.26
7.16
7.14
4.63
4.61
4.59
4.44
4.42
4.40
3.75
3.74
3.72
3.03
3.02
3.00
2.34
2.11
2.09
2.08
2.06
2.05
2.02
2.00
1.98
1.96
1.94
1.93
1.32
1.31
1.28
0.91
OH
O
O
S
O
N
N
N
9
8
7
6
5
4
3
2
1
ppm

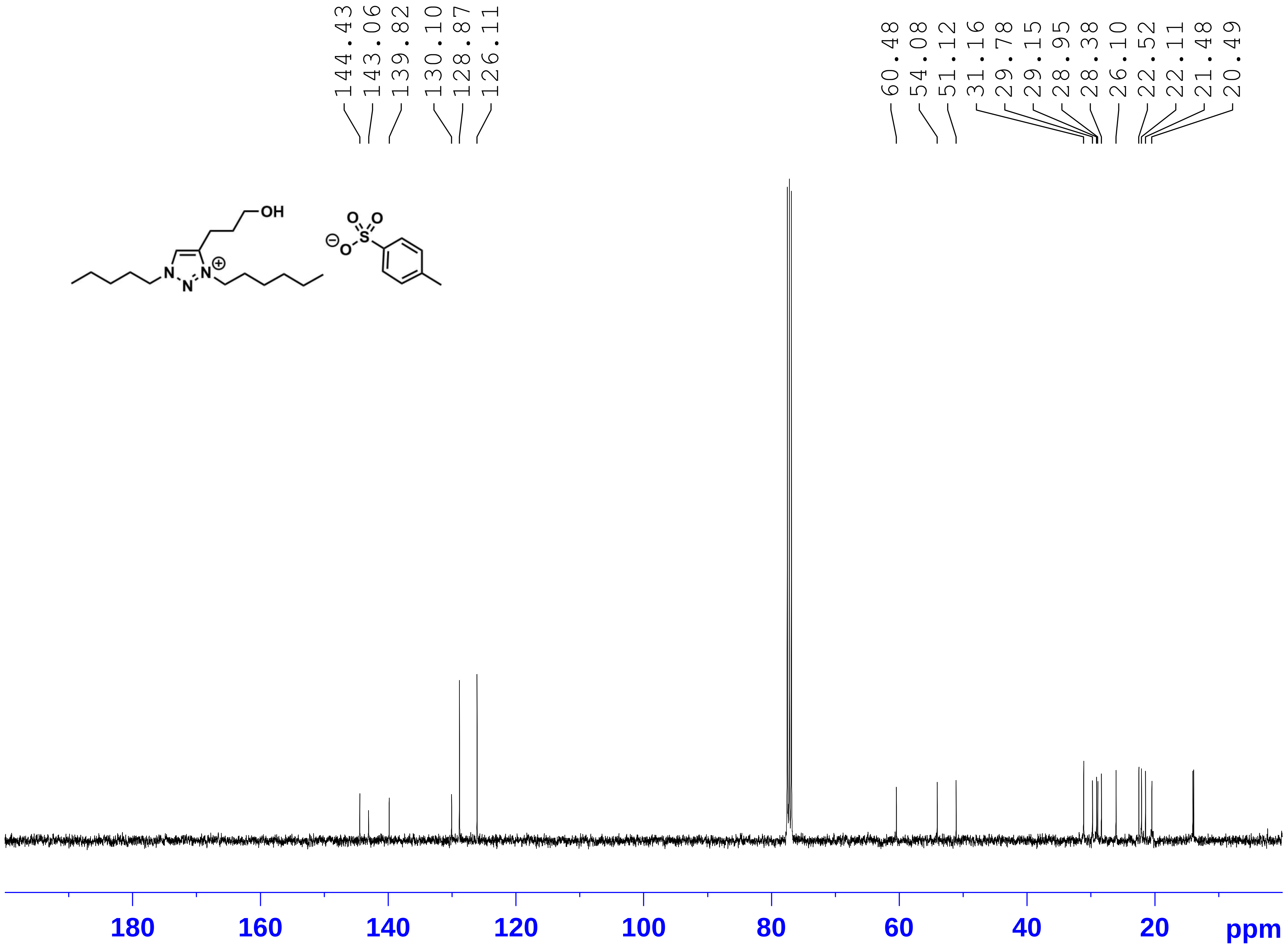

144.43
143.06
139.82
130.10
128.87
126.11
60.48
54.08
51.12
31.16
29.78
29.15
28.95
28.38
26.10
22.52
22.11
21.48
20.49
OH
N
N
N
O
O
S
O
180
160
140
120
100
80
60
40
20
ppm

Spectrum

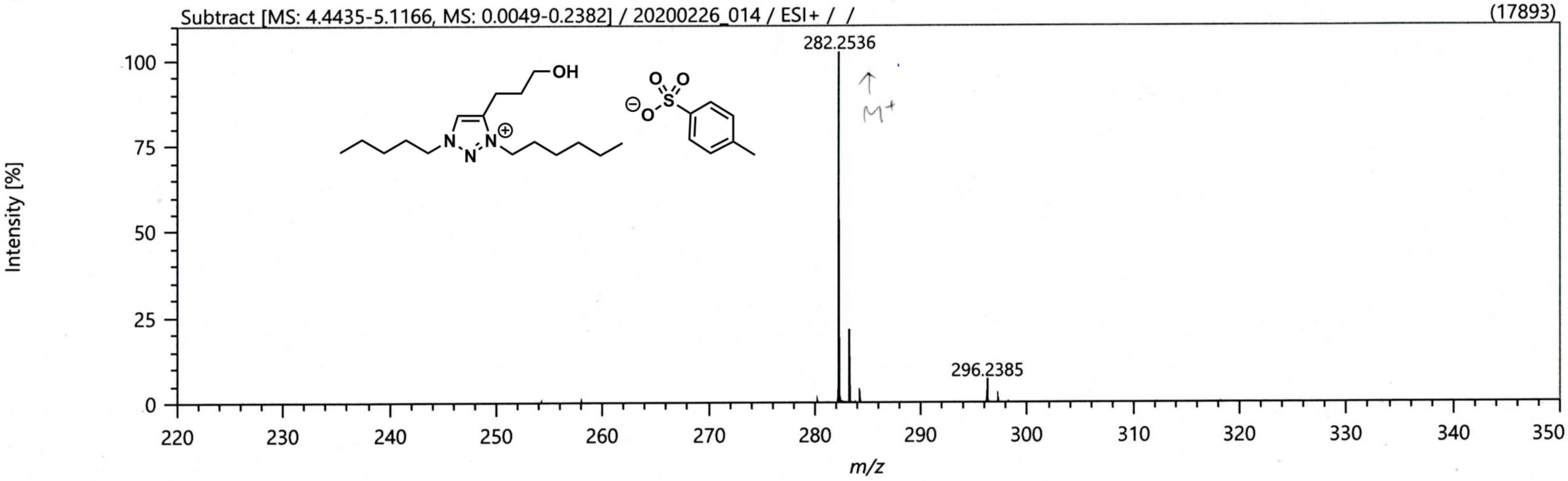

Elemental Composition

Parameters		Elements Set 1:								
Tolerance:	±10.00 ppm	Symbol	C	H	O	Na	N	S	Ni	F
Electron:	Odd/Even	Min	0	0	1	0	3	0	0	0
Charge:	+1	Max	1000	4000	1	0	3	0	0	0
DBE:	-99.0 - 999.0									

Results

Mass	Formula	Calculated Mass	Mass Difference [mDa]	Mass Difference [ppm]	DBE
282.25356	C16 H32 N3 O	282.25399	-0.43	-1.51	2.5

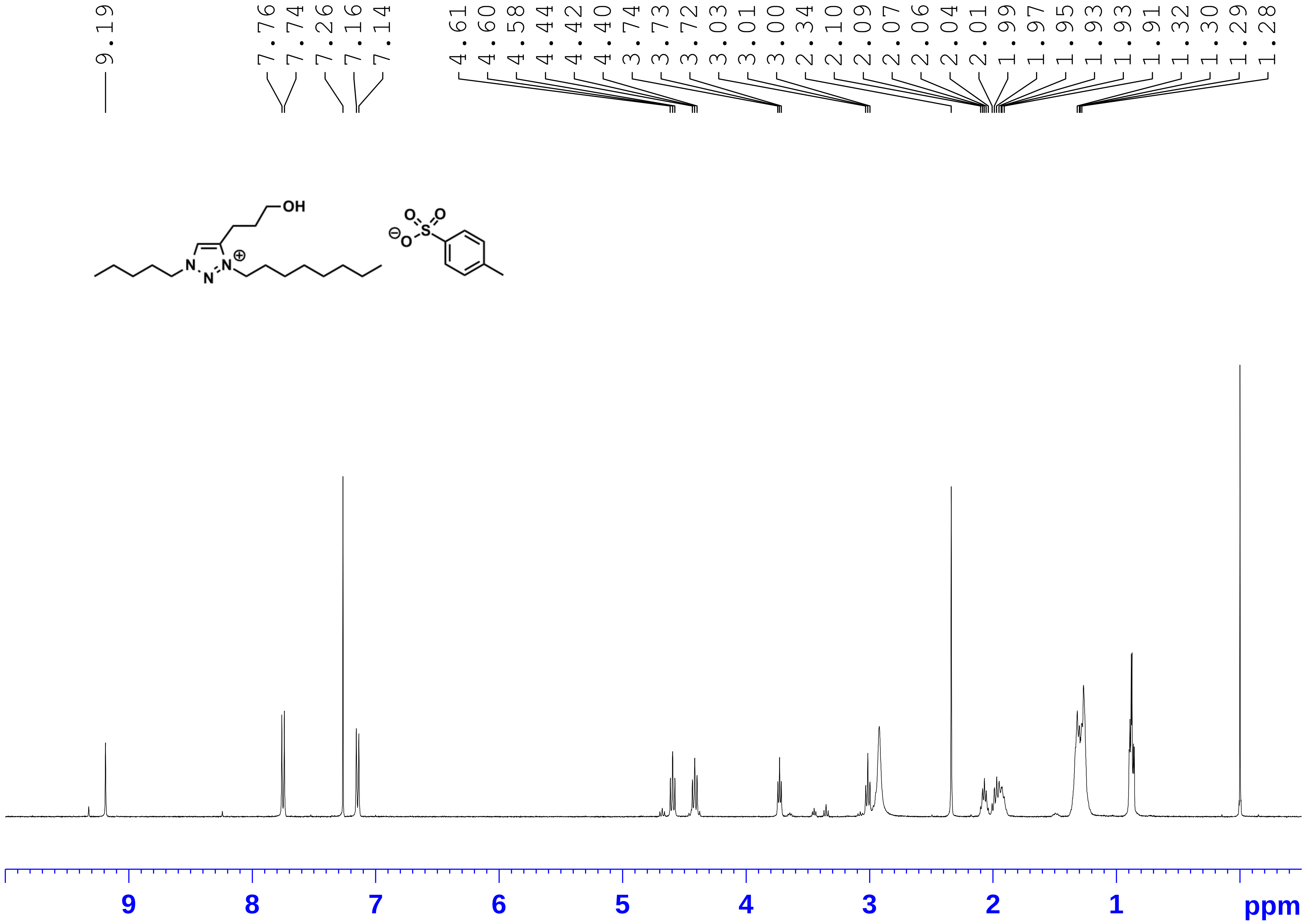

9.19
7.76
7.74
7.26
7.16
7.14
4.61
4.60
4.58
4.44
4.42
4.40
3.74
3.73
3.72
3.03
3.01
3.00
2.34
2.10
2.09
2.07
2.06
2.04
2.01
1.99
1.97
1.95
1.93
1.93
1.91
1.32
1.30
1.29
1.28
9
8
7
6
5
4
3
2
1
ppm

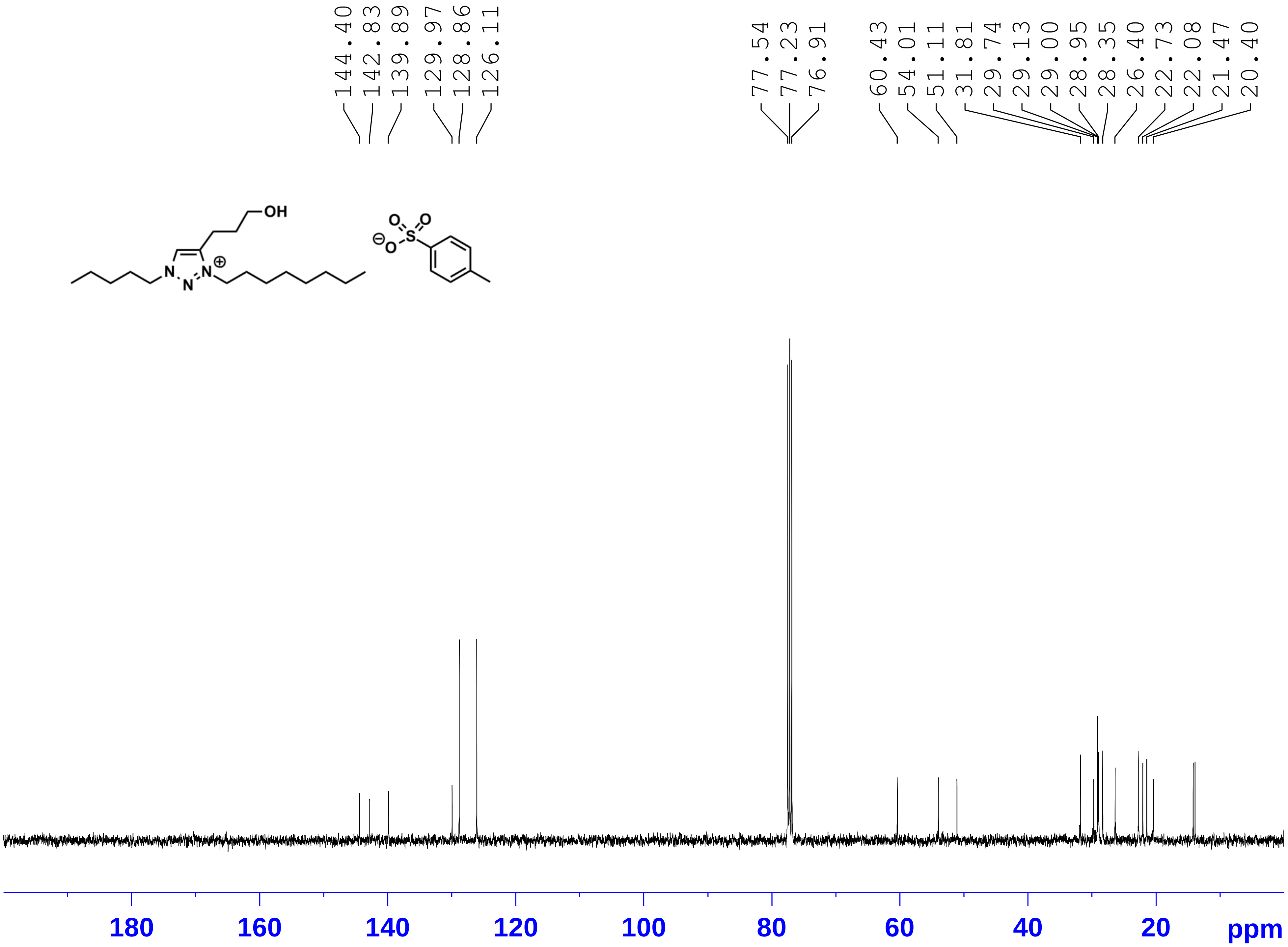

144.40
142.83
139.89
129.97
128.86
126.11
77.54
77.23
76.91
60.43
54.01
51.11
31.81
29.74
29.13
29.00
28.95
28.35
26.40
22.73
22.08
21.47
20.40
OH
N
N
N
O
O
S
O
180
160
140
120
100
80
60
40
20
ppm

Spectrum

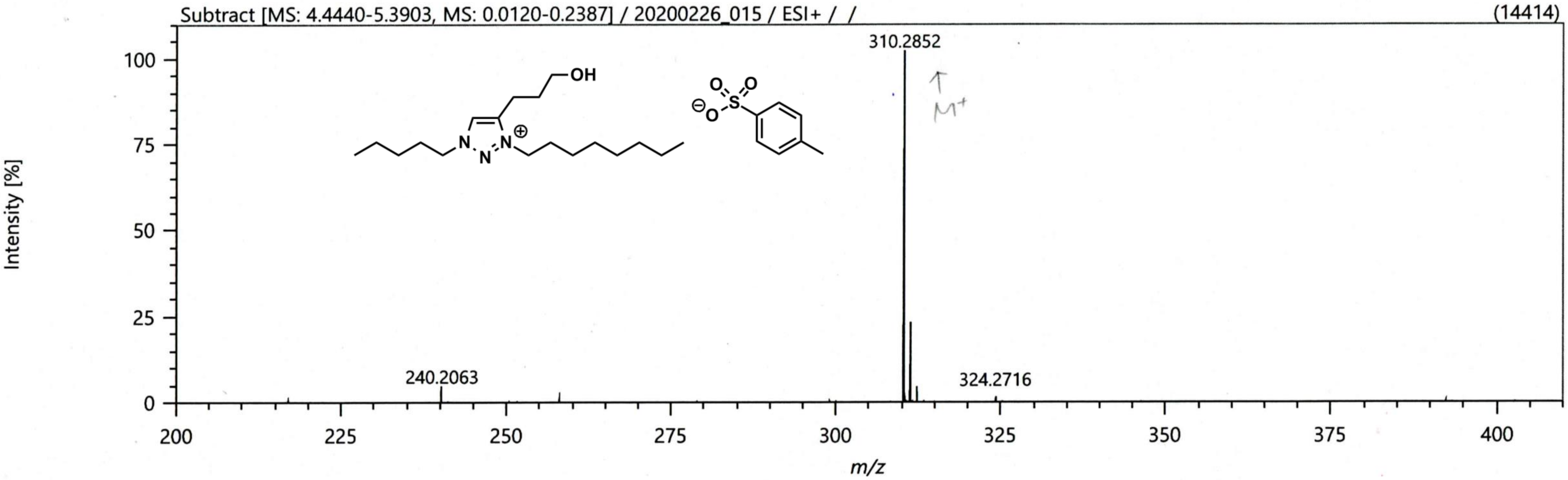

Elemental Composition

Parameters		Elements Set 1:								
Tolerance:	±10.00 ppm	Symbol	C	H	O	Na	N	S	Ni	F
Electron:	Odd/Even	Min	0	0	1	0	3	0	0	0
Charge:	+1	Max	1000	4000	1	0	3	0	0	0
DBE:	-99.0 - 999.0									

Results

Mass	Formula	Calculated Mass	Mass Difference [mDa]	Mass Difference [ppm]	DBE
310.28519	C18 H36 N3 O	310.28529	-0.10	-0.32	2.5

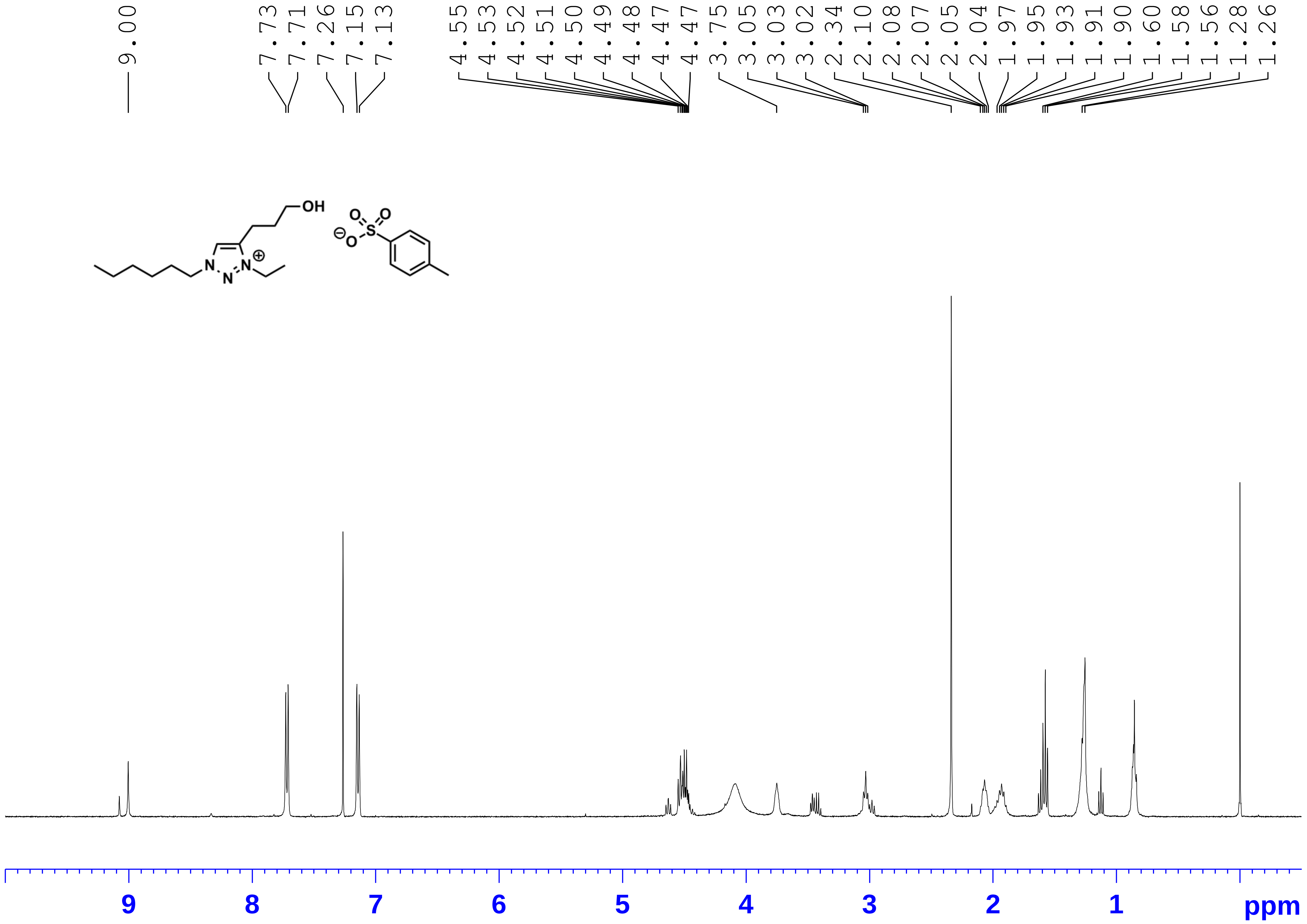
9.00
7.73
7.71
7.26
7.15
7.13
4.55
4.53
4.52
4.51
4.50
4.49
4.48
4.47
4.47
3.75
3.05
3.03
3.02
2.34
2.10
2.08
2.07
2.05
2.04
1.97
1.95
1.93
1.91
1.90
1.60
1.58
1.56
1.28
1.26
OH
O
S
O
O
N
N
N
9
8
7
6
5
4
3
2
1
ppm

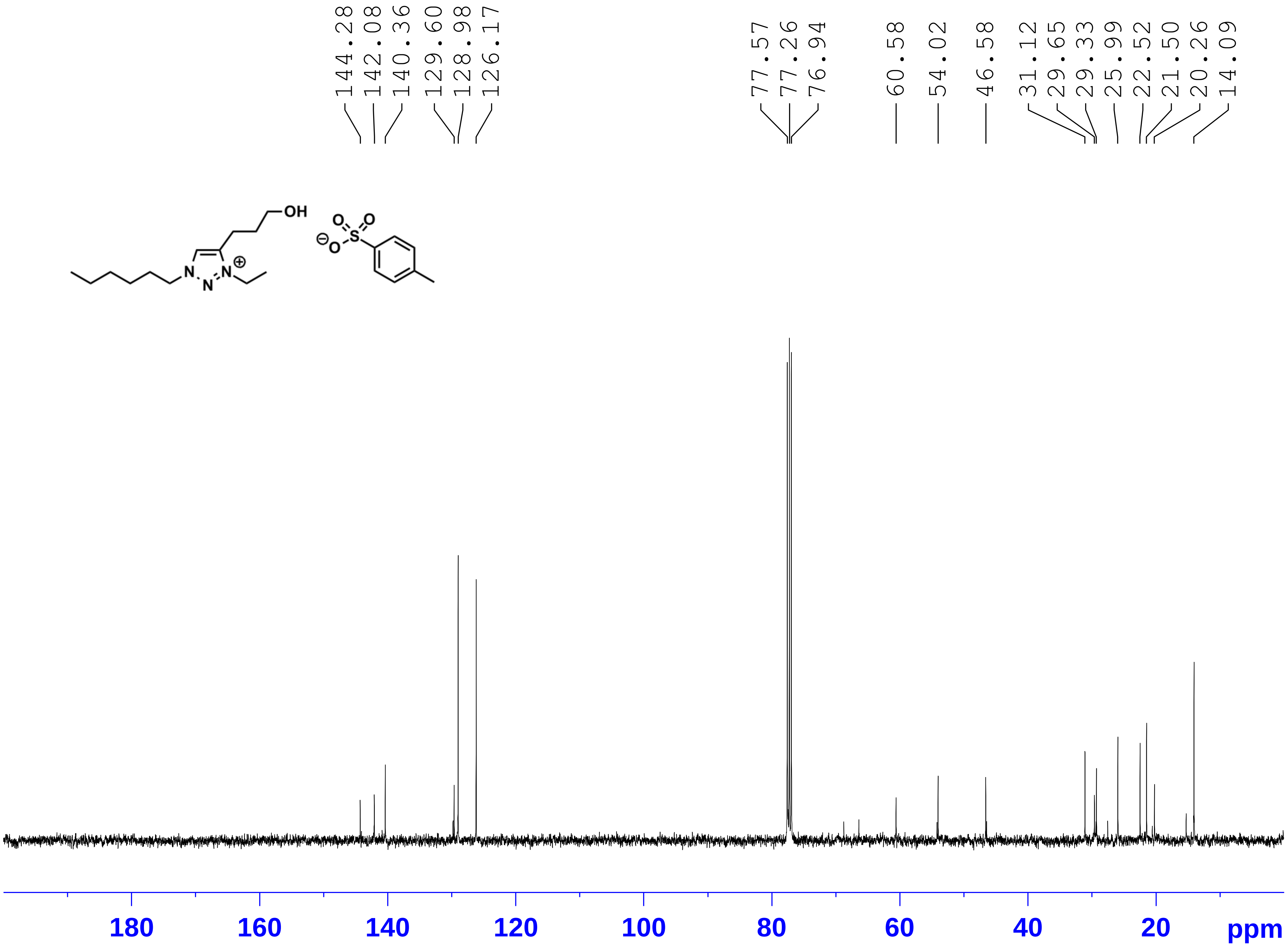
144.28
142.08
140.36
129.60
128.98
126.17
77.57
77.26
76.94
60.58
54.02
46.58
31.12
29.65
29.33
25.99
22.52
21.50
20.26
14.09
OH
O
S
O
O
N
N
N
180
160
140
120
100
80
60
40
20
ppm

Spectrum

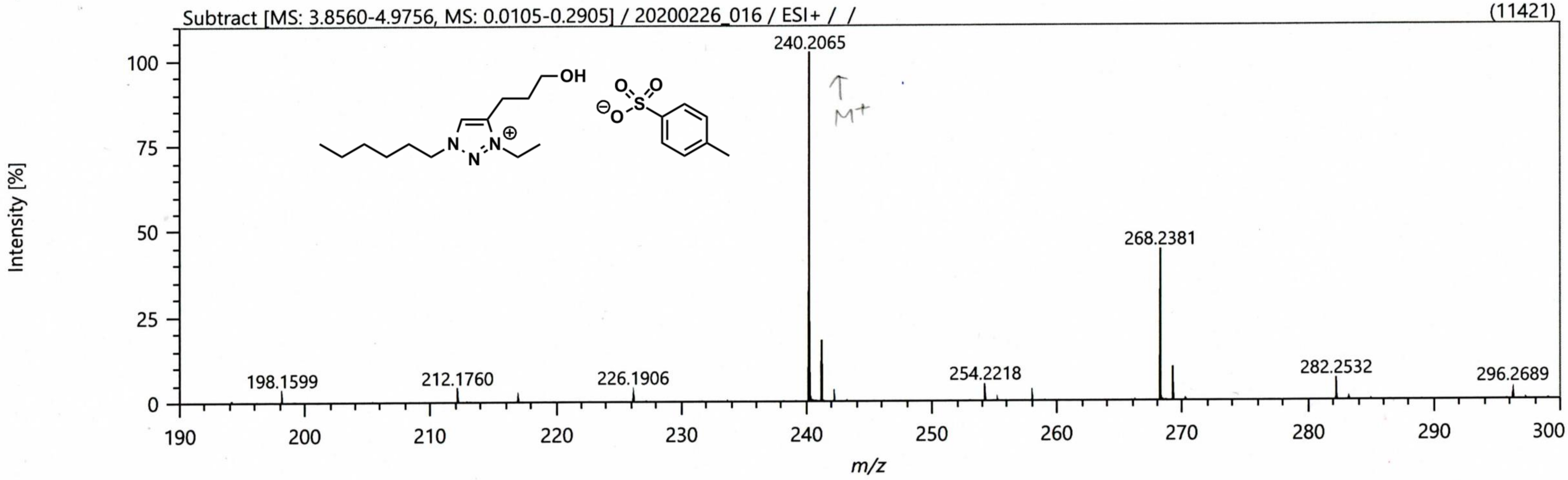

Elemental Composition

Parameters		Elements Set 1:								
Tolerance:	±10.00 ppm	Symbol	C	H	O	Na	N	S	Ni	F
Electron:	Odd/Even	Min	0	0	1	0	3	0	0	0
Charge:	+1	Max	1000	4000	1	0	3	0	0	0
DBE:	-99.0 - 999.0									

Results

Mass	Formula	Calculated Mass	Mass Difference [mDa]	Mass Difference [ppm]	DBE
240.20648	C13 H26 N3 O	240.20704	-0.56	-2.34	2.5

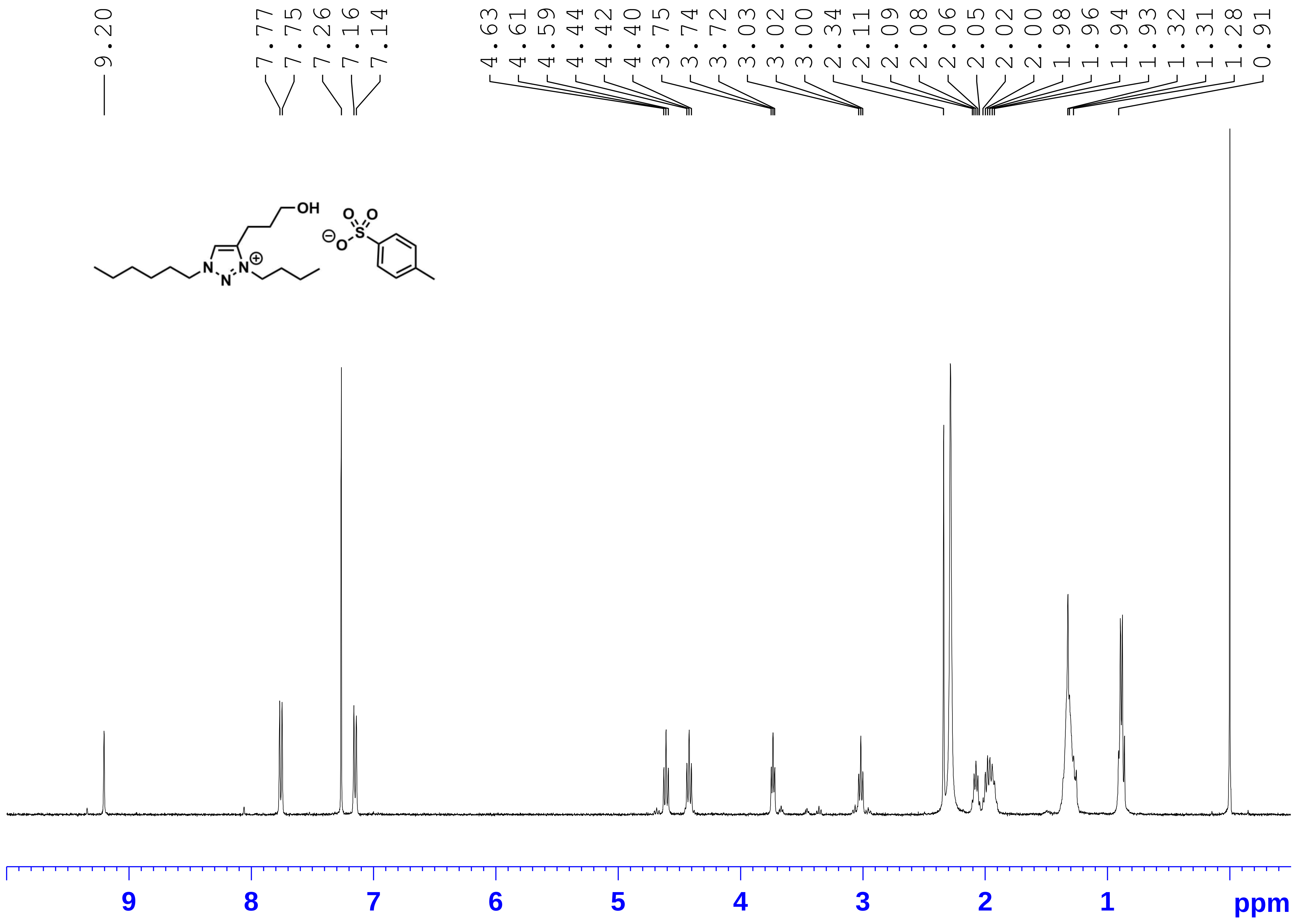

9.20
7.77
7.75
7.26
7.16
7.14
4.63
4.61
4.59
4.44
4.42
4.40
3.75
3.74
3.72
3.03
3.02
3.00
2.34
2.11
2.09
2.08
2.06
2.05
2.02
2.00
1.98
1.96
1.94
1.93
1.32
1.31
1.28
0.91
OH
O
O
S
O
N
N
N
9
8
7
6
5
4
3
2
1
ppm

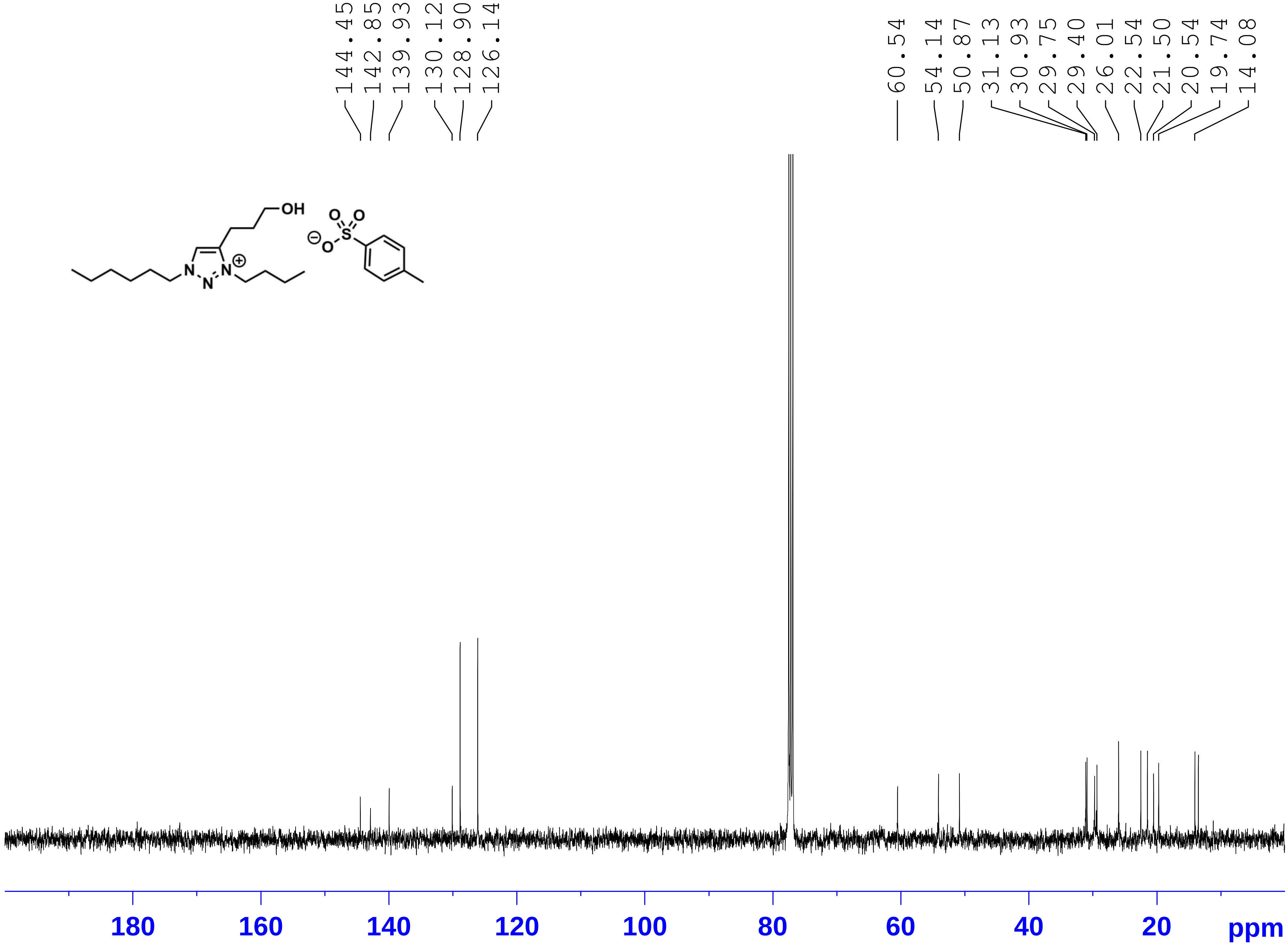
144.45
142.85
139.93
130.12
128.90
126.14
60.54
54.14
50.87
31.13
30.93
29.75
29.40
26.01
22.54
21.50
20.54
19.74
14.08
OH
N
N
N
O
O
S
O
180
160
140
120
100
80
60
40
20
ppm

Spectrum

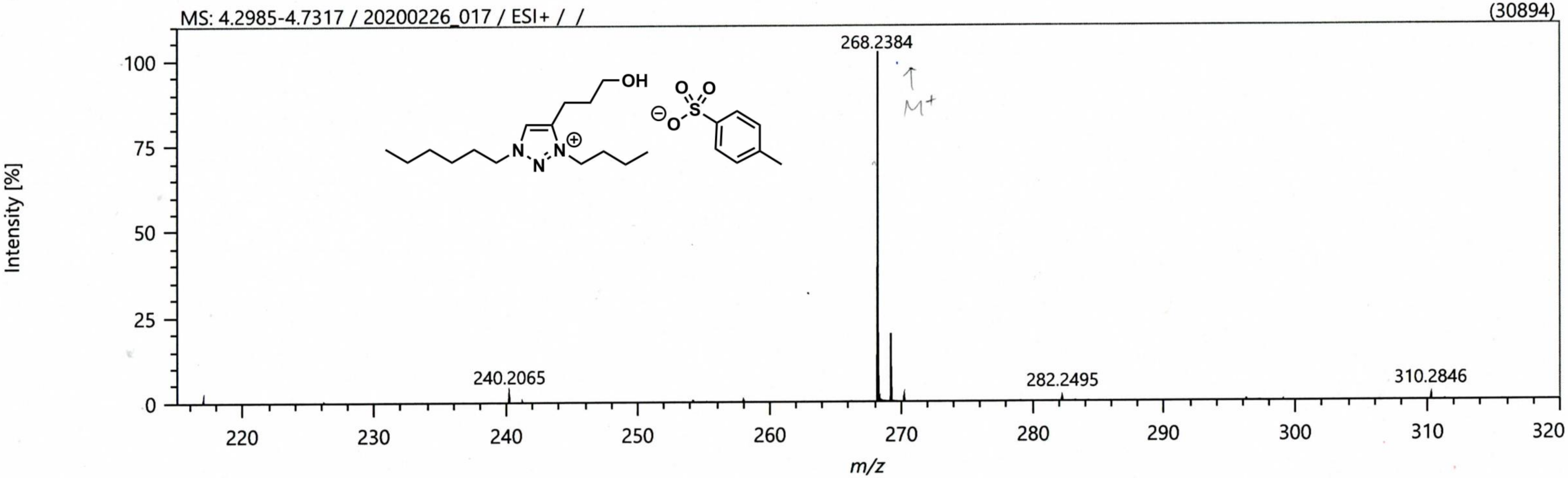

Elemental Composition

Parameters		Elements Set 1:								
Tolerance:	±10.00 ppm	Symbol	C	H	O	Na	N	S	Ni	F
Electron:	Odd/Even	Min	0	0	1	0	3	0	0	0
Charge:	+1	Max	1000	4000	1	0	3	0	0	0
DBE:	-99.0 - 999.0									

Results

Mass	Formula	Calculated Mass	Mass Difference [mDa]	Mass Difference [ppm]	DBE
268.23842	C15 H30 N3 O	268.23834	0.08	0.32	2.5

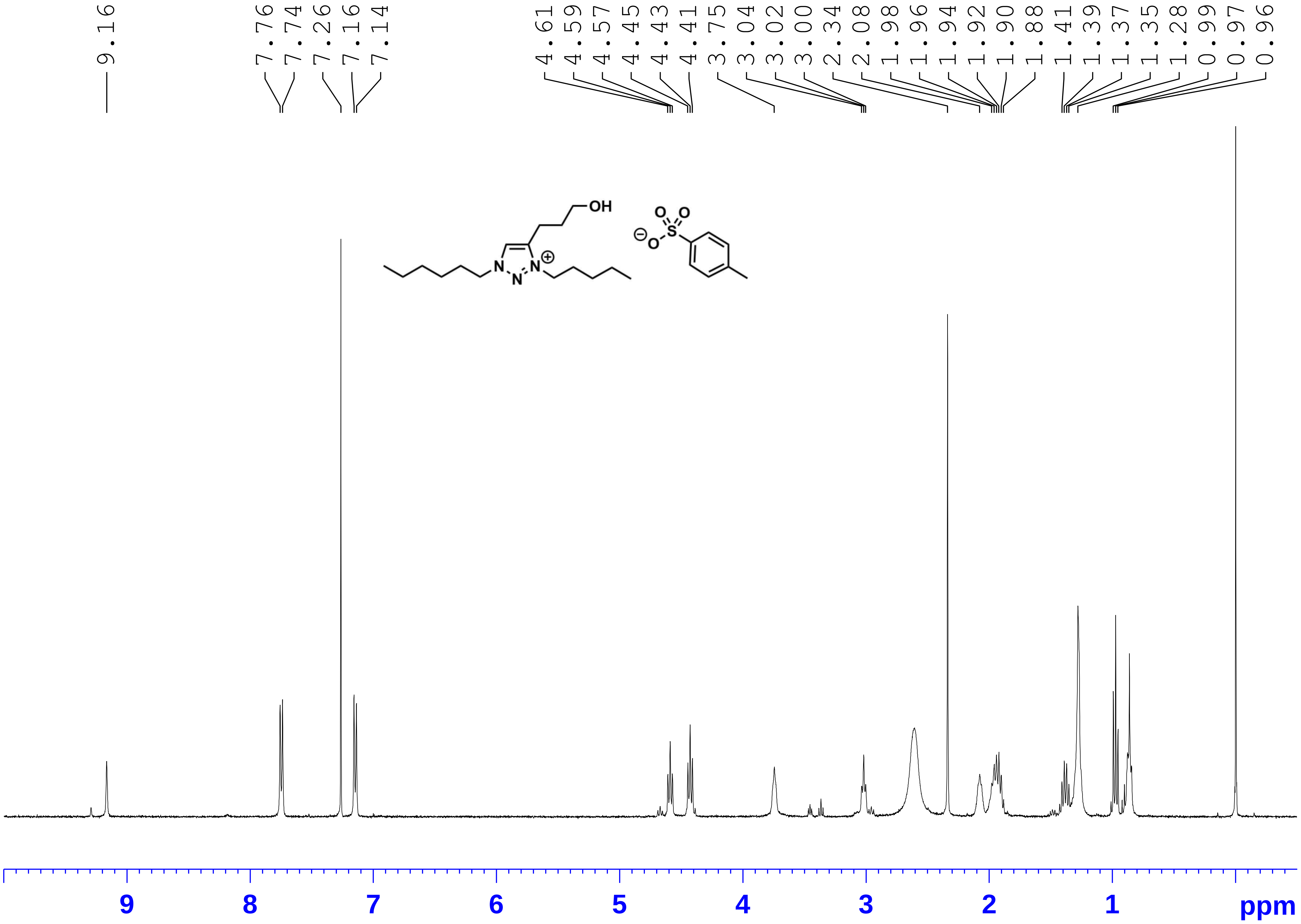
9.16
7.76
7.74
7.26
7.16
7.14
4.61
4.59
4.57
4.45
4.43
4.41
3.75
3.04
3.02
3.00
2.34
2.08
1.98
1.96
1.94
1.92
1.90
1.88
1.41
1.39
1.37
1.35
1.28
0.99
0.97
0.96
OH
N
N
N
O
O
S
O
9
8
7
6
5
4
3
2
1
ppm

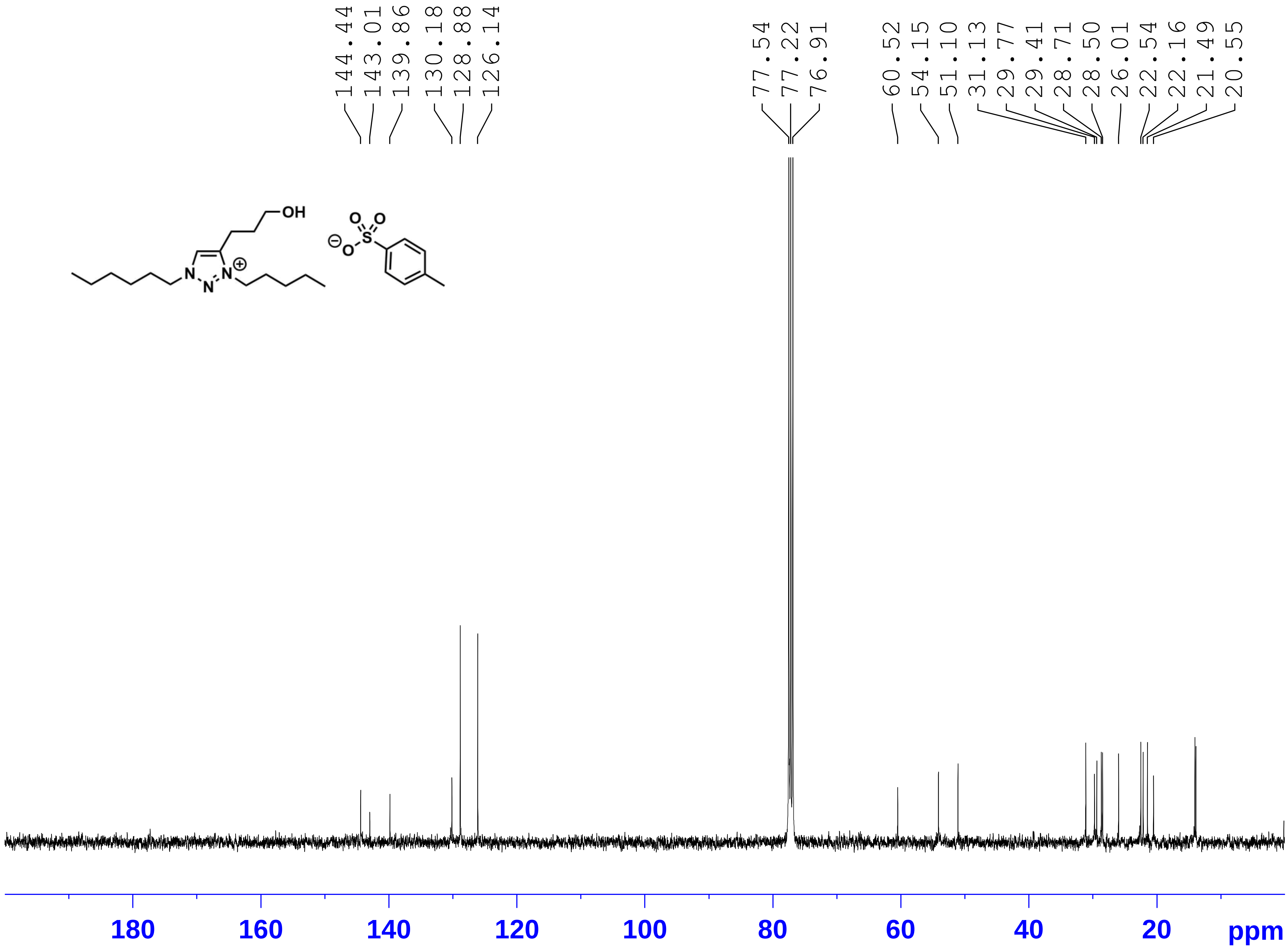
144.44
143.01
139.86
130.18
128.88
126.14
77.54
77.22
76.91
60.52
54.15
51.10
31.13
29.77
29.41
28.71
28.50
26.01
22.54
22.16
21.49
20.55
OH
O
O
S
O
N
N
N
180
160
140
120
100
80
60
40
20
ppm

Spectrum

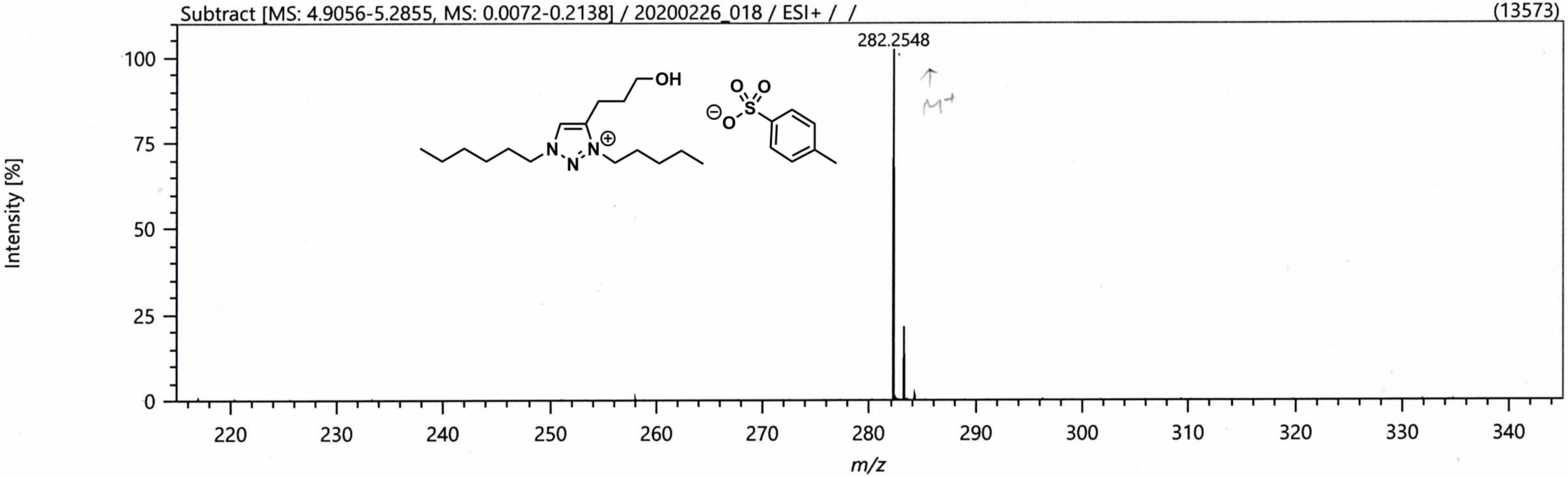

Elemental Composition

Parameters

Tolerance:	±10.00 ppm
Electron:	Odd/Even
Charge:	+1
DBE:	-99.0 - 999.0

Elements Set 1:

Symbol	C	H	O	Na	N	S	Ni	F
Min	0	0	1	0	3	0	0	0
Max	1000	4000	1	0	3	0	0	0

Results

Mass	Formula	Calculated Mass	Mass Difference [mDa]	Mass Difference [ppm]	DBE
282.25480	C16 H32 N3 O	282.25399	0.82	2.89	2.5

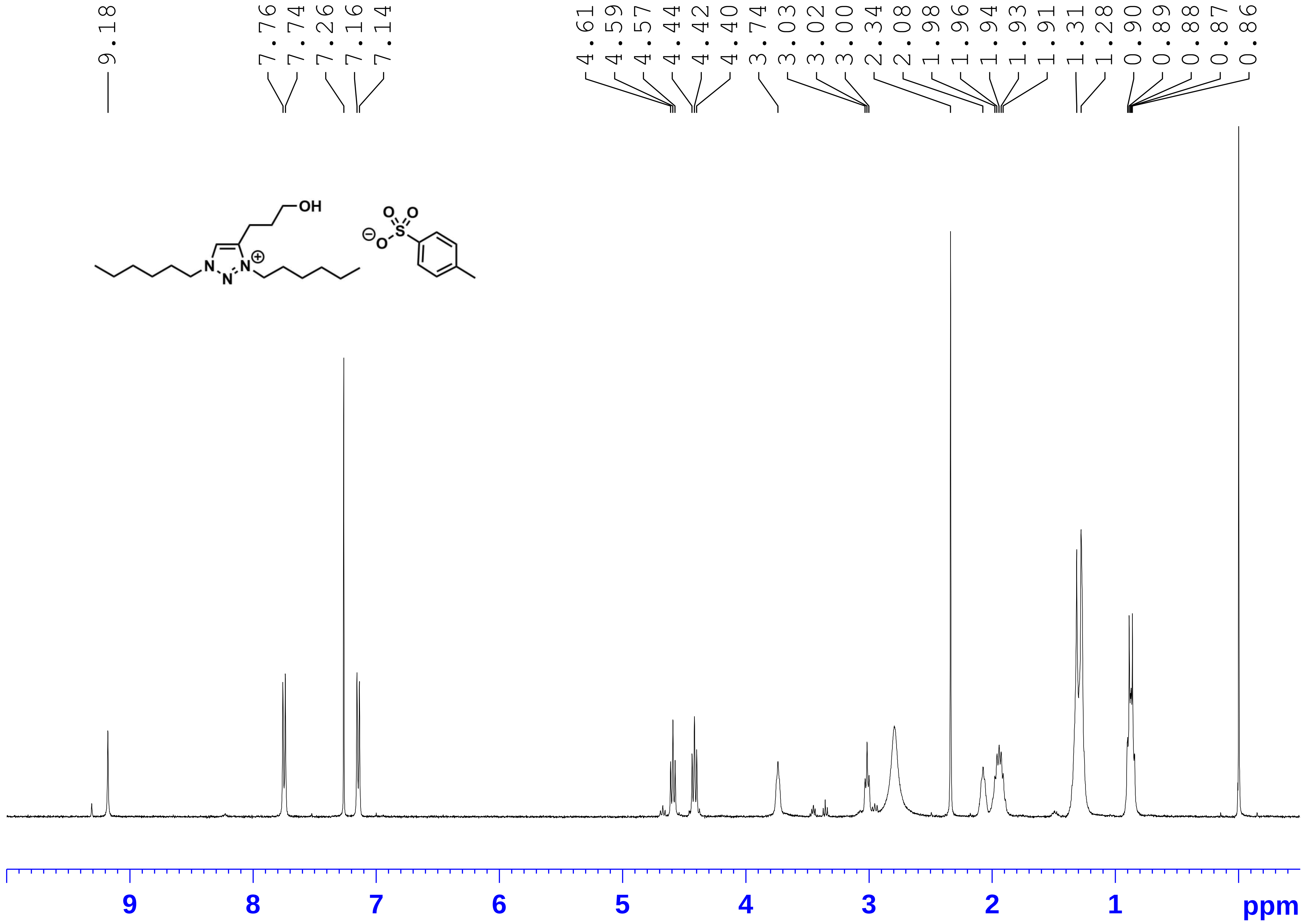
9.18
7.76
7.74
7.26
7.16
7.14
4.61
4.59
4.57
4.44
4.42
4.40
3.74
3.03
3.02
3.00
2.34
2.08
1.98
1.96
1.94
1.93
1.91
1.31
1.28
0.90
0.89
0.88
0.87
0.86
OH
N
N
N
O
O
O
S
9
8
7
6
5
4
3
2
1
ppm

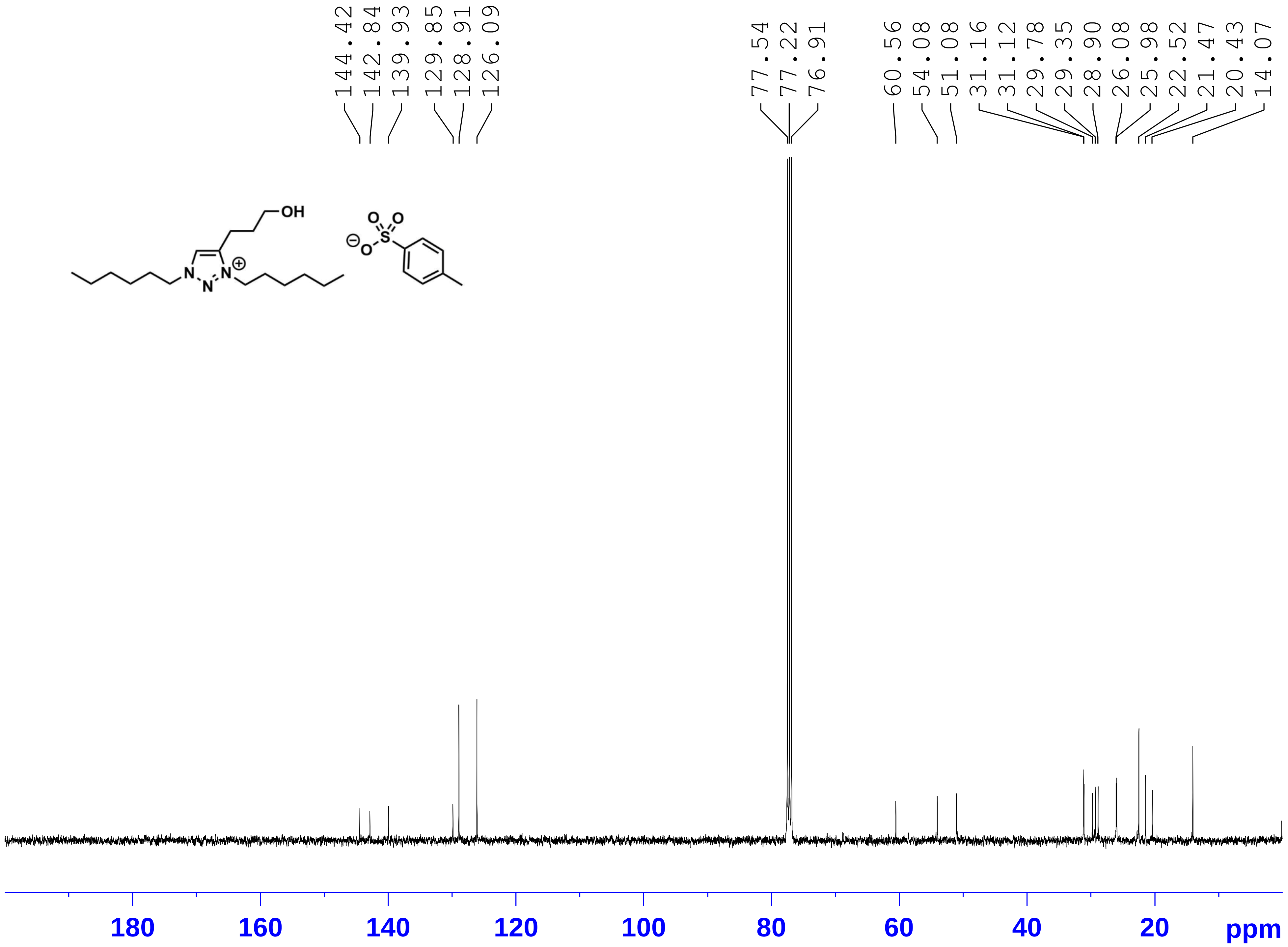

144.42
142.84
139.93
129.85
128.91
126.09
77.54
77.22
76.91
60.56
54.08
51.08
31.16
31.12
29.78
29.35
28.90
26.08
25.98
22.52
21.47
20.43
14.07
OH
N
N
N
O
O
S
O
180
160
140
120
100
80
60
40
20
ppm

Spectrum

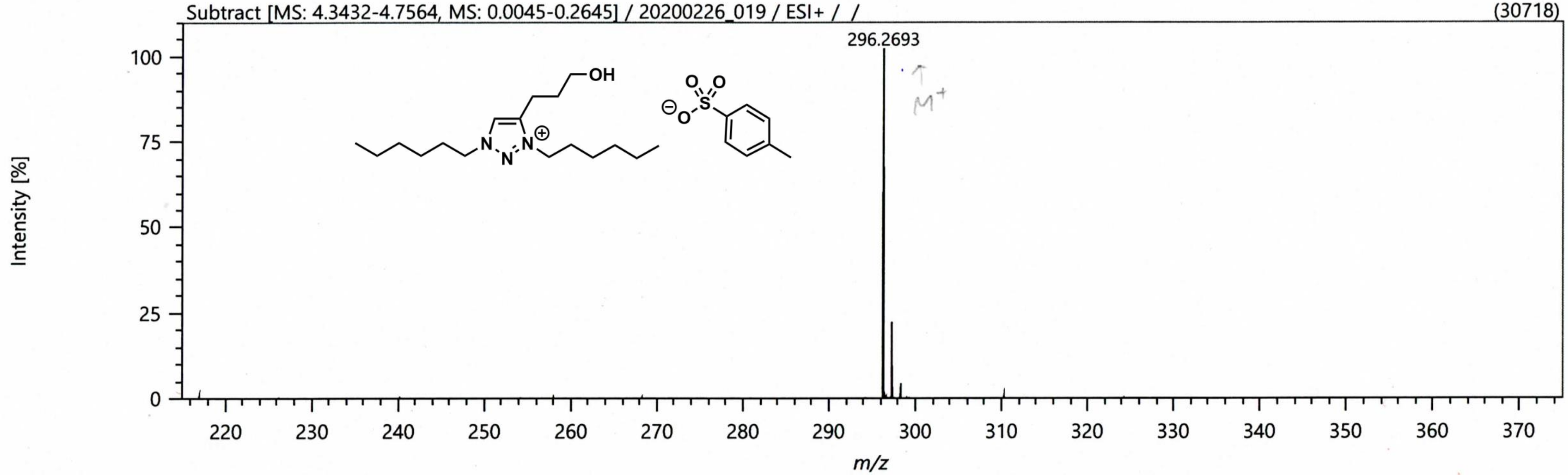

Elemental Composition

Parameters

Tolerance:	±10.00 ppm
Electron:	Odd/Even
Charge:	+1
DBE:	-99.0 - 999.0

Elements Set 1:

Symbol	C	H	O	Na	N	S	Ni	F
Min	0	0	1	0	3	0	0	0
Max	1000	4000	1	0	3	0	0	0

Results

Mass	Formula	Calculated Mass	Mass Difference [mDa]	Mass Difference [ppm]	DBE
296.26930	C17 H34 N3 O	296.26964	-0.34	-1.14	2.5

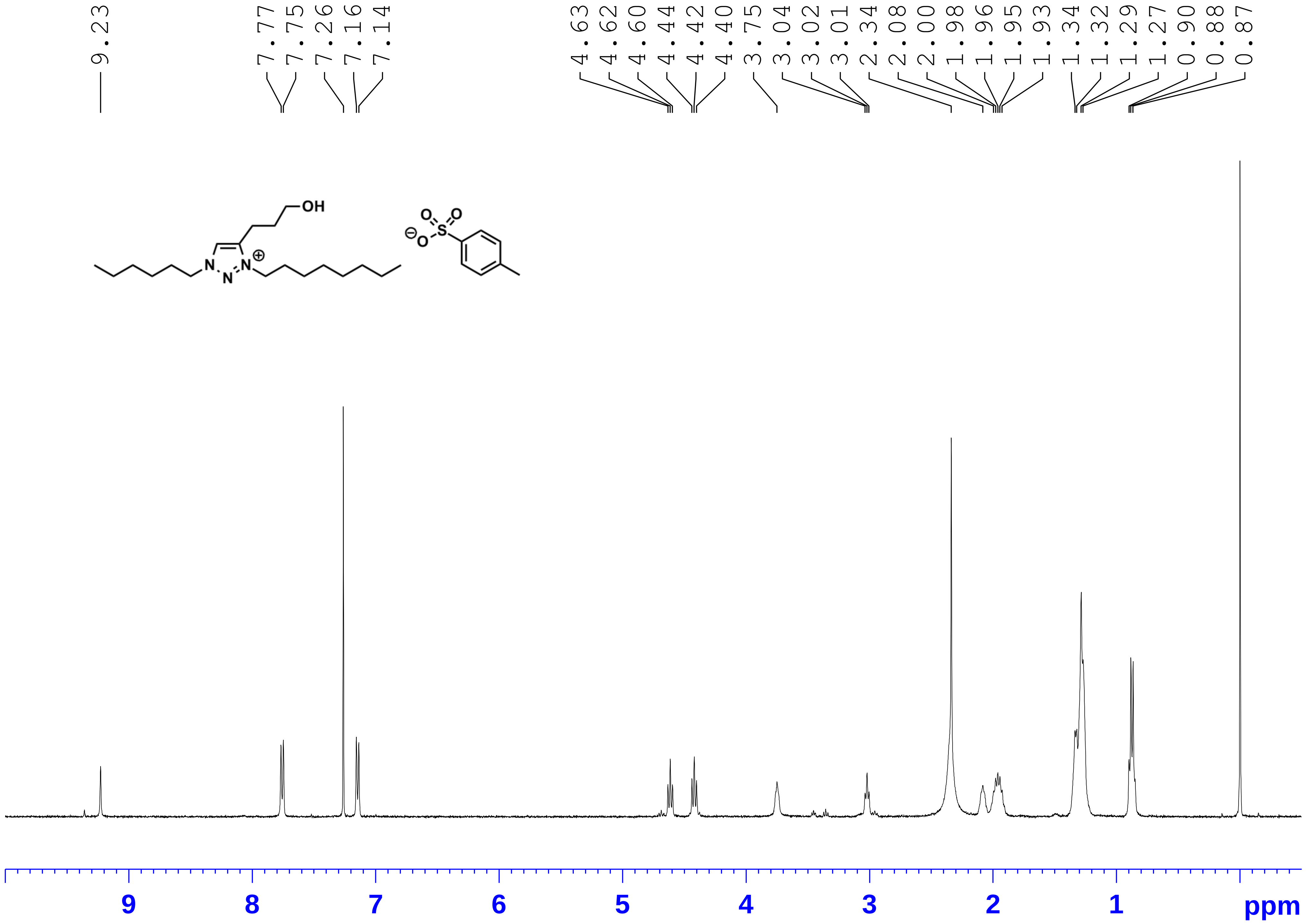

9.23
7.77
7.75
7.26
7.16
7.14
4.63
4.62
4.60
4.44
4.42
4.40
3.75
3.04
3.02
3.01
2.34
2.08
2.00
1.98
1.96
1.95
1.93
1.34
1.32
1.29
1.27
0.90
0.88
0.87
OH
N
N
N
O
O
S
O
9
8
7
6
5
4
3
2
1
ppm

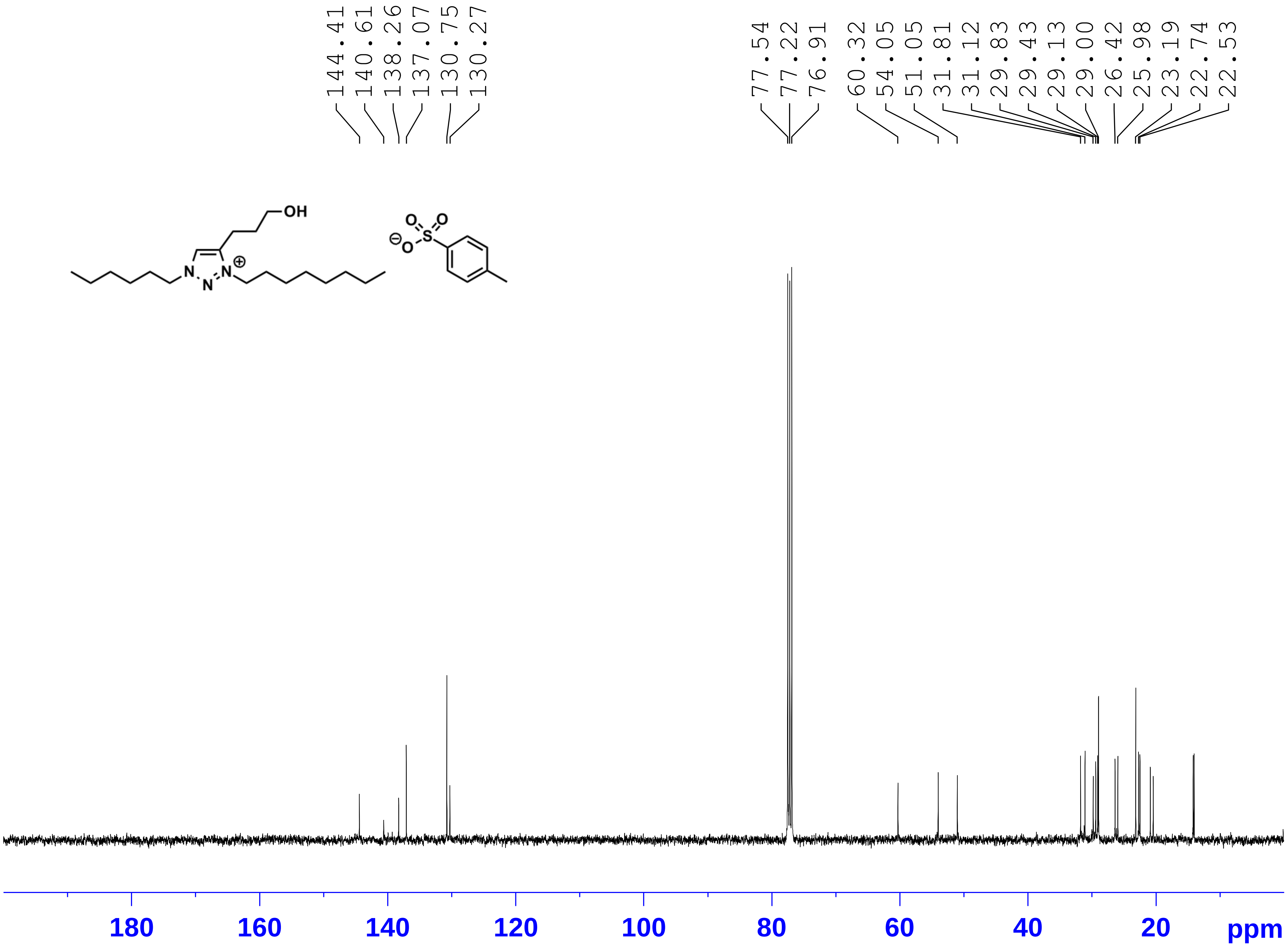
144.41
140.61
138.26
137.07
130.75
130.27
77.54
77.22
76.91
60.32
54.05
51.05
31.81
31.12
29.83
29.43
29.13
29.00
26.42
25.98
23.19
22.74
22.53
OH
O
S
N
180
160
140
120
100
80
60
40
20
ppm

Spectrum

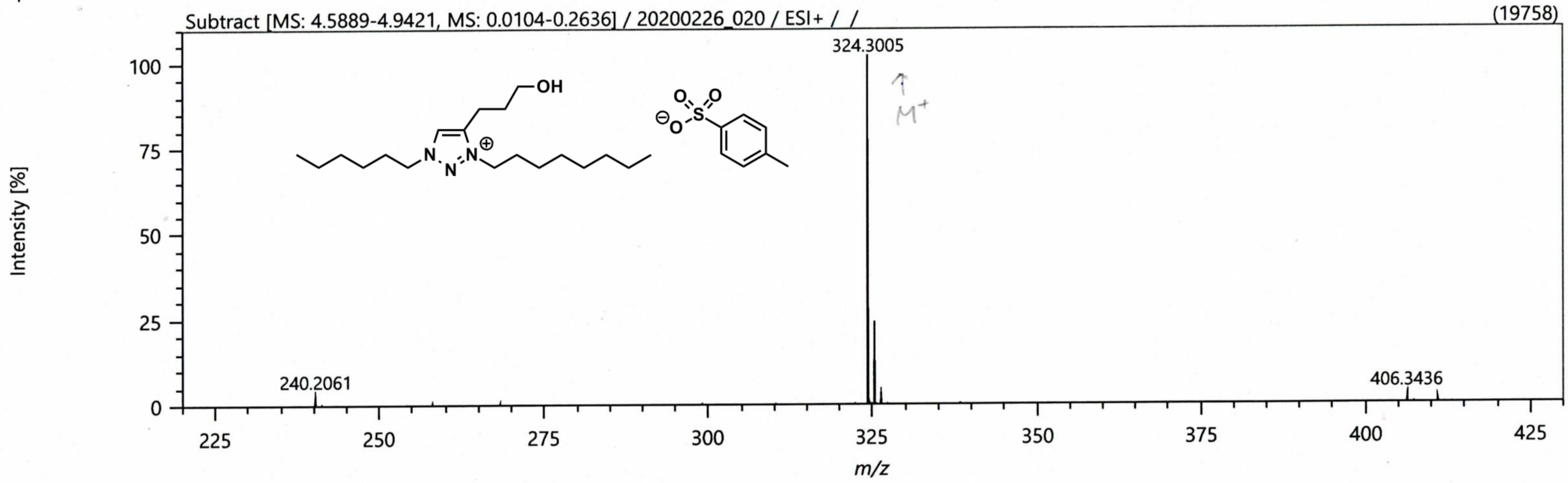

Elemental Composition

Parameters

Tolerance:	±10.00 ppm
Electron:	Odd/Even
Charge:	+1
DBE:	-99.0 - 999.0

Elements Set 1:

Symbol	C	H	O	Na	N	S	Ni	F
Min	0	0	1	0	3	0	0	0
Max	1000	4000	1	0	3	0	0	0

Results

Mass	Formula	Calculated Mass	Mass Difference [mDa]	Mass Difference [ppm]	DBE
324.30047	C19 H38 N3 O	324.30094	-0.47	-1.45	2.5

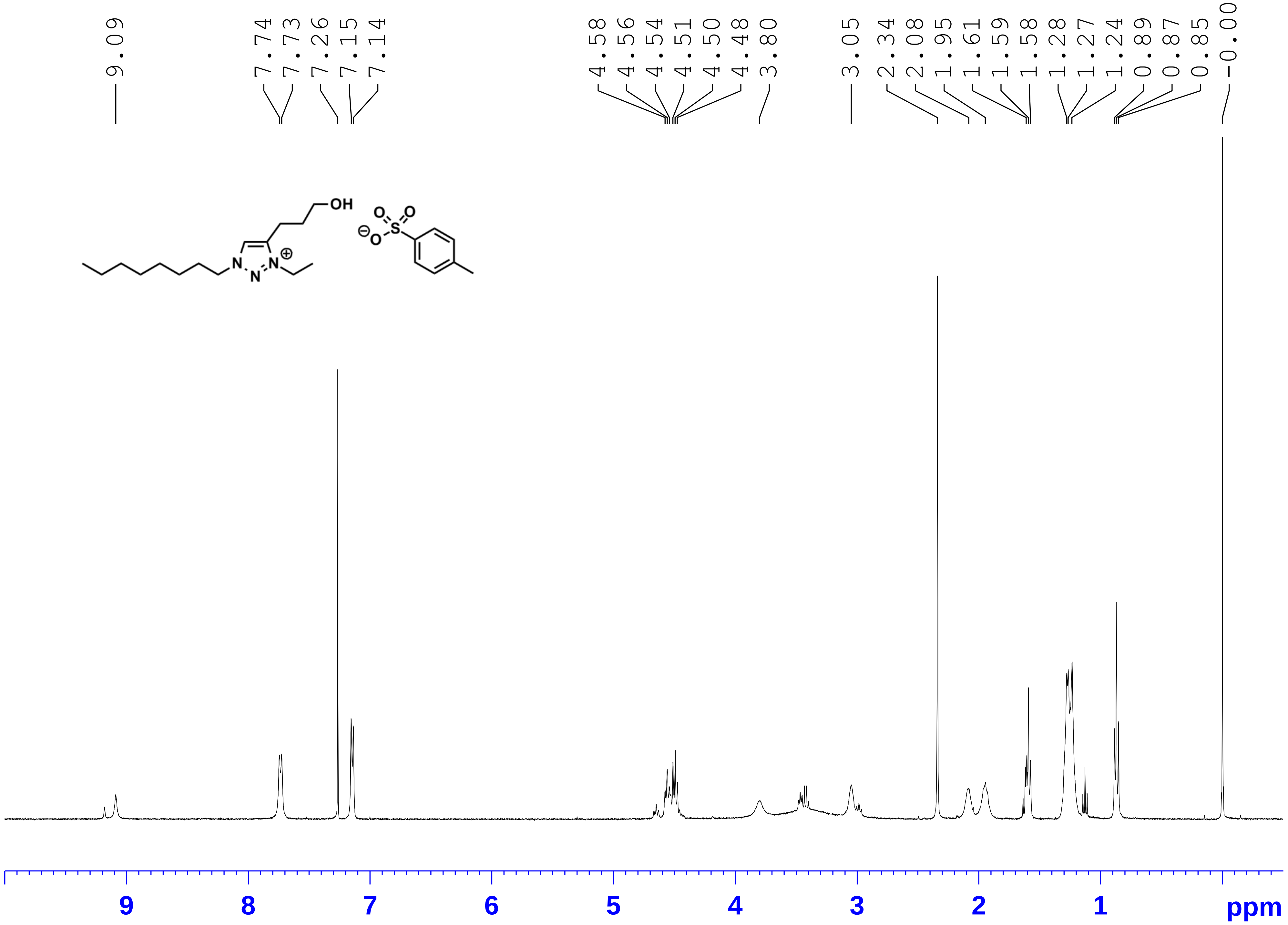
9.09
7.74
7.73
7.26
7.15
7.14
4.58
4.56
4.54
4.51
4.50
4.48
3.80
3.05
2.34
2.08
1.95
1.61
1.59
1.58
1.28
1.27
1.24
0.89
0.87
0.85
-0.00
OH
N
N
N
O
O
S
O
9
8
7
6
5
4
3
2
1
ppm

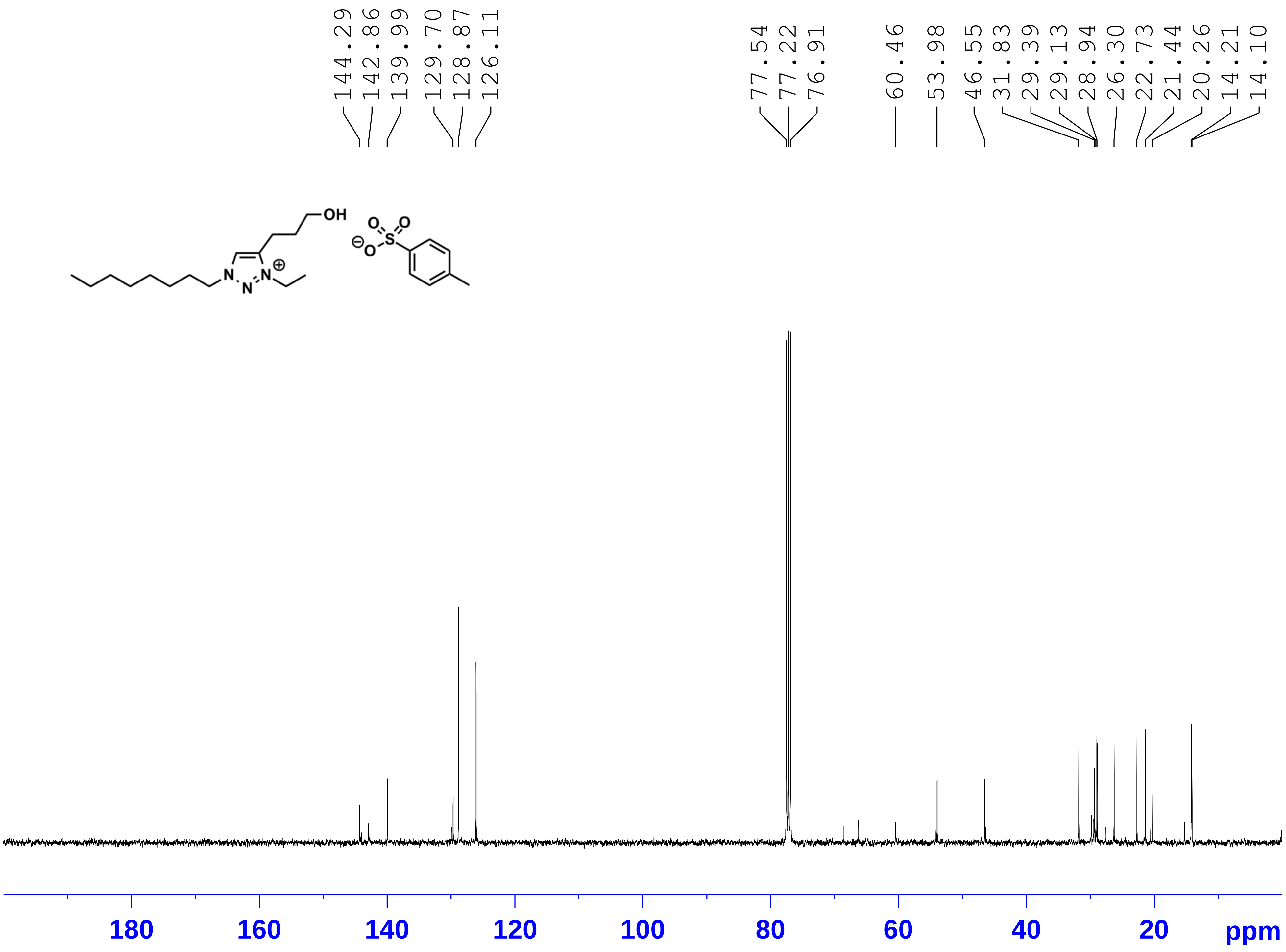
144.29
142.86
139.99
129.70
128.87
126.11
77.54
77.22
76.91
60.46
53.98
46.55
31.83
29.39
29.13
28.94
26.30
22.73
21.44
20.26
14.21
14.10
OH
O
S
O
O
N
N
N
180
160
140
120
100
80
60
40
20
ppm

Spectrum

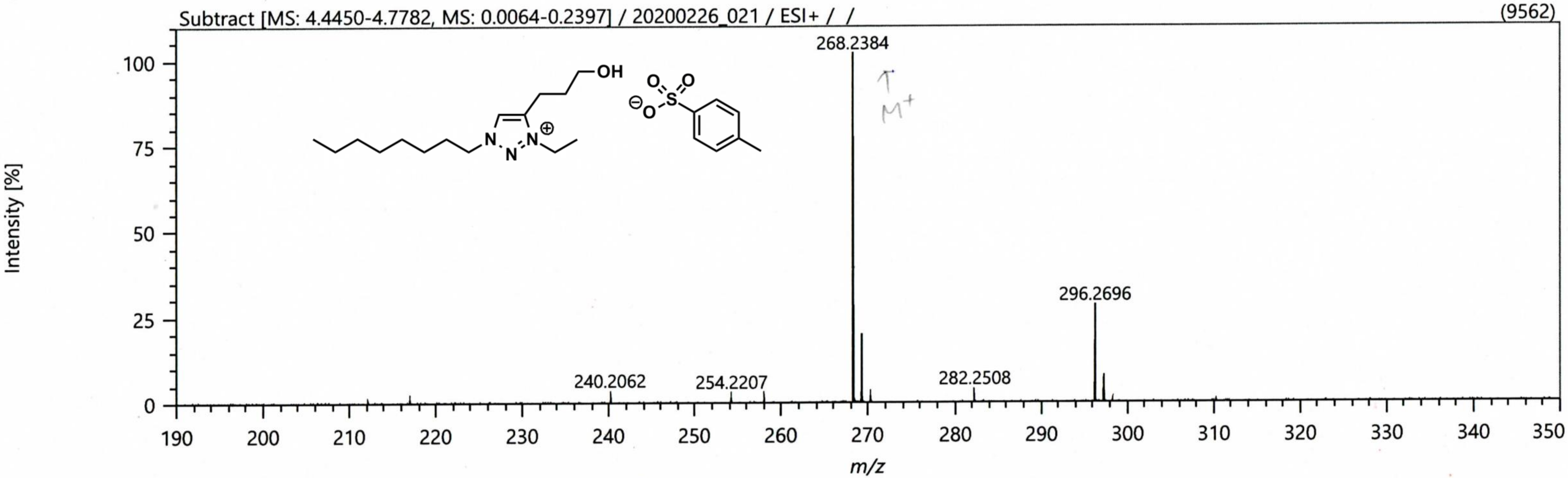

Elemental Composition

Parameters

Tolerance:	±10.00 ppm
Electron:	Odd/Even
Charge:	+1
DBE:	-99.0 - 999.0

Elements Set 1:

Symbol	C	H	O	Na	N	S	Ni	F
Min	0	0	1	0	3	0	0	0
Max	1000	4000	1	0	3	0	0	0

Results

Mass	Formula	Calculated Mass	Mass Difference [mDa]	Mass Difference [ppm]	DBE
268.23837	C15 H30 N3 O	268.23834	0.03	0.13	2.5

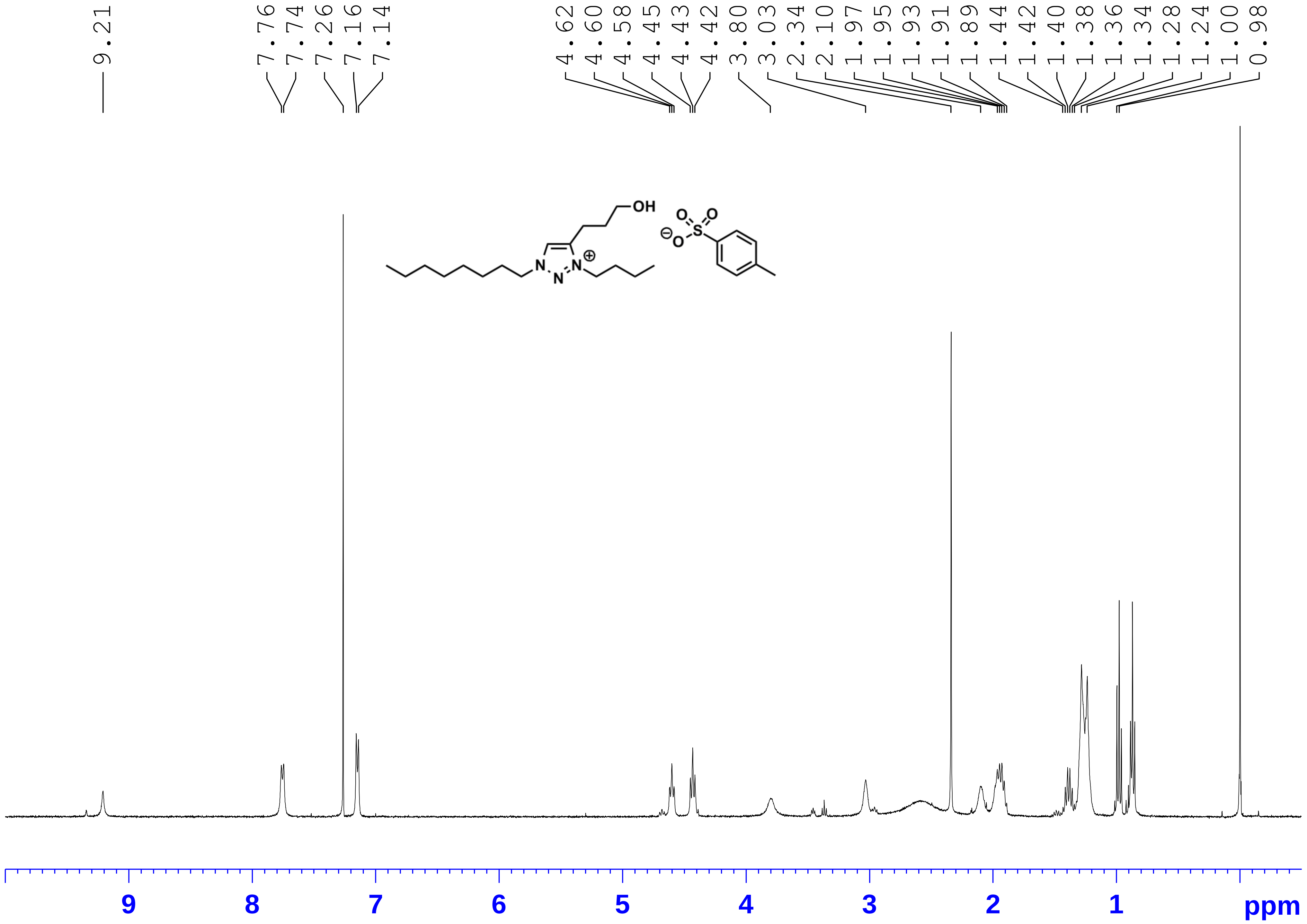
9.21
7.76
7.74
7.26
7.16
7.14
4.62
4.60
4.58
4.45
4.43
4.42
3.80
3.03
2.34
2.10
1.97
1.95
1.93
1.91
1.89
1.44
1.42
1.40
1.38
1.36
1.34
1.28
1.24
1.00
0.98
OH
9
8
7
6
5
4
3
2
1
ppm

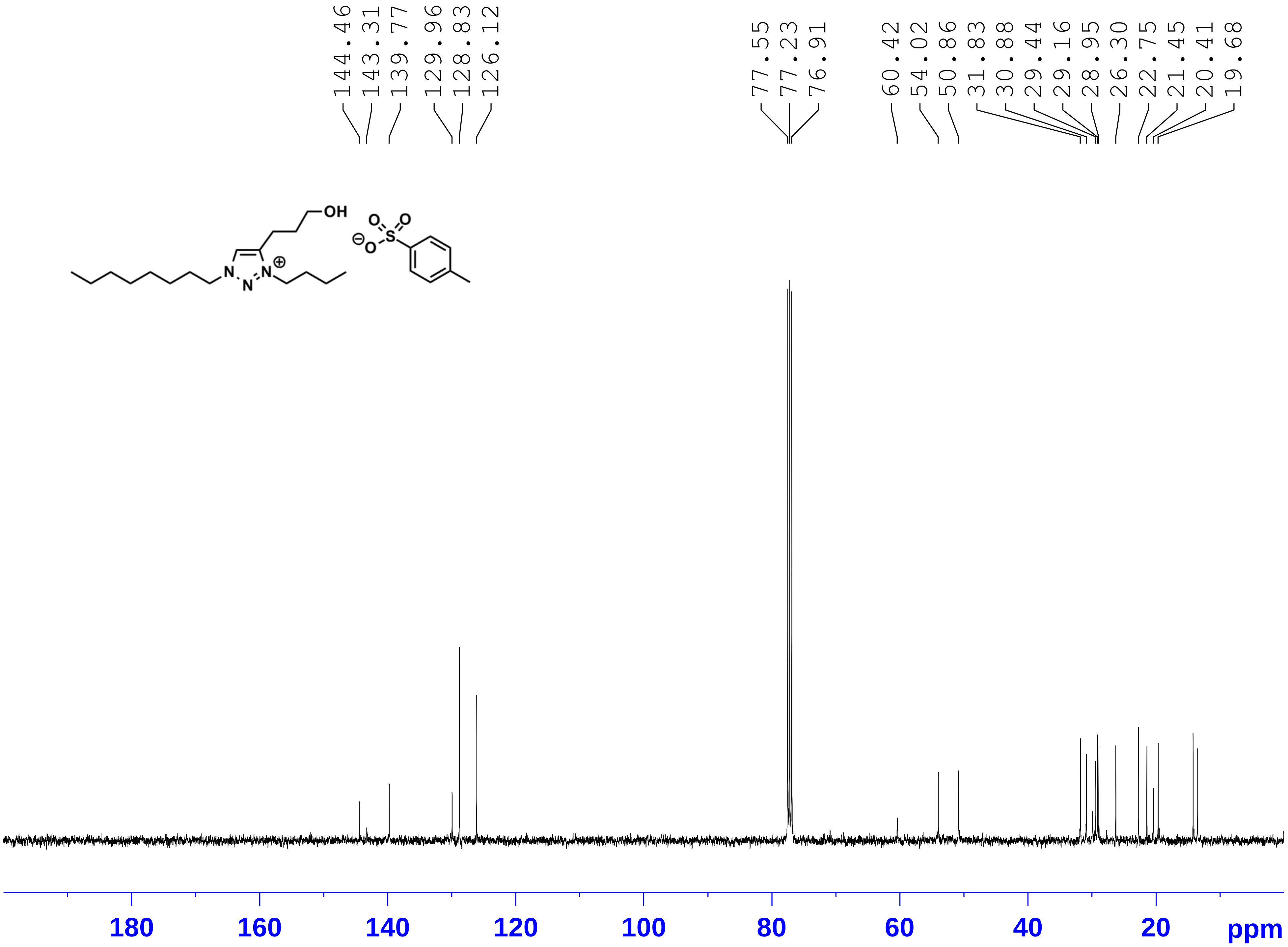
144.46
143.31
139.77
129.96
128.83
126.12
77.55
77.23
76.91
60.42
54.02
50.86
31.83
30.88
29.44
29.16
28.95
26.30
22.75
21.45
20.41
19.68
OH
O
S
O
O
N
N
N
180
160
140
120
100
80
60
40
20
ppm

Spectrum

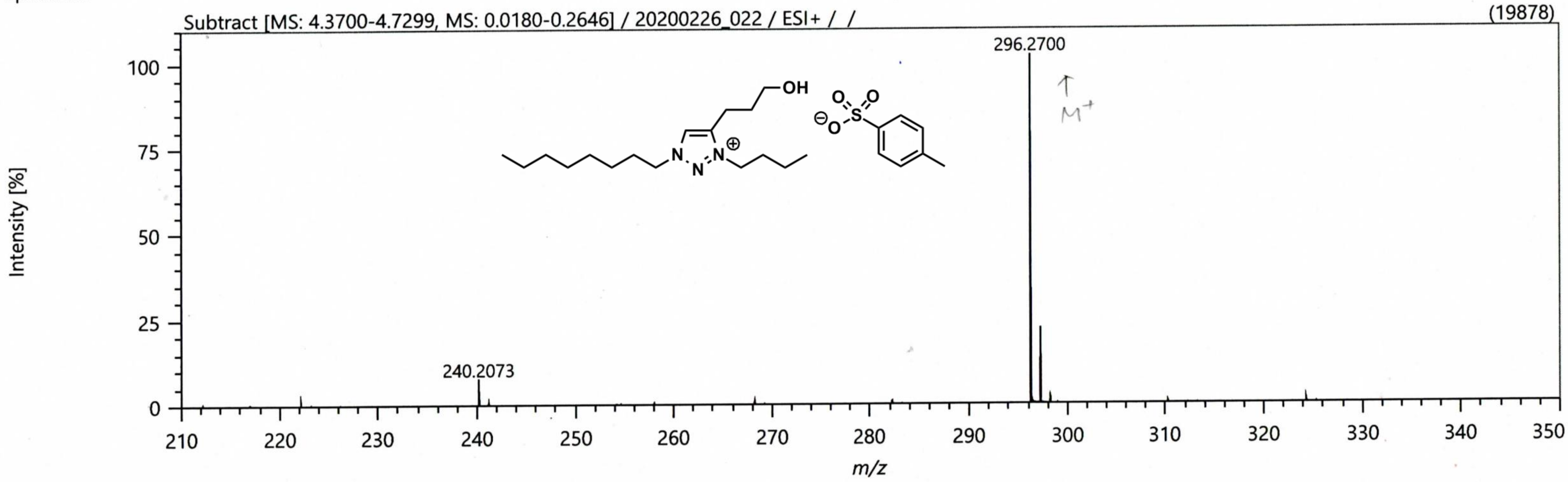

Elemental Composition

Parameters

Tolerance: ±10.00 ppm
Electron: Odd/Even
Charge: +1
DBE: -99.0 - 999.0

Elements Set 1:

Symbol	C	H	O	Na	N	S	Ni	F
Min	0	0	1	0	3	0	0	0
Max	1000	4000	1	0	3	0	0	0

Results

Mass	Formula	Calculated Mass	Mass Difference [mDa]	Mass Difference [ppm]	DBE
296.26996	C17 H34 N3 O	296.26964	0.32	1.08	2.5

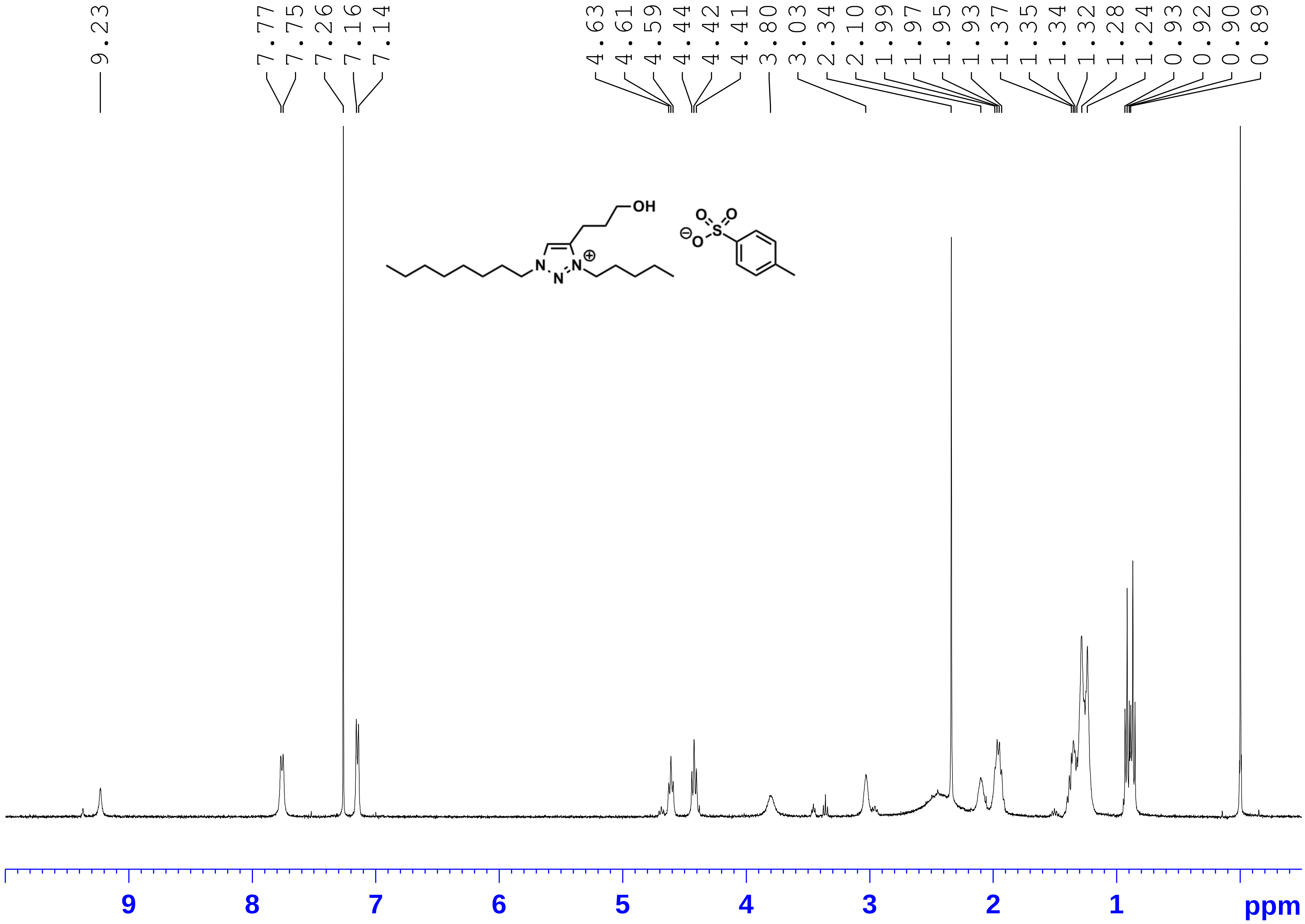
9.23
7.77
7.75
7.26
7.16
7.14
4.63
4.61
4.59
4.44
4.42
4.41
3.80
3.03
2.34
2.10
1.99
1.97
1.95
1.93
1.37
1.35
1.34
1.32
1.28
1.24
0.93
0.92
0.90
0.89
OH
N
N
N
O
O
S
O
9
8
7
6
5
4
3
2
1
ppm

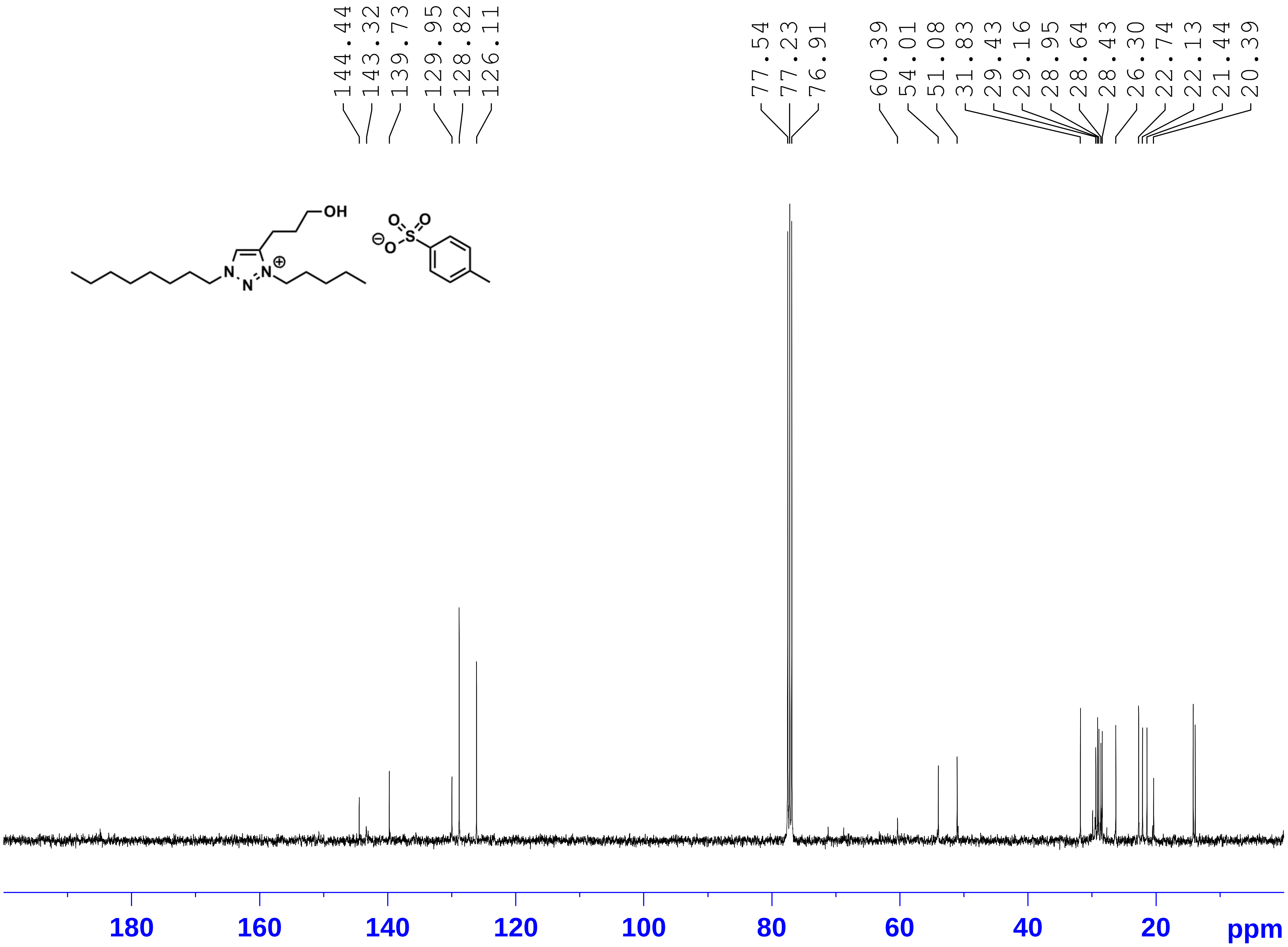
144.44
143.32
139.73
129.95
128.82
126.11
77.54
77.23
76.91
60.39
54.01
51.08
31.83
29.43
29.16
28.95
28.64
28.43
26.30
22.74
22.13
21.44
20.39
OH
O
S
O
O
N
N
N
180
160
140
120
100
80
60
40
20
ppm

Spectrum

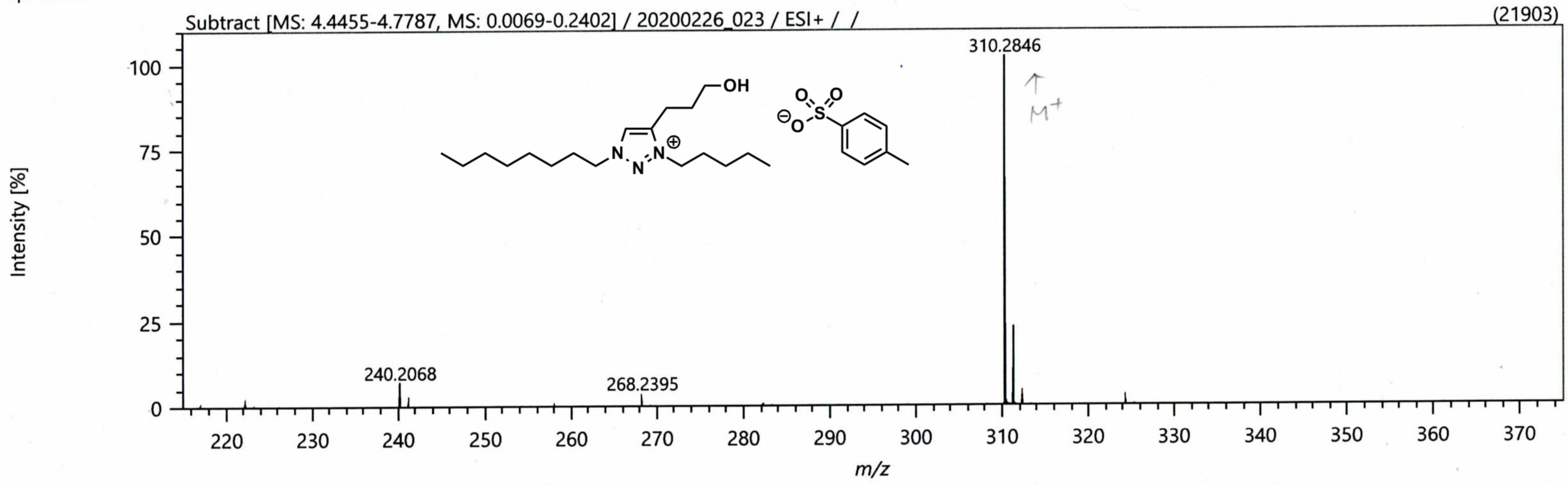

Elemental Composition

Parameters

Tolerance: ±10.00 ppm

Electron: Odd/Even

Charge: +1

DBE: -99.0 - 999.0

Elements Set 1:

Symbol	C	H	O	Na	N	S	Ni	F
Min	0	0	1	0	3	0	0	0
Max	1000	4000	1	0	3	0	0	0

Results

Mass	Formula	Calculated Mass	Mass Difference [mDa]	Mass Difference [ppm]	DBE
310.28464	C18 H36 N3 O	310.28529	-0.65	-2.08	2.5

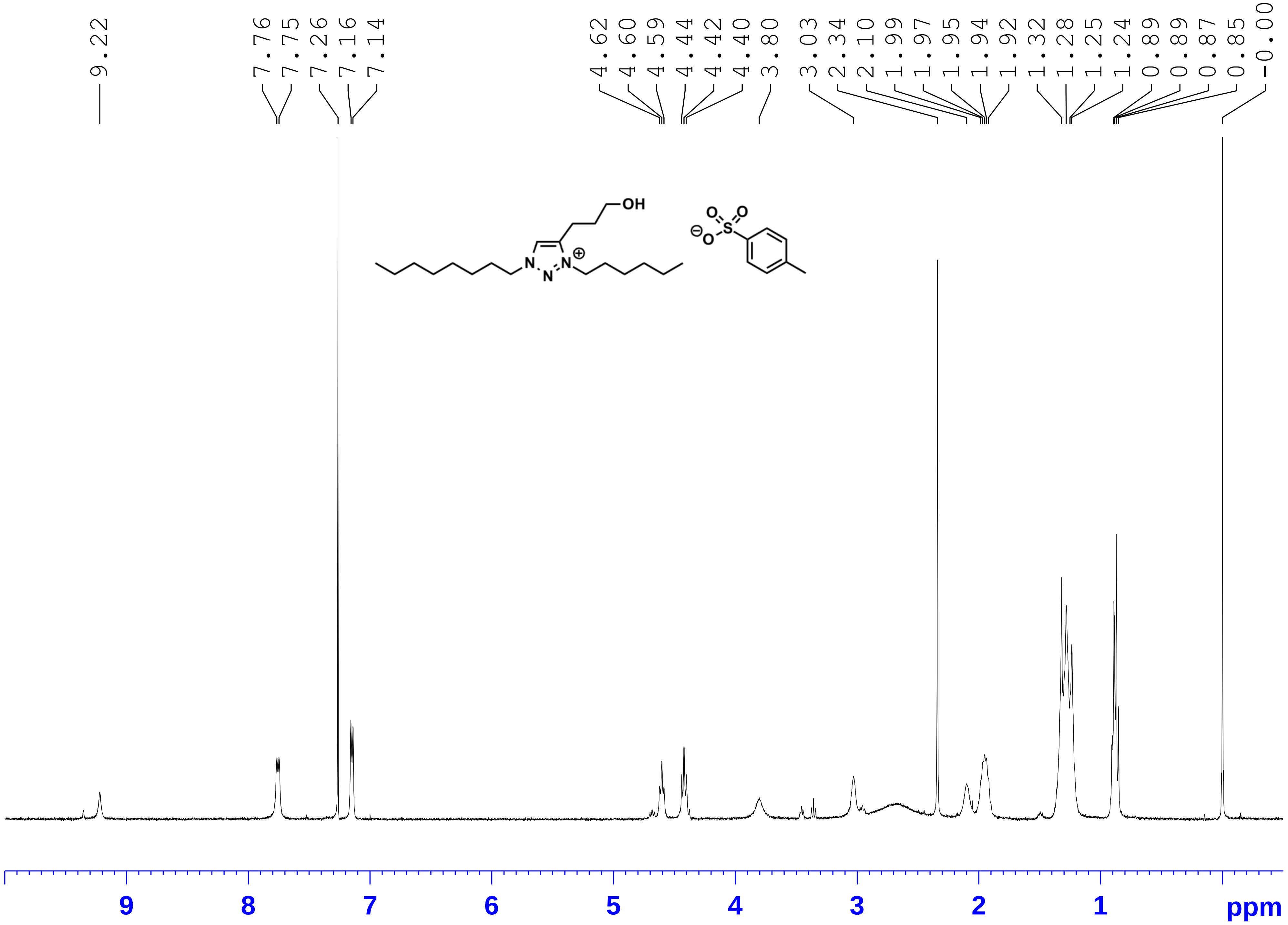
9.22
7.76
7.75
7.26
7.16
7.14
4.62
4.60
4.59
4.44
4.42
4.40
3.80
3.03
2.34
2.10
1.99
1.97
1.95
1.94
1.92
1.32
1.28
1.25
1.24
0.89
0.89
0.87
0.85
-0.00
OH
O
O
S
O
N
N
N
9
8
7
6
5
4
3
2
1
ppm

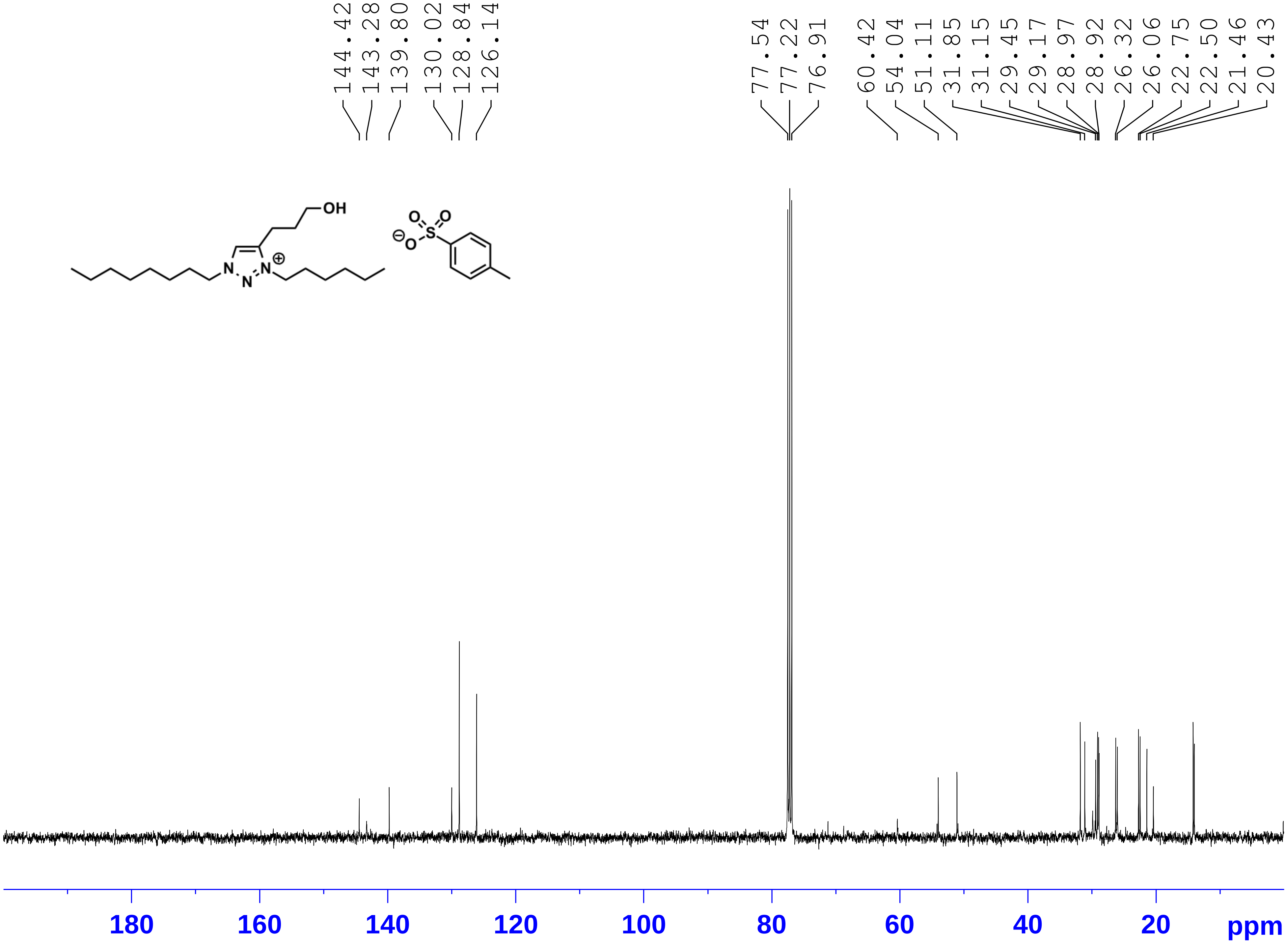
144.42
143.28
139.80
130.02
128.84
126.14
77.54
77.22
76.91
60.42
54.04
51.11
31.85
31.15
29.45
29.17
28.97
28.92
26.32
26.06
22.75
22.50
21.46
20.43
OH
O
O
S
O
N
N
N
180
160
140
120
100
80
60
40
20
ppm

Spectrum

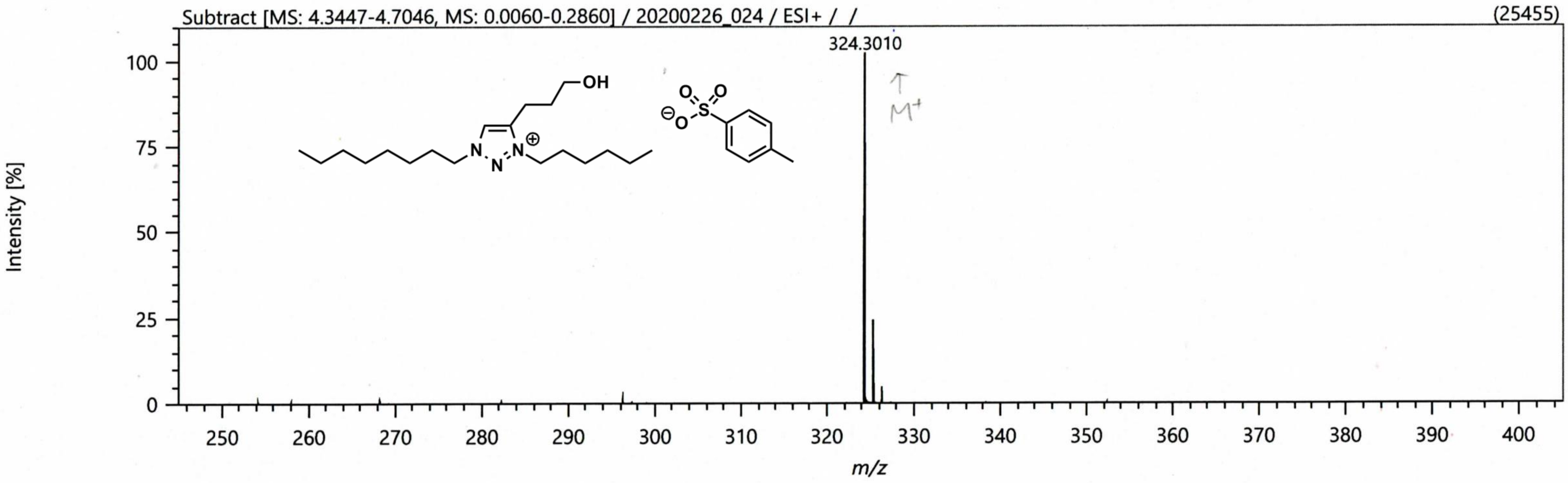

Elemental Composition

Parameters		Elements Set 1:								
Tolerance:	±10.00 ppm	Symbol	C	H	O	Na	N	S	Ni	F
Electron:	Odd/Even	Min	0	0	1	0	3	0	0	0
Charge:	+1	Max	1000	4000	1	0	3	0	0	0
DBE:	-99.0 - 999.0									

Results

Mass	Formula	Calculated Mass	Mass Difference [mDa]	Mass Difference [ppm]	DBE
324.30103	C19 H38 N3 O	324.30094	0.09	0.29	2.5

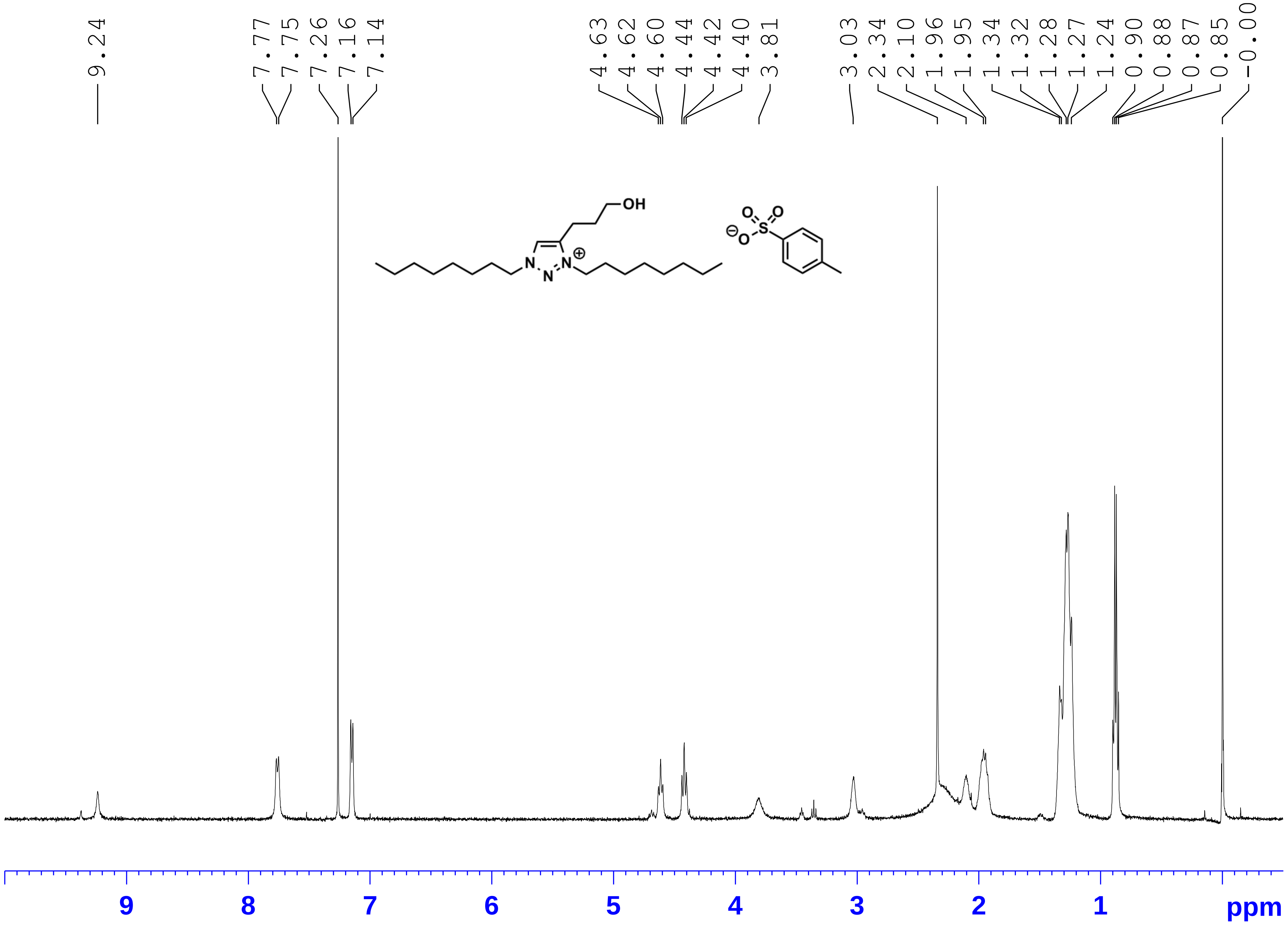
9.24
7.77
7.75
7.26
7.16
7.14
4.63
4.62
4.60
4.44
4.42
4.40
3.81
3.03
2.34
2.10
1.96
1.95
1.34
1.32
1.28
1.27
1.24
0.90
0.88
0.87
0.85
-0.00
OH
N
N
N
O
S
O
O
9
8
7
6
5
4
3
2
1
ppm

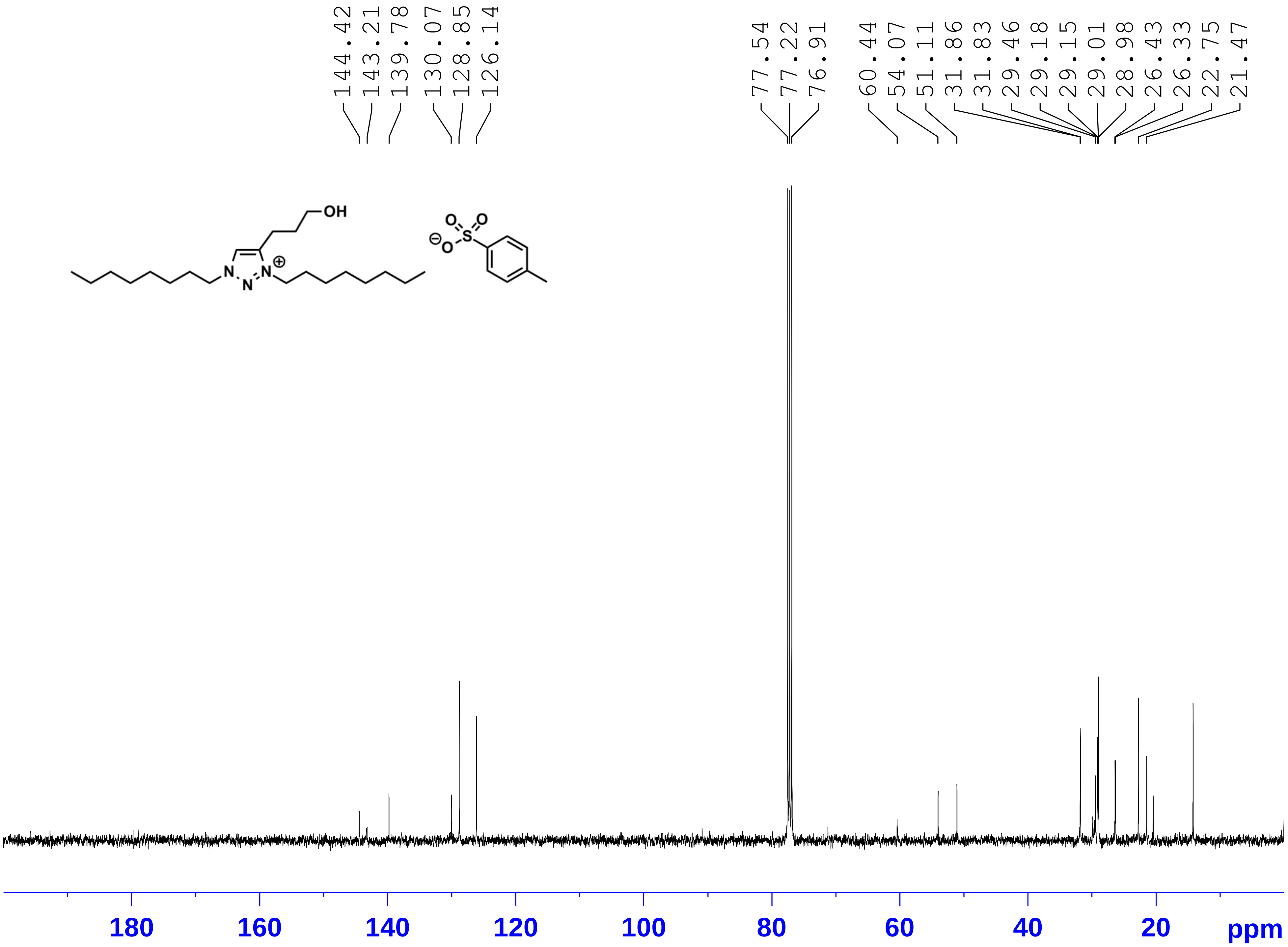
144.42
143.21
139.78
130.07
128.85
126.14
77.54
77.22
76.91
60.44
54.07
51.11
31.86
31.83
29.46
29.18
29.15
29.01
28.98
26.43
26.33
22.75
21.47
OH
N
N
N
O
O
O
S
180
160
140
120
100
80
60
40
20
ppm

Spectrum

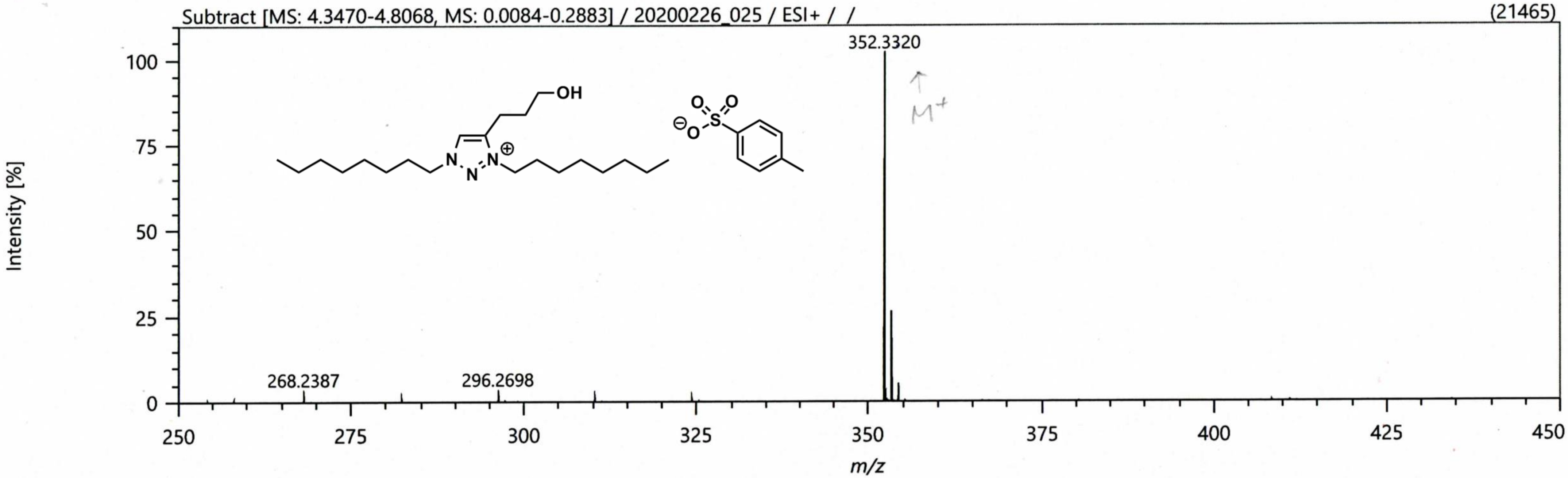

Elemental Composition

Parameters

Tolerance:	±10.00 ppm
Electron:	Odd/Even
Charge:	+1
DBE:	-99.0 - 999.0

Elements Set 1:

Symbol	C	H	O	Na	N	S	Ni	F
Min	0	0	1	0	3	0	0	0
Max	1000	4000	1	0	3	0	0	0

Results

Mass	Formula	Calculated Mass	Mass Difference [mDa]	Mass Difference [ppm]	DBE
352.33201	C21 H42 N3 O	352.33224	-0.23	-0.66	2.5

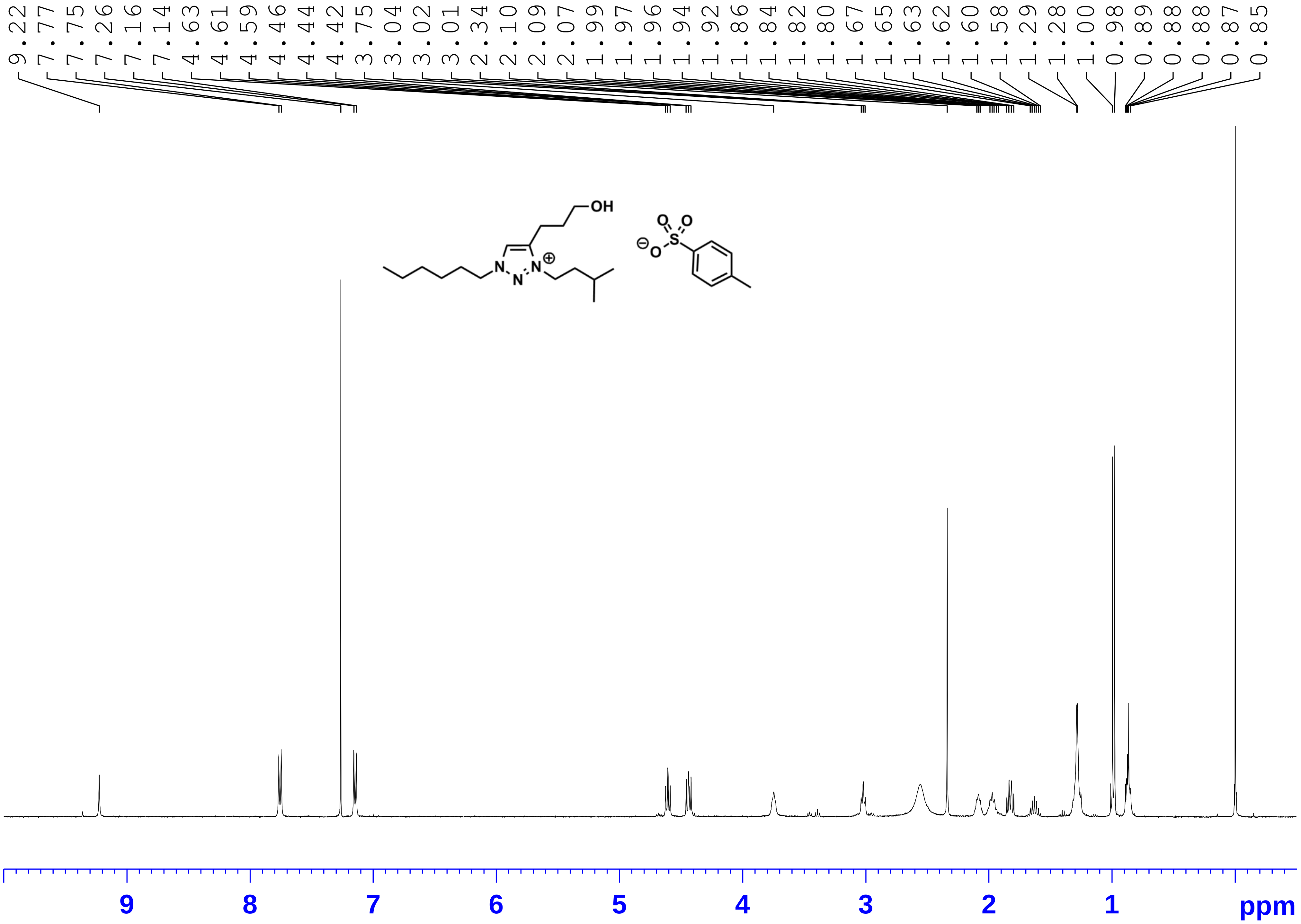
9.22
7.77
7.75
7.26
7.16
7.14
4.63
4.61
4.59
4.46
4.44
4.42
3.75
3.04
3.02
3.01
2.34
2.10
2.09
2.07
1.99
1.97
1.96
1.94
1.92
1.86
1.84
1.82
1.80
1.67
1.65
1.63
1.62
1.60
1.58
1.29
1.28
1.00
0.98
0.89
0.88
0.88
0.87
0.85
OH
N
N
N
O
O
S
O
9
8
7
6
5
4
3
2
1
ppm

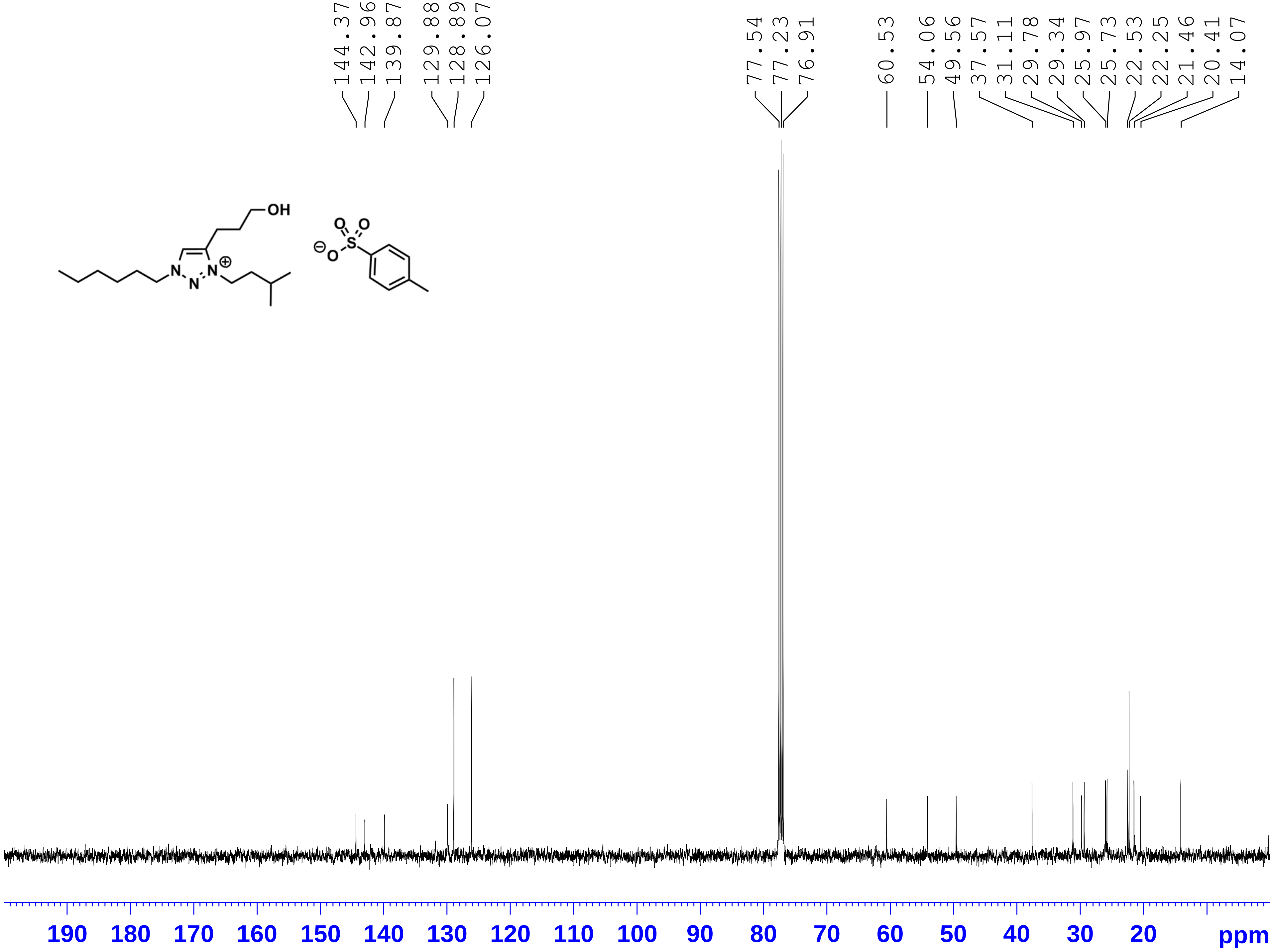
144.37
142.96
139.87
129.88
128.89
126.07
77.54
77.23
76.91
60.53
54.06
49.56
37.57
31.11
29.78
29.34
25.97
25.73
22.53
22.25
21.46
20.41
14.07
OH
O
O
S
O
N
N
N
190
180
170
160
150
140
130
120
110
100
90
80
70
60
50
40
30
20
ppm

Spectrum

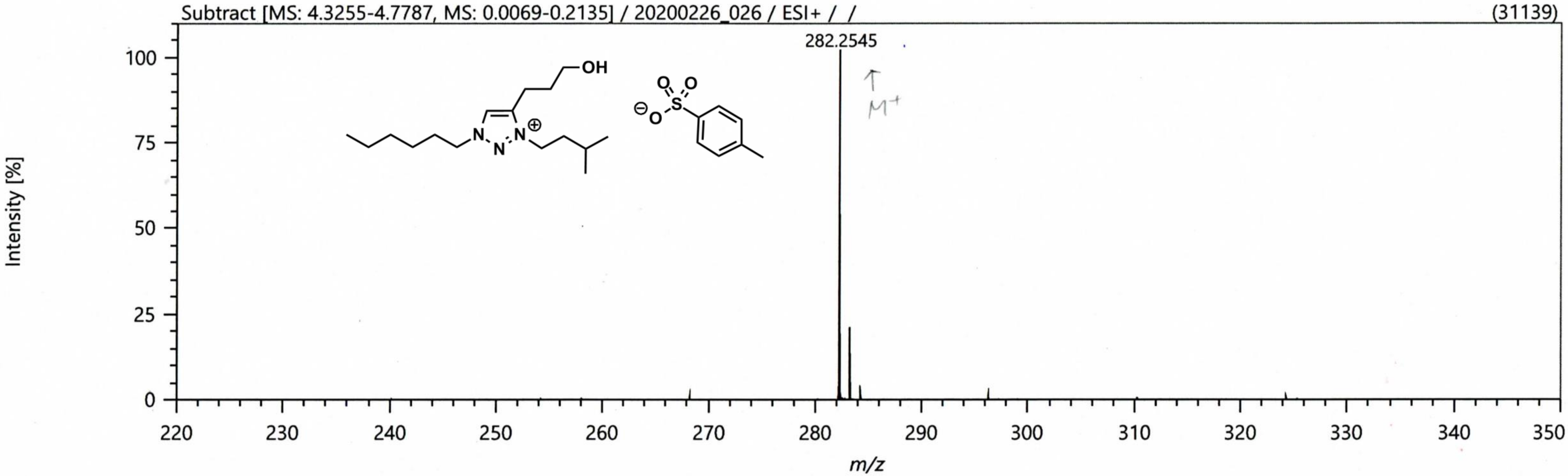

Elemental Composition

Parameters		Elements Set 1:								
Tolerance:	±10.00 ppm	Symbol	C	H	O	Na	N	S	Ni	F
Electron:	Odd/Even	Min	0	0	1	0	3	0	0	0
Charge:	+1	Max	1000	4000	1	0	3	0	0	0
DBE:	-99.0 - 999.0									

Results

Mass	Formula	Calculated Mass	Mass Difference [mDa]	Mass Difference [ppm]	DBE
282.25452	C16 H32 N3 O	282.25399	0.53	1.87	2.5

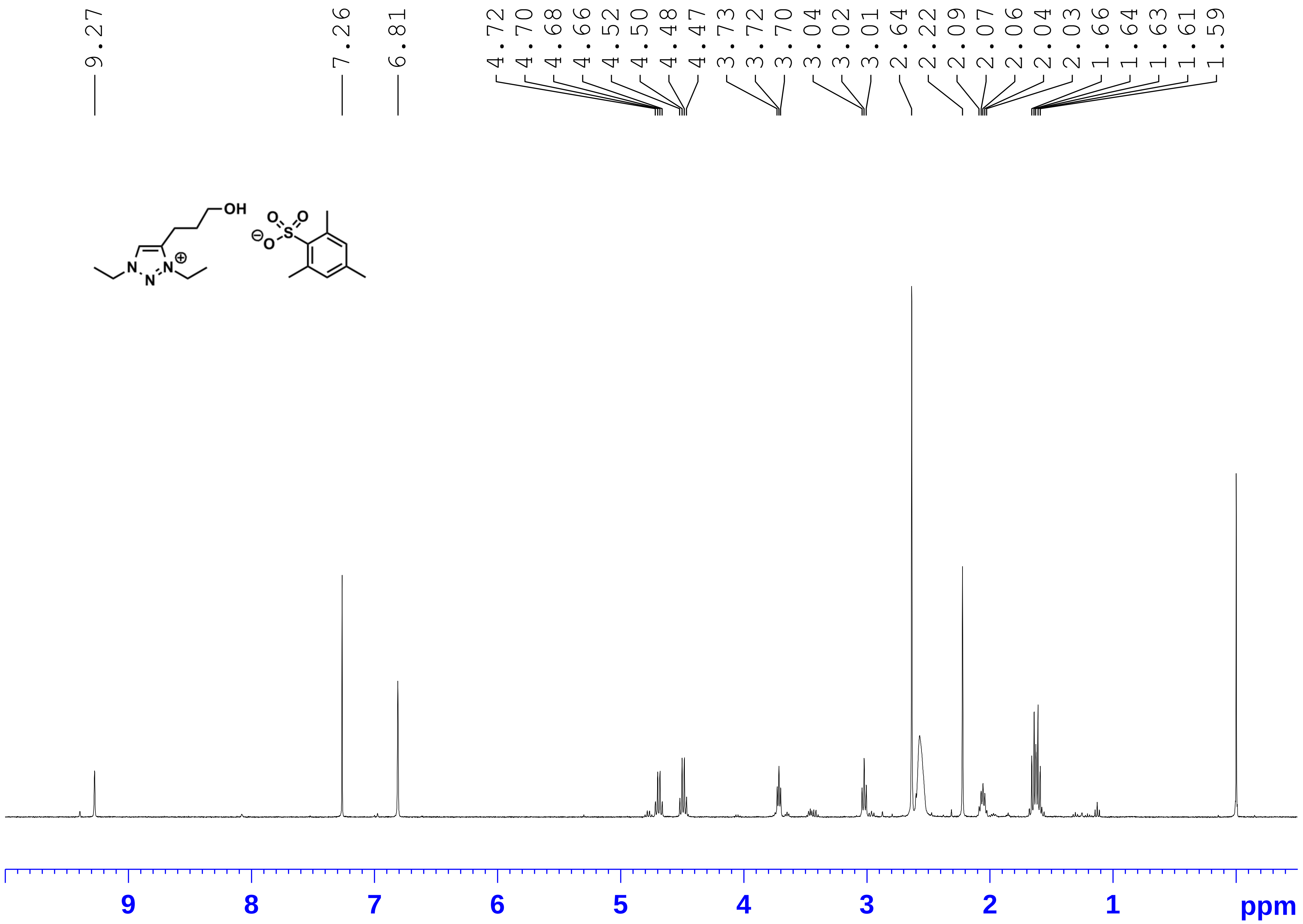
9.27
7.26
6.81
4.72
4.70
4.68
4.66
4.52
4.50
4.48
4.47
3.73
3.72
3.70
3.04
3.02
3.01
2.64
2.22
2.09
2.07
2.06
2.04
2.03
1.66
1.64
1.63
1.61
1.59
OH
O
S
O
O
N
N
N
9
8
7
6
5
4
3
2
1
ppm

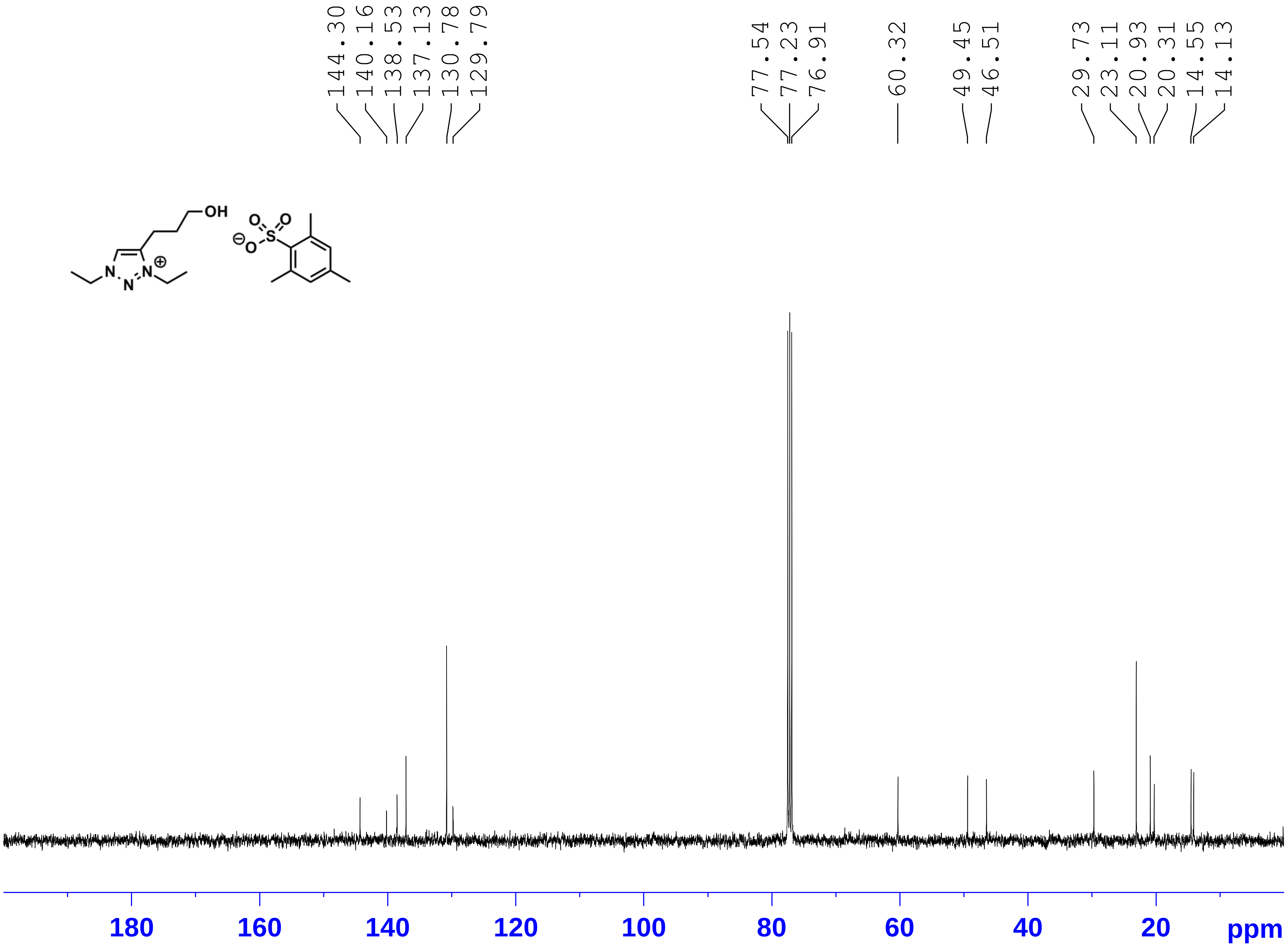
144.30
140.16
138.53
137.13
130.78
129.79
77.54
77.23
76.91
60.32
49.45
46.51
29.73
23.11
20.93
20.31
14.55
14.13
OH
O
O
S
O
N
N
N
180
160
140
120
100
80
60
40
20
ppm

Spectrum

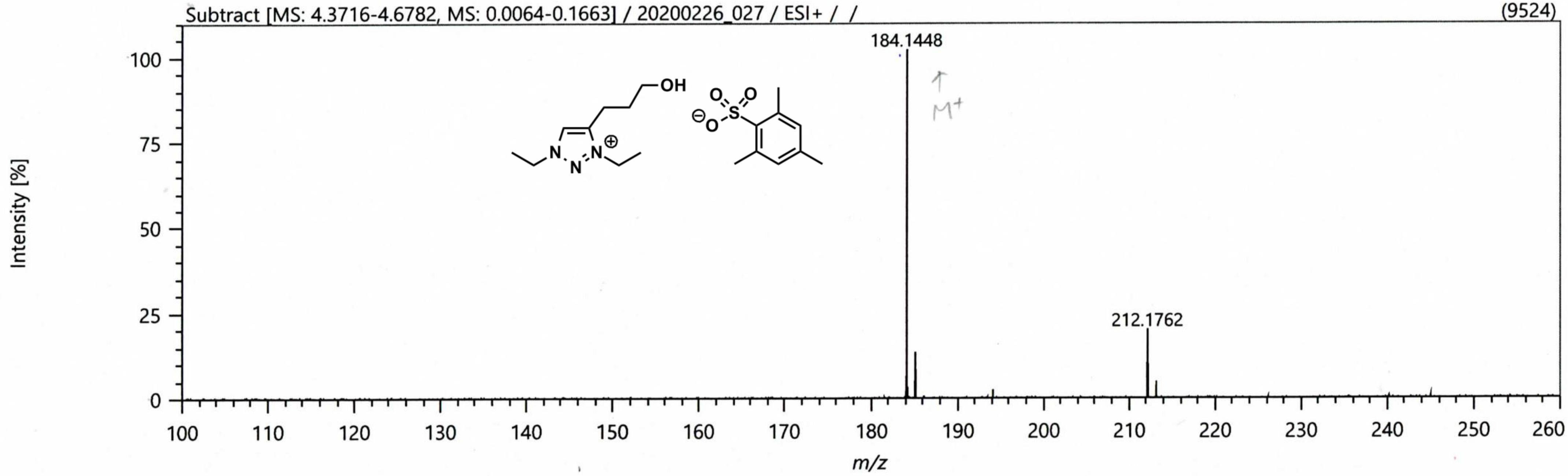

Elemental Composition

Parameters

Tolerance:	±10.00 ppm
Electron:	Odd/Even
Charge:	+1
DBE:	-99.0 - 999.0

Elements Set 1:

Symbol	C	H	O	Na	N	S	Ni	F
Min	0	0	1	0	3	0	0	0
Max	1000	4000	1	0	3	0	0	0

Results

Mass	Formula	Calculated Mass	Mass Difference [mDa]	Mass Difference [ppm]	DBE
184.14478	C9 H18 N3 O	184.14444	0.35	1.88	2.5

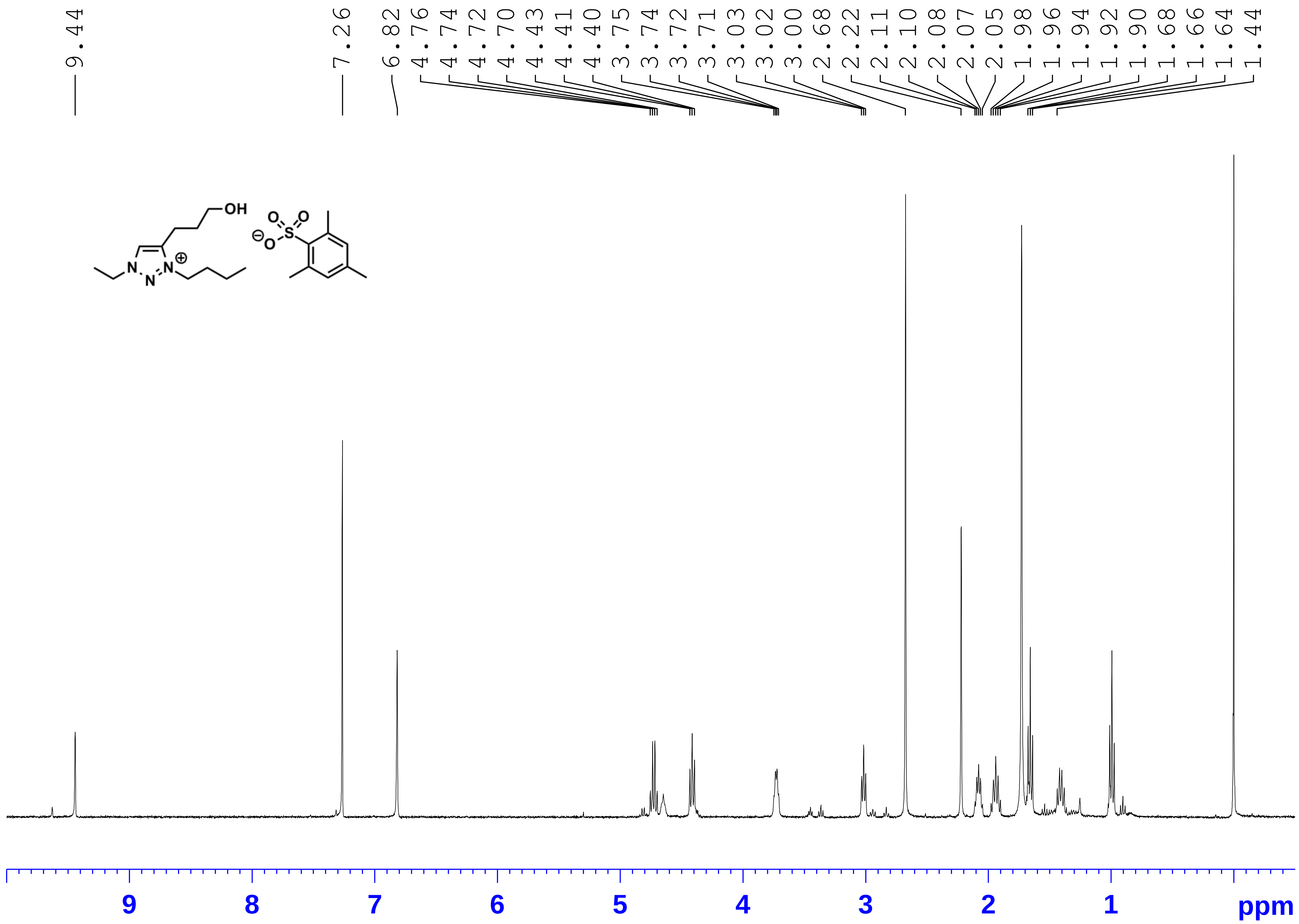
9.44
7.26
6.82
4.76
4.74
4.72
4.70
4.43
4.41
4.40
3.75
3.74
3.72
3.71
3.03
3.02
3.00
2.68
2.22
2.11
2.10
2.08
2.07
2.05
1.98
1.96
1.94
1.92
1.90
1.68
1.66
1.64
1.44
OH
O
S
O
O
N
N
N
9
8
7
6
5
4
3
2
1
ppm

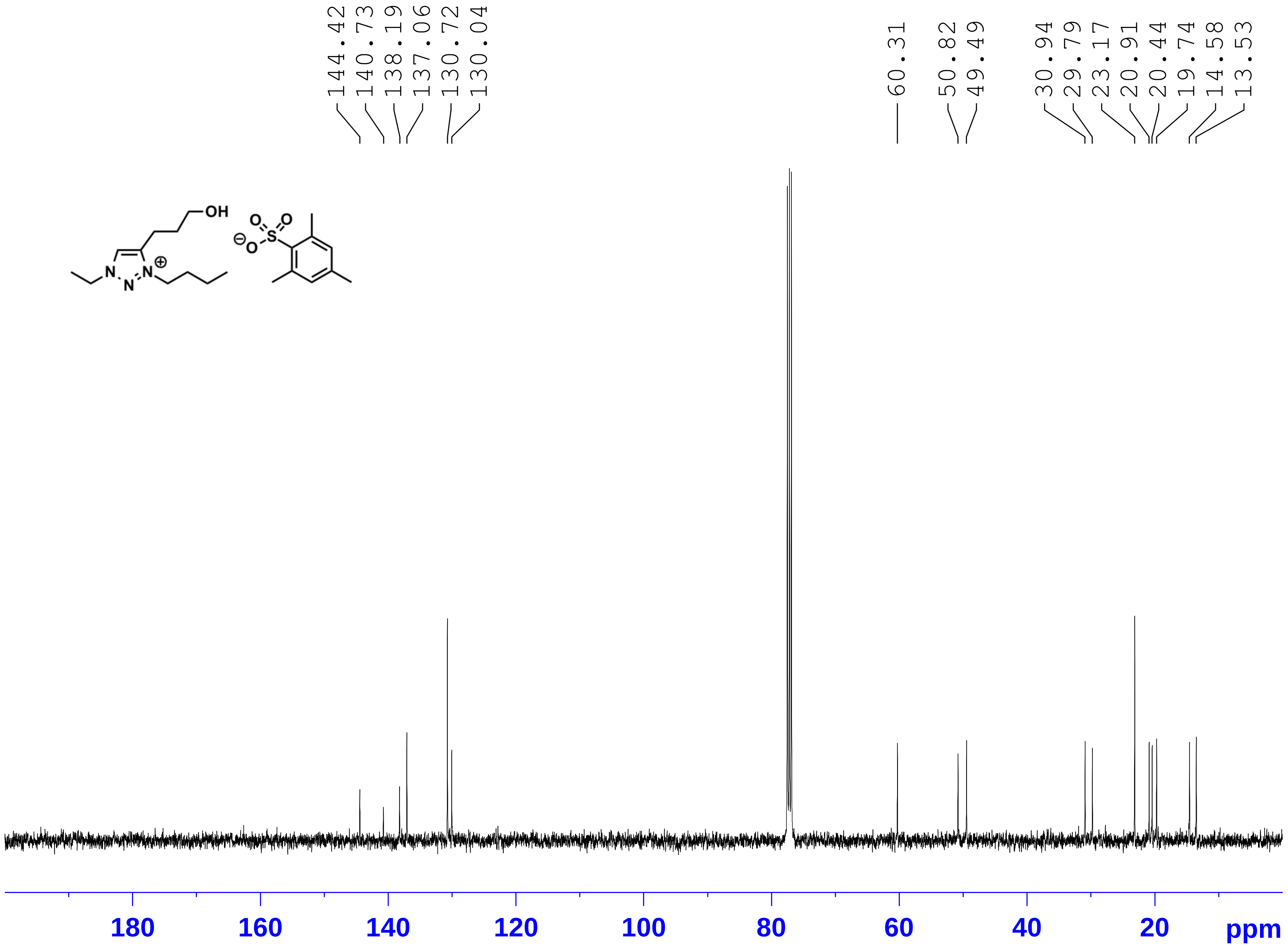
144.42
140.73
138.19
137.06
130.72
130.04
60.31
50.82
49.49
30.94
29.79
23.17
20.91
20.44
19.74
14.58
13.53
OH
O
S
N
180
160
140
120
100
80
60
40
20
ppm

Spectrum

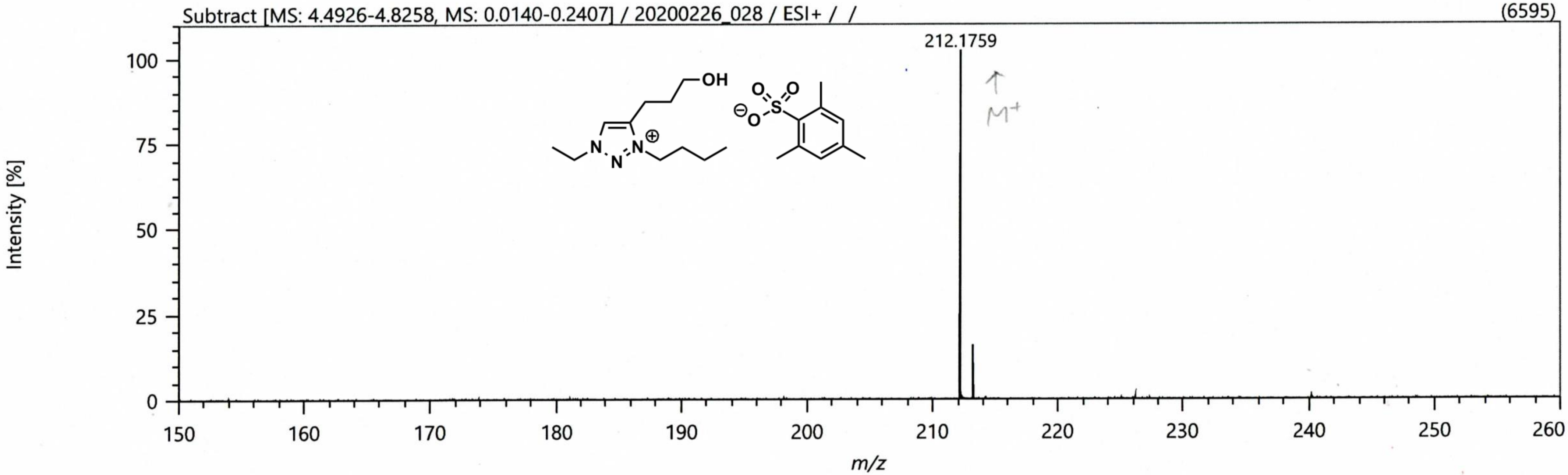

Elemental Composition

Parameters		Elements Set 1:								
Tolerance:	±10.00 ppm	Symbol	C	H	O	Na	N	S	Ni	F
Electron:	Odd/Even	Min	0	0	1	0	3	0	0	0
Charge:	+1	Max	1000	4000	1	0	3	0	0	0
DBE:	-99.0 - 999.0									

Results

Mass	Formula	Calculated Mass	Mass Difference [mDa]	Mass Difference [ppm]	DBE
212.17589	C11 H22 N3 O	212.17574	0.15	0.71	2.5

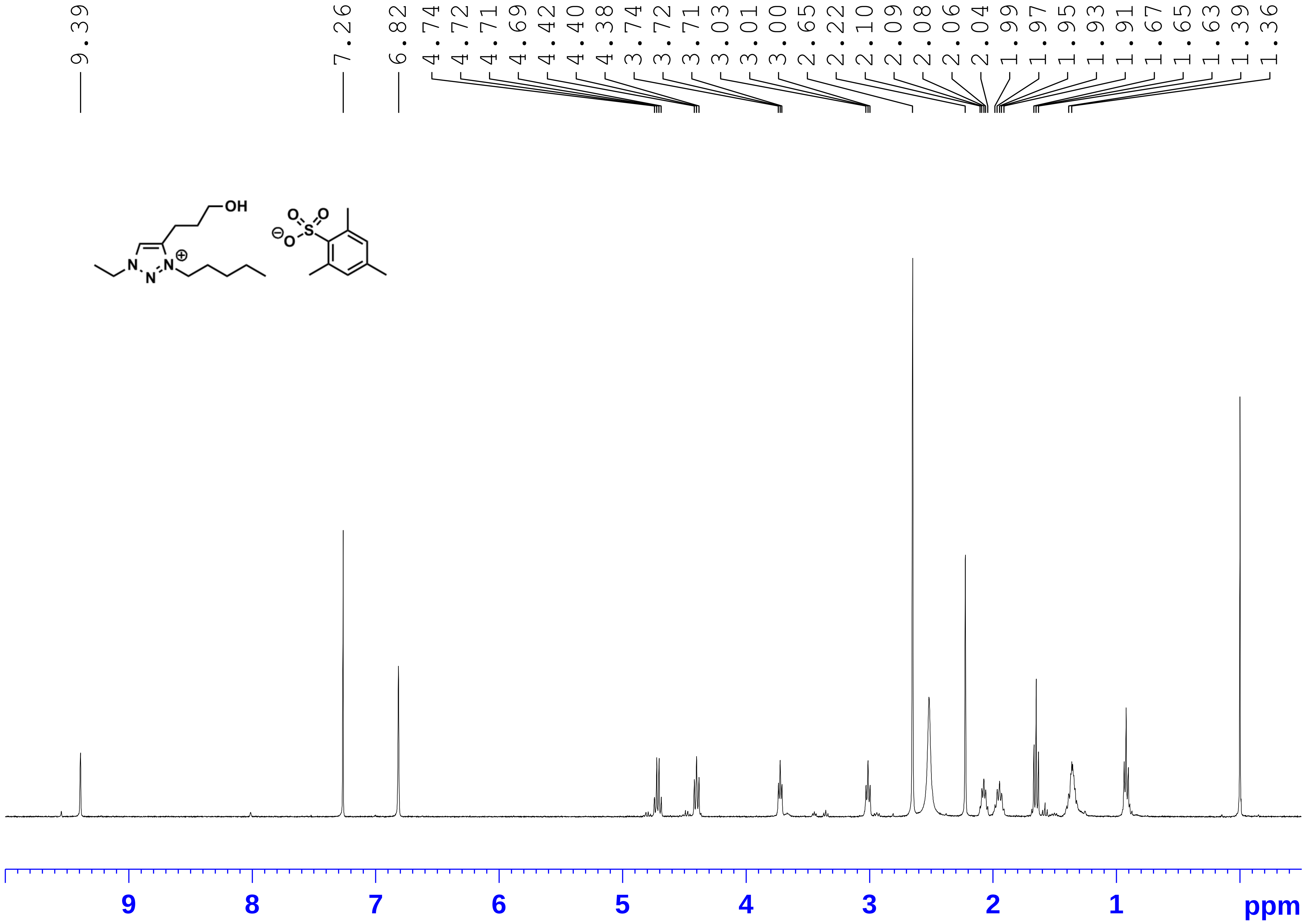
9.39
7.26
6.82
4.74
4.72
4.71
4.69
4.42
4.40
4.38
3.74
3.72
3.71
3.03
3.01
3.00
2.65
2.22
2.10
2.09
2.08
2.06
2.04
1.99
1.97
1.95
1.93
1.91
1.67
1.65
1.63
1.39
1.36
OH
N
N
N
O
S
O
O
9
8
7
6
5
4
3
2
1
ppm

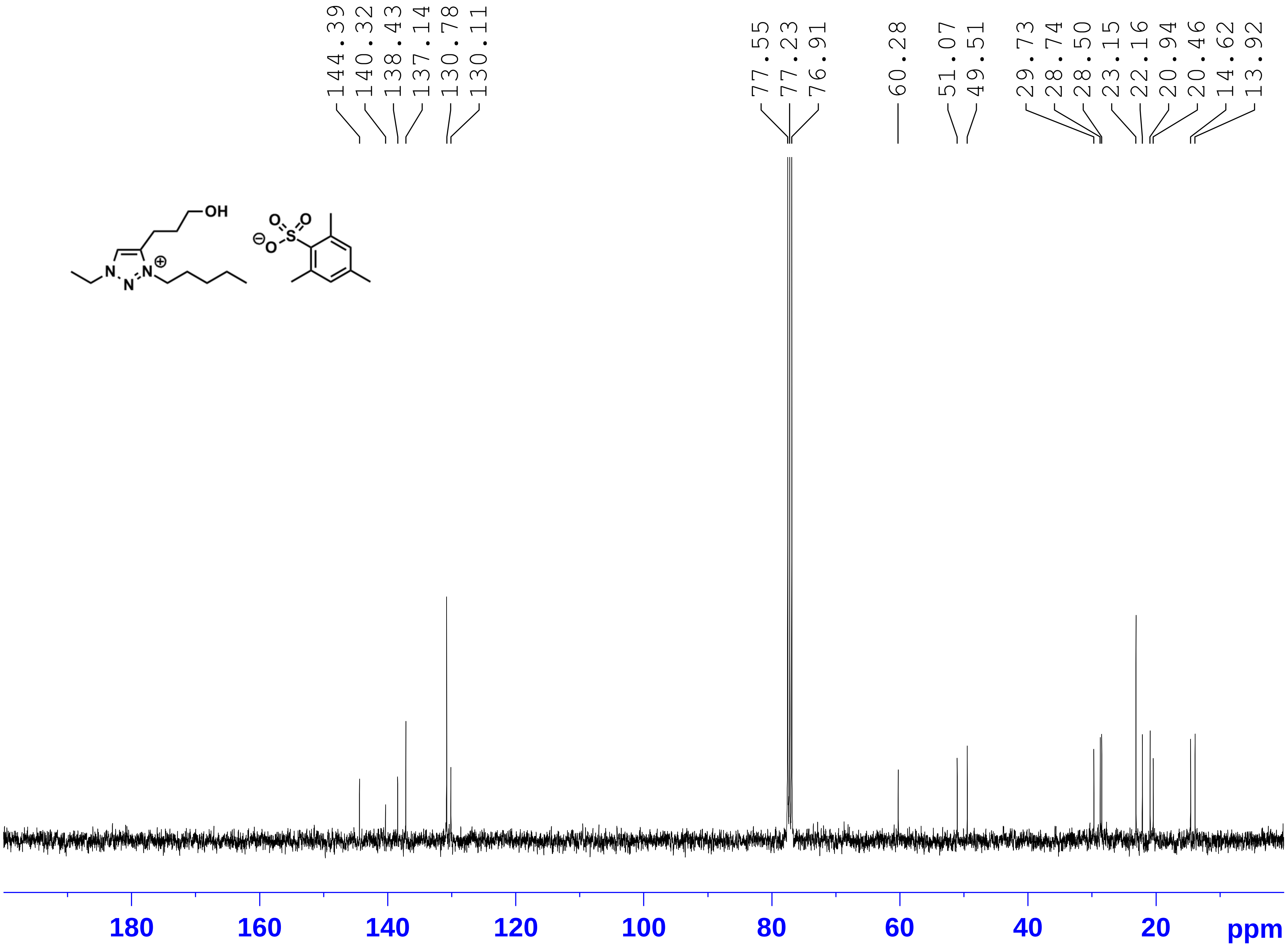
144.39
140.32
138.43
137.14
130.78
130.11
77.55
77.23
76.91
60.28
51.07
49.51
29.73
28.74
28.50
23.15
22.16
20.94
20.46
14.62
13.92
OH
180
160
140
120
100
80
60
40
20
ppm

Spectrum

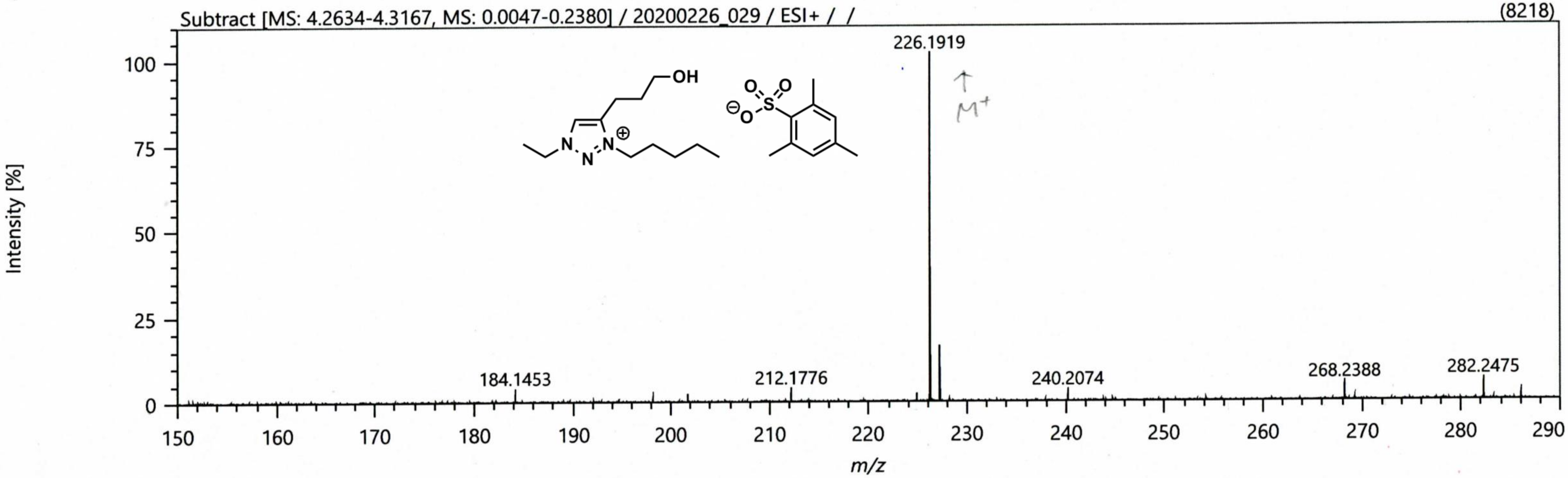

Elemental Composition

Parameters		Elements Set 1:								
		Symbol	C	H	O	Na	N	S	Ni	F
Tolerance:	±10.00 ppm	Min	0	0	1	0	3	0	0	0
Electron:	Odd/Even	Max	1000	4000	1	0	3	0	0	0
Charge:	+1									
DBE:	-99.0 - 999.0									

Results

Mass	Formula	Calculated Mass	Mass Difference [mDa]	Mass Difference [ppm]	DBE
226.19188	C12 H24 N3 O	226.19139	0.49	2.15	2.5

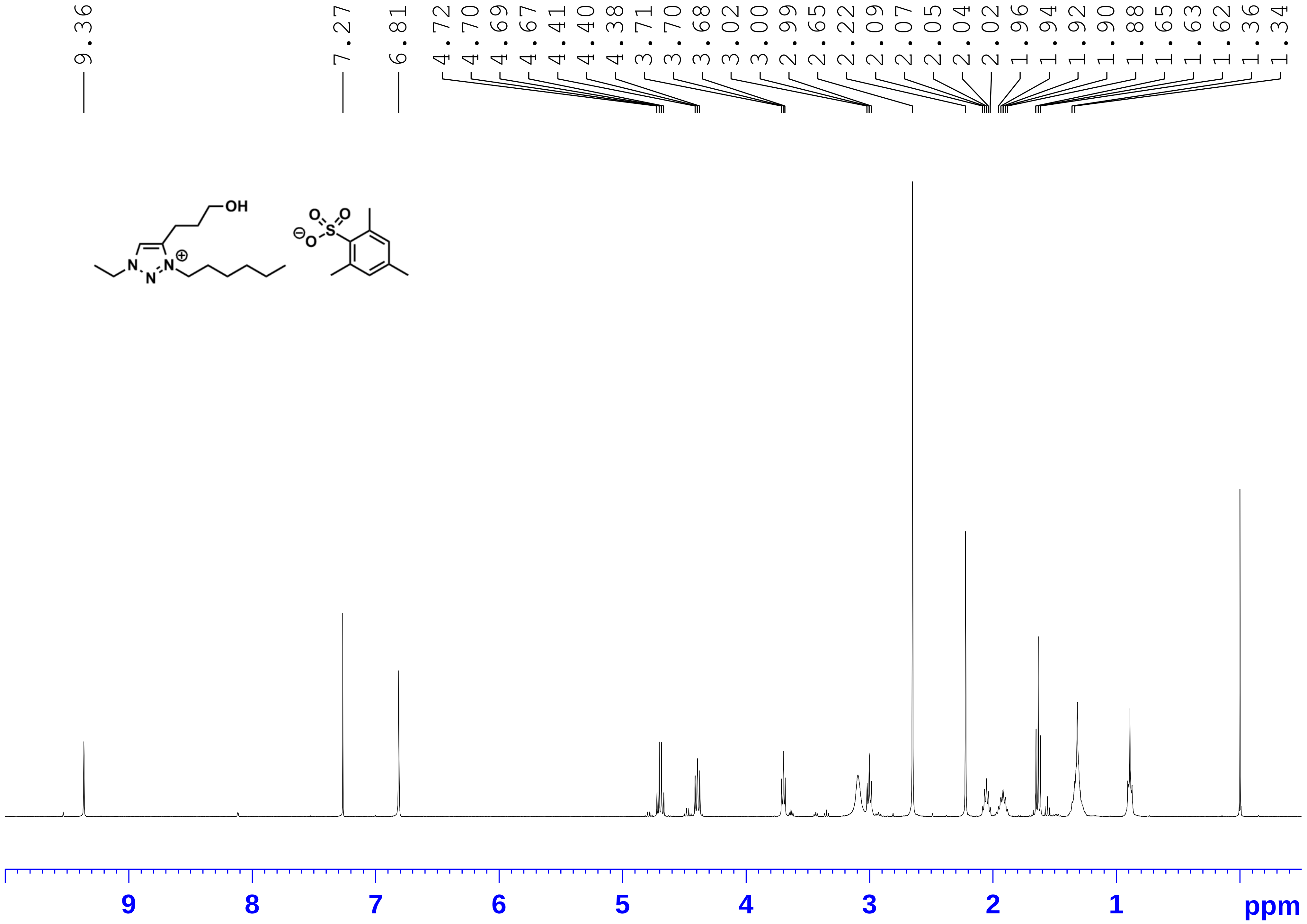
9.36
7.27
6.81
4.72
4.70
4.69
4.67
4.41
4.40
4.38
3.71
3.70
3.68
3.02
3.00
2.99
2.65
2.22
2.09
2.07
2.05
2.04
2.02
1.96
1.94
1.92
1.90
1.88
1.65
1.63
1.62
1.36
1.34
OH
N
N
N
O
S
O
O
9
8
7
6
5
4
3
2
1
ppm

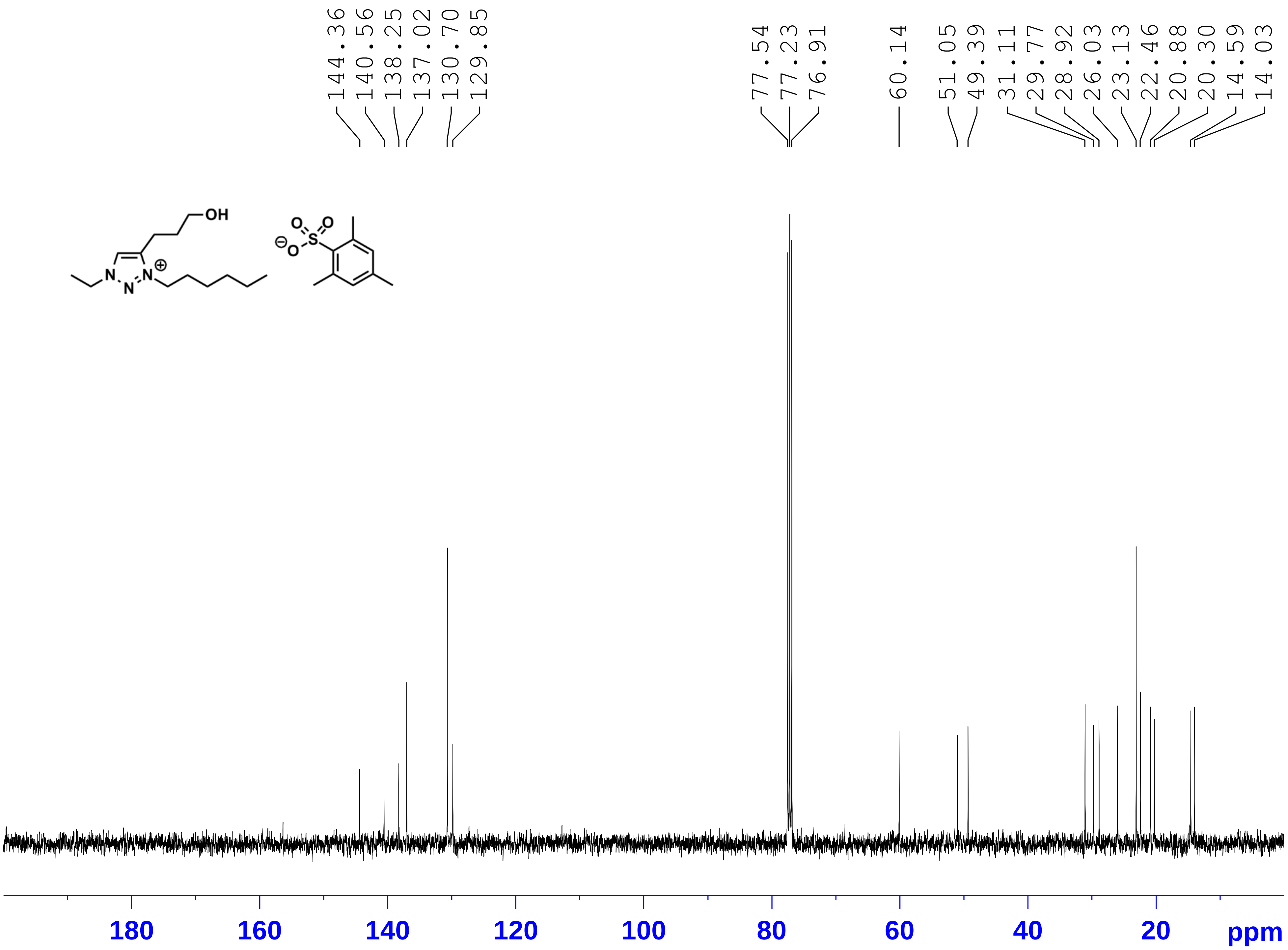
144.36
140.56
138.25
137.02
130.70
129.85
77.54
77.23
76.91
60.14
51.05
49.39
31.11
29.77
28.92
26.03
23.13
22.46
20.88
20.30
14.59
14.03
OH
O
S
O
O
N
N
N
180
160
140
120
100
80
60
40
20
ppm

Spectrum

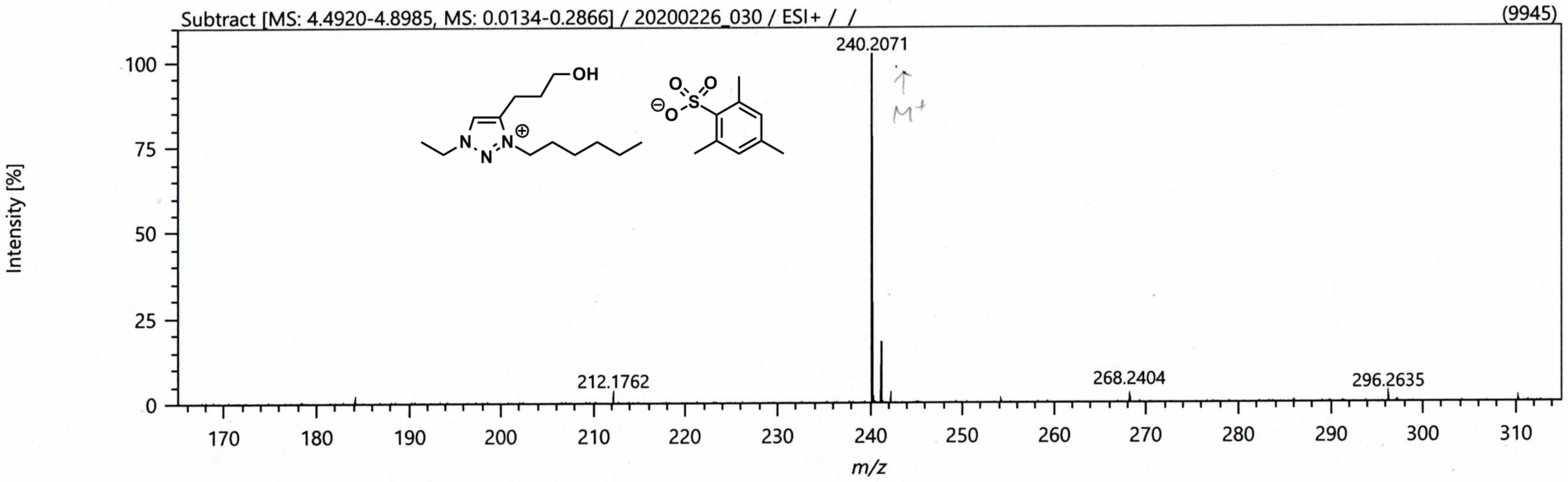

Elemental Composition

Parameters

Tolerance:	±10.00 ppm
Electron:	Odd/Even
Charge:	+1
DBE:	-99.0 - 999.0

Elements Set 1:

Symbol	C	H	O	Na	N	S	Ni	F
Min	0	0	1	0	3	0	0	0
Max	1000	4000	1	0	3	0	0	0

Results

Mass	Formula	Calculated Mass	Mass Difference [mDa]	Mass Difference [ppm]	DBE
240.20707	C13 H26 N3 O	240.20704	0.03	0.13	2.5

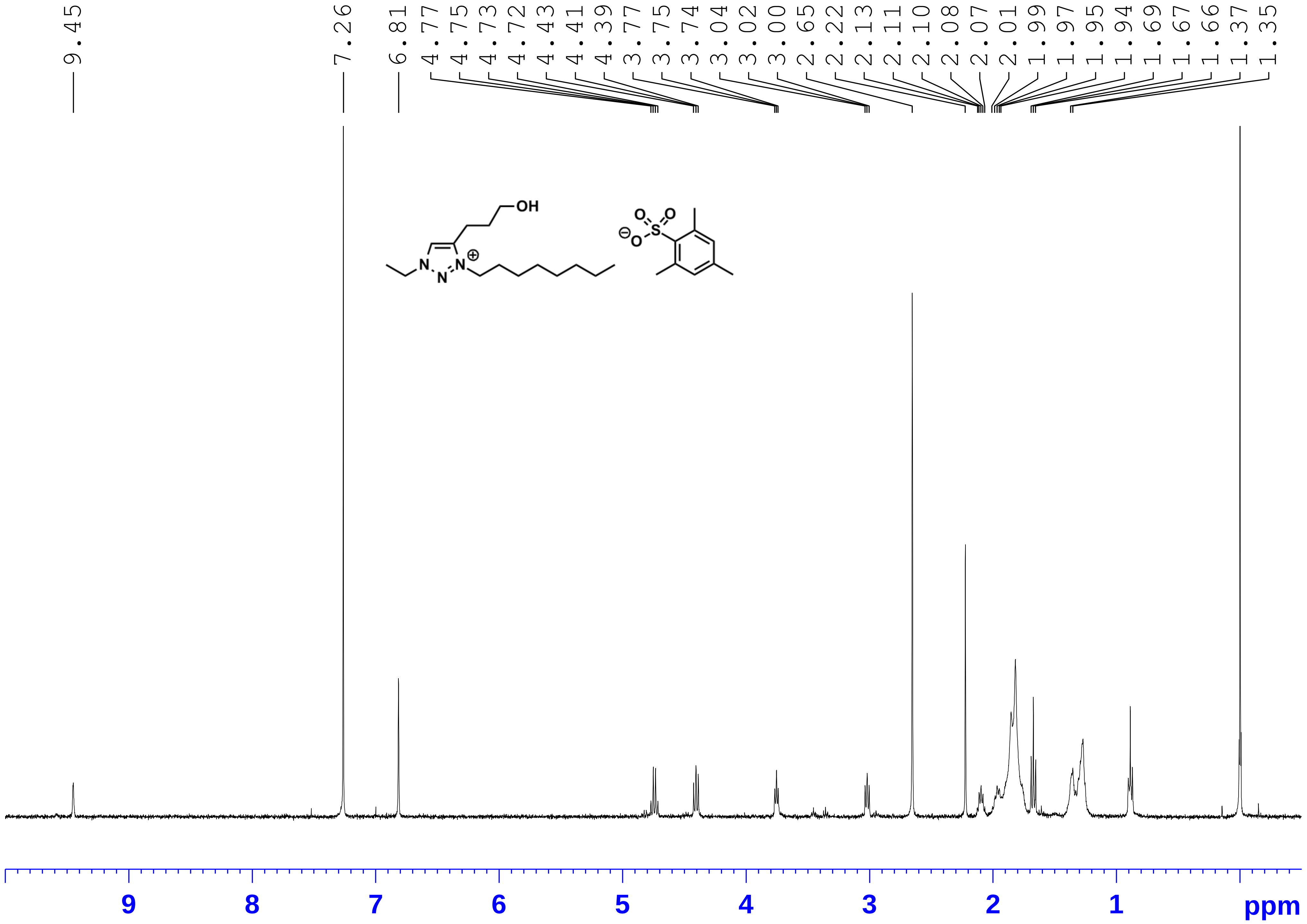
9.45
7.26
6.81
4.77
4.75
4.73
4.72
4.43
4.41
4.39
3.77
3.75
3.74
3.04
3.02
3.00
2.65
2.22
2.13
2.11
2.10
2.08
2.07
2.01
1.99
1.97
1.95
1.94
1.69
1.67
1.66
1.37
1.35
OH
N
N
N
O
O
S
O
9
8
7
6
5
4
3
2
1
ppm

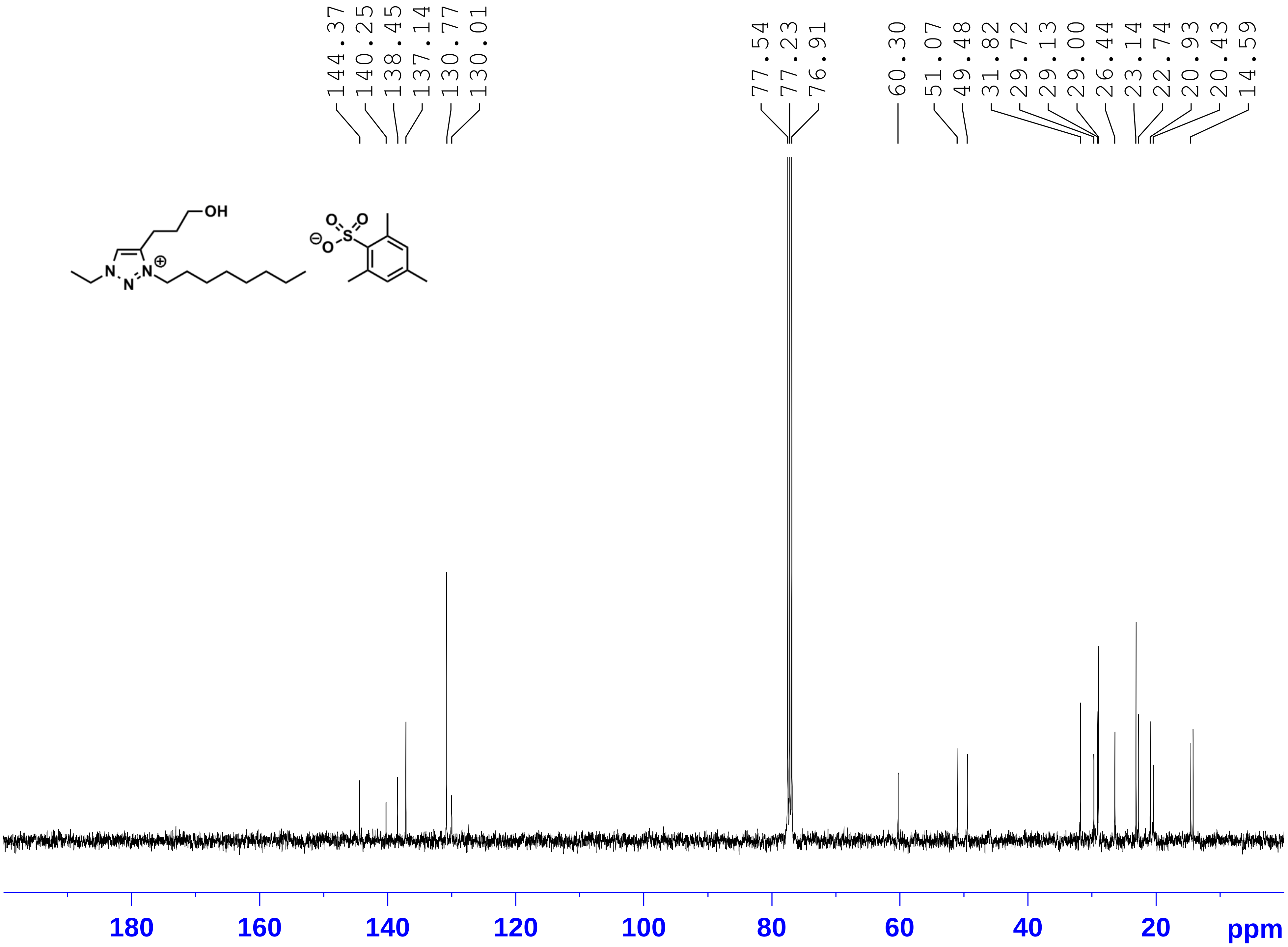
144.37
140.25
138.45
137.14
130.77
130.01
77.54
77.23
76.91
60.30
51.07
49.48
31.82
29.72
29.13
29.00
26.44
23.14
22.74
20.93
20.43
14.59
OH
N
N
N
O
O
S
O
180
160
140
120
100
80
60
40
20
ppm

Spectrum

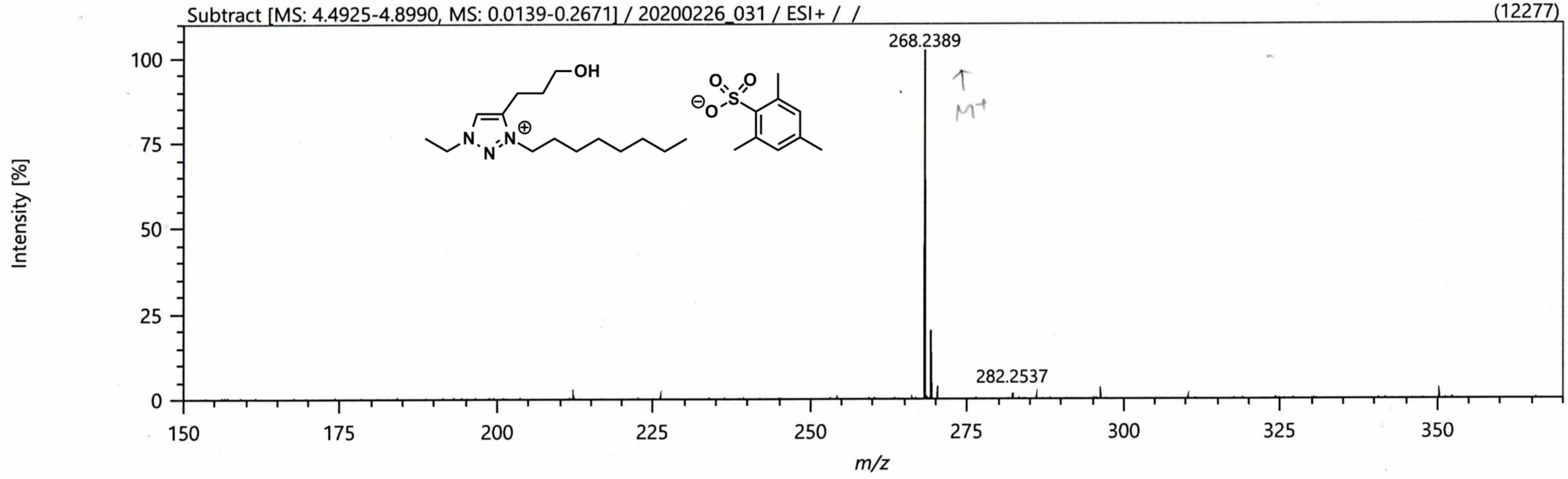

Elemental Composition

Parameters	
Tolerance:	±10.00 ppm
Electron:	Odd/Even
Charge:	+1
DBE:	-99.0 - 999.0

Elements Set 1:

Symbol	C	H	O	Na	N	S	Ni	F
Min	0	0	1	0	3	0	0	0
Max	1000	4000	1	0	3	0	0	0

Results

Mass	Formula	Calculated Mass	Mass Difference [mDa]	Mass Difference [ppm]	DBE
268.23893	C15 H30 N3 O	268.23834	0.59	2.19	2.5

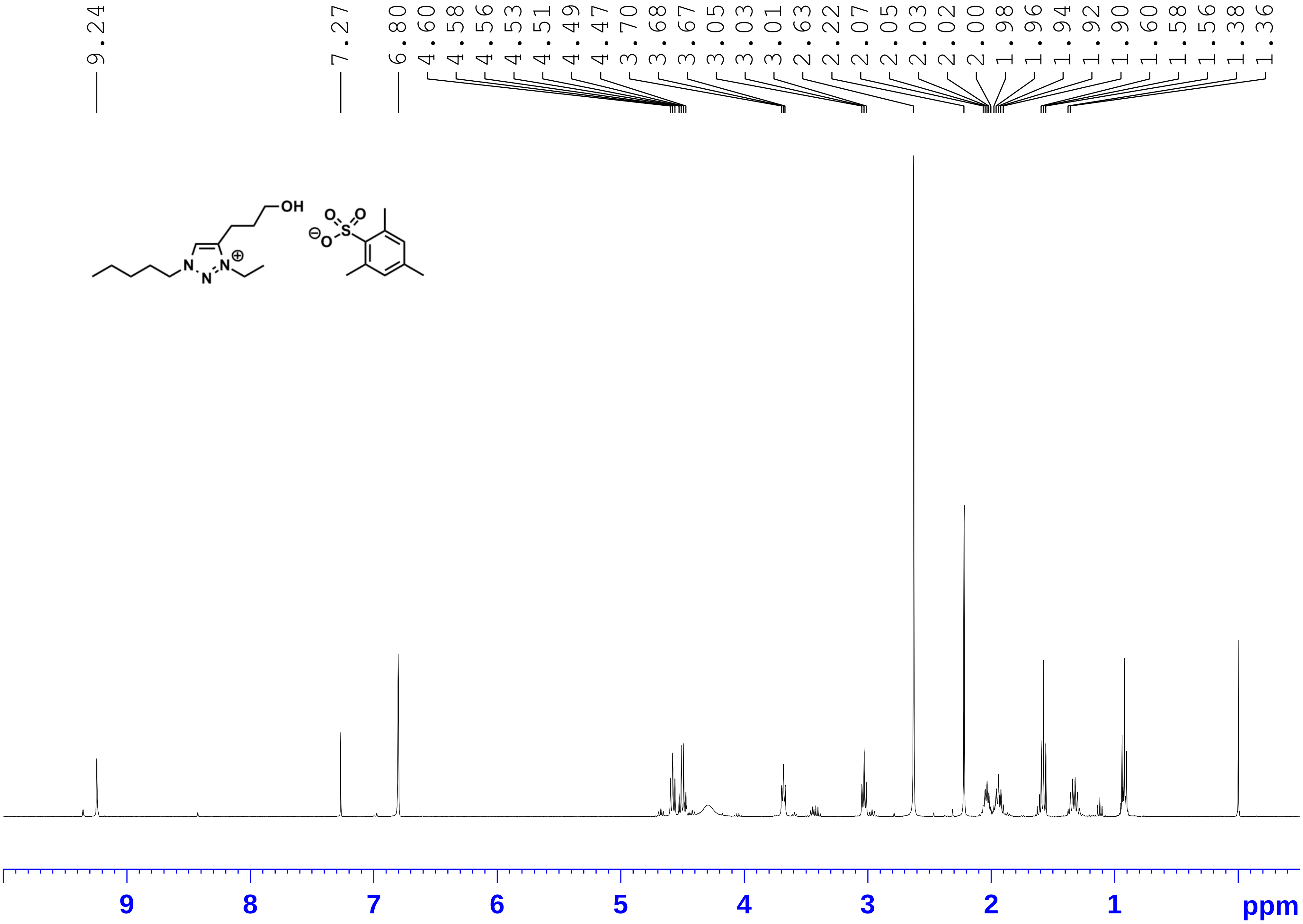
9.24
7.27
6.80
4.60
4.58
4.56
4.53
4.51
4.49
4.47
3.70
3.68
3.67
3.05
3.03
3.01
2.63
2.22
2.07
2.05
2.03
2.02
2.00
1.98
1.96
1.94
1.92
1.90
1.60
1.58
1.56
1.38
1.36
OH
9
8
7
6
5
4
3
2
1
ppm

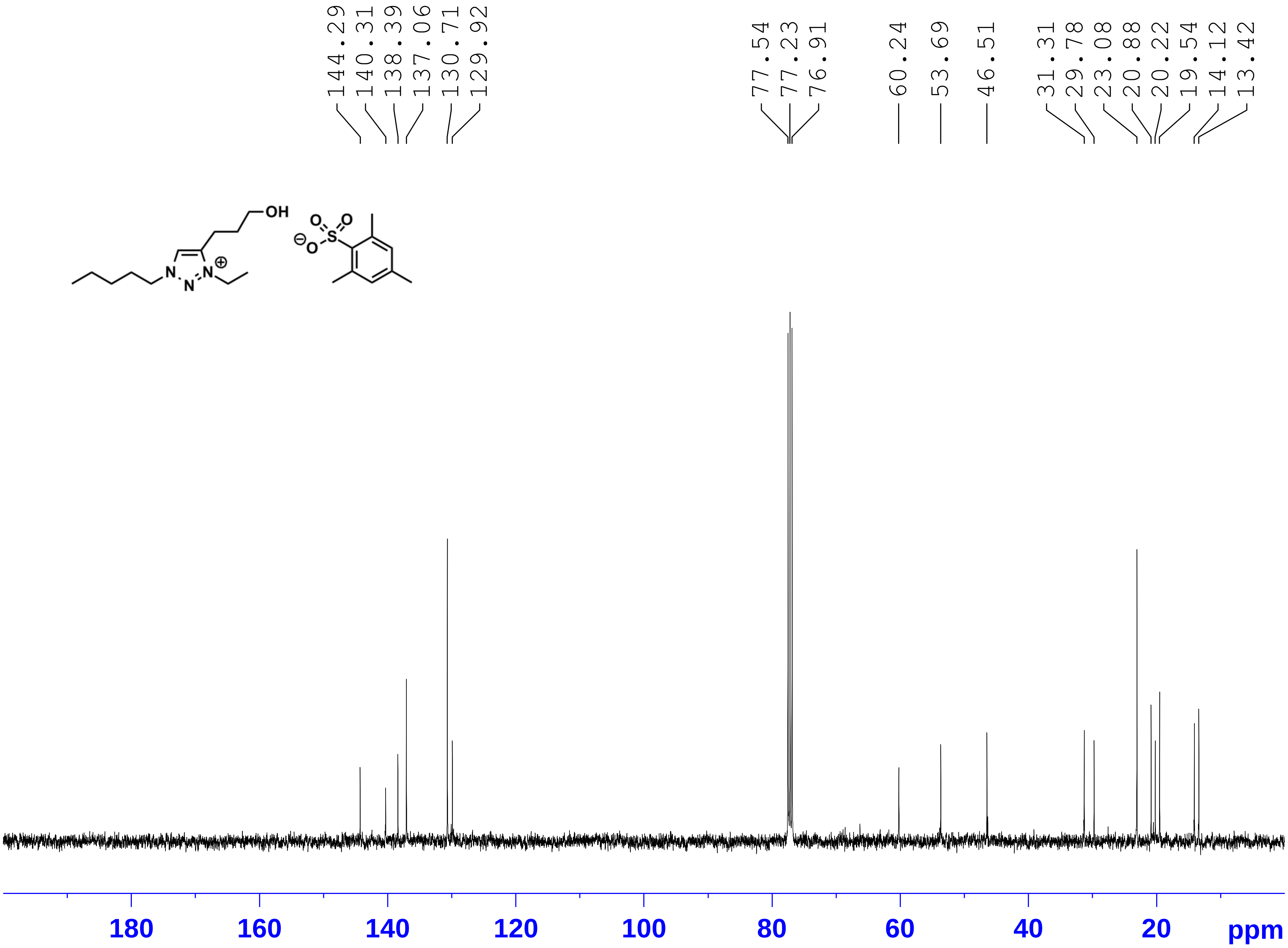

144.29
140.31
138.39
137.06
130.71
129.92
77.54
77.23
76.91
60.24
53.69
46.51
31.31
29.78
23.08
20.88
20.22
19.54
14.12
13.42
OH
N
N
N
O
O
S
O
180
160
140
120
100
80
60
40
20
ppm

Spectrum

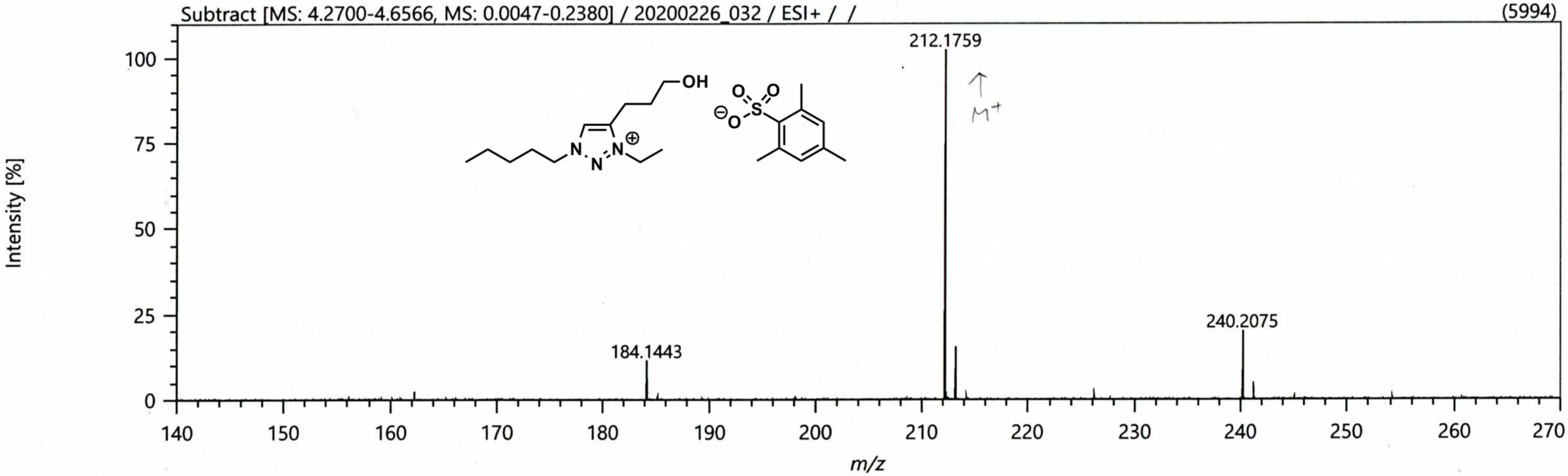

Elemental Composition

Parameters		Elements Set 1:								
Tolerance:	±10.00 ppm	Symbol	C	H	O	Na	N	S	Ni	F
Electron:	Odd/Even	Min	0	0	1	0	3	0	0	0
Charge:	+1	Max	1000	4000	1	0	3	0	0	0
DBE:	-99.0 - 999.0									

Results

Mass	Formula	Calculated Mass	Mass Difference [mDa]	Mass Difference [ppm]	DBE
212.17589	C11 H22 N3 O	212.17574	0.15	0.72	2.5

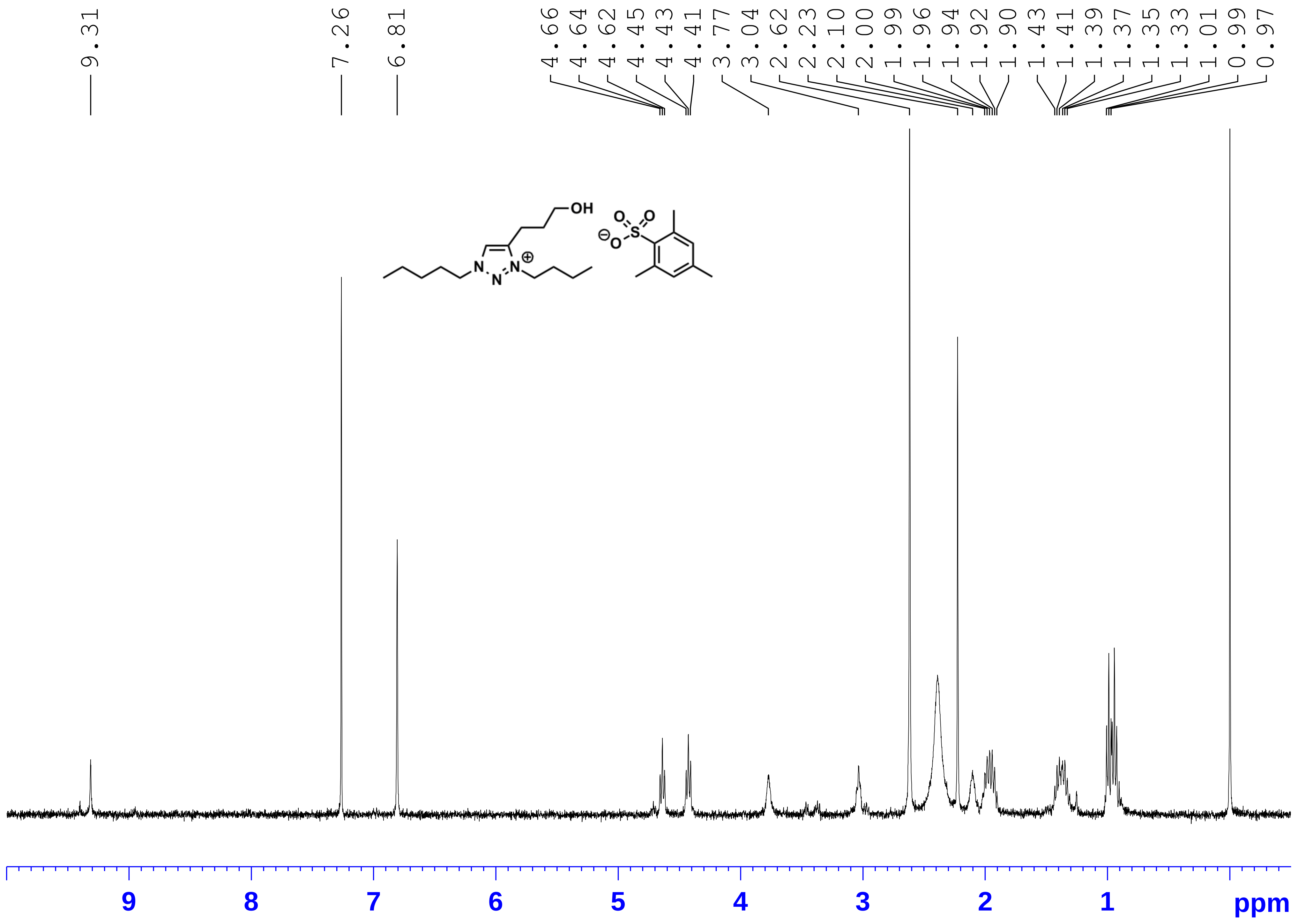
9.31
7.26
6.81
4.66
4.64
4.62
4.45
4.43
4.41
3.77
3.04
2.62
2.23
2.10
2.00
1.99
1.96
1.94
1.92
1.90
1.43
1.41
1.39
1.37
1.35
1.33
1.01
0.99
0.97
OH
O
O
S
O
N
N
N
9
8
7
6
5
4
3
2
1
ppm

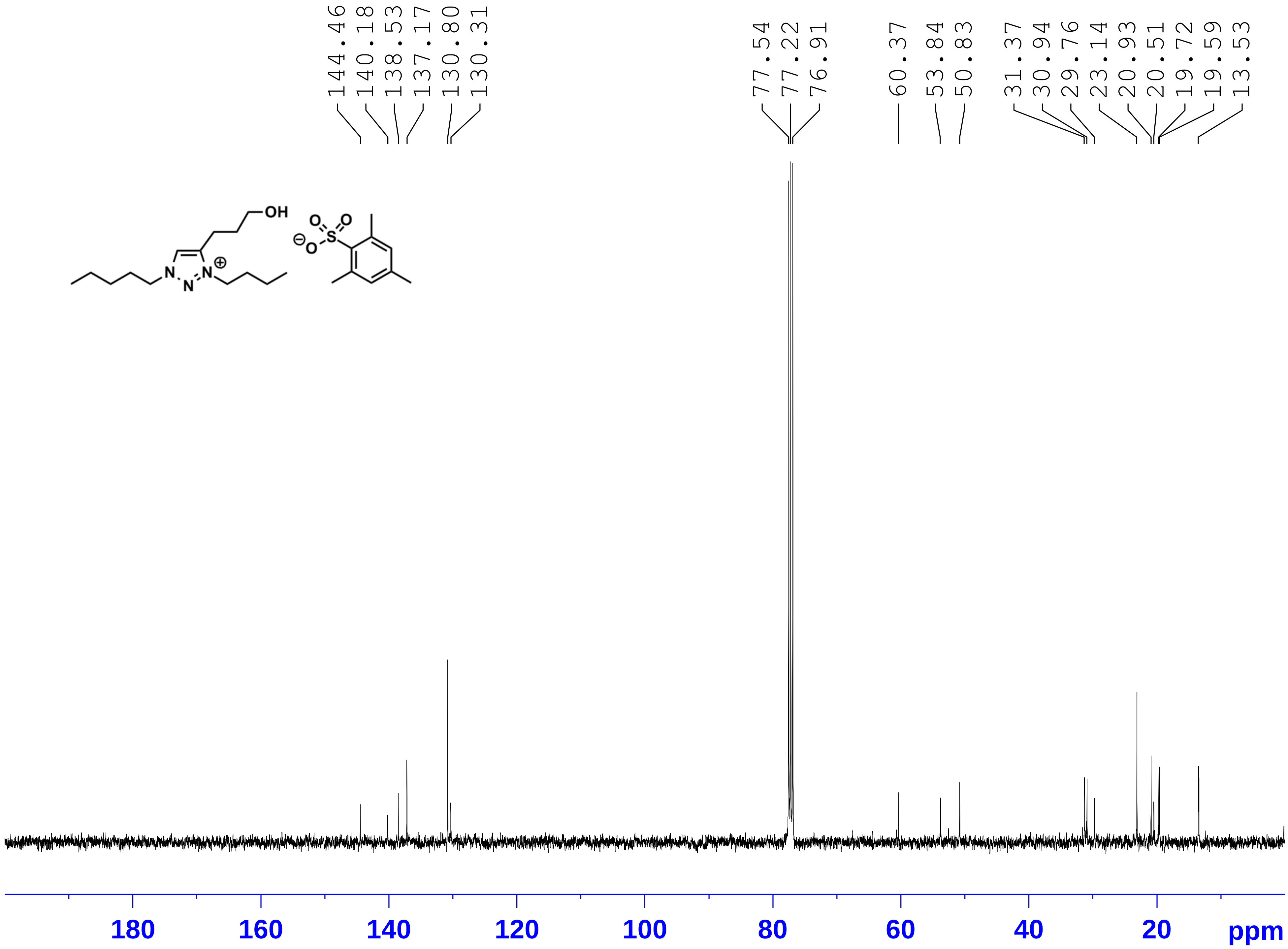
144.46
140.18
138.53
137.17
130.80
130.31
77.54
77.22
76.91
60.37
53.84
50.83
31.37
30.94
29.76
23.14
20.93
20.51
19.72
19.59
13.53
OH
180
160
140
120
100
80
60
40
20
ppm

Spectrum

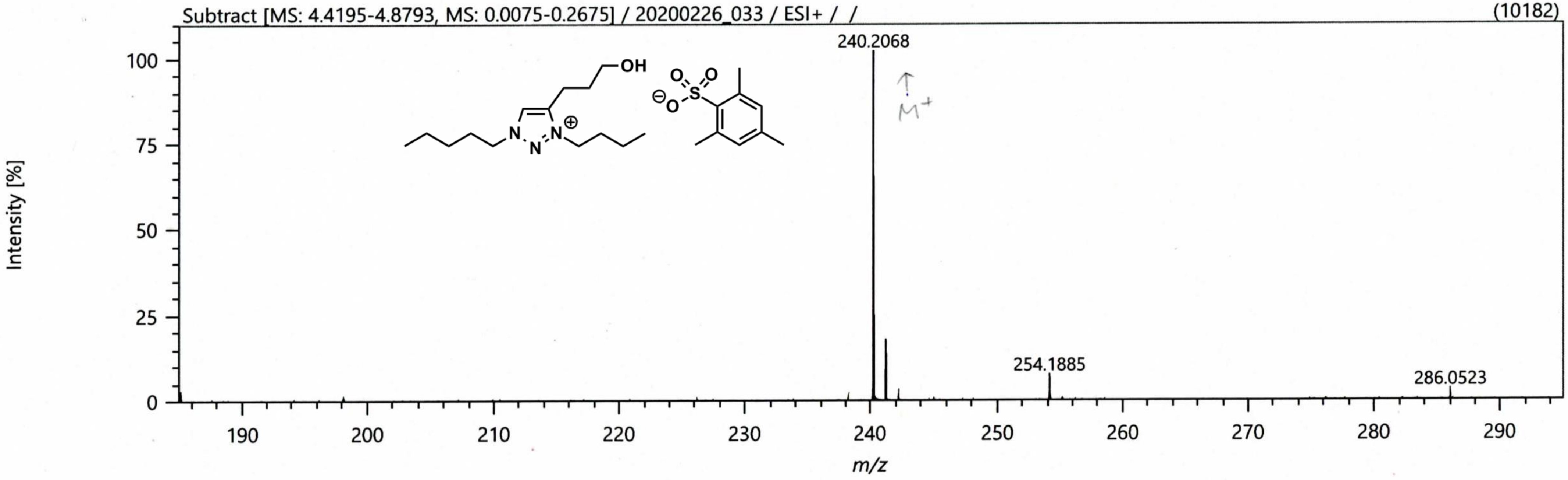

Elemental Composition

Parameters		Elements Set 1:								
Tolerance:	±10.00 ppm	Symbol	C	H	O	Na	N	S	Ni	F
Electron:	Odd/Even	Min	0	0	1	0	3	0	0	0
Charge:	+1	Max	1000	4000	1	0	3	0	0	0
DBE:	-99.0 - 999.0									

Results

Mass	Formula	Calculated Mass	Mass Difference [mDa]	Mass Difference [ppm]	DBE
240.20679	C13 H26 N3 O	240.20704	-0.24	-1.02	2.5

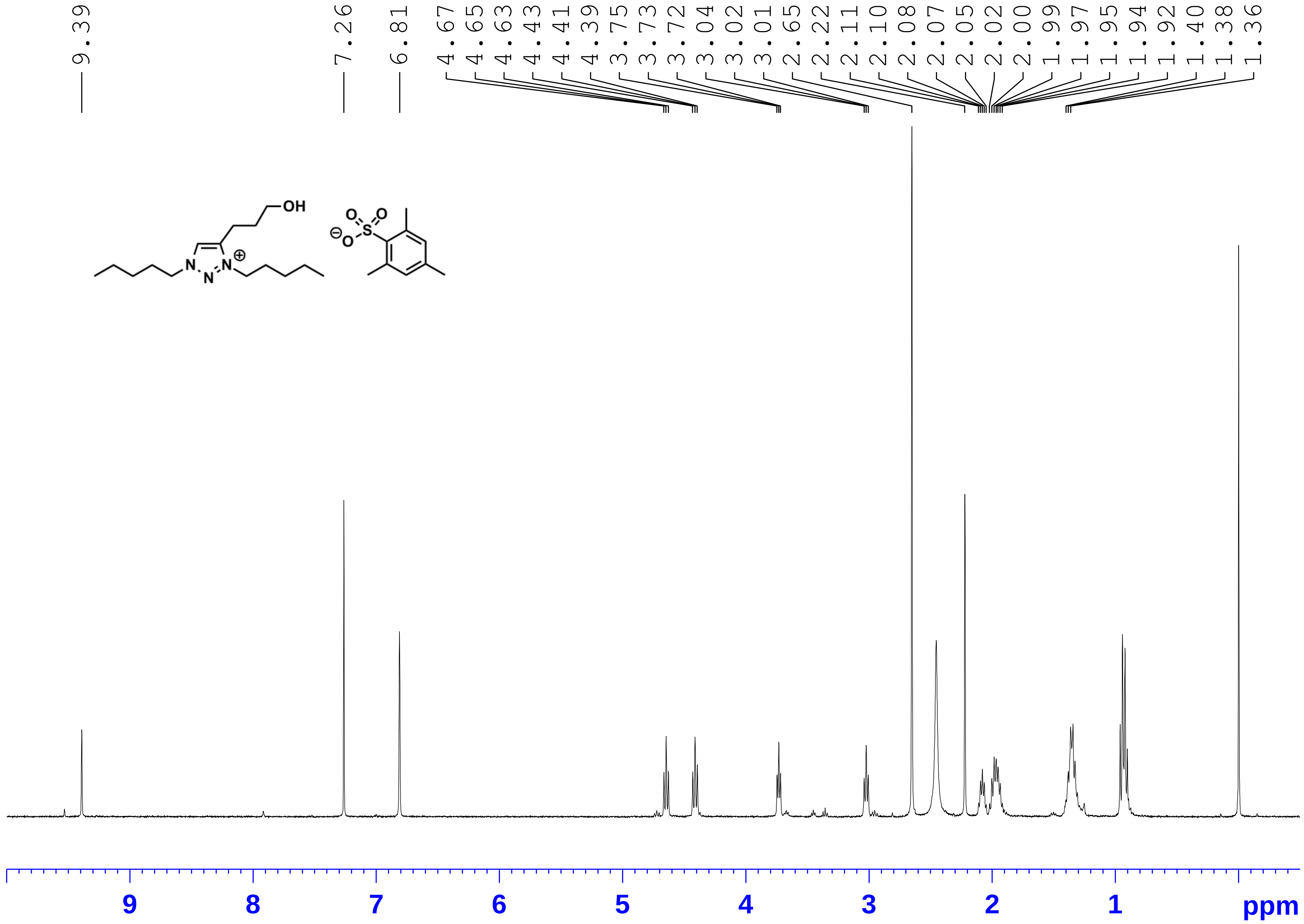
9.39
7.26
6.81
4.67
4.65
4.63
4.43
4.41
4.39
3.75
3.73
3.72
3.04
3.02
3.01
2.65
2.22
2.11
2.10
2.08
2.07
2.05
2.02
2.00
1.99
1.97
1.95
1.94
1.92
1.40
1.38
1.36
OH
N
N
N
O
O
S
O
9
8
7
6
5
4
3
2
1
ppm

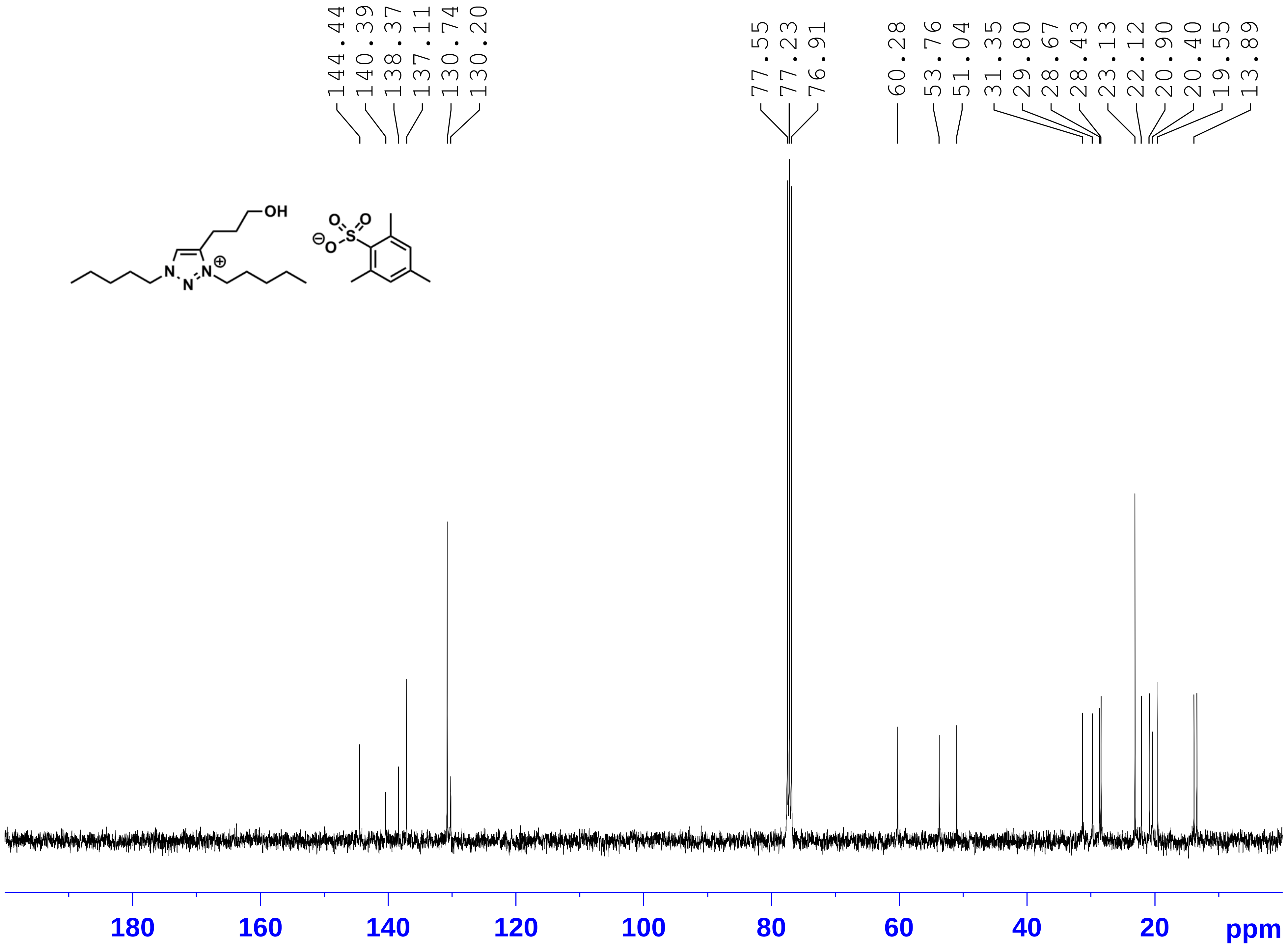
144.44
140.39
138.37
137.11
130.74
130.20
77.55
77.23
76.91
60.28
53.76
51.04
31.35
29.80
28.67
28.43
23.13
22.12
20.90
20.40
19.55
13.89
OH
O
S
O
O
N
N
N
180
160
140
120
100
80
60
40
20
ppm

Spectrum

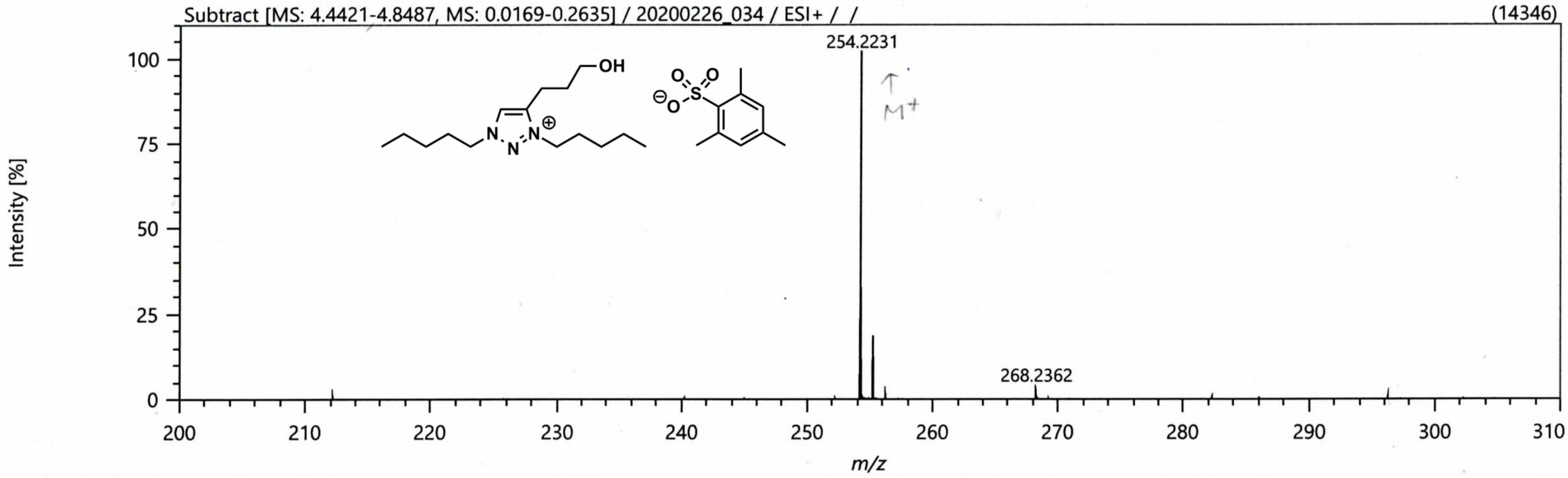

Elemental Composition

Parameters

Tolerance:	±10.00 ppm
Electron:	Odd/Even
Charge:	+1
DBE:	-99.0 - 999.0

Elements Set 1:

Symbol	C	H	O	Na	N	S	Ni	F
Min	0	0	1	0	3	0	0	0
Max	1000	4000	1	0	3	0	0	0

Results

Mass	Formula	Calculated Mass	Mass Difference [mDa]	Mass Difference [ppm]	DBE
254.22315	C14 H28 N3 O	254.22269	0.46	1.80	2.5

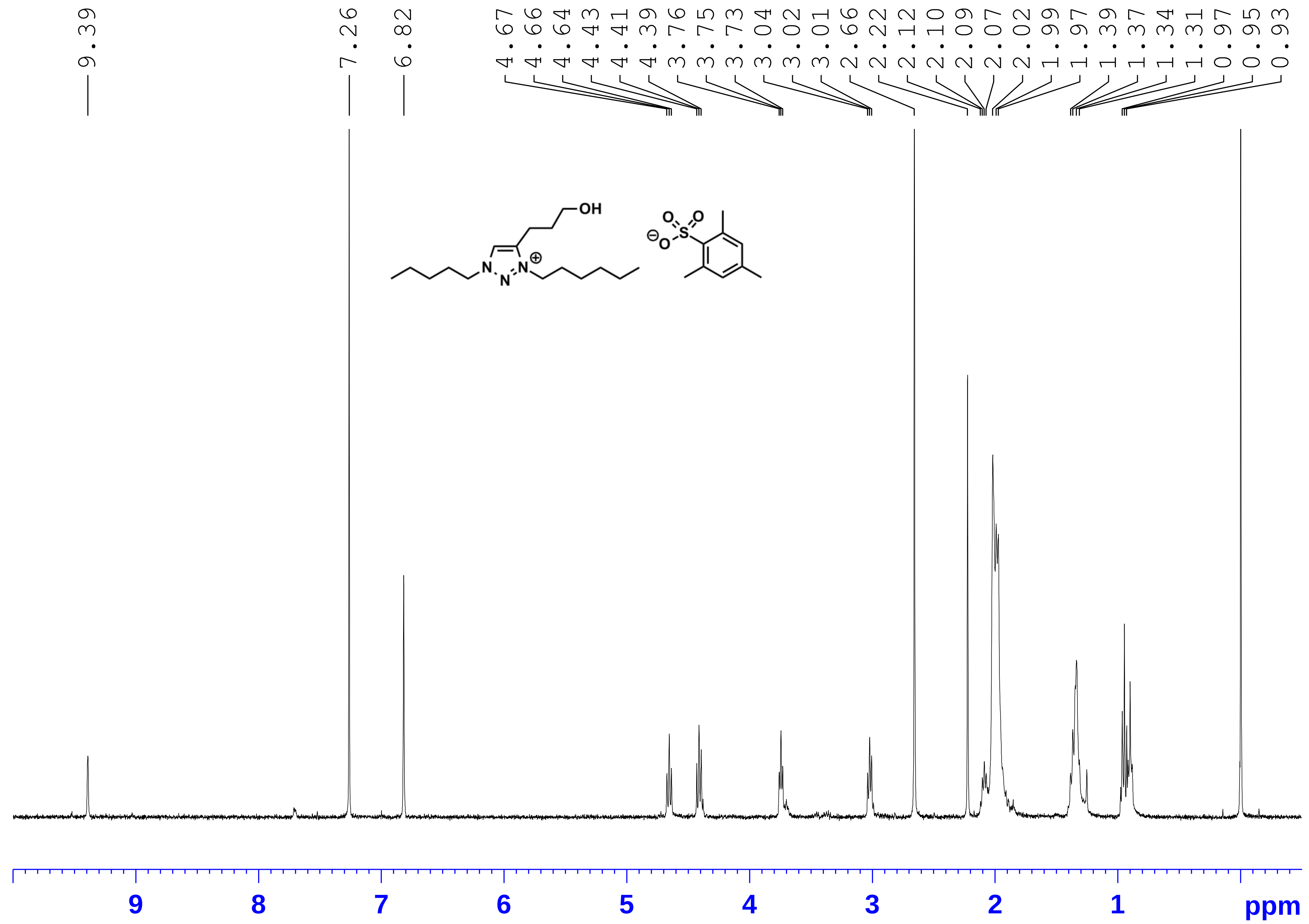
9.39
7.26
6.82
4.67
4.66
4.64
4.43
4.41
4.39
3.76
3.75
3.73
3.04
3.02
3.01
2.66
2.22
2.12
2.10
2.09
2.07
2.02
1.99
1.97
1.39
1.37
1.34
1.31
0.97
0.95
0.93
OH
O
S
O
O
N
N
N
9
8
7
6
5
4
3
2
1
ppm

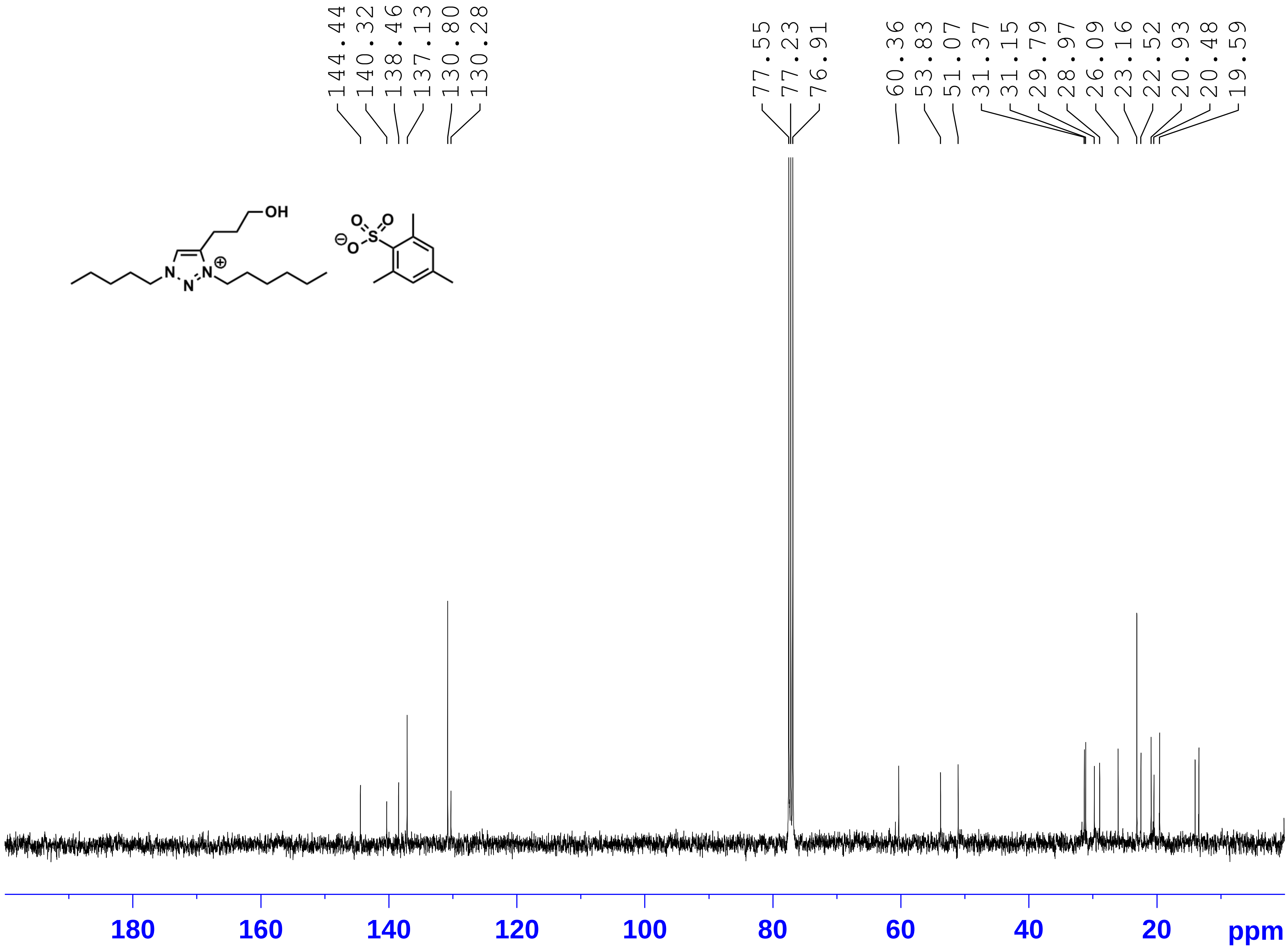
144.44
140.32
138.46
137.13
130.80
130.28
77.55
77.23
76.91
60.36
53.83
51.07
31.37
31.15
29.79
28.97
26.09
23.16
22.52
20.93
20.48
19.59
OH
N
N
N
O
S
O
O
180
160
140
120
100
80
60
40
20
ppm

Spectrum

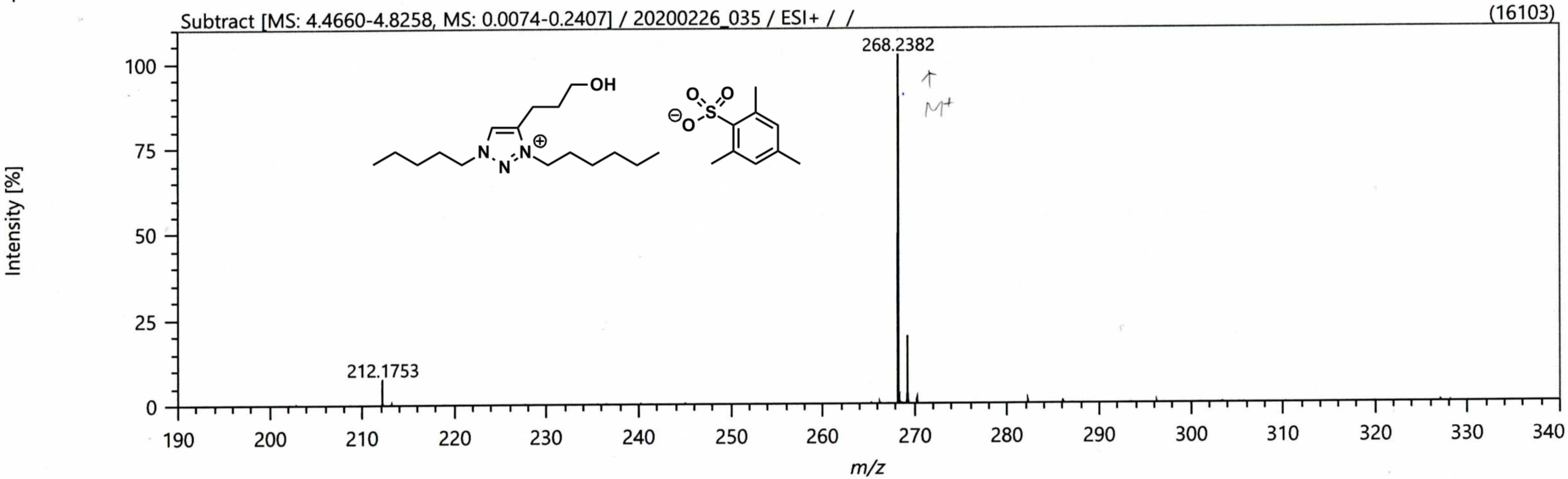

Elemental Composition

Parameters

Tolerance:	±10.00 ppm
Electron:	Odd/Even
Charge:	+1
DBE:	-99.0 - 999.0

Elements Set 1:

Symbol	C	H	O	Na	N	S	Ni	F
Min	0	0	1	0	3	0	0	0
Max	1000	4000	1	0	3	0	0	0

Results

Mass	Formula	Calculated Mass	Mass Difference [mDa]	Mass Difference [ppm]	DBE
268.23824	C15 H30 N3 O	268.23834	-0.10	-0.36	2.5

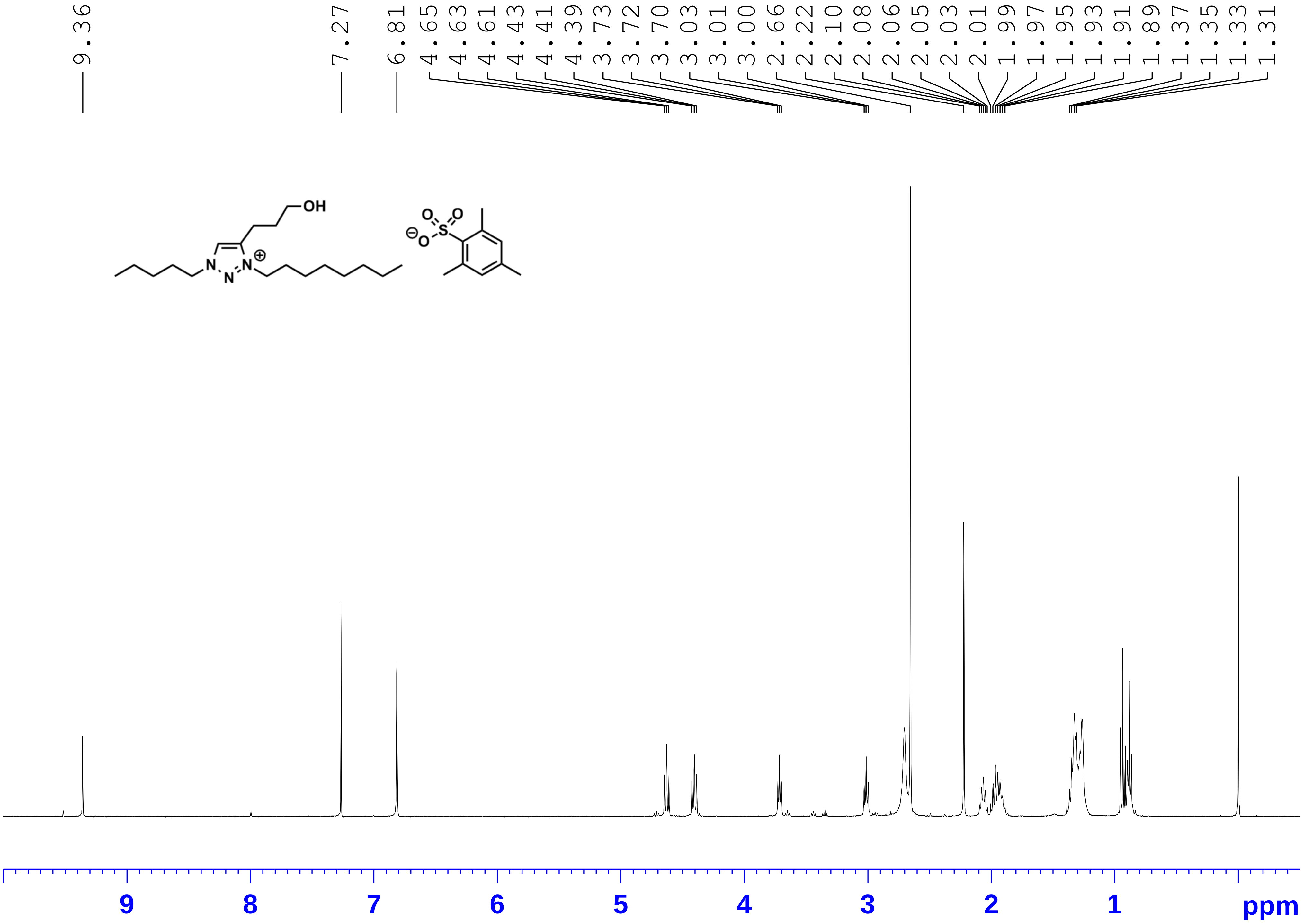

9.36
7.27
6.81
4.65
4.63
4.61
4.43
4.41
4.39
3.73
3.72
3.70
3.03
3.01
3.00
2.66
2.22
2.10
2.08
2.06
2.05
2.03
2.01
1.99
1.97
1.95
1.93
1.91
1.89
1.37
1.35
1.33
1.31
OH
N
N
N
O
O
S
O
9
8
7
6
5
4
3
2
1
ppm

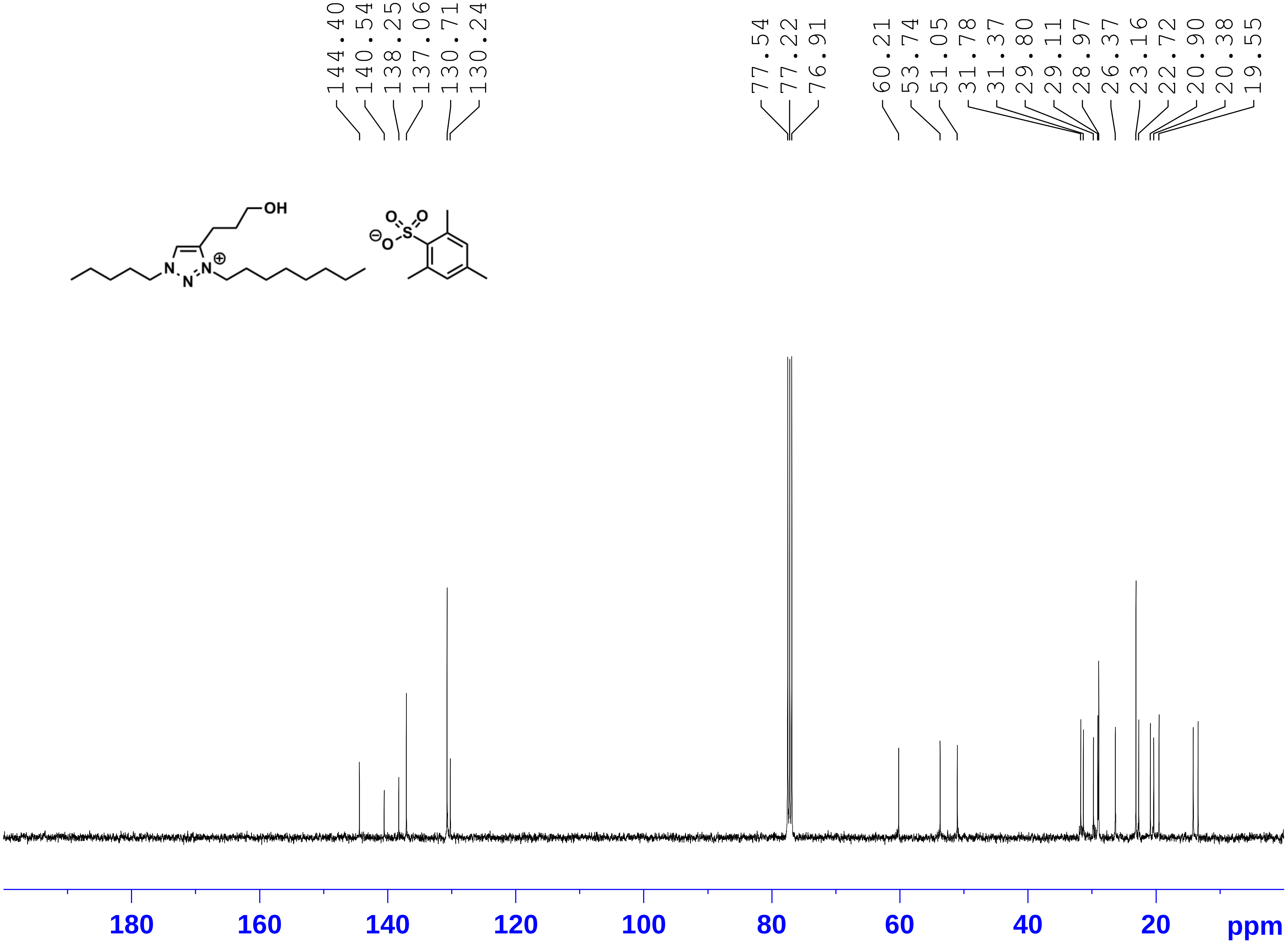
144.40
140.54
138.25
137.06
130.71
130.24
77.54
77.22
76.91
60.21
53.74
51.05
31.78
31.37
29.80
29.11
28.97
26.37
23.16
22.72
20.90
20.38
19.55
OH
N
N
N
O
O
O
S
180
160
140
120
100
80
60
40
20
ppm

Spectrum

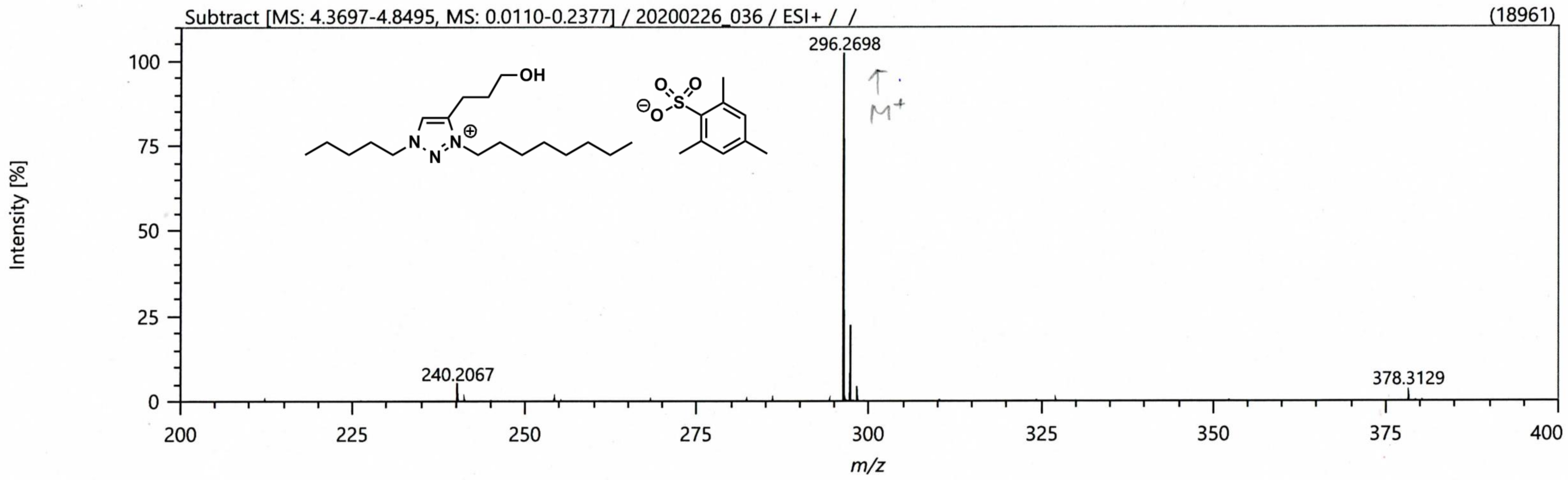

Elemental Composition

Parameters

Tolerance: ±10.00 ppm

Electron: Odd/Even

Charge: +1

DBE: -99.0 - 999.0

Elements Set 1:

Symbol	C	H	O	Na	N	S	Ni	F
Min	0	0	1	0	3	0	0	0
Max	1000	4000	1	0	3	0	0	0

Results

Mass	Formula	Calculated Mass	Mass Difference [mDa]	Mass Difference [ppm]	DBE
296.26982	C17 H34 N3 O	296.26964	0.18	0.62	2.5

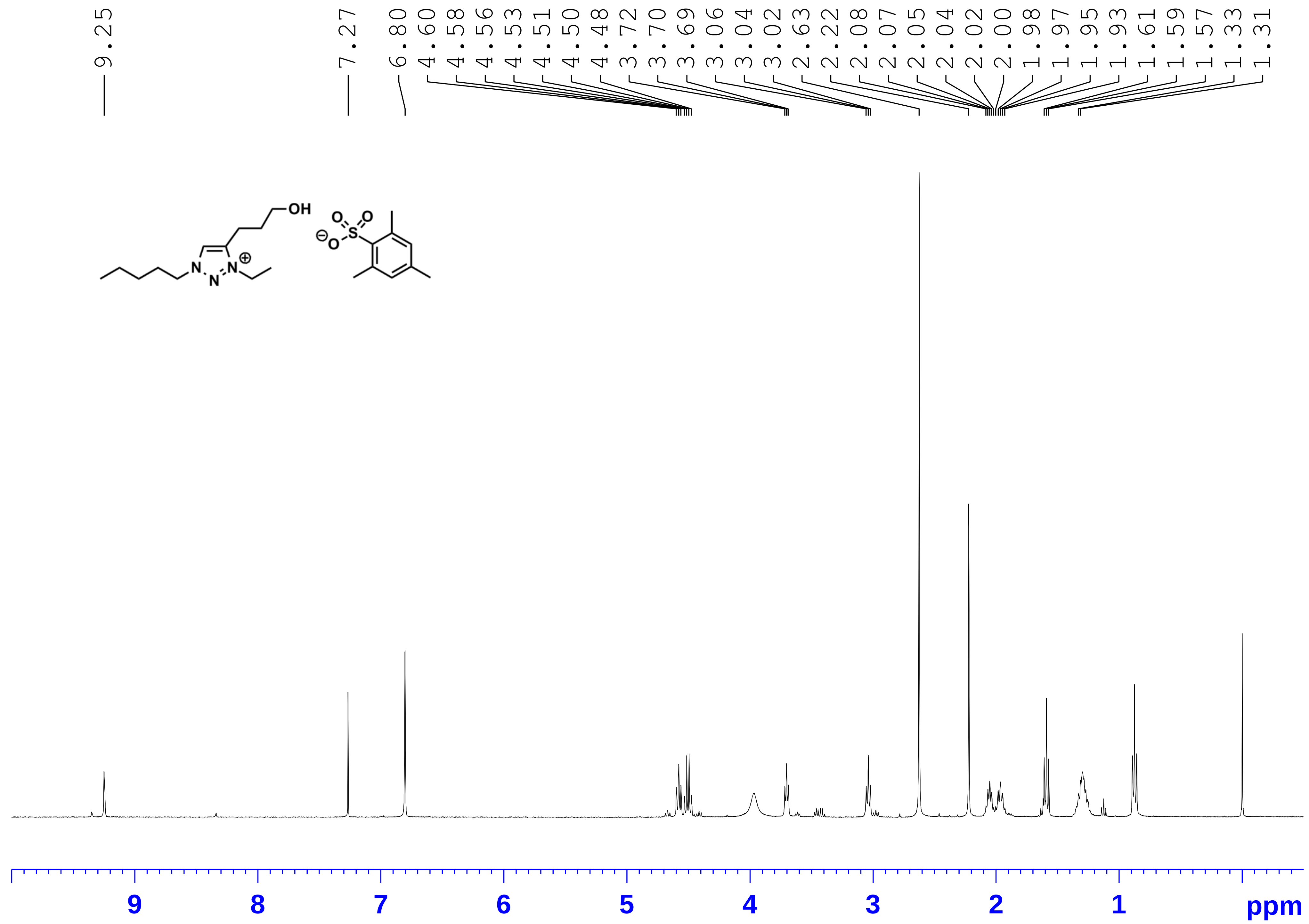
9.25
7.27
6.80
4.60
4.58
4.56
4.53
4.51
4.50
4.48
3.72
3.70
3.69
3.06
3.04
3.02
2.63
2.22
2.08
2.07
2.05
2.04
2.02
2.00
1.98
1.97
1.95
1.93
1.61
1.59
1.57
1.33
1.31
OH
N
N
N
O
O
S
O
9
8
7
6
5
4
3
2
1
ppm

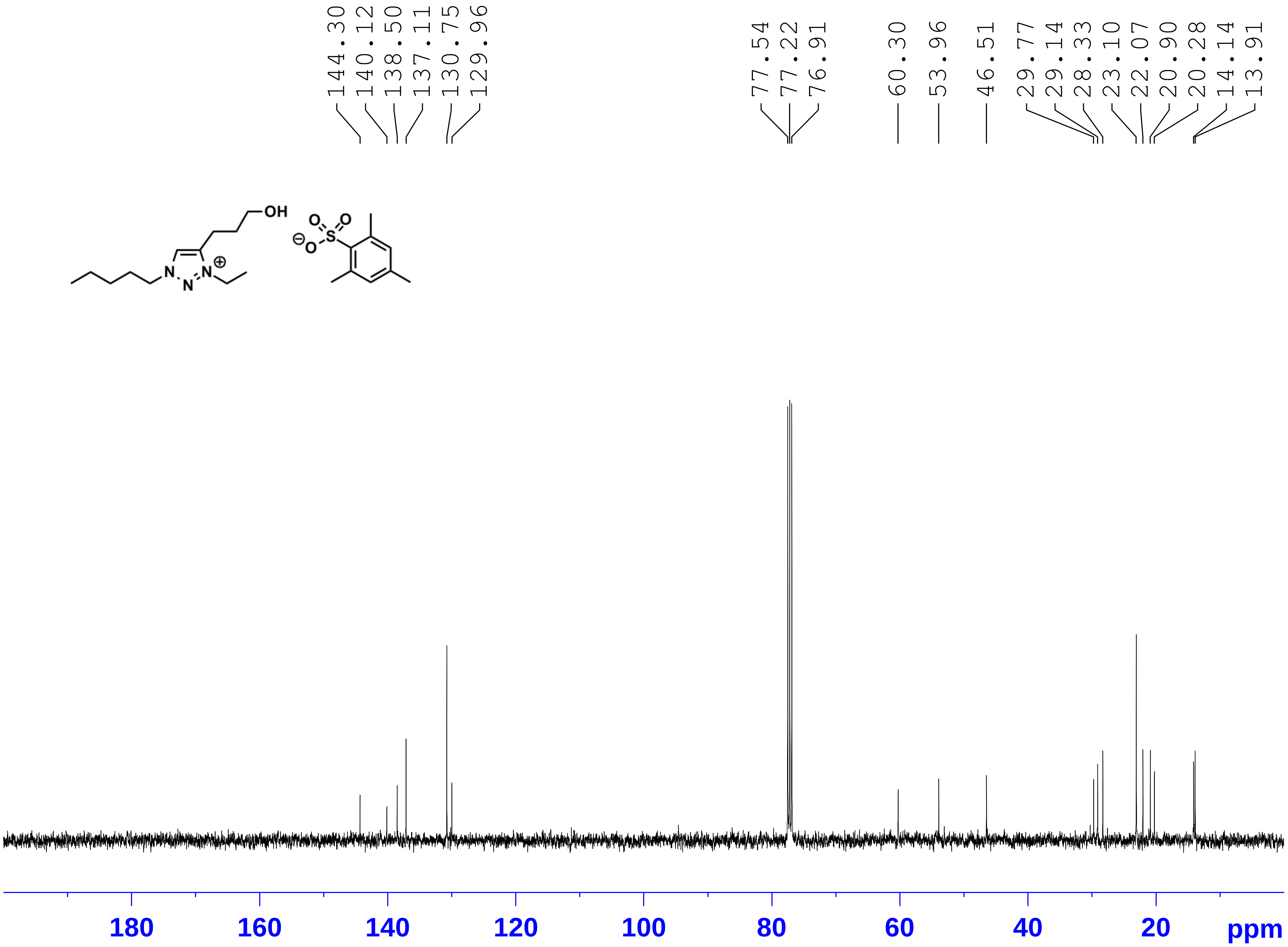
144.30
140.12
138.50
137.11
130.75
129.96
77.54
77.22
76.91
60.30
53.96
46.51
29.77
29.14
28.33
23.10
22.07
20.90
20.28
14.14
13.91
OH
O
O
S
O
N
N
N
180
160
140
120
100
80
60
40
20
ppm

Spectrum

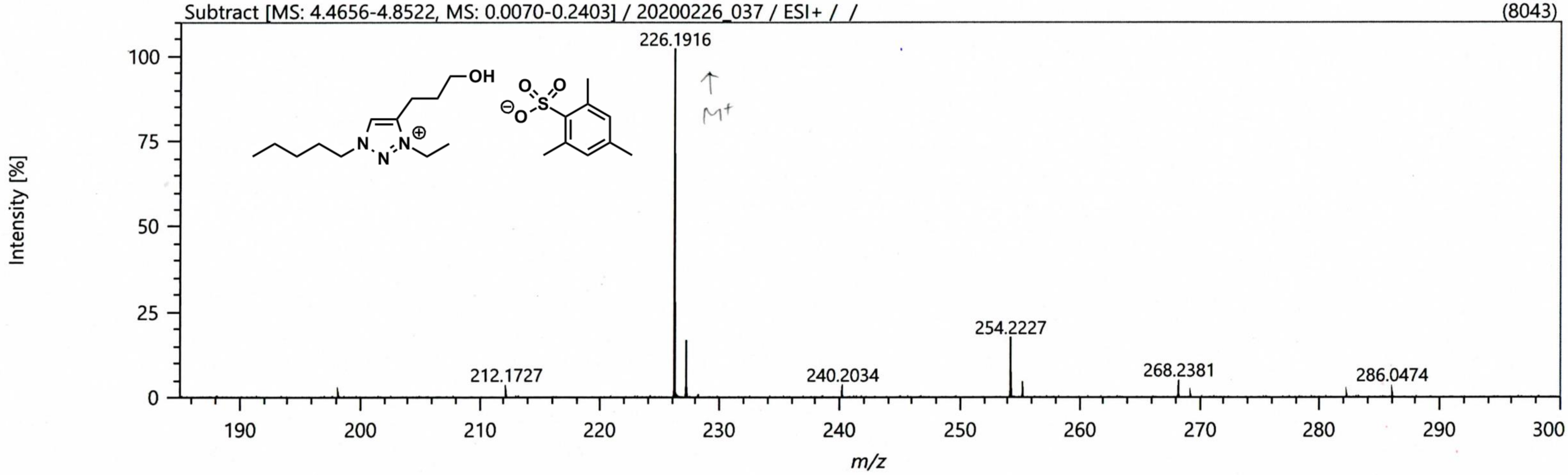

Elemental Composition

Parameters		Elements Set 1:								
		Symbol	C	H	O	Na	N	S	Ni	F
Tolerance:	±10.00 ppm	Min	0	0	1	0	3	0	0	0
Electron:	Odd/Even	Max	1000	4000	1	0	3	0	0	0
Charge:	+1									
DBE:	-99.0 - 999.0									

Results

Mass	Formula	Calculated Mass	Mass Difference [mDa]	Mass Difference [ppm]	DBE
226.19159	C12 H24 N3 O	226.19139	0.20	0.89	2.5

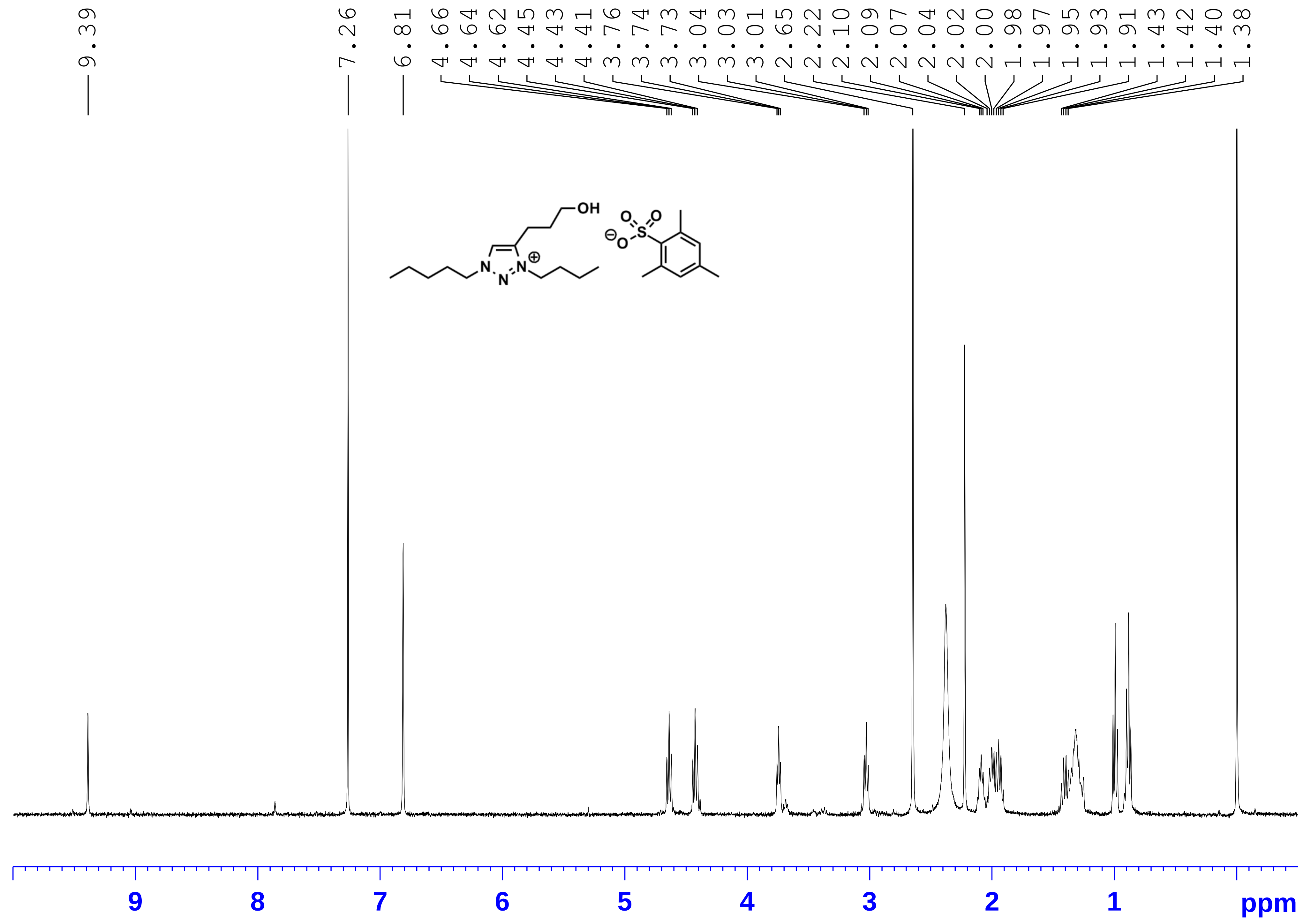

9.39
7.26
6.81
4.66
4.64
4.62
4.45
4.43
4.41
3.76
3.74
3.73
3.04
3.03
3.01
2.65
2.22
2.10
2.09
2.07
2.04
2.02
2.00
1.98
1.97
1.95
1.93
1.91
1.43
1.42
1.40
1.38
OH
N
N
N
O
S
O
O
9
8
7
6
5
4
3
2
1
ppm

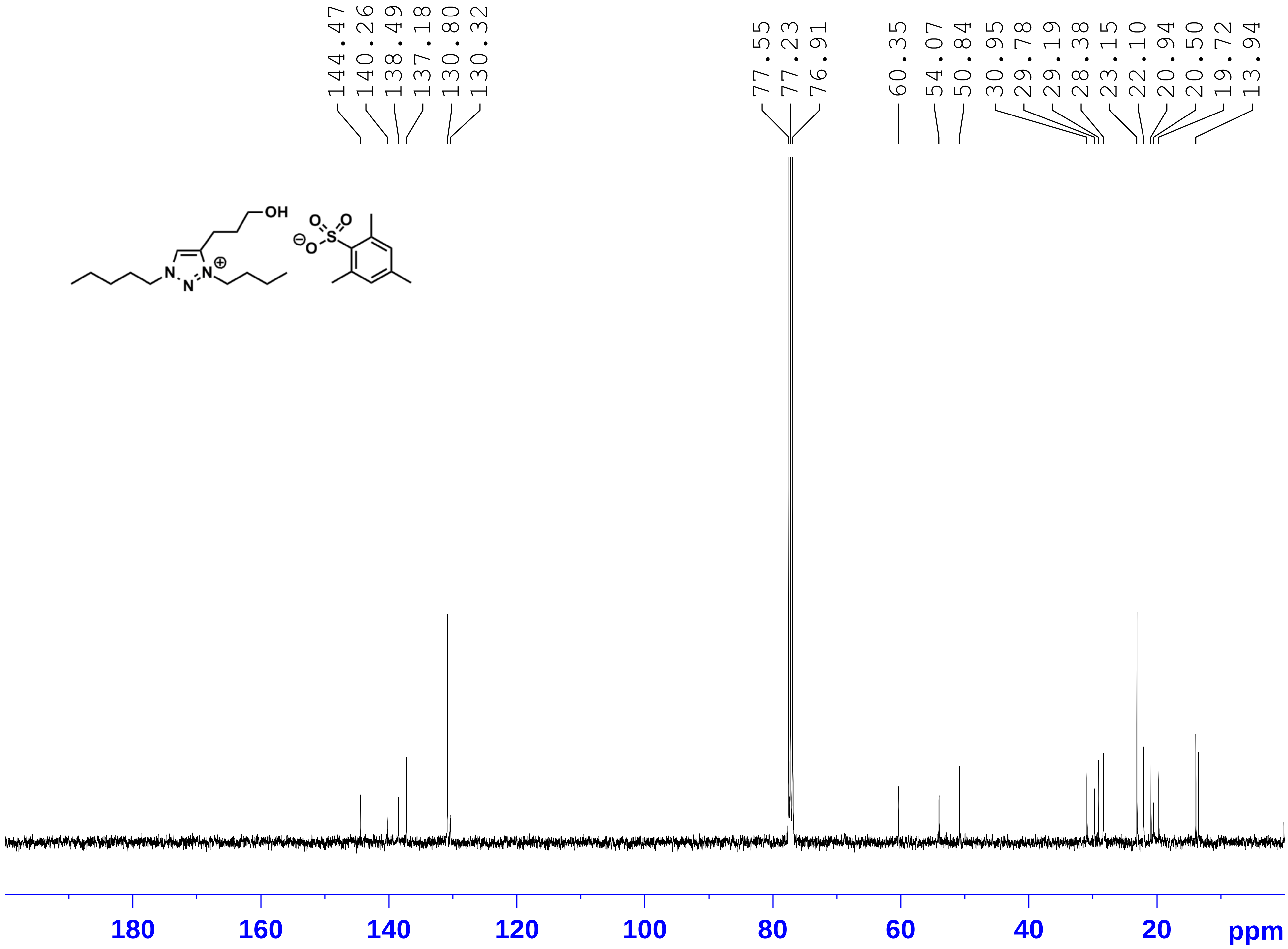
144.47
140.26
138.49
137.18
130.80
130.32
77.55
77.23
76.91
60.35
54.07
50.84
30.95
29.78
29.19
28.38
23.15
22.10
20.94
20.50
19.72
13.94
180
160
140
120
100
80
60
40
20
ppm

Spectrum

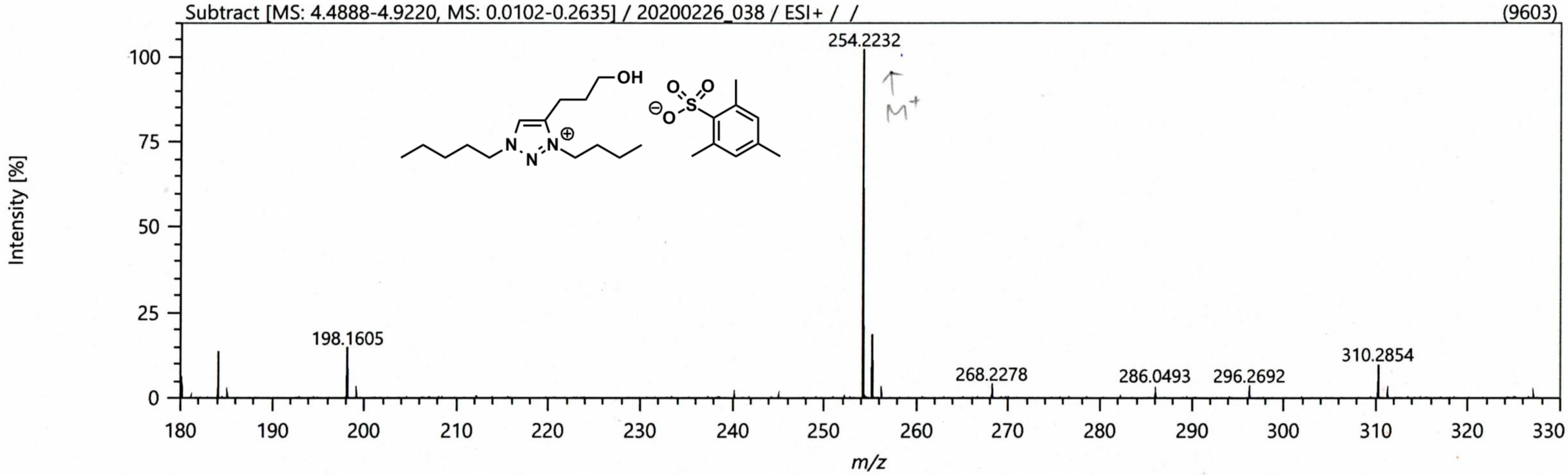

Elemental Composition

Parameters

Tolerance:	±10.00 ppm
Electron:	Odd/Even
Charge:	+1
DBE:	-99.0 - 999.0

Elements Set 1:

Symbol	C	H	O	Na	N	S	Ni	F
Min	0	0	1	0	3	0	0	0
Max	1000	4000	1	0	3	0	0	0

Results

Mass	Formula	Calculated Mass	Mass Difference [mDa]	Mass Difference [ppm]	DBE
254.22320	C14 H28 N3 O	254.22269	0.52	2.03	2.5

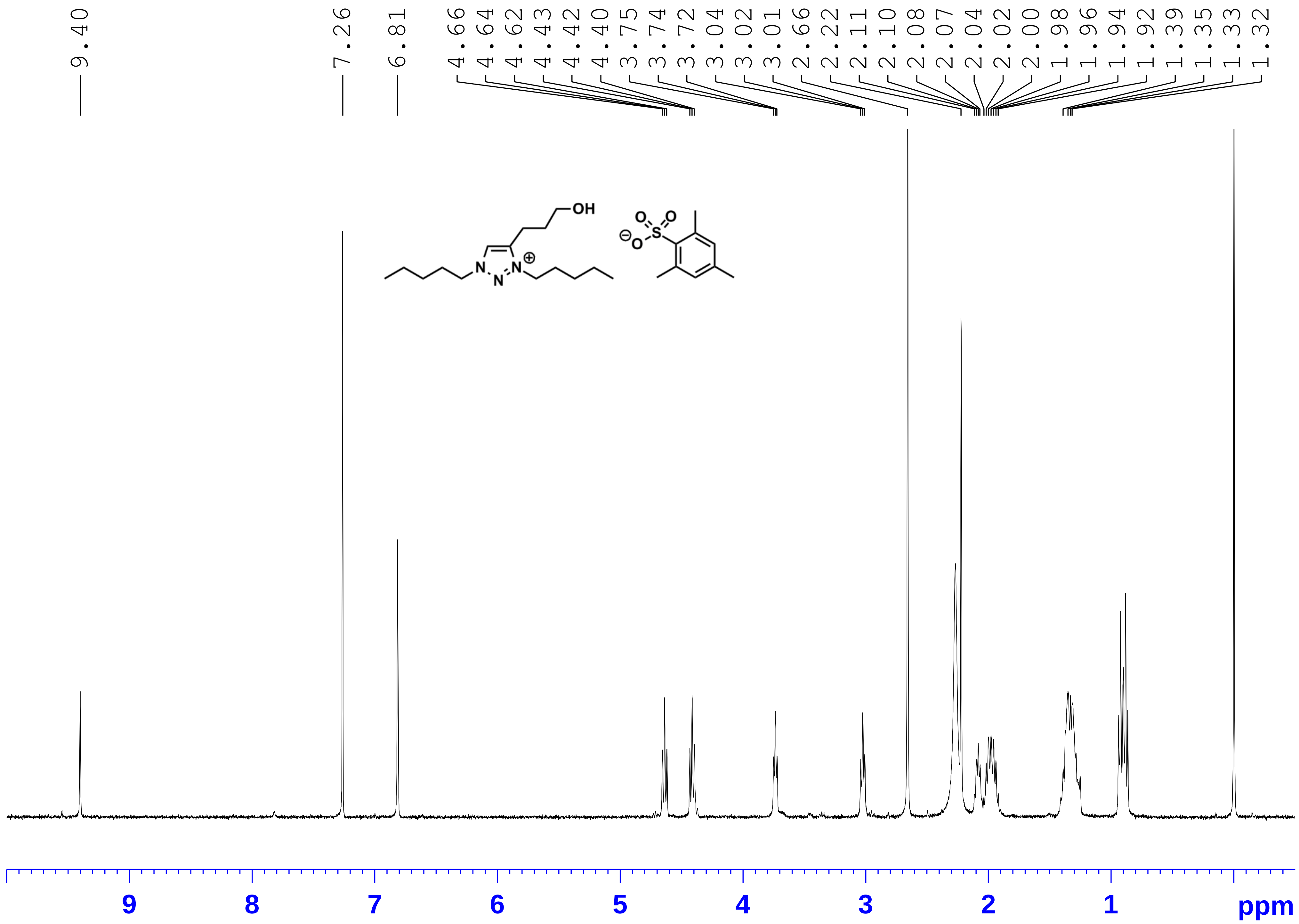
9.40
7.26
6.81
4.66
4.64
4.62
4.43
4.42
4.40
3.75
3.74
3.72
3.04
3.02
3.01
2.66
2.22
2.11
2.10
2.08
2.07
2.04
2.02
2.00
1.98
1.96
1.94
1.92
1.39
1.35
1.33
1.32
OH
N
N
N
⊕
O
O
S
⊖O
9
8
7
6
5
4
3
2
1
ppm

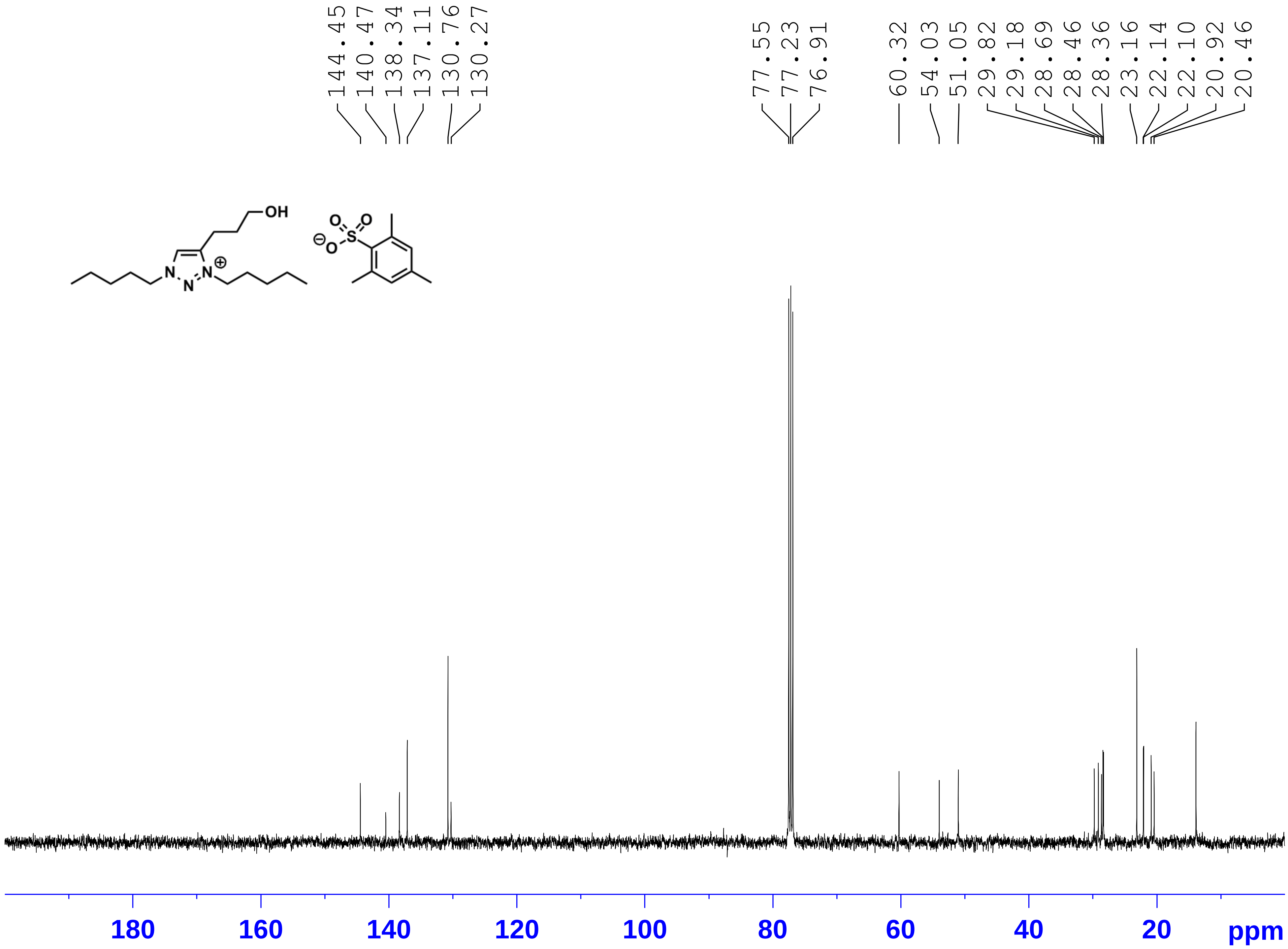
144.45
140.47
138.34
137.11
130.76
130.27
77.55
77.23
76.91
60.32
54.03
51.05
29.82
29.18
28.69
28.46
28.36
23.16
22.14
22.10
20.92
20.46
OH
N
N
N
O
O
O
S
180
160
140
120
100
80
60
40
20
ppm

Spectrum

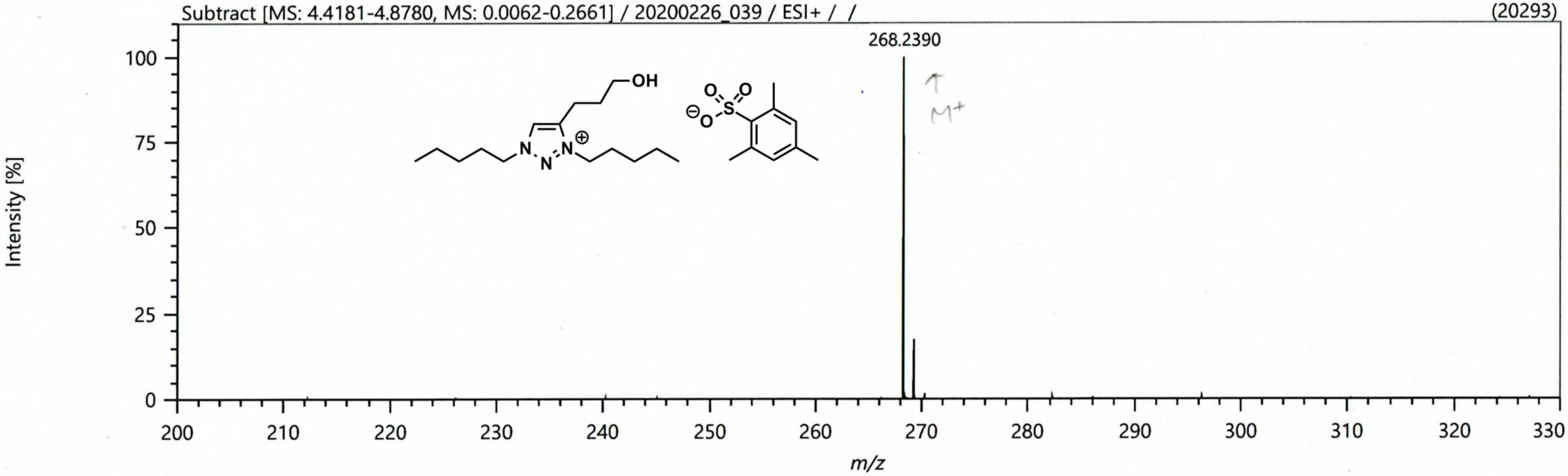

Elemental Composition

Parameters		Elements Set 1:								
Tolerance:	±10.00 ppm	Symbol	C	H	O	Na	N	S	Ni	F
Electron:	Odd/Even	Min	0	0	1	0	3	0	0	0
Charge:	+1	Max	1000	4000	1	0	3	0	0	0
DBE:	-99.0 - 999.0									

Results

Mass	Formula	Calculated Mass	Mass Difference [mDa]	Mass Difference [ppm]	DBE
268.23895	C15 H30 N3 O	268.23834	0.61	2.29	2.5

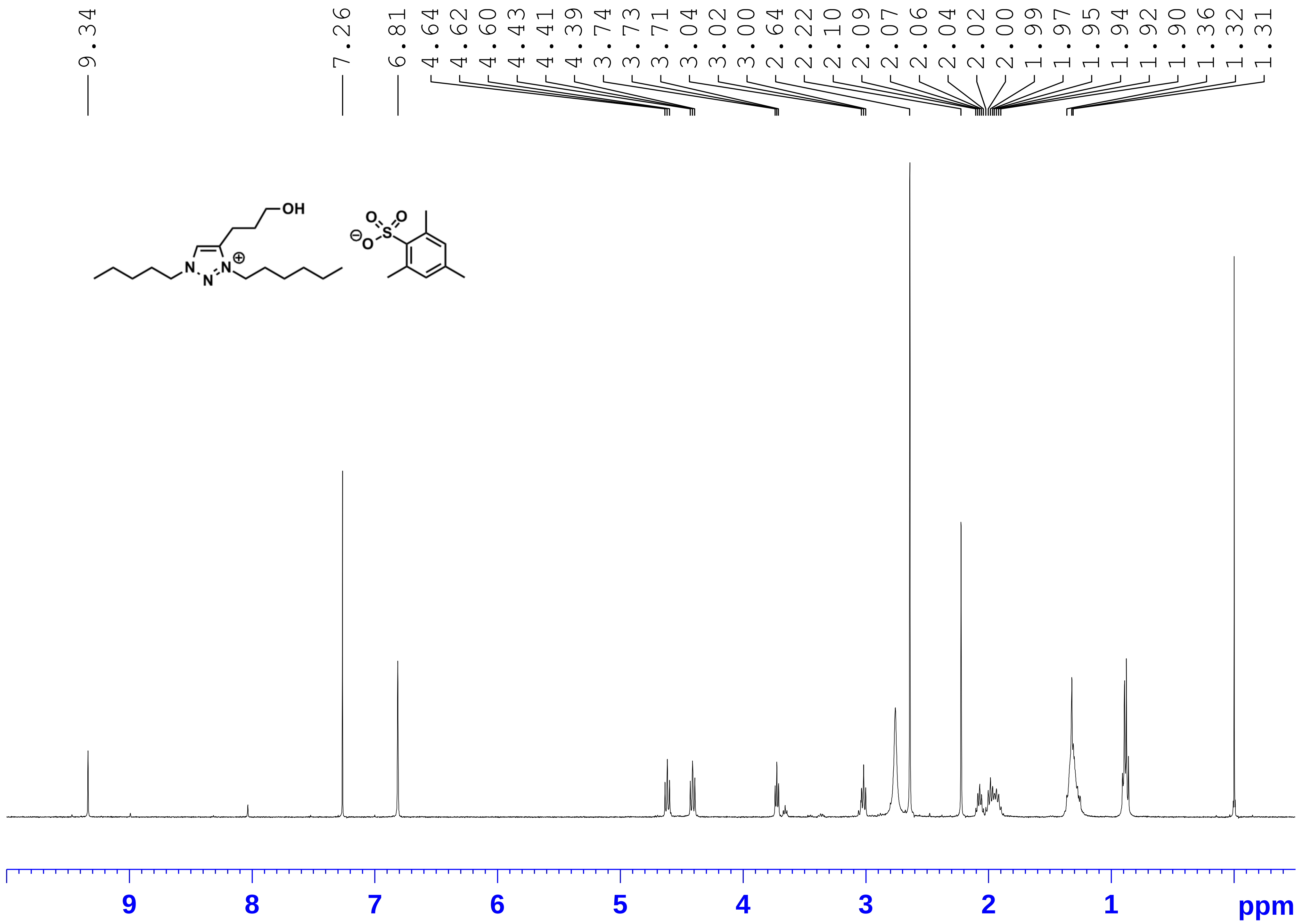

9.34
7.26
6.81
4.64
4.62
4.60
4.43
4.41
4.39
3.74
3.73
3.71
3.04
3.02
3.00
2.64
2.22
2.10
2.09
2.07
2.06
2.04
2.02
2.00
1.99
1.97
1.95
1.94
1.92
1.90
1.36
1.32
1.31
OH
N
N
N
O
S
O
O
9
8
7
6
5
4
3
2
1
ppm

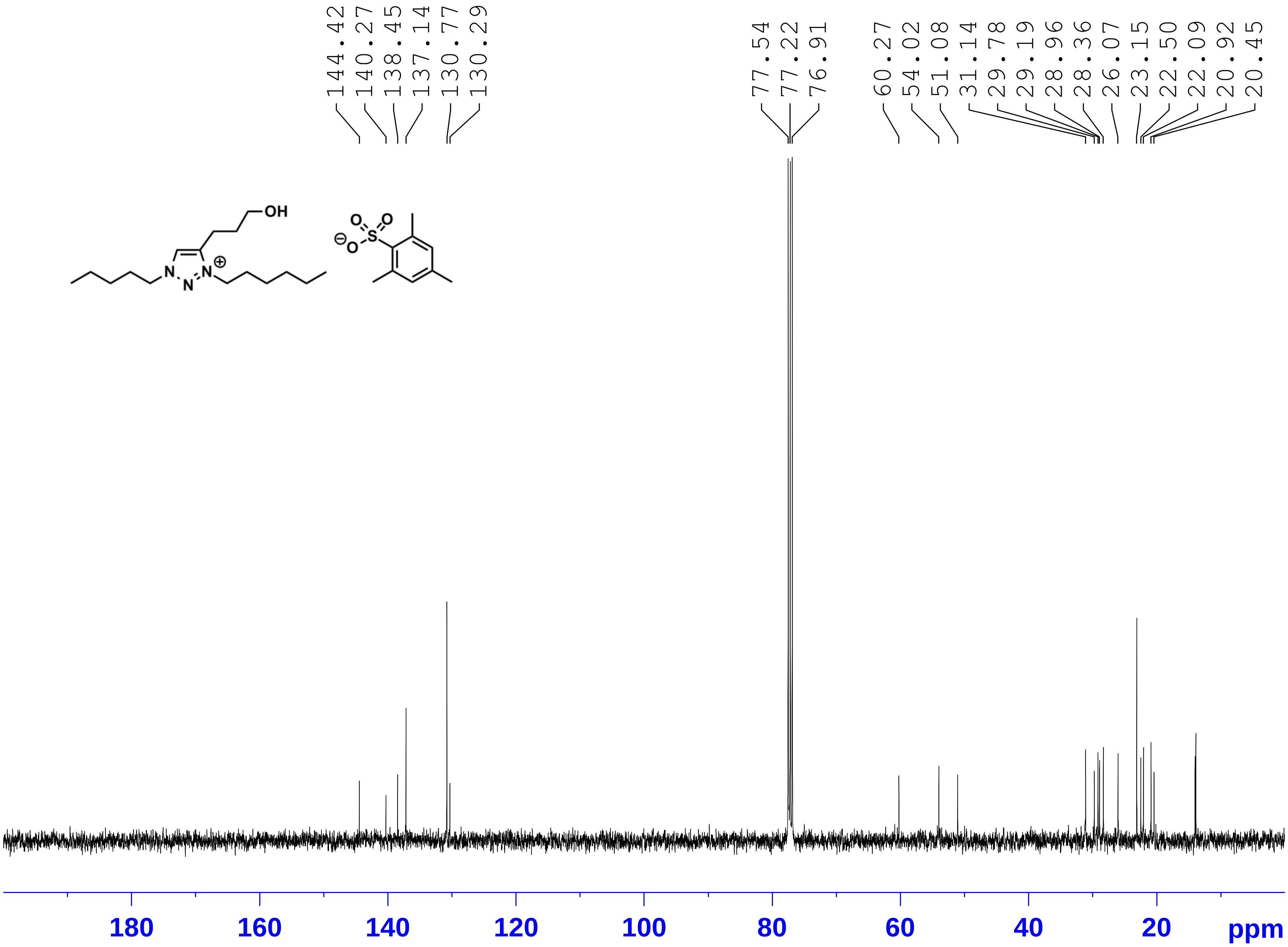
144.42
140.27
138.45
137.14
130.77
130.29
77.54
77.22
76.91
60.27
54.02
51.08
31.14
29.78
29.19
28.96
28.36
26.07
23.15
22.50
22.09
20.92
20.45
OH
O
S
O
O
N
N
N
180
160
140
120
100
80
60
40
20
ppm

Spectrum

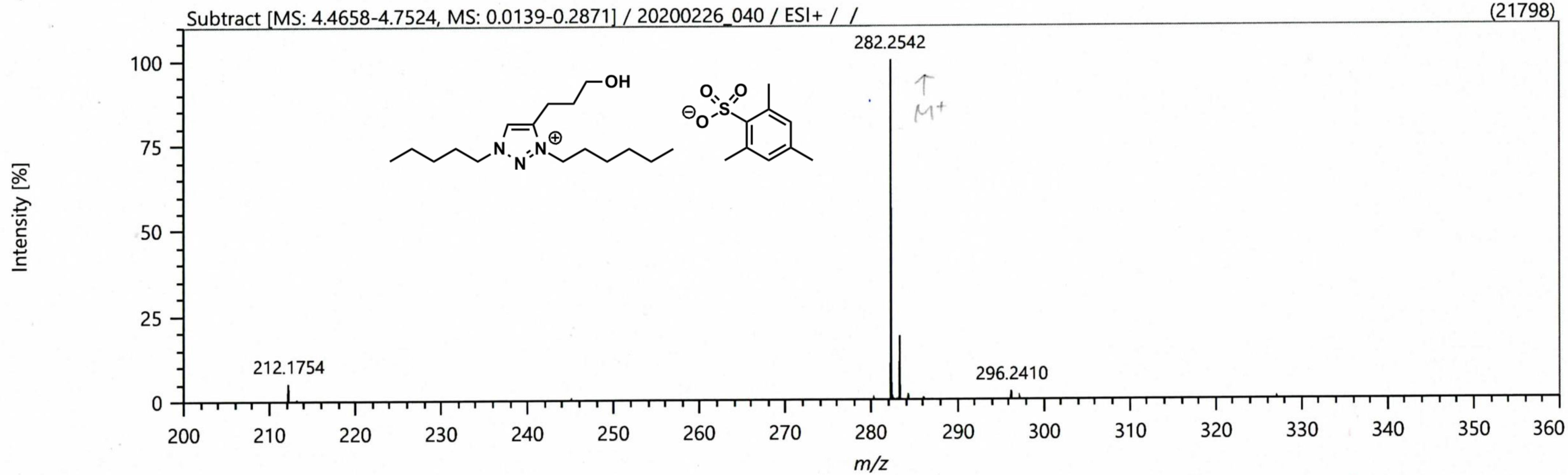

Elemental Composition

Parameters	
Tolerance:	±10.00 ppm
Electron:	Odd/Even
Charge:	+1
DBE:	-99.0 - 999.0

Elements Set 1:

Symbol	C	H	O	Na	N	S	Ni	F
Min	0	0	1	0	3	0	0	0
Max	1000	4000	1	0	3	0	0	0

Results

Mass	Formula	Calculated Mass	Mass Difference [mDa]	Mass Difference [ppm]	DBE
282.25418	C16 H32 N3 O	282.25399	0.19	0.69	2.5

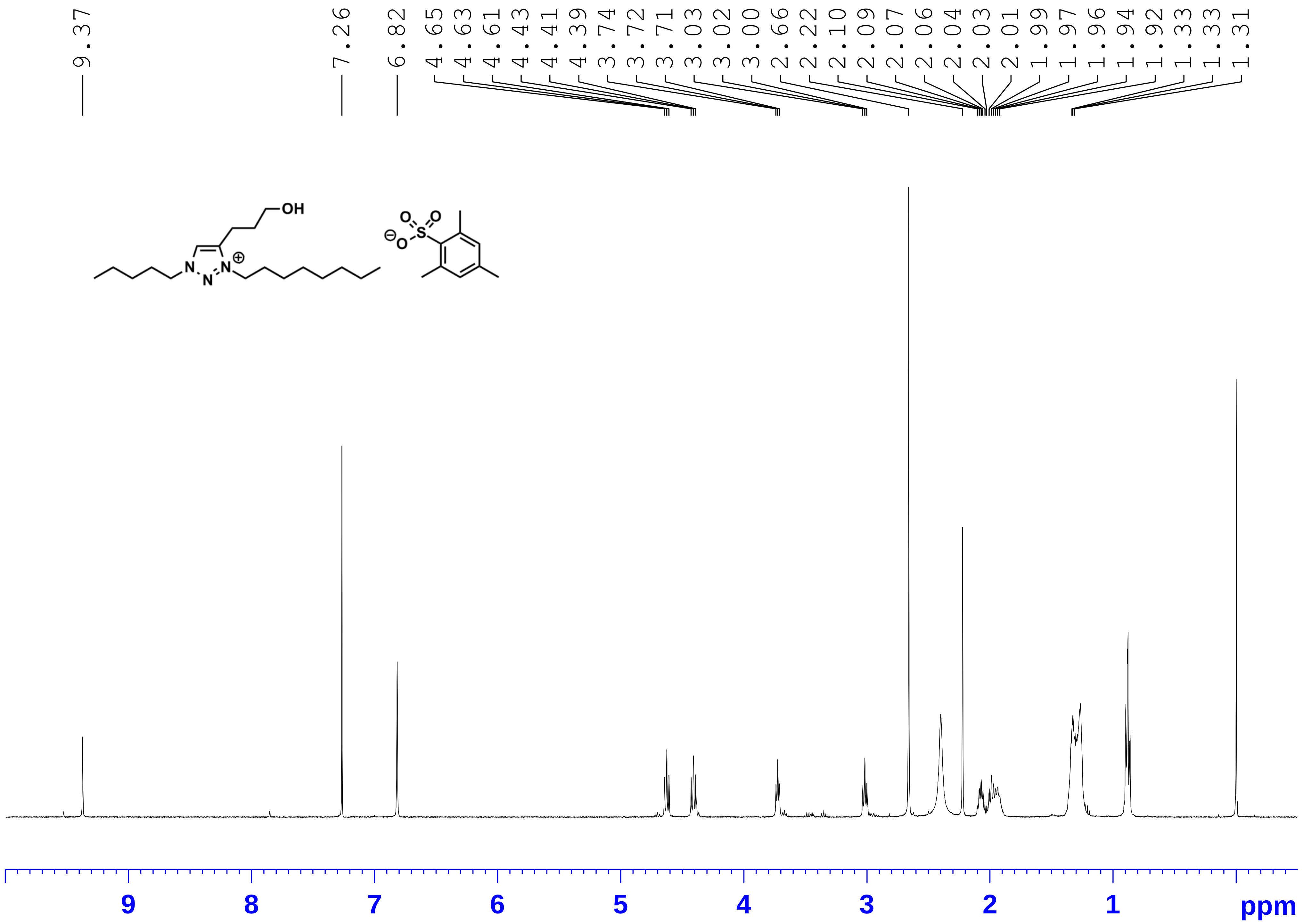
9.37
7.26
6.82
4.65
4.63
4.61
4.43
4.41
4.39
3.74
3.72
3.71
3.03
3.02
3.00
2.66
2.22
2.10
2.09
2.07
2.06
2.04
2.03
2.01
1.99
1.97
1.96
1.94
1.92
1.33
1.33
1.31
OH
N
N
N
O
S
O
O
9
8
7
6
5
4
3
2
1
ppm

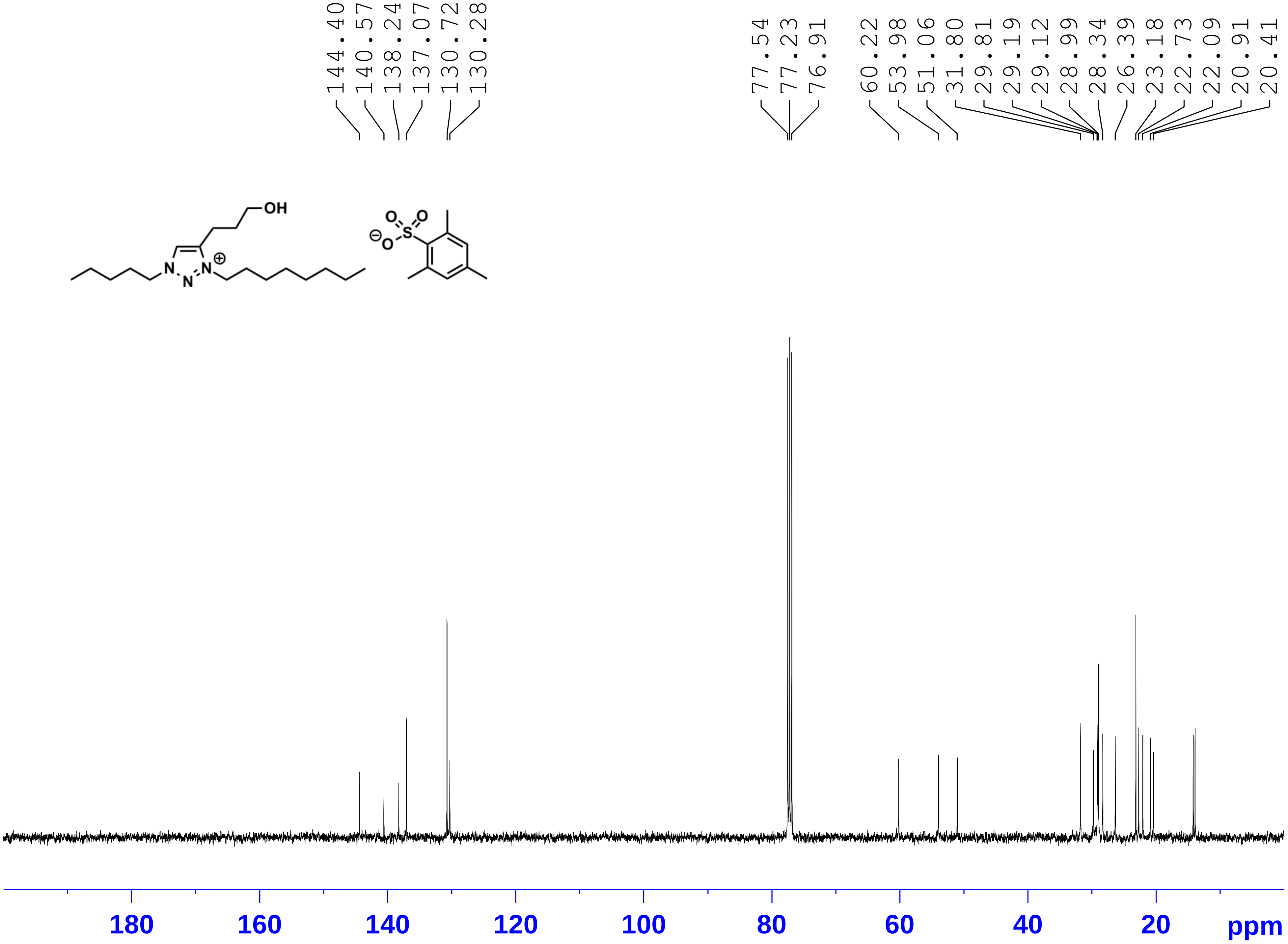

144.40
140.57
138.24
137.07
130.72
130.28
77.54
77.23
76.91
60.22
53.98
51.06
31.80
29.81
29.19
29.12
28.99
28.34
26.39
23.18
22.73
22.09
20.91
20.41
OH
N
N
N
O
O
S
O
180
160
140
120
100
80
60
40
20
ppm

Spectrum

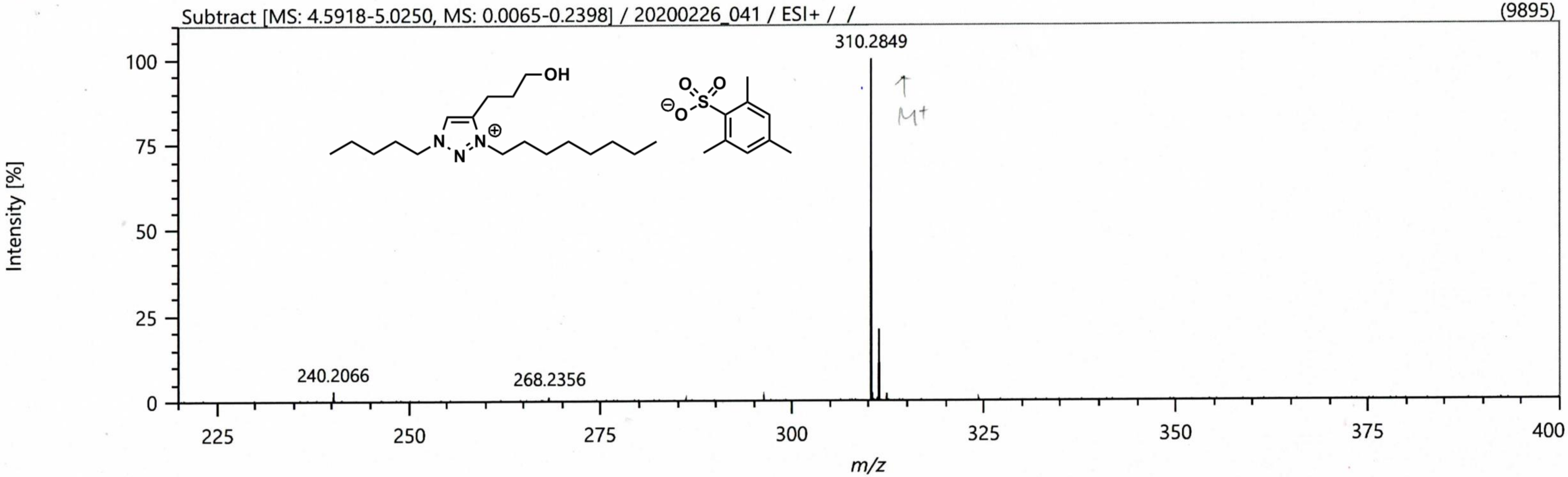

Elemental Composition

Parameters

Tolerance:	±10.00 ppm
Electron:	Odd/Even
Charge:	+1
DBE:	-99.0 - 999.0

Elements Set 1:

Symbol	C	H	O	Na	N	S	Ni	F
Min	0	0	1	0	3	0	0	0
Max	1000	4000	1	0	3	0	0	0

Results

Mass	Formula	Calculated Mass	Mass Difference [mDa]	Mass Difference [ppm]	DBE
310.28487	C18 H36 N3 O	310.28529	-0.42	-1.35	2.5

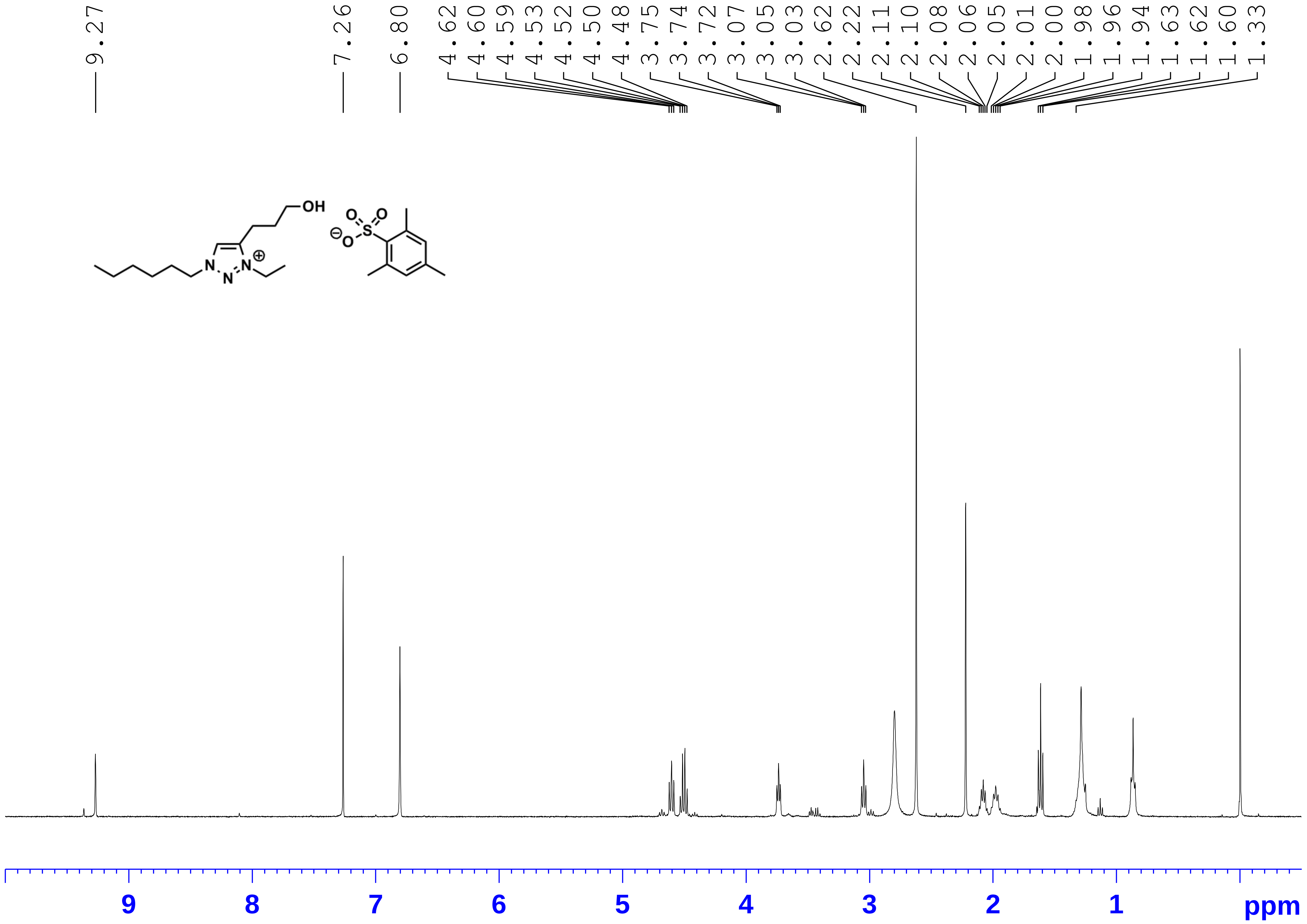
9.27
7.26
6.80
4.62
4.60
4.59
4.53
4.52
4.50
4.48
3.75
3.74
3.72
3.07
3.05
3.03
2.62
2.22
2.11
2.10
2.08
2.06
2.05
2.01
2.00
1.98
1.96
1.94
1.63
1.62
1.60
1.33
OH
O
S
O
O
N
N
N
9
8
7
6
5
4
3
2
1
ppm

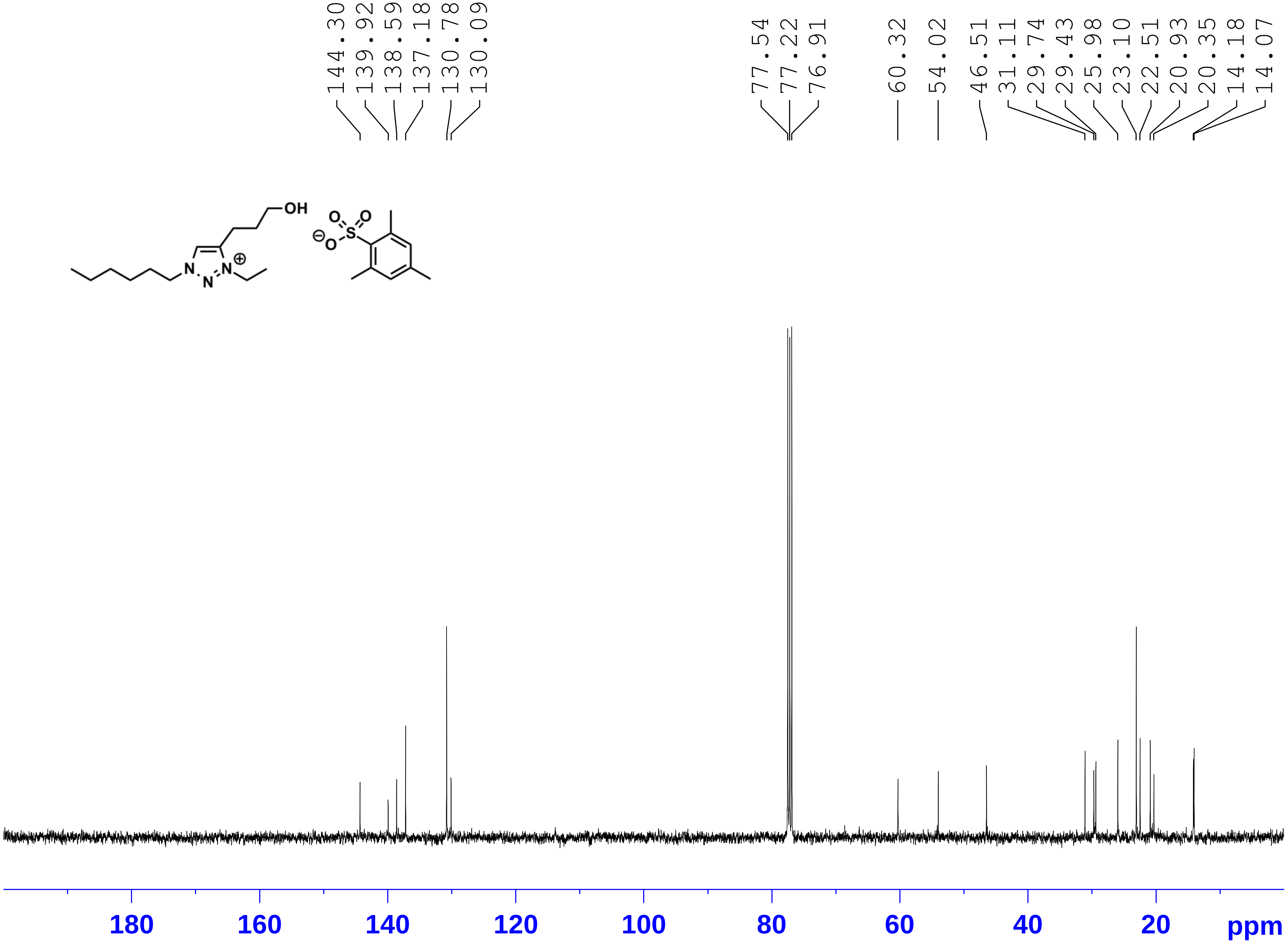
144.30
139.92
138.59
137.18
130.78
130.09
77.54
77.22
76.91
60.32
54.02
46.51
31.11
29.74
29.43
25.98
23.10
22.51
20.93
20.35
14.18
14.07
OH
O
S
O
O
N
N
N
180
160
140
120
100
80
60
40
20
ppm

Spectrum

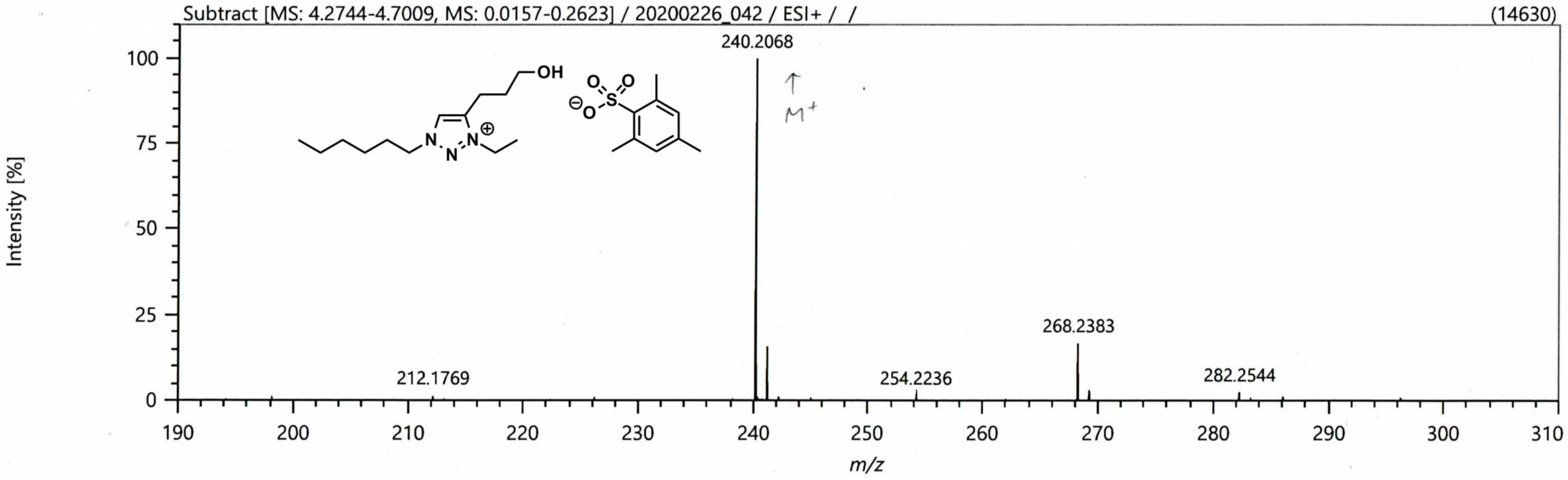

Elemental Composition

Parameters		Elements Set 1:								
Tolerance:	±10.00 ppm	Symbol	C	H	O	Na	N	S	Ni	F
Electron:	Odd/Even	Min	0	0	1	0	3	0	0	0
Charge:	+1	Max	1000	4000	1	0	3	0	0	0
DBE:	-99.0 - 999.0									

Results

Mass	Formula	Calculated Mass	Mass Difference [mDa]	Mass Difference [ppm]	DBE
240.20682	C13 H26 N3 O	240.20704	-0.22	-0.90	2.5

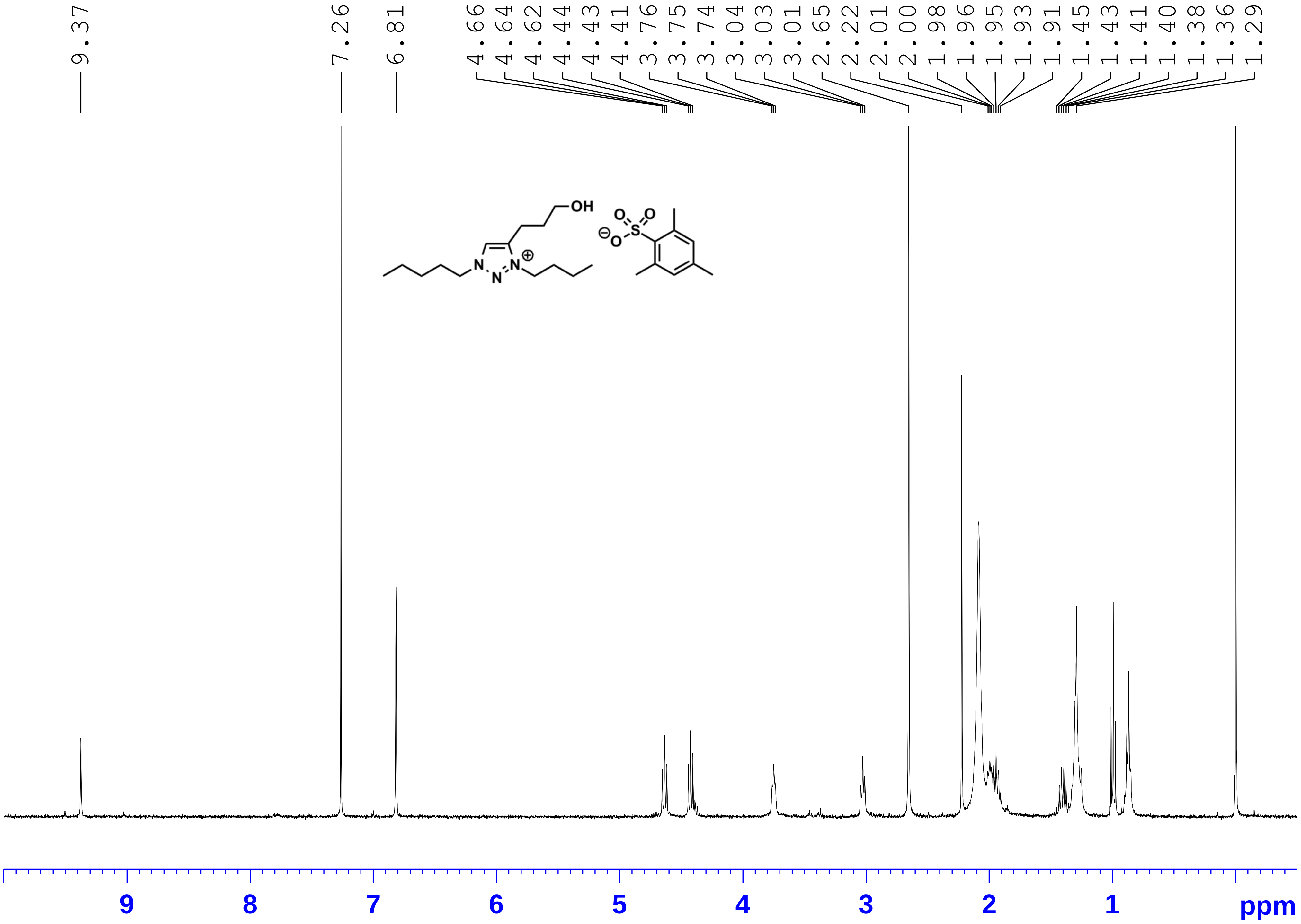
9.37
7.26
6.81
4.66
4.64
4.62
4.44
4.43
4.41
3.76
3.75
3.74
3.04
3.03
3.01
2.65
2.22
2.01
2.00
1.98
1.96
1.95
1.93
1.91
1.45
1.43
1.41
1.40
1.38
1.36
1.29
OH
N
N
N
O
O
S
O
9
8
7
6
5
4
3
2
1
ppm

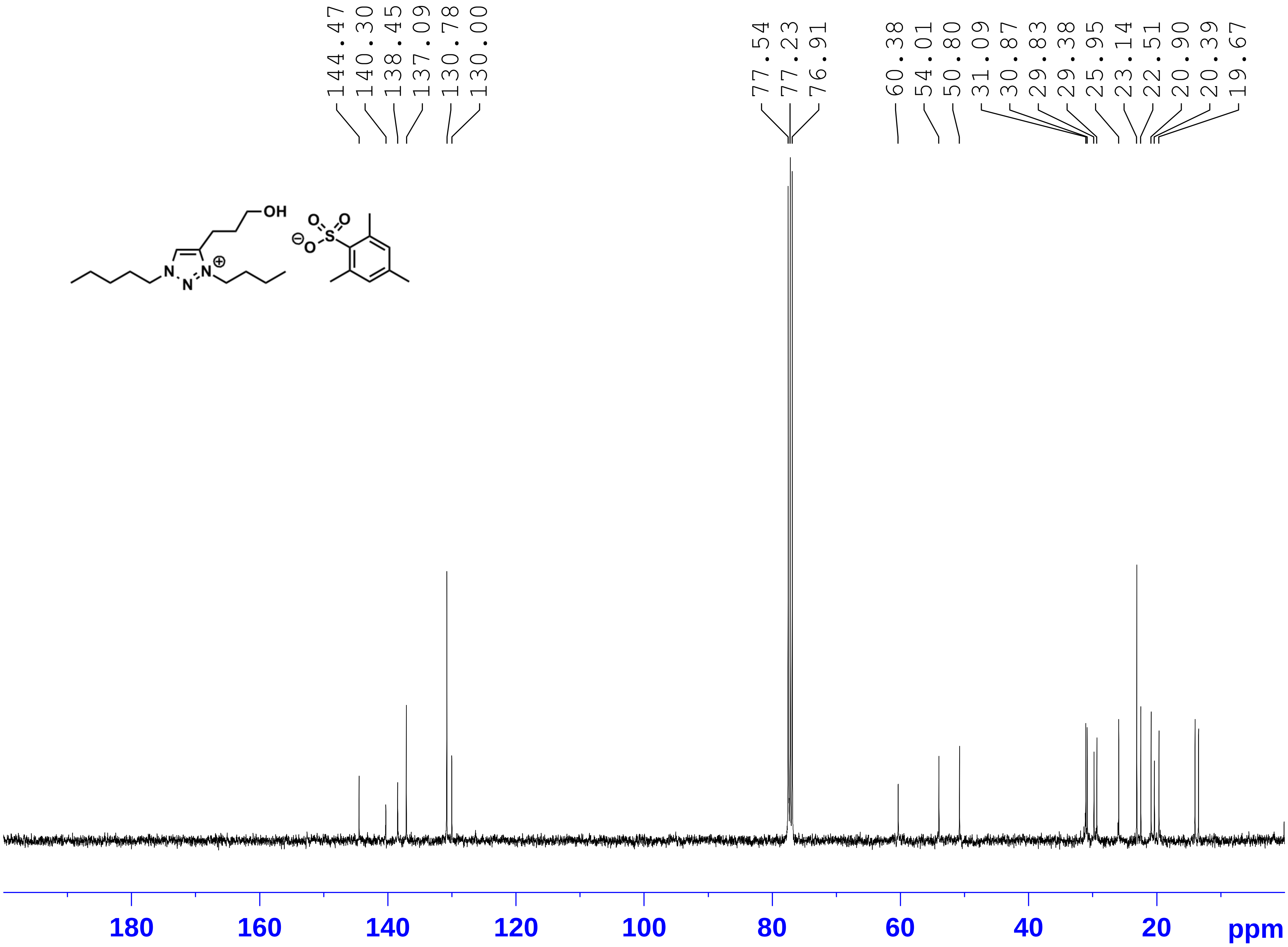
144.47
140.30
138.45
137.09
130.78
130.00
77.54
77.23
76.91
60.38
54.01
50.80
31.09
30.87
29.83
29.38
25.95
23.14
22.51
20.90
20.39
19.67
OH
O
S
O
O
N
N
N
180
160
140
120
100
80
60
40
20
ppm

Spectrum

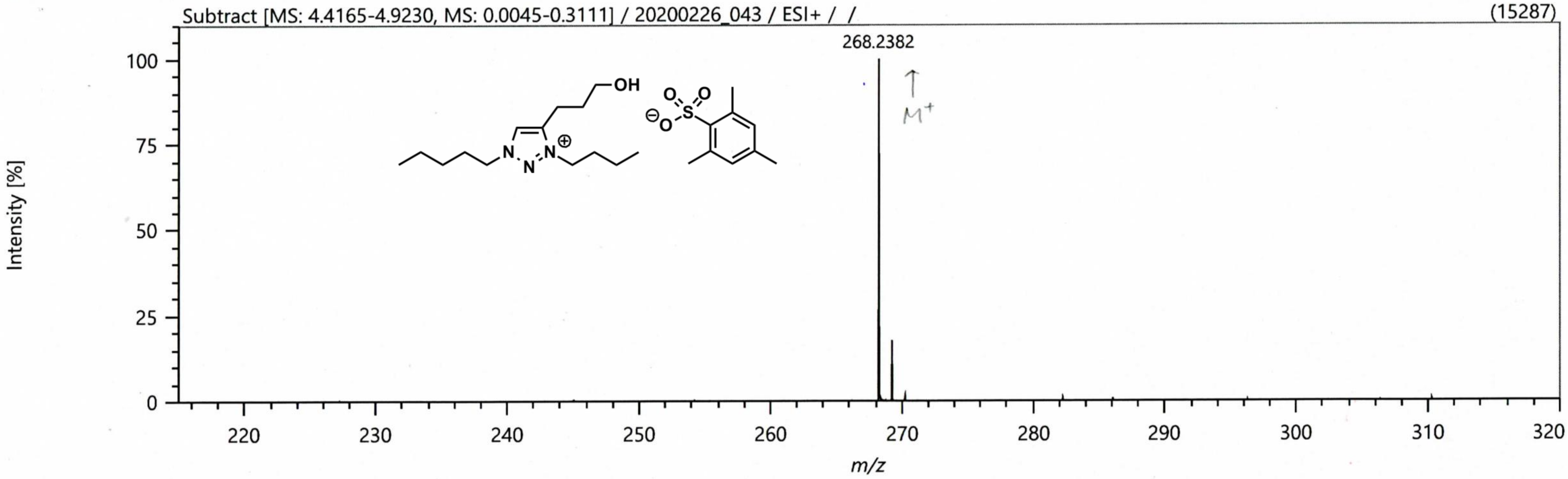

Elemental Composition

Parameters	
Tolerance:	±10.00 ppm
Electron:	Odd/Even
Charge:	+1
DBE:	-99.0 - 999.0

Elements Set 1:

Symbol	C	H	O	Na	N	S	Ni	F
Min	0	0	1	0	3	0	0	0
Max	1000	4000	1	0	3	0	0	0

Results

Mass	Formula	Calculated Mass	Mass Difference [mDa]	Mass Difference [ppm]	DBE
268.23824	C15 H30 N3 O	268.23834	-0.10	-0.37	2.5

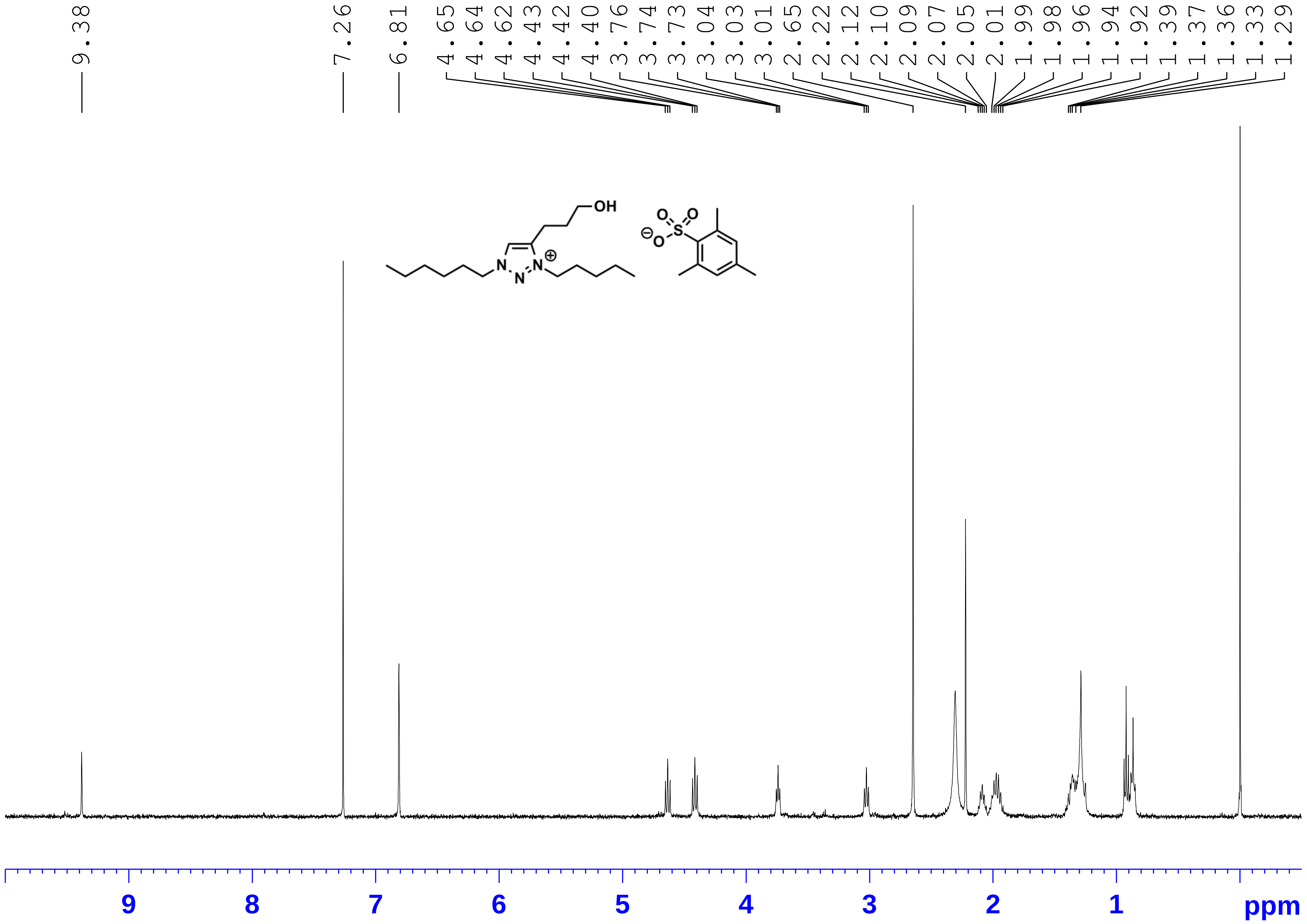

9.38
7.26
6.81
4.65
4.64
4.62
4.43
4.42
4.40
3.76
3.74
3.73
3.04
3.03
3.01
2.65
2.22
2.12
2.10
2.09
2.07
2.05
2.01
1.99
1.98
1.96
1.94
1.92
1.39
1.37
1.36
1.33
1.29
OH
N
N
N
O
O
S
O
9
8
7
6
5
4
3
2
1
ppm

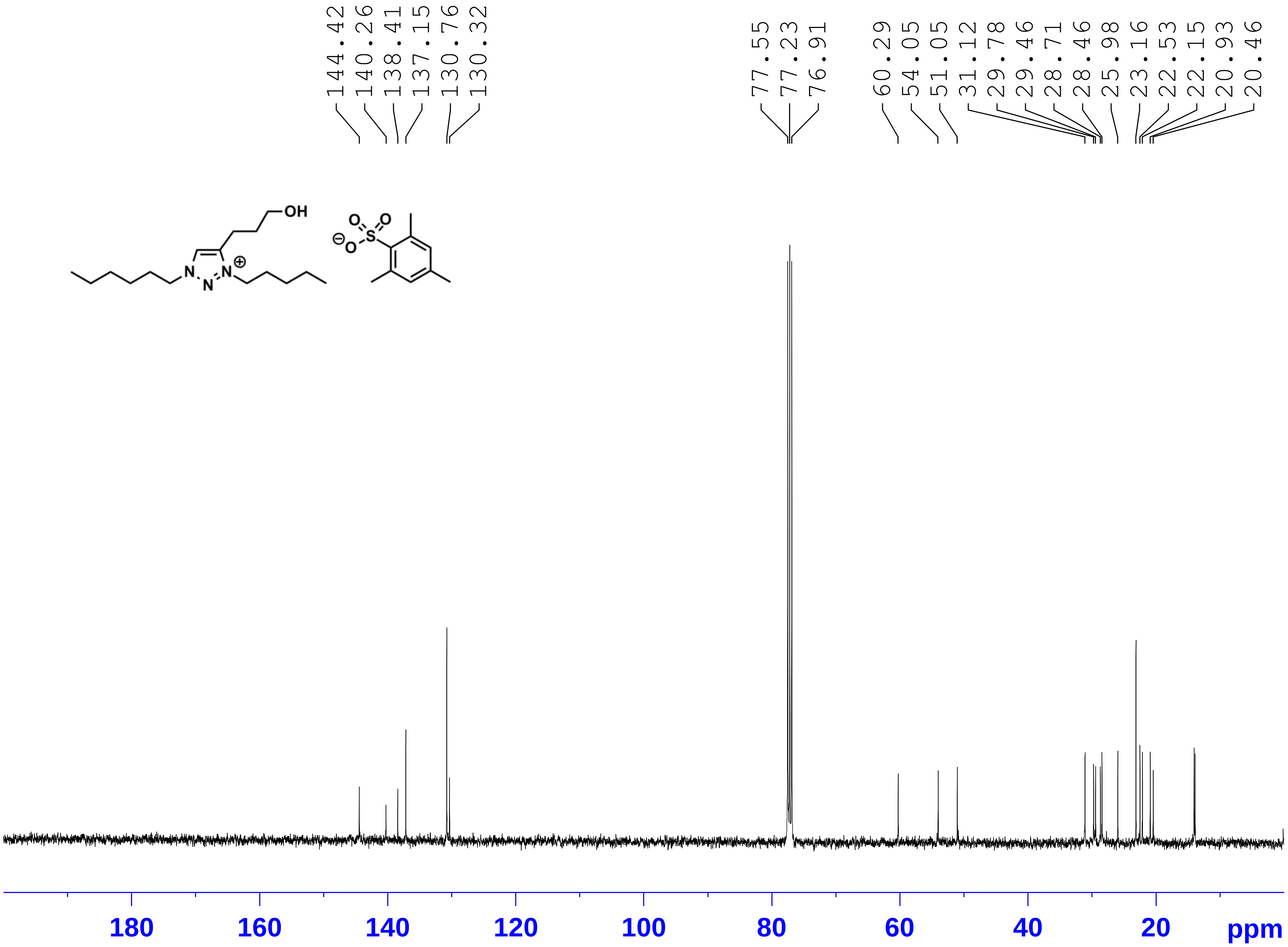
144.42
140.26
138.41
137.15
130.76
130.32
77.55
77.23
76.91
60.29
54.05
51.05
31.12
29.78
29.46
28.71
28.46
25.98
23.16
22.53
22.15
20.93
20.46
OH
O
S
O
O
N
N
N
180
160
140
120
100
80
60
40
20
ppm

Spectrum

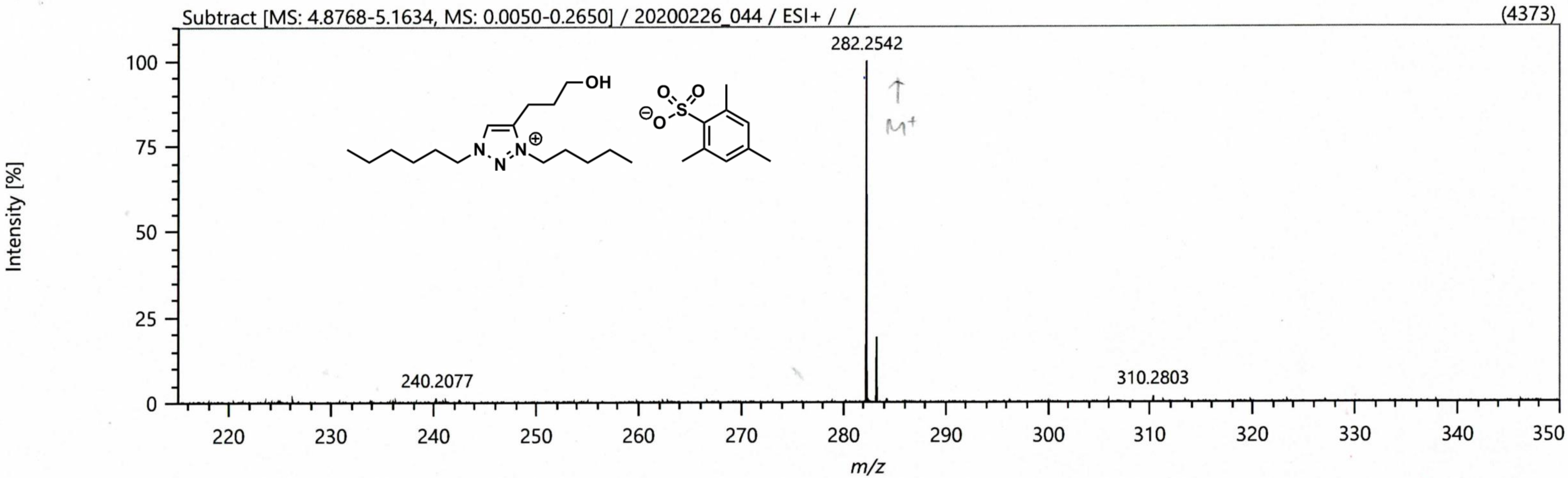

Elemental Composition

Parameters		Elements Set 1:								
Tolerance:	±10.00 ppm	Symbol	C	H	O	Na	N	S	Ni	F
Electron:	Odd/Even	Min	0	0	1	0	3	0	0	0
Charge:	+1	Max	1000	4000	1	0	3	0	0	0
DBE:	-99.0 - 999.0									

Results

Mass	Formula	Calculated Mass	Mass Difference [mDa]	Mass Difference [ppm]	DBE
282.25421	C16 H32 N3 O	282.25399	0.22	0.80	2.5

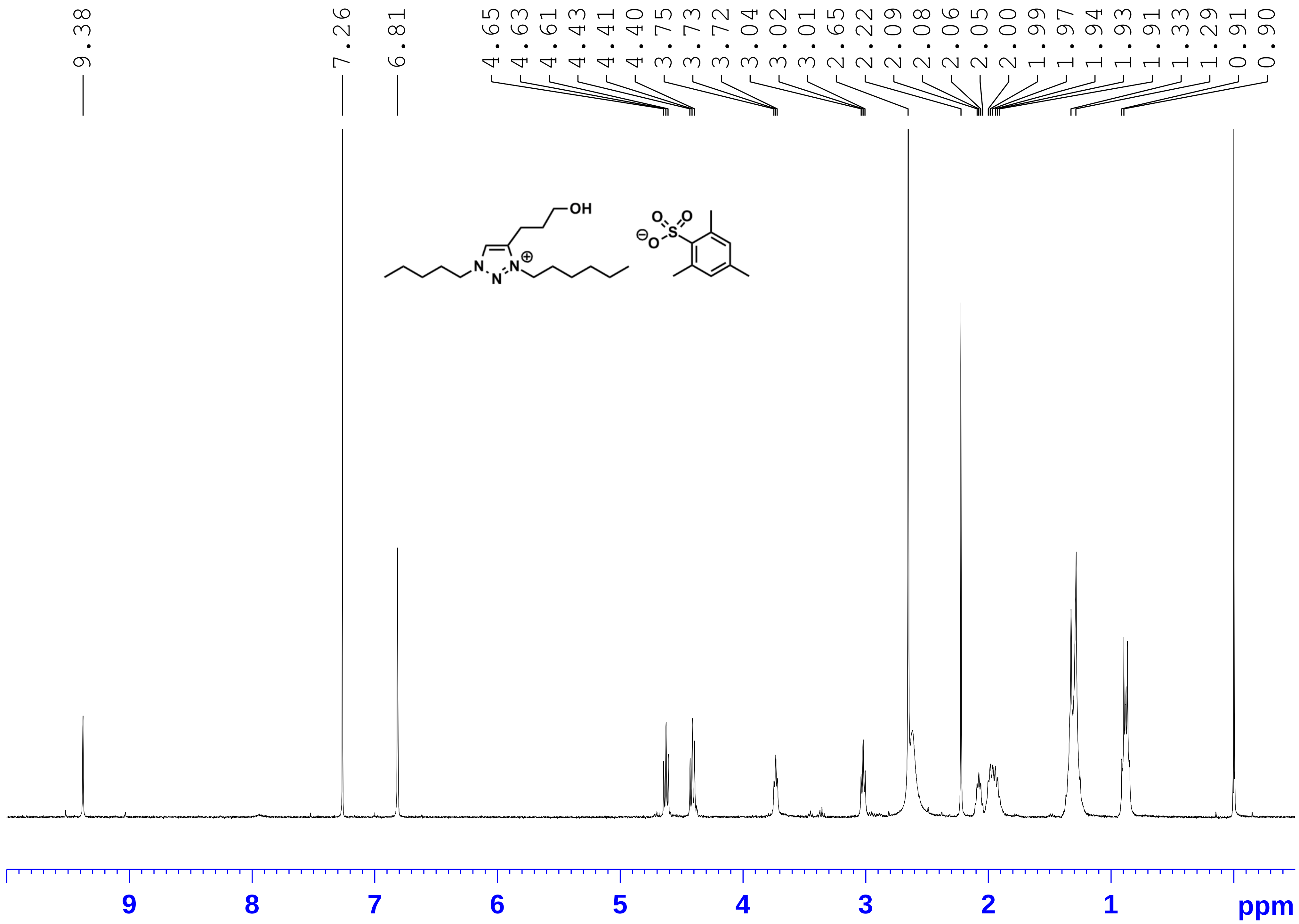
9.38
7.26
6.81
4.65
4.63
4.61
4.43
4.41
4.40
3.75
3.73
3.72
3.04
3.02
3.01
2.65
2.22
2.09
2.08
2.06
2.05
2.00
1.99
1.97
1.94
1.93
1.91
1.33
1.29
0.91
0.90
OH
N
N
N
S
O
O
O
9
8
7
6
5
4
3
2
1
ppm

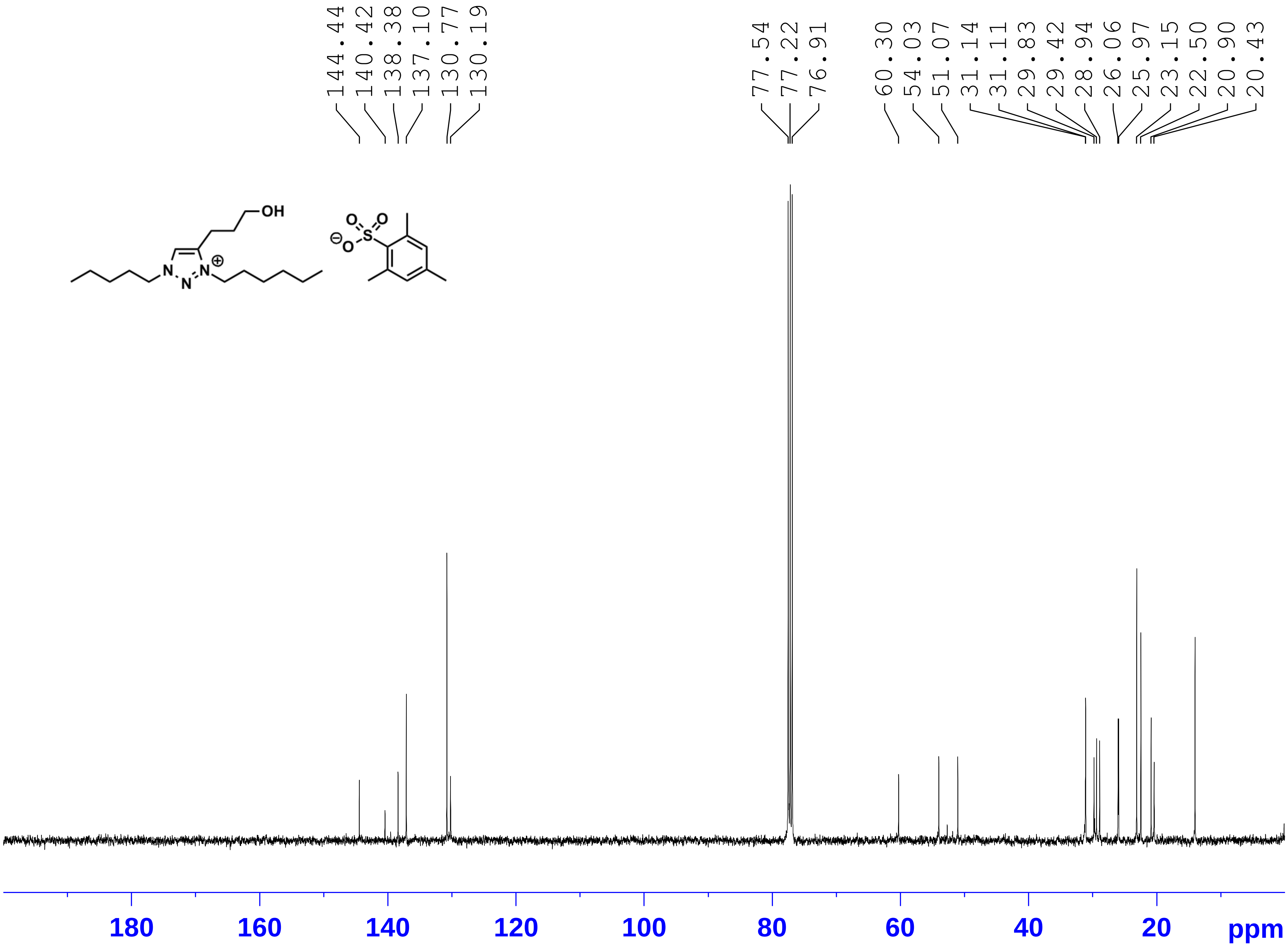
144.44
140.42
138.38
137.10
130.77
130.19
77.54
77.22
76.91
60.30
54.03
51.07
31.14
31.11
29.83
29.42
28.94
26.06
25.97
23.15
22.50
20.90
20.43
OH
N
N
N
O
O
O
S
180
160
140
120
100
80
60
40
20
ppm

Spectrum

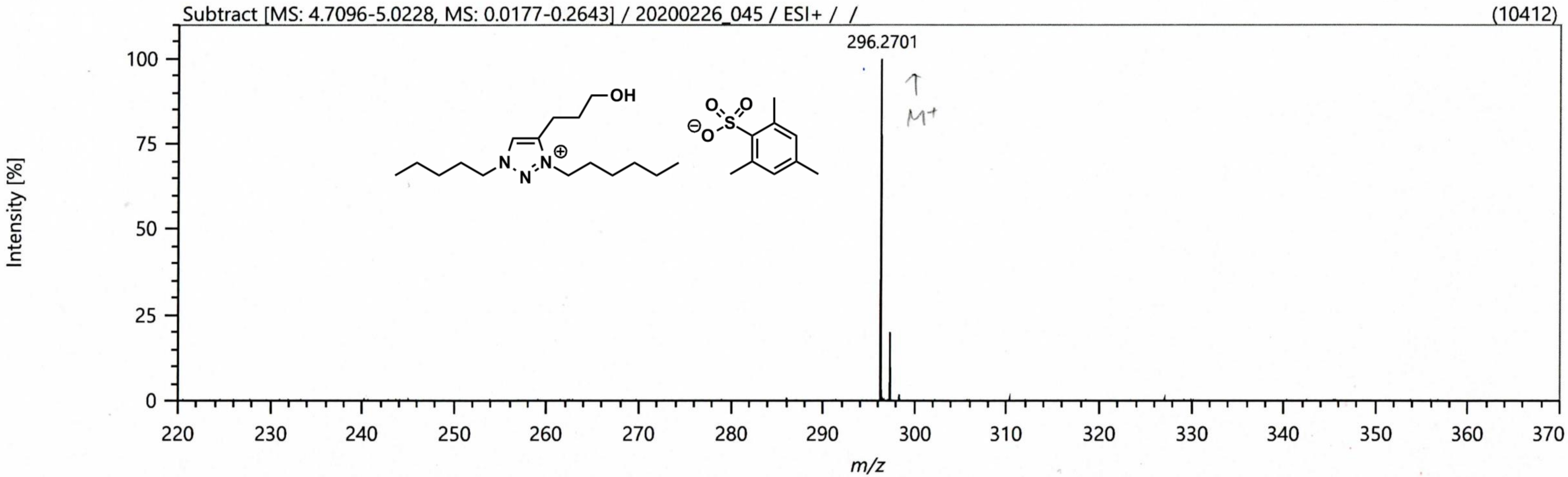

Elemental Composition

Parameters	
Tolerance:	±10.00 ppm
Electron:	Odd/Even
Charge:	+1
DBE:	-99.0 - 999.0

Elements Set 1:

Symbol	C	H	O	Na	N	S	Ni	F
Min	0	0	1	0	3	0	0	0
Max	1000	4000	1	0	3	0	0	0

Results

Mass	Formula	Calculated Mass	Mass Difference [mDa]	Mass Difference [ppm]	DBE
296.27011	C17 H34 N3 O	296.26964	0.47	1.58	2.5

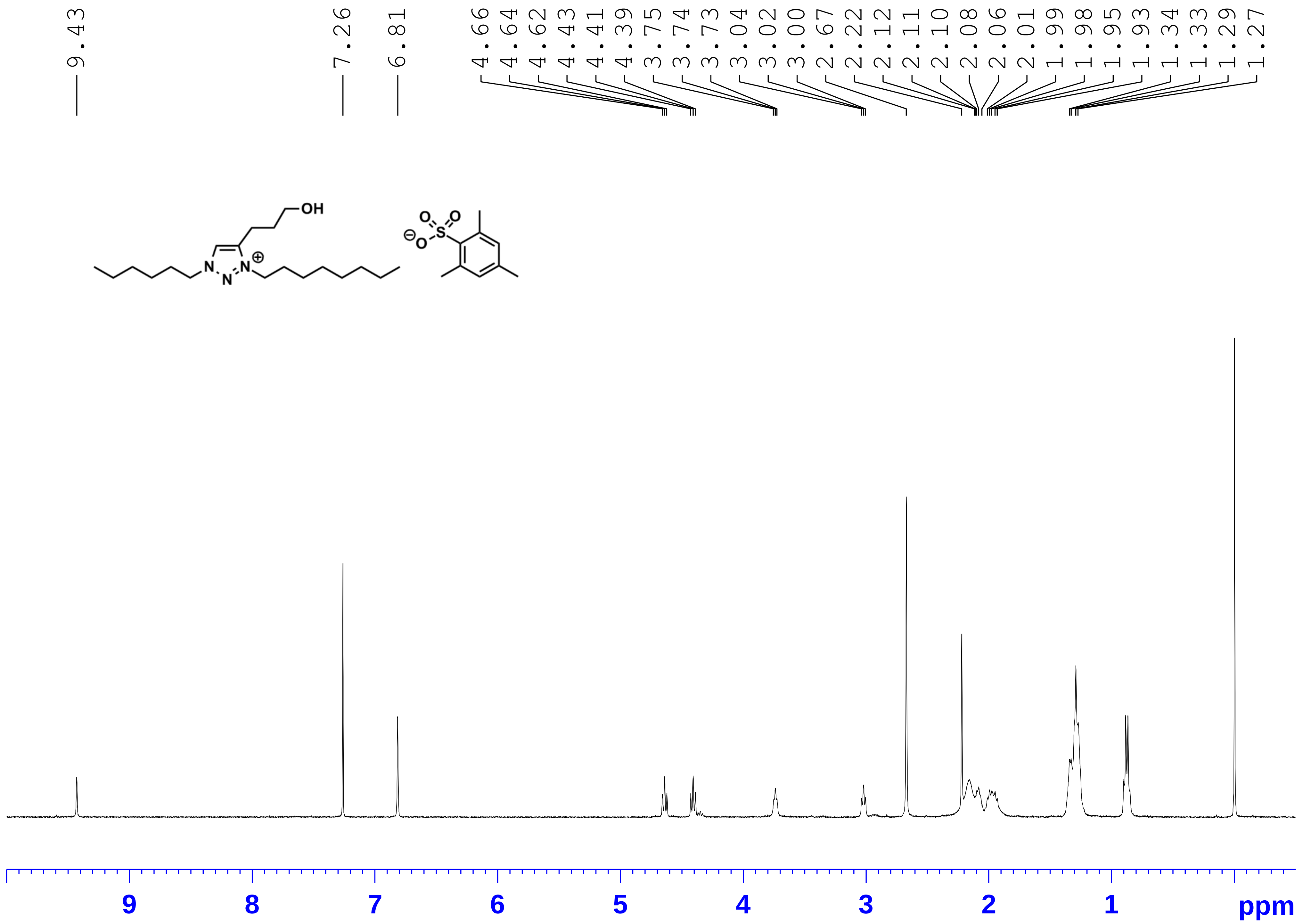
9.43
7.26
6.81
4.66
4.64
4.62
4.43
4.41
4.39
3.75
3.74
3.73
3.04
3.02
3.00
2.67
2.22
2.12
2.11
2.10
2.08
2.06
2.01
1.99
1.98
1.95
1.93
1.34
1.33
1.29
1.27
OH
9
8
7
6
5
4
3
2
1
ppm

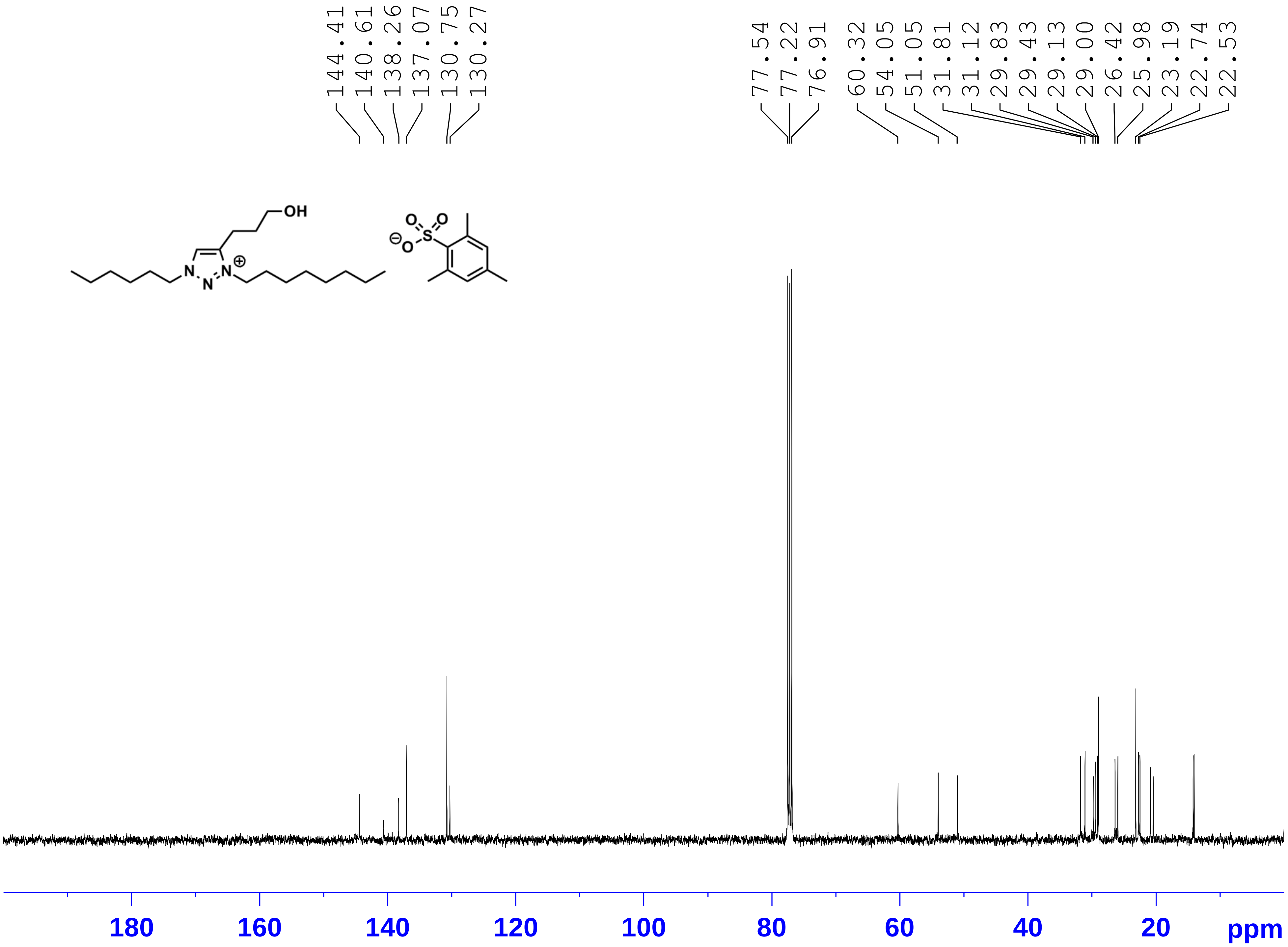

144.41
140.61
138.26
137.07
130.75
130.27
77.54
77.22
76.91
60.32
54.05
51.05
31.81
31.12
29.83
29.43
29.13
29.00
26.42
25.98
23.19
22.74
22.53
OH
N
N
N
O
O
S
O
180
160
140
120
100
80
60
40
20
ppm

Spectrum

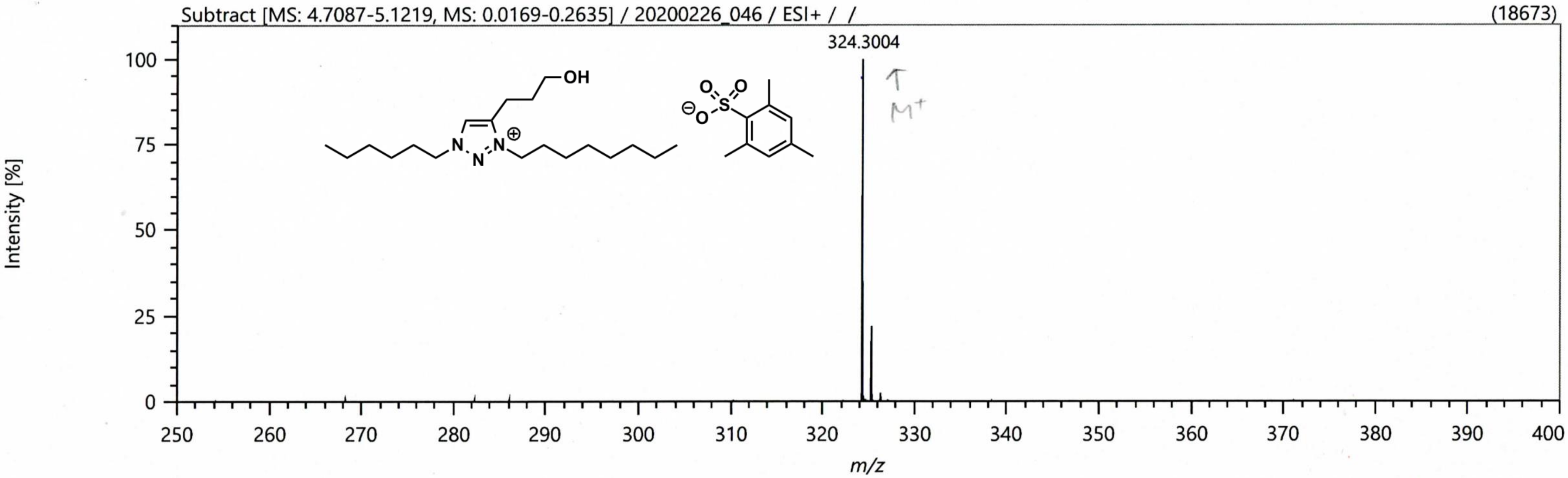

Elemental Composition

Parameters

Tolerance:	±10.00 ppm
Electron:	Odd/Even
Charge:	+1
DBE:	-99.0 - 999.0

Elements Set 1:

Symbol	C	H	O	Na	N	S	Ni	F
Min	0	0	1	0	3	0	0	0
Max	1000	4000	1	0	3	0	0	0

Results

Mass	Formula	Calculated Mass	Mass Difference [mDa]	Mass Difference [ppm]	DBE
324.30042	C19 H38 N3 O	324.30094	-0.51	-1.59	2.5

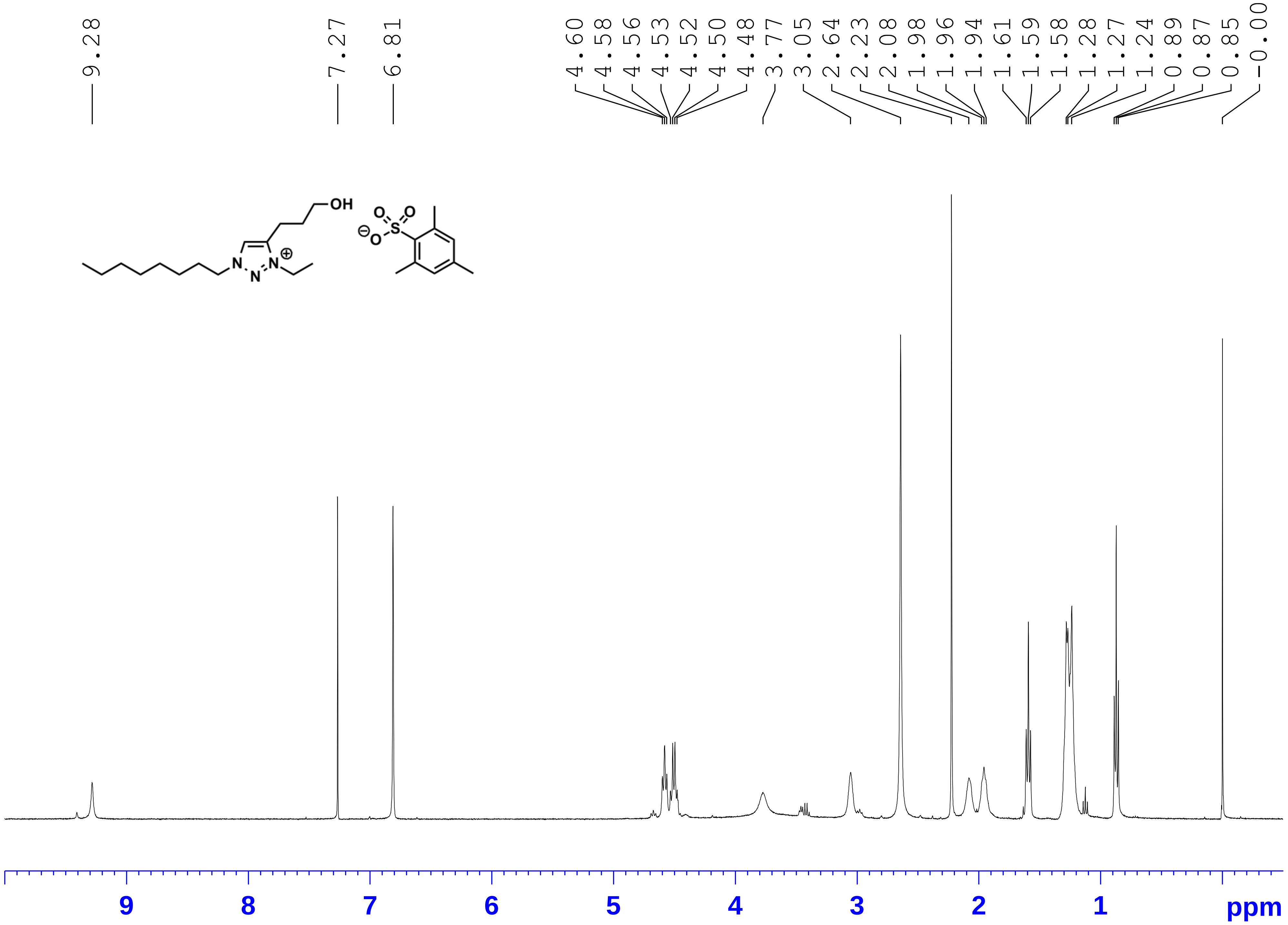
9.28
7.27
6.81
4.60
4.58
4.56
4.53
4.52
4.50
4.48
3.77
3.05
2.64
2.23
2.08
1.98
1.96
1.94
1.61
1.59
1.58
1.28
1.27
1.24
0.89
0.87
0.85
-0.00
OH
O
O
S
O
N
N
N
9
8
7
6
5
4
3
2
1
ppm

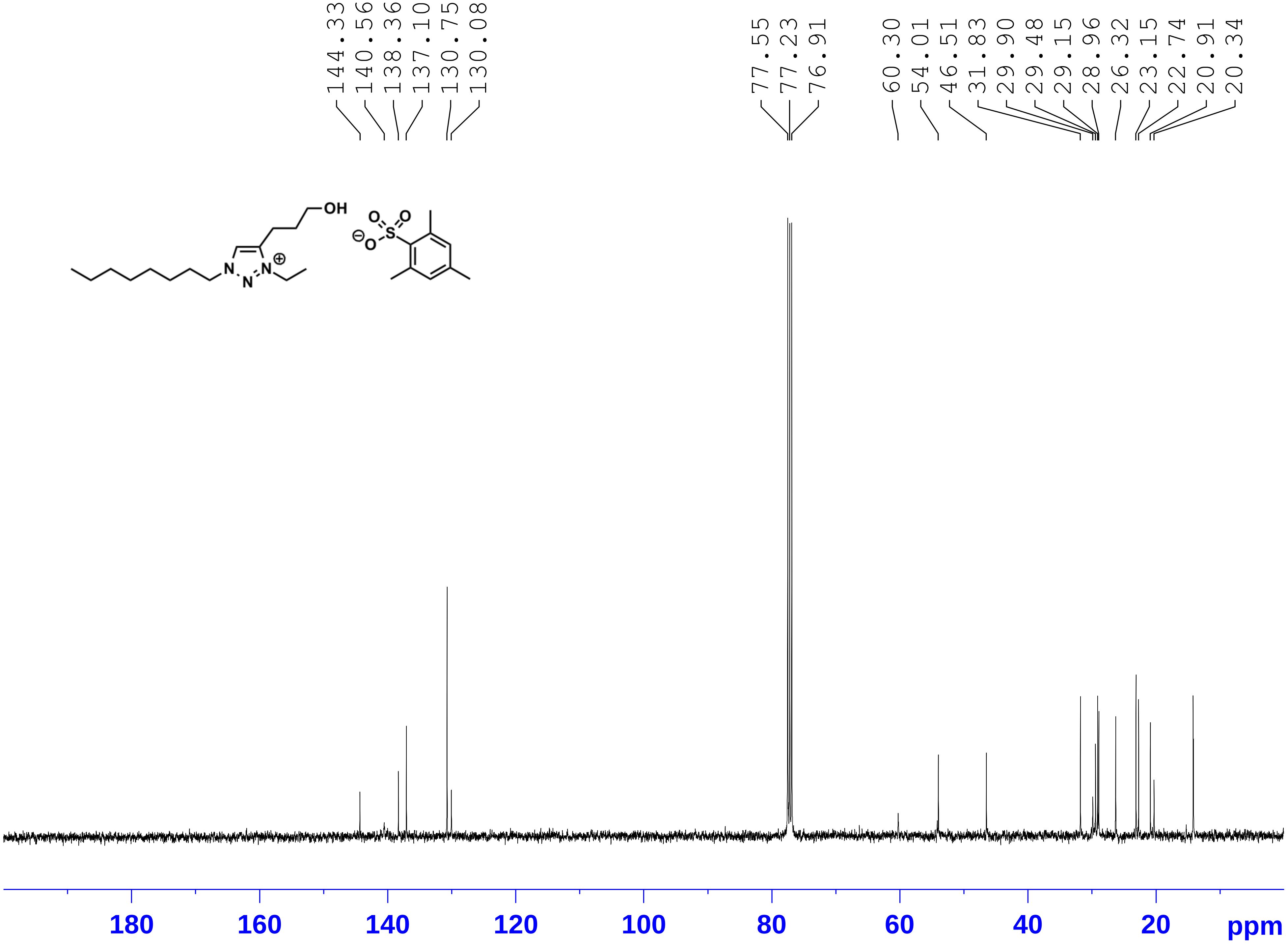
144.33
140.56
138.36
137.10
130.75
130.08
77.55
77.23
76.91
60.30
54.01
46.51
31.83
29.90
29.48
29.15
28.96
26.32
23.15
22.74
20.91
20.34
OH
O
S
O
O
N
N
N
180
160
140
120
100
80
60
40
20
ppm

Spectrum

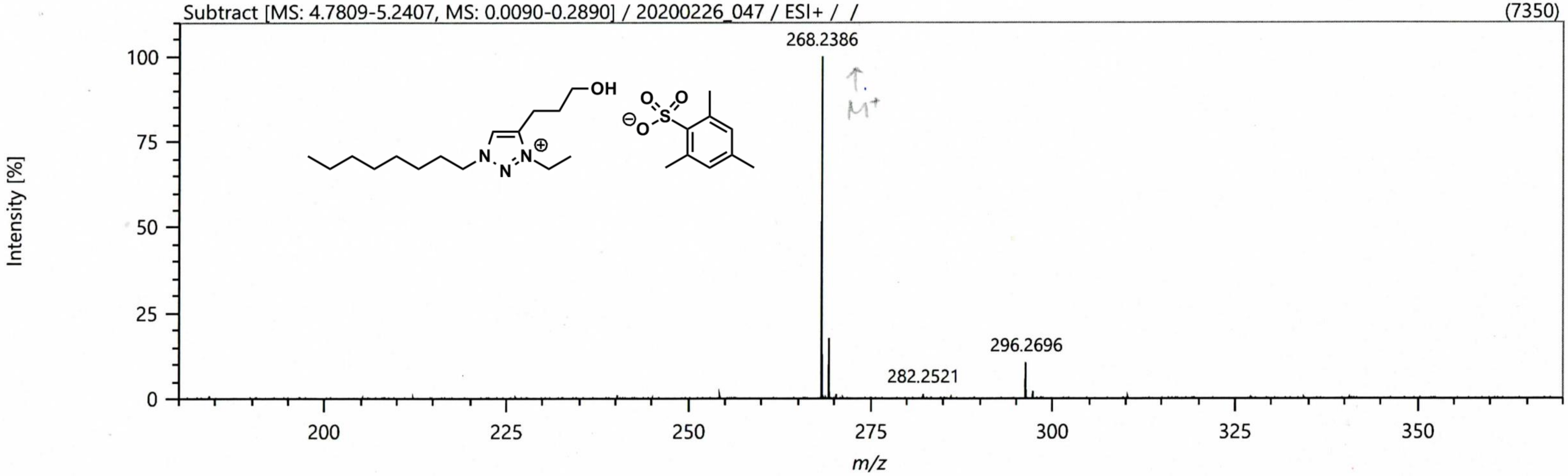

Elemental Composition

Parameters	
Tolerance:	±10.00 ppm
Electron:	Odd/Even
Charge:	+1
DBE:	-99.0 - 999.0

Elements Set 1:

Symbol	C	H	O	Na	N	S	Ni	F
Min	0	0	1	0	3	0	0	0
Max	1000	4000	1	0	3	0	0	0

Results

Mass	Formula	Calculated Mass	Mass Difference [mDa]	Mass Difference [ppm]	DBE
268.23857	C15 H30 N3 O	268.23834	0.23	0.87	2.5

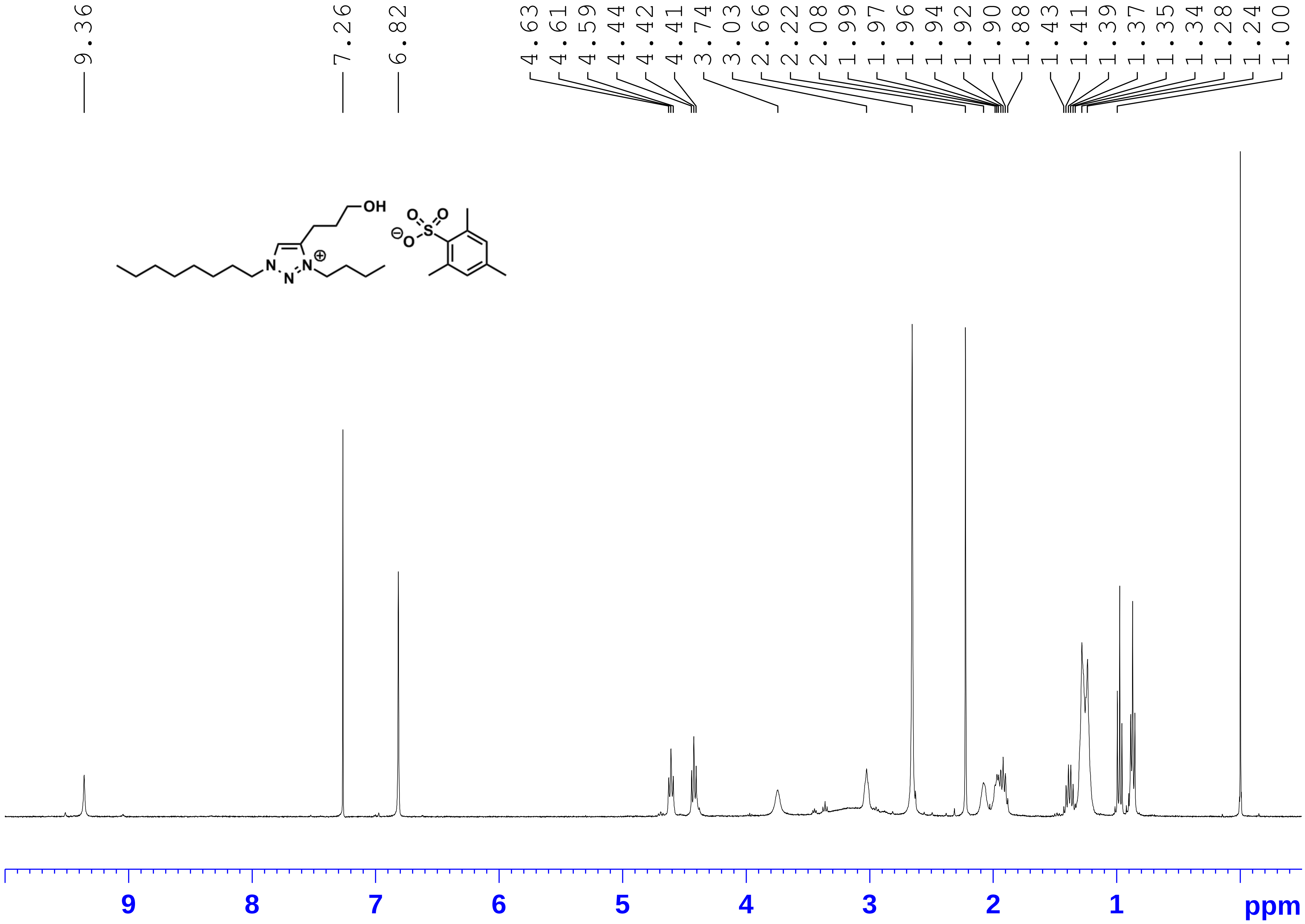
9.36
7.26
6.82
4.63
4.61
4.59
4.44
4.42
4.41
3.74
3.03
2.66
2.22
2.08
1.99
1.97
1.96
1.94
1.92
1.90
1.88
1.43
1.41
1.39
1.37
1.35
1.34
1.28
1.24
1.00
OH
N
N
N
O
S
O
O
9
8
7
6
5
4
3
2
1
ppm

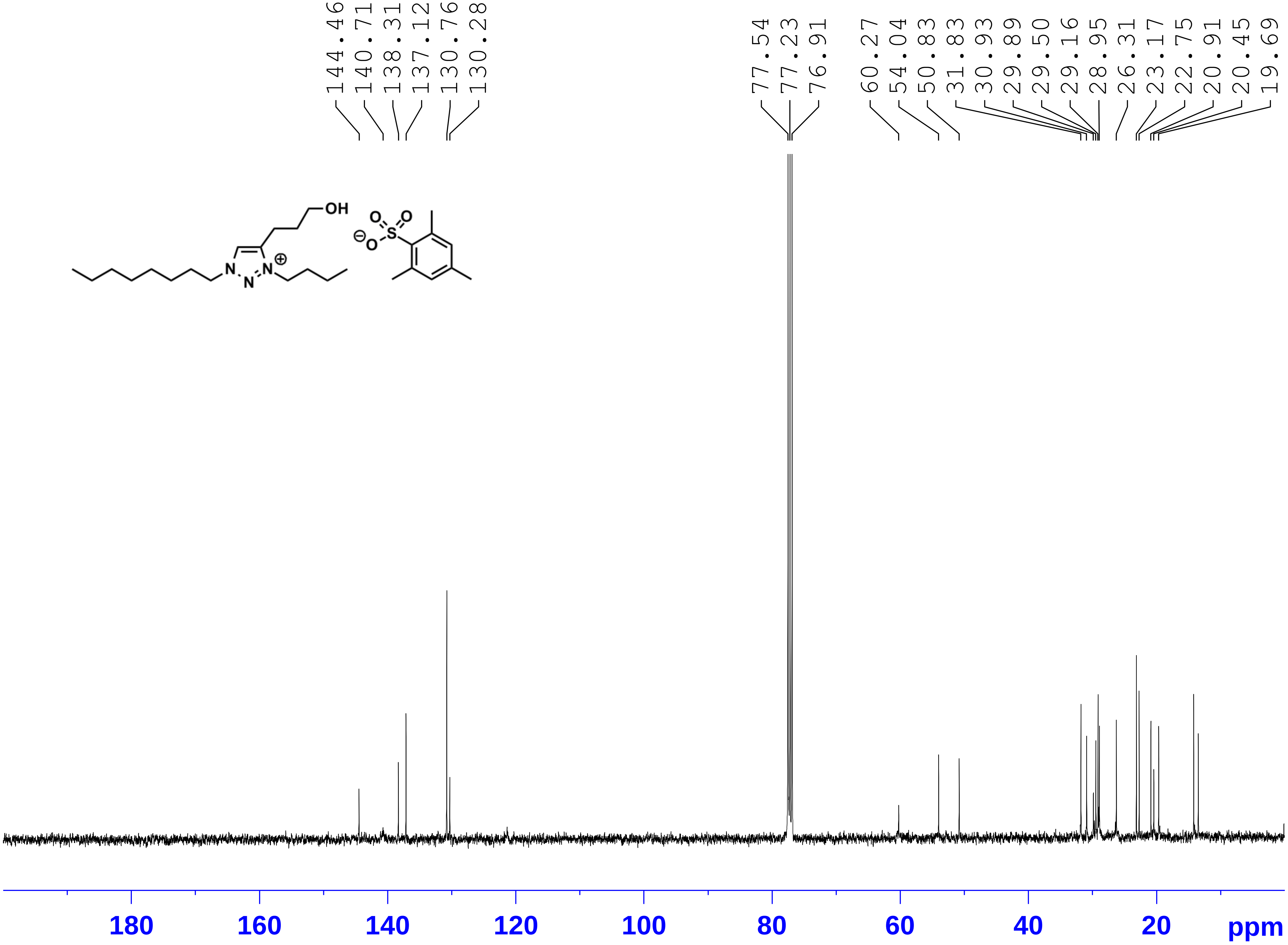
144.46
140.71
138.31
137.12
130.76
130.28
77.54
77.23
76.91
60.27
54.04
50.83
31.83
30.93
29.89
29.50
29.16
28.95
26.31
23.17
22.75
20.91
20.45
19.69
OH
O
S
O
O
N
N
N
180
160
140
120
100
80
60
40
20
ppm

Spectrum

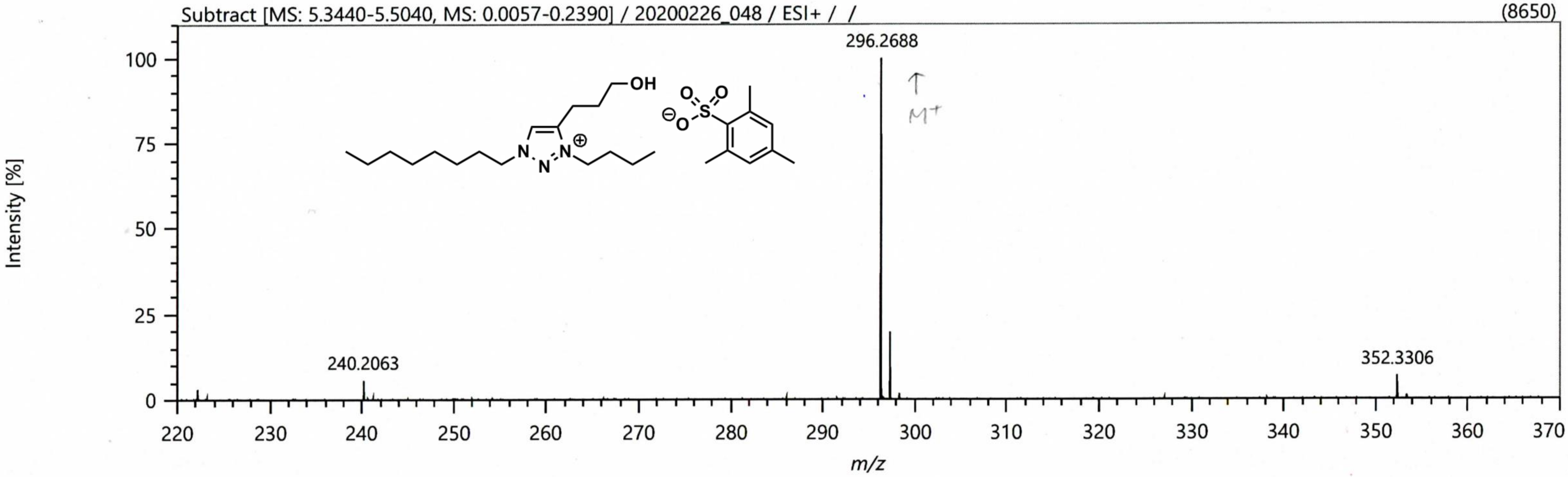

Elemental Composition

Parameters	
Tolerance:	±10.00 ppm
Electron:	Odd/Even
Charge:	+1
DBE:	-99.0 - 999.0

Elements Set 1:

Symbol	C	H	O	Na	N	S	Ni	F
Min	0	0	1	0	3	0	0	0
Max	1000	4000	1	0	3	0	0	0

Results

Mass	Formula	Calculated Mass	Mass Difference [mDa]	Mass Difference [ppm]	DBE
296.26879	C17 H34 N3 O	296.26964	-0.85	-2.85	2.5

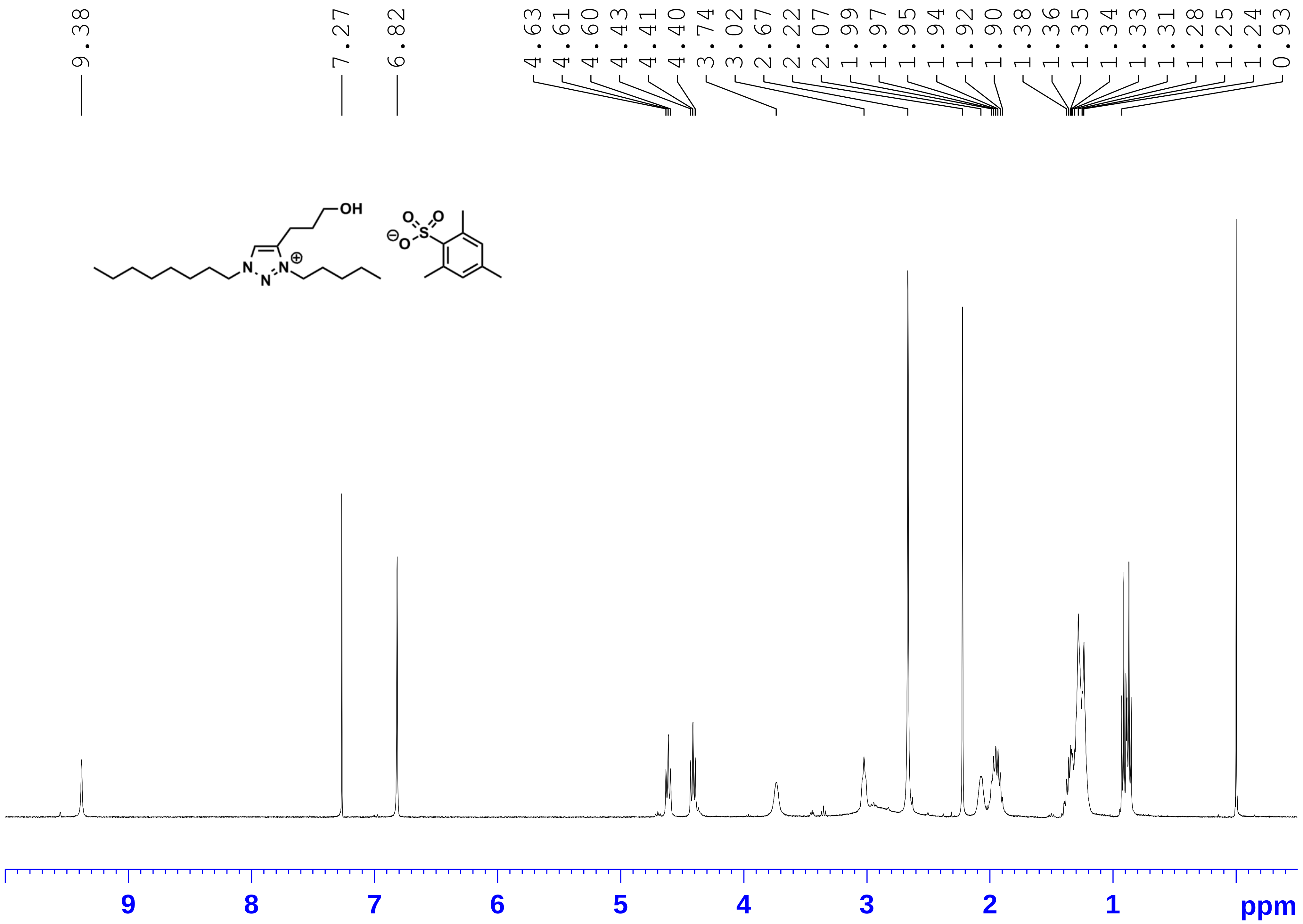
9.38
7.27
6.82
4.63
4.61
4.60
4.43
4.41
4.40
3.74
3.02
2.67
2.22
2.07
1.99
1.97
1.95
1.94
1.92
1.90
1.38
1.36
1.35
1.34
1.33
1.31
1.28
1.25
1.24
0.93
OH
N
N
N
O
O
S
O
9
8
7
6
5
4
3
2
1
ppm

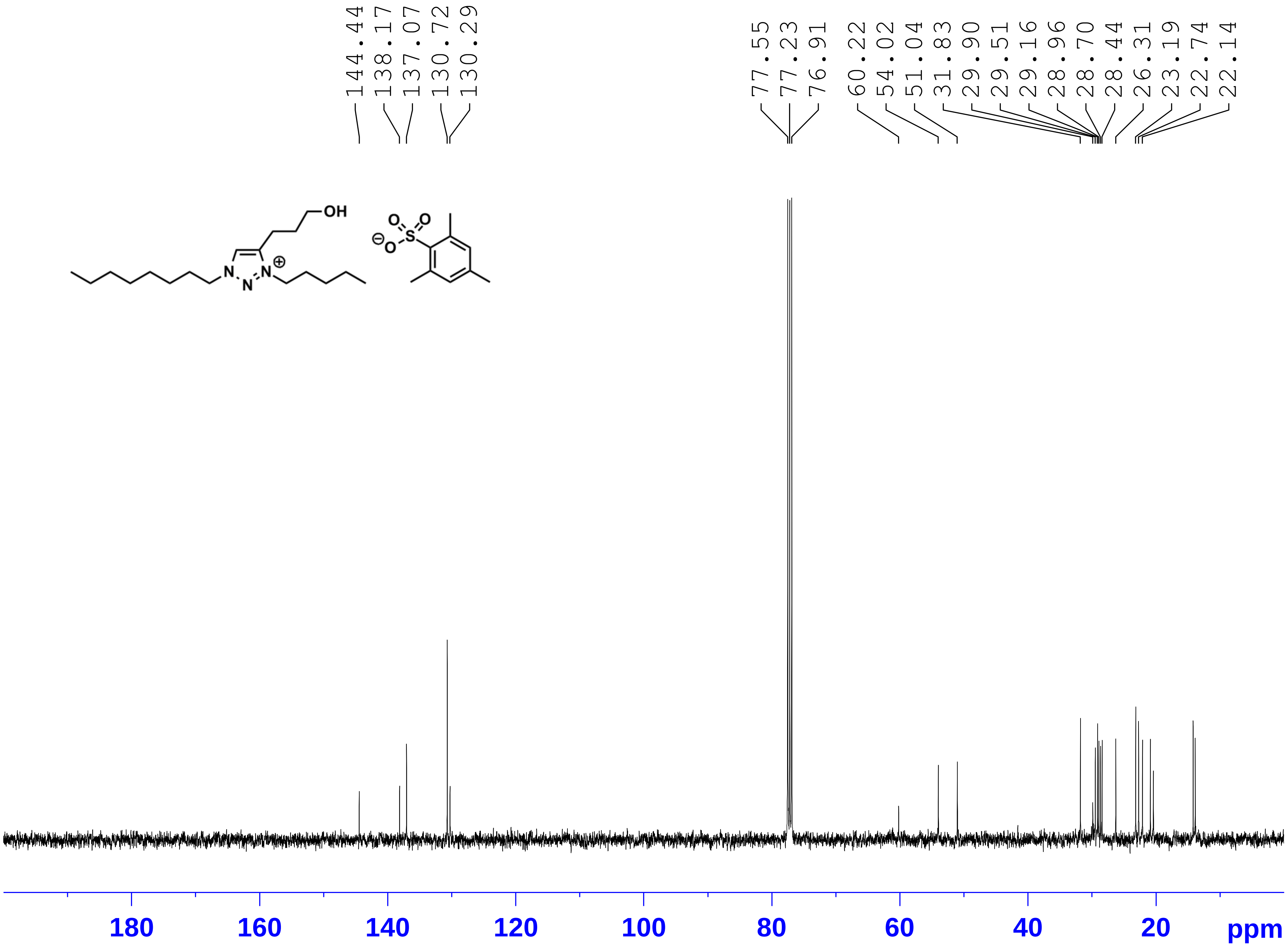
144.44
138.17
137.07
130.72
130.29
77.55
77.23
76.91
60.22
54.02
51.04
31.83
29.90
29.51
29.16
28.96
28.70
28.44
26.31
23.19
22.74
22.14
OH
O
S
N
180
160
140
120
100
80
60
40
20
ppm

Spectrum

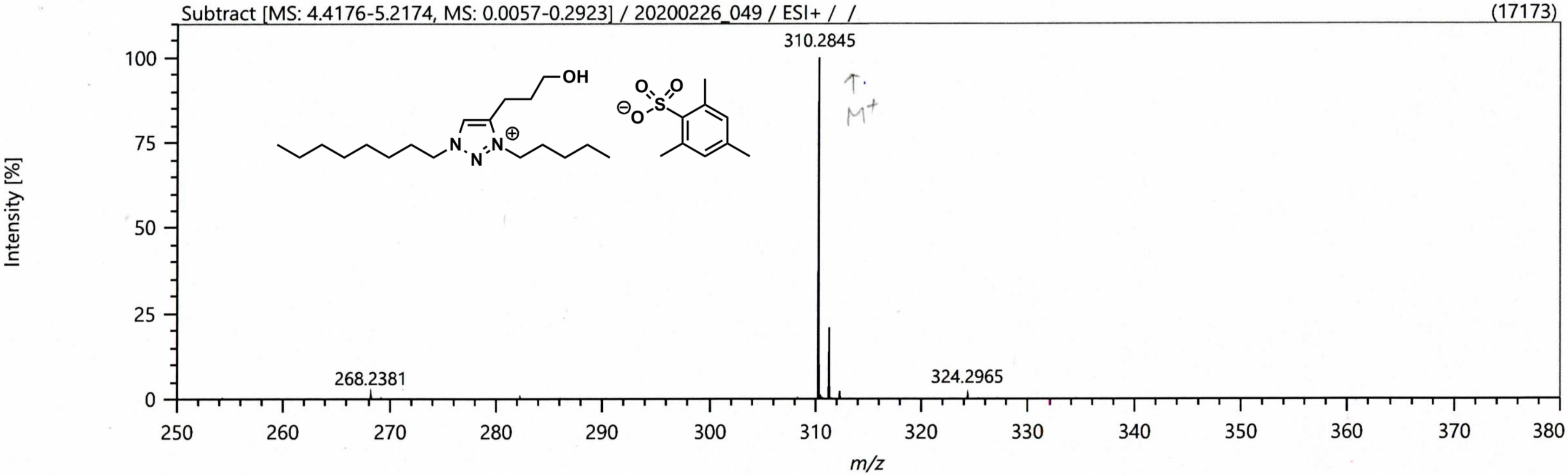

Elemental Composition

Parameters

Tolerance:	±10.00 ppm
Electron:	Odd/Even
Charge:	+1
DBE:	-99.0 - 999.0

Elements Set 1:

Symbol	C	H	O	Na	N	S	Ni	F
Min	0	0	1	0	3	0	0	0
Max	1000	4000	1	0	3	0	0	0

Results

Mass	Formula	Calculated Mass	Mass Difference [mDa]	Mass Difference [ppm]	DBE
310.28450	C18 H36 N3 O	310.28529	-0.79	-2.53	2.5

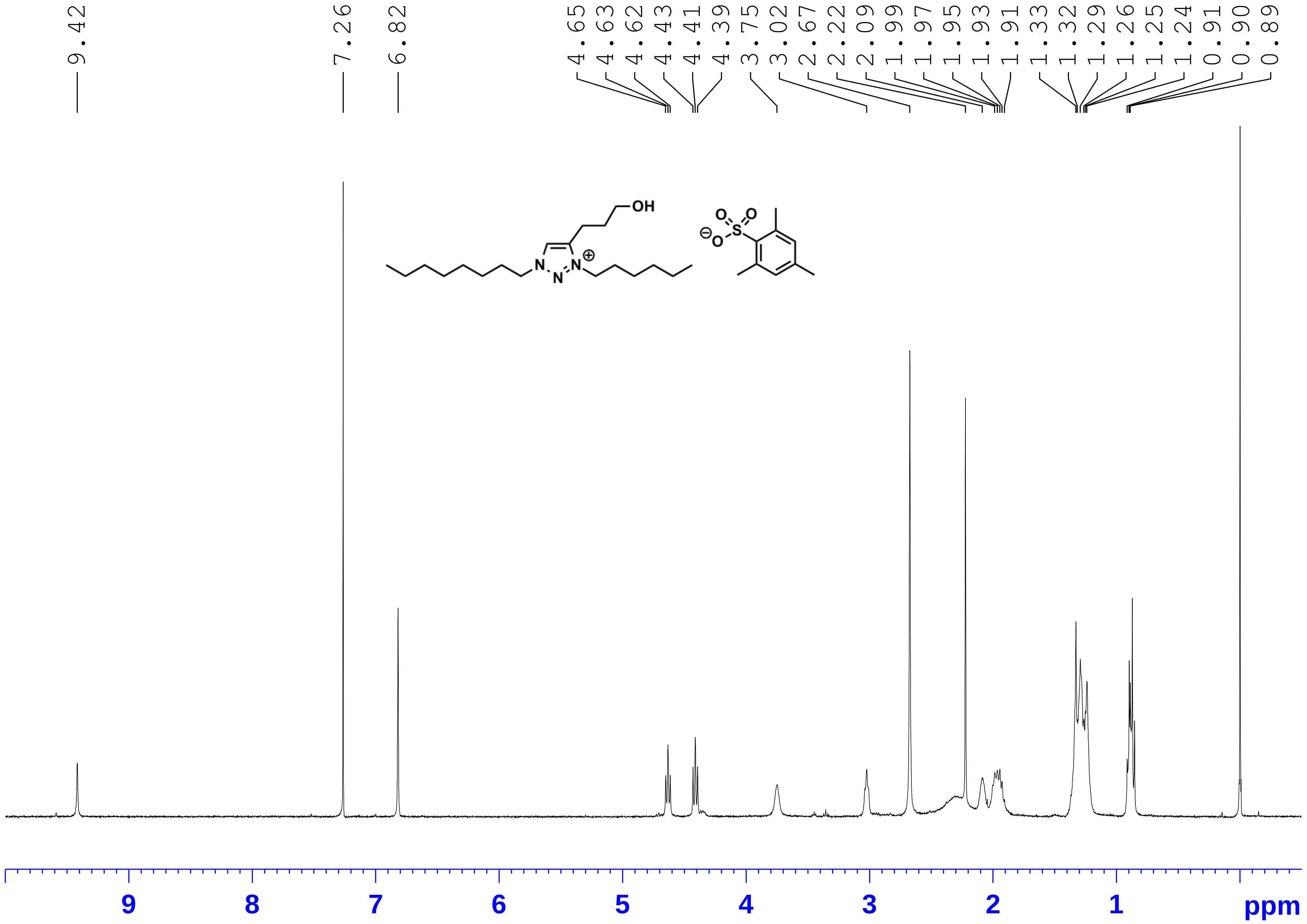
9.42
7.26
6.82
4.65
4.63
4.62
4.43
4.41
4.39
3.75
3.02
2.67
2.22
2.09
1.99
1.97
1.95
1.93
1.91
1.33
1.32
1.29
1.26
1.25
1.24
0.91
0.90
0.89
OH
N
N
N
O
O
O
S
9
8
7
6
5
4
3
2
1
ppm

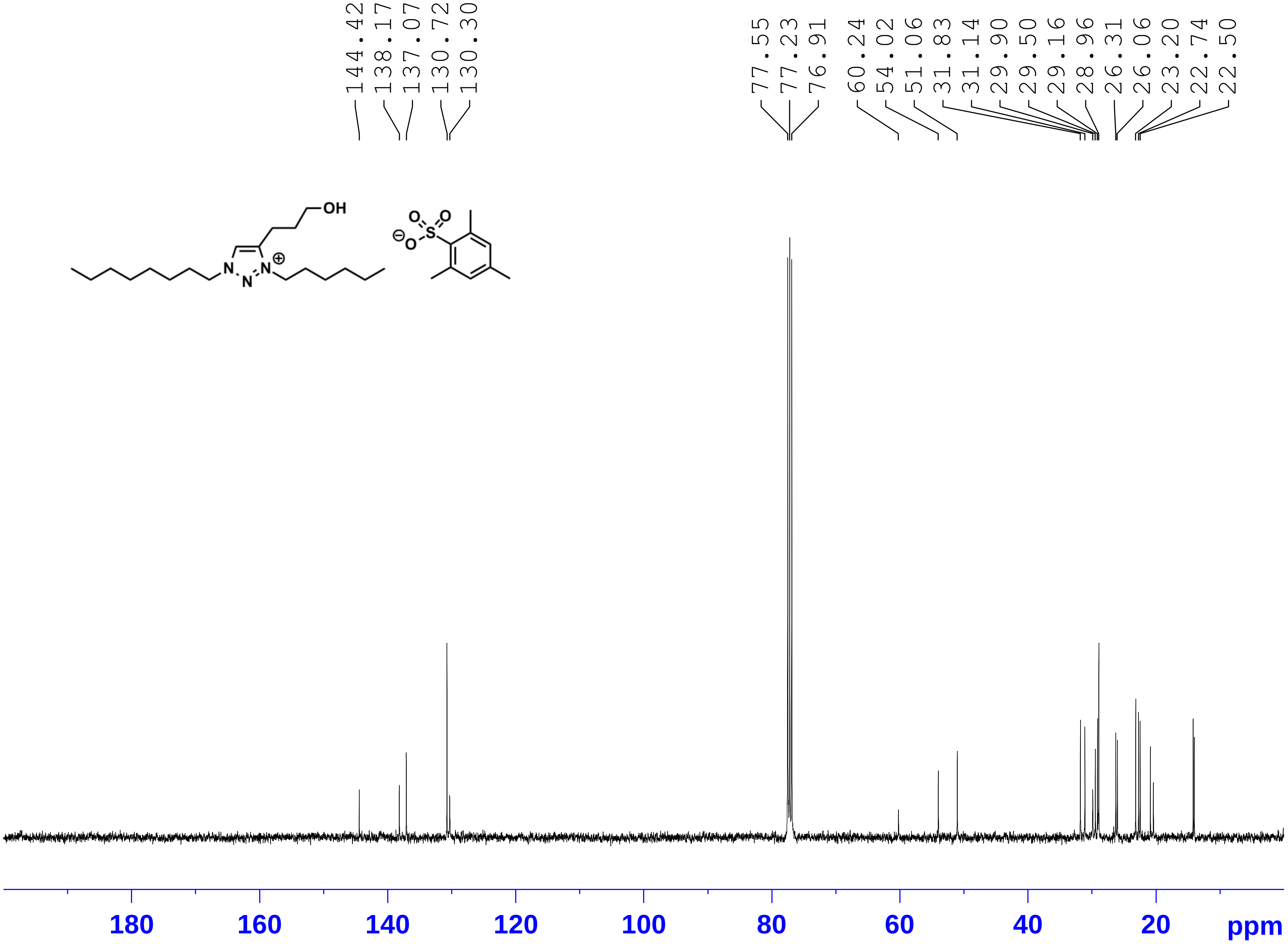
144.42
138.17
137.07
130.72
130.30
77.55
77.23
76.91
60.24
54.02
51.06
31.83
31.14
29.90
29.50
29.16
28.96
26.31
26.06
23.20
22.74
22.50
OH
N
N
N
O
O
S
O
180
160
140
120
100
80
60
40
20
ppm

Spectrum

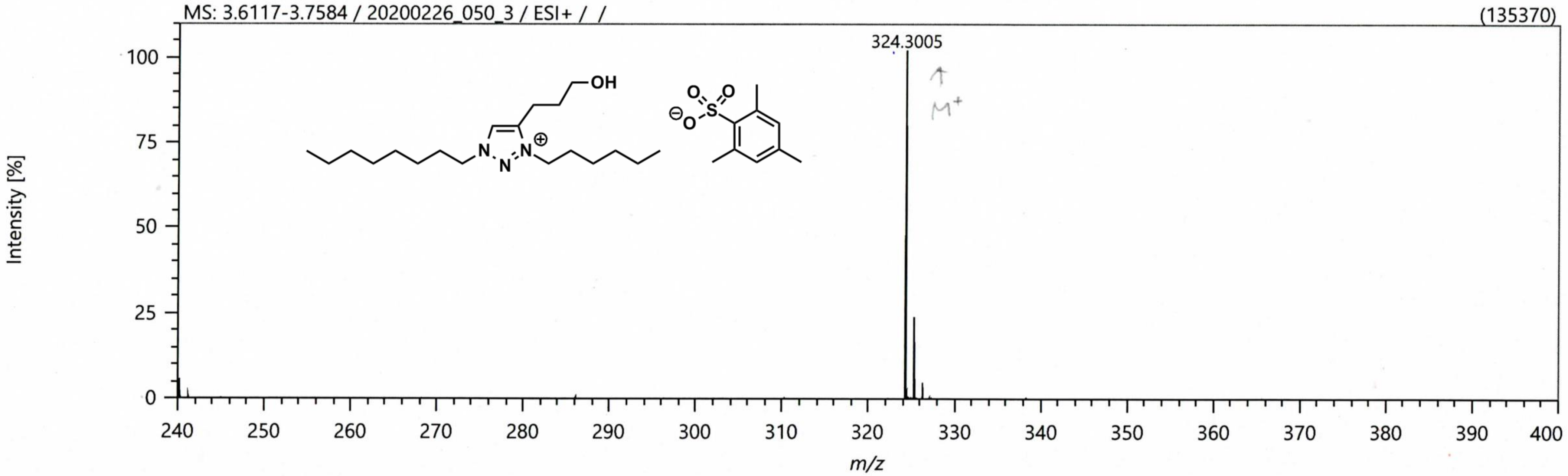

Elemental Composition

Parameters

Tolerance:	±10.00 ppm
Electron:	Odd/Even
Charge:	+1
DBE:	-99.0 - 999.0

Elements Set 1:

Symbol	C	H	O	Na	N	S	Ni	F
Min	0	0	1	0	3	0	0	0
Max	1000	4000	1	0	3	0	0	0

Results

Mass	Formula	Calculated Mass	Mass Difference [mDa]	Mass Difference [ppm]	DBE
324.30053	C19 H38 N3 O	324.30094	-0.41	-1.26	2.5

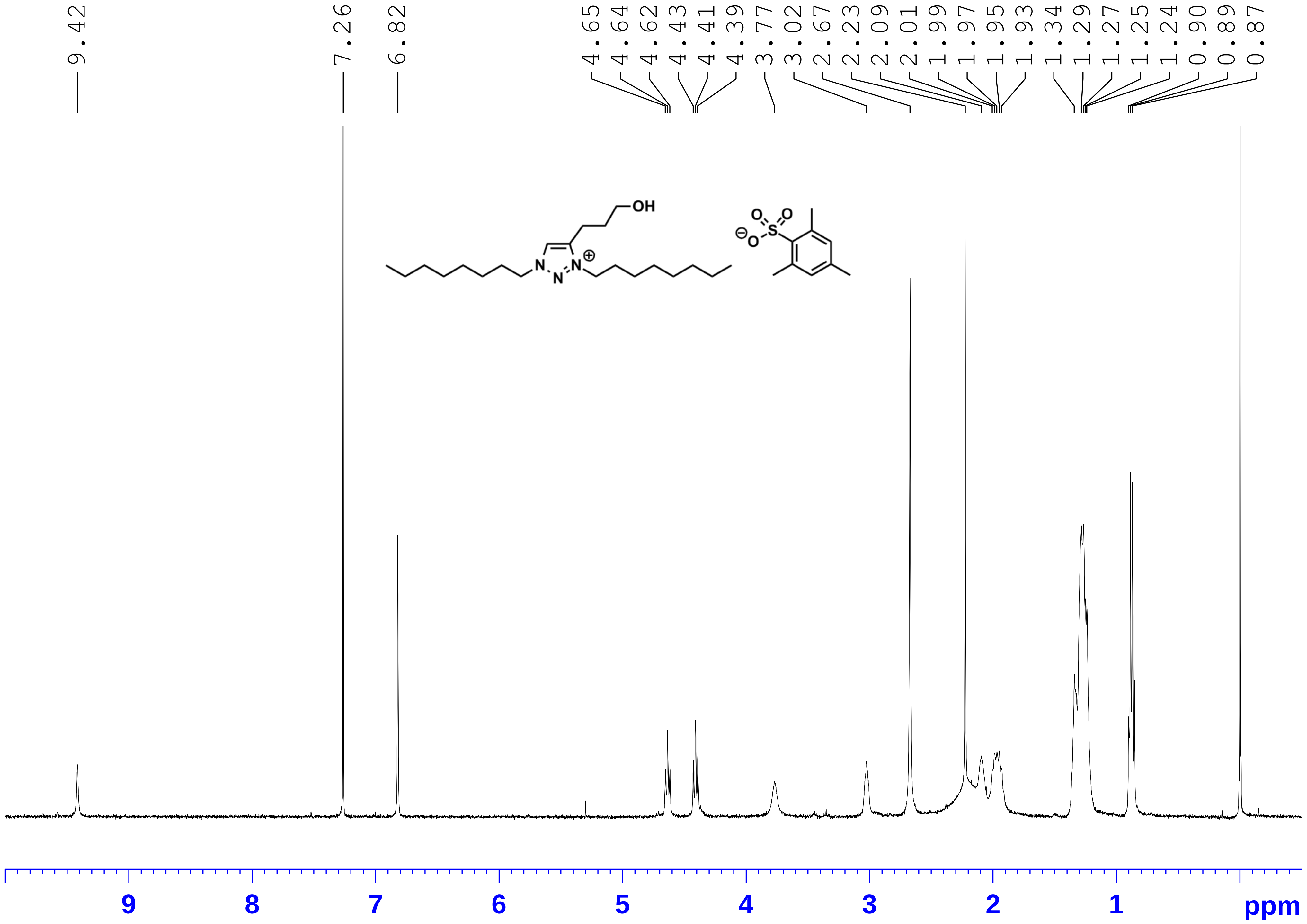

9.42
7.26
6.82
4.65
4.64
4.62
4.43
4.41
4.39
3.77
3.02
2.67
2.23
2.09
2.01
1.99
1.97
1.95
1.93
1.34
1.29
1.27
1.25
1.24
0.90
0.89
0.87
OH
N
N
N
O
S
O
O
9
8
7
6
5
4
3
2
1
ppm

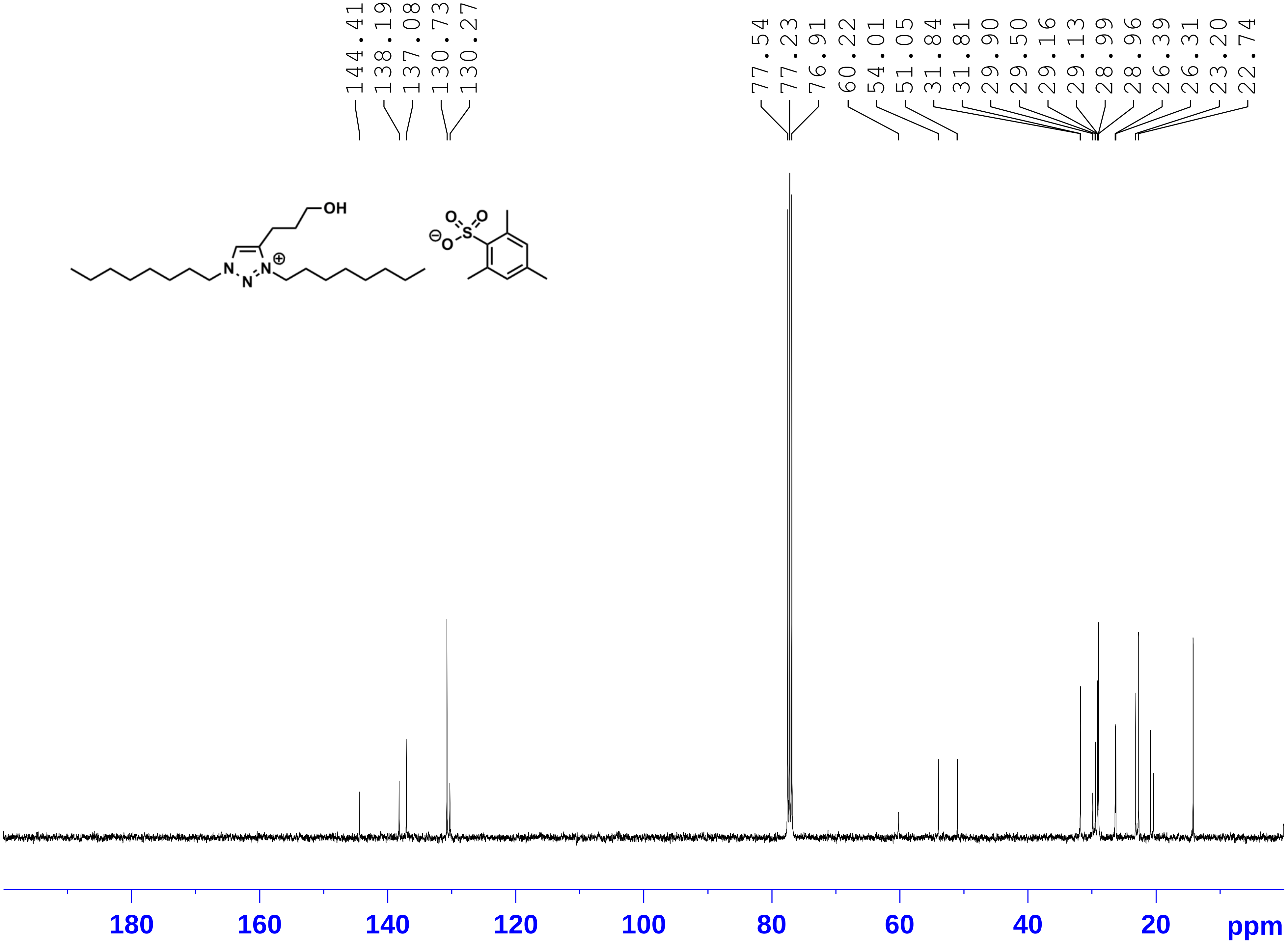
144.41
138.19
137.08
130.73
130.27
77.54
77.23
76.91
60.22
54.01
51.05
31.84
31.81
29.90
29.50
29.16
29.13
28.99
28.96
26.39
26.31
23.20
22.74
OH
N
N
N
S
O
O
O
180
160
140
120
100
80
60
40
20
ppm

Spectrum

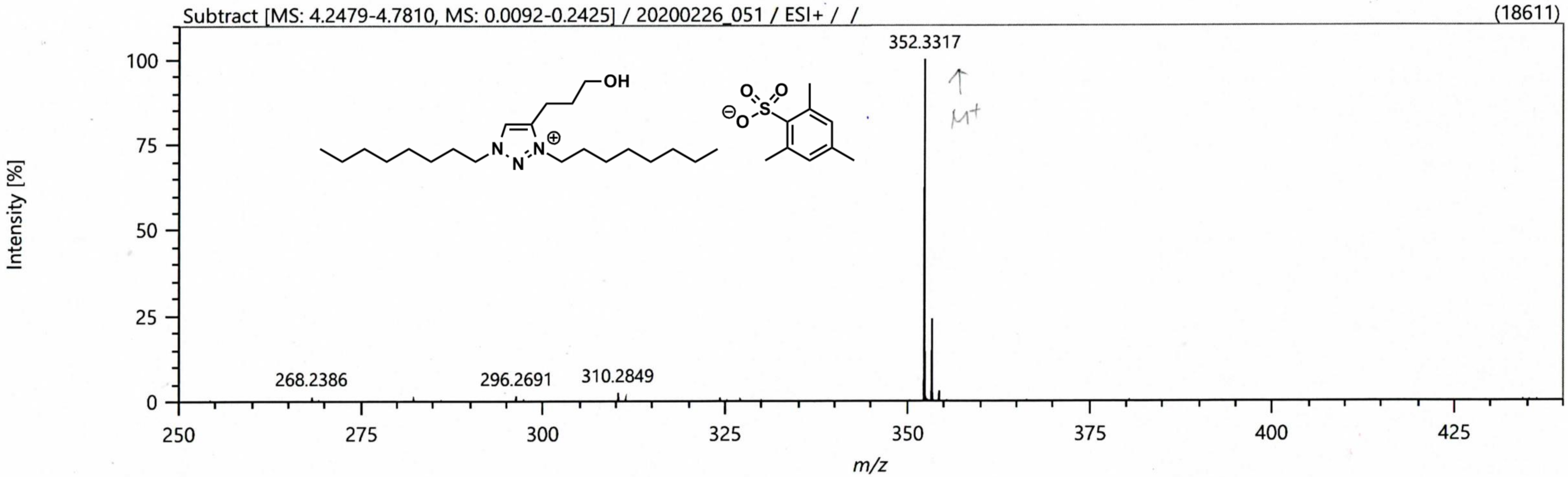

Elemental Composition

Parameters

Tolerance:	±10.00 ppm
Electron:	Odd/Even
Charge:	+1
DBE:	-99.0 - 999.0

Elements Set 1:

Symbol	C	H	O	Na	N	S	Ni	F
Min	0	0	1	0	3	0	0	0
Max	1000	4000	1	0	3	0	0	0

Results

Mass	Formula	Calculated Mass	Mass Difference [mDa]	Mass Difference [ppm]	DBE
352.33166	C21 H42 N3 O	352.33224	-0.58	-1.64	2.5

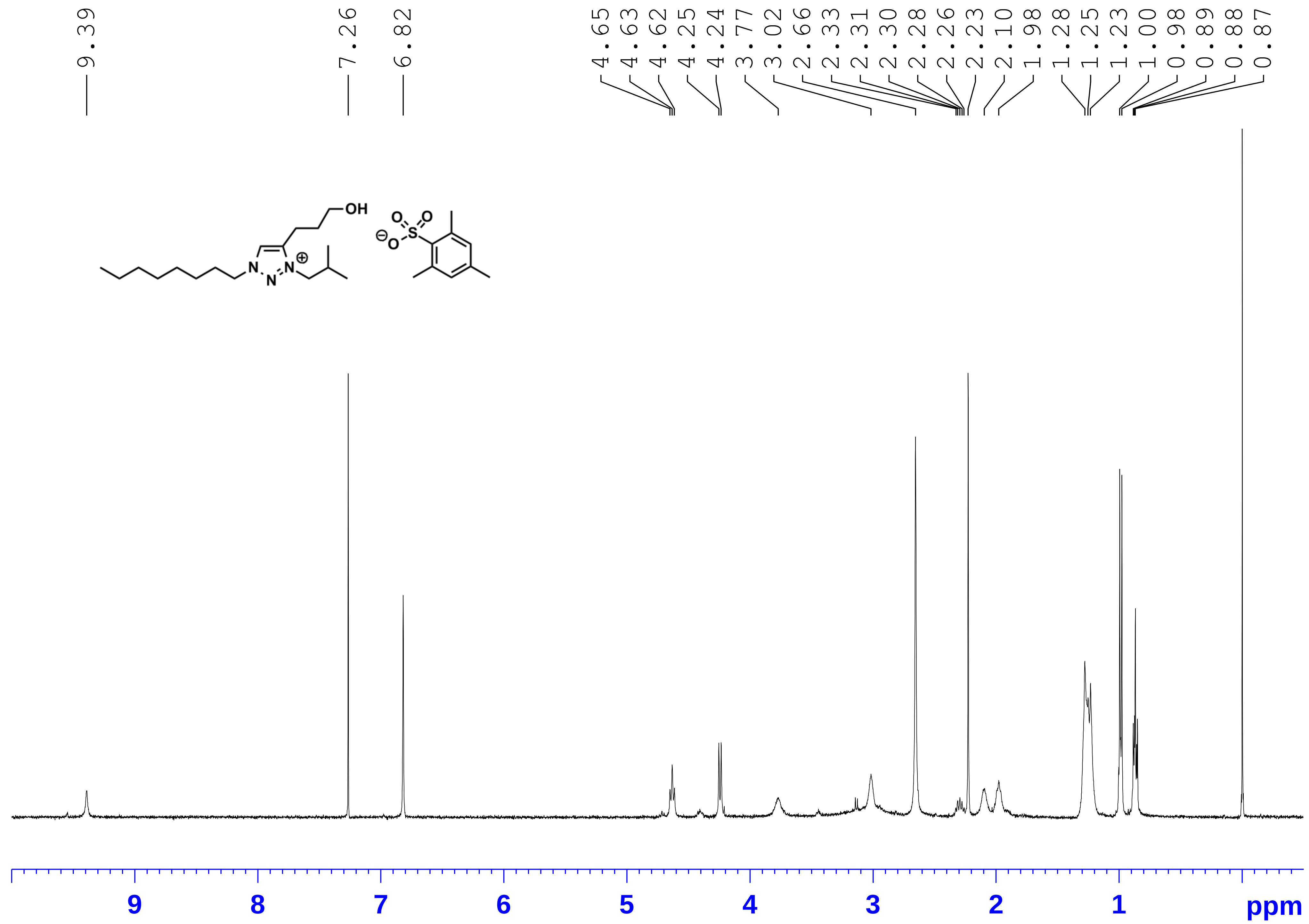

9.39
7.26
6.82
4.65
4.63
4.62
4.25
4.24
3.77
3.02
2.66
2.33
2.31
2.30
2.28
2.26
2.23
2.10
1.98
1.28
1.25
1.23
1.00
0.98
0.89
0.88
0.87
OH
N
N
N
O
S
O
O
9
8
7
6
5
4
3
2
1
ppm

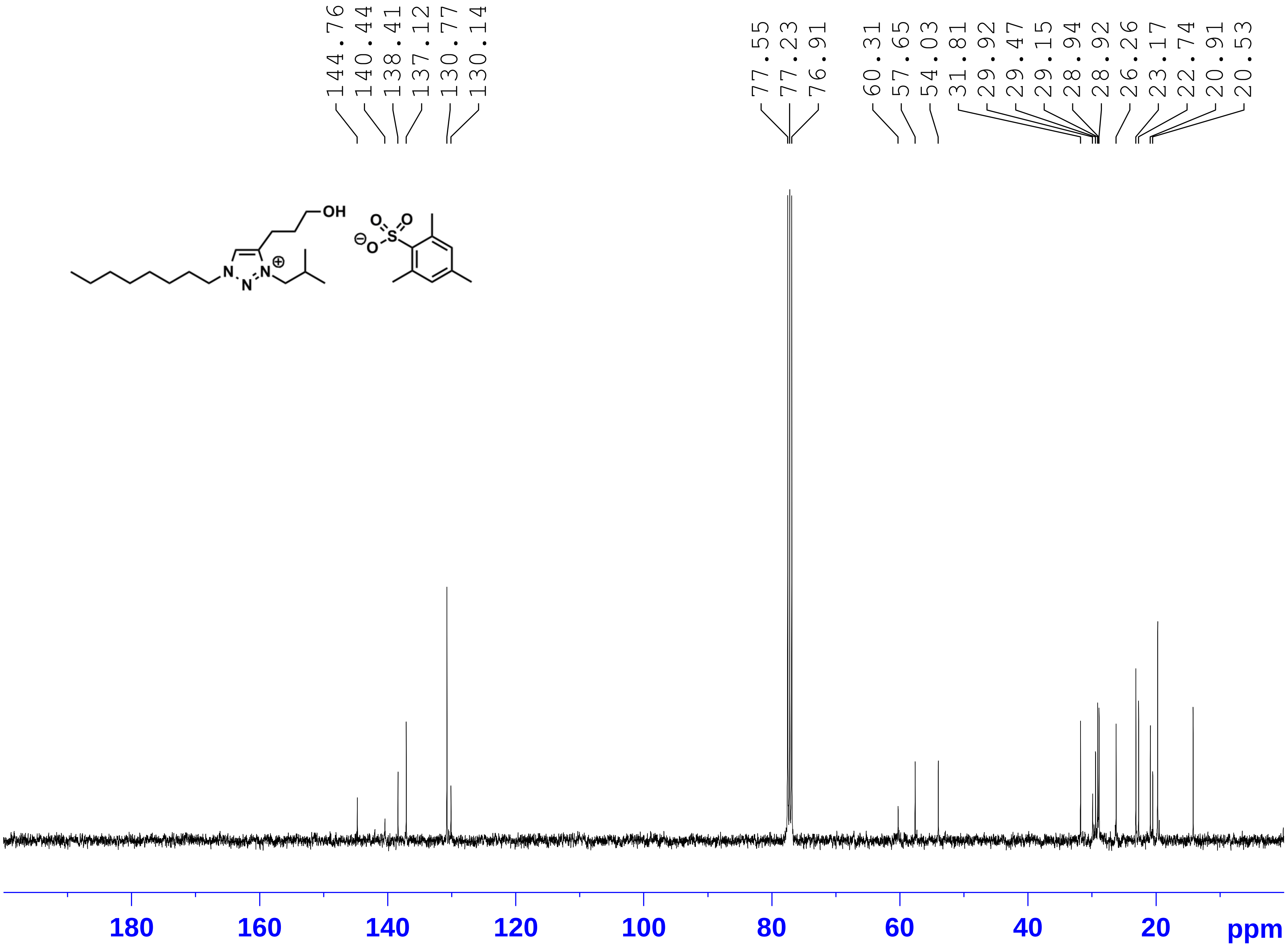
144.76
140.44
138.41
137.12
130.77
130.14
77.55
77.23
76.91
60.31
57.65
54.03
31.81
29.92
29.47
29.15
28.94
28.92
26.26
23.17
22.74
20.91
20.53
OH
O
S
N
180
160
140
120
100
80
60
40
20
ppm

Spectrum

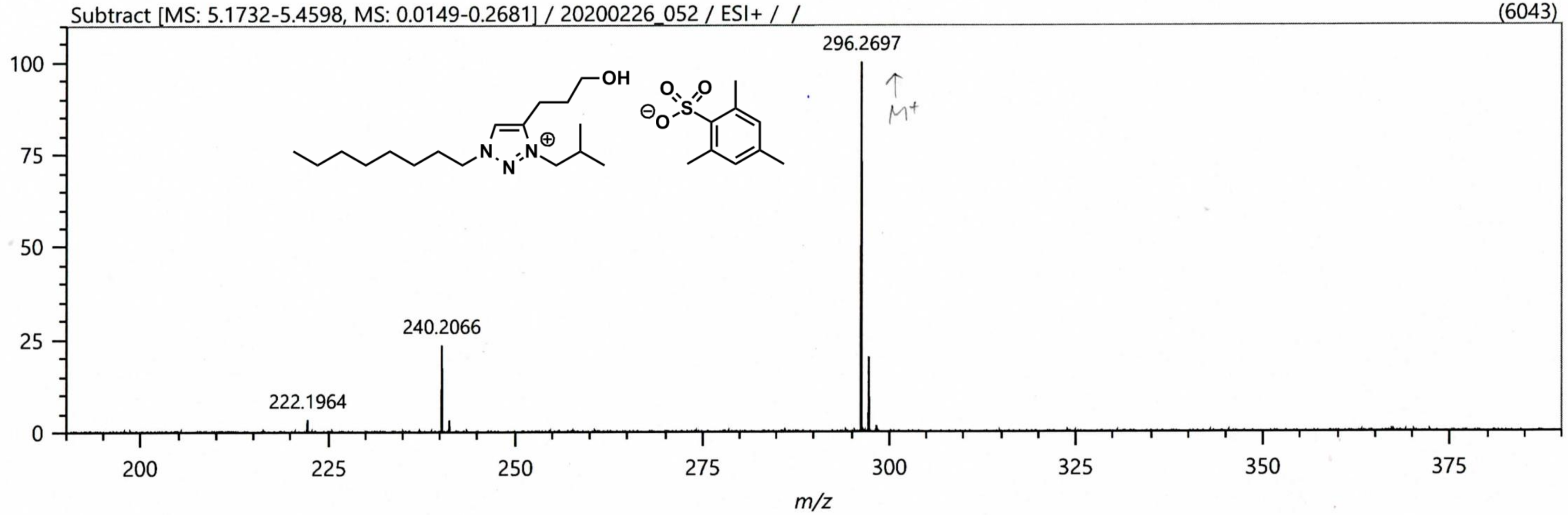

Elemental Composition

Parameters		Elements Set 1:								
Tolerance:	±10.00 ppm	Symbol	C	H	O	Na	N	S	Ni	F
Electron:	Odd/Even	Min	0	0	1	0	3	0	0	0
Charge:	+1	Max	1000	4000	1	0	3	0	0	0
DBE:	-99.0 - 999.0									

Results

Mass	Formula	Calculated Mass	Mass Difference [mDa]	Mass Difference [ppm]	DBE
296.26969	C17 H34 N3 O	296.26964	0.05	0.18	2.5
