# Supplementary material for: Combinatorial discovery of small-molecule 1,2,3-triazolium ionic liquids exhibiting lower critical solution temperature phase transition
Source: Sci Rep. 2020 Oct 26;10:18247. doi: 10.1038/s41598-020-75392-z (PMC7589527; doi:10.1038/s41598-020-75392-z)
Supplement: Supplementary file 4 — Supplementary Information 4. [file 41598_2020_75392_MOESM4_ESM.pdf]

Supporting Information (ESI-3) for [R_2-R_3-C4OH-tr][OTs] (**e1**-**e25**) and [R_2-R_3-C4OH-tr][OTMBS] (**f1**-**f26**) Ionic Liquids:

Combinatorial Discovery of Small-Molecule 1,2,3-Triazolium Ionic Liquids Exhibiting Lower Critical Solution Temperature Phase Transition

Yen-Ho Chu*, Mou-Fu Cheng and Yung-Hsin Chiang

* Corresponding author. E-mail: cheyhc@ccu.edu.tw

Department of Chemistry and Biochemistry, National Chung Cheng University, Chiayi 62102, Taiwan, Republic of China

Combinatorial Synthesis of a Library of 25 [R2-R3-C4OH-tr][OTs] Room-Temperature Ionic Liquids

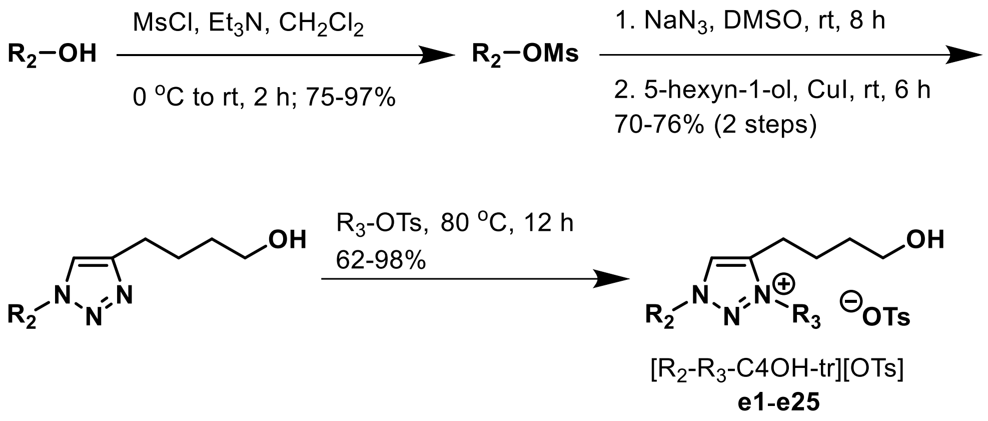

Generalized Procedure for the Synthesis of 1,4-Disubstituted 1,2,3-Triazoles (R_2-C4OH-tr)

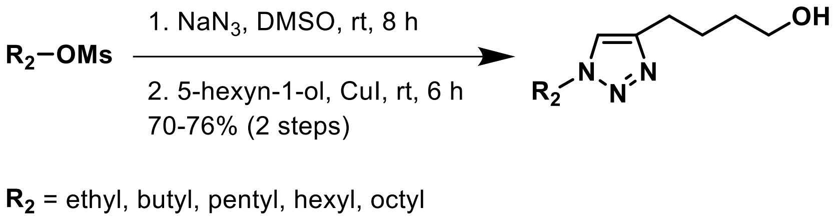

To a round-bottomed flask containing alkyl methanesulfonate (R_2-OMs, 6.6 mmol) in dimethyl sulfoxide (1.0 M) was added sodium azide (13.3 mmol, 2.0 equiv) and the reaction was allowed to proceed at room temperature for 8 h. The progress of reaction could be monitored by TLC. The resulting reaction mixture was then added copper(I) iodide (0.7 mmol, 0.1 equiv) and 5-hexyn-1-ol (7.9 mmol 1.2 equiv). The subsequent [3+2] cycloaddition reaction was proceeded for 6 h at room temperature. The progress of reaction again was readily monitored by TLC. After reaction completed, crude product was filtered and concentrated under reduced pressure. The residue was purified by column chromatography (ethyl acetate/hexane = 1:1 to 1:0) to finally afford the desired R_2-C4OH-tr.

Et-C4OH-tr: 70% yield, light yellow liquid; ^{1}H NMR (400 MHz, $CDCl_3$) δ 1.54 (t, *J* = 8.0 Hz, NCH_2CH_3, 3H), 1.61-1.68 (m, $CH{=}CCH_2CH_2$, 2H), 1.74-1.81 (m,

C*H*$_2$CH$_2$OH, 2H), 2.76 (t, *J* = 8.0 Hz, CH=CC*H*$_2$, 2H), 3.68 (t, *J* = 6.0 Hz, CH$_2$C*H*$_2$OH, 2H), 4.37 (q, *J* = 6.0 Hz, NC*H*$_2$CH$_3$, 2H), 7.29 (s, C*H*=CCH$_2$, 1H).

Bu-C4OH-tr: 72% yield, light yellow liquid; ^{1}H NMR (400 MHz, CDCl$_3$) δ 0.95 (t, *J* = 8.0 Hz, CH$_2$CH$_2$C*H*$_3$, 3H), 1.31-1.40 (m, CH$_2$C*H*$_2$CH$_3$, 2H), 1.61-1.68 (m, C*H*$_2$CH$_2$CH$_3$, 2H), 1.74-1.81 (m, CH=CCH$_2$C*H*$_2$, 2H), 1.83-1.91 (m, C*H*$_2$CH$_2$OH, 2H), 2.76 (t, *J* = 6.0 Hz, CH=CC*H*$_2$, 2H), 3.68 (t, *J* = 6.0 Hz, CH$_2$C*H*$_2$OH, 2H), 4.31 (t, *J* = 6.0 Hz, NC*H*$_2$CH$_2$, 2H), 7.27 (s, C*H*=CCH$_2$, 1H).

Pent-C4OH-tr: 73% yield, light yellow liquid; ^{1}H NMR (400 MHz, CDCl$_3$) δ 0.90 (t, *J* = 8.0 Hz, CH$_2$CH$_2$C*H*$_3$, 3H), 1.28-1.39 (m, 2 x CH$_2$, 4H), 1.61-1.65 (m, CH$_2$, 2H), 1.74-1.82 (m, CH=CCH$_2$C*H*$_2$, 2H), 1.85-1.92 (m, C*H*$_2$CH$_2$OH, 2H), 2.76 (t, *J* = 8.0 Hz, CH=CC*H*$_2$, 2H), 3.69 (q, *J* = 6.0 Hz, CH$_2$C*H*$_2$OH, 2H), 4.30 (t, *J* = 6.0 Hz, NC*H*$_2$CH$_2$, 2H), 7.27 (s, C*H*=CCH$_2$, 1H).

Hex-C4OH-tr: 76% yield, light yellow liquid; ^{1}H NMR (400 MHz, CDCl$_3$) δ 0.88 (t, *J* = 8.0 Hz, CH$_2$CH$_2$C*H*$_3$, 3H), 1.28-1.34 (m, 3 x CH$_2$, 6H), 1.61-1.68 (m, CH$_2$, 2H), 1.74-1.81 (m, CH=CCH$_2$C*H*$_2$, 2H), 1.84-1.91 (m, C*H*$_2$CH$_2$OH, 2H), 2.76 (t, *J* = 6.0 Hz, CH=CC*H*$_2$, 2H), 3.68 (q, *J* = 6.0 Hz, CH$_2$C*H*$_2$OH, 2H), 4.30 (t, *J* = 8.0 Hz, NC*H*$_2$CH$_2$, 2H), 7.27 (s, C*H*=CCH$_2$, 1H).

Oct-C4OH-tr: 75% yield, light yellow liquid; ^{1}H NMR (400 MHz, CDCl$_3$) δ 0.88 (t, *J* = 6.0 Hz, CH$_2$CH$_2$C*H*$_3$, 3H), 1.26-1.31 (m, 5 x CH$_2$, 10H), 1.61-1.65 (m, CH$_2$, 2H),

1.74-1.82 (m, CH=CCH$_2$C*H*$_2$, 2H), 1.84-1.91 (m, C*H*$_2$CH$_2$OH, 2H), 2.76 (t, *J* = 8.0 Hz, CH=CC*H*$_2$, 2H), 3.68 (t, *J* = 6.0 Hz, CH$_2$C*H*$_2$OH, 2H), 4.30 (t, *J* = 8.0 Hz, NC*H*$_2$, 2H), 7.27 (s, C*H*=CCH$_2$, 1H).

Generalized Procedure for the Synthesis of [R$_2$-R$_3$-C4OH-tr][OTs] Ionic Liquids

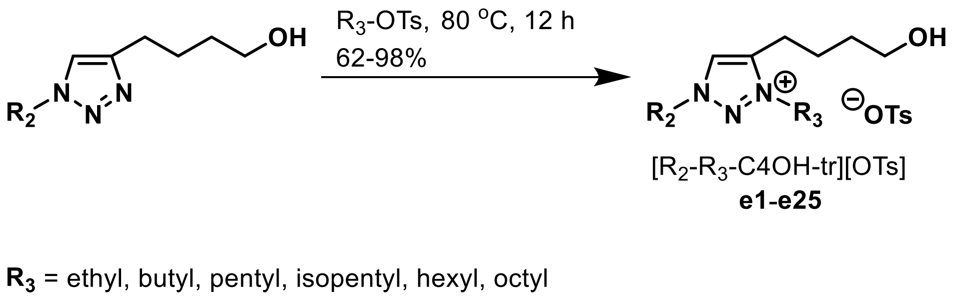

To a round-bottomed flask containing R$_2$-C4OH-tr (0.5 mmol) was added alkyl 4-methylbenzenesulfonate (R$_3$-OTs, 0.7 mmol, 1.3 equiv). The mixture was heated at 80 °C for 12 h. The progress of the ionic liquid forming reaction could be readily monitored by TLC. After reaction was completed, the reaction mixture was washed with ether (3 mL, 3 times) and then with *n*-hexane (3 mL, 3 times) to finally afford the desired [R$_2$-R$_3$-C4OH-tr][OTs] as room-temperature ionic liquids (**e1-e25**).

[Et-Et-C4OH-tr][OTs] (e1): 96% yield, light yellow liquid; ^{1}H NMR (400 MHz, CDCl$_3$) δ 1.55-1.71 (m, 2 x CH$_3$ + CH=CCH$_2$C*H*$_2$, 8H), 1.94-2.01 (m, C*H*$_2$CH$_2$OH, 2H),

2.34 (s, *p*-CH_3, 3H), 2.90 (t, *J* = 8.0 Hz, CH=CC*H*$_2$, 2H), 3.43 (t, *J* = 6.0 Hz, CH_2C*H*$_2$OH, 2H), 4.45 (q, *J* = 8.0 Hz, CNC*H*$_2$$CH_3$, 2H), 4.63 (q, *J* = 6.0 Hz, CHNC*H*$_2$$CH_3$, 2H), 7.14 (d, *J* = 8.0 Hz, aryl H, 2H), 7.71 (d, *J* = 8.0 Hz, aryl H, 2H), 9.04 (s, C*H*=CCH_2, 1H); ^{13}C NMR (100 MHz, $CDCl_3$) δ 14.01, 14.40, 21.47, 22.95, 23.49, 31.12, 46.53, 49.44, 61.39, 126.13, 128.96, 129.12, 140.46, 141.82, 144.24; EI-HRMS *m/z* [M^+] calcd for $C_{10}H_{20}N_3O$ 198.1606, found 198.1597.

[Et-Bu-C4OH-tr][OTs] (e2): 98% yield, light yellow liquid; ^{1}H NMR (400 MHz, $CDCl_3$) δ 0.96 (t, *J* = 8.0 Hz, CH_2CH_2C*H*$_3$, 3H), 1.32-1.40 (m, CH_2C*H*$_2$$CH_3$, 2H), 1.60 (t, *J* = 8.0 Hz, NCH_2C*H*$_3$, 3H), 1.66-1.72 (m, CH=CCH_2C*H*$_2$, 2H), 1.85-1.92 (m, C*H*$_2$$CH_2CH_3$, 2H), 1.94-2.02 (m, C*H*$_2$$CH_2$OH, 2H), 2.34 (s, *p*-C*H*$_3$, 3H), 2.88 (t, *J* = 8.0 Hz, CH=CC*H*$_2$, 2H), 3.69 (t, *J* = 6.0 Hz, CH_2C*H*$_2$OH, 2H), 4.38 (t, *J* = 6.0 Hz, NC*H*$_2$$CH_2$, 2H), 4.65 (q, *J* = 8.0 Hz, NC*H*$_2$$CH_3$, 2H), 7.14 (d, *J* = 8.0 Hz, aryl *H*, 2H), 7.72 (d, *J* = 8.0 Hz, aryl *H*, 2H), 9.14 (s, C*H*=CCH_2, 1H); ^{13}C NMR (100 MHz, $CDCl_3$) δ 13.52, 14.45, 19.69, 21.46, 23.12, 23.56, 30.79, 31.29, 49.48, 50.82, 61.33, 126.07, 128.90, 129.29, 140.08, 142.50, 144.39; EI-HRMS *m/z* [M^+] calcd for $C_{12}H_{24}N_3O$ 226.1919, found 226.1910.

[Et-Pent-C4OH-tr][OTs] (e3): 97% yield, light yellow liquid; ^{1}H NMR (400 MHz, $CDCl_3$) δ 0.90 (t, *J* = 6.0 Hz, CH_2CH_2C*H*$_3$, 3H), 1.28-1.38 (m, 2 x CH_2, 4H), 1.60 (t, *J* = 6.0 Hz, NCH_2C*H*$_3$, 3H), 1.66-1.72 (m, CH=CCH_2C*H*$_2$, 2H), 1.87-2.02 (m, NCH_2C*H*$_2$

+ CH_2CH$_2$OH, 4H), 2.34 (s, *p*-CH$_3$, 3H), 2.88 (t, J = 6.0 Hz, CH=CCH_2, 2H), 3.69 (t, J = 6.0 Hz, CH$_2$CH_2OH, 2H), 4.37 (t, J = 8.0 Hz, NCH_2CH$_2$, 2H), 4.65 (q, J = 8.0 Hz, NCH_2CH$_3$, 2H), 7.14 (d, J = 8.0 Hz, aryl H, 2H), 7.72 (d, J = 8.0 Hz, aryl H, 2H), 9.15 (s, CH=CCH$_2$, 1H); ^{13}C NMR (100 MHz, CDCl$_3$) δ 13.90, 14.49, 21.47, 22.13, 23.12, 23.57, 28.45, 28.58, 31.29, 49.49, 51.07, 61.34, 126.12, 128.90, 129.37, 140.15, 142.35, 144.39; EI-HRMS *m/z* [M$^+$] calcd for $C_{13}H_{26}N_3O$ 240.2076, found 240.2064.

[Et-Hex-C4OH-tr][OTs] (e4): 84% yield, light yellow liquid; ^{1}H NMR (400 MHz, CDCl$_3$) δ 0.89 (t, J = 4.0 Hz, CH$_2$CH$_2$CH_3, 3H), 1.28-1.36 (m, 3 x CH$_2$, 6H), 1.62 (t, J = 6.0 Hz, NCH$_2$CH_3, 3H), 1.68-1.73 (m, CH=CCH$_2$CH_2, 2H), 1.87-1.93 (m, NCH$_2$CH_2, 2H), 1.96-2.03 (m, CH_2CH$_2$OH, 2H), 2.34 (s, *p*-CH$_3$, 3H), 2.89 (t, J = 8.0 Hz, CH=CCH_2, 2H), 3.70 (t, J = 6.0 Hz, CH$_2$CH_2OH, 2H), 4.37 (t, J = 8.0 Hz, NCH_2CH$_2$, 2H), 4.67 (q, J = 8.0 Hz, NCH_2CH$_3$, 2H), 7.14 (d, J = 8.0 Hz, aryl H, 2H), 7.73 (d, J = 8.0 Hz, aryl H, 2H), 9.19 (s, CH=CCH$_2$, 1H); ^{13}C NMR (100 MHz, CDCl$_3$) δ 14.03, 14.47, 21.45, 22.46, 23.08, 23.54, 26.04, 28.81, 31.12, 31.28, 49.44, 51.06, 61.30, 126.08, 128.87, 129.29, 140.07, 142.48, 144.37; EI-HRMS *m/z* [M$^+$] calcd for $C_{14}H_{28}N_3O$ 254.2232, found 254.2225.

[Et-Oct-C4OH-tr][OTs] (e5): 87% yield, light yellow liquid; ^{1}H NMR (400 MHz, CDCl$_3$) δ 0.88 (t, J = 8.0 Hz, CH$_2$CH$_2$CH_3, 3H), 1.26-1.34 (m, 5 x CH$_2$, 10H), 1.64 (t, J = 8.0 Hz, NCH$_2$CH_3, 3H), 1.68-1.74 (m, CH=CCH$_2$CH_2, 2H), 1.89-1.96 (m,

NCH_2CH_2, 2H), 1.98-2.06 (m, CH_2CH_2OH, 2H), 2.34 (s, *p*-CH_3, 3H), 2.90 (t, *J* = 8.0 Hz, CH=CCH_2, 2H), 3.71 (t, *J* = 6.0 Hz, CH_2CH_2OH, 2H), 4.38 (t, *J* = 6.0 Hz, NCH_2CH_2, 2H), 4.70 (q, *J* = 8.0 Hz, NCH_2CH_3, 2H), 7.15 (d, *J* = 8.0 Hz, aryl H, 2H), 7.74 (d, *J* = 8.0 Hz, aryl H, 2H), 9.25 (s, CH=CCH_2, 1H); ^{13}C NMR (100 MHz, $CDCl_3$) δ 14.21, 14.52, 21.47, 22.72, 23.12, 23.58, 26.41, 28.90, 28.99, 29.11, 31.34, 31.81, 49.49, 51.09, 61.30, 126.11, 128.88, 129.44, 140.04, 142.55, 144.39; EI-HRMS *m/z* [M^+] calcd for $C_{16}H_{32}N_3O$ 282.2545, found 282.2535.

[Bu-Et-C4OH-tr][OTs] (e6): 98% yield, light yellow liquid; ^{1}H NMR (400 MHz, $CDCl_3$) δ 0.91 (t, *J* = 6.0 Hz, $CH_2CH_2CH_3$, 3H), 1.30-1.37 (m, $CH_2CH_2CH_3$, 2H), 1.58 (t, *J* = 6.0 Hz, NCH_2CH_3 , 3H), 1.65-1.74 (m, CH=CCH_2CH_2, 2H), 1.87-2.01 (m, NCH_2CH_2 + CH_2CH_2OH, 4H), 2.34 (s, *p*-CH_3, 3H), 2.90-2.94 (m, CH=CCH_2, 2H), 3.71 (t, *J* = 6.0 Hz, CH_2CH_2OH, 2H), 4.47 (q, *J* = 6.0 Hz, NCH_2CH_3, 2H), 4.57 (t, *J* = 8.0 Hz, NCH_2CH_2, 2H), 7.14 (d, *J* = 8.0 Hz, aryl H, 2H), 7.72 (d, *J* = 8.0 Hz, aryl H, 2H), 9.06 (s, CH=CCH_2, 1H); ^{13}C NMR (100 MHz, $CDCl_3$) δ 13.44, 14.00, 19.55, 21.45, 22.99, 23.53, 31.20, 31.23, 46.50, 53.74, 61.35, 126.05, 128.94, 129.32, 140.15, 142.42, 144.30; EI-HRMS *m/z* [M^+] calcd for $C_{12}H_{24}N_3O$ 226.1919, found 226.1911.

[Bu-Bu-C4OH-tr][OTs] (e7): 89% yield, light yellow liquid; ^{1}H NMR (400 MHz, $CDCl_3$) δ 0.92-1.00 (m, 2 x CH_3 , 6H), 1.31-1.42 (m, 2 x CH_2CH_3 , 4H), 1.67-1.74 (m, CH=CCH_2CH_2, 2H), 1.88-2.06 (m, 2 x NCH_2CH_2 + CH_2CH_2OH, 6H), 2.34 (s, *p*-CH_3,

3H), 2.91 (t, *J* = 8.0 Hz, CH=CC*H*$_2$, 2H), 3.71 (t, *J* = 6.0 Hz, CH$_2$C*H*$_2$OH, 2H), 4.40 (t, *J* = 6.0 Hz, CNC*H*$_2$, 2H), 4.63 (t, *J* = 6.0 Hz, CHNC*H*$_2$, 2H), 7.15 (d, *J* = 8.0 Hz, aryl *H*, 2H), 7.75 (d, *J* = 8.0 Hz, aryl *H*, 2H), 9.24 (s, C*H*=CCH$_2$, 1H); ^{13}C NMR (100 MHz, CDCl$_3$) δ 13.44, 13.51, 19.53, 19.65, 21.44, 23.13, 23.63, 30.79, 31.25, 31.40, 50.79, 53.77, 61.26, 126.05, 128.85, 129.59, 139.86, 142.92, 144.44; EI-HRMS *m/z* [M$^+$] calcd for $C_{14}H_{28}N_3O$ 254.2232, found 254.2223.

[Bu-Pent-C4OH-tr][OTs] (e8): 94% yield, light yellow liquid; ^{1}H NMR (400 MHz, CDCl$_3$) δ 0.89-0.94 (m, 2 x CH$_3$, 6H), 1.28-1.37 (m, 3 x CH$_2$, 6H), 1.66-1.73 (m, CH=CCH$_2$C*H*$_2$, 2H), 1.88-2.01 (m, 2 x NCH$_2$C*H*$_2$ + C*H*$_2$CH$_2$OH, 6H), 2.34 (s, *p*-CH$_3$, 3H), 2.90 (t, *J* = 8.0 Hz, CH=CC*H*$_2$, 2H), 3.70 (t, *J* = 6.0 Hz, CH$_2$C*H*$_2$OH, 2H), 4.39 (t, *J* = 6.0 Hz, CNC*H*$_2$, 2H), 4.60 (t, *J* = 8.0 Hz, CHNC*H*$_2$, 2H), 7.14 (d, *J* = 8.0 Hz, aryl H, 2H), 7.73 (d, *J* = 8.0 Hz, aryl H, 2H), 9.18 (s, C*H*=CCH$_2$, 1H); ^{13}C NMR (100 MHz, CDCl$_3$) δ 13.45, 13.91, 19.55, 21.47, 22.13, 23.14, 23.62, 28.44, 28.58, 31.28, 31.31, 51.06, 53.81, 61.35, 126.14, 128.89, 129.71, 140.08, 142.47, 144.43; EI-HRMS *m/z* [M$^+$] calcd for $C_{15}H_{30}N_3O$ 268.2389, found 268.2376.

[Bu-Hex-C4OH-tr][OTs] (e9): 91% yield, light yellow liquid; ^{1}H NMR (400 MHz, CDCl$_3$) δ 0.87-0.94 (m, 2 x CH$_3$, 6H), 1.28-1.36 (m, 4 x CH$_2$, 8H), 1.67-1.73 (m, CH=CCH$_2$C*H*$_2$, 2H), 1.85-2.02 (m, 2 x NCH$_2$C*H*$_2$ + C*H*$_2$CH$_2$OH, 6H), 2.34 (s, *p*-C*H*$_3$, 3H), 2.90 (t, *J* = 8.0 Hz, CH=CC*H*$_2$, 2H), 3.70 (t, *J* = 6.0 Hz, CH$_2$C*H*$_2$OH, 2H), 4.38 (t,

J = 6.0 Hz, CNCH_2, 2H), 4.61 (t, J = 8.0 Hz, CHNCH_2, 2H), 7.15 (d, J = 8.0 Hz, aryl *H*, 2H), 7.73 (d, J = 8.0 Hz, aryl *H*, 2H), 9.19 (s, C*H*=CCH_2, 1H); ^{13}C NMR (100 MHz, $CDCl_3$) δ 13.43, 14.03, 19.54, 21.45, 22.49, 23.15, 23.62, 26.04, 28.81, 31.13, 31.25, 31.39, 51.04, 53.79, 61.30, 126.07, 128.87, 129.64, 139.91, 142.82, 144.42; EI-HRMS *m/z* [M^+] calcd for $C_{16}H_{32}N_3O$ 282.2545, found 282.2532.

[Bu-Oct-C4OH-tr][OTs] (e10): 83% yield, light yellow liquid; ^{1}H NMR (400 MHz, $CDCl_3$) δ 0.86-0.94 (m, 2 x CH_3 , 6H), 1.26-1.35 (m, 6 x CH_2, 12H), 1.66-1.73 (m, CH=CCH_2CH_2, 2H), 1.87-2.02 (m, 2 x NCH_2CH_2 + CH_2CH_2OH, 6H), 2.34 (s, *p*-CH_3, 3H), 2.90 (t, J = 8.0 Hz, CH=CCH_2, 2H), 3.70 (t, J = 6.0 Hz, CH_2CH_2OH, 2H), 4.38 (t, J = 6.0 Hz, CNCH_2, 2H), 4.61 (t, J = 6.0 Hz, CHNCH_2, 2H), 7.14 (d, J = 8.0 Hz, aryl H, 2H), 7.73 (d, J = 8.0 Hz, aryl H, 2H), 9.19 (s, C*H*=CCH_2, 1H); ^{13}C NMR (100 MHz, $CDCl_3$) δ 13.47, 14.22, 19.58, 21.49, 22.74, 23.18, 23.64, 26.41, 28.91, 28.99, 29.14, 31.31, 31.34, 31.81, 51.08, 53.85, 61.35, 126.15, 128.89, 129.81, 140.08, 142.48, 144.42; EI-HRMS *m/z* [M^+] calcd for $C_{18}H_{36}N_3O$ 310.2858, found 310.2845.

[Pent-Et-C4OH-tr][OTs] (e11): 85% yield, light yellow liquid; ^{1}H NMR (400 MHz, $CDCl_3$) δ 0.87 (t, J = 6.0 Hz, CH_3 , 3H), 1.27-1.35 (m, 2 x CH_2, 4H), 1.59 (t, J = 6.0 Hz, CH_3 , 3H), 1.68-1.75 (m, CH=CCH_2CH_2, 2H), 1.94-2.02 (m, NCH_2CH_2 + CH_2CH_2OH, 4H), 2.34 (s, *p*-CH_3, 3H), 2.93 (t, J = 8.0 Hz, CH=CCH_2, 2H), 3.72 (t, J = 6.0 Hz, CH_2CH_2OH, 2H), 4.48 (q, J = 6.0 Hz, CNCH_2, 2H), 4.57 (t, J = 6.0 Hz, CHNCH_2, 2H),

7.15 (d, J = 8.0 Hz, aryl H, 2H), 7.72 (d, J = 8.0 Hz, aryl H, 2H), 9.07 (s, CH=CCH_2, 1H); ^{13}C NMR (100 MHz, $CDCl_3$) δ 13.93, 14.09, 21.50, 22.10, 23.03, 23.56, 28.37, 29.06, 31.17, 46.54, 54.06, 61.42, 126.18, 128.96, 129.53, 140.42, 141.89, 144.28; EI-HRMS m/z [M^+] calcd for $C_{13}H_{26}N_3O$ 240.2076, found 240.2065.

[Pent-Bu-C4OH-tr][OTs] (e12): 95% yield, light yellow liquid; ^1H NMR (400 MHz, $CDCl_3$) δ 0.86 (t, J = 6.0 Hz, CH_3, 3H), 0.97 (t, J = 8.0 Hz, CH_3, 3H), 1.25-1.40 (m, 3 x CH_2, 6H), 1.66-1.73 (m, CH=CCH_2CH_2, 2H), 1.88-2.01 (m, 2 x NCH_2CH_2 + CH_2CH_2OH, 6H), 2.34 (s, p-CH_3, 3H), 2.90 (t, J = 8.0 Hz, CH=CCH_2, 2H), 3.70 (t, J = 6.0 Hz, CH_2CH_2OH, 2H), 4.40 (t, J = 6.0 Hz, $CNCH_2$, 2H), 4.58 (t, J = 8.0 Hz, $CHNCH_2$, 2H), 7.14 (d, J = 8.0 Hz, aryl H, 2H), 7.73 (d, J = 8.0 Hz, aryl H, 2H), 9.16 (s, CH=CCH_2, 1H); ^{13}C NMR (100 MHz, $CDCl_3$) δ 13.52, 13.94, 19.67, 21.47, 22.08, 23.14, 23.62, 28.34, 29.10, 30.82, 31.23, 50.83, 54.04, 61.31, 126.13, 128.86, 129.74, 140.00, 142.61, 144.43; EI-HRMS m/z [M^+] calcd for $C_{15}H_{30}N_3O$ 268.2389, found 268.2378.

[Pent-Pent-C4OH-tr][OTs] (e13): 87% yield, light yellow liquid; ^1H NMR (400 MHz, $CDCl_3$) δ 0.85-0.93 (m, 2 x CH_3 , 6H), 1.26-1.37 (m, 4 x CH_2, 8H), 1.66-1.72 (m, CH=CCH_2CH_2, 2H), 1.89-2.04 (m, 2 x NCH_2CH_2 + CH_2CH_2OH, 6H), 2.34 (s, p-CH_3, 3H), 2.90 (t, J = 8.0 Hz, CH=CCH_2, 2H), 3.69 (t, J = 6.0 Hz, CH_2CH_2OH, 2H), 4.39 (t, J = 5.0 Hz, $CNCH_2$, 2H), 4.60 (t, J = 8.0 Hz, $CHNCH_2$, 2H), 7.14 (d, J = 8.0 Hz, aryl H, 2H), 7.74 (d, J = 8.0 Hz, aryl H, 2H), 9.19 (s, CH=CCH_2, 1H); ^{13}C NMR (100 MHz,

$CDCl_3$) δ 13.91, 13.92, 21.45, 22.08, 22.13, 23.17, 23.65, 28.34, 28.43, 28.57, 29.07, 31.47, 51.01, 54.03, 61.26, 126.06, 128.85, 129.62, 139.82, 143.01, 144.42; EI-HRMS *m/z* [M^+] calcd for $C_{16}H_{32}N_3O$ 282.2545, found 282.2533.

[Pent-Hex-C4OH-tr][OTs] (e14): 83% yield, light yellow liquid; ^{1}H NMR (400 MHz, $CDCl_3$) δ 0.85-0.91 (m, 2 x CH_3 , 6H), 1.25-1.32 (m, 5 x CH_2, 10H), 1.66-1.73 (m, $CH{=}CCH_2CH_2$, 2H), 1.90-2.04 (m, 2 x NCH_2CH_2 + CH_2CH_2OH, 6H), 2.34 (s, *p*-CH_3, 3H), 2.91 (t, *J* = 6.0 Hz, $CH{=}CCH_2$, 2H), 3.70 (t, *J* = 6.0 Hz, CH_2CH_2OH, 2H), 4.39 (t, *J* = 8.0 Hz, $CNCH_2$, 2H), 4.61 (t, *J* = 8.0 Hz, $CHNCH_2$, 2H), 7.14 (d, *J* = 8.0 Hz, aryl *H*, 2H), 7.74 (d, *J* = 8.0 Hz, aryl *H*, 2H), 9.22 (s, $CH{=}CCH_2$, 1H); ^{13}C NMR (100 MHz, $CDCl_3$) δ 13.93, 14.05, 21.47, 22.10, 22.50, 23.19, 23.70, 26.07, 28.37, 28.89, 29.14, 31.14, 31.45, 51.09, 54.10, 61.29, 126.12, 128.83, 129.90, 139.79, 143.06, 144.44; EI-HRMS *m/z* [M^+] calcd for $C_{17}H_{34}N_3O$ 296.2702, found 296.2699.

[Pent-Oct-C4OH-tr][OTs] (e15): 88% yield, light yellow liquid; ^{1}H NMR (400 MHz, $CDCl_3$) δ 0.86-0.90 (m, 2 x CH_3 , 6H), 1.26-1.34 (m, 7 x CH_2, 10H), 1.69-1.75 (m, $CH{=}CCH_2CH_2$, 2H), 1.89-2.07 (m, 2 x NCH_2CH_2 + CH_2CH_2OH, 6H), 2.34 (s, *p*-CH_3, 3H), 2.92 (t, *J* = 8.0 Hz, $CH{=}CCH_2$, 2H), 3.72 (t, *J* = 6.0 Hz, CH_2CH_2OH, 2H), 4.39 (t, *J* = 8.0 Hz, $CNCH_2$, 2H), 4.62 (t, *J* = 8.0 Hz, $CHNCH_2$, 2H), 7.15 (d, *J* = 8.0 Hz, aryl H, 2H), 7.74 (d, *J* = 8.0 Hz, aryl H, 2H), 9.23 (s, $CH{=}CCH_2$, 1H); ^{13}C NMR (100 MHz, $CDCl_3$) δ 13.96, 14.24, 21.51, 22.12, 22.76, 23.24, 23.67, 26.45, 28.39, 28.95, 29.01,

29.13, 29.16, 31.33, 31.83, 51.10, 54.15, 61.42, 126.19, 128.93, 129.88, 140.19, 142.30, 144.39; EI-HRMS *m/z* [M^+] calcd for $C_{19}H_{38}N_3O$ 324.3015, found 324.3017.

[Hex-Et-C4OH-tr][OTs] (e16): 88% yield, light yellow liquid; ^{1}H NMR (400 MHz, $CDCl_3$) δ 0.84-0.88 (m, CH_3, 3H), 1.26-1.28 (m, 3 x CH_2, 6H), 1.58 (t, *J* = 8.0 Hz, CH_3, 3H), 1.66-1.73 (m, CH=CCH$_2$C*H*$_2$, 2H), 1.90-2.00 (m, NCH$_2$C*H*$_2$ + C*H*$_2$CH$_2$OH, 4H), 2.34 (s, *p*-CH_3, 3H), 2.92 (t, *J* = 8.0 Hz, CH=CC*H*$_2$, 2H), 3.70 (t, *J* = 6.0 Hz, CH$_2$C*H*$_2$OH, 2H), 4.47 (q, *J* = 8.0 Hz, CNC*H*$_2$, 4H), 4.55 (t, *J* = 8.0 Hz, CHNC*H*$_2$, 4H), 7.14 (d, *J* = 8.0 Hz, aryl H, 2H), 7.72 (d, *J* = 8.0 Hz, aryl H, 2H), 9.07 (s, C*H*=CCH$_2$, 1H); ^{13}C NMR (100 MHz, $CDCl_3$) δ 14.09, 14.12, 21.50, 22.52, 23.06, 23.60, 26.01, 29.35, 31.13, 31.26, 46.53, 54.10, 61.37, 126.16, 128.92, 129.64, 140.19, 142.32, 144.30; EI-HRMS *m/z* [M^+] calcd for $C_{14}H_{28}N_3O$ 254.2232, found 254.2222.

[Hex-Bu-C4OH-tr][OTs] (e17): 85% yield, light yellow liquid; ^{1}H NMR (400 MHz, $CDCl_3$) δ 0.86 (t, *J* = 8.0 Hz, CH_3, 3H), 0.98 (t, *J* = 8.0 Hz, CH_3, 3H), 1.25-1.31 (m, 3 x CH_2, 6H), 1.36-1.43 (m, CH_2, 2H), 1.68-1.75 (m, CH=CCH$_2$C*H*$_2$, 2H), 1.86-2.06 (m, 2 x NCH$_2$C*H*$_2$ + C*H*$_2$CH$_2$OH, 6H), 2.34 (s, *p*-C*H*$_3$, 3H), 2.92 (t, *J* = 6.0 Hz, CH=CC*H*$_2$, 2H), 3.72 (t, *J* = 6.0 Hz, CH$_2$C*H*$_2$OH, 2H), 4.40 (t, *J* = 8.0 Hz, CNC*H*$_2$, 2H), 4.61 (t, *J* = 8.0 Hz, CHNC*H*$_2$, 2H), 7.14 (d, *J* = 8.0 Hz, aryl *H*, 2H), 7.74 (d, *J* = 8.0 Hz, aryl *H*, 2H), 9.21 (s, C*H*=CCH$_2$, 1H); ^{13}C NMR (100 MHz, $CDCl_3$) δ 13.54, 14.08, 19.71, 21.50, 22.53, 23.20, 23.66, 26.00, 29.38, 30.86, 31.12, 31.34, 50.84, 54.13, 61.37,

126.18, 128.89, 129.84, 140.06, 142.50, 144.43; EI-HRMS *m/z* [M^+] calcd for $C_{16}H_{32}N_3O$ 282.2545, found 282.2532.

[Hex-Pent-C4OH-tr][OTs] (e18): 83% yield, light yellow liquid; ^{1}H NMR (400 MHz, $CDCl_3$) δ 0.86 (t, *J* = 6.0 Hz, CH_3, 3H), 0.92 (t, *J* = 8.0 Hz, CH_3, 3H), 1.25-1.39 (m, 5 x CH_2, 10H), 1.67-1.74 (m, $CH{=}CCH_2CH_2$, 2H), 1.90-2.06 (m, 2 x NCH_2CH_2 + CH_2CH_2OH, 6H), 2.34 (s, *p*-CH_3, 3H), 2.91 (t, *J* = 8.0 Hz, $CH{=}CCH_2$, 2H), 3.71 (t, *J* = 6.0 Hz, CH_2CH_2OH, 2H), 4.39 (t, *J* = 8.0 Hz, $CNCH_2$, 2H), 4.63 (t, *J* = 6.0 Hz, $CHNCH_2$, 2H), 7.15 (d, *J* = 8.0 Hz, aryl *H*, 2H), 7.75 (d, *J* = 8.0 Hz, aryl *H*, 2H), 9.24 (s, $CH{=}CCH_2$, 1H); ^{13}C NMR (100 MHz, $CDCl_3$) δ 13.89, 14.06, 21.45, 22.13, 22.51, 23.15, 23.68, 25.96, 28.43, 28.60, 29.37, 31.11, 31.44, 51.06, 54.06, 61.25, 126.09, 128.81, 129.77, 139.73, 143.12, 144.45; EI-HRMS *m/z* [M^+] calcd for $C_{17}H_{34}N_3O$ 296.2702, found 296.2700.

[Hex-Hex-C4OH-tr][OTs] (e19): 68% yield, light yellow liquid; ^{1}H NMR (400 MHz, $CDCl_3$) δ 0.84-0.91 (m, 2 x CH_3 , 6H), 1.25-1.32 (m, 6 x CH_2, 12H), 1.67-1.73 (m, $CH{=}CCH_2CH_2$, 2H), 1.91-2.03 (m, 2 x NCH_2CH_2 + CH_2CH_2OH, 6H), 2.34 (s, *p*-CH_3, 3H), 2.91 (t, *J* = 8.0 Hz, $CH{=}CCH_2$, 2H), 3.70 (t, *J* = 6.0 Hz, CH_2CH_2OH, 2H), 4.39 (t, *J* = 8.0 Hz, $CNCH_2$, 2H), 4.62 (t, *J* = 8.0 Hz, $CHNCH_2$, 2H), 7.14 (d, *J* = 8.0 Hz, aryl *H*, 2H), 7.74 (d, *J* = 8.0 Hz, aryl *H*, 2H), 9.24 (s, $CH{=}CCH_2$, 1H)); ^{13}C NMR (100 MHz, $CDCl_3$) δ 14.06, 14.07, 21.47, 22.51, 22.52, 23.19, 23.70, 25.99, 26.07, 28.90, 29.40,

31.13, 31.15, 31.45, 51.09, 54.11, 61.28, 126.12, 128.83, 129.88, 139.79, 143.07, 144.45; EI-HRMS *m/z* [M^+] calcd for $C_{18}H_{36}N_3O$ 310.2858, found 310.2858.

[Hex-Oct-C4OH-tr][OTs] (c20): 97% yield, light yellow liquid; ^{1}H NMR (400 MHz, $CDCl_3$) δ 0.85-0.90 (m, 2 x CH_3 , 6H), 1.28-1.32 (m, 8 x CH_2, 16H), 1.67-1.73 (m, CH=CCH_2CH_2, 2H), 1.93-2.06 (m, 2 x NCH_2CH_2 + CH_2OH, 6H), 2.34 (s, *p*-CH_3, 3H), 2.92 (t, *J* = 6.0 Hz, CH=CCH_2, 2H), 3.70 (t, *J* = 6.0 Hz, CH_2CH_2OH, 2H), 4.39 (t, *J* = 8.0 Hz, $CNCH_2$, 2H), 4.63 (t, *J* = 8.0 Hz, $CHNCH_2$, 2H), 7.14 (d, *J* = 8.0 Hz, aryl H, 2H), 7.75 (d, *J* = 8.0 Hz, aryl H, 2H), 9.28 (s, C*H*=CCH_2, 1H); ^{13}C NMR (100 MHz, $CDCl_3$) δ 14.08, 14.22, 21.48, 22.53, 22.75, 23.20, 23.68, 26.00, 26.42, 28.95, 29.00, 29.14, 29.41, 31.13, 31.43, 31.82, 51.08, 54.12, 61.29, 126.13, 128.83, 129.93, 139.80, 142.97, 144.41; EI-HRMS *m/z* [M^+] calcd for $C_{20}H_{40}N_3O$ 338.3171, found 338.3159.

[Oct-Et-C4OH-tr][OTs] (e21): 97% yield, light yellow liquid; ^{1}H NMR (400 MHz, $CDCl_3$) δ 0.87 (t, *J* = 6.0 Hz, CH_3, 3H), 1.23-1.28 (m, 5 x CH_2, 10H), 1.60 (t, *J* = 8.0 Hz, CH_3, 3H), 1.68-1.76 (m, CH=CCH_2CH_2, 2H), 1.92-2.03 (m, NCH_2CH_2 + CH_2CH_2OH, 2H), 2.34 (s, *p*-CH_3, 3H), 2.93 (t, *J* = 8.0 Hz, CH=CCH_2, 2H), 3.72 (t, *J* = 6.0 Hz, CH_2CH_2OH, 2H), 4.48 (q, *J* = 8.0 Hz, $CNCH_2$, 2H), 4.57 (t, *J* = 8.0 Hz, $CHNCH_2$, 2H), 7.14 (d, *J* = 8.0 Hz, aryl H, 2H), 7.72 (d, *J* = 8.0 Hz, aryl H, 2H), 9.08 (s, C*H*=CCH_2, 1H); ^{13}C NMR (100 MHz, $CDCl_3$) δ 14.08, 14.24, 21.50, 22.76, 23.04, 23.56, 26.34, 28.98, 29.16, 29.38, 31.16, 31.86, 46.52, 54.09, 61.44, 126.20, 128.96,

129.55, 140.40, 141.86, 144.28; EI-HRMS *m/z* [M^+] calcd for $C_{16}H_{32}N_3O$ 282.2545, found 282.2533.

[Oct-Bu-C4OH-tr][OTs] (e22): 78% yield, light yellow liquid; ^{1}H NMR (400 MHz, $CDCl_3$) δ 0.87 (t, *J* = 8.0 Hz, CH_3, 3H), 0.98 (t, *J* = 8.0 Hz, CH_3, 3H), 1.23-1.28 (m, 5 x CH_2, 10H), 1.36-1.43 (m, CH_2, 2H), 1.67-1.74 (m, $CH{=}CCH_2C\mathit{H}_2$, 2H), 1.88-2.06 (m, 2 x $NCH_2C\mathit{H}_2$ + $C\mathit{H}_2CH_2OH$, 6H), 2.34 (s, *p*-CH_3, 3H), 2.92 (t, *J* = 8.0 Hz, $CH{=}CC\mathit{H}_2$, 2H), 3.71 (t, *J* = 6.0 Hz, $CH_2C\mathit{H}_2OH$, 2H), 4.40 (t, *J* = 8.0 Hz, $CNC\mathit{H}_2$, 2H), 4.62 (t, *J* = 6.0 Hz, $CHNC\mathit{H}_2$, 2H), 7.14 (d, *J* = 8.0 Hz, aryl H, 2H), 7.74 (d, *J* = 8.0 Hz, aryl H, 2H), 9.23 (s, $C\mathit{H}{=}CCH_2$, 1H); ^{13}C NMR (100 MHz, $CDCl_3$) δ 13.54, 14.25, 19.72, 21.50, 22.77, 23.20, 23.68, 26.34, 28.98, 29.19, 29.45, 30.87, 31.37, 31.86, 50.83, 54.13, 61.35, 126.17, 128.88, 129.86, 139.99, 142.62, 144.44; EI-HRMS *m/z* [M^+] calcd for $C_{18}H_{36}N_3O$ 310.2858, found 310.2846.

[Oct-Pent-C4OH-tr][OTs] (e23): 98% yield, light yellow liquid; ^{1}H NMR (400 MHz, $CDCl_3$) δ 0.85-0.94 (m, 2 x CH_3, 6H), 1.23-1.38 (m, 7 x CH_2, 16H), 1.68-1.74 (m, $CH{=}CCH_2C\mathit{H}_2$, 2H), 1.90-2.06 (m, 2 x $NCH_2C\mathit{H}_2$ + $C\mathit{H}_2CH_2OH$, 6H), 2.34 (s, *p*-CH_3, 3H), 2.92 (t, *J* = 8.0 Hz, $CH{=}CC\mathit{H}_2$, 2H), 3.71 (t, *J* = 6.0 Hz, $CH_2C\mathit{H}_2OH$, 2H), 4.39 (t, *J* = 8.0 Hz, $CNC\mathit{H}_2$, 2H), 4.62 (t, *J* = 8.0 Hz, $CHNC\mathit{H}_2$, 2H), 7.14 (d, *J* = 8.0 Hz, aryl H, 2H), 7.74 (d, *J* = 8.0 Hz, aryl H, 2H), 9.25 (s, $C\mathit{H}{=}CCH_2$, 1H); ^{13}C NMR (100 MHz, $CDCl_3$) δ 13.91, 14.24, 21.48, 22.15, 22.76, 23.19, 23.66, 26.33, 28.46, 28.61, 28.98,

29.18, 29.43, 31.36, 31.86, 51.05, 54.10, 61.34, 126.15, 128.87, 129.82, 139.96, 142.69, 144.42; EI-HRMS *m/z* [M^+] calcd for $C_{18}H_{36}N_3O$ 324.3015, found 324.3005.

[Oct-Hex-C4OH-tr][OTs] (e24): 98% yield, light yellow liquid; ^{1}H NMR (400 MHz, $CDCl_3$) δ 0.85-0.91 (m, 2 x CH_3 , 6H), 1.23-1.32 (m, 8 x CH_2, 16H), 1.67-1.75 (m, $CH{=}CCH_2CH_2$, 2H), 1.92-2.07 (m, 2 x NCH_2CH_2 + CH_2CH_2OH, 6H), 2.34 (s, *p*-CH_3, 3H), 2.92 (t, *J* = 8.0 Hz, $CH{=}CCH_2$, 2H), 3.72 (t, *J* = 6.0 Hz, CH_2CH_2OH, 2H), 4.39 (t, *J* = 8.0 Hz, $CNCH_2$, 2H), 4.62 (t, *J* = 8.0 Hz, $CHNCH_2$, 2H), 7.14 (d, *J* = 8.0 Hz, aryl H, 2H), 7.74 (d, *J* = 8.0 Hz, aryl H, 2H), 9.24 (s, $CH{=}CCH_2$, 1H); ^{13}C NMR (100 MHz, $CDCl_3$) δ 14.05, 14.23, 21.48, 22.50, 22.76, 23.17, 23.63, 26.07, 26.32, 28.85, 28.98, 29.18, 29.41, 31.14, 31.33, 31.86, 51.07, 54.08, 61.34, 126.14, 128.87, 129.73, 139.99, 142.58, 144.40; EI-HRMS *m/z* [M^+] calcd for $C_{20}H_{40}N_3O$ 338.3171, found 338.3163.

[Oct-Oct-C4OH-tr][OTs] (e25): 94% yield, light yellow liquid; ^{1}H NMR (400 MHz, $CDCl_3$) δ 0.85-0.90 (m, 2 x CH_3 , 6H), 1.23-1.33 (m, 10 x CH_2, 20H), 1.67-1.73 (m, $CH{=}CCH_2CH_2$, 2H), 1.93-2.06 (m, 2 x NCH_2CH_2 + CH_2CH_2OH, 6H), 2.34 (s, *p*-CH_3, 3H), 2.91 (t, *J* = 8.0 Hz, $CH{=}CCH_2$, 2H), 3.72 (m, CH_2CH_2OH, 2H), 4.39 (t, *J* = 8.0 Hz, $CNCH_2$, 2H), 4.62 (t, *J* = 8.0 Hz, $CHNCH_2$, 2H), 7.14 (d, *J* = 8.0 Hz, aryl H, 2H), 7.74 (d, *J* = 8.0 Hz, aryl H, 2H), 9.25 (s, $CH{=}CCH_2$, 1H); ^{13}C NMR (100 MHz, $CDCl_3$) δ 14.23, 14.24, 21.47, 22.74, 22.75, 23.18, 23.64, 26.33, 26.41, 26.48, 28.91, 29.00, 29.01, 29.14, 29.18, 29.42, 31.82, 31.86, 51.06, 54.08, 61.38, 126.14, 128.86, 129.78,

139.91, 142.72, 144.40; EI-HRMS *m/z* [M^+] calcd for $C_{22}H_{44}N_3O$ 366.3484, found 366.3473.

Combinatorial Synthesis of a Library of 26 [R2-R3-C4OH-tr][OTMBS] Ionic Liquids

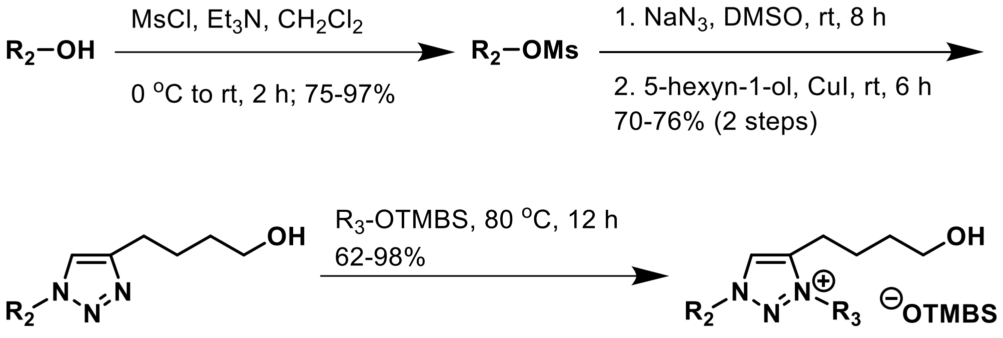

[R2-R3-C4OH-tr][OTMBS]
f1-f26

Generalized Procedure for the Synthesis of [R_2-R_3-C4OH-tr][OTMBS] Ionic Liquids

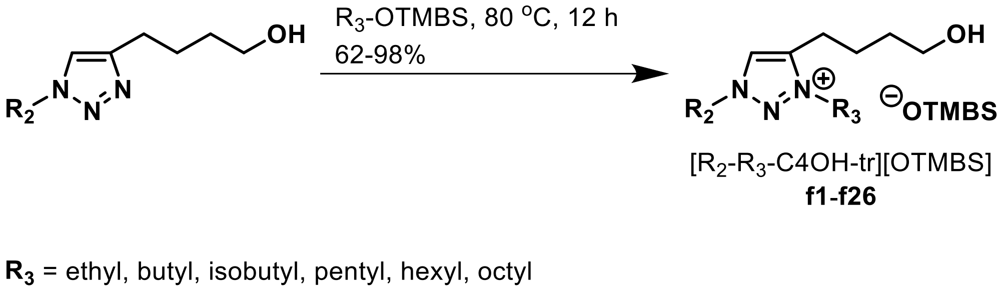

To a round-bottomed flask containing R_2-C4OH-tr (0.5 mmol) was added alkyl 2,4,6-trimethylbenzenesulfonate (R_3-OTMBS, 0.7 mmol, 1.3 equiv). The mixture was heated at 80 ºC for 12 h. The progress of the ionic liquid forming reaction could be readily monitored by TLC. After reaction was completed, the reaction mixture was washed with ether (3 mL, 3 times) and then with *n*-hexane (3 mL, 3 times) to finally afford the desired [R_2-R_3-C4OH-tr][OTMBS] as ionic liquids (**f1**-**f26**).

[Et-Et-C4OH-tr][OTMBS] (f1): 94% yield, light yellow liquid; ^{1}H NMR (400 MHz, $CDCl_3$) δ 1.59-1.73 (m, 2 x CH_3 + CH=CCH_2CH_2, 8H), 1.99-2.06 (m, CH_2CH_2OH, 2H), 2.22 (s, *p*-CH_3, 3H), 2.60 (s, 2 x *o*-CH_3, 6H), 2.94 (t, J = 6.0 Hz, CH=CCH_2, 2H), 3.70 (t, J = 6.0 Hz, CH_2CH_2OH, 2H), 4.47 (q, J = 8.0 Hz, $CNCH_2CH_3$, 2H), 4.71 (q, J = 8.0 Hz, $CHNCH_2CH_3$, 2H), 6.80 (s, aryl H, 2H), 9.31 (s, CH=CCH_2, 1H); ^{13}C NMR (100

MHz, $CDCl_3$) δ 14.10, 14.54, 20.95, 23.05, 23.09, 23.67, 31.36, 46.51, 49.53, 61.27, 129.63, 130.82, 137.29, 138.83, 139.53, 144.32; EI-HRMS *m/z* [M^+] calcd for $C_{10}H_{20}N_3O$ 198.1606, found 198.1596.

[Et-Bu-C4OH-tr][OTMBS] (f2): 86% yield, light yellow solid; mp 66-67 ºC; ^{1}H NMR (400 MHz, $CDCl_3$) δ 0.98 (t, *J* = 8.0 Hz, $CH_2CH_2CH_3$, 3H), 1.34-1.43 (m, $CH_2CH_2CH_3$, 2H), 1.63 (t, *J* = 6.0 Hz, NCH_2CH_3 , 3H), 1.68-1.72 (m, $CH{=}CCH_2CH_2$, 2H), 1.87-1.95 (m, $CH_2CH_2CH_3$, 2H), 1.98-2.05 (m, CH_2CH_2OH, 2H), 2.22 (s, *p*-CH_3, 3H), 2.63 (s, 2 x *o*-CH_3, 6H), 2.90 (t, *J* = 8.0 Hz, $CH{=}CCH_2$, 2H), 3.68 (t, *J* = 6.0 Hz, CH_2CH_2OH, 2H), 4.38 (t, *J* = 8.0 Hz, NCH_2CH_2, 2H), 4.71 (q, *J* = 8.0 Hz, NCH_2CH_3, 2H), 6.81 (s, aryl *H*, 2H), 9.37 (s, $CH{=}CCH_2$, 1H); ^{13}C NMR (100 MHz, $CDCl_3$) δ 13.47, 14.49, 19.63, 20.85, 23.06, 23.07, 23.64, 30.78, 31.40, 49.40, 50.78, 61.11, 129.44, 130.70, 137.04, 138.41, 140.25, 144.39; EI-HRMS *m/z* [M^+] calcd for $C_{12}H_{24}N_3O$ 226.1919, found 226.1909.

[Et-Pent-C4OH-tr][OTMBS] (f3): 98% yield, light yellow liquid; ^{1}H NMR (400 MHz, $CDCl_3$) δ 0.93 (t, *J* = 6.0 Hz, CH_3 , 3H), 1.34-1.39 (m, 2 x CH_2 , 4H), 1.66 (t, *J* = 8.0 Hz, NCH_2CH_3 , 3H), 1.69-1.74 (m, $CH{=}CCH_2CH_2$, 2H), 1.91-1.99 (m, NCH_2CH_2 , 2H), 2.01-2.08 (m, CH_2CH_2OH, 2H), 2.22 (s, *p*-CH_3, 3H), 2.62 (s, 2 x *o*-CH_3, 6H), 2.92 (t, *J* = 6.0 Hz, $CH{=}CCH_2$, 2H), 3.71 (t, *J* = 6.0 Hz, CH_2CH_2OH, 2H), 4.38 (t, *J* = 8.0 Hz, NCH_2CH_2, 2H), 4.74 (q, *J* = 8.0 Hz, NCH_2CH_3, 2H), 6.80 (s, aryl H, 2H), 9.44 (s,

C*H*=CCH_2, 1H); ^{13}C NMR (100 MHz, $CDCl_3$) δ 13.90, 14.55, 20.93, 22.14, 23.10, 23.22, 23.70, 28.48, 28.64, 31.44, 49.53, 51.03, 61.26, 129.72, 130.79, 137.20, 138.60, 139.93, 144.41; EI-HRMS *m/z* [M^+] calcd for $C_{13}H_{26}N_3O$ 240.2076, found 240.2067.

[Et-Hex-C4OH-tr][OTMBS] (f4): 94% yield, light yellow liquid; ^1H NMR (400 MHz, $CDCl_3$) δ 0.90 (t, *J* = 8.0 Hz, $CH_2CH_2CH_3$, 3H), 1.29-1.38 (m, 3 x CH_2, 6H), 1.66 (t, *J* = 8.0 Hz, NCH_2CH_3, 3H), 1.69-1.74 (m, $CH{=}CCH_2CH_2$, 2H), 1.90-1.98 (m, NCH_2CH_2, 2H), 2.01-2.08 (m, CH_2CH_2OH, 2H), 2.22 (s, *p*-CH_3, 3H), 2.63 (s, 2 x *o*-CH_3, 6H), 2.91 (t, *J* = 8.0 Hz, $CH{=}CCH_2$, 2H), 3.70 (t, *J* = 6.0 Hz, CH_2CH_2OH, 2H), 4.38 (t, *J* = 6.0 Hz, NCH_2CH_2, 2H), 4.74 (q, *J* = 8.0 Hz, NCH_2CH_3, 2H), 6.81 (s, aryl H, 2H), 9.46 (s, C*H*=CCH_2, 1H); ^{13}C NMR (100 MHz, $CDCl_3$) δ 14.05, 14.57, 20.93, 22.50, 23.11, 23.22, 23.72, 26.10, 28.91, 31.14, 31.47, 49.53, 51.05, 61.24, 129.78, 130.77, 137.17, 138.51, 140.10, 144.40; EI-HRMS *m/z* [M^+] calcd for $C_{14}H_{28}N_3O$ 254.2232, found 254.2222.

[Et-Oct-C4OH-tr][OTMBS] (f5): 92% yield, light yellow liquid; ^1H NMR (400 MHz, $CDCl_3$) δ 0.89 (t, *J* = 6.0 Hz, $CH_2CH_2CH_3$, 3H), 1.25-1.34 (m, 5 x CH_2, 10H), 1.63-1.72 (m, NCH_2CH_3 + $CH{=}CCH_2CH_2$, 5H), 1.89-1.95 (m, NCH_2CH_2, 2H), 1.98-2.06 (m, CH_2CH_2OH, 2H), 2.22 (s, *p*-CH_3, 3H), 2.64 (s, 2 x *o*-CH_3, 6H), 2.90 (t, *J* = 8.0 Hz, $CH{=}CCH_2$, 2H), 3.69 (t, *J* = 6.0 Hz, CH_2CH_2OH, 2H), 4.37 (t, *J* = 8.0 Hz, NCH_2CH_2, 2H), 4.73 (q, *J* = 8.0 Hz, NCH_2CH_3, 2H), 6.81 (s, aryl H, 2H), 9.44 (s, C*H*=CCH_2, 1H);

^{13}C NMR (100 MHz, $CDCl_3$) δ 14.22, 14.61, 20.92, 22.73, 23.13, 23.19, 23.71, 26.42, 28.98, 29.98, 29.11, 31.50, 31.80, 49.51, 51.07, 61.19, 129.79, 130.74, 137.14, 138.40, 140.29, 144.41; EI-HRMS *m/z* [M^+] calcd for $C_{16}H_{32}N_3O$ 282.2545, found 282.2532.

[Bu-Et-C4OH-tr][OTMBS] (f6): 91% yield, light yellow liquid; ^1H NMR (400 MHz, $CDCl_3$) δ 0.94 (t, *J* = 6.0 Hz, $CH_2CH_2CH_3$, 3H), 1.31-1.40 (m, $CH_2CH_2CH_3$, 2H), 1.61 (t, *J* = 6.0 Hz, NCH_2CH_3 , 3H), 1.66-1.73 (m, $CH{=}CCH_2CH_2$, 2H), 1.94-2.05 (m, NCH_2CH_2 + CH_2CH_2OH, 4H), 2.22 (s, *p*-CH_3, 3H), 2.63 (s, 2 x *o*-CH_3, 6H), 2.94 (t, *J* = 8.0 Hz, $CH{=}CCH_2$, 2H), 3.69 (t, *J* = 6.0 Hz, CH_2CH_2OH, 2H), 4.48 (q, *J* = 8.0 Hz, NCH_2CH_3, 2H), 4.64 (t, *J* = 8.0 Hz, NCH_2CH_2, 2H), 6.80 (s, aryl H, 2H), 9.36 (s, $CH{=}CCH_2$, 1H); ^{13}C NMR (100 MHz, $CDCl_3$) δ 13.43, 14.06, 19.55, 20.89, 23.02, 23.10, 23.68, 31.28, 31.42, 46.46, 53.75, 61.16, 129.64, 130.75, 137.07, 138.46, 140.25, 144.32; EI-HRMS *m/z* [M^+] calcd for $C_{12}H_{24}N_3O$ 226.1919, found 226.1909.

[Bu-Bu-C4OH-tr][OTMBS] (f7): 62% yield, light yellow liquid; ^1H NMR (400 MHz, $CDCl_3$) δ 0.92-1.01 (m, 2 x CH_3 , 6H), 1.32-1.42 (m, 2 x $CH_2CH_2CH_3$, 4H), 1.68-1.74 (m, $CH{=}CCH_2CH_2$, 2H), 1.91-2.06 (m, 2 x NCH_2CH_2 + CH_2CH_2OH, 6H), 2.22 (s, *p*-CH_3, 3H), 2.63 (s, 2 x *o*-CH_3, 6H), 2.92 (t, *J* = 8.0 Hz, $CH{=}CCH_2$, 2H), 3.71 (t, *J* = 6.0 Hz, CH_2CH_2OH, 2H), 4.40 (t, *J* = 6.0 Hz, $CNCH_2$, 2H), 4.65 (t, *J* = 6.0 Hz, $CHNCH_2$, 2H), 6.81 (s, aryl *H*, 2H), 9.41 (s, $CH{=}CCH_2$, 1H); ^{13}C NMR (100 MHz, $CDCl_3$) δ 13.48, 13.53, 19.64, 19.76, 20.95, 23.14, 23.35, 23.88, 30.94, 31.39, 31.52, 50.80, 53.99, 61.34,

130.26, 130.81, 137.22, 138.59, 140.08, 144.45; EI-HRMS *m/z* [M^+] calcd for $C_{14}H_{28}N_3O$ 254.2232, found 254.2222.

[Bu-Pent-C4OH-tr][OTMBS] (f8): 97% yield, light yellow liquid; ^{1}H NMR (400 MHz, $CDCl_3$) δ 0.89-0.96 (m, 2 x CH_3, 6H), 1.30-1.39 (m, 3 x CH_2, 6H), 1.67-1.74 (m, $CH{=}CCH_2CH_2$, 2H), 1.92-2.08 (m, 2 x NCH_2CH_2 + CH_2CH_2OH, 6H), 2.22 (s, *p*-CH_3, 3H), 2.63 (s, 2 x *o*-CH_3, 6H), 2.92 (t, *J* = 8.0 Hz, $CH{=}CCH_2$, 2H), 3.71 (t, *J* = 6.0 Hz, CH_2CH_2OH, 2H), 4.39 (t, *J* = 8.0 Hz, $CNCH_2$, 2H), 4.66 (t, *J* = 8.0 Hz, $CHNCH_2$, 2H), 6.80 (s, aryl *H*, 2H), 9.43 (s, $CH{=}CCH_2$, 1H); ^{13}C NMR (100 MHz, $CDCl_3$) δ 13.42, 13.90, 19.58, 20.92, 22.13, 23.11, 23.23, 23.76, 28.45, 28.63, 31.35, 31.47, 51.02, 53.84, 61.25, 130.06, 130.76, 137.20, 138.52, 140.05, 144.45; EI-HRMS *m/z* [M^+] calcd for $C_{15}H_{30}N_3O$ 268.2389, found 268.2382.

[Bu-Hex-C4OH-tr][OTMBS] (f9): 98% yield, light yellow liquid; ^{1}H NMR (400 MHz, $CDCl_3$) δ 0.88-0.95 (m, 2 x CH_3 , 6H), 1.29-1.38 (m, 4 x CH_2, 8H), 1.68-1.74 (m, $CH{=}CCH_2CH_2$, 2H), 1.91-2.09 (m, 2 x NCH_2CH_2 + CH_2CH_2OH, 6H), 2.22 (s, *p*-CH_3, 3H), 2.63 (s, 2 x *o*-CH_3, 6H), 2.92 (t, *J* = 8.0 Hz, $CH{=}CCH_2$, 2H), 3.71 (t, *J* = 6.0 Hz, CH_2CH_2OH, 2H), 4.39 (t, *J* = 8.0 Hz, $CNCH_2$, 2H), 4.67 (t, *J* = 6.0 Hz, $CHNCH_2$, 2H), 6.81 (s, aryl *H*, 2H), 9.44 (s, $CH{=}CCH_2$, 1H); ^{13}C NMR (100 MHz, $CDCl_3$) δ 13.43, 14.04, 19.56, 20.92, 22.51, 23.12, 23.23, 23.74, 26.06, 28.85, 31.13, 31.30, 31.50, 51.01,

53.82, 61.24, 129.81, 130.80, 137.14, 138.54, 140.11, 144.44; EI-HRMS *m/z* [M^+] calcd for $C_{16}H_{32}N_3O$ 282.2545, found 282.2541.

[Bu-Oct-C4OH-tr][OTMBS] (f10): 91% yield, light yellow liquid; ^{1}H NMR (400 MHz, $CDCl_3$) δ 0.89 (t, *J* = 6.0 Hz, CH_3, 3H), 0.94 (t, *J* = 8.0 Hz, CH_3, 3H), 1.25-1.38 (m, 6 x CH_2, 12H), 1.66-1.73 (m, CH=CCH_2CH_2, 2H), 1.88-2.07 (m, 2 x NCH_2CH_2 + CH_2CH_2OH, 6H), 2.22 (s, *p*-CH_3, 3H), 2.64 (s, 2 x *o*-CH_3, 6H), 2.91 (t, *J* = 8.0 Hz, CH=CCH_2, 2H), 3.70 (t, *J* = 6.0 Hz, CH_2CH_2OH, 2H), 4.38 (t, *J* = 8.0 Hz, $CNCH_2$, 2H), 4.66 (t, *J* = 8.0 Hz, $CHNCH_2$, 2H), 6.80 (s, aryl H, 2H), 9.43 (s, CH=CCH_2, 1H); ^{13}C NMR (100 MHz, $CDCl_3$) δ 13.47, 14.22, 19.59, 20.93, 22.74, 23.14, 23.22, 23.77, 26.40, 28.97, 28.97, 29.12, 31.39, 31.51, 31.80, 51.06, 53.85, 61.22, 130.13, 130.74, 137.19, 138.42, 140.20, 144.44; EI-HRMS *m/z* [M^+] calcd for $C_{18}H_{36}N_3O$ 310.2858, found 310.2850.

[Pent-Et-C4OH-tr][OTMBS] (f11): 85% yield, light yellow liquid; ^{1}H NMR (400 MHz, $CDCl_3$) δ 0.88 (t, *J* = 8.0 Hz, CH_3, 3H), 1.29-1.33 (m, 2 x CH_2, 4H), 1.63 (t, *J* = 6.0 Hz, CH_3, 3H), 1.69-1.75 (m, CH=CCH_2CH_2, 2H), 1.97-2.09 (m, NCH_2CH_2 + CH_2CH_2OH, 4H), 2.22 (s, *p*-CH_3, 3H), 2.61 (s, 2 x *o*-CH_3, 6H), 2.95 (t, *J* = 8.0 Hz, CH=CCH_2, 2H), 3.72 (t, *J* = 6.0 Hz, CH_2CH_2OH, 2H), 4.48 (q, *J* = 8.0 Hz, NCH_2CH_3, 2H), 4.64 (t, *J* = 8.0 Hz, NCH_2CH_2, 2H), 6.80 (s, aryl H, 2H), 9.36 (s, CH=CCH_2, 1H); ^{13}C NMR (100 MHz, $CDCl_3$) δ 13.93, 14.14, 20.93, 22.10, 23.09, 23.10, 23.71, 28.38,

29.16, 31.40, 46.49, 54.08, 61.24, 129.93, 130.79, 137.22, 138.64, 139.84, 144.32; EI-HRMS *m/z* [M^+] calcd for $C_{13}H_{26}N_3O$ 240.2076, found 240.2065.

[Pent-Bu-C4OH-tr][OTMBS] (f12): 74% yield, light yellow liquid; ^{1}H NMR (400 MHz, $CDCl_3$) δ 0.88 (t, *J* = 8.0 Hz, CH_3, 3H), 1.00 (t, *J* = 8.0 Hz, CH_3, 3H), 1.28-1.43 (m, 3 x CH_2, 6H), 1.67-1.74 (m, CH=CCH_2CH_2, 2H), 1.92-2.08 (m, 2 x NCH_2CH_2 + CH_2CH_2OH, 6H), 2.22 (s, *p*-CH_3, 3H), 2.66 (s, 2 x *o*-CH_3, 6H), 2.93 (t, *J* = 8.0 Hz, CH=CCH_2, 2H), 3.71 (t, *J* = 6.0 Hz, CH_2CH_2OH, 2H), 4.40 (t, *J* = 8.0 Hz, $CNCH_2$, 2H), 4.67 (t, *J* = 8.0 Hz, $CHNCH_2$, 2H), 6.81 (s, aryl *H*, 2H), 9.52 (s, C*H*=CCH_2, 1H); ^{13}C NMR (100 MHz, $CDCl_3$) δ 13.53, 13.95, 19.72, 20.94, 22.11, 23.18, 23.28, 23.82, 28.39, 29.20, 30.91, 31.55, 50.79, 54.13, 61.23, 130.18, 130.77, 137.13, 138.38, 140.42, 144.44; EI-HRMS *m/z* [M^+] calcd for $C_{15}H_{30}N_3O$ 268.2389, found 268.2386.

[Pent-Pent-C4OH-tr][OTMBS] (f13): 67% yield, light yellow liquid; ^{1}H NMR (400 MHz, $CDCl_3$) δ 0.86-0.94 (m, 2 x CH_3, 6H), 1.28-1.39 (m, 4 x CH_2, 8H), 1.66-1.73 (m, CH=CCH_2CH_2, 2H), 1.90-2.05 (m, 2 x NCH_2CH_2 + CH_2CH_2OH, 6H), 2.22 (s, *p*-CH_3, 3H), 2.65 (s, 2 x *o*-CH_3, 6H), 2.92 (t, *J* = 8.0 Hz, CH=CCH_2, 2H), 3.69 (t, *J* = 6.0 Hz, CH_2CH_2OH, 2H), 4.39 (t, *J* = 6.0 Hz, $CNCH_2$, 2H), 4.65 (t, *J* = 8.0 Hz, $CHNCH_2$, 2H), 6.81 (s, aryl *H*, 2H), 9.47 (s, C*H*=CCH_2, 1H); ^{13}C NMR (100 MHz, $CDCl_3$) δ 13.92, 13.94, 20.92, 22.10, 22.14, 23.17, 23.26, 23.79, 28.37, 28.46, 28.64, 29.15, 31.57, 50.99,

54.09, 61.21, 130.03, 130.77, 137.11, 138.37, 140.41, 144.43; EI-HRMS *m/z* [M^+] calcd for $C_{16}H_{32}N_3O$ 282.2545, found 282.2545.

[Pent-Hex-C4OH-tr][OTMBS] (f14): 63% yield, light yellow liquid; ^{1}H NMR (400 MHz, $CDCl_3$) δ 0.86-0.91 (m, 2 x CH_3 , 6H), 1.28-1.33 (m, 5 x CH_2, 10H), 1.67-1.73 (m, $CH{=}CCH_2CH_2$, 2H), 1.91-2.06 (m, 2 x NCH_2CH_2 + CH_2CH_2OH, 6H), 2.22 (s, *p*-CH_3, 3H), 2.65 (s, 2 x *o*-CH_3, 6H), 2.92 (t, *J* = 8.0 Hz, $CH{=}CCH_2$, 2H), 3.70 (t, *J* = 6.0 Hz, CH_2CH_2OH, 2H), 4.38 (t, *J* = 6.0 Hz, $CNCH_2$, 2H), 4.66 (t, *J* = 8.0 Hz, $CHNCH_2$, 2H), 6.81 (s, aryl *H*, 2H), 9.49 (s, $CH{=}CCH_2$, 1H); ^{13}C NMR (100 MHz, $CDCl_3$) δ 13.76, 13.88, 20.75, 21.93, 22.33, 23.00, 23.10, 23.64, 25.91, 28.20, 28.76, 29.01, 30.96, 31.37, 50.86, 53.95, 61.05, 129.99, 130.60, 136.95, 138.23, 140.18, 144.23; EI-HRMS *m/z* [M^+] calcd for $C_{17}H_{34}N_3O$ 296.2702, found 296.2695.

[Pent-Oct-C4OH-tr][OTMBS] (f15): 90% yield, light yellow liquid; ^{1}H NMR (400 MHz, $CDCl_3$) δ 0.86-0.90 (m, 2 x CH_3 , 6H), 1.27-1.34 (m, 7 x CH_2, 8H), 1.67-1.73 (m, $CH{=}CCH_2CH_2$, 2H), 1.90-2.07 (m, 2 x NCH_2CH_2 + CH_2CH_2OH, 6H), 2.22 (s, *p*-CH_3, 3H), 2.64 (s, 2 x *o*-CH_3, 6H), 2.91 (t, *J* = 8.0 Hz, $CH{=}CCH_2$, 2H), 3.71 (t, *J* = 6.0 Hz, CH_2CH_2OH, 2H), 4.39 (t, *J* = 6.0 Hz, $CNCH_2$, 2H), 4.65 (t, *J* = 8.0 Hz, $CHNCH_2$, 2H), 6.81 (s, aryl H, 2H), 9.43 (s, $CH{=}CCH_2$, 1H); ^{13}C NMR (100 MHz, $CDCl_3$) δ 13.94, 14.22, 20.94, 22.11, 22.74, 23.15, 23.25, 23.79, 26.42, 28.39, 28.99, 28.99, 29.13, 29.21,

31.51, 31.81, 51.06, 54.11, 61.25, 130.19, 130.76, 137.21, 138.45, 140.17, 144.42; EI-HRMS *m/z* [M^+] calcd for $C_{19}H_{38}N_3O$ 324.3015, found 324.3009.

[Hex-Et-C4OH-tr][OTMBS] (f16): 83% yield, light yellow liquid; ^{1}H NMR (400 MHz, $CDCl_3$) δ 0.87 (t, *J* = 6.0 Hz, CH_3, 3H), 1.26-1.34 (m, 3 x CH_2, 6H), 1.62 (t, *J* = 8.0 Hz, CH_3, 3H), 1.68-1.75 (m, $CH{=}CCH_2CH_2$, 2H), 1.96-2.07 (m, NCH_2CH_2 + CH_2CH_2OH, 4H), 2.22 (s, *p*-CH_3, 3H), 2.63 (s, 2 x *o*-CH_3, 6H), 2.95 (t, *J* = 8.0 Hz, $CH{=}CCH_2$, 2H), 3.71 (t, *J* = 6.0 Hz, CH_2CH_2OH, 2H), 4.48 (q, *J* = 8.0 Hz, NCH_2CH_3, 2H), 4.64 (t, *J* = 8.0 Hz, NCH_2CH_2, 2H), 6.80 (s, aryl H, 2H), 9.38 (s, $CH{=}CCH_2$, 1H); ^{13}C NMR (100 MHz, $CDCl_3$) δ 14.07, 14.17, 20.93, 22.52, 23.13, 23.14, 23.75, 26.01, 29.44, 31.13, 31.45, 46.48, 54.10, 60.20, 130.01, 130.75, 137.16, 138.44, 140.21, 144.32; EI-HRMS *m/z* [M^+] calcd for $C_{14}H_{28}N_3O$ 254.2232, found 254.2225.

[Hex-Bu-C4OH-tr][OTMBS] (f17): 70% yield, light yellow liquid; ^{1}H NMR (400 MHz, $CDCl_3$) δ 0.86 (t, *J* = 6.0 Hz, CH_3, 3H), 1.00 (t, *J* = 8.0 Hz, CH_3, 3H), 1.27-1.33 (m, 3 x CH_2, 6H), 1.68-1.43 (m, CH_2, 2H), 1.69-1.75 (m, $CH{=}CCH_2CH_2$, 2H), 1.90-2.01 (m, 2 x NCH_2CH_2 + CH_2CH_2OH, 6H), 2.22 (s, *p*-CH_3, 3H), 2.64 (s, 2 x *o*-CH_3, 6H), 2.93 (t, *J* = 8.0 Hz, $CH{=}CCH_2$, 2H), 3.72 (t, *J* = 6.0 Hz, CH_2CH_2OH, 2H), 4.40 (t, *J* = 8.0 Hz, $CNCH_2$, 2H), 4.66 (t, *J* = 8.0 Hz, $CHNCH_2$, 2H), 6.81 (s, aryl *H*, 2H), 9.47 (s, $CH{=}CCH_2$, 1H); ^{13}C NMR (100 MHz, $CDCl_3$) δ 13.52, 14.07, 19.71, 20.93, 22.53, 23.15, 23.26, 23.80, 26.00, 29.45, 30.90, 31.12, 31.50, 50.80, 54.13, 61.24, 130.15,

130.77, 137.17, 138.46, 140.20, 144.45; EI-HRMS *m/z* [M^+] calcd for $C_{16}H_{32}N_3O$ 282.2545, found 282.2534.

[Hex-Pent-C4OH-tr][OTMBS] (f18): 64% yield, light yellow liquid; ^{1}H NMR (400 MHz, $CDCl_3$) δ 0.86 (t, *J* = 6.0 Hz, CH_3, 3H), 0.92 (t, *J* = 8.0 Hz, CH_3, 3H), 1.28-1.40 (m, 5 x CH_2, 10H), 1.67-1.73 (m, CH=CCH_2CH_2, 2H), 1.90-2.08 (m, 2 x NCH_2CH_2 + CH_2CH_2OH, 6H), 2.22 (s, *p*-CH_3, 3H), 2.65 (s, 2 x *o*-CH_3, 6H), 2.92 (t, *J* = 8.0 Hz, CH=CCH_2, 2H), 3.70 (t, *J* = 6.0 Hz, CH_2CH_2OH, 2H), 4.39 (t, *J* = 8.0 Hz, CNCH_2, 2H), 4.65 (t, *J* = 8.0 Hz, CHNCH_2, 2H), 6.81 (s, aryl H, 2H), 9.47 (s, C*H*=CCH_2, 1H); ^{13}C NMR (100 MHz, $CDCl_3$) δ 13.91, 14.07, 20.93, 22.15, 22.53, 23.18, 23.24, 23.81, 26.00, 28.46, 28.68, 29.47, 31.13, 31.53, 51.03, 54.12, 61.21, 130.22, 130.73, 137.14, 138.30, 140.44, 144.44; EI-HRMS *m/z* [M^+] calcd for $C_{17}H_{34}N_3O$ 296.2702, found 296.2688.

[Hex-Hex-C4OH-tr][OTMBS] (f19): 97% yield, light yellow liquid; ^{1}H NMR (400 MHz, $CDCl_3$) δ 0.85-0.92 (m, 2 x CH_3 , 6H), 1.29-1.34 (m, 6 x CH_2, 12H), 1.67-1.74 (m, CH=CCH_2CH_2, 2H), 1.91-2.09 (m, 2 x NCH_2CH_2 + CH_2CH_2OH, 6H), 2.22 (s, *p*-CH_3, 3H), 2.67 (s, 2 x *o*-CH_3, 6H), 2.92 (t, *J* = 8.0 Hz, CH=CCH_2, 2H), 3.71 (t, *J* = 6.0 Hz, CH_2CH_2OH, 2H), 4.39 (t, *J* = 6.0 Hz, CNCH_2, 2H), 4.67 (t, *J* = 8.0 Hz, CHNCH_2, 2H), 6.81 (s, aryl H, 2H), 9.53 (s, C*H*=CCH_2, 1H); ^{13}C NMR (100 MHz, $CDCl_3$) δ 14.07, 14.07, 20.92, 22.50, 22.53, 23.20, 23.24, 23.82, 26.00, 26.07, 28.95, 29.48, 31.13, 31.14,

31.56, 51.04, 54.12, 61.19, 130.27, 130.71, 137.09, 138.17, 140.69, 144.43; EI-HRMS *m/z* [M^+] calcd for $C_{18}H_{36}N_3O$ 310.2858, found 310.2845.

[Hex-Oct-C4OH-tr][OTMBS] (f20): 77% yield, light yellow liquid; ^{1}H NMR (400 MHz, $CDCl_3$) δ 0.85-0.90 (m, 2 x CH_3 , 6H), 1.25-1.34 (m, 8 x CH, 16H), 1.67-1.73 (m, CH=CCH$_2$C*H*$_2$, 2H), 1.92-2.08 (m, 2 x NCH$_2$C*H*$_2$ + C*H*$_2$CH$_2$OH, 6H), 2.22 (s, *p*-CH$_3$, 3H), 2.65 (s, 2 x *o*-CH$_3$, 6H), 2.92 (t, *J* = 8.0 Hz, CH=CC*H*$_2$, 2H), 3.71 (t, *J* = 6.0 Hz, CH$_2$C*H*$_2$OH, 2H), 4.38 (t, *J* = 6.0 Hz, CNC*H*$_2$, 2H), 4.66 (t, *J* = 8.0 Hz, CHNC*H*$_2$, 2H), 6.81 (s, aryl H, 2H), 9.48 (s, C*H*=CCH$_2$, 1H); ^{13}C NMR (100 MHz, $CDCl_3$) δ 14.08, 14.22, 20.94, 22.54, 22.75, 23.19, 23.16, 23.82, 26.01, 26.43, 28.99, 29.00, 29.13, 29.48, 31.14, 31.55, 31.82, 51.05, 54.14, 61.22, 130.28, 130.74, 137.15, 138.29, 140.47, 144.43; EI-HRMS *m/z* [M^+] calcd for $C_{20}H_{40}N_3O$ 338.3171, found 338.3157.

[Oct-Et-C4OH-tr][OTMBS] (f21): 62% yield, light yellow liquid; ^{1}H NMR (400 MHz, $CDCl_3$) δ 0.87 (t, *J* = 8.0 Hz, CH$_3$, 3H), 1.24-1.30 (m, 5 x CH$_2$, 10H), 1.62 (t, *J* = 6.0 Hz, CH$_3$, 3H), 1.68-1.74 (m, CH=CCH$_2$C*H*$_2$, 2H), 1.97-2.07 (m, NCH$_2$C*H*$_2$ + C*H*$_2$CH$_2$OH, 4H), 2.22 (s, *p*-CH$_3$, 3H), 2.62 (s, 2 x *o*-CH$_3$, 6H), 2.95 (t, *J* = 8.0 Hz, CH=CC*H*$_2$, 2H), 3.71 (t, *J* = 6.0 Hz, CH$_2$C*H*$_2$OH, 2H), 4.48 (q, *J* = 8.0 Hz, NC*H*$_2$CH$_3$, 2H), 4.63 (t, *J* = 6.0 Hz, NC*H*$_2$CH$_2$, 2H), 6.80 (s, aryl H, 2H), 9.35 (s, C*H*=CCH$_2$, 1H); ^{13}C NMR (100 MHz, $CDCl_3$) δ 14.18, 14.25, 20.95, 22.76, 23.12, 23.12, 23.75, 26.36,

28.99, 29.18, 29.50, 31.44, 31.85, 46.48, 54.14, 61.26, 130.06, 130.79, 137.23, 138.59, 139.90, 144.31; EI-HRMS *m/z* [M^+] calcd for $C_{16}H_{32}N_3O$ 282.2545, found 282.2534.

[Oct-Bu-C4OH-tr][OTMBS] (f22): 94% yield, light yellow liquid; ^{1}H NMR (400 MHz, $CDCl_3$) δ 0.87 (t, *J* = 8.0 Hz, CH_3, 3H), 0.99 (t, *J* = 6.0 Hz, CH_3, 3H), 1.24-1.30 (m, 5 x CH_2, 10H), 1.35-1.45 (m, CH_2, 2H), 1.68-1.74 (m, CH=CCH_2CH_2, 2H), 1.90-2.05 (m, 2 x NCH_2CH_2 + CH_2CH_2OH, 6H), 2.22 (s, *p*-CH_3, 3H), 2.64 (s, 2 x *o*-CH_3, 6H), 2.93 (t, *J* = 8.0 Hz, CH=CCH_2, 2H), 3.71 (t, *J* = 6.0 Hz, CH_2CH_2OH, 2H), 4.40 (t, *J* = 8.0 Hz, $CNCH_2$, 2H), 4.65 (t, *J* = 6.0 Hz, $CHNCH_2$, 2H), 6.80 (s, aryl H, 2H), 9.46 (s, CH=CCH_2, 1H); ^{13}C NMR (100 MHz, $CDCl_3$) δ 13.53, 14.24, 19.72, 20.94, 22.76, 23.15, 23.27, 23.81, 26.34, 28.97, 29.18, 29.51, 30.91, 31.50, 31.84, 50.80, 54.15, 61.26, 130.23, 130.76, 137.20, 138.45, 140.17, 144.44; EI-HRMS *m/z* [M^+] calcd for $C_{18}H_{36}N_3O$ 310.2858, found 310.2849.

[Oct-Pent-C4OH-tr][OTMBS] (f23): 83% yield, light yellow liquid; ^{1}H NMR (400 MHz, $CDCl_3$) δ 0.85-0.95 (m, 2 x CH_3 , 6H), 1.24-1.40 (m, 7 x CH_2, 14H), 1.68-1.75 (m, CH=CCH_2CH_2, 2H), 1.92-2.07 (m, 2 x NCH_2CH_2 + CH_2CH_2OH, 6H), 2.22 (s, *p*-CH_3, 3H), 2.64 (s, 2 x *o*-CH_3, 6H), 2.92 (t, *J* = 6.0 Hz, CH=CCH_2, 2H), 3.72 (t, *J* = 6.0 Hz, CH_2CH_2OH, 2H), 4.39 (t, *J* = 8.0 Hz, $CNCH_2$, 2H), 4.66 (t, *J* = 8.0 Hz, $CHNCH_2$, 2H), 6.81 (s, aryl H, 2H), 9.48 (s, CH=CCH_2, 1H); ^{13}C NMR (100 MHz, $CDCl_3$) δ 13.91, 14.24, 20.93, 22.15, 22.76, 23.17, 23.26, 23.81, 26.35, 28.47, 28.67, 28.98, 29.18, 29.51,

31.52, 31.85, 51.02, 54.13, 61.25, 130.19, 130.75, 137.17, 138.38, 140.30, 144.43; EI-HRMS *m/z* [M$^+$] calcd for $C_{19}H_{38}N_3O$ 324.3015, found 324.3005.

[Oct-Hex-C4OH-tr][OTMBS] (f24): 83% yield, light yellow liquid; ^{1}H NMR (400 MHz, $CDCl_3$) δ 0.85-0.91 (m, 2 x CH_3, 6H), 1.24-1.33 (m, 8 x CH_2, 16H), 1.67-1.73 (m, $CH{=}CCH_2CH_2$, 2H), 1.89-2.07 (m, 2 x NCH_2CH_2 + CH_2CH_2OH, 6H), 2.22 (s, *p*-CH_3, 3H), 2.65 (s, 2 x *o*-CH_3, 6H), 2.92 (t, J = 8.0 Hz, $CH{=}CCH_2$, 2H), 3.70 (t, J = 6.0 Hz, CH_2CH_2OH, 2H), 4.39 (t, J = 6.0 Hz, $CNCH_2$, 2H), 4.65 (t, J = 6.0 Hz, $CHNCH_2$, 2H), 6.81 (s, aryl H, 2H), 9.46 (s, $CH{=}CCH_2$, 1H); ^{13}C NMR (100 MHz, $CDCl_3$) δ 14.06, 14.23, 20.93, 22.51, 22.76, 23.18, 23.24, 23.80, 26.08, 26.34, 28.94, 28.98, 29.18, 29.52, 31.14, 31.54, 31.85, 51.04, 54.11, 61.21, 130.21, 130.72, 137.13, 138.27, 140.47, 144.43; EI-HRMS *m/z* [M$^+$] calcd for $C_{20}H_{40}N_3O$ 338.3171, found 338.3158.

[Oct-Oct-C4OH-tr][OTMBS] (f25): 67% yield, light yellow liquid; ^{1}H NMR (400 MHz, $CDCl_3$) δ 0.85-0.90 (m, 2 x CH_3, 6H), 1.24-1.34 (m, 10 x CH_2, 20H), 1.67-1.74 (m, $CH{=}CCH_2CH_2$, 2H), 1.93-2.06 (m, 2 x NCH_2CH_2 + CH_2CH_2OH, 2H), 2.22 (s, *p*-CH_3, 3H), 2.65 (s, 2 x *o*-CH_3, 6H), 2.91 (t, J = 8.0 Hz, $CH{=}CCH_2$, 2H), 3.71 (t, J = 6.0 Hz, CH_2CH_2OH, 2H), 4.38 (t, J = 8.0 Hz, $CNCH_2$, 2H), 4.66 (t, J = 8.0 Hz, $CHNCH_2$, 2H), 6.81 (s, aryl H, 2H), 9.47 (s, $CH{=}CCH_2$, 1H); ^{13}C NMR (100 MHz, $CDCl_3$) δ 14.23, 14.24, 20.94, 22.75, 22.76, 23.18, 23.26, 23.81, 26.35, 26.43, 28.99, 29.00, 29.00, 29.13,

29.19, 29.52, 31.54, 31.82, 31.86, 51.04, 54.05, 61.24, 130.24, 130.75, 137.16, 138.33, 140.38, 144.41; EI-HRMS *m/z* [M^+] calcd for $C_{22}H_{44}N_3O$ 366.3484, found 366.3475.

[Oct-*i*-Bu-C4OH-tr][OTMBS] (f26): 86% yield, light yellow liquid; ^{1}H NMR (400 MHz, $CDCl_3$) δ 0.85-0.89 (m, CH_3, 3H), 1.01 (d, *J* = 4.0 Hz, 2 x CHC*H*$_3$, 6H), 1.23-1.30 (m, 5 x CH_2, 12H), 1.73-1.79 (m, CH=CCH$_2$C*H*$_2$, 2H), 1.98 (m, NCH$_2$C*H*$_2$ + C*H*$_2$CH$_2$OH, 4H), 2.23 (s, *p*-CH$_3$, 3H), 2.28-2.34 (m, C*H*CH$_3$, 1H), 2.56 (s, 2 x *o*-CH$_3$, 6H), 2.92 (t, *J* = 6.0 Hz, CH=CC*H*$_2$, 2H), 3.76 (t, *J* = 6.0 Hz, CH$_2$C*H*$_2$OH, 2H), 4.24 (d, *J* = 4.0 Hz, CNC*H*$_2$, 2H), 4.65 (t, *J* = 8.0 Hz, CHNC*H*$_2$, 2H), 6.79 (s, aryl H, 2H), 9.29 (s, C*H*=CCH$_2$, 1H); ^{13}C NMR (100 MHz, $CDCl_3$) δ 14.27, 19.89, 19.90, 21.00, 22.78, 22.99, 23.52, 23.81, 26.35, 28.96, 29.19, 29.45, 31.32, 31.86, 54.25, 57.77, 61.59, 130.01, 130.95, 137.55, 138.38, 139.40, 144.74; EI-HRMS *m/z* [M^+] calcd for $C_{18}H_{36}N_3O$ 310.2858, found 310.2851.

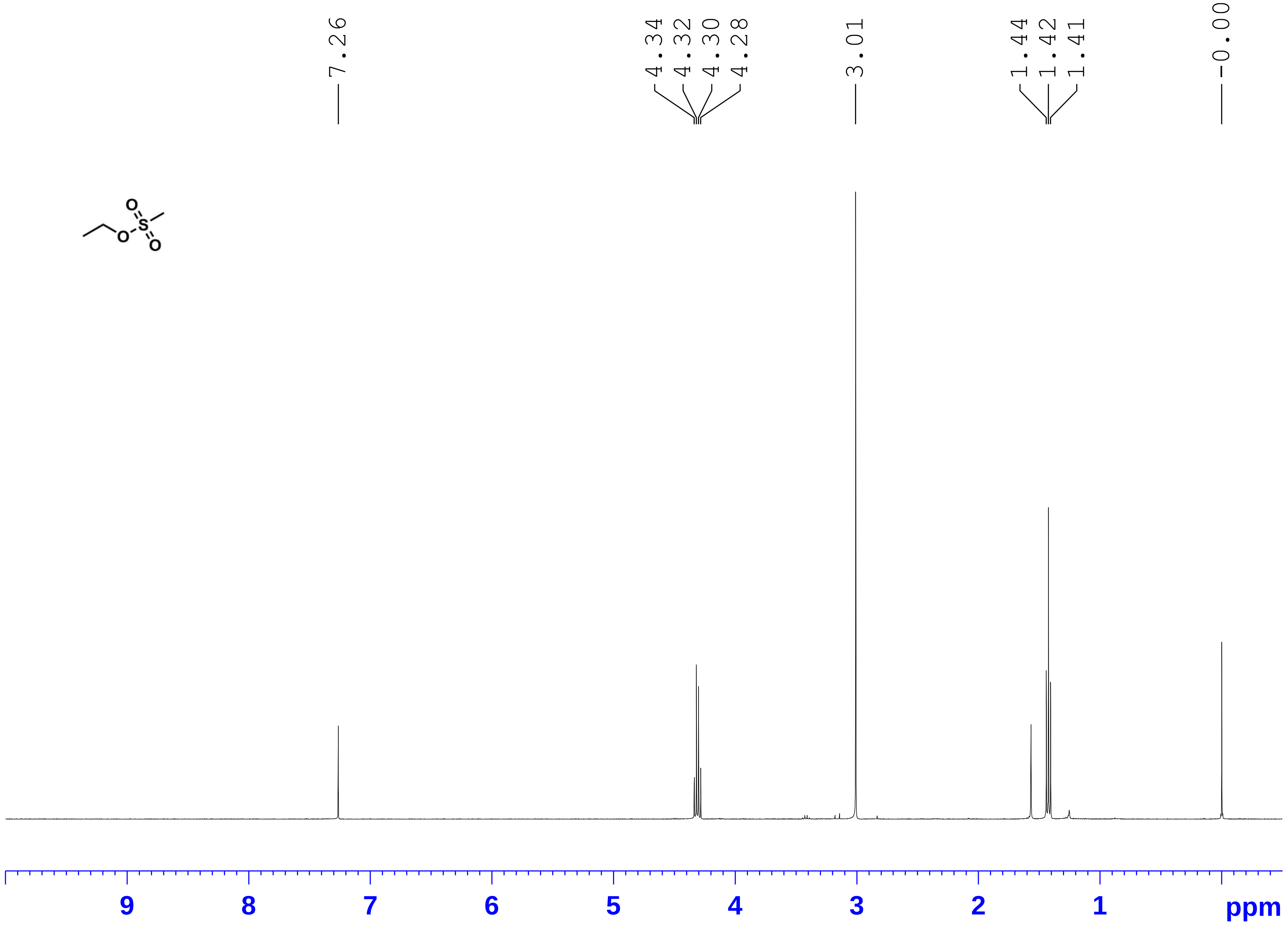
7.26
4.34
4.32
4.30
4.28
3.01
1.44
1.42
1.41
0.00
9
8
7
6
5
4
3
2
1
ppm

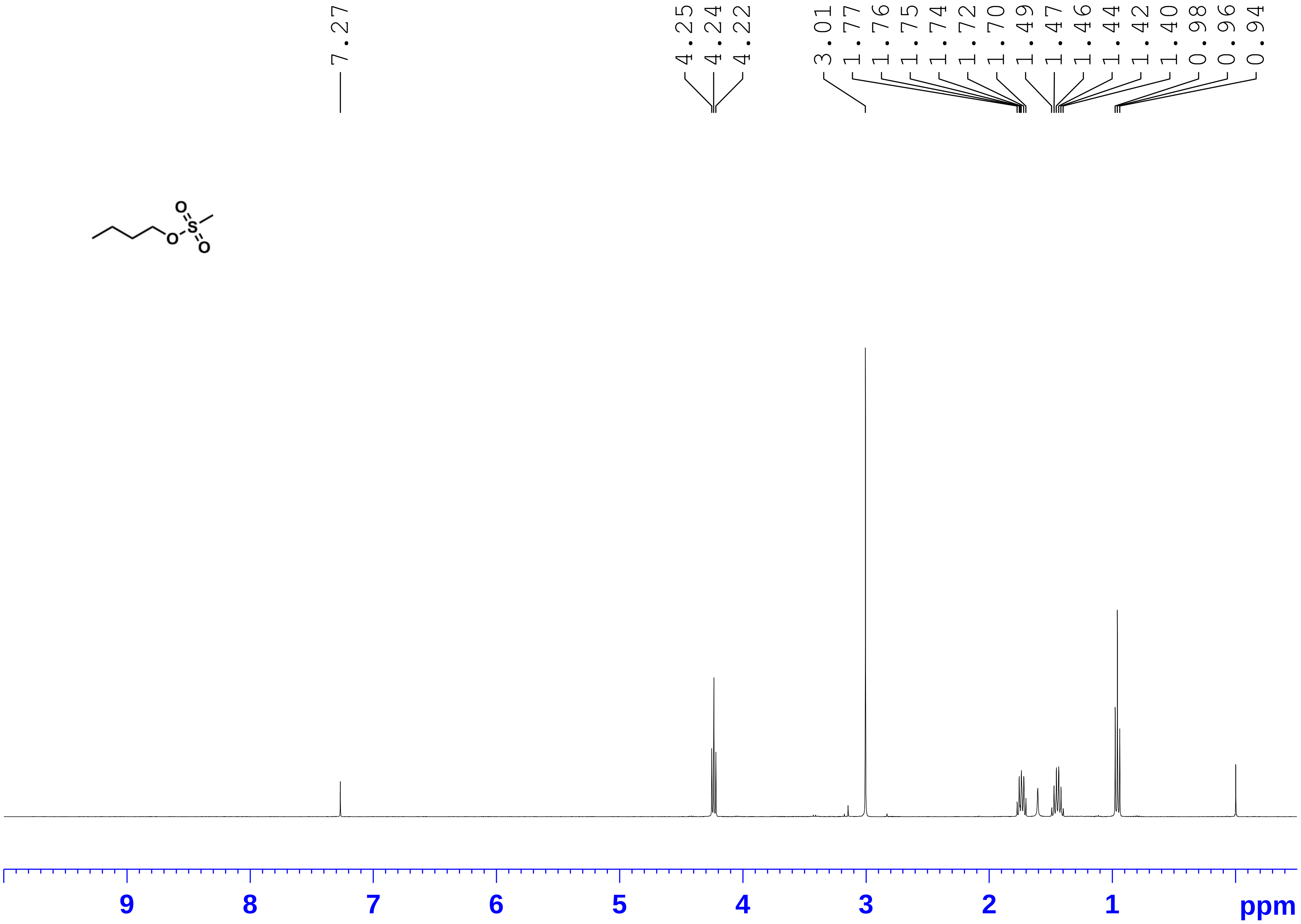
7.27
4.25
4.24
4.22
3.01
1.77
1.76
1.75
1.74
1.72
1.70
1.49
1.47
1.46
1.44
1.42
1.40
0.98
0.96
0.94
O
S
O
O
9
8
7
6
5
4
3
2
1
ppm

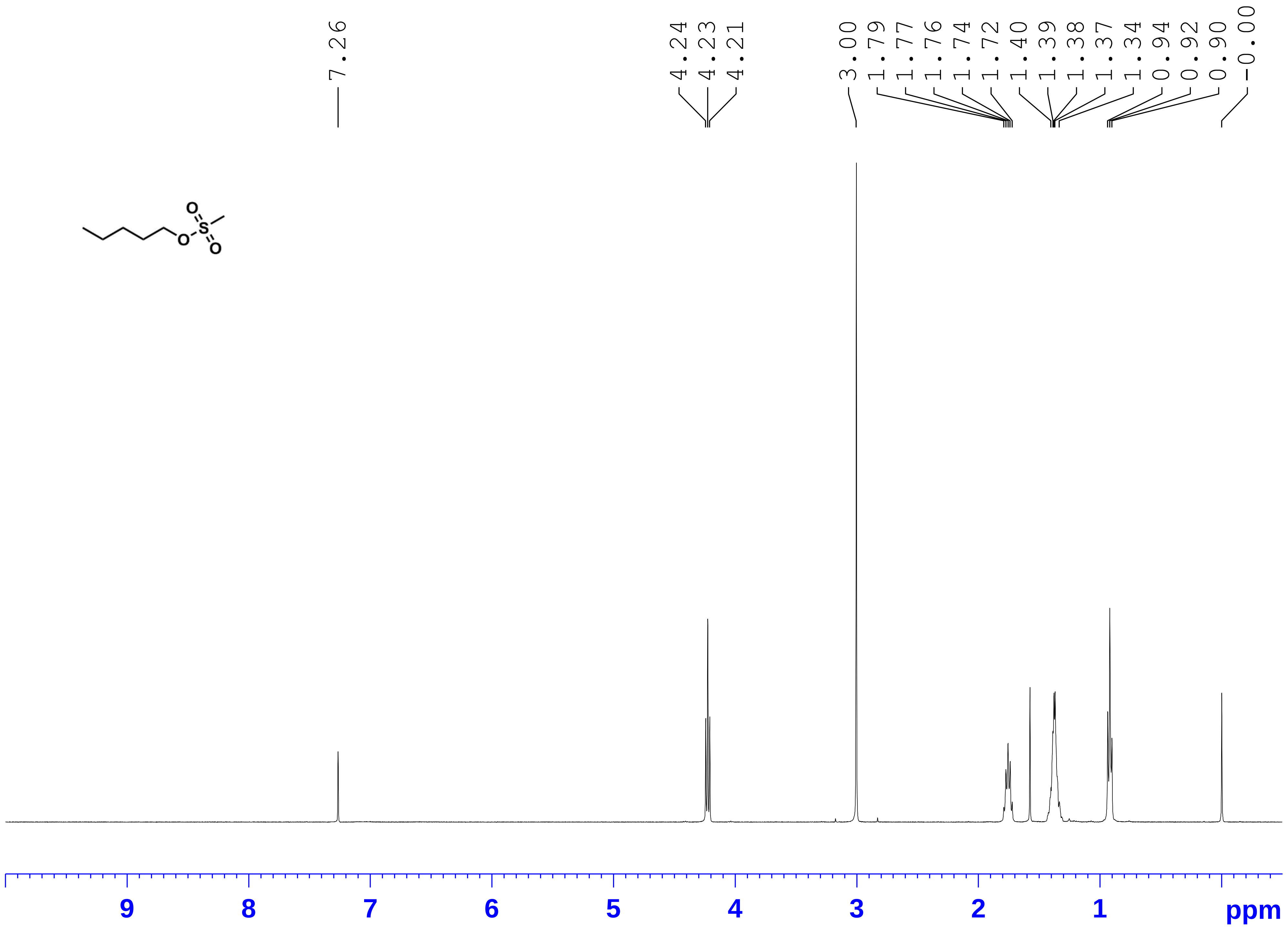
7.26
4.24
4.23
4.21
3.00
1.79
1.77
1.76
1.74
1.72
1.40
1.39
1.38
1.37
1.34
0.94
0.92
0.90
0.00
9
8
7
6
5
4
3
2
1
ppm
O
S
O
O

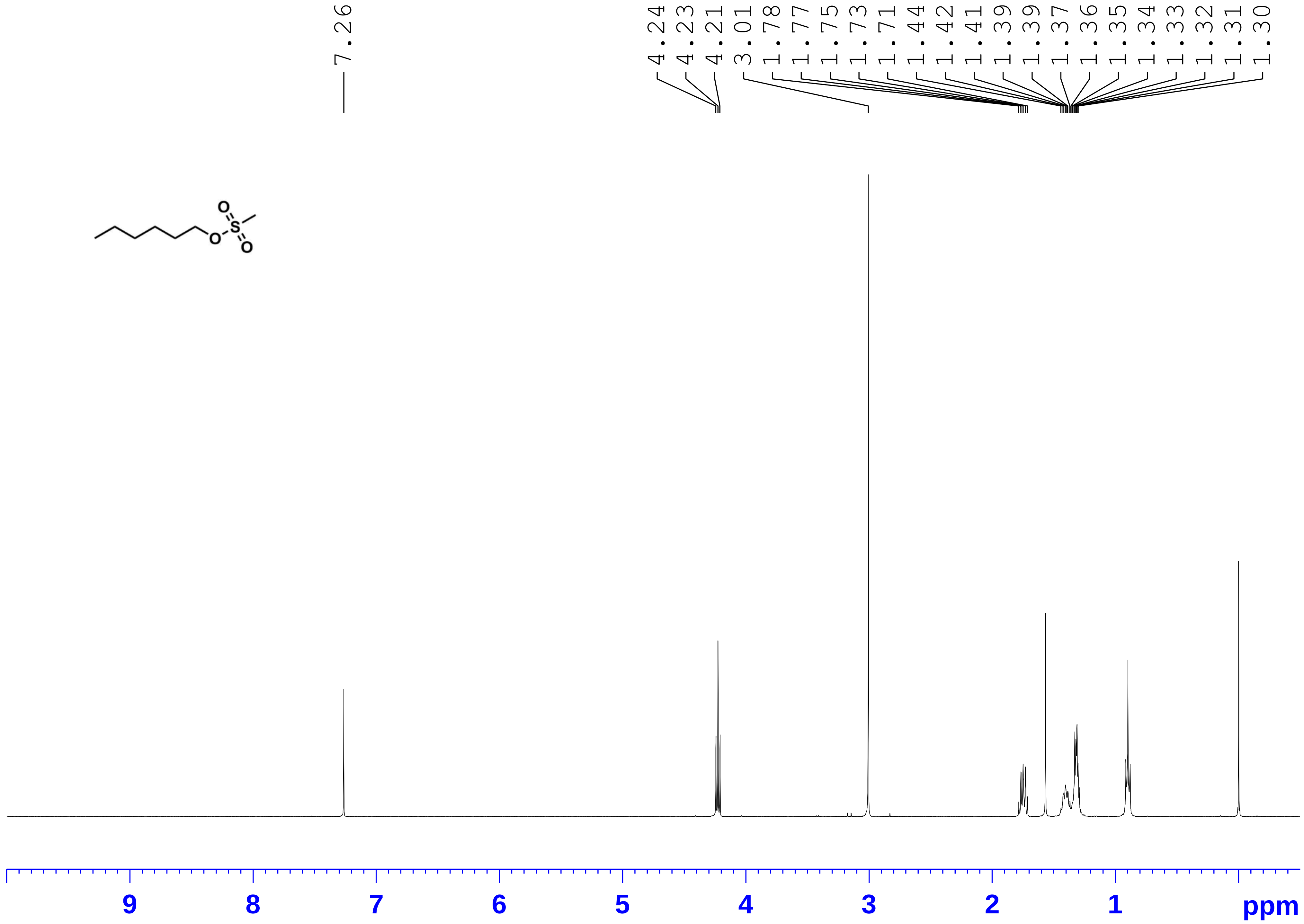

7.26
4.24
4.23
4.21
3.01
1.78
1.77
1.75
1.73
1.71
1.44
1.42
1.41
1.39
1.39
1.37
1.36
1.35
1.34
1.33
1.32
1.31
1.30
O
O
S
O
9
8
7
6
5
4
3
2
1
ppm

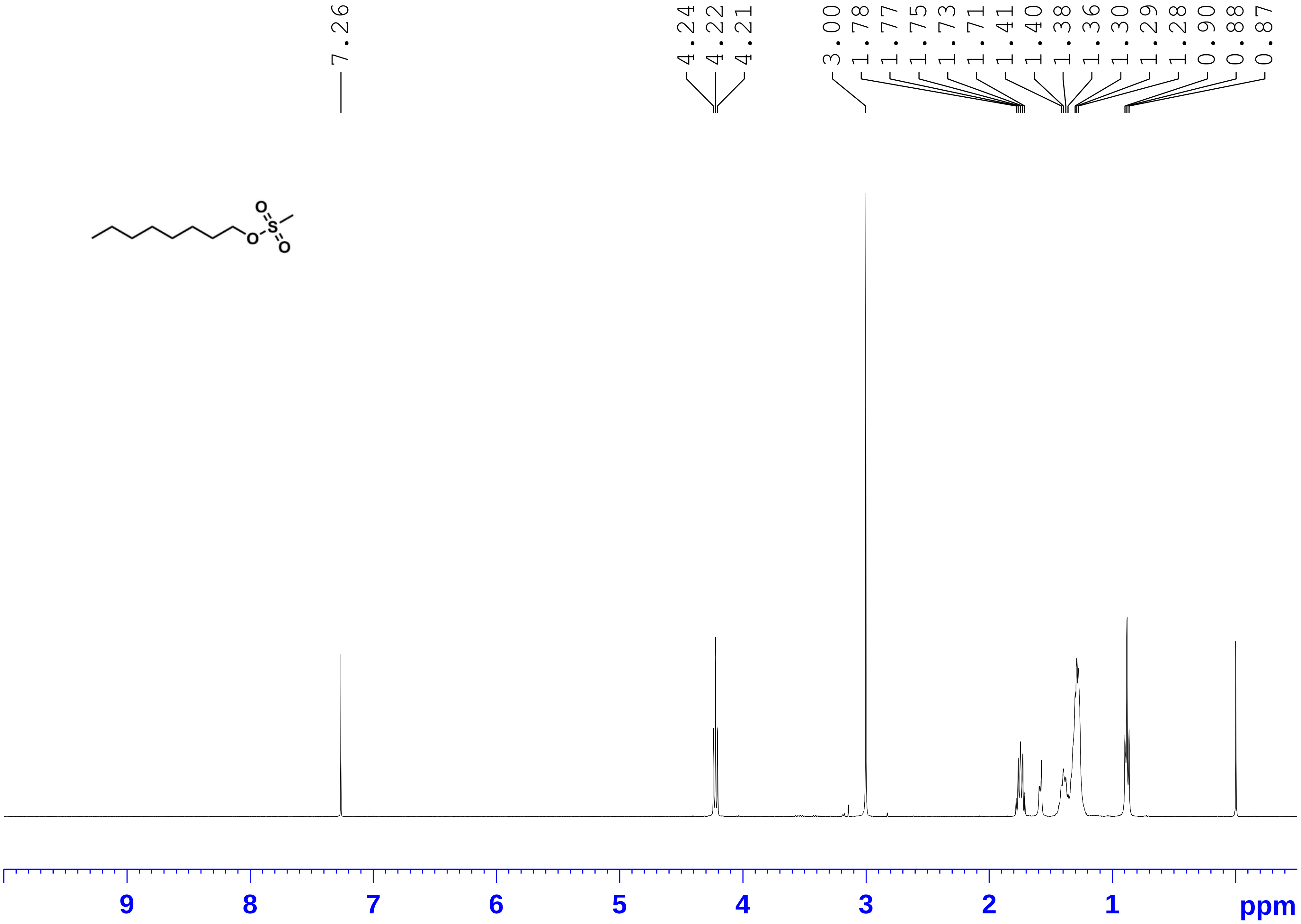

7.26
4.24
4.22
4.21
3.00
1.78
1.77
1.75
1.73
1.71
1.41
1.40
1.38
1.36
1.30
1.29
1.28
0.90
0.88
0.87
9
8
7
6
5
4
3
2
1
ppm

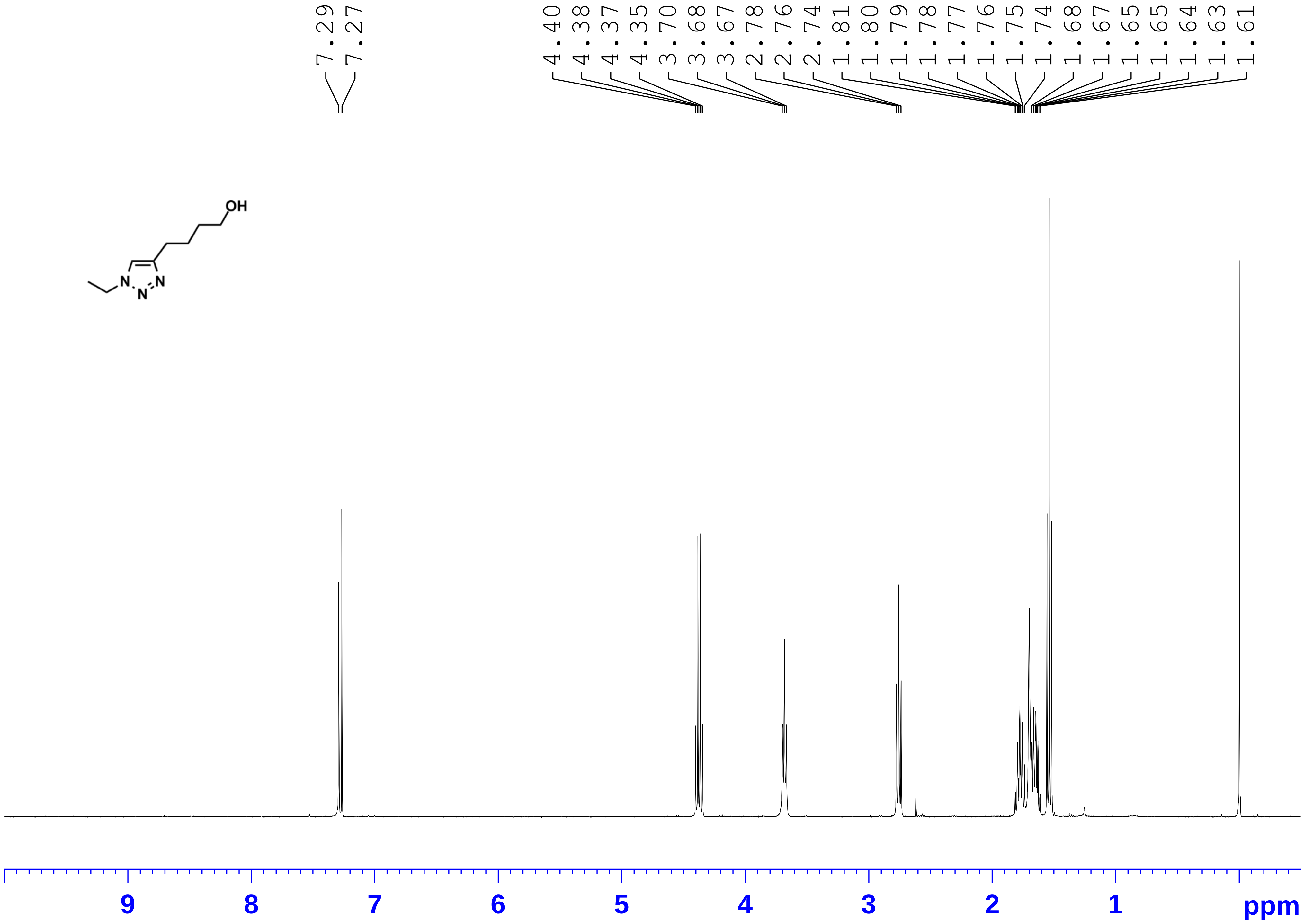

7.29
7.27
4.40
4.38
4.37
4.35
3.70
3.68
3.67
2.78
2.76
2.74
1.81
1.80
1.79
1.78
1.77
1.76
1.75
1.74
1.68
1.67
1.65
1.65
1.64
1.63
1.61
OH
N
N
N
9
8
7
6
5
4
3
2
1
ppm

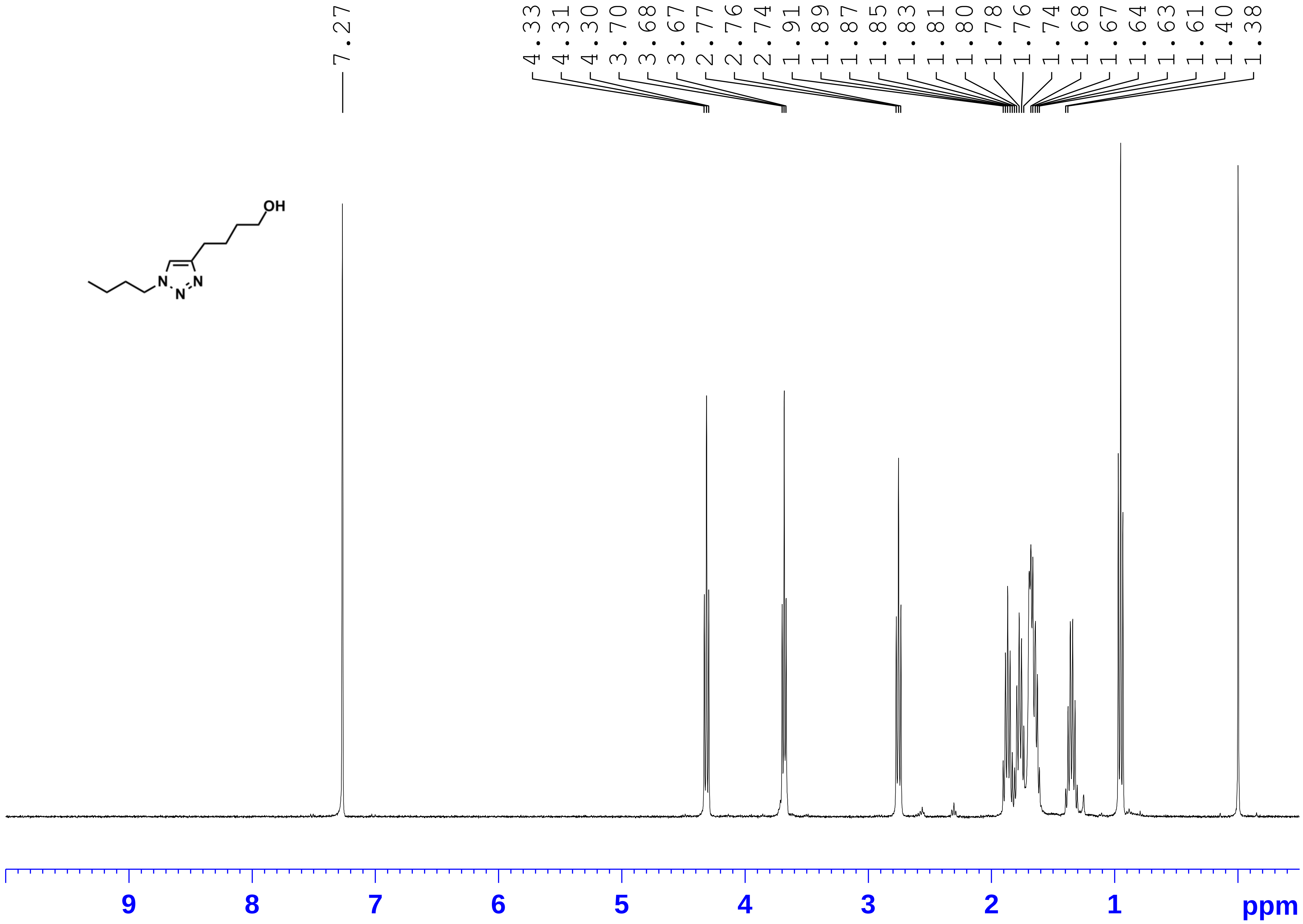

7.27
4.33
4.31
4.30
3.70
3.68
3.67
2.77
2.76
2.74
1.91
1.89
1.87
1.85
1.83
1.81
1.80
1.78
1.76
1.74
1.68
1.67
1.64
1.63
1.61
1.40
1.38
OH
N
N
N
9
8
7
6
5
4
3
2
1
ppm

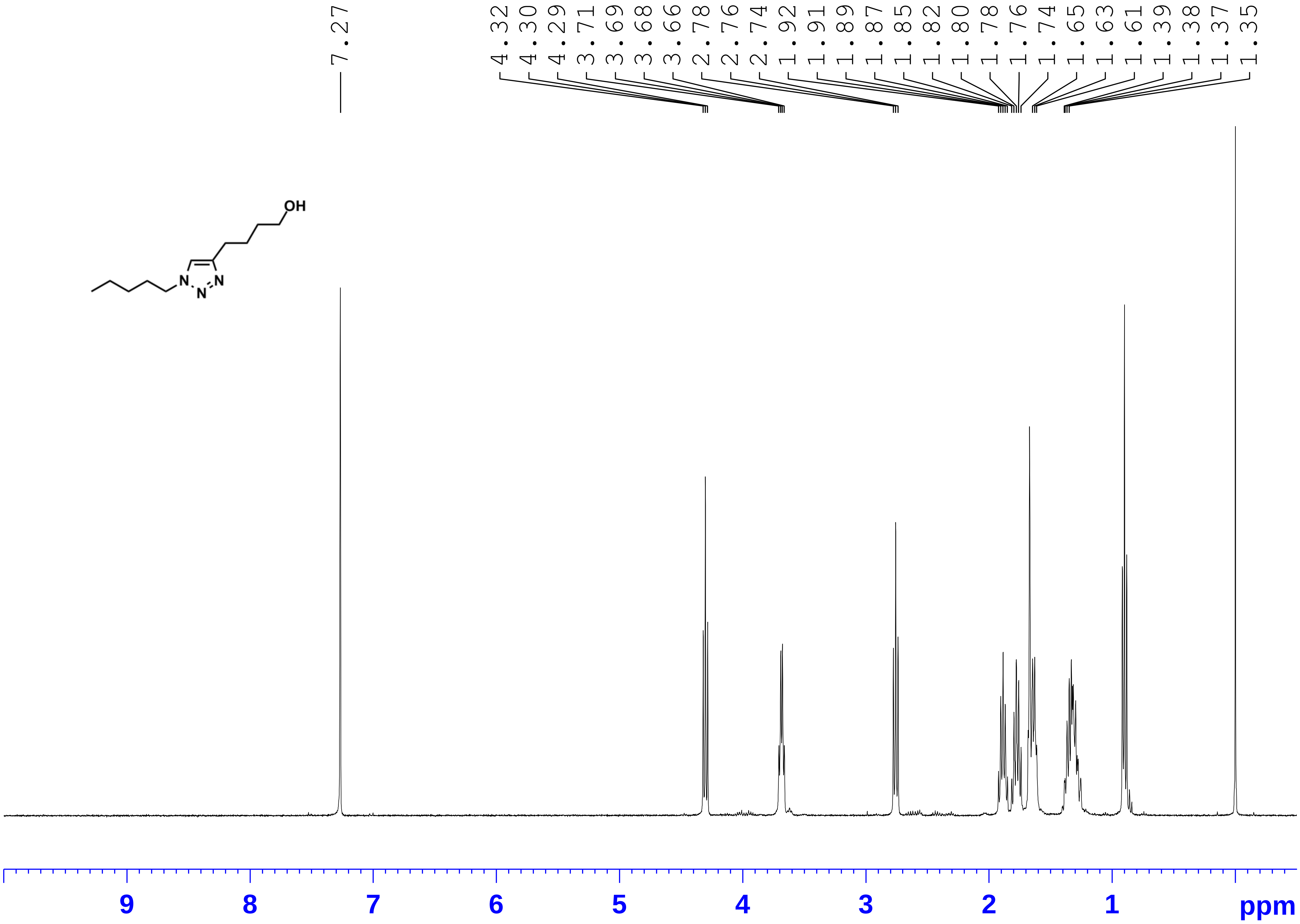

7.27
4.32
4.30
4.29
3.71
3.69
3.68
3.66
2.78
2.76
2.74
1.92
1.91
1.89
1.87
1.85
1.82
1.80
1.78
1.76
1.74
1.65
1.63
1.61
1.39
1.38
1.37
1.35
OH
N
N
N
9
8
7
6
5
4
3
2
1
ppm

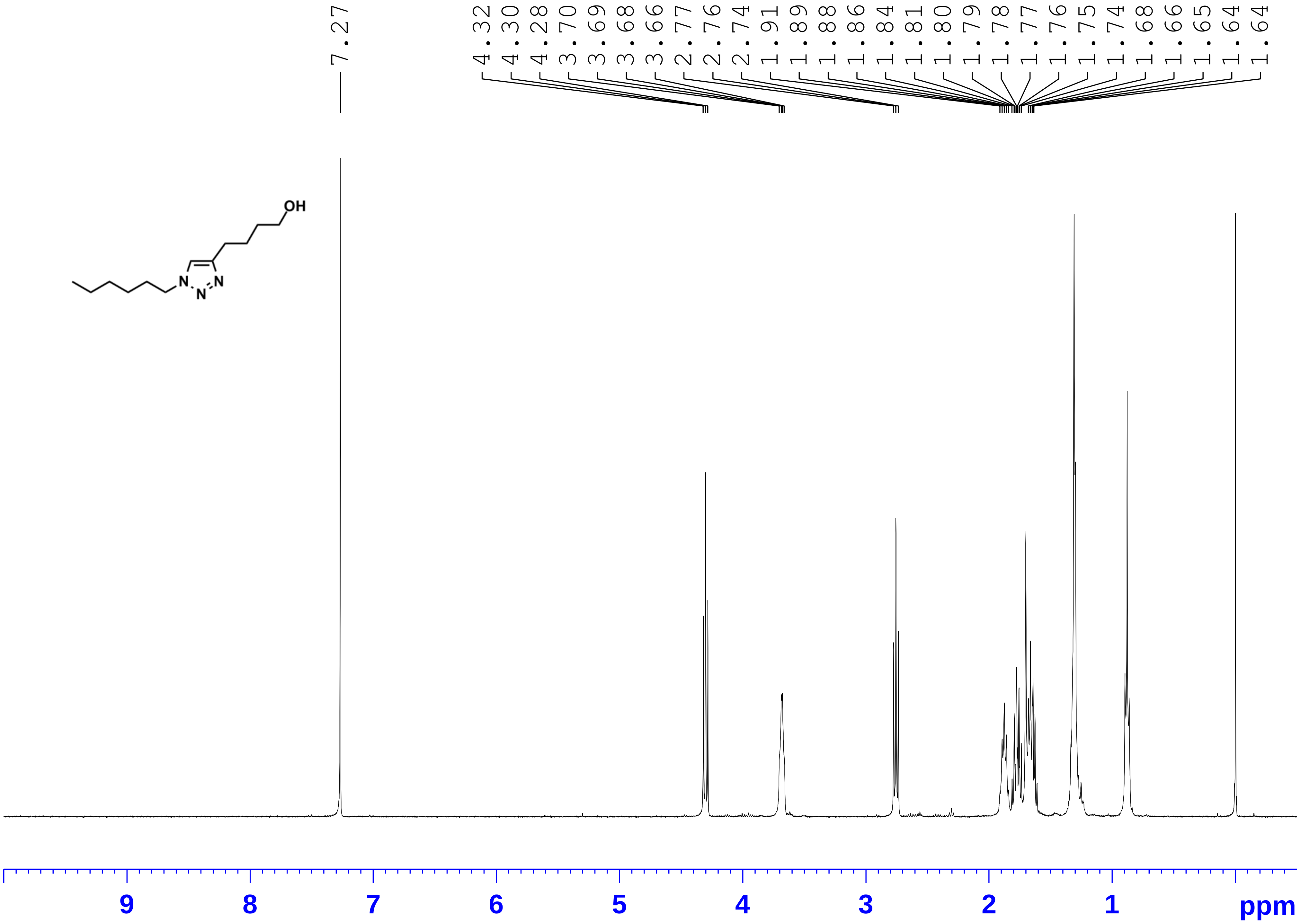

7.27
4.32
4.30
4.28
3.70
3.69
3.68
3.66
2.77
2.76
2.74
1.91
1.89
1.88
1.86
1.84
1.81
1.80
1.79
1.78
1.77
1.76
1.75
1.74
1.68
1.66
1.65
1.64
1.64
OH
N
N
N
9
8
7
6
5
4
3
2
1
ppm

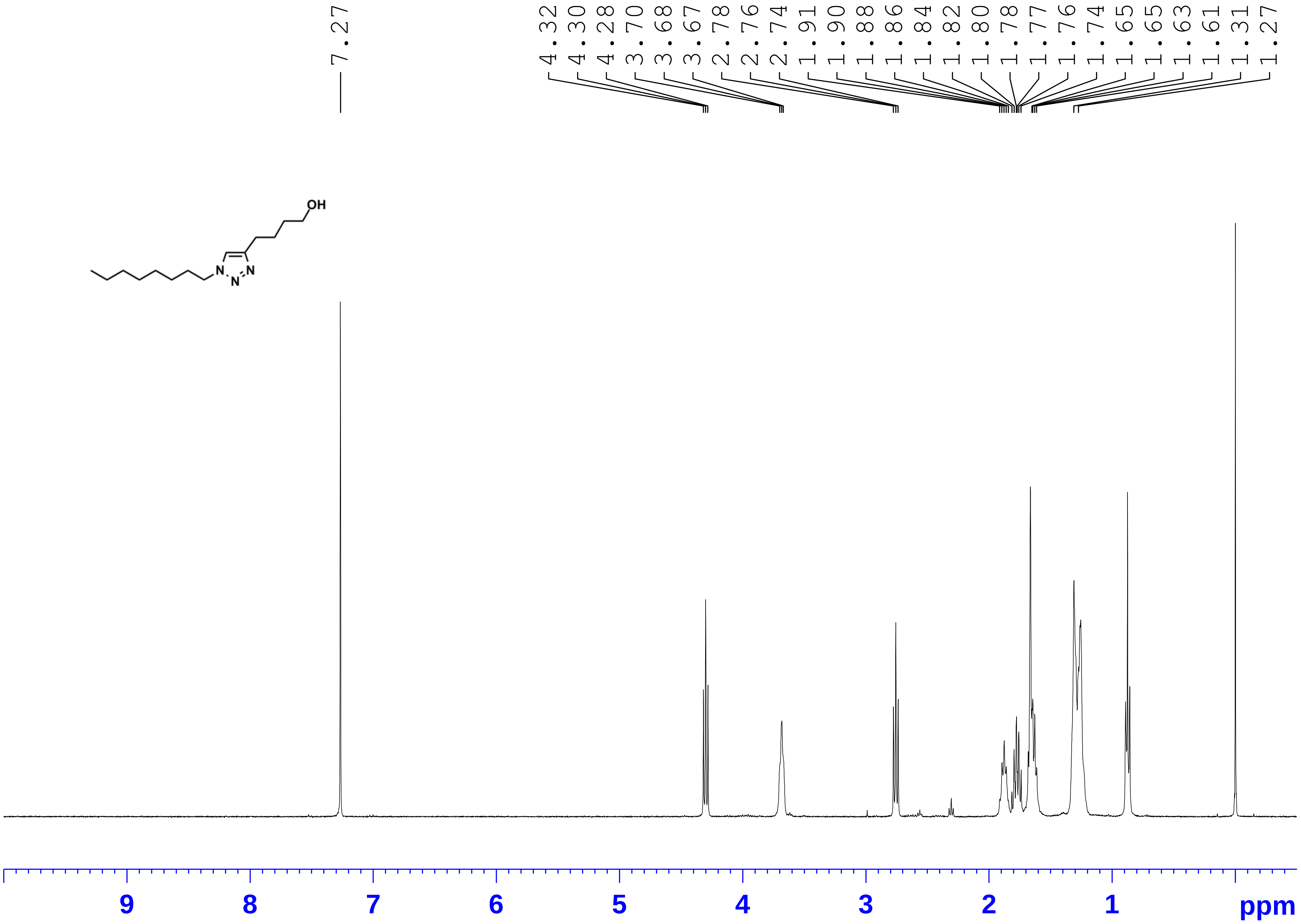

7.27
4.32
4.30
4.28
3.70
3.68
3.67
2.78
2.76
2.74
1.91
1.90
1.88
1.86
1.84
1.82
1.80
1.78
1.77
1.76
1.74
1.65
1.65
1.63
1.61
1.31
1.27
OH
N
N
N
9
8
7
6
5
4
3
2
1
ppm

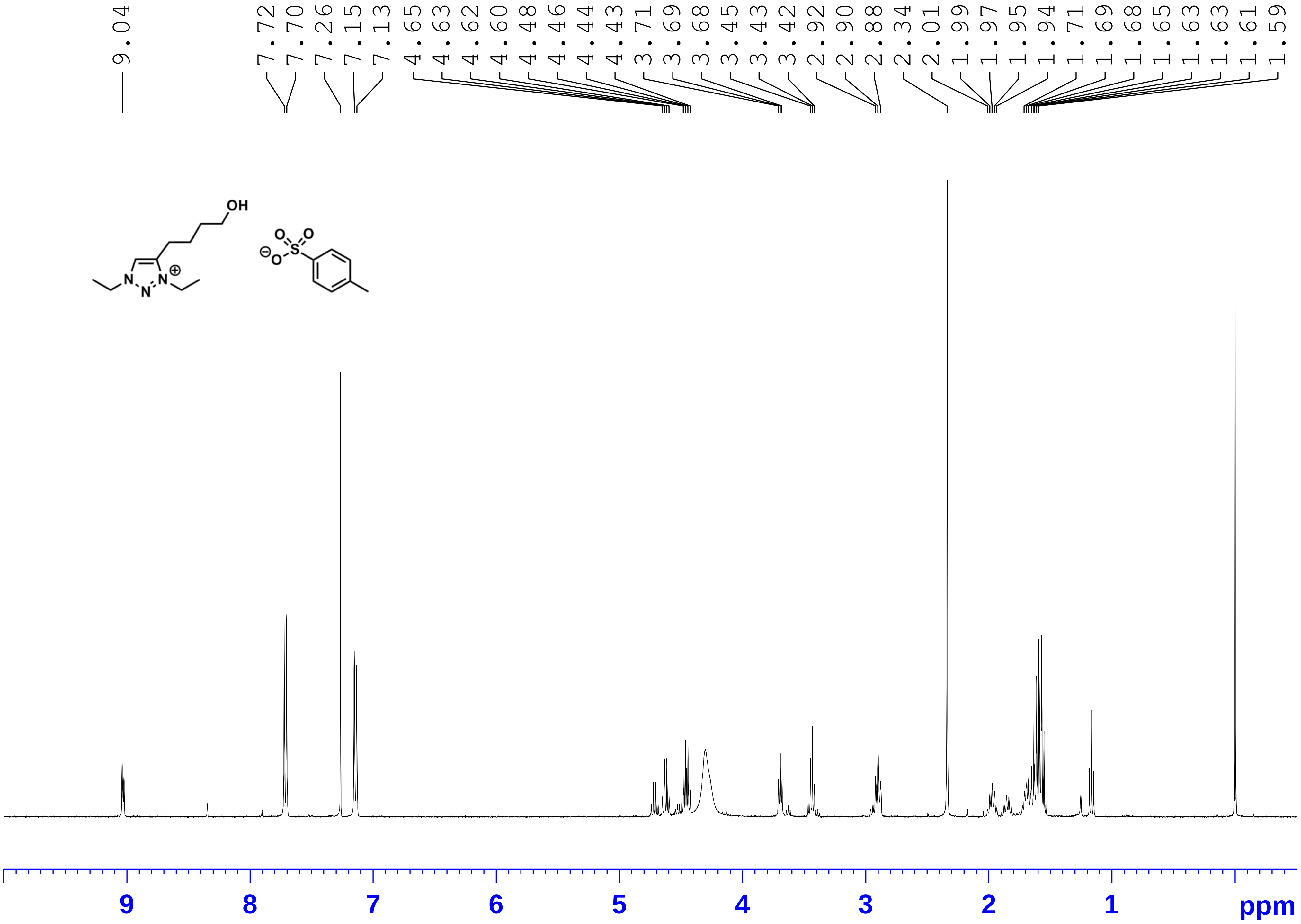

9.04
7.72
7.70
7.26
7.15
7.13
4.65
4.63
4.62
4.60
4.48
4.46
4.44
4.43
3.71
3.69
3.68
3.45
3.43
3.42
2.92
2.90
2.88
2.34
2.01
1.99
1.97
1.95
1.94
1.71
1.69
1.68
1.65
1.63
1.63
1.61
1.59
OH
O
S
O
O
N
N
N
9
8
7
6
5
4
3
2
1
ppm

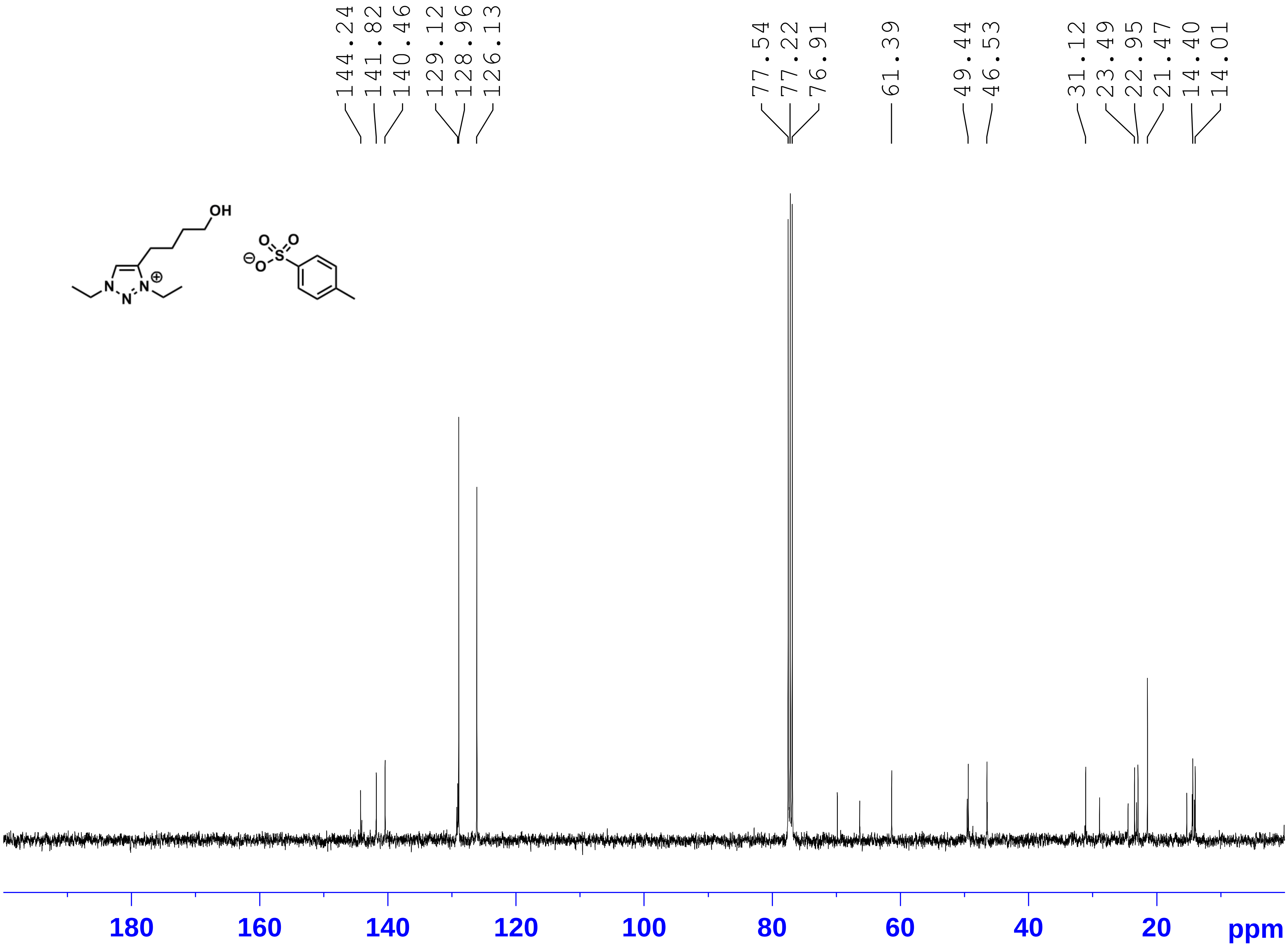

144.24
141.82
140.46
129.12
128.96
126.13
77.54
77.22
76.91
61.39
49.44
46.53
31.12
23.49
22.95
21.47
14.40
14.01
OH
180
160
140
120
100
80
60
40
20
ppm

Spectrum

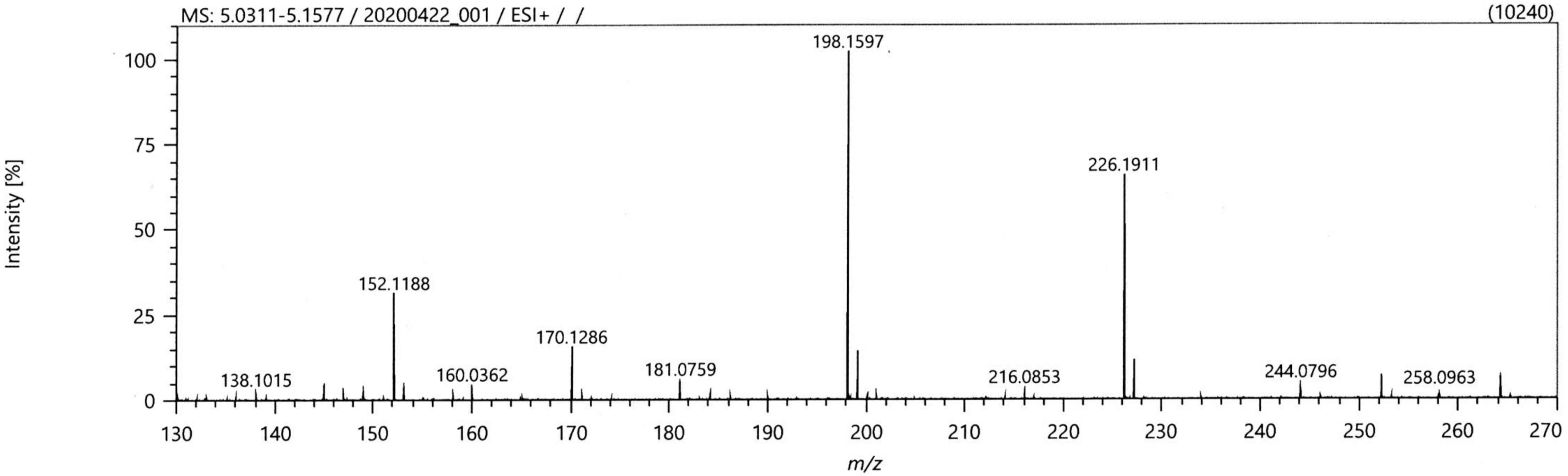

Elemental Composition

Parameters

Tolerance:	±10.00 ppm
Electron:	Odd/Even
Charge:	+1
DBE:	-99.0 - 999.0

Elements Set 1:

Symbol	C	H	O	Na	N	S
Min	0	0	1	0	3	0
Max	1000	4000	1	0	3	0

Results

Mass	Formula	Calculated Mass	Mass Difference [mDa]	Mass Difference [ppm]	DBE
198.15972	C10 H20 N3 O	198.16009	-0.37	-1.87	2.5

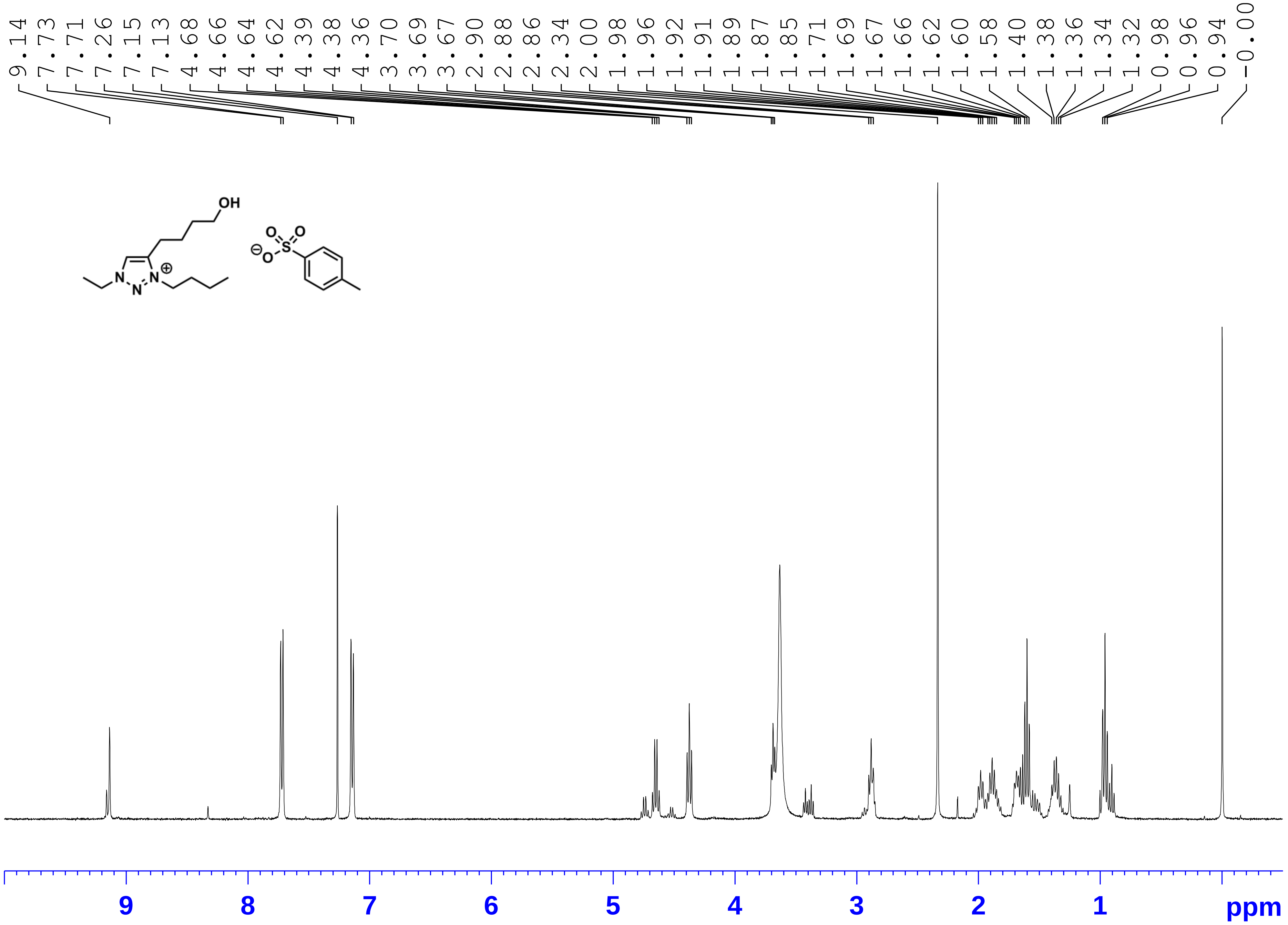

9.14
7.73
7.71
7.26
7.15
7.13
4.68
4.66
4.64
4.62
4.39
4.38
4.36
3.70
3.69
3.67
2.90
2.88
2.86
2.34
2.00
1.98
1.96
1.92
1.91
1.89
1.87
1.85
1.71
1.69
1.67
1.66
1.62
1.60
1.58
1.40
1.38
1.36
1.34
1.32
0.98
0.96
0.94
-0.00
OH
9
8
7
6
5
4
3
2
1
ppm

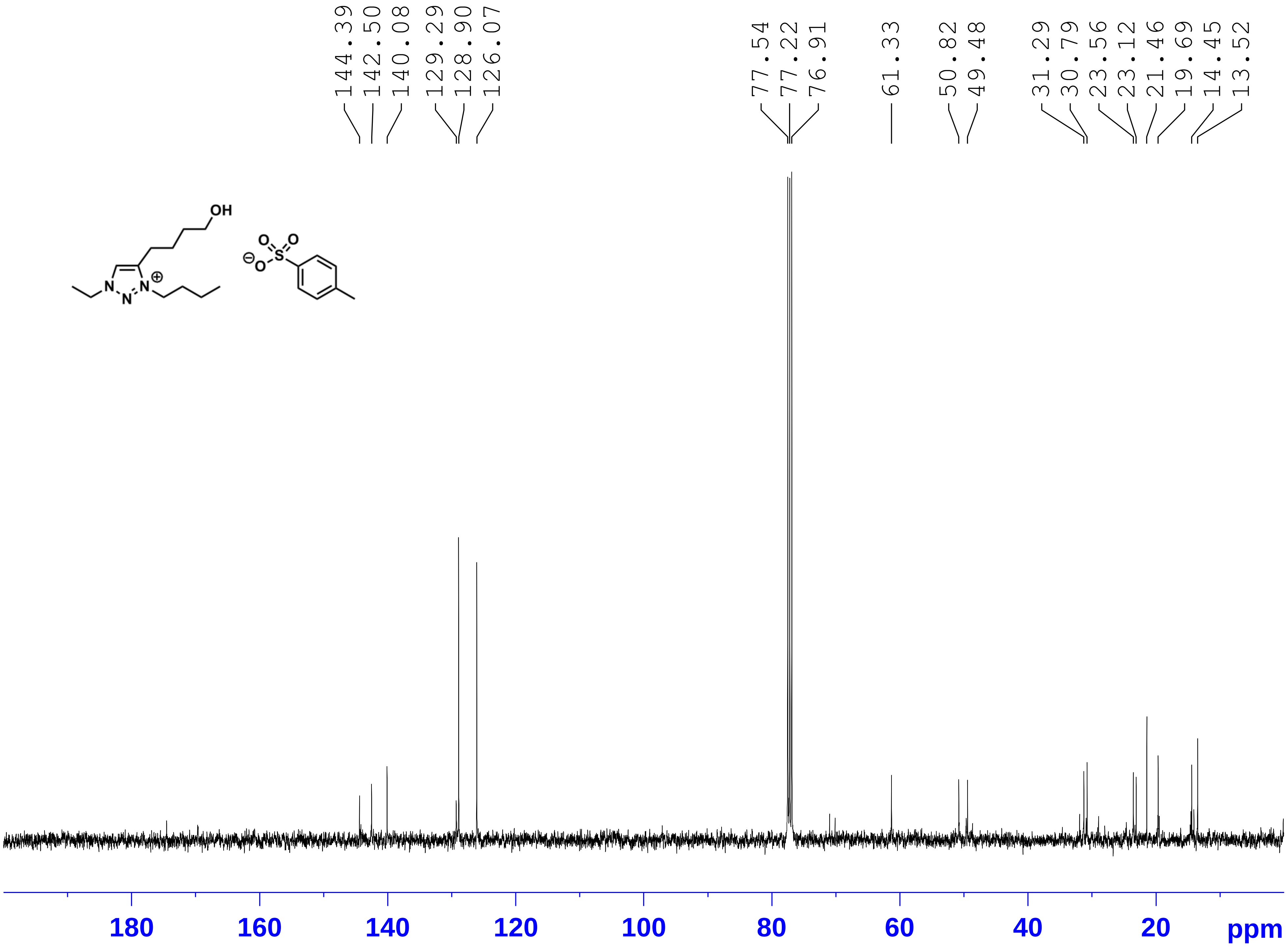

144.39
142.50
140.08
129.29
128.90
126.07
77.54
77.22
76.91
61.33
50.82
49.48
31.29
30.79
23.56
23.12
21.46
19.69
14.45
13.52
OH
O
S
O
O
N
N
N
180
160
140
120
100
80
60
40
20
ppm

Spectrum

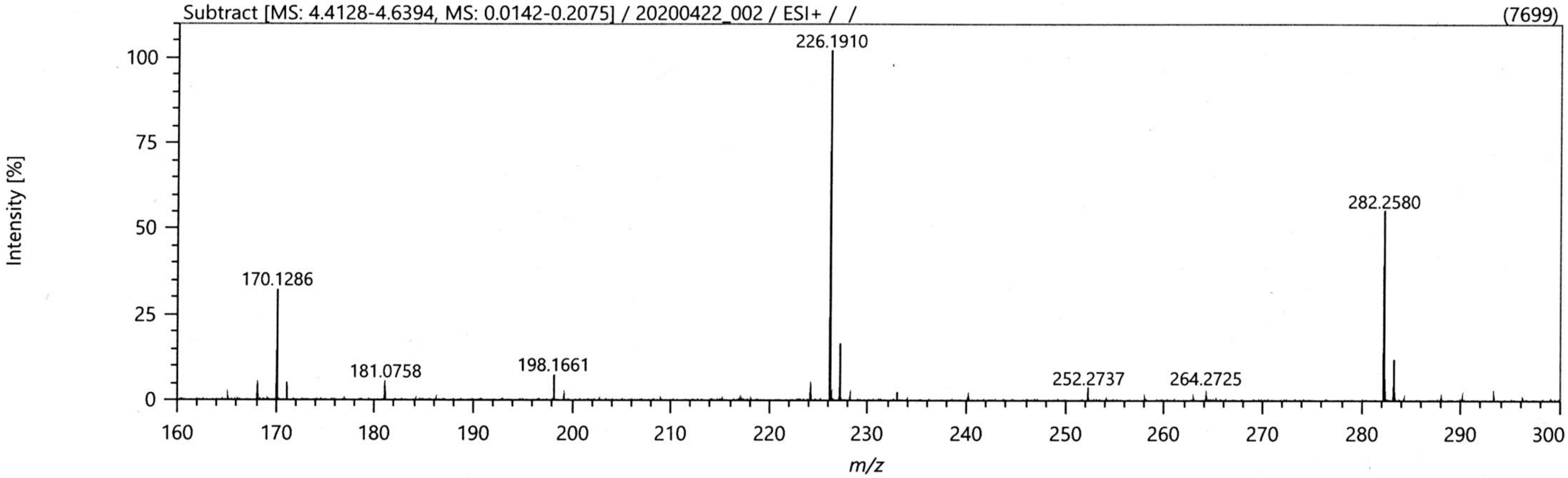

Elemental Composition

Parameters		Elements Set 1:						
Tolerance:	±10.00 ppm	Symbol	C	H	O	Na	N	S
Electron:	Odd/Even	Min	0	0	1	0	3	0
Charge:	+1	Max	1000	4000	1	0	3	0
DBE:	-99.0 - 999.0							

Results

Mass	Formula	Calculated Mass	Mass Difference [mDa]	Mass Difference [ppm]	DBE
226.19098	C12 H24 N3 O	226.19139	-0.41	-1.82	2.5

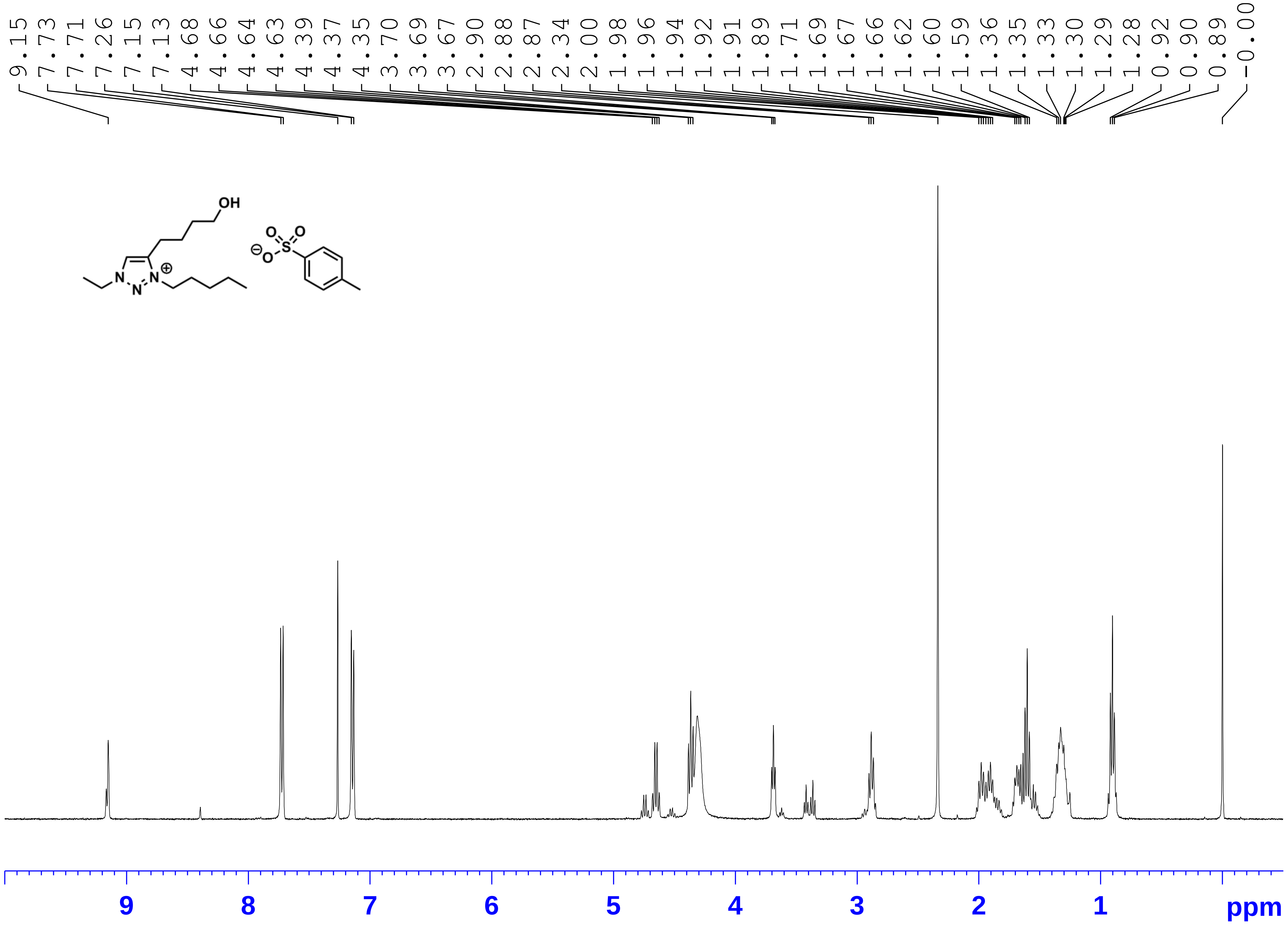
9.15
7.73
7.71
7.26
7.15
7.13
4.68
4.66
4.64
4.63
4.39
4.37
4.35
3.70
3.69
3.67
2.90
2.88
2.87
2.34
2.00
1.98
1.96
1.94
1.92
1.91
1.89
1.71
1.69
1.67
1.66
1.62
1.60
1.59
1.36
1.35
1.33
1.30
1.29
1.28
0.92
0.90
0.89
-0.00
OH
N
N
N
O
S
O
O
9
8
7
6
5
4
3
2
1
ppm

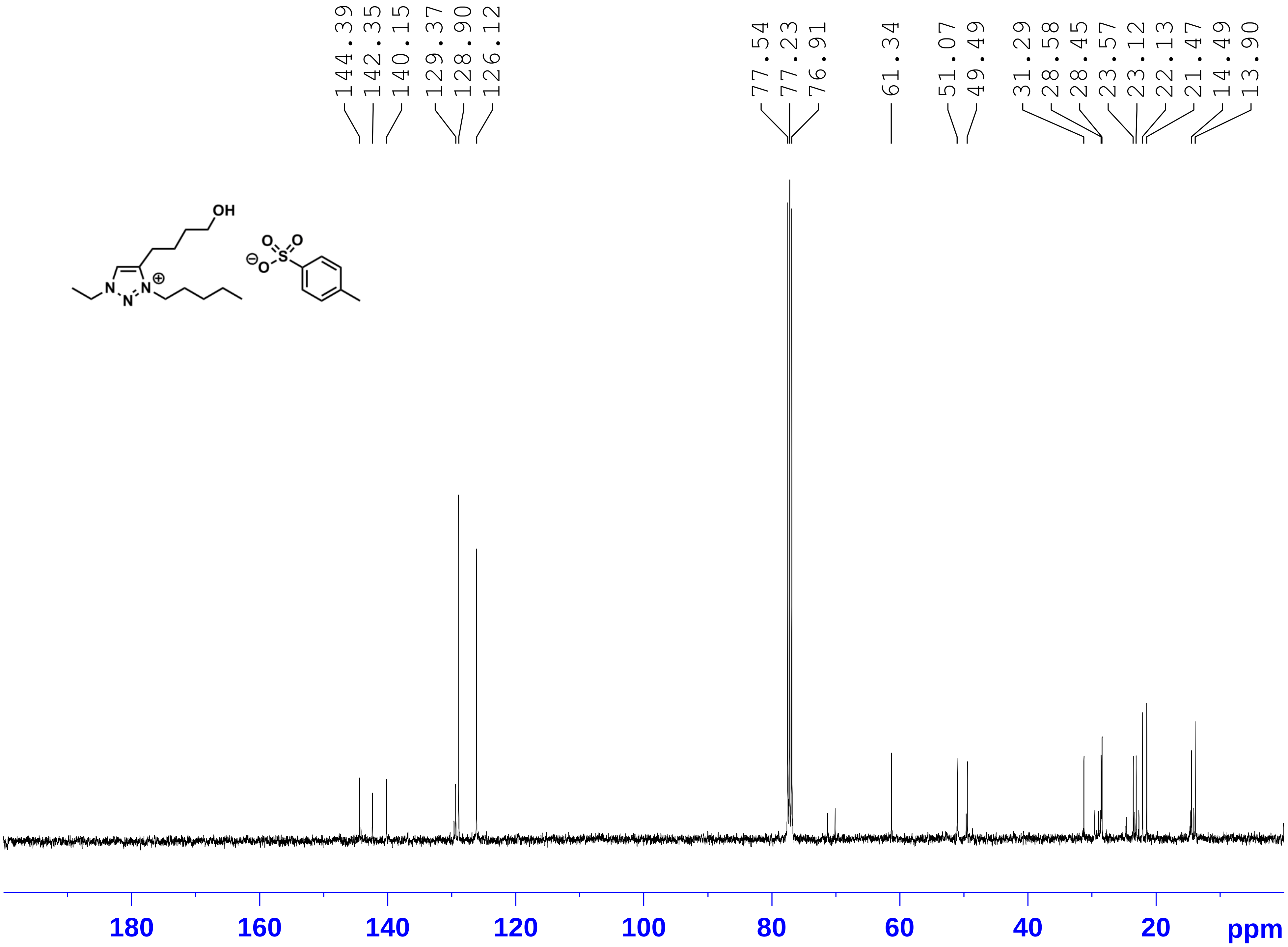

144.39
142.35
140.15
129.37
128.90
126.12
77.54
77.23
76.91
61.34
51.07
49.49
31.29
28.58
28.45
23.57
23.12
22.13
21.47
14.49
13.90
OH
180
160
140
120
100
80
60
40
20
ppm

Spectrum

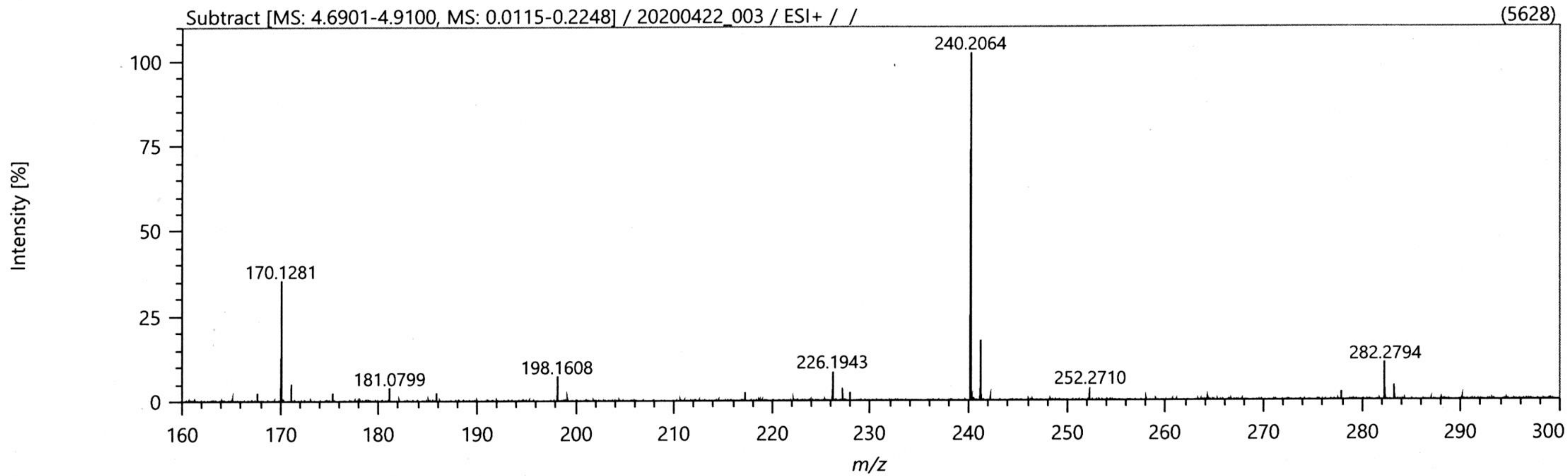

Elemental Composition

Parameters

Tolerance:	±10.00 ppm
Electron:	Odd/Even
Charge:	+1
DBE:	-99.0 - 999.0

Elements Set 1:

Symbol	C	H	O	Na	N	S
Min	0	0	1	0	3	0
Max	1000	4000	1	0	3	0

Results

Mass	Formula	Calculated Mass	Mass Difference [mDa]	Mass Difference [ppm]	DBE
240.20635	C13 H26 N3 O	240.20704	-0.69	-2.86	2.5

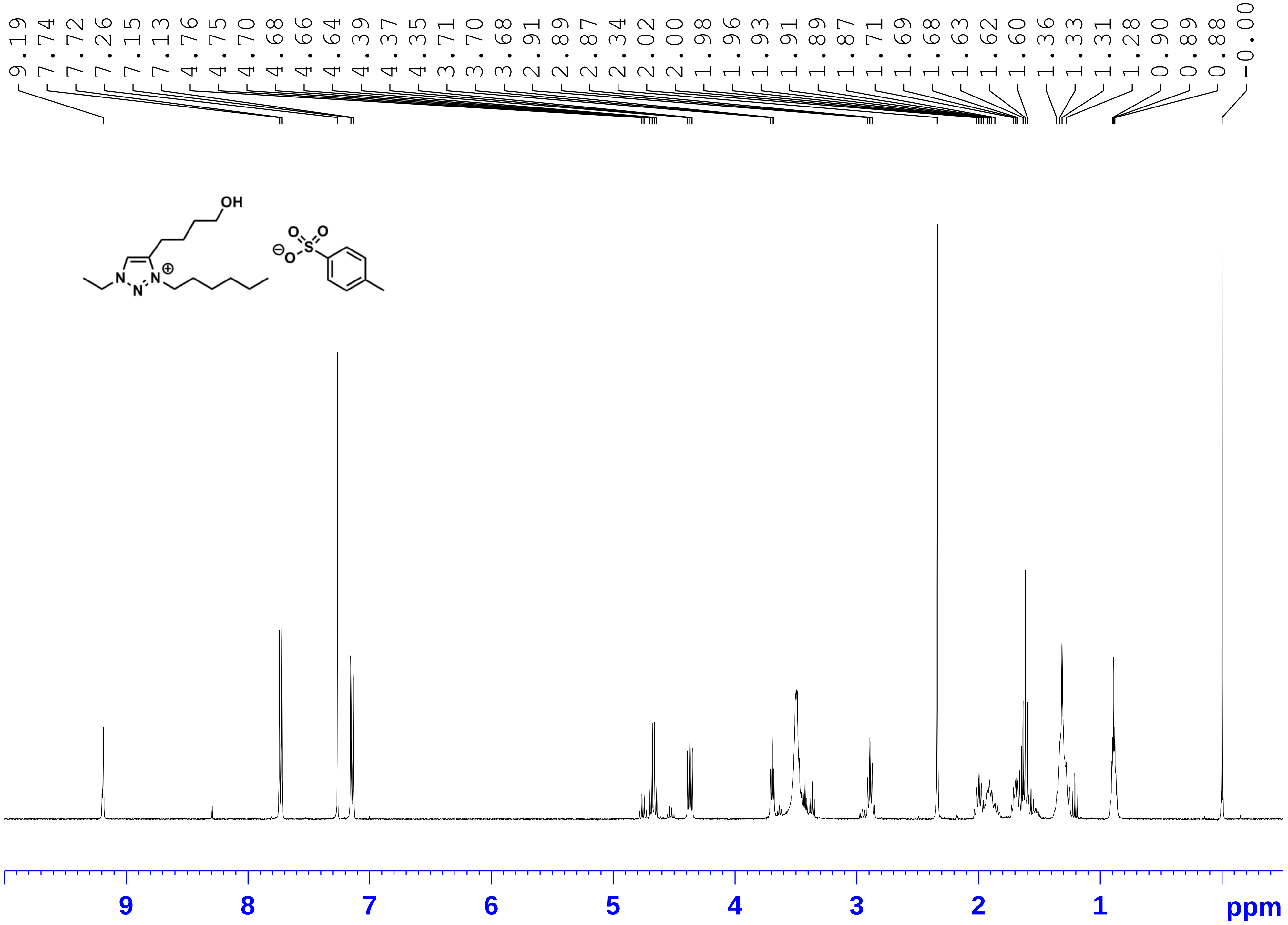
9.19
7.74
7.72
7.26
7.15
7.13
4.76
4.75
4.70
4.68
4.66
4.64
4.39
4.37
4.35
3.71
3.70
3.68
2.91
2.89
2.87
2.34
2.02
2.00
1.98
1.96
1.93
1.91
1.89
1.87
1.71
1.69
1.68
1.63
1.62
1.60
1.36
1.33
1.31
1.28
0.90
0.89
0.88
-0.00
OH
N
N
N
O
O
S
O
9
8
7
6
5
4
3
2
1
ppm

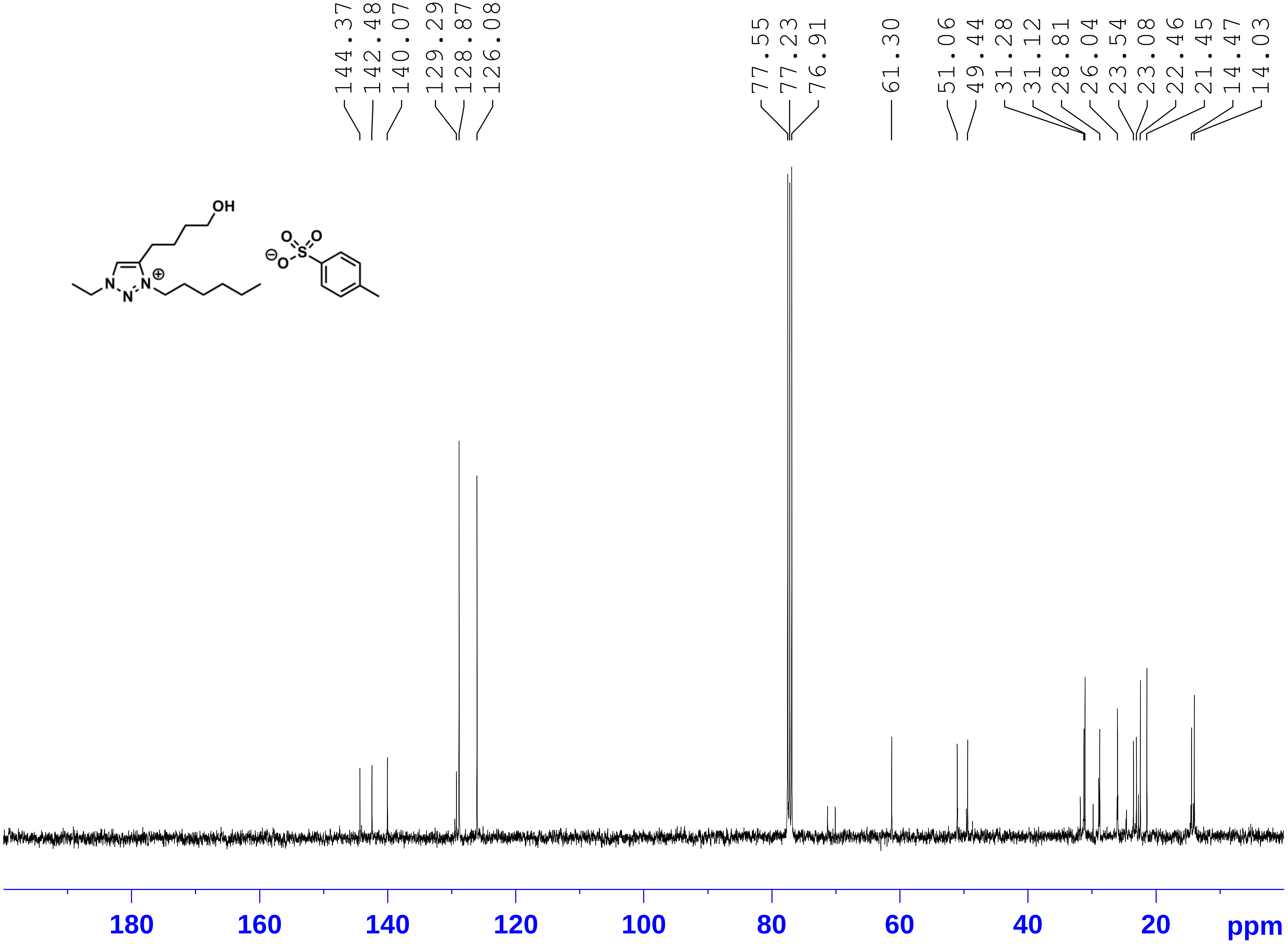

144.37
142.48
140.07
129.29
128.87
126.08
77.55
77.23
76.91
61.30
51.06
49.44
31.28
31.12
28.81
26.04
23.54
23.08
22.46
21.45
14.47
14.03
OH
O
S
O
O
N
N
N
180
160
140
120
100
80
60
40
20
ppm

Spectrum

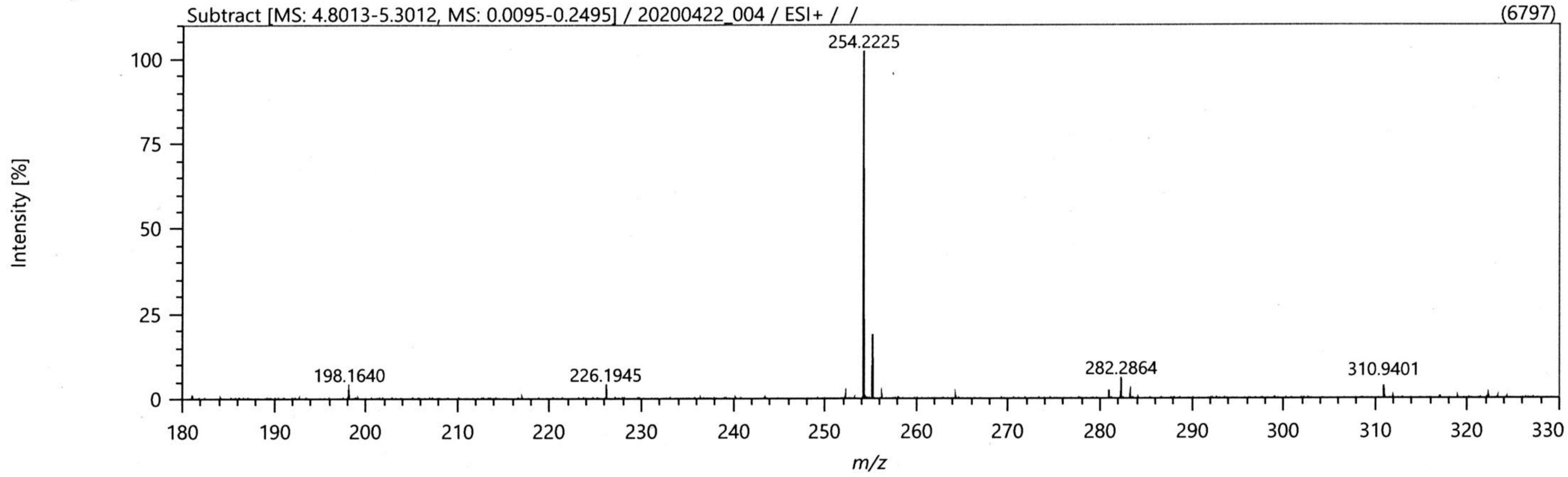

Elemental Composition

Parameters

Tolerance:	±10.00 ppm
Electron:	Odd/Even
Charge:	+1
DBE:	-99.0 - 999.0

Elements Set 1:

Symbol	C	H	O	Na	N	S
Min	0	0	1	0	3	0
Max	1000	4000	1	0	3	0

Results

Mass	Formula	Calculated Mass	Mass Difference [mDa]	Mass Difference [ppm]	DBE
254.22251	C14 H28 N3 O	254.22269	-0.18	-0.71	2.5

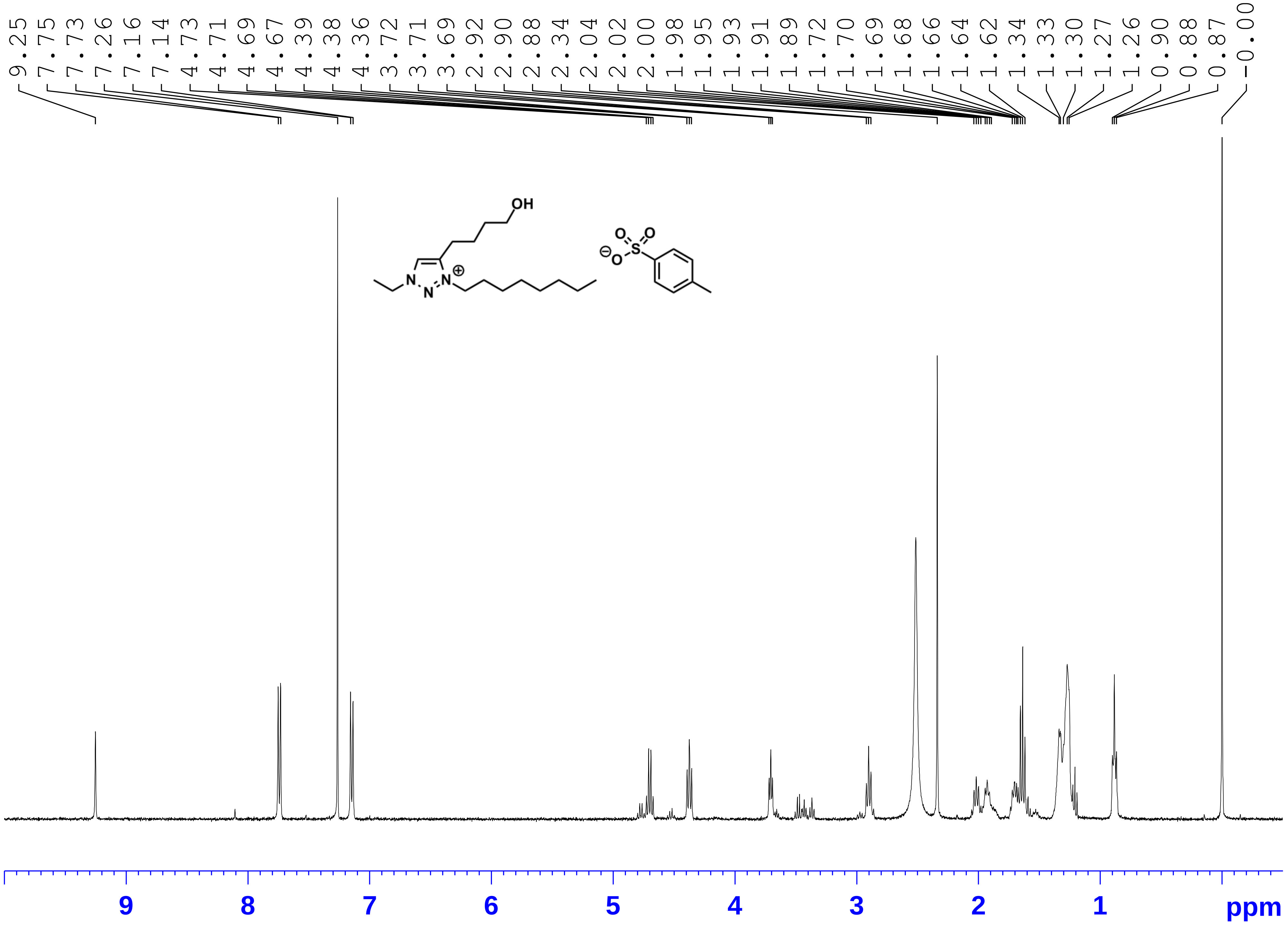
9.25
7.75
7.73
7.26
7.16
7.14
4.73
4.71
4.69
4.67
4.39
4.38
4.36
3.72
3.71
3.69
2.92
2.90
2.88
2.34
2.04
2.02
2.00
1.98
1.95
1.93
1.91
1.89
1.72
1.70
1.69
1.68
1.66
1.64
1.62
1.34
1.33
1.30
1.27
1.26
0.90
0.88
0.87
-0.00
OH
N
N
N
O
S
O
O
9
8
7
6
5
4
3
2
1
ppm

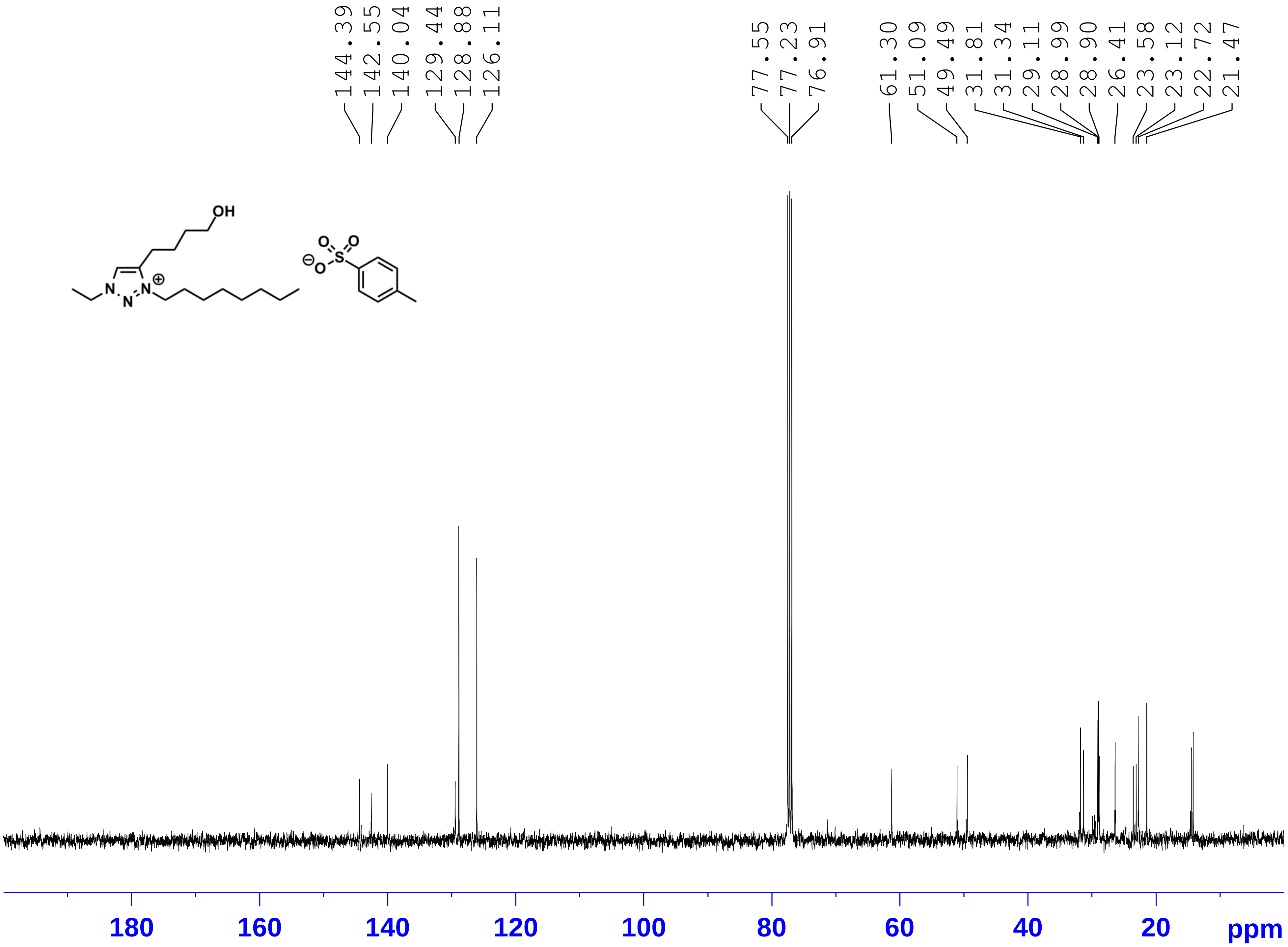
144.39
142.55
140.04
129.44
128.88
126.11
77.55
77.23
76.91
61.30
51.09
49.49
31.81
31.34
29.11
28.99
28.90
26.41
23.58
23.12
22.72
21.47
OH
N
N
N
O
O
S
O
180
160
140
120
100
80
60
40
20
ppm

Spectrum

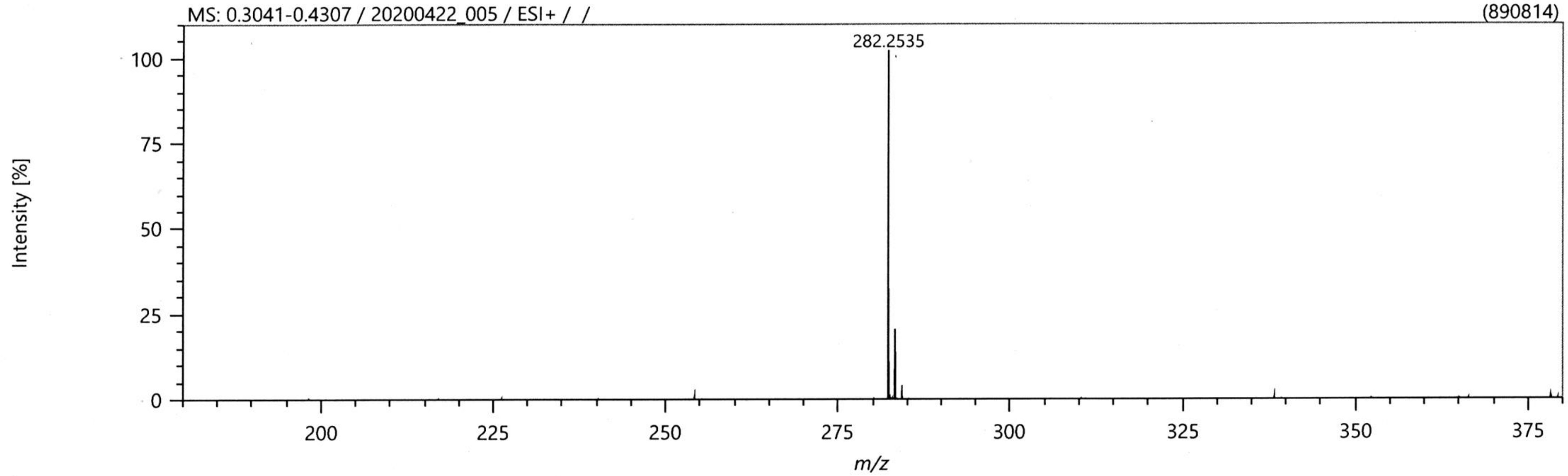

Elemental Composition

Parameters		Elements Set 1:						
Tolerance:	±10.00 ppm	Symbol	C	H	O	Na	N	F
Electron:	Odd/Even	Min	0	0	1	0	3	0
Charge:	+1	Max	1000	4000	1	0	3	0
DBE:	-99.0 - 999.0							

Results

Mass	Formula	Calculated Mass	Mass Difference [mDa]	Mass Difference [ppm]	DBE
282.25355	C16 H32 N3 O	282.25399	-0.44	-1.56	2.5

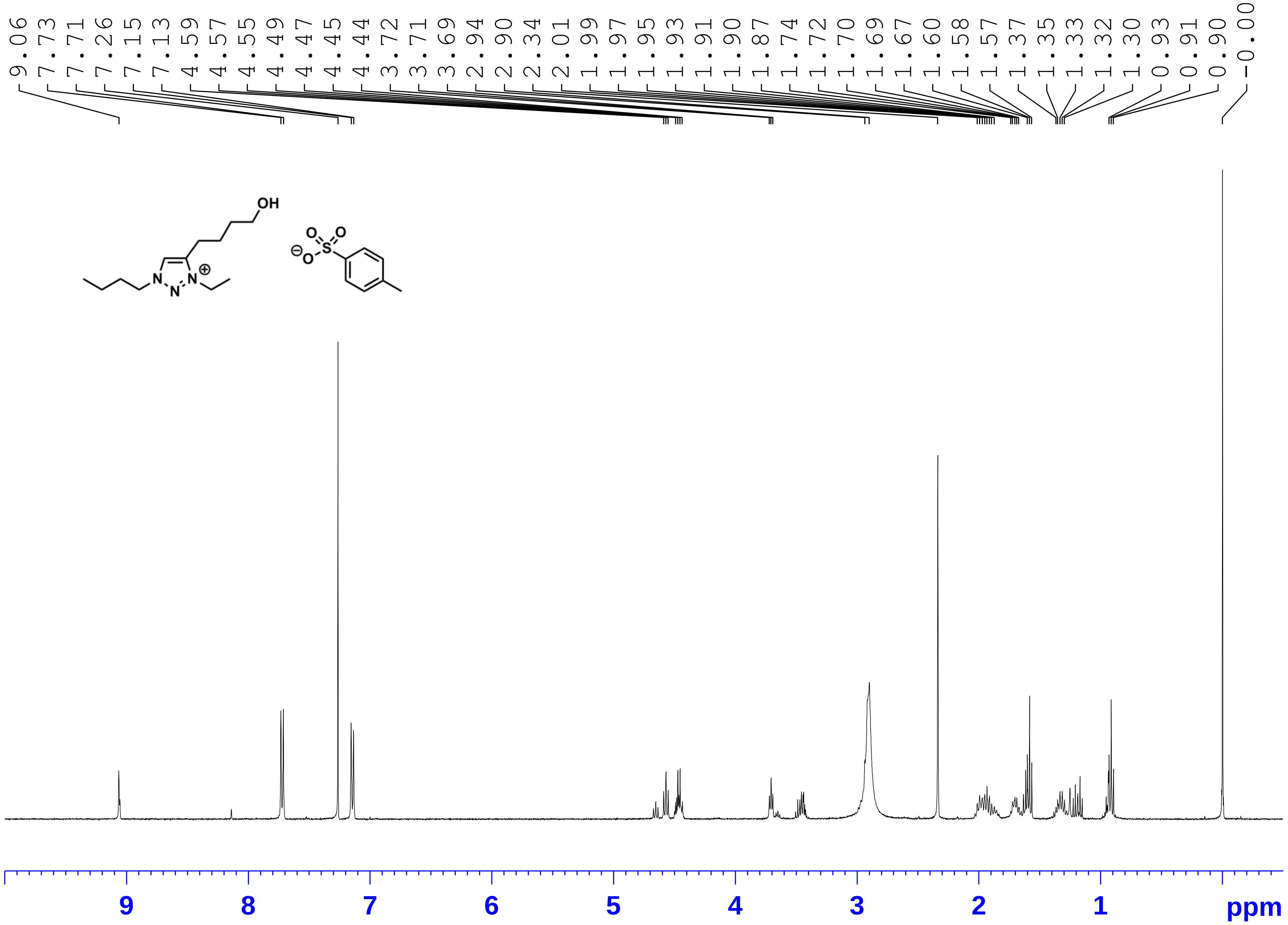

9.06
7.73
7.71
7.26
7.15
7.13
4.59
4.57
4.55
4.49
4.47
4.45
4.44
3.72
3.71
3.69
2.94
2.90
2.34
2.01
1.99
1.97
1.95
1.93
1.91
1.90
1.87
1.74
1.72
1.70
1.69
1.67
1.60
1.58
1.57
1.37
1.35
1.33
1.32
1.30
0.93
0.91
0.90
-0.00
OH
N
N
N
O
S
O
O
9
8
7
6
5
4
3
2
1
ppm

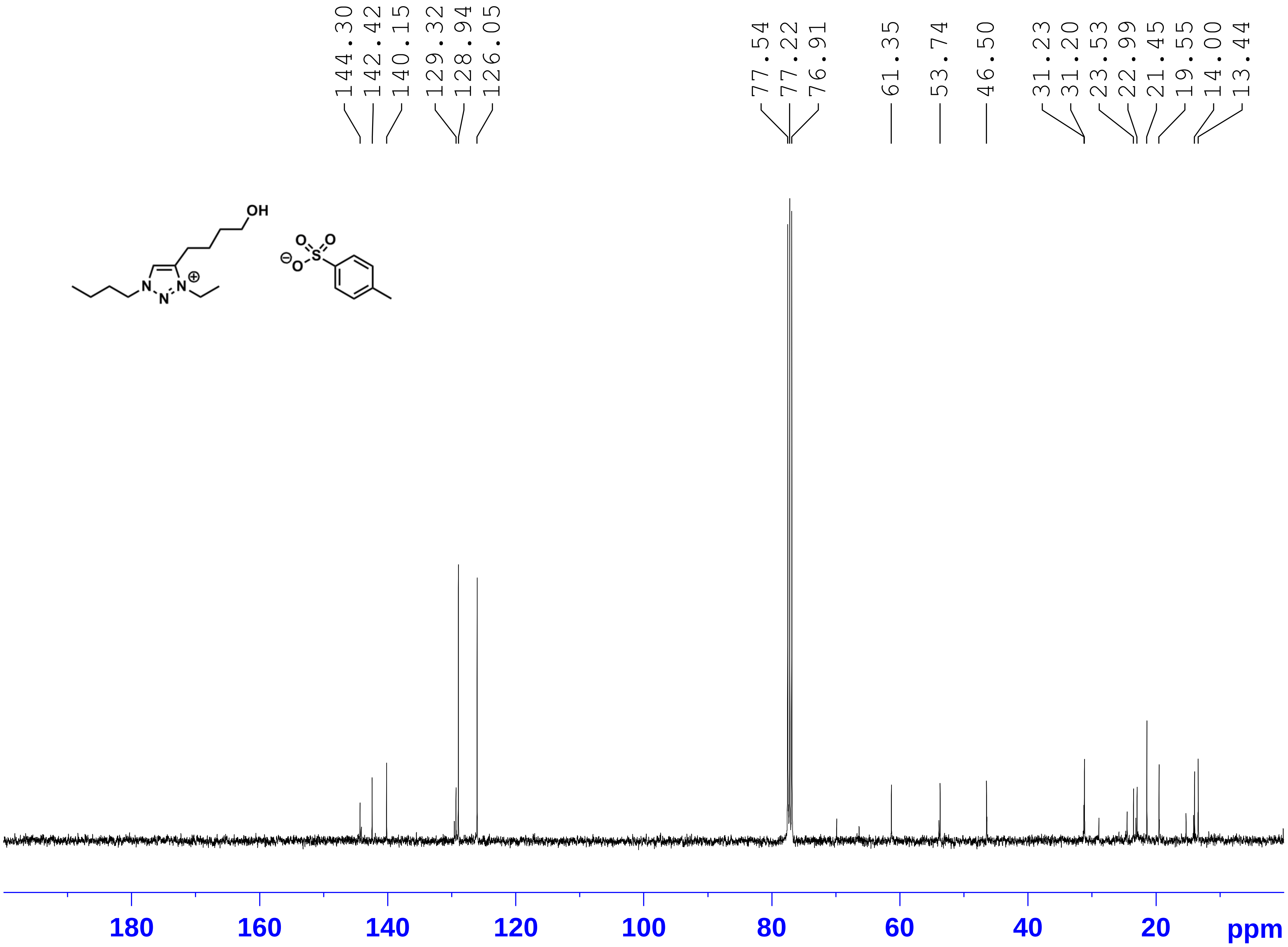
144.30
142.42
140.15
129.32
128.94
126.05
77.54
77.22
76.91
61.35
53.74
46.50
31.23
31.20
23.53
22.99
21.45
19.55
14.00
13.44
OH
180
160
140
120
100
80
60
40
20
ppm

Spectrum

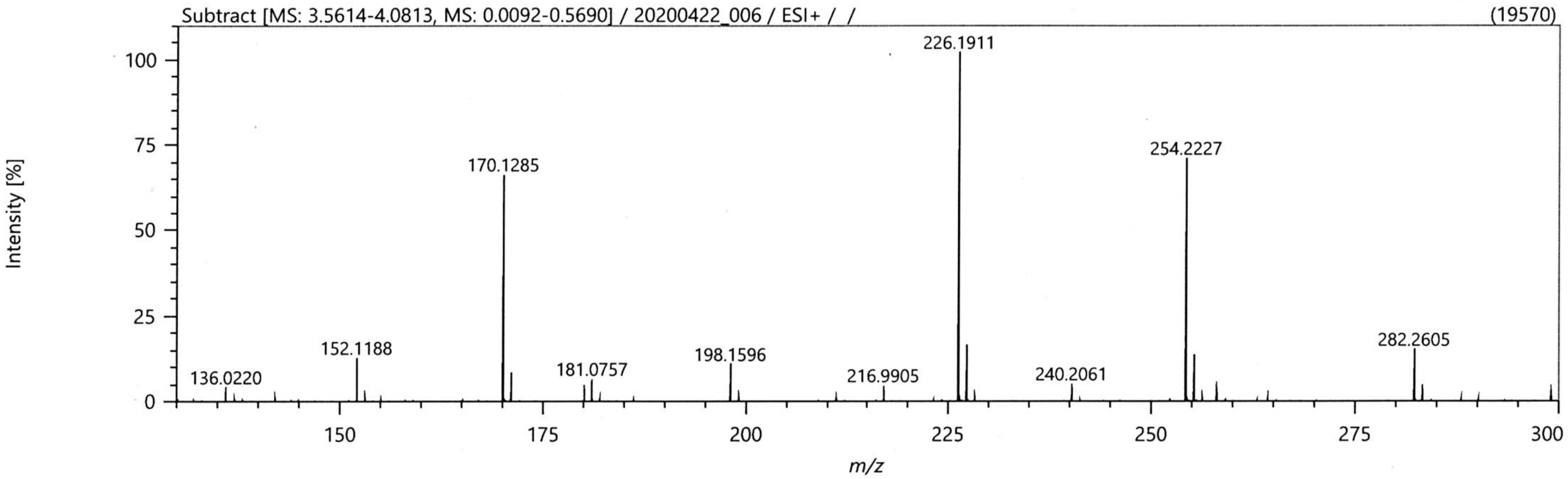

Elemental Composition

Parameters		Elements Set 1:						
Tolerance:	±10.00 ppm	Symbol	C	H	O	Na	N	F
Electron:	Odd/Even	Min	0	0	1	0	3	0
Charge:	+1	Max	1000	4000	1	0	3	0
DBE:	-99.0 - 999.0							

Results

Mass	Formula	Calculated Mass	Mass Difference [mDa]	Mass Difference [ppm]	DBE
226.19113	C12 H24 N3 O	226.19139	-0.26	-1.15	2.5

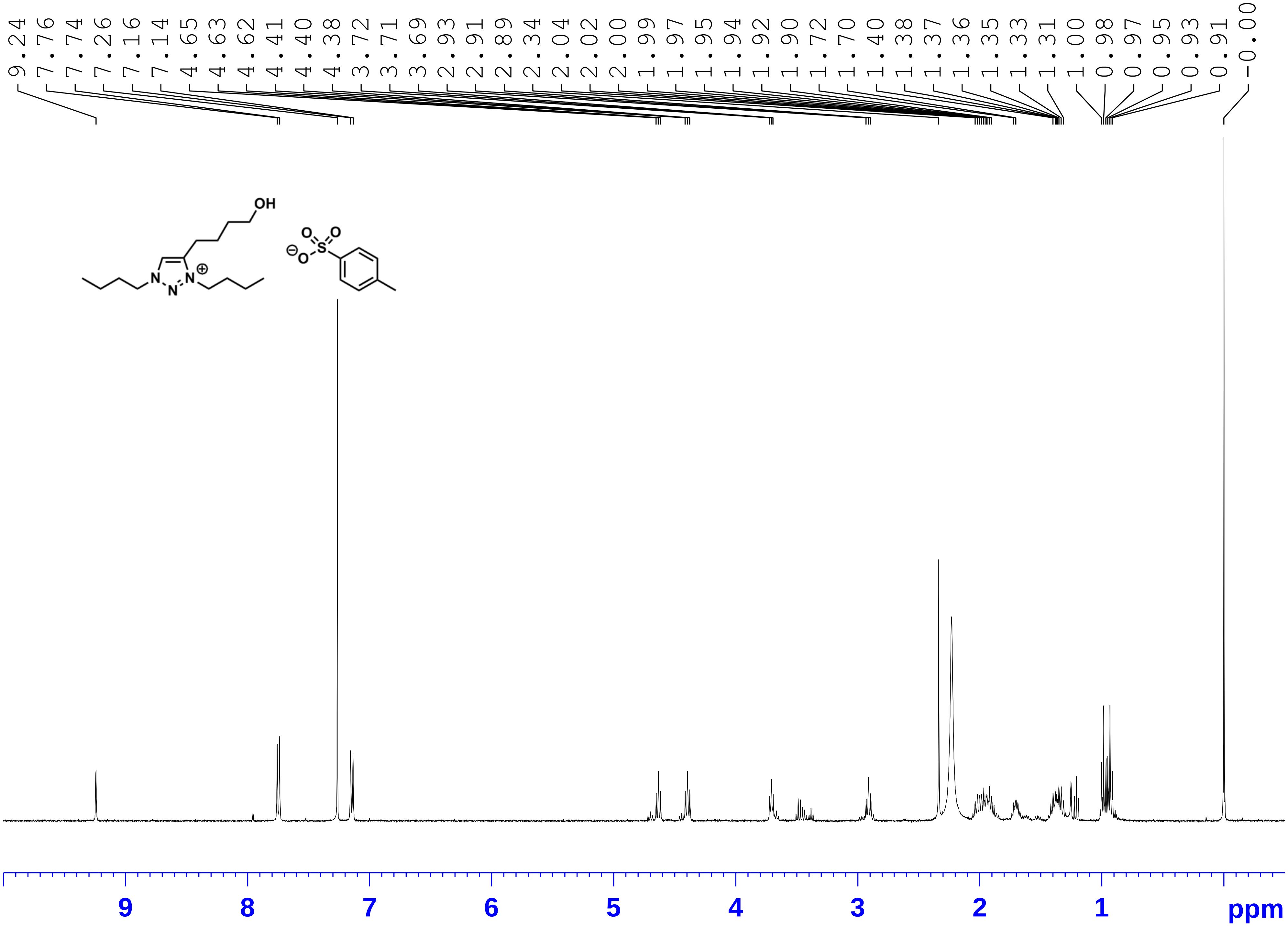
9.24
7.76
7.74
7.26
7.16
7.14
4.65
4.63
4.62
4.41
4.40
4.38
3.72
3.71
3.69
2.93
2.91
2.89
2.34
2.04
2.02
2.00
1.99
1.97
1.95
1.94
1.92
1.90
1.72
1.70
1.40
1.38
1.37
1.36
1.35
1.33
1.31
1.00
0.98
0.97
0.95
0.93
0.91
-0.00
OH
9
8
7
6
5
4
3
2
1
ppm

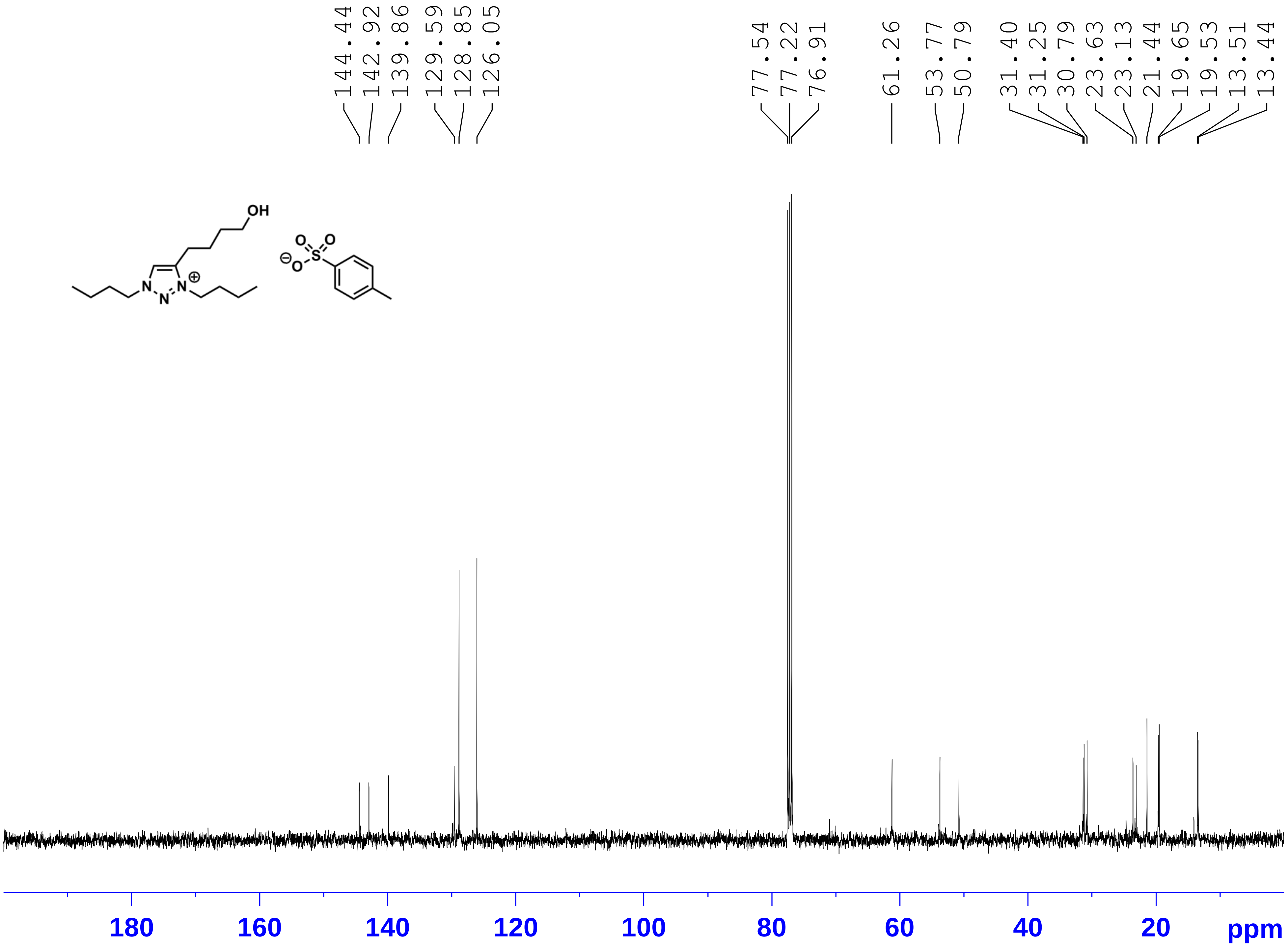

144.44
142.92
139.86
129.59
128.85
126.05
77.54
77.22
76.91
61.26
53.77
50.79
31.40
31.25
30.79
23.63
23.13
21.44
19.65
19.53
13.51
13.44
OH
180
160
140
120
100
80
60
40
20
ppm

Spectrum

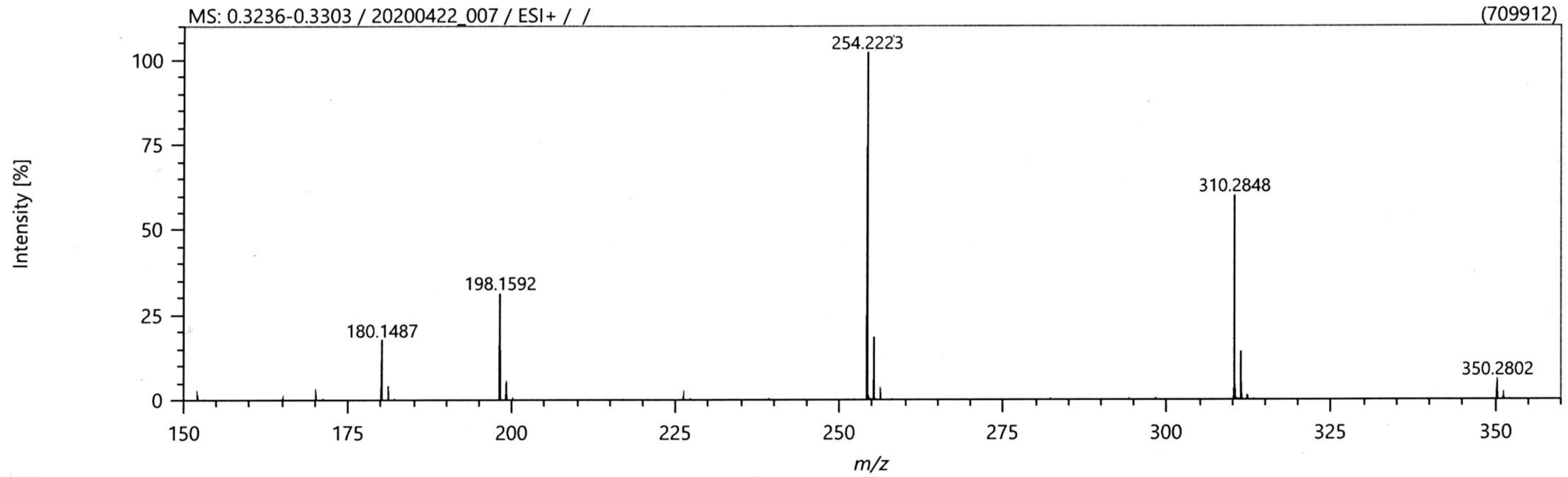

Elemental Composition

Parameters		Elements Set 1:						
Tolerance:	±10.00 ppm	Symbol	C	H	O	Na	N	F
Electron:	Odd/Even	Min	0	0	1	0	3	0
Charge:	+1	Max	1000	4000	1	0	3	0
DBE:	-99.0 - 999.0							

Results

Mass	Formula	Calculated Mass	Mass Difference [mDa]	Mass Difference [ppm]	DBE
254.22229	C14 H28 N3 O	254.22269	-0.40	-1.57	2.5

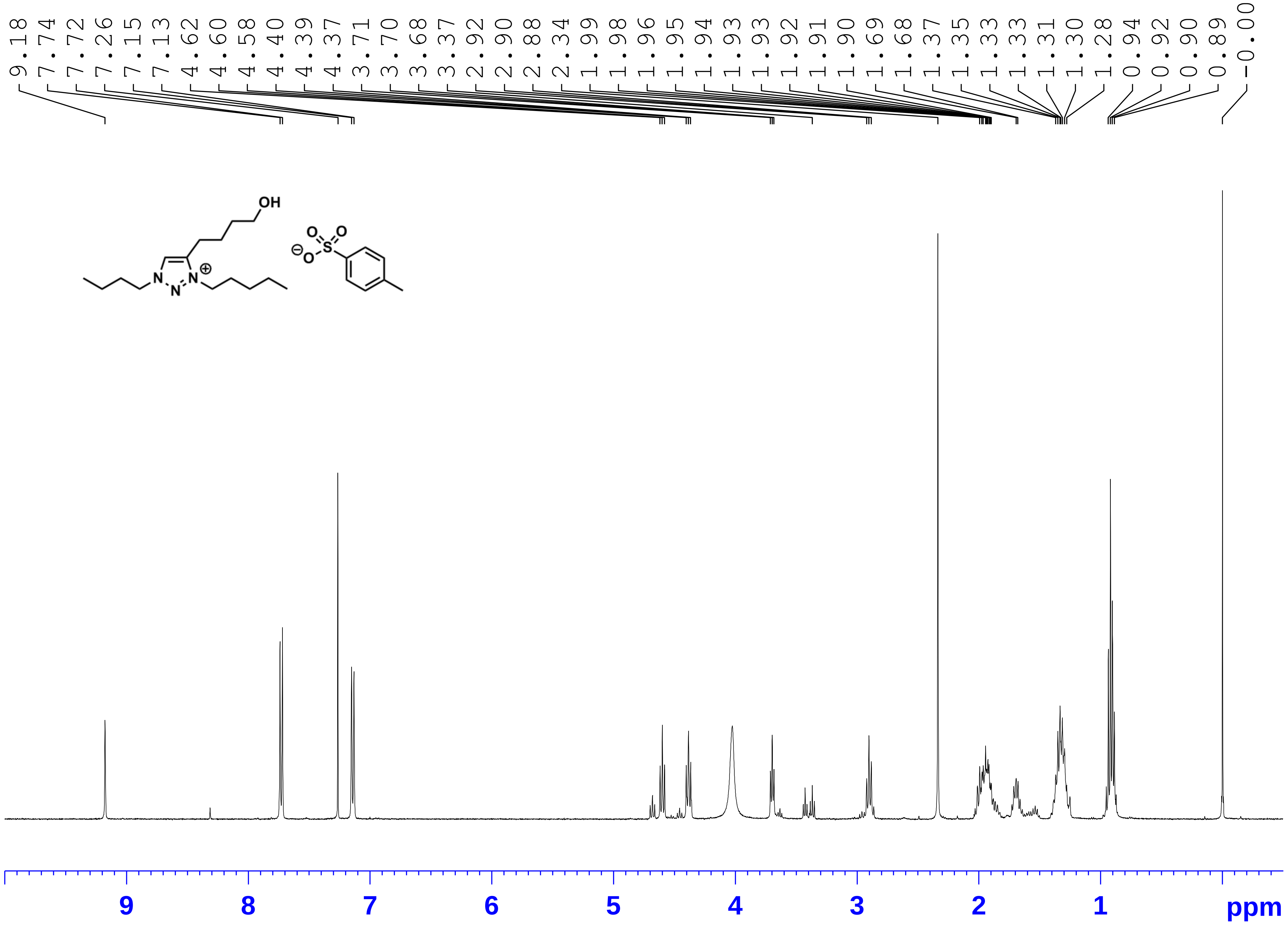

9.18
7.74
7.72
7.26
7.15
7.13
4.62
4.60
4.58
4.40
4.39
4.37
3.71
3.70
3.68
3.37
2.92
2.90
2.88
2.34
1.99
1.98
1.96
1.95
1.94
1.93
1.93
1.92
1.91
1.90
1.69
1.68
1.37
1.35
1.33
1.33
1.31
1.30
1.28
0.94
0.92
0.90
0.89
-0.00
OH
O
S
O
N
N
N
9
8
7
6
5
4
3
2
1
ppm

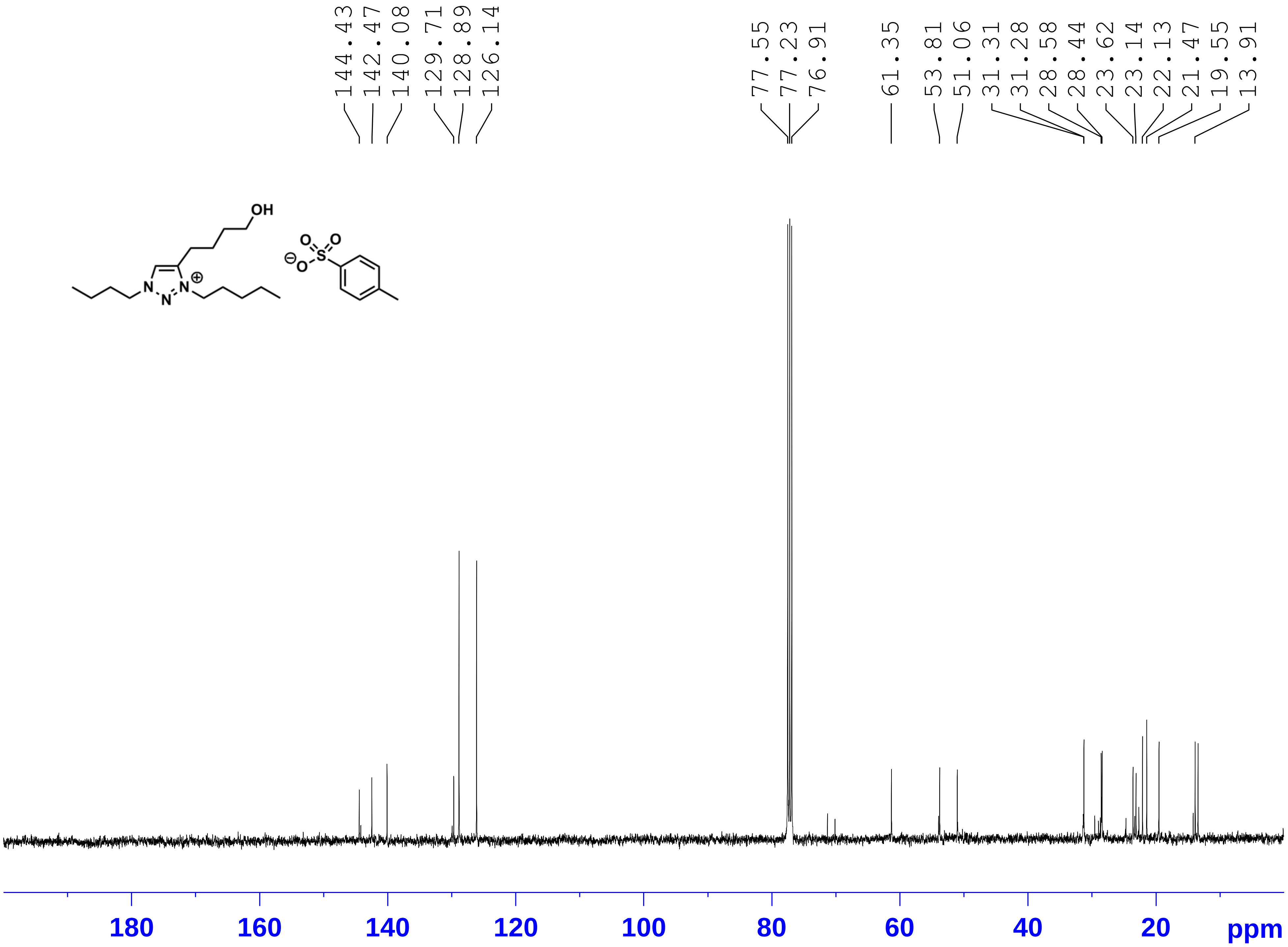

144.43
142.47
140.08
129.71
128.89
126.14
77.55
77.23
76.91
61.35
53.81
51.06
31.31
31.28
28.58
28.44
23.62
23.14
22.13
21.47
19.55
13.91
OH
N
N
N
O
O
S
O
180
160
140
120
100
80
60
40
20
ppm

Spectrum

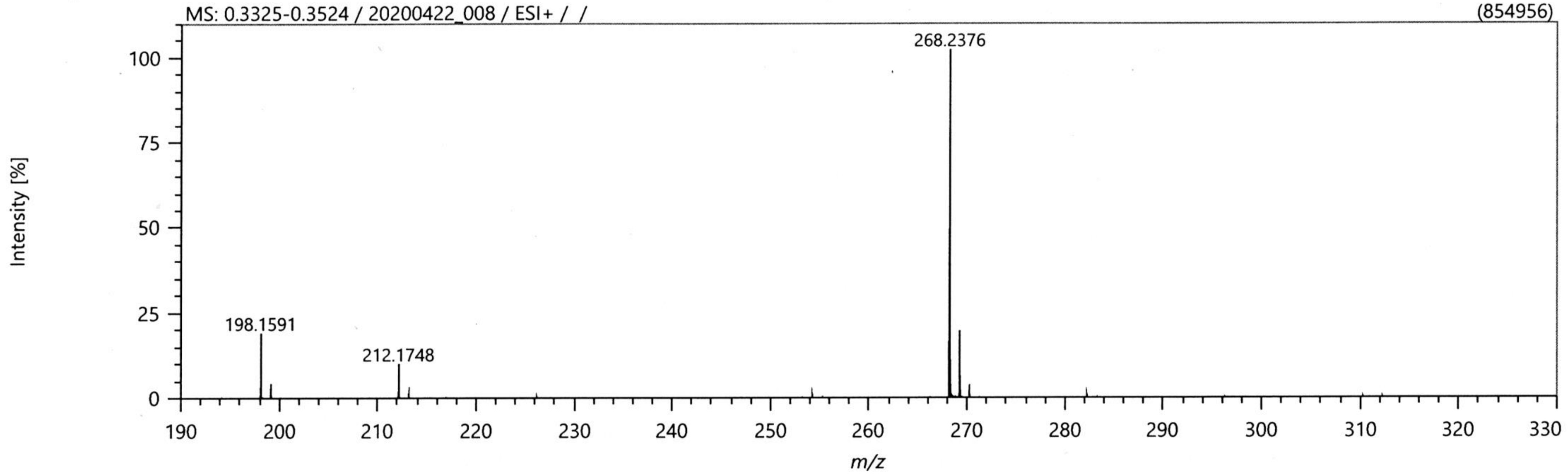

Elemental Composition

Parameters

Tolerance:	±10.00 ppm
Electron:	Odd/Even
Charge:	+1
DBE:	-99.0 - 999.0

Elements Set 1:

Symbol	C	H	O	Na	N	F
Min	0	0	1	0	3	0
Max	1000	4000	1	0	3	0

Results

Mass	Formula	Calculated Mass	Mass Difference [mDa]	Mass Difference [ppm]	DBE
268.23759	C15 H30 N3 O	268.23834	-0.75	-2.78	2.5

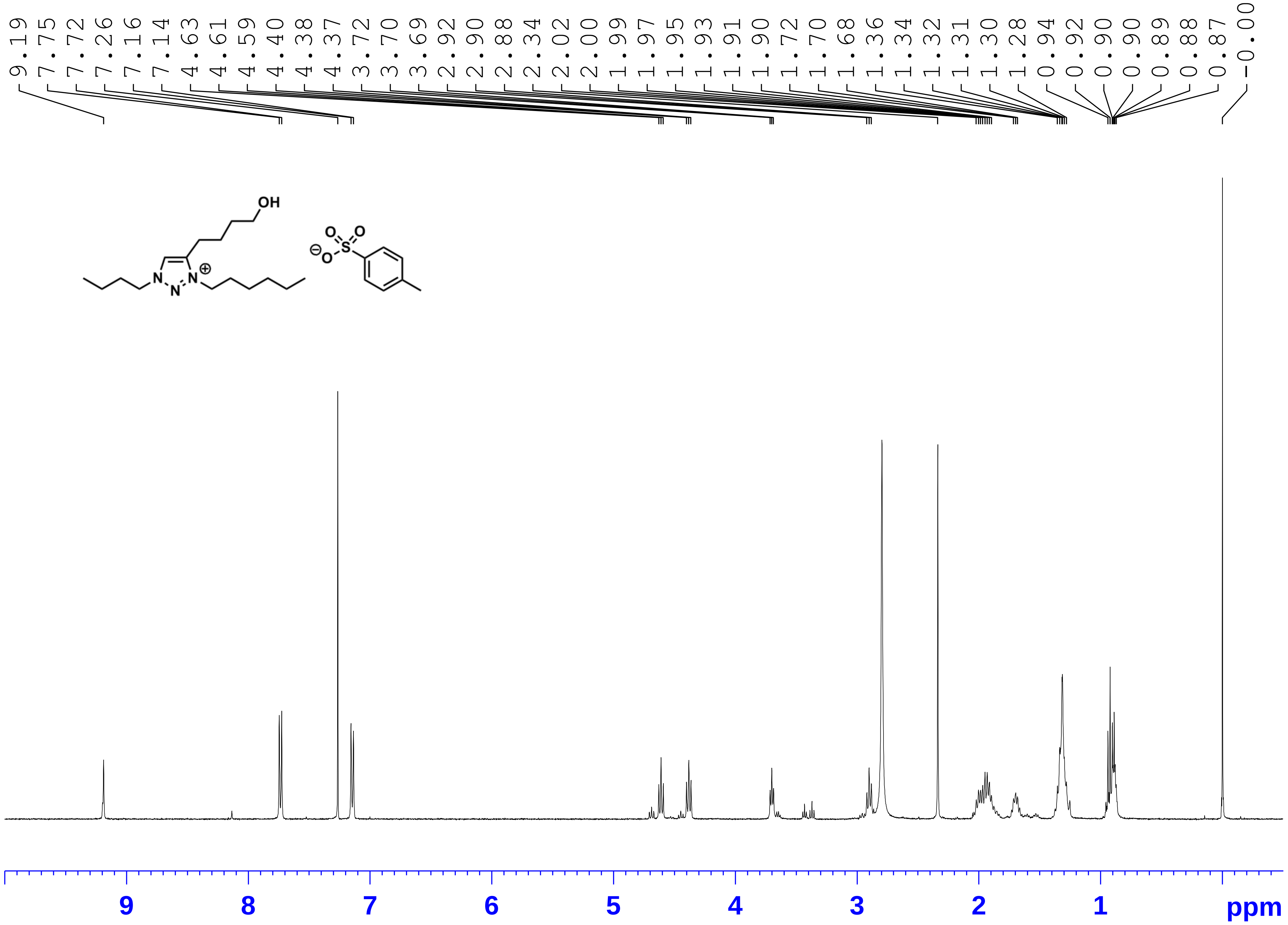

9.19
7.75
7.72
7.26
7.16
7.14
4.63
4.61
4.59
4.40
4.38
4.37
3.72
3.70
3.69
2.92
2.90
2.88
2.34
2.02
2.00
1.99
1.97
1.95
1.93
1.91
1.90
1.72
1.70
1.68
1.36
1.34
1.32
1.31
1.30
1.28
0.94
0.92
0.90
0.90
0.89
0.88
0.87
-0.00
OH
O
S
O
N
N
N
9
8
7
6
5
4
3
2
1
ppm

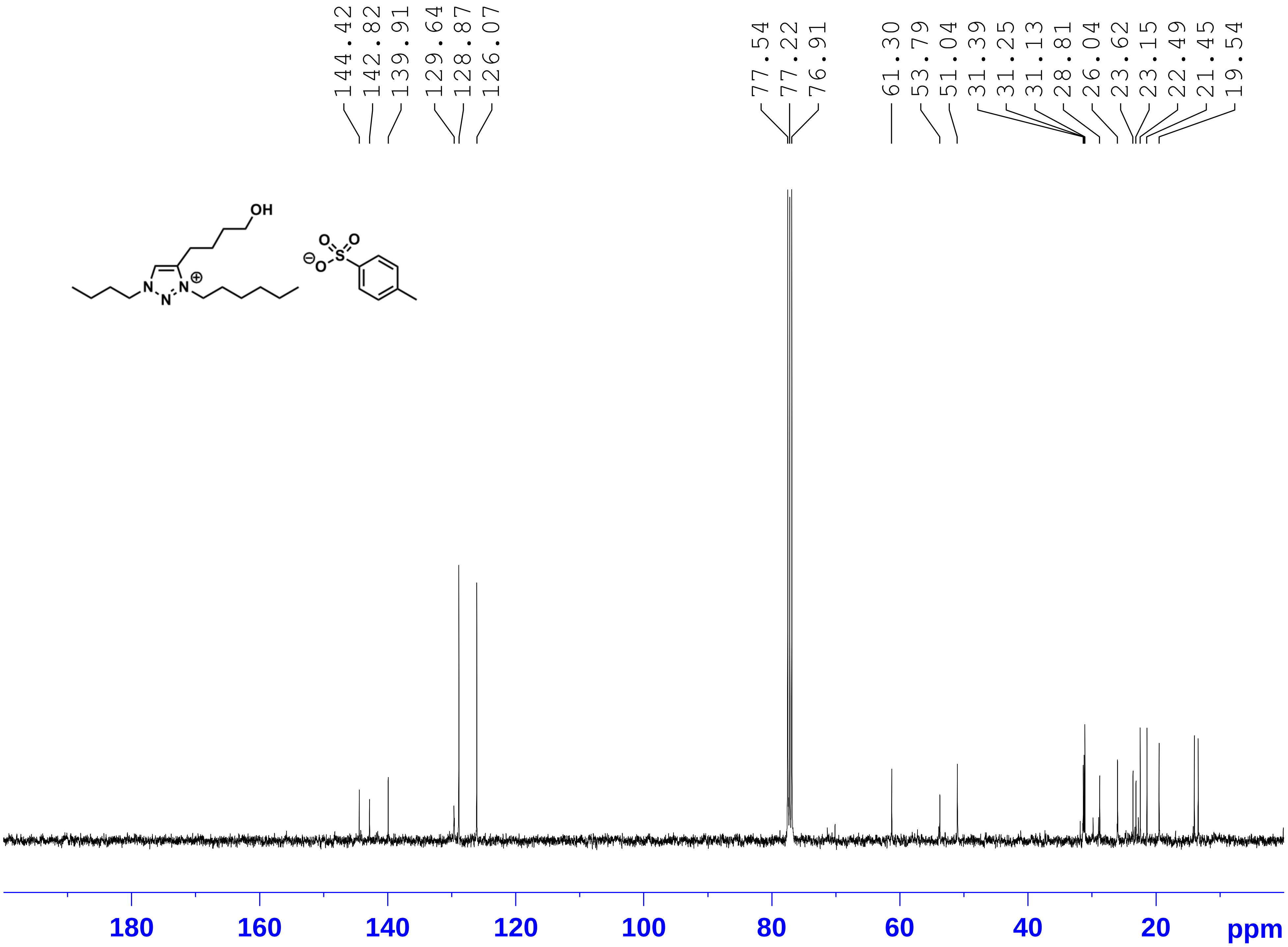
144.42
142.82
139.91
129.64
128.87
126.07
77.54
77.22
76.91
61.30
53.79
51.04
31.39
31.25
31.13
28.81
26.04
23.62
23.15
22.49
21.45
19.54
OH
N
N
N
O
O
O
S
180
160
140
120
100
80
60
40
20
ppm

Spectrum

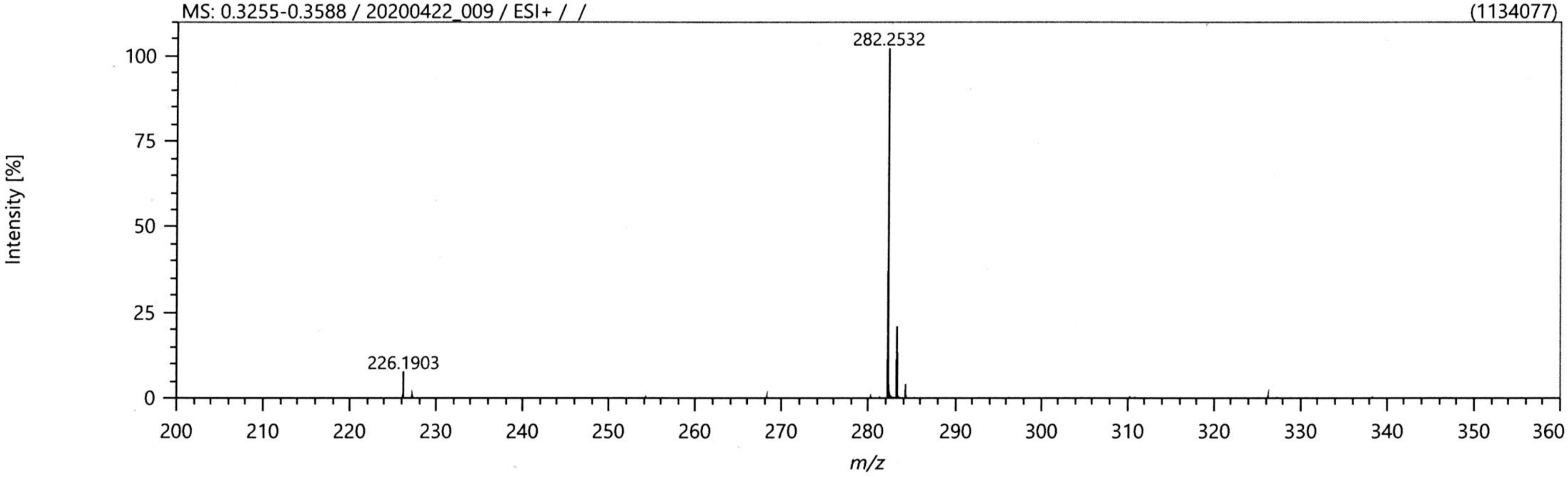

Elemental Composition

Parameters		Elements Set 1:						
Tolerance:	±10.00 ppm	Symbol	C	H	O	Na	N	F
Electron:	Odd/Even	Min	0	0	1	0	3	0
Charge:	+1	Max	1000	4000	1	0	3	0
DBE:	-99.0 - 999.0							

Results

Mass	Formula	Calculated Mass	Mass Difference [mDa]	Mass Difference [ppm]	DBE
282.25318	C16 H32 N3 O	282.25399	-0.81	-2.88	2.5

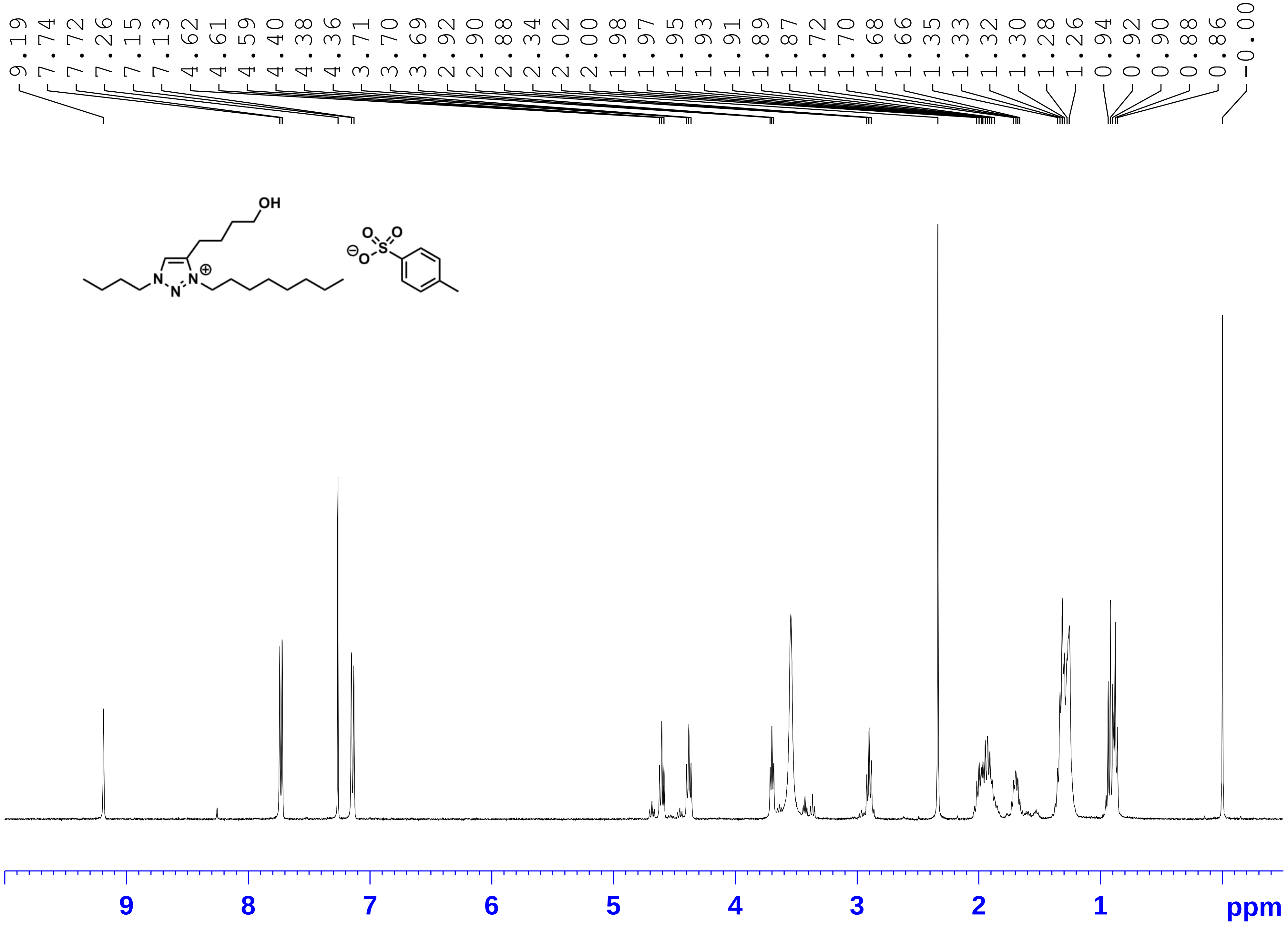
9.19
7.74
7.72
7.26
7.15
7.13
4.62
4.61
4.59
4.40
4.38
4.36
3.71
3.70
3.69
2.92
2.90
2.88
2.34
2.02
2.00
1.98
1.97
1.95
1.93
1.91
1.89
1.87
1.72
1.70
1.68
1.66
1.35
1.33
1.32
1.30
1.28
1.26
0.94
0.92
0.90
0.88
0.86
-0.00
OH
N
N
N
O
S
O
O
9
8
7
6
5
4
3
2
1
ppm

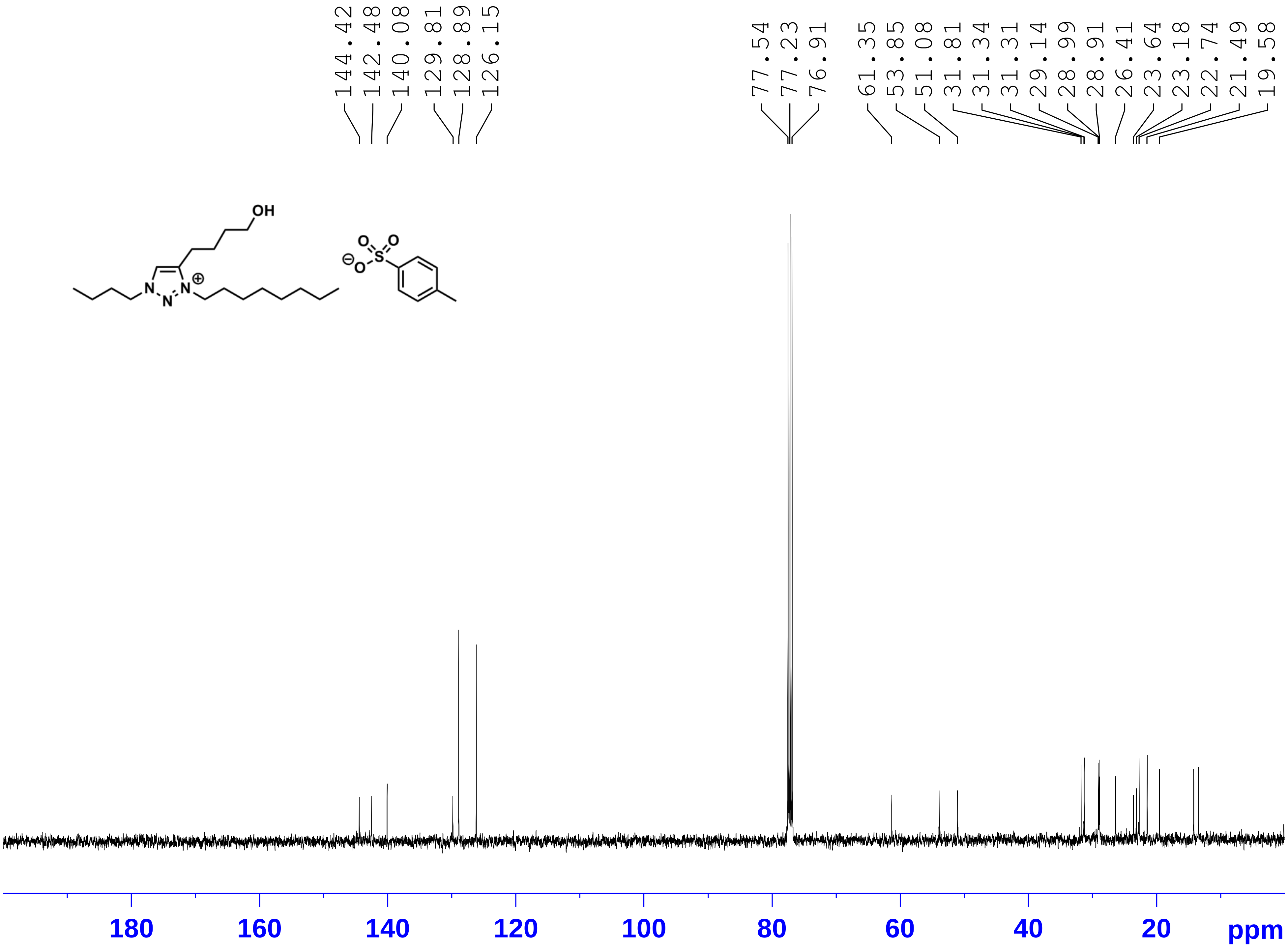

144.42
142.48
140.08
129.81
128.89
126.15
77.54
77.23
76.91
61.35
53.85
51.08
31.81
31.34
31.31
29.14
28.99
28.91
26.41
23.64
23.18
22.74
21.49
19.58
OH
O
S
O
O
N
N
N
180
160
140
120
100
80
60
40
20
ppm

Spectrum

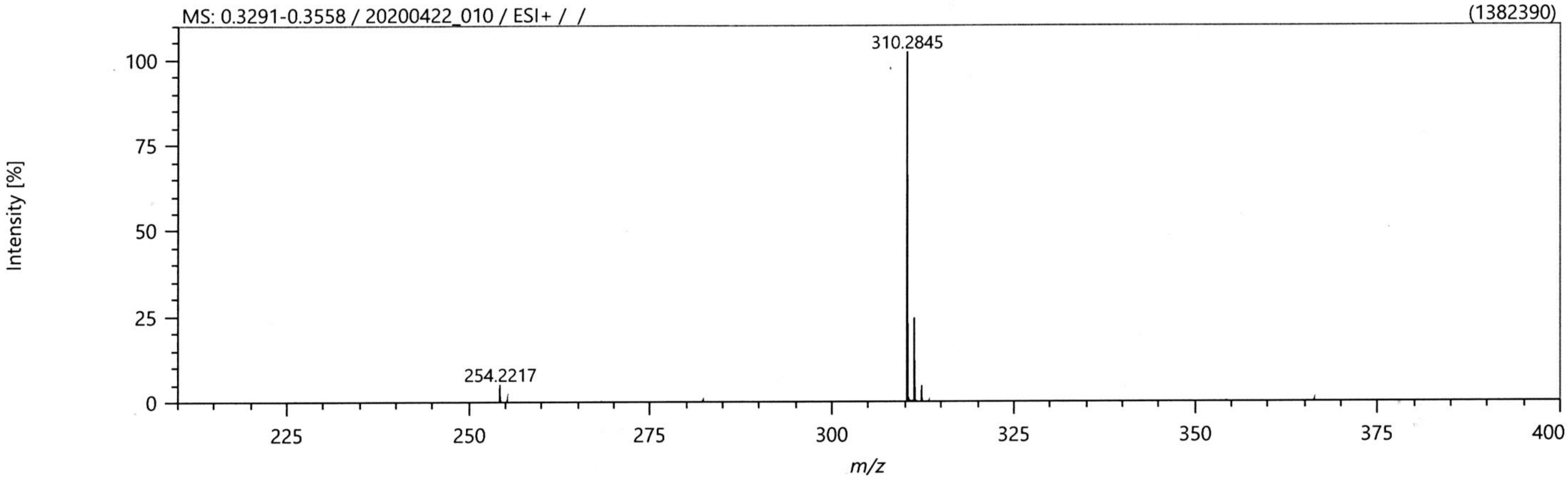

Elemental Composition

Parameters		Elements Set 1:						
Tolerance:	±10.00 ppm	Symbol	C	H	O	Na	N	F
Electron:	Odd/Even	Min	0	0	1	0	3	0
Charge:	+1	Max	1000	4000	1	0	3	0
DBE:	-99.0 - 999.0							

Results

Mass	Formula	Calculated Mass	Mass Difference [mDa]	Mass Difference [ppm]	DBE
310.28452	C18 H36 N3 O	310.28529	-0.77	-2.47	2.5

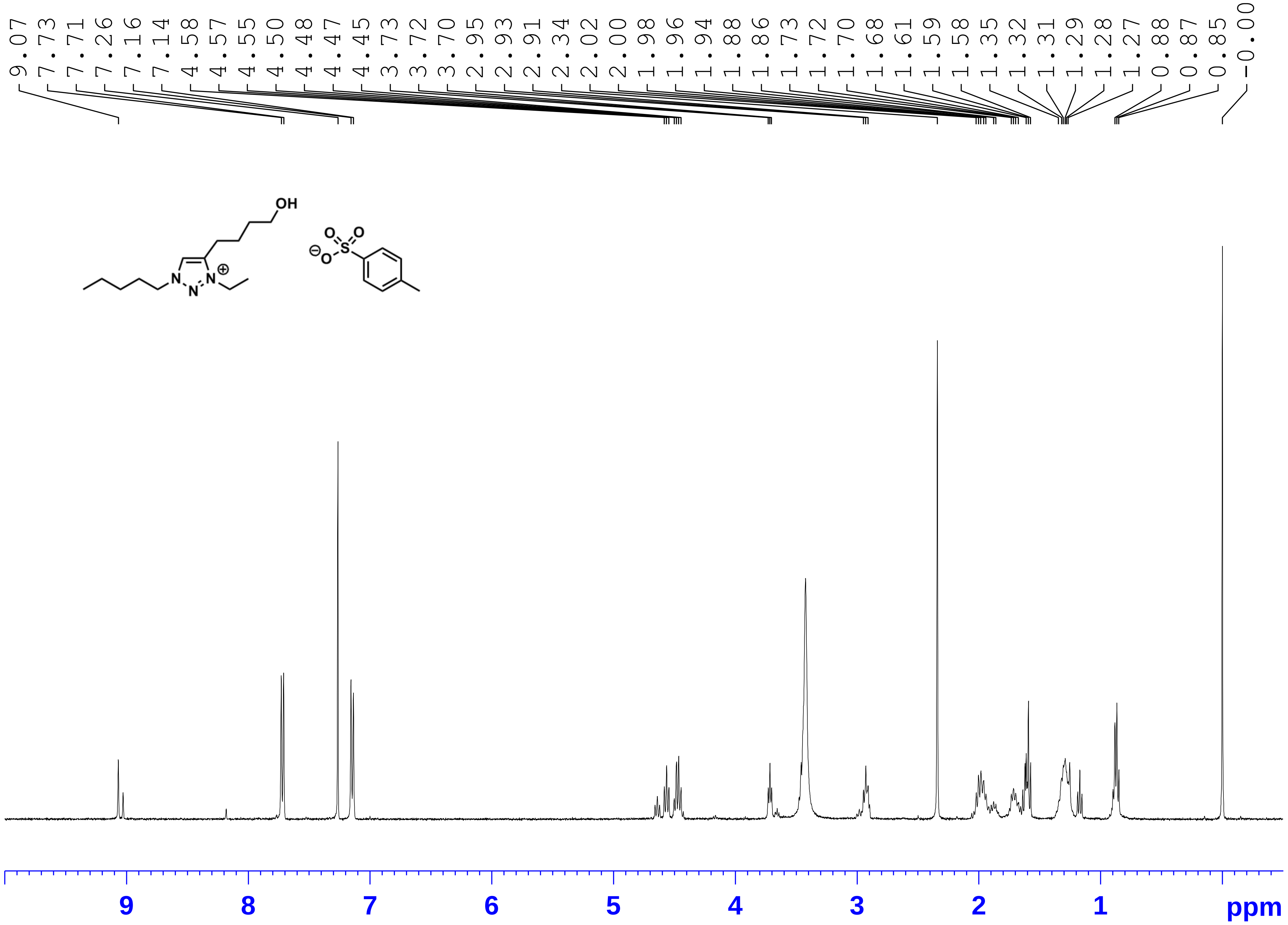

9.07
7.73
7.71
7.26
7.16
7.14
4.58
4.57
4.55
4.50
4.48
4.47
4.45
3.73
3.72
3.70
2.95
2.93
2.91
2.34
2.02
2.00
1.98
1.96
1.94
1.88
1.86
1.73
1.72
1.70
1.68
1.61
1.59
1.58
1.35
1.32
1.31
1.29
1.28
1.27
0.88
0.87
0.85
-0.00
OH
N
N
N
O
S
O
O
9
8
7
6
5
4
3
2
1
ppm

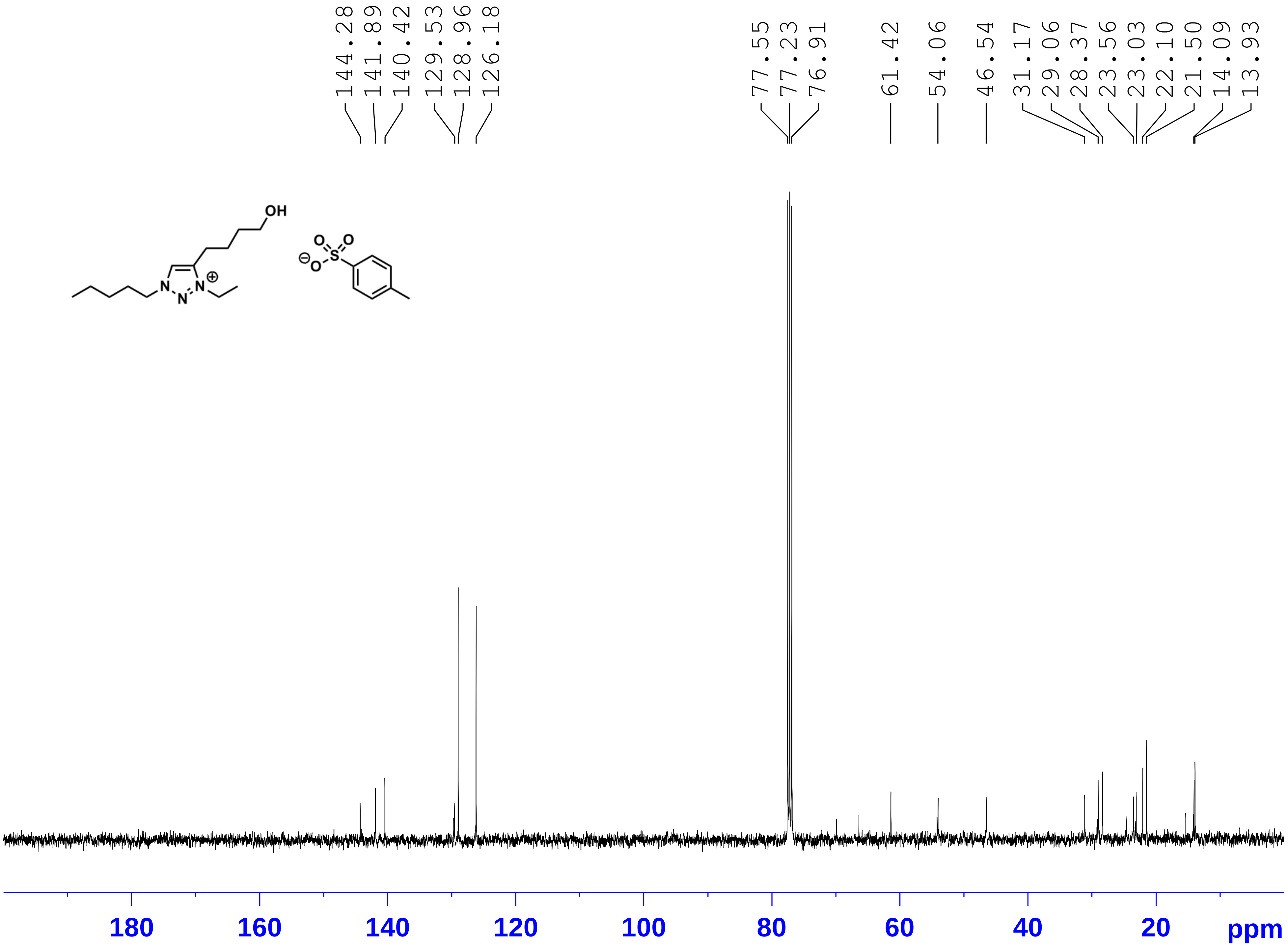

144.28
141.89
140.42
129.53
128.96
126.18
77.55
77.23
76.91
61.42
54.06
46.54
31.17
29.06
28.37
23.56
23.03
22.10
21.50
14.09
13.93
OH
N
N
N
⊕
O
O
S
⊖O
180
160
140
120
100
80
60
40
20
ppm

Spectrum

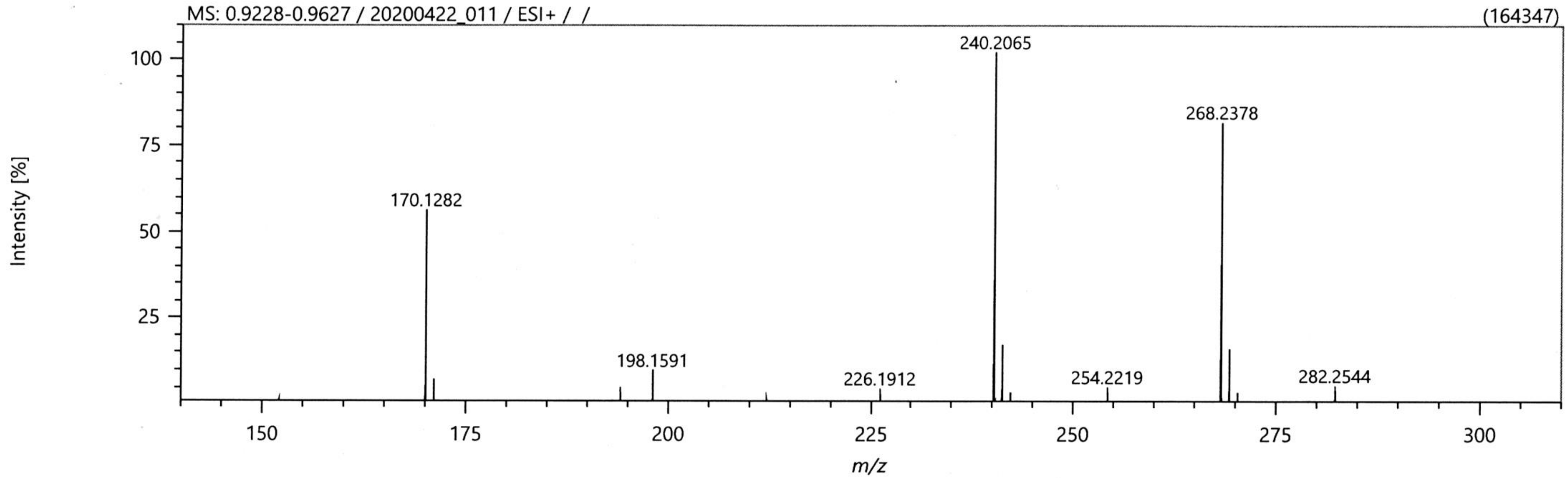

Elemental Composition

Parameters		Elements Set 1:						
Tolerance:	±10.00 ppm	Symbol	C	H	O	Na	N	F
Electron:	Odd/Even	Min	0	0	1	0	3	0
Charge:	+1	Max	1000	4000	1	0	3	0
DBE:	-99.0 - 999.0							

Results

Mass	Formula	Calculated Mass	Mass Difference [mDa]	Mass Difference [ppm]	DBE
240.20649	C13 H26 N3 O	240.20704	-0.55	-2.29	2.5

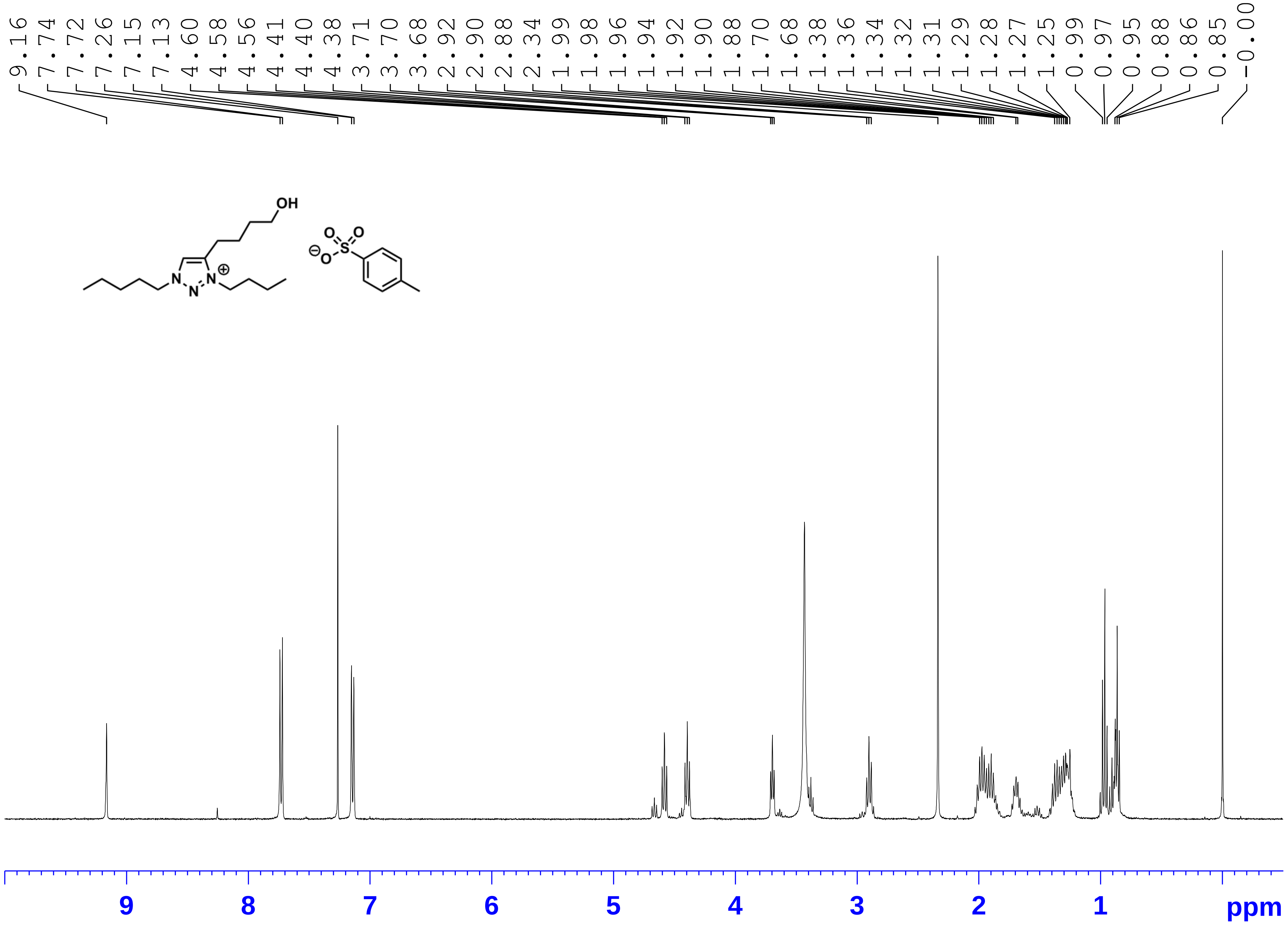
9.16
7.74
7.72
7.26
7.15
7.13
4.60
4.58
4.56
4.41
4.40
4.38
3.71
3.70
3.68
2.92
2.90
2.88
2.34
1.99
1.98
1.96
1.94
1.92
1.90
1.88
1.70
1.68
1.38
1.36
1.34
1.32
1.31
1.29
1.28
1.27
1.25
0.99
0.97
0.95
0.88
0.86
0.85
-0.00
OH
N
N
N
O
O
O
S
9
8
7
6
5
4
3
2
1
ppm

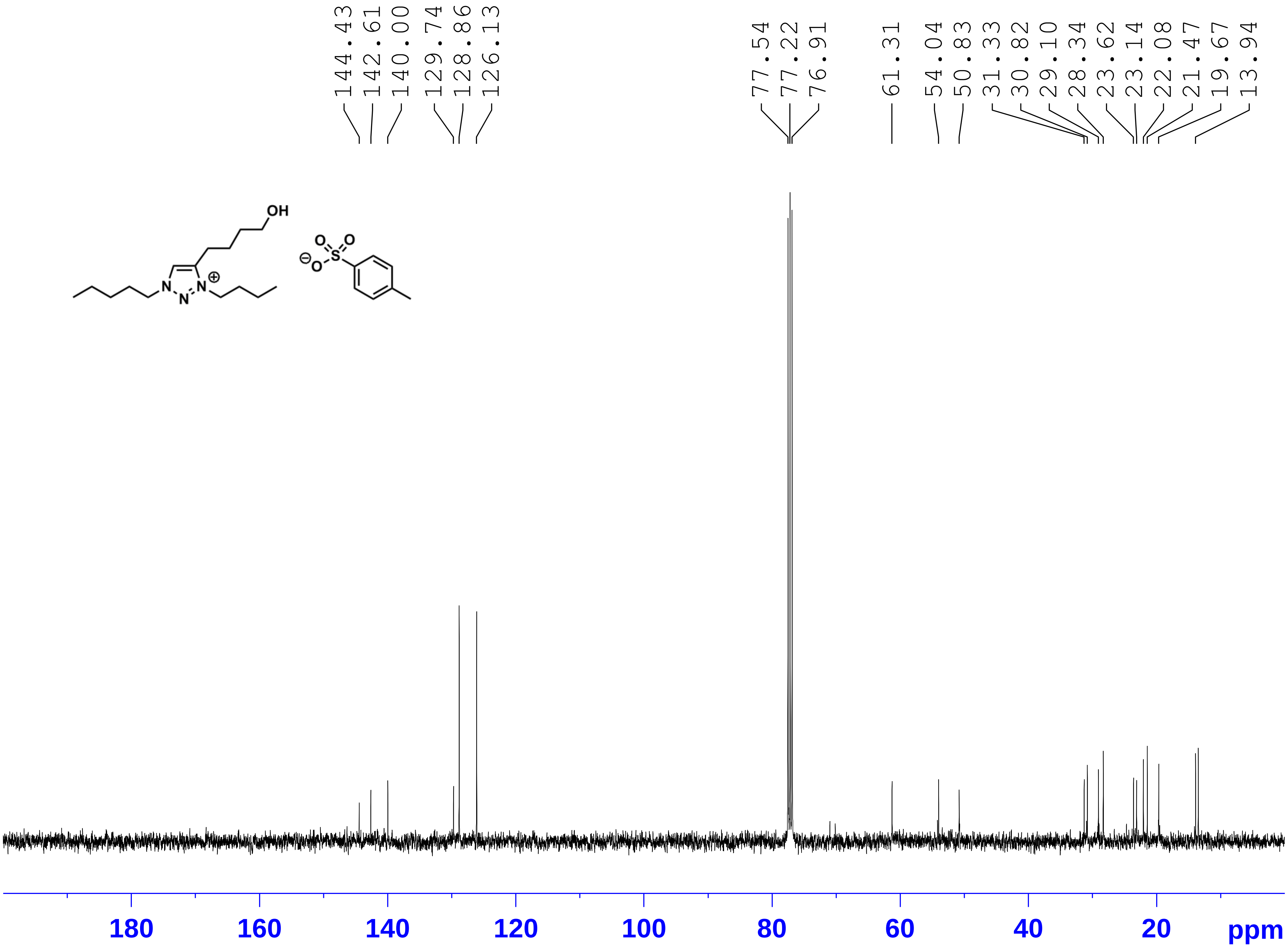
144.43
142.61
140.00
129.74
128.86
126.13
77.54
77.22
76.91
61.31
54.04
50.83
31.33
30.82
29.10
28.34
23.62
23.14
22.08
21.47
19.67
13.94
OH
N
N
N
O
O
S
O
180
160
140
120
100
80
60
40
20
ppm

Spectrum

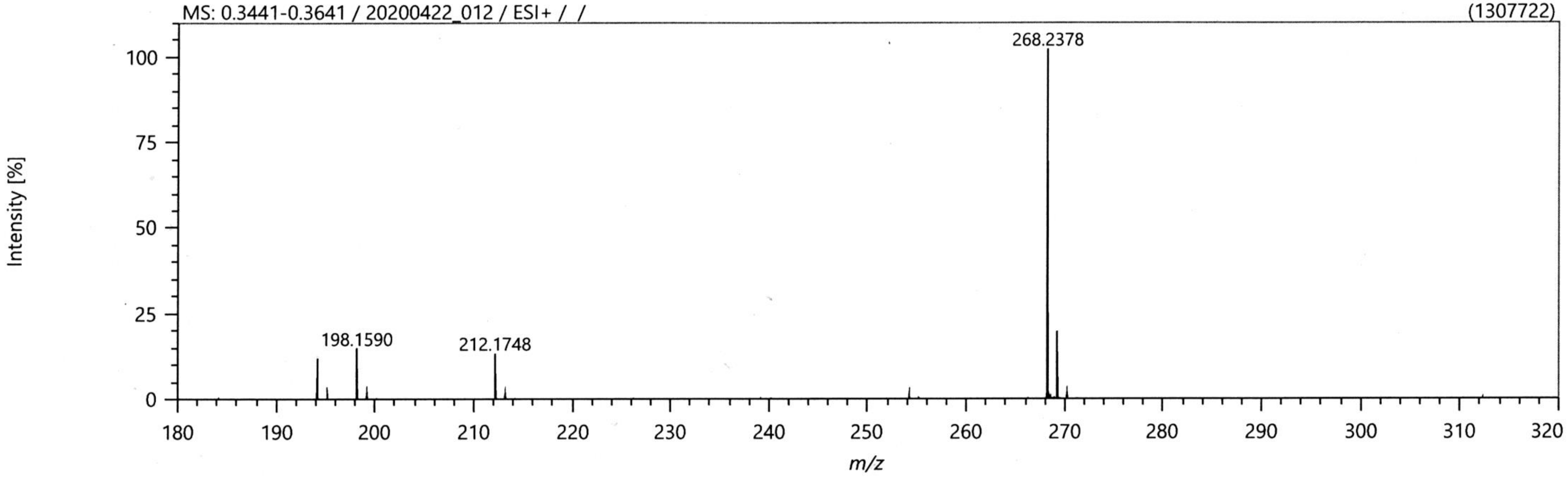

Elemental Composition

Parameters

Tolerance:	±10.00 ppm
Electron:	Odd/Even
Charge:	+1
DBE:	-99.0 - 999.0

Elements Set 1:

Symbol	C	H	O	Na	N	F
Min	0	0	1	0	3	0
Max	1000	4000	1	0	3	0

Results

Mass	Formula	Calculated Mass	Mass Difference [mDa]	Mass Difference [ppm]	DBE
268.23775	C15 H30 N3 O	268.23834	-0.59	-2.18	2.5

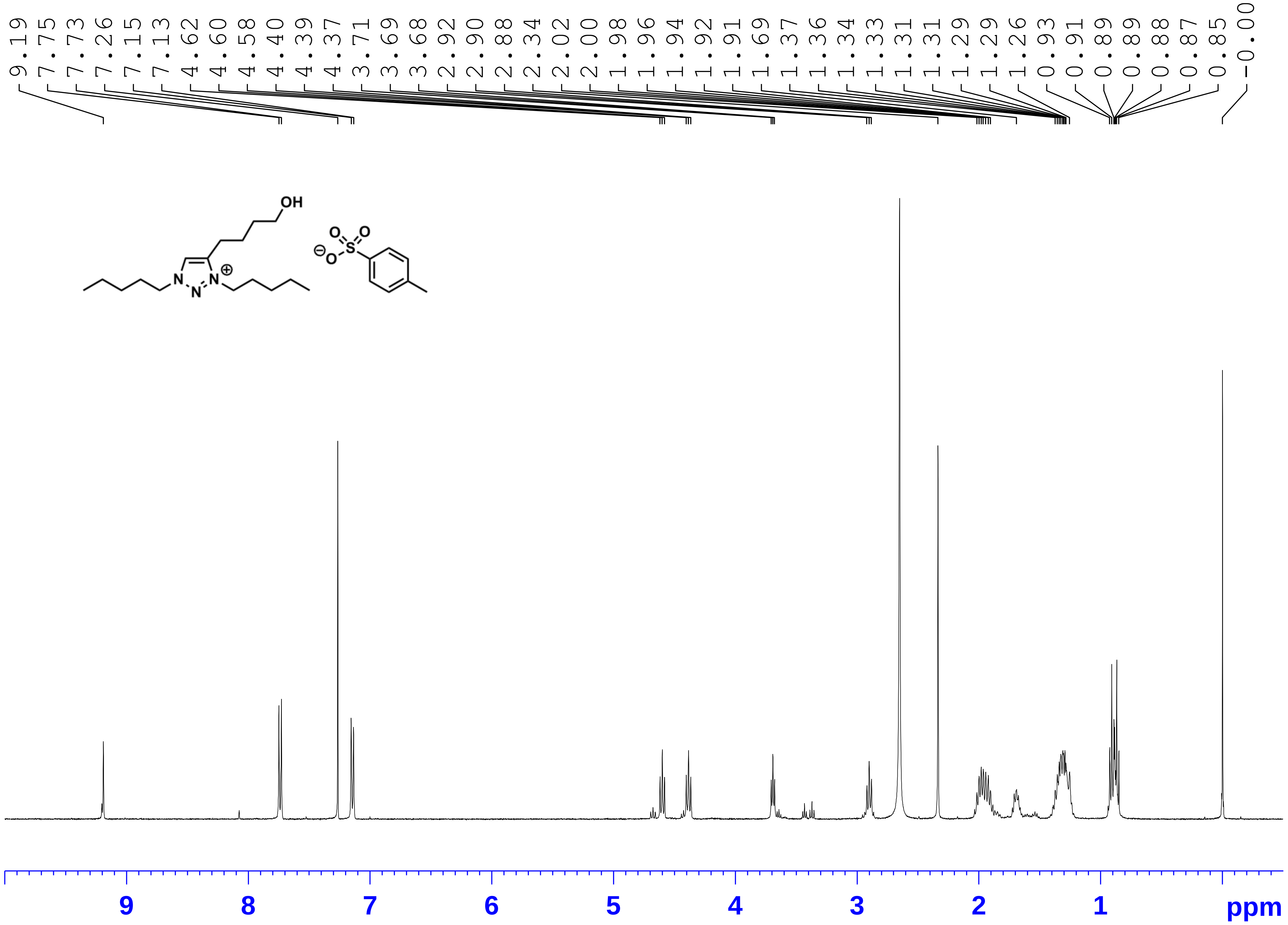
9.19
7.75
7.73
7.26
7.15
7.13
4.62
4.60
4.58
4.40
4.39
4.37
3.71
3.69
3.68
2.92
2.90
2.88
2.34
2.02
2.00
1.98
1.96
1.94
1.92
1.91
1.69
1.37
1.36
1.34
1.33
1.31
1.31
1.29
1.29
1.26
0.93
0.91
0.89
0.89
0.88
0.87
0.85
-0.00
OH
9
8
7
6
5
4
3
2
1
ppm

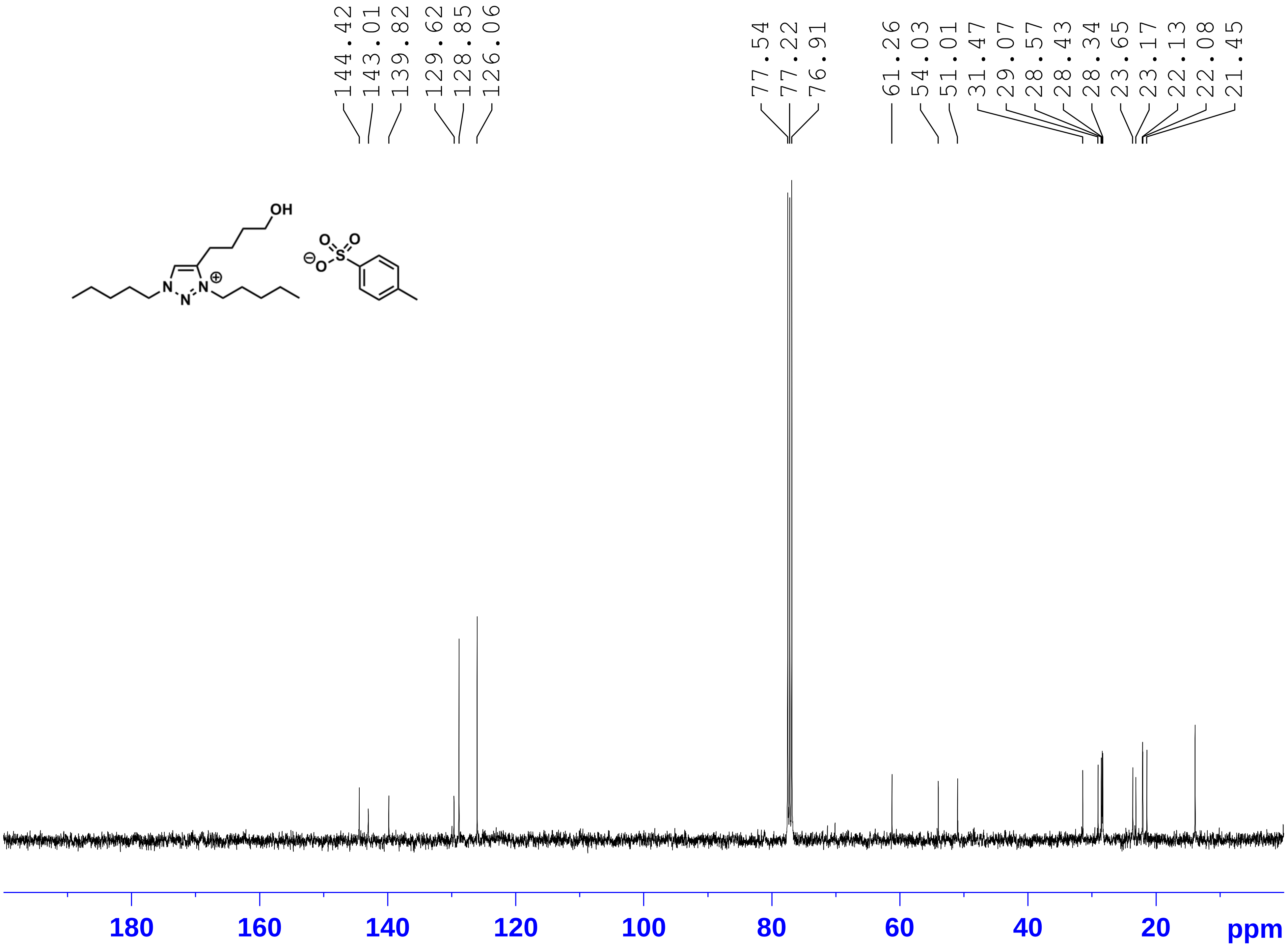

144.42
143.01
139.82
129.62
128.85
126.06
77.54
77.22
76.91
61.26
54.03
51.01
31.47
29.07
28.57
28.43
28.34
23.65
23.17
22.13
22.08
21.45
OH
N
N
N
⊕
O
O
S
⊖
O
180
160
140
120
100
80
60
40
20
ppm

Spectrum

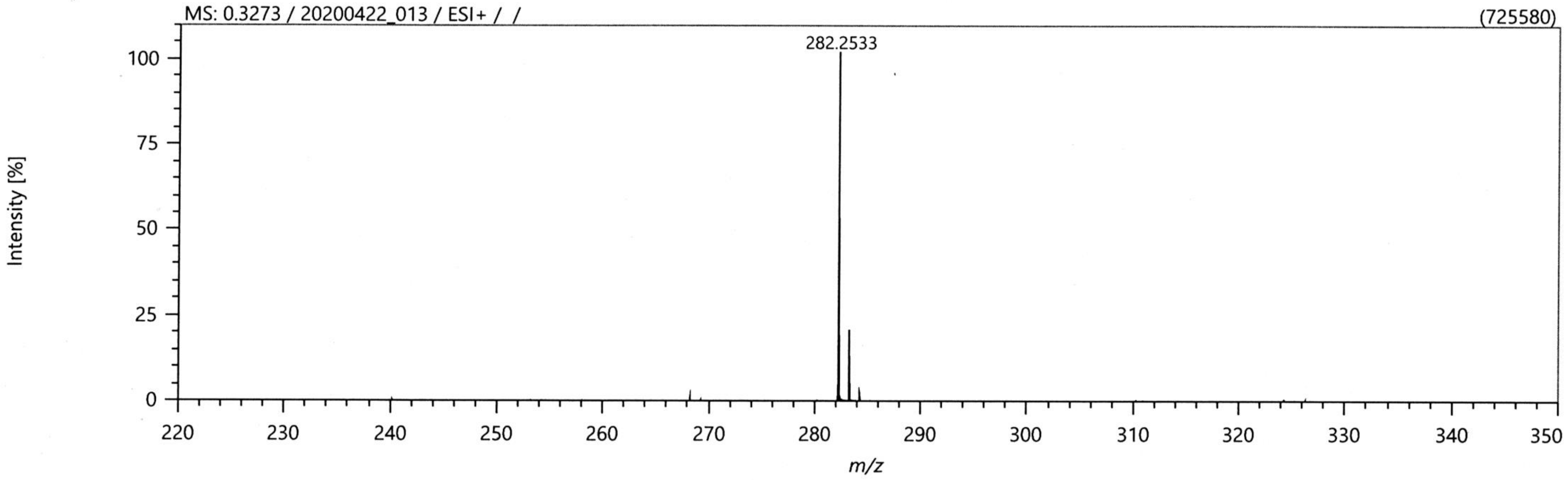

Elemental Composition

Parameters	
Tolerance:	±10.00 ppm
Electron:	Odd/Even
Charge:	+1
DBE:	-99.0 - 999.0

Elements Set 1:

Symbol	C	H	O	Na	N	F
Min	0	0	1	0	3	0
Max	1000	4000	1	0	3	0

Results

Mass	Formula	Calculated Mass	Mass Difference [mDa]	Mass Difference [ppm]	DBE
282.25328	C16 H32 N3 O	282.25399	-0.71	-2.52	2.5

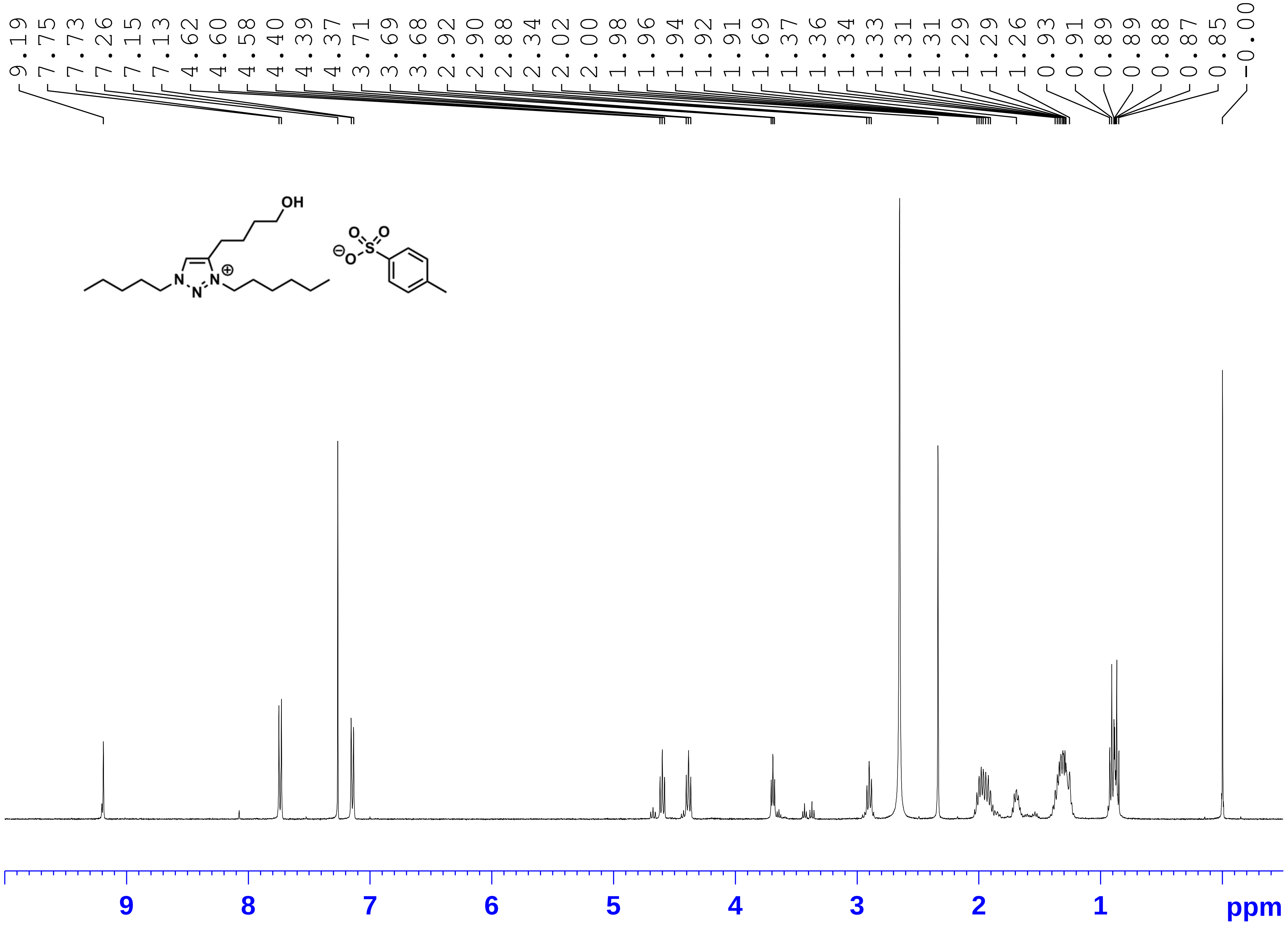

9.19
7.75
7.73
7.26
7.15
7.13
4.62
4.60
4.58
4.40
4.39
4.37
3.71
3.69
3.68
2.92
2.90
2.88
2.34
2.02
2.00
1.98
1.96
1.94
1.92
1.91
1.69
1.37
1.36
1.34
1.33
1.31
1.31
1.29
1.29
1.26
0.93
0.91
0.89
0.89
0.88
0.87
0.85
-0.00
OH
N
N
N
O
O
S
O
9
8
7
6
5
4
3
2
1
ppm

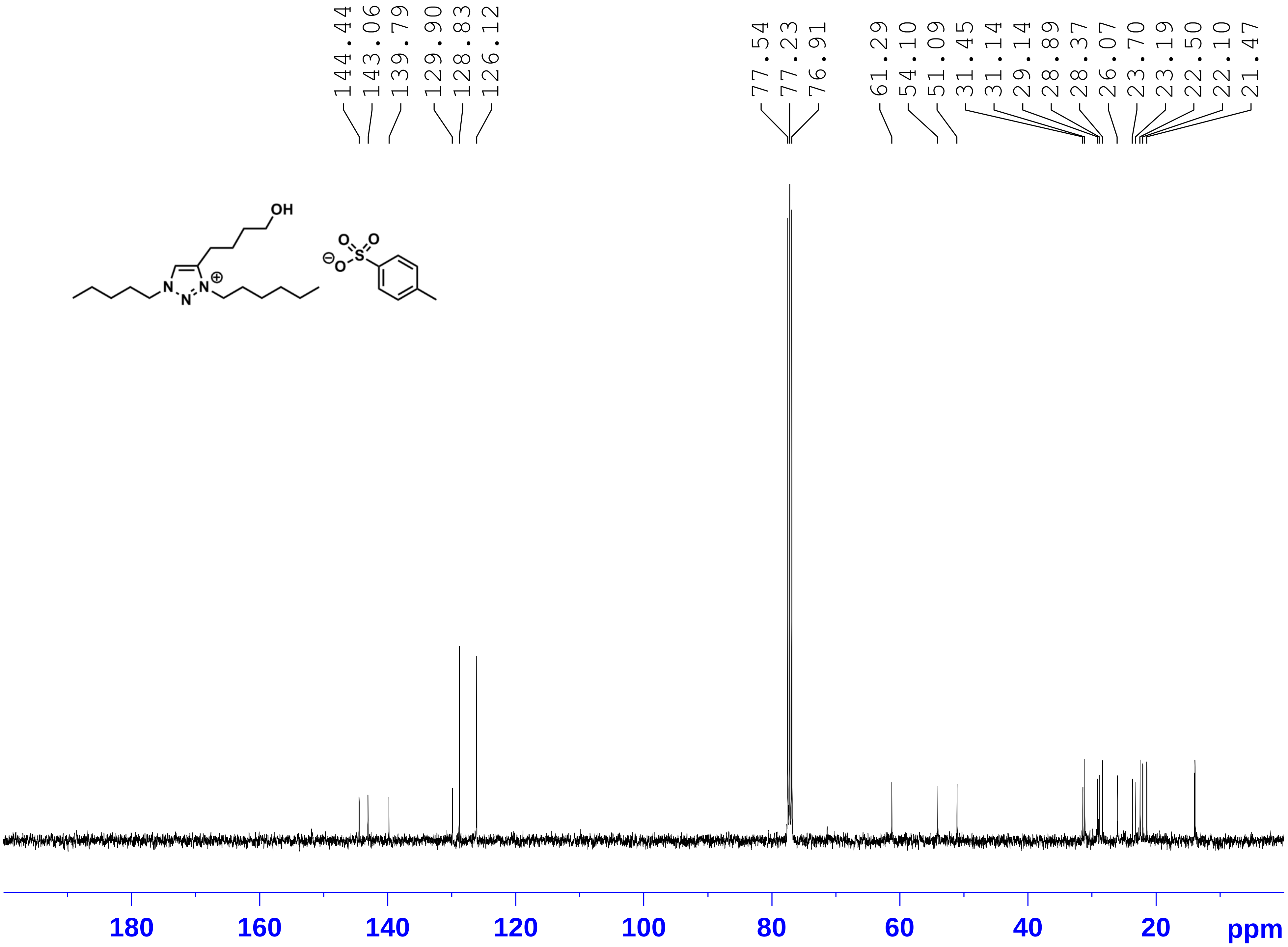

144.44
143.06
139.79
129.90
128.83
126.12
77.54
77.23
76.91
61.29
54.10
51.09
31.45
31.14
29.14
28.89
28.37
26.07
23.70
23.19
22.50
22.10
21.47
OH
180
160
140
120
100
80
60
40
20
ppm

Spectrum

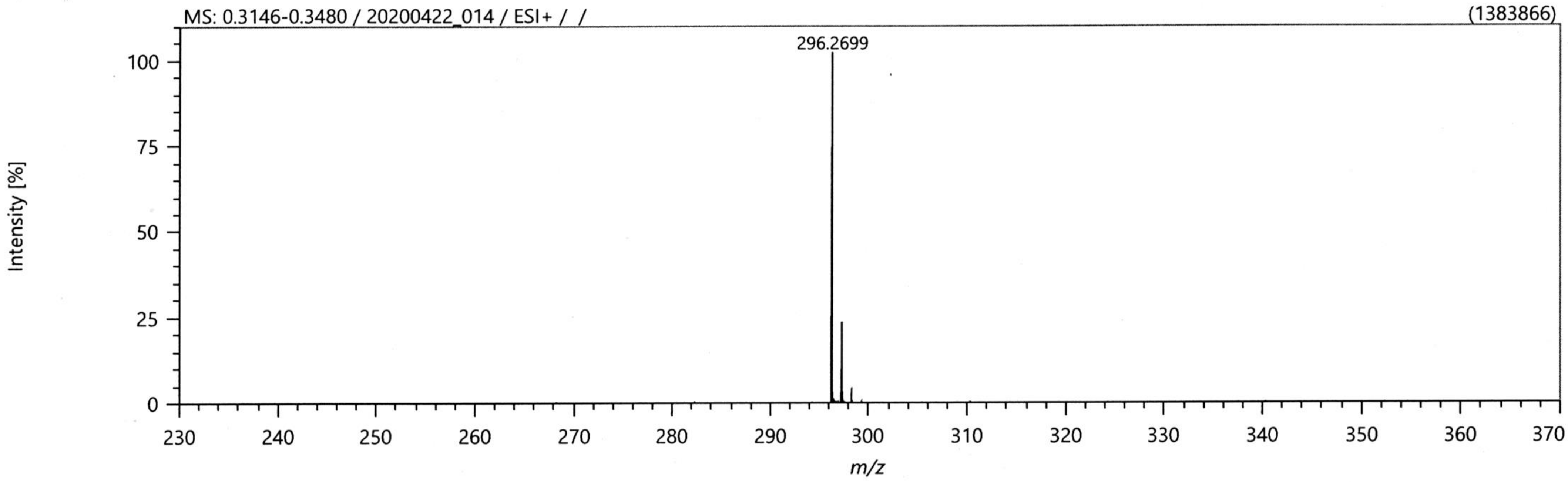

Elemental Composition

Parameters		Elements Set 1:						
Tolerance:	±10.00 ppm	Symbol	C	H	O	Na	N	F
Electron:	Odd/Even	Min	0	0	1	0	3	0
Charge:	+1	Max	1000	4000	1	0	3	0
DBE:	-99.0 - 999.0							

Results

Mass	Formula	Calculated Mass	Mass Difference [mDa]	Mass Difference [ppm]	DBE
296.26988	C17 H34 N3 O	296.26964	0.24	0.81	2.5

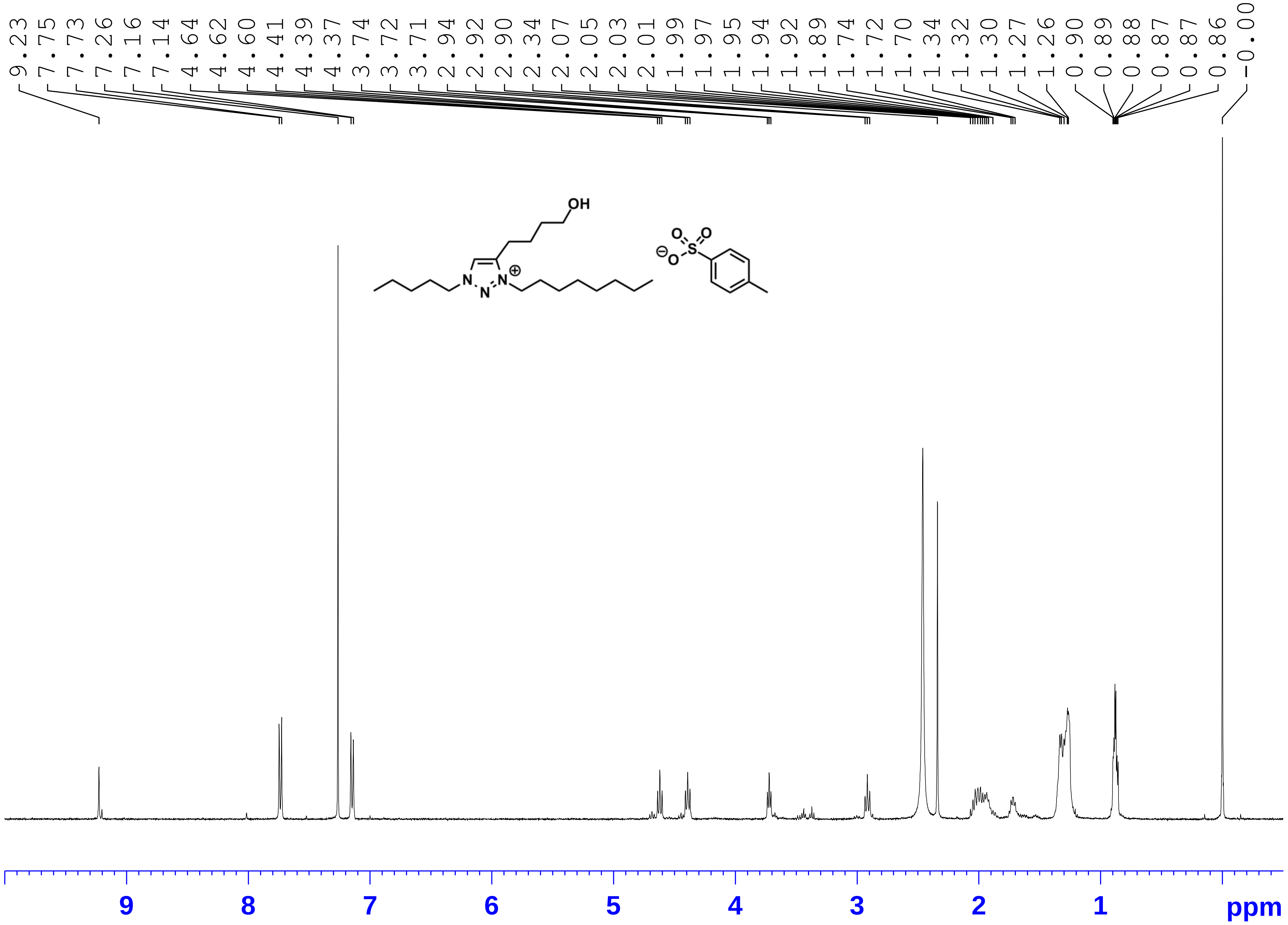

9.23
7.75
7.73
7.26
7.16
7.14
4.64
4.62
4.60
4.41
4.39
4.37
3.74
3.72
3.71
2.94
2.92
2.90
2.34
2.07
2.05
2.03
2.01
1.99
1.97
1.95
1.94
1.92
1.89
1.74
1.72
1.70
1.34
1.32
1.30
1.27
1.26
0.90
0.89
0.88
0.87
0.87
0.86
-0.00
OH
N
N
N
O
O
S
O
9
8
7
6
5
4
3
2
1
ppm

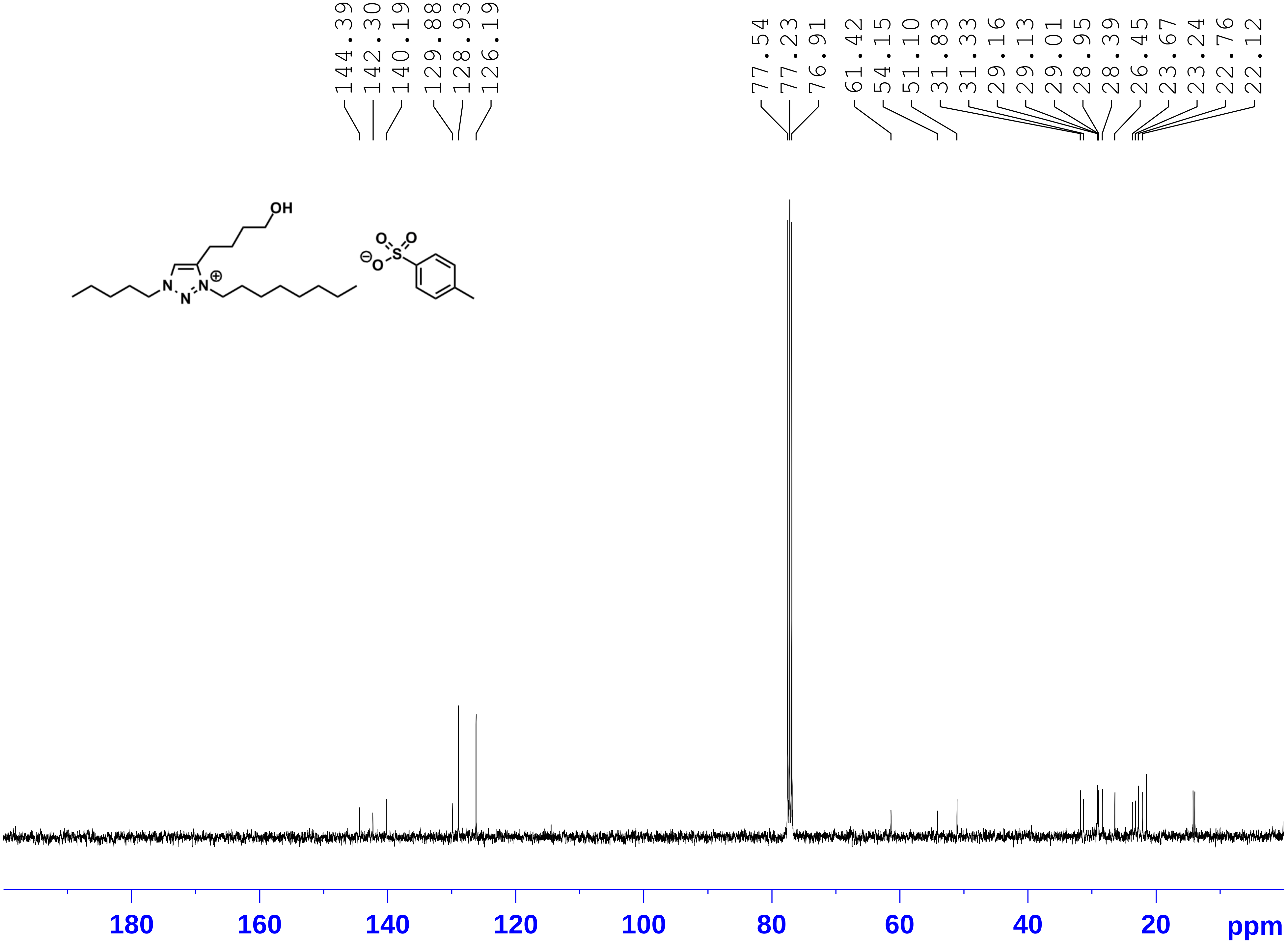
144.39
142.30
140.19
129.88
128.93
126.19
77.54
77.23
76.91
61.42
54.15
51.10
31.83
31.33
29.16
29.13
29.01
28.95
28.39
26.45
23.67
23.24
22.76
22.12
OH
N
N
N
O
S
O
O
180
160
140
120
100
80
60
40
20
ppm

Spectrum

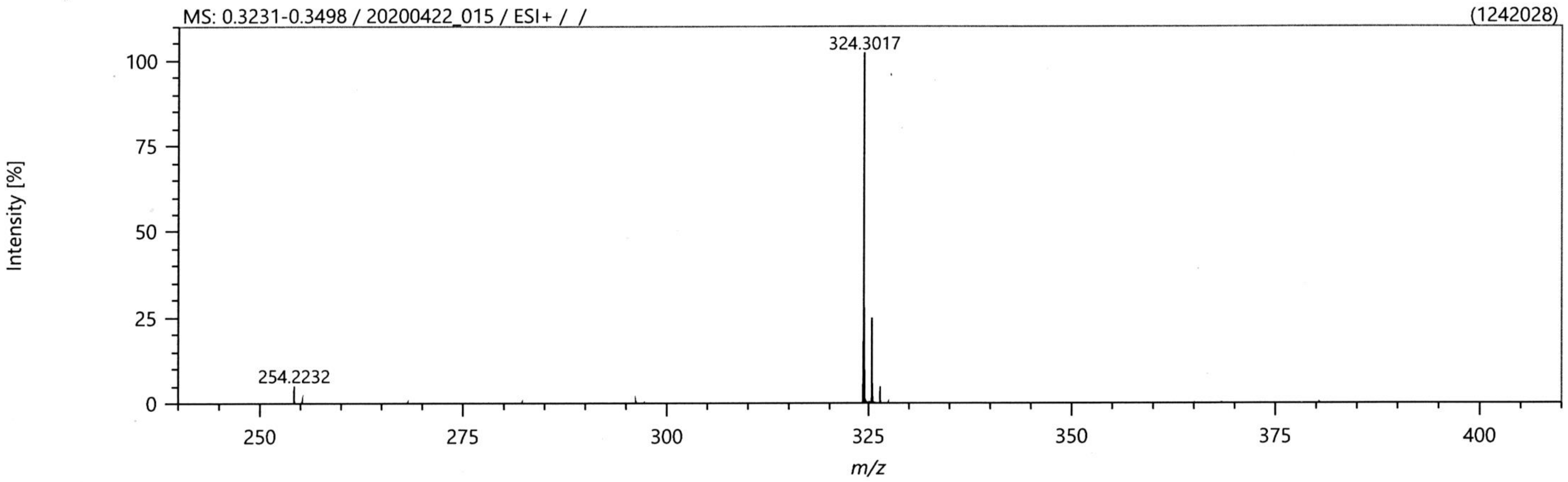

Elemental Composition

Parameters	
Tolerance:	±10.00 ppm
Electron:	Odd/Even
Charge:	+1
DBE:	-99.0 - 999.0

Elements Set 1:

Symbol	C	H	O	Na	N	F
Min	0	0	1	0	3	0
Max	1000	4000	1	0	3	0

Results

Mass	Formula	Calculated Mass	Mass Difference [mDa]	Mass Difference [ppm]	DBE
324.30169	C19 H38 N3 O	324.30094	0.75	2.30	2.5

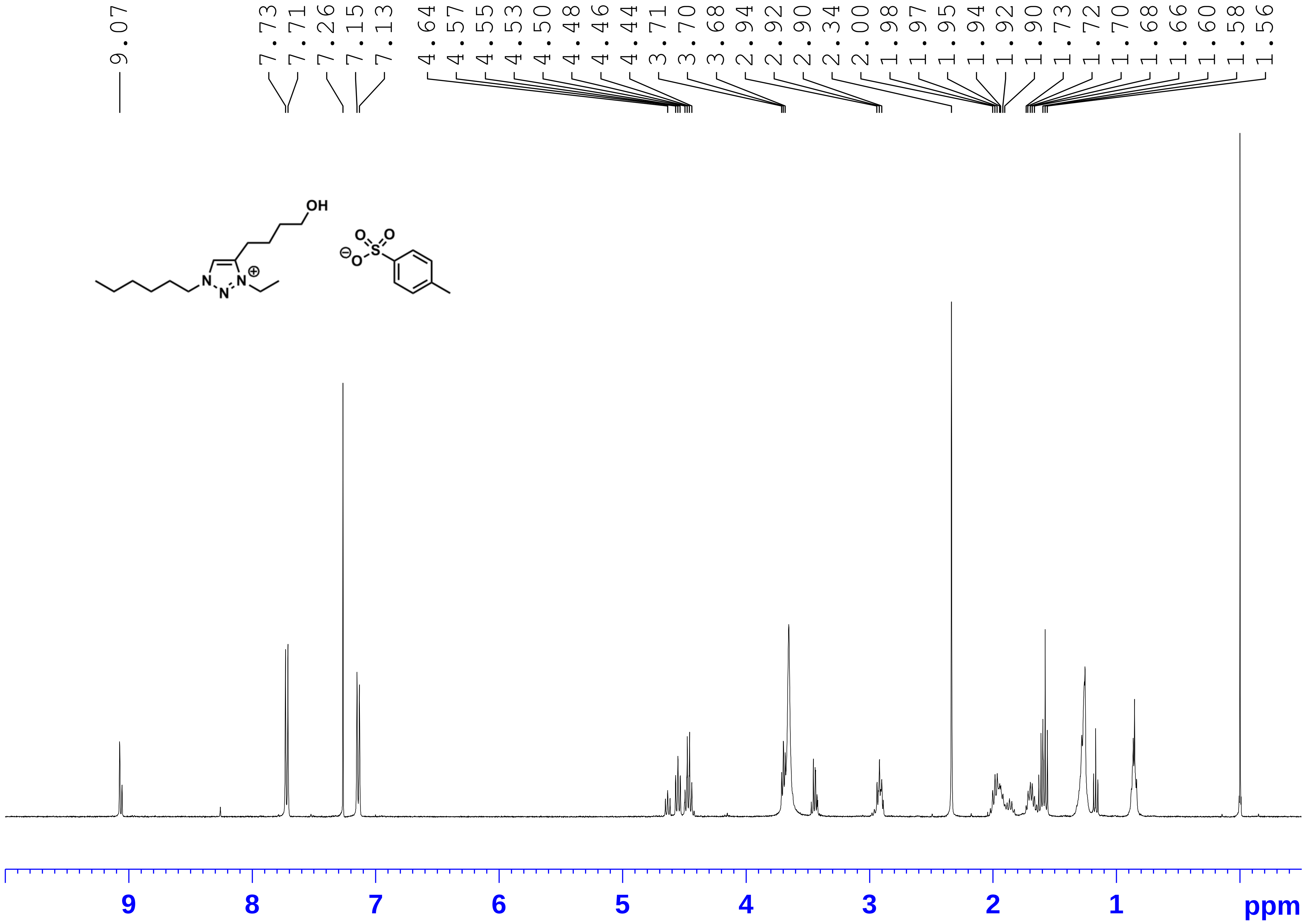
9.07
7.73
7.71
7.26
7.15
7.13
4.64
4.57
4.55
4.53
4.50
4.48
4.46
4.44
3.71
3.70
3.68
2.94
2.92
2.90
2.34
2.00
1.98
1.97
1.95
1.94
1.92
1.90
1.73
1.72
1.70
1.68
1.66
1.60
1.58
1.56
OH
N
N
N
O
S
O
O
9
8
7
6
5
4
3
2
1
ppm

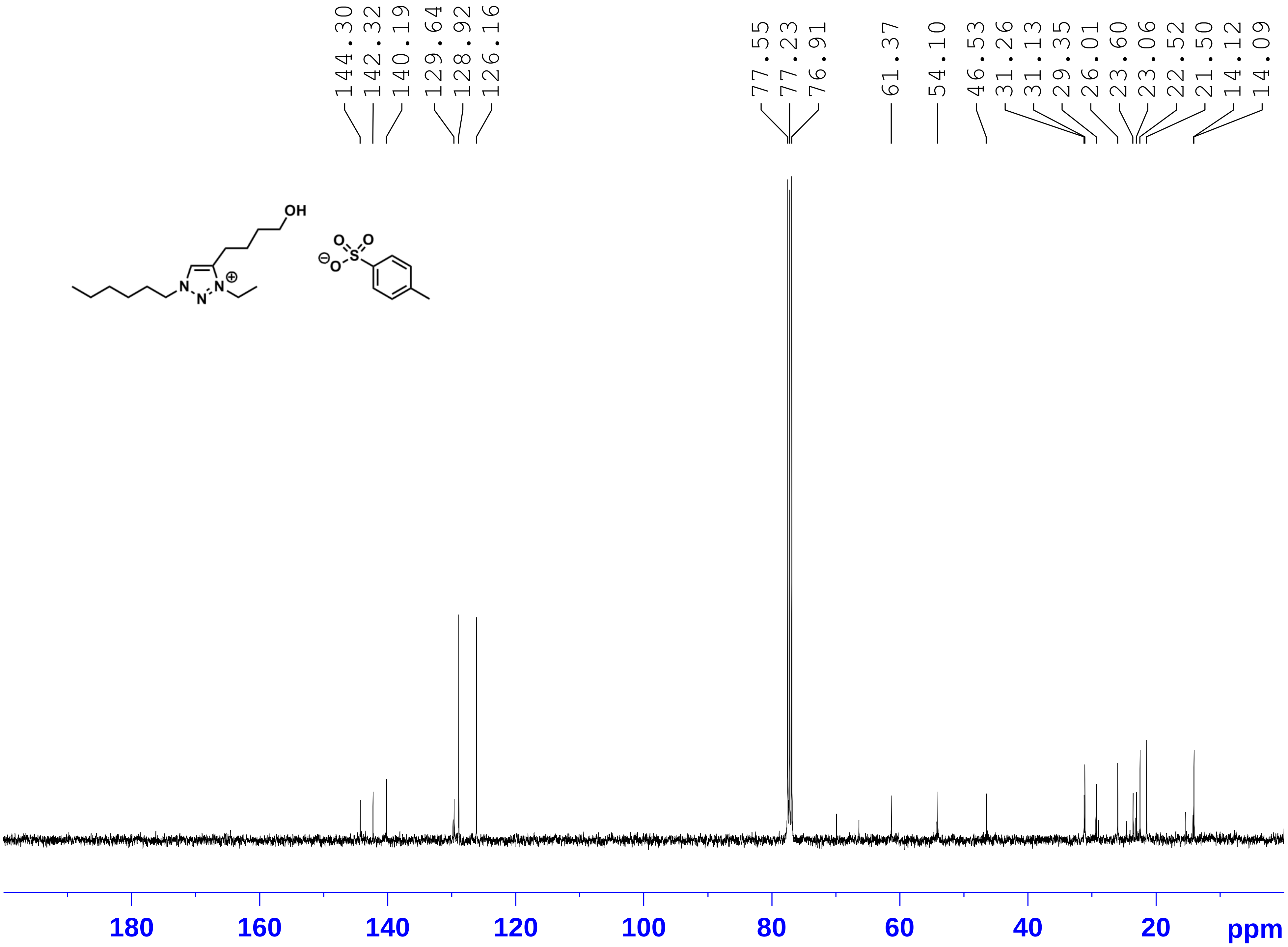
144.30
142.32
140.19
129.64
128.92
126.16
77.55
77.23
76.91
61.37
54.10
46.53
31.26
31.13
29.35
26.01
23.60
23.06
22.52
21.50
14.12
14.09
OH
N
N
N
O
O
O
S
180
160
140
120
100
80
60
40
20
ppm

Spectrum

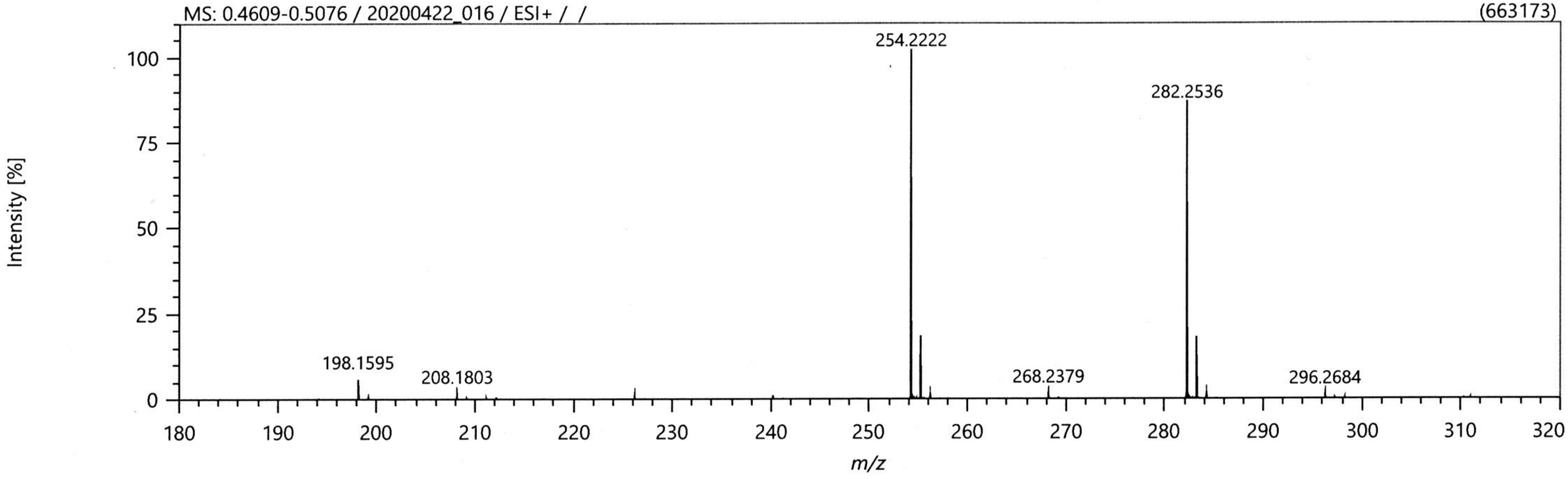

Elemental Composition

Parameters

Tolerance:	±10.00 ppm
Electron:	Odd/Even
Charge:	+1
DBE:	-99.0 - 999.0

Elements Set 1:

Symbol	C	H	O	Na	N	F
Min	0	0	1	0	3	0
Max	1000	4000	1	0	3	0

Results

Mass	Formula	Calculated Mass	Mass Difference [mDa]	Mass Difference [ppm]	DBE
254.22221	C14 H28 N3 O	254.22269	-0.48	-1.88	2.5

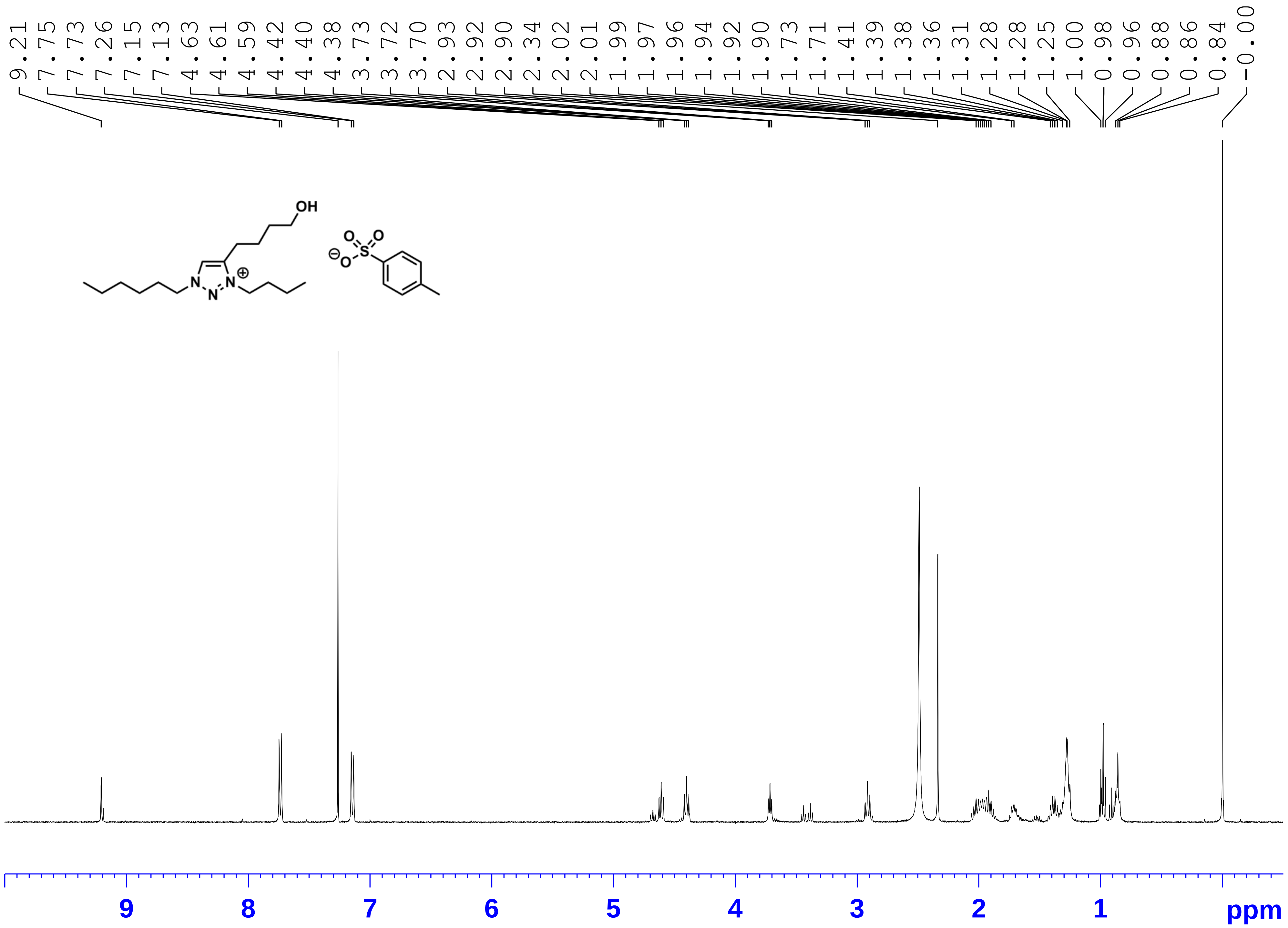
9.21
7.75
7.73
7.26
7.15
7.13
4.63
4.61
4.59
4.42
4.40
4.38
3.73
3.72
3.70
2.93
2.92
2.90
2.34
2.02
2.01
1.99
1.97
1.96
1.94
1.92
1.90
1.73
1.71
1.41
1.39
1.38
1.36
1.31
1.28
1.28
1.25
1.00
0.98
0.96
0.88
0.86
0.84
-0.00
OH
N
N
N
O
O
S
O
9
8
7
6
5
4
3
2
1
ppm

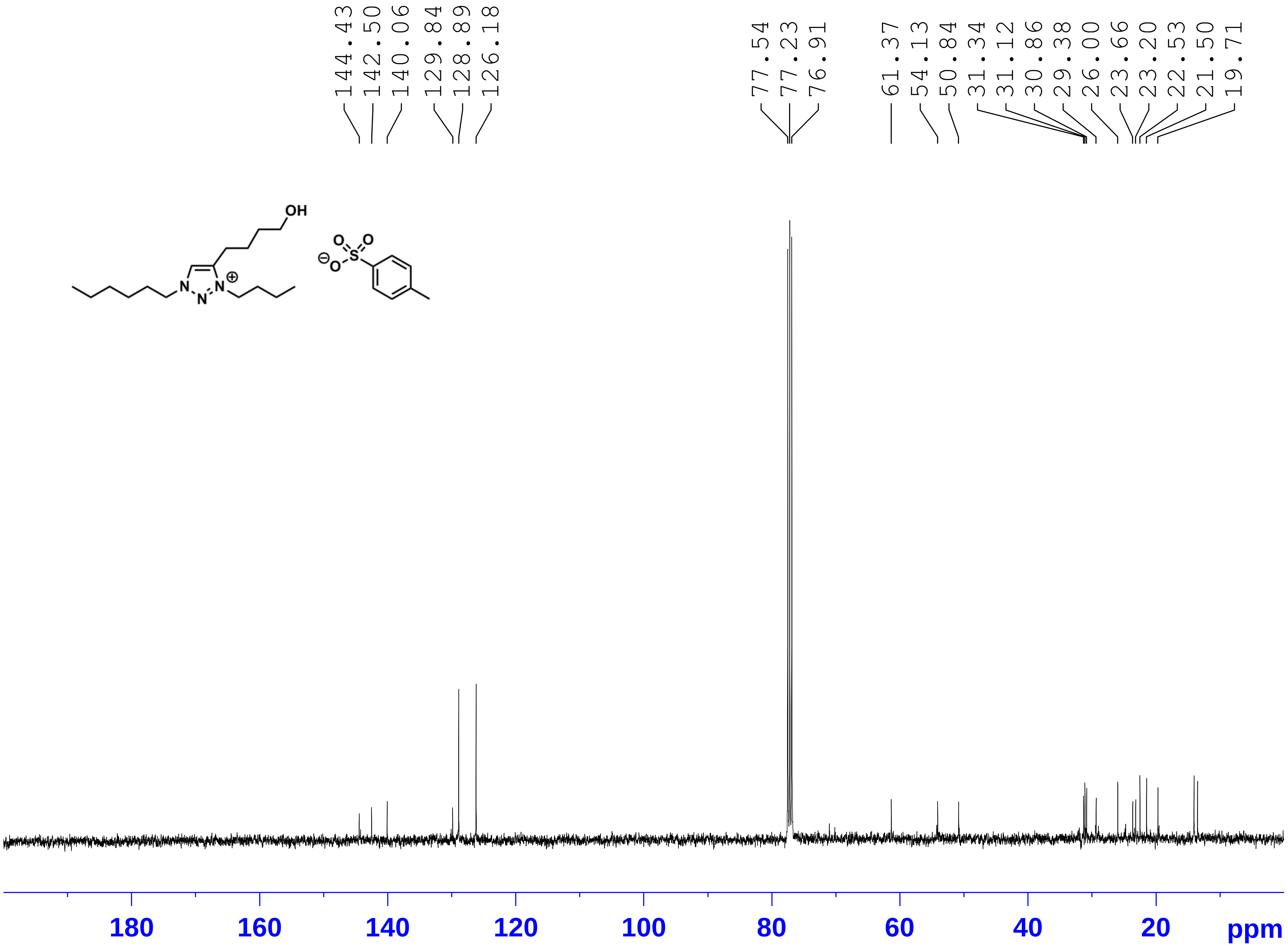
144.43
142.50
140.06
129.84
128.89
126.18
77.54
77.23
76.91
61.37
54.13
50.84
31.34
31.12
30.86
29.38
26.00
23.66
23.20
22.53
21.50
19.71
OH
O
S
O
O
N
N
N
180
160
140
120
100
80
60
40
20
ppm

Spectrum

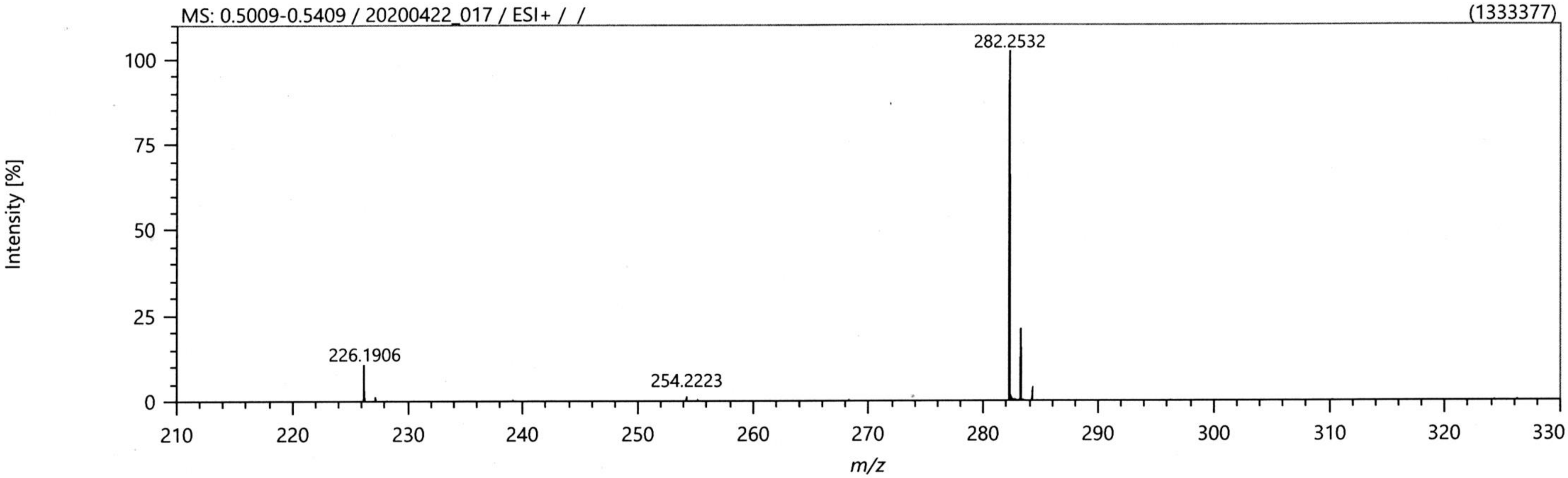

Elemental Composition

Parameters	
Tolerance:	±10.00 ppm
Electron:	Odd/Even
Charge:	+1
DBE:	-99.0 - 999.0

Elements Set 1:

Symbol	C	H	O	Na	N	F
Min	0	0	1	0	3	0
Max	1000	4000	1	0	3	0

Results

Mass	Formula	Calculated Mass	Mass Difference [mDa]	Mass Difference [ppm]	DBE
282.25322	C16 H32 N3 O	282.25399	-0.77	-2.72	2.5

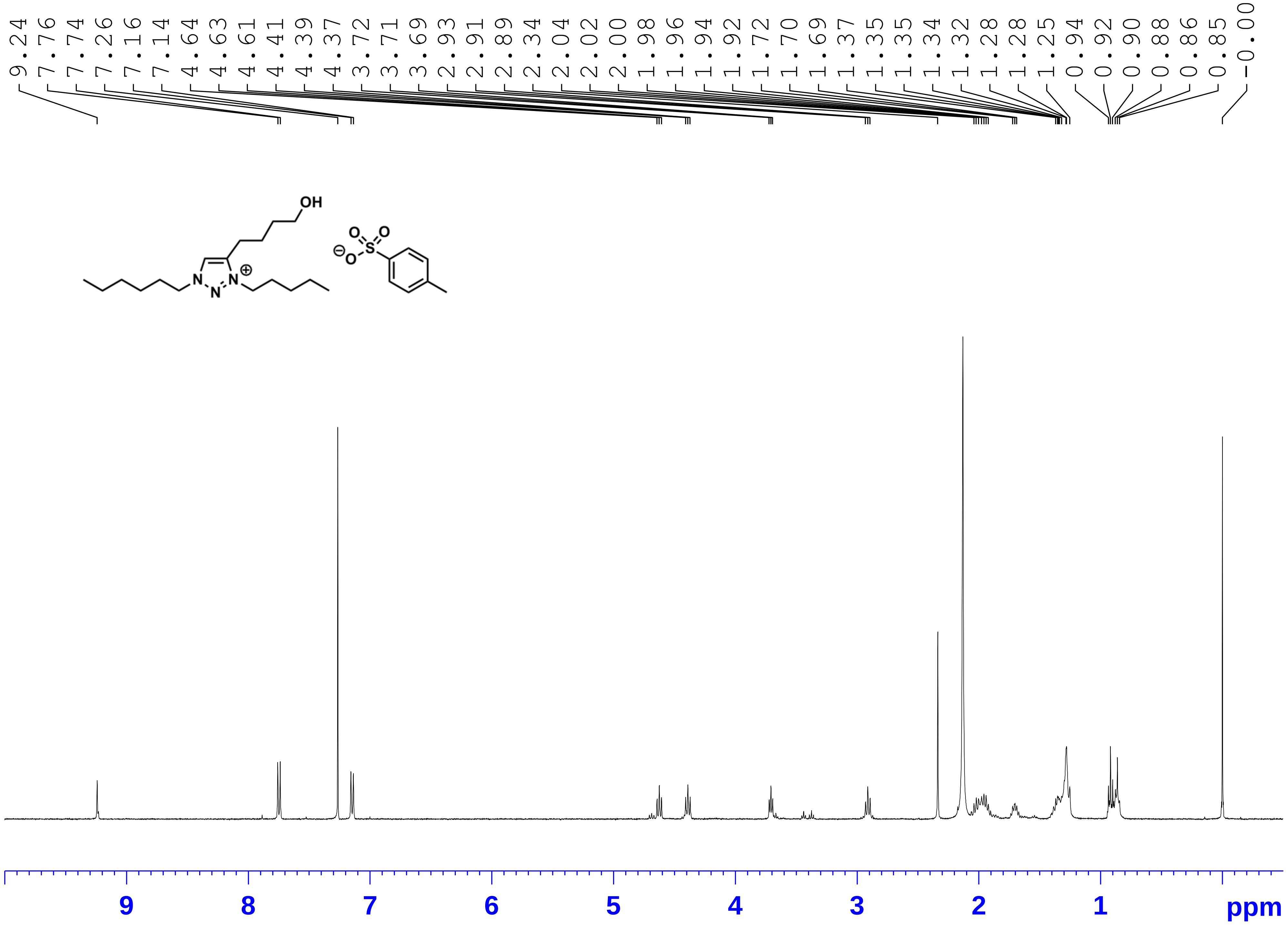

9.24
7.76
7.74
7.26
7.16
7.14
4.64
4.63
4.61
4.41
4.39
4.37
3.72
3.71
3.69
2.93
2.91
2.89
2.34
2.04
2.02
2.00
1.98
1.96
1.94
1.92
1.72
1.70
1.69
1.37
1.35
1.35
1.34
1.32
1.28
1.28
1.25
0.94
0.92
0.90
0.88
0.86
0.85
-0.00
OH
O
O
S
O
N
N
N
9
8
7
6
5
4
3
2
1
ppm

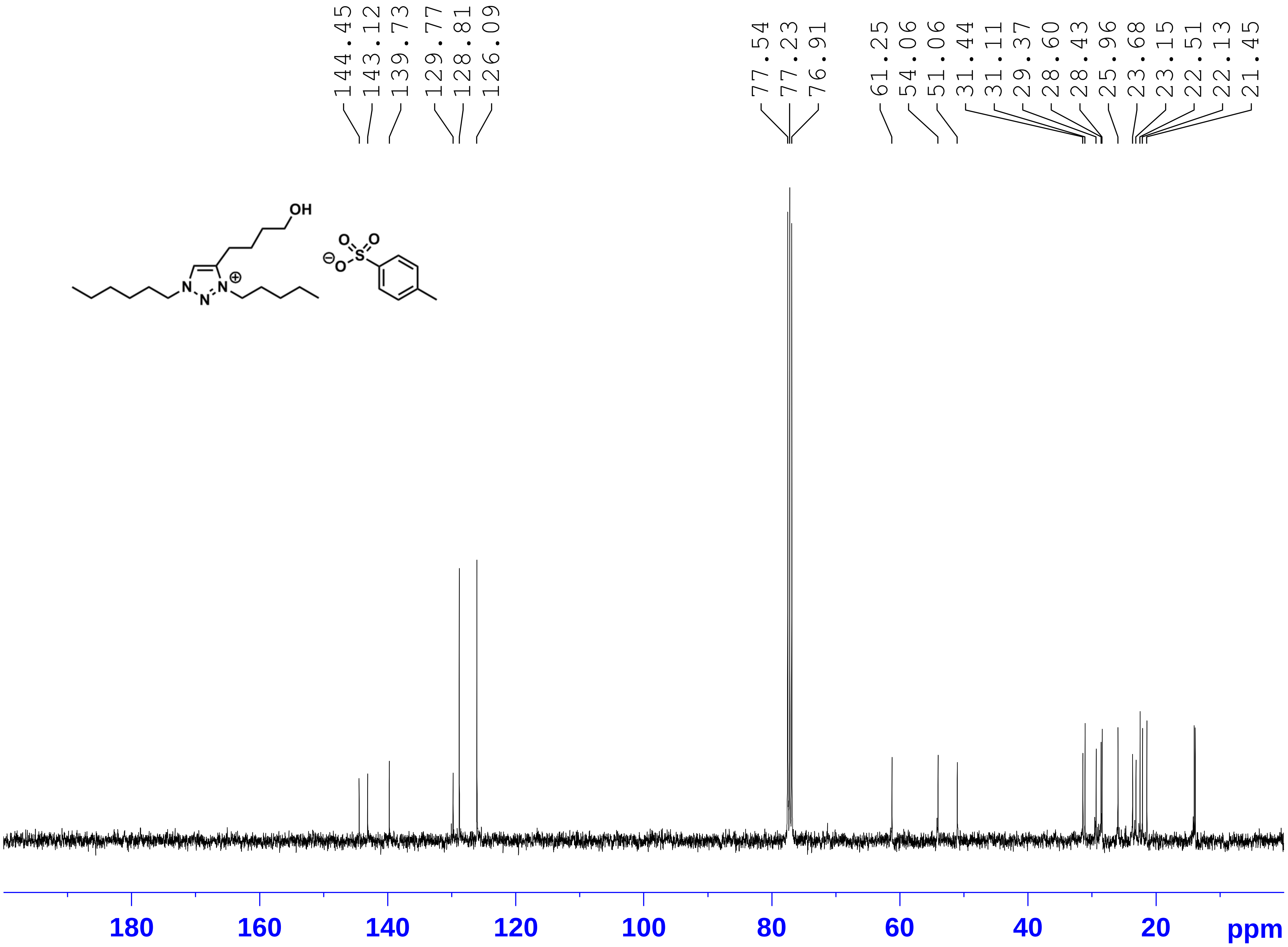

144.45
143.12
139.73
129.77
128.81
126.09
77.54
77.23
76.91
61.25
54.06
51.06
31.44
31.11
29.37
28.60
28.43
25.96
23.68
23.15
22.51
22.13
21.45
OH
O
S
N
180
160
140
120
100
80
60
40
20
ppm

Spectrum

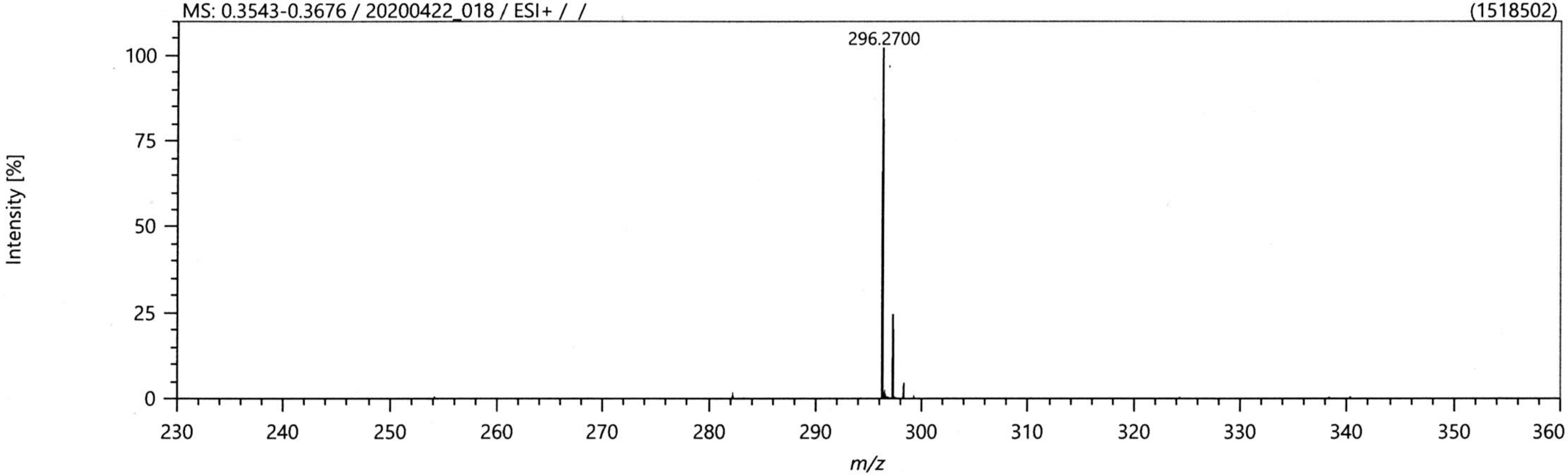

Elemental Composition

Parameters	
Tolerance:	±10.00 ppm
Electron:	Odd/Even
Charge:	+1
DBE:	-99.0 - 999.0

Elements Set 1:

Symbol	C	H	O	Na	N	F
Min	0	0	1	0	3	0
Max	1000	4000	1	0	3	0

Results

Mass	Formula	Calculated Mass	Mass Difference [mDa]	Mass Difference [ppm]	DBE
296.27003	C17 H34 N3 O	296.26964	0.40	1.34	2.5

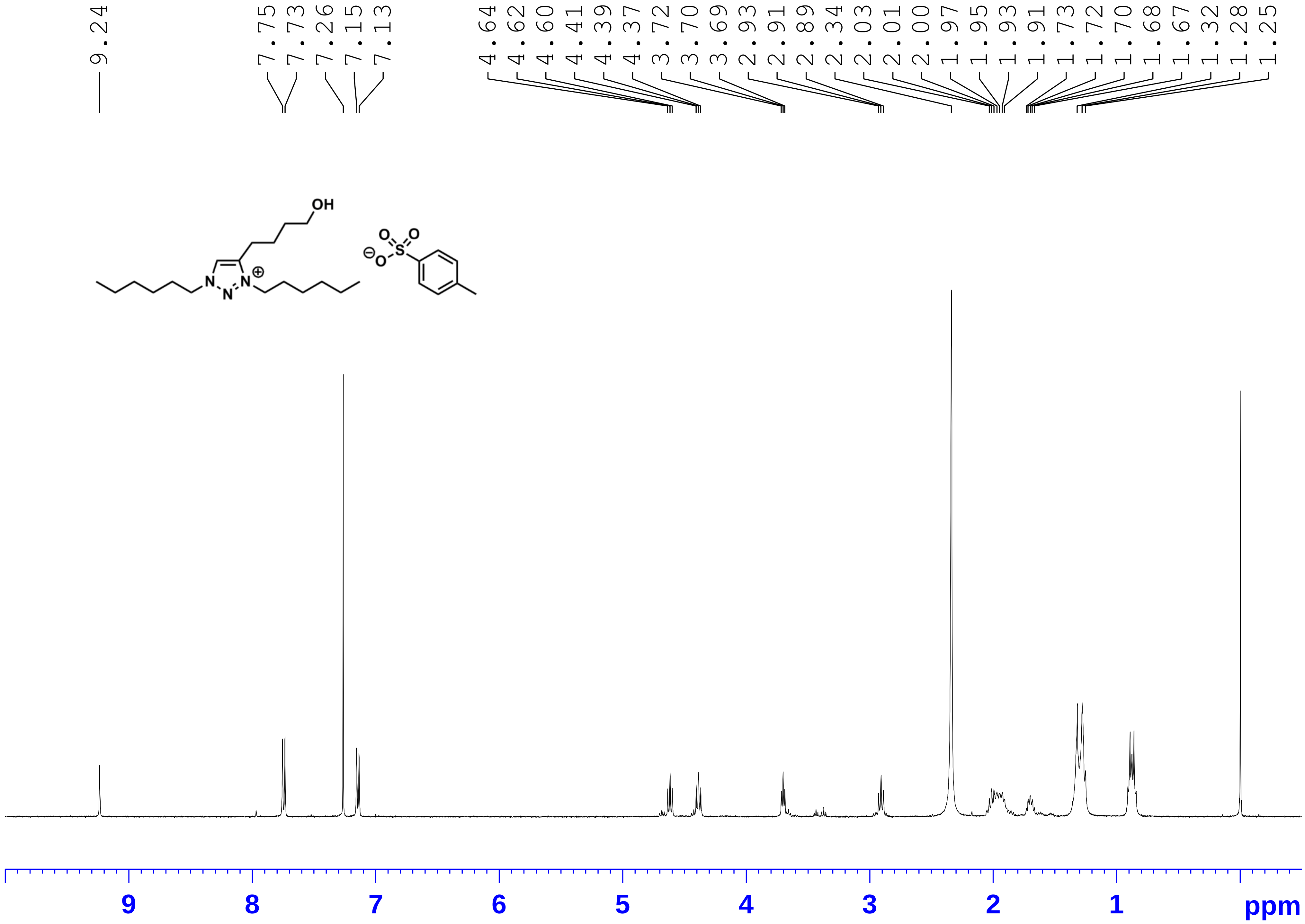
9.24
7.75 7.73 7.26 7.15 7.13
4.64 4.62 4.60 4.41 4.39 4.37 3.72 3.70 3.69 2.93 2.91 2.89 2.34 2.03 2.01 2.00 1.97 1.95 1.93 1.91 1.73 1.72 1.70 1.68 1.67 1.32 1.28 1.25
OH
N
N
N
O
O
S
O
9 8 7 6 5 4 3 2 1 ppm

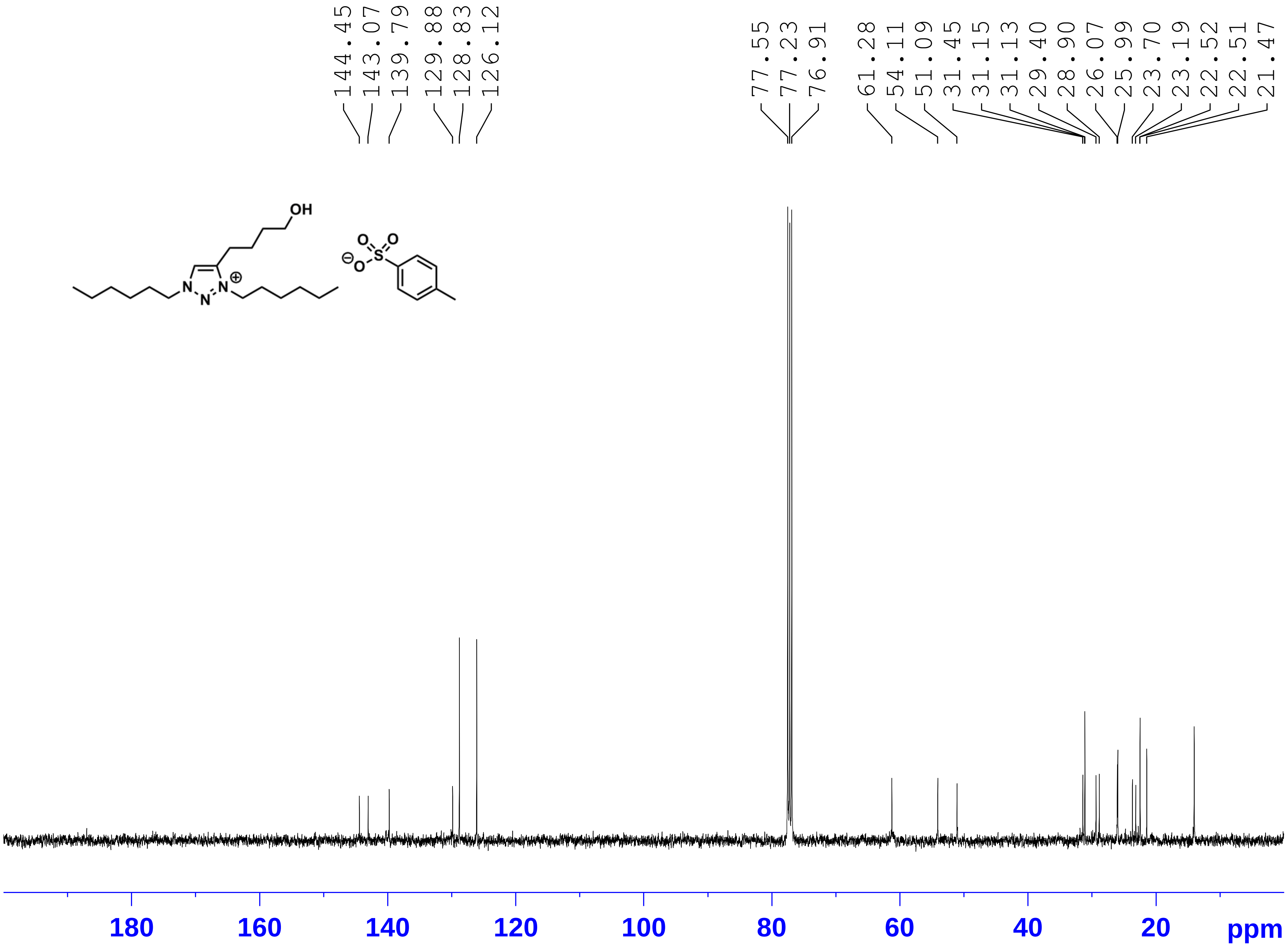

144.45
143.07
139.79
129.88
128.83
126.12
77.55
77.23
76.91
61.28
54.11
51.09
31.45
31.15
31.13
29.40
28.90
26.07
25.99
23.70
23.19
22.52
22.51
21.47
OH
O
S
O
O
N
N
N
180
160
140
120
100
80
60
40
20
ppm

Spectrum

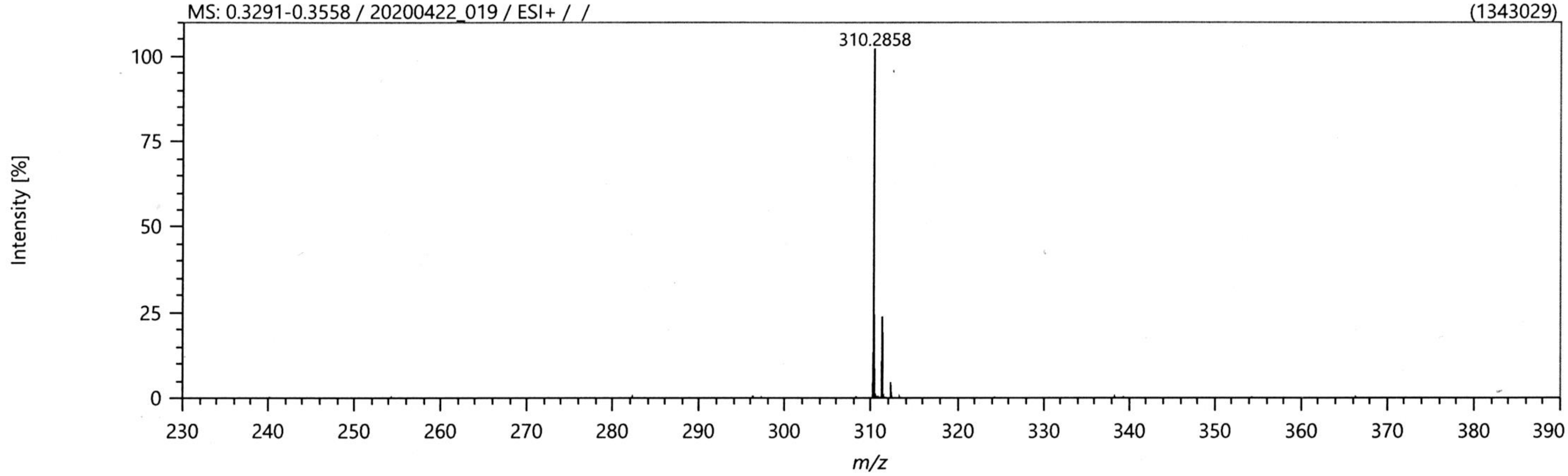

Elemental Composition

Parameters

Tolerance:	±10.00 ppm
Electron:	Odd/Even
Charge:	+1
DBE:	-99.0 - 999.0

Elements Set 1:

Symbol	C	H	O	Na	N	F
Min	0	0	1	0	3	0
Max	1000	4000	1	0	3	0

Results

Mass	Formula	Calculated Mass	Mass Difference [mDa]	Mass Difference [ppm]	DBE
310.28581	C18 H36 N3 O	310.28529	0.52	1.67	2.5

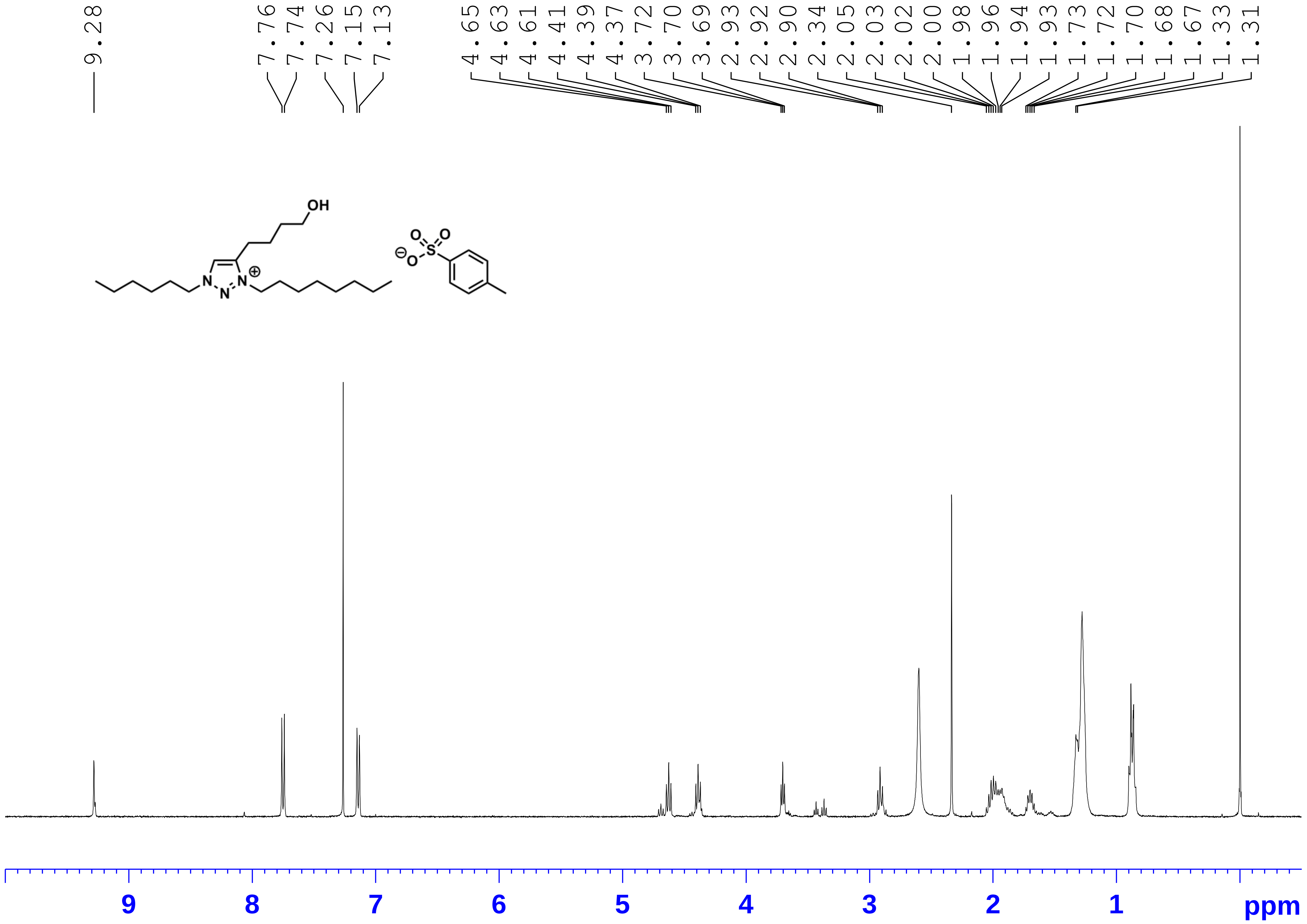

9.28
7.76
7.74
7.26
7.15
7.13
4.65
4.63
4.61
4.41
4.39
4.37
3.72
3.70
3.69
2.93
2.92
2.90
2.34
2.05
2.03
2.02
2.00
1.98
1.96
1.94
1.93
1.73
1.72
1.70
1.68
1.67
1.33
1.31
OH
N
N
N
O
O
S
O
9
8
7
6
5
4
3
2
1
ppm

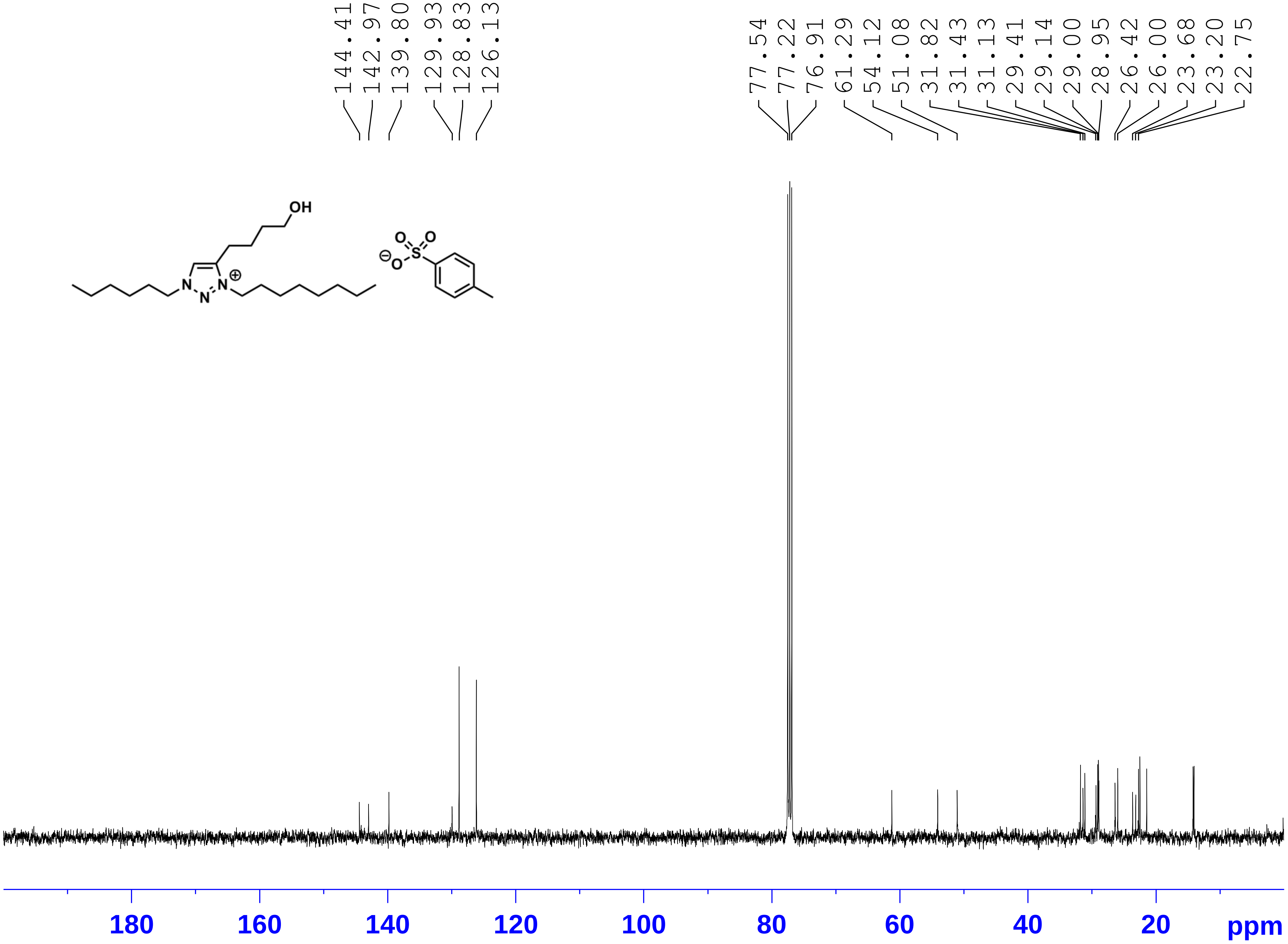
144.41
142.97
139.80
129.93
128.83
126.13
77.54
77.22
76.91
61.29
54.12
51.08
31.82
31.43
31.13
29.41
29.14
29.00
28.95
26.42
26.00
23.68
23.20
22.75
OH
N
N
N
S
O
O
O
180
160
140
120
100
80
60
40
20
ppm

Spectrum

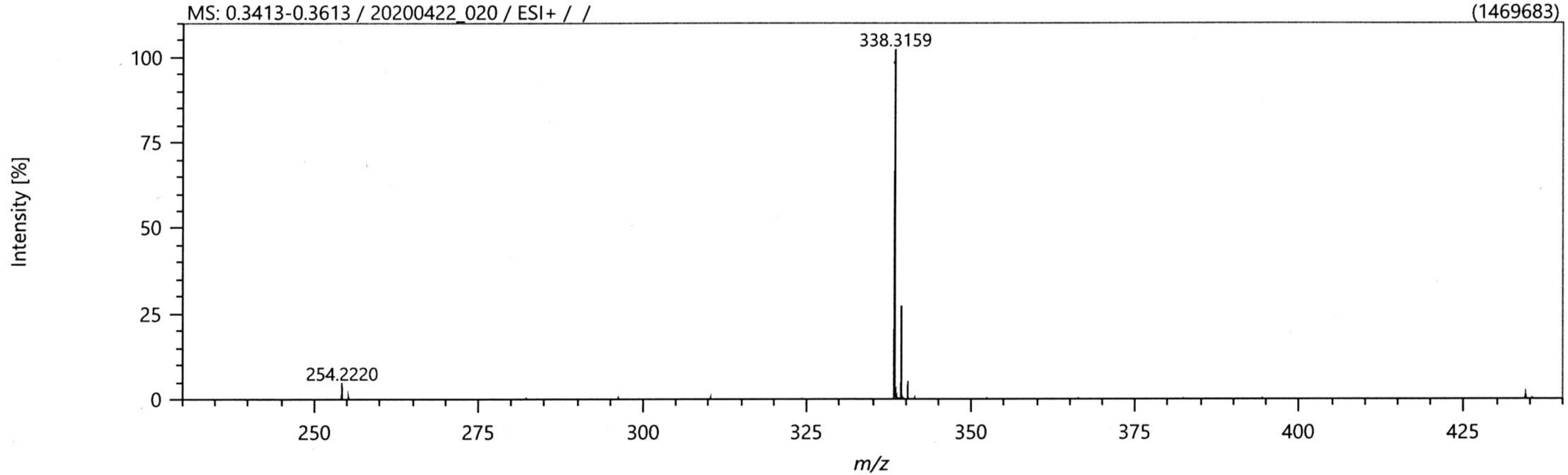

Elemental Composition

Parameters	
Tolerance:	±10.00 ppm
Electron:	Odd/Even
Charge:	+1
DBE:	-99.0 - 999.0

Elements Set 1:

Symbol	C	H	O	Na	N	F
Min	0	0	1	0	3	0
Max	1000	4000	1	0	3	0

Results

Mass	Formula	Calculated Mass	Mass Difference [mDa]	Mass Difference [ppm]	DBE
338.31587	C20 H40 N3 O	338.31659	-0.72	-2.13	2.5

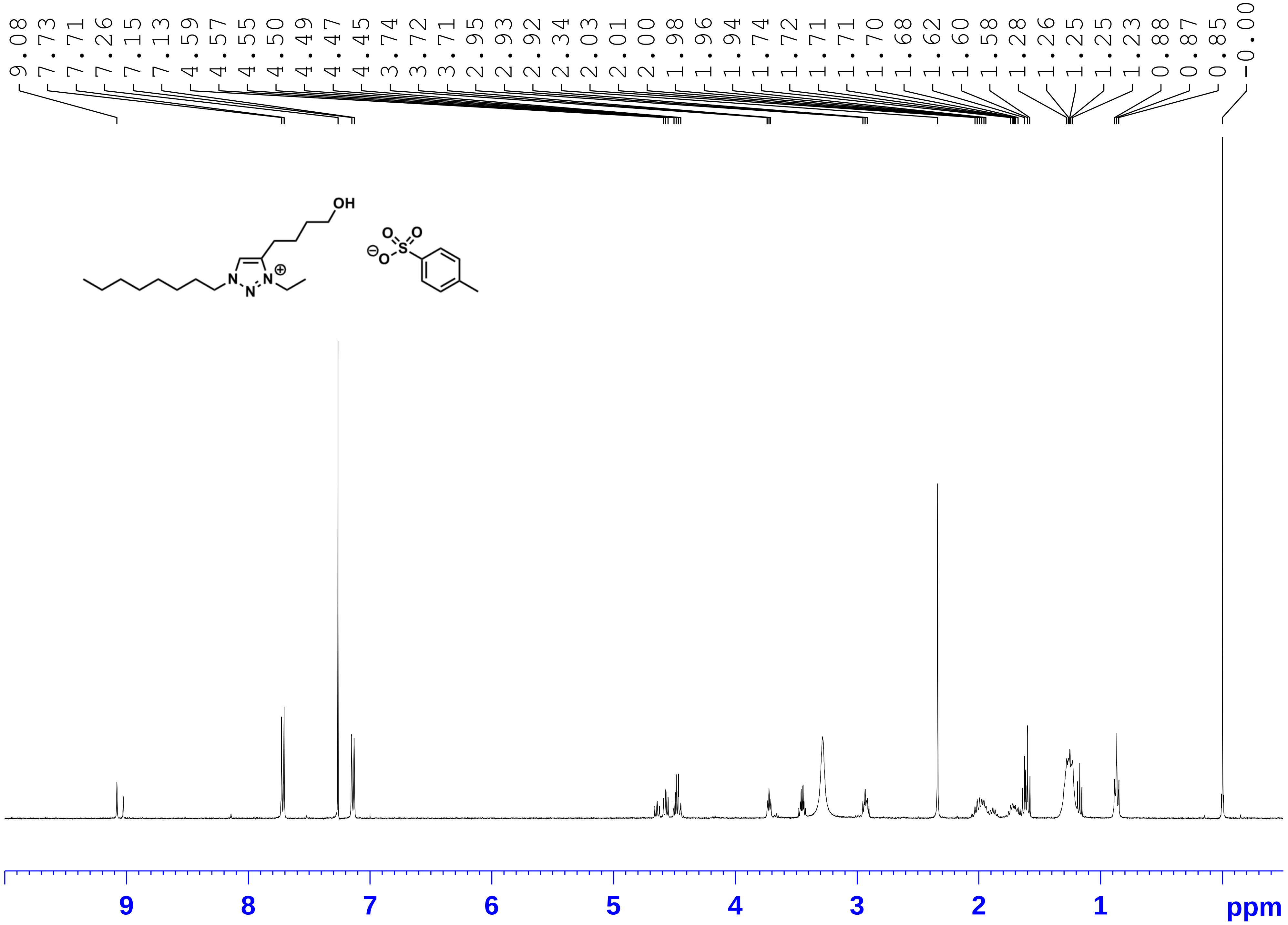
9.08
7.73
7.71
7.26
7.15
7.13
4.59
4.57
4.55
4.50
4.49
4.47
4.45
3.74
3.72
3.71
2.95
2.93
2.92
2.34
2.03
2.01
2.00
1.98
1.96
1.94
1.74
1.72
1.71
1.71
1.70
1.68
1.62
1.60
1.58
1.28
1.26
1.25
1.25
1.23
0.88
0.87
0.85
-0.00
OH
O
S
O
O
N
N
N
9
8
7
6
5
4
3
2
1
ppm

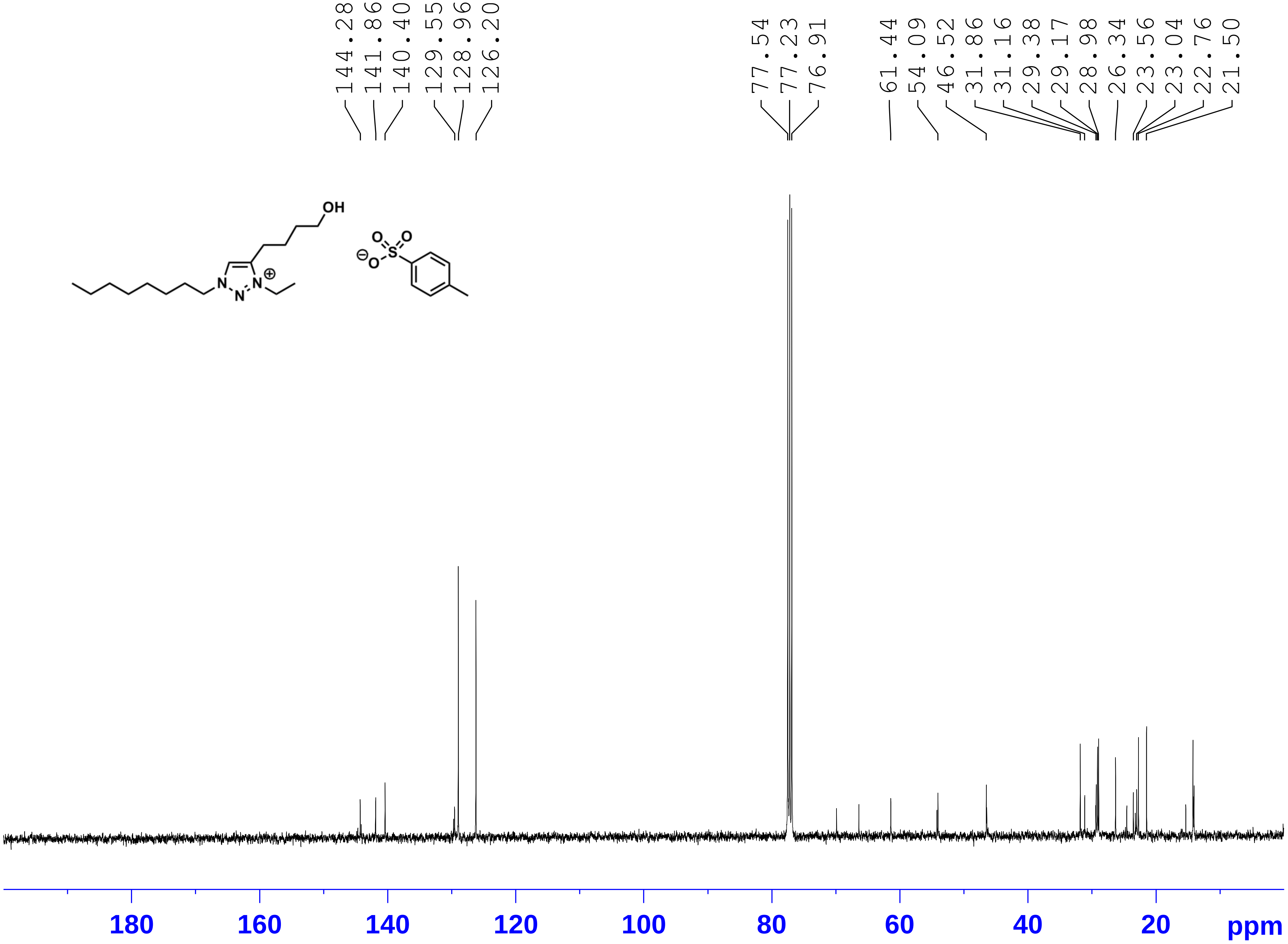
144.28
141.86
140.40
129.55
128.96
126.20
77.54
77.23
76.91
61.44
54.09
46.52
31.86
31.16
29.38
29.17
28.98
26.34
23.56
23.04
22.76
21.50
OH
O
S
N
180
160
140
120
100
80
60
40
20
ppm

Spectrum

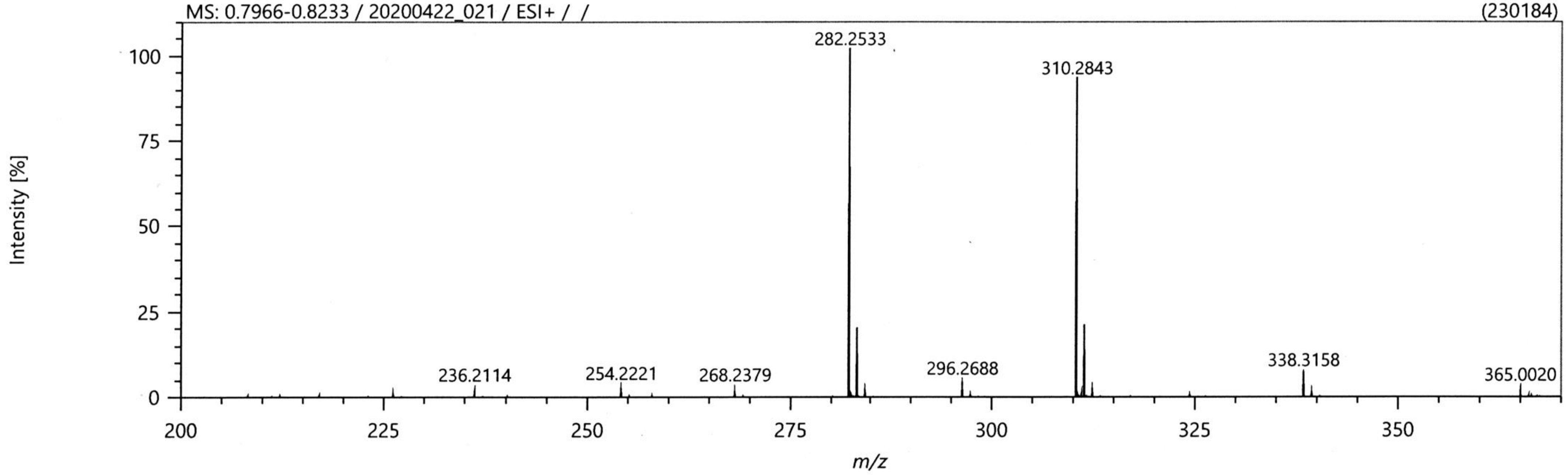

Elemental Composition

Parameters		Elements Set 1:						
Tolerance:	±10.00 ppm	Symbol	C	H	O	Na	N	F
Electron:	Odd/Even	Min	0	0	1	0	3	0
Charge:	+1	Max	1000	4000	1	0	3	0
DBE:	-99.0 - 999.0							

Results

Mass	Formula	Calculated Mass	Mass Difference [mDa]	Mass Difference [ppm]	DBE
282.25332	C16 H32 N3 O	282.25399	-0.67	-2.38	2.5

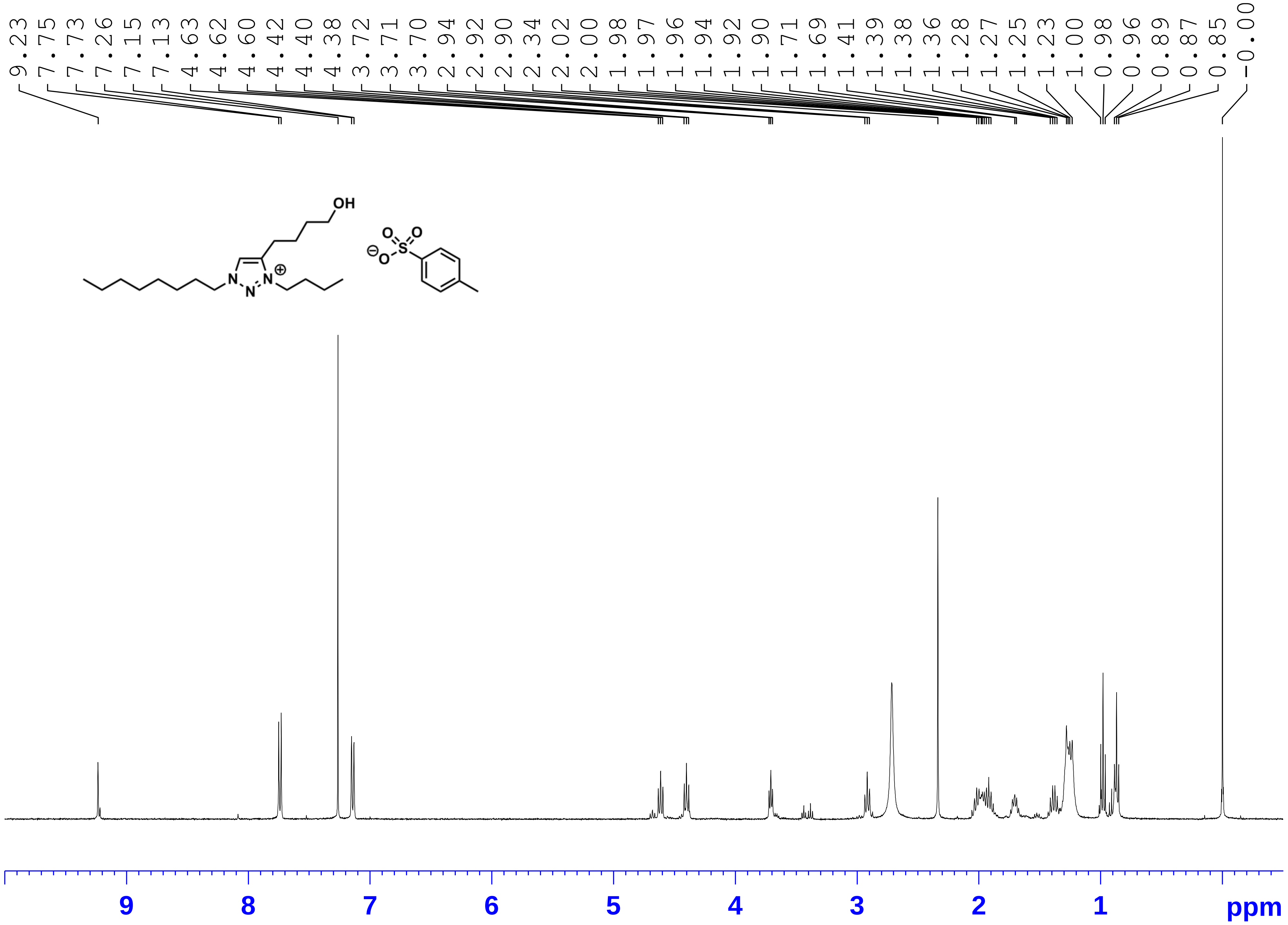

9.23
7.75
7.73
7.26
7.15
7.13
4.63
4.62
4.60
4.42
4.40
4.38
3.72
3.71
3.70
2.94
2.92
2.90
2.34
2.02
2.00
1.98
1.97
1.96
1.94
1.92
1.90
1.71
1.69
1.41
1.39
1.38
1.36
1.28
1.27
1.25
1.23
1.00
0.98
0.96
0.89
0.87
0.85
-0.00
OH
N
N
N
O
O
S
O
9
8
7
6
5
4
3
2
1
ppm

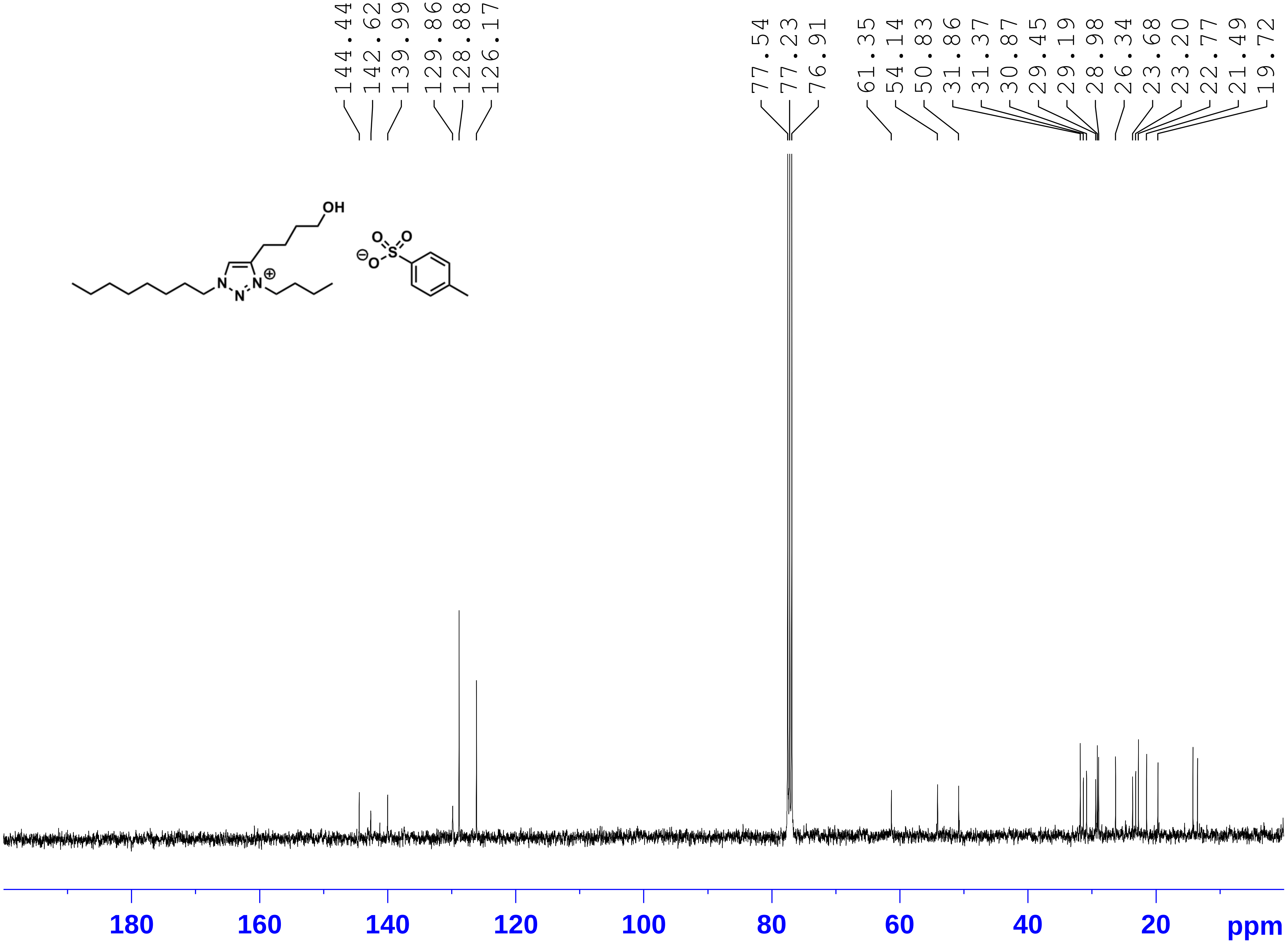
144.44
142.62
139.99
129.86
128.88
126.17
77.54
77.23
76.91
61.35
54.14
50.83
31.86
31.37
30.87
29.45
29.19
28.98
26.34
23.68
23.20
22.77
21.49
19.72
OH
180
160
140
120
100
80
60
40
20
ppm

Spectrum

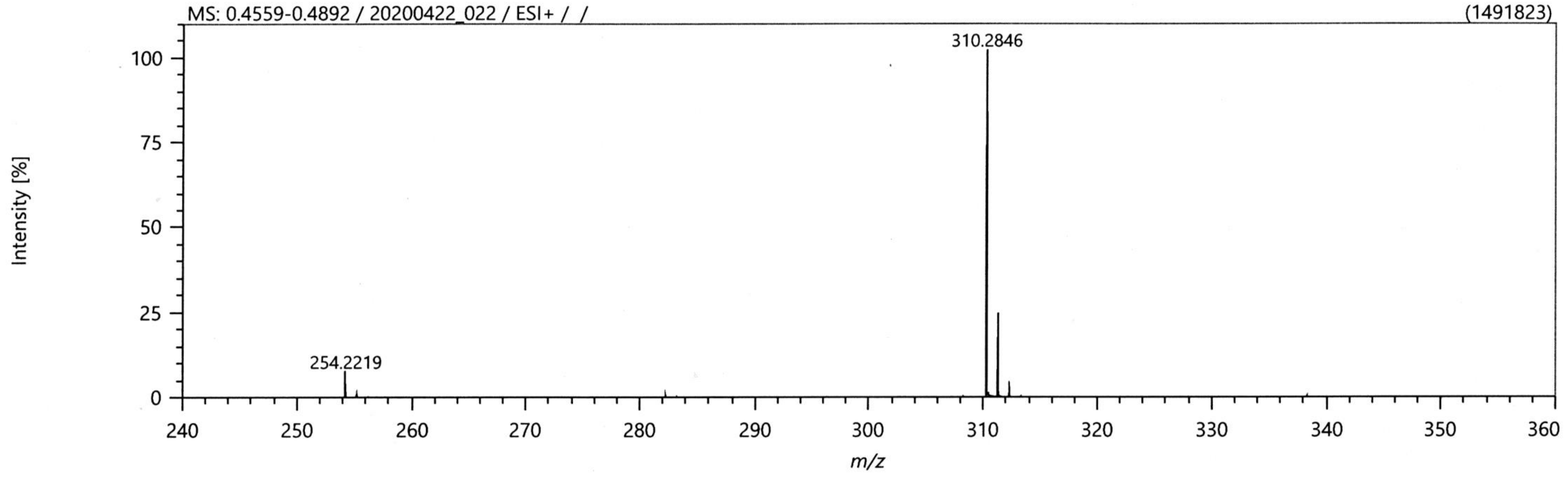

Elemental Composition

Parameters	
Tolerance:	±10.00 ppm
Electron:	Odd/Even
Charge:	+1
DBE:	-99.0 - 999.0

Elements Set 1:

Symbol	C	H	O	Na	N	F
Min	0	0	1	0	3	0
Max	1000	4000	1	0	3	0

Results

Mass	Formula	Calculated Mass	Mass Difference [mDa]	Mass Difference [ppm]	DBE
310.28464	C18 H36 N3 O	310.28529	-0.65	-2.09	2.5

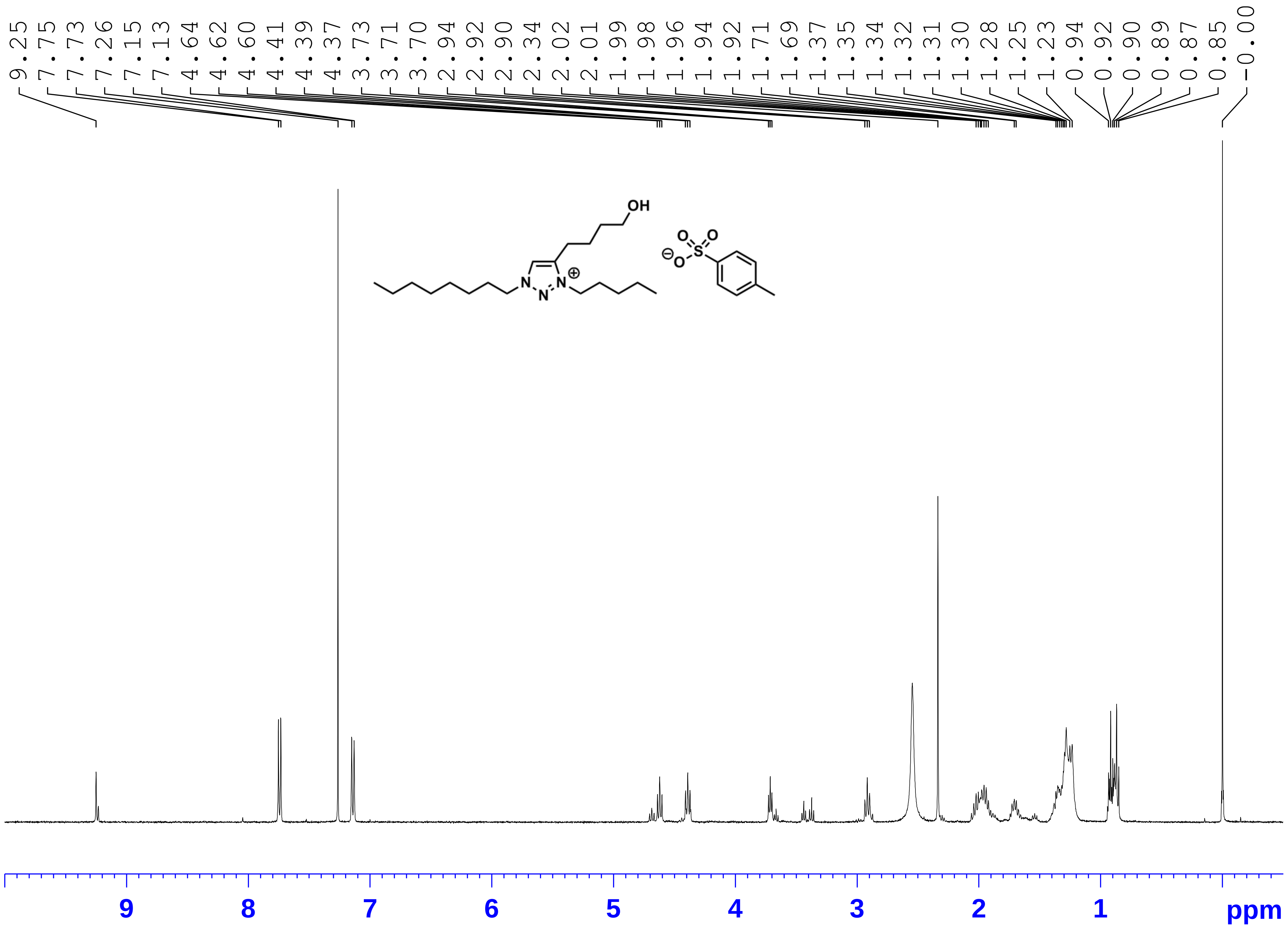
9.25
7.75
7.73
7.26
7.15
7.13
4.64
4.62
4.60
4.41
4.39
4.37
3.73
3.71
3.70
2.94
2.92
2.90
2.34
2.02
2.01
1.99
1.98
1.96
1.94
1.92
1.71
1.69
1.37
1.35
1.34
1.32
1.31
1.30
1.28
1.25
1.23
0.94
0.92
0.90
0.89
0.87
0.85
-0.00
OH
9
8
7
6
5
4
3
2
1
ppm

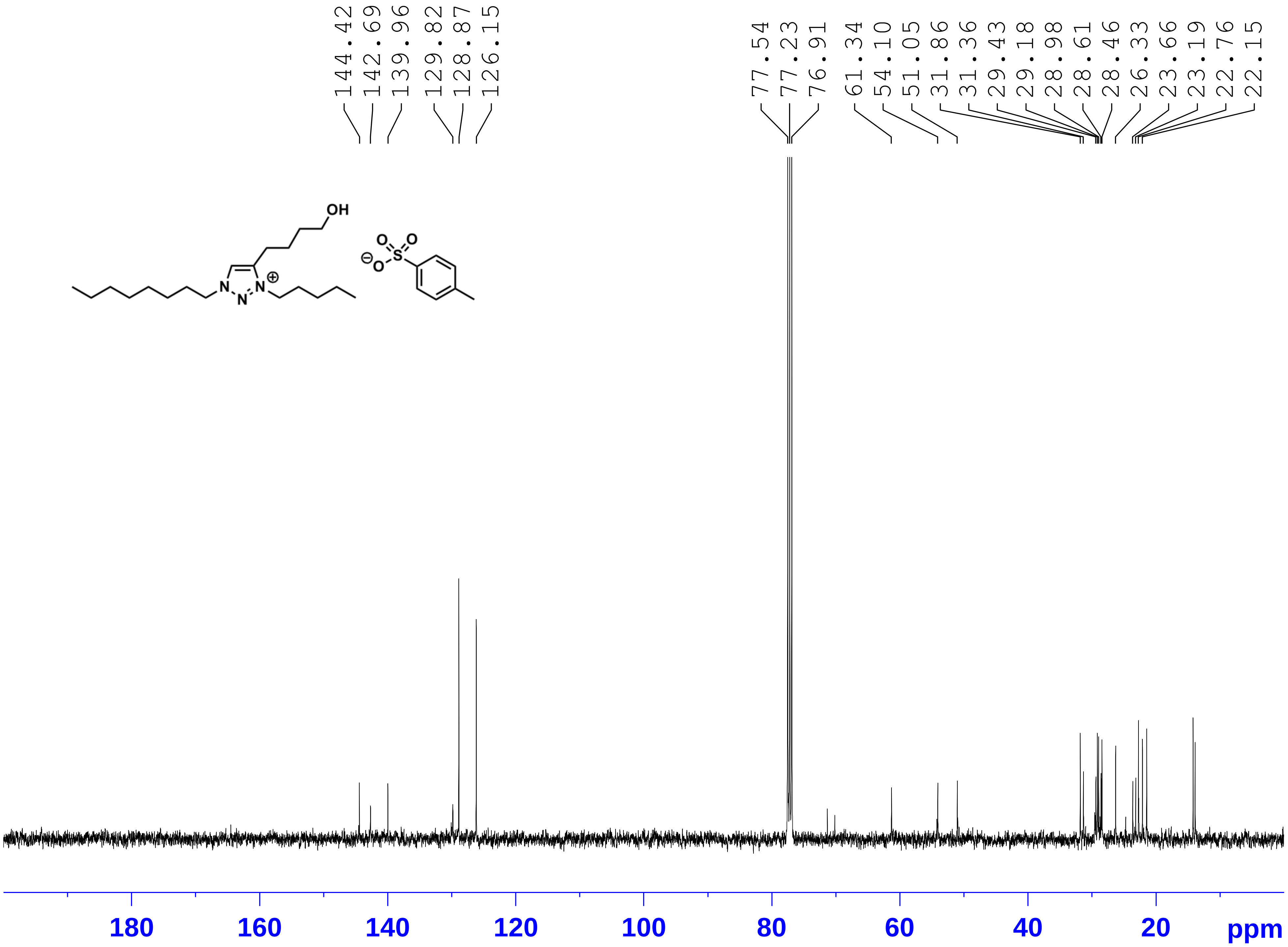

144.42
142.69
139.96
129.82
128.87
126.15
77.54
77.23
76.91
61.34
54.10
51.05
31.86
31.36
29.43
29.18
28.98
28.61
28.46
26.33
23.66
23.19
22.76
22.15
OH
N
N
N
O
O
S
O
180
160
140
120
100
80
60
40
20
ppm

Spectrum

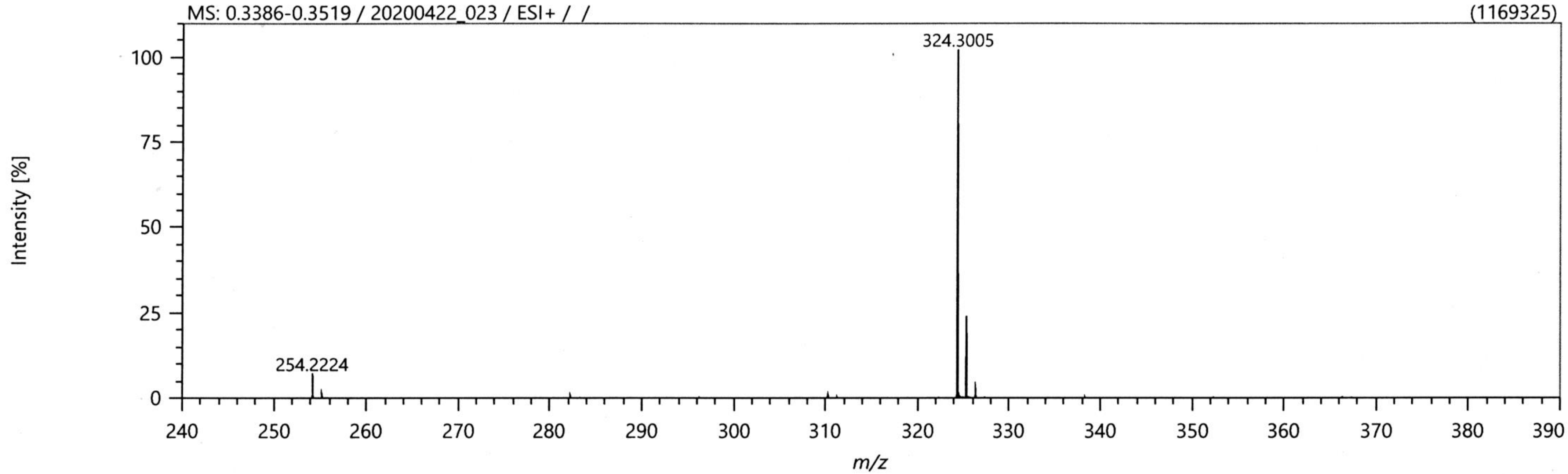

Elemental Composition

Parameters	
Tolerance:	±10.00 ppm
Electron:	Odd/Even
Charge:	+1
DBE:	-99.0 - 999.0

Elements Set 1:

Symbol	C	H	O	Na	N	F
Min	0	0	1	0	3	0
Max	1000	4000	1	0	3	0

Results

Mass	Formula	Calculated Mass	Mass Difference [mDa]	Mass Difference [ppm]	DBE
324.30049	C19 H38 N3 O	324.30094	-0.45	-1.38	2.5

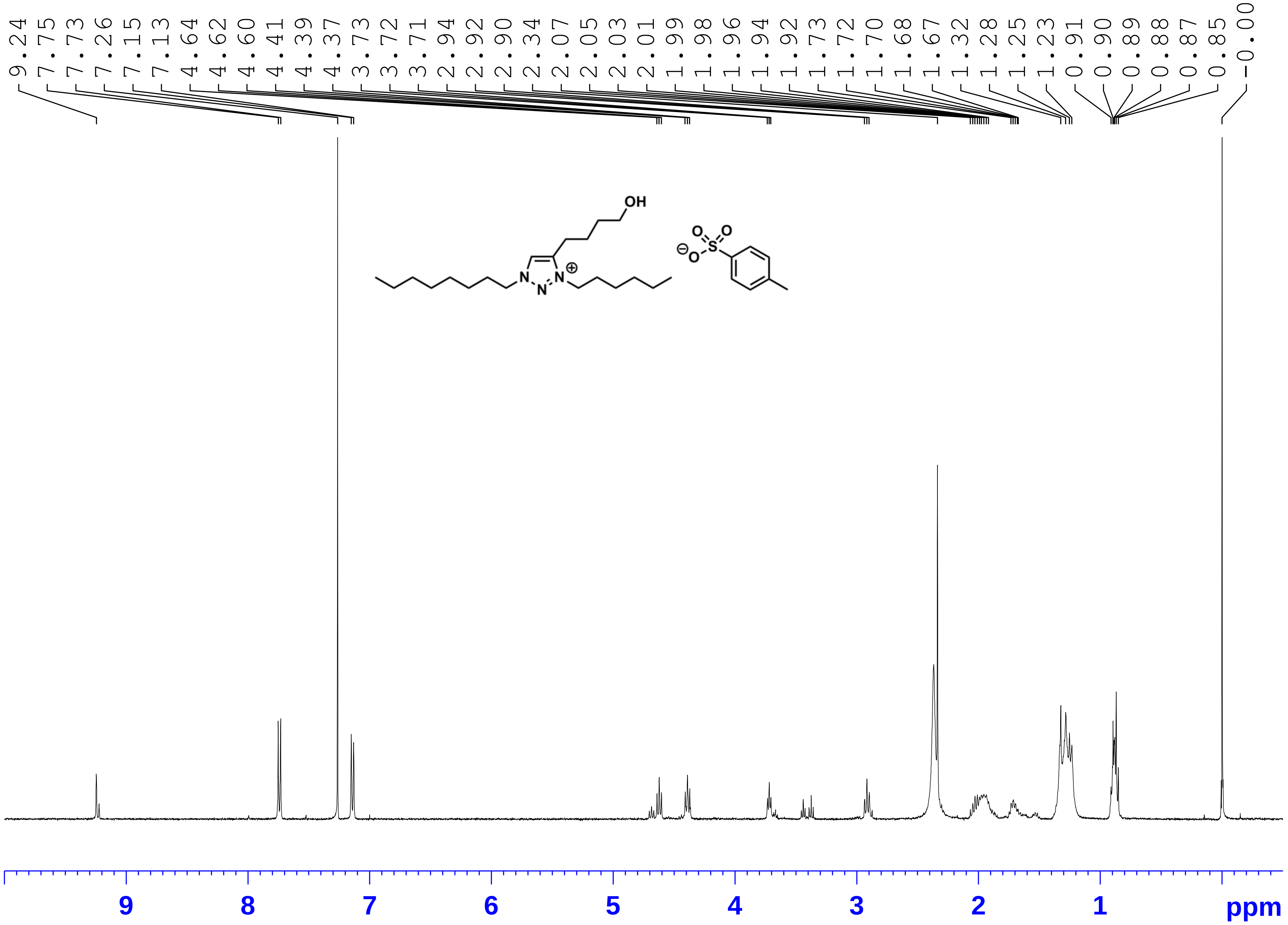

9.24
7.75
7.73
7.26
7.15
7.13
4.64
4.62
4.60
4.41
4.39
4.37
3.73
3.72
3.71
2.94
2.92
2.90
2.34
2.07
2.05
2.03
2.01
1.99
1.98
1.96
1.94
1.92
1.73
1.72
1.70
1.68
1.67
1.32
1.28
1.25
1.23
0.91
0.90
0.89
0.88
0.87
0.85
-0.00
OH
9
8
7
6
5
4
3
2
1
ppm

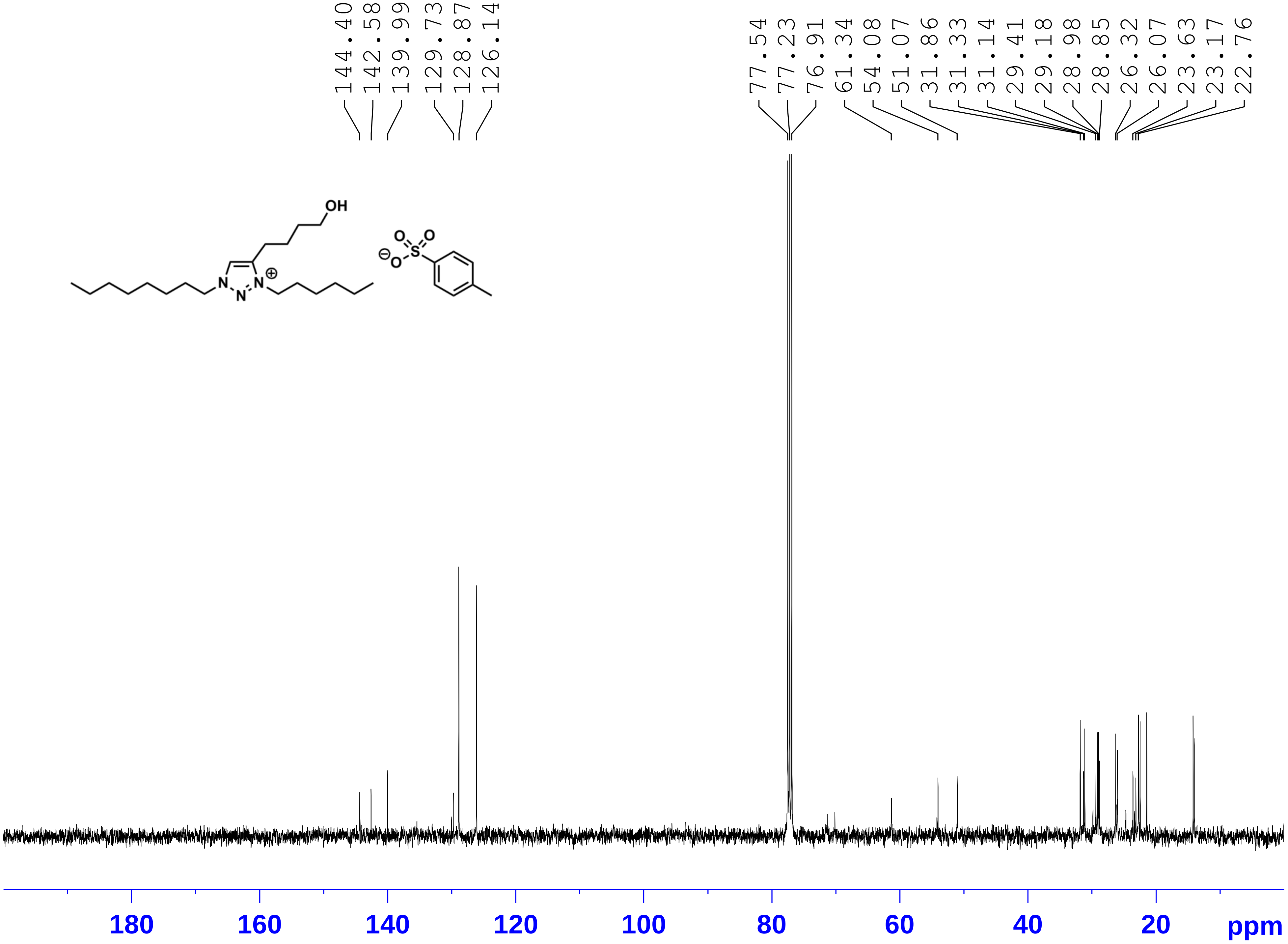
144.40
142.58
139.99
129.73
128.87
126.14
77.54
77.23
76.91
61.34
54.08
51.07
31.86
31.33
31.14
29.41
29.18
28.98
28.85
26.32
26.07
23.63
23.17
22.76
OH
N
N
N
O
S
O
O
180
160
140
120
100
80
60
40
20
ppm

Spectrum

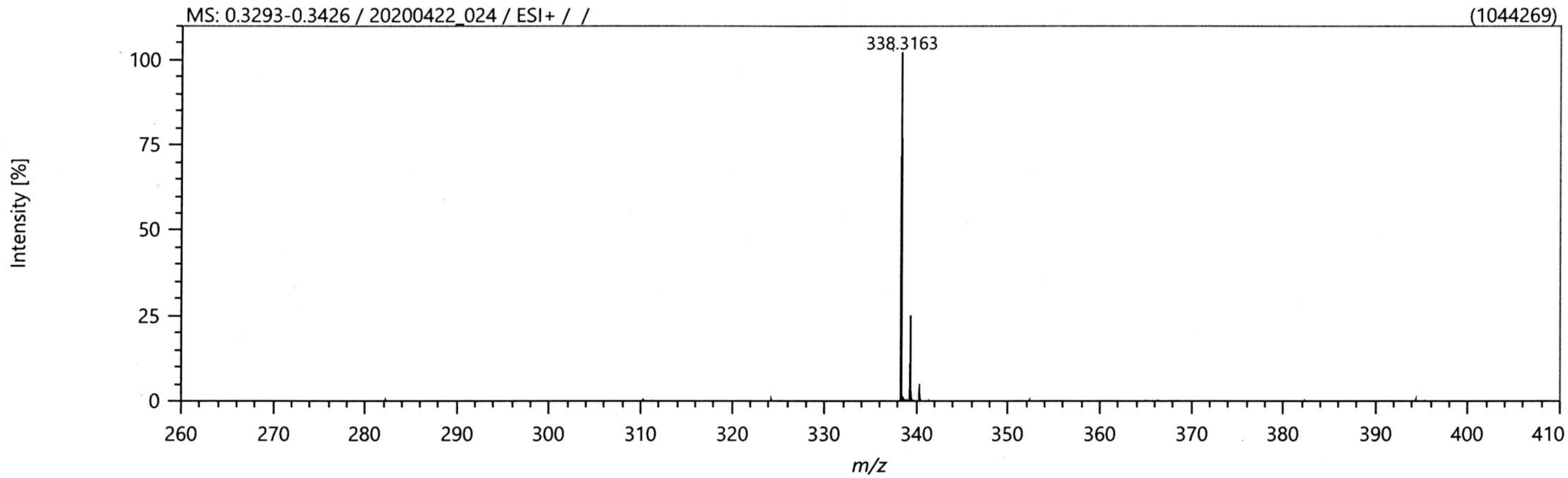

Elemental Composition

Parameters		Elements Set 1:						
Tolerance:	±10.00 ppm	Symbol	C	H	O	Na	N	F
Electron:	Odd/Even	Min	0	0	1	0	3	0
Charge:	+1	Max	1000	4000	1	0	3	0
DBE:	-99.0 - 999.0							

Results

Mass	Formula	Calculated Mass	Mass Difference [mDa]	Mass Difference [ppm]	DBE
338.31627	C20 H40 N3 O	338.31659	-0.32	-0.93	2.5

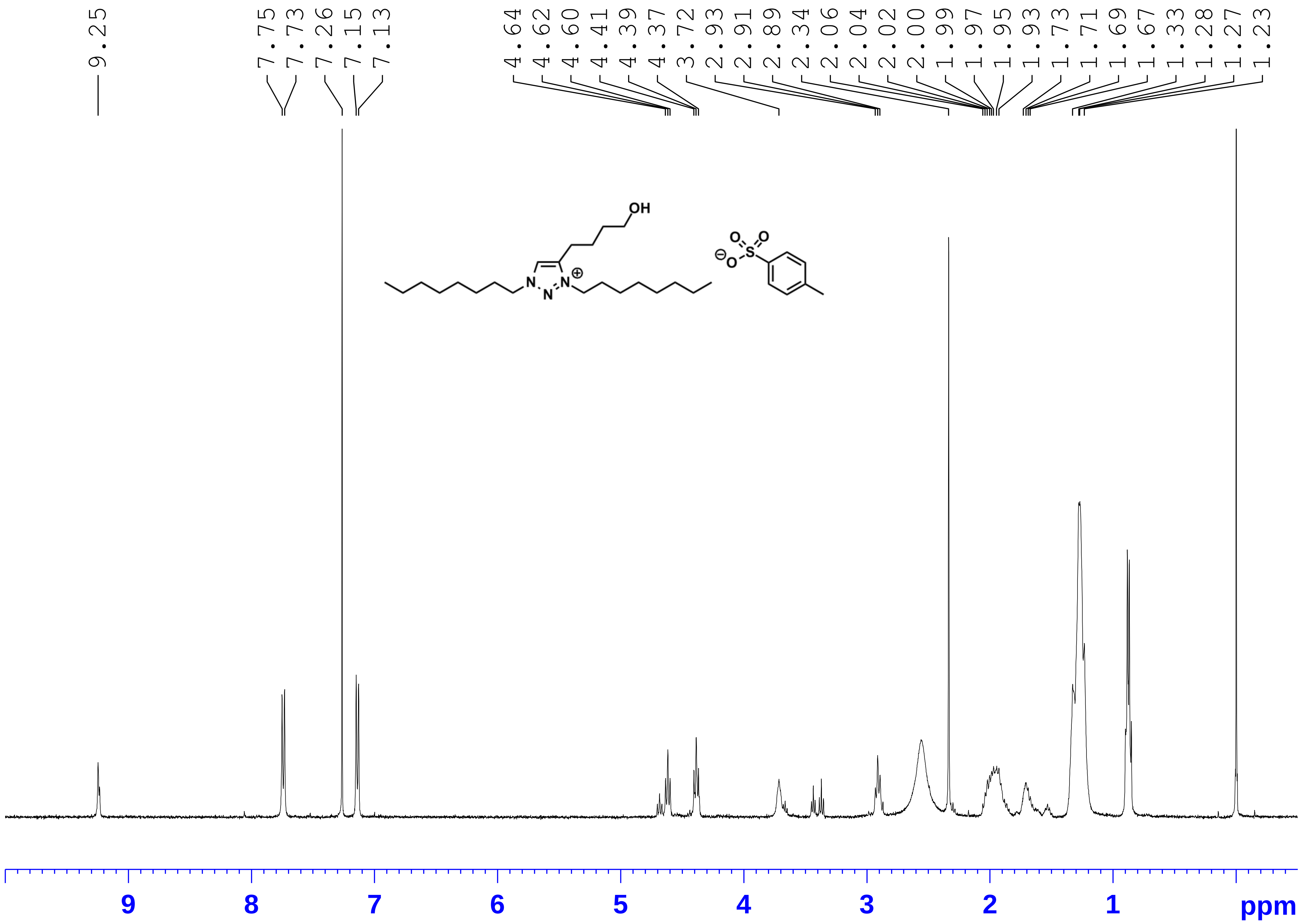
9.25
7.75
7.73
7.26
7.15
7.13
4.64
4.62
4.60
4.41
4.39
4.37
3.72
2.93
2.91
2.89
2.34
2.06
2.04
2.02
2.00
1.99
1.97
1.95
1.93
1.73
1.71
1.69
1.67
1.33
1.28
1.27
1.23
OH
N
N
N
S
O
O
O
9
8
7
6
5
4
3
2
1
ppm

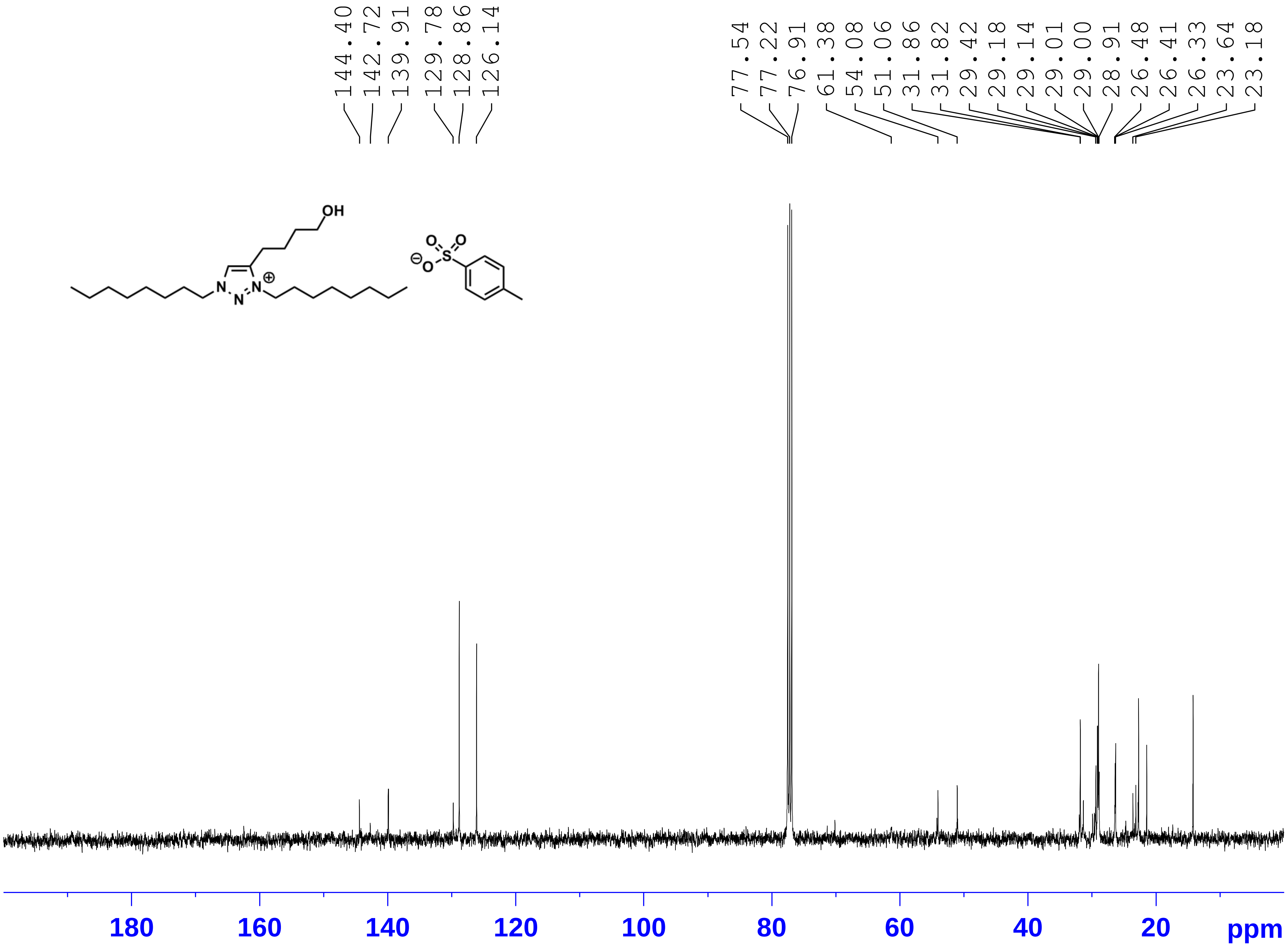
144.40
142.72
139.91
129.78
128.86
126.14
77.54
77.22
76.91
61.38
54.08
51.06
31.86
31.82
29.42
29.18
29.14
29.01
29.00
28.91
26.48
26.41
26.33
23.64
23.18
OH
N
N
N
O
O
S
O
180
160
140
120
100
80
60
40
20
ppm

Spectrum

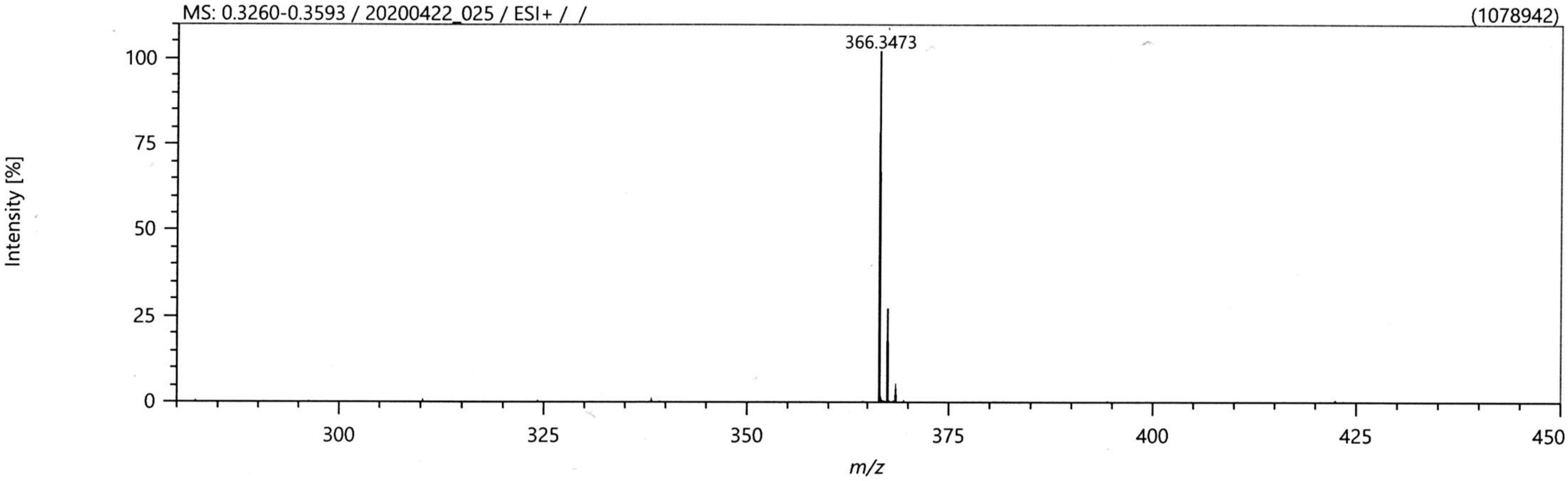

Elemental Composition

Parameters		Elements Set 1:						
Tolerance:	±10.00 ppm	Symbol	C	H	O	Na	N	F
Electron:	Odd/Even	Min	0	0	1	0	3	0
Charge:	+1	Max	1000	4000	1	0	3	0
DBE:	-99.0 - 999.0							

Results

Mass	Formula	Calculated Mass	Mass Difference [mDa]	Mass Difference [ppm]	DBE
366.34730	C22 H44 N3 O	366.34789	-0.59	-1.61	2.5

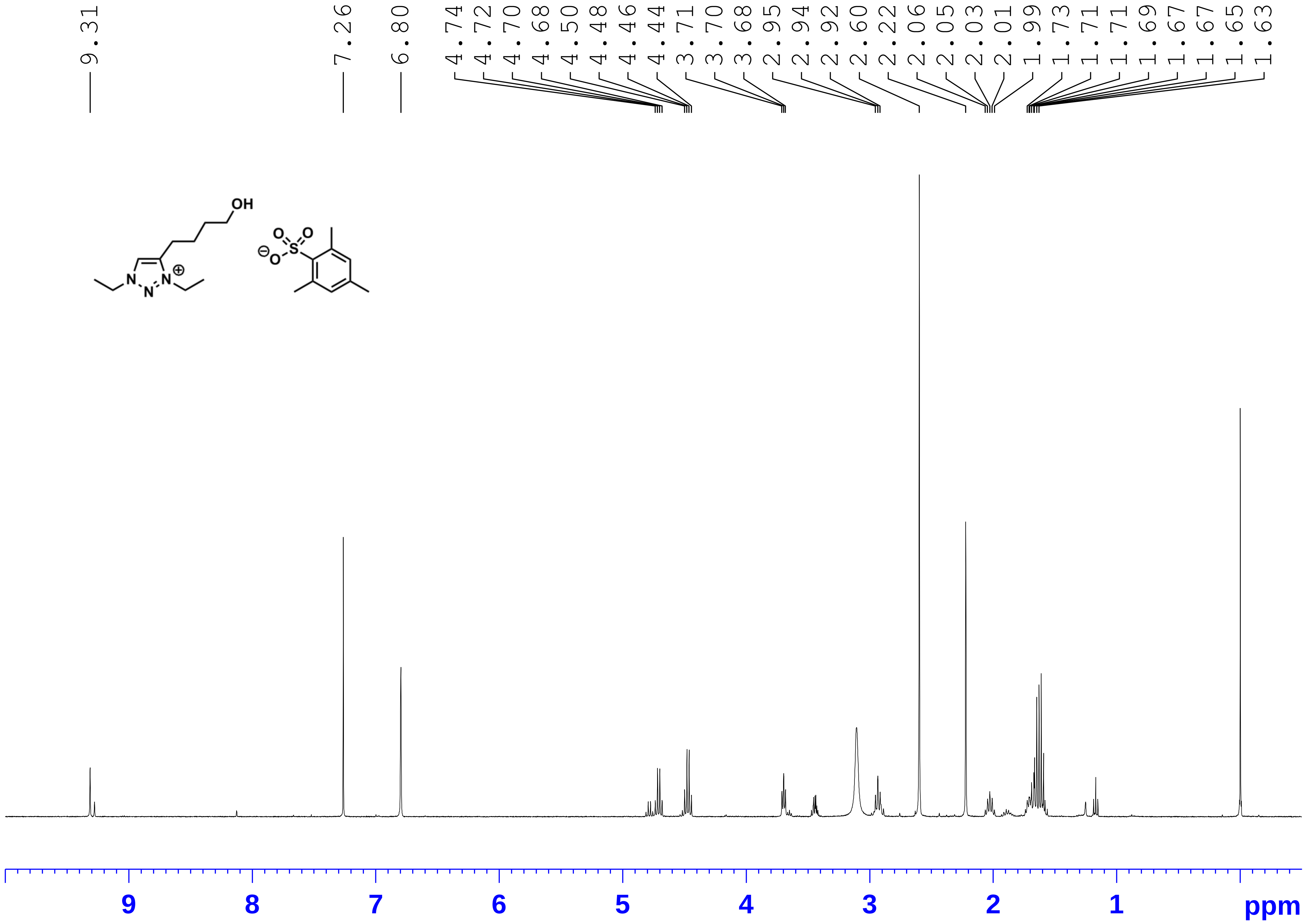
9.31
7.26
6.80
4.74
4.72
4.70
4.68
4.50
4.48
4.46
4.44
3.71
3.70
3.68
2.95
2.94
2.92
2.60
2.22
2.06
2.05
2.03
2.01
1.99
1.73
1.71
1.71
1.69
1.67
1.67
1.65
1.63
OH
N
N
N
O
O
O
S
9
8
7
6
5
4
3
2
1
ppm

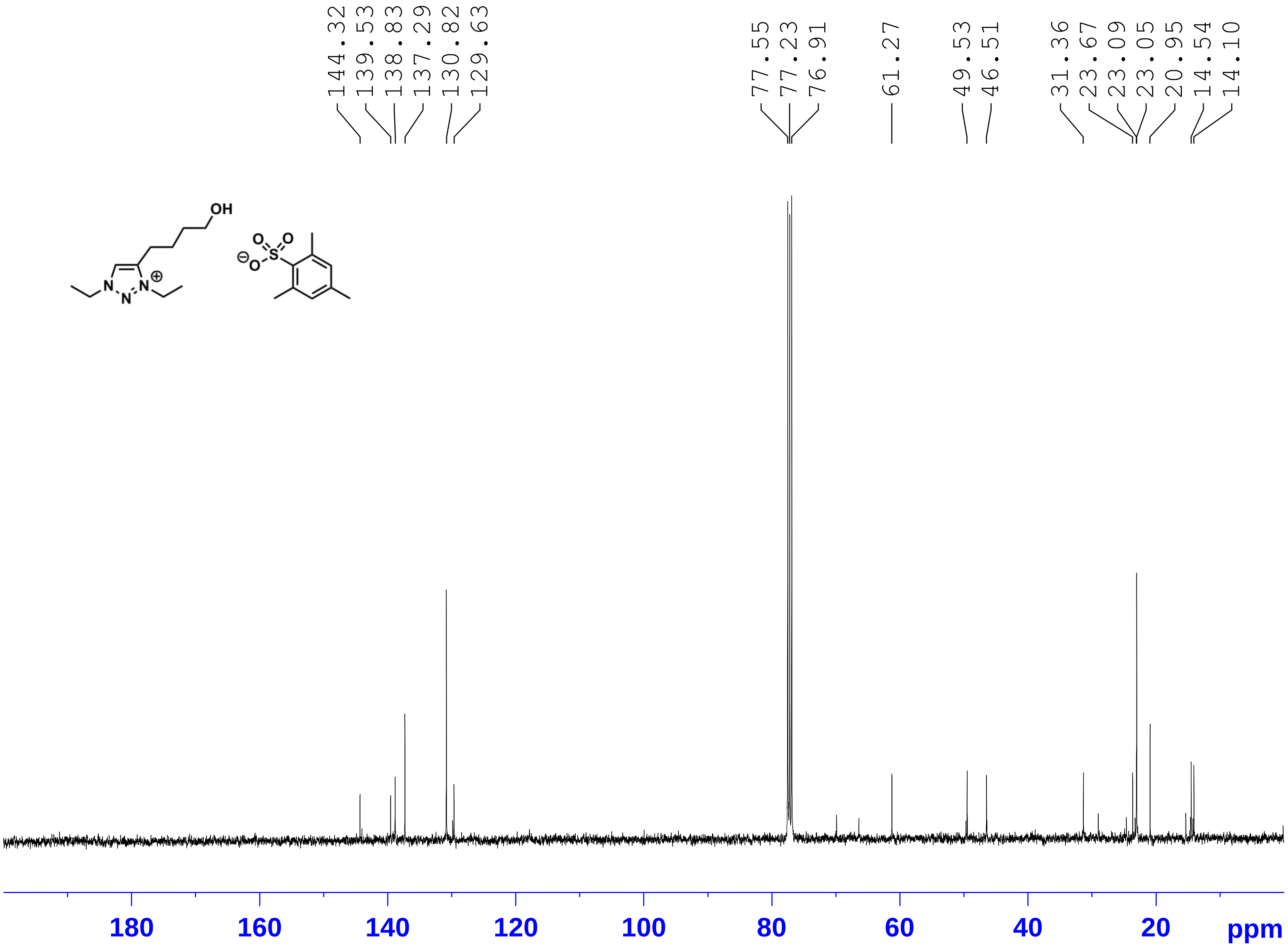

144.32
139.53
138.83
137.29
130.82
129.63
77.55
77.23
76.91
61.27
49.53
46.51
31.36
23.67
23.09
23.05
20.95
14.54
14.10
OH
O
S
N
180
160
140
120
100
80
60
40
20
ppm

Spectrum

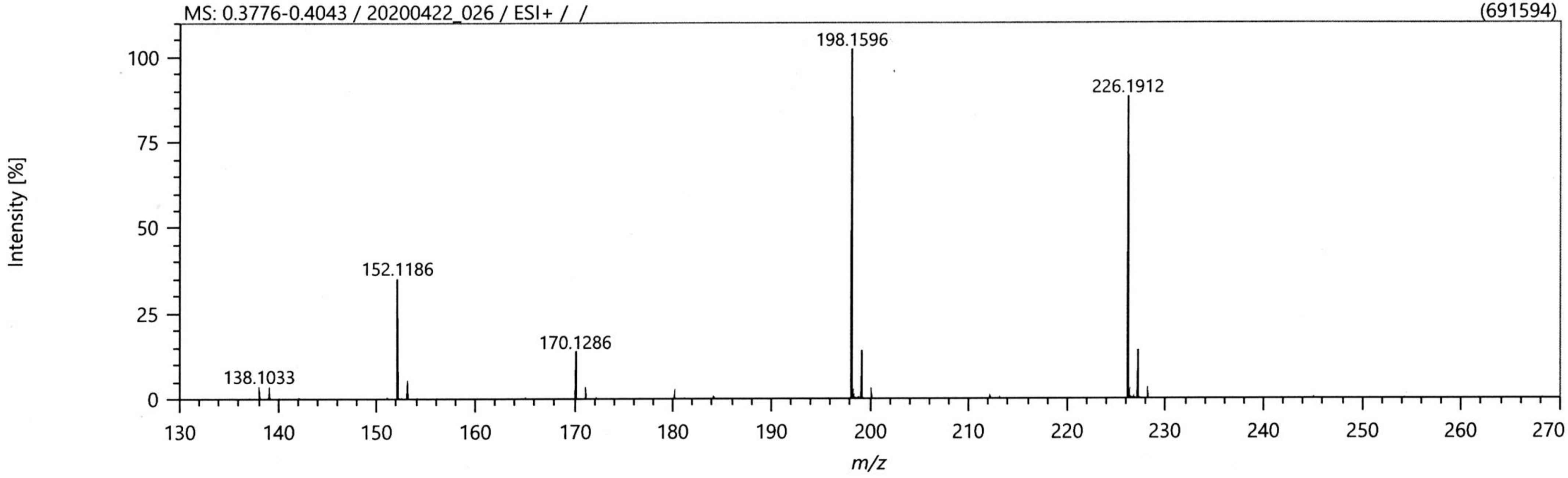

Elemental Composition

Parameters

Tolerance:	±10.00 ppm	Symbol	C	H	O	Na	N	F
Electron:	Odd/Even	Min	0	0	1	0	3	0
Charge:	+1	Max	1000	4000	1	0	3	0
DBE:	-99.0 - 999.0							

Elements Set 1:

Results

Mass	Formula	Calculated Mass	Mass Difference [mDa]	Mass Difference [ppm]	DBE
198.15956	C10 H20 N3 O	198.16009	-0.53	-2.69	2.5

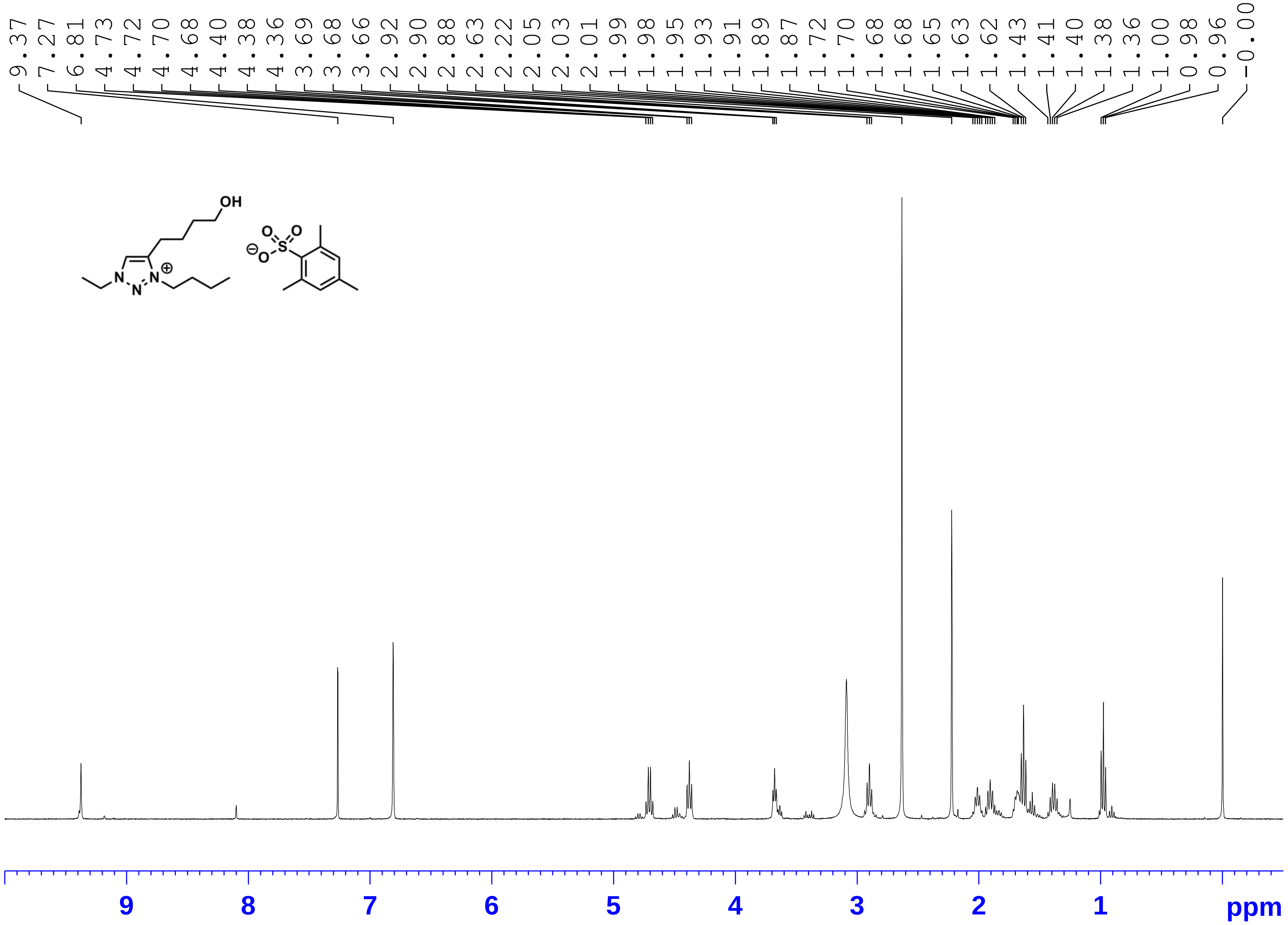
9.37
7.27
6.81
4.73
4.72
4.70
4.68
4.40
4.38
4.36
3.69
3.68
3.66
2.92
2.90
2.88
2.63
2.22
2.05
2.03
2.01
1.99
1.98
1.95
1.93
1.91
1.89
1.87
1.72
1.70
1.68
1.68
1.65
1.63
1.62
1.43
1.41
1.40
1.38
1.36
1.00
0.98
0.96
-0.00
OH
N
N
N
O
O
S
O
9
8
7
6
5
4
3
2
1
ppm

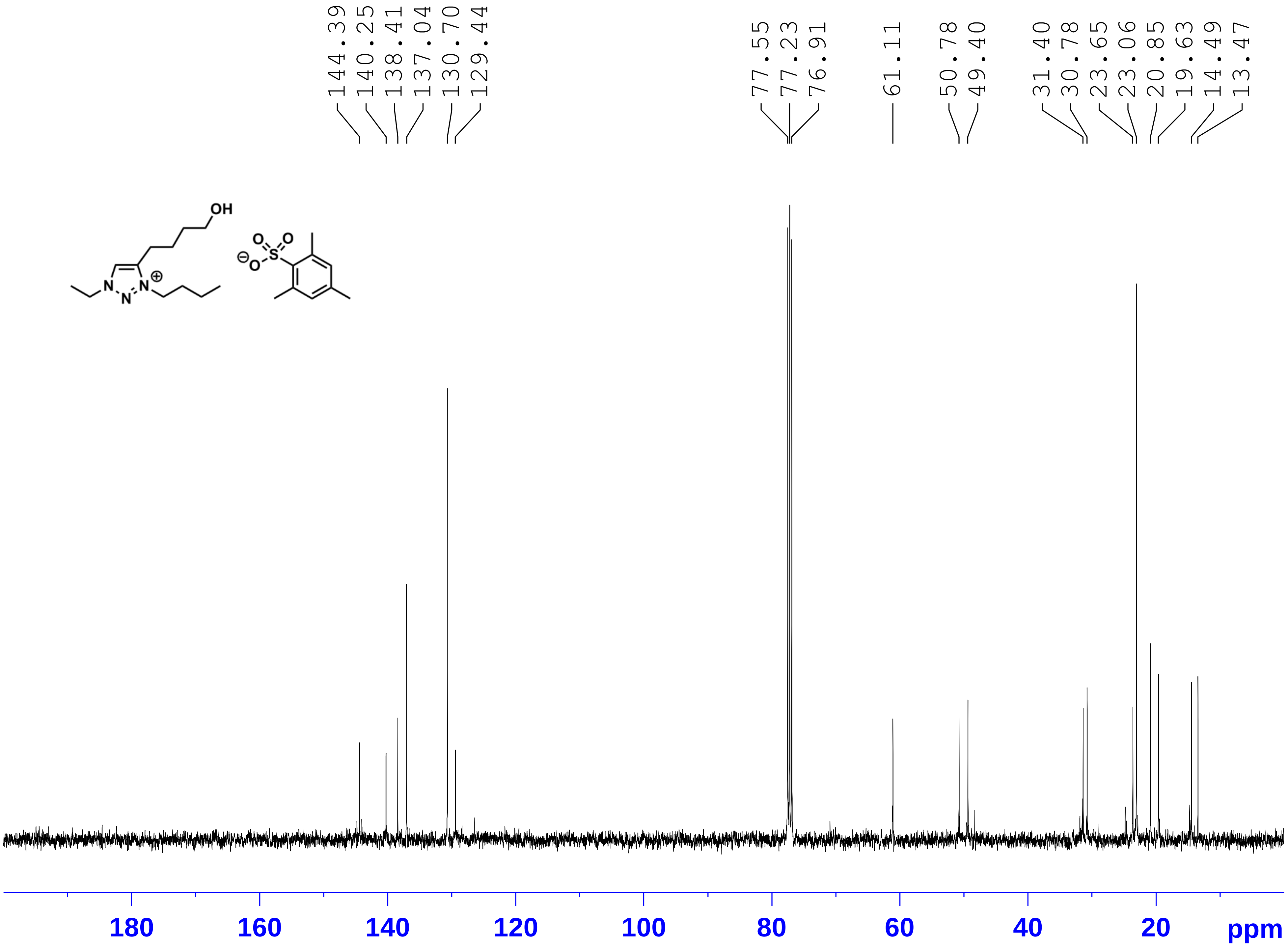
144.39
140.25
138.41
137.04
130.70
129.44
77.55
77.23
76.91
61.11
50.78
49.40
31.40
30.78
23.65
23.06
20.85
19.63
14.49
13.47
OH
180
160
140
120
100
80
60
40
20
ppm

Spectrum

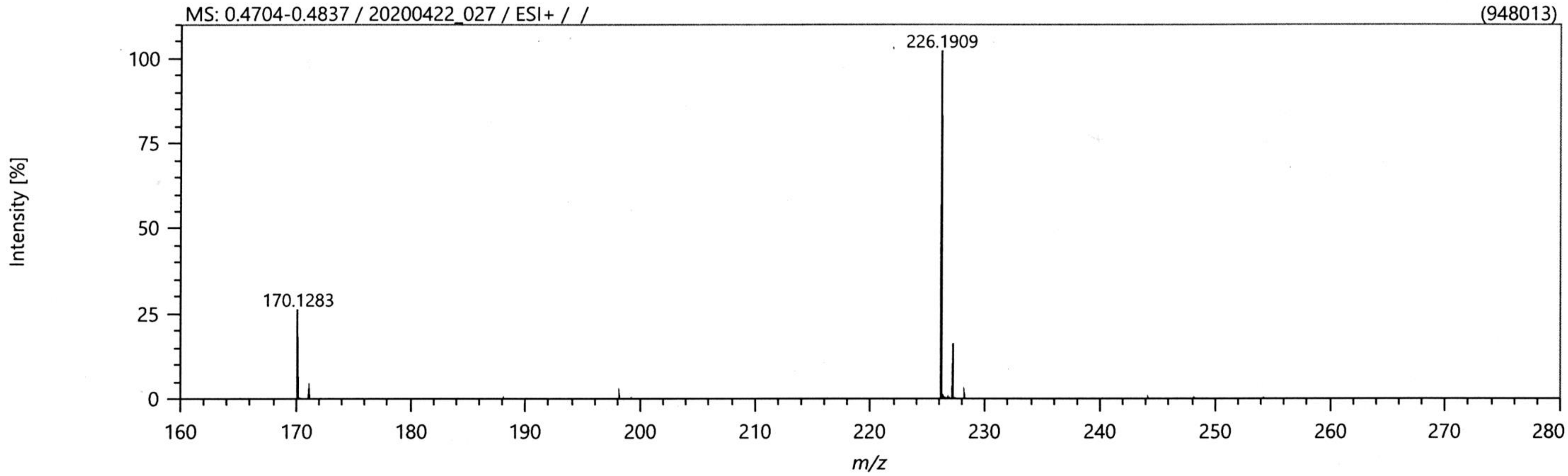

Elemental Composition

Parameters		Elements Set 1:						
Tolerance:	±10.00 ppm	Symbol	C	H	O	Na	N	F
Electron:	Odd/Even	Min	0	0	1	0	3	0
Charge:	+1	Max	1000	4000	1	0	3	0
DBE:	-99.0 - 999.0							

Results

Mass	Formula	Calculated Mass	Mass Difference [mDa]	Mass Difference [ppm]	DBE
226.19090	C12 H24 N3 O	226.19139	-0.49	-2.17	2.5

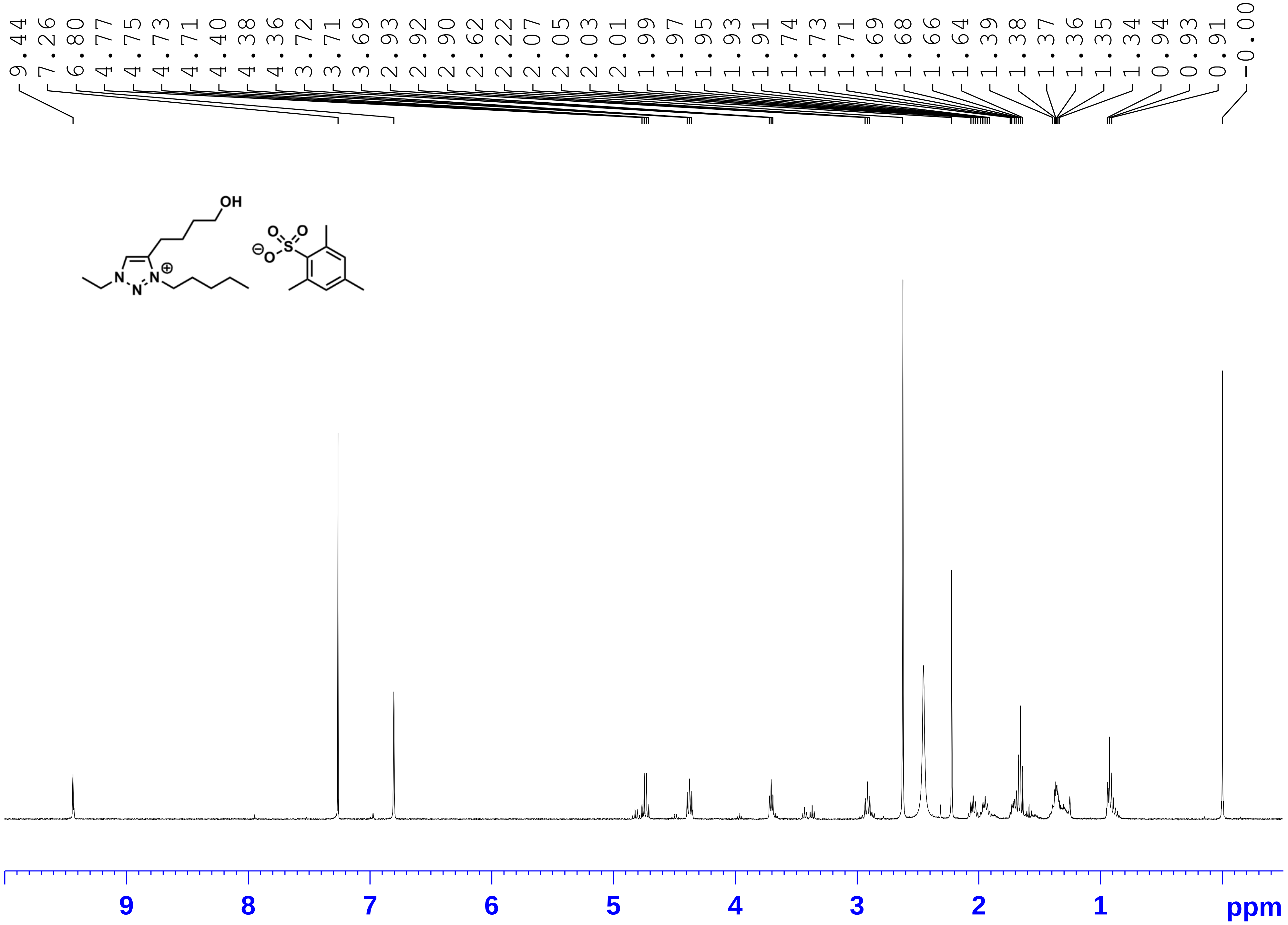

9.44
7.26
6.80
4.77
4.75
4.73
4.71
4.40
4.38
4.36
3.72
3.71
3.69
2.93
2.92
2.90
2.62
2.22
2.07
2.05
2.03
2.01
1.99
1.97
1.95
1.93
1.91
1.74
1.73
1.71
1.69
1.68
1.66
1.64
1.39
1.38
1.37
1.36
1.35
1.34
0.94
0.93
0.91
-0.00
OH
9
8
7
6
5
4
3
2
1
ppm

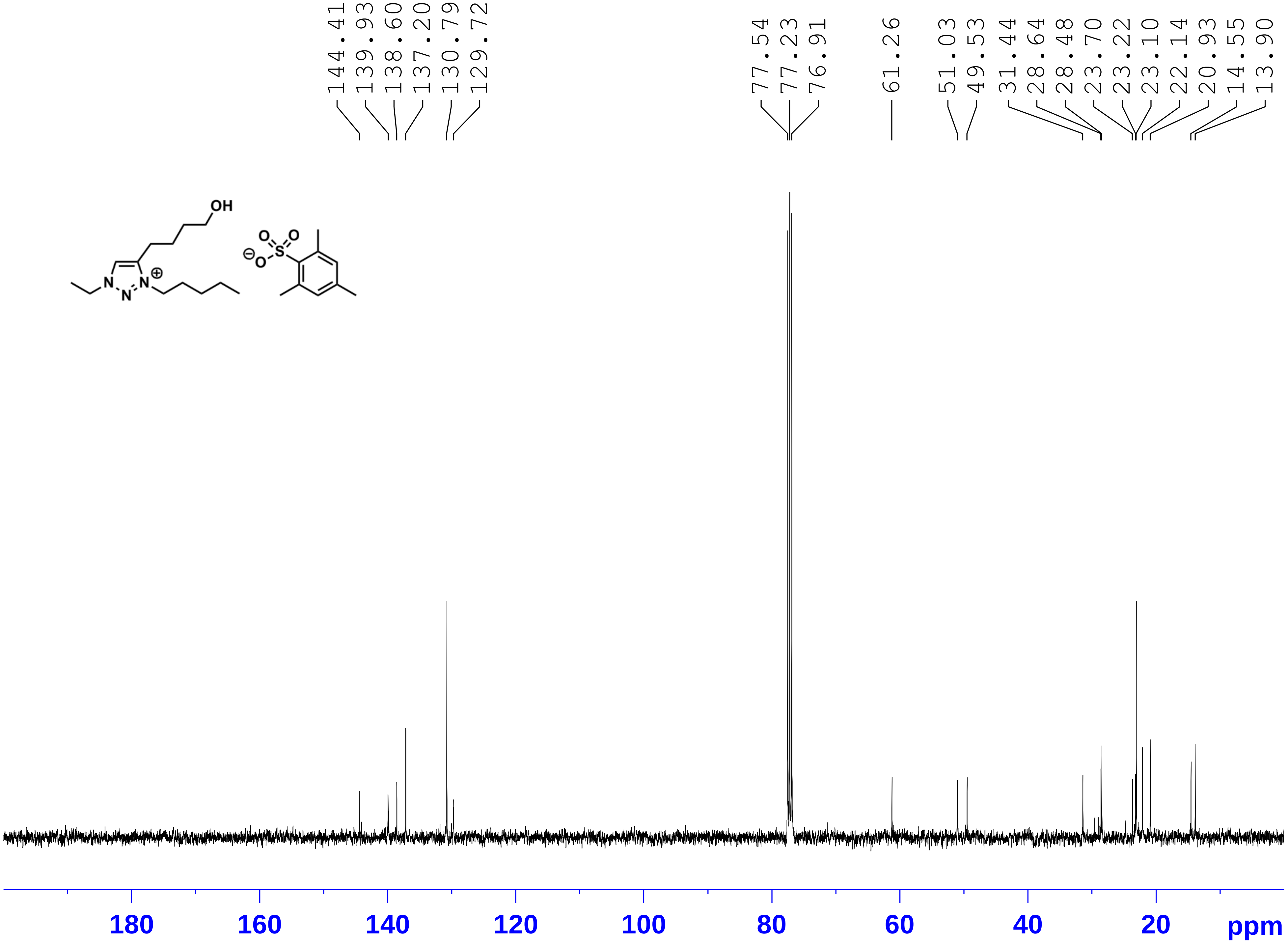
144.41
139.93
138.60
137.20
130.79
129.72
77.54
77.23
76.91
61.26
51.03
49.53
31.44
28.64
28.48
23.70
23.22
23.10
22.14
20.93
14.55
13.90
OH
O
S
N
180
160
140
120
100
80
60
40
20
ppm

Spectrum

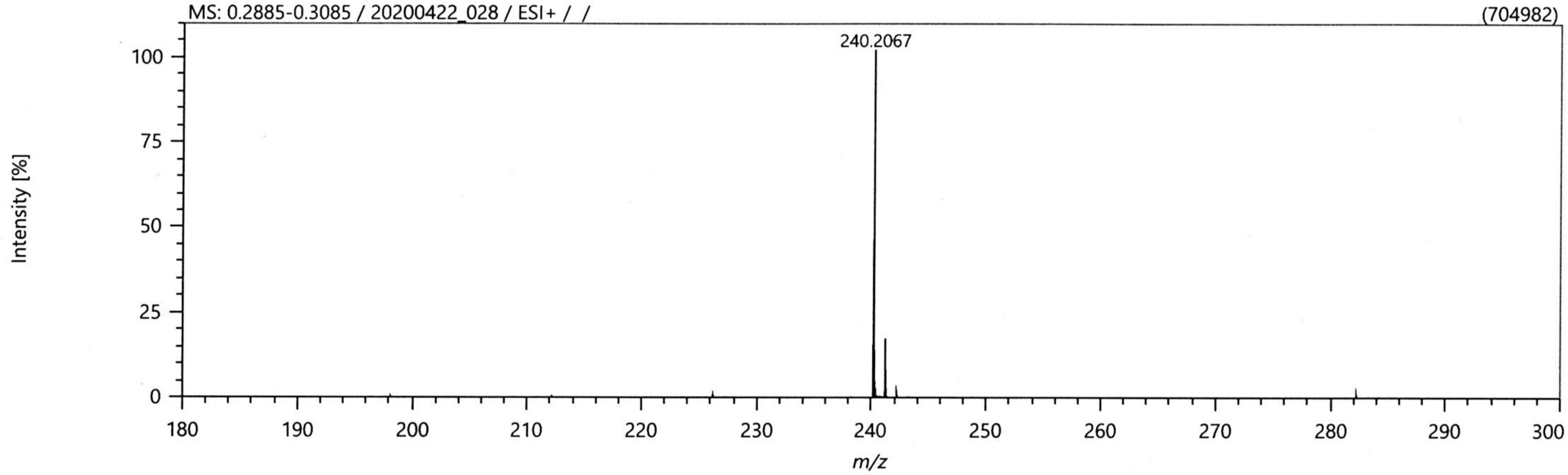

Elemental Composition

Parameters		Elements Set 1:						
Tolerance:	±10.00 ppm	Symbol	C	H	O	Na	N	F
Electron:	Odd/Even	Min	0	0	1	0	3	0
Charge:	+1	Max	1000	4000	1	0	3	0
DBE:	-99.0 - 999.0							

Results

Mass	Formula	Calculated Mass	Mass Difference [mDa]	Mass Difference [ppm]	DBE
240.20671	C13 H26 N3 O	240.20704	-0.33	-1.37	2.5

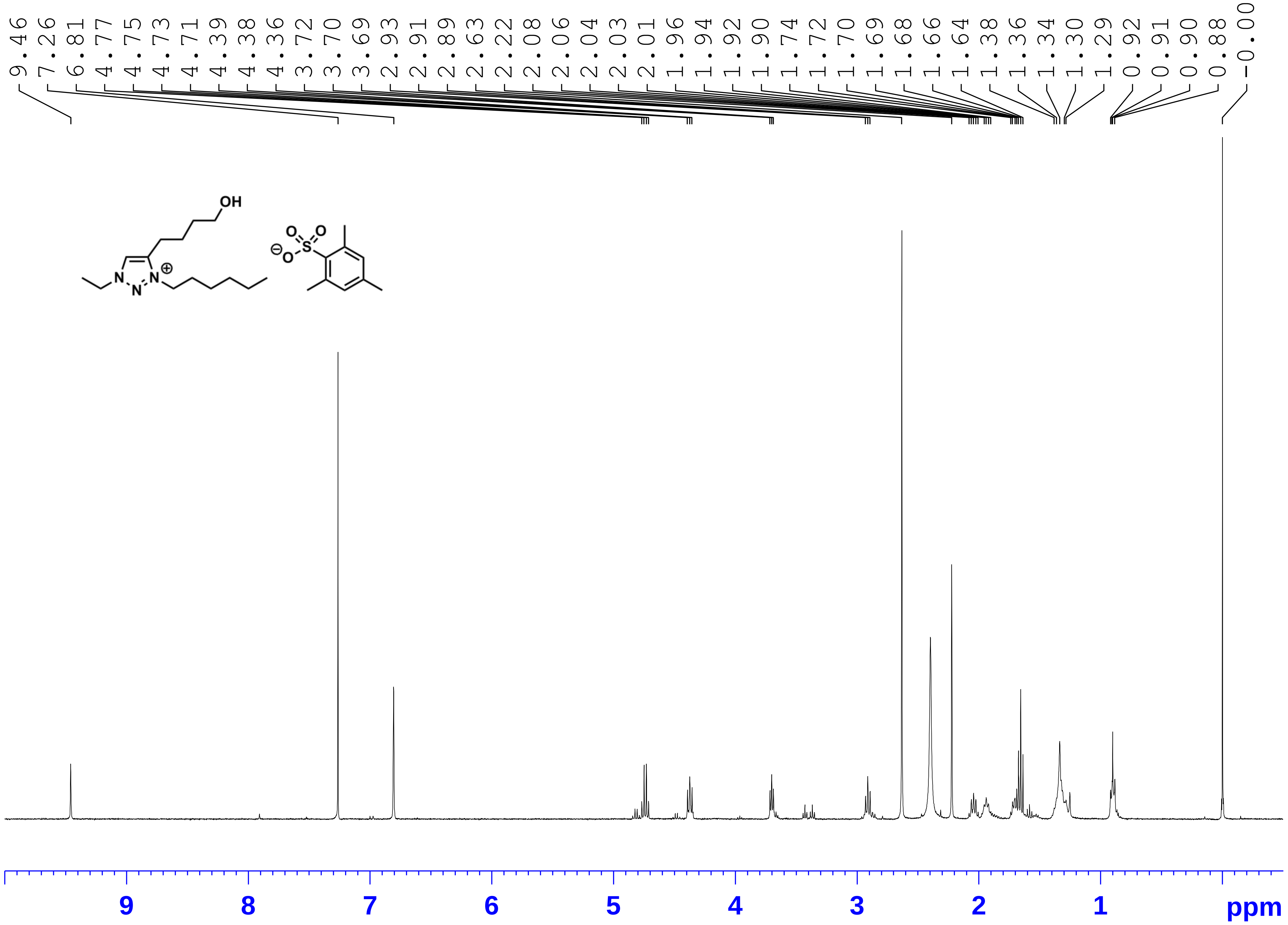
9.46
7.26
6.81
4.77
4.75
4.73
4.71
4.39
4.38
4.36
3.72
3.70
3.69
2.93
2.91
2.89
2.63
2.22
2.08
2.06
2.04
2.03
2.01
1.96
1.94
1.92
1.90
1.74
1.72
1.70
1.69
1.68
1.66
1.64
1.38
1.36
1.34
1.30
1.29
0.92
0.91
0.90
0.88
-0.00
OH
N
N
N
O
S
O
O
9
8
7
6
5
4
3
2
1
ppm

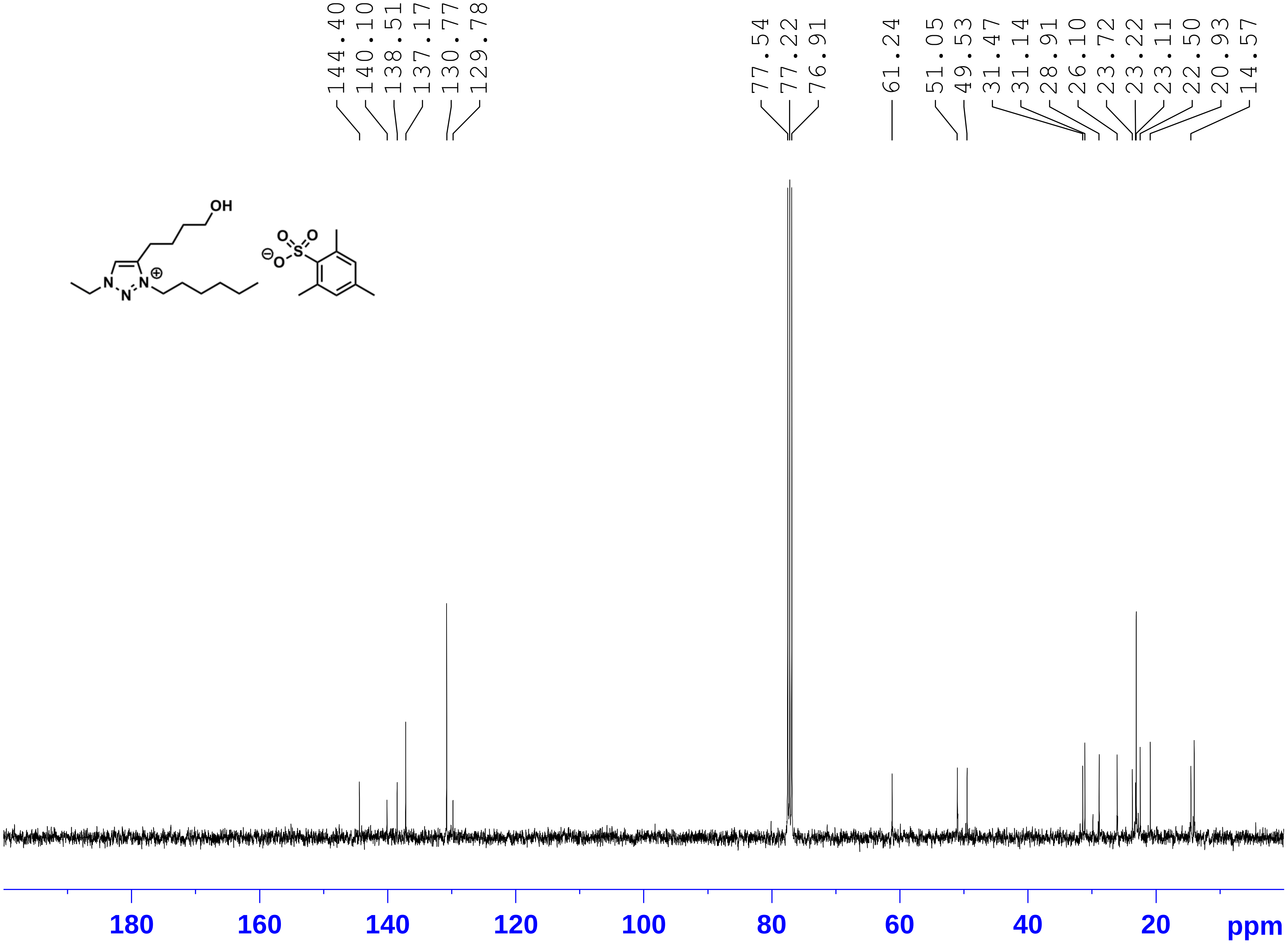

144.40
140.10
138.51
137.17
130.77
129.78
77.54
77.22
76.91
61.24
51.05
49.53
31.47
31.14
28.91
26.10
23.72
23.22
23.11
22.50
20.93
14.57
OH
180
160
140
120
100
80
60
40
20
ppm

Spectrum

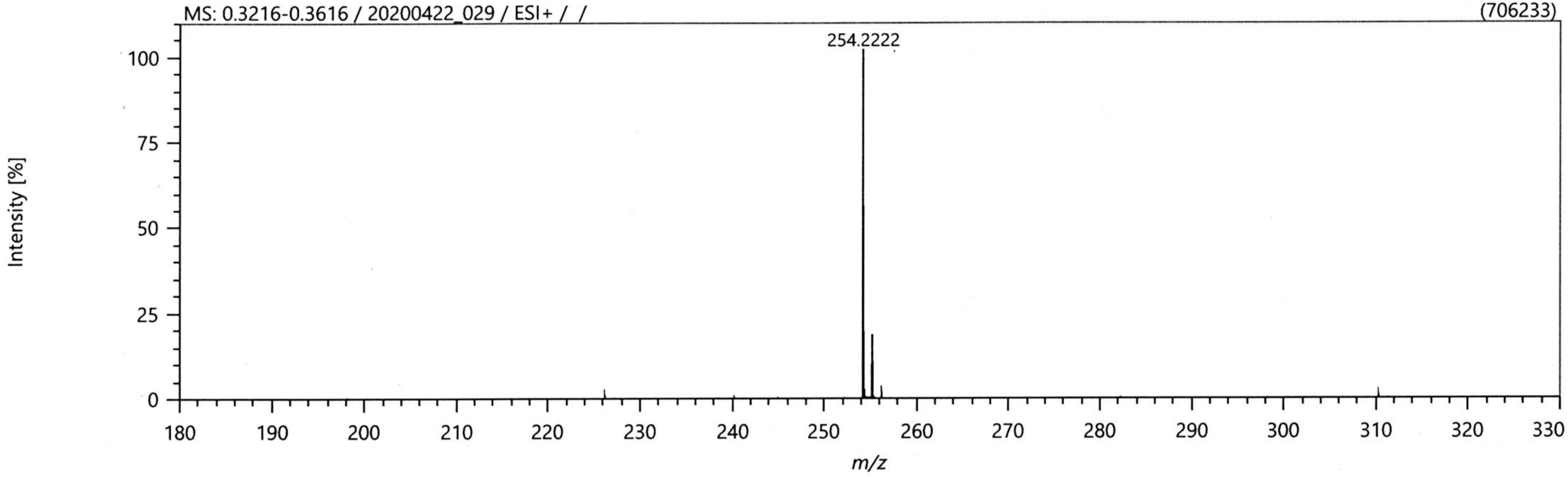

Elemental Composition

Parameters	
Tolerance:	±10.00 ppm
Electron:	Odd/Even
Charge:	+1
DBE:	-99.0 - 999.0

Elements Set 1:

Symbol	C	H	O	Na	N	F
Min	0	0	1	0	3	0
Max	1000	4000	1	0	3	0

Results

Mass	Formula	Calculated Mass	Mass Difference [mDa]	Mass Difference [ppm]	DBE
254.22221	C14 H28 N3 O	254.22269	-0.48	-1.87	2.5

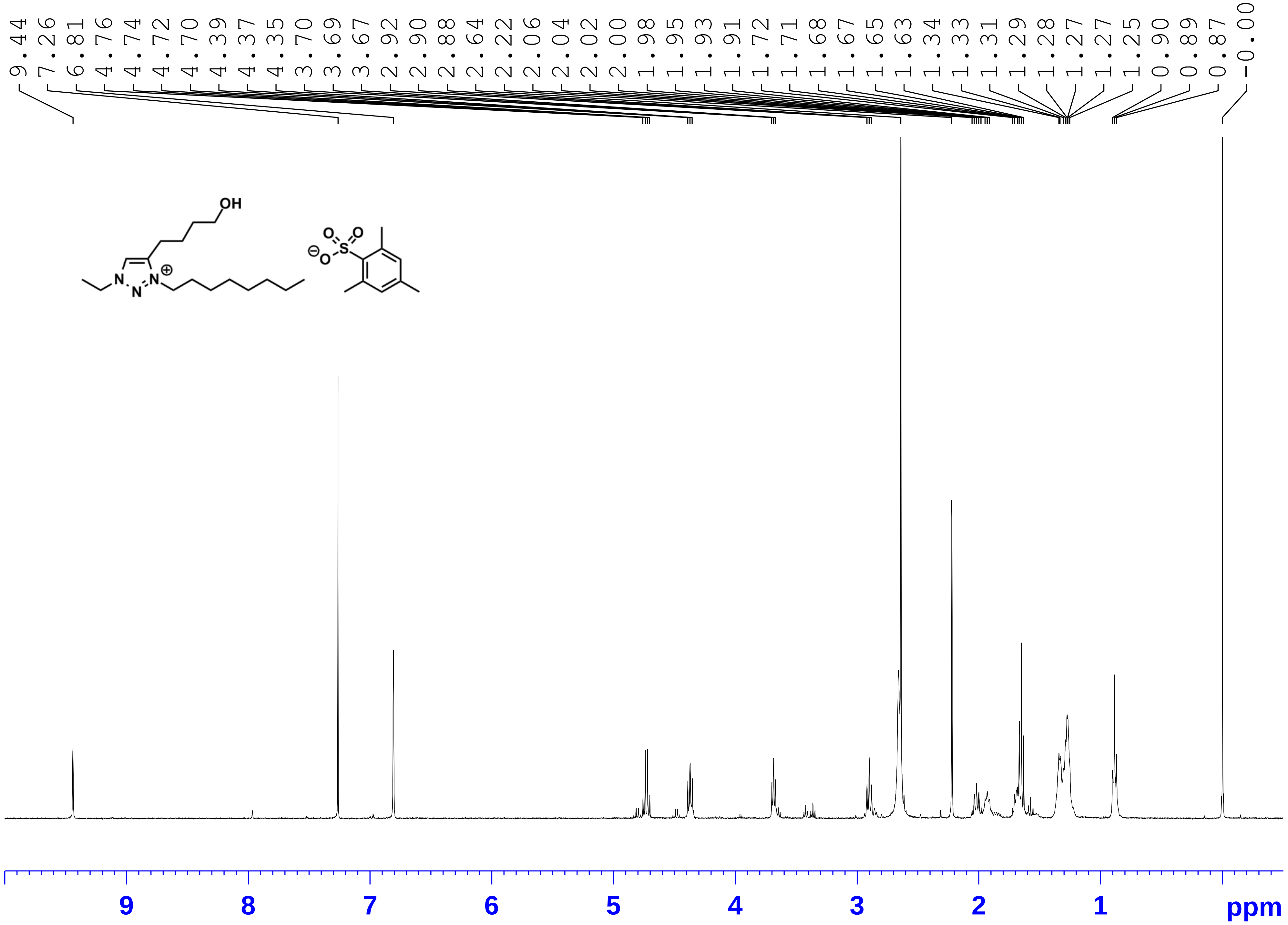

9.44
7.26
6.81
4.76
4.74
4.72
4.70
4.39
4.37
4.35
3.70
3.69
3.67
2.92
2.90
2.88
2.64
2.22
2.06
2.04
2.02
2.00
1.98
1.95
1.93
1.91
1.72
1.71
1.68
1.67
1.65
1.63
1.34
1.33
1.31
1.29
1.28
1.27
1.27
1.25
0.90
0.89
0.87
-0.00
OH
O
O
S
O
N
N
N
9
8
7
6
5
4
3
2
1
ppm

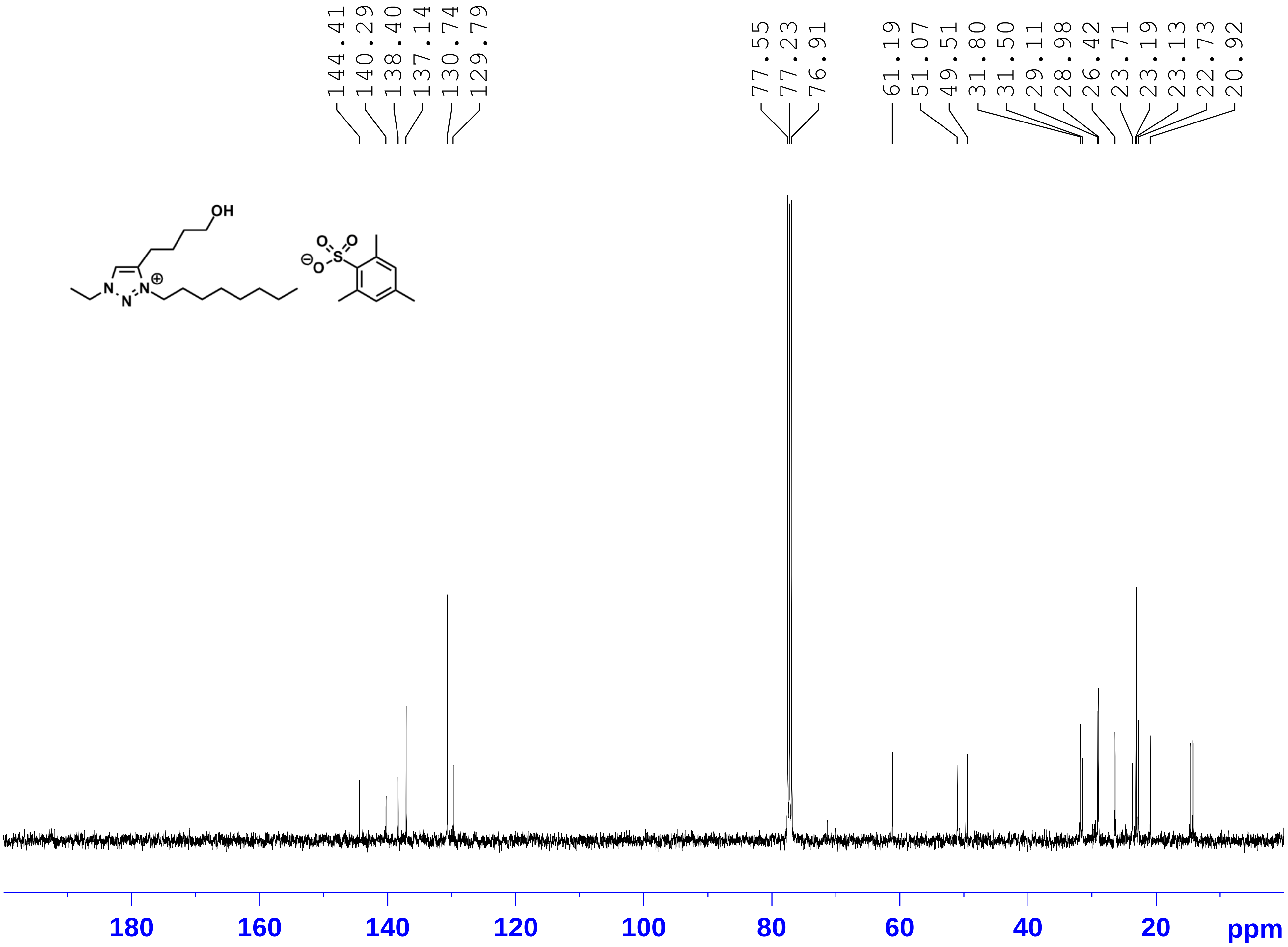

144.41
140.29
138.40
137.14
130.74
129.79
77.55
77.23
76.91
61.19
51.07
49.51
31.80
31.50
29.11
28.98
26.42
23.71
23.19
23.13
22.73
20.92
OH
N
N
N
S
O
O
O
180
160
140
120
100
80
60
40
20
ppm

Spectrum

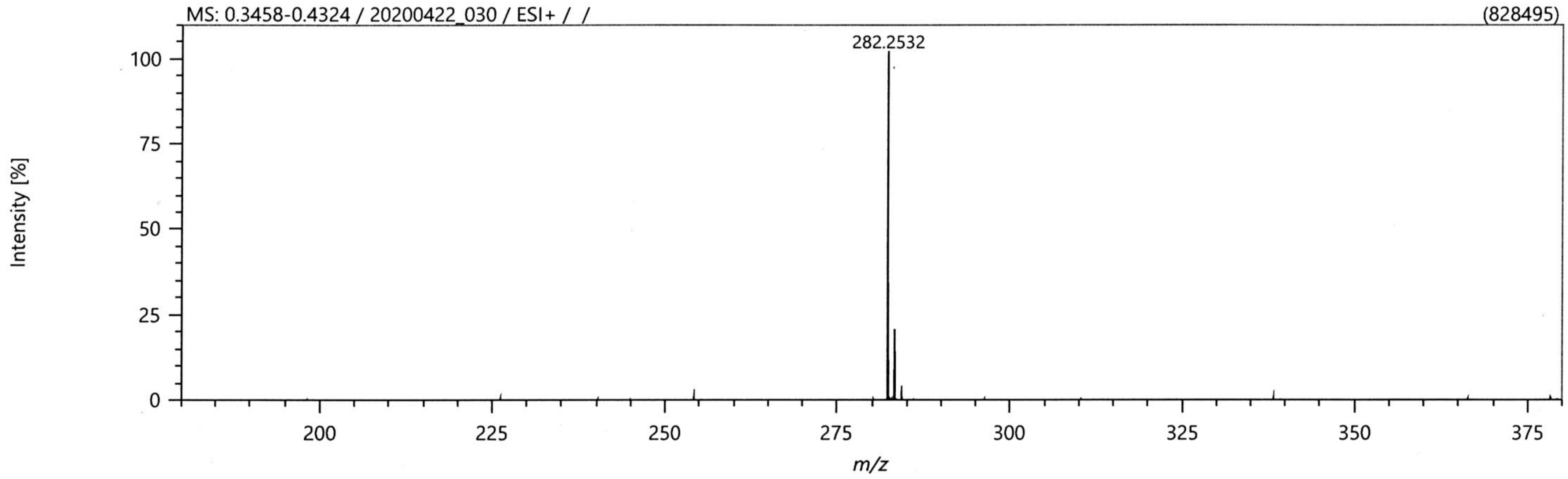

Elemental Composition

Parameters		Elements Set 1:						
Tolerance:	±10.00 ppm	Symbol	C	H	O	Na	N	F
Electron:	Odd/Even	Min	0	0	1	0	3	0
Charge:	+1	Max	1000	4000	1	0	3	0
DBE:	-99.0 - 999.0							

Results

Mass	Formula	Calculated Mass	Mass Difference [mDa]	Mass Difference [ppm]	DBE
282.25325	C16 H32 N3 O	282.25399	-0.74	-2.63	2.5

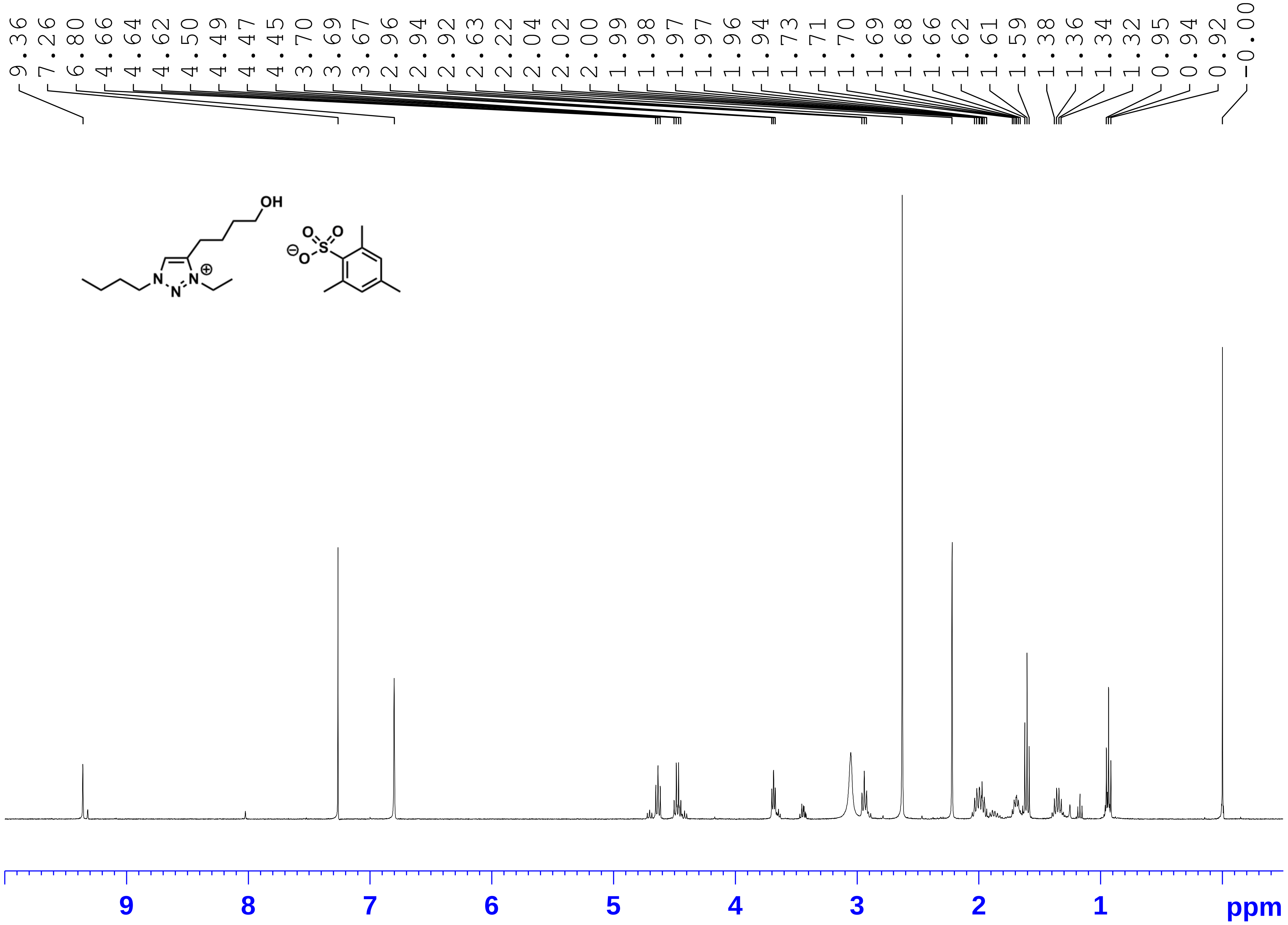
9.36
7.26
6.80
4.66
4.64
4.62
4.50
4.49
4.47
4.45
3.70
3.69
3.67
2.96
2.94
2.92
2.63
2.22
2.04
2.02
2.00
1.99
1.98
1.97
1.97
1.96
1.94
1.73
1.71
1.70
1.69
1.68
1.66
1.62
1.61
1.59
1.38
1.36
1.34
1.32
0.95
0.94
0.92
-0.00
OH
O
S
O
O
N
N
N
9
8
7
6
5
4
3
2
1
ppm

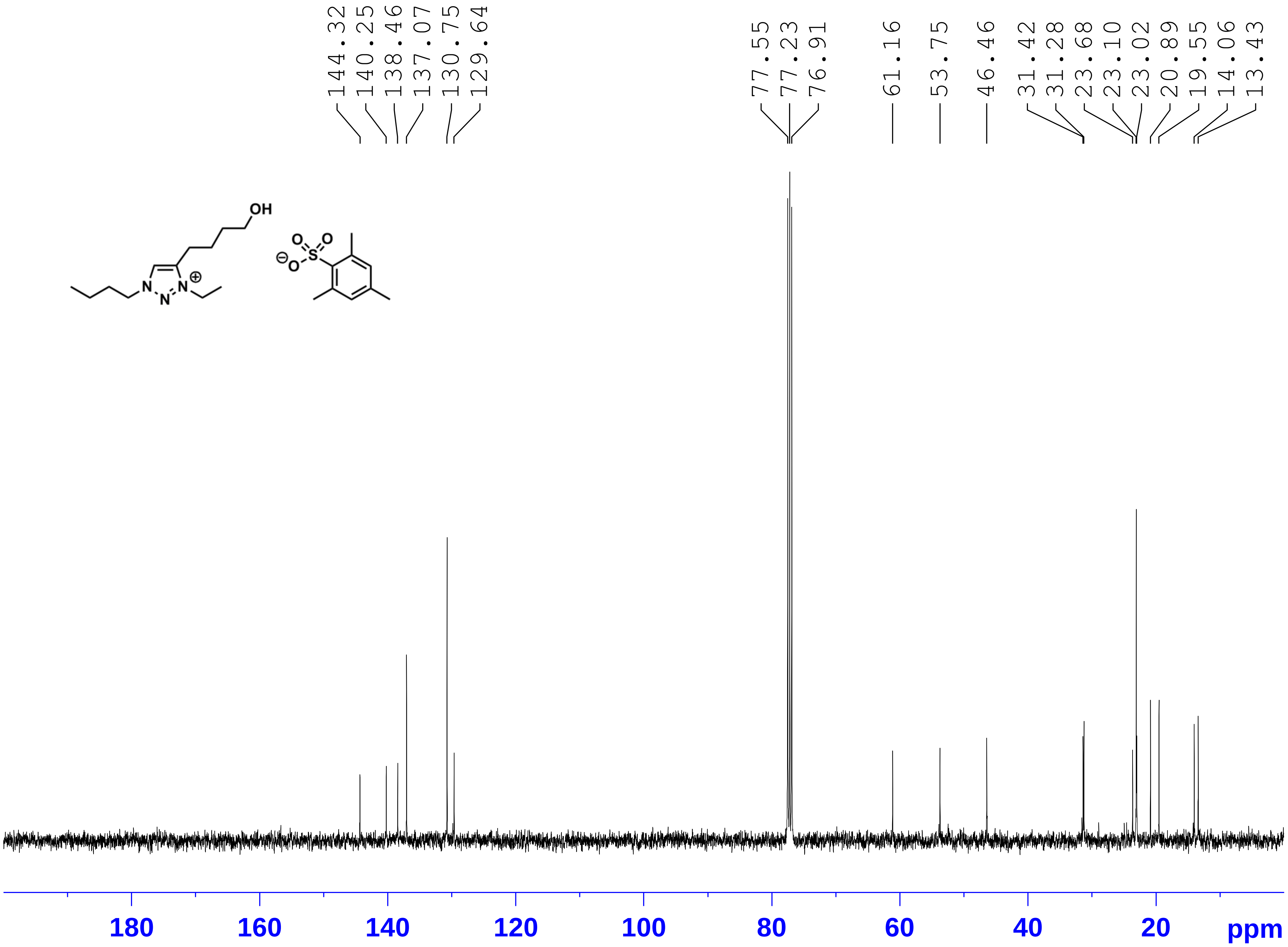

144.32
140.25
138.46
137.07
130.75
129.64
77.55
77.23
76.91
61.16
53.75
46.46
31.42
31.28
23.68
23.10
23.02
20.89
19.55
14.06
13.43
OH
N
N
N
O
O
S
O
180
160
140
120
100
80
60
40
20
ppm

Spectrum

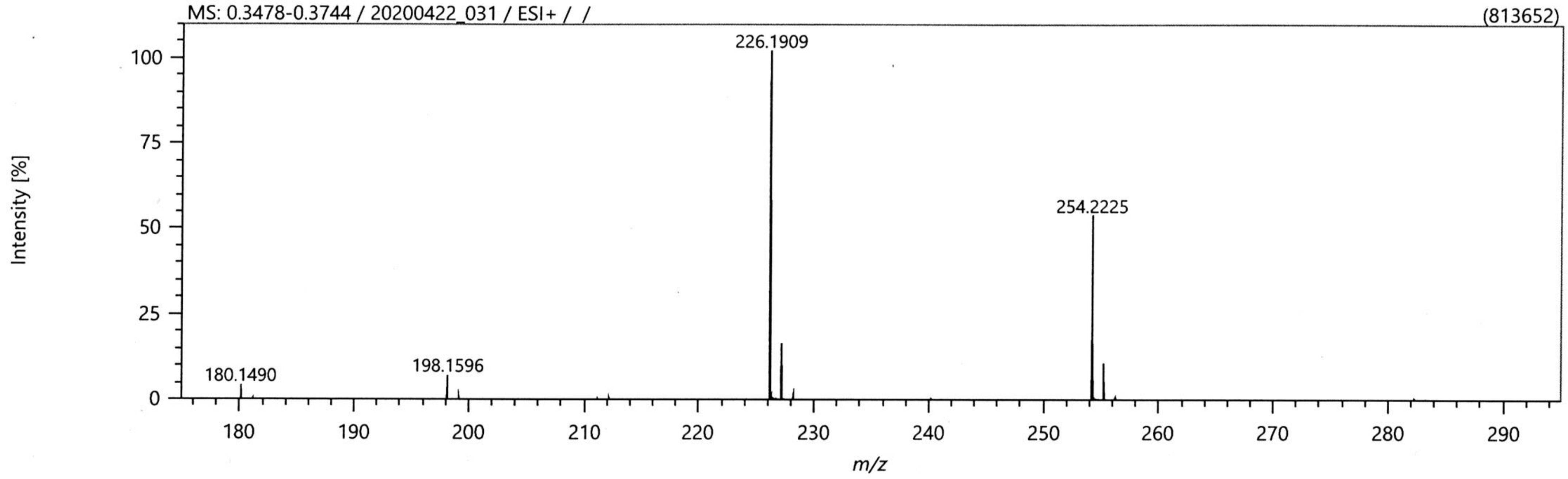

Elemental Composition

Parameters		Elements Set 1:						
Tolerance:	±10.00 ppm	Symbol	C	H	O	Na	N	F
Electron:	Odd/Even	Min	0	0	1	0	3	0
Charge:	+1	Max	1000	4000	1	0	3	0
DBE:	-99.0 - 999.0							

Results

Mass	Formula	Calculated Mass	Mass Difference [mDa]	Mass Difference [ppm]	DBE
226.19091	C12 H24 N3 O	226.19139	-0.48	-2.13	2.5

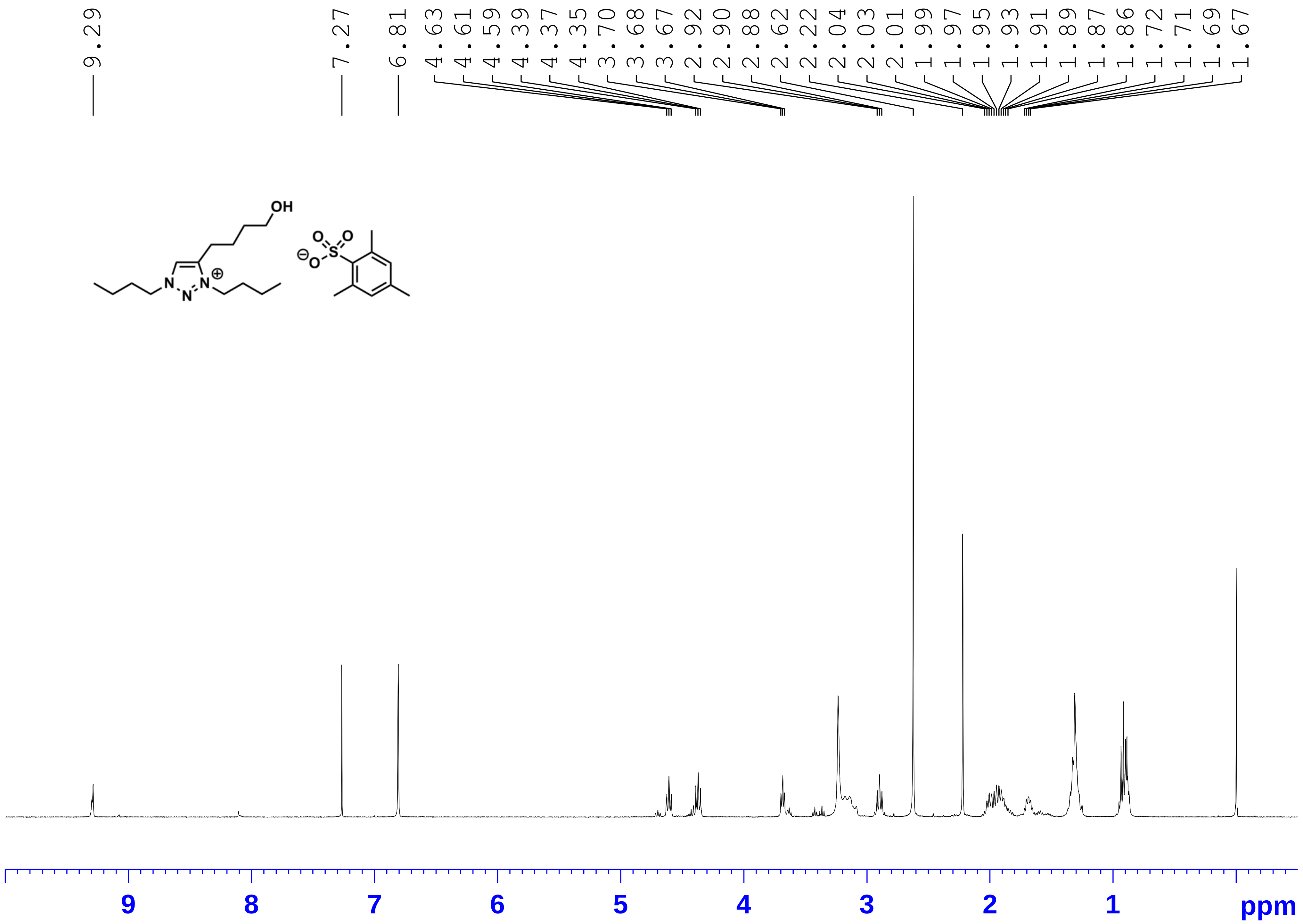

9.29
7.27
6.81
4.63
4.61
4.59
4.39
4.37
4.35
3.70
3.68
3.67
2.92
2.90
2.88
2.62
2.22
2.04
2.03
2.01
1.99
1.97
1.95
1.93
1.91
1.89
1.87
1.86
1.72
1.71
1.69
1.67
OH
N
N
N
O
O
S
O
9
8
7
6
5
4
3
2
1
ppm

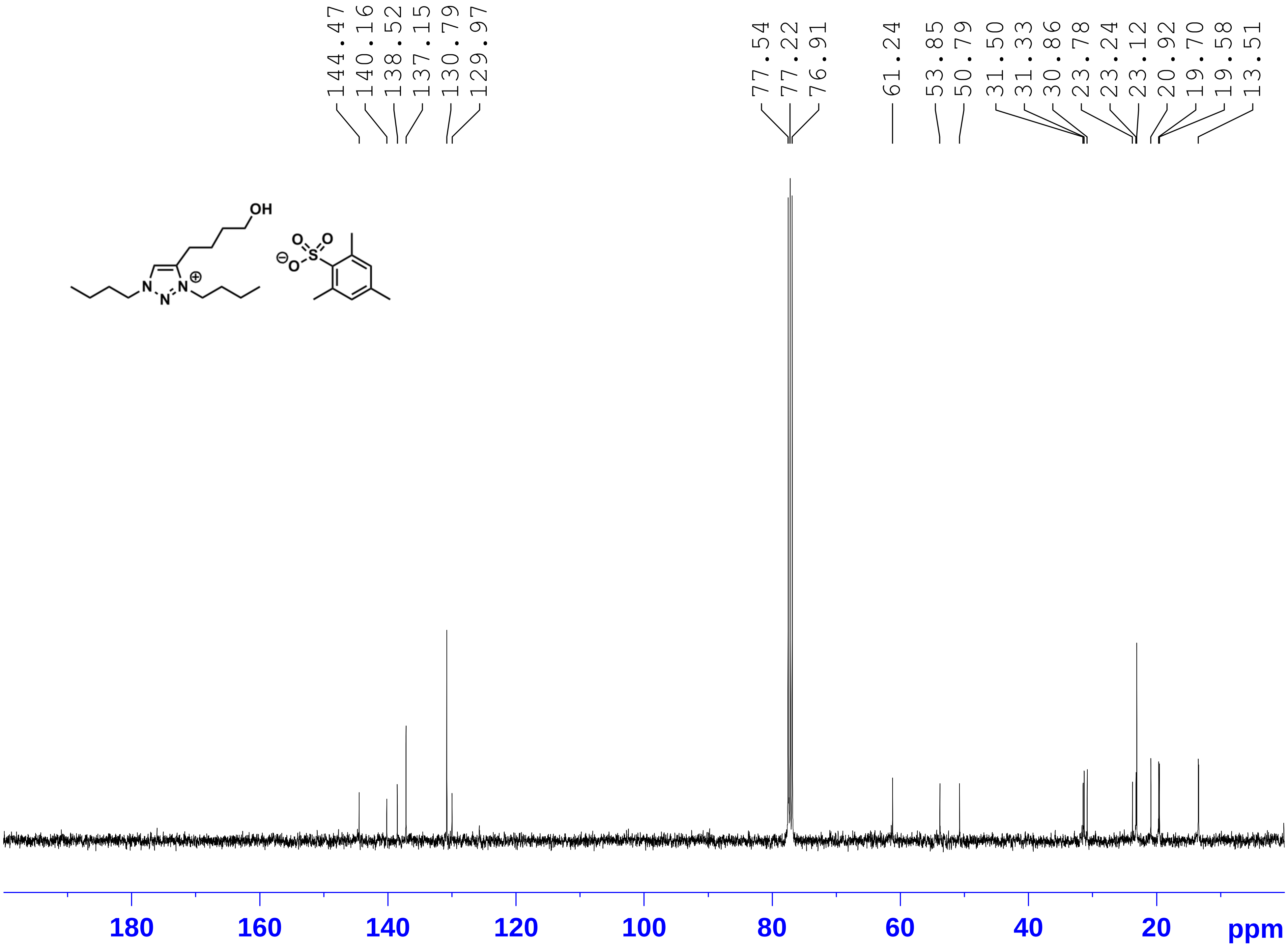
144.47
140.16
138.52
137.15
130.79
129.97
77.54
77.22
76.91
61.24
53.85
50.79
31.50
31.33
30.86
23.78
23.24
23.12
20.92
19.70
19.58
13.51
180
160
140
120
100
80
60
40
20
ppm

Spectrum

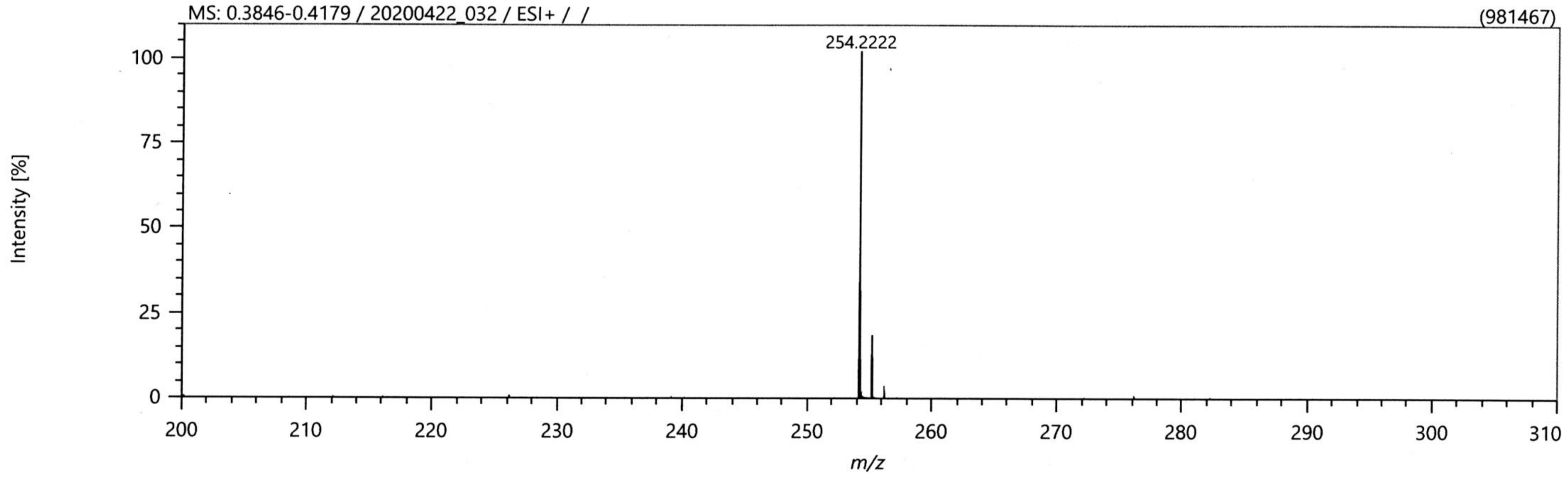

Elemental Composition

Parameters

Tolerance:	±10.00 ppm
Electron:	Odd/Even
Charge:	+1
DBE:	-99.0 - 999.0

Elements Set 1:

Symbol	C	H	O	Na	N	F
Min	0	0	1	0	3	0
Max	1000	4000	1	0	3	0

Results

Mass	Formula	Calculated Mass	Mass Difference [mDa]	Mass Difference [ppm]	DBE
254.22224	C14 H28 N3 O	254.22269	-0.45	-1.77	2.5

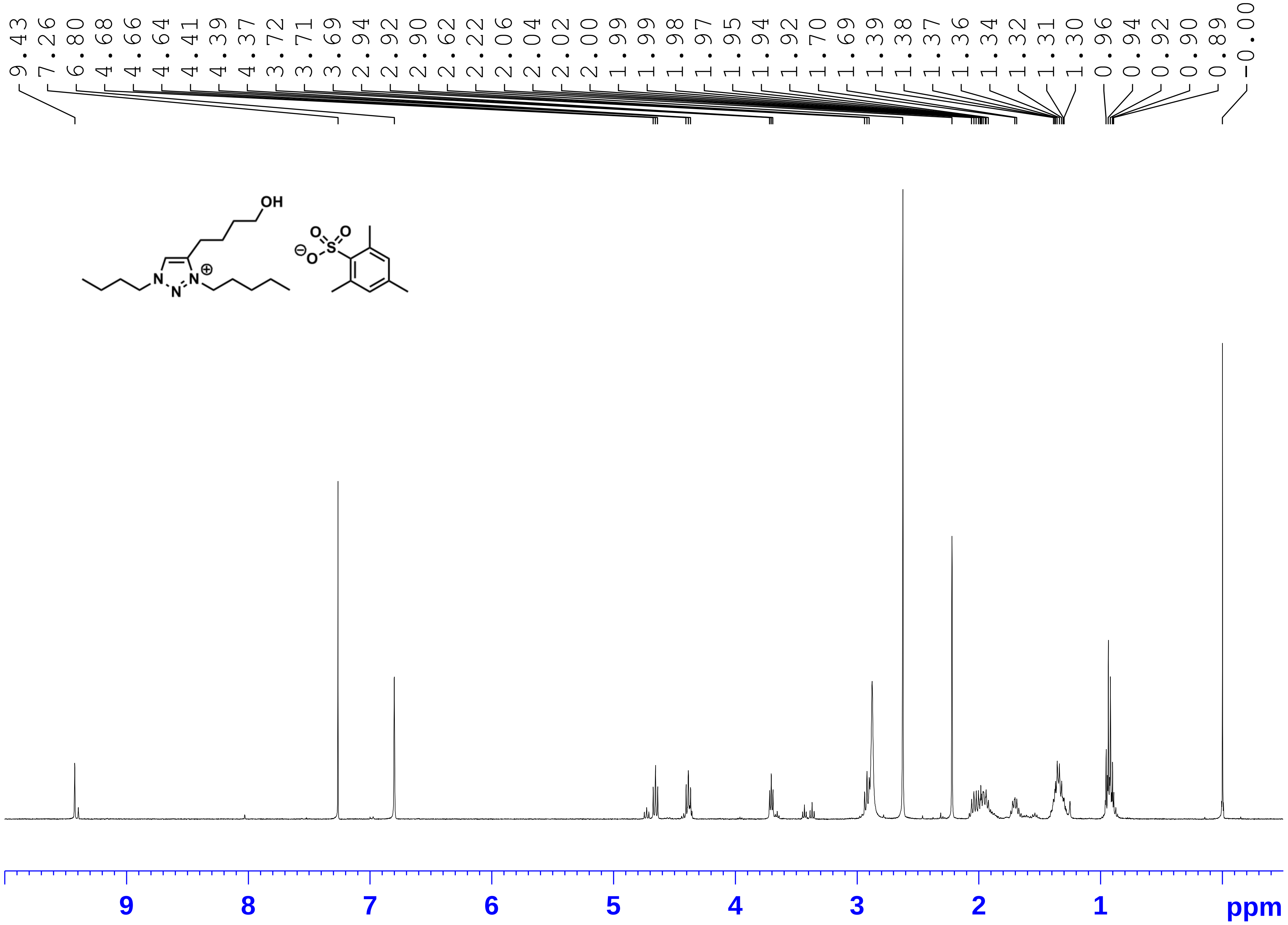
9.43
7.26
6.80
4.68
4.66
4.64
4.41
4.39
4.37
3.72
3.71
3.69
2.94
2.92
2.90
2.62
2.22
2.06
2.04
2.02
2.00
1.99
1.99
1.98
1.97
1.95
1.94
1.92
1.70
1.69
1.39
1.38
1.37
1.36
1.34
1.32
1.31
1.30
0.96
0.94
0.92
0.90
0.89
-0.00
OH
N
N
N
O
S
O
O
9
8
7
6
5
4
3
2
1
ppm

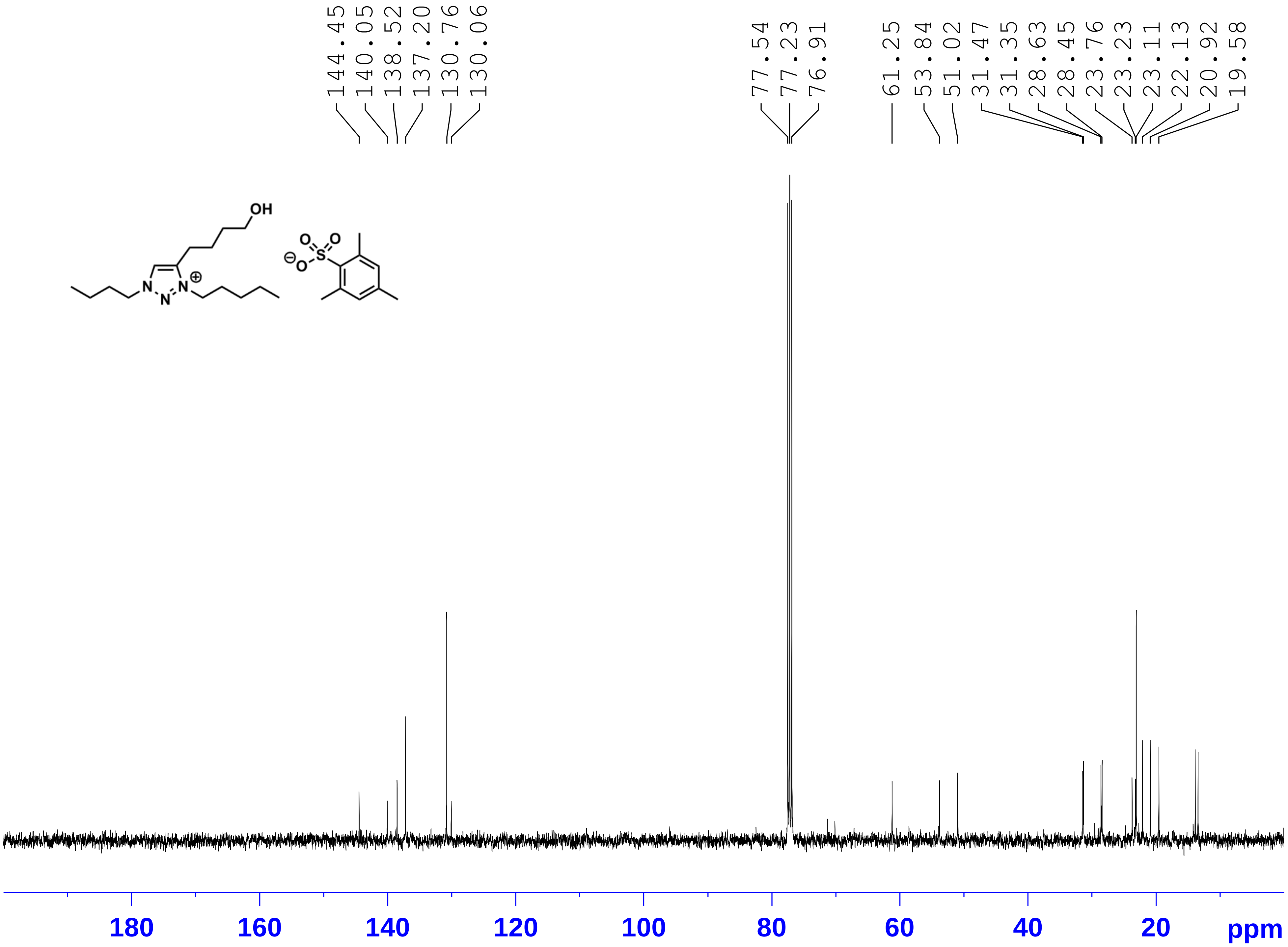
144.45
140.05
138.52
137.20
130.76
130.06
77.54
77.23
76.91
61.25
53.84
51.02
31.47
31.35
28.63
28.45
23.76
23.23
23.11
22.13
20.92
19.58
OH
180
160
140
120
100
80
60
40
20
ppm

Spectrum

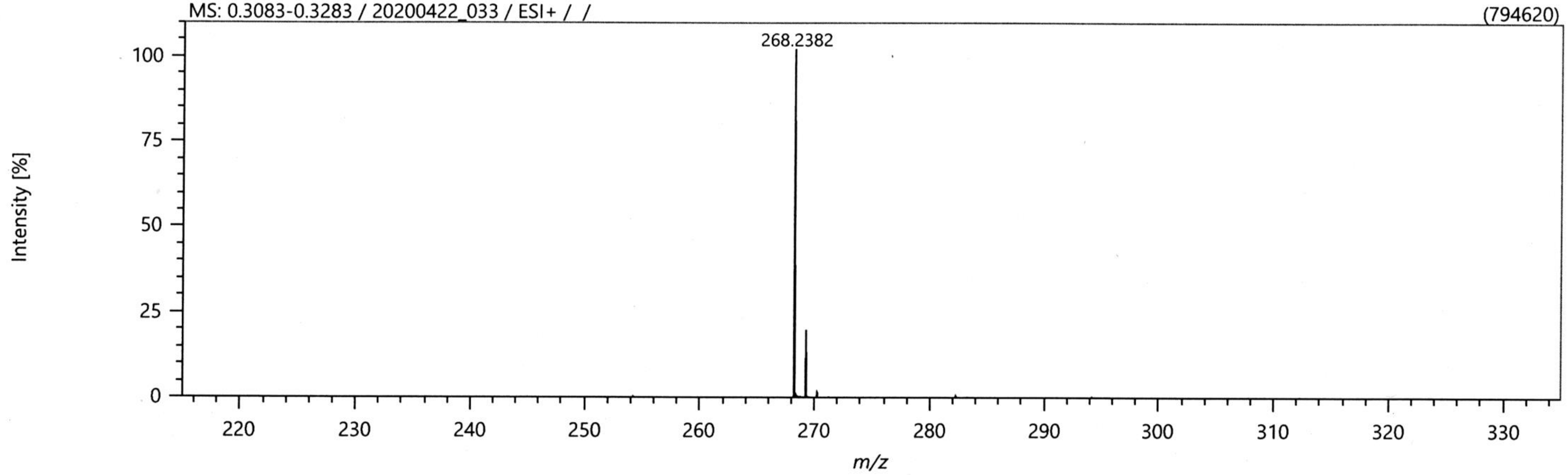

Elemental Composition

Parameters	
Tolerance:	±10.00 ppm
Electron:	Odd/Even
Charge:	+1
DBE:	-99.0 - 999.0

Elements Set 1:

Symbol	C	H	O	Na	N	F
Min	0	0	1	0	3	0
Max	1000	4000	1	0	3	0

Results

Mass	Formula	Calculated Mass	Mass Difference [mDa]	Mass Difference [ppm]	DBE
268.23819	C15 H30 N3 O	268.23834	-0.15	-0.56	2.5

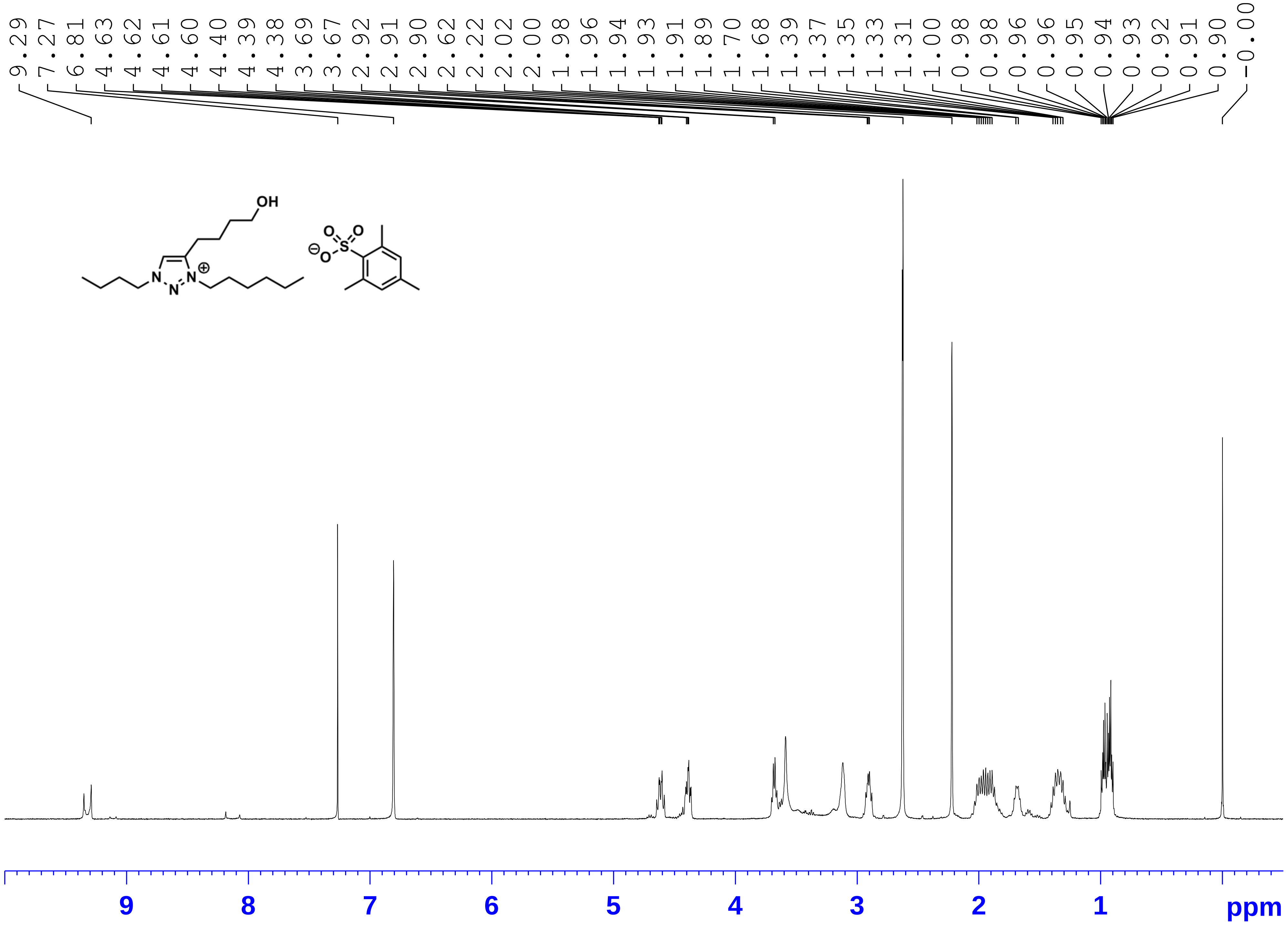
9.29
7.27
6.81
4.63
4.62
4.61
4.60
4.40
4.39
4.38
3.69
3.67
2.92
2.91
2.90
2.62
2.22
2.02
2.00
1.98
1.96
1.94
1.93
1.91
1.89
1.70
1.68
1.39
1.37
1.35
1.33
1.31
1.00
0.98
0.98
0.96
0.96
0.95
0.94
0.93
0.92
0.91
0.90
-0.00
OH
N
N
N
O
S
O
O
9
8
7
6
5
4
3
2
1
ppm

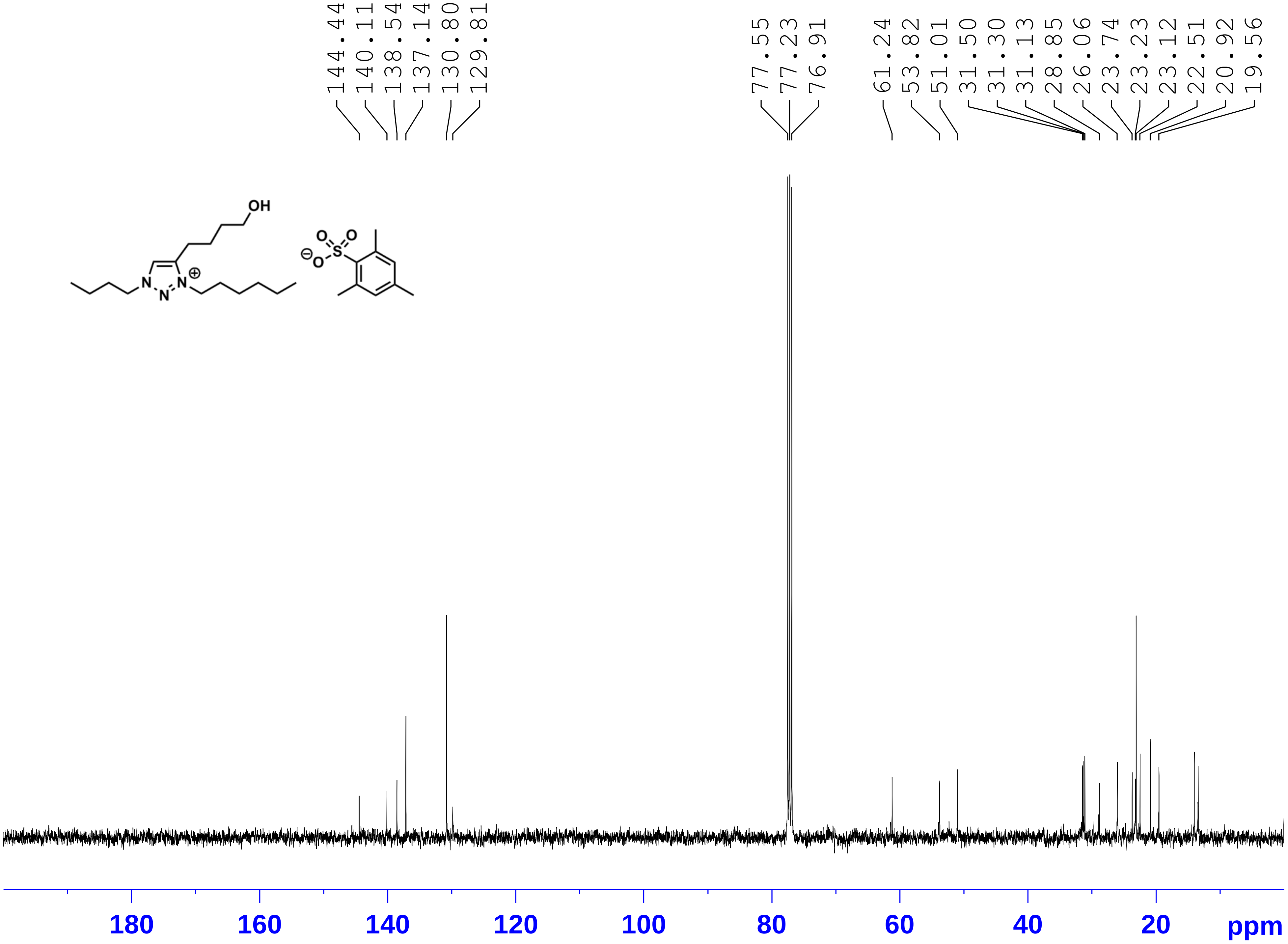
144.44
140.11
138.54
137.14
130.80
129.81
77.55
77.23
76.91
61.24
53.82
51.01
31.50
31.30
31.13
28.85
26.06
23.74
23.23
23.12
22.51
20.92
19.56
OH
O
S
N
180
160
140
120
100
80
60
40
20
ppm

Spectrum

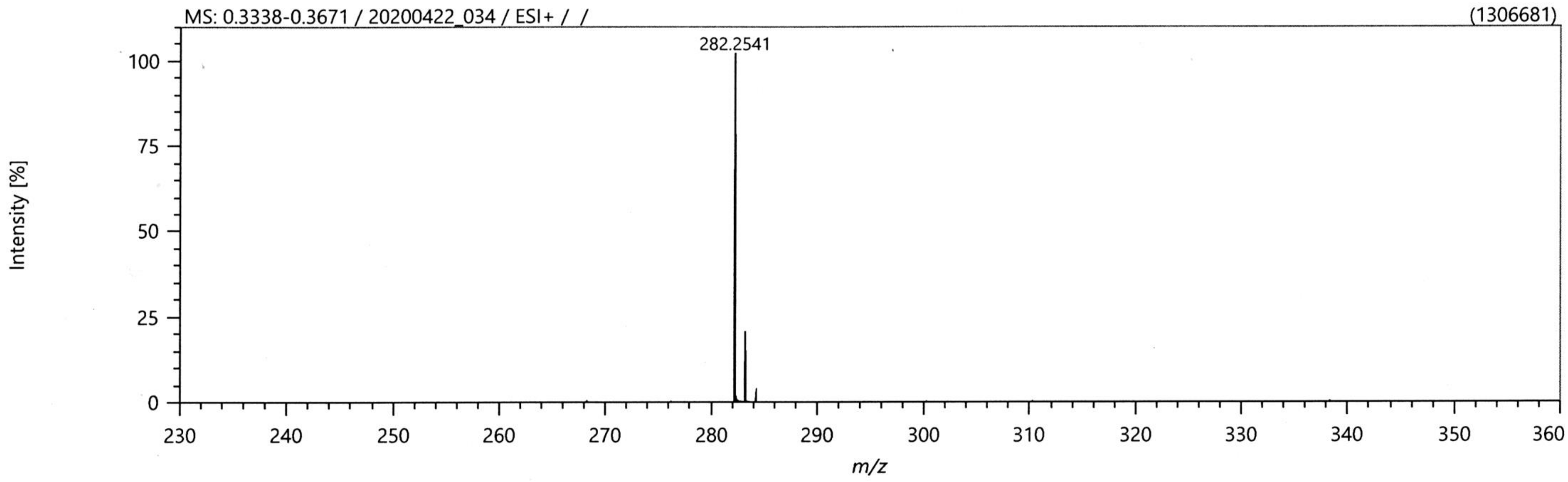

Elemental Composition

Parameters	
Tolerance:	±10.00 ppm
Electron:	Odd/Even
Charge:	+1
DBE:	-99.0 - 999.0

Elements Set 1:

Symbol	C	H	O	Na	N	F
Min	0	0	1	0	3	0
Max	1000	4000	1	0	3	0

Results

Mass	Formula	Calculated Mass	Mass Difference [mDa]	Mass Difference [ppm]	DBE
282.25408	C16 H32 N3 O	282.25399	0.09	0.31	2.5

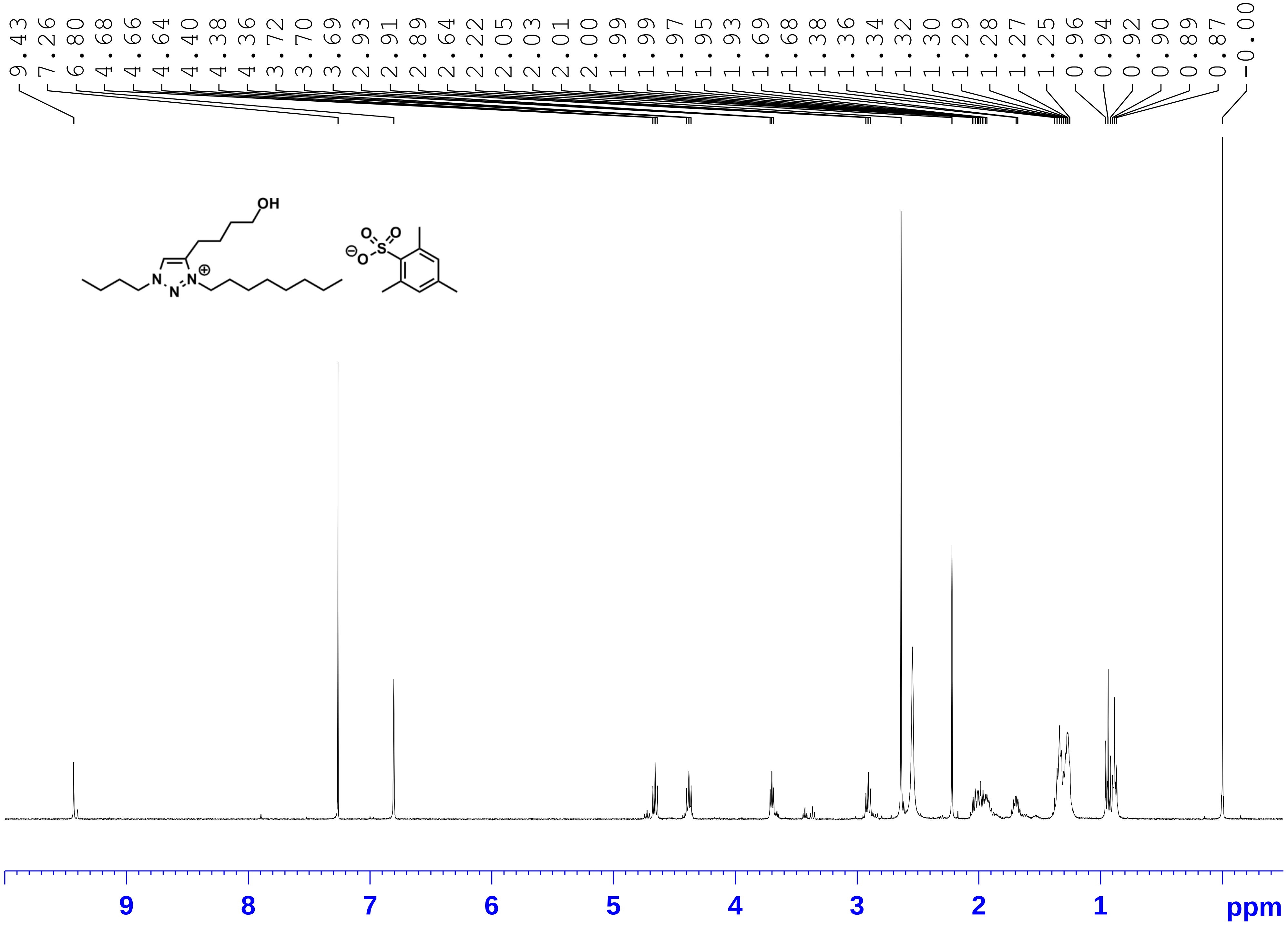

9.43
7.26
6.80
4.68
4.66
4.64
4.40
4.38
4.36
3.72
3.70
3.69
2.93
2.91
2.89
2.64
2.22
2.05
2.03
2.01
2.00
1.99
1.99
1.97
1.95
1.93
1.69
1.68
1.38
1.36
1.34
1.32
1.30
1.29
1.28
1.27
1.25
0.96
0.94
0.92
0.90
0.89
0.87
-0.00
OH
9
8
7
6
5
4
3
2
1
ppm

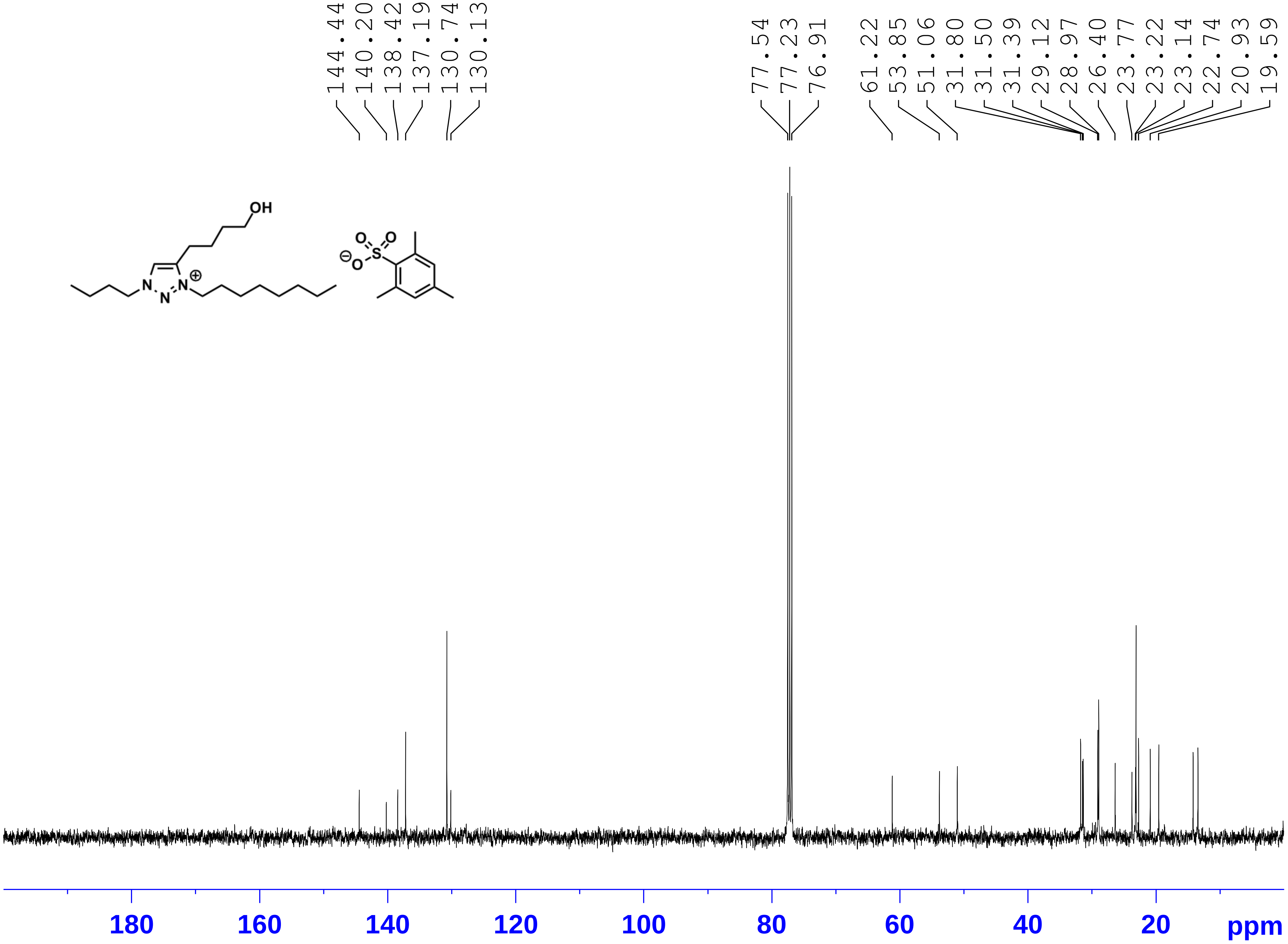
144.44
140.20
138.42
137.19
130.74
130.13
77.54
77.23
76.91
61.22
53.85
51.06
31.80
31.50
31.39
29.12
28.97
26.40
23.77
23.22
23.14
22.74
20.93
19.59
OH
N
N
N
O
O
O
S
180
160
140
120
100
80
60
40
20
ppm

Spectrum

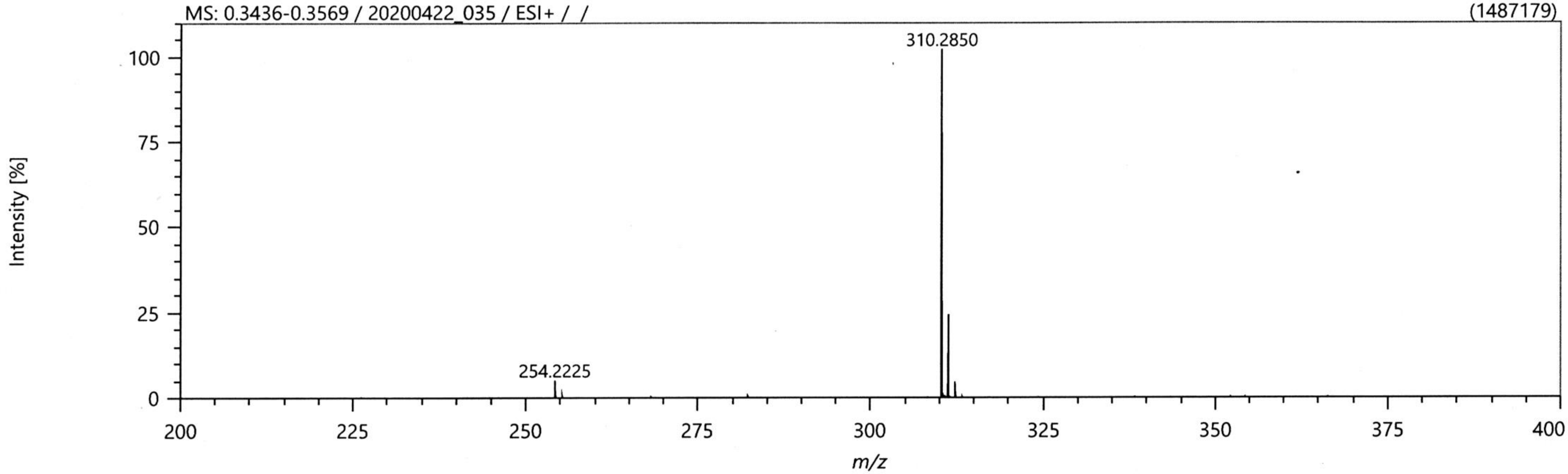

Elemental Composition

Parameters		Elements Set 1:						
		Symbol	C	H	O	Na	N	F
Tolerance:	±10.00 ppm	Min	0	0	1	0	3	0
Electron:	Odd/Even	Max	1000	4000	1	0	3	0
Charge:	+1							
DBE:	-99.0 - 999.0							

Results

Mass	Formula	Calculated Mass	Mass Difference [mDa]	Mass Difference [ppm]	DBE
310.28505	C18 H36 N3 O	310.28529	-0.24	-0.78	2.5

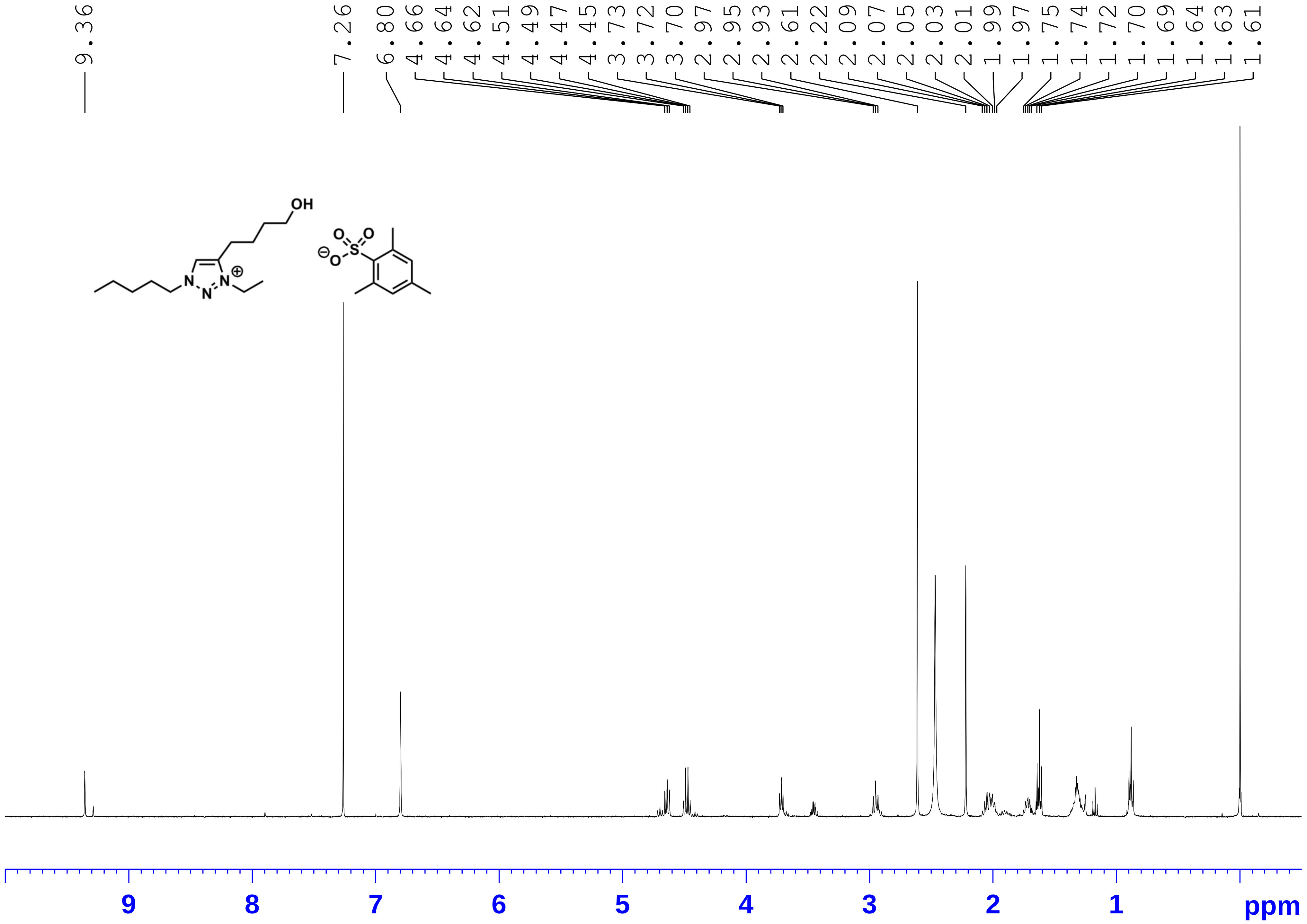

9.36
7.26
6.80
4.66
4.64
4.62
4.51
4.49
4.47
4.45
3.73
3.72
3.70
2.97
2.95
2.93
2.61
2.22
2.09
2.07
2.05
2.03
2.01
1.99
1.97
1.75
1.74
1.72
1.70
1.69
1.64
1.63
1.61
OH
O
O
S
O
N
N
N
9
8
7
6
5
4
3
2
1
ppm

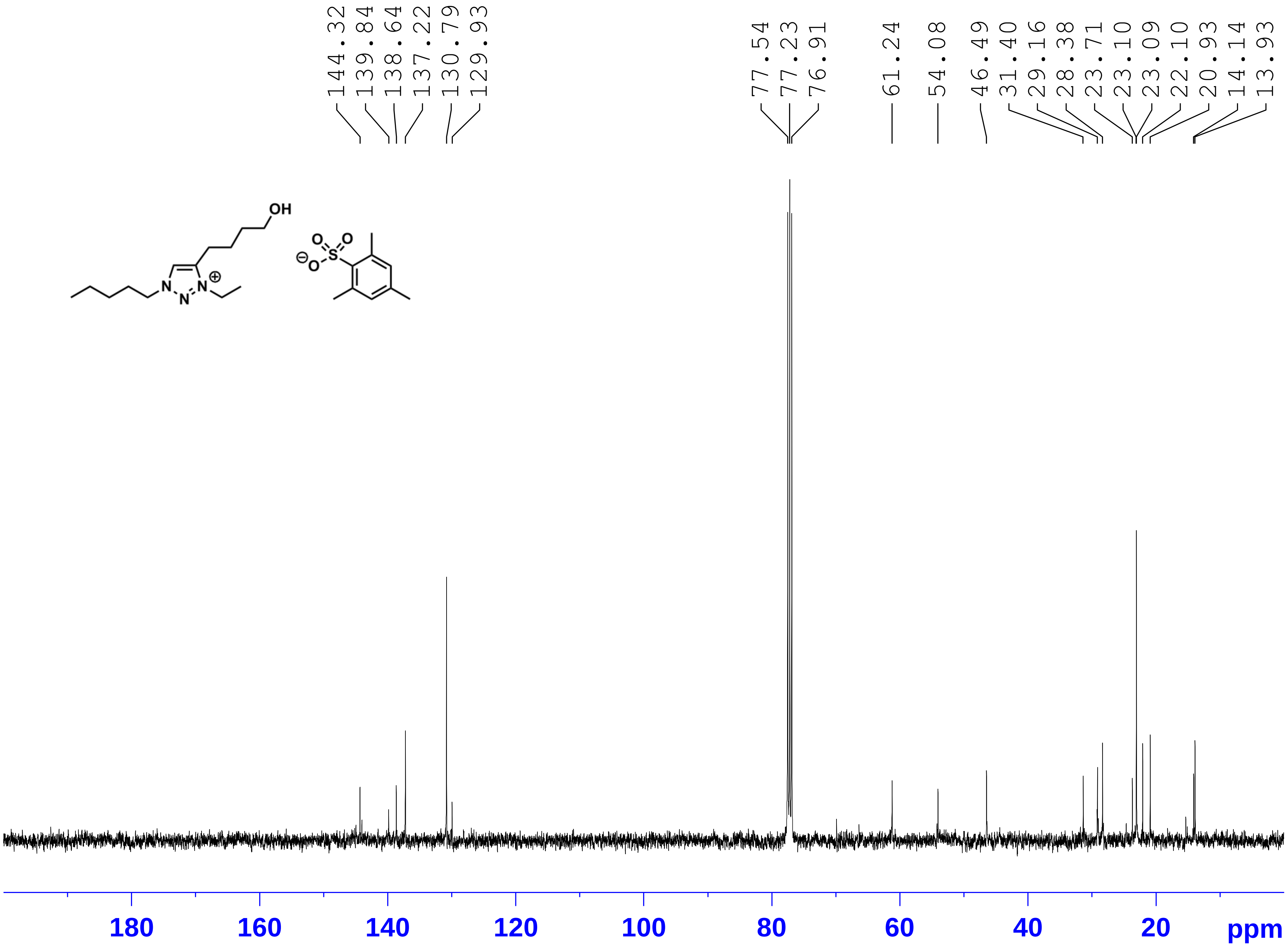

144.32
139.84
138.64
137.22
130.79
129.93
77.54
77.23
76.91
61.24
54.08
46.49
31.40
29.16
28.38
23.71
23.10
23.09
22.10
20.93
14.14
13.93
OH
N
N
N
O
O
S
O
180
160
140
120
100
80
60
40
20
ppm

Spectrum

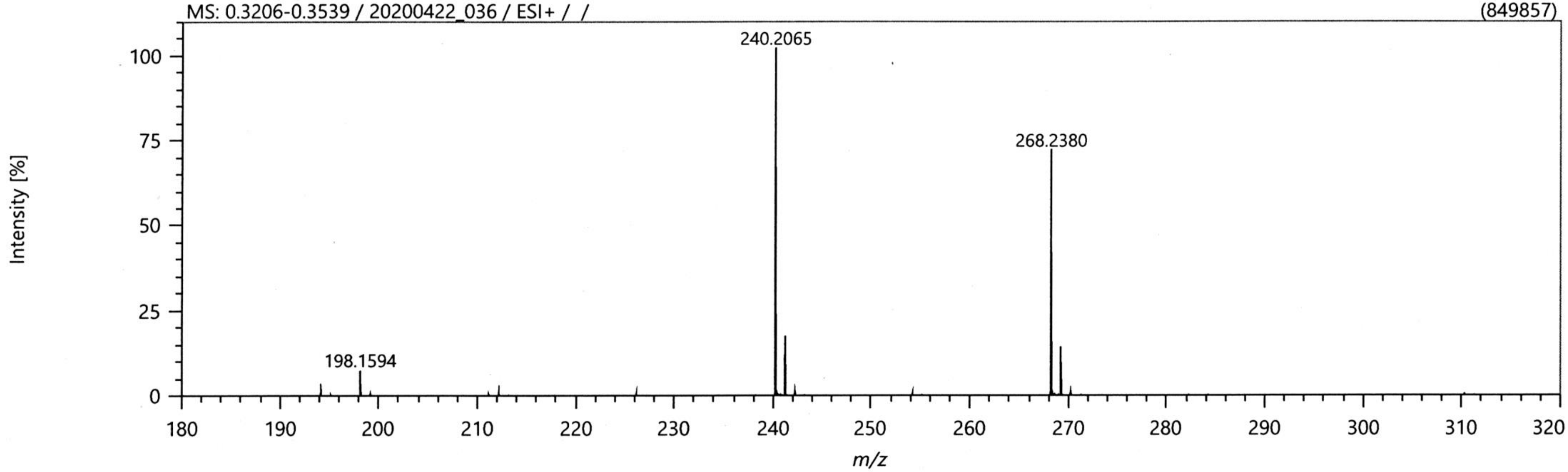

Elemental Composition

Parameters		Elements Set 1:						
Tolerance:	±10.00 ppm	Symbol	C	H	O	Na	N	F
Electron:	Odd/Even	Min	0	0	1	0	3	0
Charge:	+1	Max	1000	4000	1	0	3	0
DBE:	-99.0 - 999.0							

Results

Mass	Formula	Calculated Mass	Mass Difference [mDa]	Mass Difference [ppm]	DBE
240.20653	C13 H26 N3 O	240.20704	-0.51	-2.12	2.5

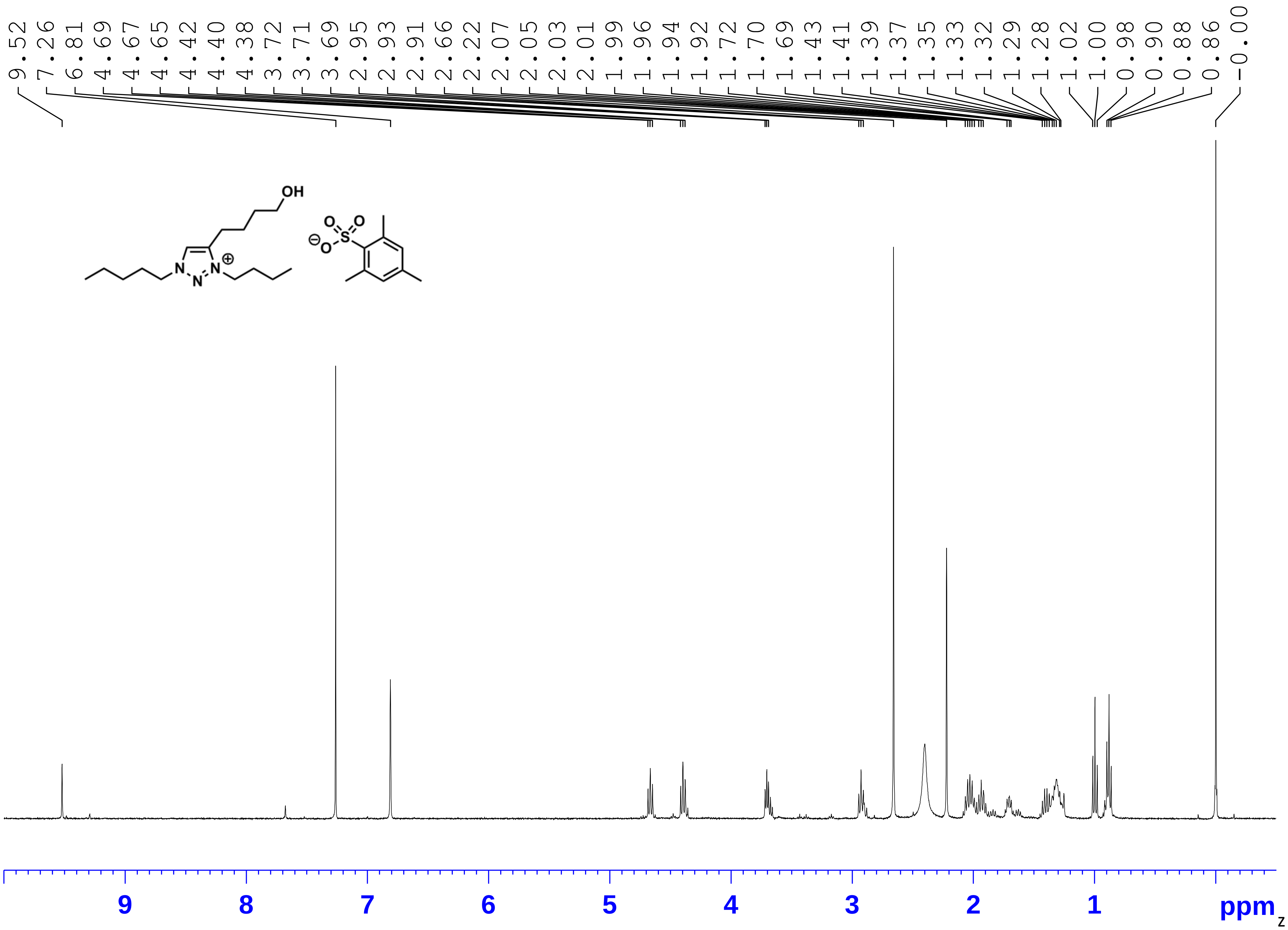
9.52
7.26
6.81
4.69
4.67
4.65
4.42
4.40
4.38
3.72
3.71
3.69
2.95
2.93
2.91
2.66
2.22
2.07
2.05
2.03
2.01
1.99
1.96
1.94
1.92
1.72
1.70
1.69
1.43
1.41
1.39
1.37
1.35
1.33
1.32
1.29
1.28
1.02
1.00
0.98
0.90
0.88
0.86
-0.00
OH
N
N
N
O
S
O
O
9
8
7
6
5
4
3
2
1
ppm

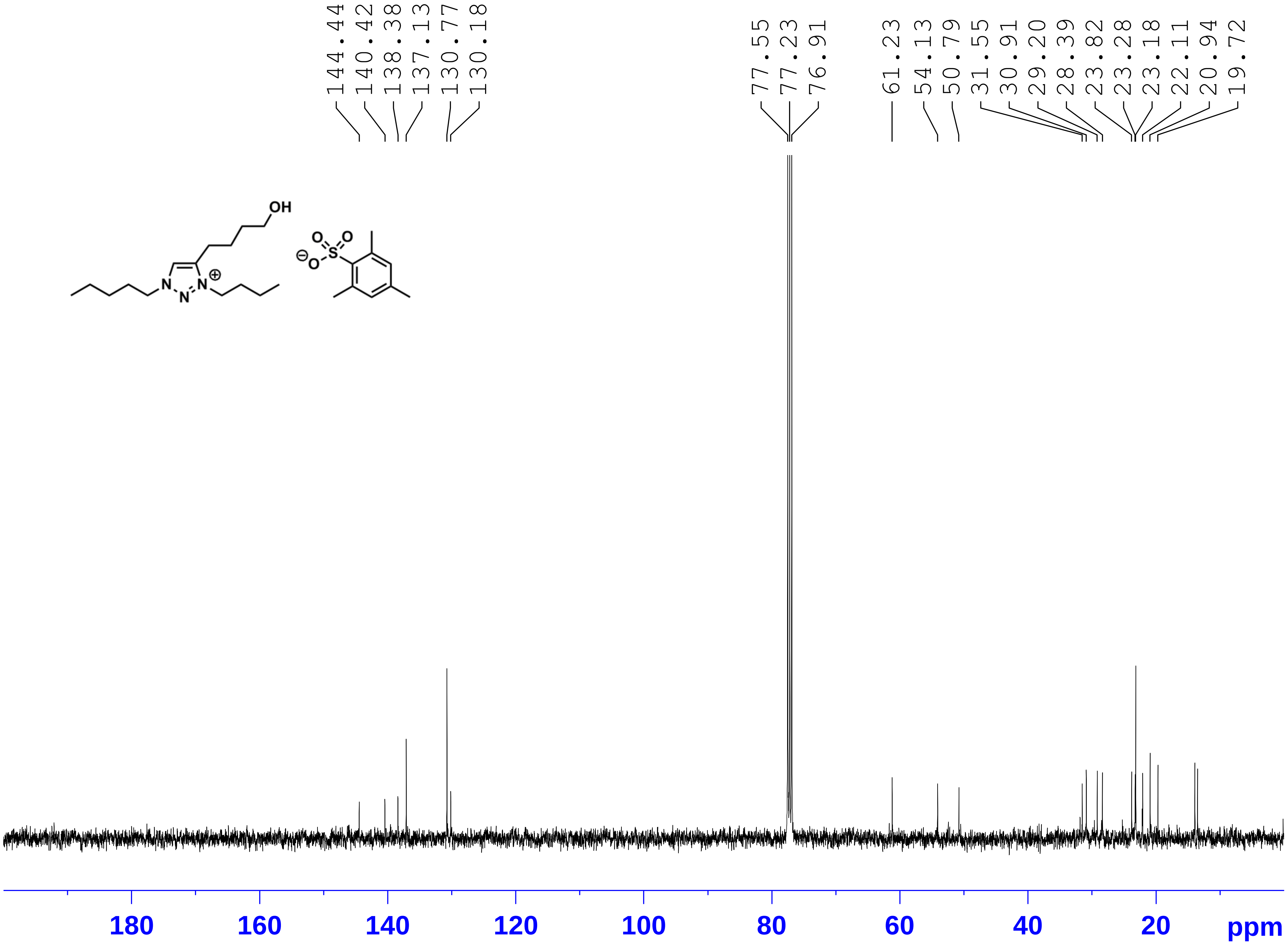
144.44
140.42
138.38
137.13
130.77
130.18
77.55
77.23
76.91
61.23
54.13
50.79
31.55
30.91
29.20
28.39
23.82
23.28
23.18
22.11
20.94
19.72
OH
O
O
S
O
N
N
N
180
160
140
120
100
80
60
40
20
ppm

Spectrum

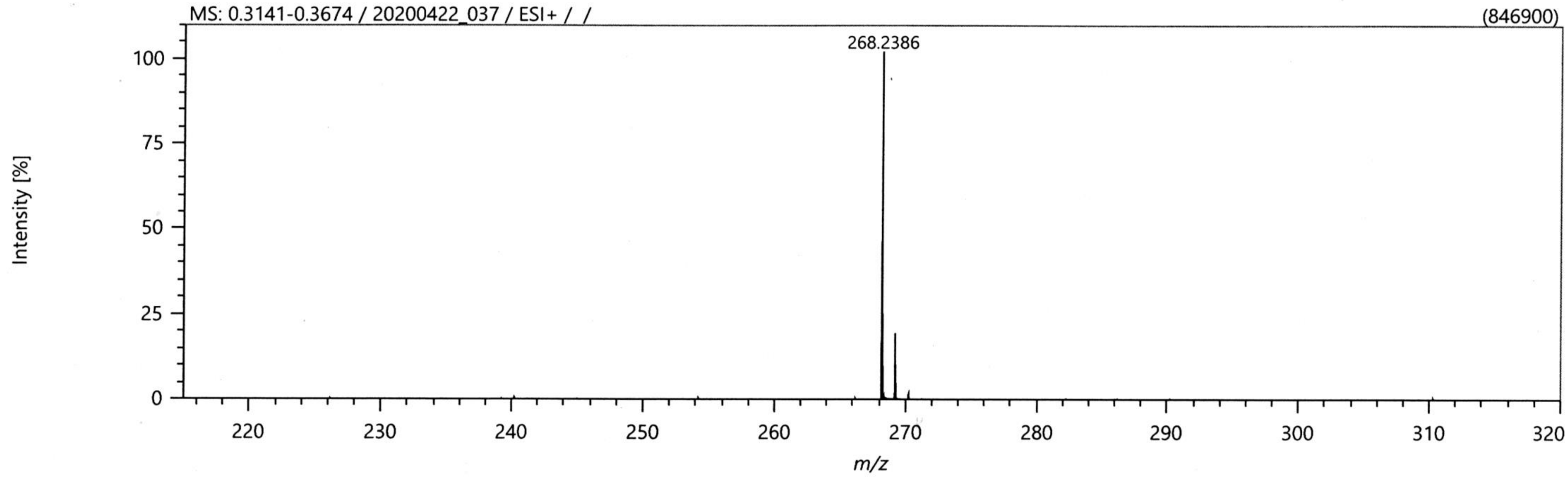

Elemental Composition

Parameters

Tolerance:	±10.00 ppm
Electron:	Odd/Even
Charge:	+1
DBE:	-99.0 - 999.0

Elements Set 1:

Symbol	C	H	O	Na	N	F
Min	0	0	1	0	3	0
Max	1000	4000	1	0	3	0

Results

Mass	Formula	Calculated Mass	Mass Difference [mDa]	Mass Difference [ppm]	DBE
268.23856	C15 H30 N3 O	268.23834	0.22	0.82	2.5

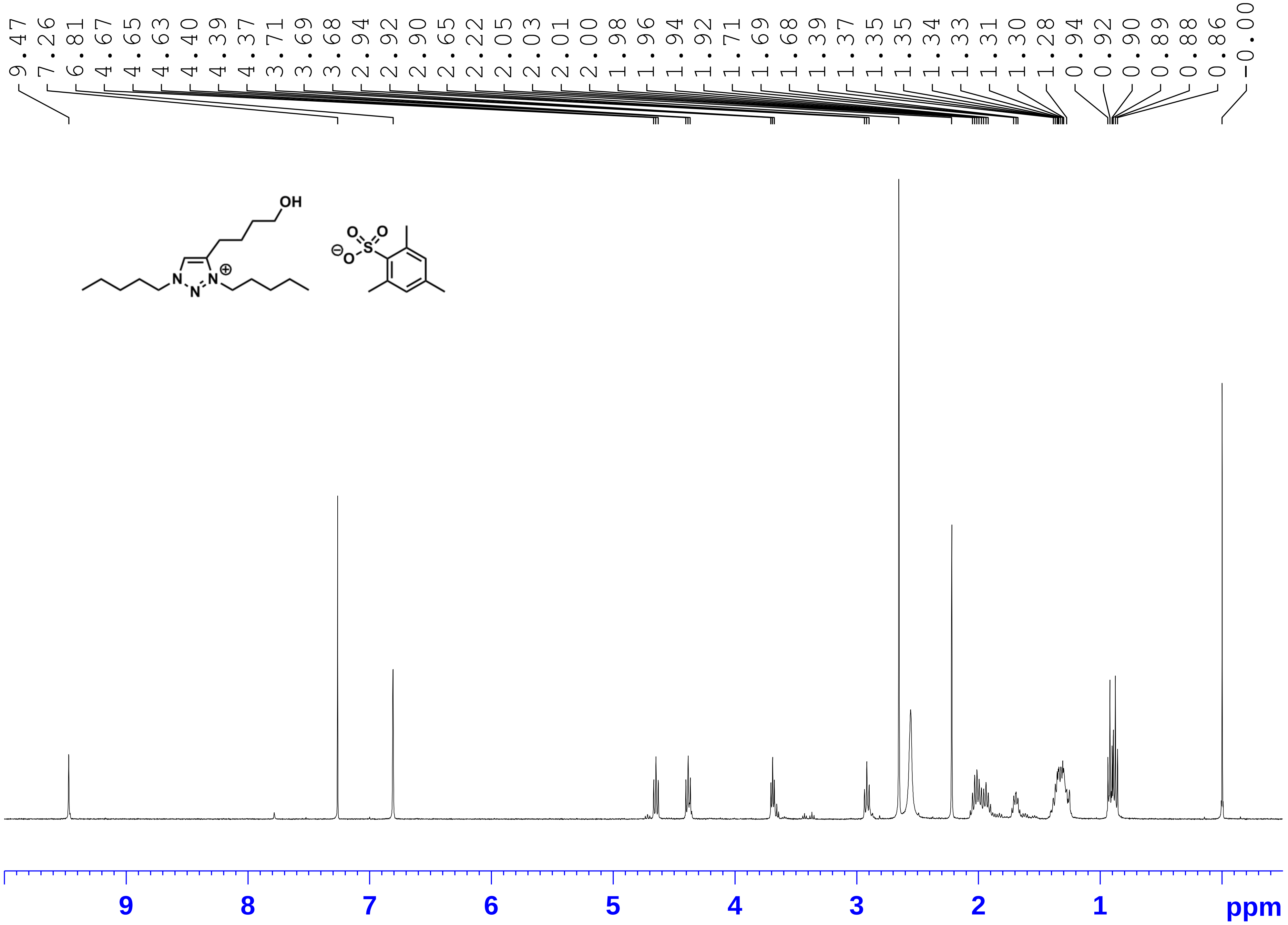
9.47
7.26
6.81
4.67
4.65
4.63
4.40
4.39
4.37
3.71
3.69
3.68
2.94
2.92
2.90
2.65
2.22
2.05
2.03
2.01
2.00
1.98
1.96
1.94
1.92
1.71
1.69
1.68
1.39
1.37
1.35
1.35
1.34
1.33
1.31
1.30
1.28
0.94
0.92
0.90
0.89
0.88
0.86
-0.00
OH
9
8
7
6
5
4
3
2
1
ppm

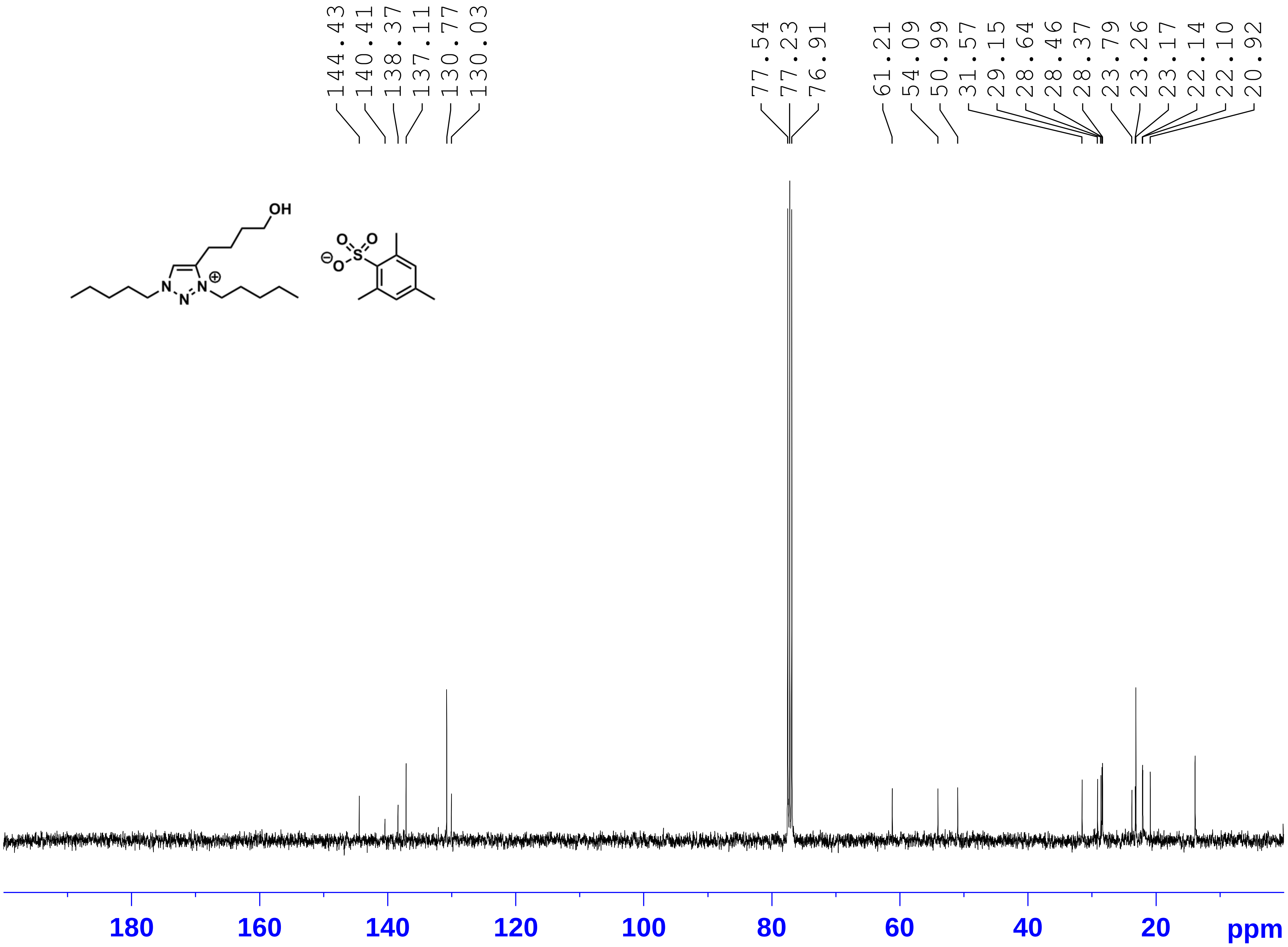
144.43
140.41
138.37
137.11
130.77
130.03
77.54
77.23
76.91
61.21
54.09
50.99
31.57
29.15
28.64
28.46
28.37
23.79
23.26
23.17
22.14
22.10
20.92
OH
180
160
140
120
100
80
60
40
20
ppm

Spectrum

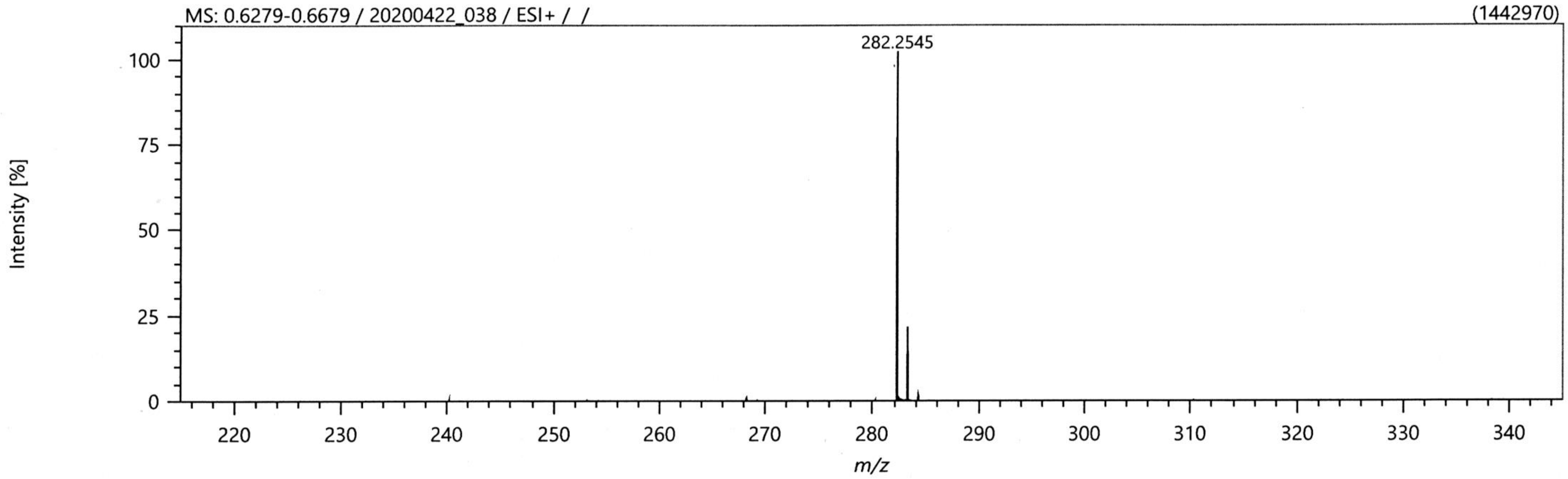

Elemental Composition

Parameters

Tolerance:	±10.00 ppm
Electron:	Odd/Even
Charge:	+1
DBE:	-99.0 - 999.0

Elements Set 1:

Symbol	C	H	O	Na	N	F
Min	0	0	1	0	3	0
Max	1000	4000	1	0	3	0

Results

Mass	Formula	Calculated Mass	Mass Difference [mDa]	Mass Difference [ppm]	DBE
282.25455	C16 H32 N3 O	282.25399	0.56	1.98	2.5

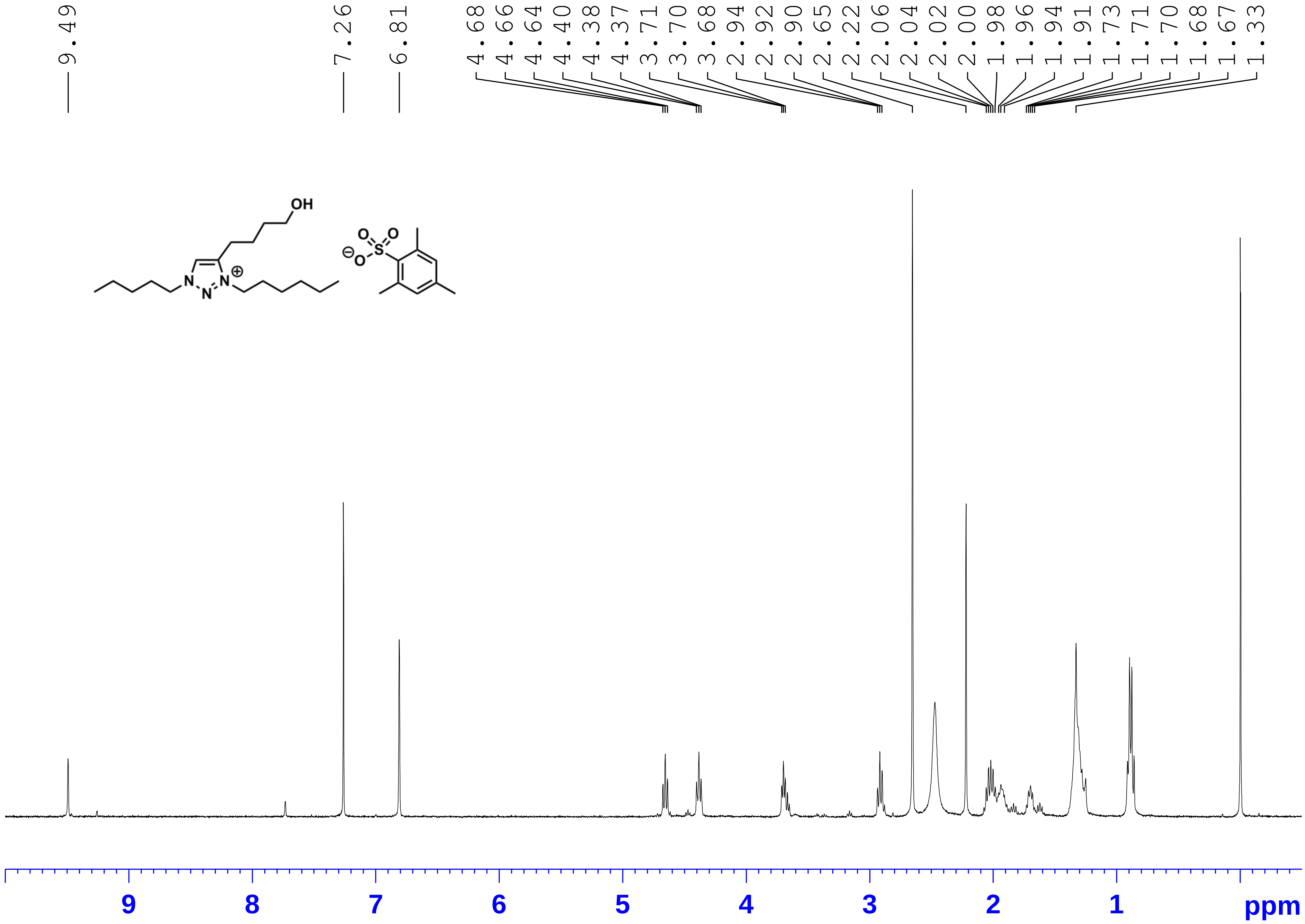

9.49
7.26
6.81
4.68
4.66
4.64
4.40
4.38
4.37
3.71
3.70
3.68
2.94
2.92
2.90
2.65
2.22
2.06
2.04
2.02
2.00
1.98
1.96
1.94
1.91
1.73
1.71
1.70
1.68
1.67
1.33
OH
N
N
N
O
O
S
O
9
8
7
6
5
4
3
2
1
ppm

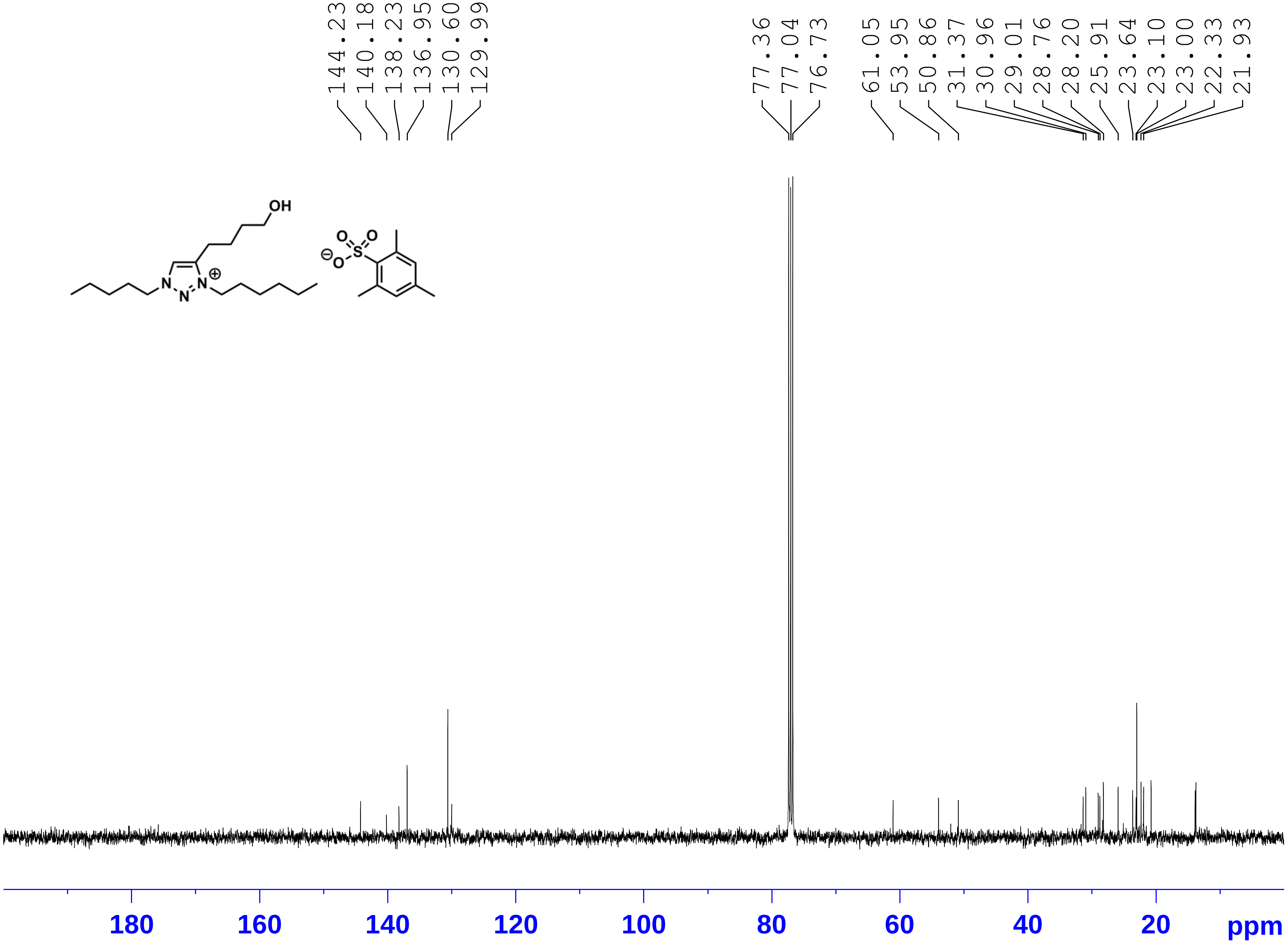
144.23
140.18
138.23
136.95
130.60
129.99
77.36
77.04
76.73
61.05
53.95
50.86
31.37
30.96
29.01
28.76
28.20
25.91
23.64
23.10
23.00
22.33
21.93
OH
O
S
N
180
160
140
120
100
80
60
40
20
ppm

Spectrum

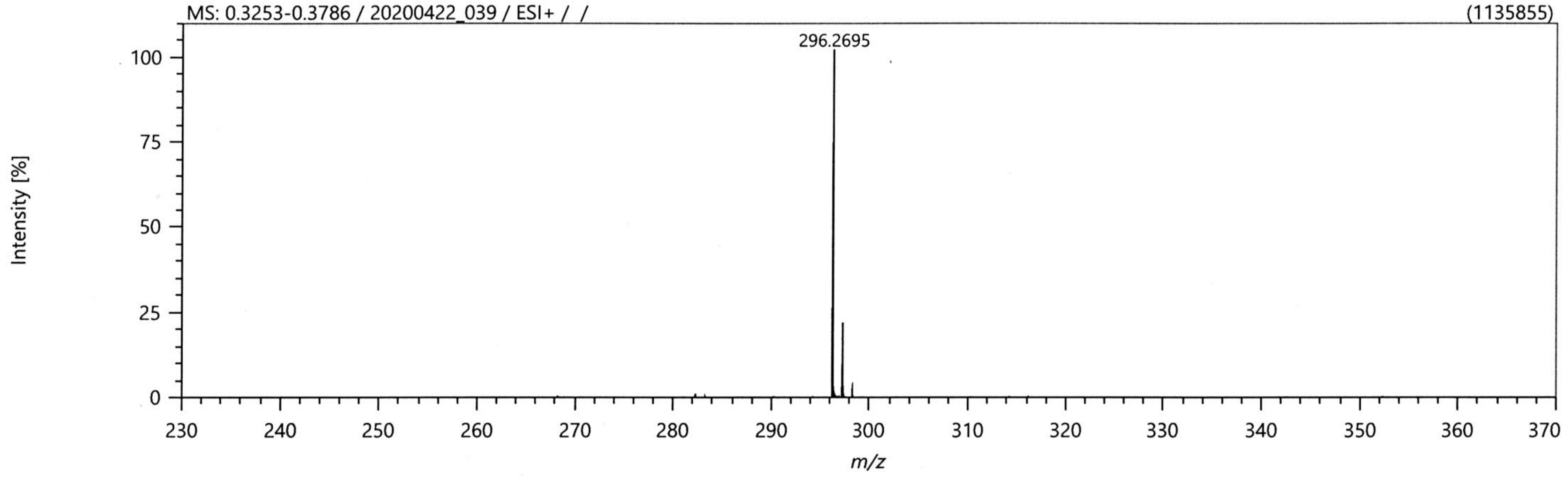

Elemental Composition

Parameters

		Elements Set 1:						
Tolerance:	±10.00 ppm	Symbol	C	H	O	Na	N	F
Electron:	Odd/Even	Min	0	0	1	0	3	0
Charge:	+1	Max	1000	4000	1	0	3	0
DBE:	-99.0 - 999.0							

Results

Mass	Formula	Calculated Mass	Mass Difference [mDa]	Mass Difference [ppm]	DBE
296.26947	C17 H34 N3 O	296.26964	-0.17	-0.57	2.5

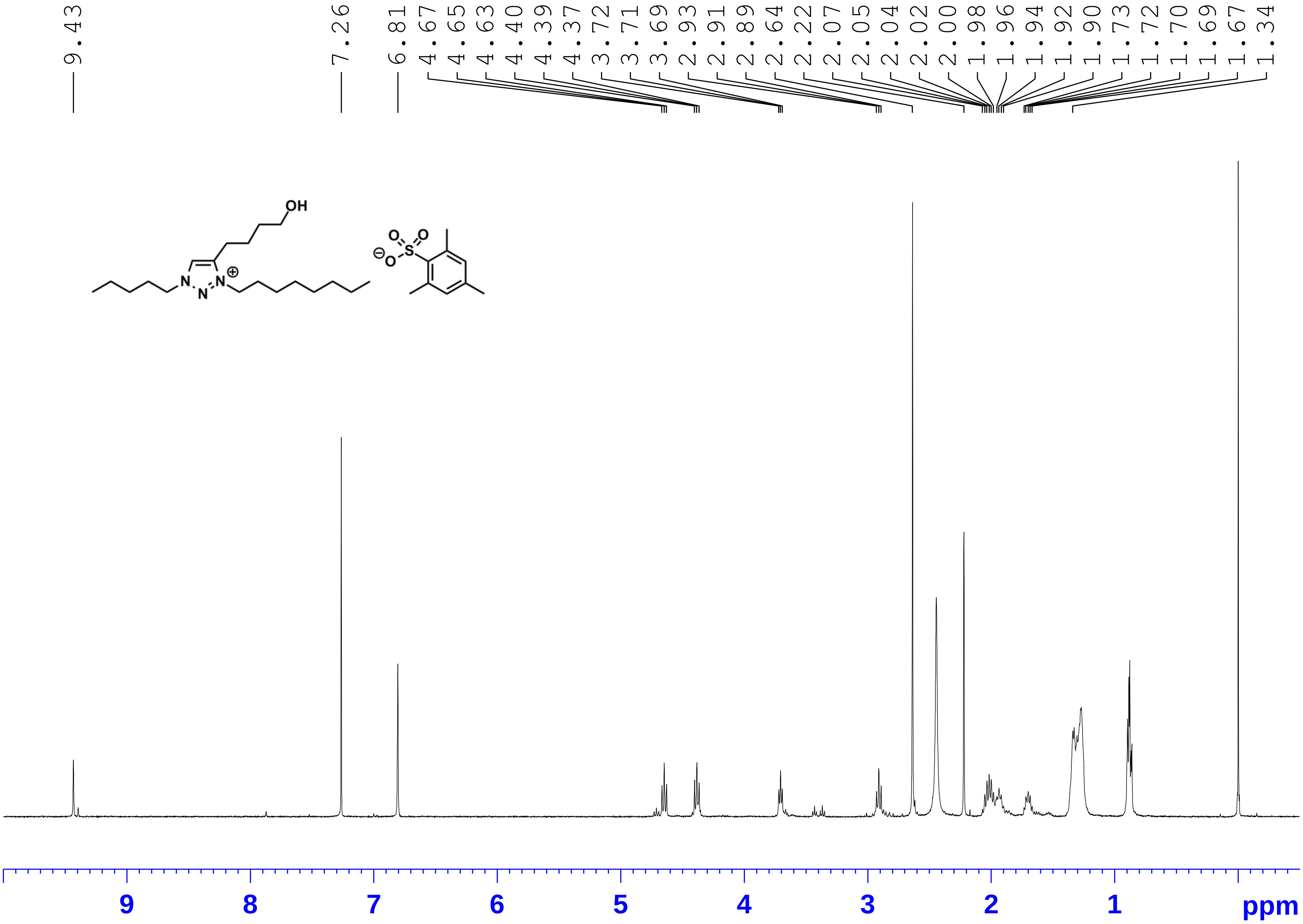

9.43
7.26
6.81
4.67
4.65
4.63
4.40
4.39
4.37
3.72
3.71
3.69
2.93
2.91
2.89
2.64
2.22
2.07
2.05
2.04
2.02
2.00
1.98
1.96
1.94
1.92
1.90
1.73
1.72
1.70
1.69
1.67
1.34
OH
N
N
N
O
O
S
O
9
8
7
6
5
4
3
2
1
ppm

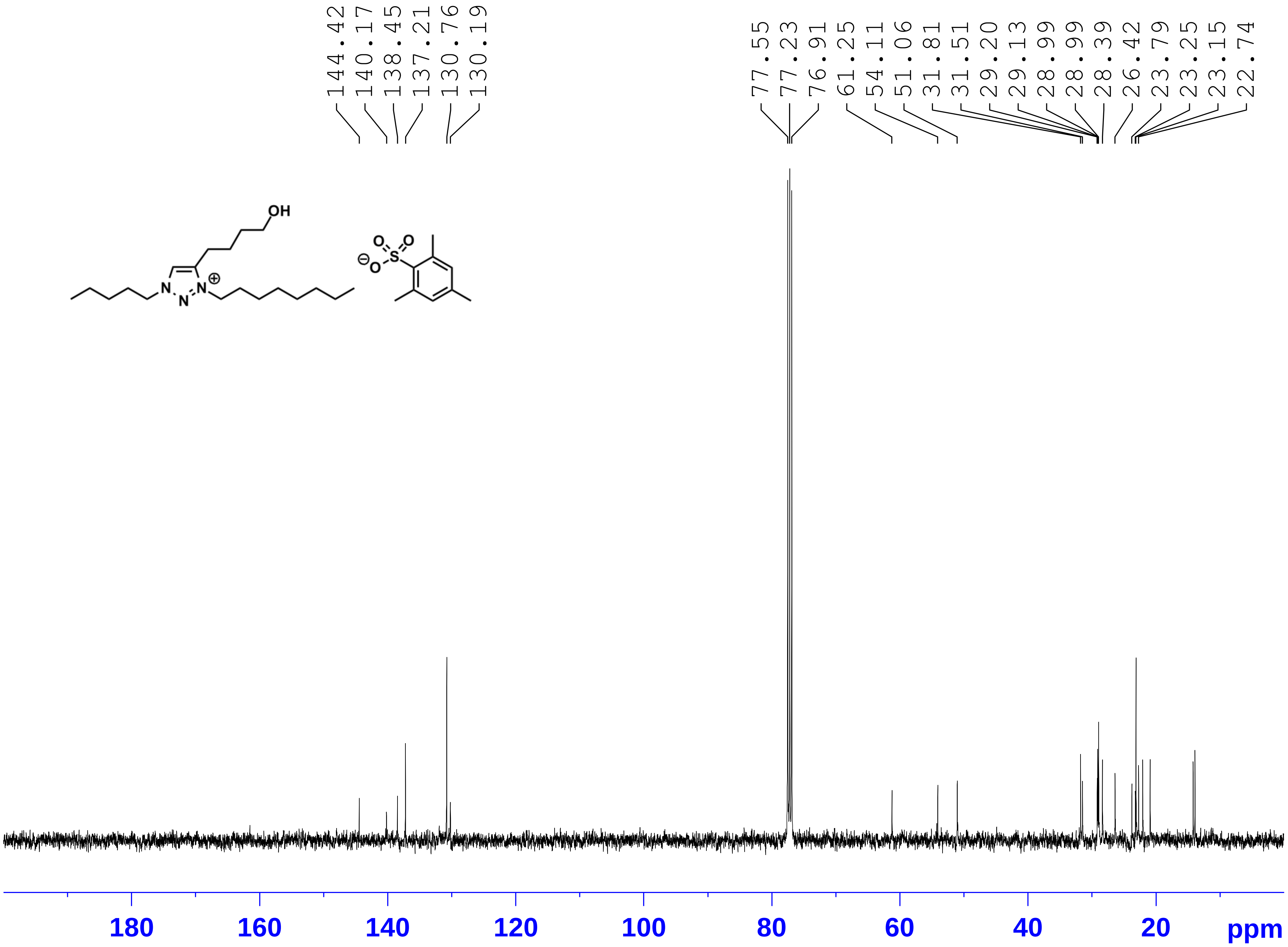
144.42
140.17
138.45
137.21
130.76
130.19
77.55
77.23
76.91
61.25
54.11
51.06
31.81
31.51
29.20
29.13
28.99
28.99
28.39
26.42
23.79
23.25
23.15
22.74
OH
N
N
N
⊕
⊖
O
S
O
O
180
160
140
120
100
80
60
40
20
ppm

Spectrum

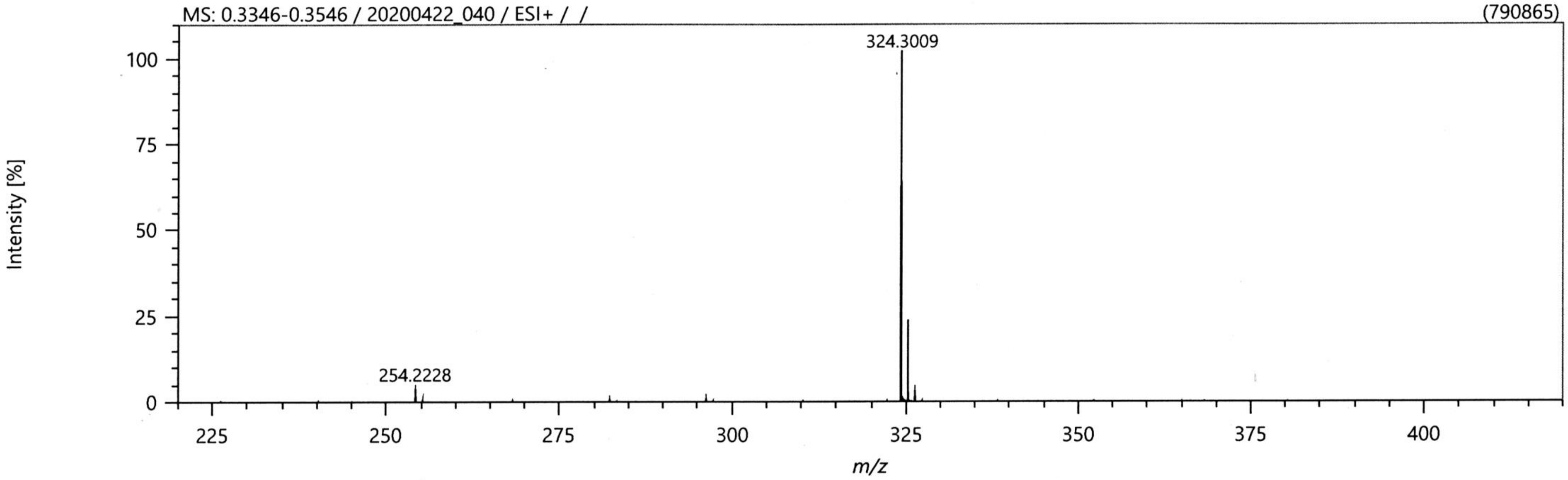

Elemental Composition

Parameters		Elements Set 1:						
Tolerance:	±10.00 ppm	Symbol	C	H	O	Na	N	F
Electron:	Odd/Even	Min	0	0	1	0	3	0
Charge:	+1	Max	1000	4000	1	0	3	0
DBE:	-99.0 - 999.0							

Results

Mass	Formula	Calculated Mass	Mass Difference [mDa]	Mass Difference [ppm]	DBE
324.30093	C19 H38 N3 O	324.30094	-0.01	-0.02	2.5

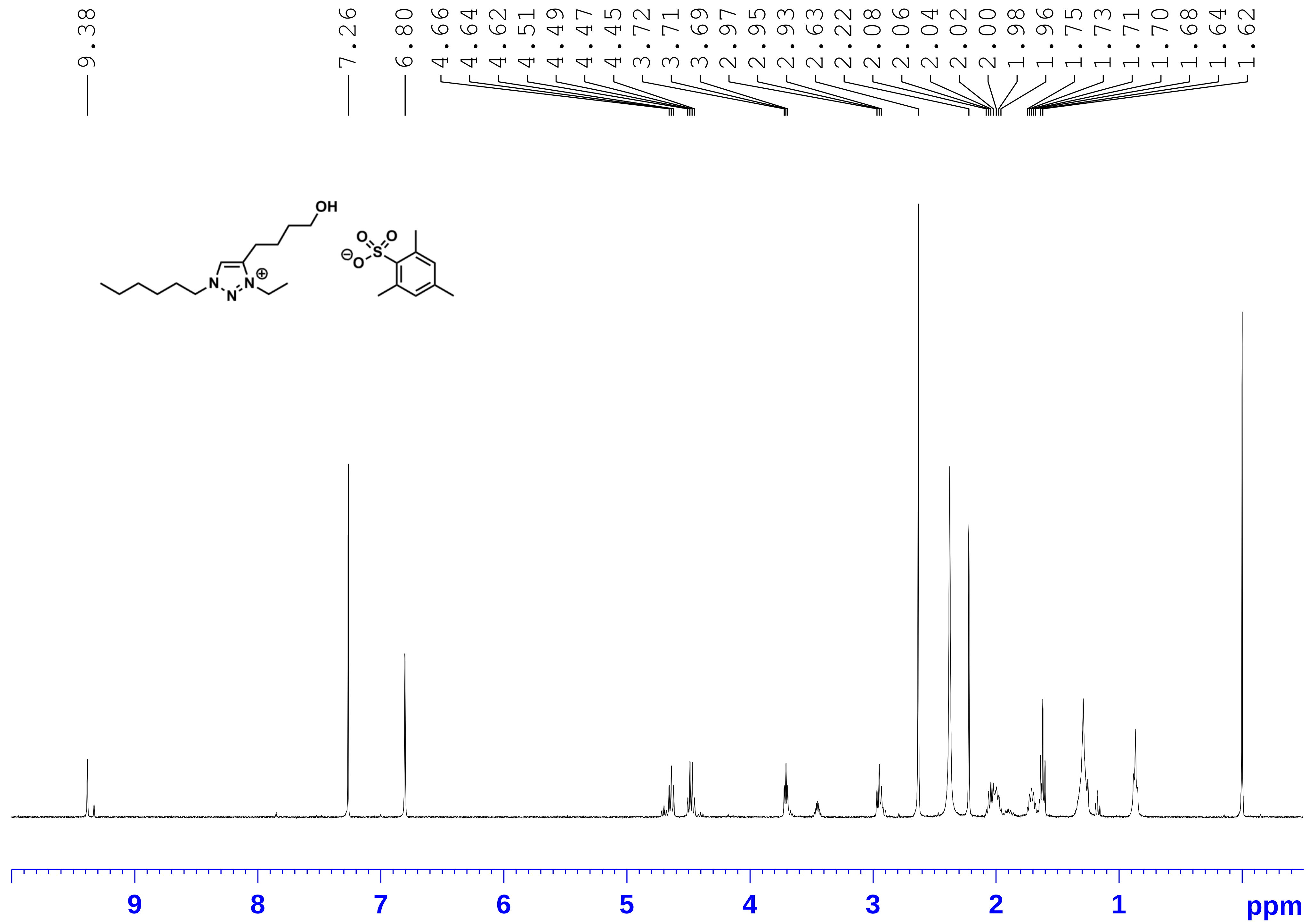
9.38
7.26
6.80
4.66
4.64
4.62
4.51
4.49
4.47
4.45
3.72
3.71
3.69
2.97
2.95
2.93
2.63
2.22
2.08
2.06
2.04
2.02
2.00
1.98
1.96
1.75
1.73
1.71
1.70
1.68
1.64
1.62
OH
9
8
7
6
5
4
3
2
1
ppm

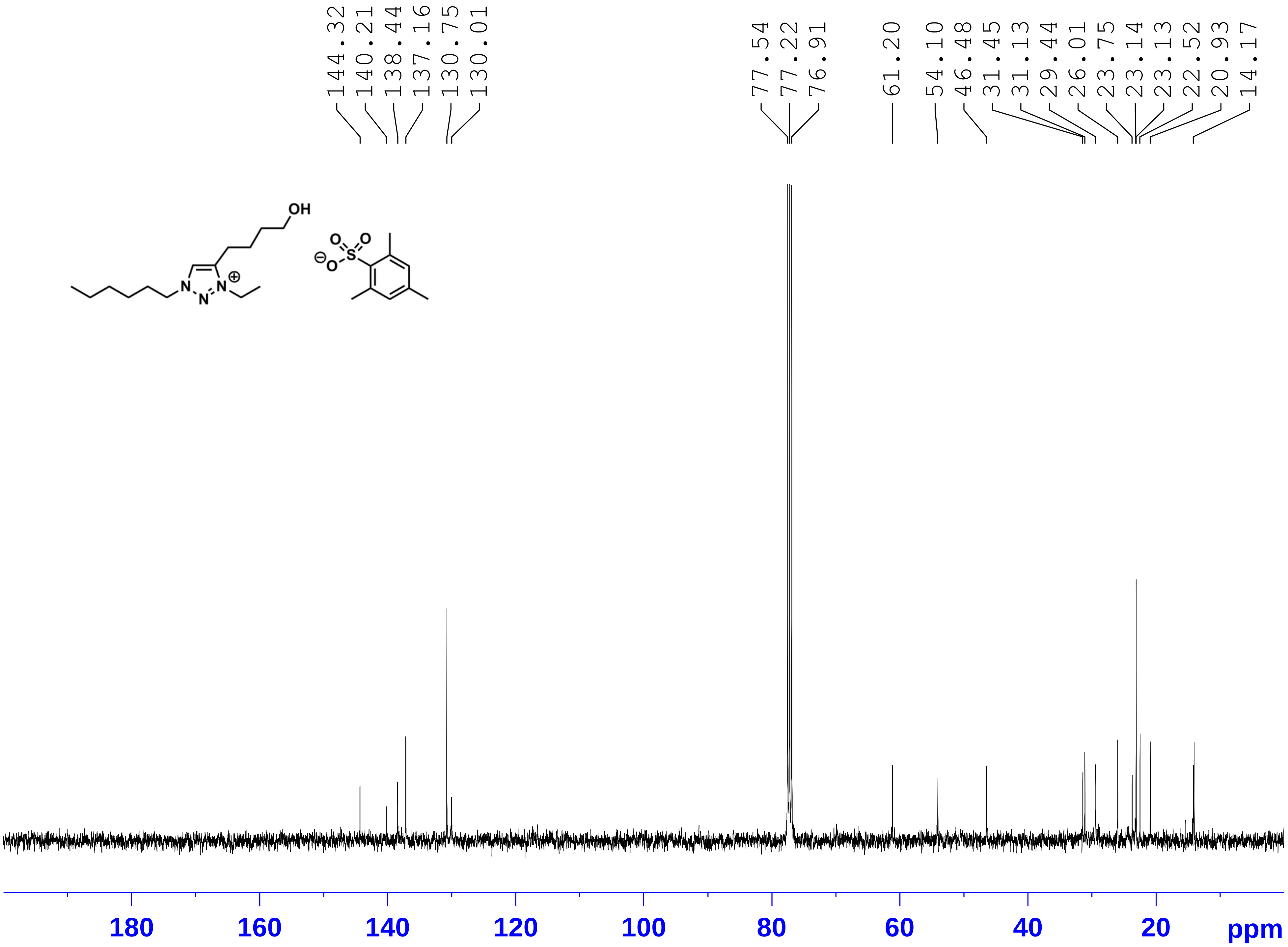
144.32
140.21
138.44
137.16
130.75
130.01
77.54
77.22
76.91
61.20
54.10
46.48
31.45
31.13
29.44
26.01
23.75
23.14
23.13
22.52
20.93
14.17
OH
180
160
140
120
100
80
60
40
20
ppm

Spectrum

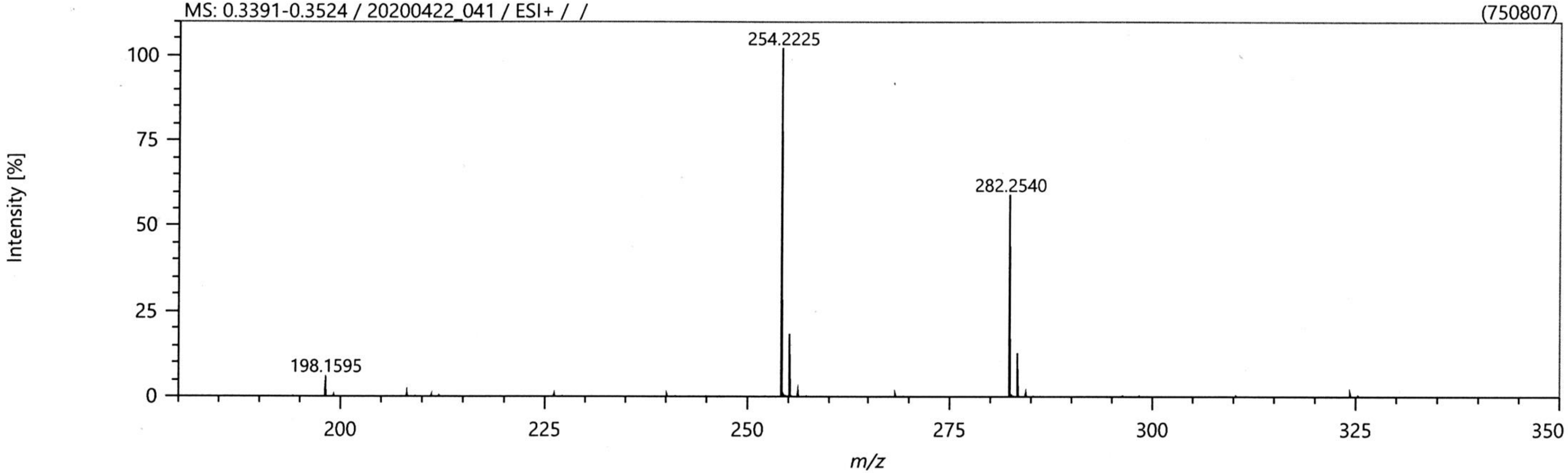

Elemental Composition

Parameters		Elements Set 1:						
Tolerance:	±10.00 ppm	Symbol	C	H	O	Na	N	F
Electron:	Odd/Even	Min	0	0	1	0	3	0
Charge:	+1	Max	1000	4000	1	0	3	0
DBE:	-99.0 - 999.0							

Results

Mass	Formula	Calculated Mass	Mass Difference [mDa]	Mass Difference [ppm]	DBE
254.22254	C14 H28 N3 O	254.22269	-0.15	-0.58	2.5

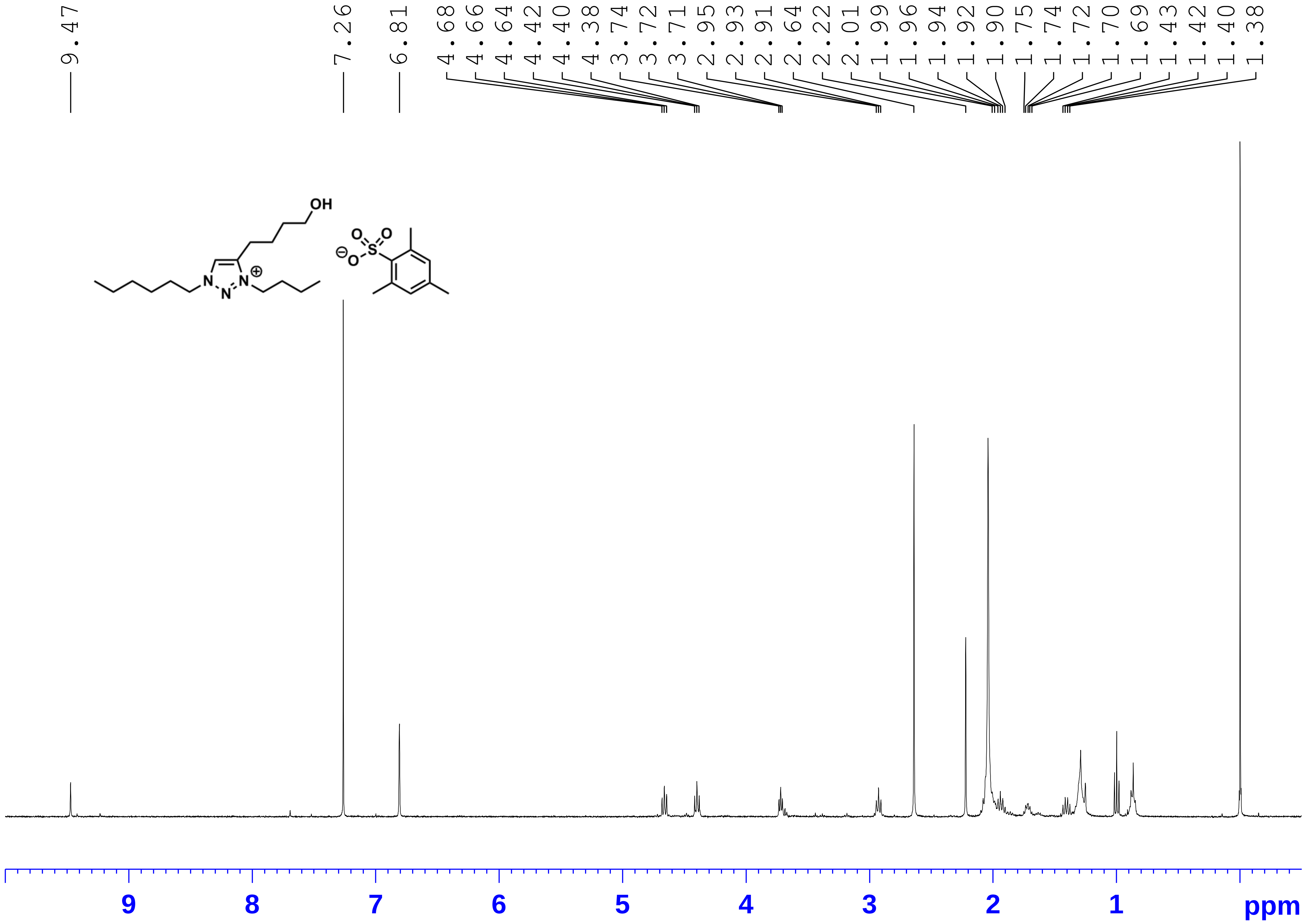
9.47
7.26
6.81
4.68
4.66
4.64
4.42
4.40
4.38
3.74
3.72
3.71
2.95
2.93
2.91
2.64
2.22
2.01
1.99
1.96
1.94
1.92
1.90
1.75
1.74
1.72
1.70
1.69
1.43
1.42
1.40
1.38
OH
9
8
7
6
5
4
3
2
1
ppm

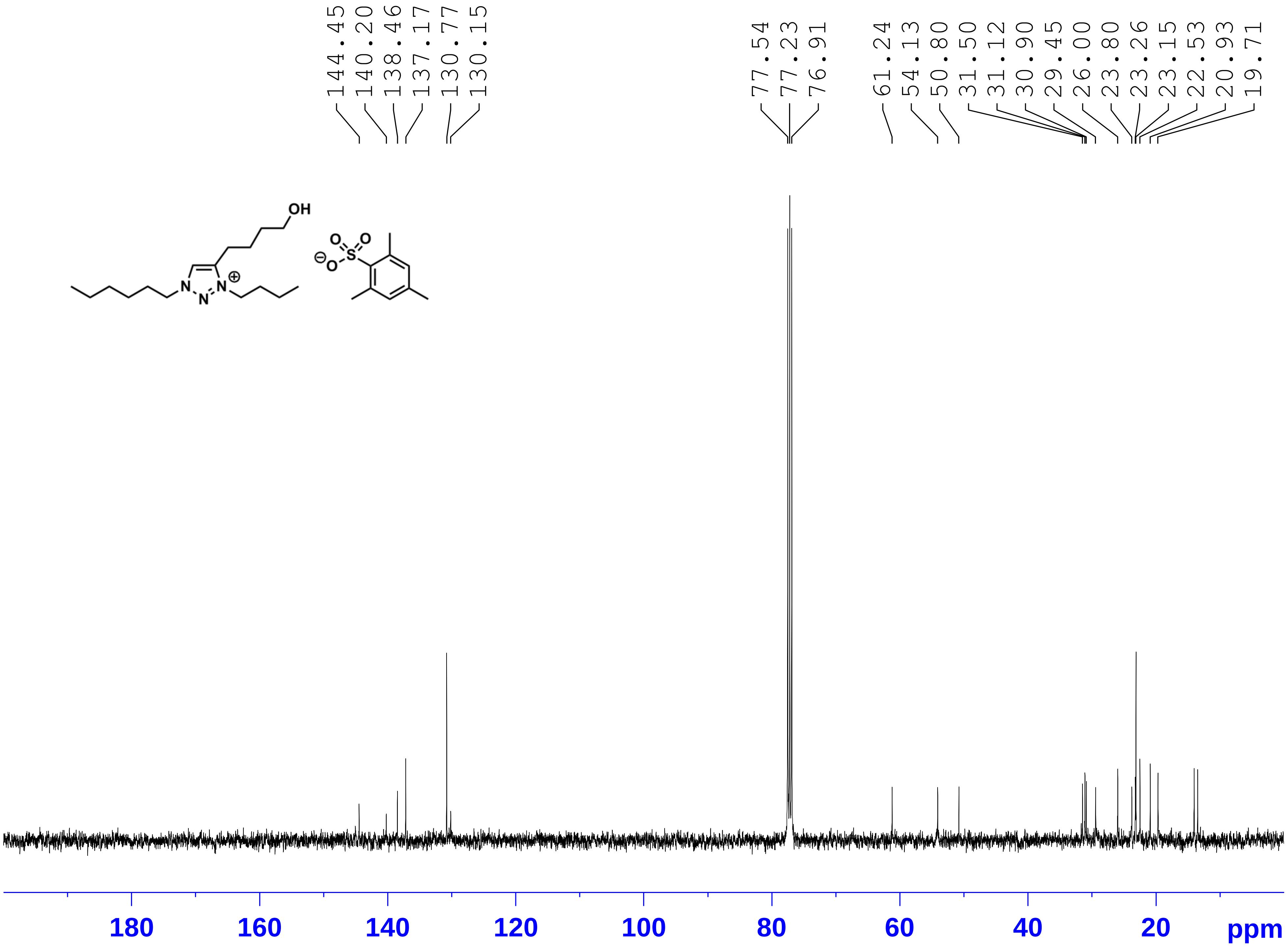
144.45
140.20
138.46
137.17
130.77
130.15
77.54
77.23
76.91
61.24
54.13
50.80
31.50
31.12
30.90
29.45
26.00
23.80
23.26
23.15
22.53
20.93
19.71
OH
O
O
S
O
N
N
N
180
160
140
120
100
80
60
40
20
ppm

Spectrum

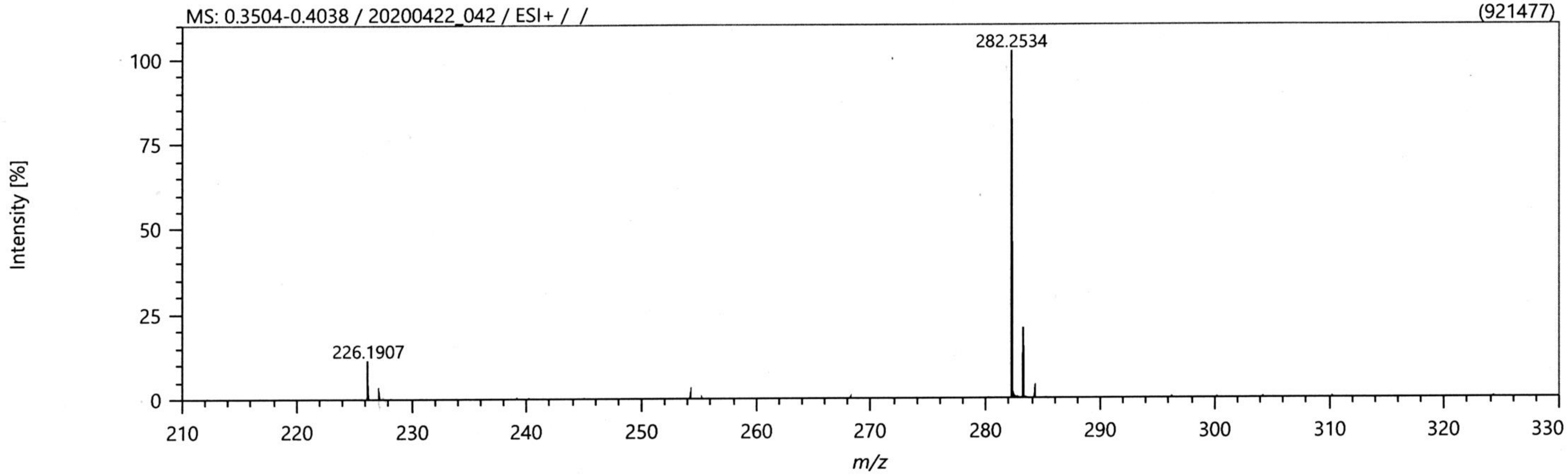

Elemental Composition

Parameters

Tolerance:	±10.00 ppm
Electron:	Odd/Even
Charge:	+1
DBE:	-99.0 - 999.0

Elements Set 1:

Symbol	C	H	O	Na	N	F
Min	0	0	1	0	3	0
Max	1000	4000	1	0	3	0

Results

Mass	Formula	Calculated Mass	Mass Difference [mDa]	Mass Difference [ppm]	DBE
282.25341	C16 H32 N3 O	282.25399	-0.58	-2.05	2.5

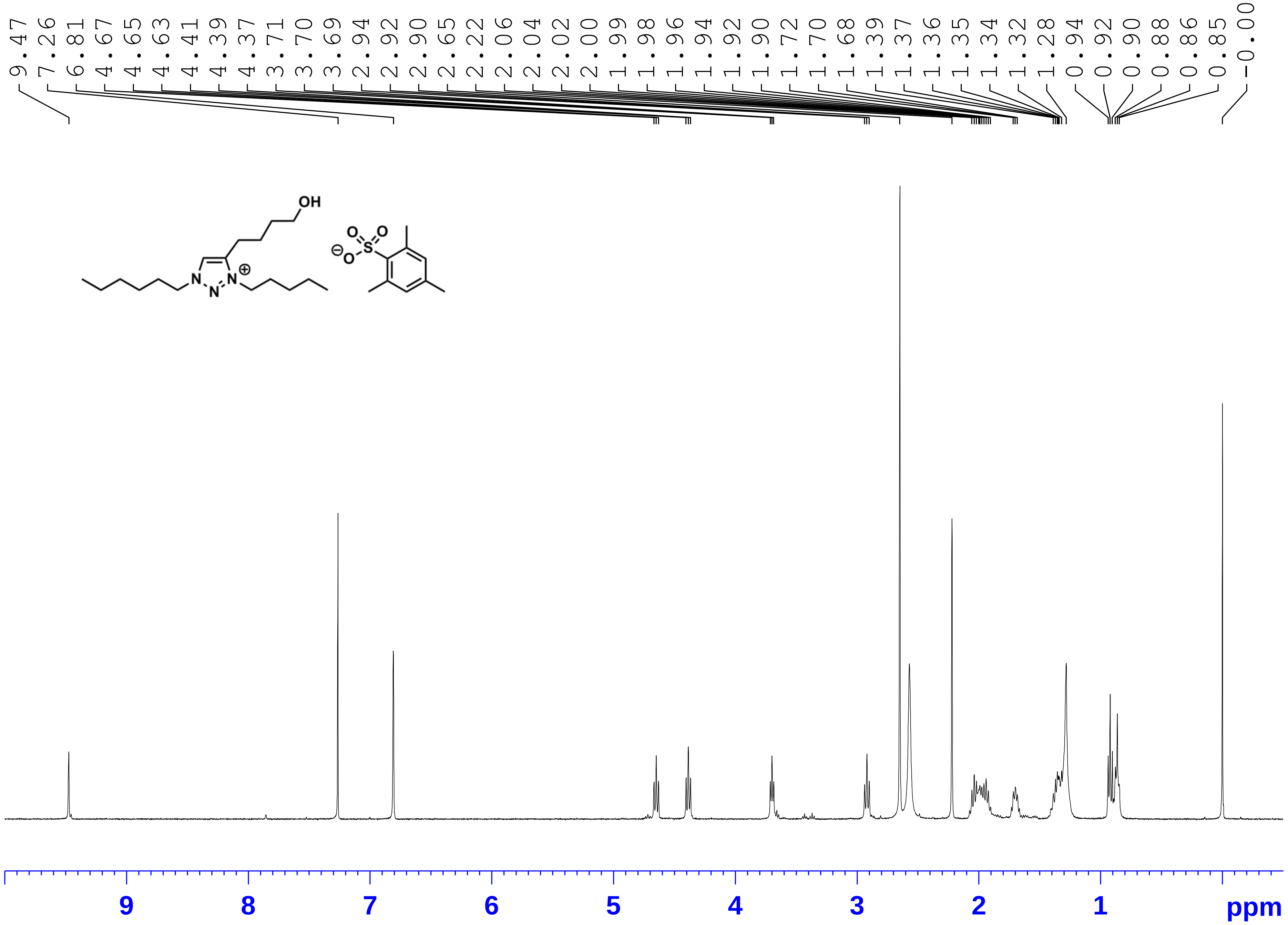

9.47
7.26
6.81
4.67
4.65
4.63
4.41
4.39
4.37
3.71
3.70
3.69
2.94
2.92
2.90
2.65
2.22
2.06
2.04
2.02
2.00
1.99
1.98
1.96
1.94
1.92
1.90
1.72
1.70
1.68
1.39
1.37
1.36
1.35
1.34
1.32
1.28
0.94
0.92
0.90
0.88
0.86
0.85
-0.00
OH
N
N
N
O
S
O
O
9
8
7
6
5
4
3
2
1
ppm

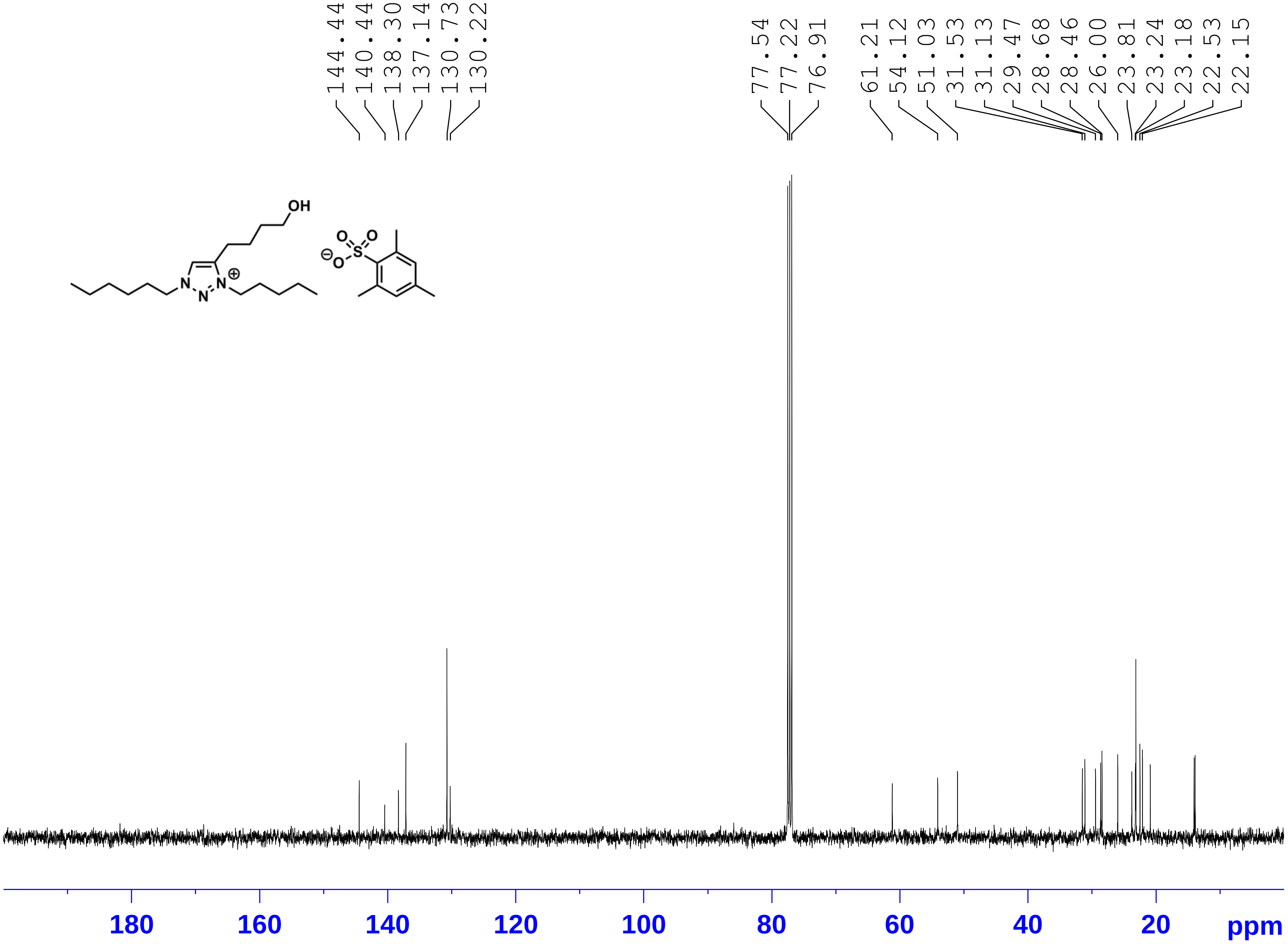
144.44
140.44
138.30
137.14
130.73
130.22
77.54
77.22
76.91
61.21
54.12
51.03
31.53
31.13
29.47
28.68
28.46
26.00
23.81
23.24
23.18
22.53
22.15
OH
N
N
N
O
O
S
O
180
160
140
120
100
80
60
40
20
ppm

Spectrum

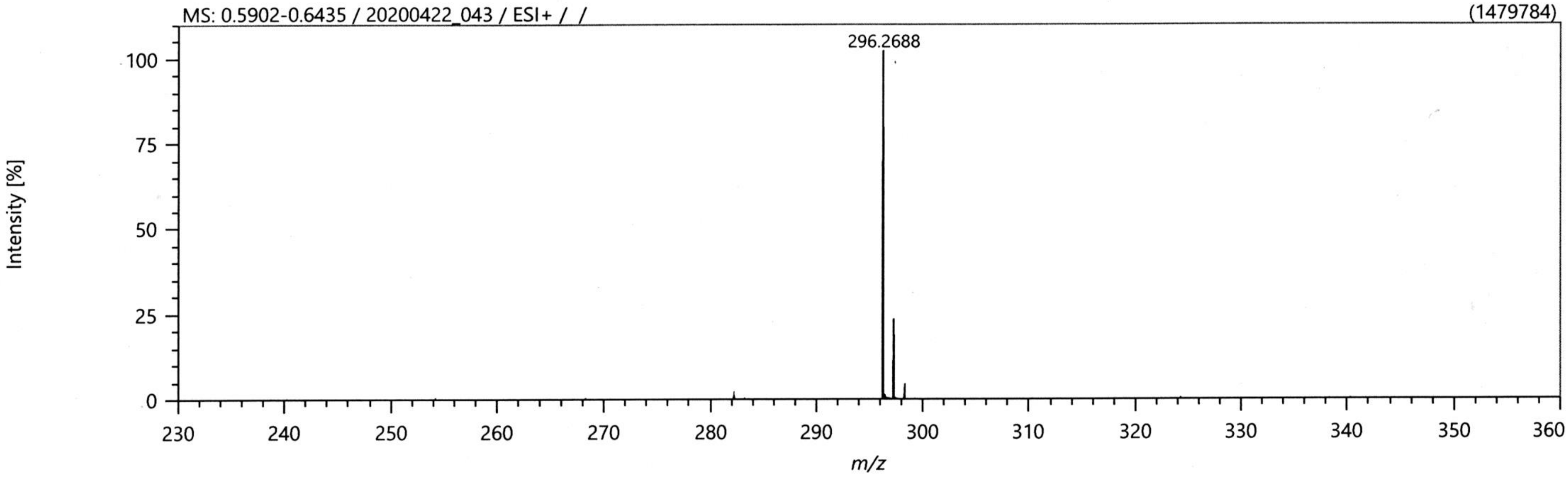

Elemental Composition

Parameters		Elements Set 1:						
Tolerance:	±10.00 ppm	Symbol	C	H	O	Na	N	F
Electron:	Odd/Even	Min	0	0	1	0	3	0
Charge:	+1	Max	1000	4000	1	0	3	0
DBE:	-99.0 - 999.0							

Results

Mass	Formula	Calculated Mass	Mass Difference [mDa]	Mass Difference [ppm]	DBE
296.26876	C17 H34 N3 O	296.26964	-0.87	-2.95	2.5

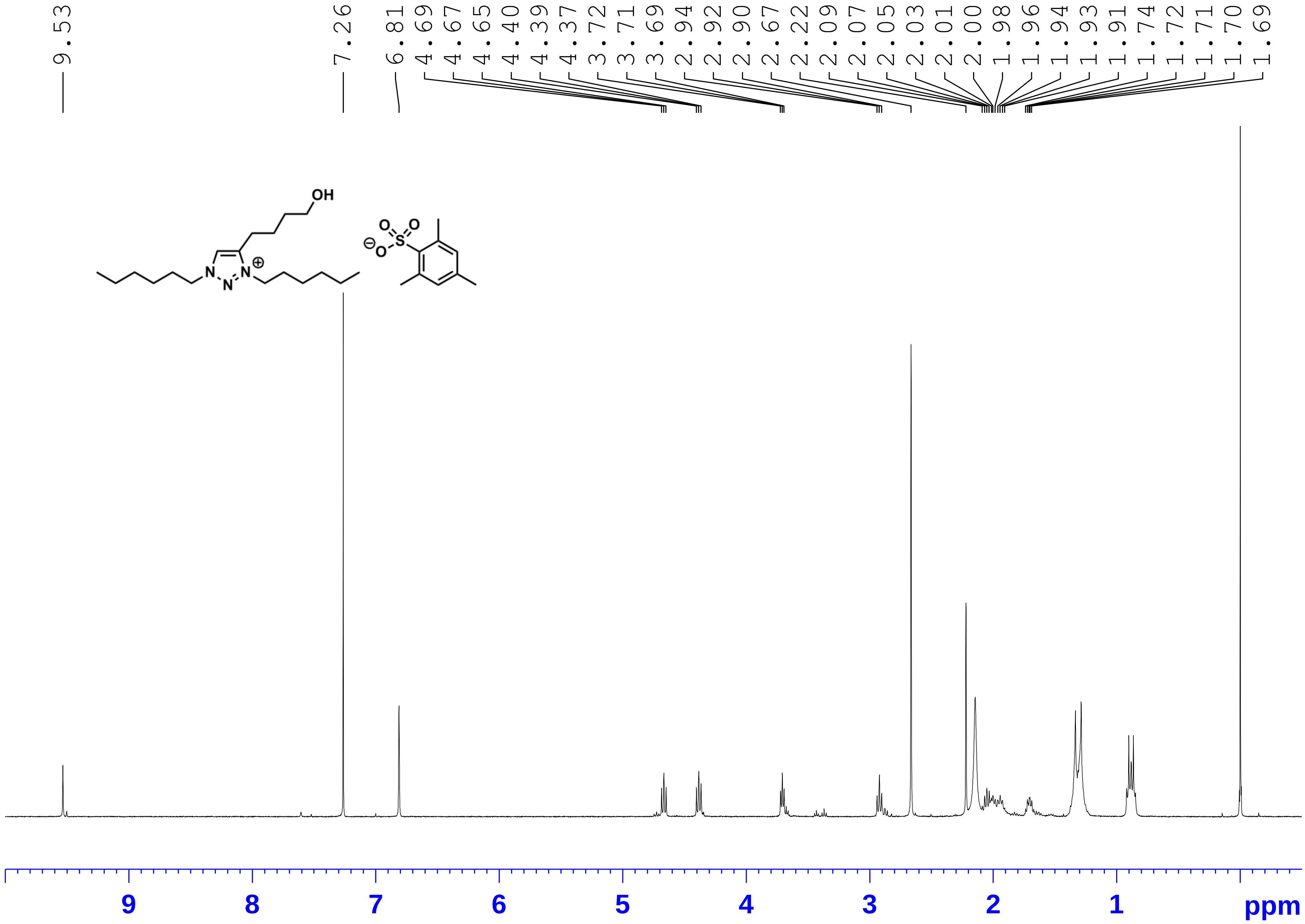

9.53
7.26
6.81
4.69
4.67
4.65
4.40
4.39
4.37
3.72
3.71
3.69
2.94
2.92
2.90
2.67
2.22
2.09
2.07
2.05
2.03
2.01
2.00
1.98
1.96
1.94
1.93
1.91
1.74
1.72
1.71
1.70
1.69
OH
O
O
S
O
N
N
N
9
8
7
6
5
4
3
2
1
ppm

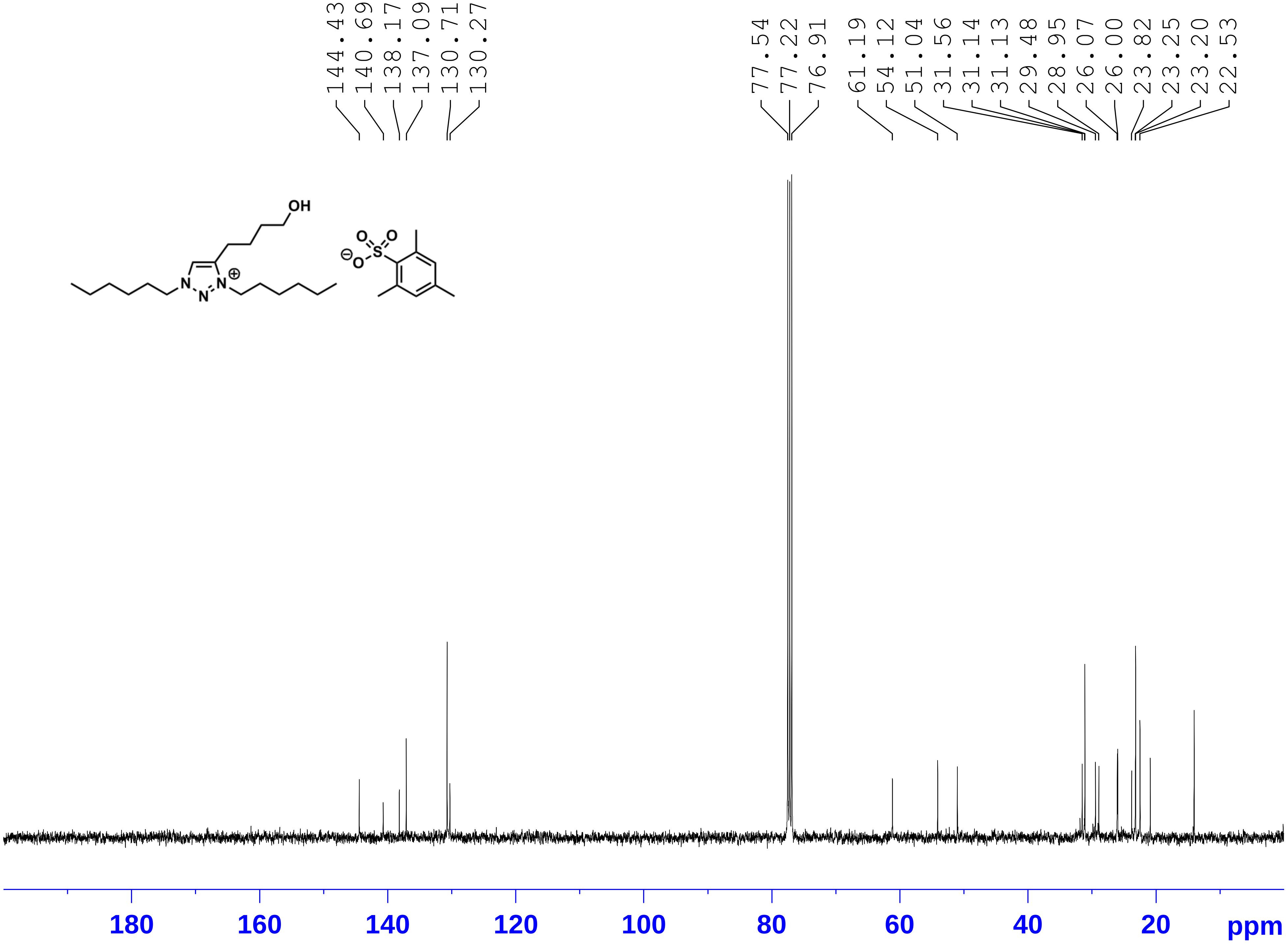
144.43
140.69
138.17
137.09
130.71
130.27
77.54
77.22
76.91
61.19
54.12
51.04
31.56
31.14
31.13
29.48
28.95
26.07
26.00
23.82
23.25
23.20
22.53
OH
N
N
N
S
O
O
O
180
160
140
120
100
80
60
40
20
ppm

Spectrum

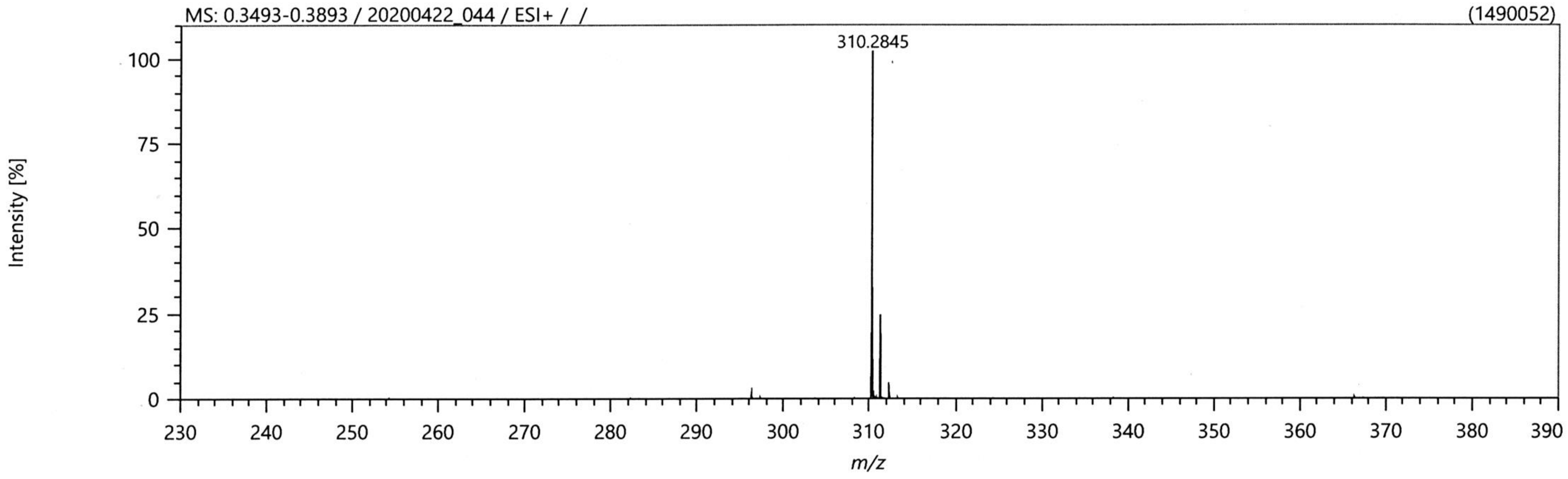

Elemental Composition

Parameters		Elements Set 1:						
Tolerance:	±10.00 ppm	Symbol	C	H	O	Na	N	F
Electron:	Odd/Even	Min	0	0	1	0	3	0
Charge:	+1	Max	1000	4000	1	0	3	0
DBE:	-99.0 - 999.0							

Results

Mass	Formula	Calculated Mass	Mass Difference [mDa]	Mass Difference [ppm]	DBE
310.28455	C18 H36 N3 O	310.28529	-0.74	-2.39	2.5

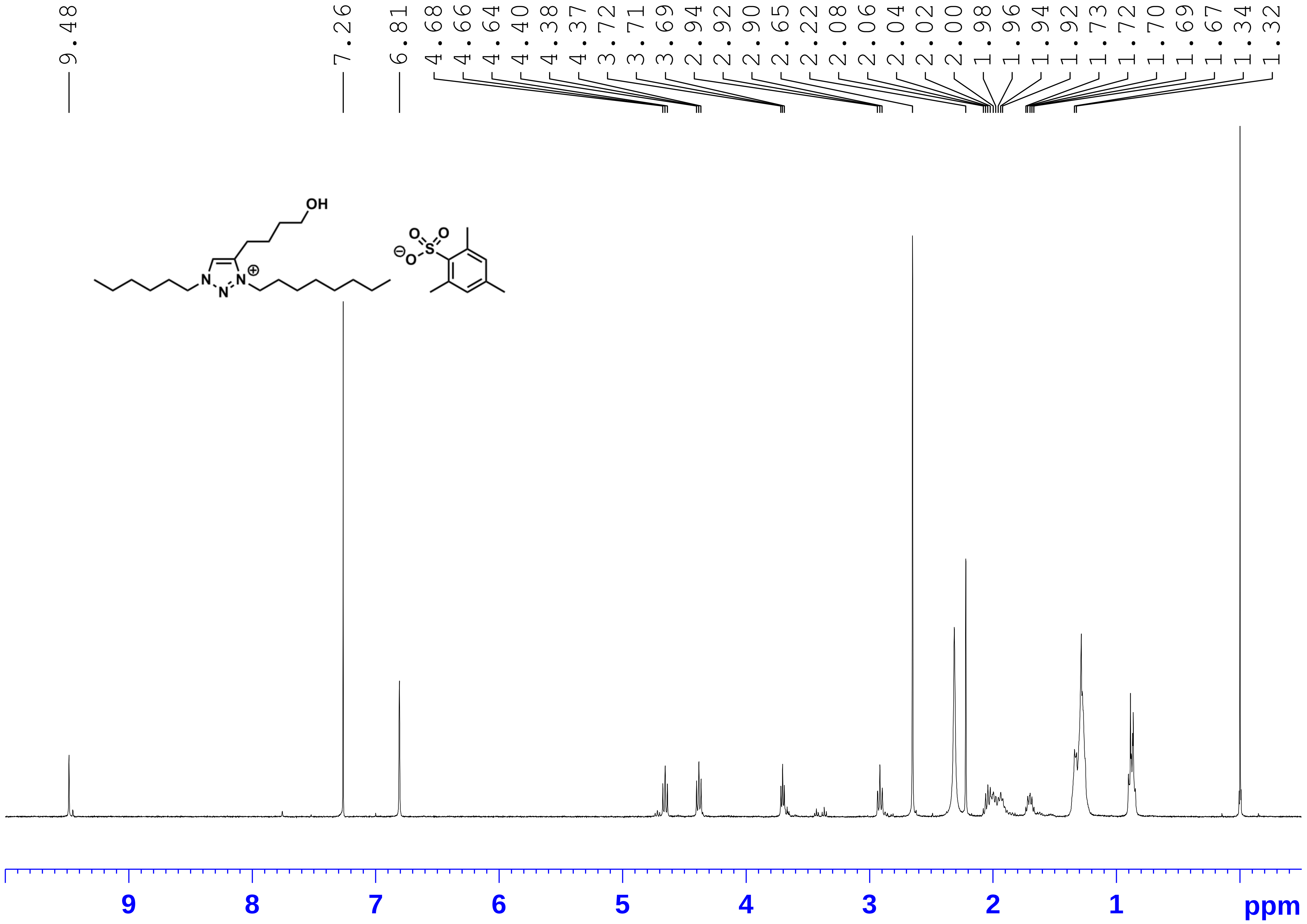

9.48
7.26
6.81
4.68
4.66
4.64
4.40
4.38
4.37
3.72
3.71
3.69
2.94
2.92
2.90
2.65
2.22
2.08
2.06
2.04
2.02
2.00
1.98
1.96
1.94
1.92
1.73
1.72
1.70
1.69
1.67
1.34
1.32
OH
N
N
N
O
S
O
O
9
8
7
6
5
4
3
2
1
ppm

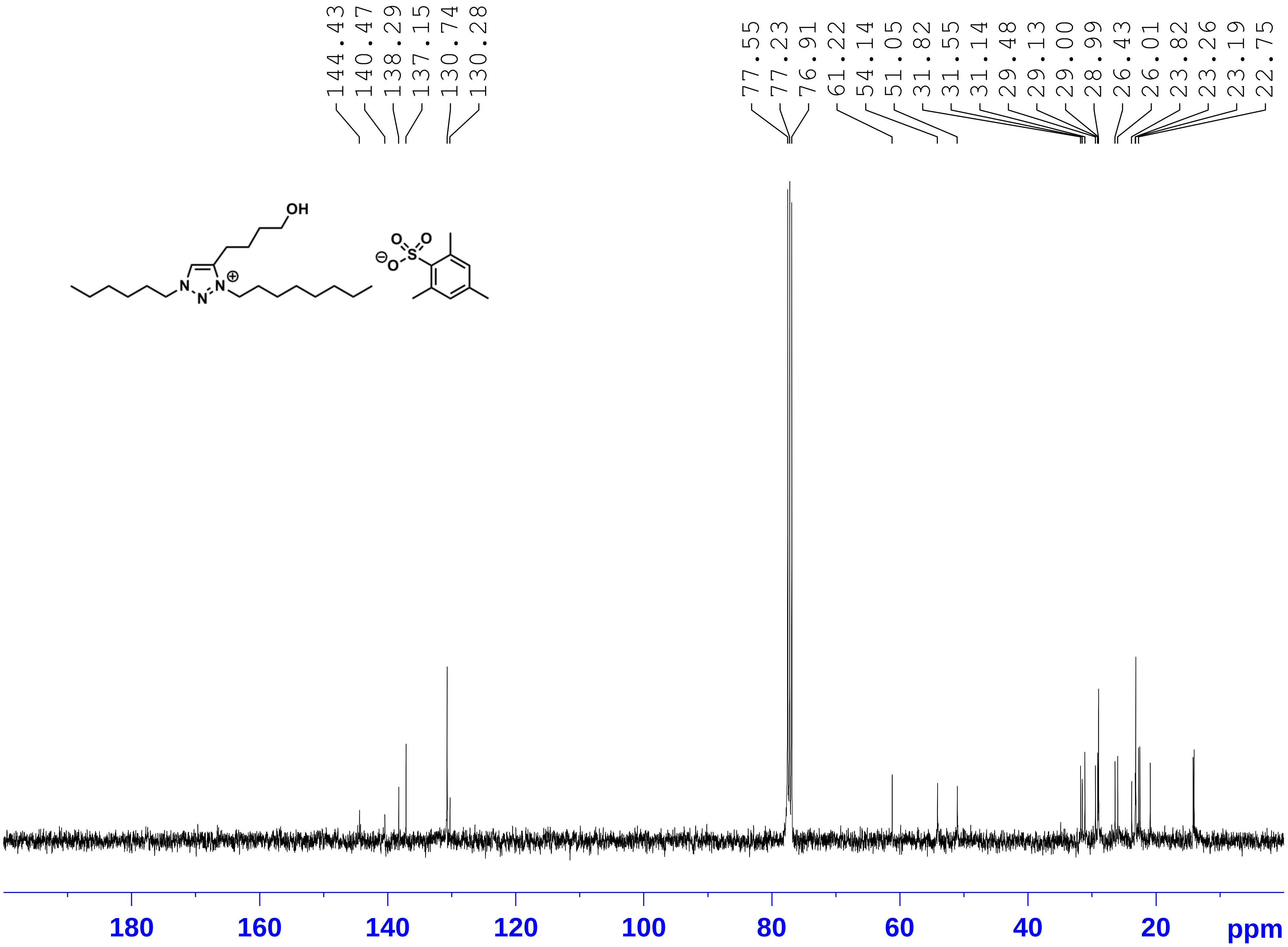

144.43
140.47
138.29
137.15
130.74
130.28
77.55
77.23
76.91
61.22
54.14
51.05
31.82
31.55
31.14
29.48
29.13
29.00
28.99
26.43
26.01
23.82
23.26
23.19
22.75
OH
N
N
N
O
S
180
160
140
120
100
80
60
40
20
ppm

Spectrum

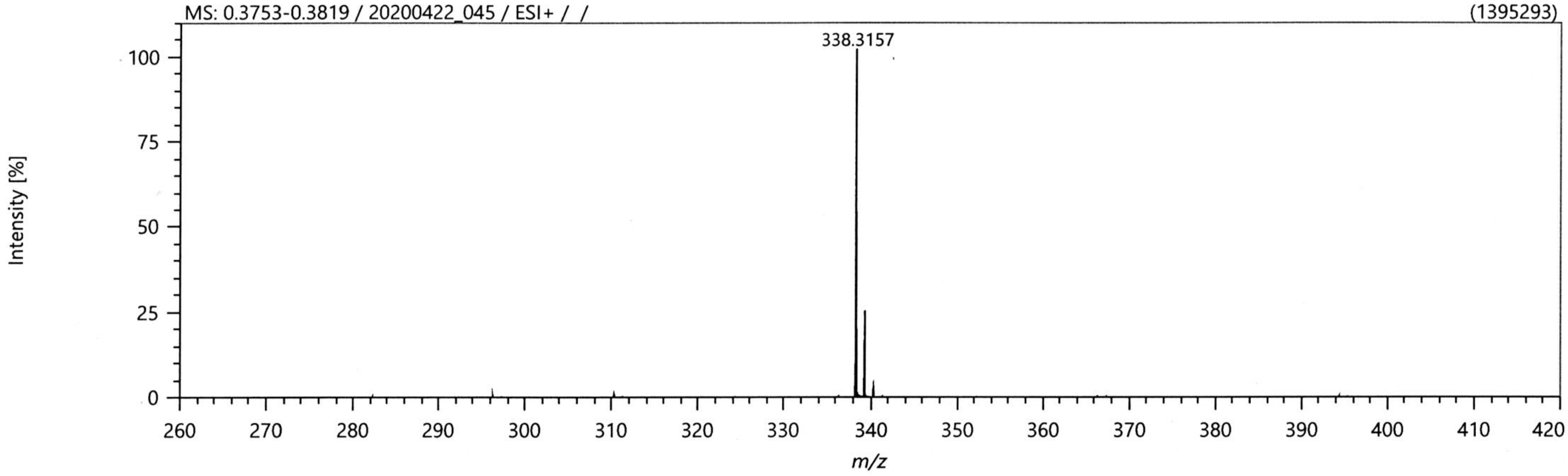

Elemental Composition

Parameters		Elements Set 1:						
Tolerance:	±10.00 ppm	Symbol	C	H	O	Na	N	F
Electron:	Odd/Even	Min	0	0	1	0	3	0
Charge:	+1	Max	1000	4000	1	0	3	0
DBE:	-99.0 - 999.0							

Results

Mass	Formula	Calculated Mass	Mass Difference [mDa]	Mass Difference [ppm]	DBE
338.31573	C20 H40 N3 O	338.31659	-0.86	-2.55	2.5

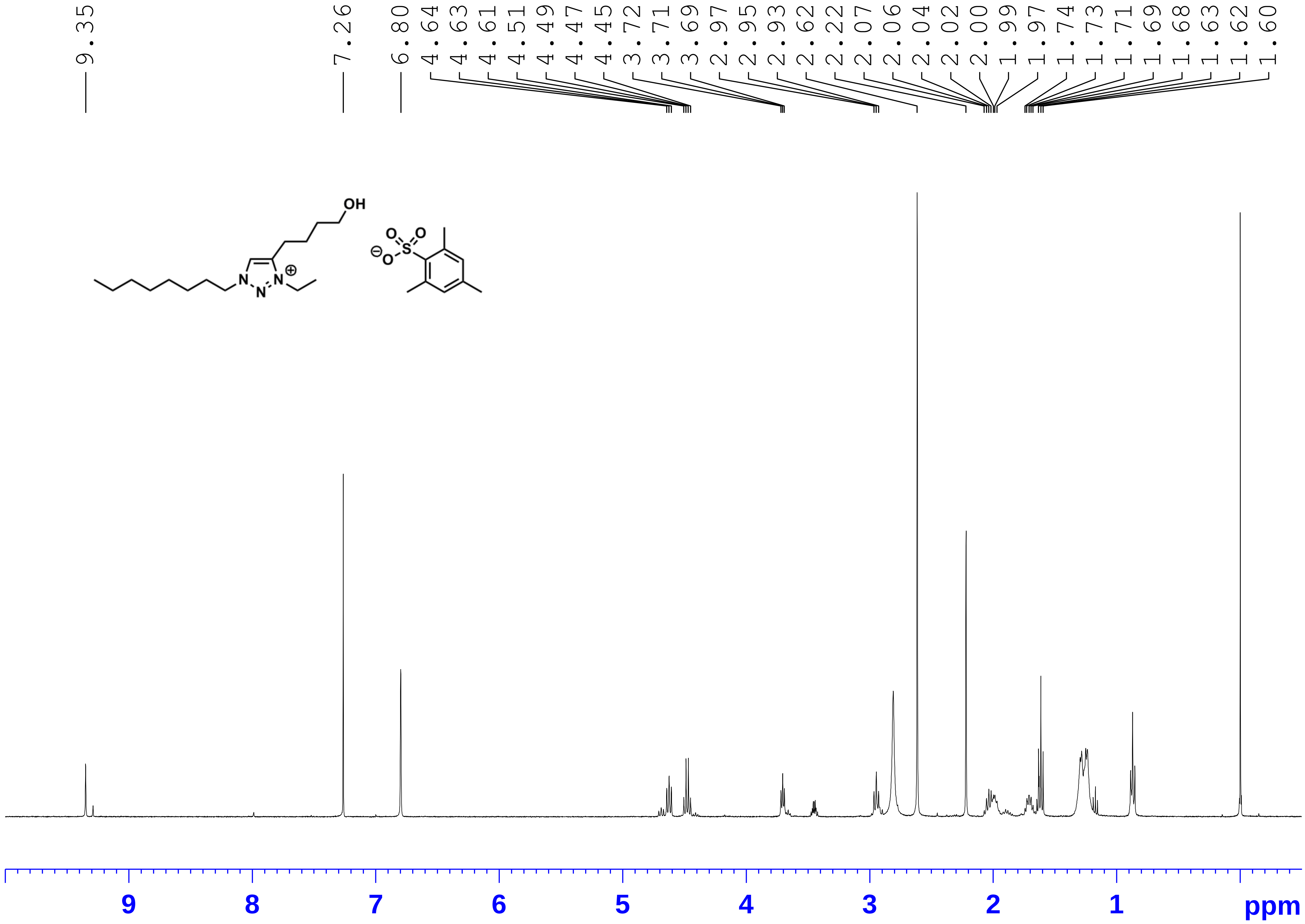
9.35
7.26
6.80
4.64
4.63
4.61
4.51
4.49
4.47
4.45
3.72
3.71
3.69
2.97
2.95
2.93
2.62
2.22
2.07
2.06
2.04
2.02
2.00
1.99
1.97
1.74
1.73
1.71
1.69
1.68
1.63
1.62
1.60
OH
N
N
N
O
O
S
O
9
8
7
6
5
4
3
2
1
ppm

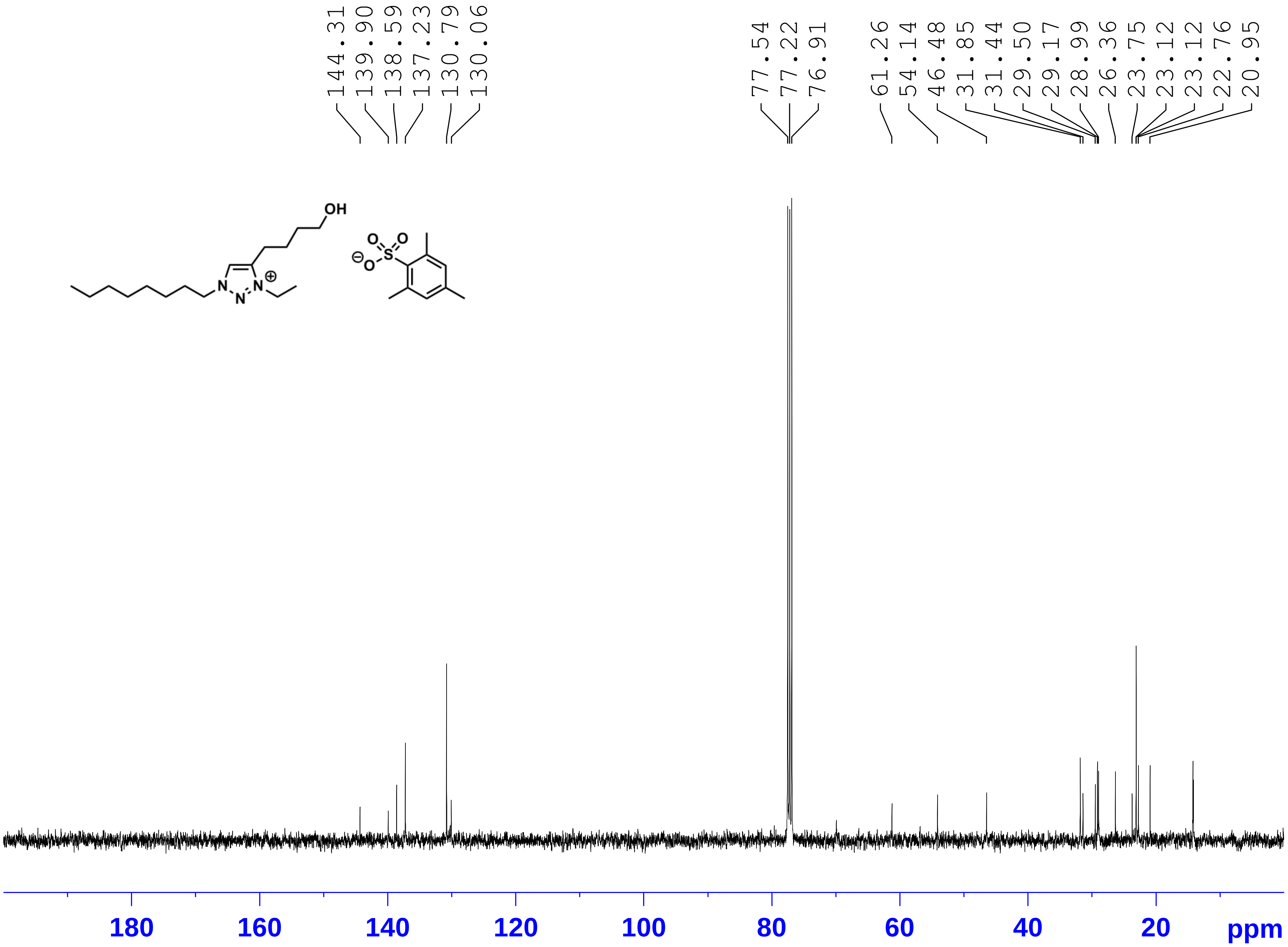

144.31
139.90
138.59
137.23
130.79
130.06
77.54
77.22
76.91
61.26
54.14
46.48
31.85
31.44
29.50
29.17
28.99
26.36
23.75
23.12
23.12
22.76
20.95
OH
O
S
O
O
N
N
N
180
160
140
120
100
80
60
40
20
ppm

Spectrum

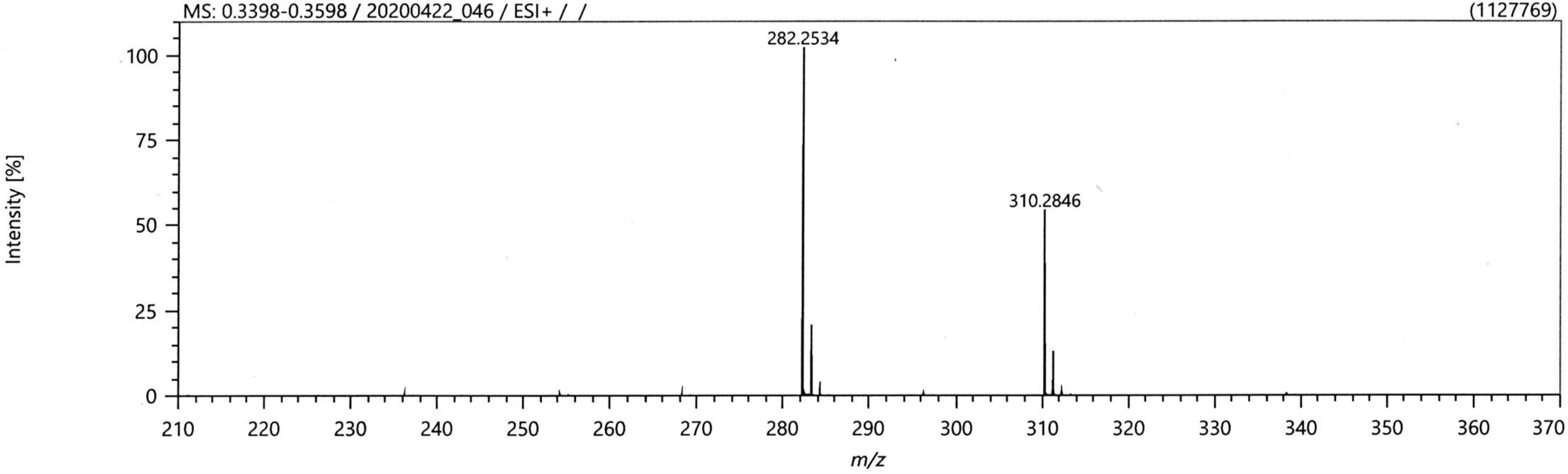

Elemental Composition

Parameters		Elements Set 1:						
Tolerance:	±10.00 ppm	Symbol	C	H	O	Na	N	F
Electron:	Odd/Even	Min	0	0	1	0	3	0
Charge:	+1	Max	1000	4000	1	0	3	0
DBE:	-99.0 - 999.0							

Results

Mass	Formula	Calculated Mass	Mass Difference [mDa]	Mass Difference [ppm]	DBE
282.25342	C16 H32 N3 O	282.25399	-0.57	-2.02	2.5

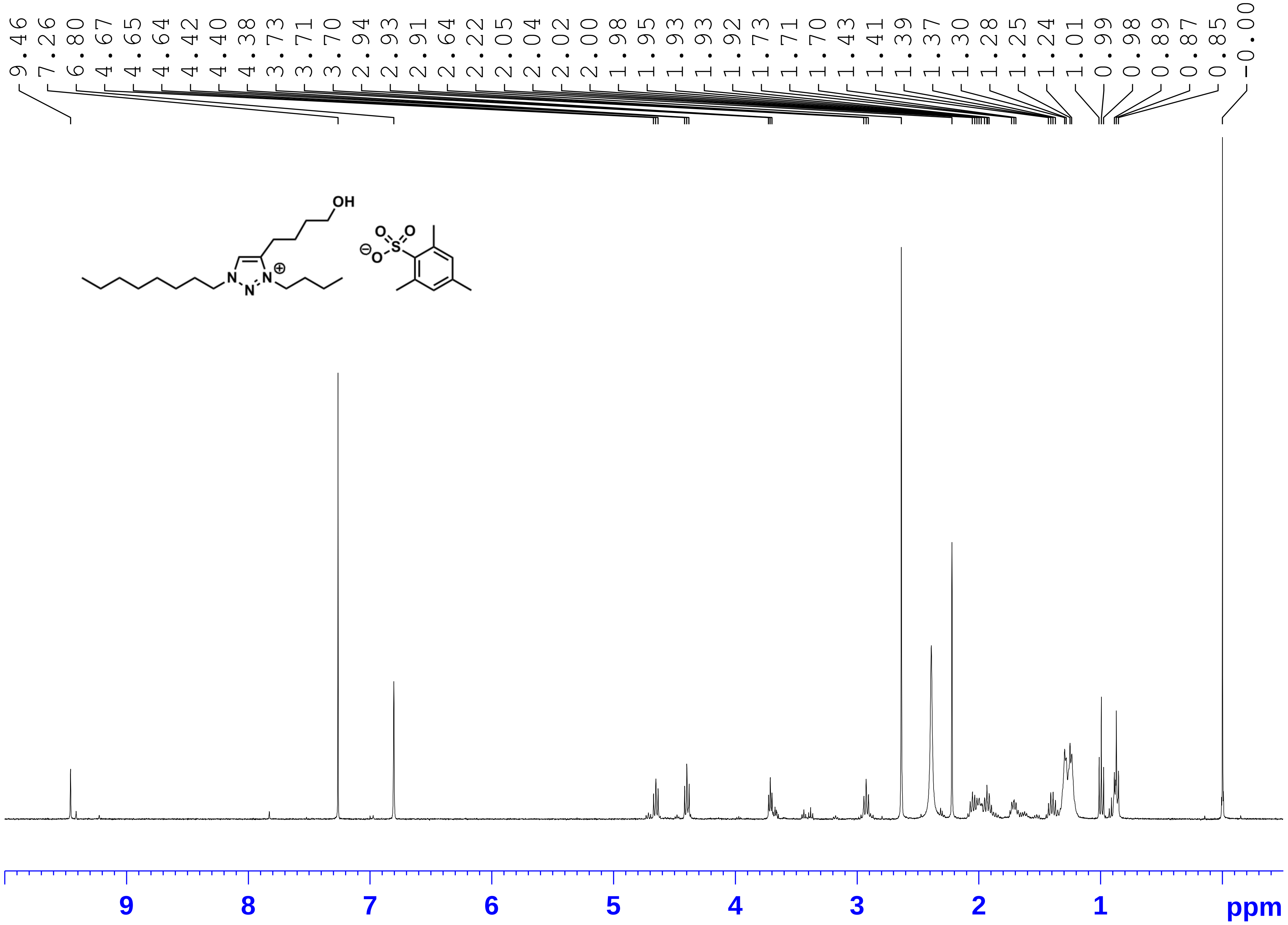
9.46
7.26
6.80
4.67
4.65
4.64
4.42
4.40
4.38
3.73
3.71
3.70
2.94
2.93
2.91
2.64
2.22
2.05
2.04
2.02
2.00
1.98
1.95
1.93
1.93
1.92
1.73
1.71
1.70
1.43
1.41
1.39
1.37
1.30
1.28
1.25
1.24
1.01
0.99
0.98
0.89
0.87
0.85
-0.00
OH
O
S
N
9
8
7
6
5
4
3
2
1
ppm

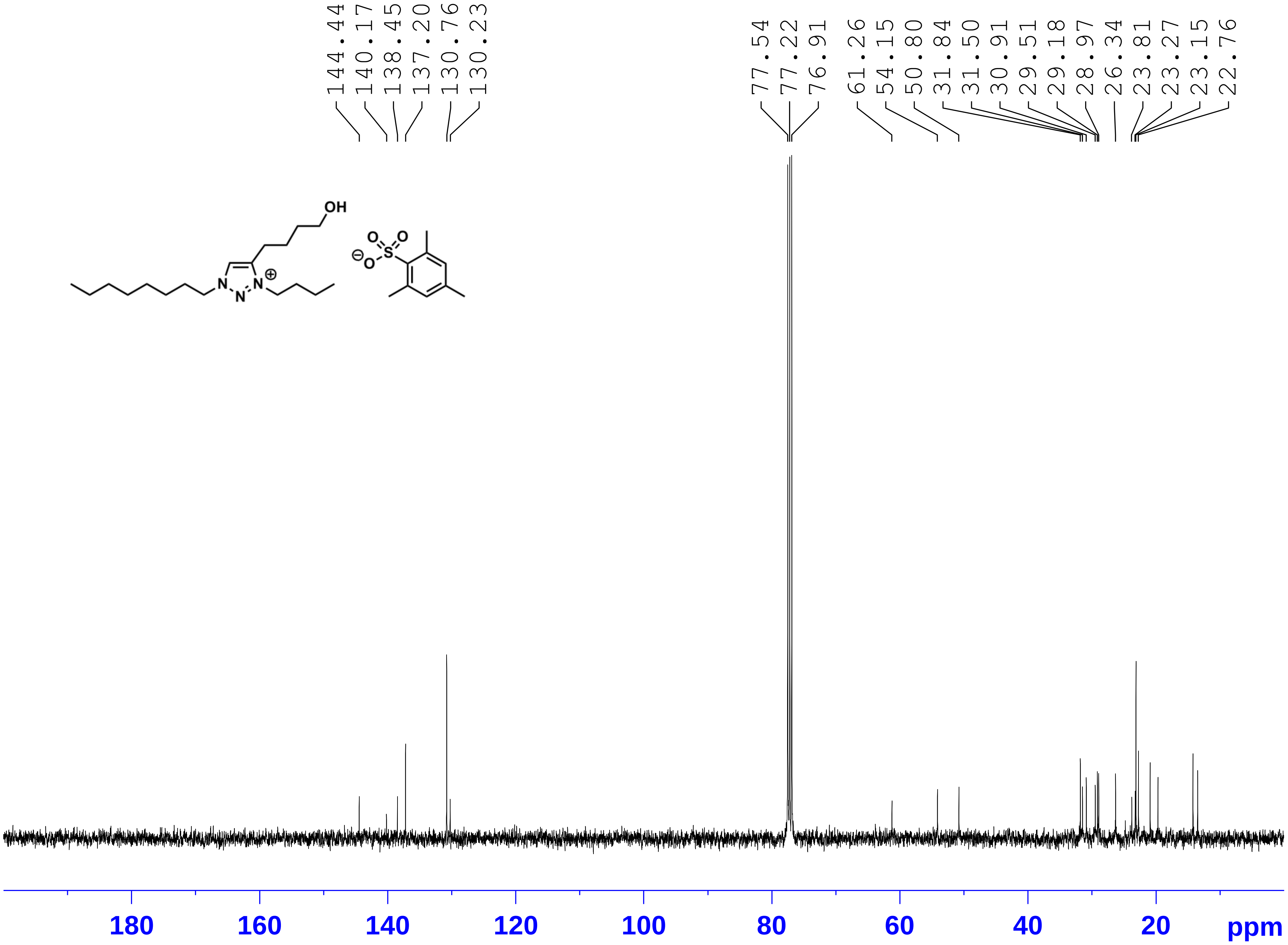

144.44
140.17
138.45
137.20
130.76
130.23
77.54
77.22
76.91
61.26
54.15
50.80
31.84
31.50
30.91
29.51
29.18
28.97
26.34
23.81
23.27
23.15
22.76
OH
180
160
140
120
100
80
60
40
20
ppm

Spectrum

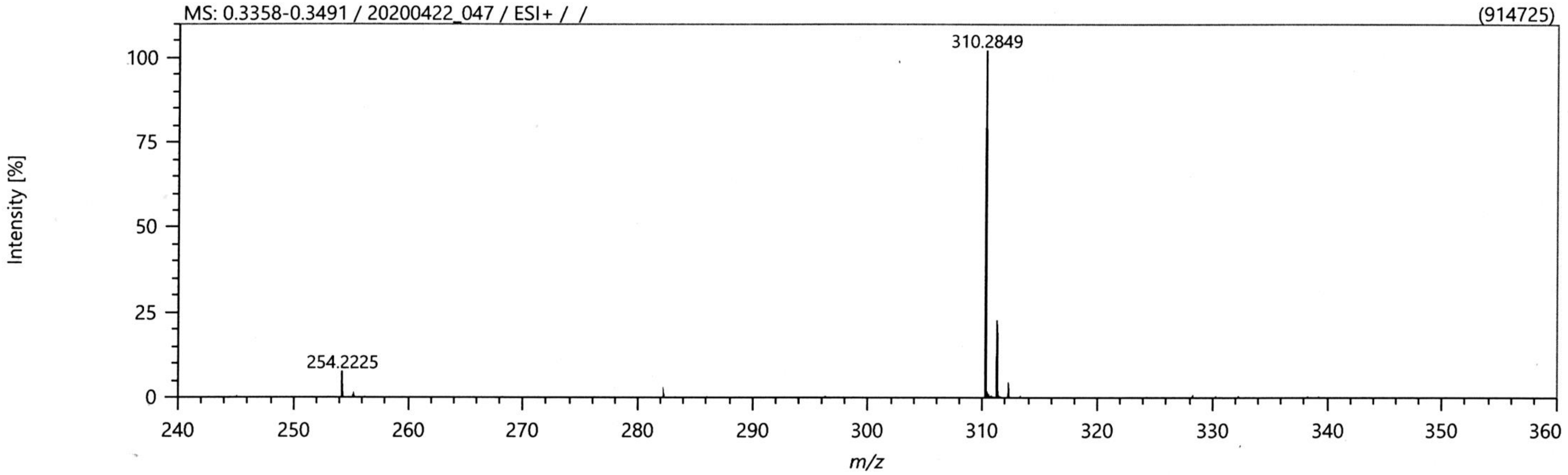

Elemental Composition

Parameters

Tolerance:	±10.00 ppm
Electron:	Odd/Even
Charge:	+1
DBE:	-99.0 - 999.0

Elements Set 1:

Symbol	C	H	O	Na	N	F
Min	0	0	1	0	3	0
Max	1000	4000	1	0	3	0

Results

Mass	Formula	Calculated Mass	Mass Difference [mDa]	Mass Difference [ppm]	DBE
310.28489	C18 H36 N3 O	310.28529	-0.40	-1.30	2.5

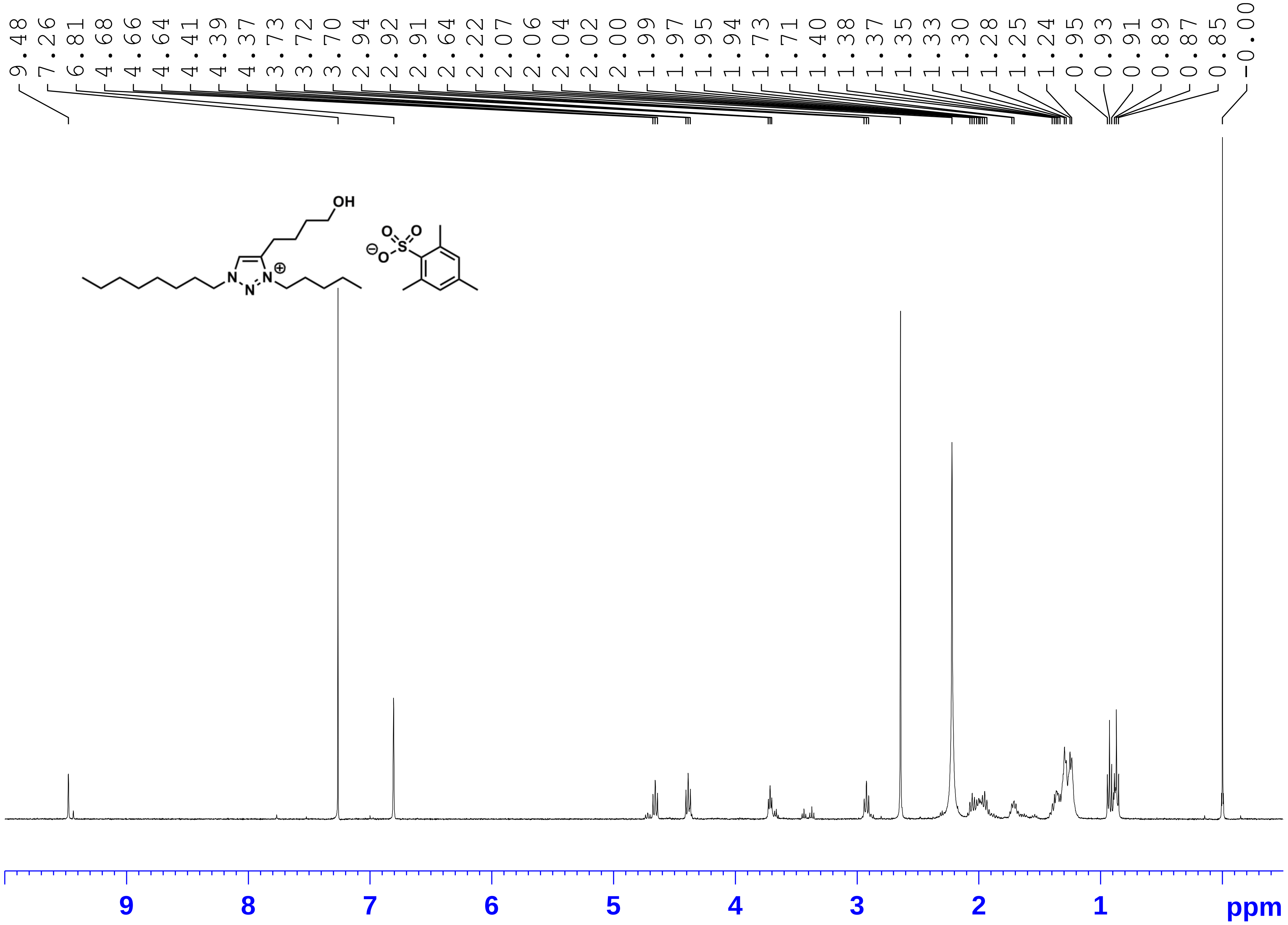

9.48
7.26
6.81
4.68
4.66
4.64
4.41
4.39
4.37
3.73
3.72
3.70
2.94
2.92
2.91
2.64
2.22
2.07
2.06
2.04
2.02
2.00
1.99
1.97
1.95
1.94
1.73
1.71
1.40
1.38
1.37
1.35
1.33
1.30
1.28
1.25
1.24
0.95
0.93
0.91
0.89
0.87
0.85
-0.00
OH
N
N
N
O
S
O
O
9
8
7
6
5
4
3
2
1
ppm

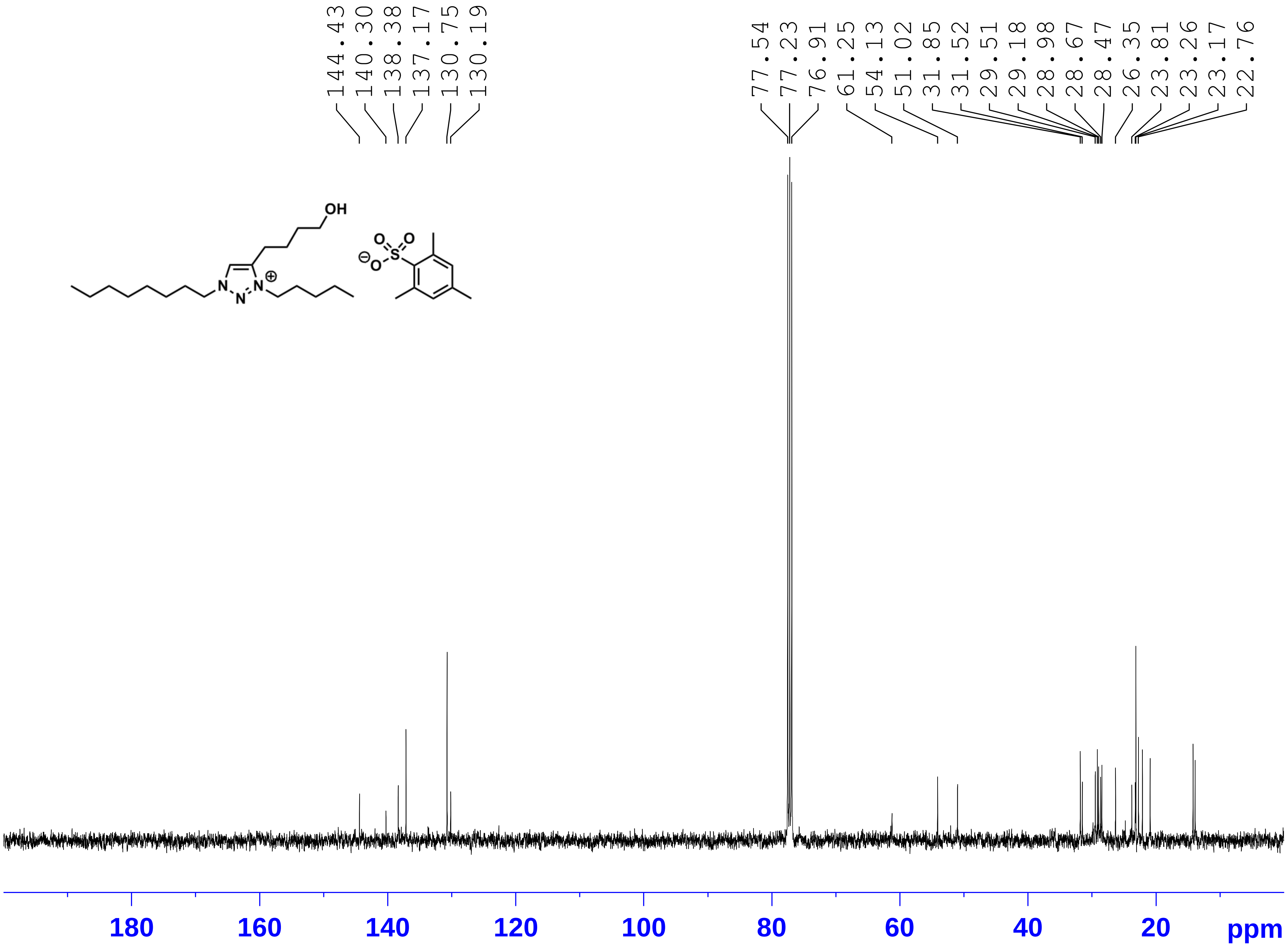
144.43
140.30
138.38
137.17
130.75
130.19
77.54
77.23
76.91
61.25
54.13
51.02
31.85
31.52
29.51
29.18
28.98
28.67
28.47
26.35
23.81
23.26
23.17
22.76
OH
O
S
O
N
N
N
180
160
140
120
100
80
60
40
20
ppm

Spectrum

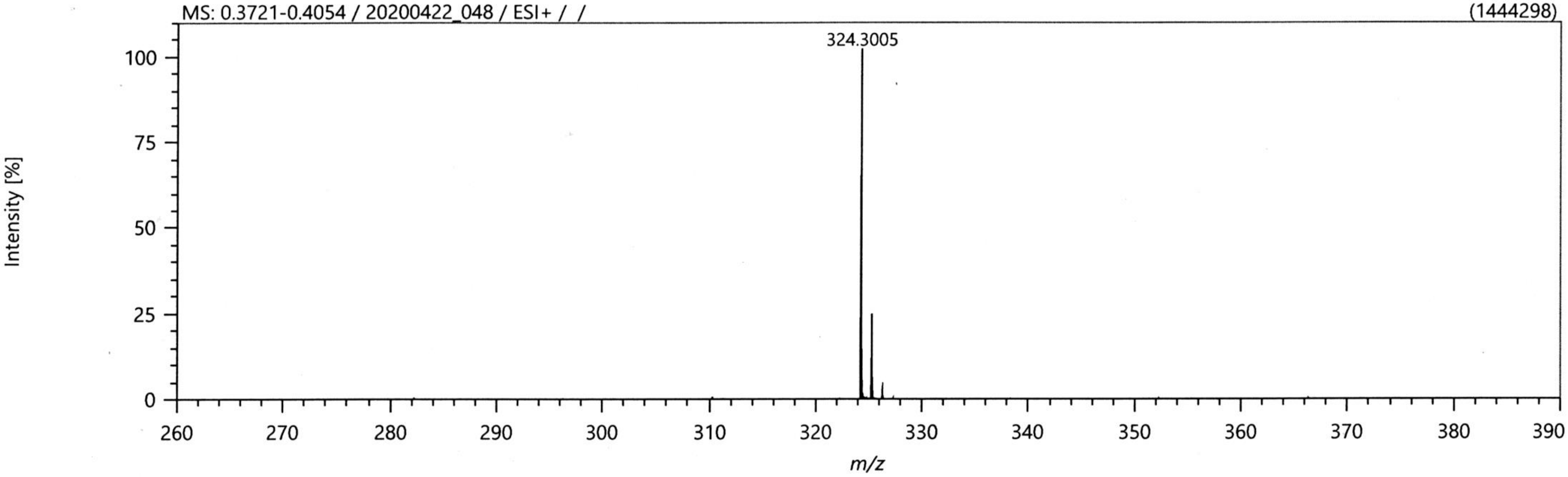

Elemental Composition

Parameters	
Tolerance:	±10.00 ppm
Electron:	Odd/Even
Charge:	+1
DBE:	-99.0 - 999.0

Elements Set 1:

Symbol	C	H	O	Na	N	F
Min	0	0	1	0	3	0
Max	1000	4000	1	0	3	0

Results

Mass	Formula	Calculated Mass	Mass Difference [mDa]	Mass Difference [ppm]	DBE
324.30050	C19 H38 N3 O	324.30094	-0.44	-1.36	2.5

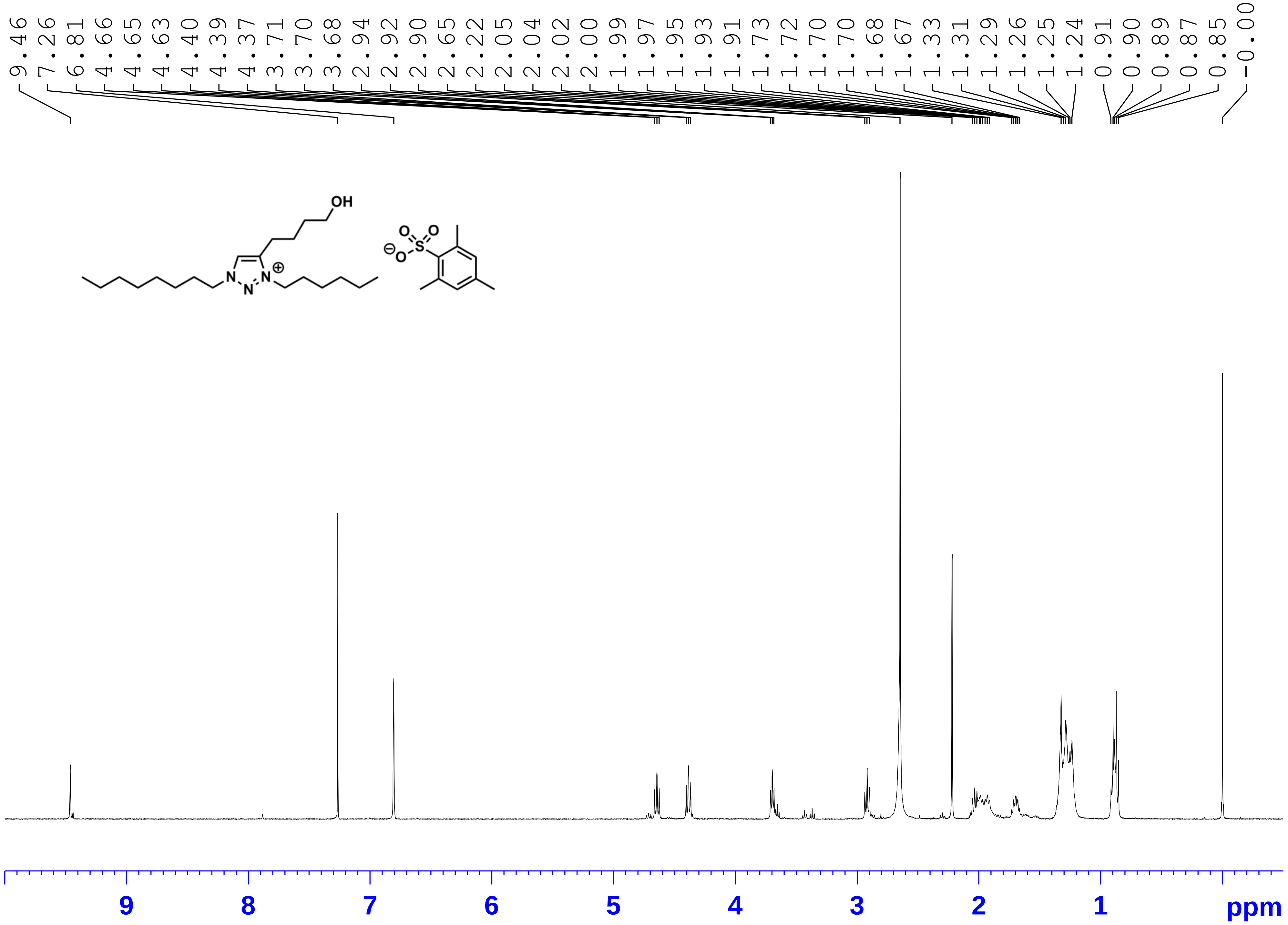

9.46
7.26
6.81
4.66
4.65
4.63
4.40
4.39
4.37
3.71
3.70
3.68
2.94
2.92
2.90
2.65
2.22
2.05
2.04
2.02
2.00
1.99
1.97
1.95
1.93
1.91
1.73
1.72
1.70
1.70
1.68
1.67
1.33
1.31
1.29
1.26
1.25
1.24
0.91
0.90
0.89
0.87
0.85
-0.00
OH
N
N
N
O
S
O
O
9
8
7
6
5
4
3
2
1
ppm

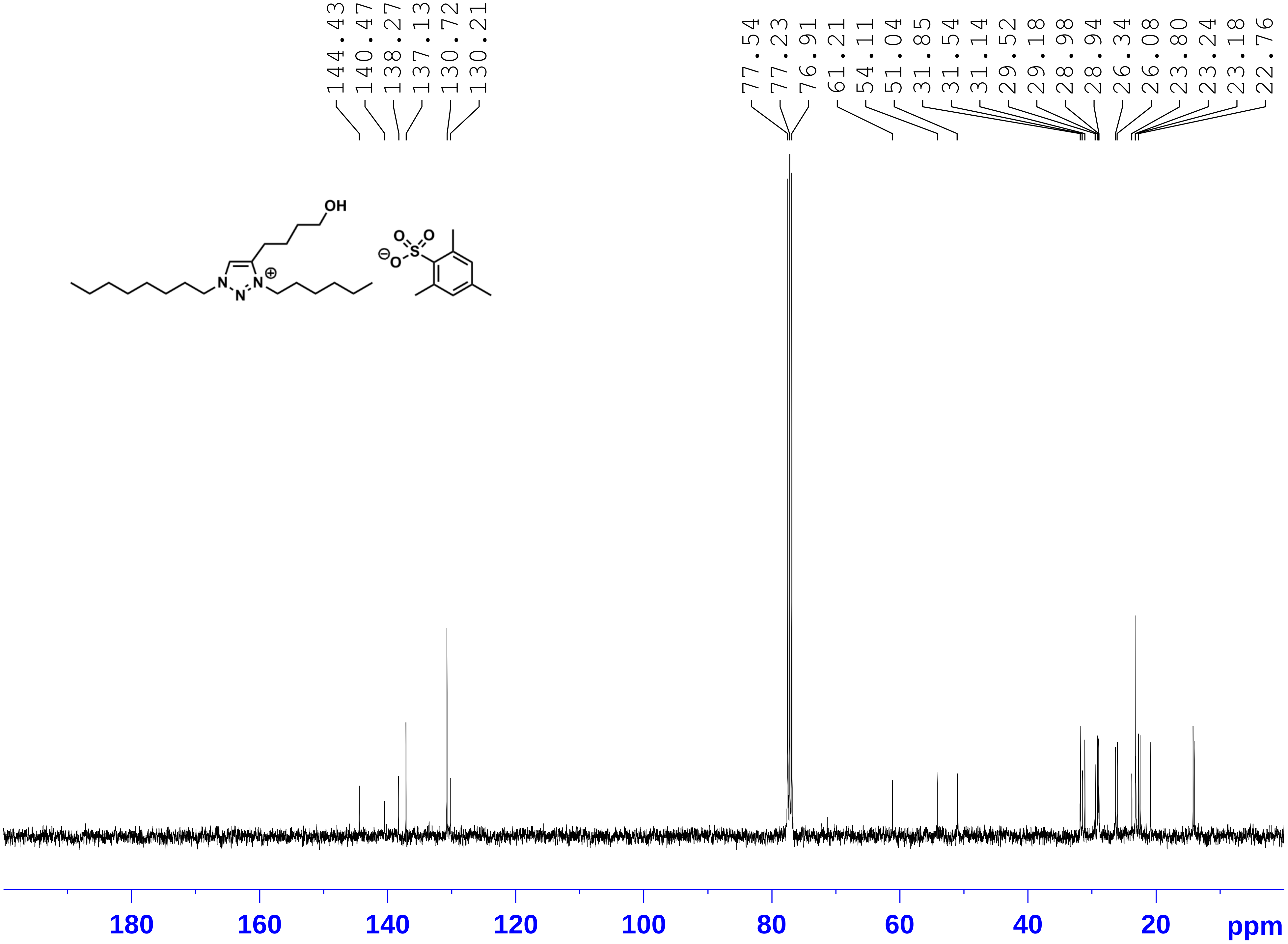

144.43
140.47
138.27
137.13
130.72
130.21
77.54
77.23
76.91
61.21
54.11
51.04
31.85
31.54
31.14
29.52
29.18
28.98
28.94
26.34
26.08
23.80
23.24
23.18
22.76
OH
O
S
O
N
N
N
180
160
140
120
100
80
60
40
20
ppm

Spectrum

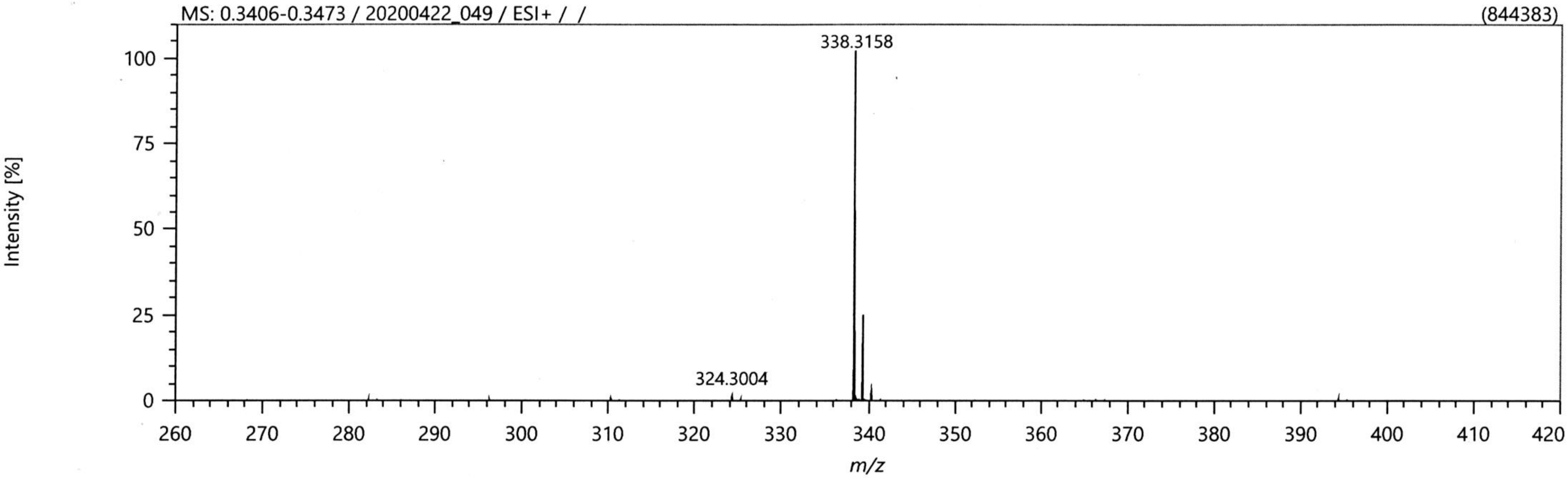

Elemental Composition

Parameters

Tolerance:	±10.00 ppm
Electron:	Odd/Even
Charge:	+1
DBE:	-99.0 - 999.0

Elements Set 1:

Symbol	C	H	O	Na	N	F
Min	0	0	1	0	3	0
Max	1000	4000	1	0	3	0

Results

Mass	Formula	Calculated Mass	Mass Difference [mDa]	Mass Difference [ppm]	DBE
338.31578	C20 H40 N3 O	338.31659	-0.81	-2.39	2.5

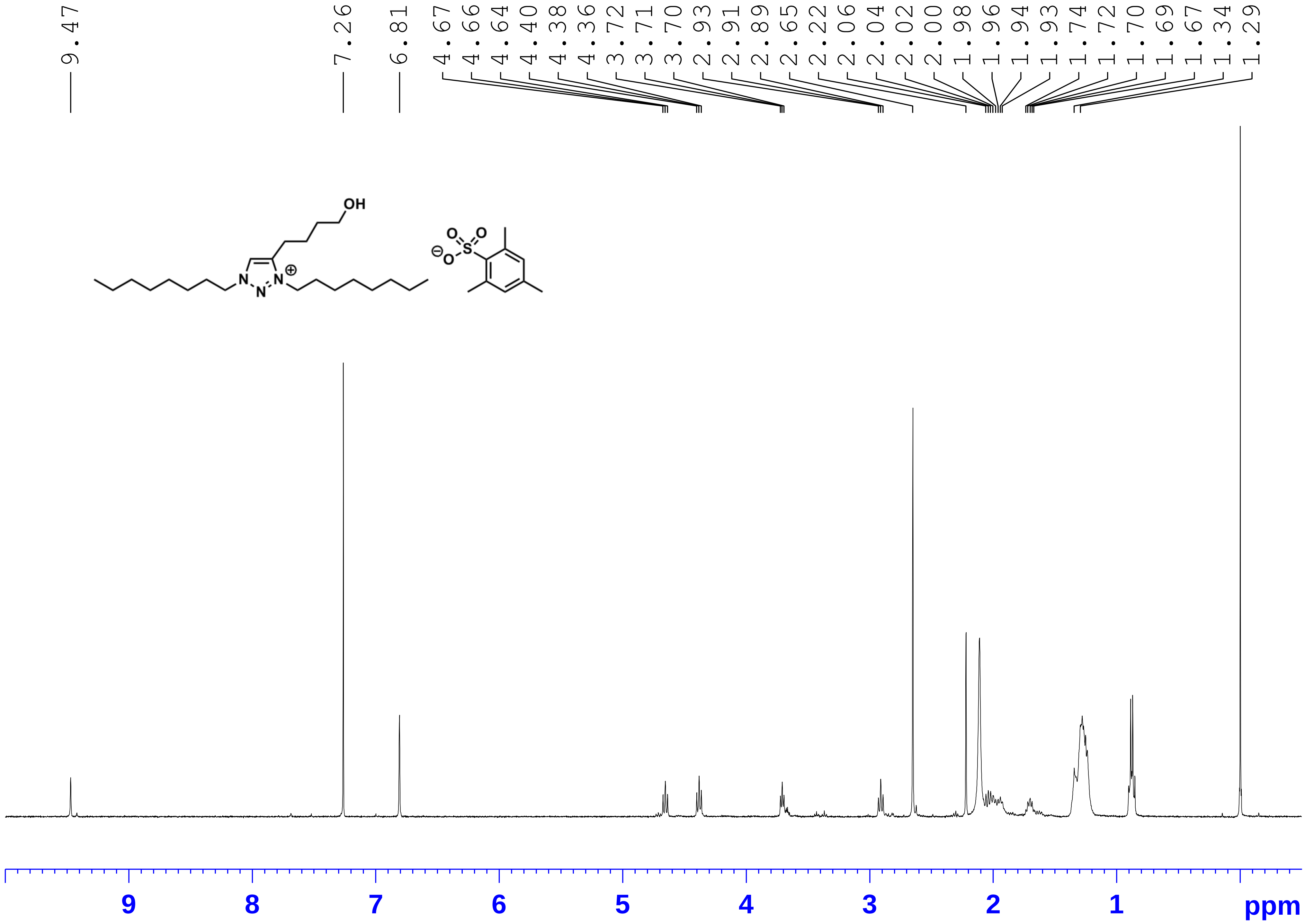

9.47
7.26
6.81
4.67
4.66
4.64
4.40
4.38
4.36
3.72
3.71
3.70
2.93
2.91
2.89
2.65
2.22
2.06
2.04
2.02
2.00
1.98
1.96
1.94
1.93
1.74
1.72
1.70
1.69
1.67
1.34
1.29
OH
N
N
N
O
O
S
O
9
8
7
6
5
4
3
2
1
ppm

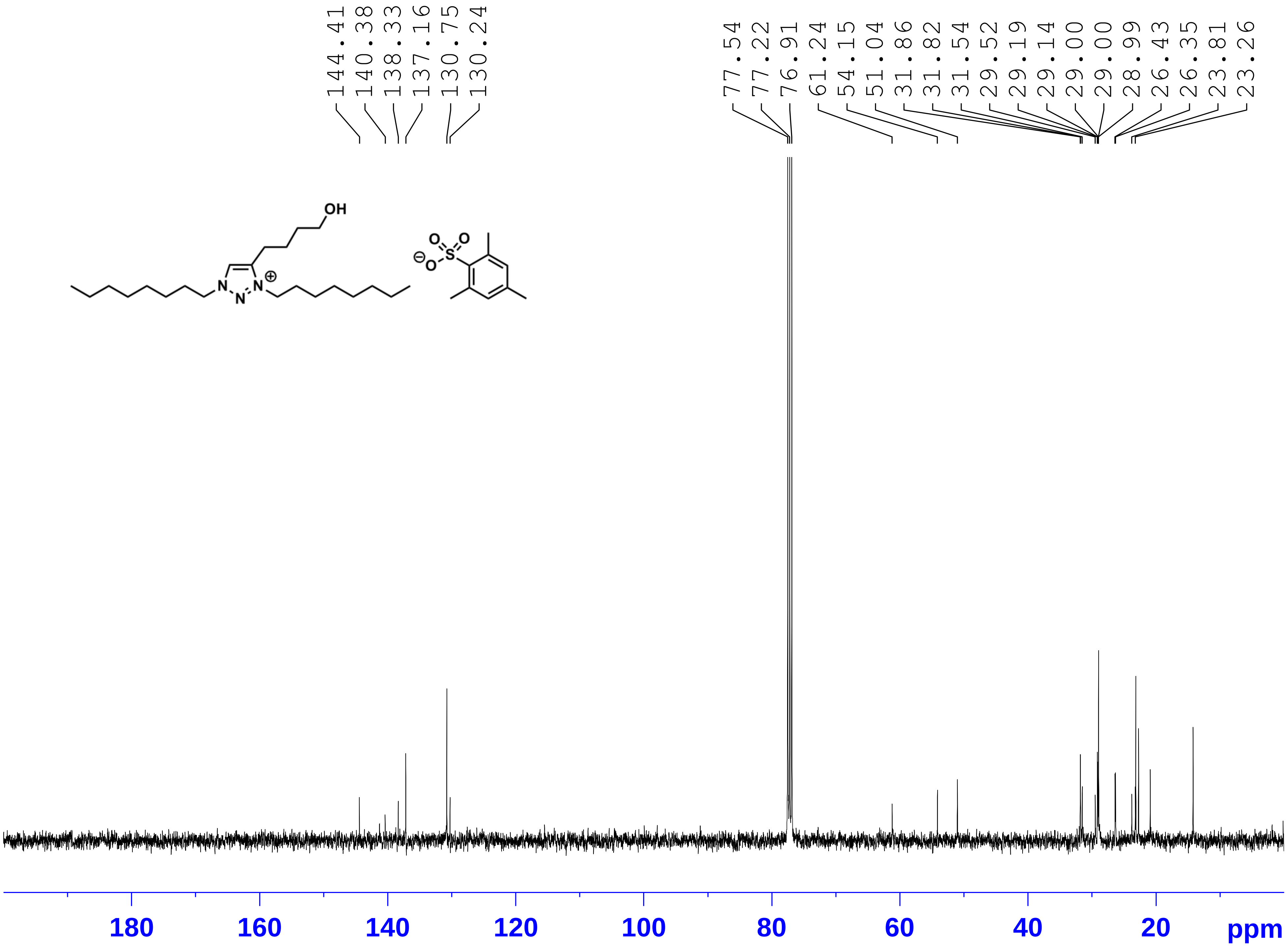
144.41
140.38
138.33
137.16
130.75
130.24
77.54
77.22
76.91
61.24
54.15
51.04
31.86
31.82
31.54
29.52
29.19
29.14
29.00
29.00
28.99
26.43
26.35
23.81
23.26
OH
N
N
N
O
O
S
O
180
160
140
120
100
80
60
40
20
ppm

Spectrum

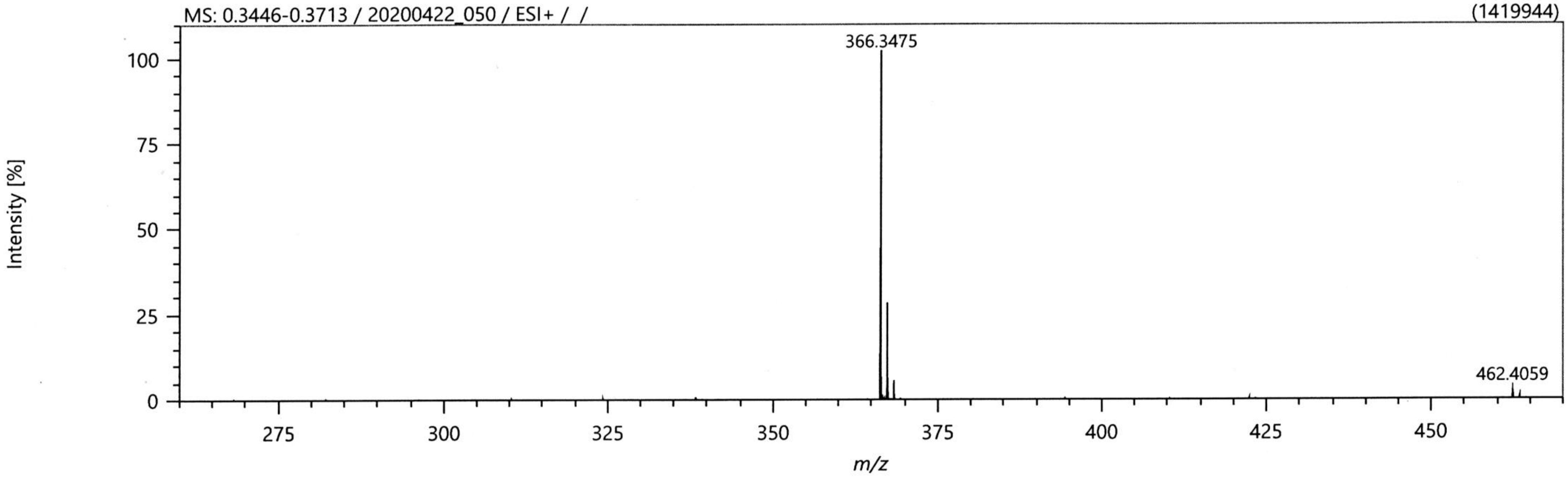

Elemental Composition

Parameters		Elements Set 1:						
Tolerance:	±10.00 ppm	Symbol	C	H	O	Na	N	F
Electron:	Odd/Even	Min	0	0	1	0	3	0
Charge:	+1	Max	1000	4000	1	0	3	0
DBE:	-99.0 - 999.0							

Results

Mass	Formula	Calculated Mass	Mass Difference [mDa]	Mass Difference [ppm]	DBE
366.34752	C22 H44 N3 O	366.34789	-0.37	-1.01	2.5

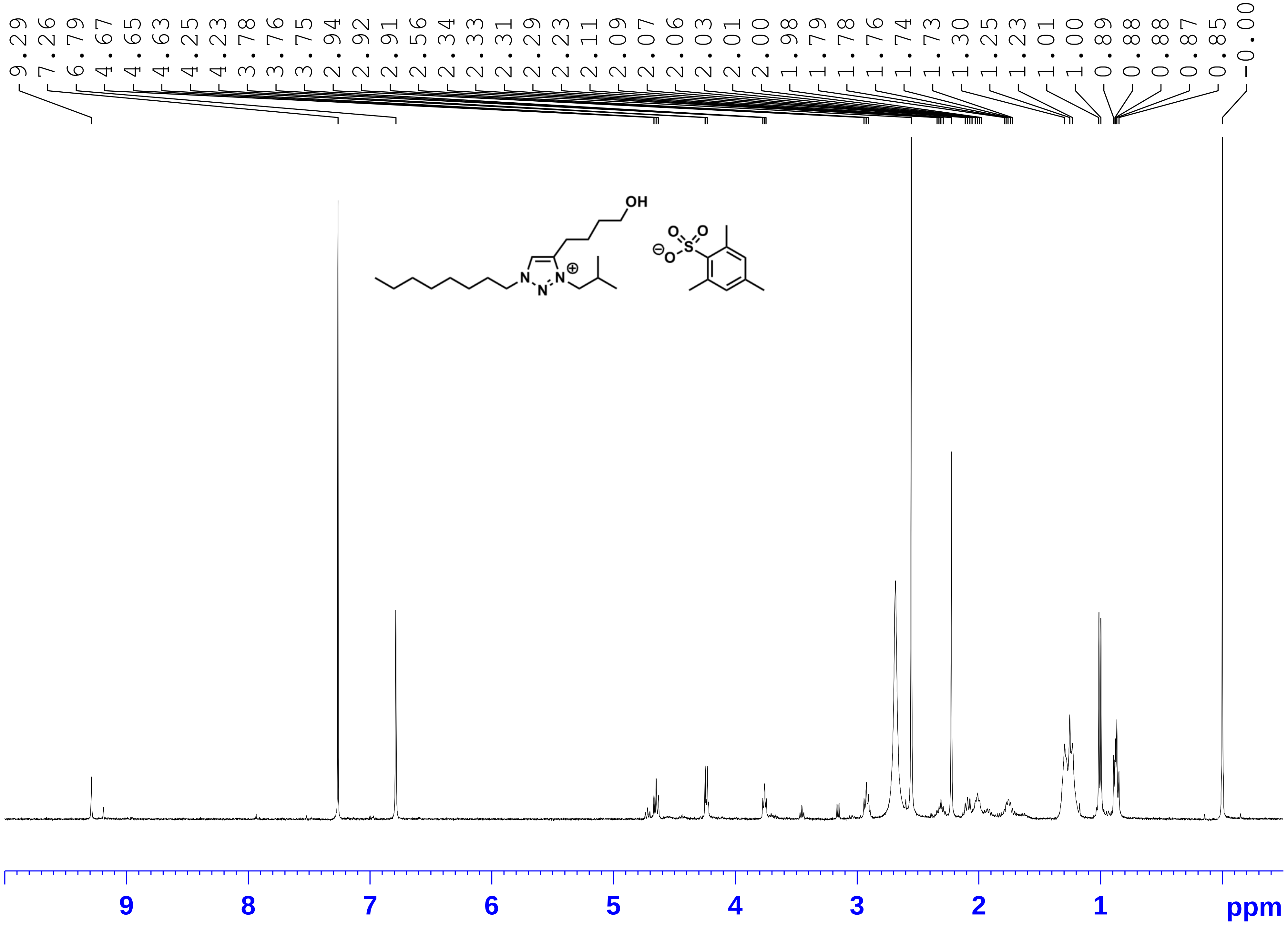

9.29
7.26
6.79
4.67
4.65
4.63
4.25
4.23
3.78
3.76
3.75
2.94
2.92
2.91
2.56
2.34
2.33
2.31
2.29
2.23
2.11
2.09
2.07
2.06
2.03
2.01
2.00
1.98
1.79
1.78
1.76
1.74
1.73
1.30
1.25
1.23
1.01
1.00
0.89
0.88
0.88
0.87
0.85
-0.00
OH
9
8
7
6
5
4
3
2
1
ppm

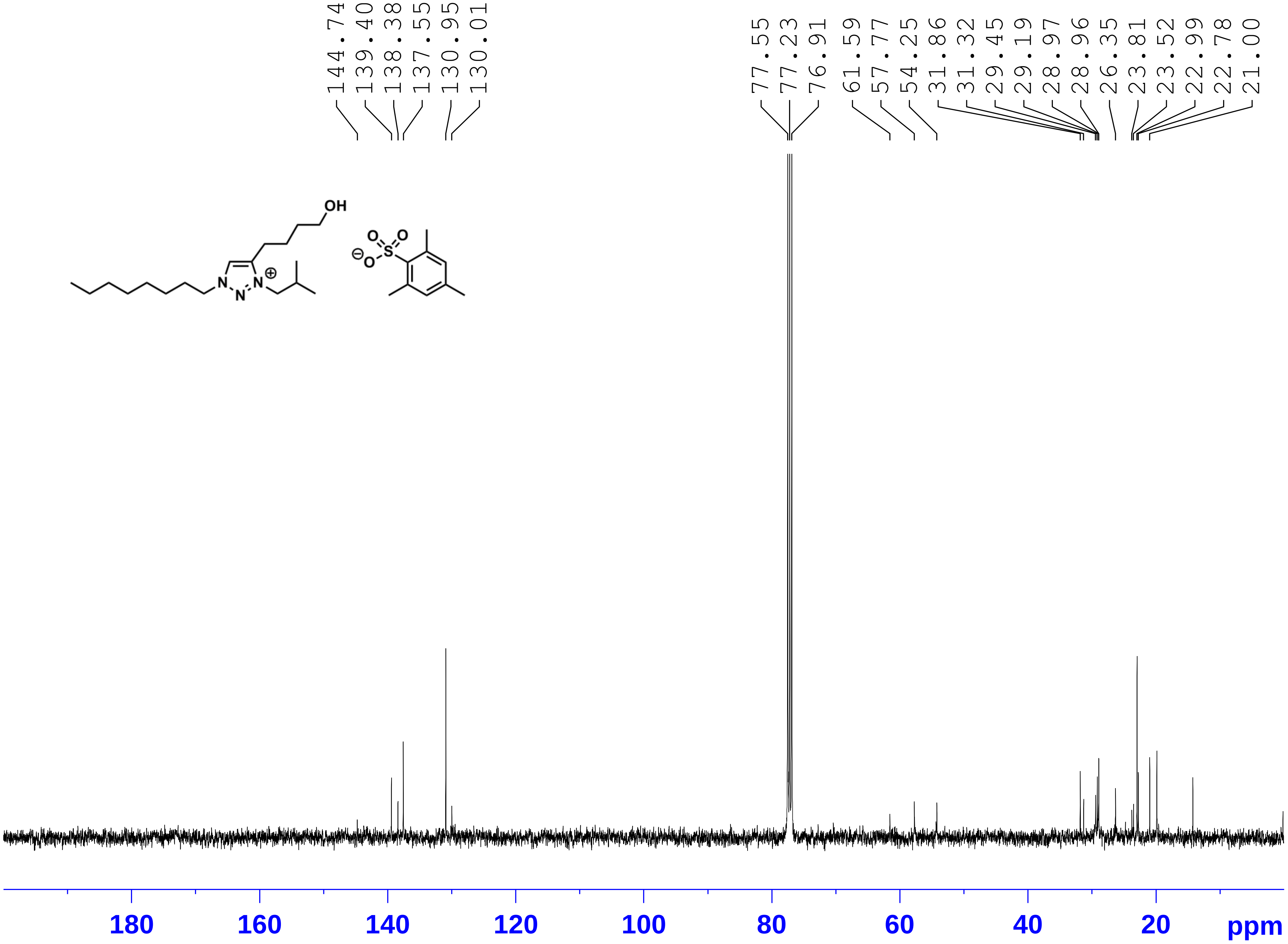
144.74
139.40
138.38
137.55
130.95
130.01
77.55
77.23
76.91
61.59
57.77
54.25
31.86
31.32
29.45
29.19
28.97
28.96
26.35
23.81
23.52
22.99
22.78
21.00
OH
O
S
O
O
N
N
N
180
160
140
120
100
80
60
40
20
ppm

Spectrum

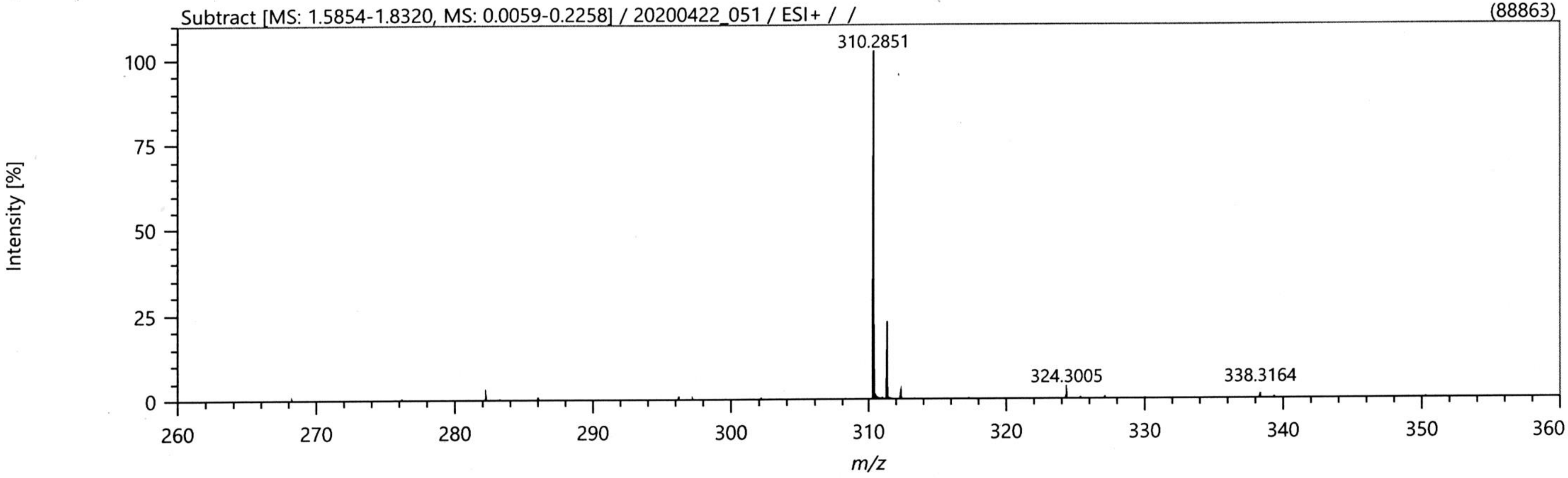

Elemental Composition

Parameters		Elements Set 1:						
Tolerance:	±10.00 ppm	Symbol	C	H	O	Na	N	F
Electron:	Odd/Even	Min	0	0	1	0	3	0
Charge:	+1	Max	1000	4000	1	0	3	0
DBE:	-99.0 - 999.0							

Results

Mass	Formula	Calculated Mass	Mass Difference [mDa]	Mass Difference [ppm]	DBE
310.28511	C18 H36 N3 O	310.28529	-0.18	-0.58	2.5
